# Supplementary material for: Novel Adomaviruses Associated with Blotchy Bass Syndrome in Black Basses (Micropterus spp.)
Source: bioRxiv. 2025 Jun 5:2025.06.01.657292. Preprint. [Version 2] doi: 10.1101/2025.06.01.657292 (PMC12478380; doi:10.1101/2025.06.01.657292)
Supplement: Supplement 1 [file media-1.pdf]

Supplemental Figure 1: Diagnostic case report of the first recorded observation of blotchy bass syndrome in largemouth bass.

ANIMAL PATHOLOGY RECORD
DEPARTMENT OF ANIMAL PATHOLOGY
COLLEGE OF RESOURCE DEVELOPMENT
UNIVERSITY OF RHODE ISLAND
KINGSTON, R. I. 02881

Date Submitted: 11-20-84

Accession No.: 0 293

Date Received:

Veterinarian	Address	Phone
Dr. Richard Wolke	25 Main Street, Carolina, RI	792-2334 (work)

Owner	Address
Marty Wencek	811 Middlebridge Rd., Narragansett, RI 02882

Ident.	Animal	Breed	Color	Age	Sex	Weight	Prev. Acces.
Micropterus	salmoides				female		

Clinical Diagnosis:

History and Clinical Summary:
Fish collected by means of angling. Caught in Roundout Creek, tributary to the Hudson River Kingston, NY. A total of 81 bass were taken over an 8 hour period (7:30 a.m. - 3:30 p.m.). Dark external patches occurred only on fish over approx. 30 cm in length. The larger the specimmn, the more numerous the black patches. Not all fish over 30 cm exhibited these markings. They occurred on approx. 33% of fish between 30 cm and 35 cm. Specimens greater than 35 cm showed these markings approx. 50%+ of the time. The number of patches varied from fish to fish, some more covered than others. Patches were ~~xxx~~ most numerous on either side of the body, the fins, particularly the dorsal, anal

Specimen Submitted: Live Animal ☐ Dead Animal ☒ Tissues ☐

Preservation: Fresh ☒ Frozen ☐ Fixed ☐

Condition of Specimen When Received at Lab:

Biopsy Data Exact Location:

Size: 42 cm

Duration:

Encapsulated: YES ☐ NO ☐

Lymph node involvement: YES ☐ NO ☐

Autopsy Data Natural Death ☐

Mode of Euthanasia:

Time and Date of Death:

Time and Date of Autopsy:

Tissues Submitted:

Findings:
and caudal, and on the lips. Fish were taken from 1 meter to 5 meters deep on 3" plastic tail jig lures and 4" plastic worms -- from weedy areas (submergents) to rocky shorelines - also adjacent to sunken barges. Water temp. ~9°C. Clarity <1 meter. Tidal amplitude approx. 1 meter. Weather conditions overcast, 38°, northerly breeze.

There are round to rectangular pigmented (black) areas above lateral line and on dorsal fin. Areas are 4 x 2 to 0.5 x 1 cm and do not extend into dermis. They number 8. The anal fin also has such an area. Mesentary was adhering to the peritoneum.

SEE OVER

Diagnoses: Peritonitis, Granulomatous, Verminous, Melanosis

Classification: P8.2a U3C

Tissues saved ☐
Photographs ☐
Radiographs ☐

Pathologist: R. E. Wolke

Date Prepared: 12-14684

Fee:
