# Supplementary material for: Novel Adomaviruses Associated with Blotchy Bass Syndrome in Black Basses (Micropterus spp.)
Source: bioRxiv. 2025 Jun 5:2025.06.01.657292. Preprint. [Version 2] doi: 10.1101/2025.06.01.657292 (PMC12478380; doi:10.1101/2025.06.01.657292)

Supplemental Figure 2: Circular genome maps of Micropterus dolomieu adomavirus 1 (MdA-1) and Micropterus nigricans adomavirus 1 (MnA-1). Core adomavirus ORFs are colored in non-gray.

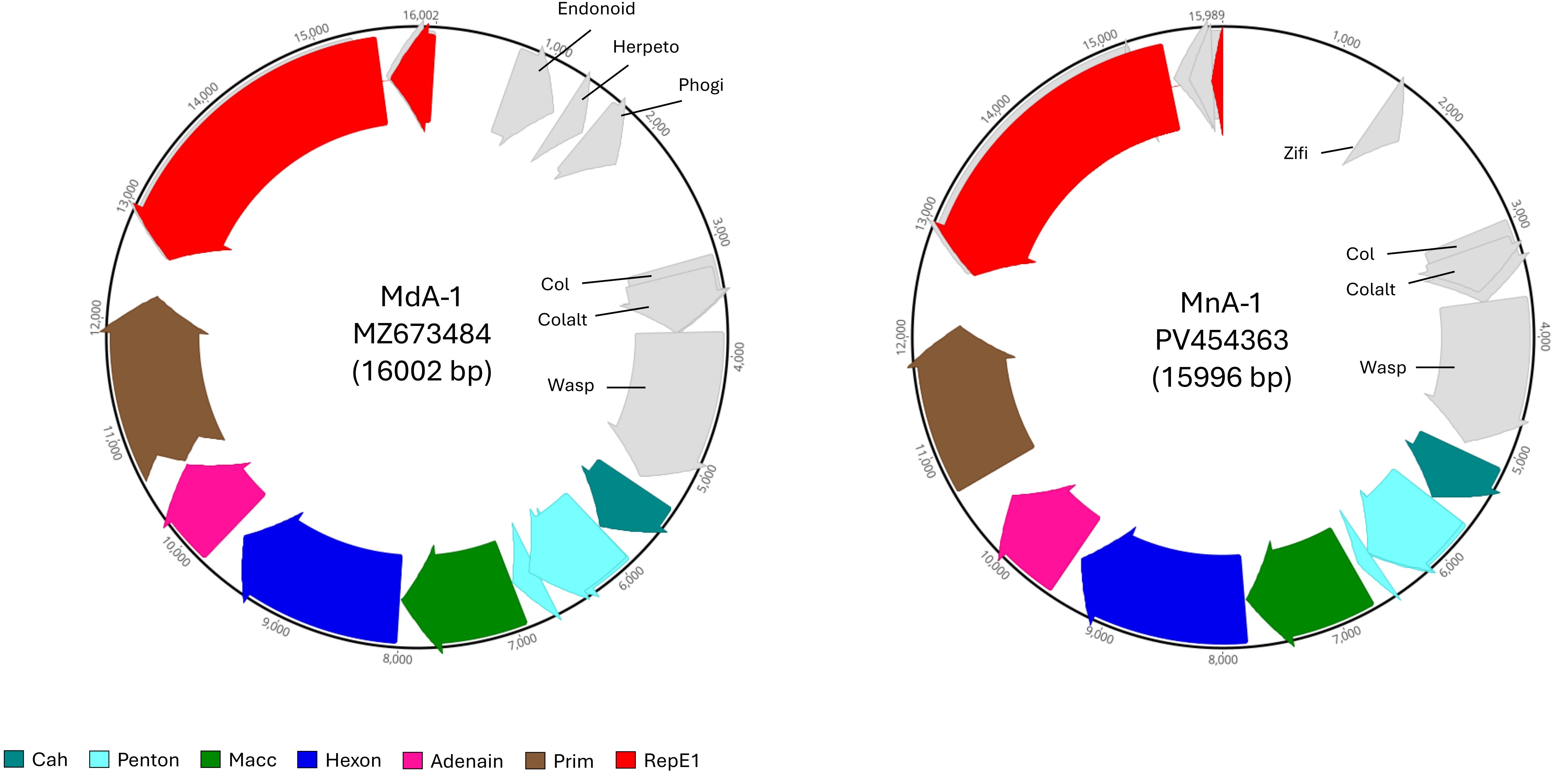

Supplement: Supplement 2 [file media-2.pdf]
