# Supplementary material for: Novel Adomaviruses Associated with Blotchy Bass Syndrome in Black Basses (Micropterus spp.)
Source: bioRxiv. 2025 Jun 5:2025.06.01.657292. Preprint. [Version 2] doi: 10.1101/2025.06.01.657292 (PMC12478380; doi:10.1101/2025.06.01.657292)

Supplemental Figure 3: Gross and histological presentation of the hyperpigmented melanistic lesions (HPML) associated with MdA-1 infection and the mucoid lesions associated with MdA-2 in smallmouth bass.

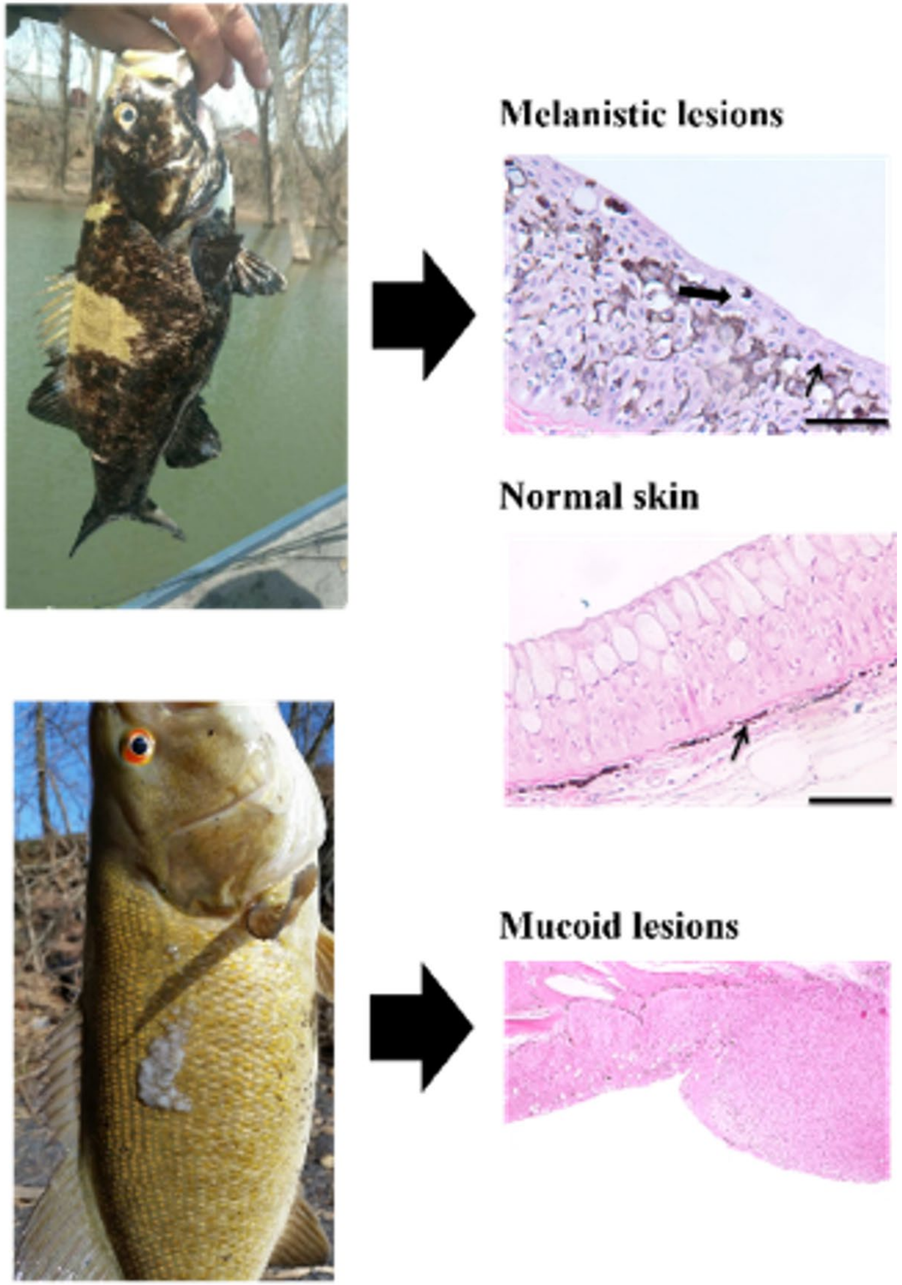

Supplement: Supplement 3 [file media-3.pdf]
