# Supplementary material for: Novel Adomaviruses Associated with Blotchy Bass Syndrome in Black Basses (Micropterus spp.)
Source: bioRxiv. 2025 Jun 5:2025.06.01.657292. Preprint. [Version 2] doi: 10.1101/2025.06.01.657292 (PMC12478380; doi:10.1101/2025.06.01.657292)

Supplemental Figure 4. RNAScope analysis of HPMLs in infected smallmouth bass skin sampled during the fall. The adenain transcript of MdA-1 was targeted. Cells positive for adomavirus nucleic acids are restricted to the epidermis. While positive signal was sometimes observed in melanocytes, it was more commonly observed in non-pigmented, epithelial cells of the epidermis.

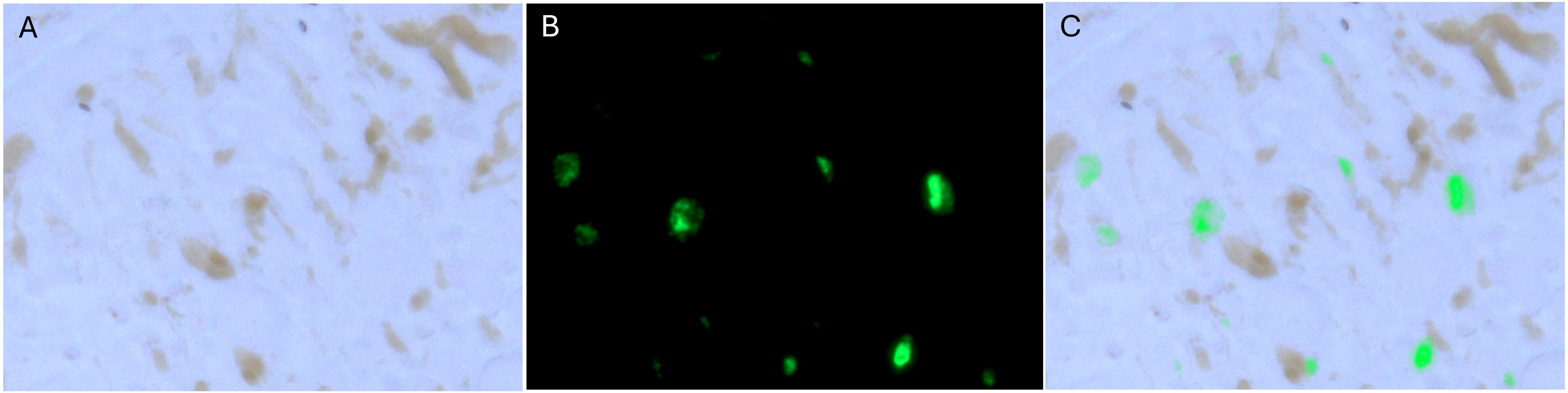

Supplement: Supplement 4 [file media-4.pdf]
