# Supplementary material for: Novel Adomaviruses Associated with Blotchy Bass Syndrome in Black Basses (Micropterus spp.)
Source: bioRxiv. 2025 Jun 5:2025.06.01.657292. Preprint. [Version 2] doi: 10.1101/2025.06.01.657292 (PMC12478380; doi:10.1101/2025.06.01.657292)

Supplemental Figure 5. RNAScope analysis of HPMLS in infected largemouth bass sampled in the spring. The adenain transcript of MnA-1 was targeted. Cells positive for adomavirus nucleic acids are observed in the epidermis, but most are observed in the basement membrane.

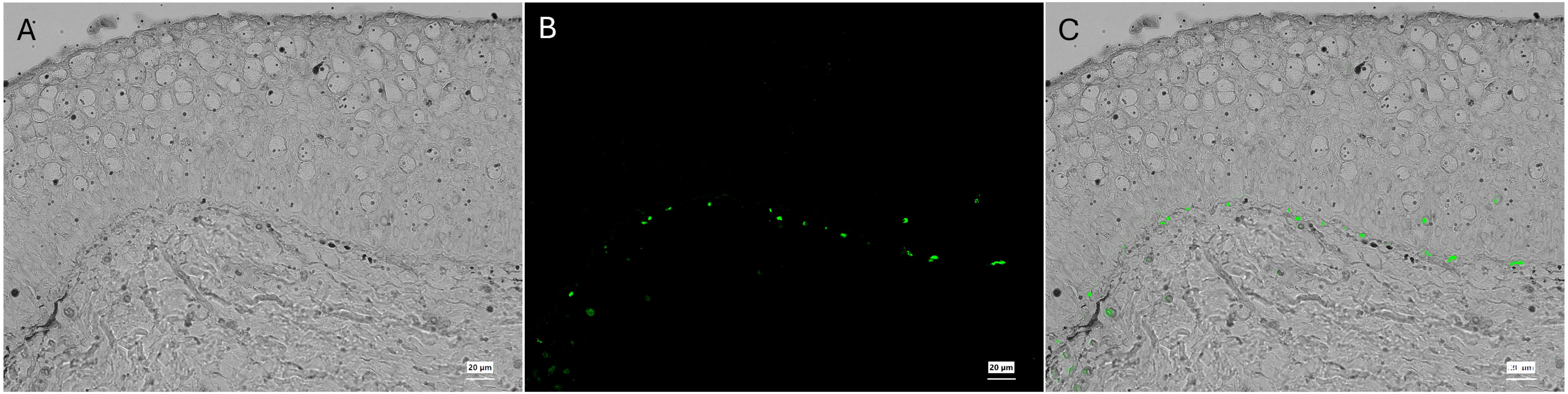

Supplement: Supplement 5 [file media-5.pdf]
