# Supplementary material for: Novel Adomaviruses Associated with Blotchy Bass Syndrome in Black Basses (Micropterus spp.)
Source: bioRxiv. 2025 Jun 5:2025.06.01.657292. Preprint. [Version 2] doi: 10.1101/2025.06.01.657292 (PMC12478380; doi:10.1101/2025.06.01.657292)
Supplement: Supplement 6 [file media-6.pdf]

Col

Pairwise Identity = 63.4%

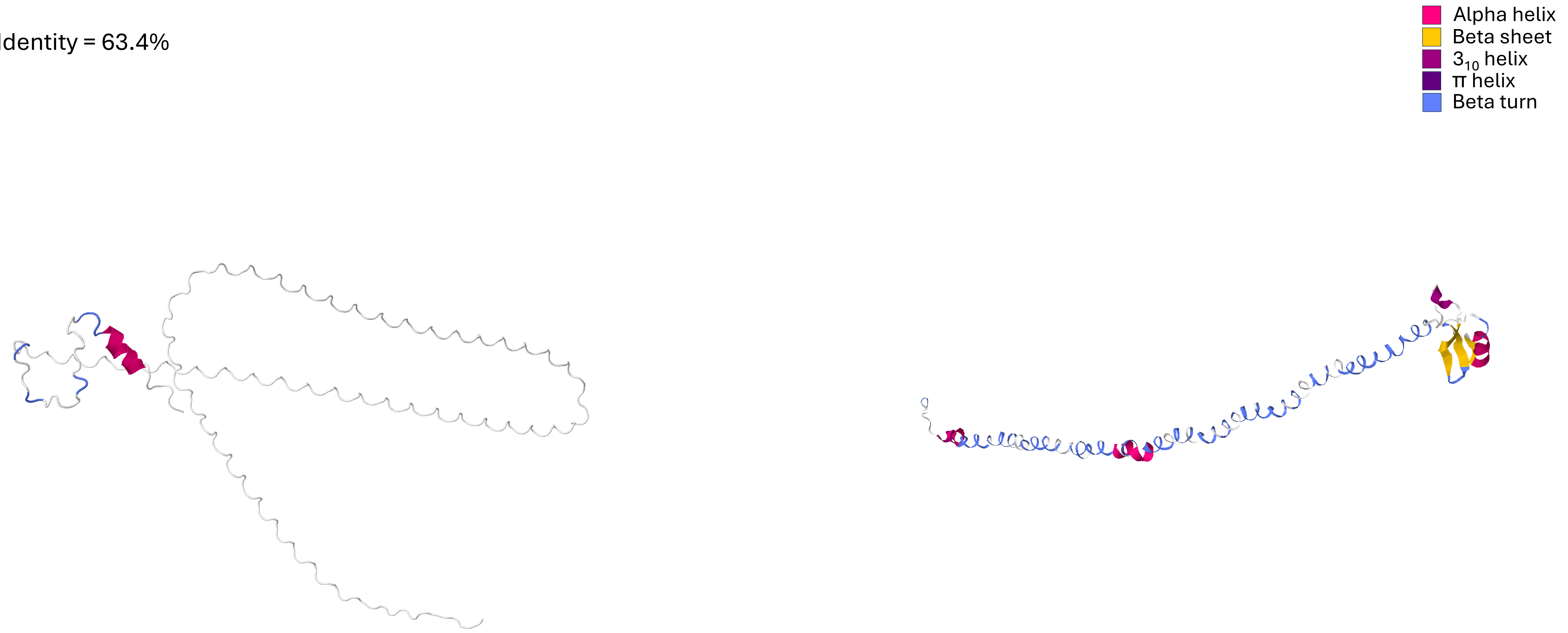

MdA-1
(UFQ21626)

MnA-1
(XQZ12361)

Colalt

Pairwise Identity = 41.0%

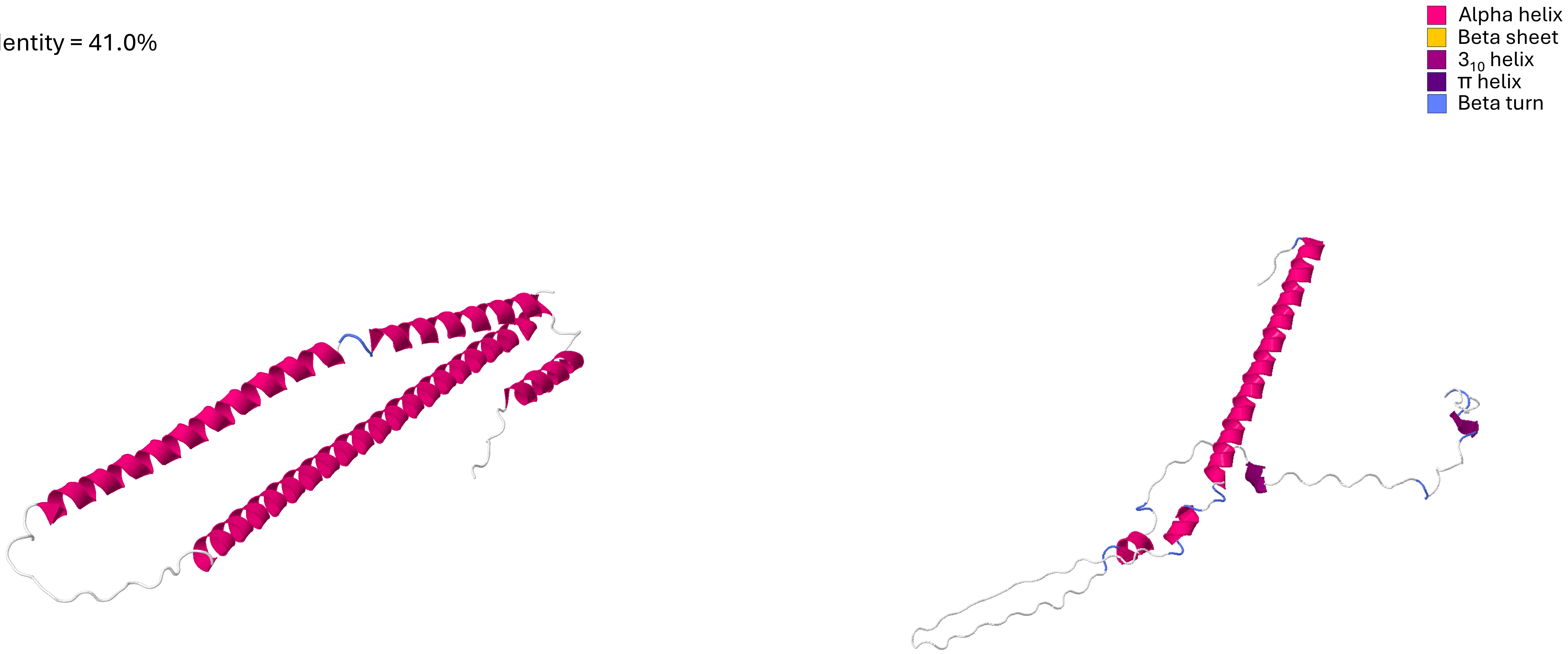

MdA-1
(UFQ21627)

MnA-1
(XQZ12362)

Wasp

Pairwise Identity = 51.7%

Alpha helix
Beta sheet
3_{10} helix
π helix
Beta turn

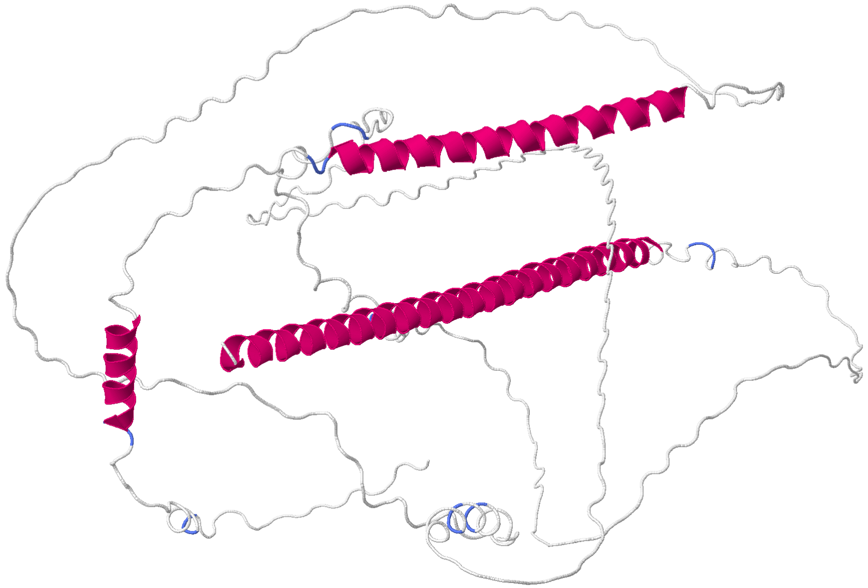

MdA-1
(UFQ21628)

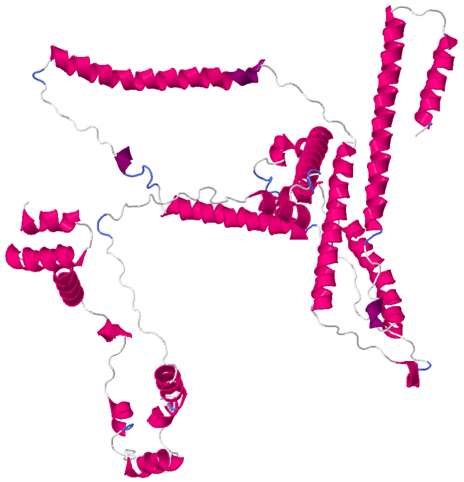

MnA-1
(XQZ12363)

Cah

Pairwise Identity = 82.5%

Alpha helix
Beta sheet
3_{10} helix
π helix
Beta turn

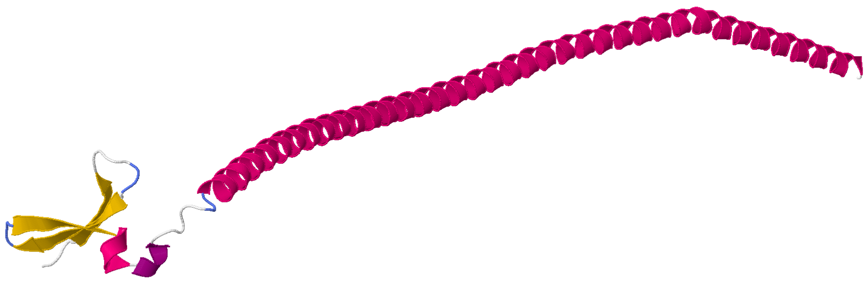

MdA-1
(UFQ21629)

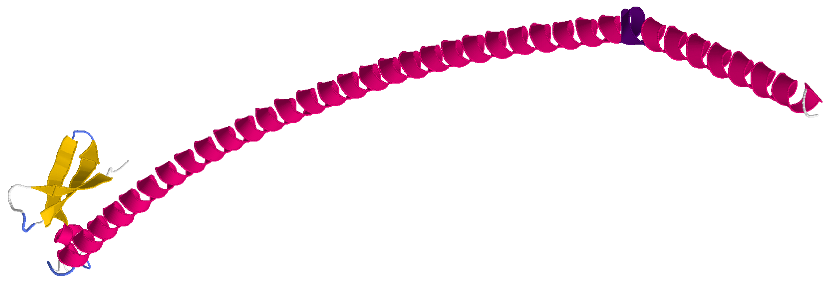

MnA-1
(XQZ12364)

Macc

Pairwise Identity = 74.0%

- Alpha helix
- Beta sheet
- 3_{10} helix
- π helix
- Beta turn

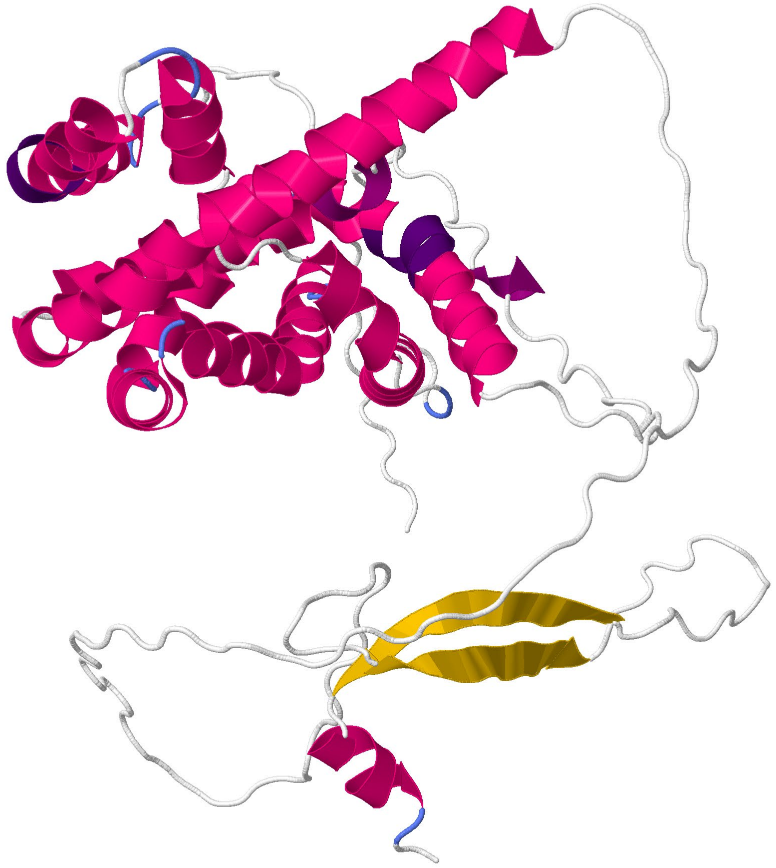

MdA-1
(UFQ21631)

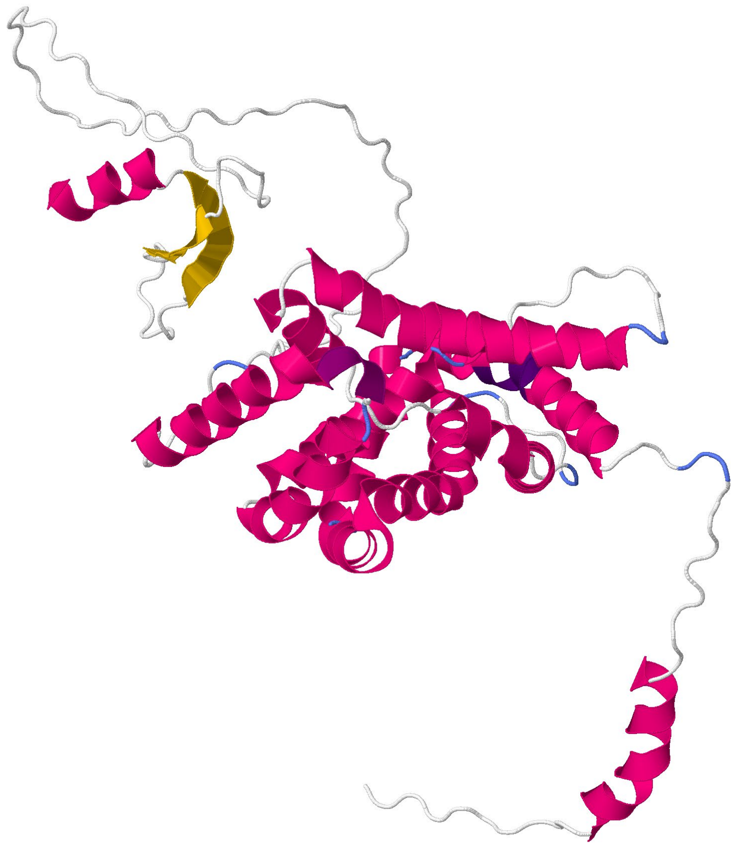

MnA-1
(XQZ12367)

Penton

Pairwise Identity = 86.8%

Alpha helix
Beta sheet
3_{10} helix
π helix
Beta turn

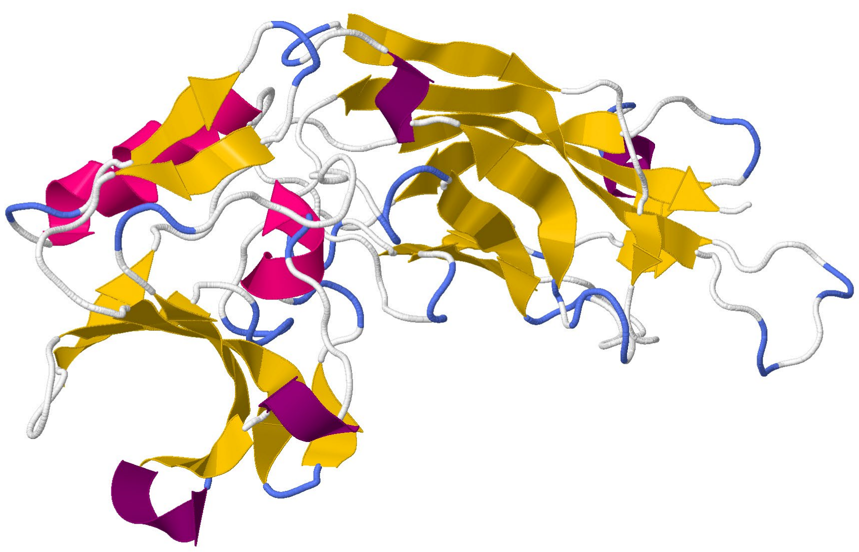

MdA-1
(UFQ21630)

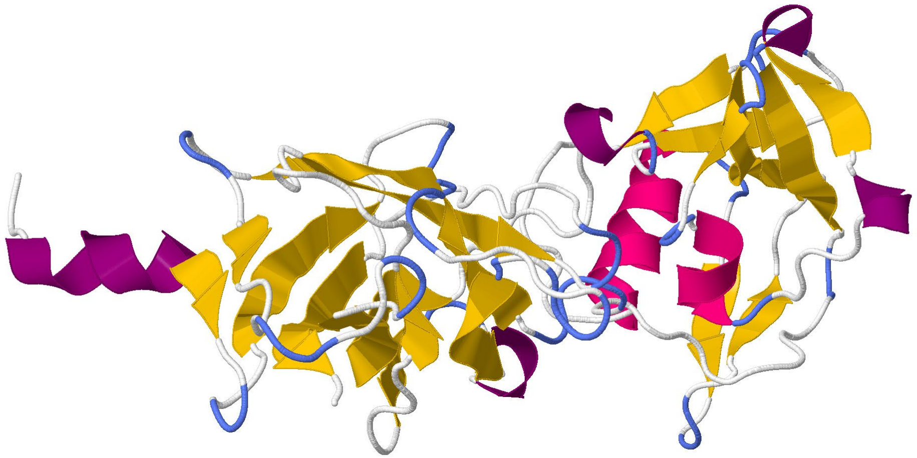

MnA-1
(XQZ12365)

Penton+

Pairwise Identity = 83.4%

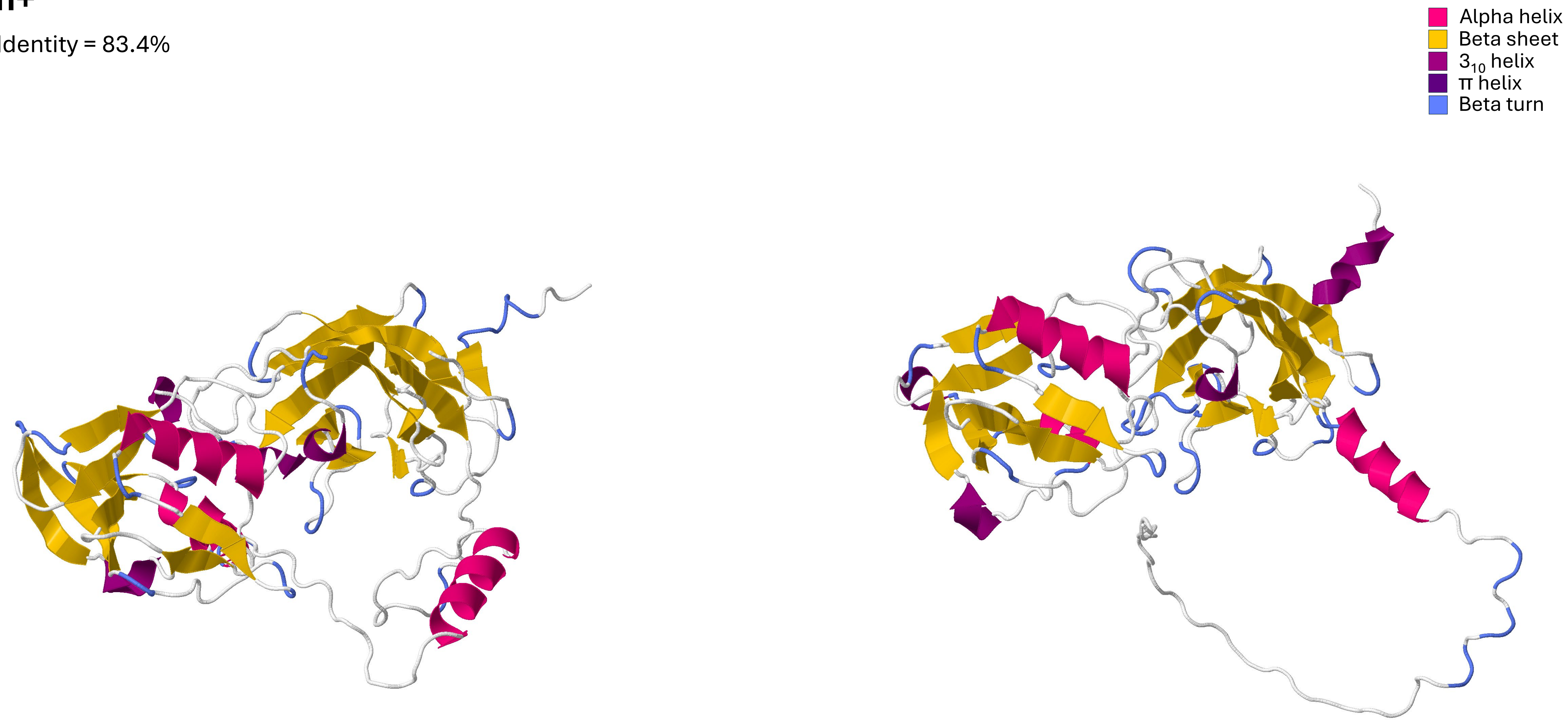

MdA-1
(XQU54337)

MnA-1
(XQZ12366)

Hexon

Pairwise Identity = 92.2%

- Alpha helix
- Beta sheet
- 3_{10} helix
- π helix
- Beta turn

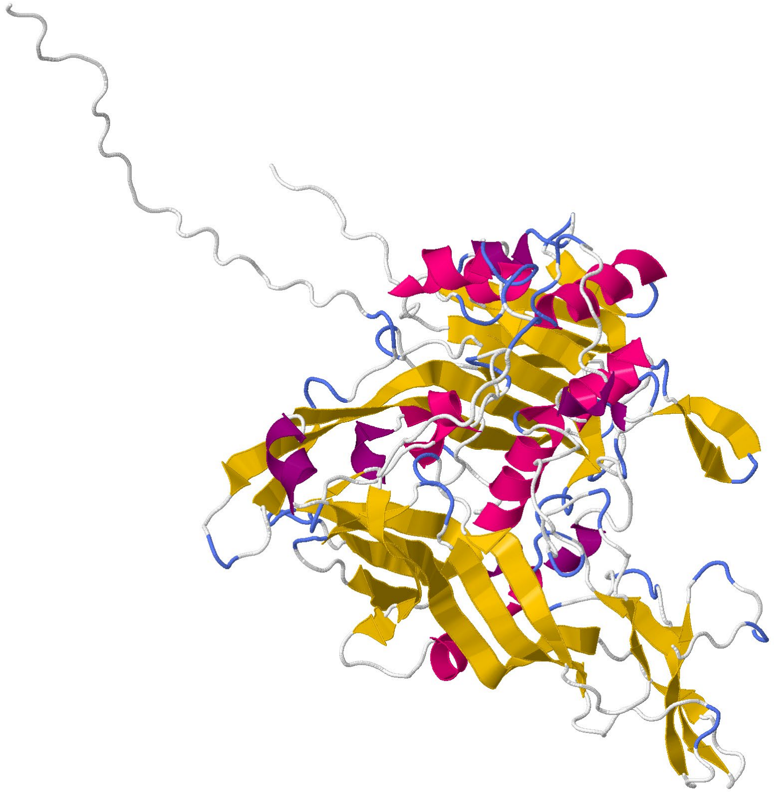

MdA-1
(UFQ21632)

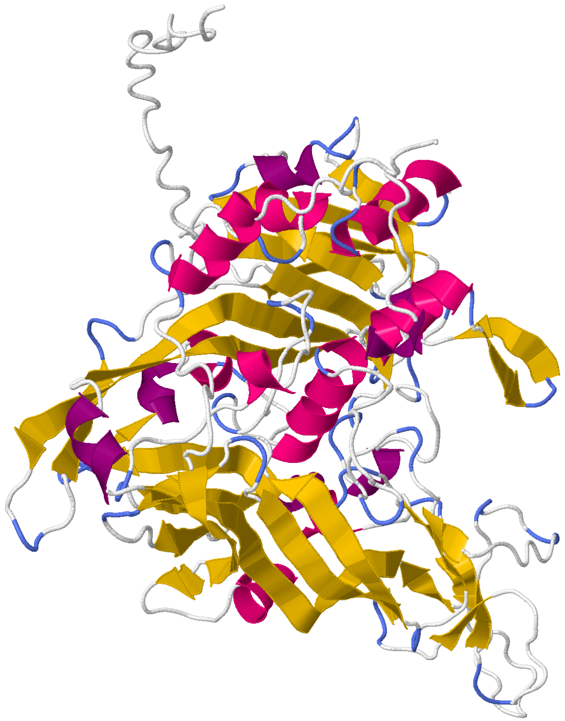

MnA-1
(XQZ1236)

Adenain

Pairwise Identity = 72.4%

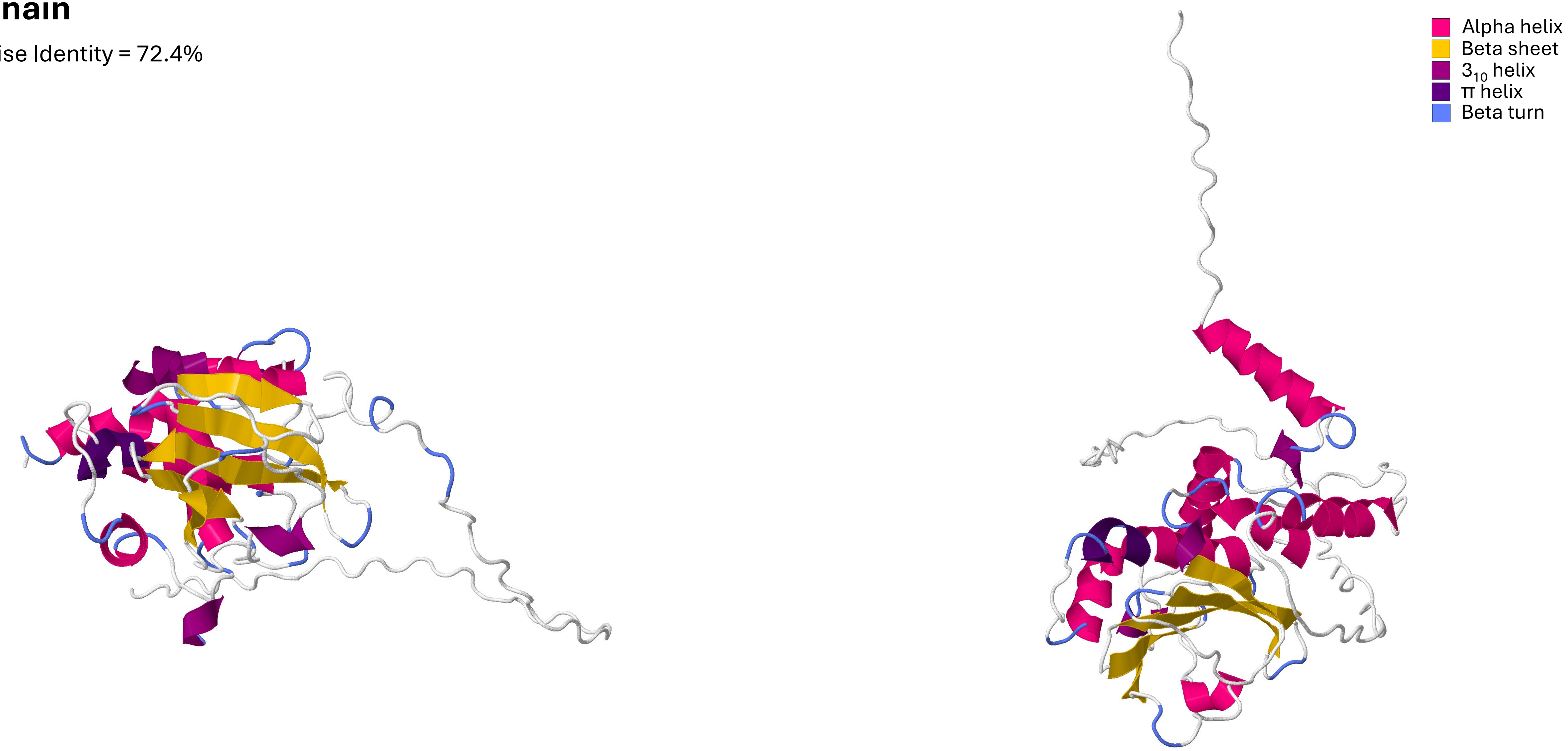

MdA-1
(UFQ21633)

MnA-1
(XQZ12369)

Prim

Pairwise Identity = 76.9%

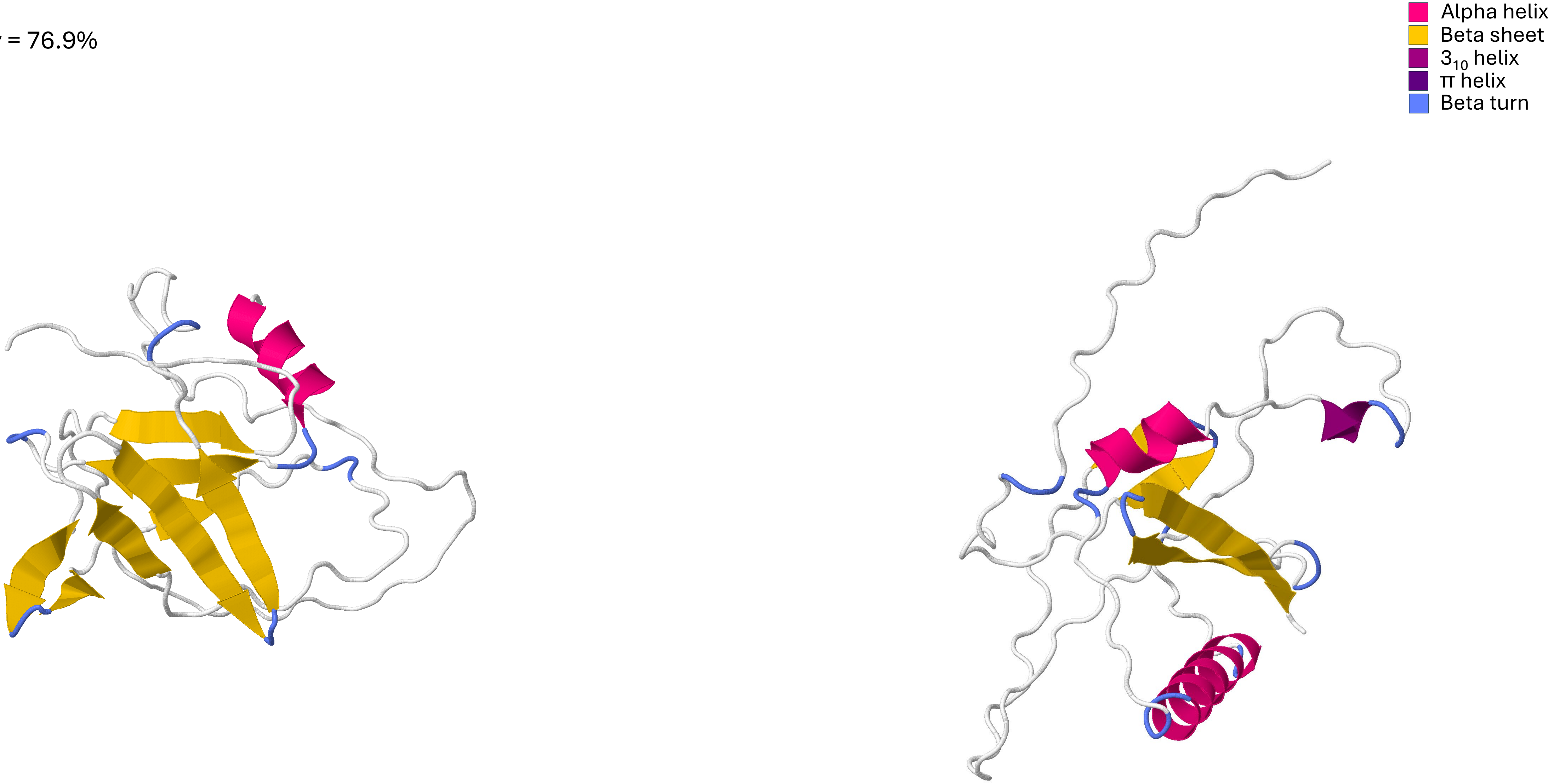

MdA-1
(UFQ21634)

MnA-1
(XQZ12370)

RepE1

Pairwise Identity = 78.9%

- Alpha helix
- Beta sheet
- 3_{10} helix
- π helix
- Beta turn

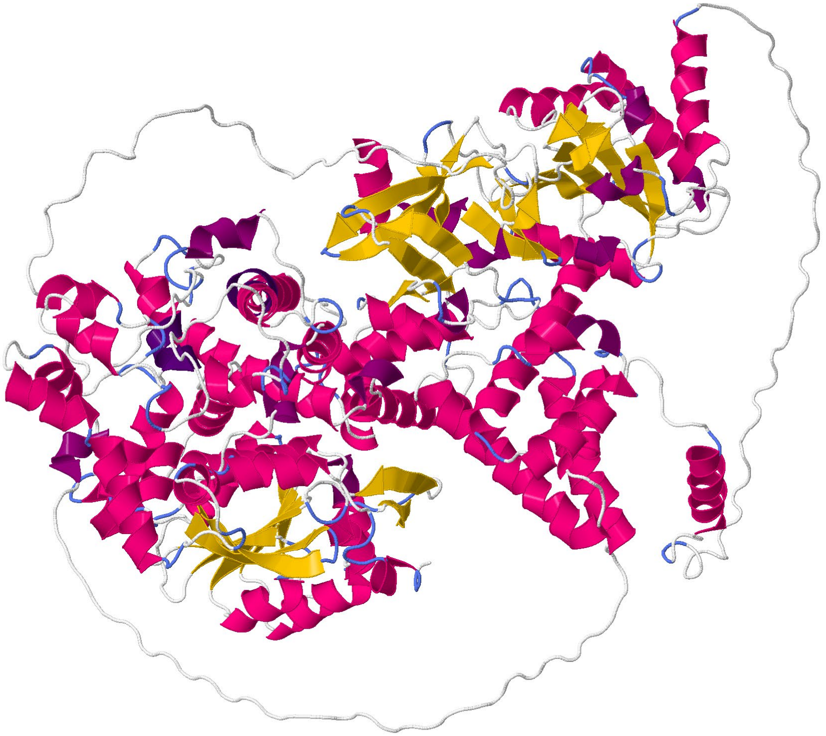

MdA-1
(UFQ21635)

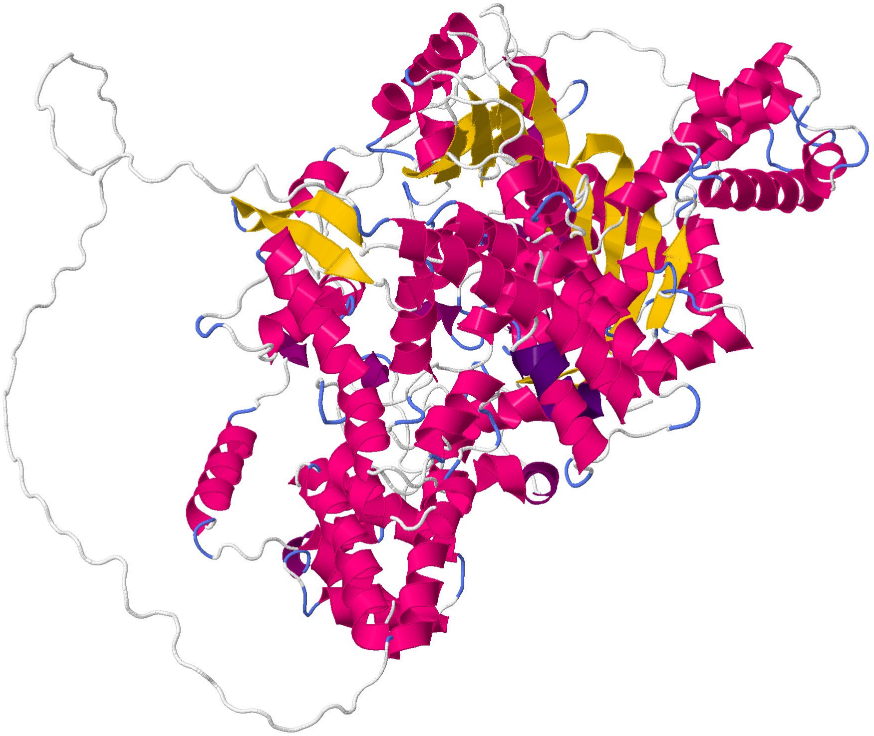

MnA-1
(XQZ12371)

SET

Pairwise Identity = 62.0%

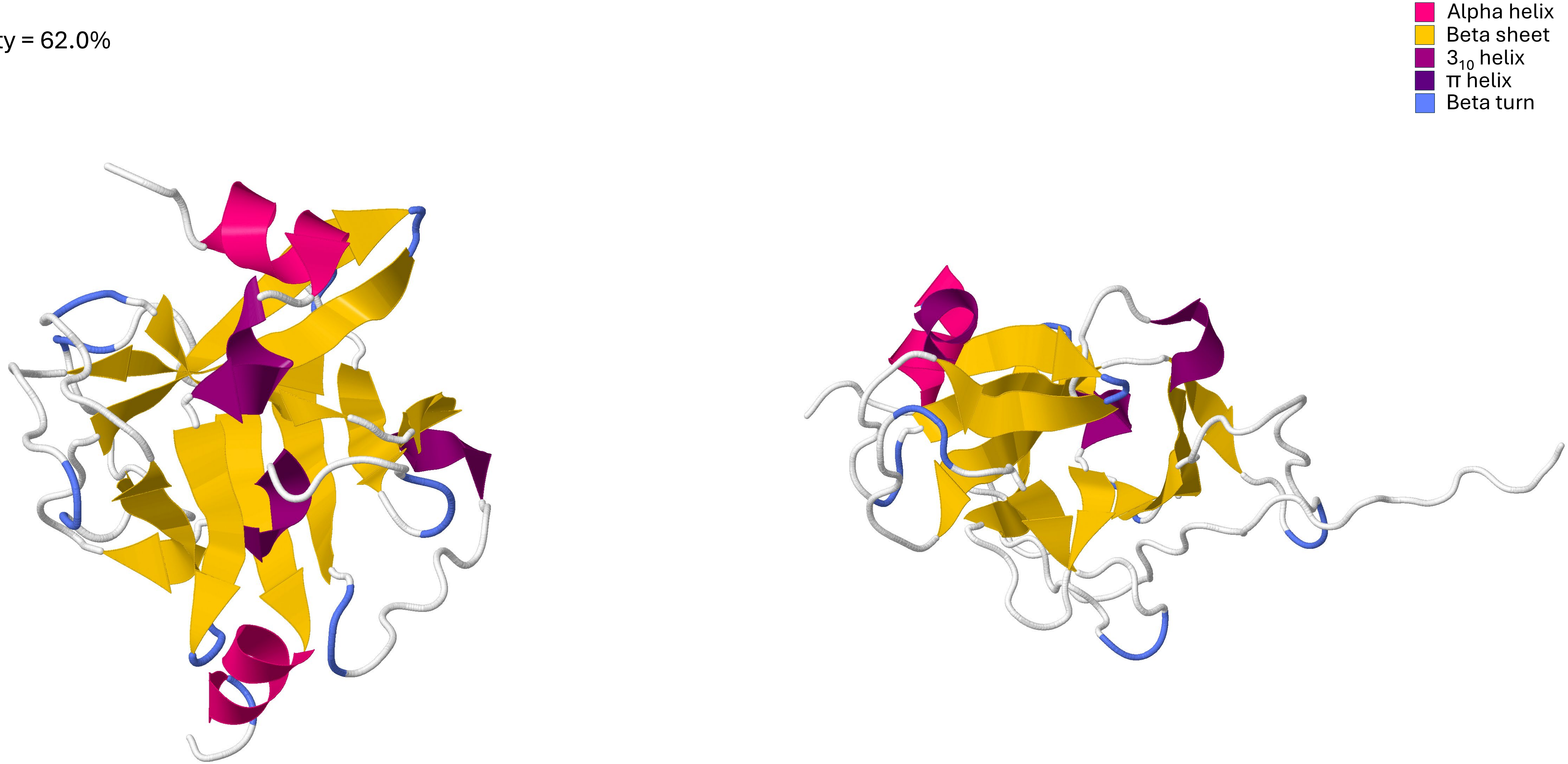

MdA-1 (UFQ21636)

MnA-1 (XQZ12374)

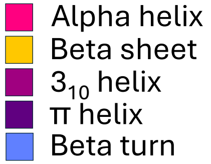

Zifi

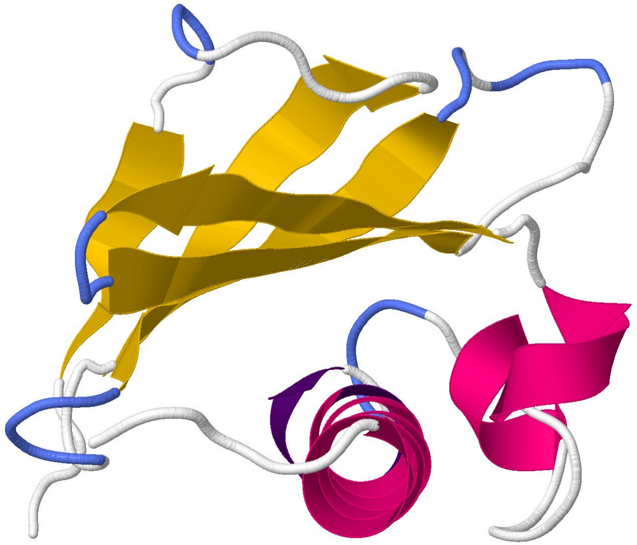

MnA-1
(XQZ12360)

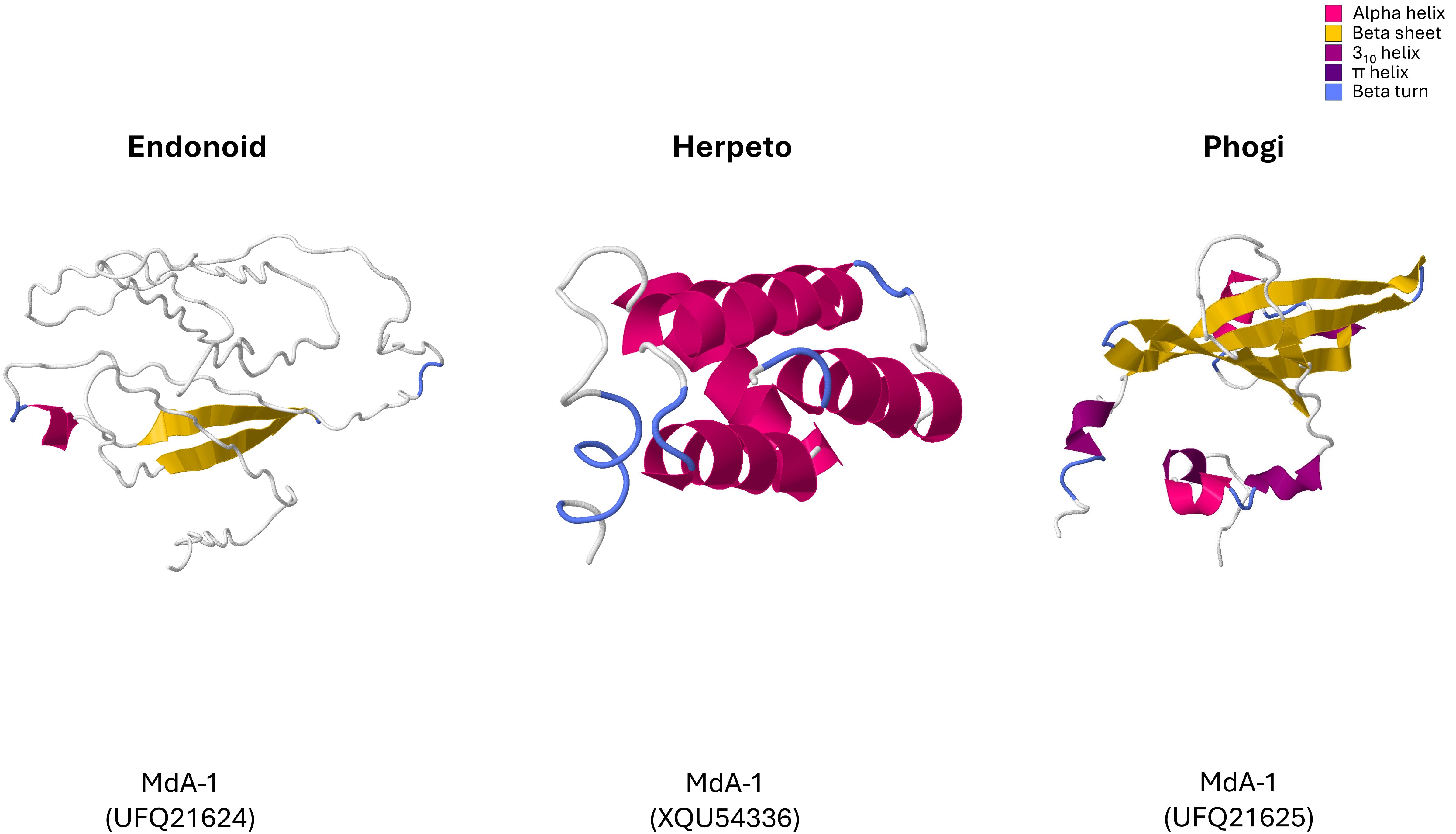
Alpha helix
Beta sheet
3_{10} helix
π helix
Beta turn
Endonoid
Herpeto
Phogi
MdA-1
(UFQ21624)
MdA-1
(XQU54336)
MdA-1
(UFQ21625)
