# Supplementary material for: Novel Adomaviruses Associated with Blotchy Bass Syndrome in Black Basses (Micropterus spp.)
Source: bioRxiv. 2025 Jun 5:2025.06.01.657292. Preprint. [Version 2] doi: 10.1101/2025.06.01.657292 (PMC12478380; doi:10.1101/2025.06.01.657292)

Supplemental Figure 7: MAFFT alignment of two MdA-1 genomes originating from different Pennsylvania rivers sampled during different years. SNPs are indicated with vertical lines. Open reading frames are identified.

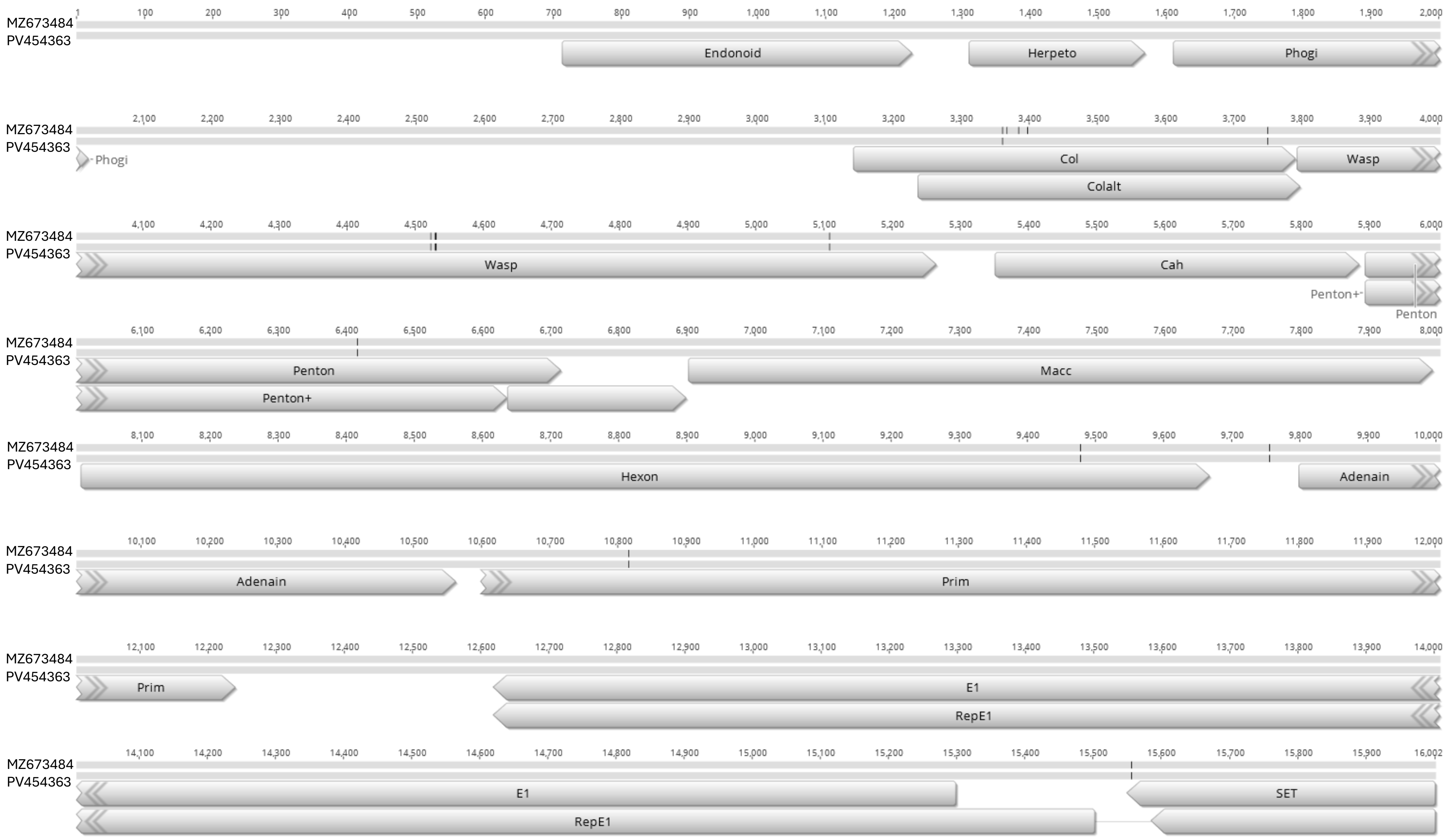

Supplement: Supplement 7 [file media-7.pdf]
