# Supplementary material for: Novel Adomaviruses Associated with Blotchy Bass Syndrome in Black Basses (Micropterus spp.)
Source: bioRxiv. 2025 Jun 5:2025.06.01.657292. Preprint. [Version 2] doi: 10.1101/2025.06.01.657292 (PMC12478380; doi:10.1101/2025.06.01.657292)

Supplemental Figure 8: MAFFT genome alignment of adomaviruses in the same phylogenetic clade as MdA-1 and MnA-1. Core adomavirus ORFs are colored in non-gray. Identity graph depicts similarity/dissimilarity across the genomes (sliding window = 1).

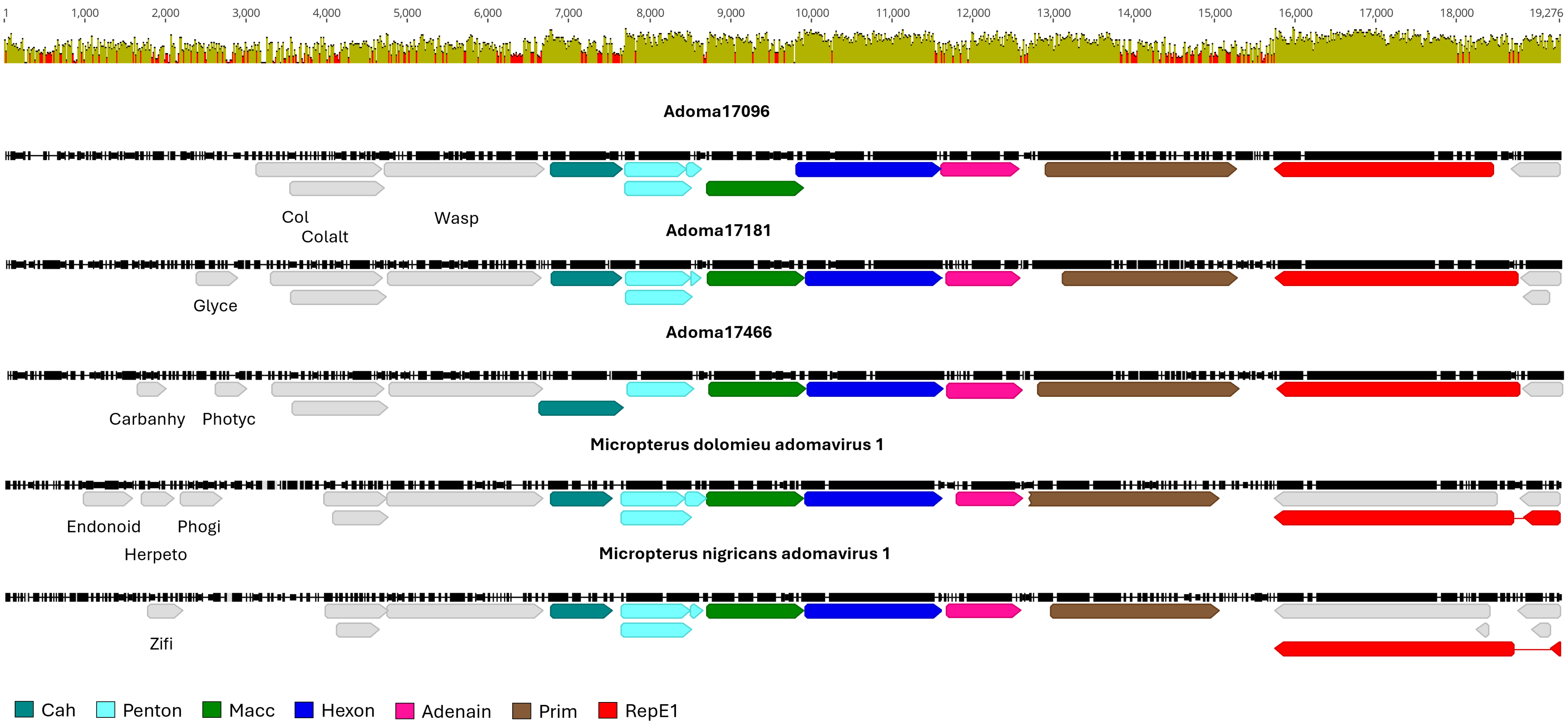

Supplement: Supplement 8 [file media-8.pdf]
