# Supplementary material for: Novel Adomaviruses Associated with Blotchy Bass Syndrome in Black Basses (Micropterus spp.)
Source: bioRxiv. 2025 Jun 5:2025.06.01.657292. Preprint. [Version 2] doi: 10.1101/2025.06.01.657292 (PMC12478380; doi:10.1101/2025.06.01.657292)
Supplement: Supplement 11 [file media-11.pdf]

Virus	Protein	Best hit	Name	Probablility	E-Value	Score	Target Length
MdA-1	Prim	6RB4_A	DNA primase small subunit; Primase, DNA-dependent RNA polymerase, ATP, priming, replication; HET: EDO; 1.5A {Homo sapien	100	1.4-66	541.6	410
MdA-1	RepE1	P26543	VE1_HPV58 Replication protein E1 OS=Human papillomavirus 58 OX=10598 GN=E1 PE=3 SV=1	100	1.7-58	547.5	644
MnA-1	Prim	6RB4_A	DNA primase small subunit; Primase, DNA-dependent RNA polymerase, ATP, priming, replication; HET: EDO; 1.5A	100	1.40E-62	515.8	410
MnA-1	RepE1	P26543	VE1_HPV58 Replication protein E1 OS=Human papillomavirus 58 OX=10598 GN=E1 PE=3 SV=1	100	1.30E-65	602.7	644
MdA-1	Adenain	4EKF_A	Adenain; alpha and beta protein (a+b), Hydrolase; HET: CSD; 0.98A {Human adenovirus 2} SCOP: d.3.1.7	99.96	1.50E-28	157	204
MnA-1	Adenain	P19119	PRO_ADEB3 Protease OS=Bovine adenovirus B serotype 3 OX=10510 GN=L3 PE=3 SV=1	99.95	1.60E-27	214	204
MnA-1	SET	9EH2_I	Histone-lysine N-methyltransferase SETD2; SETD2, Transcription, H3K36me3, TRANSFERASE-RNA-DNA complex; HET: TPO, SEP; 3.	99.44	1.50E-12	90.57	1133
MdA-1	SET	cd10545	SET_AtSUVH-like; SET domain found in Arabidopsis thaliana histone H3-K9 methyltransferases (SUVHs) and similar proteins.	99.41	5.00E-11	68.52	236
MnA-1	Penton	Q5UQU9	YL356_MIMIV Uncharacterized protein L356 OS=Acanthamoeba polyphaga mimivirus OX=212035 GN=MIMI_L356 PE=4 SV=1	99.36	1.40E-10	117.4	621
MdA-1	Penton	Q5UQU9	P1v1; giant virus, nucleocytoplasmic large DNA viruses (NCLDVs), viral assembly, Paramecium bursaria chlorella virus 1	98.72	1.10E-06	91.48	621
MdA-1	Hexon	PF21738.1	DJR_capsid ; Double jelly roll capsid-like protein	97.35	0.045	57.29	316
MnA-1	Col	PF13717.11	zinc_ribbon_4; zinc-ribbon domain	97.2	0.00016	0.3	37
MnA-1	Hexon	PF21738.1	DJR_capsid ; Double jelly roll capsid-like protein	97.11	0.1	54.79	316
MnA-1	Macc	PF05829.16	Adeno_PX ; Adenovirus late L2 mu core protein (Protein X)	97.1	0.0014	49.75	42
MdA-1	Wasp	PF02514.20	CobN-Mg_chel ; CobN/Magnesium Chelatase	96.46	0.0031	71.18	1189
MdA-1	Macc	PF05829.16	Adeno_PX ; Adenovirus late L2 mu core protein (Protein X)	96.14	0.019	44.2	42
MnA-1	Cah	P04507	SIGM1_REOVJ Outer capsid protein sigma-1 OS=Reovirus type 2 (strain D5/Jones) OX=10885 GN=S1 PE=3 SV=3	91.58	13	35.5	462
MdA-1	Cah	P04507	SIGM1_REOVJ Outer capsid protein sigma-1 OS=Reovirus type 2 (strain D5/Jones) OX=10885 GN=S1 PE=3 SV=3	90.5	26	33.57	462
MdA-1	Phogi	cd05016	SIS_PGI_2; Phosphoglucose isomerase (PGI) contains two SIS (Sugar ISomerase) domains.	60.54	20	24.22	192
MnA-1	Zifi	PF14608.10	zf-CCCH_2 ; RNA-binding, Nab2-type zinc finger	54.44	9.2	18.61	18
MnA-1	Wasp	PF14357.10	DUF4404 ; Domain of unknown function (DUF4404)	51.82	26	28.47	85
MnA-1	Colalt	7TOP_PR	PR20; Ribosomal binding peptide, ALS/FTD-associated dipeptide repeat protein, RIBOSOME; 2.4A {Saccharomyces cerevisiae}	48.29	100	21.11	40
MdA-1	Herpeto	PF14409.11	Herpeto_peptide ; Ribosomally synthesized peptide in Herpetosiphon	36.76	32	23.38	62
MdA-1	Col	2KLZ_A	Ataxin-3; UIM, Ataxin-3, Ubiquitin-binding, Hydrolase, Neurodegeneration, Nucleus, Phosphoprotein, Spinocerebellar ataxi	27.89	110	17.3	52
MdA-1	Endonoid	PF17785.5	PUA_3 ; PUA-like domain	27.53	41	19.62	64
MdA-1	Colalt	8Q7N_T	Transcription elongation regulator 1; spliceosome, pre-catalytic spliceosome, spliceosomal B complex, SPLICING; 3.1A	7.74	810	23.03	1095
