# Supplementary material for: Novel Adomaviruses Associated with Blotchy Bass Syndrome in Black Basses (Micropterus spp.)
Source: bioRxiv. 2025 Jun 5:2025.06.01.657292. Preprint. [Version 2] doi: 10.1101/2025.06.01.657292 (PMC12478380; doi:10.1101/2025.06.01.657292)
Supplement: Supplement 12 [file media-12.pdf]

DALI results: MdA-1 Endonoid

No:	Chain	Z	rmsd	lali	nres	%id	PDB Description
1:00	s001-A	22.3	20.5	170	0	0	NA

DALI results: MdA-1 Herpeto

No:	*Chain*	*Z*	*rmsd*	*lali*	*nres*	*%id*	*PDB*	*Description*
1:00	7ok0-C	6	2	55	393	5	MOLECULE:	DNA-DIRECTED RNA POLYMERASE SUBUNIT A'
2:00	7oqy-C	5.8	2	54	383	6	MOLECULE:	DNA-DIRECTED RNA POLYMERASE SUBUNIT A'
3:00	8p2i-C	5.7	2	52	390	8	MOLECULE:	DNA-DIRECTED RNA POLYMERASE SUBUNIT RPO1N
4:00	8cro-C	5.6	1.9	52	390	8	MOLECULE:	DNA-DIRECTED RNA POLYMERASE SUBUNIT RPO1N
5:00	7oq4-C	5.5	1.9	54	393	6	MOLECULE:	DNA-DIRECTED RNA POLYMERASE SUBUNIT A'
6:00	4ayb-C	5.4	1.7	52	376	12	MOLECULE:	DNA-DIRECTED RNA POLYMERASE
7:00	8rbo-C	5.4	2	53	390	8	MOLECULE:	DNA-DIRECTED RNA POLYMERASE SUBUNIT RPO1N
8:00	8orq-C	5.3	2	52	388	8	MOLECULE:	DNA-DIRECTED RNA POLYMERASE SUBUNIT RPO1N
9:00	6kf9-C	5	2	51	388	12	MOLECULE:	DNA-DIRECTED RNA POLYMERASE SUBUNIT
10:00	6kf3-C	5	1.9	51	388	12	MOLECULE:	DNA-DIRECTED RNA POLYMERASE SUBUNIT
11:00	8oki-C	5	1.9	52	390	8	MOLECULE:	DNA-DIRECTED RNA POLYMERASE SUBUNIT RPO1N
12:00	2pmz-G	5	1.9	51	279	4	MOLECULE:	DNA-DIRECTED RNA POLYMERASE SUBUNIT A
13:00	1k30-A	5	1.9	54	363	9	MOLECULE:	GLYCEROL-3-PHOSPHATE ACYLTRANSFERASE
14:00	6kf4-C	4.9	2.1	51	388	12	MOLECULE:	DNA-DIRECTED RNA POLYMERASE SUBUNIT
15:00	6swr-E	4.9	2.1	55	222	11	MOLECULE:	NANOBODY, MALTOSE/MALTODEXTRIN-BINDING PERIPLASMI
16:00	4qiw-C	4.8	1.7	47	369	13	MOLECULE:	DNA-DIRECTED RNA POLYMERASE
17:00	1iuq-A	4.8	1.9	54	357	9	MOLECULE:	GLYCEROL-3-PHOSPHATE ACYLTRANSFERASE
18:00	7jgs-C	4.7	3.3	60	614	13	MOLECULE:	ORIGIN RECOGNITION COMPLEX SUBUNIT 2
19:00	3hkz-M	4.7	1.8	51	370	4	MOLECULE:	DNA-DIRECTED RNA POLYMERASE SUBUNIT A'
20:00	2px0-B	4.7	3.3	54	258	11	MOLECULE:	FLAGELLAR BIOSYNTHESIS PROTEIN FLHF
21:00	2waq-C	4.6	1.8	49	367	6	MOLECULE:	DNA-DIRECTED RNA POLYMERASE RPO1N SUBUNIT
22:00	2y0s-Y	4.6	1.9	49	368	8	MOLECULE:	DNA-DIRECTED RNA POLYMERASE
23:00	2y0s-C	4.6	1.9	49	368	8	MOLECULE:	DNA-DIRECTED RNA POLYMERASE
24:00:00	8vej-A	4.6	2.2	54	240	7	MOLECULE:	CHD_BUTTRESS
25:00:00	6hd8-B	4.5	2.1	55	232	11	MOLECULE:	NANOBODY,MALTOSE/MALTODEXTRIN-BINDING PERIPLASMIC
26:00:00	6hd9-B	4.5	2.1	55	232	11	MOLECULE:	NANOBODY,MALTOSE/MALTODEXTRIN-BINDING PERIPLASMIC
27:00:00	2wb1-C	4.5	1.8	49	367	6	MOLECULE:	DNA-DIRECTED RNA POLYMERASE RPO1N SUBUNIT
28:00:00	6xj1-A	4.5	3.2	58	266	3	MOLECULE:	CELL DIVISION CONTROL PROTEIN 15
29:00:00	2pmz-C	4.5	1.9	51	279	4	MOLECULE:	DNA-DIRECTED RNA POLYMERASE SUBUNIT A
30:00:00	4qiw-M	4.5	1.7	47	369	13	MOLECULE:	DNA-DIRECTED RNA POLYMERASE
31:00:00	8v2d-c	4.5	3.1	75	314	5	MOLECULE:	O43_129 COMPONENT B
32:00:00	8v3b-V	4.5	3.1	75	314	5	MOLECULE:	O43_129_+4 COMPONENT A
33:00:00	8v3b-I	4.5	3.2	77	314	5	MOLECULE:	O43_129_+4 COMPONENT A
34:00:00	6hdc-B	4.5	2.1	55	232	11	MOLECULE:	NANOBODY,MALTOSE/MALTODEXTRIN-BINDING PERIPLASMIC
35:00:00	6swr-B	4.5	2.1	55	230	11	MOLECULE:	NANOBODY, MALTOSE/MALTODEXTRIN-BINDING PERIPLASMI
36:00:00	2px0-C	4.5	3.3	54	258	11	MOLECULE:	FLAGELLAR BIOSYNTHESIS PROTEIN FLHF
37:00:00	7yh8-A	4.5	2.6	53	62	11	MOLECULE:	L-19437
38:00:00	3adb-B	4.5	3	57	247	12	MOLECULE:	L-SERYL-TRNA(SEC) KINASE
39:00:00	7yh8-C	4.5	2.6	53	62	11	MOLECULE:	L-19437
40:00:00	1nxh-A	4.5	2.5	57	124	7	MOLECULE:	MTH396 PROTEIN
41:00:00	1nxh-B	4.5	2.5	57	118	7	MOLECULE:	MTH396 PROTEIN
42:00:00	8v2d-k	4.4	3.1	75	314	5	MOLECULE:	O43_129 COMPONENT B
43:00:00	8v2d-Z	4.4	3	75	314	5	MOLECULE:	O43_129 COMPONENT B
44:00:00	3syn-C	4.4	3.3	54	258	11	MOLECULE:	FLAGELLAR BIOSYNTHESIS PROTEIN FLHF
45:00:00	3syn-D	4.4	3.3	54	258	11	MOLECULE:	FLAGELLAR BIOSYNTHESIS PROTEIN FLHF
46:00:00	3syn-B	4.4	3.3	54	258	11	MOLECULE:	FLAGELLAR BIOSYNTHESIS PROTEIN FLHF
47:00:00	3syn-A	4.4	3.3	54	258	11	MOLECULE:	FLAGELLAR BIOSYNTHESIS PROTEIN FLHF
48:00:00	7jk3-C	4.4	3.1	60	614	10	MOLECULE:	ORIGIN RECOGNITION COMPLEX SUBUNIT 2
49:00:00	7bve-A	4.4	2.4	58	1034	5	MOLECULE:	INTEGRAL MEMBRANE INDOLYLACETYLINOSITOL
50:00:00	8v3b-U	4.4	3.5	62	314	10	MOLECULE:	O43_129_+4 COMPONENT A
51:00:00	8v2d-f	4.4	3.5	69	314	1	MOLECULE:	O43_129 COMPONENT B
52:00:00	8v3b-L	4.4	2.9	59	314	5	MOLECULE:	O43_129_+4 COMPONENT A
53:00:00	7jzm-A	4.3	3.8	55	64	13	MOLECULE:	LCB3
54:00:00	2px0-A	4.3	3.3	54	258	11	MOLECULE:	FLAGELLAR BIOSYNTHESIS PROTEIN FLHF

55:00:00	3add-A	4.3	3.5	57	251	5	MOLECULE:	L-SERYL-TRNA(SEC) KINASE
56:00:00	2wb1-Y	4.3	1.8	49	367	6	MOLECULE:	DNA-DIRECTED RNA POLYMERASE RPO1N SUBUNIT
57:00:00	8v3b-R	4.3	3.1	75	314	5	MOLECULE:	O43_129_+4 COMPONENT A
58:00:00	1r4g-A	4.3	2.5	49	53	6	MOLECULE:	RNA POLYMERASE ALPHA SUBUNIT
59:00:00	8v3b-C	4.3	3.1	75	314	5	MOLECULE:	O43_129_+4 COMPONENT A
60:00:00	8v2d-X	4.3	3.1	76	314	5	MOLECULE:	O43_129 COMPONENT B
61:00:00	2px0-F	4.3	3.4	54	258	11	MOLECULE:	FLAGELLAR BIOSYNTHESIS PROTEIN FLHF
62:00:00	3add-B	4.3	2.8	52	239	12	MOLECULE:	L-SERYL-TRNA(SEC) KINASE
63:00:00	7ctg-C	4.3	3.5	61	592	7	MOLECULE:	ORIGIN RECOGNITION COMPLEX SUBUNIT 1
64:00:00	3a4m-A	4.3	3.6	68	236	9	MOLECULE:	L-SERYL-TRNA(SEC) KINASE
65:00:00	8v3b-H	4.3	3.7	65	314	9	MOLECULE:	O43_129_+4 COMPONENT A
66:00:00	7upq-G	4.3	3.9	74	198	3	MOLECULE:	DHT03 PROTEIN A
67:00:00	8v3b-J	4.3	3.3	60	314	5	MOLECULE:	O43_129_+4 COMPONENT A
68:00:00	8fbi-A	4.3	3.1	64	274	8	MOLECULE:	KWOCA_39
69:00:00	3a4m-B	4.3	3.8	68	236	12	MOLECULE:	L-SERYL-TRNA(SEC) KINASE
70:00:00	8vej-B	4.3	2.3	54	242	7	MOLECULE:	CHD_BUTTRESS
71:00:00	7jk2-C	4.3	2.7	56	609	11	MOLECULE:	ORIGIN RECOGNITION COMPLEX SUBUNIT 2
72:00:00	8v3b-W	4.2	3.1	76	314	5	MOLECULE:	O43_129_+4 COMPONENT A
73:00:00	8v2d-g	4.2	3.1	75	314	5	MOLECULE:	O43_129 COMPONENT B
74:00:00	4xgc-C	4.2	3.2	61	567	11	MOLECULE:	ORIGIN RECOGNITION COMPLEX SUBUNIT 2
75:00:00	3adc-A	4.2	2.8	53	246	11	MOLECULE:	L-SERYL-TRNA(SEC) KINASE
76:00:00	2px0-E	4.2	3.2	53	258	11	MOLECULE:	FLAGELLAR BIOSYNTHESIS PROTEIN FLHF
77:00:00	8v3b-X	4.2	3.7	63	314	10	MOLECULE:	O43_129_+4 COMPONENT A
78:00:00	8v3b-P	4.2	3.8	64	314	9	MOLECULE:	O43_129_+4 COMPONENT A
79:00:00	8v2d-m	4.2	2.9	59	176	12	MOLECULE:	O43_129 COMPONENT B
80:00:00	8v3b-T	4.2	2.7	56	314	5	MOLECULE:	O43_129_+4 COMPONENT A
81:00:00	3a4n-A	4.2	3.4	66	237	11	MOLECULE:	L-SERYL-TRNA(SEC) KINASE
82:00:00	8v3b-N	4.2	3.5	61	314	11	MOLECULE:	O43_129_+4 COMPONENT A
83:00:00	8v3b-K	4.1	3.2	76	314	5	MOLECULE:	O43_129_+4 COMPONENT A
84:00:00	8v2d-a	4.1	3.2	75	314	5	MOLECULE:	O43_129 COMPONENT B
85:00:00	2px3-A	4.1	3.2	54	256	11	MOLECULE:	FLAGELLAR BIOSYNTHESIS PROTEIN FLHF
86:00:00	5uj8-C	4.1	3.1	62	553	6	MOLECULE:	ORIGIN RECOGNITION COMPLEX SUBUNIT 3
87:00:00	7bvf-B	4.1	2.3	55	1054	4	MOLECULE:	PROBABLE ARABINOSYLTRANSFERASE B
88:00:00	8v2d-b	4.1	3.1	77	314	6	MOLECULE:	O43_129 COMPONENT B
89:00:00	8v3b-S	4.1	3.2	78	314	6	MOLECULE:	O43_129_+4 COMPONENT A
90:00:00	3am1-A	4.1	2.8	52	236	12	MOLECULE:	L-SERYL-TRNA(SEC) KINASE
91:00:00	3adc-B	4.1	2.8	51	239	12	MOLECULE:	L-SERYL-TRNA(SEC) KINASE
92:00:00	8v3b-G	4.1	3.8	65	314	9	MOLECULE:	O43_129_+4 COMPONENT A
93:00:00	8v2d-e	4.1	2.8	55	314	5	MOLECULE:	O43_129 COMPONENT B
94:00:00	3vr5-A	4.1	3.3	67	588	9	MOLECULE:	V-TYPE SODIUM ATPASE CATALYTIC SUBUNIT A
95:00:00	7tmp-C	4.1	4.5	66	592	5	MOLECULE:	H(+)-TRANSPORTING TWO-SECTOR ATPASE
96:00:00	3vr4-A	4.1	3.1	69	588	10	MOLECULE:	V-TYPE SODIUM ATPASE CATALYTIC SUBUNIT A
97:00:00	8v2d-J	4.1	3.6	64	314	9	MOLECULE:	O43_129 COMPONENT B
98:00:00	8v3b-M	4.1	2.9	64	314	2	MOLECULE:	O43_129_+4 COMPONENT A
99:00:00	8v3b-O	4.1	3.5	63	314	10	MOLECULE:	O43_129_+4 COMPONENT A
0:00	4p1w-C	4	3.9	58	399	10	MOLECULE:	ATG29
1:00	7jzn-E	4	3.7	54	64	13	MOLECULE:	SPIKE GLYCOPROTEIN
2:00	6vmt-A	4	3.1	64	99	9	MOLECULE:	PROGRAMMED CELL DEATH PROTEIN 5 HOMOLOG
3:00	7jzn-F	4	3.8	54	64	13	MOLECULE:	SPIKE GLYCOPROTEIN
4:00	8v3b-F	4	3.1	75	314	5	MOLECULE:	O43_129_+4 COMPONENT A
5:00	8v2d-h	4	3.1	75	314	5	MOLECULE:	O43_129 COMPONENT B
6:00	1vdz-A	4	3	70	513	10	MOLECULE:	A-TYPE ATPASE SUBUNIT A
7:00	2v3c-C	4	2.7	56	403	9	MOLECULE:	SIGNAL RECOGNITION PARTICLE 19 KDA PROTEIN
8:00	8ia1-A	4	2.2	54	361	6	MOLECULE:	GLYCEROL-3-PHOSPHATE ACYLTRANSFERASE, CHLOROPLAST
9:00	8ia1-B	4	2.2	54	361	6	MOLECULE:	GLYCEROL-3-PHOSPHATE ACYLTRANSFERASE, CHLOROPLAST
10:00	1j8m-F	4	2.5	55	295	4	MOLECULE:	SIGNAL RECOGNITION 54 KDA PROTEIN

11:00	2px0-G	4	3.2	54	258	11	MOLECULE:	FLAGELLAR BIOSYNTHESIS PROTEIN FLHF
12:00	6x0o-A	4	2.8	58	1037	3	MOLECULE:	INTEGRAL MEMBRANE INDOLYLACETYLINOSITOL
13:00	6ty9-A	4	3.8	76	1208	7	MOLECULE:	RNA-DEPENDENT RNA POLYMERASE
14:00	3a4l-A	4	3.6	67	236	9	MOLECULE:	L-SERYL-TRNA(SEC) KINASE
15:00	1oks-A	4	2.1	48	53	8	MOLECULE:	RNA POLYMERASE ALPHA SUBUNIT
16:00	3a4l-B	4	3.7	68	237	9	MOLECULE:	L-SERYL-TRNA(SEC) KINASE
17:00	2px0-D	4	3.4	54	258	11	MOLECULE:	FLAGELLAR BIOSYNTHESIS PROTEIN FLHF
18:00	3a4n-B	4	3.4	66	237	12	MOLECULE:	L-SERYL-TRNA(SEC) KINASE
19:00	7bvf-A	4	2.7	57	1087	9	MOLECULE:	PROBABLE ARABINOSYLTRANSFERASE B
20:00	7tmr-A	4	2.9	64	593	8	MOLECULE:	H(+)-TRANSPORTING TWO-SECTOR ATPASE
21:00	7c2n-A	4	2.8	54	900	7	MOLECULE:	DRUG EXPORTERS OF THE RND SUPERFAMILY-LIKE PROTEI
22:00	8v2d-Y	4	3.4	62	314	10	MOLECULE:	O43_129 COMPONENT B
23:00	8v3b-B	4	2.9	64	314	2	MOLECULE:	O43_129_+4 COMPONENT A
24:00:00	7cos-I	4	3	65	583	6	MOLECULE:	V-TYPE SODIUM ATPASE CATALYTIC SUBUNIT A
25:00:00	3hkz-C	3.9	2.1	51	370	6	MOLECULE:	DNA-DIRECTED RNA POLYMERASE SUBUNIT A'
26:00:00	8v3b-D	3.9	3.2	75	314	5	MOLECULE:	O43_129_+4 COMPONENT A
27:00:00	8v2d-l	3.9	3.2	76	314	5	MOLECULE:	O43_129 COMPONENT B
28:00:00	5mj4-C	3.9	4	56	81	2	MOLECULE:	INTERLEUKIN-12 SUBUNIT BETA
29:00:00	5lxj-A	3.9	3.4	51	53	6	MOLECULE:	PHOSPHOPROTEIN
30:00:00	6em5-R	3.9	3.3	55	120	7	MOLECULE:	5.8S RIBOSOMAL RNA
31:00:00	4og9-C	3.9	4	56	81	2	MOLECULE:	INTERLEUKIN-12 SUBUNIT BETA
32:00:00	7tcd-A	3.9	3.4	66	436	11	MOLECULE:	D13 LOV2-DARPIN FUSION
33:00:00	3adb-A	3.9	2.7	51	250	12	MOLECULE:	L-SERYL-TRNA(SEC) KINASE
34:00:00	7coq-A	3.9	3	66	586	9	MOLECULE:	V-TYPE SODIUM ATPASE CATALYTIC SUBUNIT A
35:00:00	8v2d-j	3.9	3.4	64	314	0	MOLECULE:	O43_129 COMPONENT B
36:00:00	7upq-J	3.9	3.6	75	197	4	MOLECULE:	DHT03 PROTEIN A
37:00:00	7cop-B	3.9	2.8	59	586	5	MOLECULE:	V-TYPE SODIUM ATPASE CATALYTIC SUBUNIT A
38:00:00	7qps-A	3.9	2.3	54	322	6	MOLECULE:	ACT DOMAIN PROTEIN
39:00:00	7bv1-C	3.8	3.7	53	63	4	MOLECULE:	NSP12
40:00:00	8ce8-b	3.8	3.4	70	219	11	MOLECULE:	CYTOCHROME C BIOGENESIS ATP-BINDING EXPORT PROTEI
41:00:00	4oe8-C	3.8	3.8	57	87	2	MOLECULE:	INTERLEUKIN-12 SUBUNIT BETA
42:00:00	6zr2-n	3.8	4.5	58	177	3	MOLECULE:	NADH-UBIQUINONE OXIDOREDUCTASE CHAIN 3
43:00:00	8kdb-B	3.8	2.5	51	65	12	MOLECULE:	RNA-DIRECTED RNA POLYMERASE L
44:00:00	8v9u-A	3.8	2.5	52	81	8	MOLECULE:	DNA (CYTOSINE-5)-METHYLTRANSFERASE 1
45:00:00	8kdc-B	3.8	2.6	51	65	12	MOLECULE:	RNA-DIRECTED RNA POLYMERASE L
46:00:00	8etc-R	3.8	3	51	124	6	MOLECULE:	RNA (2151-MER)
47:00:00	5f3p-B	3.8	4.1	60	188	10	MOLECULE:	PUTATIVE UNCHARACTERIZED PROTEIN
48:00:00	7jgr-C	3.8	3.1	59	612	8	MOLECULE:	ORIGIN RECOGNITION COMPLEX SUBUNIT 2
49:00:00	6s2v-C	3.8	2.8	58	336	3	MOLECULE:	(P)PPGPP SYNTHETASE I, SPOT/RELA
50:00:00	5vre-D	3.8	3	54	188	6	MOLECULE:	PUTATIVE INTEGRAL MEMBRANE PROTEIN
51:00:00	7dqd-K	3.8	4.2	61	584	7	MOLECULE:	V-TYPE SODIUM ATPASE CATALYTIC SUBUNIT A
52:00:00	5uj8-D	3.8	3.1	62	553	6	MOLECULE:	ORIGIN RECOGNITION COMPLEX SUBUNIT 3
53:00:00	6vq9-A	3.8	4.5	65	600	5	MOLECULE:	ATPASE H+-TRANSPORTING V1 SUBUNIT A
54:00:00	8rgr-n	3.8	4.4	56	178	4	MOLECULE:	NADH DEHYDROGENASE [UBIQUINONE] IRON-SULFUR PROTE
55:00:00	7jzn-G	3.8	3.8	54	64	13	MOLECULE:	SPIKE GLYCOPROTEIN
56:00:00	7w1t-p	3.8	4.5	58	178	3	MOLECULE:	NADH DEHYDROGENASE [UBIQUINONE] FLAVOPROTEIN 1,
57:00:00	5mj3-C	3.8	3.6	57	87	0	MOLECULE:	INTERLEUKIN-12 SUBUNIT BETA
58:00:00	7jpq-C	3.8	3.2	60	587	5	MOLECULE:	ORIGIN RECOGNITION COMPLEX SUBUNIT 2
59:00:00	8rgq-n	3.8	4.4	56	178	4	MOLECULE:	NADH DEHYDROGENASE [UBIQUINONE] IRON-SULFUR PROTE
60:00:00	8rgp-n	3.8	4.5	57	178	4	MOLECULE:	NADH DEHYDROGENASE [UBIQUINONE] IRON-SULFUR PROTE
61:00:00	8v3b-E	3.8	2	43	314	12	MOLECULE:	O43_129_+4 COMPONENT A
62:00:00	8v3b-A	3.8	2.1	43	314	12	MOLECULE:	O43_129_+4 COMPONENT A
63:00:00	7fde-C	3.8	2.8	66	594	8	MOLECULE:	V-TYPE PROTON ATPASE SUBUNIT C
64:00:00	5zea-H	3.8	3.1	67	586	9	MOLECULE:	V-TYPE SODIUM ATPASE CATALYTIC SUBUNIT A
65:00:00	7dqd-J	3.8	3.9	74	584	5	MOLECULE:	V-TYPE SODIUM ATPASE CATALYTIC SUBUNIT A
66:00:00	6qum-B	3.8	3	67	578	10	MOLECULE:	V-TYPE ATP SYNTHASE ALPHA CHAIN

67:00:00	6vq6-A	3.7	4.5	65	600	5	MOLECULE:	ATPASE H+-TRANSPORTING V1 SUBUNIT A
68:00:00	4d7x-A	3.7	2.2	50	86	6	MOLECULE:	MEDIATOR OF RNA POLYMERASE II TRANSCRIPTION SUBUN
69:00:00	6j73-A	3.7	3	53	539	6	MOLECULE:	ISONIAZID INDUCIBLE GENE PROTEIN INIA
70:00:00	6ajf-A	3.7	5.2	61	901	8	MOLECULE:	DRUG EXPORTERS OF THE RND SUPERFAMILY-LIKE PROTEI
71:00:00	7udl-A	3.7	3.2	56	278	9	MOLECULE:	DESIGNED HELICAL REPEAT PROTEIN (DHR) RPB_PLP1_R6
72:00:00	6dfk-G	3.7	2.7	55	213	0	MOLECULE:	SUBUNIT OF PROTEASEOME ACTIVATOR COMPLEX,PUTATIVE
73:00:00	6dfk-C	3.7	3.5	59	222	0	MOLECULE:	SUBUNIT OF PROTEASEOME ACTIVATOR COMPLEX,PUTATIVE
74:00:00	1t6o-A	3.7	2.1	46	49	9	MOLECULE:	PHOSPHOPROTEIN
75:00:00	7qvb-A	3.7	4.3	67	213	3	MOLECULE:	DNA DAMAGE RESPONSE PROTEIN C
76:00:00	8ovt-C	3.7	4.3	72	286	8	MOLECULE:	YEGT GLYCOSYLTRANSFERASE
77:00:00	3ctd-A	3.7	5.6	60	150	10	MOLECULE:	PUTATIVE ATPASE, AAA FAMILY
78:00:00	2qex-P	3.7	2.9	50	143	6	MOLECULE:	23S RIBOSOMAL RNA
79:00:00	7zd6-t	3.7	5.1	54	177	6	MOLECULE:	NADH-UBIQUINONE OXIDOREDUCTASE CHAIN 3
80:00:00	7w2y-p	3.7	4.5	57	178	4	MOLECULE:	NADH DEHYDROGENASE [UBIQUINONE] FLAVOPROTEIN 1,
81:00:00	7r44-n	3.7	3	52	172	6	MOLECULE:	NADH-UBIQUINONE OXIDOREDUCTASE CHAIN 3
82:00:00	7r4f-n	3.7	3	52	171	6	MOLECULE:	NADH-UBIQUINONE OXIDOREDUCTASE CHAIN 3
83:00:00	7w1u-p	3.7	4.4	58	178	3	MOLECULE:	NADH DEHYDROGENASE [UBIQUINONE] FLAVOPROTEIN 1,
84:00:00	7w2u-p	3.7	4.5	57	178	4	MOLECULE:	NADH DEHYDROGENASE [UBIQUINONE] FLAVOPROTEIN 1,
85:00:00	7w0y-p	3.7	4.5	58	178	3	MOLECULE:	NADH DEHYDROGENASE [UBIQUINONE] FLAVOPROTEIN 1,
86:00:00	7w1v-p	3.7	4.3	56	178	4	MOLECULE:	NADH DEHYDROGENASE [UBIQUINONE] FLAVOPROTEIN 1,
87:00:00	7r4g-n	3.7	3	52	171	6	MOLECULE:	NADH-UBIQUINONE OXIDOREDUCTASE CHAIN 3
88:00:00	7r46-n	3.7	3	52	171	6	MOLECULE:	NADH-UBIQUINONE OXIDOREDUCTASE CHAIN 3
89:00:00	7w1z-p	3.7	4.6	56	178	5	MOLECULE:	NADH DEHYDROGENASE [UBIQUINONE] FLAVOPROTEIN 1,
90:00:00	7v31-p	3.7	4.4	56	178	4	MOLECULE:	NADH DEHYDROGENASE [UBIQUINONE] FLAVOPROTEIN 1,
91:00:00	8ow0-N	3.7	2.6	50	391	14	MOLECULE:	C0N3 DNA
92:00:00	8q0o-n	3.7	3.1	53	171	6	MOLECULE:	NADH-UBIQUINONE OXIDOREDUCTASE CHAIN 3
93:00:00	7w4e-p	3.7	4.4	57	178	4	MOLECULE:	NADH DEHYDROGENASE [UBIQUINONE] FLAVOPROTEIN 1,
94:00:00	8ca3-n	3.7	4.5	57	177	4	MOLECULE:	NADH-UBIQUINONE OXIDOREDUCTASE CHAIN 3
95:00:00	7b93-n	3.7	4.5	57	178	4	MOLECULE:	NADH-UBIQUINONE OXIDOREDUCTASE CHAIN 3
96:00:00	7vyg-p	3.7	4.5	57	178	4	MOLECULE:	NADH DEHYDROGENASE [UBIQUINONE] IRON-SULFUR PROTE
97:00:00	5cbn-A	3.7	3.2	56	176	5	MOLECULE:	MALTOSE-BINDING PERIPLASMIC PROTEIN
98:00:00	7w2r-p	3.7	4.5	57	178	4	MOLECULE:	NADH DEHYDROGENASE [UBIQUINONE] FLAVOPROTEIN 1,
99:00:00	3x38-D	3.7	3.3	56	77	5	MOLECULE:	MITOCHONDRIAL MORPHOGENESIS PROTEIN SLD7
0:00	8v2d-d	3.7	3.5	61	314	8	MOLECULE:	O43_129 COMPONENT B
1:00	7bvg-A	3.7	2.6	56	1078	4	MOLECULE:	INTEGRAL MEMBRANE INDOLYLACETYLINOSITOL
2:00	7bvc-A	3.7	2.7	57	1078	5	MOLECULE:	INTEGRAL MEMBRANE INDOLYLACETYLINOSITOL
3:00	5bw9-B	3.7	3.7	65	594	6	MOLECULE:	V-TYPE PROTON ATPASE CATALYTIC SUBUNIT A
4:00	7bvc-B	3.7	3	60	1063	2	MOLECULE:	INTEGRAL MEMBRANE INDOLYLACETYLINOSITOL
5:00	7upp-A	3.7	3.8	80	312	5	MOLECULE:	DHT03 PROTEIN A
6:00	5bw9-b	3.7	3.7	65	589	6	MOLECULE:	V-TYPE PROTON ATPASE CATALYTIC SUBUNIT A
7:00	7fdb-C	3.7	3	63	594	8	MOLECULE:	YEAST VACUOLAR ATPASE A SUBUNIT
8:00	7qps-B	3.7	2.3	53	303	6	MOLECULE:	ACT DOMAIN PROTEIN
9:00	6r10-C	3.7	3.2	68	577	1	MOLECULE:	V-TYPE ATP SYNTHASE ALPHA CHAIN
10:00	8iwh-g	3.6	3.7	55	120	5	MOLECULE:	PHOTOSYSTEM II PROTEIN D1
11:00	2m6u-A	3.6	2.8	56	82	13	MOLECULE:	CHOLINE BINDING PROTEIN A
12:00	4k12-B	3.6	2.8	56	82	13	MOLECULE:	COMPLEMENT FACTOR H
13:00	7d4f-C	3.6	3.6	53	63	4	MOLECULE:	NON-STRUCTURAL PROTEIN 8
14:00	7nfu-B	3.6	3.4	63	213	10	MOLECULE:	NUCLEOID OCCLUSION PROTEIN
15:00	3x38-A	3.6	3.3	56	78	5	MOLECULE:	MITOCHONDRIAL MORPHOGENESIS PROTEIN SLD7
16:00	6t6v-A	3.6	2.5	52	742	6	MOLECULE:	NITRIC OXIDE REDUCTASE SUBUNIT B
17:00	8v2d-K	3.6	3.2	76	314	5	MOLECULE:	O43_129 COMPONENT B
18:00	8ap8-d	3.6	3	54	270	4	MOLECULE:	OSCP
19:00	7v2c-p	3.6	4.5	57	178	4	MOLECULE:	NADH DEHYDROGENASE [UBIQUINONE] FLAVOPROTEIN 1,
20:00	7ak6-n	3.6	4.5	57	176	4	MOLECULE:	NADH-UBIQUINONE OXIDOREDUCTASE CHAIN 3
21:00	1st6-A	3.6	3.9	59	1049	10	MOLECULE:	VINCULIN
22:00	5wlw-F	3.6	2.6	52	83	6	MOLECULE:	CYSTEINE DESULFURASE, MITOCHONDRIAL

23:00	6zkg-t	3.6	5	53	177	6	MOLECULE:	NADH DEHYDROGENASE [UBIQUINONE] FLAVOPROTEIN 1,
24:00:00	6o7w-A	3.6	4	62	593	3	MOLECULE:	V-TYPE PROTON ATPASE SUBUNIT D
25:00:00	3g71-P	3.6	3	50	143	4	MOLECULE:	23S RIBOSOMAL RNA
26:00:00	5uj8-A	3.6	3	60	553	7	MOLECULE:	ORIGIN RECOGNITION COMPLEX SUBUNIT 3
27:00:00	7qpr-C	3.6	3	57	678	7	MOLECULE:	ACT DOMAIN PROTEIN
28:00:00	2otj-P	3.6	2.9	50	143	6	MOLECULE:	23S RIBOSOMAL RNA
29:00:00	7coq-B	3.6	2.8	60	586	5	MOLECULE:	V-TYPE SODIUM ATPASE CATALYTIC SUBUNIT A
30:00:00	3ctd-B	3.6	3.6	56	158	11	MOLECULE:	PUTATIVE ATPASE, AAA FAMILY
31:00:00	7vc0-p	3.6	4.6	56	178	5	MOLECULE:	NADH DEHYDROGENASE [UBIQUINONE] IRON-SULFUR PROTE
32:00:00	7vzv-p	3.6	4.5	57	178	4	MOLECULE:	NADH DEHYDROGENASE [UBIQUINONE] FLAVOPROTEIN 1,
33:00:00	7r42-n	3.6	3	52	172	6	MOLECULE:	NADH-UBIQUINONE OXIDOREDUCTASE CHAIN 3
34:00:00	3ccm-P	3.6	3	50	143	4	MOLECULE:	50S RIBOSOMAL PROTEIN L2P
35:00:00	7vys-p	3.6	4.5	57	178	4	MOLECULE:	NADH DEHYDROGENASE [UBIQUINONE] IRON-SULFUR PROTE
36:00:00	7v2h-p	3.6	4.4	56	178	4	MOLECULE:	NADH DEHYDROGENASE [UBIQUINONE] FLAVOPROTEIN 1,
37:00:00	7r48-n	3.6	3	52	171	6	MOLECULE:	NADH-UBIQUINONE OXIDOREDUCTASE CHAIN 3
38:00:00	1yhq-P	3.6	2.9	50	143	6	MOLECULE:	23S RIBOSOMAL RNA
39:00:00	7r45-n	3.6	3	52	171	6	MOLECULE:	NADH-UBIQUINONE OXIDOREDUCTASE CHAIN 3
40:00:00	7w0r-p	3.6	4.4	56	178	4	MOLECULE:	NADH DEHYDROGENASE [UBIQUINONE] FLAVOPROTEIN 1,
41:00:00	3ccv-P	3.6	3	50	143	4	MOLECULE:	50S RIBOSOMAL PROTEIN L2P
42:00:00	7r4c-n	3.6	3	52	171	6	MOLECULE:	NADH-UBIQUINONE OXIDOREDUCTASE CHAIN 3
43:00:00	7vbp-p	3.6	4.5	58	178	3	MOLECULE:	NADH DEHYDROGENASE [UBIQUINONE] IRON-SULFUR PROTE
44:00:00	8q49-n	3.6	3	52	171	6	MOLECULE:	NADH-UBIQUINONE OXIDOREDUCTASE CHAIN 3
45:00:00	7zdm-t	3.6	5	54	177	6	MOLECULE:	NADH DEHYDROGENASE [UBIQUINONE] FLAVOPROTEIN 1,
46:00:00	8q0a-n	3.6	2.9	52	171	6	MOLECULE:	NADH-UBIQUINONE OXIDOREDUCTASE CHAIN 3
47:00:00	8rgt-n	3.6	4.5	56	178	4	MOLECULE:	NADH DEHYDROGENASE [UBIQUINONE] IRON-SULFUR PROTE
48:00:00	7r47-n	3.6	3	52	171	6	MOLECULE:	NADH-UBIQUINONE OXIDOREDUCTASE CHAIN 3
49:00:00	7w1p-p	3.6	4.5	57	178	4	MOLECULE:	NADH DEHYDROGENASE [UBIQUINONE] FLAVOPROTEIN 1,
50:00:00	8q46-n	3.6	3	52	171	6	MOLECULE:	NADH-UBIQUINONE OXIDOREDUCTASE CHAIN 3
51:00:00	1vql-P	3.6	3	50	143	4	MOLECULE:	23S RIBOSOMAL RNA
52:00:00	7w4g-p	3.6	4.6	57	178	5	MOLECULE:	NADH DEHYDROGENASE [UBIQUINONE] FLAVOPROTEIN 1,
53:00:00	7v33-p	3.6	4.5	57	178	4	MOLECULE:	NADH DEHYDROGENASE [UBIQUINONE] FLAVOPROTEIN 1,
54:00:00	7vxs-p	3.6	4.5	57	178	4	MOLECULE:	NADH DEHYDROGENASE [UBIQUINONE] IRON-SULFUR PROTE
55:00:00	8c2s-n	3.6	4.5	56	177	4	MOLECULE:	NADH-UBIQUINONE OXIDOREDUCTASE CHAIN 3
56:00:00	7r41-n	3.6	3	52	172	6	MOLECULE:	NADH-UBIQUINONE OXIDOREDUCTASE CHAIN 3
57:00:00	8om1-n	3.6	4.5	56	178	4	MOLECULE:	NADH-UBIQUINONE OXIDOREDUCTASE CHAIN 3
58:00:00	5f3o-A	3.6	3.9	56	194	7	MOLECULE:	PUTATIVE UNCHARACTERIZED PROTEIN
59:00:00	8q4a-n	3.6	3.2	53	171	6	MOLECULE:	NADH-UBIQUINONE OXIDOREDUCTASE CHAIN 3
60:00:00	8g28-B	3.6	3.6	56	104	9	MOLECULE:	ATPASE, AAA FAMILY
61:00:00	3x38-C	3.6	3.2	55	79	5	MOLECULE:	MITOCHONDRIAL MORPHOGENESIS PROTEIN SLD7
62:00:00	7qsm-n	3.6	3	52	171	6	MOLECULE:	NADH-UBIQUINONE OXIDOREDUCTASE CHAIN 3
63:00:00	8g28-A	3.6	3.7	56	108	9	MOLECULE:	ATPASE, AAA FAMILY
64:00:00	7bve-B	3.6	2.7	57	1034	9	MOLECULE:	INTEGRAL MEMBRANE INDOLYLACETYLINOSITOL
65:00:00	7zdh-t	3.6	5.1	54	177	6	MOLECULE:	NADH-UBIQUINONE OXIDOREDUCTASE CHAIN 3
66:00:00	7r4d-n	3.6	3	52	171	6	MOLECULE:	NADH-UBIQUINONE OXIDOREDUCTASE CHAIN 3
67:00:00	8ca5-n	3.6	4.4	58	177	3	MOLECULE:	NADH DEHYDROGENASE [UBIQUINONE] 1 ALPHA SUBCOMPLE
68:00:00	3i56-P	3.6	2.9	50	143	6	MOLECULE:	50S RIBOSOMAL PROTEIN L2P
69:00:00	7v2e-p	3.6	4.5	58	178	3	MOLECULE:	NADH DEHYDROGENASE [UBIQUINONE] FLAVOPROTEIN 1,
70:00:00	7tmr-E	3.6	3.5	69	593	7	MOLECULE:	H(+)-TRANSPORTING TWO-SECTOR ATPASE
71:00:00	3j9v-A	3.6	3.1	68	593	7	MOLECULE:	V-TYPE PROTON ATPASE SUBUNIT A, VACUOLAR ISOFORM
72:00:00	7qpr-A	3.6	2.6	56	687	5	MOLECULE:	ACT DOMAIN PROTEIN
73:00:00	5h78-A	3.6	9.1	56	192	7	MOLECULE:	CAMP-DEPENDENT PROTEIN KINASE TYPE II-ALPHA REGUL
74:00:00	8ovt-A	3.5	4.1	69	288	10	MOLECULE:	YEGT GLYCOSYLTRANSFERASE
75:00:00	8ovw-N	3.5	2.7	50	391	14	MOLECULE:	CENTROMERE-BINDING PROTEIN 1
76:00:00	7qpr-D	3.5	3	56	689	9	MOLECULE:	ACT DOMAIN PROTEIN
77:00:00	3x38-B	3.5	3.2	55	79	5	MOLECULE:	MITOCHONDRIAL MORPHOGENESIS PROTEIN SLD7
78:00:00	6hdb-B	3.5	2.3	56	232	11	MOLECULE:	NANOBODY,MALTOSE/MALTODEXTRIN-BINDING PERIPLASMIC

79:00:00	6r3q-A	3.5	3.5	56	841	11	MOLECULE:	ADENYLATE CYCLASE 9
80:00:00	7ose-A	3.5	3.3	55	503	9	MOLECULE:	CYTOCHROME BD-II UBIQUINOL OXIDASE SUBUNIT 1
81:00:00	6hda-B	3.5	3.1	58	232	10	MOLECULE:	NANOBODY,MALTOSE/MALTODEXTRIN-BINDING PERIPLASMIC
82:00:00	8ce8-B	3.5	3.7	70	219	10	MOLECULE:	CYTOCHROME C BIOGENESIS ATP-BINDING EXPORT PROTEI
83:00:00	2qw6-C	3.5	3.2	52	83	12	MOLECULE:	AAA ATPASE, CENTRAL REGION
84:00:00	7saz-A	3.5	3.7	54	217	6	MOLECULE:	GLDM
85:00:00	6dfk-L	3.5	2.6	55	233	0	MOLECULE:	SUBUNIT OF PROTEASEOME ACTIVATOR COMPLEX,PUTATIVE
86:00:00	1k73-Q	3.5	2.9	50	143	6	MOLECULE:	23S RRNA
87:00:00	1kd1-Q	3.5	3.1	50	143	4	MOLECULE:	23S RRNA
88:00:00	7bw4-C	3.5	4.1	57	65	2	MOLECULE:	REPLICASE POLYPROTEIN 1AB
89:00:00	7udj-H	3.5	3.4	64	192	6	MOLECULE:	4XPAW PEPTIDE
90:00:00	1q86-Q	3.5	2.9	50	143	6	MOLECULE:	23S RIBOSOMAL RNA
91:00:00	1vq7-P	3.5	2.9	50	143	6	MOLECULE:	23S RIBOSOMAL RNA
92:00:00	1yjn-P	3.5	3	50	143	4	MOLECULE:	23S RIBOSOMAL RNA
93:00:00	8ovt-D	3.5	4	68	286	9	MOLECULE:	YEGT GLYCOSYLTRANSFERASE
94:00:00	7ak5-n	3.5	4.4	55	176	5	MOLECULE:	NADH-UBIQUINONE OXIDOREDUCTASE CHAIN 3
95:00:00	7w1o-p	3.5	4.5	57	178	4	MOLECULE:	NADH DEHYDROGENASE [UBIQUINONE] FLAVOPROTEIN 1,
96:00:00	6zkv-t	3.5	5	53	177	6	MOLECULE:	NADH DEHYDROGENASE [UBIQUINONE] FLAVOPROTEIN 1,
97:00:00	7qsn-n	3.5	3.1	52	171	8	MOLECULE:	NADH-UBIQUINONE OXIDOREDUCTASE CHAIN 3
98:00:00	7vwl-p	3.5	4.4	56	178	4	MOLECULE:	NADH DEHYDROGENASE [UBIQUINONE] IRON-SULFUR PROTE
99:00:00	7vy1-p	3.5	4.6	57	178	5	MOLECULE:	NADH DEHYDROGENASE [UBIQUINONE] IRON-SULFUR PROTE
0:00	5xtd-p	3.5	3.1	53	172	4	MOLECULE:	NADH DEHYDROGENASE [UBIQUINONE] FLAVOPROTEIN 1,
1:00	7qso-n	3.5	3.3	53	171	6	MOLECULE:	NADH-UBIQUINONE OXIDOREDUCTASE CHAIN 3
2:00	6zkd-t	3.5	5	54	177	6	MOLECULE:	NADH DEHYDROGENASE [UBIQUINONE] FLAVOPROTEIN 1,
3:00	3cma-P	3.5	3	50	142	6	MOLECULE:	50S RIBOSOMAL PROTEIN L2P
4:00	7qsk-n	3.5	3	52	172	6	MOLECULE:	NADH-UBIQUINONE OXIDOREDUCTASE CHAIN 3
5:00	1kqs-O	3.5	2.9	50	143	6	MOLECULE:	23S RRNA
6:00	8q25-n	3.5	3.2	53	171	6	MOLECULE:	NADH-UBIQUINONE OXIDOREDUCTASE CHAIN 3
7:00	8q47-n	3.5	3	51	171	6	MOLECULE:	NADH-UBIQUINONE OXIDOREDUCTASE CHAIN 3
8:00	3i55-P	3.5	2.9	50	143	6	MOLECULE:	23S RIBOSOMAL RNA
9:00	6zki-t	3.5	5	53	177	6	MOLECULE:	NADH DEHYDROGENASE [UBIQUINONE] FLAVOPROTEIN 1,
10:00	7bvg-B	3.5	2.5	55	1063	4	MOLECULE:	INTEGRAL MEMBRANE INDOLYLACETYLINOSITOL
11:00	1q82-Q	3.5	2.9	50	143	6	MOLECULE:	23S RIBOSOMAL RNA
12:00	7w2k-p	3.5	4.5	57	178	4	MOLECULE:	NADH DEHYDROGENASE [UBIQUINONE] FLAVOPROTEIN 1,
13:00	3cce-P	3.5	2.9	50	143	6	MOLECULE:	50S RIBOSOMAL PROTEIN L2P
14:00	7v2r-p	3.5	4.4	56	178	4	MOLECULE:	NADH DEHYDROGENASE [UBIQUINONE] FLAVOPROTEIN 1,
15:00	1vqo-P	3.5	3	50	143	4	MOLECULE:	23S RIBOSOMAL RNA
16:00	7zdp-t	3.5	2.9	52	177	6	MOLECULE:	COMPLEX I-49KD
17:00	7w4n-p	3.5	4.4	57	178	4	MOLECULE:	NADH DEHYDROGENASE [UBIQUINONE] FLAVOPROTEIN 1,
18:00	1qvg-O	3.5	2.9	50	143	6	MOLECULE:	23S RIBOSOMAL RNA
19:00	1yij-P	3.5	2.9	50	143	6	MOLECULE:	23S RIBOSOMAL RNA
20:00	1vqk-P	3.5	2.9	50	143	6	MOLECULE:	23S RIBOSOMAL RNA
21:00	5d80-b	3.5	3.4	72	598	4	MOLECULE:	V-TYPE PROTON ATPASE CATALYTIC SUBUNIT A
22:00	1nji-Q	3.5	2.9	50	143	6	MOLECULE:	23S RIBOSOMAL RNA
23:00	1yi2-P	3.5	2.9	50	143	6	MOLECULE:	23S RIBOSOMAL RNA
24:00:00	1s72-P	3.5	2.9	50	143	6	MOLECULE:	23S RIBOSOMAL RNA
25:00:00	1vqn-P	3.5	3	50	143	4	MOLECULE:	23S RIBOSOMAL RNA
26:00:00	8q45-n	3.5	3	52	171	6	MOLECULE:	NADH-UBIQUINONE OXIDOREDUCTASE CHAIN 3
27:00:00	8beh-n	3.5	2.7	52	109	4	MOLECULE:	NADH-UBIQUINONE OXIDOREDUCTASE CHAIN 5
28:00:00	3cd6-P	3.5	2.9	50	143	6	MOLECULE:	50S RIBOSOMAL PROTEIN L2P
29:00:00	6zkb-t	3.5	5.1	53	177	6	MOLECULE:	MITOCHONDRIAL COMPLEX I, 49 KDA SUBUNIT
30:00:00	1qvf-O	3.5	2.9	50	143	6	MOLECULE:	23S RIBOSOMAL RNA
31:00:00	1m1k-Q	3.5	2.9	50	143	6	MOLECULE:	23S RRNA
32:00:00	1vq8-P	3.5	2.9	50	143	6	MOLECULE:	23S RIBOSOMAL RNA
33:00:00	7vyi-p	3.5	4.6	57	178	5	MOLECULE:	NADH DEHYDROGENASE [UBIQUINONE] IRON-SULFUR PROTE
34:00:00	1jj2-O	3.5	3.1	50	143	4	MOLECULE:	23S RRNA

35:00:00	7zeb-t	3.5	5	54	177	6	MOLECULE:	NADH DEHYDROGENASE [UBIQUINONE] IRON-SULFUR PROTE
36:00:00	8q1p-n	3.5	3.1	53	171	6	MOLECULE:	NADH-UBIQUINONE OXIDOREDUCTASE CHAIN 3
37:00:00	8q48-n	3.5	3.1	52	171	8	MOLECULE:	NADH-UBIQUINONE OXIDOREDUCTASE CHAIN 3
38:00:00	1vqp-P	3.5	2.9	50	143	6	MOLECULE:	23S RIBOSOMAL RNA
39:00:00	6wm4-B	3.5	3	66	600	11	MOLECULE:	V-TYPE PROTON ATPASE 116 KDA SUBUNIT A ISOFORM 1
40:00:00	7upq-A	3.5	3.7	67	198	9	MOLECULE:	DHT03 PROTEIN A
41:00:00	6wm3-C	3.5	3.1	61	600	5	MOLECULE:	V-TYPE PROTON ATPASE 116 KDA SUBUNIT A ISOFORM 1
42:00:00	7qpr-B	3.5	2.6	56	687	5	MOLECULE:	ACT DOMAIN PROTEIN
43:00:00	8v3b-Q	3.5	3	62	314	2	MOLECULE:	O43_129_+4 COMPONENT A
44:00:00	3j9t-C	3.5	3.1	69	593	7	MOLECULE:	V-TYPE PROTON ATPASE SUBUNIT D
45:00:00	8v2d-i	3.5	3	60	314	0	MOLECULE:	O43_129 COMPONENT B
46:00:00	3j9v-C	3.5	3.3	69	593	7	MOLECULE:	V-TYPE PROTON ATPASE SUBUNIT A, VACUOLAR ISOFORM
47:00:00	3jb9-R	3.4	2.8	59	575	0	MOLECULE:	PRE-MRNA-SPLICING FACTOR SPP42
48:00:00	7xyq-B	3.4	3.5	52	124	13	MOLECULE:	CD274 MOLECULE
49:00:00	3bbz-A	3.4	2.4	45	48	4	MOLECULE:	P PROTEIN
50:00:00	2g8l-B	3.4	2.7	52	286	6	MOLECULE:	287AA LONG HYPOTHETICAL PROTEIN
51:00:00	5gmk-d	3.4	2.8	59	549	0	MOLECULE:	PRE-MRNA-SPLICING FACTOR 8
52:00:00	6ybw-z	3.4	6	60	160	5	MOLECULE:	40S RIBOSOMAL PROTEIN S4, X ISOFORM
53:00:00	6qfo-A	3.4	4	55	1348	4	MOLECULE:	PEGA DOMAIN-CONTAINING PROTEIN,PEGA DOMAIN-CONTAI
54:00:00	7v2d-p	3.4	4.6	57	178	5	MOLECULE:	NADH DEHYDROGENASE [UBIQUINONE] FLAVOPROTEIN 1,
55:00:00	7cxm-C	3.4	3.6	53	72	4	MOLECULE:	NSP12
56:00:00	3gn4-A	3.4	3.1	51	129	6	MOLECULE:	MYOSIN-VI
57:00:00	6m71-C	3.4	3.8	55	70	4	MOLECULE:	SARS-COV-2 NSP 12
58:00:00	5h7a-F	3.4	2.2	46	188	7	MOLECULE:	IMMUNOGLOBULIN G-BINDING PROTEIN A
59:00:00	6k68-L	3.4	3	49	50	4	MOLECULE:	3MNZ VARIABLE HEAVY CHAIN
60:00:00	7cxn-C	3.4	3.5	53	72	4	MOLECULE:	NSP12
61:00:00	6zkn-t	3.4	5	53	177	6	MOLECULE:	NADH DEHYDROGENASE [UBIQUINONE] FLAVOPROTEIN 1,
62:00:00	6zkm-t	3.4	5	53	177	6	MOLECULE:	NADH DEHYDROGENASE [UBIQUINONE] FLAVOPROTEIN 1,
63:00:00	8tl7-R	3.4	2.3	56	694	5	MOLECULE:	COMPUTATIONALLY DESIGNED PROTEIN
64:00:00	6g72-n	3.4	4.5	57	176	5	MOLECULE:	NADH-UBIQUINONE OXIDOREDUCTASE CHAIN 3
65:00:00	6g2j-n	3.4	4.5	57	176	5	MOLECULE:	NADH-UBIQUINONE OXIDOREDUCTASE CHAIN 3
66:00:00	5nbz-B	3.4	3.1	58	237	9	MOLECULE:	WZZB
67:00:00	3ze5-B	3.4	3.2	66	116	14	MOLECULE:	DIACYLGLYCEROL KINASE
68:00:00	1k8a-Q	3.4	2.9	50	143	6	MOLECULE:	23S RRNA
69:00:00	5ujm-C	3.4	3	60	553	7	MOLECULE:	ORIGIN RECOGNITION COMPLEX SUBUNIT 1
70:00:00	7ohg-A	3.4	2.5	53	351	4	MOLECULE:	GUANOSINE-3',5'-BIS(DIPHOSPHATE) 3'-PYROPHOSPHOHY
71:00:00	8acu-A	3.4	2.3	50	349	8	MOLECULE:	GTP PYROPHOSPHOKINASE
72:00:00	1n8r-Q	3.4	2.9	50	143	6	MOLECULE:	23S RIBOSOMAL RNA
73:00:00	7w32-p	3.4	4.5	56	178	5	MOLECULE:	NADH DEHYDROGENASE [UBIQUINONE] FLAVOPROTEIN 1,
74:00:00	8q1u-n	3.4	2.5	50	171	6	MOLECULE:	NADH-UBIQUINONE OXIDOREDUCTASE CHAIN 3
75:00:00	7v3m-p	3.4	4.6	56	178	5	MOLECULE:	NADH DEHYDROGENASE [UBIQUINONE] FLAVOPROTEIN 1,
76:00:00	3cc7-P	3.4	3	50	143	4	MOLECULE:	50S RIBOSOMAL PROTEIN L2P
77:00:00	8q0f-n	3.4	3	52	171	6	MOLECULE:	NADH-UBIQUINONE OXIDOREDUCTASE CHAIN 3
78:00:00	7zdj-t	3.4	4.9	54	177	6	MOLECULE:	COMPLEX I-49KD
79:00:00	7w4k-p	3.4	4.4	56	178	5	MOLECULE:	NADH DEHYDROGENASE [UBIQUINONE] FLAVOPROTEIN 1,
80:00:00	7qsl-n	3.4	3	52	172	6	MOLECULE:	NADH-UBIQUINONE OXIDOREDUCTASE CHAIN 3
81:00:00	6zke-t	3.4	5.1	53	177	6	MOLECULE:	NADH DEHYDROGENASE [UBIQUINONE] FLAVOPROTEIN 1,
82:00:00	7cos-H	3.4	3.5	72	586	4	MOLECULE:	V-TYPE SODIUM ATPASE CATALYTIC SUBUNIT A
83:00:00	7w2l-p	3.4	4.5	57	178	4	MOLECULE:	NADH DEHYDROGENASE [UBIQUINONE] FLAVOPROTEIN 1,
84:00:00	6zkc-t	3.4	5.1	54	177	6	MOLECULE:	NADH DEHYDROGENASE [UBIQUINONE] FLAVOPROTEIN 1,
85:00:00	6qev-B	3.4	3	51	1346	2	MOLECULE:	PEGA DOMAIN-CONTAINING PROTEIN,PEGA DOMAIN-CONTAI
86:00:00	7k8b-A	3.4	3.2	58	726	7	MOLECULE:	DRUG EXPORTERS OF THE RND SUPERFAMILY-LIKE PROTEI
87:00:00	7v32-p	3.4	4.4	56	178	4	MOLECULE:	NADH DEHYDROGENASE [UBIQUINONE] FLAVOPROTEIN 1,
88:00:00	7w35-p	3.4	4.4	56	178	4	MOLECULE:	NADH DEHYDROGENASE [UBIQUINONE] FLAVOPROTEIN 1,
89:00:00	7arb-n	3.4	2.9	52	109	6	MOLECULE:	NADH-UBIQUINONE OXIDOREDUCTASE CHAIN 3
90:00:00	5xtc-p	3.4	3.1	52	172	6	MOLECULE:	NADH DEHYDROGENASE [UBIQUINONE] IRON-SULFUR PROTE

91:00:00	7w4j-p	3.4	4.4	56	178	5	MOLECULE:	NADH DEHYDROGENASE [UBIQUINONE] FLAVOPROTEIN 1,
92:00:00	1q81-Q	3.4	2.9	50	143	6	MOLECULE:	23S RIBOSOMAL RNA
93:00:00	3ccu-P	3.4	2.9	50	143	6	MOLECULE:	50S RIBOSOMAL PROTEIN L2P
94:00:00	3g6e-P	3.4	3	50	143	4	MOLECULE:	23S RIBOSOMAL RNA
95:00:00	7aqw-n	3.4	2.9	52	109	6	MOLECULE:	NADH-UBIQUINONE OXIDOREDUCTASE CHAIN 5
96:00:00	7w0h-p	3.4	4.6	57	178	5	MOLECULE:	NADH DEHYDROGENASE [UBIQUINONE] FLAVOPROTEIN 1,
97:00:00	6vqb-B	3.4	3.5	68	600	10	MOLECULE:	ATPASE H+-TRANSPORTING V1 SUBUNIT A
98:00:00	6r0z-B	3.4	4	71	578	1	MOLECULE:	V-TYPE ATP SYNTHASE ALPHA CHAIN
99:00:00	5cwo-B	3.4	3.4	67	219	9	MOLECULE:	DESIGNED HELICAL REPEAT PROTEIN
0:00	3j9v-E	3.4	3.1	70	593	9	MOLECULE:	V-TYPE PROTON ATPASE SUBUNIT A, VACUOLAR ISOFORM
1:00	7tmq-C	3.4	3.8	66	590	8	MOLECULE:	H(+)-TRANSPORTING TWO-SECTOR ATPASE
2:00	5knc-B	3.4	3.2	68	588	10	MOLECULE:	V-TYPE SODIUM ATPASE CATALYTIC SUBUNIT A
3:00	7khr-B	3.4	3.3	63	591	3	MOLECULE:	V-TYPE PROTON ATPASE CATALYTIC SUBUNIT A
4:00	5y5z-C	3.4	3	67	576	3	MOLECULE:	V-TYPE ATP SYNTHASE ALPHA CHAIN
5:00	6ly8-C	3.4	3.2	69	577	3	MOLECULE:	V-TYPE ATP SYNTHASE ALPHA CHAIN
6:00	5cwo-A	3.4	3.2	61	219	10	MOLECULE:	DESIGNED HELICAL REPEAT PROTEIN
7:00	7fda-C	3.4	3.3	62	594	6	MOLECULE:	YEAST VACUOLAR ATPASE A SUBUNIT
8:00	2zhg-A	3.3	3	58	121	9	MOLECULE:	REDOX-SENSITIVE TRANSCRIPTIONAL ACTIVATOR SOXR
9:00	6l7r-A	3.3	2.7	49	105	12	MOLECULE:	PUTATIVE SPINDLE POLE BODY COMPONENT ALP6 PROTEIN
10:00	8jpa-A	3.3	3.2	61	139	7	MOLECULE:	DE NOVO DESIGN CAVITATED PROTEIN
11:00	4uxv-A	3.3	3.5	53	522	9	MOLECULE:	SEPTATION RING FORMATION REGULATOR EZRA
12:00	6rie-A	3.3	3.1	61	1285	7	MOLECULE:	DNA-DEPENDENT RNA POLYMERASE SUBUNIT RPO147
13:00	6l1q-A	3.3	5.3	56	267	13	MOLECULE:	CBBQ PROTEIN
14:00	7d2w-A	3.3	2.6	58	125	10	MOLECULE:	PRESAN DOMAIN-CONTAINING PROTEIN
15:00	8bob-C	3.3	3.6	57	412	0	MOLECULE:	PROTEIN MALY
16:00	2n5j-A	3.3	3.2	44	49	18	MOLECULE:	RIBONUCLEASE ZC3H12A
17:00	7obq-y	3.3	4.2	60	454	8	MOLECULE:	SRP RNA
18:00	2hjm-A	3.3	2.1	50	91	4	MOLECULE:	HYPOTHETICAL PROTEIN PF1176
19:00	7ng0-A	3.3	3.4	61	199	10	MOLECULE:	NUCLEOID OCCLUSION PROTEIN
20:00	5u5q-A	3.3	3.7	67	1434	6	MOLECULE:	DNA-DIRECTED RNA POLYMERASE II SUBUNIT RPB1
21:00	7bzf-C	3.3	3.8	55	68	4	MOLECULE:	RNA-DIRECTED RNA POLYMERASE
22:00	7bv2-C	3.3	2.8	49	63	4	MOLECULE:	NSP12
23:00	6cgh-A	3.3	3	59	89	2	MOLECULE:	DNAJ HOMOLOG SUBFAMILY C MEMBER 2
24:00:00	7rmx-A	3.3	2.7	53	229	4	MOLECULE:	TUNABLE SYMMETRIC PROTEIN, D_3_212
25:00:00	6uxe-B	3.3	2.3	49	85	6	MOLECULE:	CYSTEINE DESULFURASE, MITOCHONDRIAL
26:00:00	5i6r-A	3.3	3.2	57	419	2	MOLECULE:	SLIT-ROBO RHO GTPASE-ACTIVATING PROTEIN 2
27:00:00	6zkh-t	3.3	5	53	177	6	MOLECULE:	NADH DEHYDROGENASE [UBIQUINONE] FLAVOPROTEIN 1,
28:00:00	7d2w-B	3.3	2.6	58	125	3	MOLECULE:	PRESAN DOMAIN-CONTAINING PROTEIN
29:00:00	2ffj-B	3.3	2.9	51	271	6	MOLECULE:	CONSERVED HYPOTHETICAL PROTEIN
30:00:00	6dfk-N	3.3	2.6	55	233	0	MOLECULE:	SUBUNIT OF PROTEASEOME ACTIVATOR COMPLEX,PUTATIVE
31:00:00	1w2b-O	3.3	3.3	50	143	4	MOLECULE:	23S RRNA
32:00:00	2g8l-A	3.3	2.7	52	284	6	MOLECULE:	287AA LONG HYPOTHETICAL PROTEIN
33:00:00	3vr6-A	3.3	4.4	62	587	6	MOLECULE:	V-TYPE SODIUM ATPASE CATALYTIC SUBUNIT A
34:00:00	7bx8-B	3.3	2.9	58	1038	3	MOLECULE:	INTEGRAL MEMBRANE INDOLYLACETYLINOSITOL
35:00:00	3ccq-P	3.3	3.1	50	143	4	MOLECULE:	50S RIBOSOMAL PROTEIN L2P
36:00:00	6vq8-B	3.3	3.5	68	600	10	MOLECULE:	ATPASE H+-TRANSPORTING V1 SUBUNIT A
37:00:00	6zkf-t	3.3	5.1	52	177	6	MOLECULE:	NADH DEHYDROGENASE [UBIQUINONE] FLAVOPROTEIN 1,
38:00:00	7w4q-p	3.3	4.6	57	178	5	MOLECULE:	NADH DEHYDROGENASE [UBIQUINONE] FLAVOPROTEIN 1,
39:00:00	7w4d-p	3.3	4.5	58	178	3	MOLECULE:	NADH DEHYDROGENASE [UBIQUINONE] FLAVOPROTEIN 1,
40:00:00	3ow2-O	3.3	3	50	143	4	MOLECULE:	23S RIBOSOMAL RNA
41:00:00	3jb7-A	3.3	3.4	74	1198	4	MOLECULE:	CPV RNA-DEPENDENT RNA POLYMERASE
42:00:00	3ccj-P	3.3	2.9	50	143	6	MOLECULE:	50S RIBOSOMAL PROTEIN L2P
43:00:00	8q0m-n	3.3	3.1	52	171	6	MOLECULE:	NADH-UBIQUINONE OXIDOREDUCTASE CHAIN 3
44:00:00	3g4s-P	3.3	3	50	143	4	MOLECULE:	23S RIBOSOMAL RNA
45:00:00	7v30-p	3.3	4.5	56	178	5	MOLECULE:	NADH DEHYDROGENASE [UBIQUINONE] FLAVOPROTEIN 1,
46:00:00	1yit-P	3.3	3	50	143	4	MOLECULE:	23S RIBOSOMAL RNA

47:00:00	8olt-n	3.3	4.5	57	177	4	MOLECULE:	NADH-UBIQUINONE OXIDOREDUCTASE CHAIN 3
48:00:00	7jpo-C	3.3	3.1	60	594	5	MOLECULE:	ORIGIN RECOGNITION COMPLEX SUBUNIT 1
49:00:00	6ajj-A	3.3	4.4	58	901	9	MOLECULE:	DRUG EXPORTERS OF THE RND SUPERFAMILY-LIKE PROTEI
50:00:00	6o7x-E	3.3	3.1	67	593	7	MOLECULE:	V-TYPE PROTON ATPASE SUBUNIT C
51:00:00	7dqc-A	3.3	3.8	63	586	8	MOLECULE:	V-TYPE SODIUM ATPASE CATALYTIC SUBUNIT A
52:00:00	7cor-A	3.3	3.2	68	587	9	MOLECULE:	V-TYPE SODIUM ATPASE CATALYTIC SUBUNIT A
53:00:00	6ly8-A	3.3	3.2	69	577	1	MOLECULE:	V-TYPE ATP SYNTHASE ALPHA CHAIN
54:00:00	6o7v-C	3.3	3.3	67	593	7	MOLECULE:	V-TYPE PROTON ATPASE SUBUNIT D
55:00:00	3kdw-A	3.2	3.2	61	206	11	MOLECULE:	PUTATIVE SUGAR BINDING PROTEIN
56:00:00	5mmj-c	3.2	8.2	52	216	10	MOLECULE:	50S RIBOSOMAL PROTEIN L31
57:00:00	1xfi-A	3.2	3.4	54	343	6	MOLECULE:	UNKNOWN PROTEIN
58:00:00	6zko-t	3.2	5	54	177	6	MOLECULE:	NADH DEHYDROGENASE [UBIQUINONE] FLAVOPROTEIN 1,
59:00:00	6v85-F	3.2	2.4	47	47	4	MOLECULE:	RNA-DIRECTED RNA POLYMERASE L
60:00:00	1sb0-A	3.2	2.3	49	87	4	MOLECULE:	PROTEIN CBP
61:00:00	7jlt-C	3.2	3.8	53	81	4	MOLECULE:	NON-STRUCTURAL PROTEIN 7
62:00:00	8df7-A	3.2	3.3	54	850	4	MOLECULE:	TOPOISOMERASE V
63:00:00	5oxf-C	3.2	3.4	64	601	9	MOLECULE:	GTP-BINDING PROTEIN
64:00:00	8hra-E	3.2	6.4	59	834	10	MOLECULE:	ARCHAEAL ATPASE
65:00:00	8hr9-J	3.2	6.2	58	835	10	MOLECULE:	ARCHAEAL ATPASE
66:00:00	7v2k-p	3.2	4.5	56	178	5	MOLECULE:	NADH DEHYDROGENASE [UBIQUINONE] FLAVOPROTEIN 1,
67:00:00	4uq8-W	3.2	2.7	54	72	0	MOLECULE:	NADH UBIQUINONE OXIDOREDUCTASE CHAIN 3
68:00:00	6poo-A	3.2	3.2	54	273	4	MOLECULE:	BIBA
69:00:00	6rjx-A	3.2	3.1	54	157	2	MOLECULE:	LYSM DOMAIN PROTEIN
70:00:00	4heo-B	3.2	2.4	48	60	4	MOLECULE:	PHOSPHOPROTEIN
71:00:00	3cc4-P	3.2	2.9	50	143	6	MOLECULE:	50S RIBOSOMAL PROTEIN L2P
72:00:00	2qa4-P	3.2	2.9	50	143	6	MOLECULE:	23S RIBOSOMAL RNA
73:00:00	2px0-H	3.2	3.3	54	258	11	MOLECULE:	FLAGELLAR BIOSYNTHESIS PROTEIN FLHF
74:00:00	6r0w-B	3.2	4.6	60	577	7	MOLECULE:	V-TYPE ATP SYNTHASE ALPHA CHAIN
75:00:00	3vr2-A	3.2	3	59	586	5	MOLECULE:	V-TYPE SODIUM ATPASE CATALYTIC SUBUNIT A
76:00:00	7upq-D	3.2	3.6	78	198	10	MOLECULE:	DHT03 PROTEIN A
77:00:00	7vye-p	3.2	4.5	57	178	4	MOLECULE:	NADH DEHYDROGENASE [UBIQUINONE] IRON-SULFUR PROTE
78:00:00	7w20-p	3.2	4.4	56	178	4	MOLECULE:	NADH DEHYDROGENASE [UBIQUINONE] FLAVOPROTEIN 1,
79:00:00	7w31-p	3.2	4.6	57	178	5	MOLECULE:	NADH DEHYDROGENASE [UBIQUINONE] FLAVOPROTEIN 1,
80:00:00	3ccr-P	3.2	2.9	50	143	6	MOLECULE:	50S RIBOSOMAL PROTEIN L2P
81:00:00	3a5c-B	3.2	3.6	72	561	6	MOLECULE:	V-TYPE ATP SYNTHASE ALPHA CHAIN
82:00:00	1yj9-P	3.2	2.9	50	143	6	MOLECULE:	23S RIBOSOMAL RNA
83:00:00	7w4m-p	3.2	4.5	56	178	5	MOLECULE:	NADH DEHYDROGENASE [UBIQUINONE] FLAVOPROTEIN 1,
84:00:00	7v2f-p	3.2	4.5	57	178	4	MOLECULE:	NADH DEHYDROGENASE [UBIQUINONE] FLAVOPROTEIN 1,
85:00:00	3bge-A	3.2	5.8	59	163	12	MOLECULE:	PREDICTED ATPASE
86:00:00	6r10-B	3.2	3.3	69	577	1	MOLECULE:	V-TYPE ATP SYNTHASE ALPHA CHAIN
87:00:00	7fdb-A	3.2	3.3	58	594	7	MOLECULE:	YEAST VACUOLAR ATPASE A SUBUNIT
88:00:00	6r0w-C	3.2	3.2	67	577	1	MOLECULE:	V-TYPE ATP SYNTHASE ALPHA CHAIN
89:00:00	7nad-R	3.2	3.3	52	134	8	MOLECULE:	25S RRNA
90:00:00	6r0z-A	3.2	3	67	577	3	MOLECULE:	V-TYPE ATP SYNTHASE ALPHA CHAIN
91:00:00	6o7w-C	3.2	3.2	68	593	7	MOLECULE:	V-TYPE PROTON ATPASE SUBUNIT D
92:00:00	5y60-C	3.2	4	65	576	3	MOLECULE:	V-TYPE ATP SYNTHASE ALPHA CHAIN
93:00:00	6r0y-C	3.2	3.2	66	577	2	MOLECULE:	V-TYPE ATP SYNTHASE ALPHA CHAIN
94:00:00	7jk5-C	3.2	3.2	59	590	12	MOLECULE:	ORIGIN RECOGNITION COMPLEX SUBUNIT 2
95:00:00	3gqb-C	3.2	2.9	66	578	3	MOLECULE:	V-TYPE ATP SYNTHASE ALPHA CHAIN
96:00:00	5knc-A	3.2	3.4	70	588	9	MOLECULE:	V-TYPE SODIUM ATPASE CATALYTIC SUBUNIT A
97:00:00	6s2t-A	3.2	2.5	52	350	4	MOLECULE:	(P)PPGPP SYNTHETASE I, SPOT/RELA
98:00:00	6xby-B	3.2	3.6	72	577	3	MOLECULE:	V-TYPE PROTON ATPASE CATALYTIC SUBUNIT A
99:00:00	4zil-A	3.1	3.5	63	210	2	MOLECULE:	DSBA OXIDOREDUCTASE
0:00	3u5z-B	3.1	5.1	60	320	7	MOLECULE:	DNA POLYMERASE ACCESSORY PROTEIN 44
1:00	5cwc-A	3.1	2.5	54	196	2	MOLECULE:	DESIGNED HELICAL REPEAT PROTEIN
2:00	7sgz-A	3.1	3.3	56	418	2	MOLECULE:	CHECKPOINT PROTEIN RAD24

3:00	5lc5-n	3.1	2.7	52	166	6	MOLECULE:	NADH-UBIQUINONE OXIDOREDUCTASE CHAIN 3
4:00	6wc9-A	3.1	5.2	55	367	7	MOLECULE:	ENDOSOMAL/LYSOSOMAL POTASSIUM CHANNEL TMEM175
5:00	5z8q-A	3.1	3.6	56	101	9	MOLECULE:	HEAT SHOCK PROTEIN SSA1
6:00	5ys9-A	3.1	3.7	66	692	8	MOLECULE:	ACYL-COENZYME A OXIDASE 3
7:00	6qyi-B	3.1	5.6	57	519	4	MOLECULE:	4-HYDROXYPHENYLACETATE 3-MONOOXYGENASE OXYGENASE
8:00	8hr9-L	3.1	6.2	57	833	11	MOLECULE:	ARCHAEAL ATPASE
9:00	2hz8-A	3.1	4.4	58	115	5	MOLECULE:	DE NOVO DESIGNED DIIRON PROTEIN
10:00	6nur-C	3.1	3.7	53	70	4	MOLECULE:	NSP12
11:00	8q0q-n	3.1	3.1	53	171	6	MOLECULE:	NADH-UBIQUINONE OXIDOREDUCTASE CHAIN 3
12:00	6m5i-A	3.1	3.7	53	81	4	MOLECULE:	SARS-COV-2 NSP8
13:00	5lfj-B	3.1	4.4	52	113	0	MOLECULE:	BACTERIAL PROTEASOME ACTIVATOR
14:00	7rkc-A	3.1	3.6	67	220	7	MOLECULE:	D_3_633
15:00	7tdr-A	3.1	2.4	58	289	10	MOLECULE:	34K2 SALIVARY PROTEIN
16:00	2ewf-A	3.1	3.9	54	587	6	MOLECULE:	NICKING ENDONUCLEASE N.BSPD6I
17:00	2vda-A	3.1	3.8	75	828	5	MOLECULE:	TRANSLOCASE SUBUNIT SECA
18:00	8t03-A	3.1	3	63	199	3	MOLECULE:	PROTEIN MYOMAKER
19:00	8buy-A	3.1	2.7	59	134	5	MOLECULE:	GRANULE ASSOCIATED RAC AND RHOG EFFECTOR PROTEIN
20:00	7vfp-F	3.1	3.7	68	220	10	MOLECULE:	CYTOCHROME C BIOGENESIS ATP-BINDING EXPORT PROTEI
21:00	2otl-P	3.1	2.9	50	143	6	MOLECULE:	23S RIBOSOMAL RNA
22:00	5zea-I	3.1	4.2	60	584	7	MOLECULE:	V-TYPE SODIUM ATPASE CATALYTIC SUBUNIT A
23:00	5vre-B	3.1	3.1	54	188	6	MOLECULE:	PUTATIVE INTEGRAL MEMBRANE PROTEIN
24:00:00	3cpw-O	3.1	2.9	50	143	6	MOLECULE:	23S RIBOSOMAL RNA
25:00:00	6o7v-E	3.1	3.6	71	593	3	MOLECULE:	V-TYPE PROTON ATPASE SUBUNIT D
26:00:00	6zkj-t	3.1	4.9	53	177	6	MOLECULE:	NADH DEHYDROGENASE [UBIQUINONE] FLAVOPROTEIN 1,
27:00:00	3cc2-P	3.1	2.9	50	143	6	MOLECULE:	50S RIBOSOMAL PROTEIN L2P
28:00:00	6zkl-t	3.1	2.9	52	177	6	MOLECULE:	NADH DEHYDROGENASE [UBIQUINONE] FLAVOPROTEIN 1,
29:00:00	5cbo-G	3.1	2.9	49	176	6	MOLECULE:	MBP3-16,IMMUNOGLOBULIN G-BINDING PROTEIN A
30:00:00	5ze9-A	3.1	3.6	72	588	4	MOLECULE:	V-TYPE SODIUM ATPASE CATALYTIC SUBUNIT A
31:00:00	6vq7-B	3.1	3.6	73	600	4	MOLECULE:	ATPASE H+-TRANSPORTING V1 SUBUNIT A
32:00:00	3a5d-B	3.1	3.7	74	561	5	MOLECULE:	V-TYPE ATP SYNTHASE ALPHA CHAIN
33:00:00	3ccs-P	3.1	3	50	143	4	MOLECULE:	50S RIBOSOMAL PROTEIN L2P
34:00:00	1vqm-P	3.1	3	50	143	4	MOLECULE:	23S RIBOSOMAL RNA
35:00:00	6qep-A	3.1	3.2	51	1341	2	MOLECULE:	PEGA DOMAIN-CONTAINING PROTEIN,ENGBF-DARPIN FUSIO
36:00:00	3ccl-P	3.1	2.9	50	143	6	MOLECULE:	50S RIBOSOMAL PROTEIN L2P
37:00:00	1vq4-P	3.1	3.1	50	143	4	MOLECULE:	23S RIBOSOMAL RNA
38:00:00	5voz-A	3.1	3.6	69	592	3	MOLECULE:	V-TYPE PROTON ATPASE CATALYTIC SUBUNIT A,V-TYPE P
39:00:00	6sh9-B	3.1	4	56	1339	4	MOLECULE:	ENDO-ALPHA-N-ACETYLGALACTOSAMINIDASE,DARPIN 4B D1
40:00:00	7fda-E	3.1	3.6	64	594	6	MOLECULE:	YEAST VACUOLAR ATPASE A SUBUNIT
41:00:00	5y5z-A	3.1	3.1	66	576	2	MOLECULE:	V-TYPE ATP SYNTHASE ALPHA CHAIN
42:00:00	6qle-N	3.1	2.2	47	371	15	MOLECULE:	CENTRAL KINETOCHORE SUBUNIT MCM16,CENTRAL KINETOC
43:00:00	7fda-A	3.1	4.6	67	594	4	MOLECULE:	YEAST VACUOLAR ATPASE A SUBUNIT
44:00:00	5y5y-A	3.1	3.1	69	577	7	MOLECULE:	V-TYPE ATP SYNTHASE ALPHA CHAIN
45:00:00	6tz2-A	3.1	4.7	78	1208	6	MOLECULE:	RNA-DEPENDENT RNA POLYMERASE
46:00:00	6r0y-A	3.1	3.3	69	577	1	MOLECULE:	V-TYPE ATP SYNTHASE ALPHA CHAIN
47:00:00	6wm3-A	3.1	3.6	69	600	3	MOLECULE:	V-TYPE PROTON ATPASE 116 KDA SUBUNIT A ISOFORM 1
48:00:00	3j9u-E	3.1	2.8	63	593	8	MOLECULE:	V-TYPE PROTON ATPASE SUBUNIT D
49:00:00	6ly8-B	3.1	3.1	69	577	4	MOLECULE:	V-TYPE ATP SYNTHASE ALPHA CHAIN
50:00:00	6r10-A	3.1	3.4	68	577	3	MOLECULE:	V-TYPE ATP SYNTHASE ALPHA CHAIN
51:00:00	8utk-A	3	3.3	48	55	6	MOLECULE:	23R-B04DSLF02IB
52:00:00	2a3v-B	3	2.9	50	320	4	MOLECULE:	DNA (31-MER)
53:00:00	8oyy-A	3	4.2	62	233	5	MOLECULE:	DE NOVO DESIGNED SOLUBLE GPCR-LIKE PROTEIN
54:00:00	7m9a-G	3	3.6	58	257	5	MOLECULE:	TNSC
55:00:00	7m9c-G	3	3.6	58	257	5	MOLECULE:	TNSC
56:00:00	7m9b-G	3	3.4	57	257	5	MOLECULE:	TNSC
57:00:00	7m9b-D	3	3.9	61	257	7	MOLECULE:	TNSC
58:00:00	7m9b-C	3	3.1	55	257	5	MOLECULE:	TNSC

59:00:00	7m9a-D	3	3.9	60	257	7	MOLECULE:	TNSC
60:00:00	7m9c-D	3	3.9	60	257	7	MOLECULE:	TNSC
61:00:00	7ole-A	3	3.4	60	433	3	MOLECULE:	RUVB-LIKE 1
62:00:00	3a98-A	3	3.7	58	157	7	MOLECULE:	DEDICATOR OF CYTOKINESIS PROTEIN 2
63:00:00	7a24-X	3	2.8	52	103	2	MOLECULE:	51KDA
64:00:00	5y2g-A	3	3.6	66	581	12	MOLECULE:	MALTOSE-BINDING PERIPLASMIC PROTEIN,PROTEIN B
65:00:00	6v86-F	3	2.4	44	47	5	MOLECULE:	RNA-DIRECTED RNA POLYMERASE L
66:00:00	5h7b-A	3	2.2	46	230	7	MOLECULE:	IMMUNOGLOBULIN G-BINDING PROTEIN A
67:00:00	5h7a-H	3	2.4	46	187	7	MOLECULE:	IMMUNOGLOBULIN G-BINDING PROTEIN A
68:00:00	6dlu-P	3	2.7	54	747	6	MOLECULE:	DYNAMIN-1
69:00:00	6dlc-A	3	4.3	55	105	5	MOLECULE:	DESIGNED PROTEIN DHD1:234_A
70:00:00	7a23-X	3	2.8	52	103	2	MOLECULE:	51KDA
71:00:00	8q0j-n	3	2.5	50	171	6	MOLECULE:	NADH-UBIQUINONE OXIDOREDUCTASE CHAIN 3
72:00:00	6hum-F	3	3.5	57	611	5	MOLECULE:	NAD(P)H-QUINONE OXIDOREDUCTASE SUBUNIT 1
73:00:00	6tgz-F	3	2.6	56	352	2	MOLECULE:	55 KDA IMMEDIATE-EARLY PROTEIN 1
74:00:00	3ok8-A	3	2.6	53	219	8	MOLECULE:	BRAIN-SPECIFIC ANGIOGENESIS INHIBITOR 1-ASSOCIATE
75:00:00	4dvz-A	3	4.7	58	459	9	MOLECULE:	CYTOTOXICITY-ASSOCIATED IMMUNODOMINANT ANTIGEN
76:00:00	8q7n-L	3	3.9	50	373	6	MOLECULE:	U5 SNRNA
77:00:00	8tl7-S	3	3.3	66	694	8	MOLECULE:	COMPUTATIONALLY DESIGNED PROTEIN
78:00:00	2o3l-A	3	2.5	48	83	6	MOLECULE:	HYPOTHETICAL PROTEIN
79:00:00	2qxl-B	3	2.8	52	625	2	MOLECULE:	HEAT SHOCK PROTEIN HOMOLOG SSE1
80:00:00	6bar-A	3	4.6	64	299	11	MOLECULE:	ROD SHAPE DETERMINING PROTEIN RODA
81:00:00	2wpv-A	3	9.3	50	281	6	MOLECULE:	UPF0363 PROTEIN YOR164C
82:00:00	6th1-R	3	2.8	61	360	5	MOLECULE:	IMMEDIATE EARLY PROTEIN 1
83:00:00	1vq6-P	3	2.9	50	143	6	MOLECULE:	23S RIBOSOMAL RNA
84:00:00	5knc-C	3	4.4	62	586	6	MOLECULE:	V-TYPE SODIUM ATPASE CATALYTIC SUBUNIT A
85:00:00	3vr2-B	3	3	59	586	5	MOLECULE:	V-TYPE SODIUM ATPASE CATALYTIC SUBUNIT A
86:00:00	6zks-t	3	5	53	177	6	MOLECULE:	NADH DEHYDROGENASE [UBIQUINONE] FLAVOPROTEIN 1,
87:00:00	1m90-Q	3	2.9	50	143	6	MOLECULE:	23S RRNA
88:00:00	6vq8-A	3	3.6	73	600	4	MOLECULE:	ATPASE H+-TRANSPORTING V1 SUBUNIT A
89:00:00	6zkt-t	3	5.1	53	177	6	MOLECULE:	NADH DEHYDROGENASE [UBIQUINONE] FLAVOPROTEIN 1,
90:00:00	2zxq-A	3	2.8	51	1178	0	MOLECULE:	ENDO-ALPHA-N-ACETYLGALACTOSAMINIDASE
91:00:00	3j9t-E	3	3.6	71	593	3	MOLECULE:	V-TYPE PROTON ATPASE SUBUNIT D
92:00:00	7w00-p	3	4.5	57	178	5	MOLECULE:	NADH DEHYDROGENASE [UBIQUINONE] FLAVOPROTEIN 1,
93:00:00	3cxc-O	3	2.9	50	143	6	MOLECULE:	23S RIBOSOMAL RNA
94:00:00	1vq5-P	3	2.9	50	143	6	MOLECULE:	23S RIBOSOMAL RNA
95:00:00	6vqa-B	3	3.6	70	600	3	MOLECULE:	ATPASE H+-TRANSPORTING V1 SUBUNIT A
96:00:00	1yjw-P	3	2.9	50	143	6	MOLECULE:	23S RIBOSOMAL RNA
97:00:00	6vq6-C	3	3.5	68	600	10	MOLECULE:	ATPASE H+-TRANSPORTING V1 SUBUNIT A
98:00:00	1k9m-Q	3	3.1	50	143	4	MOLECULE:	23S RRNA
99:00:00	1kc8-Q	3	2.9	50	143	6	MOLECULE:	23S RRNA
0:00	6vqb-A	3	3.6	73	600	4	MOLECULE:	ATPASE H+-TRANSPORTING V1 SUBUNIT A
1:00	7oy2-C	3	2.9	53	454	9	MOLECULE:	APPB PROTEIN
2:00	2qw6-D	3	4.4	53	86	6	MOLECULE:	AAA ATPASE, CENTRAL REGION
3:00	6zkp-t	3	5.1	54	177	6	MOLECULE:	NADH DEHYDROGENASE [UBIQUINONE] FLAVOPROTEIN 1,
4:00	5cbo-K	3	4.3	49	176	4	MOLECULE:	MBP3-16,IMMUNOGLOBULIN G-BINDING PROTEIN A
5:00	8a1a-A	3	3	51	1345	2	MOLECULE:	L1F11V1
6:00	1vq9-P	3	2.9	50	143	6	MOLECULE:	23S RIBOSOMAL RNA
7:00	3jb6-A	3	3.6	77	1196	6	MOLECULE:	RNA-DEPENDENT RNA POLYMERASE
8:00	6vq9-C	3	3.5	68	600	10	MOLECULE:	ATPASE H+-TRANSPORTING V1 SUBUNIT A
9:00	7fde-E	3	3.3	67	594	1	MOLECULE:	V-TYPE PROTON ATPASE SUBUNIT C
10:00	7bx8-A	3	3.1	59	1038	2	MOLECULE:	INTEGRAL MEMBRANE INDOLYLACETYLINOSITOL
11:00	6o7v-A	3	3.2	67	593	7	MOLECULE:	V-TYPE PROTON ATPASE SUBUNIT D
12:00	5voz-C	3	2.9	68	592	7	MOLECULE:	V-TYPE PROTON ATPASE CATALYTIC SUBUNIT A,V-TYPE P
13:00	3bge-B	3	5.5	57	163	9	MOLECULE:	PREDICTED ATPASE
14:00	6wm3-B	3	3.3	68	600	3	MOLECULE:	V-TYPE PROTON ATPASE 116 KDA SUBUNIT A ISOFORM 1

15:00	8wqb-F	3	9.3	67	627	9	MOLECULE:	CULLIN-2
16:00	8c81-B	2.9	2.7	49	539	4	MOLECULE:	PROTEIN ORM1
17:00	7xqk-B	2.9	3.6	59	276	0	MOLECULE:	GLUTATHIONE S-TRANSFERASE CLASS-MU 26 KDA ISOZYME
18:00	5iy6-U	2.9	2.7	49	170	6	MOLECULE:	DNA-DIRECTED RNA POLYMERASE II SUBUNIT RPB1
19:00	2ejq-B	2.9	3.4	65	115	2	MOLECULE:	HYPOTHETICAL PROTEIN TTHA0227
20:00	7mi8-A	2.9	3.1	66	868	9	MOLECULE:	FUSION PROTEIN OF DYNEIN AND ENDOLYSIN
21:00	7m99-C	2.9	3.8	59	257	7	MOLECULE:	TNSC
22:00	7n6i-E	2.9	4.2	61	257	7	MOLECULE:	TNIQ (HOMOLOGY MODEL)
23:00	7jlv-D	2.9	3.5	61	437	13	MOLECULE:	DISEASE RESISTANCE PROTEIN ROQ1
24:00:00	7m9c-P	2.9	3.6	57	257	5	MOLECULE:	TNSC
25:00:00	7m9a-C	2.9	3.7	59	257	7	MOLECULE:	TNSC
26:00:00	7jlv-A	2.9	3.5	61	437	13	MOLECULE:	DISEASE RESISTANCE PROTEIN ROQ1
27:00:00	1xme-A	2.9	4.2	67	557	4	MOLECULE:	CYTOCHROME C OXIDASE POLYPEPTIDE I
28:00:00	8ba0-n	2.9	4.7	55	134	7	MOLECULE:	NADH-UBIQUINONE OXIDOREDUCTASE CHAIN 3
29:00:00	8hra-B	2.9	6.4	59	835	10	MOLECULE:	ARCHAEAL ATPASE
30:00:00	3cqz-A	2.9	3.8	66	1349	9	MOLECULE:	DNA-DIRECTED RNA POLYMERASE II SUBUNIT RPB1
31:00:00	6jbq-F	2.9	3.7	57	186	7	MOLECULE:	DNA-DIRECTED RNA POLYMERASE SUBUNIT ALPHA
32:00:00	2d1l-A	2.9	3.9	57	249	2	MOLECULE:	METASTASIS SUPPRESSOR PROTEIN 1
33:00:00	6soy-A	2.9	4.1	55	323	7	MOLECULE:	ESAG6, SUBUNIT OF HETERODIMERIC TRANSFERRIN RECEP
34:00:00	8afz-A	2.9	3	58	381	5	MOLECULE:	SORTING NEXIN-1
35:00:00	5tgw-A	2.9	3.8	58	109	3	MOLECULE:	PS1
36:00:00	5wau-A	2.9	3.7	67	514	7	MOLECULE:	CYTOCHROME C OXIDASE SUBUNIT 1
37:00:00	6peq-G	2.9	4.3	55	132	2	MOLECULE:	GLUTAMATE RECEPTOR 2
38:00:00	8djk-A	2.9	3.8	56	245	7	MOLECULE:	3-HYDROXY-3-METHYLGLUTARYL-COENZYME A REDUCTASE
39:00:00	6peq-E	2.9	4.3	55	132	2	MOLECULE:	GLUTAMATE RECEPTOR 2
40:00:00	2qff-A	2.9	3.4	52	74	0	MOLECULE:	HYPOTHETICAL PROTEIN
41:00:00	1oed-A	2.9 1	0.2	57	127	12	MOLECULE:	ACETYLCHOLINE RECEPTOR PROTEIN, ALPHA CHAIN
42:00:00	1j78-A	2.9	3.4	61	433	5	MOLECULE:	VITAMIN D BINDING PROTEIN
43:00:00	1y2o-A	2.9	2.6	53	248	8	MOLECULE:	BAI1-ASSOCIATED PROTEIN 2 ISOFORM 1
44:00:00	8g6r-C	2.9	3.9	55	62	4	MOLECULE:	NSP12
45:00:00	2awy-A	2.9	3.2	58	133	10	MOLECULE:	HEMERYTHRIN-LIKE DOMAIN PROTEIN DCRH
46:00:00	8evm-A	2.9	3.1	65	235	3	MOLECULE:	CHLOROPHYLL DIMER PROTEIN DESIGNS, SPECIAL PAIR 3
47:00:00	1oed-E	2.9 1	0.1	56	128	7	MOLECULE:	ACETYLCHOLINE RECEPTOR PROTEIN, ALPHA CHAIN
48:00:00	7du0-A	2.9	3.8	51	125	4	MOLECULE:	ACRIF14
49:00:00	7o6v-A	2.9	2.7	48	71	10	MOLECULE:	VIN3-LIKE PROTEIN 2
50:00:00	7z6h-D	2.9	3.7	62	324	6	MOLECULE:	CELL CYCLE CHECKPOINT CONTROL PROTEIN RAD9A
51:00:00	3waq-A	2.9	3.7	59	134	12	MOLECULE:	HEMERYTHRIN-LIKE DOMAIN PROTEIN DCRH
52:00:00	1i4y-A	2.9	3.5	57	113	0	MOLECULE:	METHEMERYTHRIN
53:00:00	7cop-A	2.9	4.4	62	586	6	MOLECULE:	V-TYPE SODIUM ATPASE CATALYTIC SUBUNIT A
54:00:00	3j9u-C	2.9	3.7	70	593	3	MOLECULE:	V-TYPE PROTON ATPASE SUBUNIT D
55:00:00	6nuw-E	2.9	2	44	369	7	MOLECULE:	INNER KINETOCHORE SUBUNIT IML3
56:00:00	6s2u-A	2.9	2.5	51	341	4	MOLECULE:	(P)PPGPP SYNTHETASE I, SPOT/RELA
57:00:00	3vr3-C	2.9	4.3	62	584	6	MOLECULE:	V-TYPE SODIUM ATPASE CATALYTIC SUBUNIT A
58:00:00	3vr5-B	2.9	4.4	62	593	6	MOLECULE:	V-TYPE SODIUM ATPASE CATALYTIC SUBUNIT A
59:00:00	2qw6-A	2.9	2.9	47	85	4	MOLECULE:	AAA ATPASE, CENTRAL REGION
60:00:00	6qfk-A	2.9	3	51	1347	2	MOLECULE:	PEGA DOMAIN-CONTAINING PROTEIN,PEGA DOMAIN-CONTAI
61:00:00	3cme-P	2.9	3.2	50	142	4	MOLECULE:	50S RIBOSOMAL PROTEIN L2P
62:00:00	6qum-C	2.9	3.8	75	578	5	MOLECULE:	V-TYPE ATP SYNTHASE ALPHA CHAIN
63:00:00	7bvh-B	2.9	3.2	59	1035	5	MOLECULE:	INTEGRAL MEMBRANE INDOLYLACETYLINOSITOL
64:00:00	2k0n-A	2.9	2.3	50	85	8	MOLECULE:	MEDIATOR OF RNA POLYMERASE II TRANSCRIPTION
65:00:00	2k9d-A	2.9	2.3	43	44	7	MOLECULE:	PHOSPHOPROTEIN
66:00:00	6r0w-A	2.9	3.6	74	577	5	MOLECULE:	V-TYPE ATP SYNTHASE ALPHA CHAIN
67:00:00	7zme-R	2.9	2.4	49	98	8	MOLECULE:	NADH-UBIQUINONE OXIDOREDUCTASE CHAIN 1
68:00:00	7jpp-C	2.9	3.1	60	597	5	MOLECULE:	ORIGIN RECOGNITION COMPLEX SUBUNIT 1
69:00:00	6zkr-t	2.9	5.1	53	177	6	MOLECULE:	NADH DEHYDROGENASE [UBIQUINONE] FLAVOPROTEIN 1,
70:00:00	1q7y-Q	2.9	2.9	50	143	6	MOLECULE:	23S RIBOSOMAL RNA

71:00:00	7tmm-A	2.9	3.5	70	593	3	MOLECULE:	H(+)-TRANSPORTING TWO-SECTOR ATPASE
72:00:00	7ar8-n	2.9	2.7	52	109	6	MOLECULE:	NADH-UBIQUINONE OXIDOREDUCTASE CHAIN 3
73:00:00	6qld-N	2.9	2.1	45	371	9	MOLECULE:	INNER KINETOCHORE SUBUNIT MIF2
74:00:00	7tmq-A	2.9	3.9	72	591	4	MOLECULE:	H(+)-TRANSPORTING TWO-SECTOR ATPASE
75:00:00	5bw9-c	2.9	3	64	586	6	MOLECULE:	V-TYPE PROTON ATPASE CATALYTIC SUBUNIT A
76:00:00	7bvh-A	2.9	2.8	58	1035	5	MOLECULE:	INTEGRAL MEMBRANE INDOLYLACETYLINOSITOL
77:00:00	7tmo-E	2.9	4.5	66	590	5	MOLECULE:	H(+)-TRANSPORTING TWO-SECTOR ATPASE
78:00:00	5h7c-C	2.9	2.9	45	397	11	MOLECULE:	IMMUNOGLOBULIN G-BINDING PROTEIN A, DHR14
79:00:00	5bn5-A	2.9	3.7	64	562	3	MOLECULE:	V-TYPE ATP SYNTHASE ALPHA CHAIN
80:00:00	6o7w-E	2.9	3.1	69	593	7	MOLECULE:	V-TYPE PROTON ATPASE SUBUNIT D
81:00:00	5voz-E	2.9	3.1	68	592	7	MOLECULE:	V-TYPE PROTON ATPASE CATALYTIC SUBUNIT A,V-TYPE P
82:00:00	7tmq-E	2.9	4.5	66	583	5	MOLECULE:	H(+)-TRANSPORTING TWO-SECTOR ATPASE
83:00:00	8je2-D	2.9 1	0.1	68	494	3	MOLECULE:	CULLIN-2
84:00:00	7v1n-A	2.9	4.6	52	2344	6	MOLECULE:	TOXIN B
85:00:00	6vq9-B	2.9	3.5	68	600	10	MOLECULE:	ATPASE H+-TRANSPORTING V1 SUBUNIT A
86:00:00	8wqe-B	2.9	8.4	61	627	10	MOLECULE:	CULLIN-2
87:00:00	7nj0-C	2.8	3.9	65	270	3	MOLECULE:	SECURIN,SEPARIN
88:00:00	5iqc-A	2.8	3.5	59	301	2	MOLECULE:	BIFUNCTIONAL AAC/APH
89:00:00	8eov-A	2.8	2.8	47	139	9	MOLECULE:	C2HR1_4R
90:00:00	4cc9-B	2.8	2.8	50	98	6	MOLECULE:	PROTEIN VPRBP
91:00:00	4hlq-C	2.8	3.4	57	281	16	MOLECULE:	TBC1 DOMAIN FAMILY MEMBER 20
92:00:00	7m9a-A	2.8	3.6	56	257	7	MOLECULE:	TNSC
93:00:00	7m9c-O	2.8	3.8	57	257	7	MOLECULE:	TNSC
94:00:00	7m9b-A	2.8	3.8	57	257	7	MOLECULE:	TNSC
95:00:00	7m9c-C	2.8	3.9	60	257	7	MOLECULE:	TNSC
96:00:00	7m9c-A	2.8	3.6	56	257	7	MOLECULE:	TNSC
97:00:00	7m9c-B	2.8	4	61	257	7	MOLECULE:	TNSC
98:00:00	7m9b-B	2.8	4	61	257	7	MOLECULE:	TNSC
99:00:00	7m9a-B	2.8	4	61	257	7	MOLECULE:	TNSC
0:00	2qyw-A	2.8	8.9	56	96	5	MOLECULE:	VESICLE TRANSPORT THROUGH INTERACTION WITH T-SNAR
1:00	6zka-t	2.8	4.9	54	177	6	MOLECULE:	NADH-UBIQUINONE OXIDOREDUCTASE CHAIN 3
2:00	8jzl-F	2.8	3.7	55	498	9	MOLECULE:	INOSITOL PHOSPHATE PHOSPHATASE SOPB
3:00	8jzl-D	2.8	3.7	55	498	9	MOLECULE:	INOSITOL PHOSPHATE PHOSPHATASE SOPB
4:00	3rip-A	2.8	5.9	56	568	7	MOLECULE:	GAMMA-TUBULIN COMPLEX COMPONENT 4
5:00	6rfq-F	2.8	3.9	53	121	9	MOLECULE:	SUBUNIT NUAM OF NADH:UBIQUINONE OXIDOREDUCTASE (C
6:00	8jzl-C	2.8	3.7	55	498	9	MOLECULE:	INOSITOL PHOSPHATE PHOSPHATASE SOPB
7:00	5jk7-H	2.8	3.2	51	74	6	MOLECULE:	DNA DAMAGE-BINDING PROTEIN 1
8:00	1ywm-A	2.8	3	55	180	5	MOLECULE:	C PROTEIN ALPHA-ANTIGEN
9:00	6zhi-B	2.8	3.9	62	222	11	MOLECULE:	HEAT SHOCK PROTEIN 70
10:00	8hr8-E	2.8	6.1	57	833	9	MOLECULE:	ARCHAEAL ATPASE
11:00	8jzl-A	2.8	3.7	55	498	9	MOLECULE:	INOSITOL PHOSPHATE PHOSPHATASE SOPB
12:00	8bms-C	2.8	5.4	62	263	6	MOLECULE:	ENERGY-COUPLING FACTOR TRANSPORTER ATP-BINDING PR
13:00	8jzl-B	2.8	3.7	55	498	9	MOLECULE:	INOSITOL PHOSPHATE PHOSPHATASE SOPB
14:00	5oqk-A	2.8	2.5	52	147	4	MOLECULE:	VOLTAGE-GATED HYDROGEN CHANNEL 1
15:00	1k04-A	2.8	3.5	58	142	9	MOLECULE:	FOCAL ADHESION KINASE 1
16:00	3g6i-A	2.8	3.4	62	200	8	MOLECULE:	PUTATIVE OUTER MEMBRANE PROTEIN, PART OF CARBOHYD
17:00	8hdv-A	2.8	3.1	52	137	6	MOLECULE:	DE NOVO DESIGN CAVITATED PROTEIN
18:00	2hmz-A	2.8	3.7	59	113	3	MOLECULE:	HEMERYTHRIN
19:00	5x5y-F	2.8	3.7	64	351	3	MOLECULE:	PROBABLE ATP-BINDING COMPONENT OF ABC TRANSPORTER
20:00	1dow-A	2.8	3.7	58	205	5	MOLECULE:	ALPHA-CATENIN
21:00	5lqx-Y	2.8	2.9	55	122	9	MOLECULE:	ATP SYNTHASE SUBUNIT F
22:00	6v3f-A	2.8	4.1	69	1234	3	MOLECULE:	NPC1-LIKE INTRACELLULAR CHOLESTEROL TRANSPORTER 1
23:00	7qsd-n	2.8	3	52	172	6	MOLECULE:	NADH-UBIQUINONE OXIDOREDUCTASE CHAIN 3
24:00:00	8jzl-E	2.8	3.7	55	498	9	MOLECULE:	INOSITOL PHOSPHATE PHOSPHATASE SOPB
25:00:00	8buy-B	2.8	3.4	56	126	5	MOLECULE:	GRANULE ASSOCIATED RAC AND RHOG EFFECTOR PROTEIN
26:00:00	6xby-A	2.8	4.4	64	597	5	MOLECULE:	V-TYPE PROTON ATPASE CATALYTIC SUBUNIT A

27:00:00	6xbw-C	2.8	4.3	63	597	5	MOLECULE:	V-TYPE PROTON ATPASE CATALYTIC SUBUNIT A
28:00:00	7oiw-A	2.8	2.6	51	327	8	MOLECULE:	GTP PYROPHOSPHOKINASE
29:00:00	7fdc-E	2.8	3.2	63	594	8	MOLECULE:	YEAST VACUOLAR ATPASE A SUBUNIT
30:00:00	7dqe-C	2.8	4.4	62	584	6	MOLECULE:	V-TYPE SODIUM ATPASE CATALYTIC SUBUNIT A
31:00:00	5lnk-t	2.8	3	52	166	6	MOLECULE:	MITOCHONDRIAL COMPLEX I, 51 KDA SUBUNIT
32:00:00	6wm2-A	2.8	3.7	73	600	4	MOLECULE:	V-TYPE PROTON ATPASE SUBUNIT E 1
33:00:00	5ze9-B	2.8	3.6	72	592	4	MOLECULE:	V-TYPE SODIUM ATPASE CATALYTIC SUBUNIT A
34:00:00	6vqa-C	2.8	3.1	65	600	11	MOLECULE:	ATPASE H+-TRANSPORTING V1 SUBUNIT A
35:00:00	8a19-A	2.8	3	51	1345	2	MOLECULE:	L1E4V1
36:00:00	8pql-H	2.8	9.6	64	597	13	MOLECULE:	UBIQUITIN-CONJUGATING ENZYME E2 R2
37:00:00	6wlz-C	2.8	3.6	73	600	4	MOLECULE:	V-TYPE PROTON ATPASE CATALYTIC SUBUNIT A
38:00:00	5h76-A	2.8	4.2	55	197	7	MOLECULE:	DARPIN,IMMUNOGLOBULIN G-BINDING PROTEIN A
39:00:00	6aji-A	2.8	4.7	59	873	10	MOLECULE:	DRUG EXPORTERS OF THE RND SUPERFAMILY-LIKE PROTEI
40:00:00	6rx4-A	2.8	4.3	55	474	5	MOLECULE:	CYTOCHROME BD-I UBIQUINOL OXIDASE SUBUNIT 1
41:00:00	6zl1-C	2.8	3.3	61	122	2	MOLECULE:	ALBUMIN
42:00:00	7k8a-A	2.8	2.7	56	722	7	MOLECULE:	DRUG EXPORTERS OF THE RND SUPERFAMILY-LIKE PROTEI
43:00:00	3j0j-C	2.8	3.6	71	561	6	MOLECULE:	V-TYPE ATP SYNTHASE ALPHA CHAIN
44:00:00	8b9z-n	2.8	4.5	55	134	7	MOLECULE:	NADH-UBIQUINONE OXIDOREDUCTASE CHAIN 3
45:00:00	6r0z-C	2.8	3.7	73	577	4	MOLECULE:	V-TYPE ATP SYNTHASE ALPHA CHAIN
46:00:00	6wlz-A	2.8	3.6	72	600	3	MOLECULE:	V-TYPE PROTON ATPASE CATALYTIC SUBUNIT A
47:00:00	6wlz-B	2.8	3.3	68	600	4	MOLECULE:	V-TYPE PROTON ATPASE CATALYTIC SUBUNIT A
48:00:00	5d80-A	2.8	3.1	67	591	6	MOLECULE:	V-TYPE PROTON ATPASE CATALYTIC SUBUNIT A
49:00:00	7tmo-A	2.8	3.8	62	592	10	MOLECULE:	H(+)-TRANSPORTING TWO-SECTOR ATPASE
50:00:00	6s2v-B	2.8	3.5	59	326	8	MOLECULE:	(P)PPGPP SYNTHETASE I, SPOT/RELA
51:00:00	7khr-A	2.8	2.9	62	589	2	MOLECULE:	V-TYPE PROTON ATPASE CATALYTIC SUBUNIT A
52:00:00	7w4c-p	2.8	4.7	57	178	0	MOLECULE:	NADH DEHYDROGENASE [UBIQUINONE] FLAVOPROTEIN 1,
53:00:00	5uql-A	2.8	3.7	64	542	9	MOLECULE:	TOXIN A
54:00:00	7fdb-E	2.8	3.8	73	594	3	MOLECULE:	YEAST VACUOLAR ATPASE A SUBUNIT
55:00:00	7d5i-A	2.8	3.1	50	443	4	MOLECULE:	CYTOCHROME D UBIQUINOL OXIDASE SUBUNIT 1
56:00:00	3j9t-A	2.8	2.8	63	593	8	MOLECULE:	V-TYPE PROTON ATPASE SUBUNIT D
57:00:00	6xby-C	2.8	3.3	67	583	1	MOLECULE:	V-TYPE PROTON ATPASE CATALYTIC SUBUNIT A
58:00:00	5h78-B	2.8	9.1	70	192	7	MOLECULE:	CAMP-DEPENDENT PROTEIN KINASE TYPE II-ALPHA REGUL
59:00:00	7cor-C	2.8	3.2	66	584	8	MOLECULE:	V-TYPE SODIUM ATPASE CATALYTIC SUBUNIT A
60:00:00	6xbw-A	2.8	4.4	59	589	5	MOLECULE:	V-TYPE PROTON ATPASE CATALYTIC SUBUNIT A
61:00:00	5d80-a	2.8	3.5	72	591	6	MOLECULE:	V-TYPE PROTON ATPASE CATALYTIC SUBUNIT A
62:00:00	6bzh-B	2.7	1.9	38	192	8	MOLECULE:	PROBABLE ATP-DEPENDENT RNA HELICASE DDX58
63:00:00	6dan-B	2.7	3.3	55	328	7	MOLECULE:	PHDJ
64:00:00	7tjk-F	2.7	2.7	52	262	8	MOLECULE:	ORIGIN RECOGNITION COMPLEX SUBUNIT 1
65:00:00	8bmw-N	2.7	3.6	55	251	7	MOLECULE:	CRISPR-ASSOCIATED SMALL SUBUNIT PROTEIN (TYPE III
66:00:00	7m9b-F	2.7	3.8	58	257	7	MOLECULE:	TNSC
67:00:00	7m9c-F	2.7	3.7	58	257	7	MOLECULE:	TNSC
68:00:00	7m99-G	2.7	3.3	55	257	7	MOLECULE:	TNSC
69:00:00	7jlv-B	2.7	3.6	61	437	11	MOLECULE:	DISEASE RESISTANCE PROTEIN ROQ1
70:00:00	7m9a-F	2.7	3.8	58	257	7	MOLECULE:	TNSC
71:00:00	7m99-H	2.7	3.3	55	257	7	MOLECULE:	TNSC
72:00:00	7n6i-J	2.7	3.3	55	257	7	MOLECULE:	TNIQ (HOMOLOGY MODEL)
73:00:00	7n6i-I	2.7	3.3	55	257	7	MOLECULE:	TNIQ (HOMOLOGY MODEL)
74:00:00	7n6i-G	2.7	3.3	55	257	7	MOLECULE:	TNIQ (HOMOLOGY MODEL)
75:00:00	7m99-B	2.7	3.3	55	257	7	MOLECULE:	TNSC
76:00:00	7n6i-F	2.7	3.8	58	257	7	MOLECULE:	TNIQ (HOMOLOGY MODEL)
77:00:00	8gyw-A	2.7	3	61	380	7	MOLECULE:	CHOLINE/ETHANOLAMINEPHOSPHOTRANSFERASE 1
78:00:00	5l1a-A	2.7	2.8	54	108	7	MOLECULE:	UNCHARACTERIZED PROTEIN
79:00:00	7uni-C	2.7	3.5	53	242	2	MOLECULE:	SP2-ZNPPAM DESIGNED CHLOROPHYLL DIMER PROTEIN
80:00:00	8j1x-A	2.7	3.8	54	174	6	MOLECULE:	NEAR-INFRARED FLUORESCENT PROTEIN
81:00:00	3dpn-A	2.7	1.9	48	537	13	MOLECULE:	PROTEIN CT_858
82:00:00	6y93-A	2.7	3.1	53	105	6	MOLECULE:	NUCLEOID OCCLUSION PROTEIN

83:00:00	5h77-A	2.7	5	51	84	4	MOLECULE:	CAMP-DEPENDENT PROTEIN KINASE TYPE II-ALPHA REGUL
84:00:00	7ar9-N	2.7	4.3	60	375	13	MOLECULE:	ND3
85:00:00	3h0g-A	2.7	3.7	64	1496	5	MOLECULE:	DNA-DIRECTED RNA POLYMERASE II SUBUNIT RPB1
86:00:00	4by1-A	2.7	3.8	67	1427	9	MOLECULE:	DNA-DIRECTED RNA POLYMERASE II SUBUNIT RPB1
87:00:00	7opb-D	2.7	2.6	43	55	5	MOLECULE:	INTERLEUKIN-7 RECEPTOR SUBUNIT ALPHA
88:00:00	5ul2-A	2.7	4	71	731	1	MOLECULE:	OXSB PROTEIN
89:00:00	1sfo-A	2.7	3.5	66	1395	5	MOLECULE:	RNA STRAND
90:00:00	6xss-A	2.7	3.6	70	260	4	MOLECULE:	C4_NAT_HFUSE-7900
91:00:00	6rfr-C	2.7	4.3	69	438	7	MOLECULE:	SUBUNIT NUAM OF NADH:UBIQUINONE OXIDOREDUCTASE (C
92:00:00	8ga6-A	2.7	6.8	58	476	5	MOLECULE:	THR6
93:00:00	6z5l-A	2.7	5.8	63	251	5	MOLECULE:	MATRIX PROTEIN 1
94:00:00	6zkq-t	2.7	5.1	53	177	6	MOLECULE:	NADH DEHYDROGENASE [UBIQUINONE] FLAVOPROTEIN 1,
95:00:00	7ctt-C	2.7	3.9	55	63	4	MOLECULE:	NSP12
96:00:00	4h6h-B	2.7	3.1	51	80	8	MOLECULE:	FIBRINOGEN-BINDING PROTEIN
97:00:00	5luf-W	2.7	3	53	72	0	MOLECULE:	CYTOCHROME B-C1 COMPLEX SUBUNIT 1, MITOCHONDRIAL
98:00:00	8a1g-A	2.7	4.2	53	218	8	MOLECULE:	SORTING NEXIN-1
99:00:00	4up6-A	2.7	3.4	57	114	7	MOLECULE:	DIACYLGLYCEROL KINASE
0:00	1wgw-A	2.7	2.9	54	99	7	MOLECULE:	SIGNAL RECOGINITION PARTICLE 54
1:00	2p61-A	2.7	3.7	56	114	5	MOLECULE:	HYPOTHETICAL PROTEIN TM_1646
2:00	8e55-A	2.7	3.2	59	195	0	MOLECULE:	SG135
3:00	3h74-A	2.7	3.6	64	278	6	MOLECULE:	PYRIDOXAL KINASE
4:00	7dfh-C	2.7	4.5	55	63	5	MOLECULE:	RNA-DIRECTED RNA POLYMERAS
5:00	4nsr-E	2.7	3.1	56	89	4	MOLECULE:	IMMUNITY PROTEIN
6:00	4nsr-A	2.7	3.1	56	89	4	MOLECULE:	IMMUNITY PROTEIN
7:00	8ub3-k	2.7	4	58	224	3	MOLECULE:	DPHF7 FILAMENT
8:00	2ozn-B	2.7	3.9	56	131	4	MOLECULE:	O-GLCNACASE NAGJ
9:00	4uis-C	2.7	3.9	69	196	0	MOLECULE:	GAMMA-SECRETASE
10:00	7sb2-A	2.7	3.8	57	299	5	MOLECULE:	GLDM
11:00	5nvs-A	2.7	3.3	62	932	0	MOLECULE:	DYNEIN HEAVY CHAIN
12:00	1i5n-D	2.7	3.6	60	129	3	MOLECULE:	CHEMOTAXIS PROTEIN CHEA
13:00	3o42-A	2.7	3.8	55	197	9	MOLECULE:	GP41-5
14:00	5i9k-A	2.7	3.2	56	123	9	MOLECULE:	MICROSOMAL GLUTATHIONE S-TRANSFERASE 1
15:00	2qup-A	2.7	2.9	57	119	11	MOLECULE:	BH1478 PROTEIN
16:00	2e2h-A	2.7	4	68	1398	7	MOLECULE:	5'-R(*AP*UP*CP*GP*AP*GP*AP*GP*GP*A)-3'
17:00	5knb-B	2.7	4.3	62	592	6	MOLECULE:	V-TYPE SODIUM ATPASE CATALYTIC SUBUNIT A
18:00	6o7x-A	2.7	3.9	61	593	3	MOLECULE:	V-TYPE PROTON ATPASE SUBUNIT C
19:00	6vq7-C	2.7	4.5	65	600	5	MOLECULE:	ATPASE H+-TRANSPORTING V1 SUBUNIT A
20:00	7cos-A	2.7	3	59	586	5	MOLECULE:	V-TYPE SODIUM ATPASE CATALYTIC SUBUNIT A
21:00	7zm7-R	2.7	2.6	50	98	8	MOLECULE:	NADH-UBIQUINONE OXIDOREDUCTASE CHAIN 1
22:00	7zmh-R	2.7	2.4	49	98	6	MOLECULE:	NADH-UBIQUINONE OXIDOREDUCTASE CHAIN 1
23:00	6zie-A	2.7	4	56	123	5	MOLECULE:	CMPX-383B
24:00:00	7khr-C	2.7	3.6	72	600	4	MOLECULE:	V-TYPE PROTON ATPASE CATALYTIC SUBUNIT A
25:00:00	7fde-A	2.7	3.6	72	594	1	MOLECULE:	V-TYPE PROTON ATPASE SUBUNIT C
26:00:00	5knd-C	2.7	3.6	72	586	4	MOLECULE:	V-TYPE SODIUM ATPASE CATALYTIC SUBUNIT A
27:00:00	7dqc-B	2.7	3.7	71	586	4	MOLECULE:	V-TYPE SODIUM ATPASE CATALYTIC SUBUNIT A
28:00:00	7zmg-R	2.7	2.4	50	98	6	MOLECULE:	NADH-UBIQUINONE OXIDOREDUCTASE CHAIN 1
29:00:00	6aun-A	2.7	5.1	61	618	8	MOLECULE:	PLA2G6, IPLA2BETA
30:00:00	3ikj-A	2.7	3.9	74	513	8	MOLECULE:	V-TYPE ATP SYNTHASE ALPHA CHAIN
31:00:00	7dh0-f	2.7	3.5	52	167	8	MOLECULE:	NADH-UBIQUINONE OXIDOREDUCTASE CHAIN 2
32:00:00	6xbw-B	2.7	3.5	72	590	3	MOLECULE:	V-TYPE PROTON ATPASE CATALYTIC SUBUNIT A
33:00:00	3pvs-A	2.7	3.4	57	414	11	MOLECULE:	REPLICATION-ASSOCIATED RECOMBINATION PROTEIN A
34:00:00	6zl1-D	2.7	2.8	51	120	4	MOLECULE:	ALBUMIN
35:00:00	7dqd-B	2.7	3.1	65	584	8	MOLECULE:	V-TYPE SODIUM ATPASE CATALYTIC SUBUNIT A
36:00:00	7vz8-p	2.7	4.7	57	178	0	MOLECULE:	NADH DEHYDROGENASE [UBIQUINONE] IRON-SULFUR PROTE
37:00:00	7upp-B	2.7	3.8	81	309	9	MOLECULE:	DHT03 PROTEIN A
38:00:00	6oq7-A	2.7	4.3	59	541	7	MOLECULE:	TOXIN B

39:00:00	7fdc-A	2.7	3.1	66	594	5	MOLECULE:	YEAST VACUOLAR ATPASE A SUBUNIT
40:00:00	5d80-c	2.7	3.6	62	589	10	MOLECULE:	V-TYPE PROTON ATPASE CATALYTIC SUBUNIT A
41:00:00	3i72-A	2.7	3.1	65	514	2	MOLECULE:	A-TYPE ATP SYNTHASE CATALYTIC SUBUNIT A
42:00:00	5voy-A	2.7	3	62	592	3	MOLECULE:	V-TYPE PROTON ATPASE CATALYTIC SUBUNIT A,V-TYPE P
43:00:00	6qum-A	2.7	3.7	71	578	3	MOLECULE:	V-TYPE ATP SYNTHASE ALPHA CHAIN
44:00:00	5bw9-C	2.7	3.1	61	589	5	MOLECULE:	V-TYPE PROTON ATPASE CATALYTIC SUBUNIT A
45:00:00	3vr4-B	2.7	4	65	593	8	MOLECULE:	V-TYPE SODIUM ATPASE CATALYTIC SUBUNIT A
46:00:00	7arc-W	2.6	3	54	127	7	MOLECULE:	PSST
47:00:00	8wo8-A	2.6	3	53	463	2	MOLECULE:	PROBABLE RIBONUCLEASE FAU-1
48:00:00	5cwf-A	2.6	3.2	54	177	2	MOLECULE:	DESIGNED HELICAL REPEAT PROTEIN
49:00:00	5h75-D	2.6	2.9	43	225	9	MOLECULE:	MERSACIDIN DECARBOXYLASE,IMMUNOGLOBULIN G-BINDING
50:00:00	1ixs-A	2.6	3.3	45	50	11	MOLECULE:	HOLLIDAY JUNCTION DNA HELICASE RUVA
51:00:00	8jh7-D	2.6	3.3	55	431	9	MOLECULE:	ANTI-BRIL FAB HEAVY CHAIN
52:00:00	2fm8-C	2.6	3.4	62	220	3	MOLECULE:	SURFACE PRESENTATION OF ANTIGENS PROTEIN SPAK
53:00:00	7m9b-K	2.6	3.7	59	257	7	MOLECULE:	TNSC
54:00:00	7n6i-C	2.6	3.3	55	257	7	MOLECULE:	TNIQ (HOMOLOGY MODEL)
55:00:00	7n6i-D	2.6	3.3	55	257	7	MOLECULE:	TNIQ (HOMOLOGY MODEL)
56:00:00	7m99-E	2.6	3.3	55	257	7	MOLECULE:	TNSC
57:00:00	7m9c-K	2.6	3.3	56	257	5	MOLECULE:	TNSC
58:00:00	7m9a-K	2.6	3.7	59	257	7	MOLECULE:	TNSC
59:00:00	7jlv-G	2.6	3.5	59	437	12	MOLECULE:	DISEASE RESISTANCE PROTEIN ROQ1
60:00:00	6mbz-A	2.6	9.7	56	536	5	MOLECULE:	SIGNAL TRANSDUCER AND ACTIVATOR OF TRANSCRIPTION
61:00:00	4v3i-A	2.6	9.9	57	153	0	MOLECULE:	VCA0115
62:00:00	7a23-d	2.6	2.8	52	66	6	MOLECULE:	51KDA
63:00:00	6orb-A	2.6	3.2	64	3352	6	MOLECULE:	MIDASIN
64:00:00	2j68-A	2.6	4.1	55	680	5	MOLECULE:	BACTERIAL DYNAMIN-LIKE PROTEIN
65:00:00	3c4a-A	2.6	3.3	68	365	10	MOLECULE:	PROBABLE TRYPTOPHAN HYDROXYLASE VIOD
66:00:00	8tl7-Z	2.6	2.8	63	694	8	MOLECULE:	COMPUTATIONALLY DESIGNED PROTEIN
67:00:00	5j0l-A	2.6	2.7	53	130	2	MOLECULE:	DESIGNED PROTEIN 3L6HC2_2
68:00:00	4roe-A	2.6	3.1	57	307	7	MOLECULE:	TRANSCRIPTION FACTOR IIIB 50 KDA SUBUNIT
69:00:00	7os0-A	2.6	3.9	60	1130	3	MOLECULE:	CAS13A
70:00:00	6fh1-A	2.6	3.5	65	353	9	MOLECULE:	PROTEIN-ARGININE KINASE
71:00:00	8v2d-A	2.6	9.8	62	307	8	MOLECULE:	O43_129 COMPONENT B
72:00:00	5vhx-E	2.6	4.1	58	179	5	MOLECULE:	GLUTAMATE RECEPTOR 2,GERM CELL-SPECIFIC GENE 1-LI
73:00:00	8ub3-H	2.6	4.1	58	224	3	MOLECULE:	DPHF7 FILAMENT
74:00:00	1l2f-A	2.6	2.8	50	344	10	MOLECULE:	N UTILIZATION SUBSTANCE PROTEIN A
75:00:00	1lot-A	2.6	3.3	60	436	5	MOLECULE:	VITAMIN D-BINDING PROTEIN
76:00:00	8x6g-H	2.6	4.2	63	244	5	MOLECULE:	DNA-DIRECTED RNA POLYMERASE SUBUNIT ALPHA
77:00:00	3if8-B	2.6	3.6	65	237	9	MOLECULE:	PROTEIN ZWILCH HOMOLOG
78:00:00	4nsr-D	2.6	3.1	56	92	4	MOLECULE:	IMMUNITY PROTEIN
79:00:00	2y1d-A	2.6	4.2	70	359	11	MOLECULE:	1-DEOXY-D-XYLULOSE 5-PHOSPHATE REDUCTOISOMERASE
80:00:00	8ub3-C	2.6	4.1	58	224	3	MOLECULE:	DPHF7 FILAMENT
81:00:00	2occ-A	2.6	3.2	69	514	3	MOLECULE:	CYTOCHROME C OXIDASE
82:00:00	1nze-A	2.6	4.7	60	112	5	MOLECULE:	OXYGEN-EVOLVING ENHANCER PROTEIN 3
83:00:00	2fu2-A	2.6	3.4	47	78	11	MOLECULE:	HYPOTHETICAL PROTEIN SPY2152
84:00:00	6wiq-A	2.6	3.6	54	80	4	MOLECULE:	SARS-COV-2 NSP7
85:00:00	7tmp-E	2.6	4.4	62	590	5	MOLECULE:	H(+)-TRANSPORTING TWO-SECTOR ATPASE
86:00:00	3vr6-B	2.6	4.3	62	592	6	MOLECULE:	V-TYPE SODIUM ATPASE CATALYTIC SUBUNIT A
87:00:00	5o31-n	2.6	3	53	166	8	MOLECULE:	NADH-UBIQUINONE OXIDOREDUCTASE CHAIN 3
88:00:00	5tsj-B	2.6	3.5	70	575	4	MOLECULE:	V-TYPE ATP SYNTHASE ALPHA CHAIN
89:00:00	3se0-A	2.6	3.7	74	519	8	MOLECULE:	V-TYPE ATP SYNTHASE ALPHA CHAIN
90:00:00	5h76-C	2.6	4.3	53	197	8	MOLECULE:	DARPIN,IMMUNOGLOBULIN G-BINDING PROTEIN A
91:00:00	7cor-B	2.6	3.8	72	586	7	MOLECULE:	V-TYPE SODIUM ATPASE CATALYTIC SUBUNIT A
92:00:00	7ar7-n	2.6	2.9	52	109	6	MOLECULE:	NADH-UBIQUINONE OXIDOREDUCTASE CHAIN 3
93:00:00	6wm2-B	2.6	3.7	73	600	4	MOLECULE:	V-TYPE PROTON ATPASE SUBUNIT E 1
94:00:00	5gar-B	2.6	3.7	73	575	4	MOLECULE:	V-TYPE ATP SYNTHASE ALPHA CHAIN

95:00:00	6vq7-A	2.6	3.6	72	600	3	MOLECULE:	ATPASE H+-TRANSPORTING V1 SUBUNIT A
96:00:00	7fdc-C	2.6	3.8	73	594	3	MOLECULE:	YEAST VACUOLAR ATPASE A SUBUNIT
97:00:00	2r9g-N	2.6	6	58	180	9	MOLECULE:	AAA ATPASE, CENTRAL REGION
98:00:00	5f3p-A	2.6	4.3	56	193	7	MOLECULE:	PUTATIVE UNCHARACTERIZED PROTEIN
99:00:00	5cbo-F	2.6	2.9	50	176	6	MOLECULE:	MBP3-16,IMMUNOGLOBULIN G-BINDING PROTEIN A
0:00	5cbo-H	2.6	4.2	49	176	4	MOLECULE:	MBP3-16,IMMUNOGLOBULIN G-BINDING PROTEIN A
1:00	8q1y-n	2.6	3	52	171	6	MOLECULE:	NADH-UBIQUINONE OXIDOREDUCTASE CHAIN 3
2:00	6wm4-A	2.6	3.6	73	600	4	MOLECULE:	V-TYPE PROTON ATPASE 116 KDA SUBUNIT A ISOFORM 1
3:00	5bn4-A	2.6	3.7	63	567	3	MOLECULE:	V-TYPE ATP SYNTHASE ALPHA CHAIN
4:00	7tmr-C	2.6	3.9	73	593	4	MOLECULE:	H(+)-TRANSPORTING TWO-SECTOR ATPASE
5:00	3pvs-B	2.6	5.1	58	417	10	MOLECULE:	REPLICATION-ASSOCIATED RECOMBINATION PROTEIN A
6:00	3m4y-A	2.6	3.9	70	558	1	MOLECULE:	V-TYPE ATP SYNTHASE ALPHA CHAIN
7:00	6yxa-A	2.6	2.6	48	536	8	MOLECULE:	GTP PYROPHOSPHOKINASE
8:00	5gas-C	2.6	3.3	67	576	4	MOLECULE:	V-TYPE ATP SYNTHASE ALPHA CHAIN
9:00	5h7c-A	2.6	2.7	41	400	12	MOLECULE:	IMMUNOGLOBULIN G-BINDING PROTEIN A, DHR14
10:00	2r9g-B	2.6	6	58	180	9	MOLECULE:	AAA ATPASE, CENTRAL REGION
11:00	3w3a-J	2.6	3.2	68	577	4	MOLECULE:	V-TYPE ATP SYNTHASE ALPHA CHAIN
12:00	5voy-C	2.6	3.9	72	592	3	MOLECULE:	V-TYPE PROTON ATPASE CATALYTIC SUBUNIT A,V-TYPE P
13:00	8q7r-H	2.6 1	0.2	68	593	10	MOLECULE:	CULLIN-2
14:00	4dmw-A	2.6	3.5	67	535	6	MOLECULE:	TOXIN A
15:00	7dqe-A	2.6	3.3	65	584	5	MOLECULE:	V-TYPE SODIUM ATPASE CATALYTIC SUBUNIT A
16:00	6wm4-C	2.6	3.4	67	600	1	MOLECULE:	V-TYPE PROTON ATPASE 116 KDA SUBUNIT A ISOFORM 1
17:00	6fes-A	2.6	4.7	65	365	12	MOLECULE:	D12_BRIC2, A SYNTHETIC PROTEIN,D12_BRIC2, A SYNTH
18:00	8vei-A	2.6	3.7	47	117	9	MOLECULE:	CHD_R1
19:00	7deg-A	2.5	3.8	72	581	3	MOLECULE:	CYTOCHROME C OXIDASE SUBUNIT I
20:00	7qe7-A	2.5	4	69	1648	9	MOLECULE:	ANAPHASE-PROMOTING COMPLEX SUBUNIT 10
21:00	2ve7-B	2.5	2.2	36	303	6	MOLECULE:	KINETOCHORE PROTEIN HEC1, KINETOCHORE PROTEIN SPC
22:00	7but-A	2.5	6	57	142	2	MOLECULE:	ACINIFORM SPIDROIN
23:00	7m9c-E	2.5	3.4	58	257	5	MOLECULE:	TNSC
24:00:00	4wvm-B	2.5	4.2	67	616	4	MOLECULE:	STONUSTOXIN SUBUNIT ALPHA
25:00:00	7m99-A	2.5	3.3	54	257	7	MOLECULE:	TNSC
26:00:00	7l7v-A	2.5	4.4	52	121	0	MOLECULE:	PROBABLE DISEASE RESISTANCE PROTEIN AT5G66900
27:00:00	5wd8-A	2.5	9.8	57	90	0	MOLECULE:	UNCHARACTERIZED PROTEIN
28:00:00	8bcs-A	2.5	4.5	41	49	7	MOLECULE:	CC-HP1.0
29:00:00	8vjn-B	2.5	3.6	39	51	0	MOLECULE:	ENCAPSULIN NANOCOMPARTMENT CARGO PROTEIN ENCD
30:00:00	6w2r-A	2.5	4.1	70	218	7	MOLECULE:	JUNCTION 19 DHR54-DHR79
31:00:00	7lsv-B	2.5	3.5	60	158	7	MOLECULE:	CALMODULIN-DEPENDENT PROTEIN KINASE
32:00:00	3gtg-A	2.5	4	68	1442	9	MOLECULE:	DNA-DIRECTED RNA POLYMERASE II SUBUNIT RPB1
33:00:00	6wbf-A	2.5	3.3	66	344	6	MOLECULE:	PANNEXIN-1
34:00:00	1nu7-D	2.5	3.1	54	282	13	MOLECULE:	THROMBIN LIGHT CHAIN
35:00:00	1pc6-A	2.5	4.3	56	141	4	MOLECULE:	PROTEIN NINB
36:00:00	6sdk-D	2.5	3.4	58	199	7	MOLECULE:	STAGE 0 SPORULATION PROTEIN J
37:00:00	6hra-B	2.5	4	68	679	4	MOLECULE:	POTASSIUM-TRANSPORTING ATPASE POTASSIUM-BINDING S
38:00:00	1sxj-D	2.5	4	57	328	9	MOLECULE:	ACTIVATOR 1 95 KDA SUBUNIT
39:00:00	4bzj-C	2.5	8.5	55	693	2	MOLECULE:	PROTEIN TRANSPORT PROTEIN SEC31
40:00:00	2p5t-E	2.5	3.8	54	95	6	MOLECULE:	FRAGMENT OF PEZA HELIX-TURN-HELIX MOTIF
41:00:00	7wtu-K	2.5 1	0.6	56	1022	14	MOLECULE:	18S RRNA
42:00:00	7ytj-B	2.5	3.2	55	102	7	MOLECULE:	VACUOLAR TRANSPORTER CHAPERONE 4
43:00:00	8i13-H	2.5	9.7	53	280	6	MOLECULE:	SMC6 ISOFORM 1
44:00:00	1lq7-A	2.5	3	48	67	6	MOLECULE:	ALPHA3W
45:00:00	7jzu-A	2.5	2.6	45	55	4	MOLECULE:	LCB1
46:00:00	4y9j-B	2.5	5.5	61	593	10	MOLECULE:	PROTEIN ACDH-11, ISOFORM B
47:00:00	6rfl-C	2.5 1	0.8	53	304	4	MOLECULE:	DNA-DEPENDENT RNA POLYMERASE SUBUNIT RPO132
48:00:00	2c5i-T	2.5	9.7	55	94	9	MOLECULE:	T-SNARE AFFECTING A LATE GOLGI COMPARTMENT
49:00:00	7jzl-E	2.5	2.6	45	55	4	MOLECULE:	SPIKE GLYCOPROTEIN
50:00:00	7wtt-K	2.5 1	0.4	70	1022	11	MOLECULE:	18S RRNA

51:00:00	7jzl-G	2.5	2.6	45	55	4	MOLECULE:	SPIKE GLYCOPROTEIN
52:00:00	5e85-A	2.5	5.5	53	235	6	MOLECULE:	78 KDA GLUCOSE-REGULATED PROTEIN
53:00:00	8ub3-A	2.5	4.1	58	224	3	MOLECULE:	DPHF7 FILAMENT
54:00:00	8ub3-J	2.5	4.1	58	224	3	MOLECULE:	DPHF7 FILAMENT
55:00:00	5j4o-A	2.5	9.9	57	222	11	MOLECULE:	SPECTRIN ALPHA CHAIN, ERYTHROCYTIC 1
56:00:00	2pno-C	2.5	3.5	54	146	2	MOLECULE:	LEUKOTRIENE C4 SYNTHASE
57:00:00	7p14-A	2.5	3.2	53	329	9	MOLECULE:	XK-RELATED PROTEIN
58:00:00	5wuq-A	2.5	3.3	56	158	5	MOLECULE:	ECF RNA POLYMERASE SIGMA FACTOR SIGW
59:00:00	1kw2-A	2.5	4	59	455	8	MOLECULE:	VITAMIN D-BINDING PROTEIN
60:00:00	8j8y-A	2.5	3.3	56	129	7	MOLECULE:	IMMUNODOMINANT MEMBRANE PROTEIN
61:00:00	7nd2-A	2.5	3.9	57	330	5	MOLECULE:	PROTEIN PHOSPHATASE 1 REGULATORY SUBUNIT 21
62:00:00	6l82-A	2.5	2.9	49	98	2	MOLECULE:	SPINDLE POLE BODY COMPONENT
63:00:00	8vjn-D	2.5	3.8	40	52	0	MOLECULE:	ENCAPSULIN NANOCOMPARTMENT CARGO PROTEIN ENCD
64:00:00	7ytj-C	2.5	3.4	55	102	7	MOLECULE:	VACUOLAR TRANSPORTER CHAPERONE 4
65:00:00	1au1-A	2.5	4.3	41	166	7	MOLECULE:	INTERFERON-BETA
66:00:00	8euy-b	2.5	2.9	52	120	12	MOLECULE:	RNA (1095-MER)
67:00:00	6rth-A	2.5	4	56	504	4	MOLECULE:	RTX TOXIN AND CA2+-BINDING PROTEIN
68:00:00	5y5x-C	2.5	4.5	60	576	7	MOLECULE:	V-TYPE ATP SYNTHASE ALPHA CHAIN
69:00:00	2r9g-G	2.5	6	58	179	9	MOLECULE:	AAA ATPASE, CENTRAL REGION
70:00:00	2r9g-K	2.5	6	61	180	8	MOLECULE:	AAA ATPASE, CENTRAL REGION
71:00:00	2r9g-F	2.5	6	58	180	9	MOLECULE:	AAA ATPASE, CENTRAL REGION
72:00:00	7r43-n	2.5	3	52	172	6	MOLECULE:	NADH-UBIQUINONE OXIDOREDUCTASE CHAIN 3
73:00:00	2r9g-J	2.5	6.6	60	181	8	MOLECULE:	AAA ATPASE, CENTRAL REGION
74:00:00	8ovt-B	2.5	3.6	68	284	7	MOLECULE:	YEGT GLYCOSYLTRANSFERASE
75:00:00	2r9g-I	2.5	6	58	178	9	MOLECULE:	AAA ATPASE, CENTRAL REGION
76:00:00	6zkk-t	2.5	4.9	53	177	6	MOLECULE:	NADH DEHYDROGENASE [UBIQUINONE] FLAVOPROTEIN 1,
77:00:00	6r0y-B	2.5	4	75	577	7	MOLECULE:	V-TYPE ATP SYNTHASE ALPHA CHAIN
78:00:00	2r9g-A	2.5	6	58	180	9	MOLECULE:	AAA ATPASE, CENTRAL REGION
79:00:00	3qia-A	2.5	3.8	71	564	7	MOLECULE:	V-TYPE ATP SYNTHASE ALPHA CHAIN
80:00:00	2r9g-M	2.5	6	58	179	9	MOLECULE:	AAA ATPASE, CENTRAL REGION
81:00:00	5y5y-B	2.5	3.5	61	577	7	MOLECULE:	V-TYPE ATP SYNTHASE ALPHA CHAIN
82:00:00	4k7b-A	2.5	3.6	52	111	8	MOLECULE:	EXTRINSIC PROTEIN IN PHOTOSYSTEM II
83:00:00	8iwh-G	2.5	3.6	54	120	6	MOLECULE:	PHOTOSYSTEM II PROTEIN D1
84:00:00	2r9g-D	2.5	6	58	180	9	MOLECULE:	AAA ATPASE, CENTRAL REGION
85:00:00	3sdz-A	2.5	3.1	65	548	2	MOLECULE:	V-TYPE ATP SYNTHASE ALPHA CHAIN
86:00:00	3pvs-C	2.5	3.7	52	420	12	MOLECULE:	REPLICATION-ASSOCIATED RECOMBINATION PROTEIN A
87:00:00	6fes-D	2.5	4.9	63	363	8	MOLECULE:	D12_BRIC2, A SYNTHETIC PROTEIN,D12_BRIC2, A SYNTH
88:00:00	2vl8-B	2.5	3.6	66	533	6	MOLECULE:	CYTOTOXIN L
89:00:00	5voy-E	2.5	3.5	71	592	4	MOLECULE:	V-TYPE PROTON ATPASE CATALYTIC SUBUNIT A,V-TYPE P
90:00:00	6vq6-B	2.5	3.5	69	600	1	MOLECULE:	ATPASE H+-TRANSPORTING V1 SUBUNIT A
91:00:00	5d80-C	2.5	3.5	58	589	7	MOLECULE:	V-TYPE PROTON ATPASE CATALYTIC SUBUNIT A
92:00:00	3pvs-D	2.5	6.1	55	424	11	MOLECULE:	REPLICATION-ASSOCIATED RECOMBINATION PROTEIN A
93:00:00	5cwh-A	2.5	9.1	50	159	6	MOLECULE:	DESIGNED HELICAL REPEAT PROTEIN
94:00:00	7or1-D	2.5	3.8	54	596	6	MOLECULE:	TRANSIENT RECEPTOR POTENTIAL CATION CHANNEL SUBFA
95:00:00	2vkh-C	2.5	3.4	64	537	6	MOLECULE:	CYTOTOXIN L
96:00:00	5y81-H	2.4	3	66	154	14	MOLECULE:	TRANSCRIPTION-ASSOCIATED PROTEIN 1
97:00:00	7m99-F	2.4	4.2	60	257	7	MOLECULE:	TNSC
98:00:00	7m9b-E	2.4	3.2	56	257	5	MOLECULE:	TNSC
99:00:00	7l7b-F	2.4	4.1	62	272	6	MOLECULE:	DNA-DIRECTED RNA POLYMERASE SUBUNIT ALPHA
0:00	8f6q-B	2.4	8.3	56	199	7	MOLECULE:	C8-71
1:00	7m9a-E	2.4	3.2	56	257	5	MOLECULE:	TNSC
2:00	8vjn-A	2.4	3.8	40	52	0	MOLECULE:	ENCAPSULIN NANOCOMPARTMENT CARGO PROTEIN ENCD
3:00	7uhb-K	2.4	4.3	56	75	2	MOLECULE:	SPIKE GLYCOPROTEIN
4:00	7udc-B	2.4	9.7	58	215	5	MOLECULE:	SYNTAXIN-BINDING PROTEIN 1
5:00	7uni-B	2.4	3.8	54	245	4	MOLECULE:	SP2-ZNPPAM DESIGNED CHLOROPHYLL DIMER PROTEIN
6:00	4u5a-B	2.4	2.8	55	184	9	MOLECULE:	SPOROZOITE MICRONEME PROTEIN ESSENTIAL FOR CELL T

7:00	6sp2-A	2.4	3.8	58	366	5	MOLECULE:	MEMBRANE PROTEIN TMS1D
8:00	3ucq-A	2.4	3.5	53	651	6	MOLECULE:	AMYLOSUCRASE
9:00	8hr8-D	2.4	6	58	836	7	MOLECULE:	ARCHAEAL ATPASE
10:00	1k83-A	2.4	3.8	65	1366	9	MOLECULE:	DNA-DIRECTED RNA POLYMERASE II LARGEST SUBUNIT
11:00	3zjc-E	2.4	2.9	39	286	5	MOLECULE:	GTPASE IMAP FAMILY MEMBER 7
12:00	1y1v-A	2.4	4	66	1426	9	MOLECULE:	DNA-DIRECTED RNA POLYMERASE II LARGEST SUBUNIT
13:00	6nbq-B	2.4	2.7	52	483	19	MOLECULE:	NAD(P)H-QUINONE OXIDOREDUCTASE SUBUNIT H
14:00	6n1l-A	2.4	4	58	141	2	MOLECULE:	FIBRONECTIN-BINDING PROTEIN BBK32
15:00	6tgb-A	2.4	3.6	60	1450	13	MOLECULE:	DEDICATOR OF CYTOKINESIS PROTEIN 2
16:00	6wca-A	2.4	5.9	60	367	5	MOLECULE:	ENDOSOMAL/LYSOSOMAL POTASSIUM CHANNEL TMEM175
17:00	6nbx-F	2.4	3.5	57	655	2	MOLECULE:	NAD(P)H-QUINONE OXIDOREDUCTASE SUBUNIT 1
18:00	3k7d-A	2.4	2.6	57	497	5	MOLECULE:	GLUTAMATE-AMMONIA-LIGASE ADENYLYLTRANSFERASE
19:00	7lh7-B	2.4	4	47	135	9	MOLECULE:	BCL-2-LIKE PROTEIN 1
20:00	8ugc-A	2.4	3.5	73	384	4	MOLECULE:	FD15
21:00	5l09-B	2.4	2.1	35	164	6	MOLECULE:	QUORUM-SENSING TRANSCRIPTIONAL ACTIVATOR
22:00	6e6t-A	2.4	4.5	55	266	2	MOLECULE:	NCMC
23:00	3c7n-A	2.4	4.4	54	648	4	MOLECULE:	HEAT SHOCK PROTEIN HOMOLOG SSE1
24:00:00	3fki-A	2.4	3.8	65	1427	9	MOLECULE:	DNA-DIRECTED RNA POLYMERASE II SUBUNIT RPB1
25:00:00	2y31-A	2.4	3.5	55	242	5	MOLECULE:	PUTATIVE REPRESSOR SIMREG2
26:00:00	6fh2-B	2.4	2.9	62	304	10	MOLECULE:	PROTEIN-ARGININE KINASE
27:00:00	3l8i-A	2.4	3.6	59	198	2	MOLECULE:	PROGRAMMED CELL DEATH PROTEIN 10
28:00:00	3o1j-A	2.4	3.6	58	273	3	MOLECULE:	SENSOR PROTEIN TORS
29:00:00	6in7-B	2.4	4	58	174	7	MOLECULE:	SIGMA FACTOR ALGU NEGATIVE REGULATORY PROTEIN
30:00:00	5uz5-G	2.4	2.8	51	229	8	MOLECULE:	U1 SMALL NUCLEAR RIBONUCLEOPROTEIN 70 KDA HOMOLOG
31:00:00	2apl-A	2.4	3.5	59	149	10	MOLECULE:	HYPOTHETICAL PROTEIN PG0816
32:00:00	4nsr-C	2.4	3.2	56	93	2	MOLECULE:	IMMUNITY PROTEIN
33:00:00	2ns7-D	2.4	3.4	53	178	13	MOLECULE:	TETRACYCLINE REPRESSOR PROTEIN
34:00:00	6zyv-A	2.4 1	0.1	59	238	7	MOLECULE:	CIR PROTEIN
35:00:00	5vre-A	2.4	3	56	188	5	MOLECULE:	PUTATIVE INTEGRAL MEMBRANE PROTEIN
36:00:00	6gdj-A	2.4	3.8	51	71	8	MOLECULE:	MTO2
37:00:00	8ve7-A	2.4	3.9	57	294	9	MOLECULE:	DARPIN PROTEIN SCAFFOLD
38:00:00	6tz0-A	2.4	3.8	75	1208	7	MOLECULE:	RNA-DEPENDENT RNA POLYMERASE
39:00:00	3vr2-C	2.4	3.6	72	583	6	MOLECULE:	V-TYPE SODIUM ATPASE CATALYTIC SUBUNIT A
40:00:00	2r9g-L	2.4	6.6	62	188	8	MOLECULE:	AAA ATPASE, CENTRAL REGION
41:00:00	6qlf-N	2.4	2.2	45	371	9	MOLECULE:	INNER KINETOCHORE SUBUNIT IML3
42:00:00	2r9g-H	2.4	6	58	179	9	MOLECULE:	AAA ATPASE, CENTRAL REGION
43:00:00	3i73-A	2.4	3.8	72	512	8	MOLECULE:	A-TYPE ATP SYNTHASE CATALYTIC SUBUNIT A
44:00:00	6ty8-A	2.4	3.7	76	1196	7	MOLECULE:	RNA-DEPENDENT RNA POLYMERASE
45:00:00	5knb-A	2.4	3.9	73	587	4	MOLECULE:	V-TYPE SODIUM ATPASE CATALYTIC SUBUNIT A
46:00:00	7dqc-C	2.4	3.7	73	584	5	MOLECULE:	V-TYPE SODIUM ATPASE CATALYTIC SUBUNIT A
47:00:00	6rko-A	2.4	3.2	55	444	11	MOLECULE:	CYTOCHROME BD-I UBIQUINOL OXIDASE SUBUNIT 2
48:00:00	6tz1-A	2.4	3.8	75	1208	7	MOLECULE:	RNA-DEPENDENT RNA POLYMERASE
49:00:00	7ctf-C	2.4	3.3	61	592	5	MOLECULE:	ORIGIN RECOGNITION COMPLEX SUBUNIT 1
50:00:00	7vy9-p	2.4	4.5	58	178	3	MOLECULE:	NADH DEHYDROGENASE [UBIQUINONE] IRON-SULFUR PROTE
51:00:00	5uj8-B	2.4	3	60	553	7	MOLECULE:	ORIGIN RECOGNITION COMPLEX SUBUNIT 3
52:00:00	8v9o-E	2.4	3.5	60	299	5	MOLECULE:	TETRAHEDRAL NANOCAGE CAGE, NON-FUSION COMPONENT
53:00:00	2r9g-P	2.4	6.6	60	187	8	MOLECULE:	AAA ATPASE, CENTRAL REGION
54:00:00	5bn3-A	2.4	3.4	59	563	2	MOLECULE:	V-TYPE ATP SYNTHASE ALPHA CHAIN
55:00:00	8ffq-B	2.4	3.1	51	602	4	MOLECULE:	TRANSIENT RECEPTOR POTENTIAL CATION CHANNEL SUBFA
56:00:00	7cos-C	2.4	4.7	62	585	10	MOLECULE:	V-TYPE SODIUM ATPASE CATALYTIC SUBUNIT A
57:00:00	8ffq-A	2.4	3.1	51	602	4	MOLECULE:	TRANSIENT RECEPTOR POTENTIAL CATION CHANNEL SUBFA
58:00:00	3nd8-A	2.4	3.6	68	510	1	MOLECULE:	V-TYPE ATP SYNTHASE ALPHA CHAIN
59:00:00	6lu0-A	2.3	2.5	48	937	8	MOLECULE:	CAS12I2
60:00:00	7m9b-L	2.3	3.5	55	257	5	MOLECULE:	TNSC
61:00:00	7mbw-A	2.3	5.2	63	475	8	MOLECULE:	TRANSPOSON TN7 TRANSPOSITION PROTEIN TNSC
62:00:00	7m9c-J	2.3	3.5	58	257	5	MOLECULE:	TNSC

63:00:00	7m9a-L	2.3	3.6	56	257	5	MOLECULE:	TNSC
64:00:00	7m9a-J	2.3	3.5	58	257	5	MOLECULE:	TNSC
65:00:00	6wcc-B	2.3	2.8	55	367	7	MOLECULE:	ENDOSOMAL/LYSOSOMAL POTASSIUM CHANNEL TMEM175
66:00:00	7m9c-L	2.3	3.5	55	257	5	MOLECULE:	TNSC
67:00:00	7m9b-J	2.3	3.5	58	257	5	MOLECULE:	TNSC
68:00:00	2uui-A	2.3	2.9	57	155	5	MOLECULE:	LEUKOTRIENE C4 SYNTHASE
69:00:00	7unh-A	2.3	3.3	65	246	11	MOLECULE:	SP2 DESIGNED CHLOROPHYLL DIMER PROTEIN
70:00:00	7ag9-A	2.3	9.8	56	375	4	MOLECULE:	KAR9
71:00:00	8g9j-A	2.3	8.9	54	201	4	MOLECULE:	THR1
72:00:00	2lse-A	2.3	3.7	57	101	2	MOLECULE:	FOUR HELIX BUNDLE PROTEIN
73:00:00	6ksa-B	2.3	3.9	68	611	9	MOLECULE:	ACYL-COA DEHYDROGENASE
74:00:00	2lp4-A	2.3	4.8	69	225	10	MOLECULE:	CHEMOTAXIS PROTEIN CHEA
75:00:00	2ncg-A	2.3	4.4	59	118	7	MOLECULE:	RGA1E
76:00:00	5wah-A	2.3	2.9	54	99	6	MOLECULE:	IGA FC RECEPTOR
77:00:00	6n4n-F	2.3	3.5	63	191	6	MOLECULE:	NS3 PROTEASE
78:00:00	8a64-A	2.3	4.7	61	940	10	MOLECULE:	ENDO-BETA-N-ACETYLGLUCOSAMINIDASE F2
79:00:00	8tl7-X	2.3	2.6	62	694	10	MOLECULE:	COMPUTATIONALLY DESIGNED PROTEIN
80:00:00	5z2c-A	2.3	3.7	58	445	12	MOLECULE:	ALPHA-PROTEIN KINASE 1
81:00:00	7wtz-K	2.3 1	0.5	70	909	11	MOLECULE:	18S RRNA
82:00:00	6tjv-F	2.3	3	53	600	6	MOLECULE:	NAD(P)H-QUINONE OXIDOREDUCTASE SUBUNIT 1
83:00:00	6xke-A	2.3	2.8	54	116	4	MOLECULE:	ALBICIN
84:00:00	3h0d-A	2.3	2.9	52	155	6	MOLECULE:	CTSR
85:00:00	1vbg-A	2.3	6.1	59	874	10	MOLECULE:	PYRUVATE,ORTHOPHOSPHATE DIKINASE
86:00:00	6n4n-C	2.3	3.6	65	184	3	MOLECULE:	NS3 PROTEASE
87:00:00	1t6u-A	2.3	4.7	60	117	3	MOLECULE:	SUPEROXIDE DISMUTASE [NI]
88:00:00	4fyf-A	2.3	2.4	58	739	9	MOLECULE:	SIDF, INHIBITOR OF GROWTH FAMILY, MEMBER 3
89:00:00	7b5n-H	2.3	4.9	66	423	5	MOLECULE:	CULLIN-1
90:00:00	5uha-F	2.3	4.2	64	322	8	MOLECULE:	DNA-DIRECTED RNA POLYMERASE SUBUNIT ALPHA
91:00:00	6a2j-A	2.3	4.2	51	309	6	MOLECULE:	HEME A SYNTHASE
92:00:00	7qho-V	2.3	3.8	38	129	18	MOLECULE:	CYTOCHROME BC1 COMPLEX RIESKE IRON-SULFUR SUBUNIT
93:00:00	2v0o-A	2.3	3.4	55	273	7	MOLECULE:	FCH DOMAIN ONLY PROTEIN 2
94:00:00	7k8c-A	2.3	2.9	58	722	7	MOLECULE:	TREHALOSE MONOMYCOLATE EXPORTER MMPL3
95:00:00	5cbo-E	2.3	2.9	46	176	7	MOLECULE:	MBP3-16,IMMUNOGLOBULIN G-BINDING PROTEIN A
96:00:00	6c9k-D	2.3	4	59	294	7	MOLECULE:	DARP14 - SUBUNIT A WITH DARPIN
97:00:00	7vbl-p	2.3	4.4	56	178	4	MOLECULE:	NADH DEHYDROGENASE [UBIQUINONE] IRON-SULFUR PROTE
98:00:00	7k8d-A	2.3	4.7	62	724	10	MOLECULE:	DRUG EXPORTERS OF THE RND SUPERFAMILY-LIKE PROTEI
99:00:00	5cbo-L	2.3	3	50	176	6	MOLECULE:	MBP3-16,IMMUNOGLOBULIN G-BINDING PROTEIN A
0:00	8v9o-F	2.3	3.6	60	300	5	MOLECULE:	TETRAHEDRAL NANOCAGE CAGE, NON-FUSION COMPONENT
1:00	8v9o-D	2.3	3.6	60	301	5	MOLECULE:	TETRAHEDRAL NANOCAGE CAGE, NON-FUSION COMPONENT
2:00	7jps-C	2.3	3.2	61	591	5	MOLECULE:	ORIGIN RECOGNITION COMPLEX SUBUNIT 1
3:00	5h76-B	2.3	4.6	53	197	8	MOLECULE:	DARPIN,IMMUNOGLOBULIN G-BINDING PROTEIN A
4:00	6c9k-J	2.3	4.1	59	292	7	MOLECULE:	DARP14 - SUBUNIT A WITH DARPIN
5:00	5cbo-C	2.3	2.8	43	176	9	MOLECULE:	MBP3-16,IMMUNOGLOBULIN G-BINDING PROTEIN A
6:00	6v9v-A	2.3	3.7	53	597	6	MOLECULE:	TRANSIENT RECEPTOR POTENTIAL CATION CHANNEL SUBFA
7:00	7tmm-C	2.3	4	67	592	3	MOLECULE:	H(+)-TRANSPORTING TWO-SECTOR ATPASE
8:00	6v9v-D	2.3	3.7	53	597	6	MOLECULE:	TRANSIENT RECEPTOR POTENTIAL CATION CHANNEL SUBFA
9:00	3oe7-X	2.3	3.1	57	469	9	MOLECULE:	ATP SYNTHASE SUBUNIT ALPHA
10:00	5cbo-B	2.3	2.8	43	176	9	MOLECULE:	MBP3-16,IMMUNOGLOBULIN G-BINDING PROTEIN A
11:00	8fin-A	2.3	9.5	53	174	9	MOLECULE:	CS207A
12:00	6fes-B	2.3	4.9	66	363	12	MOLECULE:	D12_BRIC2, A SYNTHETIC PROTEIN,D12_BRIC2, A SYNTH
13:00	6ztq-n	2.3	4.6	56	177	0	MOLECULE:	NADH-UBIQUINONE OXIDOREDUCTASE CHAIN 3
14:00	7bwr-A	2.3	5.3	54	1058	4	MOLECULE:	INTEGRAL MEMBRANE INDOLYLACETYLINOSITOL
15:00	7w4f-p	2.3	4.7	57	178	0	MOLECULE:	NADH DEHYDROGENASE [UBIQUINONE] FLAVOPROTEIN 1,
16:00	6fes-C	2.3	3.8	58	363	14	MOLECULE:	D12_BRIC2, A SYNTHETIC PROTEIN,D12_BRIC2, A SYNTH
17:00	4dmv-A	2.3	4	50	537	6	MOLECULE:	TOXIN A
18:00	7n6b-A	2.3	3.2	49	757	6	MOLECULE:	MMPL3 TRANSPORTER

19:00	5bw9-a	2.3	4.3	64	591	5	MOLECULE:	V-TYPE PROTON ATPASE CATALYTIC SUBUNIT A
20:00	3qjy-A	2.3	3.1	65	556	2	MOLECULE:	V-TYPE ATP SYNTHASE ALPHA CHAIN
21:00	6ej7-A	2.2	3.2	47	706	11	MOLECULE:	XYLOSYLTRANSFERASE 1
22:00	2kob-A	2.2	2.9	43	108	5	MOLECULE:	UNCHARACTERIZED PROTEIN
23:00	7o8c-A	2.2	2.3	43	582	2	MOLECULE:	SURFACE GLYCAN-BINDING PROTEIN BO2743
24:00:00	7n6i-H	2.2	3.3	54	257	7	MOLECULE:	TNIQ (HOMOLOGY MODEL)
25:00:00	6ahx-A	2.2	3.9	40	60	8	MOLECULE:	PUTATIVE CYTOSOLIC PROTEIN
26:00:00	6gcs-F	2.2	3.9	53	119	9	MOLECULE:	75-KDA PROTEIN (NUAM)
27:00:00	6irr-A	2.2	8.9	48	133	8	MOLECULE:	DISRUPTED IN SCHIZOPHRENIA 1 HOMOLOG,CYCLIC AMP-D
28:00:00	3qwe-A	2.2	3.7	56	260	2	MOLECULE:	GEM-INTERACTING PROTEIN
29:00:00	3p8c-F	2.2	4.8	43	156	5	MOLECULE:	CYTOPLASMIC FMR1-INTERACTING PROTEIN 1
30:00:00	7vqo-A	2.2	4.2	65	1180	6	MOLECULE:	AMS1, NBR1 AND MALE FUSION PROTEIN
31:00:00	3s4w-A	2.2 1	0.2	64	1206	2	MOLECULE:	FANCONI ANEMIA GROUP I PROTEIN HOMOLOG
32:00:00	7aqr-V	2.2	3.9	53	140	4	MOLECULE:	NADH DEHYDROGENASE [UBIQUINONE] IRON-SULFUR PROTE
33:00:00	2uxw-A	2.2	3.7	66	567	8	MOLECULE:	VERY-LONG-CHAIN SPECIFIC ACYL-COA DEHYDROGENASE
34:00:00	5ijj-A	2.2	9	54	175	4	MOLECULE:	SPX DOMAIN
35:00:00	7s0r-A	2.2	3.1	49	85	12	MOLECULE:	C PROTEIN BETA ANTIGEN
36:00:00	6r9r-A	2.2	4.8	55	454	7	MOLECULE:	DNA (5'-R(P*AP*AP*AP*A)-3')
37:00:00	4m0m-A	2.2	3.4	67	721	7	MOLECULE:	PUTATIVE UNCHARACTERIZED PROTEIN
38:00:00	2dgj-A	2.2	3.3	48	246	4	MOLECULE:	HYPOTHETICAL PROTEIN EBHA
39:00:00	8ebt-C	2.2 1	1.3	60	191	5	MOLECULE:	GENERAL TRANSCRIPTION AND DNA REPAIR FACTOR IIH H
40:00:00	7wu0-K	2.2 1	0.3	60	909	13	MOLECULE:	18S RRNA
41:00:00	7mkk-B	2.2	4.6	54	73	7	MOLECULE:	SMALL OVARY, ISOFORM A
42:00:00	4dyl-A	2.2	3.5	57	376	4	MOLECULE:	TYROSINE-PROTEIN KINASE FES/FPS
43:00:00	6xj1-B	2.2	3.1	58	277	3	MOLECULE:	CELL DIVISION CONTROL PROTEIN 15
44:00:00	4he8-A	2.2	3.9	41	92	5	MOLECULE:	NADH-QUINONE OXIDOREDUCTASE SUBUNIT 7
45:00:00	1k05-B	2.2	3.4	56	142	9	MOLECULE:	FOCAL ADHESION KINASE 1
46:00:00	6oeh-A	2.2	3	57	470	0	MOLECULE:	POLYALA MODEL OF OMCC I-LAYER
47:00:00	6z35-A	2.2	3.9	56	109	2	MOLECULE:	MAQUETTE 2-1IP
48:00:00	3t38-A	2.2	3.7	52	199	6	MOLECULE:	ARSENATE REDUCTASE
49:00:00	6f1v-m	2.2	3.6	54	217	7	MOLECULE:	CYTOPLASMIC DYNEIN 1 HEAVY CHAIN 1
50:00:00	4nsr-B	2.2	2.7	51	90	2	MOLECULE:	IMMUNITY PROTEIN
51:00:00	4nsr-F	2.2	2.8	51	89	2	MOLECULE:	IMMUNITY PROTEIN
52:00:00	2hxo-A	2.2	3	51	207	10	MOLECULE:	PUTATIVE TETR-FAMILY TRANSCRIPTIONAL REGULATOR
53:00:00	1q0k-A	2.2	3.9	56	117	0	MOLECULE:	SUPEROXIDE DISMUTASE [NI]
54:00:00	1i4d-A	2.2	3.4	53	188	6	MOLECULE:	ARFAPTIN 2
55:00:00	8c5v-Q	2.2	3.6	42	516	5	MOLECULE:	CHEMOTAXIS PROTEIN CHEA
56:00:00	2pmr-A	2.2	4.1	49	76	10	MOLECULE:	UNCHARACTERIZED PROTEIN
57:00:00	1rke-A	2.2	6.4	61	262	3	MOLECULE:	VINCULIN
58:00:00	2ip6-A	2.2	2.9	50	87	8	MOLECULE:	PAPB
59:00:00	6dfk-I	2.2	3.8	54	236	7	MOLECULE:	SUBUNIT OF PROTEASEOME ACTIVATOR COMPLEX,PUTATIVE
60:00:00	2hye-C	2.2 1	0.6	56	719	11	MOLECULE:	DNA DAMAGE-BINDING PROTEIN 1
61:00:00	3les-A	2.2	4.2	62	179	5	MOLECULE:	RNA POLYMERASE SIGMA FACTOR
62:00:00	6xh5-F	2.2	8.6	59	348	5	MOLECULE:	HELICAL FUSION DESIGN
63:00:00	6c9k-T	2.2	3.9	57	294	9	MOLECULE:	DARP14 - SUBUNIT A WITH DARPIN
64:00:00	7cop-C	2.2	3.7	72	584	4	MOLECULE:	V-TYPE SODIUM ATPASE CATALYTIC SUBUNIT A
65:00:00	5vre-C	2.2	3.1	56	188	5	MOLECULE:	PUTATIVE INTEGRAL MEMBRANE PROTEIN
66:00:00	6c9k-I	2.2	4	59	293	7	MOLECULE:	DARP14 - SUBUNIT A WITH DARPIN
67:00:00	3i4l-A	2.2	3.8	74	524	8	MOLECULE:	A-TYPE ATP SYNTHASE CATALYTIC SUBUNIT A
68:00:00	6c9k-B	2.2	4	57	292	9	MOLECULE:	DARP14 - SUBUNIT A WITH DARPIN
69:00:00	5knd-B	2.2	3.6	72	592	4	MOLECULE:	V-TYPE SODIUM ATPASE CATALYTIC SUBUNIT A
70:00:00	6c9k-Q	2.2	4.5	60	293	8	MOLECULE:	DARP14 - SUBUNIT A WITH DARPIN
71:00:00	7w4l-p	2.2	4.5	56	178	5	MOLECULE:	NADH DEHYDROGENASE [UBIQUINONE] FLAVOPROTEIN 1,
72:00:00	6c9k-K	2.2	4	57	291	9	MOLECULE:	DARP14 - SUBUNIT A WITH DARPIN
73:00:00	6c9k-S	2.2	4	57	291	9	MOLECULE:	DARP14 - SUBUNIT A WITH DARPIN
74:00:00	7pty-B	2.2 1	1	67	865	6	MOLECULE:	DELTA-LATROINSECTOTOXIN-LT1A

75:00:00	6c9k-A	2.2	4.5	60	293	8	MOLECULE:	DARP14 - SUBUNIT A WITH DARPIN
76:00:00	5cwl-A	2.2 1	0.1	53	177	8	MOLECULE:	DESIGNED HELICAL REPEAT PROTEIN
77:00:00	5x09-A	2.2	3.6	68	530	1	MOLECULE:	V-TYPE ATP SYNTHASE ALPHA CHAIN,V-TYPE ATP SYNTHA
78:00:00	7yks-D	2.2	3.1	52	591	10	MOLECULE:	TRANSIENT RECEPTOR POTENTIAL CATION CHANNEL SUBFA
79:00:00	2bvm-A	2.2	3.7	50	541	10	MOLECULE:	TOXIN B
80:00:00	7wnx-A	2.2	3	56	734	2	MOLECULE:	TREHALOSE MONOMYCOLATE EXPORTER MMPL3
81:00:00	7dqd-C	2.2	4.2	74	584	7	MOLECULE:	V-TYPE SODIUM ATPASE CATALYTIC SUBUNIT A
82:00:00	7ose-D	2.2	4.4	52	503	13	MOLECULE:	CYTOCHROME BD-II UBIQUINOL OXIDASE SUBUNIT 1
83:00:00	7jk4-C	2.2	6.9	59	612	3	MOLECULE:	ORIGIN RECOGNITION COMPLEX SUBUNIT 2
84:00:00	6v9v-C	2.2	3.7	53	597	6	MOLECULE:	TRANSIENT RECEPTOR POTENTIAL CATION CHANNEL SUBFA
85:00:00	5cbo-I	2.2	2.7	43	176	9	MOLECULE:	MBP3-16,IMMUNOGLOBULIN G-BINDING PROTEIN A
86:00:00	7lkm-A	2.1	2.5	39	149	10	MOLECULE:	PILUS BIOGENESIS PROTEIN
87:00:00	7m9c-H	2.1	3.9	59	257	7	MOLECULE:	TNSC
88:00:00	7m9a-H	2.1	3.3	54	257	4	MOLECULE:	TNSC
89:00:00	7m9b-H	2.1	3.3	54	257	4	MOLECULE:	TNSC
90:00:00	7p2p-C	2.1	4.3	40	174	8	MOLECULE:	SIGNAL PEPTIDASE COMPLEX CATALYTIC SUBUNIT SEC11A
91:00:00	8e1e-B	2.1	4.3	50	232	4	MOLECULE:	SG122_C3
92:00:00	6gvw-B	2.1	6.6	44	262	5	MOLECULE:	BRCA1-A COMPLEX SUBUNIT ABRAXAS 1
93:00:00	6l7o-G	2.1	3	39	192	13	MOLECULE:	NAD(P)H-QUINONE OXIDOREDUCTASE SUBUNIT 1
94:00:00	7phk-E	2.1	7.7	47	393	6	MOLECULE:	POTASSIUM VOLTAGE-GATED CHANNEL, SHAW-RELATED SUB
95:00:00	8tl7-Y	2.1	2.6	62	694	10	MOLECULE:	COMPUTATIONALLY DESIGNED PROTEIN
96:00:00	7e1w-B	2.1	4.6	52	524	8	MOLECULE:	CYTOCHROME C OXIDASE SUBUNIT 2
97:00:00	7zmb-F	2.1	4	53	238	6	MOLECULE:	NADH-UBIQUINONE OXIDOREDUCTASE CHAIN 1
98:00:00	5flz-B	2.1	4.9	61	565	11	MOLECULE:	SPINDLE POLE BODY COMPONENT SPC97
99:00:00	1r9t-A	2.1	4	65	1395	9	MOLECULE:	RNA STRAND
0:00	8j0h-A	2.1	8.9	56	134	9	MOLECULE:	UNCHARACTERIZED PROTEIN C4H3.06
1:00	3rgu-A	2.1	4.7	53	88	8	MOLECULE:	FIMBRIAE-ASSOCIATED PROTEIN FAP1
2:00	5jbr-A	2.1	8.5	45	149	4	MOLECULE:	UNCHARACTERIZED PROTEIN BCAV_2135
3:00	4uy3-A	2.1	2.9	53	194	8	MOLECULE:	SEPTATION RING FORMATION REGULATOR EZRA
4:00	4noo-B	2.1	2.7	50	95	2	MOLECULE:	VGRG PROTEIN
5:00	4nqi-D	2.1	3.3	53	232	0	MOLECULE:	SH3 DOMAIN-CONTAINING PROTEIN
6:00	8bpr-G	2.1	3.5	65	193	5	MOLECULE:	DNA REPLICATION AND REPAIR PROTEIN RECF
7:00	7e1x-B	2.1	4.6	52	524	8	MOLECULE:	CYTOCHROME C OXIDASE SUBUNIT 2
8:00	3ats-A	2.1	6.3	58	352	5	MOLECULE:	PUTATIVE UNCHARACTERIZED PROTEIN
9:00	8w41-A	2.1	4	55	1162	0	MOLECULE:	CALMODULIN-1
10:00	3hai-A	2.1	3.2	51	294	4	MOLECULE:	HUMAN PACSIN1 F-BAR
11:00	6irw-A	2.1	4.5	56	495	11	MOLECULE:	PHOSPHORYLATED CTD-INTERACTING FACTOR 1
12:00	8e7s-O	2.1	9.5	49	269	2	MOLECULE:	CYTOCHROME B-C1 COMPLEX SUBUNIT 1, MITOCHONDRIAL
13:00	8pmq-2	2.1	7.8	55	355	11	MOLECULE:	E3 UBIQUITIN-PROTEIN LIGASE RMD5
14:00	6tjv-C	2.1	3.4	39	114	13	MOLECULE:	NAD(P)H-QUINONE OXIDOREDUCTASE SUBUNIT 1
15:00	3t46-A	2.1	4.2	53	75	9	MOLECULE:	SCIN-D
16:00	6q8x-A	2.1	4	41	117	5	MOLECULE:	NADH-QUINONE OXIDOREDUCTASE SUBUNIT 1
17:00	4bpd-F	2.1	2.7	46	92	15	MOLECULE:	DIACYLGLYCEROL KINASE
18:00	8ia1-C	2.1	2.3	54	361	6	MOLECULE:	GLYCEROL-3-PHOSPHATE ACYLTRANSFERASE, CHLOROPLAST
19:00	3ckd-B	2.1	4.5	55	244	4	MOLECULE:	INVASION PLASMID ANTIGEN, SECRETED BY THE MXI-SPA
20:00	7wej-A	2.1	3.3	49	270	8	MOLECULE:	WD REPEAT-CONTAINING PROTEIN 47
21:00	1n5u-A	2.1	3.7	63	583	2	MOLECULE:	SERUM ALBUMIN
22:00	1k40-A	2.1	3.3	57	126	9	MOLECULE:	ADHESION KINASE
23:00	4brb-F	2.1	3	47	92	15	MOLECULE:	DIACYLGLYCEROL KINASE
24:00:00	7khc-F	2.1	3.7	55	447	9	MOLECULE:	DNA-DIRECTED RNA POLYMERASE SUBUNIT ALPHA
25:00:00	2z0v-A	2.1	3.8	52	223	4	MOLECULE:	SH3-CONTAINING GRB2-LIKE PROTEIN 3
26:00:00	6fbv-F	2.1	3.6	60	293	3	MOLECULE:	DNA-DIRECTED RNA POLYMERASE SUBUNIT ALPHA
27:00:00	1z23-A	2.1	3.9	57	163	7	MOLECULE:	CRK-ASSOCIATED SUBSTRATE
28:00:00	4by9-C	2.1	3.2	51	366	2	MOLECULE:	SSR26
29:00:00	1lvf-A	2.1	9.5	53	106	8	MOLECULE:	SYNTAXIN 6
30:00:00	1m6t-A	2.1	3.7	55	106	4	MOLECULE:	SOLUBLE CYTOCHROME B562

31:00:00	7rmy-A	2.1	3.8	75	266	4	MOLECULE:	DE NOVO DESIGNED TUNABLE HOMODIMER, D_3-337
32:00:00	3kyj-A	2.1	4.1	58	129	3	MOLECULE:	PUTATIVE HISTIDINE PROTEIN KINASE
33:00:00	2c2l-A	2.1	2.7	42	281	0	MOLECULE:	CARBOXY TERMINUS OF HSP70-INTERACTING PROTEIN
34:00:00	8swd-D	2.1	3	39	53	10	MOLECULE:	2-OXOGLUTARATE:ACCEPTOR OXIDOREDUCTASE
35:00:00	1ysm-A	2.1	2.2	38	55	8	MOLECULE:	CALCYCLIN-BINDING PROTEIN
36:00:00	4jza-A	2.1	3.9	61	817	10	MOLECULE:	UNCHARACTERIZED PROTEIN
37:00:00	7m4m-A	2.1	6.9	42	173	5	MOLECULE:	E3 UBIQUITIN-PROTEIN LIGASE RNF216
38:00:00	3l32-B	2.1	1.6	35	45	3	MOLECULE:	PHOSPHOPROTEIN
39:00:00	6vq8-C	2.1	3.7	73	600	4	MOLECULE:	ATPASE H+-TRANSPORTING V1 SUBUNIT A
40:00:00	2qw6-B	2.1	3.7	48	85	6	MOLECULE:	AAA ATPASE, CENTRAL REGION
41:00:00	7r72-R	2.1	3.2	52	134	8	MOLECULE:	25S RRNA
42:00:00	5f3q-B	2.1	2.9	53	176	6	MOLECULE:	PUTATIVE UNCHARACTERIZED PROTEIN
43:00:00	5f3o-B	2.1	3.1	55	189	5	MOLECULE:	PUTATIVE UNCHARACTERIZED PROTEIN
44:00:00	7tts-F	2.1	4.9	59	433	15	MOLECULE:	CASEINOLYTIC PEPTIDASE B PROTEIN HOMOLOG
45:00:00	7cte-C	2.1	3.2	60	592	5	MOLECULE:	ORIGIN RECOGNITION COMPLEX SUBUNIT 2
46:00:00	6xh5-E	2.1	8.5	61	348	5	MOLECULE:	HELICAL FUSION DESIGN
47:00:00	3vr4-C	2.1	3.3	64	586	5	MOLECULE:	V-TYPE SODIUM ATPASE CATALYTIC SUBUNIT A
48:00:00	5zea-A	2.1	3.3	65	587	5	MOLECULE:	V-TYPE SODIUM ATPASE CATALYTIC SUBUNIT A
49:00:00	7uby-B	2.1	3.8	51	529	6	MOLECULE:	GLUCOSYLTRANSFERASE TCDA
50:00:00	5zea-G	2.1	3.6	72	587	4	MOLECULE:	V-TYPE SODIUM ATPASE CATALYTIC SUBUNIT A
51:00:00	7or0-B	2.1	3.8	54	596	6	MOLECULE:	TRANSIENT RECEPTOR POTENTIAL CATION CHANNEL SUBFA
52:00:00	7dqe-B	2.1	3.3	66	584	6	MOLECULE:	V-TYPE SODIUM ATPASE CATALYTIC SUBUNIT A
53:00:00	7zmb-R	2.1	2.9	49	98	8	MOLECULE:	NADH-UBIQUINONE OXIDOREDUCTASE CHAIN 1
54:00:00	1pjq-B	2	4	60	456	8	MOLECULE:	SIROHEME SYNTHASE
55:00:00	7p61-A	2	4.9	43	106	14	MOLECULE:	NADH-QUINONE OXIDOREDUCTASE SUBUNIT F
56:00:00	4a4z-A	2	3.8	37	874	8	MOLECULE:	ANTIVIRAL HELICASE SKI2
57:00:00	7upp-C	2	3.9	42	75	5	MOLECULE:	DHT03 PROTEIN A
58:00:00	7d0i-F	2	4.6	45	424	7	MOLECULE:	AUTOPHAGY-RELATED PROTEIN 9
59:00:00	5lnk-A	2	4.8	44	115	9	MOLECULE:	MITOCHONDRIAL COMPLEX I, 51 KDA SUBUNIT
60:00:00	6ziy-A	2	4	41	117	5	MOLECULE:	NADH-QUINONE OXIDOREDUCTASE SUBUNIT 1
61:00:00	8ebs-C	2.0 1	0.7	67	358	4	MOLECULE:	TFIIH BASAL TRANSCRIPTION FACTOR COMPLEX HELICASE
62:00:00	6jhm-A	2	4.5	66	494	8	MOLECULE:	CHLOROPHENOL MONOOXYGENASE
63:00:00	8ab0-G	2	3.7	64	193	6	MOLECULE:	RECOMBINATION PROTEIN RECR
64:00:00	6khi-C	2	3.8	41	120	12	MOLECULE:	NAD(P)H-QUINONE OXIDOREDUCTASE SUBUNIT 1
65:00:00	6l80-C	2	2.9	47	107	4	MOLECULE:	GAMMA-TUBULIN COMPLEX SUBUNIT MOD21
66:00:00	7nyh-A	2	4.7	43	86	14	MOLECULE:	NADH-QUINONE OXIDOREDUCTASE SUBUNIT A
67:00:00	6l7p-C	2	3.7	41	121	12	MOLECULE:	NAD(P)H-QUINONE OXIDOREDUCTASE SUBUNIT 1
68:00:00	6vp0-E	2	7.8	52	406	10	MOLECULE:	DIACYLGLYCEROL O-ACYLTRANSFERASE 1
69:00:00	6ptq-A	2	8.2	48	482	6	MOLECULE:	PHOTORECEPTOR-HISTIDINE KINASE BPHP
70:00:00	8tl7-V	2	3	64	694	8	MOLECULE:	COMPUTATIONALLY DESIGNED PROTEIN
71:00:00	8f6q-A	2	9	66	199	9	MOLECULE:	C8-71
72:00:00	1eo0-A	2	2.4	47	77	13	MOLECULE:	TRANSCRIPTION ELONGATION FACTOR S-II
73:00:00	8om1-A	2	4.1	42	115	14	MOLECULE:	NADH-UBIQUINONE OXIDOREDUCTASE CHAIN 3
74:00:00	8swd-C	2	3.1	39	53	10	MOLECULE:	2-OXOGLUTARATE:ACCEPTOR OXIDOREDUCTASE
75:00:00	7tad-C	2.0 1	0	53	210	9	MOLECULE:	REGULATORY PROTEIN NPR1
76:00:00	7d0i-L	2	4.6	45	424	7	MOLECULE:	AUTOPHAGY-RELATED PROTEIN 9
77:00:00	6umr-A	2	2.9	56	418	5	MOLECULE:	DAMAGE-CONTROL PHOSPHATASE DUF89
78:00:00	6zku-t	2	5.1	53	177	6	MOLECULE:	NADH DEHYDROGENASE [UBIQUINONE] FLAVOPROTEIN 1,
79:00:00	7jpr-C	2	3	58	593	5	MOLECULE:	ORIGIN RECOGNITION COMPLEX SUBUNIT 1
80:00:00	4mix-A	2	3.6	68	276	3	MOLECULE:	PUTATIVE INSECTICIDAL TOXIN
81:00:00	3mfy-A	2	3.6	70	512	3	MOLECULE:	V-TYPE ATP SYNTHASE ALPHA CHAIN
82:00:00	8pwl-A	2.0 1	0.8	66	830	6	MOLECULE:	E3 UBIQUITIN-PROTEIN LIGASE HACE1
83:00:00	7tmp-A	2	3.2	64	593	5	MOLECULE:	H(+)-TRANSPORTING TWO-SECTOR ATPASE
84:00:00	3srz-A	2	3.7	64	537	9	MOLECULE:	TOXIN A
85:00:00	7yks-B	2	2.9	52	591	10	MOLECULE:	TRANSIENT RECEPTOR POTENTIAL CATION CHANNEL SUBFA

DALI results: MdA-1 Phogi

No:	Chain	Z	rmsd	lali	nres	%id	PDB	Description
1:00	7r5s-P	5.8	5.7	72	224	1	MOLECULE:	CENTROMERE PROTEIN H
2:00	7qoo-P	5.6	7.1	83	233	1	MOLECULE:	CENTROMERE PROTEIN C
3:00	7pkn-P	5.6	6.8	74	224	3	MOLECULE:	CENTROMERE PROTEIN H
4:00	7uik-p	5.3	5.2	73	895	4	MOLECULE:	DNA (38-MER)
5:00	1wzn-A	5.2	4.2	70	245	7	MOLECULE:	SAM-DEPENDENT METHYLTRANSFERASE
6:00	7ywx-P	5.2	5.6	72	224	1	MOLECULE:	CENTROMERE PROTEIN H
7:00	6i7s-G	5	5.4	88	863	6	MOLECULE:	PROTEIN DISULFIDE-ISOMERASE
8:00	3u1w-C	5	4.7	78	250	6	MOLECULE:	HYPOTHETICAL PERIPLASMIC PROTEIN
9:00	5d16-A	4.9	3	64	161	6	MOLECULE:	TRANSPOSON TN7 TRANSPOSITION PROTEI
10:00	7xhn-P	4.9	7.8	79	224	4	MOLECULE:	CENTROMERE PROTEIN O
11:00	6lk8-J	4.8	5.2	72	1026	11	MOLECULE:	MGC83295 PROTEIN
12:00	7uic-p	4.8	5.1	72	903	4	MOLECULE:	MEDIATOR OF RNA POLYMERASE II TRANS
13:00	8eoj-B	4.8	6.9	92	781	4	MOLECULE:	PROTEIN DISULFIDE-ISOMERASE
14:00	5o68-D	4.8	7.1	90	271	7	MOLECULE:	FAPF
15:00	7yyh-P	4.8	7.3	68	219	7	MOLECULE:	HISTONE H3-LIKE CENTROMERIC PROTEIN
16:00	7r5v-P	4.8	7.4	69	219	7	MOLECULE:	CENTROMERE PROTEIN H
17:00	7uil-6	4.8	5.2	73	903	4	MOLECULE:	MEDIATOR OF RNA POLYMERASE II TRANS
18:00	7px8-A	4.7	4.9	74	700	5	MOLECULE:	ACYLAMINO-ACID-RELEASING ENZYME
19:00	5o68-H	4.7	7.1	88	272	6	MOLECULE:	FAPF
20:00	5o68-K	4.7	7.1	89	272	6	MOLECULE:	FAPF
21:00	7qun-A	4.7	4.9	73	695	5	MOLECULE:	ACYLAMINO-ACID-RELEASING ENZYME
22:00	7qun-C	4.7	4.6	73	694	5	MOLECULE:	ACYLAMINO-ACID-RELEASING ENZYME
23:00	7px8-B	4.7	4.9	72	700	6	MOLECULE:	ACYLAMINO-ACID-RELEASING ENZYME
24:00:00	7qun-B	4.7	5	73	694	5	MOLECULE:	ACYLAMINO-ACID-RELEASING ENZYME
25:00:00	7qun-D	4.7	4.6	73	694	5	MOLECULE:	ACYLAMINO-ACID-RELEASING ENZYME
26:00:00	3cnx-C	4.6	5.3	65	148	9	MOLECULE:	UNCHARACTERIZED PROTEIN
27:00:00	6zce-p	4.6	6.4	76	646	8	MOLECULE:	EUKARYOTIC TRANSLATION INITIATION F
28:00:00	5o65-B	4.6	6	89	296	4	MOLECULE:	FAPF
29:00:00	7s7u-A	4.6	6.7	83	511	4	MOLECULE:	INICSNFR 3.0 FLUORESCENT NICOTINE S
30:00:00	8cas-p	4.6	4.9	70	646	9	MOLECULE:	40S RIBOSOMAL PROTEIN S0-A
31:00:00	5o68-I	4.6	5.4	76	256	12	MOLECULE:	FAPF
32:00:00	4ik8-A	4.6	6.7	83	395	2	MOLECULE:	RCAMP, GREEN FLUORESCENT PROTEIN
33:00:00	3wlc-A	4.6	6.7	82	392	2	MOLECULE:	MYOSIN LIGHT CHAIN KINASE, GREEN FL
34:00:00	4it7-A	4.5	3.7	65	107	5	MOLECULE:	CPI
35:00:00	6qvi-A	4.5	7.4	76	518	8	MOLECULE:	COMZ
36:00:00	5tgn-A	4.5	3.8	57	109	12	MOLECULE:	UNCHARACTERIZED PROTEIN
37:00:00	3d9r-B	4.5	6.8	74	134	7	MOLECULE:	KETOSTEROID ISOMERASE-LIKE PROTEIN
38:00:00	6i7s-H	4.5	5.2	86	862	6	MOLECULE:	PROTEIN DISULFIDE-ISOMERASE
39:00:00	7s7v-C	4.5	8	87	506	3	MOLECULE:	INICSNFR 3.0 FLUORESCENT NICOTINE S
40:00:00	6e8a-A	4.4	2.8	56	148	4	MOLECULE:	DUF1795 DOMAIN-CONTAINING PROTEIN
41:00:00	7wb4-E	4.4	4.8	76	1363	4	MOLECULE:	OUTER NUP133
42:00:00	2o5p-A	4.4	12.2	100	772	6	MOLECULE:	FERRIPYOVERDINE RECEPTOR
43:00:00	5dl5-A	4.4	7.7	79	416	9	MOLECULE:	MEMBRANE PROTEIN
44:00:00	5tgn-C	4.4	4.1	56	107	11	MOLECULE:	UNCHARACTERIZED PROTEIN

45:00:00	3blz-A	4.4	3.6	57	124	9	MOLECULE:	NTF2-LIKE PROTEIN OF UNKNOWN FUNCTI
46:00:00	7jta-B	4.4	3.3	52	56	4	MOLECULE:	NTF2-LIKE NUCLEASE/ANTI-CRISPR
47:00:00	7pb8-P	4.4	7.7	75	224	8	MOLECULE:	CENTROMERE PROTEIN O
48:00:00	6zu9-p	4.4	5.4	70	646	9	MOLECULE:	18S RIBOSOMAL RNA
49:00:00	5o67-C	4.4	6.4	84	284	6	MOLECULE:	FAPF
50:00:00	3u1w-B	4.4	3.2	74	248	1	MOLECULE:	HYPOTHETICAL PERIPLASMIC PROTEIN
51:00:00	5o65-A	4.4	7.1	89	276	6	MOLECULE:	FAPF
52:00:00	7s7t-A	4.4	6.7	83	509	4	MOLECULE:	INICSNFR 3.0 FLUORESCENT NICOTINE S
53:00:00	3ek4-A	4.4	6.7	82	348	2	MOLECULE:	MYOSIN LIGHT CHAIN KINASE, GREEN FL
54:00:00	7jta-A	4.3	3.6	52	56	4	MOLECULE:	NTF2-LIKE NUCLEASE/ANTI-CRISPR
55:00:00	3gzr-A	4.3	4.5	68	143	6	MOLECULE:	UNCHARACTERIZED PROTEIN WITH A NTF2
56:00:00	2w4y-A	4.3	4	69	122	4	MOLECULE:	CAULOBACTER 5 VIRUS-LIKE PARTICLE
57:00:00	2d73-A	4.3	3	62	717	11	MOLECULE:	ALPHA-GLUCOSIDASE SUSB
58:00:00	7zkh-A	4.3	5.6	74	267	9	MOLECULE:	METHYLTRANSFERASE
59:00:00	6nd4-H	4.3	5	74	834	7	MOLECULE:	ETS RRNA
60:00:00	6bjt-B	4.3	2.9	55	130	4	MOLECULE:	DUF4440 DOMAIN-CONTAINING PROTEIN
61:00:00	7syf-A	4.3	9.7	80	1391	9	MOLECULE:	PHOSPHATIDYLINOSITOL 3,4,5-TRISPHOS
62:00:00	8cah-p	4.3	5.5	70	646	6	MOLECULE:	EUKARYOTIC TRANSLATION INITIATION F
63:00:00	3cnx-A	4.3	5.3	65	138	9	MOLECULE:	UNCHARACTERIZED PROTEIN
64:00:00	3wld-A	4.3	6.8	83	393	2	MOLECULE:	MYOSIN LIGHT CHAIN KINASE, GREEN FL
65:00:00	7aug-A	4.3	6.8	82	398	2	MOLECULE:	RSGCAMP1.3
66:00:00	3u1w-A	4.3	3.1	74	249	1	MOLECULE:	HYPOTHETICAL PERIPLASMIC PROTEIN
67:00:00	4pr7-A	4.2	6.8	73	171	7	MOLECULE:	OLIGOGALACTURONATE-SPECIFIC PORIN K
68:00:00	8feh-D	4.2	6.5	70	268	7	MOLECULE:	MINOR CAPSID PROTEIN A1 FUSION
69:00:00	5yvf-A	4.2	3.7	73	322	4	MOLECULE:	BFA1
70:00:00	6i1m-A	4.2	4	61	93	3	MOLECULE:	CYSTATIN
71:00:00	5fq6-M	4.2	9.5	95	948	9	MOLECULE:	PUTATIVE LIPOPROTEIN
72:00:00	7sjy-A	4.2	8.3	78	313	4	MOLECULE:	ANTI-SIGMA-I FACTOR RSGI9
73:00:00	6iqt-A	4.2	5.3	68	135	10	MOLECULE:	CAG PATHOGENICITY ISLAND PROTEIN (C
74:00:00	6r2q-B	4.2	7.4	91	649	4	MOLECULE:	CYSTATHIONINE BETA-SYNTHASE
75:00:00	7oqe-P	4.2	5.5	74	1186	8	MOLECULE:	PROTEIN NAM8
76:00:00	4lzi-A	4.1	7.1	69	277	10	MOLECULE:	MULTICYSTATIN
77:00:00	8cli-B	4.1	4	66	689	8	MOLECULE:	GENERAL TRANSCRIPTION FACTOR 3C POL
78:00:00	6dbj-B	4.1	6	77	352	12	MOLECULE:	RECOMBINATION ACTIVATING GENE 1 - M
79:00:00	4p42-A	4.1	7.4	85	455	8	MOLECULE:	EXTENDED SYNAPTOTAGMIN-2
80:00:00	3f8x-B	4.1	2.8	49	132	4	MOLECULE:	PUTATIVE DELTA-5-3-KETOSTEROID ISOM
81:00:00	6zrd-A	4.1	6.3	77	411	5	MOLECULE:	HISTONE-BINDING PROTEIN RBBP4
82:00:00	6jp6-A	4.1	4.7	72	998	4	MOLECULE:	TRNA (GUANOSINE(34)-2'-O)-METHYLTRA
83:00:00	6m63-A	4.1	6.8	81	359	2	MOLECULE:	CHIMERA OF CYCLIC NUCLEOTIDE-GATED
84:00:00	3cu3-A	4.1	6.4	74	162	7	MOLECULE:	DOMAIN OF UNKNOWN FUNCTION WITH A C
85:00:00	3fsd-A	4.1	6	67	121	4	MOLECULE:	NTF2-LIKE PROTEIN OF UNKNOWN FUNCTI
86:00:00	5d17-I	4.1	3.1	65	158	5	MOLECULE:	TRANSPOSON TN7 TRANSPOSITION PROTEI
87:00:00	8bys-A	4	9.8	85	323	5	MOLECULE:	S-LAYER HOMOLOGY DOMAIN-CONTAINING
88:00:00	7qe7-I	4	6.4	69	742	6	MOLECULE:	ANAPHASE-PROMOTING COMPLEX SUBUNIT
89:00:00	2ags-A	4	7	66	634	6	MOLECULE:	SIALIDASE
90:00:00	7oc9-A	4	5.1	70	133	7	MOLECULE:	BD0675

91:00:00	7cd7-A	4	3.6	64	99	8	MOLECULE:	GREEN FLUORESCENT PROTEIN
92:00:00	2obd-A	4	5	79	472	8	MOLECULE:	CHOLESTERYL ESTER TRANSFER PROTEIN
93:00:00	1uyn-X	4	6.9	94	279	7	MOLECULE:	NALP
94:00:00	8sy3-A	4	5.7	69	209	7	MOLECULE:	BURP DOMAIN-CONTAINING PROTEIN
95:00:00	4xzv-B	4	4.1	65	142	6	MOLECULE:	MALTOSE-BINDING PERIPLASMIC PROTEIN
96:00:00	3vu4-A	4	4.1	66	319	5	MOLECULE:	KMHSV2
97:00:00	3cnx-B	4	4.9	61	146	10	MOLECULE:	UNCHARACTERIZED PROTEIN
98:00:00	5o68-B	4	6.4	84	274	6	MOLECULE:	FAPF
99:00:00	7px8-D	4	4.8	71	699	4	MOLECULE:	ACYLAMINO-ACID-RELEASING ENZYME
0:00	7px8-C	4	4.5	70	699	4	MOLECULE:	ACYLAMINO-ACID-RELEASING ENZYME
1:00	7whm-A	3.9	4.7	96	1214	5	MOLECULE:	PPPDE DOMAIN-CONTAINING PROTEIN
2:00	6qp8-A	3.9	4.5	82	622	7	MOLECULE:	IP13724P
3:00	6zmq-A	3.9	6	61	589	5	MOLECULE:	CYTOCHROME C-TYPE BIOGENESIS PROTEI
4:00	7agv-F	3.9	9.8	51	163	8	MOLECULE:	K(+)/H(+) ANTIPORTER SUBUNIT KHTT
5:00	3csl-A	3.9	7	97	753	4	MOLECULE:	HASR PROTEIN
6:00	1ww1-A	3.9	7.6	73	250	8	MOLECULE:	TRNASE Z
7:00	8rf0-A	3.9	4.4	71	2780	4	MOLECULE:	CYCLIC BETA-(1,2)-GLUCAN SYNTHASE N
8:00	4aip-B	3.9	7.1	97	665	6	MOLECULE:	FE-REGULATED PROTEIN B
9:00	5gyd-C	3.9	7.6	84	228	10	MOLECULE:	MITOCHONDRIAL DISTRIBUTION AND MORP
10:00	2gjv-A	3.9	2.9	71	136	7	MOLECULE:	PUTATIVE CYTOPLASMIC PROTEIN
11:00	4epa-A	3.9	11.8	97	632	3	MOLECULE:	PESTICIN RECEPTOR
12:00	8asw-A	3.9	5.6	58	1255	5	MOLECULE:	ELONGATOR COMPLEX PROTEIN 1
13:00	3nm7-A	3.9	2.3	52	84	6	MOLECULE:	UNCHARACTERIZED PROTEIN
14:00	8e2g-A	3.9	6.2	66	523	8	MOLECULE:	BACULOVIRAL IAP REPEAT-CONTAINING P
15:00	7uil-p	3.9	9.1	73	903	8	MOLECULE:	MEDIATOR OF RNA POLYMERASE II TRANS
16:00	7xho-P	3.9	7.1	80	224	1	MOLECULE:	CENTROMERE PROTEIN C
17:00	6ht9-B	3.8	4.5	70	316	7	MOLECULE:	ASTACIN
18:00	3lh4-A	3.8	4.6	64	115	5	MOLECULE:	SECRETED CYSTATIN
19:00	2pff-B	3.8	4.3	76	2006	0	MOLECULE:	FATTY ACID SYNTHASE SUBUNIT ALPHA
20:00	3v89-A	3.8	9.3	95	853	5	MOLECULE:	TRANSFERRIN-BINDING PROTEIN A
21:00	1jmx-B	3.8	5.3	73	339	8	MOLECULE:	AMINE DEHYDROGENASE
22:00	6w3g-A	3.8	3.8	58	109	10	MOLECULE:	RD1NTF2_04
23:00	8ovw-O	3.8	7.3	78	241	5	MOLECULE:	CENTROMERE-BINDING PROTEIN 1
24:00:00	5jrk-A	3.8	4.2	63	695	6	MOLECULE:	DIPEPTIDYL AMINOPEPTIDASES/ACYLAMIN
25:00:00	3e99-A	3.8	5.7	74	149	7	MOLECULE:	BENZOATE 1,2-DIOXYGENASE BETA SUBUN
26:00:00	7qog-A	3.8	4.4	97	657	4	MOLECULE:	PORTAL PROTEIN GP20
27:00:00	3os7-A	3.8	4.3	82	338	5	MOLECULE:	GALACTOSE MUTAROTASE-LIKE PROTEIN
28:00:00	7apk-m	3.8	10.7	84	549	11	MOLECULE:	THO COMPLEX SUBUNIT 1
29:00:00	5mq0-t	3.8	2.8	58	438	7	MOLECULE:	YEAST UBC4 GENE FOR UBIQUITIN-CONJU
30:00:00	7t5q-I	3.8	6.1	90	283	2	MOLECULE:	ACTIN-RELATED PROTEIN 3
31:00:00	4hbr-A	3.8	4.1	78	140	5	MOLECULE:	PUTATIVE PERIPLASMIC PROTEIN
32:00:00	6baq-F	3.8	4.8	89	211	8	MOLECULE:	BPI FOLD-CONTAINING FAMILY A MEMBER
33:00:00	7el9-A	3.8	6.1	75	1731	0	MOLECULE:	RNA-DIRECTED RNA POLYMERASE L
34:00:00	8ovw-P	3.8	7.5	66	257	11	MOLECULE:	CENTROMERE-BINDING PROTEIN 1
35:00:00	8g8v-A	3.8	4.5	77	254	8	MOLECULE:	GTP CYCLOHYDROLASE FOLE2
36:00:00	7zb1-B	3.8	4.3	70	725	4	MOLECULE:	PROLYL ENDOPEPTIDASE

37:00:00	5tgn-B	3.8	3.7	56	108	9	MOLECULE:	UNCHARACTERIZED PROTEIN
38:00:00	7oqb-P	3.8	5.6	72	1186	8	MOLECULE:	U2 SNRNP COMPONENT HSH155
39:00:00	3ke7-B	3.7	4.4	62	132	16	MOLECULE:	PUTATIVE KETOSTEROID ISOMERASE
40:00:00	3dcz-A	3.7	3.3	70	170	7	MOLECULE:	PUTATIVE RNFG SUBUNIT OF ELECTRON T
41:00:00	6cb1-s	3.7	3.4	69	512	7	MOLECULE:	35S PRE-RIBOSOMAL RNA MISCRNA
42:00:00	6h04-I	3.7	8	79	501	1	MOLECULE:	COMPLEMENT COMPONENT C9
43:00:00	3db7-A	3.7	2.8	52	127	2	MOLECULE:	PUTATIVE CALCIUM-REGULATED PERIPLAS
44:00:00	1mg2-A	3.7	4	55	382	7	MOLECULE:	METHYLAMINE DEHYDROGENASE, HEAVY CH
45:00:00	2o3o-I	3.7	8	70	244	9	MOLECULE:	YYCI PROTEIN
46:00:00	7ep9-A	3.7	4	65	660	3	MOLECULE:	S9 FAMILY PEPTIDASE
47:00:00	1yem-B	3.7	5.9	56	166	0	MOLECULE:	CONSERVED HYPOTHETICAL PROTEIN PFU-
48:00:00	7n3v-A	3.7	4.5	67	266	10	MOLECULE:	LMCA
49:00:00	8c7g-A	3.7	5.3	67	607	3	MOLECULE:	MIC1 DOMAIN-CONTAINING PROTEIN
50:00:00	3wpv-A	3.7	4.9	80	538	8	MOLECULE:	BETA-FRUCTOFURANOSIDASE
51:00:00	4zkq-A	3.7	9.6	68	388	1	MOLECULE:	PUTATIVE UNCHARACTERIZED PROTEIN
52:00:00	7vud-A	3.7	4.8	74	535	7	MOLECULE:	CAROTENOID CLEAVAGE DIOXYGENASE 1
53:00:00	2qkd-A	3.7	8.4	66	384	5	MOLECULE:	ZINC FINGER PROTEIN ZPR1
54:00:00	7esn-A	3.7	3.6	64	435	2	MOLECULE:	L-RHAMNOSE-ALPHA-1,4-D-GLUCURONATE
55:00:00	6aig-A	3.7	7	64	258	8	MOLECULE:	DNA POLYMERASE SLIDING CLAMP 1
56:00:00	7wb4-f	3.7	5.5	61	321	7	MOLECULE:	OUTER NUP133
57:00:00	4jf7-A	3.7	4.7	67	501	6	MOLECULE:	HEMAGGLUTININ-NEURAMINIDASE
58:00:00	2oaj-A	3.7	6.5	77	875	5	MOLECULE:	PROTEIN SNI1
59:00:00	4h5j-B	3.7	5.5	73	347	3	MOLECULE:	GUANINE NUCLEOTIDE-EXCHANGE FACTOR
60:00:00	6h3i-A	3.7	6	70	2124	10	MOLECULE:	PROTEIN INVOLVED IN GLIDING MOTILIT
61:00:00	4q35-A	3.7	8.8	99	758	9	MOLECULE:	LPS-ASSEMBLY PROTEIN LPTD
62:00:00	5tf2-A	3.7	4.7	55	338	5	MOLECULE:	PROLACTIN REGULATORY ELEMENT-BINDIN
63:00:00	3en8-A	3.7	7.9	64	128	11	MOLECULE:	UNCHARACTERIZED NTF-2 LIKE PROTEIN
64:00:00	2wjq-A	3.7	6	61	205	0	MOLECULE:	PROBABLE N-ACETYLNEURAMINIC ACID OU
65:00:00	7btx-L	3.7	10.2	75	314	7	MOLECULE:	MITOCHONDRIAL OUTER MEMBRANE BETA-B
66:00:00	8xcj-F	3.7	8	62	420	6	MOLECULE:	MALTOPORIN
67:00:00	6ehd-A	3.7	6.3	89	322	4	MOLECULE:	OMPT PROTEIN
68:00:00	6lk8-E	3.7	13.6	65	1030	2	MOLECULE:	MGC83295 PROTEIN
69:00:00	8dte-A	3.7	4.7	62	172	16	MOLECULE:	PERIPLASMIC TRANSPORT PROTEIN
70:00:00	7e7w-C	3.7	3.2	66	264	9	MOLECULE:	FUCOSE-BINDING LECTIN PROTEIN,FUCOS
71:00:00	5kuy-G	3.7	3	57	84	7	MOLECULE:	HEMAGGLUTININ HA1
72:00:00	5bka-B	3.7	5.3	71	131	11	MOLECULE:	HYDROXYLACYL-COA DEHYDROGENASE
73:00:00	5ml9-B	3.7	4	62	103	3	MOLECULE:	LOW AFFINITY IMMUNOGLOBULIN GAMMA F
74:00:00	7wb4-J	3.7	5.2	73	1021	5	MOLECULE:	OUTER NUP133
75:00:00	5d17-C	3.7	3.1	67	158	4	MOLECULE:	TRANSPOSON TN7 TRANSPOSITION PROTEI
76:00:00	8p0w-A	3.6	3.2	48	190	4	MOLECULE:	COMM DOMAIN-CONTAINING PROTEIN 1
77:00:00	7mhu-A	3.6	7.1	68	374	13	MOLECULE:	EXO-ALPHA-SIALIDASE
78:00:00	6sg8-B	3.6	5.6	70	381	6	MOLECULE:	HEMAGGLUTININ-NEURAMINIDASE
79:00:00	5hax-A	3.6	5.9	74	698	11	MOLECULE:	NUCLEOPORIN NUP170
80:00:00	6sgo-A	3.6	5.5	67	136	3	MOLECULE:	SECRETED PROTEIN
81:00:00	6l77-A	3.6	3.5	67	214	7	MOLECULE:	MS5A
82:00:00	5bw0-F	3.6	2.7	53	91	6	MOLECULE:	TYPE II SECRETION SYSTEM PROTEIN J

83:00:00	1rn7-A	3.6	4.4	68	112	7	MOLECULE:	CYSTATIN D
84:00:00	6c0f-A	3.6	5.6	70	394	9	MOLECULE:	SACCHAROMYCES CEREVISIAE S288C 35S
85:00:00	9c4o-A	3.6	7.8	93	661	4	MOLECULE:	PYRROLOQUINOLINE QUINONE TRANSPORTE
86:00:00	6em5-m	3.6	3.4	72	645	11	MOLECULE:	5.8S RIBOSOMAL RNA
87:00:00	5own-A	3.6	7.9	82	295	4	MOLECULE:	PERFORIN-LIKE PROTEIN 1
88:00:00	4rhz-A	3.6	3.7	79	259	8	MOLECULE:	CRY23AA1
89:00:00	5vob-D	3.6	8.7	62	170	6	MOLECULE:	ENVELOPE GLYCOPROTEIN H
90:00:00	6z37-A	3.6	6.7	82	418	5	MOLECULE:	TODX
91:00:00	6w4q-C	3.6	4.3	62	758	10	MOLECULE:	TAIL FIBER
92:00:00	7e4h-A	3.6	6.8	76	439	4	MOLECULE:	SORTING ASSEMBLY MACHINERY 50 KDA S
93:00:00	2h36-X	3.6	2.5	49	108	6	MOLECULE:	HYPOTHETICAL PROTEIN SIFV0014
94:00:00	7eeb-F	3.6	5	67	1042	9	MOLECULE:	ENHANCED GREEN FLUORESCENT PROTEIN,
95:00:00	8cpz-B	3.6	10.4	69	2122	9	MOLECULE:	TCDA1
96:00:00	7eeb-H	3.6	6.1	75	916	5	MOLECULE:	ENHANCED GREEN FLUORESCENT PROTEIN,
97:00:00	8ppr-K	3.6	6.5	62	246	5	MOLECULE:	KINETOCHORE-ASSOCIATED PROTEIN DSN1
98:00:00	6cp9-E	3.6	8.6	62	119	10	MOLECULE:	CDIA
99:00:00	5nqz-A	3.6	5.2	70	267	9	MOLECULE:	FACTOR H BINDING PROTEIN,MAJOR OUTE
0:00	4hh4-C	3.6	5.9	65	257	5	MOLECULE:	CCBJ
1:00	8tie-g	3.6	6.7	76	1157	5	MOLECULE:	NUCLEOPORIN NUP120
2:00	2rjz-A	3.6	4.2	71	135	4	MOLECULE:	PILO PROTEIN
3:00	6zlt-B	3.6	8.3	90	933	4	MOLECULE:	SUSD HOMOLOG
4:00	3kvn-X	3.6	4.5	65	628	3	MOLECULE:	ESTERASE ESTA
5:00	7bc4-B	3.6	7.4	70	2054	4	MOLECULE:	FATTY ACID SYNTHASE SUBUNIT ALPHA
6:00	7st9-H	3.6	8.6	59	316	10	MOLECULE:	CHECKPOINT PROTEIN RAD24
7:00	2x2i-D	3.6	5.5	57	1025	9	MOLECULE:	ALPHA-1,4-GLUCAN LYASE ISOZYME 1
8:00	7kd9-F	3.6	6.3	83	225	7	MOLECULE:	GALLATE DECARBOXYLASE
9:00	7e7w-B	3.6	3.2	64	264	13	MOLECULE:	FUCOSE-BINDING LECTIN PROTEIN,FUCOS
10:00	6w40-B	3.6	2.4	48	115	2	MOLECULE:	DENOVO NTF2
11:00	5d17-A	3.6	3.2	67	159	4	MOLECULE:	TRANSPOSON TN7 TRANSPOSITION PROTEI
12:00	5d17-L	3.6	3.2	67	158	4	MOLECULE:	TRANSPOSON TN7 TRANSPOSITION PROTEI
13:00	6gel-B	3.6	7	68	528	3	MOLECULE:	GREEN FLUORESCENT PROTEIN,OPTIMIZED
14:00	5d17-F	3.6	3.3	67	158	4	MOLECULE:	TRANSPOSON TN7 TRANSPOSITION PROTEI
15:00	8j1g-B	3.5	5.3	76	313	7	MOLECULE:	ORNITHINE CYCLODEAMINASE FAMILY PRO
16:00	6hsy-A	3.5	4.5	59	190	5	MOLECULE:	TOLUENE TOLERANCE PROTEIN TTG2D
17:00	1y8c-A	3.5	7.6	61	246	7	MOLECULE:	S-ADENOSYLMETHIONINE-DEPENDENT METH
18:00	2gsk-A	3.5	12.7	96	590	7	MOLECULE:	VITAMIN B12 TRANSPORTER BTUB
19:00	8dml-B	3.5	3.8	61	133	3	MOLECULE:	VTRA
20:00	7krw-A	3.5	6.1	74	608	12	MOLECULE:	CHAPERONE PROTEIN DNAK FUSED WITH S
21:00	8y7e-3	3.5	3.5	57	1193	9	MOLECULE:	PRE-MRNA
22:00	3boy-A	3.5	4.9	83	150	4	MOLECULE:	5'-R(*UP*UP*UP*AP*GP*UP*UP*UP*UP*UP
23:00	4zn4-B	3.5	4.9	68	411	6	MOLECULE:	SQT1
24:00:00	2gtl-N	3.5	3.8	69	220	1	MOLECULE:	EXTRACELLULAR GLOBIN 4
25:00:00	5efv-B	3.5	3.9	70	635	7	MOLECULE:	PHI ETA ORF 56-LIKE PROTEIN
26:00:00	2gvh-B	3.5	3.6	74	250	1	MOLECULE:	AGR_L_2016P
27:00:00	2h1t-A	3.5	12	61	186	3	MOLECULE:	HYPOTHETICAL PROTEIN
28:00:00	3h51-A	3.5	5.1	64	142	9	MOLECULE:	PUTATIVE CALCIUM/CALMODULIN DEPENDE

29:00:00	6ud7-A	3.5	5.4	75	426	3	MOLECULE:	DDB1- AND CUL4-ASSOCIATED FACTOR 15
30:00:00	7s00-c	3.5	6.3	66	484	6	MOLECULE:	DNA-DIRECTED RNA POLYMERASE BETA SU
31:00:00	5ldt-A	3.5	8.2	84	403	2	MOLECULE:	MOMP PORIN
32:00:00	5h3z-A	3.5	6.5	76	1113	1	MOLECULE:	UNCHARACTERIZED PROTEIN
33:00:00	5n1a-B	3.5	4	65	706	3	MOLECULE:	UTP4
34:00:00	5z8o-B	3.5	4.8	71	146	6	MOLECULE:	CYCLASE/DEHYDRASE
35:00:00	2n2l-A	3.5	3.1	59	156	3	MOLECULE:	OUTER MEMBRANE PROTEIN X
36:00:00	4uvq-A	3.5	5.6	69	263	4	MOLECULE:	THIAZOLINE OXIDASE/SUBTILISIN-LIKE
37:00:00	7e85-A	3.5	4.2	52	114	4	MOLECULE:	SNOAL-LIKE DOMAIN-CONTAINING PROTEI
38:00:00	4hvt-A	3.5	4.8	78	691	8	MOLECULE:	POST-PROLINE CLEAVING ENZYME
39:00:00	3k7c-A	3.5	4	58	108	7	MOLECULE:	PUTATIVE NTF2-LIKE TRANSPEPTIDASE
40:00:00	6abo-A	3.5	5.8	51	213	8	MOLECULE:	DNA REPAIR PROTEIN XRCC4
41:00:00	6h0f-J	3.5	6.2	74	842	4	MOLECULE:	DNA DAMAGE-BINDING PROTEIN 1,DNA DA
42:00:00	7t5q-J	3.5	6.4	92	291	5	MOLECULE:	ACTIN-RELATED PROTEIN 3
43:00:00	8ptx-B	3.5	5.1	76	800	7	MOLECULE:	ELONGATOR COMPLEX PROTEIN 1
44:00:00	7q4p-C	3.5	3.6	54	878	4	MOLECULE:	SPLICING FACTOR 3A SUBUNIT 2
45:00:00	6tzk-A	3.5	6.7	77	451	4	MOLECULE:	CELLULOSE SYNTHASE OPERON PROTEIN C
46:00:00	5mt2-A	3.5	4.8	84	352	7	MOLECULE:	BETA-GALACTOSIDASE
47:00:00	7zsk-A	3.5	5.2	72	1482	3	MOLECULE:	PUTATIVE POLYKETIDE SYNTHASE
48:00:00	3qv0-A	3.5	3.3	61	179	3	MOLECULE:	MITOCHONDRIAL ACIDIC PROTEIN MAM33
49:00:00	6z8y-A	3.5	10.7	86	658	6	MOLECULE:	PUTATIVE COPPER TRANSPORT OUTER MEM
50:00:00	3grd-A	3.5	5.5	71	133	4	MOLECULE:	UNCHARACTERIZED NTF2-SUPERFAMILY PR
51:00:00	6rw6-B	3.5	6.3	64	2485	2	MOLECULE:	TCDA1
52:00:00	7qe7-A	3.5	5.4	71	1648	10	MOLECULE:	ANAPHASE-PROMOTING COMPLEX SUBUNIT
53:00:00	3k4x-A	3.5	5.2	60	758	5	MOLECULE:	PROLIFERATING CELL NUCLEAR ANTIGEN
54:00:00	7ne0-D	3.5	4.9	60	161	8	MOLECULE:	NETRIN-1
55:00:00	5bun-C	3.5	8.9	72	433	6	MOLECULE:	OUTER MEMBRANE PROTEIN
56:00:00	3hx8-A	3.5	5.2	66	128	6	MOLECULE:	PUTATIVE KETOSTEROID ISOMERASE
57:00:00	7e7n-A	3.5	3.7	61	259	10	MOLECULE:	FUCOSE-BINDING LECTIN PROTEIN,FUCOS
58:00:00	6q0r-C	3.5	2.9	58	183	5	MOLECULE:	DNA DAMAGE-BINDING PROTEIN 1
59:00:00	7c5w-B	3.5	4.8	63	163	3	MOLECULE:	IOTA-CARBONIC ANHYDRASE
60:00:00	3rwa-F	3.5	5.3	62	232	2	MOLECULE:	FLUORESCENT PROTEIN FP480
61:00:00	3rwa-B	3.5	5.3	62	232	2	MOLECULE:	FLUORESCENT PROTEIN FP480
62:00:00	6ogb-B	3.5	9	57	225	5	MOLECULE:	CRYSTAL STRUCTURE OF GREEN FLUORESC
63:00:00	6gel-A	3.5	7.1	68	530	3	MOLECULE:	GREEN FLUORESCENT PROTEIN,OPTIMIZED
64:00:00	4w6g-B	3.5	8.5	55	217	5	MOLECULE:	FLUORESCENT PROTEIN D190C
65:00:00	4wvm-B	3.4	6.3	80	616	6	MOLECULE:	STONUSTOXIN SUBUNIT ALPHA
66:00:00	7nyw-B	3.4	12.2	72	858	7	MOLECULE:	CHROMOSOME PARTITION PROTEIN MUKB
67:00:00	7kzn-A	3.4	4.1	66	315	5	MOLECULE:	HEAVY CHAIN ALPHA
68:00:00	5nj5-A	3.4	10.6	89	480	4	MOLECULE:	METALLOPROTEASE TLDD
69:00:00	5mtz-A	3.4	6.1	75	768	5	MOLECULE:	RIBONUCLEASE Z
70:00:00	5kpe-A	3.4	4.3	58	120	5	MOLECULE:	DE NOVO BETA SHEET DESIGN PROTEIN O
71:00:00	7pd7-B	3.4	5.1	75	251	3	MOLECULE:	METHYLTRANSFERASE
72:00:00	6gie-A	3.4	11.1	77	218	6	MOLECULE:	33-36 KDA OUTER MEMBRANE PROTEIN
73:00:00	2mc8-A	3.4	3.7	63	114	5	MOLECULE:	UNCHARACTERIZED PROTEIN
74:00:00	4irt-A	3.4	4.4	65	398	5	MOLECULE:	UNCHARACTERIZED PROTEIN

75:00:00	6lvb-A	3.4	4.2	68	762	3	MOLECULE:	N,N-DIMETHYLFORMAMIDASE LARGE SUBUN
76:00:00	6tyd-V	3.4	7.5	89	219	10	MOLECULE:	LIM DOMAIN-BINDING PROTEIN 1
77:00:00	6h0a-A	3.4	5.6	77	339	5	MOLECULE:	SERUM PARAOXONASE-1 BY DIRECTED EVO
78:00:00	5tc1-M	3.4	5	77	330	4	MOLECULE:	CAPSID PROTEIN
79:00:00	7zao-A	3.4	7.6	75	429	8	MOLECULE:	SIALIDASE (NEURAMINIDASE) FAMILY PR
80:00:00	3kg7-C	3.4	5.7	80	287	5	MOLECULE:	CURH
81:00:00	6jzf-A	3.4	5.7	65	153	5	MOLECULE:	PLASTID DIVISION PROTEIN CDP1, CHLO
82:00:00	7nyw-A	3.4	12	72	685	7	MOLECULE:	CHROMOSOME PARTITION PROTEIN MUKB
83:00:00	7czf-B	3.4	4.1	62	644	2	MOLECULE:	EPHRIN TYPE-A RECEPTOR 2
84:00:00	6cd2-C	3.4	4.6	77	727	8	MOLECULE:	CHAPERONE PROTEIN PAPD
85:00:00	5a1u-E	3.4	5.3	69	822	3	MOLECULE:	ADP-RIBOSYLATION FACTOR 1
86:00:00	7azn-A	3.4	4.6	71	177	6	MOLECULE:	PROTEIN ASTER-C
87:00:00	4n58-A	3.4	4.4	64	266	6	MOLECULE:	PECTOCIN M2
88:00:00	4y25-A	3.4	7.5	85	290	7	MOLECULE:	POLY-BETA-1,6-N-ACETYL-D-GLUCOSAMIN
89:00:00	7y22-Y	3.4	4.3	65	794	8	MOLECULE:	PHAGE CONNECTOR PROTEIN
90:00:00	3pgb-A	3.4	4.8	93	740	4	MOLECULE:	PUTATIVE UNCHARACTERIZED PROTEIN
91:00:00	4o3v-B	3.4	5.5	71	140	7	MOLECULE:	VIRB8-LIKE PROTEIN OF TYPE IV SECRE
92:00:00	3h3h-B	3.4	4.3	53	120	2	MOLECULE:	UNCHARACTERIZED SNOAL-LIKE PROTEIN
93:00:00	3hsa-A	3.4	3.9	53	124	8	MOLECULE:	PLECKSTRIN HOMOLOGY DOMAIN
94:00:00	4zsv-A	3.4	11.3	65	294	2	MOLECULE:	UNCHARACTERIZED PROTEIN
95:00:00	3kvp-D	3.4	4.2	46	49	2	MOLECULE:	UNCHARACTERIZED PROTEIN YMZC
96:00:00	4rdr-A	3.4	6.6	77	706	5	MOLECULE:	ZNUD
97:00:00	1u1i-A	3.4	4.3	65	392	9	MOLECULE:	MYO-INOSITOL-1-PHOSPHATE SYNTHASE
98:00:00	6hxv-A	3.4	5.6	54	163	2	MOLECULE:	COILED-COIL DOMAIN-CONTAINING PROTE
99:00:00	8chv-B	3.4	2.1	47	147	6	MOLECULE:	TRANSCRIPTIONAL ACTIVATOR PROTEIN P
0:00	4qtq-A	3.4	3.7	62	209	6	MOLECULE:	XAC2610 PROTEIN
1:00	7e7u-B	3.4	4	62	270	10	MOLECULE:	FUCOSE-BINDING LECTIN PROTEIN,FUCOS
2:00	7kd9-I	3.4	6.5	83	226	7	MOLECULE:	GALLATE DECARBOXYLASE
3:00	6w3d-A	3.4	3.7	57	114	12	MOLECULE:	RD1NTF2_05
4:00	5bka-D	3.4	5.3	65	130	12	MOLECULE:	HYDROXYLACYL-COA DEHYDROGENASE
5:00	7kd9-C	3.4	6	83	227	7	MOLECULE:	GALLATE DECARBOXYLASE
6:00	3sg2-A	3.4	7.1	78	394	5	MOLECULE:	MYOSIN LIGHT CHAIN KINASE, GREEN FL
7:00	7rrk-C	3.4	4.9	62	213	10	MOLECULE:	FLUORESCENT PROTEIN DRONPA
8:00	3rwa-C	3.4	6.6	65	232	3	MOLECULE:	FLUORESCENT PROTEIN FP480
9:00	5o68-A	3.4	6.4	83	272	6	MOLECULE:	FAPF
10:00	5d16-B	3.4	3.2	65	157	5	MOLECULE:	TRANSPOSON TN7 TRANSPOSITION PROTEI
11:00	5d17-K	3.4	3.1	63	157	5	MOLECULE:	TRANSPOSON TN7 TRANSPOSITION PROTEI
12:00	8f2r-B	3.3	3.6	48	199	2	MOLECULE:	COMM DOMAIN-CONTAINING PROTEIN 1
13:00	5k36-F	3.3	9.5	61	215	7	MOLECULE:	EXOSOME COMPLEX COMPONENT RRP45
14:00	7kd9-A	3.3	6.9	88	231	9	MOLECULE:	GALLATE DECARBOXYLASE
15:00	6kaw-A	3.3	7.3	75	381	4	MOLECULE:	CGHA
16:00	6uio-C	3.3	4.2	67	112	7	MOLECULE:	CYSTATIN-8
17:00	3rob-A	3.3	5.4	71	131	4	MOLECULE:	UNCHARACTERIZED CONSERVED PROTEIN
18:00	5e1v-B	3.3	3.6	69	274	1	MOLECULE:	POLYKETIDE SYNTHASE PKSL
19:00	1eut-A	3.3	4.3	64	601	6	MOLECULE:	SIALIDASE
20:00	2yfr-A	3.3	5.7	80	533	5	MOLECULE:	LEVANSUCRASE

21:00	8ih8-D	3.3	2.6	56	130	7	MOLECULE:	ANTI-SIGMA-F FACTOR ANTAGONIST RSFB
22:00	3ci0-K	3.3	3.5	70	280	9	MOLECULE:	PSEUDOPILIN GSPI
23:00	8cyk-A	3.3	4.9	67	131	6	MOLECULE:	HALC1_878
24:00:00	8qca-C	3.3	4.3	71	384	6	MOLECULE:	ANTIVIRAL HELICASE SKI2
25:00:00	8jbq-A	3.3	5.6	79	692	3	MOLECULE:	HEMOLYSIN
26:00:00	6myv-C	3.3	9.1	67	526	1	MOLECULE:	SIALIDASE26
27:00:00	6urg-A	3.3	4.1	70	1202	6	MOLECULE:	CLEAVAGE AND POLYADENYLATION SPECIF
28:00:00	5hal-A	3.3	3.4	60	103	7	MOLECULE:	UNCHARACTERIZED PROTEIN
29:00:00	4i0o-A	3.3	5	77	463	5	MOLECULE:	PROTEIN ELYS
30:00:00	3emo-A	3.3	3.4	53	126	4	MOLECULE:	HIA (ADHESIN)
31:00:00	4ebr-A	3.3	4	83	157	7	MOLECULE:	UBIQUITIN-LIKE-CONJUGATING ENZYME A
32:00:00	2gia-G	3.3	4.9	63	155	6	MOLECULE:	MITOCHONDRIAL RNA-BINDING PROTEIN 2
33:00:00	4ccv-A	3.3	4.2	62	115	3	MOLECULE:	HISTIDINE-RICH GLYCOPROTEIN
34:00:00	1nkg-A	3.3	9.7	58	508	10	MOLECULE:	RHAMNOGALACTURONASE B
35:00:00	3a76-B	3.3	6.3	79	153	8	MOLECULE:	GAMMA-HEXACHLOROCYCLOHEXANE DEHYDRO
36:00:00	8f2r-I	3.3	3.4	48	198	10	MOLECULE:	COMM DOMAIN-CONTAINING PROTEIN 1
37:00:00	2rgq-B	3.3	5.5	67	134	7	MOLECULE:	DOMAIN OF UNKNOWN FUNCTION WITH A C
38:00:00	6r8f-E	3.3	4.1	64	210	0	MOLECULE:	LYS-63-SPECIFIC DEUBIQUITINASE BRCC
39:00:00	4i4k-A	3.3	5.3	63	138	2	MOLECULE:	UNCHARACTERIZED PROTEIN SGCJ
40:00:00	6j6a-A	3.3	9.7	87	442	3	MOLECULE:	ZINC-DEPENDENT PROTEASE, TLDD/PMBA
41:00:00	5h7z-B	3.3	6.5	66	302	3	MOLECULE:	UNCHARACTERIZED PROTEIN
42:00:00	5ogs-A	3.3	5.5	63	403	10	MOLECULE:	WD REPEAT AND HMG-BOX DNA-BINDING P
43:00:00	2kxg-A	3.3	3.8	68	95	7	MOLECULE:	ASPARTIC PROTEASE INHIBITOR
44:00:00	6w3w-A	3.3	5.4	54	107	11	MOLECULE:	DENOVO NTF2
45:00:00	6hdv-A	3.3	9	81	136	6	MOLECULE:	AFIFAVIDIN
46:00:00	8bmx-C	3.3	11.1	92	633	4	MOLECULE:	PUTATIVE TONB-LINKED OUTER MEMBRANE
47:00:00	8adl-J	3.3	4	52	801	2	MOLECULE:	MTC5 ISOFORM 1
48:00:00	7svm-A	3.3	5.3	89	852	7	MOLECULE:	DIPEPTIDYL PEPTIDASE 8
49:00:00	5dfz-B	3.3	6	70	1224	1	MOLECULE:	VACUOLAR PROTEIN SORTING-ASSOCIATED
50:00:00	6igb-A	3.3	4.2	68	363	4	MOLECULE:	PERIPLASMIC GLUCONOLACTONASE, PPGL
51:00:00	4ui9-A	3.3	5.8	75	1441	7	MOLECULE:	ANAPHASE-PROMOTING COMPLEX SUBUNIT
52:00:00	8xcj-A	3.3	10.3	77	422	4	MOLECULE:	MALTOPORIN
53:00:00	7pi3-A	3.3	5.9	76	328	1	MOLECULE:	CYSTEINE-RICH PROTECTIVE ANTIGEN
54:00:00	6q50-A	3.3	5.2	68	318	7	MOLECULE:	MPT-4
55:00:00	7s5n-B	3.3	5.7	65	128	2	MOLECULE:	MYMAA.17060.A
56:00:00	6ewz-A	3.3	9.3	62	202	6	MOLECULE:	GTP PYROPHOSPHOKINASE
57:00:00	4acv-B	3.3	3.1	61	122	2	MOLECULE:	PROPHAGE LAMBDALM01, ANTIGEN B
58:00:00	3f40-A	3.3	5.6	63	112	5	MOLECULE:	UNCHARACTERIZED NTF2-LIKE PROTEIN
59:00:00	6gn5-A	3.3	5.2	81	177	5	MOLECULE:	GRAM DOMAIN-CONTAINING PROTEIN 1C
60:00:00	4i8e-X	3.3	2.3	51	359	10	MOLECULE:	PLATELET BINDING PROTEIN GSPB
61:00:00	8fs6-G	3.3	3.3	59	298	3	MOLECULE:	CHECKPOINT PROTEIN RAD24
62:00:00	8jxe-A	3.3	7	68	1469	12	MOLECULE:	LDL RECEPTOR RELATED PROTEIN 2
63:00:00	1wnh-A	3.3	4.4	68	220	6	MOLECULE:	LATEXIN
64:00:00	6f37-A	3.3	3.2	54	113	9	MOLECULE:	NANO3,FUCOSE-BINDING LECTIN PROTEIN
65:00:00	8j0n-A	3.3	4.6	62	949	5	MOLECULE:	ER MEMBRANE PROTEIN COMPLEX SUBUNIT
66:00:00	1r5m-A	3.3	8.6	53	351	6	MOLECULE:	SIR4-INTERACTING PROTEIN SIF2

67:00:00	8ck0-A	3.3	4.2	90	478	7	MOLECULE:	PORTAL PROTEIN
68:00:00	8igd-A	3.3	2.9	54	82	4	MOLECULE:	DOUBLE-STRANDED RNA-BINDING DOMAIN
69:00:00	5xta-A	3.3	3.4	71	120	1	MOLECULE:	VIRK PROTEIN
70:00:00	7kpr-A	3.3	7.9	89	403	3	MOLECULE:	PROTEIN PHOSPHATASE 1H
71:00:00	4hxg-F	3.3	5.9	64	614	3	MOLECULE:	PUTATIVE UNCHARACTERIZED PROTEIN PH
72:00:00	7qok-A	3.3	6	51	1361	6	MOLECULE:	MUZZLE PROTEIN GP44
73:00:00	4rlc-A	3.3	6.9	75	135	4	MOLECULE:	OUTER MEMBRANE PORIN F
74:00:00	5uac-C	3.3	5.6	62	1342	6	MOLECULE:	DNA-DIRECTED RNA POLYMERASE SUBUNIT
75:00:00	5l8s-A	3.3	4.1	68	604	6	MOLECULE:	AMINO ACYL PEPTIDASE
76:00:00	7d9b-A	3.3	4.9	64	588	5	MOLECULE:	ALPHA-GLYCOSIDASE
77:00:00	4zgv-A	3.3	12.5	89	809	1	MOLECULE:	FERREDOXIN RECEPTOR
78:00:00	8xci-Z	3.3	7	57	383	7	MOLECULE:	TIP ATTACHMENT PROTEIN J
79:00:00	6jql-A	3.3	3.8	69	678	6	MOLECULE:	BIFUNCTIONAL PROTEIN PAAZ
80:00:00	8ghl-C	3.3	9.9	51	377	6	MOLECULE:	PROTEIN HIR1
81:00:00	6hpv-A	3.3	5.4	70	299	6	MOLECULE:	FETUIN-B
82:00:00	8ghn-C	3.3	9.9	51	377	6	MOLECULE:	PROTEIN HIR1
83:00:00	7e85-C	3.3	4	53	114	9	MOLECULE:	SNOAL-LIKE DOMAIN-CONTAINING PROTEI
84:00:00	7e85-B	3.3	4.7	55	114	11	MOLECULE:	SNOAL-LIKE DOMAIN-CONTAINING PROTEI
85:00:00	4w6r-P	3.3	8.7	56	204	7	MOLECULE:	FLUORESCENT PROTEIN D102C
86:00:00	5o67-A	3.3	7.7	80	282	9	MOLECULE:	FAPF
87:00:00	3rwa-E	3.3	5.2	62	232	2	MOLECULE:	FLUORESCENT PROTEIN FP480
88:00:00	4u1f-A	3.3	6	73	474	4	MOLECULE:	EUKARYOTIC TRANSLATION INITIATION F
89:00:00	6mxw-H	3.3	4.7	61	215	11	MOLECULE:	PHOTOSWITCHABLE PROTEIN TETDRON
90:00:00	5o68-J	3.3	7	93	268	4	MOLECULE:	FAPF
91:00:00	5o68-L	3.3	5.4	76	258	12	MOLECULE:	FAPF
92:00:00	3sg7-A	3.3	6.7	83	398	2	MOLECULE:	MYOSIN LIGHT CHAIN KINASE, GREEN FL
93:00:00	5exb-L	3.3	4.4	61	225	5	MOLECULE:	GREEN FLUORESCENT PROTEIN
94:00:00	7t4e-A	3.2	5.6	72	307	6	MOLECULE:	EPX1
95:00:00	6cb1-k	3.2	3.2	52	77	8	MOLECULE:	35S PRE-RIBOSOMAL RNA MISCRNA
96:00:00	6imj-A	3.2	3.7	70	412	3	MOLECULE:	DNA LIGASE
97:00:00	5ien-A	3.2	5.8	71	129	4	MOLECULE:	D30H_F14
98:00:00	6s6z-A	3.2	6.2	67	1083	4	MOLECULE:	BETA-GALACTOSIDASE
99:00:00	5u6y-A	3.2	5.3	66	459	9	MOLECULE:	CALCIUM/CALMODULIN-DEPENDENT PROTEI
0:00	8b61-B	3.2	4.4	60	200	7	MOLECULE:	CONSERVED HYPOTHETICAL LIPOPROTEIN
1:00	7zei-A	3.2	4.9	71	310	7	MOLECULE:	CH_GAF159A
2:00	1aq3-A	3.2	4.6	78	129	6	MOLECULE:	RNA (5'-
3:00	7jmn-P	3.2	7.3	91	810	5	MOLECULE:	MEDIATOR OF RNA POLYMERASE II TRANS
4:00	8gdl-A	3.2	2.4	60	355	7	MOLECULE:	SECRETED SALIVARY ACID PHOSPHATASE
5:00	3a1j-A	3.2	7.2	65	265	8	MOLECULE:	CELL CYCLE CHECKPOINT CONTROL PROTE
6:00	7a0h-A	3.2	5.9	79	285	3	MOLECULE:	F-ACTIN-CAPPING PROTEIN SUBUNIT ALP
7:00	7st9-G	3.2	7.5	67	340	3	MOLECULE:	CHECKPOINT PROTEIN RAD24
8:00	5zbt-A	3.2	3.5	63	212	8	MOLECULE:	LECTIN-LIKE PROTEIN
9:00	5tse-A	3.2	3.6	74	136	3	MOLECULE:	LPS-ASSEMBLY LIPOPROTEIN LPTE
10:00	5w3s-A	3.2	5.3	81	485	6	MOLECULE:	MUCOLIPIN-3 ISOFORM 1
11:00	4aee-A	3.2	6.2	70	684	7	MOLECULE:	ALPHA AMYLASE, CATALYTIC REGION
12:00	7zgr-A	3.2	6	80	1253	5	MOLECULE:	PROTEIN CFT1

13:00	6sl1-A	3.2	6.7	65	2651	3	MOLECULE:	SERINE/THREONINE-PROTEIN KINASE TEL
14:00	6r6u-A	3.2	7.5	68	458	6	MOLECULE:	CIS-ACONITATE DECARBOXYLASE
15:00	6lyq-A	3.2	8.9	91	787	5	MOLECULE:	OUTER MEMBRANE PROTEIN ASSEMBLY FAC
16:00	6m1u-A	3.2	3.6	58	122	9	MOLECULE:	RNA N6-ADENOSINE-METHYLTRANSFERASE
17:00	8gqq-A	3.2	4.1	61	99	3	MOLECULE:	CYSTATIN-A2
18:00	7o5y-D	3.2	9	71	131	11	MOLECULE:	TYPE IV PILUS BIOGENESIS PROTEIN PI
19:00	6hmj-A	3.2	9	77	359	6	MOLECULE:	PUTATIVE PAS/PAC SENSOR PROTEIN
20:00	7vcf-I	3.2	7.5	76	363	8	MOLECULE:	TIC214
21:00	3qqz-A	3.2	4.5	63	243	2	MOLECULE:	PUTATIVE UNCHARACTERIZED PROTEIN YJ
22:00	4q51-B	3.2	3.2	73	265	8	MOLECULE:	UNCHARACTERIZED PROTEIN
23:00	5ft0-A	3.2	8.7	77	256	5	MOLECULE:	GP37
24:00:00	7rxu-A	3.2	3.3	71	134	7	MOLECULE:	LIPOPROTEIN
25:00:00	2yeq-A	3.2	4.8	72	522	7	MOLECULE:	ALKALINE PHOSPHATASE D
26:00:00	5wlz-C	3.2	9.3	51	206	6	MOLECULE:	DNA REPAIR PROTEIN XRCC4,MYOSIN-7
27:00:00	7ype-A	3.2	8.3	77	286	8	MOLECULE:	E301R
28:00:00	2xe4-A	3.2	5.3	70	721	3	MOLECULE:	OLIGOPEPTIDASE B
29:00:00	3vsf-C	3.2	3.3	71	482	3	MOLECULE:	RICIN B LECTIN
30:00:00	6dhx-A	3.2	5.8	69	182	7	MOLECULE:	TIPC2
31:00:00	5nxh-A	3.2	9.8	69	546	3	MOLECULE:	LONG-TAIL FIBER PROXIMAL SUBUNIT
32:00:00	6h3i-F	3.2	5.8	81	345	4	MOLECULE:	PROTEIN INVOLVED IN GLIDING MOTILIT
33:00:00	8pjn-2	3.2	3.8	56	156	5	MOLECULE:	E3 UBIQUITIN-PROTEIN TRANSFERASE RM
34:00:00	6ekv-A	3.2	3.6	72	718	6	MOLECULE:	TOXIN COMPLEX COMPONENT ORF-X2
35:00:00	7obm-A	3.2	4.5	67	624	9	MOLECULE:	PROLYL ENDOPEPTIDASE-LIKE
36:00:00	6sb5-J	3.2	6.5	76	543	7	MOLECULE:	MACROPHAGE-EXPRESSED GENE 1 PROTEIN
37:00:00	7nri-E	3.2	4.4	56	84	7	MOLECULE:	OUTER MEMBRANE PROTEIN ASSEMBLY FAC
38:00:00	6iys-O	3.2	8.3	59	245	7	MOLECULE:	OUTER SURFACE PROTEIN A
39:00:00	6w3f-A	3.2	5.3	59	115	8	MOLECULE:	RD1NTF2_05_I64F_A80G_T94P_D101K_L10
40:00:00	5d17-H	3.2	3.4	61	156	5	MOLECULE:	TRANSPOSON TN7 TRANSPOSITION PROTEI
41:00:00	4u1f-B	3.2	6	73	475	4	MOLECULE:	EUKARYOTIC TRANSLATION INITIATION F
42:00:00	8es4-E	3.1	4.3	69	772	6	MOLECULE:	GP35
43:00:00	6bym-A	3.1	4.4	80	200	11	MOLECULE:	STEROL-BINDING PROTEIN
44:00:00	3ggr-C	3.1	6.2	72	264	3	MOLECULE:	CELL CYCLE CHECKPOINT CONTROL PROTE
45:00:00	4w78-F	3.1	2.9	56	127	4	MOLECULE:	HYDRATASE CHSH1
46:00:00	2vf9-A	3.1	5	76	131	4	MOLECULE:	COAT PROTEIN
47:00:00	8bd7-S	3.1	5.2	62	1104	3	MOLECULE:	IFT88
48:00:00	7qpg-R	3.1	5.2	65	2208	8	MOLECULE:	PROTEIN ZWILCH HOMOLOG
49:00:00	7aed-B	3.1	5.7	60	138	5	MOLECULE:	PRGL
50:00:00	3hk4-A	3.1	5.2	60	118	5	MOLECULE:	MLR7391 PROTEIN
51:00:00	8vwr-A	3.1	5.4	71	154	3	MOLECULE:	ACYL DEHYDRATASE
52:00:00	5mdq-A	3.1	9.7	89	350	7	MOLECULE:	CHITOPORIN
53:00:00	1jki-A	3.1	10.8	59	525	3	MOLECULE:	MYO-INOSITOL-1-PHOSPHATE SYNTHASE
54:00:00	1l0o-A	3.1	3.2	62	141	5	MOLECULE:	ANTI-SIGMA F FACTOR
55:00:00	8alo-B	3.1	5.1	79	148	5	MOLECULE:	CHOLERA TOXIN TRANSCRIPTIONAL ACTIV
56:00:00	2zwa-B	3.1	3.8	57	684	2	MOLECULE:	LEUCINE CARBOXYL METHYLTRANSFERASE
57:00:00	7q3e-A	3.1	6.9	71	551	7	MOLECULE:	WD REPEAT-CONTAINING AND PLANAR CEL
58:00:00	8f2r-F	3.1	3.5	49	77	6	MOLECULE:	COMM DOMAIN-CONTAINING PROTEIN 1

59:00:00	6ekt-A	3.1	5.9	81	419	4	MOLECULE:	P-47 PROTEIN
60:00:00	6a70-B	3.1	5.3	78	704	6	MOLECULE:	POLYCYSTIN-2
61:00:00	8tie-a	3.1	4.3	64	1012	3	MOLECULE:	NUCLEOPORIN NUP120
62:00:00	1fi1-A	3.1	11.8	94	707	6	MOLECULE:	FERRICHROME-IRON RECEPTOR
63:00:00	2rau-A	3.1	6.7	62	350	2	MOLECULE:	PUTATIVE ESTERASE
64:00:00	7uyx-B	3.1	5.2	68	169	6	MOLECULE:	BACTERIOPHAGE PA1C GP2
65:00:00	5k47-A	3.1	8.2	82	484	7	MOLECULE:	POLYCYSTIN-2
66:00:00	3nqz-A	3.1	6.5	70	175	10	MOLECULE:	SECRETED METALLOPROTEASE MCP02
67:00:00	4ddg-A	3.1	6.9	60	399	7	MOLECULE:	UBIQUITIN-CONJUGATING ENZYME E2 D2,
68:00:00	7yls-A	3.1	5.9	74	158	8	MOLECULE:	AROMATIC-RING-HYDROXYLATING DIOXYGE
69:00:00	4l9h-A	3.1	3.3	75	155	8	MOLECULE:	F-BOX ONLY PROTEIN 7
70:00:00	7kra-A	3.1	9.8	70	694	4	MOLECULE:	ER MEMBRANE PROTEIN COMPLEX SUBUNIT
71:00:00	2wyh-A	3.1	9.7	75	905	7	MOLECULE:	ALPHA-MANNOSIDASE
72:00:00	4o2h-A	3.1	8.4	69	137	6	MOLECULE:	PROTEIN BCAM1869
73:00:00	4a7z-A	3.1	5.2	75	881	9	MOLECULE:	ALDOS-2-ULOSE DEHYDRATASE
74:00:00	7fh6-A	3.1	9.4	77	653	3	MOLECULE:	CYLK
75:00:00	7rag-A	3.1	7.6	63	170	3	MOLECULE:	LIPOPROTEIN
76:00:00	5kph-A	3.1	3.7	55	85	7	MOLECULE:	DE NOVO BETA SHEET DESIGN PROTEIN O
77:00:00	6wlw-U	3.1	5.8	79	205	6	MOLECULE:	V-TYPE PROTON ATPASE 21 KDA PROTEOL
78:00:00	2w5n-A	3.1	4.8	74	365	1	MOLECULE:	ALPHA-L-ARABINOFURANOSIDASE
79:00:00	8tum-a	3.1	4.7	55	143	0	MOLECULE:	TYPE IV MAJOR PILIN PROTEIN PILA
80:00:00	5edf-A	3.1	7.1	77	224	1	MOLECULE:	FRPC OPERON PROTEIN
81:00:00	7z8b-F	3.1	5.6	63	457	6	MOLECULE:	CULLIN-7
82:00:00	8qc6-A	3.1	7.1	74	366	7	MOLECULE:	OXIDOREDUCTASE
83:00:00	3o61-A	3.1	8.3	51	187	6	MOLECULE:	GDP-MANNOSE PYROPHOSPHATASE NUDK
84:00:00	5u0p-Q	3.1	7	66	508	6	MOLECULE:	MEDIATOR COMPLEX SUBUNIT 14
85:00:00	8thf-A	3.1	5.4	59	900	5	MOLECULE:	SPIKE PROTEIN S1,SPIKE GLYCOPROTEIN
86:00:00	6eus-A	3.1	7.8	71	346	4	MOLECULE:	DCAP-LIKE PROTEIN
87:00:00	2x3j-A	3.1	11	67	586	7	MOLECULE:	ACSD
88:00:00	6yhk-A	3.1	3.3	81	994	6	MOLECULE:	CYTOTOXIC NECROTIZING FACTOR
89:00:00	3duk-A	3.1	3.6	56	125	5	MOLECULE:	NTF2-LIKE PROTEIN OF UNKNOWN FUNCTI
90:00:00	3ebw-A	3.1	4.8	61	157	7	MOLECULE:	PER A 4 ALLERGEN
91:00:00	8ae1-A	3.1	7.4	91	953	5	MOLECULE:	S-LAYER PROTEIN SLPA
92:00:00	5wd6-A	3.1	4	68	195	10	MOLECULE:	SHORT PALATE, LUNG AND NASAL EPITHE
93:00:00	5chx-A	3.1	5.5	55	210	4	MOLECULE:	XRCC4-MYH7-1590-1657
94:00:00	3muu-A	3.1	6.5	65	623	5	MOLECULE:	STRUCTURAL POLYPROTEIN
95:00:00	4pj2-B	3.1	3.6	59	123	0	MOLECULE:	PUTATIVE EXPORTED PROTEIN
96:00:00	6ohh-B	3.1	5.3	64	128	6	MOLECULE:	EF1P2_MFAP2B
97:00:00	8a9y-E	3.1	4.3	72	497	8	MOLECULE:	GLYCOSIDE HYDROLASE FAMILY 32
98:00:00	7okx-O	3.1	4.5	53	106	4	MOLECULE:	DNA-DIRECTED RNA POLYMERASE II SUBU
99:00:00	6te9-F	3.1	2.9	61	134	8	MOLECULE:	ADAPTOR PROTEIN RCC01688
0:00	1oh1-A	3.1	3.7	63	109	13	MOLECULE:	STAPHOSTATIN A
1:00	6w90-A	3.1	3.7	53	130	6	MOLECULE:	NTF2 FOLD PROTEIN LOOP-HELIX-LOOP D
2:00	5xqi-B	3.1	4.5	55	276	2	MOLECULE:	PROTEIN ROGDI HOMOLOG
3:00	6jsh-B	3.1	4.5	70	1904	0	MOLECULE:	FATTY ACID SYNTHASE SUBUNIT BETA
4:00	8ghn-A	3.1	4.8	55	667	7	MOLECULE:	PROTEIN HIR1

5:00	5bka-C	3.1	5.1	63	126	11	MOLECULE:	HYDROXYLACYL-COA DEHYDROGENASE
6:00	7f0o-A	3.1	10	75	144	8	MOLECULE:	NSRQ
7:00	6isl-A	3.1	4.2	65	120	12	MOLECULE:	XIME, SNOAL-LIKE DOMAIN PROTEIN
8:00	3dxo-A	3.1	4.6	58	117	2	MOLECULE:	UNCHARACTERIZED SNOAL-LIKE PROTEIN
9:00	7e7w-A	3.1	3.3	68	264	9	MOLECULE:	FUCOSE-BINDING LECTIN PROTEIN,FUCOS
10:00	4nox-A	3.1	7.4	79	460	9	MOLECULE:	EUKARYOTIC TRANSLATION INITIATION F
11:00	5d17-B	3.1	3.2	60	158	5	MOLECULE:	TRANSPOSON TN7 TRANSPOSITION PROTEI
12:00	7dmx-B	3.1	5.1	57	209	7	MOLECULE:	PHOCL GREEN
13:00	3tmt-B	3.1	4.1	59	220	5	MOLECULE:	GREEN TO RED PHOTOCONVERTIBLE GPF-L
14:00	2ib6-A	3.1	4.2	62	225	6	MOLECULE:	YELLOW MUTANT CHROMO PROTEIN
15:00	6k96-B	3	8.5	75	343	3	MOLECULE:	FIVE-MEMBERED-CYCLITOL-PHOSPHATE SY
16:00	7pkn-O	3	4.7	56	210	4	MOLECULE:	CENTROMERE PROTEIN H
17:00	7rtn-A	3	11.6	62	270	5	MOLECULE:	OUTER CAPSID PROTEIN VP5
18:00	8g7w-B	3	4.6	81	1059	1	MOLECULE:	TYPE I PKS MODULE 4, MODULE 5
19:00	8s9t-B	3	11.5	71	185	7	MOLECULE:	CAS7-CAS5-CAS11
20:00	6e14-D	3	9.1	83	805	7	MOLECULE:	TYPE 1 FIMBRIN D-MANNOSE SPECIFIC A
21:00	7w63-B	3	7	77	395	6	MOLECULE:	TOXIN-COREGULATED PILUS BIOSYNTHESI
22:00	4ec6-A	3	5.5	61	109	7	MOLECULE:	PUTATIVE UNCHARACTERIZED PROTEIN
23:00	7aoh-B	3	5.8	77	1134	4	MOLECULE:	DNA-DIRECTED RNA POLYMERASE 147 KDA
24:00:00	8f2r-D	3	2.9	49	199	4	MOLECULE:	COMM DOMAIN-CONTAINING PROTEIN 1
25:00:00	2hhi-A	3	3.1	65	204	6	MOLECULE:	IMMUNOGENIC PROTEIN MPT64
26:00:00	6xd4-A	3	6.1	87	458	9	MOLECULE:	HEMOLYSIN
27:00:00	7pkq-N	3	4.2	69	195	3	MOLECULE:	MS35
28:00:00	3nja-D	3	4.1	51	105	8	MOLECULE:	PROBABLE GGDEF FAMILY PROTEIN
29:00:00	2hye-A	3	5.8	72	1140	10	MOLECULE:	DNA DAMAGE-BINDING PROTEIN 1
30:00:00	8p0w-J	3	3.2	48	202	2	MOLECULE:	COMM DOMAIN-CONTAINING PROTEIN 1
31:00:00	5x04-A	3	4.6	67	276	7	MOLECULE:	DODECANOYL-[ACYL-CARRIER-PROTEIN] H
32:00:00	6mrk-B	3	5.8	68	188	6	MOLECULE:	NUCLEAR RNA EXPORT FACTOR 2
33:00:00	6m36-I	3	2.6	48	104	4	MOLECULE:	SERINE-PROTEIN KINASE RSBW
34:00:00	5v1d-D	3	5.4	71	247	6	MOLECULE:	EIF2AK3 PROTEIN
35:00:00	6eu4-A	3	6.1	68	587	6	MOLECULE:	TAIL SPIKE PROTEIN
36:00:00	7kbj-I	3	5.4	59	153	8	MOLECULE:	NEUTRAL ALPHA-GLUCOSIDASE AB TRYPSI
37:00:00	4mkq-A	3	4.9	76	169	8	MOLECULE:	MONALYSIN
38:00:00	2ch9-A	3	5.9	73	126	3	MOLECULE:	CYSTATIN F
39:00:00	3esi-A	3	3.1	68	124	3	MOLECULE:	UNCHARACTERIZED PROTEIN
40:00:00	4h5b-A	3	2.4	67	152	3	MOLECULE:	DR_1245 PROTEIN
41:00:00	1k1x-A	3	5.1	72	636	7	MOLECULE:	4-ALPHA-GLUCANOTRANSFERASE
42:00:00	7bvj-A	3	7.1	68	313	4	MOLECULE:	OXIDOREDUCTASE, NAD-BINDING
43:00:00	7c5x-A	3	6.2	70	506	6	MOLECULE:	IOTA-CARBONIC ANHYDRASE
44:00:00	5wi2-B	3	3.6	54	93	9	MOLECULE:	CDNA FLJ56409, HIGHLY SIMILAR TO SE
45:00:00	8t0b-A	3	5.7	61	121	7	MOLECULE:	DUF1842 DOMAIN-CONTAINING PROTEIN
46:00:00	3put-B	3	3.2	71	155	4	MOLECULE:	HYPOTHETICAL CONSERVED PROTEIN
47:00:00	2lyx-A	3	4	60	87	15	MOLECULE:	UNCHARACTERIZED PROTEIN YQZG
48:00:00	8q72-A	3	13	71	750	6	MOLECULE:	JETC
49:00:00	4d0v-D	3	6	67	116	1	MOLECULE:	FIBER PROTEIN
50:00:00	8p0w-H	3	3.3	47	183	2	MOLECULE:	COMM DOMAIN-CONTAINING PROTEIN 1

51:00:00	1dwn-A	3	4.5	64	127	6	MOLECULE:	PHAGE COAT PROTEIN
52:00:00	1mm4-A	3	5.5	82	170	4	MOLECULE:	CRCA PROTEIN
53:00:00	3b8l-A	3	5.7	77	147	9	MOLECULE:	UNCHARACTERIZED PROTEIN
54:00:00	6dn4-A	3	7.4	62	275	6	MOLECULE:	BETA-LACTAMASE
55:00:00	3df6-B	3	3.1	51	99	2	MOLECULE:	ORF99
56:00:00	7eep-A	3	4.5	87	529	7	MOLECULE:	PAM1 PORTAL PROTEINS
57:00:00	3but-A	3	3.5	69	125	6	MOLECULE:	UNCHARACTERIZED PROTEIN AF_0446
58:00:00	6oh1-A	3	4.9	54	84	9	MOLECULE:	IMMUNOGLOBULIN A1 PROTEASE
59:00:00	6exp-F	3	2.9	53	103	9	MOLECULE:	SIRV3 ACRID1 (GP02) ANTI-CRISPR PRO
60:00:00	3mmy-C	3	4.2	55	355	7	MOLECULE:	MRNA EXPORT FACTOR
61:00:00	8p26-J	3	5.4	56	147	0	MOLECULE:	U2 SMALL NUCLEAR RIBONUCLEOPROTEIN
62:00:00	6u3r-A	3	3.4	51	190	2	MOLECULE:	DNAJ HOMOLOG SUBFAMILY B MEMBER 6,D
63:00:00	8gex-A	3	3.8	62	411	8	MOLECULE:	DUF4374 DOMAIN-CONTAINING PROTEIN
64:00:00	4agi-A	3	4	68	314	4	MOLECULE:	FUCOSE-SPECIFIC LECTIN FLEA
65:00:00	6yle-B	3	7.5	71	415	8	MOLECULE:	PRE-RRNA-PROCESSING PROTEIN IPI3
66:00:00	4j5t-A	3	12.6	58	788	9	MOLECULE:	MANNOSYL-OLIGOSACCHARIDE GLUCOSIDAS
67:00:00	5jtl-A	3	6	58	155	12	MOLECULE:	PROTEIN-EXPORT PROTEIN SECB
68:00:00	5cd6-A	3	4.6	80	575	6	MOLECULE:	TPR-DOMAIN CONTAINING PROTEIN
69:00:00	8jto-A	3	8.6	76	343	4	MOLECULE:	OUTER MEMBRANE PORIN (FRAGMENT)
70:00:00	3fz2-E	3	3.1	63	133	8	MOLECULE:	MINOR TAIL PROTEIN U
71:00:00	5y58-A	3	6.2	73	548	5	MOLECULE:	ATP-DEPENDENT DNA HELICASE II SUBUN
72:00:00	7uea-U	3	12.4	78	364	6	MOLECULE:	PHOTOSYSTEM P840 REACTION CENTER, L
73:00:00	8fn4-2	3	5.6	58	357	9	MOLECULE:	RNA-EDITING SUBSTRATE-BINDING COMPL
74:00:00	8bbg-B	3	5.9	68	1426	1	MOLECULE:	WD REPEAT-CONTAINING PROTEIN 19
75:00:00	6e8e-A	3	7.4	64	468	3	MOLECULE:	BETA SLIDING CLAMP,DNA MISMATCH REP
76:00:00	1sli-A	3	5.5	75	679	7	MOLECULE:	INTRAMOLECULAR TRANS-SIALIDASE
77:00:00	1xsi-A	3	4.2	53	773	8	MOLECULE:	PUTATIVE FAMILY 31 GLUCOSIDASE YICI
78:00:00	1mdc-A	3	3.1	53	131	6	MOLECULE:	INSECT FATTY ACID BINDING PROTEIN
79:00:00	8r6y-A	3	12.3	63	1991	14	MOLECULE:	RNA-DIRECTED RNA POLYMERASE L
80:00:00	4u8u-M	3	3.7	68	222	4	MOLECULE:	GLOBIN A CHAIN
81:00:00	8evk-A	3	4.9	63	115	8	MOLECULE:	DIHYDRONEOPTERIN ALDOLASE
82:00:00	7vwk-A	3	3.6	70	260	7	MOLECULE:	POLYKETIDE SYNTHASE
83:00:00	4qvh-A	3	7.8	57	596	2	MOLECULE:	MALTOSE-BINDING PERIPLASMIC PROTEIN
84:00:00	7elb-A	3	6.7	73	1937	0	MOLECULE:	RNA-DIRECTED RNA POLYMERASE L
85:00:00	7vd7-A	3	3.5	48	94	8	MOLECULE:	TOXIN
86:00:00	8ye0-C	3	5.1	73	290	8	MOLECULE:	LYNF/TRUF/PATF FAMILY PEPTIDE O-PRE
87:00:00	4xrw-A	3	11.4	64	305	6	MOLECULE:	BEXL
88:00:00	6nd4-T	3	4.8	69	812	7	MOLECULE:	ETS RRNA
89:00:00	4g75-A	3	4.5	66	292	3	MOLECULE:	PHOSPHODIESTERASE
90:00:00	7qwt-A	3	3.2	80	352	6	MOLECULE:	RIESKE (2FE-2S) DOMAIN PROTEIN
91:00:00	2g8s-A	3	4.3	70	347	9	MOLECULE:	GLUCOSE/SORBOSONE DEHYDROGENASES
92:00:00	4neh-A	3	7.4	78	1083	6	MOLECULE:	INTEGRIN ALPHA-X
93:00:00	6yai-E	3	4.4	64	726	6	MOLECULE:	CLATHRIN HEAVY CHAIN
94:00:00	6yfi-A	3	4.6	66	151	3	MOLECULE:	COAT PROTEIN
95:00:00	4gq2-P	3	3.9	64	371	6	MOLECULE:	NUCLEOPORIN NUP120
96:00:00	8pz4-A	3	9.6	81	466	4	MOLECULE:	ALGINATE PRODUCTION PROTEIN ALGE

97:00:00	3av0-B	3	12	64	365	5	MOLECULE:	DNA DOUBLE-STRAND BREAK REPAIR PROT
98:00:00	1dp4-C	3	5.6	63	429	6	MOLECULE:	ATRIAL NATRIURETIC PEPTIDE RECEPTOR
99:00:00	8uzl-A	3	4.3	68	143	6	MOLECULE:	DESIGNED TRANSMEMBRANE BETA-BARREL
0:00	6gxs-B	3	3.6	63	363	6	MOLECULE:	CV39L LECTIN
1:00	6h7f-A	3	5.5	76	674	9	MOLECULE:	BAUA
2:00	4n4r-B	3	3.2	70	151	9	MOLECULE:	LPS-ASSEMBLY PROTEIN LPTD
3:00	6isk-A	3	4.4	67	120	12	MOLECULE:	XIME, SNOAL-LIKE DOMAIN PROTEIN
4:00	7q3d-A	3	6.9	68	545	6	MOLECULE:	WD REPEAT-CONTAINING AND PLANAR CEL
5:00	7cd8-B	3	11.3	60	228	5	MOLECULE:	GREEN FLUORESCENT PROTEIN
6:00	4zf4-B	3	4.7	71	223	10	MOLECULE:	GREEN FLUORESCENT PROTEIN
7:00	2rh7-A	3	8.3	52	211	4	MOLECULE:	GREEN FLUORESCENT PROTEIN
8:00	5o67-B	3	3.9	66	290	2	MOLECULE:	FAPF
9:00	6ofm-A	3	8.7	56	229	7	MOLECULE:	GREEN FLUORESCENT PROTEIN (GFP) S6
10:00	2g16-A	3	6.5	49	62	10	MOLECULE:	GREEN FLUORESCENT PROTEIN
11:00	3evr-A	3	8.4	81	397	5	MOLECULE:	MYOSIN LIGHT CHAIN KINASE, GREEN FL
12:00	4pa0-A	3	8.3	54	974	6	MOLECULE:	MYOSIN-7,GREEN FLUORESCENT PROTEIN
13:00	5d74-B	2.9	4.2	57	234	7	MOLECULE:	PUTATIVE PHAGE LYSIN
14:00	5jk2-A	2.9	3.4	61	128	3	MOLECULE:	TP0751
15:00	8f2r-G	2.9	3.6	50	200	12	MOLECULE:	COMM DOMAIN-CONTAINING PROTEIN 1
16:00	4mtu-A	2.9	4.7	74	143	5	MOLECULE:	BETA-ALANYL-COA:AMMONIA LYASE 2
17:00	6c9j-A	2.9	5.3	63	386	2	MOLECULE:	5'-AMP-ACTIVATED PROTEIN KINASE CAT
18:00	8ptx-C	2.9	4.9	67	539	4	MOLECULE:	ELONGATOR COMPLEX PROTEIN 1
19:00	4y4v-A	2.9	6.4	72	318	8	MOLECULE:	CONSERVED HYPOTHETICAL SECRETED PRO
20:00	2kcd-A	2.9	3.7	62	120	0	MOLECULE:	UNCHARACTERIZED PROTEIN SSP0047
21:00	3zpm-A	2.9	6	92	211	13	MOLECULE:	LATHERIN
22:00	3h6j-A	2.9	7.2	68	438	7	MOLECULE:	NEURAMINIDASE
23:00	4nlm-A	2.9	5.7	58	326	2	MOLECULE:	LMO1340 PROTEIN
24:00:00	7k10-A	2.9	7.8	69	1259	3	MOLECULE:	DNA-DEPENDENT PROTEIN KINASE CATALY
25:00:00	8pbz-A	2.9	4.7	80	1003	6	MOLECULE:	MGP-OPERON PROTEIN 3
26:00:00	4j0x-B	2.9	4.7	53	366	11	MOLECULE:	RIBOSOMAL RNA-PROCESSING PROTEIN 9
27:00:00	3j9e-D	2.9	8.3	74	520	7	MOLECULE:	VP5
28:00:00	8cvo-E	2.9	2.2	46	64	7	MOLECULE:	16S RIBOSOMAL RNA
29:00:00	6rwb-A	2.9	6.2	63	1873	5	MOLECULE:	TOXIN,TOXIN COMPLEX SUBUNIT TCAB,PU
30:00:00	7qh3-B	2.9	7.6	58	148	5	MOLECULE:	RSFG
31:00:00	4v12-A	2.9	4.9	70	337	3	MOLECULE:	MAOC LIKE DOMAIN PROTEIN
32:00:00	8deo-A	2.9	5.1	57	454	9	MOLECULE:	ACCUMULATION ASSOCIATED PROTEIN
33:00:00	5c9p-A	2.9	7.4	71	354	3	MOLECULE:	PLL LECTIN
34:00:00	5mmj-y	2.9	3.8	60	116	12	MOLECULE:	50S RIBOSOMAL PROTEIN L31
35:00:00	8hpo-K	2.9	8.4	62	413	2	MOLECULE:	TRANSCRIPTIONAL REGULATORY PROTEIN
36:00:00	8oml-A	2.9	7.9	68	325	6	MOLECULE:	CELL WALL SURFACE ANCHOR FAMILY PRO
37:00:00	6qzh-A	2.9	4.4	69	744	7	MOLECULE:	HUMAN CHEMOKINE RECEPTOR 7
38:00:00	4gcz-B	2.9	7.1	59	378	8	MOLECULE:	BLUE-LIGHT PHOTORECEPTOR, SENSOR PR
39:00:00	1tp6-A	2.9	5.3	69	126	3	MOLECULE:	HYPOTHETICAL PROTEIN PA1314
40:00:00	8ovo-A	2.9	4.1	63	504	10	MOLECULE:	PUTATIVE PERIPLASMIC BINDING TRANSP
41:00:00	4lgq-A	2.9	5.2	64	134	11	MOLECULE:	PUTATIVE POLYKETIDE CYCLASE
42:00:00	3ua3-A	2.9	5.7	86	663	2	MOLECULE:	PROTEIN ARGININE N-METHYLTRANSFERAS

43:00:00	8f2r-C	2.9	3.6	48	195	4	MOLECULE:	COMM DOMAIN-CONTAINING PROTEIN 1
44:00:00	5iw9-A	2.9	3.4	50	126	8	MOLECULE:	BASEPLATE WEDGE PROTEIN GP25
45:00:00	1s28-A	2.9	2.2	43	130	9	MOLECULE:	ORF1
46:00:00	6p59-B	2.9	7.8	62	196	5	MOLECULE:	CORE-BINDING FACTOR SUBUNIT BETA
47:00:00	8cb6-F	2.9	4.8	57	110	4	MOLECULE:	LYSOSOMAL ALPHA-GLUCOSIDASE (76 KDA
48:00:00	1l0q-A	2.9	7.1	65	391	9	MOLECULE:	SURFACE LAYER PROTEIN
49:00:00	6pnq-B	2.9	5.2	64	133	2	MOLECULE:	NEUREXIN-1
50:00:00	5yk6-A	2.9	5.2	87	239	10	MOLECULE:	MAINTENANCE OF MITOCHONDRIAL MORPHO
51:00:00	6x1i-B	2.9	6.4	65	146	0	MOLECULE:	COB_ADENO_TRANS DOMAIN-CONTAINING P
52:00:00	7r2x-A	2.9	5.7	72	762	8	MOLECULE:	MANNURONIC ACID SPECIFIC LYASE
53:00:00	2k54-A	2.9	3.6	67	123	6	MOLECULE:	PROTEIN ATU0742
54:00:00	7uhy-C	2.9	5.3	69	578	1	MOLECULE:	GATOR COMPLEX PROTEIN MIOS
55:00:00	8f5o-E	2.9	6.9	66	1076	15	MOLECULE:	INTRAFLAGELLAR TRANSPORT PROTEIN 12
56:00:00	6u5j-A	2.9	5.1	72	164	8	MOLECULE:	COLLAR PA0615
57:00:00	5bp3-A	2.9	5.7	74	286	8	MOLECULE:	MYCOCEROSIC ACID SYNTHASE-LIKE POLY
58:00:00	6icl-A	2.9	3.7	62	338	3	MOLECULE:	METHYLXANTHINE N3-DEMETHYLASE NDMB
59:00:00	2ns9-A	2.9	5.3	72	152	7	MOLECULE:	HYPOTHETICAL PROTEIN APE2225
60:00:00	6sw9-F	2.9	2	47	229	0	MOLECULE:	16S RIBOSOMAL RNA
61:00:00	7vcf-B	2.9	8.5	94	651	10	MOLECULE:	TIC214
62:00:00	8d8k-E	2.9	2.9	50	291	8	MOLECULE:	PROBABLE S-ADENOSYL-L-METHIONINE-DE
63:00:00	6kyb-B	2.9	3.9	72	330	6	MOLECULE:	AUTOPHAGY-RELATED PROTEIN 18
64:00:00	6b7l-A	2.9	3.8	72	290	1	MOLECULE:	IMMUNE MODULATOR A
65:00:00	3c8w-A	2.9	4.8	69	250	6	MOLECULE:	ACETOACETATE DECARBOXYLASE ADC
66:00:00	7pge-B	2.9	8.6	77	198	3	MOLECULE:	COPPER RESISTANCE PROTEIN B
67:00:00	5nzr-K	2.9	7	70	559	4	MOLECULE:	COATOMER SUBUNIT ALPHA
68:00:00	7ktr-C	2.9	3.9	44	407	5	MOLECULE:	TRANSFORMATION/TRANSCRIPTION DOMAIN
69:00:00	3jro-A	2.9	4.6	71	701	0	MOLECULE:	FUSION PROTEIN OF PROTEIN TRANSPORT
70:00:00	6k7f-A	2.9	4.6	71	499	1	MOLECULE:	MALTOSE/MALTODEXTRIN-BINDING PERIPL
71:00:00	3eu7-A	2.9	7.2	63	313	5	MOLECULE:	PARTNER AND LOCALIZER OF BRCA2
72:00:00	6nau-A	2.9	3.7	59	334	2	MOLECULE:	6-PHOSPHOGLUCONOLACTONASE
73:00:00	7mo7-B	2.9	4.6	65	699	3	MOLECULE:	HEPATOCYTE GROWTH FACTOR
74:00:00	2zpa-B	2.9	7.8	62	662	3	MOLECULE:	UNCHARACTERIZED PROTEIN YPFI
75:00:00	8an0-A	2.9	5.7	53	282	6	MOLECULE:	PUTATIVE SECRETED PROTEIN
76:00:00	3g8z-A	2.9	4.5	62	129	6	MOLECULE:	PROTEIN OF UNKNOWN FUNCTION WITH CY
77:00:00	6rw8-B	2.9	10	69	2337	9	MOLECULE:	A COMPONENT OF INSECTICIDAL TOXIN C
78:00:00	2du7-A	2.9	7.5	70	539	11	MOLECULE:	O-PHOSPHOSERYL-TRNA SYNTHETASE
79:00:00	4zel-A	2.9	4.1	51	550	4	MOLECULE:	DOPAMINE BETA-HYDROXYLASE
80:00:00	4bg7-A	2.9	4	52	97	4	MOLECULE:	PUTATIVE TRANSCRIPTIONAL COACTIVATO
81:00:00	7jrd-A	2.9	4.6	66	566	2	MOLECULE:	LACTOFERRIN-BINDING PROTEIN B
82:00:00	2ia7-A	2.9	3.9	59	111	7	MOLECULE:	TAIL LYSOZYME, PUTATIVE
83:00:00	8rtd-S	2.9	5.7	63	223	10	MOLECULE:	TRWJ PROTEIN
84:00:00	4n6l-A	2.9	3.8	61	114	5	MOLECULE:	CYSTATIN-M
85:00:00	2xgl-A	2.9	3.6	55	90	5	MOLECULE:	COLICIN-M IMMUNITY PROTEIN
86:00:00	3lhn-A	2.9	4.2	53	108	8	MOLECULE:	LIPOPROTEIN
87:00:00	4qa8-A	2.9	4.3	59	210	3	MOLECULE:	PUTATIVE LIPOPROTEIN LPRF
88:00:00	3b33-A	2.9	4.8	49	109	6	MOLECULE:	SENSOR PROTEIN

89:00:00	1kmo-A	2.9	7.1	94	661	6	MOLECULE:	IRON(III) DICITRATE TRANSPORT PROTE
90:00:00	6l4i-B	2.9	3.3	58	90	0	MOLECULE:	SINGLE CHAIN MONELLIN
91:00:00	6l44-A	2.9	3.5	59	89	0	MOLECULE:	SINGLE CHAIN MONELLIN
92:00:00	6l44-B	2.9	3.5	59	90	2	MOLECULE:	SINGLE CHAIN MONELLIN
93:00:00	4mhc-A	2.9	6.9	68	671	10	MOLECULE:	NUCLEOPORIN NUP157
94:00:00	7xsi-A	2.9	5.3	63	130	6	MOLECULE:	SORDARIN/HYPOXYSORDARIN BIOSYNTHESI
95:00:00	3dxo-B	2.9	4.6	61	118	2	MOLECULE:	UNCHARACTERIZED SNOAL-LIKE PROTEIN
96:00:00	3ksp-A	2.9	7.9	70	129	7	MOLECULE:	CALCIUM/CALMODULIN-DEPENDENT KINASE
97:00:00	5zc1-E	2.9	3.6	57	98	14	MOLECULE:	CYSTATIN-1
98:00:00	1sq9-A	2.9	4.5	71	378	3	MOLECULE:	ANTIVIRAL PROTEIN SKI8
99:00:00	4ouq-A	2.9	3.4	53	105	8	MOLECULE:	UNCHARACTERIZED PROTEIN
0:00	6mwq-G	2.9	6.3	57	226	4	MOLECULE:	DARPIN, MUSCLE-TYPE ALDOLASE CHIMER
1:00	3o78-B	2.9	6	71	400	11	MOLECULE:	MYOSIN LIGHT CHAIN KINASE, SMOOTH M
2:00	8ill-B	2.9	8.6	54	212	2	MOLECULE:	GREEN FLUORESCENT PROTEIN
3:00	7a7v-A	2.9	8.3	55	225	5	MOLECULE:	GREEN FLUORESCENT PROTEIN
4:00	4w6i-A	2.9	7.8	52	219	13	MOLECULE:	FLUORESCENT PROTEIN D190C
5:00	4w7d-B	2.9	8.5	55	223	5	MOLECULE:	FLUORESCENT PROTEIN D21H/K26H
6:00	5o68-F	2.9	5.4	93	254	9	MOLECULE:	FAPF
7:00	6uhl-A	2.9	8.3	55	224	4	MOLECULE:	C148 MGFP-SCDNA-1
8:00	8b6t-B	2.9	8.3	55	516	4	MOLECULE:	GREEN FLUORESCENT PROTEIN,HALOALKAN
9:00	7a86-A	2.9	8.9	58	224	5	MOLECULE:	GREEN FLUORESCENT PROTEIN
10:00	2g5z-A	2.9	6.5	49	62	10	MOLECULE:	GREEN FLUORESCENT PROTEIN
11:00	6hzn-A	2.8	4	57	742	7	MOLECULE:	DERMATAN-SULFATE EPIMERASE
12:00	2erv-A	2.8	6.1	80	150	9	MOLECULE:	HYPOTHETICAL PROTEIN PAER03002360
13:00	5a5y-A	2.8	6.9	64	211	2	MOLECULE:	TRIPHOSPHATE TUNEL METALLOENZYME 3
14:00	2e1q-A	2.8	7.5	82	1307	4	MOLECULE:	XANTHINE DEHYDROGENASE/OXIDASE
15:00	3vb8-A	2.8	3.9	68	153	6	MOLECULE:	ENGINEERED PROTEIN
16:00	7wkk-D	2.8	3.7	60	1115	7	MOLECULE:	MGC83295 PROTEIN
17:00	7yqh-B	2.8	11.6	72	1066	6	MOLECULE:	STRUCTURAL MAINTENANCE OF CHROMOSOM
18:00	7x80-A	2.8	6.3	68	148	1	MOLECULE:	PLOI4
19:00	6n8s-A	2.8	6.2	77	834	9	MOLECULE:	LETHAL(2) GIANT LARVAE PROTEIN HOMO
20:00	8abv-A	2.8	5.1	78	244	3	MOLECULE:	SNOAL-LIKE DOMAIN-CONTAINING PROTEI
21:00	6al2-A	2.8	4.9	85	484	2	MOLECULE:	MEMBRANE PROTEIN INSERTASE YIDC
22:00	3g0k-A	2.8	4.7	65	129	3	MOLECULE:	PUTATIVE MEMBRANE PROTEIN
23:00	5njb-B	2.8	9.9	85	445	2	MOLECULE:	METALLOPROTEASE TLDD
24:00:00	6nu9-A	2.8	3.9	50	168	10	MOLECULE:	ZINC-BINDING NON-STRUCTURAL PROTEIN
25:00:00	5nfi-B	2.8	4.3	68	277	7	MOLECULE:	MINOR FIMBRIUM ANCHORING SUBUNIT MF
26:00:00	1bp1-A	2.8	5.6	81	456	4	MOLECULE:	BACTERICIDAL/PERMEABILITY-INCREASIN
27:00:00	3sbu-A	2.8	4.9	63	230	6	MOLECULE:	HYPOTHETICAL NTF2-LIKE PROTEIN
28:00:00	8sv7-A	2.8	5	66	272	2	MOLECULE:	TRANSPORT AND GOLGI ORGANIZATION PR
29:00:00	7nyx-A	2.8	4.8	63	1467	13	MOLECULE:	CHROMOSOME PARTITION PROTEIN MUKB
30:00:00	6p74-A	2.8	11.2	59	512	8	MOLECULE:	PUTATIVE ATP-DEPENDENT ENDONUCLEASE
31:00:00	6tlb-A	2.8	3.3	72	185	8	MOLECULE:	SERINE/THREONINE PROTEIN KINASE
32:00:00	5n6u-A	2.8	4.5	51	811	6	MOLECULE:	BETA-MANNOSIDASE
33:00:00	4pux-A	2.8	5.8	79	157	11	MOLECULE:	UNCHARACTERIZED PROTEIN
34:00:00	5tod-D	2.8	8.1	70	157	4	MOLECULE:	TRANSMEMBRANE PROTEIN 24

35:00:00	8cli-C	2.8	8.1	82	579	2	MOLECULE:	GENERAL TRANSCRIPTION FACTOR 3C POL
36:00:00	8oxh-A	2.8	2.7	51	93	6	MOLECULE:	AVRA6
37:00:00	8u7i-A	2.8	11.2	73	626	4	MOLECULE:	ENDONUCLEASE GAJA
38:00:00	7c8z-B	2.8	6	71	161	4	MOLECULE:	SALICYLATE 5-HYDROXYLASE, LARGE OXY
39:00:00	8shj-A	2.8	5	65	334	3	MOLECULE:	WD REPEAT-CONTAINING PROTEIN 91
40:00:00	5wzr-A	2.8	8.4	69	630	3	MOLECULE:	ALPHA-N-ACETYLGALACTOSAMINIDASE
41:00:00	3e1e-C	2.8	3.4	67	141	6	MOLECULE:	THIOESTERASE FAMILY PROTEIN
42:00:00	8ptx-A	2.8	4.1	70	1215	9	MOLECULE:	ELONGATOR COMPLEX PROTEIN 1
43:00:00	8iha-A	2.8	4.6	56	129	4	MOLECULE:	POLYKETIDE CYCLASE / DEHYDRASE AND
44:00:00	2cg8-C	2.8	8	70	248	3	MOLECULE:	DIHYDRONEOPTERIN ALDOLASE 6-HYDROXY
45:00:00	4ue0-A	2.8	4.1	54	115	6	MOLECULE:	FIBER
46:00:00	3wy4-B	2.8	7.4	53	421	4	MOLECULE:	ALPHA-GLUCOSIDASE
47:00:00	1jkg-A	2.8	5.5	67	139	16	MOLECULE:	P15
48:00:00	1he8-A	2.8	6.3	66	749	5	MOLECULE:	PHOSPHATIDYLINOSITOL 3-KINASE CATAL
49:00:00	5hd6-A	2.8	6.4	81	175	5	MOLECULE:	3-HYDROXYDECANOYL-[ACYL-CARRIER-PRO
50:00:00	3msw-A	2.8	6.9	71	139	3	MOLECULE:	UNCHARACTERIZED PROTEIN
51:00:00	2qiy-A	2.8	4.3	59	134	8	MOLECULE:	UBP3-ASSOCIATED PROTEIN BRE5
52:00:00	6w6m-A	2.8	5.5	64	412	5	MOLECULE:	TYPE IV PILUS SECRETIN PILQ FAMILY
53:00:00	7l5m-A	2.8	4	62	87	6	MOLECULE:	LIPOCALIN FAMILY PROTEIN
54:00:00	8vwp-A	2.8	7.8	67	502	9	MOLECULE:	LANGYA VIRUS ATTACHMENT (G) PROTEIN
55:00:00	8wxf-A	2.8	10.7	63	730	3	MOLECULE:	BIFUNCTIONAL GUANOSINE PENTAPHOSPHA
56:00:00	4rki-A	2.8	6.9	53	374	4	MOLECULE:	DNA POLYMERASE III SUBUNIT BETA
57:00:00	6nd4-L	2.8	5.3	58	473	3	MOLECULE:	ETS RRNA
58:00:00	7xxa-E	2.8	2.5	48	144	4	MOLECULE:	VP1
59:00:00	3q6k-B	2.8	5.8	68	381	10	MOLECULE:	43.2 KDA SALIVARY PROTEIN
60:00:00	7zty-A	2.8	7.8	58	345	5	MOLECULE:	CNH DOMAIN-CONTAINING PROTEIN
61:00:00	6s9u-A	2.8	3.6	64	520	5	MOLECULE:	PUTATIVE SUCROSE PHOSPHORYLASE
62:00:00	2ooj-B	2.8	6.1	69	134	4	MOLECULE:	HYPOTHETICAL PROTEIN
63:00:00	6nd4-W	2.8	4.6	61	385	2	MOLECULE:	ETS RRNA
64:00:00	6qhg-A	2.8	5.6	52	119	10	MOLECULE:	POLYMERASE
65:00:00	5mnt-B	2.8	6.1	74	421	4	MOLECULE:	A2 MATURATION PROTEIN
66:00:00	6xt9-I	2.8	8.3	62	767	5	MOLECULE:	BARDET-BIEDL SYNDROME 1 PROTEIN
67:00:00	4r5o-A	2.8	3.6	59	426	5	MOLECULE:	QUINONPROTEIN ALCOHOL DEHYDROGENASE
68:00:00	8r8q-B	2.8	9.3	54	341	6	MOLECULE:	MAJOR FACILITATOR SUPERFAMILY DOMAI
69:00:00	8dit-B	2.8	8	62	834	3	MOLECULE:	VACUOLAR PROTEIN SORTING-ASSOCIATED
70:00:00	5iwd-A	2.8	2.9	53	261	9	MOLECULE:	DNA POLYMERASE PROCESSIVITY FACTOR
71:00:00	6ozd-B	2.8	3.4	61	523	10	MOLECULE:	PUTATIVE EXPORTED PROTEIN
72:00:00	6eys-B	2.8	4.4	71	478	6	MOLECULE:	PVDP
73:00:00	6mly-C	2.8	3.6	73	774	7	MOLECULE:	BIFUNCTIONAL GH43-CE PROTEIN
74:00:00	3hrp-A	2.8	4.2	61	400	7	MOLECULE:	UNCHARACTERIZED PROTEIN
75:00:00	4gwp-B	2.8	3.6	73	492	5	MOLECULE:	MEDIATOR OF RNA POLYMERASE II TRANS
76:00:00	4exr-A	2.8	3	52	152	0	MOLECULE:	PUTATIVE LIPOPROTEIN
77:00:00	7sfn-B	2.8	3	68	153	4	MOLECULE:	OLMO - OLIGOMYCIN SPIROCYCLASE
78:00:00	8es5-A	2.8	4.2	60	146	3	MOLECULE:	ACTIVATOR OF HSP90 ATPASE
79:00:00	7wmp-a	2.8	4.7	99	559	5	MOLECULE:	PORTAL PROTEIN
80:00:00	6jq9-B	2.8	3.9	79	483	5	MOLECULE:	SHORT ULVAN LYASE

81:00:00	5n9j-A	2.8	3.1	58	566	5	MOLECULE:	MEDIATOR OF RNA POLYMERASE II TRANS
82:00:00	8chw-A	2.8	2.9	50	67	6	MOLECULE:	TRANSCRIPTIONAL ACTIVATOR PROTEIN P
83:00:00	5f75-C	2.8	3.9	67	475	4	MOLECULE:	THIOCYANATE DEHYDROGENASE
84:00:00	3was-A	2.8	4.5	69	389	9	MOLECULE:	4-O-BETA-D-MANNOSYL-D-GLUCOSE PHOSP
85:00:00	3s5m-A	2.8	8.8	58	1094	5	MOLECULE:	FALCILYSIN
86:00:00	5gqt-A	2.8	8.8	63	470	3	MOLECULE:	NITRILE-SPECIFIER PROTEIN 1
87:00:00	6rfy-D	2.8	12	65	407	6	MOLECULE:	EIS2
88:00:00	3w7s-A	2.8	8.9	71	760	6	MOLECULE:	UNCHARACTERIZED PROTEIN YGJK
89:00:00	6vbv-7	2.8	4.4	74	706	5	MOLECULE:	BARDET-BIEDL SYNDROME 18 PROTEIN
90:00:00	7tom-A	2.8	5.7	53	499	0	MOLECULE:	GLYCEROL DIBIPHYTANYL GLYCEROL TETR
91:00:00	7bgh-A	2.8	9.7	80	177	3	MOLECULE:	OUTER ENVELOPE PORE PROTEIN 21, CHL
92:00:00	3s9j-A	2.8	8.6	57	362	7	MOLECULE:	MEMBER OF DUF4221 FAMILY
93:00:00	4ew7-A	2.8	4.8	52	113	2	MOLECULE:	CONJUGATIVE TRANSFER: REGULATION
94:00:00	8h8b-A	2.8	12.5	68	1027	6	MOLECULE:	PUTATIVE RHS-FAMILY PROTEIN
95:00:00	2p4f-A	2.8	3.8	62	191	8	MOLECULE:	SIMILAR TO SP|P32453 SACCHAROMYCES
96:00:00	8y6p-Q	2.8	7.7	65	1228	2	MOLECULE:	APAF-1 RELATED KILLER DARK
97:00:00	4xi5-A	2.8	9.8	67	728	3	MOLECULE:	ENVELOPE GLYCOPROTEIN H
98:00:00	3nqh-A	2.8	3.5	54	439	6	MOLECULE:	GLYCOSYL HYDROLASE
99:00:00	4bp8-A	2.8	5.6	62	712	10	MOLECULE:	OLIGOPEPTIDASE B
0:00	1xks-A	2.8	4.6	73	374	7	MOLECULE:	NUCLEAR PORE COMPLEX PROTEIN NUP133
1:00	5bka-A	2.8	5.3	67	115	12	MOLECULE:	HYDROXYLACYL-COA DEHYDROGENASE
2:00	3eby-A	2.8	5.8	68	153	7	MOLECULE:	BETA SUBUNIT OF A PUTATIVE AROMATIC
3:00	5tph-A	2.8	5.7	61	120	10	MOLECULE:	DE NOVO NTF2 HOMODIMER
4:00	7pkq-L	2.8	4.5	72	175	7	MOLECULE:	MS35
5:00	5i97-A	2.8	5.9	64	143	8	MOLECULE:	CONJUGAL TRANSFER PROTEIN
6:00	8h2t-H	2.8	5.5	71	157	7	MOLECULE:	RIESKE (2FE-2S) DOMAIN PROTEIN
7:00	6zgn-A	2.8	5	58	101	3	MOLECULE:	PUTATIVE TRANSFER PROTEIN
8:00	8f5p-E	2.8	5.8	60	1057	3	MOLECULE:	NET DOMAIN-CONTAINING PROTEIN
9:00	5o65-C	2.8	7.3	94	278	4	MOLECULE:	FAPF
10:00	5o68-G	2.8	5.8	86	271	6	MOLECULE:	FAPF
11:00	5d17-G	2.8	3.3	61	158	5	MOLECULE:	TRANSPOSON TN7 TRANSPOSITION PROTEI
12:00	5wj3-B	2.8	8.4	55	225	5	MOLECULE:	GREEN FLUORESCENT PROTEIN
13:00	1rrx-A	2.8	8.9	58	225	5	MOLECULE:	SIGF1-GFP FUSION PROTEIN
14:00	6xu4-A	2.8	6.2	82	389	2	MOLECULE:	FGCAMP
15:00	3osr-A	2.8	6.3	82	603	2	MOLECULE:	MALTOSE-BINDING PERIPLASMIC PROTEIN
16:00	4w6r-I	2.8	9	52	209	10	MOLECULE:	FLUORESCENT PROTEIN D102C
17:00	4w75-B	2.8	8	50	187	8	MOLECULE:	FLUORESCENT PROTEIN D21H/K26C
18:00	2gu3-A	2.7	3.3	49	128	10	MOLECULE:	YPMB PROTEIN
19:00	2gey-A	2.7	5.9	71	157	7	MOLECULE:	ACLR PROTEIN
20:00	3cyg-A	2.7	5.9	66	215	3	MOLECULE:	UNCHARACTERIZED PROTEIN
21:00	1lqm-H	2.7	3.5	51	84	12	MOLECULE:	URACIL-DNA GLYCOSYLASE
22:00	2ffs-A	2.7	2.9	63	146	3	MOLECULE:	HYPOTHETICAL PROTEIN PA1206
23:00	1u5o-A	2.7	5.9	62	125	10	MOLECULE:	NUCLEAR TRANSPORT FACTOR 2
24:00:00	7eu3-7	2.7	3.9	69	317	6	MOLECULE:	NAD(P)H-QUINONE OXIDOREDUCTASE SUBU
25:00:00	4ffg-A	2.7	4.4	68	480	7	MOLECULE:	LEVAN FRUCTOTRANSFERASE
26:00:00	8hqo-a	2.7	3.4	60	160	8	MOLECULE:	PORTAL PROTEIN

27:00:00	5mtw-A	2.7	4.2	67	138	6	MOLECULE:	SECB-LIKE CHAPERONE RV1957
28:00:00	1vl4-A	2.7	10.7	87	428	11	MOLECULE:	PMBA-RELATED PROTEIN
29:00:00	8dk3-A	2.7	9.3	64	455	3	MOLECULE:	JETC
30:00:00	7cpx-A	2.7	9.3	78	2262	5	MOLECULE:	LOVASTATIN NONAKETIDE SYNTHASE, POL
31:00:00	6r5w-A	2.7	6	66	171	3	MOLECULE:	GP15 PROTEIN
32:00:00	6stx-C	2.7	4.9	61	663	11	MOLECULE:	KELCH DOMAIN-CONTAINING PROTEIN
33:00:00	2ogf-B	2.7	4.4	60	122	2	MOLECULE:	HYPOTHETICAL PROTEIN MJ0408
34:00:00	6y6k-A	2.7	4	58	1299	10	MOLECULE:	RNA-DEPENDENT RNA POLYMERASE
35:00:00	3f7s-A	2.7	4.4	69	142	6	MOLECULE:	UNCHARACTERIZED NTF2-LIKE PROTEIN
36:00:00	1nbu-A	2.7	4.3	62	118	5	MOLECULE:	PROBABLE DIHYDRONEOPTERIN ALDOLASE
37:00:00	8tnn-A	2.7	11.2	64	658	5	MOLECULE:	ENVELOPE GLYCOPROTEIN H
38:00:00	3c1a-A	2.7	4.9	62	307	5	MOLECULE:	PUTATIVE OXIDOREDUCTASE
39:00:00	4ghb-B	2.7	5	63	257	3	MOLECULE:	HYPOTHETICAL PROTEIN
40:00:00	2nyk-A	2.7	11.5	67	237	9	MOLECULE:	M157
41:00:00	6mlt-A	2.7	7.1	60	603	3	MOLECULE:	HEMOLYSIN-RELATED PROTEIN
42:00:00	5hi8-A	2.7	3.8	53	136	4	MOLECULE:	ANTENNA PROTEIN
43:00:00	1oac-B	2.7	4.4	88	723	3	MOLECULE:	COPPER AMINE OXIDASE
44:00:00	4r03-A	2.7	3.9	65	109	5	MOLECULE:	UNCHARACTERIZED PROTEIN
45:00:00	8i0a-A	2.7	6.9	67	524	6	MOLECULE:	ALPHA-L-ARABINOFURANOSIDASE
46:00:00	4p5i-A	2.7	6.5	69	240	12	MOLECULE:	CHEMOKINE BINDING PROTEIN
47:00:00	8bsh-C	2.7	5.6	68	794	9	MOLECULE:	PROTEIN TRANSPORT PROTEIN SEC23
48:00:00	3dm8-A	2.7	6.6	66	135	6	MOLECULE:	UNCHARACTERIZED PROTEIN RPA4348
49:00:00	2vnv-B	2.7	3.1	60	128	3	MOLECULE:	BCLA
50:00:00	1b9l-A	2.7	5.1	63	119	2	MOLECULE:	PROTEIN (EPIMERASE)
51:00:00	7vma-A	2.7	3.5	67	620	4	MOLECULE:	AMYLO-ALPHA-1,6-GLUCOSIDASE, PUTATI
52:00:00	8hmc-B	2.7	3.5	61	1195	7	MOLECULE:	INTRAFLAGELLAR TRANSPORT PROTEIN 12
53:00:00	6s8f-F	2.7	6.1	62	2571	3	MOLECULE:	SERINE/THREONINE-PROTEIN KINASE TEL
54:00:00	5k8g-A	2.7	5.6	64	511	5	MOLECULE:	ANTIFREEZE PROTEIN
55:00:00	3er7-B	2.7	4.8	67	123	6	MOLECULE:	UNCHARACTERIZED NTF2-LIKE PROTEIN
56:00:00	6nnw-A	2.7	8.2	74	208	7	MOLECULE:	TETRONASIN
57:00:00	6v04-A	2.7	5	71	268	4	MOLECULE:	UNCHARACTERIZED SRPBCC DOMAIN-CONTA
58:00:00	3slj-A	2.7	10.2	98	305	7	MOLECULE:	SERINE PROTEASE ESPP
59:00:00	7fse-A	2.7	7.8	55	1040	15	MOLECULE:	REVERSE GYRASE
60:00:00	3alx-B	2.7	4.3	58	526	2	MOLECULE:	HEMAGGLUTININ,LINKER,CDW150
61:00:00	4l3a-A	2.7	3.4	56	469	4	MOLECULE:	INTERNALIN K
62:00:00	6sum-A	2.7	11.2	49	335	10	MOLECULE:	AMICOUMACIN KINASE
63:00:00	6kko-A	2.7	4.2	63	170	8	MOLECULE:	PUTATIVE SERINE PHOSPHATASE
64:00:00	5jzr-A	2.7	6.5	74	131	9	MOLECULE:	COAT PROTEIN
65:00:00	5hzv-A	2.7	5.1	66	598	8	MOLECULE:	MALTOSE-BINDING PERIPLASMIC PROTEIN
66:00:00	8aim-B	2.7	4.1	52	86	4	MOLECULE:	URACIL-DNA GLYCOSYLASE INHIBITOR
67:00:00	5d9g-A	2.7	4	80	246	1	MOLECULE:	TIP41-LIKE PROTEIN
68:00:00	2in5-A	2.7	4.8	69	195	10	MOLECULE:	HYPOTHETICAL LIPOPROTEIN YMCC
69:00:00	4v1a-i	2.7	3.6	70	242	6	MOLECULE:	MITORIBOSOMAL PROTEIN ML37, MRPL37
70:00:00	7p8s-A	2.7	3.6	71	293	11	MOLECULE:	LEUCOTOXIN LUKEV
71:00:00	7ohi-A	2.7	6.7	67	250	4	MOLECULE:	AP-2 COMPLEX SUBUNIT ALPHA-2
72:00:00	7okx-M	2.7	10.3	56	139	5	MOLECULE:	DNA-DIRECTED RNA POLYMERASE II SUBU

73:00:00	3n7n-B	2.7	5.8	58	164	3	MOLECULE:	MONOPOLIN COMPLEX SUBUNIT CSM1
74:00:00	7eni-C	2.7	3.3	59	126	7	MOLECULE:	CRISPR-ASSOCIATED ENDONUCLEASE CAS9
75:00:00	3klq-A	2.7	4.9	70	137	4	MOLECULE:	PUTATIVE PILUS ANCHORING PROTEIN
76:00:00	4u3v-A	2.7	6.1	67	242	3	MOLECULE:	POLYKETIDE SYNTHASE PKSR
77:00:00	7erl-A	2.7	5.2	69	545	6	MOLECULE:	BETA-XYLANASE
78:00:00	2hqs-A	2.7	3.6	55	412	13	MOLECULE:	PROTEIN TOLB
79:00:00	6h6g-A	2.7	5.1	61	2139	2	MOLECULE:	TCDB2,TCCC3
80:00:00	1qjs-A	2.7	5.8	60	408	5	MOLECULE:	HEMOPEXIN
81:00:00	8h36-A	2.7	7.2	69	567	6	MOLECULE:	E3 UBIQUITIN-PROTEIN LIGASE RBX1
82:00:00	5ts4-B	2.7	4.6	57	106	7	MOLECULE:	DENOVO NTF2
83:00:00	8a3t-C	2.7	5.9	65	1410	8	MOLECULE:	ANAPHASE-PROMOTING COMPLEX SUBUNIT
84:00:00	4mob-A	2.7	3	64	319	11	MOLECULE:	ACYL-COENZYME A THIOESTERASE 12
85:00:00	6wil-A	2.7	8.1	93	536	6	MOLECULE:	HEMOLYSIN ACTIVATOR PROTEIN CDIB
86:00:00	6goc-A	2.7	5.5	70	444	9	MOLECULE:	DUF3826 DOMAIN-CONTAINING PROTEIN
87:00:00	7to3-A	2.7	11.1	73	568	4	MOLECULE:	CAP2
88:00:00	7dna-A	2.7	8.4	55	213	5	MOLECULE:	GREEN-TO-RED PHOTOCONVERTIBLE GFP-L
89:00:00	7x2n-A	2.7	4.4	66	340	3	MOLECULE:	PYCR1
90:00:00	6vxk-B	2.7	5.2	69	747	7	MOLECULE:	SEMAPHORIN-LIKE PROTEIN 139
91:00:00	2z5b-A	2.7	4.4	41	127	10	MOLECULE:	PROTEIN YPL144W
92:00:00	1sa8-A	2.7	3.3	59	106	5	MOLECULE:	FATTY ACID-BINDING PROTEIN, INTESTI
93:00:00	6wxu-B	2.7	3.9	58	273	3	MOLECULE:	DUAL OXIDASE 1
94:00:00	6jcy-C	2.7	5.3	62	1139	6	MOLECULE:	DNA-DIRECTED RNA POLYMERASE SUBUNIT
95:00:00	3ats-A	2.7	3.8	57	352	18	MOLECULE:	PUTATIVE UNCHARACTERIZED PROTEIN
96:00:00	6vs7-A	2.7	3.2	54	409	9	MOLECULE:	ADHESIN
97:00:00	7eeb-E	2.7	4.6	64	1053	5	MOLECULE:	ENHANCED GREEN FLUORESCENT PROTEIN,
98:00:00	8axs-A	2.7	3.4	73	573	3	MOLECULE:	EXO-ALPHA-SIALIDASE
99:00:00	7lxf-A	2.7	3.1	56	134	9	MOLECULE:	PROTEIN ENABLED HOMOLOG,PHOTORECEPT
0:00	7mjp-A	2.7	4.7	59	366	5	MOLECULE:	ATP-SENSITIVE INWARD RECTIFIER POTA
1:00	8c7i-A	2.7	8.4	55	404	11	MOLECULE:	GREEN FLUORESCENT PROTEIN,TLL0404 P
2:00	3oan-A	2.7	3.3	51	116	0	MOLECULE:	ABR034WP
3:00	8h7g-C	2.7	5.3	68	3261	4	MOLECULE:	TRANSFORMATION/TRANSCRIPTION DOMAIN
4:00	2j04-A	2.7	6	70	587	10	MOLECULE:	HYPOTHETICAL PROTEIN YPL007C
5:00	3kkg-A	2.7	5	68	144	12	MOLECULE:	PUTATIVE SNOAL-LIKE POLYKETIDE CYCL
6:00	3s25-A	2.7	4.6	54	295	9	MOLECULE:	HYPOTHETICAL 7-BLADED BETA-PROPELLE
7:00	5a4o-A	2.7	3.3	47	74	2	MOLECULE:	BPSL1147
8:00	8env-a	2.7	3.1	49	102	2	MOLECULE:	SHEATH PROTEIN GP31
9:00	6dj8-A	2.7	11.8	62	384	8	MOLECULE:	BETA SLIDING CLAMP
10:00	8us3-A	2.7	5.9	88	552	10	MOLECULE:	TRANSLOCATION AND ASSEMBLY MODULE S
11:00	6p29-A	2.7	4	49	134	8	MOLECULE:	N-DEMETHYLINDOLMYCIN SYNTHASE (PLUN
12:00	3ehc-B	2.7	4.1	62	128	2	MOLECULE:	SNOAL-LIKE POLYKETIDE CYCLASE
13:00	4w4l-C	2.7	5	60	297	7	MOLECULE:	PE FAMILY PROTEIN PE25
14:00	3azo-A	2.7	6.1	83	661	6	MOLECULE:	AMINOPEPTIDASE
15:00	2yzy-A	2.7	5.2	55	163	2	MOLECULE:	PUTATIVE UNCHARACTERIZED PROTEIN TT
16:00	4ktp-A	2.7	8.2	68	767	7	MOLECULE:	GLYCOSIDE HYDROLASE FAMILY 65 CENTR
17:00	1k32-A	2.7	4.5	60	1023	3	MOLECULE:	TRICORN PROTEASE
18:00	3i2n-A	2.7	5.3	68	343	10	MOLECULE:	WD REPEAT-CONTAINING PROTEIN 92

19:00	5hx0-A	2.7	7.8	64	365	6	MOLECULE:	UNCHARACTERIZED PROTEIN DFER_1899
20:00	7emf-P	2.7	6	74	766	14	MOLECULE:	MEDIATOR OF RNA POLYMERASE II TRANS
21:00	7qrd-A	2.7	3.6	64	364	8	MOLECULE:	HISTONE-ARGININE METHYLTRANSFERASE
22:00	2ojh-A	2.7	5	54	277	7	MOLECULE:	UNCHARACTERIZED PROTEIN ATU1656/AGR
23:00	7v2w-H	2.7	6.9	71	379	4	MOLECULE:	THO COMPLEX SUBUNIT HPR1
24:00:00	7z4w-A	2.7	4.3	74	447	4	MOLECULE:	PORTAL PROTEIN
25:00:00	1yli-B	2.7	4.2	65	147	9	MOLECULE:	PUTATIVE ACYL-COA THIOESTER HYDROLA
26:00:00	4gxb-A	2.7	6	69	263	9	MOLECULE:	SORTING NEXIN-17
27:00:00	6icz-A	2.7	9.3	71	2253	8	MOLECULE:	PROTEIN MAGO NASHI HOMOLOG 2
28:00:00	4o3v-A	2.7	4.4	61	138	5	MOLECULE:	VIRB8-LIKE PROTEIN OF TYPE IV SECRE
29:00:00	6fej-B	2.7	4	60	118	5	MOLECULE:	ALL4940 PROTEIN
30:00:00	3d9r-A	2.7	4.8	66	132	14	MOLECULE:	KETOSTEROID ISOMERASE-LIKE PROTEIN
31:00:00	6isk-B	2.7	4	62	120	11	MOLECULE:	XIME, SNOAL-LIKE DOMAIN PROTEIN
32:00:00	5o7s-B	2.7	2.9	56	90	0	MOLECULE:	MONELLIN CHAIN B,MONELLIN CHAIN A
33:00:00	4q9t-A	2.7	4.9	62	384	6	MOLECULE:	NUCLEOPORIN NUP133
34:00:00	8abt-A	2.7	4.2	73	235	1	MOLECULE:	SNOAL-LIKE DOMAIN-CONTAINING PROTEI
35:00:00	5o68-E	2.7	6.3	88	273	7	MOLECULE:	FAPF
36:00:00	6og8-B	2.7	6.8	60	227	3	MOLECULE:	CRYSTAL STRUCTURE OF GREEN FLUORESC
37:00:00	6zsm-A	2.7	7	78	396	4	MOLECULE:	GREEN FLUORESCENT PROTEIN,GREEN FLU
38:00:00	3sg4-A	2.7	7.1	78	394	4	MOLECULE:	MYOSIN LIGHT CHAIN KINASE, GREEN FL
39:00:00	7lug-A	2.7	6	75	223	5	MOLECULE:	RED FLUORESCENT PNRFP B30Y MUTANT
40:00:00	5d17-D	2.7	3.3	61	157	5	MOLECULE:	TRANSPOSON TN7 TRANSPOSITION PROTEI
41:00:00	4w6k-B	2.7	3.4	51	182	4	MOLECULE:	FLUORESCENT PROTEIN D117C
42:00:00	6uhr-A	2.7	10.4	58	222	12	MOLECULE:	C148 MGFP-SCDNA-2
43:00:00	6hr1-B	2.7	8.3	55	401	4	MOLECULE:	MYOSIN LIGHT CHAIN KINASE 2, SKELET
44:00:00	7cd7-D	2.7	8.4	55	235	11	MOLECULE:	GREEN FLUORESCENT PROTEIN
45:00:00	4w6p-F	2.7	10.3	57	214	9	MOLECULE:	FLUORESCENT PROTEIN D102C
46:00:00	8yq4-A	2.7	8.6	54	213	2	MOLECULE:	MBAOJIN2 - BRIGHT AND STABLE GREEN
47:00:00	4jfg-F	2.7	10.2	55	217	13	MOLECULE:	GREEN FLUORESCENT PROTEIN
48:00:00	3ztf-A	2.7	8.4	55	236	11	MOLECULE:	GREEN FLUORESCENT PROTEIN
49:00:00	8uzh-B	2.6	3.3	52	626	4	MOLECULE:	SUMO FUSED TREHALOSE SYNTHASE (TRES
50:00:00	3hzp-A	2.6	5.2	67	128	7	MOLECULE:	NTF2-LIKE PROTEIN OF UNKNOWN FUNCTI
51:00:00	4eae-B	2.6	4.1	65	188	8	MOLECULE:	LMO1068 PROTEIN
52:00:00	7ty0-C	2.6	6.7	67	488	3	MOLECULE:	GLYCOPROTEIN G
53:00:00	6cvz-A	2.6	5.6	66	337	6	MOLECULE:	E3 UBIQUITIN-PROTEIN LIGASE RFWD3
54:00:00	4mp6-A	2.6	4.9	81	334	2	MOLECULE:	PUTATIVE ORNITHINE CYCLODEAMINASE
55:00:00	2chc-A	2.6	6.1	74	169	5	MOLECULE:	PROTEIN RV3472
56:00:00	8fcj-I	2.6	9.5	74	494	1	MOLECULE:	TYPE I-B CRISPR-ASSOCIATED PROTEIN
57:00:00	1z94-B	2.6	4.2	71	144	3	MOLECULE:	CONSERVED HYPOTHETICAL PROTEIN
58:00:00	2rfr-A	2.6	5.3	71	154	7	MOLECULE:	UNCHARACTERIZED PROTEIN
59:00:00	6dfl-A	2.6	10.2	58	241	3	MOLECULE:	LIPOPOLYSACCHARIDE CORE HEPTOSE(I)
60:00:00	5fw5-A	2.6	4.7	62	139	8	MOLECULE:	RAS GTPASE-ACTIVATING PROTEIN-BINDI
61:00:00	7ojn-L	2.6	7.3	93	2010	5	MOLECULE:	RNA-DIRECTED RNA POLYMERASE L
62:00:00	3lyd-B	2.6	3.2	59	160	3	MOLECULE:	UNCHARACTERIZED PROTEIN
63:00:00	6nob-A	2.6	6.5	76	634	3	MOLECULE:	BETA-FRUCTOFURANOSIDASE
64:00:00	3ef8-A	2.6	5.2	70	149	6	MOLECULE:	PUTATIVE SCYALONE DEHYDRATASE

65:00:00	1fw9-A	2.6	2.9	62	164	8	MOLECULE:	CHORISMATE LYASE
66:00:00	6mit-G	2.6	6.9	64	355	3	MOLECULE:	LIPOPOLYSACCHARIDE EXPORT SYSTEM AT
67:00:00	4zv3-B	2.6	3.5	63	300	3	MOLECULE:	CYTOSOLIC ACYL COENZYME A THIOESTER
68:00:00	3b18-A	2.6	4.4	72	167	7	MOLECULE:	UNCHARACTERIZED PROTEIN RV0098/MT01
69:00:00	6itd-A	2.6	5	65	328	5	MOLECULE:	SLR0355 PROTEIN
70:00:00	8jyx-A	2.6	6.2	90	565	6	MOLECULE:	MALTODEXTRIN-BINDING PROTEIN,GASDER
71:00:00	7nie-C	2.6	6.1	68	294	7	MOLECULE:	GLYCEROL KINASE
72:00:00	8u01-A	2.6	5.2	56	909	2	MOLECULE:	GLYCOSYL HYDROLASE FAMILY 2, SUGAR
73:00:00	8suc-A	2.6	5.1	62	282	10	MOLECULE:	NHL (RING FINGER B-BOX COILED COIL)
74:00:00	7alc-C	2.6	4.4	62	162	3	MOLECULE:	GTP CYCLOHYDROLASE 1
75:00:00	3db2-A	2.6	6.2	71	347	3	MOLECULE:	PUTATIVE NADPH-DEPENDENT OXIDOREDUC
76:00:00	4r7k-A	2.6	5.8	74	168	4	MOLECULE:	HYPOTHETICAL PROTEIN JHP0584
77:00:00	6xux-A	2.6	9	72	879	6	MOLECULE:	NANOBODY,GLUCOSIDASE YGJK,GLUCOSIDA
78:00:00	7s9w-B	2.6	5.6	68	529	6	MOLECULE:	DRMA
79:00:00	7wug-4	2.6	6	69	230	10	MOLECULE:	VACUOLAR IMPORT AND DEGRADATION PRO
80:00:00	6e3k-E	2.6	3.1	58	214	3	MOLECULE:	INTERFERON GAMMA
81:00:00	8h0h-A	2.6	8.6	75	145	8	MOLECULE:	UNCHARACTERIZED PROTEIN RV1546
82:00:00	7e2c-K	2.6	4.7	71	251	1	MOLECULE:	TRAPP-ASSOCIATED PROTEIN TCA17
83:00:00	8adl-C	2.6	4.5	68	782	4	MOLECULE:	MTC5 ISOFORM 1
84:00:00	3g16-A	2.6	4.4	60	156	2	MOLECULE:	UNCHARACTERIZED PROTEIN WITH CYSTAT
85:00:00	3t8n-B	2.6	4.5	72	130	8	MOLECULE:	STEROID DELTA-ISOMERASE
86:00:00	1zc4-B	2.6	3.7	57	113	5	MOLECULE:	RAS-RELATED PROTEIN RAL-A
87:00:00	4boe-A	2.6	4.3	58	154	9	MOLECULE:	JAPANIN
88:00:00	2fs2-B	2.6	4.2	73	138	7	MOLECULE:	PHENYLACETIC ACID DEGRADATION PROTE
89:00:00	5img-A	2.6	7.6	60	467	8	MOLECULE:	DIPEPTIDASE
90:00:00	8iex-A	2.6	4.8	54	88	4	MOLECULE:	PROBABLE WRKY TRANSCRIPTION FACTOR
91:00:00	6xlf-C	2.6	4.5	70	316	11	MOLECULE:	ATP-DEPENDENT MOLECULAR CHAPERONE H
92:00:00	8dek-A	2.6	4.8	69	440	3	MOLECULE:	NPCBM/NEW2 DOMAIN-CONTAINING PROTEI
93:00:00	4gb5-A	2.6	5.5	72	148	6	MOLECULE:	UNCHARACTERIZED PROTEIN
94:00:00	4rzm-B	2.6	5.1	64	283	0	MOLECULE:	EPOXIDE HYDROLASE LASB
95:00:00	8ath-A	2.6	3.9	68	168	7	MOLECULE:	LYSOSOME-ASSOCIATED MEMBRANE GLYCOP
96:00:00	5iri-A	2.6	3	52	105	8	MOLECULE:	SERINE/THREONINE-PROTEIN KINASE BRS
97:00:00	7tpr-A	2.6	10	75	1145	7	MOLECULE:	SPIKE GLYCOPROTEIN
98:00:00	7aqc-R	2.6	3.6	73	558	5	MOLECULE:	23S RIBOSOMAL RNA
99:00:00	7pkw-A	2.6	8.4	70	240	6	MOLECULE:	PUTATIVE TRANSFER PROTEIN
0:00	7jsr-A	2.6	4.6	66	1496	9	MOLECULE:	NAD-SPECIFIC GLUTAMATE DEHYDROGENAS
1:00	6gc1-C	2.6	4.6	68	551	6	MOLECULE:	NHL REPEAT-CONTAINING PROTEIN 2
2:00	6lwz-A	2.6	3.9	44	53	9	MOLECULE:	BACTERIOCIN
3:00	8e5f-A	2.6	9.6	73	293	10	MOLECULE:	C-TYPE CYTOCHROME
4:00	2ret-A	2.6	3.4	53	84	6	MOLECULE:	PSEUDOPILIN EPSI
5:00	6ppb-k	2.6	3.9	50	344	10	MOLECULE:	CAPSID VERTEX COMPONENT 1
6:00	8veh-C	2.6	4.9	55	194	5	MOLECULE:	OUTER MEMBRANE LIPOPROTEIN-SORTING
7:00	6gez-B	2.6	7	68	532	3	MOLECULE:	GREEN FLUORESCENT PROTEIN,OPTIMIZED
8:00	3adj-A	2.6	3.1	48	72	2	MOLECULE:	F21M12.9 PROTEIN
9:00	3sn0-A	2.6	2.4	46	401	4	MOLECULE:	PUTATIVE L-ALANINE-DL-GLUTAMATE EPI
10:00	6dzk-Y	2.6	3.8	59	103	12	MOLECULE:	16S RRNA

11:00	8dh8-A	2.6	5.2	69	700	6	MOLECULE:	LEPTIN RECEPTOR
12:00	6jx7-A	2.6	6.3	61	1245	7	MOLECULE:	FELINE INFECTIOUS PERITONITIS VIRUS
13:00	6bpm-A	2.6	8	87	711	6	MOLECULE:	CATECHOLATE SIDEROPHORE RECEPTOR FI
14:00	3o8e-D	2.6	5.7	58	248	0	MOLECULE:	FIBER 36.1 KDA PROTEIN
15:00	8t1l-I	2.6	4.8	65	1111	8	MOLECULE:	MEDIATOR OF RNA POLYMERASE II TRANS
16:00	6d72-C	2.6	3.9	59	177	3	MOLECULE:	SPERMIDINE N1-ACETYLTRANSFERASE
17:00	7z32-A	2.6	4.9	74	412	7	MOLECULE:	ACIDPHOSPHATASE
18:00	8umd-C	2.6	4.6	61	290	8	MOLECULE:	FLAGELLAR M-RING PROTEIN
19:00	4or8-B	2.6	3.8	61	226	8	MOLECULE:	MEMBRANE-ASSOCIATED PROTEIN VP24
20:00	7riz-A	2.6	6.6	62	306	2	MOLECULE:	BETA-PROPELLER LACTONASE
21:00	3cmb-B	2.6	6.8	78	266	8	MOLECULE:	ACETOACETATE DECARBOXYLASE
22:00	4fhn-B	2.6	4.1	77	1022	1	MOLECULE:	NUCLEOPORIN NUP37
23:00	3uc2-B	2.6	2.8	43	126	2	MOLECULE:	HYPOTHETICAL PROTEIN WITH IMMUNOGLO
24:00:00	4yhc-A	2.6	5.2	68	439	6	MOLECULE:	STEROL REGULATORY ELEMENT-BINDING P
25:00:00	8w4j-J	2.6	4	64	574	6	MOLECULE:	GLUTAMATE DEHYDROGENASE 1, MITOCHON
26:00:00	2bvb-A	2.6	4	66	137	9	MOLECULE:	MICRONEMAL PROTEIN 1
27:00:00	7uaw-A	2.6	4.1	55	125	7	MOLECULE:	ABC TRANSPORTER ATPASE
28:00:00	5x7o-A	2.6	7.6	59	1247	8	MOLECULE:	GLYCOSIDE HYDROLASE FAMILY 31 ALPHA
29:00:00	5isv-A	2.6	3.2	56	156	4	MOLECULE:	RIBOSOMAL-PROTEIN-ALANINE ACETYLTRA
30:00:00	8tar-R	2.6	4.6	63	440	2	MOLECULE:	ANAPHASE-PROMOTING COMPLEX SUBUNIT
31:00:00	8v8b-A	2.6	4.7	83	476	7	MOLECULE:	PORTAL PROTEIN
32:00:00	4qxz-A	2.6	3	53	146	8	MOLECULE:	UNCHARACTERIZED PROTEIN
33:00:00	1ei5-A	2.6	4	47	518	2	MOLECULE:	D-AMINOPEPTIDASE
34:00:00	5e6m-A	2.6	4.9	78	520	4	MOLECULE:	GLYCINE--TRNA LIGASE
35:00:00	1xsz-A	2.6	12	62	356	10	MOLECULE:	GUANINE NUCLEOTIDE EXCHANGE PROTEIN
36:00:00	2r4i-A	2.6	5.4	69	123	7	MOLECULE:	UNCHARACTERIZED PROTEIN
37:00:00	6sy0-A	2.6	3.2	50	137	2	MOLECULE:	TRANSCRIPTION FACTOR WITH AP2 DOMAI
38:00:00	2n8x-A	2.6	3.5	68	166	7	MOLECULE:	LPS-ASSEMBLY LIPOPROTEIN LPTE
39:00:00	7eeb-G	2.6	5.4	67	714	9	MOLECULE:	ENHANCED GREEN FLUORESCENT PROTEIN,
40:00:00	8sh2-D	2.6	5.9	70	384	10	MOLECULE:	KELCH DOMAIN-CONTAINING PROTEIN 2
41:00:00	8ssm-C	2.6	3.3	47	89	2	MOLECULE:	CONTACT-DEPENDENT INHIBITOR A
42:00:00	3tzg-A	2.6	5.6	76	239	0	MOLECULE:	HYPOTHETICAL PROTEIN BVU_2266
43:00:00	2q7a-A	2.6	2.9	54	152	7	MOLECULE:	CELL SURFACE HEME-BINDING PROTEIN
44:00:00	5ftx-A	2.6	3	61	651	5	MOLECULE:	SURFACE LAYER PROTEIN
45:00:00	5lql-A	2.6	3.8	62	155	8	MOLECULE:	4-HYDROXYBENZOYL-COA THIOESTERASE
46:00:00	6p2k-B	2.6	5.1	62	778	6	MOLECULE:	FIBRONECTIN TYPE III DOMAIN-CONTAIN
47:00:00	3kya-A	2.6	4.4	65	471	6	MOLECULE:	PUTATIVE PHOSPHATASE
48:00:00	5kku-B	2.6	4.3	65	289	3	MOLECULE:	POLYKETIDE SYNTHASE TYPE I
49:00:00	3wz4-G	2.6	5.1	64	140	11	MOLECULE:	DOTI
50:00:00	7jmn-N	2.6	6.4	63	198	10	MOLECULE:	MEDIATOR OF RNA POLYMERASE II TRANS
51:00:00	4akx-B	2.6	9.4	56	534	7	MOLECULE:	SPCU
52:00:00	6cbc-B	2.6	8	93	270	8	MOLECULE:	VACUOLAR PROTEIN SORTING-ASSOCIATED
53:00:00	7fhr-A	2.6	3.5	63	439	3	MOLECULE:	PUTATIVE PHTHALATE 4,5-DIOXYGENASE,
54:00:00	5boi-A	2.6	4.4	55	226	9	MOLECULE:	GERMINATION PROTEIN YPEB
55:00:00	3dmc-A	2.6	4.1	62	134	10	MOLECULE:	NTF2-LIKE PROTEIN
56:00:00	2p4o-A	2.6	7.6	54	302	9	MOLECULE:	HYPOTHETICAL PROTEIN

57:00:00	1vla-A	2.6	3.9	61	144	3	MOLECULE:	HYDROPEROXIDE RESISTANCE PROTEIN OS
58:00:00	3w5g-A	2.6	4.6	56	705	5	MOLECULE:	BETA-GALACTOSIDASE
59:00:00	8r1o-F	2.6	12.9	70	281	4	MOLECULE:	RRP45
60:00:00	3wz3-A	2.6	5.1	71	137	8	MOLECULE:	TRAM PROTEIN
61:00:00	8avg-A	2.6	4.3	62	603	3	MOLECULE:	ELONGATOR COMPLEX PROTEIN 1
62:00:00	8cuk-B	2.6	7.1	67	334	9	MOLECULE:	E3 UBIQUITIN-PROTEIN LIGASE PEP5
63:00:00	5u35-A	2.6	3.9	57	124	9	MOLECULE:	DE NOVO NTF2 WITH LARGE CAVITY
64:00:00	6isl-B	2.6	4.2	63	120	8	MOLECULE:	XIME, SNOAL-LIKE DOMAIN PROTEIN
65:00:00	3gwr-A	2.6	5.2	65	126	2	MOLECULE:	PUTATIVE CALCIUM/CALMODULIN-DEPENDE
66:00:00	8cuk-C	2.6	7	64	330	14	MOLECULE:	E3 UBIQUITIN-PROTEIN LIGASE PEP5
67:00:00	5a5u-B	2.6	8.1	65	613	8	MOLECULE:	EUKARYOTIC INITIATION FACTOR 3
68:00:00	6l4j-A	2.6	2.8	52	90	0	MOLECULE:	SINGLE CHAIN MONELLIN
69:00:00	5tpj-A	2.6	4.4	55	122	11	MOLECULE:	DENOVO NTF2
70:00:00	5d17-E	2.6	3.4	63	158	5	MOLECULE:	TRANSPOSON TN7 TRANSPOSITION PROTEI
71:00:00	6quh-C	2.6	9.8	59	228	8	MOLECULE:	GREEN FLUORESCENT PROTEIN
72:00:00	5b61-F	2.6	9	58	223	5	MOLECULE:	GREEN FLUORESCENT PROTEIN
73:00:00	2ib6-C	2.6	10.8	56	225	11	MOLECULE:	YELLOW MUTANT CHROMO PROTEIN
74:00:00	5wj2-B	2.6	8.7	57	226	7	MOLECULE:	CYAN FLUORESCENT PROTEIN
75:00:00	4w76-B	2.6	9.8	59	226	7	MOLECULE:	FLUORESCENT PROTEIN D21H/K26C
76:00:00	7sws-C	2.6	7.6	54	217	6	MOLECULE:	CHROMOPROTEIN AMILCP
77:00:00	7amb-D	2.6	8.7	57	229	7	MOLECULE:	GREEN FLUORESCENT PROTEIN
78:00:00	4tzg-E	2.6	4.4	63	217	8	MOLECULE:	FLUORESCENT PROTEIN
79:00:00	7rrh-D	2.6	9.7	63	215	5	MOLECULE:	FLUORESCENT PROTEIN DRONPA
80:00:00	6og9-B	2.6	5.9	53	226	6	MOLECULE:	CRYSTAL STRUCTURE OF GREEN FLUORESC
81:00:00	6fll-A	2.6	10.3	60	223	5	MOLECULE:	GREEN FLUORESCENT PROTEIN
82:00:00	3ls3-B	2.6	4.4	62	214	8	MOLECULE:	PADRON0.9
83:00:00	5hzu-D	2.6	8.4	52	212	6	MOLECULE:	FLUORESCENT PROTEIN DRONPA
84:00:00	5dpi-F	2.6	10	58	224	5	MOLECULE:	GREEN FLUORESCENT PROTEIN
85:00:00	2ib6-E	2.6	10.8	55	225	11	MOLECULE:	YELLOW MUTANT CHROMO PROTEIN
86:00:00	5dtl-H	2.6	9.7	63	219	2	MOLECULE:	GREEN TO RED PHOTOCONVERTIBLE GFP-L
87:00:00	5cm8-A	2.5	3.5	44	419	5	MOLECULE:	RAL GUANINE NUCLEOTIDE DISSOCIATION
88:00:00	3zwl-D	2.5	4.7	63	352	10	MOLECULE:	EUKARYOTIC TRANSLATION INITIATION F
89:00:00	3ge2-A	2.5	3	46	89	2	MOLECULE:	LIPOPROTEIN, PUTATIVE
90:00:00	3ub1-D	2.5	6.6	65	253	11	MOLECULE:	ORF13-LIKE PROTEIN
91:00:00	8h93-B	2.5	6.5	65	367	6	MOLECULE:	NACHT, LRR AND PYD DOMAINS-CONTAINI
92:00:00	8bo9-A	2.5	3.3	66	174	3	MOLECULE:	NON STRUCTURAL POLYPROTEIN
93:00:00	4ps6-A	2.5	3.8	60	130	3	MOLECULE:	INHIBITOR OF VERTEBRATE LYSOZYME
94:00:00	2l4v-A	2.5	3.2	65	135	8	MOLECULE:	CYSTATIN
95:00:00	2r5x-B	2.5	2.9	48	118	4	MOLECULE:	UNCHARACTERIZED CONSERVED PROTEIN
96:00:00	3edp-A	2.5	8.2	83	223	4	MOLECULE:	LIN2111 PROTEIN
97:00:00	8ju3-A	2.5	10.5	72	290	6	MOLECULE:	TAIL FIBER PROTEIN S,TAIL FIBER PRO
98:00:00	3hdp-A	2.5	11.7	53	132	8	MOLECULE:	GLYOXALASE-I
99:00:00	7myj-C	2.5	5	63	464	3	MOLECULE:	5'-AMP-ACTIVATED PROTEIN KINASE CAT
0:00	4ava-A	2.5	4.5	77	327	6	MOLECULE:	LYSINE ACETYLTRANSFERASE
1:00	5c9i-A	2.5	4.1	59	768	5	MOLECULE:	PROTEIN RELATED TO PENICILLIN ACYLA
2:00	8a43-B	2.5	3.5	65	1131	2	MOLECULE:	DNA-DIRECTED RNA POLYMERASE I SUBUN

3:00	4rl8-C	2.5	7.2	89	268	7	MOLECULE:	PROTEIN INVOLVED IN META-PATHWAY OF
4:00	8d82-A	2.5	3.5	53	589	0	MOLECULE:	SOLUBLE INTERLEUKIN-6 RECEPTOR SUBU
5:00	7acv-B	2.5	3.4	50	127	8	MOLECULE:	LMW SLP
6:00	1xs0-C	2.5	3.2	63	129	5	MOLECULE:	INHIBITOR OF VERTEBRATE LYSOZYME
7:00	3exz-B	2.5	4.3	64	147	6	MOLECULE:	MAOC-LIKE DEHYDRATASE
8:00	7cbl-A	2.5	7.6	69	211	4	MOLECULE:	FLAGELLAR L-RING PROTEIN
9:00	8gun-A	2.5	5.4	70	166	1	MOLECULE:	TYPE VII SECRETION SYSTEM PROTEIN E
10:00	6zwm-C	2.5	5.4	71	319	13	MOLECULE:	SERINE/THREONINE-PROTEIN KINASE MTO
11:00	2rqf-A	2.5	7.8	86	227	5	MOLECULE:	HEMOLYMPH JUVENILE HORMONE BINDING
12:00	8k44-A	2.5	6.7	72	280	6	MOLECULE:	VP9
13:00	3fgy-A	2.5	4.3	57	135	12	MOLECULE:	UNCHARACTERIZED NTF2-LIKE PROTEIN
14:00	8t2v-B	2.5	9.7	66	718	11	MOLECULE:	INTEGRIN ALPHA-IIB
15:00	3q63-F	2.5	4.3	69	139	6	MOLECULE:	MLL2253 PROTEIN
16:00	6xp5-Q	2.5	7.2	72	476	6	MOLECULE:	MEDIATOR OF RNA POLYMERASE II TRANS
17:00	4xb6-C	2.5	7.5	78	353	8	MOLECULE:	ALPHA-D-RIBOSE 1-METHYLPHOSPHONATE
18:00	7ml9-A	2.5	5	81	295	5	MOLECULE:	INSECTICIDAL PROTEIN
19:00	6gut-B	2.5	5.3	58	239	3	MOLECULE:	23S RRNA PSEUDOURIDINE SYNTHASE D,P
20:00	5b2o-A	2.5	5.2	58	1455	3	MOLECULE:	CRISPR-ASSOCIATED ENDONUCLEASE CAS9
21:00	5awh-A	2.5	6.4	68	759	4	MOLECULE:	UNCHARACTERIZED PROTEIN
22:00	5vgl-A	2.5	5.4	57	154	2	MOLECULE:	LACHRYMATORY-FACTOR SYNTHASE
23:00	8cgm-A	2.5	11.8	57	190	7	MOLECULE:	OUTER MEMBRANE LIPOPROTEIN CARRIER
24:00:00	2wgo-A	2.5	3.9	55	98	5	MOLECULE:	RANASPUMIN-2
25:00:00	4i60-A	2.5	4.7	74	128	3	MOLECULE:	AVIDIN
26:00:00	6s50-A	2.5	4	70	248	4	MOLECULE:	STREPTAVIDIN
27:00:00	4fvs-A	2.5	4.8	76	214	5	MOLECULE:	PUTATIVE LIPOPROTEIN
28:00:00	4jhy-A	2.5	3.9	61	156	7	MOLECULE:	HYPOTHETICAL PROTEIN
29:00:00	1tlw-A	2.5	11.7	93	251	6	MOLECULE:	NUCLEOSIDE-SPECIFIC CHANNEL-FORMING
30:00:00	1lo7-A	2.5	5.6	65	140	6	MOLECULE:	4-HYDROXYBENZOYL-COA THIOESTERASE
31:00:00	4ak1-A	2.5	5.4	58	600	5	MOLECULE:	BT_4661
32:00:00	2joe-A	2.5	4.3	59	139	7	MOLECULE:	HYPOTHETICAL LIPOPROTEIN YEHR
33:00:00	3qoo-A	2.5	6.8	72	135	7	MOLECULE:	UNCHARACTERIZED PROTEIN
34:00:00	5dyv-A	2.5	6.4	64	135	6	MOLECULE:	YD REPEAT-CONTAINING PROTEIN
35:00:00	6kme-A	2.5	5.1	68	284	4	MOLECULE:	PHYTOCHROMOBILIN SYNTHASE
36:00:00	6qzk-A	2.5	5.2	75	746	15	MOLECULE:	CLOSTRIDIUM BUTYRICUM ARGONAUTE
37:00:00	8i6v-D	2.5	5.9	65	678	2	MOLECULE:	VACUOLAR TRANSPORTER CHAPERONE COMP
38:00:00	4rul-A	2.5	9.8	60	821	5	MOLECULE:	DNA TOPOISOMERASE 1
39:00:00	6gvw-C	2.5	4.1	77	383	1	MOLECULE:	BRCA1-A COMPLEX SUBUNIT ABRAXAS 1
40:00:00	7omm-B	2.5	2.7	55	128	7	MOLECULE:	LPS-ASSEMBLY PROTEIN LPTD
41:00:00	1nbh-A	2.5	8.6	70	293	7	MOLECULE:	GLYCINE N-METHYLTRANSFERASE
42:00:00	4uw8-A	2.5	6.2	72	401	6	MOLECULE:	L-SHAPED TAIL FIBER PROTEIN
43:00:00	5h3k-A	2.5	9.6	79	535	5	MOLECULE:	SLR0280 PROTEIN
44:00:00	3fbq-A	2.5	4.7	68	283	4	MOLECULE:	CONSERVED DOMAIN PROTEIN
45:00:00	8th8-H	2.5	7.5	67	820	3	MOLECULE:	DYNEIN REGULATORY COMPLEX PROTEIN 1
46:00:00	5w5y-O	2.5	3.7	76	640	7	MOLECULE:	DNA-DIRECTED RNA POLYMERASE I SUBUN
47:00:00	7qb6-A	2.5	4.3	74	164	4	MOLECULE:	NODULIN-13
48:00:00	1yfq-A	2.5	5.8	55	342	2	MOLECULE:	CELL CYCLE ARREST PROTEIN BUB3

49:00:00	4e72-A	2.5	7	62	218	5	MOLECULE:	UNCHARACTERIZED PROTEIN
50:00:00	3ewk-A	2.5	5.2	54	227	0	MOLECULE:	SENSOR PROTEIN
51:00:00	3wyb-A	2.5	4.7	66	327	8	MOLECULE:	MESO-DIAMINOPIMELATE D-DEHYDROGENAS
52:00:00	6jp4-A	2.5	9	75	770	1	MOLECULE:	ALGINATE LYASE
53:00:00	6m15-A	2.5	5.6	71	965	7	MOLECULE:	SPIKE GLYCOPROTEIN
54:00:00	3f14-A	2.5	2.9	53	112	13	MOLECULE:	UNCHARACTERIZED NTF2-LIKE PROTEIN
55:00:00	5h2d-A	2.5	10.1	84	418	7	MOLECULE:	KLLA0C04147P
56:00:00	7bi2-A	2.5	5.4	59	1021	7	MOLECULE:	PHOSPHATIDYLINOSITOL 4-PHOSPHATE 3-
57:00:00	3icy-A	2.5	9.8	56	118	4	MOLECULE:	SENSOR PROTEIN
58:00:00	6xzq-C	2.5	8.8	65	772	8	MOLECULE:	RNA (5'-
59:00:00	4tmd-A	2.5	3.8	69	190	4	MOLECULE:	UNCHARACTERIZED PROTEIN
60:00:00	7dsk-A	2.5	3	53	471	2	MOLECULE:	4F2 CELL-SURFACE ANTIGEN HEAVY CHAI
61:00:00	2x12-A	2.5	2.8	55	200	0	MOLECULE:	FIMBRIAE-ASSOCIATED PROTEIN FAP1
62:00:00	1lsh-B	2.5	6.8	70	174	4	MOLECULE:	LIPOVITELLIN (LV-1N, LV-1C)
63:00:00	7sg7-S	2.5	3.7	65	471	6	MOLECULE:	GENE 14 PROTEIN
64:00:00	3o77-A	2.5	8.5	81	388	4	MOLECULE:	MYOSIN LIGHT CHAIN KINASE, SMOOTH M
65:00:00	1bh3-A	2.5	6.2	80	289	5	MOLECULE:	PORIN
66:00:00	6r9t-A	2.5	5.5	53	2185	8	MOLECULE:	TALIN-1
67:00:00	6b12-B	2.5	3.7	52	153	2	MOLECULE:	TNE2
68:00:00	5h1j-A	2.5	3.4	55	693	11	MOLECULE:	GEM-ASSOCIATED PROTEIN 5
69:00:00	5wox-A	2.5	7.2	53	145	8	MOLECULE:	UNCHARACTERIZED PROTEIN
70:00:00	7djl-A	2.5	4.4	61	473	8	MOLECULE:	PROTEIN SUPPRESSOR OF QUENCHING 1,
71:00:00	2ia9-D	2.5	3.7	44	99	5	MOLECULE:	PUTATIVE SEPTATION PROTEIN SPOVG
72:00:00	5mqr-A	2.5	3.6	61	1082	2	MOLECULE:	BETA-L-ARABINOBIOSIDASE
73:00:00	4id2-A	2.5	3.6	64	136	8	MOLECULE:	UNCHARACTERIZED PROTEIN
74:00:00	5o8o-A	2.5	7	75	332	3	MOLECULE:	MITOCHONDRIAL IMPORT RECEPTOR SUBUN
75:00:00	3k1l-A	2.5	7	57	376	0	MOLECULE:	FANCL
76:00:00	7su6-B	2.5	4.6	56	111	5	MOLECULE:	7,8-DIHYDRONEOPTERIN ALDOLASE
77:00:00	6vbu-2	2.5	3	47	659	6	MOLECULE:	BARDET-BIEDL SYNDROME 18 PROTEIN
78:00:00	2iv9-A	2.5	3.6	67	236	4	MOLECULE:	AP-2 COMPLEX SUBUNIT BETA-2
79:00:00	6u9f-A	2.5	4.7	51	717	4	MOLECULE:	PDPA
80:00:00	5xfm-A	2.5	5.6	93	632	8	MOLECULE:	ALPHA-GLUCOSIDASE
81:00:00	7lc1-B	2.5	4.4	56	218	4	MOLECULE:	ISOFORM 2B OF GTPASE KRAS
82:00:00	6f2p-A	2.5	6.5	74	1040	7	MOLECULE:	PAENIBACILLUS XANTHAN LYASE
83:00:00	1cvm-A	2.5	3.9	67	353	4	MOLECULE:	PHYTASE
84:00:00	3dzm-A	2.5	7	81	208	2	MOLECULE:	HYPOTHETICAL CONSERVED PROTEIN
85:00:00	3u97-A	2.5	3.1	45	77	11	MOLECULE:	RIBONUCLEASE TOXIN BRNT
86:00:00	6a9e-A	2.5	7.2	55	339	7	MOLECULE:	ENDOLYSIN,AUTOPHAGY-RELATED PROTEIN
87:00:00	4lxr-A	2.5	3.8	42	755	7	MOLECULE:	PROTEIN TOLL
88:00:00	5uwa-A	2.5	5	67	185	12	MOLECULE:	PROBABLE PHOSPHOLIPID-BINDING PROTE
89:00:00	2bib-A	2.5	5.1	59	540	2	MOLECULE:	TEICHOIC ACID PHOSPHORYLCHOLINE EST
90:00:00	5ho2-A	2.5	4.4	80	803	4	MOLECULE:	EXTRACELLULAR ARABINANASE
91:00:00	8d2j-B	2.5	5.3	48	483	6	MOLECULE:	IPD113_COW
92:00:00	7qzq-A	2.5	3.6	50	344	14	MOLECULE:	KELCH REPEAT AND BTB DOMAIN-CONTAIN
93:00:00	8g6e-b	2.5	3.8	56	256	7	MOLECULE:	PROTEASOME SUBUNIT ALPHA TYPE-6
94:00:00	1tu1-A	2.5	3.2	55	144	4	MOLECULE:	HYPOTHETICAL PROTEIN PA0094

95:00:00	5fr8-A	2.5	13.8	93	707	4	MOLECULE:	TONB-DEPENDENT SIDEROPHORE RECEPTOR
96:00:00	3q9d-A	2.5	1.9	38	175	11	MOLECULE:	PROTEIN CPN_0803/CP_1068/CPJ0803/CP
97:00:00	3k5j-A	2.5	5.1	51	182	2	MOLECULE:	SUPPRESSOR OF FUSED FAMILY PROTEIN
98:00:00	5if6-A	2.5	5.7	72	134	7	MOLECULE:	OHP9_1C
99:00:00	8qrl-D	2.5	4	62	343	2	MOLECULE:	12S MITOCHONDRIAL RRNA
0:00	7qrj-B	2.5	6.5	66	182	6	MOLECULE:	ZAV_19 PROTEIN
1:00	6ulg-N	2.5	7.9	56	483	2	MOLECULE:	FOLLICULIN
2:00	1nr0-A	2.5	9.7	63	610	5	MOLECULE:	ACTIN INTERACTING PROTEIN 1
3:00	2fkj-A	2.5	2.6	40	361	5	MOLECULE:	OUTER SURFACE PROTEIN A
4:00	6yug-A	2.5	4	59	143	5	MOLECULE:	DIAMINE ACETYLTRANSFERASE
5:00	5m1i-A	2.5	7	68	539	9	MOLECULE:	ALPHA-GALACTOSIDASE
6:00	6n7p-M	2.5	3.3	54	107	2	MOLECULE:	U1 SMALL NUCLEAR RIBONUCLEOPROTEIN
7:00	3o4j-C	2.5	4.6	64	579	6	MOLECULE:	ACYLAMINO-ACID-RELEASING ENZYME
8:00	4fyg-A	2.5	4.4	83	743	5	MOLECULE:	SIDF, INHIBITOR OF GROWTH FAMILY, M
9:00	6cl5-A	2.5	9.1	69	374	4	MOLECULE:	TAIL FIBER PROTEIN
10:00	5w0p-C	2.5	5	76	850	7	MOLECULE:	ENDOLYSIN,RHODOPSIN,S-ARRESTIN
11:00	7wug-Y	2.5	4.8	67	508	9	MOLECULE:	VACUOLAR IMPORT AND DEGRADATION PRO
12:00	8smu-A	2.5	9.1	59	393	10	MOLECULE:	HTAACR2 INTEGRAL FUSION WITHIN ENHA
13:00	6itw-A	2.5	3.2	58	221	7	MOLECULE:	TYPE VI IMMUNITY PROTEIN ATU4351
14:00	8jnx-A	2.5	5.3	64	662	2	MOLECULE:	ALPHA-AMYLASE
15:00	8g9t-K	2.5	4	50	582	8	MOLECULE:	ACRIC9
16:00	5ztm-A	2.5	10.4	54	168	7	MOLECULE:	DOSAGE COMPENSATION REGULATOR
17:00	6qam-A	2.5	7.3	65	211	6	MOLECULE:	OUTER MEMBRANE PROTEIN ALKL
18:00	6rlb-C	2.5	6	61	494	5	MOLECULE:	O6-ALKYLGUANINE-DNA ALKYLTRANSFERAS
19:00	3afl-A	2.5	4.9	73	766	7	MOLECULE:	OLIGO ALGINATE LYASE
20:00	8ewi-A	2.5	4.7	71	1775	6	MOLECULE:	E3 UBIQUITIN-PROTEIN LIGASE UBR5
21:00	5mwv-A	2.5	5.1	72	281	4	MOLECULE:	OUTER MEMBRANE PROTEIN G
22:00	6nd4-Q	2.5	4.5	64	862	6	MOLECULE:	ETS RRNA
23:00	6le0-A	2.5	5.9	67	209	3	MOLECULE:	ABMU
24:00:00	8p98-A	2.5	10.4	90	621	3	MOLECULE:	VITAMIN B12 TRANSPORTER BTUB
25:00:00	7agv-A	2.5	2.9	56	160	5	MOLECULE:	K(+)/H(+) ANTIPORTER SUBUNIT KHTT
26:00:00	8h2t-E	2.5	5.6	70	157	7	MOLECULE:	RIESKE (2FE-2S) DOMAIN PROTEIN
27:00:00	8f5p-C	2.5	6.1	62	1176	8	MOLECULE:	NET DOMAIN-CONTAINING PROTEIN
28:00:00	3gwr-B	2.5	5.4	66	128	2	MOLECULE:	PUTATIVE CALCIUM/CALMODULIN-DEPENDE
29:00:00	5trv-A	2.5	3.6	56	118	9	MOLECULE:	DENOVO NTF2
30:00:00	7qh3-C	2.5	9.9	63	137	6	MOLECULE:	RSFG
31:00:00	8h2t-A	2.5	5.5	71	157	7	MOLECULE:	RIESKE (2FE-2S) DOMAIN PROTEIN
32:00:00	5u9o-D	2.5	5.1	67	146	4	MOLECULE:	PLASTID DIVISION PROTEIN CDP1, CHLO
33:00:00	8aha-A	2.5	11.1	65	237	2	MOLECULE:	GREEN FLUORESCENT PROTEIN
34:00:00	5o68-C	2.5	6.4	84	262	6	MOLECULE:	FAPF
35:00:00	4w7x-B	2.5	6.1	57	220	4	MOLECULE:	FLUORESCENT PROTEIN E115C/T118H
36:00:00	4uer-b	2.5	7.6	81	480	2	MOLECULE:	EIF1A
37:00:00	5k1h-B	2.5	5.9	61	554	3	MOLECULE:	EUKARYOTIC TRANSLATION INITIATION F
38:00:00	8am4-A	2.5	8.2	54	229	9	MOLECULE:	GREEN FLUORESCENT PROTEIN
39:00:00	4w7f-A	2.5	8.2	53	213	9	MOLECULE:	FLUORESCENT PROTEIN E124H/K126H
40:00:00	5exb-J	2.5	4	73	221	8	MOLECULE:	GREEN FLUORESCENT PROTEIN

41:00:00	4jfg-A	2.5	8.9	57	225	5	MOLECULE:	GREEN FLUORESCENT PROTEIN
42:00:00	3ssy-A	2.5	10.7	69	227	6	MOLECULE:	GREEN FLUORESCENT PROTEIN
43:00:00	1emc-A	2.5	8.2	54	224	9	MOLECULE:	GREEN FLUORESCENT PROTEIN
44:00:00	3bpq-D	2.4	2.9	44	86	0	MOLECULE:	ANTITOXIN RELB3
45:00:00	5yx4-A	2.4	4.8	65	232	6	MOLECULE:	CHALCONE-FLAVONONE ISOMERASE FAMILY
46:00:00	7aak-A	2.4	4.9	71	345	6	MOLECULE:	PORPHOBILINOGEN DEAMINASE
47:00:00	2imj-A	2.4	4.3	68	158	9	MOLECULE:	HYPOTHETICAL PROTEIN DUF1348
48:00:00	1lsh-A	2.4	8.1	93	955	11	MOLECULE:	LIPOVITELLIN (LV-1N, LV-1C)
49:00:00	4lee-A	2.4	5.1	64	313	3	MOLECULE:	AGGLUTININ-LIKE PROTEIN 3
50:00:00	8wkl-G	2.4	4.2	66	307	8	MOLECULE:	STRICTOSIDINE SYNTHASE
51:00:00	2ztb-B	2.4	3.7	71	248	1	MOLECULE:	CRYSTAL PROTEIN
52:00:00	3dgc-S	2.4	3.2	54	207	6	MOLECULE:	INTERLEUKIN-22
53:00:00	3bb9-B	2.4	4.7	60	125	5	MOLECULE:	PUTATIVE ORPHAN PROTEIN
54:00:00	4l8p-A	2.4	6.1	73	168	1	MOLECULE:	BILE ACID 7A-DEHYDRATASE, BAIE
55:00:00	6td9-B	2.4	3.4	71	247	6	MOLECULE:	PA1624
56:00:00	3ty1-A	2.4	5.3	87	384	1	MOLECULE:	HYPOTHETICAL ALDOSE 1-EPIMERASE
57:00:00	3rby-A	2.4	4.1	69	245	4	MOLECULE:	UNCHARACTERIZED PROTEIN YLR301W
58:00:00	5jrb-K	2.4	8.1	66	188	6	MOLECULE:	DNA REPAIR PROTEIN RAD52 HOMOLOG
59:00:00	3g1j-A	2.4	3.2	49	90	6	MOLECULE:	INTEGRON CASSETTE PROTEIN
60:00:00	3pu2-B	2.4	4.6	69	153	4	MOLECULE:	UNCHARACTERIZED PROTEIN
61:00:00	2wb7-A	2.4	5.3	74	525	4	MOLECULE:	PT26-6P
62:00:00	5gv0-A	2.4	4.8	68	162	7	MOLECULE:	LYSOSOME-ASSOCIATED MEMBRANE GLYCOP
63:00:00	2qzi-A	2.4	3	49	101	10	MOLECULE:	UNCHARACTERIZED PROTEIN
64:00:00	9be2-A	2.4	10.1	70	335	6	MOLECULE:	NUCLEOID-ASSOCIATED PROTEIN YEJK
65:00:00	8r54-A	2.4	4.4	79	1982	8	MOLECULE:	TENEURIN-3
66:00:00	1l3a-C	2.4	6.6	60	182	2	MOLECULE:	P24: PLANT TRANSCRIPTIONAL REGULATO
67:00:00	7vqo-A	2.4	9	77	1180	4	MOLECULE:	AMS1, NBR1 AND MALE FUSION PROTEIN
68:00:00	3k0z-A	2.4	5.6	72	149	7	MOLECULE:	PUTATIVE POLYKETIDE CYCLASE
69:00:00	7f13-A	2.4	10.4	78	154	9	MOLECULE:	DCR3
70:00:00	7zr1-D	2.4	10.2	72	780	7	MOLECULE:	DOUBLE-STRAND BREAK REPAIR PROTEIN
71:00:00	4esq-A	2.4	4.8	72	194	4	MOLECULE:	SERINE/THREONINE PROTEIN KINASE
72:00:00	6xrb-A	2.4	2.9	60	150	10	MOLECULE:	SCIW
73:00:00	3mi6-D	2.4	5.8	84	743	11	MOLECULE:	ALPHA-GALACTOSIDASE
74:00:00	8rkh-A	2.4	8.1	66	219	6	MOLECULE:	ZONA PELLUCIDA SPERM-BINDING PROTEI
75:00:00	6ipv-A	2.4	3	68	221	6	MOLECULE:	CQSB2
76:00:00	2il5-A	2.4	6.7	72	162	7	MOLECULE:	HYPOTHETICAL PROTEIN
77:00:00	3ggr-B	2.4	7.3	61	278	3	MOLECULE:	CELL CYCLE CHECKPOINT CONTROL PROTE
78:00:00	4ql0-A	2.4	7.4	89	520	4	MOLECULE:	FILAMENTOUS HEMAGGLUTININ TRANSPORT
79:00:00	6e5u-A	2.4	4.7	70	339	10	MOLECULE:	NUCLEAR RNA EXPORT FACTOR 1
80:00:00	5da9-A	2.4	10.9	71	433	7	MOLECULE:	PUTATIVE UNCHARACTERIZED PROTEIN,PU
81:00:00	4kc5-A	2.4	5.5	83	914	2	MOLECULE:	RHIE PROTEIN
82:00:00	3c5p-A	2.4	9.5	78	194	14	MOLECULE:	PROTEIN BAS0735 OF UNKNOWN FUNCTION
83:00:00	4kwy-A	2.4	4.6	67	137	9	MOLECULE:	PUTATIVE UNCHARACTERIZED PROTEIN
84:00:00	5lal-B	2.4	6.1	69	158	7	MOLECULE:	DIRIGENT PROTEIN 6
85:00:00	3bcy-A	2.4	2.9	57	146	5	MOLECULE:	PROTEIN YER067W
86:00:00	4dkk-A	2.4	2.1	45	104	2	MOLECULE:	DOUBLE-STRANDED RNA-BINDING PROTEIN

87:00:00	6zu9-Q	2.4	9.9	70	226	4	MOLECULE:	18S RIBOSOMAL RNA
88:00:00	2fpn-A	2.4	5.9	64	205	5	MOLECULE:	YWMB
89:00:00	3v9o-A	2.4	6.6	63	121	5	MOLECULE:	DIHYDRONEOPTERIN ALDOLASE
90:00:00	2w9j-B	2.4	3.4	51	82	4	MOLECULE:	SIGNAL RECOGNITION PARTICLE SUBUNIT
91:00:00	4qan-A	2.4	4.4	65	385	2	MOLECULE:	HYPOTHETICAL PROTEIN
92:00:00	5k21-B	2.4	4.6	63	123	11	MOLECULE:	PYOCYANIN DEMETHYLASE
93:00:00	1vjh-A	2.4	4.3	59	120	5	MOLECULE:	BET V I ALLERGEN FAMILY
94:00:00	4ol4-A	2.4	3.5	63	177	8	MOLECULE:	PROLINE-RICH 28 KDA ANTIGEN
95:00:00	1t6a-A	2.4	4.2	52	124	10	MOLECULE:	RBSTP2229 GENE PRODUCT
96:00:00	7pw9-A	2.4	8.2	63	1952	6	MOLECULE:	SERINE/THREONINE-PROTEIN KINASE SMG
97:00:00	8kg3-A	2.4	5.4	67	311	7	MOLECULE:	OS06G0623700 PROTEIN
98:00:00	3gek-A	2.4	4.6	60	132	8	MOLECULE:	PUTATIVE THIOESTERASE YHDA
99:00:00	7w7i-B	2.4	7.4	80	342	5	MOLECULE:	FCTA DOMAIN-CONTAINING PROTEIN
0:00	5e4m-A	2.4	4.5	61	177	2	MOLECULE:	HYDROXYNITRILE LYASE
1:00	4fnv-A	2.4	6	76	659	9	MOLECULE:	HEPARINASE III PROTEIN, HEPARITIN S
2:00	7bcz-A	2.4	4.1	59	242	3	MOLECULE:	4'-PHOSPHOPANTETHEINYL TRANSFERASE
3:00	3qq2-B	2.4	7.6	89	250	6	MOLECULE:	BRKA AUTOTRANSPORTER
4:00	8e4g-P	2.4	7.5	76	793	5	MOLECULE:	PORTAL PROTEIN
5:00	7brm-A	2.4	6.6	84	257	5	MOLECULE:	CURLI PRODUCTION ASSEMBLY/TRANSPORT
6:00	3gzb-A	2.4	5.3	78	154	6	MOLECULE:	PUTATIVE SNOAL-LIKE POLYKETIDE CYCL
7:00	6z9c-A	2.4	3.6	70	258	4	MOLECULE:	POLYMERASE DELTA-INTERACTING PROTEI
8:00	3n91-A	2.4	5.5	74	315	9	MOLECULE:	UNCHARACTERIZED PROTEIN
9:00	4wp5-A	2.4	8.5	81	199	4	MOLECULE:	MRNA EXPORT PROTEIN
10:00	4dk6-C	2.4	4.6	38	87	8	MOLECULE:	SINGLE DOMAIN ANTIBODY VHH
11:00	1bqs-A	2.4	3.7	58	209	9	MOLECULE:	PROTEIN (MUCOSAL ADDRESSIN CELL ADH
12:00	4hg6-A	2.4	5.8	81	747	9	MOLECULE:	CELLULOSE SYNTHASE SUBUNIT A
13:00	4u3q-B	2.4	3.4	48	99	13	MOLECULE:	17 KDA LIPOPROTEIN
14:00	6em5-d	2.4	4.7	53	107	2	MOLECULE:	5.8S RIBOSOMAL RNA
15:00	3aki-A	2.4	6.5	72	448	3	MOLECULE:	PUTATIVE SECRETED ALPHA L-ARABINOFU
16:00	1dml-E	2.4	6.6	53	275	13	MOLECULE:	DNA POLYMERASE PROCESSIVITY FACTOR
17:00	5yje-B	2.4	11.5	59	318	7	MOLECULE:	PROTEIN HIRA
18:00	4ll4-C	2.4	5	65	281	8	MOLECULE:	THIOREDOXIN-INTERACTING PROTEIN
19:00	5a1v-K	2.4	8	50	1125	10	MOLECULE:	ADP-RIBOSYLATION FACTOR 1
20:00	8a9b-B	2.4	6.3	84	809	4	MOLECULE:	LIPID BINDING PROTEIN P116 (MPN213)
21:00	7z6e-C	2.4	6.8	63	534	8	MOLECULE:	SERINE/THREONINE-PROTEIN KINASE MRC
22:00	6orj-A	2.4	12.1	61	243	3	MOLECULE:	PHIKZ164
23:00	3af5-A	2.4	4.3	58	638	5	MOLECULE:	PUTATIVE UNCHARACTERIZED PROTEIN PH
24:00:00	5hl8-A	2.4	3.2	48	81	2	MOLECULE:	TYPE II SECRETION SYSTEM PROTEIN L
25:00:00	6gj1-D	2.4	4.1	59	121	3	MOLECULE:	PUTATIVE TYPE VI SECRETION PROTEIN
26:00:00	6z3b-A	2.4	8.1	59	371	7	MOLECULE:	GFO/IDH/MOCA FAMILY OXIDOREDUCTASE
27:00:00	8cm1-A	2.4	7	63	179	3	MOLECULE:	OUTER-MEMBRANE LIPOPROTEIN LOLB
28:00:00	8y19-A	2.4	6.3	61	1208	7	MOLECULE:	SPIKE GLYCOPROTEIN
29:00:00	7rpy-A	2.4	3.2	66	241	6	MOLECULE:	COHESIN-CONTAINING PROTEIN
30:00:00	7oix-A	2.4	7.2	81	265	5	MOLECULE:	SYNCYTIN-2
31:00:00	3u4y-A	2.4	4.4	63	319	11	MOLECULE:	UNCHARACTERIZED PROTEIN
32:00:00	5nzu-D	2.4	8.3	61	425	11	MOLECULE:	COATOMER SUBUNIT ALPHA

33:00:00	3hw2-A	2.4	5.1	62	308	3	MOLECULE:	PROTEIN SIFA
34:00:00	8ahx-G	2.4	5.1	74	197	4	MOLECULE:	ION-TRANSLOCATING OXIDOREDUCTASE CO
35:00:00	8uci-A	2.4	7.4	52	381	4	MOLECULE:	ATP DEPENDENT DNA LIGASE
36:00:00	1n7u-A	2.4	6.1	75	554	7	MOLECULE:	ADSORPTION PROTEIN P2
37:00:00	7z4f-J	2.4	5.3	92	603	9	MOLECULE:	PUTATIVE STRUCTURAL PROTEIN
38:00:00	7bjt-A	2.4	7.8	69	727	4	MOLECULE:	ALGINATE LYASE, FAMILY PL17
39:00:00	7ylz-A	2.4	14.8	78	576	4	MOLECULE:	HYDROXYAMIDOTRANSFERASE
40:00:00	8dys-A	2.4	5.2	62	409	5	MOLECULE:	EUKARYOTIC TRANSLATION INITIATION F
41:00:00	2pmq-B	2.4	6.1	52	374	6	MOLECULE:	MANDELATE RACEMASE/MUCONATE LACTONI
42:00:00	3fvz-A	2.4	5.1	56	329	9	MOLECULE:	PEPTIDYL-GLYCINE ALPHA-AMIDATING MO
43:00:00	6rkw-B	2.4	3.1	47	781	6	MOLECULE:	DNA GYRASE SUBUNIT A
44:00:00	4ozq-B	2.4	10	78	687	6	MOLECULE:	CHIMERA OF MALTOSE-BINDING PERIPLAS
45:00:00	4k15-A	2.4	9.9	60	138	2	MOLECULE:	LMO2686 PROTEIN
46:00:00	7eu9-A	2.4	6.3	70	1078	4	MOLECULE:	CAS12I1 D647A MUTANT
47:00:00	6trq-C	2.4	9.6	52	336	6	MOLECULE:	M7GPPPX DIPHOSPHATASE
48:00:00	4asc-A	2.4	4.3	68	308	0	MOLECULE:	KELCH REPEAT AND BTB DOMAIN-CONTAIN
49:00:00	5jyx-B	2.4	6.8	63	109	2	MOLECULE:	ARCHEAOSINE SYNTHASE QUEF-LIKE
50:00:00	4fvk-A	2.4	5.3	71	367	3	MOLECULE:	NEURAMINIDASE
51:00:00	6chg-A	2.4	4.1	61	305	2	MOLECULE:	KLLA0E24487P
52:00:00	5oa3-0	2.4	9.1	63	479	5	MOLECULE:	EUKARYOTIC TRANSLATION INITIATION F
53:00:00	3b7c-A	2.4	4.8	66	121	5	MOLECULE:	UNCHARACTERIZED PROTEIN
54:00:00	8pel-E	2.4	10	67	304	4	MOLECULE:	RRP45
55:00:00	5vzt-B	2.4	5.9	69	418	4	MOLECULE:	S-PHASE KINASE-ASSOCIATED PROTEIN 1
56:00:00	8t8s-B	2.4	6.7	66	664	8	MOLECULE:	SORTILIN
57:00:00	5c0p-A	2.4	3.8	65	284	5	MOLECULE:	ENDO-ARABINASE
58:00:00	5y6q-C	2.4	6.3	82	748	9	MOLECULE:	ALDEHYDE OXIDASE SMALL SUBUNIT
59:00:00	8etf-C	2.4	7.2	62	330	8	MOLECULE:	CHOLOYLGLYCINE HYDROLASE
60:00:00	1lqs-R	2.4	3.4	61	207	7	MOLECULE:	INTERLEUKIN-10 RECEPTOR ALPHA CHAIN
61:00:00	6m76-A	2.4	8.5	64	923	3	MOLECULE:	LPXTG-MOTIF CELL WALL ANCHOR DOMAIN
62:00:00	6zyg-A	2.4	3.1	43	113	2	MOLECULE:	PROTEALYSIN-ASSOCIATED PROTEIN
63:00:00	4fmb-A	2.4	4.4	67	351	1	MOLECULE:	CYSTEINE PROTEASE-LIKE VIRA
64:00:00	6mfi-A	2.4	5.2	51	264	6	MOLECULE:	METALLO-BETA-LACTAMASE
65:00:00	5lox-1	2.4	6.6	75	241	7	MOLECULE:	PEPTIDASE
66:00:00	7vnx-A	2.4	7.5	48	216	8	MOLECULE:	TKARKI
67:00:00	7uwf-A	2.4	3.3	54	358	13	MOLECULE:	WD REPEAT-CONTAINING PROTEIN 18
68:00:00	7bkb-k	2.4	3.8	45	386	4	MOLECULE:	COB--COM HETERODISULFIDE REDUCTASE
69:00:00	7mge-A	2.4	5.5	61	349	3	MOLECULE:	WD REPEAT-CONTAINING PROTEIN 41
70:00:00	5dky-A	2.4	3.2	64	928	3	MOLECULE:	ALPHA GLUCOSIDASE-LIKE PROTEIN
71:00:00	7c5w-A	2.4	4.9	61	164	5	MOLECULE:	IOTA-CARBONIC ANHYDRASE
72:00:00	7plc-D	2.4	4.6	64	286	2	MOLECULE:	SMP-30/CGR1 FAMILY PROTEIN
73:00:00	2oit-A	2.4	5.5	74	434	4	MOLECULE:	NUCLEOPORIN 214KDA
74:00:00	6uio-B	2.4	4.8	55	110	7	MOLECULE:	CYSTATIN-8
75:00:00	7epn-A	2.4	4.7	61	133	7	MOLECULE:	SNOAL-LIKE DOMAIN-CONTAINING PROTEI
76:00:00	8ghl-G	2.4	6.2	57	276	7	MOLECULE:	PROTEIN HIR1
77:00:00	6h01-C	2.4	4.9	57	230	7	MOLECULE:	GREEN FLUORESCENT PROTEIN
78:00:00	4w6l-A	2.4	5.8	51	204	4	MOLECULE:	FLUORESCENT PROTEIN D117C

79:00:00	3adf-B	2.4	5	60	209	5	MOLECULE:	MONOMERIC AZAMI GREEN
80:00:00	2whu-A	2.4	10.8	58	215	9	MOLECULE:	LARGE STOKES SHIFT FLUORESCENT PROT
81:00:00	2wht-C	2.4	10.9	59	215	8	MOLECULE:	LARGE STOKES SHIFT FLUORESCENT PROT
82:00:00	5hzo-A	2.4	8.4	54	228	9	MOLECULE:	GREEN FLUORESCENT PROTEIN
83:00:00	3evv-A	2.4	6.9	76	385	5	MOLECULE:	MYOSIN LIGHT CHAIN KINASE, GREEN FL
84:00:00	6fp8-A	2.4	8.1	54	216	11	MOLECULE:	GFP-LIKE FLUORESCENT CHROMOPROTEIN
85:00:00	8ill-A	2.4	8.5	54	213	2	MOLECULE:	GREEN FLUORESCENT PROTEIN
86:00:00	3svs-C	2.4	11	57	222	11	MOLECULE:	MKATE S158A/S143C
87:00:00	6wv5-A	2.4	10.4	56	370	9	MOLECULE:	VITAMIN K EPOXIDE REDUCTASE CYS43SE
88:00:00	8i4o-C	2.4	5.2	75	206	7	MOLECULE:	SPLIT GREEN FLOURESCENT PROTEIN
89:00:00	6y1g-C	2.4	7.6	45	221	4	MOLECULE:	GFP-LIKE NON-FLUORESCENT CHROMOPROT
90:00:00	3mgf-B	2.4	4.7	61	209	5	MOLECULE:	FLUORESCENT PROTEIN
91:00:00	4w77-B	2.4	8.2	54	217	9	MOLECULE:	FLUORESCENT PROTEIN D21H/K26C
92:00:00	4w6r-N	2.4	5.6	50	174	4	MOLECULE:	FLUORESCENT PROTEIN D102C
93:00:00	5f9g-A	2.4	6.9	77	228	5	MOLECULE:	PNGFP1.5-Y.CRO,GREEN FLUORESCENT PR
94:00:00	5exb-C	2.4	8.2	54	223	6	MOLECULE:	GREEN FLUORESCENT PROTEIN
95:00:00	6nql-C	2.4	8.4	52	216	6	MOLECULE:	FLUORESCENT PROTEIN DRONPA
96:00:00	8i4o-I	2.4	5.1	75	204	7	MOLECULE:	SPLIT GREEN FLOURESCENT PROTEIN
97:00:00	6uru-A	2.4	10	82	500	5	MOLECULE:	IACHSNFR PRECURSOR
98:00:00	4jfg-H	2.4	8.4	55	225	5	MOLECULE:	GREEN FLUORESCENT PROTEIN
99:00:00	2ib5-F	2.4	10.8	56	225	11	MOLECULE:	CHROMO PROTEIN
0:00	5dpi-E	2.4	11.5	47	225	6	MOLECULE:	GREEN FLUORESCENT PROTEIN
1:00	5ox9-A	2.4	9.6	60	226	5	MOLECULE:	GREEN FLUORESCENT PROTEIN
2:00	8i4o-E	2.4	5.2	74	203	5	MOLECULE:	SPLIT GREEN FLOURESCENT PROTEIN
3:00	6un4-A	2.4	9.5	62	245	6	MOLECULE:	GREEN FLUORESCENT PROTEIN
4:00	8i4o-A	2.4	5	74	208	5	MOLECULE:	SPLIT GREEN FLOURESCENT PROTEIN
5:00	8jxg-A	2.3	4.7	63	1114	3	MOLECULE:	RAT RAP
6:00	2kie-A	2.3	5.1	56	124	2	MOLECULE:	INOSITOL POLYPHOSPHATE 5-PHOSPHATAS
7:00	2hjs-A	2.3	5.7	67	334	6	MOLECULE:	USG-1 PROTEIN HOMOLOG
8:00	1yax-A	2.3	6	66	153	8	MOLECULE:	VIRULENCE SENSOR PROTEIN PHOQ, SENS
9:00	2jgs-B	2.3	4.2	70	116	11	MOLECULE:	CIRCULAR PERMUTANT OF AVIDIN
10:00	8dl1-A	2.3	6	53	713	8	MOLECULE:	ALPHA AMYLASE, CATALYTIC DOMAIN PRO
11:00	4f8b-B	2.3	4.4	63	145	0	MOLECULE:	NADPH-DEPENDENT 7-CYANO-7-DEAZAGUAN
12:00	7kra-G	2.3	5.4	74	151	7	MOLECULE:	ER MEMBRANE PROTEIN COMPLEX SUBUNIT
13:00	5coz-A	2.3	4.9	85	347	7	MOLECULE:	UNCHARACTERIZED PROTEIN
14:00	5k36-C	2.3	10.1	67	340	6	MOLECULE:	EXOSOME COMPLEX COMPONENT RRP45
15:00	5bsz-A	2.3	4.2	68	248	6	MOLECULE:	N-METHYLTRANSFERASE
16:00	2vzs-A	2.3	6.2	63	857	5	MOLECULE:	EXO-BETA-D-GLUCOSAMINIDASE
17:00	3en1-B	2.3	6.5	74	180	8	MOLECULE:	BENZENE 1,2-DIOXYGENASE SUBUNIT ALP
18:00	7dpy-A	2.3	3	53	90	4	MOLECULE:	BRUCELLA ABORTUS PHIA
19:00	4imh-A	2.3	9.1	61	347	10	MOLECULE:	HEMIN DEGRADING FACTOR
20:00	1tbu-B	2.3	3	52	98	6	MOLECULE:	PEROXISOMAL ACYL-COENZYME A THIOEST
21:00	4x2m-A	2.3	5.8	73	177	11	MOLECULE:	MTR2
22:00	3m83-A	2.3	6.5	70	325	0	MOLECULE:	ACETYL XYLAN ESTERASE
23:00	5imm-A	2.3	3.8	53	120	8	MOLECULE:	NANOBODY
24:00:00	3l5z-A	2.3	3.3	60	143	3	MOLECULE:	TRANSCRIPTIONAL REGULATOR, GNTR FAM

25:00:00	8b7d-A	2.3	4.4	62	147	3	MOLECULE:	TRANSMEMBRANE PROTEIN 106B
26:00:00	7rpo-A	2.3	5.6	58	252	7	MOLECULE:	DNA POLYMERASE SLIDING CLAMP 1
27:00:00	7tmu-C	2.3	5	70	146	6	MOLECULE:	SRPBCC FAMILY PROTEIN
28:00:00	8gy2-A	2.3	4.6	79	723	4	MOLECULE:	ALCOHOL DEHYDROGENASE (QUINONE), DE
29:00:00	7ns9-A	2.3	6.5	58	178	5	MOLECULE:	TRIPHOSPHATE TUNNEL METALLOENZYME S
30:00:00	6f7k-B	2.3	3.9	61	547	7	MOLECULE:	TAILSPIKE
31:00:00	3bws-A	2.3	4	70	407	3	MOLECULE:	PROTEIN LP49
32:00:00	4mis-A	2.3	3.7	49	98	2	MOLECULE:	PUTATIVE UNCHARACTERIZED PROTEIN
33:00:00	6k4e-B	2.3	10.9	68	246	7	MOLECULE:	HAMP DOMAIN-CONTAINING PROTEIN
34:00:00	7b2a-A	2.3	4.9	65	162	5	MOLECULE:	CIRPA5
35:00:00	5eq7-A	2.3	4.8	55	258	4	MOLECULE:	INOSITOL MONOPHOSPHATASE
36:00:00	8ts6-A	2.3	5.8	85	500	2	MOLECULE:	PORTAL PROTEIN
37:00:00	7cu9-B	2.3	4.5	62	193	11	MOLECULE:	TUBE-FORMING PROTEIN IN MYCOBACTERI
38:00:00	4e6f-B	2.3	6.1	64	173	11	MOLECULE:	UNCHARACTERIZED PROTEIN
39:00:00	8e1m-C	2.3	5.1	74	264	7	MOLECULE:	MITOCHONDRIAL IMPORT INNER MEMBRANE
40:00:00	7mo0-B	2.3	2.6	45	126	4	MOLECULE:	GTP-BINDING NUCLEAR PROTEIN RAN
41:00:00	5g25-A	2.3	2.9	45	82	2	MOLECULE:	TYPE-IV LIKE COMPETENCE PILIN TTHA1
42:00:00	7d1b-A	2.3	9.3	62	302	6	MOLECULE:	LEUCINE AMINOPEPTIDASE
43:00:00	2wzp-A	2.3	5.4	59	266	3	MOLECULE:	PUTATIVE RECEPTOR BINDING PROTEIN
44:00:00	4jp0-A	2.3	4.5	83	378	5	MOLECULE:	43.8 KDA INSECTICIDAL CRYSTAL PROTE
45:00:00	2k4v-A	2.3	4.9	58	125	2	MOLECULE:	UNCHARACTERIZED PROTEIN PA1076
46:00:00	3nwz-A	2.3	5.3	62	155	5	MOLECULE:	BH2602 PROTEIN
47:00:00	7zlv-h	2.3	3.6	69	527	3	MOLECULE:	PROBABLE CENTRAL STRAIGHT FIBER
48:00:00	5nwm-A	2.3	10.6	65	132	3	MOLECULE:	NUCLEAR RECEPTOR COACTIVATOR 1
49:00:00	2xgr-A	2.3	4.9	78	197	5	MOLECULE:	SPD1 NUCLEASE
50:00:00	3gvj-A	2.3	3.7	80	670	8	MOLECULE:	ENDO-N-ACETYLNEURAMINIDASE
51:00:00	3rqb-B	2.3	6.9	68	265	9	MOLECULE:	UNCHARACTERIZED PROTEIN
52:00:00	1hpw-A	2.3	9.5	65	129	5	MOLECULE:	FIMBRIAL PROTEIN
53:00:00	4eg9-A	2.3	5.4	81	231	4	MOLECULE:	UNCHARACTERIZED PROTEIN SAOUHSC_027
54:00:00	3hfi-A	2.3	2.8	55	137	5	MOLECULE:	PUTATIVE REGULATOR
55:00:00	2d42-A	2.3	2.6	48	249	2	MOLECULE:	NON-TOXIC CRYSTAL PROTEIN
56:00:00	4ffu-K	2.3	3.2	59	135	10	MOLECULE:	OXIDASE
57:00:00	2m7o-A	2.3	3.3	43	70	14	MOLECULE:	UNCHARACTERIZED PROTEIN
58:00:00	2a8i-A	2.3	7.6	91	341	5	MOLECULE:	THREONINE ASPARTASE 1
59:00:00	4fzq-A	2.3	4.3	52	79	2	MOLECULE:	UNCHARACTERIZED PROTEIN CONSERVED I
60:00:00	2wbv-B	2.3	5	60	186	8	MOLECULE:	FIBER PROTEIN
61:00:00	8wzb-G	2.3	8.6	59	387	3	MOLECULE:	DPY30 DOMAIN CONTAINING 2
62:00:00	2gxf-A	2.3	3.2	53	119	2	MOLECULE:	HYPOTHETICAL PROTEIN YYBH
63:00:00	4q28-A	2.3	3.6	51	110	6	MOLECULE:	PERIPLAKIN
64:00:00	5e27-A	2.3	8.9	58	226	7	MOLECULE:	RESUSCITATION-PROMOTING FACTOR RPFB
65:00:00	1su3-B	2.3	4	55	416	5	MOLECULE:	INTERSTITIAL COLLAGENASE
66:00:00	7kqq-B	2.3	6.5	60	306	5	MOLECULE:	WD REPEAT-CONTAINING PROTEIN 55
67:00:00	4ggt-B	2.3	4.1	63	106	5	MOLECULE:	BRADAVIDIN 2
68:00:00	5f0j-B	2.3	6.2	75	306	4	MOLECULE:	VACUOLAR PROTEIN SORTING-ASSOCIATED
69:00:00	7vmb-A	2.3	6.1	67	359	7	MOLECULE:	IQ MOTIF AND SEC7 DOMAIN-CONTAINING
70:00:00	5ukg-A	2.3	9.1	79	393	3	MOLECULE:	K-GECO

71:00:00	5gzl-A	2.3	3.9	77	358	3	MOLECULE:	LYSINE CYCLODEAMINASE
72:00:00	3lju-X	2.3	3.8	56	373	9	MOLECULE:	ARF-GAP WITH DUAL PH DOMAIN-CONTAIN
73:00:00	8ffz-E	2.3	4.8	69	518	7	MOLECULE:	TRANSCRIPTION FACTOR IIIA
74:00:00	3ijc-A	2.3	4.9	60	357	0	MOLECULE:	POLYCOMB PROTEIN EED
75:00:00	5gyy-B	2.3	7.2	67	405	3	MOLECULE:	S-RECEPTOR KINASE SRK9
76:00:00	7pfp-A	2.3	6.9	63	563	5	MOLECULE:	UROMODULIN
77:00:00	5wru-E	2.3	5.2	56	311	4	MOLECULE:	PROBABLE INORGANIC PYROPHOSPHATASE
78:00:00	8ey4-A	2.3	2	39	88	10	MOLECULE:	CYS_RICH_CPCC DOMAIN-CONTAINING PRO
79:00:00	6upk-G	2.3	3	45	423	4	MOLECULE:	HISTONE H3.1
80:00:00	3kk7-A	2.3	7.5	77	502	1	MOLECULE:	PUTATIVE CELL INVASION PROTEIN WITH
81:00:00	2dsb-A	2.3	10.6	47	206	11	MOLECULE:	ADP-SUGAR PYROPHOSPHATASE
82:00:00	8dit-C	2.3	5.3	69	736	4	MOLECULE:	VACUOLAR PROTEIN SORTING-ASSOCIATED
83:00:00	6um1-A	2.3	5	53	2208	8	MOLECULE:	CATION-INDEPENDENT MANNOSE-6-PHOSPH
84:00:00	8eec-A	2.3	5.1	67	317	4	MOLECULE:	ISOFORM 2 OF MITOGEN-ACTIVATED PROT
85:00:00	5hr7-A	2.3	8.2	63	361	11	MOLECULE:	TRNA GLU
86:00:00	5ch1-A	2.3	5.2	62	481	5	MOLECULE:	PUTATIVE POLYCOMB PROTEIN EED
87:00:00	3rri-A	2.3	3.9	51	132	4	MOLECULE:	GLYOXALASE/BLEOMYCIN RESISTANCE PRO
88:00:00	5fql-A	2.3	5.3	83	507	7	MOLECULE:	IDURONATE-2-SULFATASE
89:00:00	8c8g-B	2.3	6.5	57	1195	7	MOLECULE:	PUTATIVE BOTULINUM-LIKE TOXIN WO
90:00:00	7w1m-B	2.3	12	61	528	7	MOLECULE:	STRUCTURAL MAINTENANCE OF CHROMOSOM
91:00:00	1r0u-A	2.3	4.6	60	142	5	MOLECULE:	PROTEIN YWIB
92:00:00	1kit-A	2.3	5.3	69	757	3	MOLECULE:	SIALIDASE
93:00:00	8jxc-A	2.3	4.6	65	1337	11	MOLECULE:	LDL RECEPTOR RELATED PROTEIN 2
94:00:00	7usl-C	2.3	17.3	64	715	6	MOLECULE:	INTEGRIN ALPHA-M
95:00:00	4fzm-A	2.3	4.7	65	276	2	MOLECULE:	BACTERIOCIN
96:00:00	3jbr-F	2.3	5.9	58	872	0	MOLECULE:	VOLTAGE-DEPENDENT L-TYPE CALCIUM CH
97:00:00	5fzp-A	2.3	3.7	57	344	2	MOLECULE:	DISPASE AUTOLYSIS-INDUCING PROTEIN
98:00:00	3dm0-A	2.3	5	64	675	5	MOLECULE:	MALTOSE-BINDING PERIPLASMIC PROTEIN
99:00:00	7klj-A	2.3	4.3	48	324	8	MOLECULE:	ISOFORM 2 OF KINESIN-LIKE PROTEIN K
0:00	6bxr-A	2.3	5.7	57	273	0	MOLECULE:	MITOCHONDRIAL ASSOCIATION FACTOR 1
1:00	7woo-D	2.3	5.9	69	1398	12	MOLECULE:	NUCLEOPORIN NIC96
2:00	3mlq-A	2.3	5	74	186	8	MOLECULE:	DNA-DIRECTED RNA POLYMERASE SUBUNIT
3:00	4orl-A	2.3	2.5	52	110	8	MOLECULE:	UNCHARACTERIZED PROTEIN
4:00	8t1l-L	2.3	3.2	58	559	2	MOLECULE:	MEDIATOR OF RNA POLYMERASE II TRANS
5:00	6nd4-O	2.3	4.4	60	832	10	MOLECULE:	ETS RRNA
6:00	5k1c-C	2.3	8.3	65	407	8	MOLECULE:	UBIQUITIN CARBOXYL-TERMINAL HYDROLA
7:00	5o63-B	2.3	3	54	161	7	MOLECULE:	RESTRICTION ENDONUCLEASE UBALAI
8:00	3f5o-A	2.3	2.9	65	138	5	MOLECULE:	THIOESTERASE SUPERFAMILY MEMBER 2
9:00	1gju-A	2.3	8	51	636	4	MOLECULE:	MALTODEXTRIN GLYCOSYLTRANSFERASE
10:00	8of7-A	2.3	4.5	70	143	14	MOLECULE:	RHS FAMILY PROTEIN
11:00	4i27-A	2.3	10.5	68	361	10	MOLECULE:	DNA NUCLEOTIDYLEXOTRANSFERASE
12:00	8f5o-C	2.3	4.8	55	1179	0	MOLECULE:	INTRAFLAGELLAR TRANSPORT PROTEIN 12
13:00	5ixh-B	2.3	7.2	59	161	8	MOLECULE:	YCEI-LIKE DOMAIN PROTEIN
14:00	3no2-A	2.3	3.7	64	274	3	MOLECULE:	UNCHARACTERIZED PROTEIN
15:00	5i8u-C	2.3	7.7	46	203	0	MOLECULE:	ADP-RIBOSE PYROPHOSPHATASE
16:00	6nd4-S	2.3	7.6	68	481	1	MOLECULE:	ETS RRNA

17:00	8pbz-B	2.3	5.7	56	1380	5	MOLECULE:	MGP-OPERON PROTEIN 3
18:00	2y3u-A	2.3	4.5	67	679	4	MOLECULE:	COLLAGENASE
19:00	6fpt-A	2.3	5.7	58	391	5	MOLECULE:	E3 UBIQUITIN-PROTEIN LIGASE TRIM71
20:00	3htx-D	2.3	4.4	55	796	5	MOLECULE:	HEN1
21:00	2z2o-C	2.3	5.7	59	299	3	MOLECULE:	VIRGINIAMYCIN B LYASE
22:00	8glo-A	2.3	3.8	60	255	7	MOLECULE:	HEMOPHILIN
23:00	6w17-F	2.3	5.3	55	168	4	MOLECULE:	ACTIN-RELATED PROTEIN 3
24:00:00	3kny-A	2.3	8.8	76	194	7	MOLECULE:	HYPOTHETICAL PROTEIN BT_3535
25:00:00	6ah0-W	2.3	6	58	463	3	MOLECULE:	U5SNRNA
26:00:00	8wqr-B	2.3	7.4	59	356	5	MOLECULE:	ACTIVATING MOLECULE IN BECN1-REGULA
27:00:00	6rlb-D	2.3	5.1	66	446	9	MOLECULE:	O6-ALKYLGUANINE-DNA ALKYLTRANSFERAS
28:00:00	3w0s-A	2.3	2.8	45	298	2	MOLECULE:	HYGROMYCIN-B 4-O-KINASE
29:00:00	8ttb-B	2.3	3.4	58	426	9	MOLECULE:	SERINE/THREONINE-PROTEIN PHOSPHATAS
30:00:00	6wg3-A	2.3	10.6	61	561	5	MOLECULE:	STRUCTURAL MAINTENANCE OF CHROMOSOM
31:00:00	8q7n-F	2.3	6	60	431	8	MOLECULE:	U5 SNRNA
32:00:00	5ncl-A	2.3	13.5	58	418	5	MOLECULE:	SERINE/THREONINE-PROTEIN KINASE CBK
33:00:00	4ci8-A	2.3	9.2	56	640	4	MOLECULE:	ECHINODERM MICROTUBULE-ASSOCIATED P
34:00:00	7xp3-A	2.3	4.4	58	153	2	MOLECULE:	NAC DOMAIN-CONTAINING PROTEIN 92
35:00:00	2por-A	2.3	7.9	78	301	5	MOLECULE:	PORIN
36:00:00	7wh0-A	2.3	5.7	66	529	6	MOLECULE:	BETA-CAROTENE 15,15'-MONOOXYGENASE
37:00:00	8h6h-A	2.3	5.2	70	986	4	MOLECULE:	CELLODEXTRIN PHOSPHORYLASE
38:00:00	8boz-J	2.3	5.5	54	216	9	MOLECULE:	TRANSMEMBRANE PROTEIN
39:00:00	2cwz-A	2.3	4.5	67	138	6	MOLECULE:	THIOESTERASE FAMILY PROTEIN
40:00:00	4gk9-A	2.3	4.2	54	279	4	MOLECULE:	AGGLUTININ (BOA)
41:00:00	3i0y-A	2.3	4.6	67	138	4	MOLECULE:	PUTATIVE POLYKETIDE CYCLASE
42:00:00	2p9w-A	2.3	5.4	66	333	8	MOLECULE:	MAL S 1 ALLERGENIC PROTEIN
43:00:00	8ajm-B	2.3	5.2	60	395	3	MOLECULE:	DNA DAMAGE-BINDING PROTEIN 1
44:00:00	2jox-A	2.3	3.5	54	106	4	MOLECULE:	CHURCHILL PROTEIN
45:00:00	4g1e-A	2.3	7.8	75	964	15	MOLECULE:	INTEGRIN ALPHA-V
46:00:00	7kpz-B	2.3	5.7	58	126	7	MOLECULE:	HUPZ
47:00:00	1sjw-A	2.3	7.5	64	142	5	MOLECULE:	NOGALONIC ACID METHYL ESTER CYCLASE
48:00:00	8ghn-B	2.3	5.7	65	800	5	MOLECULE:	PROTEIN HIR1
49:00:00	4po7-A	2.3	7	59	658	2	MOLECULE:	SORTILIN
50:00:00	4r1k-A	2.3	5.8	62	135	3	MOLECULE:	UNCHARACTERIZED PROTEIN
51:00:00	5tgn-D	2.3	4	56	109	11	MOLECULE:	UNCHARACTERIZED PROTEIN
52:00:00	7c5v-A	2.3	5.2	59	164	0	MOLECULE:	IOTA-CARBONIC ANHYDRASE
53:00:00	1mwt-A	2.3	6.3	72	635	11	MOLECULE:	PBP2A
54:00:00	7c5v-B	2.3	4.9	62	163	6	MOLECULE:	IOTA-CARBONIC ANHYDRASE
55:00:00	7aqo-E	2.3	5.5	59	348	7	MOLECULE:	THO COMPLEX SUBUNIT 2
56:00:00	7kd9-E	2.3	6.6	82	219	7	MOLECULE:	GALLATE DECARBOXYLASE
57:00:00	5ts4-A	2.3	4.9	48	101	6	MOLECULE:	DENOVO NTF2
58:00:00	3ec9-A	2.3	5.4	63	130	3	MOLECULE:	UNCHARACTERIZED NTF2-LIKE PROTEIN
59:00:00	7uhy-B	2.3	3.8	58	761	5	MOLECULE:	GATOR COMPLEX PROTEIN MIOS
60:00:00	7plc-C	2.3	6	64	286	2	MOLECULE:	SMP-30/CGR1 FAMILY PROTEIN
61:00:00	4xzz-B	2.3	5.4	70	316	9	MOLECULE:	CONSERVED HYPOTHETICAL SECRETED PRO
62:00:00	5d17-M	2.3	3.3	62	159	5	MOLECULE:	TRANSPOSON TN7 TRANSPOSITION PROTEI

63:00:00	4w6p-B	2.3	5.7	50	214	4	MOLECULE:	FLUORESCENT PROTEIN D102C
64:00:00	4w6g-A	2.3	6.8	58	218	3	MOLECULE:	FLUORESCENT PROTEIN D190C
65:00:00	7lqo-A	2.3	5.9	75	229	5	MOLECULE:	RED FLUORESCENT PEROXYNITRITE BIOSE
66:00:00	5ukg-B	2.3	8.9	81	389	2	MOLECULE:	K-GECO
67:00:00	4w6p-A	2.3	10.6	57	217	4	MOLECULE:	FLUORESCENT PROTEIN D102C
68:00:00	7a7n-A	2.3	6.6	61	227	3	MOLECULE:	GREEN FLUORESCENT PROTEIN
69:00:00	4w7a-C	2.3	10.7	58	220	9	MOLECULE:	FLUORESCENT PROTEIN D21H/K26C
70:00:00	1yhg-B	2.3	8.1	53	224	13	MOLECULE:	GREEN FLUORESCENT PROTEIN
71:00:00	6un6-B	2.3	4.7	71	230	10	MOLECULE:	GREEN FLUORESCENT PROTEIN
72:00:00	6jc6-D	2.3	4.4	63	220	2	MOLECULE:	SHCP
73:00:00	4ik3-A	2.3	11.9	80	393	5	MOLECULE:	RCAMP, GREEN FLUORESCENT PROTEIN
74:00:00	4emq-F	2.3	4.4	62	213	6	MOLECULE:	FLUORESCENT PROTEIN DRONPA
75:00:00	5exb-D	2.3	9.3	58	221	7	MOLECULE:	GREEN FLUORESCENT PROTEIN
76:00:00	3lvc-B	2.3	8.3	55	226	9	MOLECULE:	GREEN FLUORESCENT PROTEIN
77:00:00	7y96-A	2.3	10.3	57	309	9	MOLECULE:	GREEN FLUORESCENT PROTEIN,MEMBRANE
78:00:00	8bxt-A	2.3	8.5	54	214	2	MOLECULE:	STAYGOLD
79:00:00	7qlj-A	2.3	4.7	62	219	5	MOLECULE:	RSKIIRO
80:00:00	4w6n-C	2.3	8.7	57	218	7	MOLECULE:	FLUORESCENT PROTEIN D117C
81:00:00	8i4o-G	2.3	5.8	72	205	7	MOLECULE:	SPLIT GREEN FLOURESCENT PROTEIN
82:00:00	6lnp-D	2.3	10.4	56	362	7	MOLECULE:	FUSION PROTEIN OF GREEN FLUORESCENT
83:00:00	6ofk-A	2.3	8.9	58	225	5	MOLECULE:	GREEN FLUORESCENT PROTEIN (GFP) S6
84:00:00	6ofn-B	2.3	5.3	70	236	10	MOLECULE:	GREEN FLUORESCENT PROTEIN (GFP) S6
85:00:00	6ya9-A	2.3	6.4	75	400	1	MOLECULE:	RSCGAMP
86:00:00	4w6r-F	2.3	8.4	53	201	9	MOLECULE:	FLUORESCENT PROTEIN D102C
87:00:00	2ib5-H	2.3	11.1	57	225	11	MOLECULE:	CHROMO PROTEIN
88:00:00	3mgf-D	2.3	5.1	60	212	5	MOLECULE:	FLUORESCENT PROTEIN
89:00:00	3wck-A	2.3	4.7	67	217	3	MOLECULE:	MONOMERIC PHOTOSENITIZING FLUORESCE
90:00:00	2odr-A	2.2	7.7	65	491	6	MOLECULE:	PHOSPHOSERYL-TRNA SYNTHETASE
91:00:00	1s5a-B	2.2	5.2	68	143	9	MOLECULE:	HYPOTHETICAL PROTEIN YESE
92:00:00	3d4e-A	2.2	7.3	45	162	2	MOLECULE:	PUTATIVE BETA-LACTAMASE INHIBITOR P
93:00:00	2amu-A	2.2	2.7	52	132	6	MOLECULE:	PUTATIVE SUPEROXIDE REDUCTASE
94:00:00	2jmu-A	2.2	3.3	62	224	2	MOLECULE:	THIAMINE-TRIPHOSPHATASE
95:00:00	7lhg-C	2.2	6.9	76	805	1	MOLECULE:	P FIMBRIAL USHER PROTEIN PAPC
96:00:00	1qab-E	2.2	4.4	69	180	12	MOLECULE:	PROTEIN (TRANSTHYRETIN)
97:00:00	3j2m-A	2.2	4	65	211	3	MOLECULE:	TAIL CONNECTOR PROTEIN GP15
98:00:00	2q78-F	2.2	8.5	71	136	6	MOLECULE:	UNCHARACTERIZED PROTEIN
99:00:00	4gah-B	2.2	4	66	195	6	MOLECULE:	THIOESTERASE SUPERFAMILY MEMBER 4
0:00	8ih7-B	2.2	4	63	317	3	MOLECULE:	4-HYDROXY-2-OXOVALERATE ALDOLASE
1:00	3bc9-A	2.2	4.3	54	585	7	MOLECULE:	ALPHA AMYLASE, CATALYTIC REGION
2:00	7orl-A	2.2	7.3	82	2183	7	MOLECULE:	RNA (5'-D(*(GTG))-R(P*AP*AP*UP*AP*C
3:00	4rqo-B	2.2	4.2	68	448	6	MOLECULE:	L-SERINE DEHYDRATASE
4:00	4q6u-B	2.2	5	64	324	3	MOLECULE:	UNCHARACTERIZED PROTEIN
5:00	8bad-B	2.2	4.9	88	351	6	MOLECULE:	BINARY TOXIN A-LIKE PROTEIN
6:00	8k49-T	2.2	5.7	74	627	5	MOLECULE:	VP2
7:00	7wb4-c	2.2	3.8	59	346	2	MOLECULE:	OUTER NUP133
8:00	1f13-A	2.2	12.2	68	721	4	MOLECULE:	CELLULAR COAGULATION FACTOR XIII ZY

9:00	8r1o-D	2.2	10.4	63	233	5	MOLECULE:	RRP45
10:00	5jxl-A	2.2	3.4	74	851	7	MOLECULE:	FLAGELLAR HOOK PROTEIN FLGE
11:00	5zx9-A	2.2	2.7	57	165	2	MOLECULE:	ALANINE AND PROLINE-RICH SECRETED P
12:00	6ihc-F	2.2	4.1	67	153	4	MOLECULE:	3-HYDROXYACYL-[ACYL-CARRIER-PROTEIN
13:00	8x6f-C	2.2	6.6	65	1153	3	MOLECULE:	DNA-DIRECTED RNA POLYMERASE SUBUNIT
14:00	7p2p-C	2.2	4.3	72	174	6	MOLECULE:	SIGNAL PEPTIDASE COMPLEX CATALYTIC
15:00	3ceb-A	2.2	6.8	47	194	6	MOLECULE:	D-AMINOACID AMINOTRANSFERASE-LIKE P
16:00	4doh-B	2.2	3.5	51	193	2	MOLECULE:	INTERLEUKIN-20
17:00	4uoj-A	2.2	5.3	74	917	7	MOLECULE:	BETA-MANNOSIDASE GH2
18:00	7k4o-A	2.2	6.2	78	554	10	MOLECULE:	CARBOXYLIC ESTER HYDROLASE
19:00	1jmo-A	2.2	3.4	67	427	7	MOLECULE:	THROMBIN, LIGHT CHAIN
20:00	4pw1-B	2.2	5.9	56	215	5	MOLECULE:	UNCHARACTERIZED PROTEIN
21:00	5fmv-A	2.2	3.3	57	351	7	MOLECULE:	RECEPTOR-TYPE TYROSINE-PROTEIN PHOS
22:00	2xem-D	2.2	3.4	60	146	13	MOLECULE:	DYNE7
23:00	2bmo-B	2.2	5.9	82	194	7	MOLECULE:	OXYGENASE-ALPHA NBDO
24:00:00	3bnv-D	2.2	4.3	64	143	9	MOLECULE:	CJ0977
25:00:00	6wum-a	2.2	11.3	90	445	7	MOLECULE:	SAM35
26:00:00	4l8n-A	2.2	9.2	72	448	6	MOLECULE:	PDZ DOMAIN PROTEIN
27:00:00	3r87-A	2.2	3.7	58	132	7	MOLECULE:	PUTATIVE UNCHARACTERIZED PROTEIN
28:00:00	3fig-B	2.2	4.1	64	578	5	MOLECULE:	2-ISOPROPYLMALATE SYNTHASE
29:00:00	6ged-A	2.2	3.8	76	295	4	MOLECULE:	PRGB
30:00:00	2v5y-A	2.2	3.4	59	564	5	MOLECULE:	RECEPTOR-TYPE TYROSINE-PROTEIN PHOS
31:00:00	6zyx-Y	2.2	8.9	90	1067	4	MOLECULE:	DYNEIN HEAVY CHAIN, OUTER ARM PROTE
32:00:00	2ve7-B	2.2	6.5	61	303	5	MOLECULE:	KINETOCHORE PROTEIN HEC1, KINETOCHO
33:00:00	6hiu-A	2.2	7	65	143	3	MOLECULE:	CYTOCHROME P460
34:00:00	8oe4-D	2.2	3.7	56	510	2	MOLECULE:	INTERLEUKIN-12 SUBUNIT BETA
35:00:00	2kd2-A	2.2	2.8	47	94	4	MOLECULE:	FAS APOPTOTIC INHIBITORY MOLECULE 1
36:00:00	3rd4-B	2.2	4.3	50	82	6	MOLECULE:	UNCHARACTERIZED PROTEIN
37:00:00	2ymw-B	2.2	5.5	77	685	9	MOLECULE:	L-LYSINE 6-OXIDASE
38:00:00	2dyt-A	2.2	5.6	82	213	1	MOLECULE:	AUTOPHAGY-RELATED PROTEIN 3
39:00:00	5cn2-A	2.2	3.1	53	115	11	MOLECULE:	ADP-RIBOSYLATION FACTOR-BINDING PRO
40:00:00	4e0s-B	2.2	7.5	85	898	6	MOLECULE:	COMPLEMENT C5
41:00:00	3to3-B	2.2	9.2	53	595	8	MOLECULE:	PETROBACTIN BIOSYNTHESIS PROTEIN AS
42:00:00	8uq9-a	2.2	3.6	66	433	6	MOLECULE:	E3 UBIQUITIN-PROTEIN LIGASE RNF168,
43:00:00	8pel-C	2.2	11.3	69	311	10	MOLECULE:	RRP45
44:00:00	8hbn-A	2.2	4.1	66	371	5	MOLECULE:	MRNA EXPORT FACTOR MEX67
45:00:00	3w15-A	2.2	6.4	55	336	5	MOLECULE:	PEROXISOMAL TARGETING SIGNAL 2 RECE
46:00:00	1z24-A	2.2	4.2	59	189	8	MOLECULE:	INSECTICYANIN A FORM
47:00:00	4ycz-A	2.2	4.3	58	713	5	MOLECULE:	FUSION PROTEIN OF SEC13 AND NUP145C
48:00:00	6nqz-B	2.2	4.5	60	215	2	MOLECULE:	FLAGELLAR COILING PROTEIN B
49:00:00	5j6q-A	2.2	10.1	63	595	6	MOLECULE:	CELL WALL BINDING PROTEIN CWP8
50:00:00	6j6q-F	2.2	4.8	65	161	2	MOLECULE:	PRE-MRNA-SPLICING FACTOR 8
51:00:00	6fwv-A	2.2	4.3	68	522	3	MOLECULE:	COLLAGEN ADHESION PROTEIN
52:00:00	3fka-B	2.2	4.8	58	120	3	MOLECULE:	UNCHARACTERIZED NTF-2 LIKE PROTEIN
53:00:00	7uic-n	2.2	10	82	220	5	MOLECULE:	MEDIATOR OF RNA POLYMERASE II TRANS
54:00:00	3qs3-H	2.2	3.4	65	177	12	MOLECULE:	FIMBRILLIN MATB HOMOLOG, ECPD

55:00:00	5u8o-B	2.2	3.8	55	360	0	MOLECULE:	ZN-DEPENDENT HYDROLASE
56:00:00	2ooi-B	2.2	9.1	67	158	6	MOLECULE:	SA0254 PROTEIN
57:00:00	6wo0-A	2.2	5.7	67	359	7	MOLECULE:	PROTEIN ARTEMIS
58:00:00	2f1z-B	2.2	2.9	52	481	10	MOLECULE:	UBIQUITIN CARBOXYL-TERMINAL HYDROLA
59:00:00	3i0o-A	2.2	4.2	51	329	2	MOLECULE:	SPECTINOMYCIN PHOSPHOTRANSFERASE
60:00:00	6s62-A	2.2	4.9	59	485	3	MOLECULE:	PROPIONATE CATABOLIC PROTEIN PRPD
61:00:00	8dfv-K	2.2	5.6	57	253	7	MOLECULE:	ENDORIBONUCLEASE DCR-1
62:00:00	4ckn-C	2.2	3.2	57	181	7	MOLECULE:	SAS-6
63:00:00	4q1z-A	2.2	3.7	48	389	8	MOLECULE:	PUTATIVE LIPOPROTEIN
64:00:00	3c6k-D	2.2	8.3	52	348	4	MOLECULE:	SPERMINE SYNTHASE
65:00:00	6wes-A	2.2	3.8	63	158	8	MOLECULE:	TOX3
66:00:00	4jgl-A	2.2	4.6	67	152	7	MOLECULE:	HYPOTHETICAL PROTEIN
67:00:00	7vcf-F	2.2	6.5	79	402	9	MOLECULE:	TIC214
68:00:00	1ss4-A	2.2	3	44	149	9	MOLECULE:	GLYOXALASE FAMILY PROTEIN
69:00:00	1im0-A	2.2	7.2	90	262	3	MOLECULE:	OUTER MEMBRANE PHSOPHOLIPASE A
70:00:00	6rap-E	2.2	5.1	73	275	4	MOLECULE:	AFP1
71:00:00	4cg4-A	2.2	4	62	376	8	MOLECULE:	PYRIN
72:00:00	8khg-A	2.2	4.5	63	271	3	MOLECULE:	ITACONYL-COA HYDRATASE
73:00:00	5c0w-B	2.2	11.9	70	244	4	MOLECULE:	EXOSOME COMPLEX COMPONENT RRP45
74:00:00	7e5c-A	2.2	6.7	79	439	3	MOLECULE:	XAA-PRO DIPEPTIDASE
75:00:00	3soy-A	2.2	5.1	65	142	8	MOLECULE:	NTF2-LIKE SUPERFAMILY PROTEIN
76:00:00	3osm-A	2.2	3.4	59	119	3	MOLECULE:	SERINE/THREONINE-PROTEIN KINASE KCC
77:00:00	6w40-A	2.2	4.6	57	120	11	MOLECULE:	DENOVO NTF2
78:00:00	7zbo-A	2.2	5.2	75	348	8	MOLECULE:	AMINE DEHYDROGENASE
79:00:00	6vbu-5	2.2	4.2	59	300	7	MOLECULE:	BARDET-BIEDL SYNDROME 18 PROTEIN
80:00:00	3b7f-A	2.2	4.5	63	368	8	MOLECULE:	GLYCOSYL HYDROLASE, BNR REPEAT
81:00:00	1rwi-A	2.2	4.8	63	256	11	MOLECULE:	SERINE/THREONINE-PROTEIN KINASE PKN
82:00:00	7e38-B	2.2	7.3	65	305	5	MOLECULE:	DEOXYPODOPHYLLOTOXIN SYNTHASE
83:00:00	5g24-A	2.2	4.8	63	200	5	MOLECULE:	TYPE-IV LIKE PILIN TTHA1219
84:00:00	7pgr-F	2.2	9.1	64	2423	9	MOLECULE:	NEUROFIBROMIN
85:00:00	8hsb-B	2.2	3.2	59	159	10	MOLECULE:	CDNG
86:00:00	7lyt-A	2.2	3.2	59	712	3	MOLECULE:	CASPHI
87:00:00	3azv-A	2.2	4.4	66	418	3	MOLECULE:	D/C MOSAIC NEUROTOXIN
88:00:00	7zm6-B	2.2	6	80	433	5	MOLECULE:	ATTACHMENT PROTEIN
89:00:00	6qm7-H	2.2	3.8	58	229	5	MOLECULE:	PROTEASOME ALPHA1 CHAIN
90:00:00	3e8p-A	2.2	3.6	70	153	7	MOLECULE:	UNCHARACTERIZED PROTEIN
91:00:00	6qmu-A	2.2	5.6	68	184	4	MOLECULE:	NEUTROPHIL GELATINASE-ASSOCIATED LI
92:00:00	4p07-A	2.2	3.8	71	569	8	MOLECULE:	ARYLSULFATE SULFOTRANSFERASE ASST
93:00:00	5aiw-A	2.2	4.5	69	127	7	MOLECULE:	TRAH
94:00:00	8hbn-B	2.2	5.1	69	165	10	MOLECULE:	MRNA EXPORT FACTOR MEX67
95:00:00	8fne-A	2.2	7.6	68	525	4	MOLECULE:	MALTOSE/MALTODEXTRIN-BINDING PERIPL
96:00:00	8ee7-A	2.2	10	64	456	11	MOLECULE:	PTUA
97:00:00	8cp6-B	2.2	16.1	73	1084	10	MOLECULE:	TOXIN PROTEIN TSE5
98:00:00	7q05-E	2.2	5.2	64	409	5	MOLECULE:	TEREPHTHALATE 1,2-DIOXYGENASE, TERM
99:00:00	7mni-C	2.2	4.1	61	412	5	MOLECULE:	NUCLEAR PORE COMPLEX PROTEIN NUP88
0:00	6qz4-A	2.2	7.9	80	568	6	MOLECULE:	MONO(2-HYDROXYETHYL) TEREPHTHALATE

1:00	5mzv-C	2.2	6.5	61	293	3	MOLECULE:	INTERLEUKIN-12 SUBUNIT BETA
2:00	5je1-A	2.2	9.1	54	245	6	MOLECULE:	METHYL TRANSFERASE
3:00	4wl2-A	2.2	6.1	61	346	2	MOLECULE:	PUTATIVE EXPORTED CHOLOYLGLYCINE HY
4:00	6t9i-D	2.2	4.9	59	566	7	MOLECULE:	TRANSCRIPTION FACTOR SPT20
5:00	4xnn-A	2.2	5.4	66	445	8	MOLECULE:	CELLOBIOHYDROLASE CHBI
6:00	4fi1-A	2.2	12.8	54	371	6	MOLECULE:	CASEIN KINASE II SUBUNIT ALPHA
7:00	4v0n-D	2.2	5.5	59	304	5	MOLECULE:	BARDET-BIEDL SYNDROME 1 PROTEIN
8:00	4nkq-A	2.2	5.3	47	412	11	MOLECULE:	CYTOKINE RECEPTOR COMMON SUBUNIT BE
9:00	7qpg-B	2.2	9.5	59	591	5	MOLECULE:	PROTEIN ZWILCH HOMOLOG
10:00	2p3p-B	2.2	3.1	63	198	6	MOLECULE:	HYPOTHETICAL PROTEIN
11:00	4epc-A	2.2	3.5	50	152	2	MOLECULE:	N-ACETYLMURAMOYL-L-ALANINE AMIDASE
12:00	5o9z-L	2.2	4.5	56	459	4	MOLECULE:	PRE-MRNA-PROCESSING-SPLICING FACTOR
13:00	2vdu-D	2.2	4.9	62	376	0	MOLECULE:	TRNA (GUANINE-N(7)-)-METHYLTRANSFER
14:00	8onf-A	2.2	7.5	66	258	5	MOLECULE:	CELL WALL SURFACE ANCHOR FAMILY PRO
15:00	6zxf-z	2.2	3.8	52	373	10	MOLECULE:	PRE-18S RIBOSOMAL RNA
16:00	6a5g-B	2.2	6.1	65	157	5	MOLECULE:	[4+2] AND [4+6] CYCLASE STMD
17:00	6i9f-A	2.2	4.6	51	155	4	MOLECULE:	FATTY ACID-BINDING PROTEIN HOMOLOG
18:00	8day-A	2.2	4.2	58	399	5	MOLECULE:	DIMETHYLALLYLTRYPTOPHAN SYNTHASE 1
19:00	4dvy-P	2.2	5.7	61	656	5	MOLECULE:	CYTOTOXICITY-ASSOCIATED IMMUNODOMIN
20:00	6yvd-D	2.2	9.9	65	414	3	MOLECULE:	CONDENSIN COMPLEX SUBUNIT 2
21:00	6dgv-A	2.2	10.4	89	553	3	MOLECULE:	FLUORESCENT GABA SENSOR PRECURSOR
22:00	3cje-A	2.2	7	67	150	9	MOLECULE:	OSMC-LIKE PROTEIN
23:00	8qx8-B	2.2	8.4	57	728	2	MOLECULE:	VACUOLAR PROTEIN SORTING-ASSOCIATED
24:00:00	3s5t-A	2.2	7.3	61	253	2	MOLECULE:	DUF3298 FAMILY PROTEIN
25:00:00	6pon-A	2.2	3.2	66	255	8	MOLECULE:	ADHERENCE AND VIRULENCE PROTEIN A
26:00:00	8c47-B	2.2	4.9	47	151	9	MOLECULE:	PROFILIN
27:00:00	5oc7-D	2.2	4.1	47	112	11	MOLECULE:	BREAKPOINT CLUSTER REGION PROTEIN,P
28:00:00	2pso-B	2.2	9.3	57	198	11	MOLECULE:	STAR-RELATED LIPID TRANSFER PROTEIN
29:00:00	1k4n-A	2.2	9	54	183	2	MOLECULE:	PROTEIN EC4020
30:00:00	8opr-A	2.2	7.5	62	651	6	MOLECULE:	S-LAYER PROTEIN EA1
31:00:00	5cl2-A	2.2	3.7	60	246	2	MOLECULE:	SPORULATION-CONTROL PROTEIN SPO0M
32:00:00	5k3w-A	2.2	5.1	64	298	5	MOLECULE:	CPUTA1
33:00:00	7c3c-B	2.2	4.2	74	346	1	MOLECULE:	AOFLEA
34:00:00	5yww-A	2.2	8.9	59	485	3	MOLECULE:	NUCLEOTIDE BINDING PROTEIN PINC
35:00:00	1eqr-A	2.2	4.3	64	590	13	MOLECULE:	ASPARTYL-TRNA SYNTHETASE
36:00:00	6eke-C	2.2	4	40	43	5	MOLECULE:	LECTIN
37:00:00	8qyn-N	2.2	3.6	57	216	7	MOLECULE:	PROTEASOME SUBUNIT ALPHA TYPE-2
38:00:00	5cww-B	2.2	6	71	538	6	MOLECULE:	NUCLEOPORIN NUP145N
39:00:00	8beq-A	2.2	4.1	66	534	3	MOLECULE:	FRUCTOFURANOSIDASE FROM RHODOTORULA
40:00:00	2o62-A	2.2	6.3	60	269	8	MOLECULE:	HYPOTHETICAL PROTEIN
41:00:00	6wg3-B	2.2	11.4	62	693	8	MOLECULE:	STRUCTURAL MAINTENANCE OF CHROMOSOM
42:00:00	3e29-B	2.2	3.5	69	135	3	MOLECULE:	UNCHARACTERIZED PROTEIN Q7WE92_BORB
43:00:00	1ns0-B	2.2	3.6	48	346	4	MOLECULE:	GALACTOSE MUTAROTASE
44:00:00	5ejq-A	2.2	5.8	69	501	6	MOLECULE:	MYOSIN-I HEAVY CHAIN
45:00:00	6b3y-A	2.2	4.6	49	225	2	MOLECULE:	DENN DOMAIN-CONTAINING PROTEIN 3
46:00:00	4z48-A	2.2	8	69	240	1	MOLECULE:	UNCHARACTERIZED PROTEIN

47:00:00	5ine-A	2.2	6.1	51	348	6	MOLECULE:	PRE-GLYCOPROTEIN POLYPROTEIN GP COM
48:00:00	2fwv-A	2.2	3.8	65	190	5	MOLECULE:	HYPOTHETICAL PROTEIN MTUBF_01000852
49:00:00	8osv-A	2.2	5.2	53	248	8	MOLECULE:	GDMF
50:00:00	4yy8-B	2.2	6.5	71	377	11	MOLECULE:	KELCH PROTEIN
51:00:00	7zgr-D	2.2	4.7	58	395	3	MOLECULE:	PROTEIN CFT1
52:00:00	6qx6-A	2.2	5.5	63	264	11	MOLECULE:	FERREDOXIN BILIN REDUCTASE PLASTID
53:00:00	7pab-A	2.2	3.2	54	415	7	MOLECULE:	NUCLEAR EGRESS PROTEIN 2,NUCLEAR EG
54:00:00	5mus-B	2.2	8	64	328	6	MOLECULE:	L PROTEIN
55:00:00	5xn7-B	2.2	10.1	55	551	2	MOLECULE:	PUTATIVE RTX-TOXIN
56:00:00	4je3-A	2.2	5.3	67	244	10	MOLECULE:	CENTRAL KINETOCHORE SUBUNIT IML3
57:00:00	6n90-A	2.2	8.2	52	88	6	MOLECULE:	UNCHARACTERIZED PROTEIN
58:00:00	1ve3-B	2.2	8.7	66	227	5	MOLECULE:	HYPOTHETICAL PROTEIN PH0226
59:00:00	3u0s-A	2.2	7.6	75	327	0	MOLECULE:	DIISOPROPYL-FLUOROPHOSPHATASE
60:00:00	1vhz-A	2.2	8.5	44	186	9	MOLECULE:	ADP COMPOUNDS HYDROLASE NUDE
61:00:00	7ztq-A	2.2	6.3	55	236	5	MOLECULE:	CAROTENOID-BINDING PROTEIN
62:00:00	6y86-A	2.2	9.1	53	425	6	MOLECULE:	MEMBRANE PROTEIN INSERTASE YIDC
63:00:00	6f3a-h	2.2	6.5	53	401	11	MOLECULE:	ARP1 ACTIN RELATED PROTEIN 1 HOMOLO
64:00:00	4wp5-B	2.2	6.1	73	171	10	MOLECULE:	MRNA EXPORT PROTEIN
65:00:00	7jmv-A	2.2	6.2	85	223	6	MOLECULE:	PEA PATHOGENICITY PROTEIN 2
66:00:00	7f0o-B	2.2	8.9	70	149	9	MOLECULE:	NSRQ
67:00:00	4dki-A	2.2	5.3	68	642	9	MOLECULE:	PENICILLIN-BINDING PROTEIN 2'
68:00:00	7jmr-A	2.2	6.4	87	224	5	MOLECULE:	PEA PATHOGENICITY PROTEIN 2
69:00:00	7z6e-A	2.2	7.6	60	519	7	MOLECULE:	SERINE/THREONINE-PROTEIN KINASE MRC
70:00:00	5bka-F	2.2	5	68	134	10	MOLECULE:	HYDROXYLACYL-COA DEHYDROGENASE
71:00:00	8ghl-I	2.2	9.4	51	377	6	MOLECULE:	PROTEIN HIR1
72:00:00	6chg-D	2.2	5.1	61	408	3	MOLECULE:	KLLA0E24487P
73:00:00	8boz-N	2.2	5.6	52	206	10	MOLECULE:	TRANSMEMBRANE PROTEIN
74:00:00	8ghn-I	2.2	9.4	51	377	6	MOLECULE:	PROTEIN HIR1
75:00:00	4q53-B	2.2	2.4	51	109	8	MOLECULE:	UNCHARACTERIZED PROTEIN
76:00:00	8boz-L	2.2	5.5	51	206	8	MOLECULE:	TRANSMEMBRANE PROTEIN
77:00:00	2gex-A	2.2	4.8	65	146	5	MOLECULE:	SNOL
78:00:00	7f0z-A	2.2	8.8	69	139	7	MOLECULE:	NSRQ
79:00:00	8t1m-B	2.2	6	66	116	8	MOLECULE:	DUF1842 DOMAIN-CONTAINING PROTEIN
80:00:00	7yao-B	2.2	3.6	52	213	0	MOLECULE:	OXSTAYGOLD
81:00:00	3st0-A	2.2	10.3	57	226	2	MOLECULE:	GREEN FLUORESCENT PROTEIN
82:00:00	6xu4-B	2.2	8.6	78	382	5	MOLECULE:	FGCAMP
83:00:00	3osr-B	2.2	6.5	82	600	4	MOLECULE:	MALTOSE-BINDING PERIPLASMIC PROTEIN
84:00:00	5b61-A	2.2	8.4	55	222	5	MOLECULE:	GREEN FLUORESCENT PROTEIN
85:00:00	2ah8-A	2.2	6.6	62	232	3	MOLECULE:	GREEN FLUORESCENT PROTEIN
86:00:00	5yr2-B	2.2	6.1	74	228	4	MOLECULE:	GREEN FLUORESCENT PROTEIN
87:00:00	4w73-B	2.2	11.7	58	214	3	MOLECULE:	FLUORESCENT PROTEIN E115C/T118H
88:00:00	3rwa-G	2.2	5.2	62	232	2	MOLECULE:	FLUORESCENT PROTEIN FP480
89:00:00	4jfg-B	2.2	5.5	52	222	4	MOLECULE:	GREEN FLUORESCENT PROTEIN
90:00:00	5yr2-A	2.2	6	74	226	5	MOLECULE:	GREEN FLUORESCENT PROTEIN
91:00:00	6gqh-A	2.2	7.2	59	226	7	MOLECULE:	GREEN FLUORESCENT PROTEIN
92:00:00	5du0-A	2.2	9.8	59	227	7	MOLECULE:	GREEN FLUORESCENT PROTEIN

93:00:00	6nqq-F	2.2	4.3	59	219	8	MOLECULE: FLUORESCENT PROTEIN DRONPA
94:00:00	7y40-B	2.2	7.2	47	213	6	MOLECULE: STAYGOLD
95:00:00	3ek8-A	2.2	6.7	76	393	5	MOLECULE: MYOSIN LIGHT CHAIN KINASE, GREEN FL
96:00:00	2ib5-C	2.2	11	57	225	11	MOLECULE: CHROMO PROTEIN
97:00:00	7dna-F	2.2	5	56	205	7	MOLECULE: GREEN-TO-RED PHOTOCONVERTIBLE GFP-L
98:00:00	8gyf-A	2.2	7.2	47	213	6	MOLECULE: STAYGOLD(K192Y)
99:00:00	4w7x-C	2.2	8.7	57	223	7	MOLECULE: FLUORESCENT PROTEIN E115C/T118H
0:00	5wj2-A	2.2	8.2	55	234	5	MOLECULE: CYAN FLUORESCENT PROTEIN
1:00	8i4o-K	2.2	5.8	71	204	7	MOLECULE: SPLIT GREEN FLOURESCENT PROTEIN
2:00	3bxc-G	2.2	9.9	59	218	0	MOLECULE: FAR-RED FLUORESCENT PROTEIN MKATE
3:00	5jzl-A	2.2	10.6	68	231	9	MOLECULE: GREEN FLUORESCENT PROTEIN
4:00	8j2k-B	2.2	8.7	47	213	6	MOLECULE: STAYGOLD(N137A, Q140S)
5:00	6un7-A	2.2	6	68	226	10	MOLECULE: GREEN FLUORESCENT PROTEIN
6:00	8ovp-A	2.2	4.6	71	500	4	MOLECULE: PUTATIVE PERIPLASMIC BINDING TRANSP
7:00	8j2h-B	2.2	7.5	50	214	6	MOLECULE: STAYGOLD(N137A)
8:00	5ktg-A	2.2	8.4	54	308	4	MOLECULE: GREEN FLUORESCENT PROTEIN, BCL-2 HO
9:00	8xld-A	2.2	7.3	48	213	6	MOLECULE: STAYGOLD
10:00	2p4m-G	2.2	8.5	53	218	6	MOLECULE: GFP-LIKE NON-FLUORESCENT CHROMOPROT
11:00	4gio-A	2.1	2.7	51	96	6	MOLECULE: PUTATIVE LIPOPROTEIN
12:00	3k67-A	2.1	4.1	63	156	3	MOLECULE: PUTATIVE DEHYDRATASE AF1124
13:00	5dl7-A	2.1	7.9	91	402	4	MOLECULE: PORIN
14:00	5xev-A	2.1	9.1	79	409	8	MOLECULE: XAA-PRO DIPEPTIDASE,PEPTIDASE-RELAT
15:00	7n34-A	2.1	6.8	60	108	5	MOLECULE: CAP15
16:00	6pwv-D	2.1	5.8	70	357	4	MOLECULE: RETINOBLASTOMA-BINDING PROTEIN 5
17:00	5mr3-B	2.1	4.1	63	117	5	MOLECULE: EGG-LYSIN
18:00	4ruw-A	2.1	7	63	386	3	MOLECULE: ENDONUCLEASE/EXONUCLEASE/PHOSPHATAS
19:00	1ln1-A	2.1	4.1	54	203	6	MOLECULE: PHOSPHATIDYLCHOLINE TRANSFER PROTEI
20:00	7jjn-A	2.1	4.8	54	514	2	MOLECULE: GLYCOSIDASES
21:00	6e57-A	2.1	3.6	53	399	2	MOLECULE: SURFACE GLYCAN BINDING PROTEIN B
22:00	3mes-B	2.1	4.5	50	358	6	MOLECULE: CHOLINE KINASE
23:00	8oq3-D	2.1	6.8	63	1556	8	MOLECULE: COMPLEMENT C3
24:00:00	5flv-M	2.1	4.9	65	229	12	MOLECULE: HOMEOBOX PROTEIN NKX-2.5, T-BOX TRA
25:00:00	4e1t-A	2.1	8.6	82	245	6	MOLECULE: INVASIN
26:00:00	2pyw-A	2.1	4	55	417	5	MOLECULE: UNCHARACTERIZED PROTEIN
27:00:00	4tq2-A	2.1	4.9	72	177	7	MOLECULE: PUTATIVE PHYCOERYTHRIN LYASE
28:00:00	1i78-A	2.1	7.4	92	297	5	MOLECULE: PROTEASE VII
29:00:00	2onf-A	2.1	3.4	59	140	7	MOLECULE: HYPOTHETICAL PROTEIN TA0195
30:00:00	3og6-B	2.1	3.5	60	200	5	MOLECULE: INTERLEUKIN-29
31:00:00	6k7l-A	2.1	6.2	67	992	7	MOLECULE: PHOSPHOLIPID-TRANSPORTING ATPASE
32:00:00	1zxf-A	2.1	5.7	67	155	7	MOLECULE: CALC
33:00:00	8ew9-A	2.1	6.3	73	241	5	MOLECULE: ALTERED INHERITANCE OF MITOCHONDRIA
34:00:00	5npy-A	2.1	4.3	75	472	5	MOLECULE: FLAGELLAR BASAL BODY PROTEIN
35:00:00	3n72-B	2.1	5.2	61	152	5	MOLECULE: PUTATIVE ACTIVATOR OF HSP90
36:00:00	6ph4-B	2.1	5.2	56	460	4	MOLECULE: BLUE-LIGHT-ACTIVATED HISTIDINE KINA
37:00:00	4mjt-A	2.1	6.6	82	236	7	MOLECULE: MONALYSIN
38:00:00	8p1l-A	2.1	10.6	71	2036	7	MOLECULE: RNA-DIRECTED RNA POLYMERASE L

39:00:00	3uv1-A	2.1	5.9	82	190	6	MOLECULE:	DER F 7 ALLERGEN
40:00:00	5zki-B	2.1	4.6	77	304	8	MOLECULE:	NUCLEASE EXOG, MITOCHONDRIAL
41:00:00	5yz0-A	2.1	3	57	2362	4	MOLECULE:	SERINE/THREONINE-PROTEIN KINASE ATR
42:00:00	6egt-A	2.1	4.3	57	445	5	MOLECULE:	GLYCOPROTEIN
43:00:00	4nzd-A	2.1	6.4	66	210	2	MOLECULE:	INTERLEUKIN-21 RECEPTOR
44:00:00	3rwx-A	2.1	7.5	75	254	3	MOLECULE:	HYPOTHETICAL BACTERIAL OUTER MEMBRA
45:00:00	3p51-A	2.1	4.5	68	145	1	MOLECULE:	UNCHARACTERIZED PROTEIN
46:00:00	3tw5-A	2.1	5.5	80	344	1	MOLECULE:	TRANSGLUTAMINASE ELICITOR
47:00:00	2o5c-A	2.1	9.5	61	634	3	MOLECULE:	5'-D(*CP*GP*CP*AP*AP*CP*TP*T)-3'
48:00:00	2j8f-A	2.1	4.5	51	341	10	MOLECULE:	LYSOZYME
49:00:00	3oml-A	2.1	10	72	532	7	MOLECULE:	PEROXISOMAL MULTIFUNCTIONAL ENZYME
50:00:00	8hgn-A	2.1	4.6	70	353	6	MOLECULE:	BETA-METHYLMALYL-COA DEHYDRATASE
51:00:00	3tek-A	2.1	3.9	58	139	0	MOLECULE:	THERMODBP-SINGLE STRANDED DNA BINDI
52:00:00	6agf-B	2.1	8.5	64	173	6	MOLECULE:	SODIUM CHANNEL PROTEIN TYPE 4 SUBUN
53:00:00	4qni-A	2.1	5.7	73	321	5	MOLECULE:	UNCHARACTERIZED PROTEIN
54:00:00	6jjt-D	2.1	7.3	73	133	4	MOLECULE:	PHNH
55:00:00	3zt9-A	2.1	5.7	65	192	6	MOLECULE:	SERINE PHOSPHATASE
56:00:00	1j6r-A	2.1	3.8	68	197	16	MOLECULE:	METHIONINE SYNTHASE
57:00:00	4p2i-A	2.1	4	50	117	0	MOLECULE:	MKIAA0254 PROTEIN
58:00:00	8a16-B	2.1	4.1	53	244	8	MOLECULE:	RECEPTOR-TYPE TYROSINE-PROTEIN PHOS
59:00:00	6zgd-A	2.1	5.6	58	288	9	MOLECULE:	PROTON-GATED ION CHANNEL
60:00:00	2wpw-A	2.1	4	59	328	3	MOLECULE:	ORF14
61:00:00	2ntk-A	2.1	5.9	51	206	2	MOLECULE:	IMP CYCLOHYDROLASE
62:00:00	5jpq-i	2.1	5.4	57	659	7	MOLECULE:	WD40 DOMAIN PROTEINS
63:00:00	4i2x-E	2.1	3.4	51	314	8	MOLECULE:	FABOX117 LIGHT CHAIN
64:00:00	3ie5-B	2.1	3.6	72	164	7	MOLECULE:	PHENOLIC OXIDATIVE COUPLING PROTEIN
65:00:00	2cuh-A	2.1	5.3	59	115	7	MOLECULE:	TENASCIN-X
66:00:00	5yq0-C	2.1	7.2	99	304	8	MOLECULE:	COFJ
67:00:00	1zwy-B	2.1	4.3	61	180	5	MOLECULE:	HYPOTHETICAL UPF0244 PROTEIN VC0702
68:00:00	2j2z-A	2.1	7.9	64	216	11	MOLECULE:	CHAPERONE PROTEIN PAPD
69:00:00	6hsw-A	2.1	8.5	70	422	9	MOLECULE:	CARBOHYDRATE ESTERASE FAMILY 15 DOM
70:00:00	5v77-A	2.1	3.3	46	106	0	MOLECULE:	UNCHARACTERIZED PROTEIN
71:00:00	4hrv-B	2.1	4.4	63	144	8	MOLECULE:	PUTATIVE LIPOPROTEIN GNA1162
72:00:00	7pvh-A	2.1	4.4	77	195	1	MOLECULE:	POR SECRETION SYSTEM PROTEIN PORN/G
73:00:00	5ymx-A	2.1	4.2	51	202	4	MOLECULE:	MUTUAL GLIDING-MOTILITY PROTEIN MGL
74:00:00	2ehb-D	2.1	3.7	53	126	6	MOLECULE:	CALCINEURIN B-LIKE PROTEIN 4
75:00:00	5dz8-A	2.1	5.7	69	167	1	MOLECULE:	BSPA (BSPA_V)
76:00:00	1vi8-B	2.1	4.5	60	146	7	MOLECULE:	HYPOTHETICAL PROTEIN YDII
77:00:00	2mvb-A	2.1	7.6	67	165	3	MOLECULE:	UNCHARACTERIZED PROTEIN
78:00:00	4gbs-B	2.1	6.5	58	186	5	MOLECULE:	PUTATIVE LIPOPROTEIN
79:00:00	4mjg-A	2.1	3.2	61	177	2	MOLECULE:	HYPOTHETICAL PROTEIN
80:00:00	3pcs-B	2.1	4.5	86	355	2	MOLECULE:	ESPG
81:00:00	4lsd-A	2.1	3.6	51	98	4	MOLECULE:	FIBRONECTIN TYPE III DOMAIN-CONTAIN
82:00:00	6ycq-A	2.1	8.2	65	347	2	MOLECULE:	AUXIN RESPONSE FACTOR 1
83:00:00	4qdc-A	2.1	4.1	78	369	6	MOLECULE:	3-KETOSTEROID 9ALPHA-HYDROXYLASE OX
84:00:00	3osq-A	2.1	8.8	78	607	4	MOLECULE:	MALTOSE-BINDING PERIPLASMIC PROTEIN

85:00:00	8f3h-A	2.1	5.5	70	643	3	MOLECULE:	PENICILLIN BINDING PROTEIN 5
86:00:00	7bny-B	2.1	7.8	68	140	6	MOLECULE:	GENOME POLYPROTEIN
87:00:00	4aq1-A	2.1	4.8	57	721	7	MOLECULE:	SBSB PROTEIN
88:00:00	3fid-A	2.1	6.1	82	296	2	MOLECULE:	PUTATIVE OUTER MEMBRANE PROTEIN (LP
89:00:00	3nz3-A	2.1	4.5	56	100	5	MOLECULE:	PUTATIVE UNCHARACTERIZED PROTEIN
90:00:00	3kog-A	2.1	4.7	75	226	5	MOLECULE:	PUTATIVE PORE-FORMING TOXIN
91:00:00	6hq9-A	2.1	2.9	43	60	7	MOLECULE:	DNA EXCISION REPAIR PROTEIN ERCC-6-
92:00:00	8fn9-F	2.1	3.9	55	103	5	MOLECULE:	TENASCIN-R
93:00:00	2ikk-A	2.1	4.2	58	146	12	MOLECULE:	HYPOTHETICAL TRANSCRIPTIONAL REGULA
94:00:00	5m1u-A	2.1	6.4	49	127	10	MOLECULE:	CURLI PRODUCTION ASSEMBLY/TRANSPORT
95:00:00	5mob-A	2.1	5.9	77	193	6	MOLECULE:	SLPYL1_ABA
96:00:00	6cfw-K	2.1	5.9	68	166	4	MOLECULE:	MONOVALENT CATION/H+ ANTIPORTER SUB
97:00:00	6e0a-B	2.1	4.7	68	262	3	MOLECULE:	METHYL-ACCEPTING CHEMOTAXIS PROTEIN
98:00:00	4les-A	2.1	3.1	61	142	7	MOLECULE:	PROTEIN - CONSERVED HYPOTHETICAL
99:00:00	7vgr-A	2.1	3.8	57	198	4	MOLECULE:	YN7756_1 FAB LIGHT CHAIN
0:00	5a1u-G	2.1	4.1	61	811	3	MOLECULE:	ADP-RIBOSYLATION FACTOR 1
1:00	5lth-A	2.1	3.3	64	334	5	MOLECULE:	HEME DEPENDENT OXIDATIVE N-DEMETHYL
2:00	8bl4-e	2.1	4.4	59	149	7	MOLECULE:	PHAGE TAIL SHEATH FAMILY PROTEIN
3:00	5yvq-A	2.1	4	56	358	7	MOLECULE:	TAIL FIBER PROTEIN S
4:00	7ct1-A	2.1	5.9	74	263	3	MOLECULE:	FUSION PROTEIN SORTING NEXIN-27 AND
5:00	7xyz-B	2.1	3.2	61	456	5	MOLECULE:	TRIPARTITE MOTIF-CONTAINING PROTEIN
6:00	5mje-A	2.1	5.9	45	111	4	MOLECULE:	CYTOTOXIC TRANSLATIONAL REPRESSOR O
7:00	5af0-C	2.1	6	57	239	5	MOLECULE:	MAELSTROM
8:00	7kyx-A	2.1	4.5	63	397	6	MOLECULE:	CORONIN-6
9:00	2x2h-A	2.1	4.4	65	1025	2	MOLECULE:	ALPHA-1,4-GLUCAN LYASE ISOZYME 1
10:00	7np8-B	2.1	4.1	67	620	10	MOLECULE:	COENZYME F420-DEPENDENT SULFITE RED
11:00	6zls-A	2.1	3.6	56	292	5	MOLECULE:	HISTIDINE KINASE
12:00	5u30-A	2.1	4.3	67	1085	4	MOLECULE:	CRISPR-ASSOCIATED ENDONUCLEASE C2C1
13:00	8wb2-A	2.1	2.8	57	179	4	MOLECULE:	TEMPERATURE-INDUCED LIPOCALIN-1
14:00	3j1w-A	2.1	3.9	50	106	4	MOLECULE:	PROTEIN PRGH
15:00	6ef3-A	2.1	5.5	70	247	7	MOLECULE:	PROTEASOME SUBUNIT BETA TYPE-1
16:00	2dso-C	2.1	4.6	69	323	6	MOLECULE:	DRP35
17:00	4qvs-A	2.1	9.3	60	217	7	MOLECULE:	S-LAYER DOMAIN-CONTAINING PROTEIN
18:00	5yyl-A	2.1	6.4	64	405	5	MOLECULE:	MAJOR ROYAL JELLY PROTEIN 1
19:00	5opq-A	2.1	4.3	64	659	9	MOLECULE:	3,6-ANHYDRO-D-GALACTOSIDASE
20:00	4fca-A	2.1	5.5	64	482	6	MOLECULE:	CONSERVED DOMAIN PROTEIN
21:00	3acp-A	2.1	8.6	52	417	8	MOLECULE:	WD REPEAT-CONTAINING PROTEIN YGL004
22:00	1ecg-B	2.1	14.6	75	501	11	MOLECULE:	GLUTAMINE PHOSPHORIBOSYLPYROPHOSPHA
23:00	4bq6-A	2.1	3	61	205	3	MOLECULE:	NEOGENIN
24:00:00	7dqx-E	2.1	8.7	58	293	7	MOLECULE:	6-HYDROXYPSEUDOOXYNICOTINE DEHYDROG
25:00:00	6peu-B	2.1	9.3	54	385	2	MOLECULE:	UNCHARACTERIZED PROTEIN MJ1094
26:00:00	4c2m-I	2.1	7.3	43	124	2	MOLECULE:	DNA-DIRECTED RNA POLYMERASE I SUBUN
27:00:00	5z4g-A	2.1	3.5	67	145	6	MOLECULE:	SAHS4
28:00:00	7w2t-A	2.1	7.5	73	773	4	MOLECULE:	GLUCOSYLCERAMIDASE
29:00:00	3v7d-B	2.1	3.9	47	450	4	MOLECULE:	SUPPRESSOR OF KINETOCHORE PROTEIN 1
30:00:00	8kei-D	2.1	3.6	62	164	10	MOLECULE:	CYTOCHROME B-245 LIGHT CHAIN

31:00:00	8f2r-E	2.1	3.1	46	205	13	MOLECULE:	COMM DOMAIN-CONTAINING PROTEIN 1
32:00:00	5hx2-A	2.1	6.5	57	1030	5	MOLECULE:	BASEPLATE WEDGE PROTEIN GP7
33:00:00	8e98-D	2.1	12.2	67	638	7	MOLECULE:	GLUTAMATE RECEPTOR IONOTROPIC, NMDA
34:00:00	5mc9-A	2.1	4.4	51	597	8	MOLECULE:	LAMININ SUBUNIT ALPHA-1
35:00:00	6nd4-I	2.1	4.2	49	487	0	MOLECULE:	ETS RRNA
36:00:00	2h9u-A	2.1	3	51	101	4	MOLECULE:	DNA/RNA-BINDING PROTEIN ALBA 2
37:00:00	1fwx-A	2.1	4.8	59	591	5	MOLECULE:	NITROUS OXIDE REDUCTASE
38:00:00	3b77-A	2.1	7.4	60	188	12	MOLECULE:	UNCHARACTERIZED PROTEIN
39:00:00	4jmh-A	2.1	2.6	47	200	6	MOLECULE:	CLAMP SHC1_PY239/240
40:00:00	2x5h-D	2.1	3.6	45	94	7	MOLECULE:	ORF 131
41:00:00	8bn0-A	2.1	6.6	62	342	0	MOLECULE:	PUTATIVE SURFACE LAYER PROTEIN
42:00:00	6nd4-J	2.1	3.7	69	493	9	MOLECULE:	ETS RRNA
43:00:00	6il9-A	2.1	3	43	490	7	MOLECULE:	FRUCTURONATE-TAGATURONATE EPIMERASE
44:00:00	5oj3-A	2.1	5.3	55	322	4	MOLECULE:	PHOTOSYSTEM II STABILITY/ASSEMBLY F
45:00:00	8p4k-A	2.1	3.9	47	599	2	MOLECULE:	CORE PROTEIN OPG136
46:00:00	6rtv-A	2.1	8.2	49	386	8	MOLECULE:	4-O-METHYL-GLUCURONOYL METHYLESTERA
47:00:00	4wju-A	2.1	5.8	62	457	3	MOLECULE:	RIBOSOME ASSEMBLY PROTEIN 4
48:00:00	5i08-A	2.1	6.2	66	958	9	MOLECULE:	SPIKE GLYCOPROTEIN, ENVELOPE GLYCOP
49:00:00	5c0x-K	2.1	6.4	55	350	7	MOLECULE:	EXOSOME COMPLEX COMPONENT RRP45
50:00:00	1g6o-A	2.1	4.9	49	323	8	MOLECULE:	CAG-ALPHA
51:00:00	4kha-A	2.1	7.4	53	360	4	MOLECULE:	SPT16M-HISTONE H2B 1.1 CHIMERA
52:00:00	5cvo-D	2.1	4.7	54	612	2	MOLECULE:	WD REPEAT-CONTAINING PROTEIN 48
53:00:00	6rte-A	2.1	3.5	56	464	7	MOLECULE:	CYTOCHROME C
54:00:00	7wj7-A	2.1	10	57	242	11	MOLECULE:	SERINE/THREONINE PROTEIN KINASE
55:00:00	8y6b-D	2.1	4.7	68	517	4	MOLECULE:	DISINTEGRIN AND METALLOPROTEINASE D
56:00:00	6muq-A	2.1	4.3	64	267	8	MOLECULE:	MUREIN-DD-ENDOPEPTIDASE
57:00:00	6gkw-A	2.1	4.5	50	351	4	MOLECULE:	PUTATIVE PHAGE XKDK-LIKE PROTEIN
58:00:00	5o60-H	2.1	2.8	48	151	2	MOLECULE:	50S RIBOSOMAL PROTEIN BL37
59:00:00	8ide-A	2.1	6.4	46	929	4	MOLECULE:	N(6)-L-THREONYLCARBAMOYLADENINE SYN
60:00:00	8v1k-A	2.1	5.5	72	185	3	MOLECULE:	OUTER-MEMBRANE LIPOPROTEIN CARRIER
61:00:00	7s69-A	2.1	4.4	52	174	6	MOLECULE:	N-ACETYLGLUCOSAMINE-1-PHOSPHOTRANSF
62:00:00	5uc2-C	2.1	4	56	242	0	MOLECULE:	DOMAIN OF UNKNOWN FUNCTION DUF1849
63:00:00	2hqv-A	2.1	8	56	172	2	MOLECULE:	AGR_C_4470P
64:00:00	8h8p-B	2.1	4.9	80	355	5	MOLECULE:	THIOMORPHOLINE-CARBOXYLATE DEHYDROG
65:00:00	6h41-A	2.1	4.7	69	309	4	MOLECULE:	INTERLEUKIN-5 RECEPTOR SUBUNIT ALPH
66:00:00	6sno-A	2.1	5.7	66	573	5	MOLECULE:	PHOSPHOGLUCOMUTASE-1
67:00:00	8k2v-A	2.1	9.3	56	214	5	MOLECULE:	METHYLCROTONOYL-COA CARBOXYLASE SUB
68:00:00	6cp9-D	2.1	4.7	57	116	12	MOLECULE:	CDIA
69:00:00	7w42-B	2.1	10.7	61	402	8	MOLECULE:	UNCHARACTERIZED ATPASE YJOB
70:00:00	7krj-C	2.1	8.8	50	133	10	MOLECULE:	HEAT SHOCK PROTEIN HSP 90-ALPHA
71:00:00	5w5p-A	2.1	7.3	92	610	4	MOLECULE:	TAIL FIBER PROTEIN
72:00:00	6ahr-F	2.1	5.2	59	129	7	MOLECULE:	H1 RNA
73:00:00	5owg-A	2.1	4.4	56	181	5	MOLECULE:	PCYX_EBK42635
74:00:00	7kzn-D	2.1	4.3	65	456	6	MOLECULE:	HEAVY CHAIN ALPHA
75:00:00	8acs-A	2.1	6.4	65	598	14	MOLECULE:	FAD-DEPENDENT OXIDOREDUCTASE
76:00:00	5ltw-D	2.1	2.4	44	98	7	MOLECULE:	14-3-3 PROTEIN SIGMA

77:00:00	1w94-A	2.1	3.2	48	155	6	MOLECULE:	PROBABLE BRIX-DOMAIN RIBOSOMAL BIOG
78:00:00	5ejr-A	2.1	4.7	58	500	3	MOLECULE:	MYOSIN-I HEAVY CHAIN
79:00:00	5ek5-B	2.1	2.5	38	120	5	MOLECULE:	IRMA
80:00:00	7apk-F	2.1	5.3	67	337	0	MOLECULE:	THO COMPLEX SUBUNIT 1
81:00:00	6fhm-A	2.1	6.9	58	193	9	MOLECULE:	OUTER-MEMBRANE LIPOPROTEIN CARRIER
82:00:00	1qqg-B	2.1	6.2	56	210	5	MOLECULE:	INSULIN RECEPTOR SUBSTRATE 1
83:00:00	7et0-C	2.1	5.4	66	684	3	MOLECULE:	BACTERIA FACTOR B
84:00:00	8orn-B	2.1	6.6	57	180	7	MOLECULE:	OUTER-MEMBRANE LIPOPROTEIN CARRIER
85:00:00	6jt5-A	2.1	7.5	57	409	4	MOLECULE:	EXTRACELLULAR PQQ-DEPENDENT SUGAR D
86:00:00	6y9c-A	2.1	3.4	60	365	8	MOLECULE:	CARNITINE MONOOXYGENASE OXYGENASE S
87:00:00	5ezu-A	2.1	3	47	76	4	MOLECULE:	PROTEIN A46
88:00:00	1x1g-A	2.1	3.8	53	129	8	MOLECULE:	PLECKSTRIN 2
89:00:00	3ejv-A	2.1	6	65	159	2	MOLECULE:	UNCHARACTERIZED PROTEIN WITH CYSTAT
90:00:00	8bl3-A	2.1	4.7	65	141	3	MOLECULE:	SCIG12
91:00:00	3zfo-A	2.1	3.6	53	150	2	MOLECULE:	N-TERMINAL PROTEASE NPRO
92:00:00	6wqc-A	2.1	5	54	293	7	MOLECULE:	N-TERMINAL ACETYLTRANSFERASE, GNAT
93:00:00	6yzg-A	2.1	4.6	58	279	2	MOLECULE:	SURFACE-ASSOCIATED PROTEIN CSHB
94:00:00	5gt1-A	2.1	3.3	46	153	9	MOLECULE:	CHOLINE BINDING PROTEIN A
95:00:00	1m1g-C	2.1	3.8	50	244	2	MOLECULE:	TRANSCRIPTION ANTITERMINATION PROTE
96:00:00	7lt1-A	2.1	8	62	403	3	MOLECULE:	PROTEIN MB21D2
97:00:00	6ffy-A	2.1	6.8	56	918	2	MOLECULE:	VPS10 DOMAIN-CONTAINING RECEPTOR SO
98:00:00	4tqj-A	2.1	6.8	61	403	8	MOLECULE:	LECTIN 2
99:00:00	7nnl-B	2.1	5	65	682	11	MOLECULE:	POTASSIUM-TRANSPORTING ATPASE POTAS
0:00	8oml-B	2.1	4.1	66	317	0	MOLECULE:	CELL WALL SURFACE ANCHOR FAMILY PRO
1:00	7f10-B	2.1	9.5	68	140	4	MOLECULE:	NSRQ
2:00	2w2c-A	2.1	5.2	61	128	7	MOLECULE:	CALCIUM/CALMODULIN-DEPENDENT PROTEI
3:00	7f0y-A	2.1	9.6	67	141	3	MOLECULE:	NSRQ
4:00	3ff0-A	2.1	6.7	76	162	4	MOLECULE:	PHENAZINE BIOSYNTHESIS PROTEIN PHZB
5:00	4y4v-B	2.1	5.1	70	317	10	MOLECULE:	CONSERVED HYPOTHETICAL SECRETED PRO
6:00	8jxd-A	2.1	6.2	62	493	2	MOLECULE:	LDL RECEPTOR RELATED PROTEIN 2
7:00	4x2h-B	2.1	5.6	69	175	12	MOLECULE:	PUTATIVE MRNA EXPORT PROTEIN
8:00	5yzn-A	2.1	4.9	57	649	7	MOLECULE:	ACYL-PEPTIDE HYDROLASE, PUTATIVE
9:00	7f14-A	2.1	9.3	70	148	4	MOLECULE:	DCR3
10:00	5evh-A	2.1	6.8	64	121	3	MOLECULE:	UNCHARACTERIZED PROTEIN
11:00	6swy-4	2.1	4.6	68	217	4	MOLECULE:	VACUOLAR IMPORT AND DEGRADATION PRO
12:00	8ovn-A	2.1	9.6	78	503	5	MOLECULE:	PUTATIVE PERIPLASMIC BINDING TRANSP
13:00	6vio-D	2.1	6.5	59	225	3	MOLECULE:	GREEN FLUORESCENT PROTEIN GFP
14:00	3evu-A	2.1	12.5	79	393	5	MOLECULE:	MYOSIN LIGHT CHAIN KINASE, GREEN FL
15:00	4w7e-A	2.1	6.8	59	221	3	MOLECULE:	FLUORESCENT PROTEIN D21H/K26H
16:00	4bdu-B	2.1	7.8	63	295	3	MOLECULE:	GREEN FLUORESCENT PROTEIN, APOPTOSI
17:00	3ek7-A	2.1	12.3	79	389	6	MOLECULE:	MYOSIN LIGHT CHAIN KINASE, GREEN FL
18:00	5dpi-D	2.1	5.9	56	225	4	MOLECULE:	GREEN FLUORESCENT PROTEIN
19:00	3sg5-A	2.1	12.3	78	387	5	MOLECULE:	MYOSIN LIGHT CHAIN KINASE, GREEN FL
20:00	6uru-B	2.1	8.8	74	500	4	MOLECULE:	IACHSNFR PRECURSOR
21:00	3sg6-A	2.1	12.1	78	383	4	MOLECULE:	MYOSIN LIGHT CHAIN KINASE, GREEN FL
22:00	7bwn-M	2.1	8.4	55	280	5	MOLECULE:	CHIMERA OF GREEN FLUORESCENT PROTEI

23:00	7bwn-J	2.1	10	57	280	5	MOLECULE:	CHIMERA OF GREEN FLUORESCENT PROTEI
24:00:00	6wva-A	2.1	6.8	68	411	3	MOLECULE:	VITAMIN K EPOXIDE REDUCTASE-LIKE PR
25:00:00	3k1k-B	2.1	6.4	58	224	3	MOLECULE:	GREEN FLUORESCENT PROTEIN
26:00:00	8j3j-A	2.1	8.6	57	214	9	MOLECULE:	STAYGOLD(Q140S, Y187F)
27:00:00	6efr-A	2.1	9.3	78	503	4	MOLECULE:	INICSNFR 1.0, A GENETICALLY ENCODED
28:00:00	4ik9-A	2.1	12.3	78	393	5	MOLECULE:	RCAMP, GREEN FLUORESCENT PROTEIN
29:00:00	8ovp-B	2.1	6.1	74	497	4	MOLECULE:	PUTATIVE PERIPLASMIC BINDING TRANSP
30:00:00	3sg3-A	2.1	7.1	78	394	4	MOLECULE:	MYOSIN LIGHT CHAIN KINASE, GREEN FL
31:00:00	4ik4-A	2.1	13.3	74	395	7	MOLECULE:	RCAMP, GREEN FLUORESCENT PROTEIN
32:00:00	6zsn-A	2.1	7	78	397	4	MOLECULE:	GREEN FLUORESCENT PROTEIN,GREEN FLU
33:00:00	7st4-A	2.1	7.8	79	385	5	MOLECULE:	JGCAMP8.410.80
34:00:00	4zf4-A	2.1	4.7	71	226	10	MOLECULE:	GREEN FLUORESCENT PROTEIN
35:00:00	2wiq-A	2.1	10	57	225	9	MOLECULE:	KILLERRED
36:00:00	7amf-C	2.1	11.2	72	234	7	MOLECULE:	GREEN FLUORESCENT PROTEIN
37:00:00	3vic-D	2.1	9.4	55	218	2	MOLECULE:	GFP-LIKE NON-FLUORESCENT CHROMOPROT
38:00:00	4ndj-A	2.1	11.6	47	279	9	MOLECULE:	GREEN FLUORESCENT PROTEIN, CHIMERIC
39:00:00	8bxt-B	2.1	7.1	48	213	6	MOLECULE:	STAYGOLD
40:00:00	7rrh-F	2.1	10.2	63	213	2	MOLECULE:	FLUORESCENT PROTEIN DRONPA
41:00:00	7bwn-C	2.1	8.2	55	280	5	MOLECULE:	CHIMERA OF GREEN FLUORESCENT PROTEI
42:00:00	4w7r-D	2.1	8.8	57	225	9	MOLECULE:	FLUORESCENT PROTEIN E124H/K126C
43:00:00	2ote-B	2.1	11.8	49	213	6	MOLECULE:	GFP-LIKE FLUORESCENT CHROMOPROTEIN
44:00:00	3cgl-E	2.1	10.4	65	212	0	MOLECULE:	GFP-LIKE FLUORESCENT CHROMOPROTEIN
45:00:00	7rri-D	2.1	10.3	65	219	2	MOLECULE:	FLUORESCENT PROTEIN DRONPA
46:00:00	5mfc-D	2.1	8.9	58	237	5	MOLECULE:	YIIIM5AII
47:00:00	6ofo-A	2.1	8.9	61	220	3	MOLECULE:	GREEN FLUORESCENT PROTEIN (GFP) S1
48:00:00	5hzt-I	2.1	11.6	51	212	6	MOLECULE:	FLUORESCENT PROTEIN DRONPA
49:00:00	7o7c-A	2.1	10.7	71	232	3	MOLECULE:	GREEN FLUORESCENT PROTEIN
50:00:00	4w6k-A	2.1	10.9	47	194	11	MOLECULE:	FLUORESCENT PROTEIN D117C
51:00:00	2oiw-A	2	3	58	133	7	MOLECULE:	PUTATIVE 4-HYDROXYBENZOYL-COA THIOE
52:00:00	6uhd-A	2	13.4	86	322	2	MOLECULE:	HISTONE ACETYLTRANSFERASE TYPE B CA
53:00:00	7v9a-B	2	4.5	68	377	4	MOLECULE:	TELOMERASE CAJAL BODY PROTEIN 1
54:00:00	2hq7-B	2	8.6	64	142	9	MOLECULE:	PROTEIN, RELATED TO GENERAL STRESS
55:00:00	1a87-A	2	4.1	54	297	6	MOLECULE:	COLICIN N
56:00:00	6lix-B	2	11	69	196	7	MOLECULE:	CHROMOPHORE LYASE CRL, CHLOROPLASTI
57:00:00	6shc-A	2	5.7	62	273	8	MOLECULE:	SERINE/THREONINE-PROTEIN KINASE/END
58:00:00	2yrl-A	2	4.3	55	102	5	MOLECULE:	KIAA1837 PROTEIN
59:00:00	4lmi-B	2	5.5	62	136	8	MOLECULE:	UNCHARACTERIZED PROTEIN
60:00:00	4ge1-A	2	3.8	68	195	10	MOLECULE:	BIOGENIC AMINE-BINDING PROTEIN
61:00:00	8ok9-C	2	5.6	65	236	9	MOLECULE:	PIWI PROTEIN AF_1318
62:00:00	3jvn-A	2	3.6	54	122	4	MOLECULE:	ACETYLTRANSFERASE
63:00:00	3w81-A	2	10.3	76	616	5	MOLECULE:	ALPHA-L-IDURONIDASE
64:00:00	5oj2-B	2	2.6	47	692	9	MOLECULE:	MAM DOMAIN-CONTAINING GLYCOSYLPHOSP
65:00:00	5hdf-B	2	8.7	46	320	9	MOLECULE:	HYDROLASE
66:00:00	4v3g-A	2	11	90	330	3	MOLECULE:	CYMA PROTEIN
67:00:00	2dc1-A	2	4.5	62	236	3	MOLECULE:	L-ASPARTATE DEHYDROGENASE
68:00:00	2qml-A	2	4.4	59	193	3	MOLECULE:	BH2621 PROTEIN

69:00:00	6dv3-A	2	7.3	77	500	4	MOLECULE:	PROTEIN INVG
70:00:00	2qp2-A	2	8.1	73	498	10	MOLECULE:	UNKNOWN PROTEIN
71:00:00	2r41-C	2	2.8	55	106	9	MOLECULE:	UNCHARACTERIZED PROTEIN
72:00:00	3ubg-B	2	4.1	52	310	6	MOLECULE:	NEURAL-CADHERIN
73:00:00	6gef-A	2	6.9	60	388	12	MOLECULE:	TYPE IV SECRETION SYSTEM PROTEIN DO
74:00:00	2mpo-A	2	4.6	75	182	9	MOLECULE:	MIC2-ASSOCIATED PROTEIN
75:00:00	7cun-G	2	4	64	895	6	MOLECULE:	INTEGRATOR COMPLEX SUBUNIT 1
76:00:00	3c5m-B	2	9	65	378	3	MOLECULE:	OLIGOGALACTURONATE LYASE
77:00:00	7qof-d	2	4.1	56	333	2	MOLECULE:	MAJOR CAPSID PROTEIN GP32
78:00:00	7nky-O	2	1.9	42	427	10	MOLECULE:	RNA (5'-
79:00:00	7s8v-B	2	8.6	75	518	3	MOLECULE:	INSULIN-LIKE GROWTH FACTOR 1 RECEPT
80:00:00	8as8-A	2	10	70	597	4	MOLECULE:	JETC
81:00:00	6e5d-A	2	5.6	59	173	5	MOLECULE:	LIPID BINDING PROTEIN LPQN
82:00:00	2fkc-A	2	4.2	65	247	6	MOLECULE:	5'-D(*CP*CP*AP*GP*CP*GP*CP*TP*GP*G)
83:00:00	5n83-C	2	6.9	69	196	0	MOLECULE:	FIBER
84:00:00	7e2c-I	2	9.6	64	799	6	MOLECULE:	TRAPP-ASSOCIATED PROTEIN TCA17
85:00:00	4kh6-B	2	5	58	596	7	MOLECULE:	NUCLEOSIDE-TRIPHOSPHATASE 2
86:00:00	5nbs-A	2	8.1	62	842	5	MOLECULE:	BETA-GLUCOSIDASE
87:00:00	3e8w-A	2	7.5	82	219	5	MOLECULE:	TAKEOUT-LIKE PROTEIN 1
88:00:00	8fbe-A	2	4.5	73	144	3	MOLECULE:	NEUROTOXIN COMPLEX COMPONENT ORF-X1
89:00:00	4m8r-A	2	3.9	64	406	8	MOLECULE:	HYPOTHETICAL PROTEIN
90:00:00	2d7v-A	2	3.5	60	155	13	MOLECULE:	HYPOTHETICAL PROTEIN VCA0330
91:00:00	8i5f-A	2	8.3	62	433	2	MOLECULE:	DEDICATOR OF CYTOKINESIS PROTEIN 10
92:00:00	4r1k-B	2	4.8	58	136	2	MOLECULE:	UNCHARACTERIZED PROTEIN
93:00:00	3fh1-A	2	3.4	56	122	2	MOLECULE:	UNCHARACTERIZED NTF2-LIKE PROTEIN
94:00:00	6uf2-A	2	3.1	50	125	6	MOLECULE:	BIOFILM-RELATED PROTEIN
95:00:00	2eab-A	2	7.4	66	888	6	MOLECULE:	ALPHA-FUCOSIDASE
96:00:00	6v4n-M	2	4.6	69	391	1	MOLECULE:	NEURAMINIDASE
97:00:00	6f3h-B	2	10.2	69	790	4	MOLECULE:	EXORIBONUCLEASE II, MITOCHONDRIAL
98:00:00	6k9k-A	2	9.5	70	2501	4	MOLECULE:	SERINE-PROTEIN KINASE ATM
99:00:00	5aor-A	2	19.6	66	1009	3	MOLECULE:	DOSAGE COMPENSATION REGULATOR
0:00	3nqn-A	2	6.3	58	151	10	MOLECULE:	UNCHARACTERIZED PROTEIN
1:00	7vgh-B	2	4.3	73	1182	7	MOLECULE:	CELL CYCLE CONTROL PROTEIN 50B
2:00	6j7n-A	2	7.4	64	290	5	MOLECULE:	GUANYLYLTRANSFERASE-LIKE TOXIN
3:00	1jeq-A	2	5.7	68	548	9	MOLECULE:	KU70
4:00	6u4k-A	2	3.8	52	391	10	MOLECULE:	TALIN-2
5:00	2crf-A	2	4.9	67	150	6	MOLECULE:	RAN BINDING PROTEIN 3
6:00	4ruq-B	2	5	60	237	0	MOLECULE:	FISH-EGG LECTIN
7:00	4l1n-A	2	5.1	55	161	4	MOLECULE:	CONSERVED LIPOPROTEIN, PUTATIVE
8:00	5dcj-A	2	6.9	50	118	4	MOLECULE:	OSMOLARITY SENSOR PROTEIN ENVZ
9:00	8ck1-A	2	9.7	51	826	4	MOLECULE:	TAIL NOZZLE
10:00	2fg9-A	2	5.9	63	166	6	MOLECULE:	5-NITROIMIDAZOLE ANTIBIOTIC RESISTA
11:00	2m89-A	2	3.7	63	134	5	MOLECULE:	AHA1 DOMAIN PROTEIN
12:00	3c7g-A	2	5.2	74	488	7	MOLECULE:	ENDO-1,4-BETA-XYLANASE
13:00	4k90-B	2	5.2	62	207	11	MOLECULE:	EXTRACELLULAR METALLOPROTEINASE MEP
14:00	1d09-B	2	3.8	56	153	4	MOLECULE:	ASPARTATE CARBAMOYLTRANSFERASE CATA

15:00	5dfz-C	2	6.8	65	783	8	MOLECULE:	VACUOLAR PROTEIN SORTING-ASSOCIATED
16:00	4std-A	2	5.9	68	164	6	MOLECULE:	SCYTALONE DEHYDRATASE
17:00	5hww-A	2	4.5	54	128	9	MOLECULE:	SENSOR HISTIDINE KINASE TODS
18:00	4qxa-B	2	6.5	56	147	7	MOLECULE:	RAS-RELATED PROTEIN RAB-9A
19:00	3tvv-B	2	4.3	51	235	2	MOLECULE:	HISTONE CHAPERONE RTT106
20:00	5csk-A	2	5.6	49	1996	4	MOLECULE:	ACETYL-COA CARBOXYLASE
21:00	8owu-E	2	3	46	115	4	MOLECULE:	TRANSPOSASE
22:00	8t1l-A	2	6.7	66	472	6	MOLECULE:	MEDIATOR OF RNA POLYMERASE II TRANS
23:00	5txc-A	2	5.1	74	653	9	MOLECULE:	ATXE2
24:00:00	5d9b-A	2	6.8	61	307	8	MOLECULE:	LUCIFERIN REGENERATING ENZYME
25:00:00	5b2g-C	2	5.5	50	350	8	MOLECULE:	ENDOLYSIN,CLAUDIN-4
26:00:00	8adl-A	2	4.4	64	633	5	MOLECULE:	MTC5 ISOFORM 1
27:00:00	8a43-A	2	2.9	49	1549	8	MOLECULE:	DNA-DIRECTED RNA POLYMERASE I SUBUN
28:00:00	7s3l-A	2	3.6	42	254	5	MOLECULE:	AMINOGLYCOSIDE PHOSPHOTRANSFERASE F
29:00:00	8usw-B	2	13	72	745	3	MOLECULE:	GLUTAMATE RECEPTOR IONOTROPIC, NMDA
30:00:00	5xmz-A	2	4.9	64	122	8	MOLECULE:	EFFECTOR PROTEIN PEVD1
31:00:00	2pcs-A	2	5.1	70	152	4	MOLECULE:	CONSERVED PROTEIN
32:00:00	6od1-B	2	3.4	54	106	4	MOLECULE:	REGULATOR OF RPOS
33:00:00	2ow6-A	2	8.6	75	1016	3	MOLECULE:	ALPHA-MANNOSIDASE 2
34:00:00	3tkn-A	2	5	48	451	10	MOLECULE:	NUCLEOPORIN NUP82
35:00:00	7jrl-A	2	3.9	57	457	5	MOLECULE:	F5/8 TYPE C DOMAIN PROTEIN
36:00:00	8a3t-B	2	4.7	58	440	10	MOLECULE:	ANAPHASE-PROMOTING COMPLEX SUBUNIT
37:00:00	9b21-A	2	7.9	49	205	0	MOLECULE:	ADP-RIBOSE PYROPHOSPHATASE
38:00:00	6qvf-B	2	3.4	62	173	6	MOLECULE:	PREPILIN-LIKE PROTEIN
39:00:00	8ae6-W	2	3.7	49	941	8	MOLECULE:	NITROGEN PERMEASE REGULATOR 3
40:00:00	4yu6-A	2	12.1	81	754	9	MOLECULE:	IMMUNE INHIBITOR A, METALLOPROTEASE
41:00:00	1ofd-A	2	7.2	64	1491	8	MOLECULE:	FERREDOXIN-DEPENDENT GLUTAMATE SYNT
42:00:00	6do4-A	2	4.9	43	319	7	MOLECULE:	KELCH DOMAIN-CONTAINING PROTEIN 2
43:00:00	5wqh-F	2	6.9	74	140	7	MOLECULE:	ISOMERASE TRT14
44:00:00	7z4f-G	2	5.5	69	922	3	MOLECULE:	PUTATIVE STRUCTURAL PROTEIN
45:00:00	3wj9-B	2	7.3	64	402	9	MOLECULE:	EUKARYOTIC TRANSLATION INITIATION F
46:00:00	7aed-A	2	4.3	55	128	5	MOLECULE:	PRGL
47:00:00	7f13-B	2	9.2	70	148	4	MOLECULE:	DCR3
48:00:00	6xzf-A	2	6	57	225	4	MOLECULE:	EGFP
49:00:00	2whs-D	2	8.9	55	226	4	MOLECULE:	LARGE STOKES SHIFT FLUORESCENT PROT
50:00:00	6nqs-D	2	4.3	59	214	8	MOLECULE:	FLUORESCENT PROTEIN DRONPA
51:00:00	4w75-A	2	12.2	54	209	2	MOLECULE:	FLUORESCENT PROTEIN D21H/K26C
52:00:00	3ekj-A	2	11.4	74	301	4	MOLECULE:	MYOSIN LIGHT CHAIN KINASE, GREEN FL
53:00:00	4pfe-B	2	9.4	60	340	3	MOLECULE:	GREEN FLUORESCENT PROTEIN
54:00:00	6tv7-A	2	7	78	397	4	MOLECULE:	RSGCAMP
55:00:00	4w6u-C	2	10.1	60	221	7	MOLECULE:	FLUORESCENT PROTEIN E115H/T118H
56:00:00	4dxp-A	2	10.4	65	215	2	MOLECULE:	LEA X121 GFP-LIKE PROTEINS
57:00:00	5eb7-A	2	9.7	63	213	2	MOLECULE:	REVERSIBLY PHOTOSWITCHING CHROMOPRO
58:00:00	7a8i-D	2	11	58	224	5	MOLECULE:	GREEN FLUORESCENT PROTEIN

DALI results: MdA-1 Col

No:	Chain	Z	rmsd	lali	nres	%id	PDB Description
1:00	s001A	7.6	62.4	215	0	0	

DALI results: MdA-1 Colalt

No:	Chain	Z	rmsd	lali	nres	%id	PDB	Description
1:00	8th8-s	6.3	11	116	187	9	MOLECULE:	DYNEIN REGULATORY COMPLEX PROTEIN 1/2 N-TERMINAL
2:00	6j9r-B	6.1	4.2	98	126	8	MOLECULE:	BRAIN TUMOR PROTEIN
3:00	7wxz-D	6	6.5	90	187	14	MOLECULE:	SPIKE PROTEIN S2'
4:00	8ga8-G	6	3.5	78	97	5	MOLECULE:	TRANSCRIPTIONAL REGULATORY PROTEIN SDS3
5:00	8fxf-A	6	2.1	78	78	10	MOLECULE:	E3 UBIQUITIN-PROTEIN LIGASE TRIM56
6:00	1pl5-S	6	3	76	76	11	MOLECULE:	REGULATORY PROTEIN SIR4
7:00	5fiy-C	6	3.6	78	78	8	MOLECULE:	PRKC APOPTOSIS WT1 REGULATOR PROTEIN
8:00	6hk9-B	5.9	2.3	78	78	5	MOLECULE:	TESTIS-EXPRESSED PROTEIN 12
9:00	3vp8-D	5.9	2.5	78	78	5	MOLECULE:	GENERAL TRANSCRIPTIONAL COREPRESSOR TUP1
10:00	3vp8-C	5.9	2.1	77	77	6	MOLECULE:	GENERAL TRANSCRIPTIONAL COREPRESSOR TUP1
11:00	8sjj-A	5.9	2.9	74	74	5	MOLECULE:	SEPTIN-14
12:00	8sjj-B	5.9	3.3	74	74	5	MOLECULE:	SEPTIN-14
13:00	8t9d-D	5.9	6.5	87	161	8	MOLECULE:	MEDIATOR OF RNA POLYMERASE II TRANSCRIPTION SUBUN
14:00	8czi-A	5.9	2.2	71	71	11	MOLECULE:	SCAFFOLDED SPIKE PROTEIN S2' HR1
15:00	8fcf-D	5.9	3.6	79	79	4	MOLECULE:	PLASMALEMMA VESICLE-ASSOCIATED PROTEIN
16:00	8fxf-D	5.9	2.1	78	78	10	MOLECULE:	E3 UBIQUITIN-PROTEIN LIGASE TRIM56
17:00	8tgt-A	5.9	1.6	76	76	8	MOLECULE:	M PROTEIN
18:00	8czi-C	5.9	2.2	71	71	11	MOLECULE:	SCAFFOLDED SPIKE PROTEIN S2' HR1
19:00	8afh-P	5.9	4.1	79	79	14	MOLECULE:	CRESCENTIN
20:00	8fxf-B	5.9	2	78	78	10	MOLECULE:	E3 UBIQUITIN-PROTEIN LIGASE TRIM56
21:00	1pl5-A	5.9	2.9	75	75	11	MOLECULE:	REGULATORY PROTEIN SIR4
22:00	8afe-F	5.9	4.1	79	79	14	MOLECULE:	CRESCENTIN
23:00	8afh-F	5.9	4.1	79	79	14	MOLECULE:	CRESCENTIN
24:00:00	8csp-U	5.9	9.8	98	174	6	MOLECULE:	28S RIBOSOMAL PROTEIN S34, MITOCHONDRIAL
25:00:00	1gk4-D	5.9	3.6	78	78	8	MOLECULE:	VIMENTIN
26:00:00	1gk4-B	5.9	4.2	79	79	5	MOLECULE:	VIMENTIN
27:00:00	2bez-C	5.9	2.5	77	77	9	MOLECULE:	E2 GLYCOPROTEIN
28:00:00	5jxc-C	5.9	3.5	78	78	10	MOLECULE:	RAS/RAP GTPASE-ACTIVATING PROTEIN SYNGAP
29:00:00	1n7s-C	5.9	5.4	79	79	8	MOLECULE:	VESICLE-ASSOCIATED MEMBRANE PROTEIN 2
30:00:00	5djn-A	5.9	2.2	77	77	10	MOLECULE:	KINESIN-LIKE PROTEIN
31:00:00	1gk4-A	5.9	3	78	79	8	MOLECULE:	VIMENTIN
32:00:00	6aoz-A	5.9	3.8	74	74	7	MOLECULE:	CASP8-ASSOCIATED PROTEIN 2
33:00:00	8x7z-A	5.8	3.6	81	83	12	MOLECULE:	HR1
34:00:00	8v0y-G	5.8	3.3	81	82	5	MOLECULE:	ACTIN, ALPHA CARDIAC MUSCLE 1
35:00:00	8t1i-B	5.8	7.6	82	158	9	MOLECULE:	MEDIATOR OF RNA POLYMERASE II TRANSCRIPTION SUBUN
36:00:00	1nyh-A	5.8	2.9	76	76	11	MOLECULE:	REGULATORY PROTEIN SIR4
37:00:00	7tik-B	5.8	1.7	71	71	10	MOLECULE:	FERRITIN, DPS FAMILY PROTEIN AND SPIKE PROTEIN S2
38:00:00	8fa2-A	5.8	2.2	71	71	10	MOLECULE:	SCAFFOLDED SPIKE PROTEIN S2' HR1
39:00:00	8fxf-C	5.8	2	78	78	10	MOLECULE:	E3 UBIQUITIN-PROTEIN LIGASE TRIM56
40:00:00	8fa1-C	5.8	1.8	71	71	10	MOLECULE:	FERRITIN, DPS FAMILY PROTEIN AND SPIKE PROTEIN S2
41:00:00	8fa2-C	5.8	2.2	71	71	10	MOLECULE:	SCAFFOLDED SPIKE PROTEIN S2' HR1
42:00:00	8fa1-A	5.8	1.8	71	71	10	MOLECULE:	FERRITIN, DPS FAMILY PROTEIN AND SPIKE PROTEIN S2
43:00:00	8czi-B	5.8	2.2	71	71	11	MOLECULE:	SCAFFOLDED SPIKE PROTEIN S2' HR1
44:00:00	7tik-C	5.8	1.7	71	71	10	MOLECULE:	FERRITIN, DPS FAMILY PROTEIN AND SPIKE PROTEIN S2
45:00:00	8fa1-B	5.8	1.8	71	71	10	MOLECULE:	FERRITIN, DPS FAMILY PROTEIN AND SPIKE PROTEIN S2
46:00:00	8sdi-A	5.8	1.9	80	80	6	MOLECULE:	TRIPARTITE MOTIF-CONTAINING PROTEIN 45
47:00:00	8fa2-B	5.8	2.2	71	71	10	MOLECULE:	SCAFFOLDED SPIKE PROTEIN S2' HR1
48:00:00	7z44-A	5.8	6.9	100	598	5	MOLECULE:	PORTAL PROTEIN
49:00:00	7tik-A	5.8	1.7	71	71	10	MOLECULE:	FERRITIN, DPS FAMILY PROTEIN AND SPIKE PROTEIN S2
50:00:00	6h9l-B	5.8	7.5	93	129	4	MOLECULE:	UNCHARACTERIZED PROTEIN
51:00:00	8sdi-D	5.8	1.9	80	80	6	MOLECULE:	TRIPARTITE MOTIF-CONTAINING PROTEIN 45
52:00:00	2wps-B	5.8	5.2	86	98	10	MOLECULE:	TRIMERIC AUTOTRANSPORTER ADHESIN FRAGMENT
53:00:00	2wps-C	5.8	5.2	86	98	9	MOLECULE:	TRIMERIC AUTOTRANSPORTER ADHESIN FRAGMENT
54:00:00	3vp8-A	5.8	2.3	75	75	8	MOLECULE:	GENERAL TRANSCRIPTIONAL COREPRESSOR TUP1

55:00:00	5djn-C	5.8	4	78	81	10	MOLECULE:	KINESIN-LIKE PROTEIN
56:00:00	3fwc-N	5.8	3.1	82	84	5	MOLECULE:	CELL DIVISION CONTROL PROTEIN 31
57:00:00	3fwc-J	5.8	1.3	77	78	5	MOLECULE:	CELL DIVISION CONTROL PROTEIN 31
58:00:00	1x8y-A	5.8	3.7	74	74	5	MOLECULE:	LAMIN A/C
59:00:00	7apk-m	5.7	3.5	71	549	3	MOLECULE:	THO COMPLEX SUBUNIT 1
60:00:00	3iv1-A	5.7	2.9	76	78	5	MOLECULE:	TUMOR SUSCEPTIBILITY GENE 101 PROTEIN
61:00:00	6eun-A	5.7	6.8	89	147	8	MOLECULE:	ADHESIN
62:00:00	3vp8-B	5.7	2.8	79	80	6	MOLECULE:	GENERAL TRANSCRIPTIONAL COREPRESSOR TUP1
63:00:00	2jee-A	5.7	2.7	77	78	8	MOLECULE:	CELL DIVISION PROTEIN ZAPB
64:00:00	3iv1-E	5.7	2.9	76	78	5	MOLECULE:	TUMOR SUSCEPTIBILITY GENE 101 PROTEIN
65:00:00	3iv1-F	5.7	3.3	77	78	5	MOLECULE:	TUMOR SUSCEPTIBILITY GENE 101 PROTEIN
66:00:00	3iv1-B	5.7	2.9	76	78	5	MOLECULE:	TUMOR SUSCEPTIBILITY GENE 101 PROTEIN
67:00:00	3iv1-H	5.7	3.1	77	78	5	MOLECULE:	TUMOR SUSCEPTIBILITY GENE 101 PROTEIN
68:00:00	2jee-D	5.7	2.7	77	78	8	MOLECULE:	CELL DIVISION PROTEIN ZAPB
69:00:00	7x2e-A	5.7	6.1	94	163	6	MOLECULE:	HARMONIN
70:00:00	2jee-B	5.7	2.7	77	78	8	MOLECULE:	CELL DIVISION PROTEIN ZAPB
71:00:00	7say-B	5.7	2.6	70	70	6	MOLECULE:	GENERAL CONTROL TRANSCRIPTION FACTOR GCN4/M PROTE
72:00:00	8v0i-F	5.7	4.6	81	82	9	MOLECULE:	ACTIN, ALPHA CARDIAC MUSCLE 1
73:00:00	8uzx-G	5.7	3.3	81	82	5	MOLECULE:	ACTIN, ALPHA CARDIAC MUSCLE 1
74:00:00	8fcf-C	5.7	2.4	81	83	9	MOLECULE:	PLASMALEMMA VESICLE-ASSOCIATED PROTEIN
75:00:00	8uz5-G	5.7	3.9	81	82	5	MOLECULE:	ACTIN, ALPHA CARDIAC MUSCLE 1
76:00:00	8sdi-B	5.7	1.3	78	80	10	MOLECULE:	TRIPARTITE MOTIF-CONTAINING PROTEIN 45
77:00:00	8uz6-G	5.7	4.6	80	82	6	MOLECULE:	ACTIN, ALPHA CARDIAC MUSCLE 1
78:00:00	8uwx-G	5.7	4.6	80	82	6	MOLECULE:	ACTIN, ALPHA CARDIAC MUSCLE 1
79:00:00	8v0i-G	5.7	3.8	80	82	6	MOLECULE:	ACTIN, ALPHA CARDIAC MUSCLE 1
80:00:00	8sdi-C	5.7	1.5	79	80	10	MOLECULE:	TRIPARTITE MOTIF-CONTAINING PROTEIN 45
81:00:00	8x7x-C	5.7	2.1	72	72	13	MOLECULE:	HR1
82:00:00	8sjj-D	5.7	2.2	83	86	5	MOLECULE:	SEPTIN-14
83:00:00	8sor-C	5.7	6.9	96	264	6	MOLECULE:	PHOSPHATIDYLINOSITOL 3-KINASE CATALYTIC SUBUNIT T
84:00:00	7zd6-q	5.7	11.8	101	139	9	MOLECULE:	NADH-UBIQUINONE OXIDOREDUCTASE CHAIN 3
85:00:00	7y5d-b	5.7	6.2	95	145	2	MOLECULE:	ATP SYNTHASE SUBUNIT A
86:00:00	1sfc-J	5.7	2.6	74	74	5	MOLECULE:	PROTEIN (SYNAPTOBREVIN 2)
87:00:00	3vem-D	5.7	1.9	78	80	5	MOLECULE:	HELICASE PROTEIN MOM1
88:00:00	8uzx-F	5.7	4.4	81	82	9	MOLECULE:	ACTIN, ALPHA CARDIAC MUSCLE 1
89:00:00	5wq4-D	5.7	3.8	72	72	11	MOLECULE:	UBIQUITIN
90:00:00	8v0y-F	5.7	4.4	80	82	11	MOLECULE:	ACTIN, ALPHA CARDIAC MUSCLE 1
91:00:00	1uix-B	5.7	2.9	68	68	12	MOLECULE:	RHO-ASSOCIATED KINASE
92:00:00	4e61-A	5.7	3.5	84	91	7	MOLECULE:	PROTEIN BIM1
93:00:00	4mvd-B	5.6	14	81	253	2	MOLECULE:	CHOLINE-PHOSPHATE CYTIDYLYLTRANSFERASE A
94:00:00	3trt-A	5.6	1.6	75	75	8	MOLECULE:	VIMENTIN
95:00:00	3iv1-C	5.6	3.3	78	78	5	MOLECULE:	TUMOR SUSCEPTIBILITY GENE 101 PROTEIN
96:00:00	3iv1-G	5.6	3	76	78	5	MOLECULE:	TUMOR SUSCEPTIBILITY GENE 101 PROTEIN
97:00:00	3iv1-D	5.6	3.2	77	78	5	MOLECULE:	TUMOR SUSCEPTIBILITY GENE 101 PROTEIN
98:00:00	5h07-D	5.6	3.8	78	78	6	MOLECULE:	POLYUBIQUITIN-C
99:00:00	8sjj-C	5.6	3.2	81	86	5	MOLECULE:	SEPTIN-14
0:00	8j9s-A	5.6	3.3	71	71	10	MOLECULE:	AMINOACYL TRNA SYNTHASE COMPLEX-INTERACTING MULTI
1:00	8fby-D	5.6	3.4	82	86	4	MOLECULE:	PLASMALEMMA VESICLE-ASSOCIATED PROTEIN
2:00	8t1l-B	5.6	8.7	89	158	3	MOLECULE:	MEDIATOR OF RNA POLYMERASE II TRANSCRIPTION SUBUN
3:00	8v0k-G	5.6	3.4	81	82	9	MOLECULE:	ACTIN, ALPHA CARDIAC MUSCLE 1
4:00	4wy4-C	5.6	3	75	78	7	MOLECULE:	VESICLE-ASSOCIATED MEMBRANE PROTEIN 8
5:00	5xau-E	5.6	3.1	72	72	8	MOLECULE:	LAMININ SUBUNIT ALPHA-5
6:00	8afh-E	5.6	3.7	79	79	14	MOLECULE:	CRESCENTIN
7:00	1fav-A	5.6	3.9	78	78	9	MOLECULE:	HIV-1 ENVELOPE PROTEIN CHIMERA
8:00	8afe-E	5.6	3.7	79	79	14	MOLECULE:	CRESCENTIN
9:00	8dd0-O	5.6	2	70	70	6	MOLECULE:	ACTIN, ALPHA CARDIAC MUSCLE 1
10:00	8sde-A	5.6	2.3	83	89	8	MOLECULE:	TRIPARTITE MOTIF-CONTAINING PROTEIN 29

11:00	8gt9-B	5.6	4.9	83	88	7	MOLECULE:	BECLIN-2
12:00	8uyd-G	5.6	4.5	80	82	11	MOLECULE:	ACTIN, ALPHA CARDIAC MUSCLE 1
13:00	8dd0-N	5.6	4	70	70	6	MOLECULE:	ACTIN, ALPHA CARDIAC MUSCLE 1
14:00	8i3e-B	5.6	6.7	93	135	6	MOLECULE:	ELKS/RAB6-INTERACTING/CAST FAMILY MEMBER 1
15:00	8fby-B	5.6	3.2	84	88	4	MOLECULE:	PLASMALEMMA VESICLE-ASSOCIATED PROTEIN
16:00	3cvf-C	5.6	3.4	74	74	8	MOLECULE:	HOMER PROTEIN HOMOLOG 3
17:00	8afh-O	5.6	3.7	79	79	14	MOLECULE:	CRESCENTIN
18:00	7ucc-J	5.6	1.7	68	68	9	MOLECULE:	PROTEIN FOSB
19:00	8dd0-J	5.6	1.8	70	70	13	MOLECULE:	ACTIN, ALPHA CARDIAC MUSCLE 1
20:00	1gk4-F	5.6	2.6	74	74	7	MOLECULE:	VIMENTIN
21:00	1sfc-F	5.6	3.6	73	73	4	MOLECULE:	PROTEIN (SYNAPTOBREVIN 2)
22:00	3tyy-B	5.6	2.2	71	71	13	MOLECULE:	LAMIN-B1
23:00	3thf-A	5.6	7.1	129	175	9	MOLECULE:	PROTEIN SHROOM
24:00:00	3klt-D	5.6	1.4	70	70	7	MOLECULE:	VIMENTIN
25:00:00	3bas-B	5.6	4.6	79	87	9	MOLECULE:	MYOSIN HEAVY CHAIN, STRIATED MUSCLE/GENERAL CONTR
26:00:00	3vem-B	5.6	1.7	79	82	6	MOLECULE:	HELICASE PROTEIN MOM1
27:00:00	3fwc-F	5.6	2.3	82	84	5	MOLECULE:	CELL DIVISION CONTROL PROTEIN 31
28:00:00	4dhx-A	5.6	2.3	72	72	6	MOLECULE:	80 KDA MCM3-ASSOCIATED PROTEIN
29:00:00	4bl6-A	5.6	2.4	79	82	14	MOLECULE:	PROTEIN BICAUDAL D
30:00:00	4dhx-D	5.6	2.2	69	69	6	MOLECULE:	80 KDA MCM3-ASSOCIATED PROTEIN
31:00:00	5whf-D	5.6	3.7	82	85	10	MOLECULE:	VIMENTIN
32:00:00	3ni0-B	5.6	2.9	80	87	10	MOLECULE:	BONE MARROW STROMAL ANTIGEN 2
33:00:00	1uix-A	5.6	3.6	69	69	12	MOLECULE:	RHO-ASSOCIATED KINASE
34:00:00	5oi7-A	5.6	4.9	83	88	17	MOLECULE:	CENTROSOMAL PROTEIN OF 85 KDA
35:00:00	3v4q-A	5.6	3.5	74	74	5	MOLECULE:	PRELAMIN-A/C
36:00:00	3vp9-B	5.6	5.1	77	77	6	MOLECULE:	GENERAL TRANSCRIPTIONAL COREPRESSOR TUP1
37:00:00	3htk-B	5.6	4	73	73	11	MOLECULE:	STRUCTURAL MAINTENANCE OF CHROMOSOMES PROTEIN 5
38:00:00	7znl-E	5.6	2.3	65	538	8	MOLECULE:	THO COMPLEX SUBUNIT 1
39:00:00	4mvd-H	5.6	14	81	253	2	MOLECULE:	CHOLINE-PHOSPHATE CYTIDYLYLTRANSFERASE A
40:00:00	8fwj-A	5.5	4.8	101	552	6	MOLECULE:	CIRCADIAN CLOCK PROTEIN KAIC
41:00:00	7znk-o	5.5	8.2	91	164	10	MOLECULE:	RNA
42:00:00	6ap0-B	5.5	3.3	81	88	4	MOLECULE:	CASP8-ASSOCIATED PROTEIN 2
43:00:00	6oqa-G	5.5	3.3	81	87	11	MOLECULE:	PEPTIDYL-PROLYL CIS-TRANS ISOMERASE FKBP1A
44:00:00	7fde-G	5.5	3.5	83	231	7	MOLECULE:	V-TYPE PROTON ATPASE SUBUNIT C
45:00:00	7yfj-B	5.5	4.4	84	92	6	MOLECULE:	PRE-MRNA-SPLICING REGULATOR WTAP
46:00:00	8uz6-F	5.5	4	80	82	5	MOLECULE:	ACTIN, ALPHA CARDIAC MUSCLE 1
47:00:00	7z8i-x	5.5	3	83	93	7	MOLECULE:	ARP1 ACTIN RELATED PROTEIN 1 HOMOLOG A
48:00:00	8uwy-F	5.5	4	80	82	5	MOLECULE:	ACTIN, ALPHA CARDIAC MUSCLE 1
49:00:00	8dd0-I	5.5	3.6	70	70	13	MOLECULE:	ACTIN, ALPHA CARDIAC MUSCLE 1
50:00:00	4ll7-G	5.5	4.2	83	87	16	MOLECULE:	SWI5-DEPENDENT HO EXPRESSION PROTEIN 3
51:00:00	8uzy-F	5.5	4.2	79	82	6	MOLECULE:	ACTIN, ALPHA CARDIAC MUSCLE 1
52:00:00	6cfz-D	5.5	2.7	72	73	3	MOLECULE:	ASK1
53:00:00	8uww-F	5.5	4	80	82	5	MOLECULE:	ACTIN, ALPHA CARDIAC MUSCLE 1
54:00:00	1gk4-C	5.5	2.8	70	70	3	MOLECULE:	VIMENTIN
55:00:00	8uz5-F	5.5	4	80	82	5	MOLECULE:	ACTIN, ALPHA CARDIAC MUSCLE 1
56:00:00	7z8i-X	5.5	4.7	83	93	7	MOLECULE:	ARP1 ACTIN RELATED PROTEIN 1 HOMOLOG A
57:00:00	8uwx-F	5.5	4	80	82	5	MOLECULE:	ACTIN, ALPHA CARDIAC MUSCLE 1
58:00:00	8gt9-C	5.5	5.1	82	90	11	MOLECULE:	BECLIN-2
59:00:00	8v01-F	5.5	4.4	79	82	6	MOLECULE:	ACTIN, ALPHA CARDIAC MUSCLE 1
60:00:00	1x79-C	5.5	3.4	80	88	9	MOLECULE:	ADP-RIBOSYLATION FACTOR BINDING PROTEIN GGA1
61:00:00	4n3y-B	5.5	3.2	81	83	6	MOLECULE:	RAB5 GDP/GTP EXCHANGE FACTOR
62:00:00	5jxc-A	5.5	4.5	81	86	11	MOLECULE:	RAS/RAP GTPASE-ACTIVATING PROTEIN SYNGAP
63:00:00	3vem-C	5.5	2	78	83	5	MOLECULE:	HELICASE PROTEIN MOM1
64:00:00	3klt-B	5.5	2.5	71	71	7	MOLECULE:	VIMENTIN
65:00:00	3vem-A	5.5	2.6	80	82	9	MOLECULE:	HELICASE PROTEIN MOM1
66:00:00	5mq4-B	5.5	4.3	79	111	4	MOLECULE:	PROTEIN KINASE C-BINDING PROTEIN 1

67:00:00	4xa6-D	5.5	4	93	105	4	MOLECULE:	GP7-MYH7(1777-1855)-EB1 CHIMERA PROTEIN
68:00:00	4bl6-C	5.5	7	83	86	13	MOLECULE:	PROTEIN BICAUDAL D
69:00:00	5whf-A	5.5	5.8	83	86	7	MOLECULE:	VIMENTIN
70:00:00	5hda-C	5.5	4.9	83	117	4	MOLECULE:	ZINC FINGER MYND DOMAIN-CONTAINING PROTEIN 11
71:00:00	6ap0-A	5.5	3.2	78	82	8	MOLECULE:	CASP8-ASSOCIATED PROTEIN 2
72:00:00	4lhz-E	5.5	5.2	76	76	4	MOLECULE:	RAS-RELATED PROTEIN RAB-8A
73:00:00	5k7b-D	5.5	3.5	80	88	4	MOLECULE:	BECLIN-2
74:00:00	4lhy-E	5.5	5.2	76	76	4	MOLECULE:	RAS-RELATED PROTEIN RAB-8A
75:00:00	4n5b-A	5.4	4.6	89	104	1	MOLECULE:	PHOSPHOPROTEIN
76:00:00	8gt9-A	5.4	6.3	82	91	12	MOLECULE:	BECLIN-2
77:00:00	7vf2-C	5.4	6.8	86	184	7	MOLECULE:	PROTEIN VIRILIZER HOMOLOG
78:00:00	3n7n-D	5.4	3.1	79	158	8	MOLECULE:	MONOPOLIN COMPLEX SUBUNIT CSM1
79:00:00	3n7n-C	5.4	4	81	158	9	MOLECULE:	MONOPOLIN COMPLEX SUBUNIT CSM1
80:00:00	4wij-B	5.4	7.5	106	301	4	MOLECULE:	SPLICING FACTOR, PROLINE- AND GLUTAMINE-RICH
81:00:00	3q8t-A	5.4	4.4	86	94	7	MOLECULE:	BECLIN-1
82:00:00	8ims-A	5.4	3.7	85	93	11	MOLECULE:	E3 UBIQUITIN-PROTEIN LIGASE TRAF7
83:00:00	6y09-C	5.4	5.3	82	86	5	MOLECULE:	RAS-RELATED PROTEIN RAB-33B
84:00:00	6otn-B	5.4	2.3	67	67	6	MOLECULE:	TROPOMYOSIN ALPHA-3 CHAIN
85:00:00	2jee-C	5.4	2.5	74	78	8	MOLECULE:	CELL DIVISION PROTEIN ZAPB
86:00:00	3cve-B	5.4	2.1	66	66	11	MOLECULE:	HOMER PROTEIN HOMOLOG 1
87:00:00	8fby-C	5.4	3.2	85	93	6	MOLECULE:	PLASMALEMMA VESICLE-ASSOCIATED PROTEIN
88:00:00	8uyd-F	5.4	3.5	79	82	6	MOLECULE:	ACTIN, ALPHA CARDIAC MUSCLE 1
89:00:00	7ucd-J	5.4	1.7	68	68	9	MOLECULE:	PROTEIN FOSB
90:00:00	7saf-B	5.4	2.7	67	70	10	MOLECULE:	GENERAL CONTROL TRANSCRIPTION FACTOR GCN4/M PROTE
91:00:00	4ll7-B	5.4	4.1	82	88	16	MOLECULE:	SWI5-DEPENDENT HO EXPRESSION PROTEIN 3
92:00:00	8uzy-G	5.4	3.9	79	82	5	MOLECULE:	ACTIN, ALPHA CARDIAC MUSCLE 1
93:00:00	7say-D	5.4	2.9	67	67	6	MOLECULE:	GENERAL CONTROL TRANSCRIPTION FACTOR GCN4/M PROTE
94:00:00	7apk-o	5.4	6.2	82	155	6	MOLECULE:	THO COMPLEX SUBUNIT 1
95:00:00	7eqb-A	5.4	2.9	68	68	3	MOLECULE:	KINESIN-LIKE PROTEIN
96:00:00	1sfc-B	5.4	3.2	72	72	4	MOLECULE:	PROTEIN (SYNAPTOBREVIN 2)
97:00:00	4ll7-C	5.4	4	83	90	19	MOLECULE:	SWI5-DEPENDENT HO EXPRESSION PROTEIN 3
98:00:00	8bs7-B	5.4	2.3	76	76	7	MOLECULE:	PHOSPHOPROTEIN
99:00:00	1n7s-B	5.4	3.6	68	68	7	MOLECULE:	VESICLE-ASSOCIATED MEMBRANE PROTEIN 2
0:00	8gt9-D	5.4	5.2	83	91	11	MOLECULE:	BECLIN-2
1:00	3cvf-D	5.4	3	72	72	8	MOLECULE:	HOMER PROTEIN HOMOLOG 3
2:00	7yg4-E	5.4	2.3	67	67	7	MOLECULE:	PROTEIN VIRILIZER HOMOLOG
3:00	1gk4-E	5.4	2.7	69	70	3	MOLECULE:	VIMENTIN
4:00	8srq-Y	5.4	5.2	86	252	7	MOLECULE:	RB1-INDUCIBLE COILED-COIL PROTEIN 1
5:00	1x79-B	5.4	5.1	85	90	8	MOLECULE:	ADP-RIBOSYLATION FACTOR BINDING PROTEIN GGA1
6:00	4ll7-F	5.4	4.5	83	91	16	MOLECULE:	SWI5-DEPENDENT HO EXPRESSION PROTEIN 3
7:00	7z8j-X	5.4	5.2	87	93	7	MOLECULE:	BICD FAMILY-LIKE CARGO ADAPTER 1
8:00	5k9l-D	5.4	5.4	83	89	10	MOLECULE:	BECLIN-2
9:00	4zku-A	5.4	5.9	85	179	9	MOLECULE:	TAIL NEEDLE PROTEIN GP26
10:00	2xdj-B	5.4	4	67	67	12	MOLECULE:	UNCHARACTERIZED PROTEIN YBGF
11:00	6tt7-K	5.4	7.8	82	208	5	MOLECULE:	ATP SYNTHASE SUBUNIT ALPHA
12:00	5vpb-D	5.4	3.2	66	66	9	MOLECULE:	PROTEIN FOSB
13:00	3vir-A	5.4	2	69	69	13	MOLECULE:	MATING-TYPE SWITCHING PROTEIN SWI5
14:00	5tby-A	5.4	11.3	99	954	6	MOLECULE:	MYOSIN-7
15:00	5m88-A	5.4	5.4	81	121	5	MOLECULE:	CORE
16:00	5lxn-A	5.4	3.9	78	81	10	MOLECULE:	TRANSFORMING ACIDIC COILED-COIL-CONTAINING PROTEI
17:00	1nkn-C	5.4	3.9	73	75	5	MOLECULE:	S2N51-GCN4
18:00	4i6m-C	5.4	2.5	69	69	6	MOLECULE:	ACTIN-RELATED PROTEIN 7
19:00	1nkn-A	5.4	3.9	73	74	5	MOLECULE:	S2N51-GCN4
20:00	5cch-B	5.4	3	67	67	6	MOLECULE:	VESICLE-ASSOCIATED MEMBRANE PROTEIN 2
21:00	5ccg-B	5.4	3	66	66	5	MOLECULE:	VESICLE-ASSOCIATED MEMBRANE PROTEIN 2
22:00	5cci-B	5.4	3	67	67	3	MOLECULE:	VESICLE-ASSOCIATED MEMBRANE PROTEIN 2

23:00	5lob-D	5.4	5.6	84	85	4	MOLECULE:	RABPHILIN-3A
24:00:00	3tnu-A	5.4	3	83	90	10	MOLECULE:	KERATIN, TYPE I CYTOSKELETAL 14
25:00:00	1l4a-C	5.4	5.1	73	73	10	MOLECULE:	SYNAPTOBREVIN
26:00:00	6mti-B	5.4	3	67	67	3	MOLECULE:	SYNAPTOTAGMIN-1
27:00:00	4lhx-E	5.4	5.2	76	76	4	MOLECULE:	RAS-RELATED PROTEIN RAB-8A
28:00:00	6mti-F	5.4	3.3	67	67	4	MOLECULE:	SYNAPTOTAGMIN-1
29:00:00	6mti-J	5.4	3	67	67	3	MOLECULE:	SYNAPTOTAGMIN-1
30:00:00	5eof-A	5.4	5.5	72	74	7	MOLECULE:	OPTINEURIN
31:00:00	8e4g-a	5.4	2.2	90	127	10	MOLECULE:	PORTAL PROTEIN
32:00:00	3v5b-A	5.4	3	74	74	8	MOLECULE:	PRELAMIN-A/C
33:00:00	6mti-N	5.4	3	67	67	3	MOLECULE:	SYNAPTOTAGMIN-1
34:00:00	6mti-R	5.4	3.3	67	67	4	MOLECULE:	SYNAPTOTAGMIN-1
35:00:00	6mti-V	5.4	3	67	67	3	MOLECULE:	SYNAPTOTAGMIN-1
36:00:00	4mvd-F	5.4	5.6	84	253	7	MOLECULE:	CHOLINE-PHOSPHATE CYTIDYLYLTRANSFERASE A
37:00:00	4om3-D	5.3	23.6	98	122	6	MOLECULE:	TRANSDUCIN-LIKE ENHANCER PROTEIN 1
38:00:00	7jts-s	5.3	9.4	97	290	2	MOLECULE:	RADIAL SPOKE PROTEIN 3
39:00:00	3q8t-B	5.3	4.1	87	95	7	MOLECULE:	BECLIN-1
40:00:00	5wsu-D	5.3	3.7	87	91	3	MOLECULE:	CALMODULIN
41:00:00	7xuz-A	5.3	2.9	84	120	10	MOLECULE:	HISTONE DEACETYLASE 4
42:00:00	2doq-D	5.3	3.9	81	83	6	MOLECULE:	CELL DIVISION CONTROL PROTEIN 31
43:00:00	5wwl-N	5.3	7.7	107	155	6	MOLECULE:	CENTROMERE PROTEIN MIS12
44:00:00	6wmz-C	5.3	6	95	292	3	MOLECULE:	SPLICING FACTOR, PROLINE- AND GLUTAMINE-RICH
45:00:00	5bw9-g	5.3	5.5	83	141	5	MOLECULE:	V-TYPE PROTON ATPASE CATALYTIC SUBUNIT A
46:00:00	5kiu-A	5.3	6.4	75	76	5	MOLECULE:	SELENOPROTEIN S
47:00:00	8arf-B	5.3	4.1	83	97	10	MOLECULE:	PROTEIN SPINDLY
48:00:00	6iak-F	5.3	4.9	71	71	11	MOLECULE:	UNCHARACTERIZED PROTEIN
49:00:00	8bs7-F	5.3	2.4	74	74	5	MOLECULE:	PHOSPHOPROTEIN
50:00:00	7znl-g	5.3	8.1	84	161	5	MOLECULE:	THO COMPLEX SUBUNIT 1
51:00:00	7wxz-B	5.3	4.4	88	185	9	MOLECULE:	SPIKE PROTEIN S2'
52:00:00	7znk-g	5.3	8.1	84	161	5	MOLECULE:	RNA
53:00:00	8v01-G	5.3	3.7	77	82	8	MOLECULE:	ACTIN, ALPHA CARDIAC MUSCLE 1
54:00:00	5toh-B	5.3	4	70	70	7	MOLECULE:	POLYMERASE COFACTOR VP35
55:00:00	7znl-G	5.3	8.1	84	161	5	MOLECULE:	THO COMPLEX SUBUNIT 1
56:00:00	7z8j-x	5.3	2.5	83	93	7	MOLECULE:	BICD FAMILY-LIKE CARGO ADAPTER 1
57:00:00	7znk-G	5.3	8.1	84	161	5	MOLECULE:	RNA
58:00:00	5c9n-A	5.3	2.6	64	64	20	MOLECULE:	GEMININ COILED-COIL DOMAIN-CONTAINING PROTEIN 1
59:00:00	4ll7-D	5.3	4	83	91	19	MOLECULE:	SWI5-DEPENDENT HO EXPRESSION PROTEIN 3
60:00:00	7yes-E	5.3	4.9	67	67	9	MOLECULE:	RNA-DIRECTED RNA POLYMERASE L
61:00:00	7yfj-A	5.3	4.1	83	97	11	MOLECULE:	PRE-MRNA-SPLICING REGULATOR WTAP
62:00:00	3cve-C	5.3	3.2	66	66	17	MOLECULE:	HOMER PROTEIN HOMOLOG 1
63:00:00	8ims-B	5.3	3.5	84	92	6	MOLECULE:	E3 UBIQUITIN-PROTEIN LIGASE TRAF7
64:00:00	8p0v-K	5.3	6.9	101	304	4	MOLECULE:	COILED-COIL DOMAIN-CONTAINING PROTEIN 93
65:00:00	6obi-A	5.3	1.8	68	68	3	MOLECULE:	MYOSIN-VI
66:00:00	8arf-A	5.3	4.1	83	96	5	MOLECULE:	PROTEIN SPINDLY
67:00:00	1jth-B	5.3	4.7	67	67	6	MOLECULE:	SNAP25
68:00:00	7puz-A	5.3	7	109	148	8	MOLECULE:	MICOS COMPLEX SUBUNIT MIC60
69:00:00	8jsm-E	5.3	5	67	67	10	MOLECULE:	RNA-DIRECTED RNA POLYMERASE L
70:00:00	5l1x-L	5.3	4.8	89	370	4	MOLECULE:	HMPV F2 SUBUNIT
71:00:00	5l1x-B	5.3	3.3	86	369	5	MOLECULE:	HMPV F2 SUBUNIT
72:00:00	8jsn-E	5.3	4.4	67	67	10	MOLECULE:	RNA-DIRECTED RNA POLYMERASE L
73:00:00	5d3a-A	5.3	4.2	79	82	4	MOLECULE:	KINESIN-LIKE PROTEIN KIF21A
74:00:00	3s4r-B	5.3	3.1	85	91	11	MOLECULE:	VIMENTIN
75:00:00	2wpr-C	5.3	5.4	85	97	6	MOLECULE:	TRIMERIC AUTOTRANSPORTER ADHESIN FRAGMENT
76:00:00	3klt-A	5.3	2.1	70	71	7	MOLECULE:	VIMENTIN
77:00:00	2xdj-E	5.3	2.8	67	67	16	MOLECULE:	UNCHARACTERIZED PROTEIN YBGF
78:00:00	2xdj-D	5.3	3.8	66	66	12	MOLECULE:	UNCHARACTERIZED PROTEIN YBGF

79:00:00	7x5g-E	5.3	4.7	80	95	10	MOLECULE:	TRANSCRIPTION FACTOR MAFG
80:00:00	3vir-B	5.3	1.1	66	66	14	MOLECULE:	MATING-TYPE SWITCHING PROTEIN SWI5
81:00:00	5oag-D	5.3	4.7	87	98	8	MOLECULE:	RAB-3A-INTERACTING PROTEIN
82:00:00	3h6p-B	5.3	3.2	67	67	7	MOLECULE:	ESAT-6 LIKE PROTEIN ESXS
83:00:00	6ano-B	5.3	3	70	70	13	MOLECULE:	CASP8-ASSOCIATED PROTEIN 2
84:00:00	6f6p-D	5.3	4.9	87	98	6	MOLECULE:	RAB-3A-INTERACTING PROTEIN
85:00:00	5cj1-C	5.3	3.1	80	98	13	MOLECULE:	GP7-MYH7-(1526-1571) CHIMERA PROTEIN
86:00:00	3b5n-B	5.3	3.8	69	69	3	MOLECULE:	SYNAPTOBREVIN HOMOLOG 1
87:00:00	2q6q-B	5.3	3	65	65	8	MOLECULE:	SPINDLE POLE BODY COMPONENT SPC42
88:00:00	3klt-C	5.3	2.4	71	71	7	MOLECULE:	VIMENTIN
89:00:00	4ytd-B	5.3	4.5	87	99	7	MOLECULE:	PROTEIN BICAUDAL D HOMOLOG 1
90:00:00	4c46-C	5.3	3.1	74	74	8	MOLECULE:	GENERAL CONTROL PROTEIN GCN4, GENERAL CONTROL PRO
91:00:00	5ccg-H	5.3	3.3	65	65	5	MOLECULE:	VESICLE-ASSOCIATED MEMBRANE PROTEIN 2
92:00:00	3u1a-B	5.3	3.5	77	84	3	MOLECULE:	SMOOTH MUSCLE TROPOMYOSIN ALPHA
93:00:00	7upz-B	5.3	4.1	68	68	9	MOLECULE:	CCAAT/ENHANCER-BINDING PROTEIN BETA
94:00:00	8ims-C	5.3	4.2	87	92	9	MOLECULE:	E3 UBIQUITIN-PROTEIN LIGASE TRAF7
95:00:00	3u1a-D	5.3	2.8	75	80	3	MOLECULE:	SMOOTH MUSCLE TROPOMYOSIN ALPHA
96:00:00	3vp9-A	5.3	2.2	70	70	11	MOLECULE:	GENERAL TRANSCRIPTIONAL COREPRESSOR TUP1
97:00:00	7tv4-D	5.3	3.4	79	79	5	MOLECULE:	NF-KAPPA-B ESSENTIAL MODULATOR
98:00:00	4wbn-E	5.3	4.4	88	120	3	MOLECULE:	TUBULIN ALPHA-1B CHAIN
99:00:00	3v4w-A	5.3	3.4	74	74	5	MOLECULE:	PRELAMIN-A/C
0:00	5cch-D	5.3	3.9	64	64	3	MOLECULE:	VESICLE-ASSOCIATED MEMBRANE PROTEIN 2
1:00	2e43-B	5.3	3.2	64	64	5	MOLECULE:	CCAAT/ENHANCER-BINDING PROTEIN BETA
2:00	4tuy-E	5.3	4.7	88	120	3	MOLECULE:	TUBULIN ALPHA-1B CHAIN
3:00	2yo3-A	5.3	5.7	80	257	6	MOLECULE:	GENERAL CONTROL PROTEIN GCN4, PUTATIVE INNER MEMB
4:00	7vhp-G	5.2	8.1	109	299	4	MOLECULE:	ATP-DEPENDENT ZINC METALLOPROTEASE FTSH
5:00	5hda-A	5.2	1.9	71	121	11	MOLECULE:	ZINC FINGER MYND DOMAIN-CONTAINING PROTEIN 11
6:00	7yhn-E	5.2	4.5	91	123	8	MOLECULE:	TUBULIN ALPHA-1B CHAIN
7:00	7uw5-A	5.2	7.6	84	723	10	MOLECULE:	MECHANOSENSITIVE CHANNEL MSCK
8:00	3trt-B	5.2	5.4	73	73	10	MOLECULE:	VIMENTIN
9:00	5oag-A	5.2	3.2	87	98	8	MOLECULE:	RAB-3A-INTERACTING PROTEIN
10:00	8r67-E	5.2	3.1	84	122	6	MOLECULE:	DETYROSINATED TUBULIN ALPHA-1B CHAIN
11:00	8b7a-E	5.2	4.5	87	121	7	MOLECULE:	TUBULIN ALPHA-1B CHAIN
12:00	5cci-D	5.2	3.9	64	64	3	MOLECULE:	VESICLE-ASSOCIATED MEMBRANE PROTEIN 2
13:00	4n78-E	5.2	2.2	66	67	5	MOLECULE:	CYTOPLASMIC FMR1-INTERACTING PROTEIN 1
14:00	7yg4-B	5.2	3.6	67	67	9	MOLECULE:	PROTEIN VIRILIZER HOMOLOG
15:00	8xwx-D	5.2	2.7	65	65	8	MOLECULE:	B-CELL RECEPTOR-ASSOCIATED PROTEIN 31
16:00	7znk-O	5.2	7.9	90	164	3	MOLECULE:	RNA
17:00	8j9s-B	5.2	4.1	67	67	9	MOLECULE:	AMINOACYL TRNA SYNTHASE COMPLEX-INTERACTING MULTI
18:00	8j0s-d	5.2	6.4	89	445	9	MOLECULE:	ATP SYNTHASE SUBUNIT A
19:00	7znl-o	5.2	7.9	90	164	3	MOLECULE:	THO COMPLEX SUBUNIT 1
20:00	8bs7-H	5.2	2.1	71	71	6	MOLECULE:	PHOSPHOPROTEIN
21:00	8rc1-E	5.2	2.3	84	89	0	MOLECULE:	TUBULIN ALPHA-1B CHAIN
22:00	8bs7-E	5.2	1.8	71	71	7	MOLECULE:	PHOSPHOPROTEIN
23:00	9b8q-N	5.2	2.7	88	106	6	MOLECULE:	V-TYPE PROTON ATPASE SUBUNIT C 1
24:00:00	7xfr-D	5.2	2.2	62	62	11	MOLECULE:	ISOFORM 2 OF WD REPEAT DOMAIN PHOSPHOINOSITIDE-IN
25:00:00	4ll7-A	5.2	3.8	80	94	13	MOLECULE:	SWI5-DEPENDENT HO EXPRESSION PROTEIN 3
26:00:00	3b5n-F	5.2	4.1	68	68	6	MOLECULE:	SYNAPTOBREVIN HOMOLOG 1
27:00:00	8ppr-P	5.2	19.7	95	176	7	MOLECULE:	KINETOCHORE-ASSOCIATED PROTEIN DSN1 HOMOLOG
28:00:00	7x5e-A	5.2	2.4	77	99	4	MOLECULE:	TRANSCRIPTION FACTOR MAFG
29:00:00	7x5f-E	5.2	4.2	79	95	9	MOLECULE:	TRANSCRIPTION FACTOR MAFG
30:00:00	7x5f-A	5.2	2.8	81	100	10	MOLECULE:	TRANSCRIPTION FACTOR MAFG
31:00:00	6mti-D	5.2	4	64	64	3	MOLECULE:	SYNAPTOTAGMIN-1
32:00:00	6mti-H	5.2	4	64	64	3	MOLECULE:	SYNAPTOTAGMIN-1
33:00:00	6mti-L	5.2	4	64	64	3	MOLECULE:	SYNAPTOTAGMIN-1
34:00:00	1gtw-B	5.2	2.8	65	67	6	MOLECULE:	CAAT/ENHANCER BINDING PROTEIN BETA

35:00:00	2wpq-A	5.2	5.2	87	99	6	MOLECULE:	TRIMERIC AUTOTRANSPORTER ADHESIN FRAGMENT
36:00:00	3mq7-C	5.2	3.8	87	99	6	MOLECULE:	BONE MARROW STROMAL ANTIGEN 2
37:00:00	3mq7-E	5.2	4	87	99	9	MOLECULE:	BONE MARROW STROMAL ANTIGEN 2
38:00:00	6bl7-B	5.2	4.1	87	98	9	MOLECULE:	PROTEIN STU2
39:00:00	2wpq-B	5.2	5	87	99	6	MOLECULE:	TRIMERIC AUTOTRANSPORTER ADHESIN FRAGMENT
40:00:00	3mqc-D	5.2	4	86	100	5	MOLECULE:	BONE MARROW STROMAL ANTIGEN 2
41:00:00	5bu8-A	5.2	5.8	83	199	6	MOLECULE:	DNA STABILIZATION PROTEIN
42:00:00	3mq7-I	5.2	3.2	87	99	7	MOLECULE:	BONE MARROW STROMAL ANTIGEN 2
43:00:00	5d80-M	5.2	5.8	94	203	6	MOLECULE:	V-TYPE PROTON ATPASE CATALYTIC SUBUNIT A
44:00:00	3mq7-D	5.2	4.9	87	99	9	MOLECULE:	BONE MARROW STROMAL ANTIGEN 2
45:00:00	3mq7-A	5.2	4.2	87	99	8	MOLECULE:	BONE MARROW STROMAL ANTIGEN 2
46:00:00	3mq7-G	5.2	3.1	87	99	8	MOLECULE:	BONE MARROW STROMAL ANTIGEN 2
47:00:00	6mti-P	5.2	3.9	64	64	3	MOLECULE:	SYNAPTOTAGMIN-1
48:00:00	7x5g-A	5.2	3.5	80	99	10	MOLECULE:	TRANSCRIPTION FACTOR MAFG
49:00:00	2wt7-A	5.2	2.4	63	63	6	MOLECULE:	PROTO-ONCOGENE PROTEIN C-FOS
50:00:00	2ba2-A	5.2	5.6	76	81	8	MOLECULE:	HYPOTHETICAL UPF0134 PROTEIN MPN010
51:00:00	3ni0-A	5.2	5.1	86	95	10	MOLECULE:	BONE MARROW STROMAL ANTIGEN 2
52:00:00	2e42-B	5.2	3.1	65	67	6	MOLECULE:	CCAAT/ENHANCER-BINDING PROTEIN BETA
53:00:00	4owf-B	5.2	4.8	82	86	5	MOLECULE:	NF-KAPPA-B ESSENTIAL MODULATOR
54:00:00	7xuz-B	5.2	3.6	86	120	9	MOLECULE:	HISTONE DEACETYLASE 4
55:00:00	6mti-T	5.2	4	64	64	3	MOLECULE:	SYNAPTOTAGMIN-1
56:00:00	6mti-X	5.2	4	64	64	3	MOLECULE:	SYNAPTOTAGMIN-1
57:00:00	4bry-B	5.2	3.8	69	69	6	MOLECULE:	GEMININ
58:00:00	3mq7-K	5.2	3.8	86	99	8	MOLECULE:	BONE MARROW STROMAL ANTIGEN 2
59:00:00	5cj1-A	5.2	3.6	81	98	14	MOLECULE:	GP7-MYH7-(1526-1571) CHIMERA PROTEIN
60:00:00	4c46-A	5.2	2.9	72	72	7	MOLECULE:	GENERAL CONTROL PROTEIN GCN4, GENERAL CONTROL PRO
61:00:00	6fia-E	5.2	5.8	82	88	4	MOLECULE:	LINE-1 RETROTRANSPOSABLE ELEMENT ORF1 PROTEIN
62:00:00	1sfc-A	5.2	4.7	69	69	10	MOLECULE:	PROTEIN (SYNAPTOBREVIN 2)
63:00:00	4o4h-E	5.2	4.5	88	123	3	MOLECULE:	TUBULIN ALPHA-1B CHAIN
64:00:00	7bjg-A	5.2	5.5	87	98	6	MOLECULE:	SD21996P
65:00:00	6abo-A	5.2	4.9	104	213	12	MOLECULE:	DNA REPAIR PROTEIN XRCC4
66:00:00	3hd7-E	5.2	5.6	83	90	2	MOLECULE:	VESICLE-ASSOCIATED MEMBRANE PROTEIN 2
67:00:00	4i4t-E	5.2	4.4	88	123	3	MOLECULE:	TUBULIN ALPHA-1B CHAIN
68:00:00	4o2b-E	5.2	4.5	88	121	3	MOLECULE:	TUBULIN ALPHA-1B CHAIN
69:00:00	1gu5-B	5.2	3.1	65	67	8	MOLECULE:	CAAT/ENHANCER BINDING PROTEIN BETA
70:00:00	4tv9-E	5.2	4.5	88	122	3	MOLECULE:	TUBULIN ALPHA-1B CHAIN
71:00:00	4i50-E	5.2	4.4	88	123	3	MOLECULE:	TUBULIN ALPHA-1B CHAIN
72:00:00	4ihj-E	5.2	4.4	88	121	3	MOLECULE:	TUBULIN ALPHA-1B CHAIN
73:00:00	1gu4-B	5.2	3.3	65	67	8	MOLECULE:	CAAT/ENHANCER BINDING PROTEIN BETA
74:00:00	5c8y-E	5.2	4.6	88	121	3	MOLECULE:	TUBULIN ALPHA
75:00:00	4o4j-E	5.2	4.5	88	123	3	MOLECULE:	TUBULIN ALPHA-1B CHAIN
76:00:00	4tv8-E	5.2	4.5	88	121	3	MOLECULE:	TUBULIN ALPHA-1B CHAIN
77:00:00	5ccg-D	5.2	4.1	64	64	3	MOLECULE:	VESICLE-ASSOCIATED MEMBRANE PROTEIN 2
78:00:00	4nqj-A	5.1	37.2	126	177	8	MOLECULE:	E3 UBIQUITIN-PROTEIN LIGASE TRIM69
79:00:00	6fia-D	5.1	23	93	104	2	MOLECULE:	LINE-1 RETROTRANSPOSABLE ELEMENT ORF1 PROTEIN
80:00:00	4bt9-B	5.1	4.3	81	238	7	MOLECULE:	PROLYL 4-HYDROXYLASE SUBUNIT ALPHA-1
81:00:00	8ppr-F	5.1	8.4	78	112	8	MOLECULE:	KINETOCHORE-ASSOCIATED PROTEIN DSN1 HOMOLOG
82:00:00	5mq4-C	5.1	5.5	78	117	5	MOLECULE:	PROTEIN KINASE C-BINDING PROTEIN 1
83:00:00	8hpo-D	5.1	6.2	92	165	7	MOLECULE:	TRANSCRIPTIONAL REGULATORY PROTEIN UME1
84:00:00	8rgq-Z	5.1	13.8	109	141	6	MOLECULE:	NADH DEHYDROGENASE [UBIQUINONE] IRON-SULFUR PROTE
85:00:00	3cve-A	5.1	2.8	64	68	9	MOLECULE:	HOMER PROTEIN HOMOLOG 1
86:00:00	4aj5-Y	5.1	3.2	78	99	8	MOLECULE:	SPINDLE AND KINETOCHORE-ASSOCIATED PROTEIN 1
87:00:00	4aj5-1	5.1	3.2	78	99	8	MOLECULE:	SPINDLE AND KINETOCHORE-ASSOCIATED PROTEIN 1
88:00:00	7x5d-A	5.1	4	87	99	7	MOLECULE:	LAMIN-A/C
89:00:00	3cve-D	5.1	1.8	61	61	16	MOLECULE:	HOMER PROTEIN HOMOLOG 1
90:00:00	3rk2-D	5.1	4.1	65	65	3	MOLECULE:	VESICLE-ASSOCIATED MEMBRANE PROTEIN 2

91:00:00	7tv4-B	5.1	4	79	81	8	MOLECULE:	NF-KAPPA-B ESSENTIAL MODULATOR
92:00:00	7saf-A	5.1	2	62	62	6	MOLECULE:	GENERAL CONTROL TRANSCRIPTION FACTOR GCN4/M PROTE
93:00:00	9b8q-O	5.1	4	84	103	5	MOLECULE:	V-TYPE PROTON ATPASE SUBUNIT C 1
94:00:00	7yet-E	5.1	5.3	67	67	9	MOLECULE:	RNA-DIRECTED RNA POLYMERASE L
95:00:00	8q85-W	5.1	5.8	81	106	5	MOLECULE:	KINETOCHORE PROTEIN NDC80
96:00:00	8k8d-A	5.1	2.1	66	68	12	MOLECULE:	CCAAT/ENHANCER-BINDING PROTEIN BETA
97:00:00	7znl-O	5.1	6.9	86	164	9	MOLECULE:	THO COMPLEX SUBUNIT 1
98:00:00	8bs7-G	5.1	1.6	68	68	6	MOLECULE:	PHOSPHOPROTEIN
99:00:00	8ard-A	5.1	1.7	62	62	13	MOLECULE:	UNCONVENTIONAL MYOSIN-VI
0:00	8h9g-K	5.1	5.7	96	198	2	MOLECULE:	ATP SYNTHASE F(0) COMPLEX SUBUNIT B1, MITOCHONDRI
1:00	8bs7-D	5.1	2.3	78	80	5	MOLECULE:	PHOSPHOPROTEIN
2:00	7x5d-B	5.1	4.2	83	94	8	MOLECULE:	LAMIN-A/C
3:00	4ll7-E	5.1	4.3	82	90	18	MOLECULE:	SWI5-DEPENDENT HO EXPRESSION PROTEIN 3
4:00	1n7s-A	5.1	4.4	63	63	13	MOLECULE:	VESICLE-ASSOCIATED MEMBRANE PROTEIN 2
5:00	1urq-B	5.1	2.7	63	63	5	MOLECULE:	M-TOMOSYN ISOFORM
6:00	4e61-B	5.1	5.6	78	84	8	MOLECULE:	PROTEIN BIM1
7:00	4ll7-H	5.1	3.8	81	91	10	MOLECULE:	SWI5-DEPENDENT HO EXPRESSION PROTEIN 3
8:00	8t1l-D	5.1	8	98	161	8	MOLECULE:	MEDIATOR OF RNA POLYMERASE II TRANSCRIPTION SUBUN
9:00	8b7c-E	5.1	5.5	90	123	3	MOLECULE:	TUBULIN ALPHA-1B CHAIN
10:00	1hjb-D	5.1	3.2	68	68	12	MOLECULE:	CCAAT/ENHANCER BINDING PROTEIN BETA
11:00	3rk2-H	5.1	4.1	65	65	3	MOLECULE:	VESICLE-ASSOCIATED MEMBRANE PROTEIN 2
12:00	7c4j-H	5.1	6.2	93	242	3	MOLECULE:	TRANSCRIPTION REGULATORY PROTEIN SNF12
13:00	1h8a-B	5.1	2.8	67	67	7	MOLECULE:	CAAT/ENHANCER BINDING PROTEIN BETA
14:00	3mq7-L	5.1	4.8	87	99	9	MOLECULE:	BONE MARROW STROMAL ANTIGEN 2
15:00	3pp5-A	5.1	3.4	63	63	13	MOLECULE:	BRK1
16:00	6vol-I	5.1	3	82	150	6	MOLECULE:	ATP SYNTHASE SUBUNIT ALPHA, CHLOROPLASTIC
17:00	3hd7-A	5.1	5.6	83	91	2	MOLECULE:	VESICLE-ASSOCIATED MEMBRANE PROTEIN 2
18:00	3mqc-A	5.1	3.3	87	100	9	MOLECULE:	BONE MARROW STROMAL ANTIGEN 2
19:00	3mqc-C	5.1	3.5	84	100	10	MOLECULE:	BONE MARROW STROMAL ANTIGEN 2
20:00	3mq7-H	5.1	4.8	87	99	9	MOLECULE:	BONE MARROW STROMAL ANTIGEN 2
21:00	3s9g-A	5.1	4.8	78	82	18	MOLECULE:	PROTEIN HEXIM1
22:00	2xdj-C	5.1	3.1	64	67	14	MOLECULE:	UNCHARACTERIZED PROTEIN YBGF
23:00	3tnu-B	5.1	4.9	86	95	9	MOLECULE:	KERATIN, TYPE I CYTOSKELETAL 14
24:00:00	3u1c-A	5.1	6.6	88	101	10	MOLECULE:	TROPOMYOSIN ALPHA-1 CHAIN
25:00:00	3b5n-A	5.1	3.9	61	61	5	MOLECULE:	SYNAPTOBREVIN HOMOLOG 1
26:00:00	5wme-D	5.1	2.3	75	89	5	MOLECULE:	CAPSID ASSEMBLY SCAFFOLDING PROTEIN,MYOSIN-7
27:00:00	3mqc-B	5.1	4.4	86	100	5	MOLECULE:	BONE MARROW STROMAL ANTIGEN 2
28:00:00	1l2p-A	5.1	1.2	61	61	7	MOLECULE:	ATP SYNTHASE B CHAIN
29:00:00	2nps-B	5.1	2.8	68	68	4	MOLECULE:	VESICLE-ASSOCIATED MEMBRANE PROTEIN 4
30:00:00	7x5e-E	5.1	3.4	80	101	10	MOLECULE:	TRANSCRIPTION FACTOR MAFG
31:00:00	5toi-B	5.1	4.7	70	70	6	MOLECULE:	POLYMERASE COFACTOR VP35
32:00:00	4ytd-A	5.1	6.4	88	96	9	MOLECULE:	PROTEIN BICAUDAL D HOMOLOG 1
33:00:00	2ba2-B	5.1	4.5	74	80	7	MOLECULE:	HYPOTHETICAL UPF0134 PROTEIN MPN010
34:00:00	5dol-A	5.1	2	61	61	7	MOLECULE:	INITIATION-CONTROL PROTEIN YABA
35:00:00	2ba2-C	5.1	4.8	74	81	7	MOLECULE:	HYPOTHETICAL UPF0134 PROTEIN MPN010
36:00:00	5ca0-E	5.1	4.7	88	121	3	MOLECULE:	TUBULIN ALPHA-1B CHAIN
37:00:00	4lhx-F	5.1	6.1	75	76	4	MOLECULE:	RAS-RELATED PROTEIN RAB-8A
38:00:00	4i55-E	5.1	4.4	88	124	3	MOLECULE:	TUBULIN ALPHA-1B CHAIN
39:00:00	4o4l-E	5.1	4.4	88	123	3	MOLECULE:	TUBULIN ALPHA-1B CHAIN
40:00:00	4o4i-E	5.1	4.4	88	121	3	MOLECULE:	TUBULIN ALPHA-1B CHAIN
41:00:00	5cb4-E	5.1	4.7	88	121	3	MOLECULE:	TUBULIN ALPHA
42:00:00	4x20-E	5.1	4.6	88	123	3	MOLECULE:	TUBULIN ALPHA CHAIN
43:00:00	4o2a-E	5.1	5	91	124	3	MOLECULE:	TUBULIN ALPHA-1B CHAIN
44:00:00	1kil-D	5.1	4.5	65	66	3	MOLECULE:	SYNAPTOBREVIN SNARE MOTIF
45:00:00	1hjb-B	5.1	2.1	65	67	12	MOLECULE:	CCAAT/ENHANCER BINDING PROTEIN BETA
46:00:00	6uci-B	5.1	1.5	61	61	7	MOLECULE:	PROTEIN FOSB

47:00:00	5ca1-E	5.1	4.6	88	121	3	MOLECULE:	TUBULIN ALPHA
48:00:00	3i00-B	5.1	2.4	74	80	7	MOLECULE:	HUNTINGTIN-INTERACTING PROTEIN 1
49:00:00	1n7s-D	5.1	4.8	66	66	3	MOLECULE:	VESICLE-ASSOCIATED MEMBRANE PROTEIN 2
50:00:00	2qa7-B	5.1	2.7	69	70	9	MOLECULE:	HUNTINGTIN-INTERACTING PROTEIN 1
51:00:00	4x1i-E	5.1	4.6	88	123	3	MOLECULE:	TUBULIN ALPHA CHAIN
52:00:00	3b5n-H	5.1	3.7	63	63	3	MOLECULE:	SYNAPTOBREVIN HOMOLOG 1
53:00:00	2qa7-D	5.1	2.8	69	70	9	MOLECULE:	HUNTINGTIN-INTERACTING PROTEIN 1
54:00:00	4x1y-E	5.1	4.5	87	123	3	MOLECULE:	TUBULIN ALPHA CHAIN
55:00:00	1h88-A	5.1	2.2	67	70	9	MOLECULE:	CCAAT/ENHANCER BINDING PROTEIN BETA
56:00:00	1ebo-B	5.1	4.3	92	112	5	MOLECULE:	EBOLA VIRUS ENVELOPE PROTEIN CHIMERA CONSISTING
57:00:00	7znk-e	5.1	2.4	65	527	8	MOLECULE:	RNA
58:00:00	7znl-e	5.1	2.4	65	527	8	MOLECULE:	THO COMPLEX SUBUNIT 1
59:00:00	7znk-E	5.1	2.4	65	538	8	MOLECULE:	RNA
60:00:00	3opc-B	5	2.8	87	131	9	MOLECULE:	UNCHARACTERIZED PROTEIN
61:00:00	7woo-G	5	13.3	89	200	12	MOLECULE:	NUCLEOPORIN NIC96
62:00:00	4he8-A	5	25.4	84	92	10	MOLECULE:	NADH-QUINONE OXIDOREDUCTASE SUBUNIT 7
63:00:00	7sqk-C	5	31.1	122	600	11	MOLECULE:	HAUS AUGMIN-LIKE COMPLEX SUBUNIT 1
64:00:00	5oxf-A	5	7.3	115	703	5	MOLECULE:	GTP-BINDING PROTEIN
65:00:00	6ian-C	5	26.7	93	432	9	MOLECULE:	INTRAFLAGELLAR TRANSPORT PROTEIN 74
66:00:00	4aj5-2	5	4.4	78	100	5	MOLECULE:	SPINDLE AND KINETOCHORE-ASSOCIATED PROTEIN 1
67:00:00	3n7n-B	5	3.7	85	164	7	MOLECULE:	MONOPOLIN COMPLEX SUBUNIT CSM1
68:00:00	3b5n-D	5	4.1	64	64	3	MOLECULE:	SYNAPTOBREVIN HOMOLOG 1
69:00:00	4aj5-4	5	3.3	77	98	8	MOLECULE:	SPINDLE AND KINETOCHORE-ASSOCIATED PROTEIN 1
70:00:00	4aj5-V	5	3.4	77	98	8	MOLECULE:	SPINDLE AND KINETOCHORE-ASSOCIATED PROTEIN 1
71:00:00	4aj5-X	5	3.8	78	98	8	MOLECULE:	SPINDLE AND KINETOCHORE-ASSOCIATED PROTEIN 1
72:00:00	3m0d-A	5	3.2	63	63	5	MOLECULE:	TNF RECEPTOR-ASSOCIATED FACTOR 2
73:00:00	6z26-B	5	4.4	87	103	8	MOLECULE:	SPINDLE ASSEMBLY ABNORMAL PROTEIN 6 HOMOLOG
74:00:00	6zpm-A	5	3.2	85	103	5	MOLECULE:	TRYPANOSOMA CRUZI KKT4 117-218
75:00:00	7y5b-b	5	10.8	104	145	3	MOLECULE:	ATP SYNTHASE SUBUNIT ALPHA
76:00:00	4m3l-A	5	1.5	60	60	8	MOLECULE:	E3 UBIQUITIN-PROTEIN LIGASE TRIM63
77:00:00	8a5a-G	5	7.1	91	110	7	MOLECULE:	CHROMATIN-REMODELING ATPASE INO80
78:00:00	3zdo-F	5	3.1	65	65	9	MOLECULE:	PHOSPHOPROTEIN
79:00:00	7wjt-B	5	1.5	60	60	8	MOLECULE:	ISOFORM 2 OF TRANSMEMBRANE PROTEIN 266
80:00:00	3rl0-X	5	3.8	63	63	2	MOLECULE:	VESICLE-ASSOCIATED MEMBRANE PROTEIN 2
81:00:00	7w2u-W	5	13.3	105	142	10	MOLECULE:	NADH DEHYDROGENASE [UBIQUINONE] FLAVOPROTEIN 1,
82:00:00	3rl0-L	5	3.9	64	64	2	MOLECULE:	VESICLE-ASSOCIATED MEMBRANE PROTEIN 2
83:00:00	7yer-E	5	5.4	67	67	9	MOLECULE:	RNA-DIRECTED RNA POLYMERASE L
84:00:00	1qbz-C	5	3.1	70	120	7	MOLECULE:	PROTEIN (SIV GP41 ECTODOMAIN)
85:00:00	8g0c-b	5	6.2	94	141	3	MOLECULE:	ATP SYNTHASE SUBUNIT ALPHA
86:00:00	8ap7-L	5	2	65	65	5	MOLECULE:	ATP SYNTHASE SUBUNIT A
87:00:00	1h89-A	5	1.9	64	64	5	MOLECULE:	CAAT/ENHANCER BINDING PROTEIN BETA
88:00:00	1gtw-A	5	2.9	65	65	8	MOLECULE:	CAAT/ENHANCER BINDING PROTEIN BETA
89:00:00	2yo3-C	5	4.1	75	257	5	MOLECULE:	GENERAL CONTROL PROTEIN GCN4, PUTATIVE INNER MEMB
90:00:00	8k8d-B	5	2.7	66	66	11	MOLECULE:	CCAAT/ENHANCER-BINDING PROTEIN BETA
91:00:00	8bs7-C	5	2.7	77	81	8	MOLECULE:	PHOSPHOPROTEIN
92:00:00	8at4-E	5	23.9	106	217	7	MOLECULE:	HAUS AUGMIN-LIKE COMPLEX SUBUNIT 1
93:00:00	8bd7-M	5	8.1	95	164	6	MOLECULE:	IFT88
94:00:00	6zpj-A	5	7.1	90	110	11	MOLECULE:	LEISHMANIA MEXICANA KKT4
95:00:00	6r0z-I	5	2.2	77	103	5	MOLECULE:	V-TYPE ATP SYNTHASE ALPHA CHAIN
96:00:00	8bs7-A	5	1.7	68	68	6	MOLECULE:	PHOSPHOPROTEIN
97:00:00	8afh-B	5	4	84	97	6	MOLECULE:	CRESCENTIN
98:00:00	1hvv-B	5	2.2	62	62	3	MOLECULE:	SYNTAXIN 1A
99:00:00	8afe-B	5	4	84	97	6	MOLECULE:	CRESCENTIN
0:00	1gd2-F	5	4.3	64	64	9	MOLECULE:	DNA (5'-
1:00	8afh-L	5	4	84	97	6	MOLECULE:	CRESCENTIN
2:00	4q9u-D	5	3.5	74	75	11	MOLECULE:	RAB5 GDP/GTP EXCHANGE FACTOR

3:00	6yjd-A	5	3.7	83	104	8	MOLECULE:	CAPSID ASSEMBLY SCAFFOLDING PROTEIN,PRELAMIN-A/C
4:00	4wpx-B	5	9.7	78	81	5	MOLECULE:	CELL DIVISION CONTROL PROTEIN 31-LIKE PROTEIN
5:00	2e43-A	5	2.2	62	62	8	MOLECULE:	CCAAT/ENHANCER-BINDING PROTEIN BETA
6:00	7f3t-A	5	4.6	85	330	11	MOLECULE:	TRANSMEMBRANE PROTEIN 120A
7:00	5l1x-F	5	5.1	88	369	3	MOLECULE:	HMPV F2 SUBUNIT
8:00	2no2-A	5	4.5	87	102	10	MOLECULE:	HUNTINGTIN-INTERACTING PROTEIN 1
9:00	4wpx-E	5	9.8	78	81	5	MOLECULE:	CELL DIVISION CONTROL PROTEIN 31-LIKE PROTEIN
10:00	5ed9-B	5	4.4	72	72	10	MOLECULE:	SUN DOMAIN-CONTAINING PROTEIN 2
11:00	1hjb-E	5	4.7	66	68	9	MOLECULE:	CCAAT/ENHANCER BINDING PROTEIN BETA
12:00	5j9t-K	5	23.9	123	269	11	MOLECULE:	HISTONE ACETYLTRANSFERASE ESA1
13:00	4n21-C	5	3.8	91	124	10	MOLECULE:	GP2 ECTODOMAIN
14:00	5j9w-G	5	11.7	101	275	10	MOLECULE:	HISTONE ACETYLTRANSFERASE ESA1
15:00	2xdj-A	5	4.3	66	66	14	MOLECULE:	UNCHARACTERIZED PROTEIN YBGF
16:00	6ys4-F	5	3.5	87	101	6	MOLECULE:	SPINDLE ASSEMBLY ABNORMAL PROTEIN 6 HOMOLOG
17:00	5vpf-C	5	2.3	64	64	6	MOLECULE:	PROTEIN FOSB
18:00	6a9p-E	5	6.1	87	102	11	MOLECULE:	GLIAL FIBRILLARY ACIDIC PROTEIN
19:00	2xu6-A	5	2.7	63	63	8	MOLECULE:	MDV1 COILED COIL
20:00	5wsu-C	5	2.4	82	90	4	MOLECULE:	CALMODULIN
21:00	3ryc-E	5	5.2	91	136	3	MOLECULE:	TUBULIN ALPHA CHAIN
22:00	3h6p-A	5	2.7	60	60	5	MOLECULE:	ESAT-6 LIKE PROTEIN ESXS
23:00	4jzl-A	5	1.8	62	62	5	MOLECULE:	B-CELL RECEPTOR-ASSOCIATED PROTEIN 31
24:00:00	5wme-B	5	2.1	74	92	8	MOLECULE:	CAPSID ASSEMBLY SCAFFOLDING PROTEIN,MYOSIN-7
25:00:00	1t3j-A	5	1.9	62	62	13	MOLECULE:	MITOFUSIN 1
26:00:00	3lt6-D	5	2.9	62	62	13	MOLECULE:	ADHESIN YADA
27:00:00	1gu4-A	5	2.8	65	65	14	MOLECULE:	CAAT/ENHANCER BINDING PROTEIN BETA
28:00:00	5d60-D	5	3	61	61	13	MOLECULE:	PUTATIVE TRANSCRIPTION FACTOR
29:00:00	3mq7-B	5	4.7	87	99	9	MOLECULE:	BONE MARROW STROMAL ANTIGEN 2
30:00:00	2wt7-B	5	4	75	90	7	MOLECULE:	PROTO-ONCOGENE PROTEIN C-FOS
31:00:00	2xu6-B	5	2.7	64	64	8	MOLECULE:	MDV1 COILED COIL
32:00:00	2q6q-A	5	2.7	62	62	11	MOLECULE:	SPINDLE POLE BODY COMPONENT SPC42
33:00:00	2wps-A	5	5.2	87	98	3	MOLECULE:	TRIMERIC AUTOTRANSPORTER ADHESIN FRAGMENT
34:00:00	6znl-N	5	5.9	84	280	11	MOLECULE:	ARP1 ACTIN RELATED PROTEIN 1 HOMOLOG A
35:00:00	2qa7-C	5	5	87	102	7	MOLECULE:	HUNTINGTIN-INTERACTING PROTEIN 1
36:00:00	3u59-D	5	5.1	89	98	4	MOLECULE:	TROPOMYOSIN BETA CHAIN
37:00:00	4n21-F	5	3.9	92	124	11	MOLECULE:	GP2 ECTODOMAIN
38:00:00	3u1a-A	5	3.3	76	82	4	MOLECULE:	SMOOTH MUSCLE TROPOMYOSIN ALPHA
39:00:00	4lhx-D	5	4.6	71	71	4	MOLECULE:	RAS-RELATED PROTEIN RAB-8A
40:00:00	1sfc-I	5	4.9	69	69	13	MOLECULE:	PROTEIN (SYNAPTOBREVIN 2)
41:00:00	4li0-D	5	3.9	69	69	10	MOLECULE:	RAS-RELATED PROTEIN RAB-8A
42:00:00	4jzl-D	5	2	61	61	5	MOLECULE:	B-CELL RECEPTOR-ASSOCIATED PROTEIN 31
43:00:00	4iij-E	5	4.6	88	122	3	MOLECULE:	TUBULIN ALPHA-1B CHAIN
44:00:00	4n21-E	5	3.9	91	121	9	MOLECULE:	GP2 ECTODOMAIN
45:00:00	4jzl-B	5	2	61	61	5	MOLECULE:	B-CELL RECEPTOR-ASSOCIATED PROTEIN 31
46:00:00	2qa7-A	5	4.6	87	99	7	MOLECULE:	HUNTINGTIN-INTERACTING PROTEIN 1
47:00:00	5ccg-J	5	3.7	63	63	3	MOLECULE:	VESICLE-ASSOCIATED MEMBRANE PROTEIN 2
48:00:00	6yf5-A	5	5.9	84	89	6	MOLECULE:	PRELAMIN-A/C,MICROTUBULE-ASSOCIATED PROTEIN RP/EB
49:00:00	3rl0-P	5	3.8	64	64	2	MOLECULE:	VESICLE-ASSOCIATED MEMBRANE PROTEIN 2
50:00:00	2e42-A	5	2.6	65	65	8	MOLECULE:	CCAAT/ENHANCER-BINDING PROTEIN BETA
51:00:00	1ebo-C	5	5.5	92	111	9	MOLECULE:	EBOLA VIRUS ENVELOPE PROTEIN CHIMERA CONSISTING
52:00:00	4g2k-B	5	5.2	86	108	12	MOLECULE:	GENERAL CONTROL PROTEIN GCN4, ENVELOPE GLYCOPROTE
53:00:00	4aj5-T	4.9	27.1	101	110	6	MOLECULE:	SPINDLE AND KINETOCHORE-ASSOCIATED PROTEIN 1
54:00:00	8wjo-B	4.9	42.1	84	283	8	MOLECULE:	STRUCTURAL MAINTENANCE OF CHROMOSOMES PROTEIN 5
55:00:00	8tek-P	4.9	3.1	78	138	5	MOLECULE:	DYNEIN REGULATORY COMPLEX PROTEIN 1/2 N-TERMINAL
56:00:00	8idc-E	4.9	4.3	95	300	9	MOLECULE:	CELL DIVISION ATP-BINDING PROTEIN FTSE
57:00:00	1m1j-D	4.9	10.8	112	194	5	MOLECULE:	FIBRINOGEN ALPHA SUBUNIT
58:00:00	4r3z-A	4.9	3.6	73	76	10	MOLECULE:	AMINOACYL TRNA SYNTHASE COMPLEX-INTERACTING MULTI

59:00:00	2p2u-B	4.9	5.7	75	153	8	MOLECULE:	HOST-NUCLEASE INHIBITOR PROTEIN GAM, PUTATIVE
60:00:00	4hl8-A	4.9	6.6	101	782	3	MOLECULE:	MAJOR VAULT PROTEIN
61:00:00	5wlz-C	4.9	7.8	100	206	7	MOLECULE:	DNA REPAIR PROTEIN XRCC4,MYOSIN-7
62:00:00	1s1c-Y	4.9	3.3	70	70	4	MOLECULE:	TRANSFORMING PROTEIN RHOA
63:00:00	4aj5-3	4.9	3.5	76	98	8	MOLECULE:	SPINDLE AND KINETOCHORE-ASSOCIATED PROTEIN 1
64:00:00	4emc-B	4.9	4	75	158	7	MOLECULE:	MONOPOLIN COMPLEX SUBUNIT CSM1
65:00:00	5d60-B	4.9	2.7	61	61	16	MOLECULE:	PUTATIVE TRANSCRIPTION FACTOR
66:00:00	4h22-B	4.9	3.2	75	83	4	MOLECULE:	LEUCINE-RICH REPEAT FLIGHTLESS-INTERACTING PROTEI
67:00:00	5ajs-B	4.9	2.5	64	64	6	MOLECULE:	THAP DOMAIN-CONTAINING PROTEIN 11
68:00:00	8q85-b	4.9	5.3	69	70	12	MOLECULE:	KINETOCHORE PROTEIN NDC80
69:00:00	4aj5-B	4.9	11.3	80	87	14	MOLECULE:	SPINDLE AND KINETOCHORE-ASSOCIATED PROTEIN 1
70:00:00	1wt6-D	4.9	3.3	63	63	5	MOLECULE:	MYOTONIN-PROTEIN KINASE
71:00:00	3b5n-L	4.9	3.5	62	62	3	MOLECULE:	SYNAPTOBREVIN HOMOLOG 1
72:00:00	7yes-D	4.9	2.7	68	68	13	MOLECULE:	RNA-DIRECTED RNA POLYMERASE L
73:00:00	7upz-A	4.9	2.3	64	64	9	MOLECULE:	CCAAT/ENHANCER-BINDING PROTEIN BETA
74:00:00	8pr4-x	4.9	2	62	92	8	MOLECULE:	ARP11
75:00:00	8xwx-F	4.9	2.5	62	66	6	MOLECULE:	B-CELL RECEPTOR-ASSOCIATED PROTEIN 31
76:00:00	7usd-C	4.9	5.1	74	191	8	MOLECULE:	CYTOPLASMIC FMR1-INTERACTING PROTEIN 1
77:00:00	7xho-K	4.9	15.7	105	230	7	MOLECULE:	CENTROMERE PROTEIN C
78:00:00	8uwy-G	4.9	4.6	73	82	5	MOLECULE:	ACTIN, ALPHA CARDIAC MUSCLE 1
79:00:00	8p1u-C	4.9	5.6	80	93	10	MOLECULE:	CELL DIVISION PROTEIN FTSL
80:00:00	8ap7-l	4.9	1.8	65	65	6	MOLECULE:	ATP SYNTHASE SUBUNIT A
81:00:00	7ucd-F	4.9	1.9	64	64	8	MOLECULE:	PROTEIN FOSB
82:00:00	8jsm-C	4.9	5.3	70	99	7	MOLECULE:	RNA-DIRECTED RNA POLYMERASE L
83:00:00	8pr2-B	4.9	2.8	87	106	8	MOLECULE:	C-JUN-AMINO-TERMINAL KINASE-INTERACTING PROTEIN 3
84:00:00	8q85-F	4.9	7.5	95	230	8	MOLECULE:	KINETOCHORE PROTEIN NDC80
85:00:00	3hrn-A	4.9	2.3	63	63	8	MOLECULE:	TRANSIENT RECEPTOR POTENTIAL (TRP) CHANNEL
86:00:00	6gbo-A	4.9	6.2	72	73	7	MOLECULE:	POLYMERASE COFACTOR VP35
87:00:00	3jb9-U	4.9	3.8	89	430	9	MOLECULE:	PRE-MRNA-SPLICING FACTOR SPP42
88:00:00	1c1g-D	4.9	11.8	105	284	6	MOLECULE:	TROPOMYOSIN
89:00:00	7xhn-U	4.9	5.7	83	140	4	MOLECULE:	CENTROMERE PROTEIN O
90:00:00	1hvv-D	4.9	3.6	61	61	3	MOLECULE:	SYNTAXIN 1A
91:00:00	3b5n-J	4.9	3.3	64	64	6	MOLECULE:	SYNAPTOBREVIN HOMOLOG 1
92:00:00	8bd7-X	4.9	7.4	87	106	2	MOLECULE:	IFT88
93:00:00	7usc-D	4.9	1.8	60	60	7	MOLECULE:	CYTOPLASMIC FMR1-INTERACTING PROTEIN 1
94:00:00	7usd-D	4.9	2.3	62	62	5	MOLECULE:	CYTOPLASMIC FMR1-INTERACTING PROTEIN 1
95:00:00	4q9u-H	4.9	3.9	77	82	10	MOLECULE:	RAB5 GDP/GTP EXCHANGE FACTOR
96:00:00	4gkw-A	4.9	3.4	84	158	6	MOLECULE:	SPINDLE ASSEMBLY ABNORMAL PROTEIN 6
97:00:00	5ylz-t	4.9	7	82	128	4	MOLECULE:	PRE-MRNA-SPLICING FACTOR 8
98:00:00	5omd-A	4.9	1.8	61	61	7	MOLECULE:	DNA DAMAGE CHECKPOINT PROTEIN LCD1
99:00:00	3rl0-f	4.9	3.6	62	62	0	MOLECULE:	VESICLE-ASSOCIATED MEMBRANE PROTEIN 2
0:00	3m0d-B	4.9	3.4	63	63	5	MOLECULE:	TNF RECEPTOR-ASSOCIATED FACTOR 2
1:00	2b9c-B	4.9	8.6	94	142	6	MOLECULE:	STRIATED-MUSCLE ALPHA TROPOMYOSIN
2:00	6dd9-D	4.9	3.3	87	121	9	MOLECULE:	SYNAPTONEMAL COMPLEX PROTEIN 3
3:00	5c3l-A	4.9	8.6	93	134	8	MOLECULE:	NUP54
4:00	3zdo-C	4.9	3.1	63	63	10	MOLECULE:	PHOSPHOPROTEIN
5:00	6v8o-J	4.9	7.2	97	115	5	MOLECULE:	HIGH TEMPERATURE LETHAL PROTEIN 1
6:00	3u59-B	4.9	5.1	91	101	3	MOLECULE:	TROPOMYOSIN BETA CHAIN
7:00	5w5d-C	4.9	3.8	61	61	7	MOLECULE:	VESICLE-ASSOCIATED MEMBRANE PROTEIN 2
8:00	1sa0-E	4.9	3.8	85	124	4	MOLECULE:	TUBULIN ALPHA CHAIN
9:00	2wz7-A	4.9	4.4	66	66	14	MOLECULE:	UNCHARACTERIZED PROTEIN YBGF
10:00	2wpr-A	4.9	5.6	87	97	7	MOLECULE:	TRIMERIC AUTOTRANSPORTER ADHESIN FRAGMENT
11:00	4b2q-t	4.9	4.6	83	129	11	MOLECULE:	ATP SYNTHASE SUBUNIT ALPHA, MITOCHONDRIAL
12:00	4zdw-B	4.9	5.9	84	91	7	MOLECULE:	RAS-RELATED PROTEIN SEC4
13:00	3vir-D	4.9	1.2	63	63	8	MOLECULE:	MATING-TYPE SWITCHING PROTEIN SWI5
14:00	2d3e-D	4.9	4	86	130	3	MOLECULE:	GENERAL CONTROL PROTEIN GCN4 AND TROPOMYOSIN 1 AL

15:00	3p8c-E	4.9	3.5	64	64	6	MOLECULE:	CYTOPLASMIC FMR1-INTERACTING PROTEIN 1
16:00	3thf-B	4.9	8	139	174	11	MOLECULE:	PROTEIN SHROOM
17:00	1sfc-D	4.9	4.7	68	74	4	MOLECULE:	PROTEIN (SYNAPTOBREVIN 2)
18:00	2kz9-A	4.9	5.7	69	69	6	MOLECULE:	V-TYPE PROTON ATPASE SUBUNIT E
19:00	3a7o-E	4.9	1.9	60	60	8	MOLECULE:	AUTOPHAGY PROTEIN 16
20:00	3lt7-B	4.9	2.9	60	60	12	MOLECULE:	ADHESIN YADA
21:00	2xdj-F	4.9	2.7	61	61	8	MOLECULE:	UNCHARACTERIZED PROTEIN YBGF
22:00	8aia-B	4.9	3.7	82	85	5	MOLECULE:	CRESCENTIN
23:00	3j97-L	4.9	3.8	66	66	5	MOLECULE:	VESICLE-FUSING ATPASE
24:00:00	5wlq-A	4.9	4.4	82	104	10	MOLECULE:	CAPSID ASSEMBLY SCAFFOLDING PROTEIN,MYOSIN-7,MICR
25:00:00	6r0w-K	4.9	5.1	85	98	4	MOLECULE:	V-TYPE ATP SYNTHASE ALPHA CHAIN
26:00:00	7yes-C	4.9	4.8	70	99	11	MOLECULE:	RNA-DIRECTED RNA POLYMERASE L
27:00:00	3j96-L	4.9	3.7	66	66	5	MOLECULE:	VESICLE-FUSING ATPASE
28:00:00	3mqb-E	4.9	5.5	91	106	3	MOLECULE:	BONE MARROW STROMAL ANTIGEN 2
29:00:00	3hd7-B	4.9	4.5	87	98	1	MOLECULE:	VESICLE-ASSOCIATED MEMBRANE PROTEIN 2
30:00:00	5ccg-G	4.9	4.1	62	63	15	MOLECULE:	VESICLE-ASSOCIATED MEMBRANE PROTEIN 2
31:00:00	5apv-B	4.9	2.9	67	67	10	MOLECULE:	GENERAL CONTROL PROTEIN GCN4
32:00:00	3mqb-B	4.9	6.2	91	106	4	MOLECULE:	BONE MARROW STROMAL ANTIGEN 2
33:00:00	3fx0-B	4.9	3.7	71	71	8	MOLECULE:	NF-KAPPA-B ESSENTIAL MODULATOR
34:00:00	4qkv-A	4.9	6.2	80	101	6	MOLECULE:	POLYMERASE I AND TRANSCRIPT RELEASE FACTOR
35:00:00	4n21-D	4.9	3.8	91	123	9	MOLECULE:	GP2 ECTODOMAIN
36:00:00	3ipd-A	4.9	5.6	85	91	4	MOLECULE:	VESICLE-ASSOCIATED MEMBRANE PROTEIN 2
37:00:00	5cch-A	4.9	3.9	63	63	14	MOLECULE:	VESICLE-ASSOCIATED MEMBRANE PROTEIN 2
38:00:00	4li0-F	4.9	3.6	69	69	4	MOLECULE:	RAS-RELATED PROTEIN RAB-8A
39:00:00	5ccg-A	4.9	4	63	63	14	MOLECULE:	VESICLE-ASSOCIATED MEMBRANE PROTEIN 2
40:00:00	5cci-A	4.9	3.9	62	62	15	MOLECULE:	VESICLE-ASSOCIATED MEMBRANE PROTEIN 2
41:00:00	4w7z-A	4.9	3	64	64	9	MOLECULE:	B-CELL RECEPTOR-ASSOCIATED PROTEIN 29
42:00:00	7xho-U	4.9	7	83	140	6	MOLECULE:	CENTROMERE PROTEIN C
43:00:00	4w7z-C	4.9	1.6	59	59	10	MOLECULE:	B-CELL RECEPTOR-ASSOCIATED PROTEIN 29
44:00:00	4w7z-D	4.9	1.7	60	60	10	MOLECULE:	B-CELL RECEPTOR-ASSOCIATED PROTEIN 29
45:00:00	4w7z-B	4.9	3	64	64	9	MOLECULE:	B-CELL RECEPTOR-ASSOCIATED PROTEIN 29
46:00:00	3rl0-T	4.9	3.7	62	62	0	MOLECULE:	VESICLE-ASSOCIATED MEMBRANE PROTEIN 2
47:00:00	4w80-A	4.9	2.9	64	64	9	MOLECULE:	B-CELL RECEPTOR-ASSOCIATED PROTEIN 29
48:00:00	1ebo-A	4.9	5.4	92	112	5	MOLECULE:	EBOLA VIRUS ENVELOPE PROTEIN CHIMERA CONSISTING
49:00:00	8fed-E	4.8	17.6	97	358	5	MOLECULE:	VIRULENCE FACTOR MCE FAMILY PROTEIN
50:00:00	6ian-A	4.8	9.8	79	306	9	MOLECULE:	INTRAFLAGELLAR TRANSPORT PROTEIN 74
51:00:00	4orh-H	4.8	4	89	142	8	MOLECULE:	UBIQUITIN-CONJUGATING ENZYME E2 VARIANT 2
52:00:00	8g0q-B	4.8	12.1	103	230	8	MOLECULE:	KINETOCHORE PROTEIN NDC80
53:00:00	5opt-P	4.8	6.8	89	249	8	MOLECULE:	ACTIVATED PROTEIN KINASE C RECEPTOR, PUTATIVE
54:00:00	2ocy-A	4.8	8.8	83	149	12	MOLECULE:	RAB GUANINE NUCLEOTIDE EXCHANGE FACTOR SEC2
55:00:00	6ff7-G	4.8	6.9	92	132	5	MOLECULE:	RNA-BINDING MOTIF PROTEIN, X-LINKED 2
56:00:00	4aj5-Z	4.8	3.3	76	97	7	MOLECULE:	SPINDLE AND KINETOCHORE-ASSOCIATED PROTEIN 1
57:00:00	8amr-A	4.8	5	97	155	4	MOLECULE:	TRIPARTITE MOTIF-CONTAINING PROTEIN 3
58:00:00	7syg-H	4.8	3.8	71	237	4	MOLECULE:	18S RRNA
59:00:00	5jvp-B	4.8	5.1	84	90	5	MOLECULE:	CHIMERA PROTEIN OF CENTROMERE-ASSOCIATED PROTEIN
60:00:00	6ys4-A	4.8	4.4	87	103	11	MOLECULE:	SPINDLE ASSEMBLY ABNORMAL PROTEIN 6 HOMOLOG
61:00:00	7bjs-A	4.8	2.8	64	69	5	MOLECULE:	KINESIN HEAVY CHAIN
62:00:00	2v4h-A	4.8	5.9	84	98	5	MOLECULE:	NF-KAPPA-B ESSENTIAL MODULATOR
63:00:00	6cfz-B	4.8	5.3	65	65	9	MOLECULE:	ASK1
64:00:00	1qbz-B	4.8	2.7	63	114	6	MOLECULE:	PROTEIN (SIV GP41 ECTODOMAIN)
65:00:00	8h9v-K	4.8	5.9	90	198	3	MOLECULE:	ATP SYNTHASE F(0) COMPLEX SUBUNIT C1, MITOCHONDRI
66:00:00	8akr-Q	4.8	4.7	79	196	9	MOLECULE:	CHLOROPLAST MEMBRANE-ASSOCIATED 30 KD PROTEIN
67:00:00	3brt-D	4.8	4	62	62	6	MOLECULE:	INHIBITOR OF NUCLEAR FACTOR KAPPA-B KINASE SUBUNI
68:00:00	3gn4-A	4.8	4.1	78	129	8	MOLECULE:	MYOSIN-VI
69:00:00	8bh1-D	4.8	7.2	71	85	8	MOLECULE:	PROBABLE PEPTIDOGLYCAN GLYCOSYLTRANSFERASE FTSW
70:00:00	1qbz-A	4.8	3.1	62	102	5	MOLECULE:	PROTEIN (SIV GP41 ECTODOMAIN)

71:00:00	7wjt-C	4.8	1.7	59	59	7	MOLECULE:	ISOFORM 2 OF TRANSMEMBRANE PROTEIN 266
72:00:00	5mq0-t	4.8	8.8	89	438	9	MOLECULE:	YEAST UBC4 GENE FOR UBIQUITIN-CONJUGATING ENZYME
73:00:00	7yer-D	4.8	2.8	68	68	13	MOLECULE:	RNA-DIRECTED RNA POLYMERASE L
74:00:00	7y8r-Q	4.8	10	76	91	11	MOLECULE:	HISTONE H3
75:00:00	8fby-A	4.8	3.5	75	89	4	MOLECULE:	PLASMALEMMA VESICLE-ASSOCIATED PROTEIN
76:00:00	8q85-V	4.8	3.9	69	117	4	MOLECULE:	KINETOCHORE PROTEIN NDC80
77:00:00	6cfz-A	4.8	5.7	66	66	8	MOLECULE:	ASK1
78:00:00	1gmj-C	4.8	1.3	59	59	2	MOLECULE:	ATPASE INHIBITOR
79:00:00	7syo-H	4.8	6.1	73	237	4	MOLECULE:	18S RRNA
80:00:00	7naf-5	4.8	15.6	84	105	5	MOLECULE:	25S RRNA
81:00:00	6lth-Q	4.8	21.7	92	105	5	MOLECULE:	TRANSCRIPTION ACTIVATOR BRG1
82:00:00	8sdj-A	4.8	4.3	87	112	7	MOLECULE:	PYRIN
83:00:00	8sdj-D	4.8	4.3	88	112	7	MOLECULE:	PYRIN
84:00:00	6h9m-A	4.8	4.4	81	94	11	MOLECULE:	COILED-COIL DOMAIN-CONTAINING PROTEIN 90B, MITOCH
85:00:00	5xau-C	4.8	4.7	70	70	4	MOLECULE:	LAMININ SUBUNIT ALPHA-5
86:00:00	7yet-C	4.8	4.4	68	99	13	MOLECULE:	RNA-DIRECTED RNA POLYMERASE L
87:00:00	8sdj-C	4.8	4.3	88	112	7	MOLECULE:	PYRIN
88:00:00	5c9n-B	4.8	3.6	61	61	20	MOLECULE:	GEMININ COILED-COIL DOMAIN-CONTAINING PROTEIN 1
89:00:00	1io4-A	4.8	2.4	63	63	11	MOLECULE:	CSF-1R PROMOTER
90:00:00	7use-D	4.8	1.9	61	62	5	MOLECULE:	CYTOPLASMIC FMR1-INTERACTING PROTEIN 1
91:00:00	8sdj-B	4.8	4.3	87	112	7	MOLECULE:	PYRIN
92:00:00	8uww-G	4.8	3.9	76	82	7	MOLECULE:	ACTIN, ALPHA CARDIAC MUSCLE 1
93:00:00	8pr2-C	4.8	5.7	87	106	8	MOLECULE:	C-JUN-AMINO-TERMINAL KINASE-INTERACTING PROTEIN 3
94:00:00	7xr0-E	4.8	5.8	90	123	4	MOLECULE:	TUBULIN ALPHA-1B CHAIN
95:00:00	7xqy-E	4.8	5.8	90	123	3	MOLECULE:	TUBULIN ALPHA-1B CHAIN
96:00:00	3b5n-I	4.8	3.2	58	58	2	MOLECULE:	SYNAPTOBREVIN HOMOLOG 1
97:00:00	3rl0-H	4.8	3.6	61	61	0	MOLECULE:	VESICLE-ASSOCIATED MEMBRANE PROTEIN 2
98:00:00	2xzr-A	4.8	4.7	87	108	6	MOLECULE:	IMMUNOGLOBULIN-BINDING PROTEIN EIBD
99:00:00	3uf1-B	4.8	4.6	89	106	8	MOLECULE:	VIMENTIN
0:00	3mq7-J	4.8	6.5	87	99	5	MOLECULE:	BONE MARROW STROMAL ANTIGEN 2
1:00	6fia-C	4.8	6.2	80	89	4	MOLECULE:	LINE-1 RETROTRANSPOSABLE ELEMENT ORF1 PROTEIN
2:00	3q0x-A	4.8	10.2	83	206	6	MOLECULE:	CENTRIOLE PROTEIN
3:00	5y60-K	4.8	3.6	87	99	7	MOLECULE:	V-TYPE ATP SYNTHASE ALPHA CHAIN
4:00	2d3e-C	4.8	5.1	81	130	10	MOLECULE:	GENERAL CONTROL PROTEIN GCN4 AND TROPOMYOSIN 1 AL
5:00	3hd7-F	4.8	4.2	83	99	1	MOLECULE:	VESICLE-ASSOCIATED MEMBRANE PROTEIN 2
6:00	4eot-A	4.8	2.5	73	92	12	MOLECULE:	TRANSCRIPTION FACTOR MAFA
7:00	5toi-A	4.8	5.2	68	68	7	MOLECULE:	POLYMERASE COFACTOR VP35
8:00	6f63-A	4.8	5.7	85	96	7	MOLECULE:	SYNAPTONEMAL COMPLEX PROTEIN 1
9:00	3m0a-A	4.8	3	62	62	5	MOLECULE:	TNF RECEPTOR-ASSOCIATED FACTOR 2
10:00	4bry-A	4.8	3.1	68	69	6	MOLECULE:	GEMININ
11:00	3zdo-D	4.8	3	64	64	11	MOLECULE:	PHOSPHOPROTEIN
12:00	3zdo-B	4.8	2.7	62	62	13	MOLECULE:	PHOSPHOPROTEIN
13:00	3lt7-E	4.8	2.9	61	61	8	MOLECULE:	ADHESIN YADA
14:00	2q2f-A	4.8	5.5	71	72	7	MOLECULE:	SELENOPROTEIN S
15:00	5gmk-q	4.8	7.4	88	387	8	MOLECULE:	PRE-MRNA-SPLICING FACTOR 8
16:00	6zqm-b	4.8	6.6	103	209	7	MOLECULE:	ATP SYNTHASE SUBUNIT ALPHA, MITOCHONDRIAL
17:00	3a7o-A	4.8	2.1	59	59	12	MOLECULE:	AUTOPHAGY PROTEIN 16
18:00	8b8b-D	4.8	3.7	85	97	11	MOLECULE:	MUNIA BORNAVIRUS 1 PHOSPHOPROTEIN
19:00	3rk2-B	4.8	2.6	59	59	3	MOLECULE:	VESICLE-ASSOCIATED MEMBRANE PROTEIN 2
20:00	8aix-F	4.8	4.7	80	85	5	MOLECULE:	CRESCENTIN
21:00	3zdo-A	4.8	3.2	63	63	13	MOLECULE:	PHOSPHOPROTEIN
22:00	2n1t-B	4.8	5	71	72	4	MOLECULE:	VESICLE-ASSOCIATED MEMBRANE PROTEIN 2
23:00	4aj5-A	4.8	11	73	79	10	MOLECULE:	SPINDLE AND KINETOCHORE-ASSOCIATED PROTEIN 1
24:00:00	3rl0-F	4.8	3	61	61	5	MOLECULE:	VESICLE-ASSOCIATED MEMBRANE PROTEIN 2
25:00:00	3rl0-R	4.8	3	61	61	5	MOLECULE:	VESICLE-ASSOCIATED MEMBRANE PROTEIN 2
26:00:00	8aix-B	4.8	4.7	80	85	5	MOLECULE:	CRESCENTIN

27:00:00	2nps-A	4.8	3.3	63	63	5	MOLECULE:	VESICLE-ASSOCIATED MEMBRANE PROTEIN 4
28:00:00	3rk2-F	4.8	2.6	59	59	3	MOLECULE:	VESICLE-ASSOCIATED MEMBRANE PROTEIN 2
29:00:00	1kil-A	4.8	5	65	65	14	MOLECULE:	SYNAPTOBREVIN SNARE MOTIF
30:00:00	2n1t-A	4.8	4.8	69	69	13	MOLECULE:	VESICLE-ASSOCIATED MEMBRANE PROTEIN 2
31:00:00	3v6i-A	4.8	5	104	186	6	MOLECULE:	V-TYPE ATP SYNTHASE SUBUNIT E
32:00:00	1h8a-A	4.8	2.7	65	68	9	MOLECULE:	CAAT/ENHANCER BINDING PROTEIN BETA
33:00:00	4li0-E	4.8	3.5	64	64	5	MOLECULE:	RAS-RELATED PROTEIN RAB-8A
34:00:00	3u1a-C	4.8	3.9	76	84	4	MOLECULE:	SMOOTH MUSCLE TROPOMYOSIN ALPHA
35:00:00	3i00-A	4.8	3.4	73	76	5	MOLECULE:	HUNTINGTIN-INTERACTING PROTEIN 1
36:00:00	2zxx-A	4.8	7.9	69	70	6	MOLECULE:	GEMININ
37:00:00	2v66-D	4.8	6.3	91	111	10	MOLECULE:	NUCLEAR DISTRIBUTION PROTEIN NUDE-LIKE 1
38:00:00	4w80-B	4.8	3	64	64	9	MOLECULE:	B-CELL RECEPTOR-ASSOCIATED PROTEIN 29
39:00:00	4w80-D	4.8	1.6	58	58	10	MOLECULE:	B-CELL RECEPTOR-ASSOCIATED PROTEIN 29
40:00:00	2v66-C	4.8	6	93	111	10	MOLECULE:	NUCLEAR DISTRIBUTION PROTEIN NUDE-LIKE 1
41:00:00	1sfc-L	4.8	4.4	67	73	3	MOLECULE:	PROTEIN (SYNAPTOBREVIN 2)
42:00:00	3rl0-D	4.8	3.7	62	62	0	MOLECULE:	VESICLE-ASSOCIATED MEMBRANE PROTEIN 2
43:00:00	3rl0-b	4.8	3.6	61	61	0	MOLECULE:	VESICLE-ASSOCIATED MEMBRANE PROTEIN 2
44:00:00	3zdo-G	4.8	2.8	64	64	5	MOLECULE:	PHOSPHOPROTEIN
45:00:00	1urq-D	4.8	4.1	62	62	0	MOLECULE:	M-TOMOSYN ISOFORM
46:00:00	3rk3-D	4.8	4.3	65	65	3	MOLECULE:	VAMP2
47:00:00	1h89-B	4.8	4.9	64	64	6	MOLECULE:	CAAT/ENHANCER BINDING PROTEIN BETA
48:00:00	5jst-A	4.8	6.2	86	428	7	MOLECULE:	MALTOSE-BINDING PERIPLASMIC PROTEIN,MITOCHONDRIAL
49:00:00	8xwx-G	4.8	2.2	57	57	9	MOLECULE:	B-CELL RECEPTOR-ASSOCIATED PROTEIN 31
50:00:00	4w80-C	4.8	1.7	59	59	10	MOLECULE:	B-CELL RECEPTOR-ASSOCIATED PROTEIN 29
51:00:00	7apk-E	4.8	4.7	61	523	11	MOLECULE:	THO COMPLEX SUBUNIT 1
52:00:00	1joc-A	4.7	3.3	75	123	9	MOLECULE:	EARLY ENDOSOMAL AUTOANTIGEN 1
53:00:00	1aa0-A	4.7	4	74	113	8	MOLECULE:	FIBRITIN
54:00:00	7eeb-C	4.7	7.6	104	278	6	MOLECULE:	ENHANCED GREEN FLUORESCENT PROTEIN,CATION CHANNEL
55:00:00	6kn7-T	4.7	15.3	67	138	9	MOLECULE:	ACTIN, ALPHA SKELETAL MUSCLE
56:00:00	8at3-A	4.7	8.2	133	286	8	MOLECULE:	HAUS AUGMIN-LIKE COMPLEX SUBUNIT 1
57:00:00	4aj5-W	4.7	4.8	85	98	8	MOLECULE:	SPINDLE AND KINETOCHORE-ASSOCIATED PROTEIN 1
58:00:00	6v92-R	4.7	4.5	79	325	11	MOLECULE:	ACTIN-RELATED PROTEIN 7
59:00:00	4emc-A	4.7	3.6	70	151	6	MOLECULE:	MONOPOLIN COMPLEX SUBUNIT CSM1
60:00:00	8bd7-W	4.7	6.3	86	114	8	MOLECULE:	IFT88
61:00:00	7eaa-C	4.7	6	91	114	12	MOLECULE:	RB1-INDUCIBLE COILED-COIL PROTEIN 1
62:00:00	3uf1-D	4.7	4.6	90	112	7	MOLECULE:	VIMENTIN
63:00:00	3b5n-C	4.7	3.2	65	70	9	MOLECULE:	SYNAPTOBREVIN HOMOLOG 1
64:00:00	7s5u-B	4.7	7.8	96	180	6	MOLECULE:	KINESIN-LIKE PROTEIN KLP61F
65:00:00	5hmo-A	4.7	8	88	131	9	MOLECULE:	UNCONVENTIONAL MYOSIN-X
66:00:00	6xor-B	4.7	5.9	71	71	10	MOLECULE:	PROTEIN SWALLOW
67:00:00	4m3l-B	4.7	1.9	58	58	14	MOLECULE:	E3 UBIQUITIN-PROTEIN LIGASE TRIM63
68:00:00	2w6a-B	4.7	1.8	56	56	5	MOLECULE:	ARF GTPASE-ACTIVATING PROTEIN GIT1
69:00:00	8b4i-D	4.7	3.7	60	60	5	MOLECULE:	MITOCHONDRIAL IMPORT RECEPTOR SUBUNIT TOM40
70:00:00	7z47-F	4.7	5.9	67	68	9	MOLECULE:	ADAPTOR PROTEIN
71:00:00	6xor-A	4.7	6.2	71	71	8	MOLECULE:	PROTEIN SWALLOW
72:00:00	4i1l-A	4.7	2.5	62	62	5	MOLECULE:	FORKHEAD BOX PROTEIN P3
73:00:00	8jsm-D	4.7	4.2	68	68	12	MOLECULE:	RNA-DIRECTED RNA POLYMERASE L
74:00:00	5kc1-J	4.7	1.6	58	58	3	MOLECULE:	AUTOPHAGY-RELATED PROTEIN 38
75:00:00	7b93-Z	4.7	13	101	143	7	MOLECULE:	NADH-UBIQUINONE OXIDOREDUCTASE CHAIN 3
76:00:00	3p8c-D	4.7	4.3	77	204	8	MOLECULE:	CYTOPLASMIC FMR1-INTERACTING PROTEIN 1
77:00:00	8afl-E	4.7	3.3	68	68	13	MOLECULE:	CRESCENTIN
78:00:00	6cp3-Z	4.7	29.2	118	155	6	MOLECULE:	ATP SYNTHASE SUBUNIT 9, MITOCHONDRIAL
79:00:00	6cfz-E	4.7	6.8	66	68	8	MOLECULE:	ASK1
80:00:00	8q85-c	4.7	4.3	62	64	5	MOLECULE:	KINETOCHORE PROTEIN NDC80
81:00:00	7yet-D	4.7	2.7	68	68	13	MOLECULE:	RNA-DIRECTED RNA POLYMERASE L
82:00:00	8e2j-C	4.7	2.4	57	57	0	MOLECULE:	DIABLO IAP-BINDING MITOCHONDRIAL PROTEIN

83:00:00	8afm-K	4.7	3.1	68	68	9	MOLECULE:	CRESCENTIN
84:00:00	8afm-E	4.7	3.3	68	68	13	MOLECULE:	CRESCENTIN
85:00:00	8b4i-C	4.7	3.6	60	60	7	MOLECULE:	MITOCHONDRIAL IMPORT RECEPTOR SUBUNIT TOM40
86:00:00	7bv6-B	4.7	2.4	61	61	5	MOLECULE:	VESICLE-ASSOCIATED MEMBRANE PROTEIN 8
87:00:00	1io4-B	4.7	3.4	65	70	12	MOLECULE:	CSF-1R PROMOTER
88:00:00	4bwd-A	4.7	4.4	61	61	8	MOLECULE:	SHORT COILED-COIL PROTEIN
89:00:00	7y5c-b	4.7	4.8	93	145	4	MOLECULE:	ATP SYNTHASE SUBUNIT ALPHA
90:00:00	1hvv-C	4.7	3.5	66	66	3	MOLECULE:	SYNTAXIN 1A
91:00:00	8b8b-B	4.7	3	81	95	7	MOLECULE:	MUNIA BORNAVIRUS 1 PHOSPHOPROTEIN
92:00:00	8afm-B	4.7	3.4	77	77	8	MOLECULE:	CRESCENTIN
93:00:00	3iox-A	4.7	2.7	80	489	4	MOLECULE:	AGI/II
94:00:00	8b8b-C	4.7	3.2	85	95	7	MOLECULE:	MUNIA BORNAVIRUS 1 PHOSPHOPROTEIN
95:00:00	8at3-E	4.7	13	111	217	7	MOLECULE:	HAUS AUGMIN-LIKE COMPLEX SUBUNIT 1
96:00:00	8dbs-X	4.7	4.9	85	156	2	MOLECULE:	ATP SYNTHASE SUBUNIT ALPHA
97:00:00	3b5n-E	4.7	4.2	58	58	10	MOLECULE:	SYNAPTOBREVIN HOMOLOG 1
98:00:00	6dd8-D	4.7	4.2	87	117	3	MOLECULE:	SYNAPTONEMAL COMPLEX PROTEIN 3
99:00:00	1ik9-A	4.7	4.8	85	207	8	MOLECULE:	DNA REPAIR PROTEIN XRCC4
0:00	3mq7-F	4.7	5.6	86	99	2	MOLECULE:	BONE MARROW STROMAL ANTIGEN 2
1:00	3v6i-B	4.7	7.7	89	101	3	MOLECULE:	V-TYPE ATP SYNTHASE SUBUNIT E
2:00	3m06-D	4.7	2.8	62	62	6	MOLECULE:	TNF RECEPTOR-ASSOCIATED FACTOR 2
3:00	3zdo-E	4.7	2.7	62	62	13	MOLECULE:	PHOSPHOPROTEIN
4:00	2v66-B	4.7	5.8	91	111	12	MOLECULE:	NUCLEAR DISTRIBUTION PROTEIN NUDE-LIKE 1
5:00	3m0a-B	4.7	2.9	62	62	6	MOLECULE:	TNF RECEPTOR-ASSOCIATED FACTOR 2
6:00	3m06-F	4.7	3.5	62	62	5	MOLECULE:	TNF RECEPTOR-ASSOCIATED FACTOR 2
7:00	3h7z-A	4.7	3.3	61	61	10	MOLECULE:	ADHESIN YADA
8:00	3rl0-J	4.7	2.7	59	59	3	MOLECULE:	VESICLE-ASSOCIATED MEMBRANE PROTEIN 2
9:00	5apv-F	4.7	6.7	67	67	3	MOLECULE:	GENERAL CONTROL PROTEIN GCN4
10:00	3rl0-d	4.7	2.7	59	59	3	MOLECULE:	VESICLE-ASSOCIATED MEMBRANE PROTEIN 2
11:00	4c46-B	4.7	3.5	71	73	1	MOLECULE:	GENERAL CONTROL PROTEIN GCN4, GENERAL CONTROL PRO
12:00	3m06-A	4.7	3.6	62	62	3	MOLECULE:	TNF RECEPTOR-ASSOCIATED FACTOR 2
13:00	2wvr-B	4.7	6.7	70	74	6	MOLECULE:	GEMININ
14:00	2x7a-A	4.7	1.8	59	59	7	MOLECULE:	BONE MARROW STROMAL ANTIGEN 2
15:00	3m06-E	4.7	3.1	62	62	6	MOLECULE:	TNF RECEPTOR-ASSOCIATED FACTOR 2
16:00	7z47-D	4.7	5.1	76	87	9	MOLECULE:	ADAPTOR PROTEIN
17:00	5f5p-E	4.7	1.8	57	57	7	MOLECULE:	PROTEIN SHROOM2
18:00	4efa-E	4.7	4	74	225	4	MOLECULE:	V-TYPE PROTON ATPASE SUBUNIT C
19:00	1t2k-D	4.7	1.6	61	61	5	MOLECULE:	31-MER
20:00	4eb6-E	4.7	4.4	91	132	8	MOLECULE:	TUBULIN ALPHA CHAIN
21:00	4x1k-E	4.7	4.8	87	123	5	MOLECULE:	TUBULIN ALPHA CHAIN
22:00	2yo3-B	4.7	6.4	80	258	6	MOLECULE:	GENERAL CONTROL PROTEIN GCN4, PUTATIVE INNER MEMB
23:00	5d80-n	4.7	3.4	71	105	7	MOLECULE:	V-TYPE PROTON ATPASE CATALYTIC SUBUNIT A
24:00:00	3lt6-C	4.7	2.4	58	58	10	MOLECULE:	ADHESIN YADA
25:00:00	8pr3-B	4.7	4.1	82	105	11	MOLECULE:	C-JUN-AMINO-TERMINAL KINASE-INTERACTING PROTEIN 3
26:00:00	4m3l-C	4.7	1.9	57	57	14	MOLECULE:	E3 UBIQUITIN-PROTEIN LIGASE TRIM63
27:00:00	2v66-E	4.7	6.5	92	111	10	MOLECULE:	NUCLEAR DISTRIBUTION PROTEIN NUDE-LIKE 1
28:00:00	5apw-B	4.7	4.4	64	64	11	MOLECULE:	GENERAL CONTROL PROTEIN GCN4
29:00:00	8xwx-E	4.7	2.3	58	58	9	MOLECULE:	B-CELL RECEPTOR-ASSOCIATED PROTEIN 31
30:00:00	3fwc-B	4.7	3.9	75	83	4	MOLECULE:	CELL DIVISION CONTROL PROTEIN 31
31:00:00	4xa1-C	4.7	9.5	90	130	4	MOLECULE:	GP7-MYH7(1173-1238)-EB1 CHIMERA PROTEIN
32:00:00	6fln-B	4.6	23.4	98	367	5	MOLECULE:	E3 UBIQUITIN/ISG15 LIGASE TRIM25
33:00:00	6fkf-p	4.6	21.7	104	143	7	MOLECULE:	ATP SYNTHASE SUBUNIT ALPHA, CHLOROPLASTIC
34:00:00	6oei-A	4.6	5.9	96	243	9	MOLECULE:	SPINDLE POLE BODY COMPONENT SPC42,SIGMA-54-DEPEND
35:00:00	7uic-c	4.6	3.1	65	110	9	MOLECULE:	MEDIATOR OF RNA POLYMERASE II TRANSCRIPTION SUBUN
36:00:00	7w20-W	4.6	10	86	142	7	MOLECULE:	NADH DEHYDROGENASE [UBIQUINONE] FLAVOPROTEIN 1,
37:00:00	3jb9-i	4.6	10.3	97	161	5	MOLECULE:	PRE-MRNA-SPLICING FACTOR SPP42
38:00:00	6ppb-l	4.6	7.2	80	83	9	MOLECULE:	CAPSID VERTEX COMPONENT 1

39:00:00	7bgf-A	4.6	4.2	87	112	5	MOLECULE:	DNA ENDONUCLEASE RBBP8,CTIP/RBBP8
40:00:00	8tgt-B	4.6	4.7	82	90	5	MOLECULE:	M PROTEIN
41:00:00	8g0p-B	4.6	3.2	86	98	7	MOLECULE:	KINETOCHORE PROTEIN NDC80 HOMOLOG
42:00:00	3zdo-H	4.6	3.1	62	62	6	MOLECULE:	PHOSPHOPROTEIN
43:00:00	3hd7-H	4.6	4.1	62	62	0	MOLECULE:	VESICLE-ASSOCIATED MEMBRANE PROTEIN 2
44:00:00	5omb-C	4.6	9.5	85	98	11	MOLECULE:	REPLICATION FACTOR A PROTEIN 1
45:00:00	6id0-s	4.6	3.2	67	67	7	MOLECULE:	PRE-MRNA-PROCESSING-SPLICING FACTOR 8
46:00:00	5mqf-L	4.6	17.1	87	336	7	MOLECULE:	PRE-MRNA-PROCESSING-SPLICING FACTOR 8
47:00:00	8e2j-D	4.6	2.2	57	57	0	MOLECULE:	DIABLO IAP-BINDING MITOCHONDRIAL PROTEIN
48:00:00	4dl0-K	4.6	2.8	68	97	6	MOLECULE:	V-TYPE PROTON ATPASE SUBUNIT C
49:00:00	7wjt-D	4.6	1.7	56	56	7	MOLECULE:	ISOFORM 2 OF TRANSMEMBRANE PROTEIN 266
50:00:00	8yxr-B	4.6	5.6	68	69	1	MOLECULE:	PHOSPHOPROTEIN
51:00:00	6un9-A	4.6	2.6	66	69	11	MOLECULE:	UNCHARACTERIZED PROTEIN
52:00:00	5mw9-H	4.6	2.4	57	57	9	MOLECULE:	CENTROSOMIN
53:00:00	3pn7-D	4.6	5.8	68	69	6	MOLECULE:	MYOSIN HEAVY CHAIN
54:00:00	8swd-A	4.6	1.3	54	54	13	MOLECULE:	2-OXOGLUTARATE:ACCEPTOR OXIDOREDUCTASE
55:00:00	7sqk-A	4.6	10.9	88	278	9	MOLECULE:	HAUS AUGMIN-LIKE COMPLEX SUBUNIT 1
56:00:00	8owi-A	4.6	1.2	55	55	4	MOLECULE:	CENTROMERE-ASSOCIATED PROTEIN E
57:00:00	8aix-A	4.6	4	81	84	5	MOLECULE:	CRESCENTIN
58:00:00	7xfr-B	4.6	4.9	66	69	6	MOLECULE:	ISOFORM 2 OF WD REPEAT DOMAIN PHOSPHOINOSITIDE-IN
59:00:00	7wjt-A	4.6	1.4	55	55	9	MOLECULE:	ISOFORM 2 OF TRANSMEMBRANE PROTEIN 266
60:00:00	8aix-E	4.6	4	81	84	5	MOLECULE:	CRESCENTIN
61:00:00	3mqb-A	4.6	6.6	95	109	9	MOLECULE:	BONE MARROW STROMAL ANTIGEN 2
62:00:00	7nad-5	4.6	4.9	69	123	6	MOLECULE:	25S RRNA
63:00:00	8aia-A	4.6	4	81	84	5	MOLECULE:	CRESCENTIN
64:00:00	7uo4-B	4.6	2.7	78	187	6	MOLECULE:	RNA-DIRECTED RNA POLYMERASE
65:00:00	8afm-H	4.6	2.7	77	77	9	MOLECULE:	CRESCENTIN
66:00:00	8k86-B	4.6	2	57	58	11	MOLECULE:	NUCLEAR FACTOR INTERLEUKIN-3-REGULATED PROTEIN
67:00:00	8swd-B	4.6	1.1	54	54	13	MOLECULE:	2-OXOGLUTARATE:ACCEPTOR OXIDOREDUCTASE
68:00:00	8ahl-J	4.6	5.2	87	120	3	MOLECULE:	CRESCENTIN
69:00:00	8q3v-W	4.6	6	74	75	8	MOLECULE:	TETRAHYDROMETHANOPTERIN S-METHYLTRANSFERASE SUBUN
70:00:00	8afl-B	4.6	3.5	77	77	8	MOLECULE:	CRESCENTIN
71:00:00	1kil-B	4.6	2.5	59	59	3	MOLECULE:	SYNAPTOBREVIN SNARE MOTIF
72:00:00	8ajb-V	4.6	5.4	90	120	3	MOLECULE:	CRESCENTIN
73:00:00	5f5p-F	4.6	1.9	58	58	9	MOLECULE:	PROTEIN SHROOM2
74:00:00	5t58-B	4.6	4.2	73	190	4	MOLECULE:	KLLA0F02343P
75:00:00	4yv4-C	4.6	1.7	57	57	4	MOLECULE:	SPINDLE ASSEMBLY ABNORMAL PROTEIN 5
76:00:00	8pr3-C	4.6	3.4	81	106	2	MOLECULE:	C-JUN-AMINO-TERMINAL KINASE-INTERACTING PROTEIN 3
77:00:00	3ipd-D	4.6	4.5	63	63	5	MOLECULE:	VESICLE-ASSOCIATED MEMBRANE PROTEIN 2
78:00:00	3n7n-A	4.6	6.4	85	163	0	MOLECULE:	MONOPOLIN COMPLEX SUBUNIT CSM1
79:00:00	3k5b-B	4.6	5.3	86	100	5	MOLECULE:	V-TYPE ATP SYNTHASE, SUBUNIT (VAPC-THERM)
80:00:00	7jg7-b	4.6	6.2	95	138	5	MOLECULE:	ATP SYNTHASE SUBUNIT ALPHA
81:00:00	3uf1-C	4.6	5.5	87	109	8	MOLECULE:	VIMENTIN
82:00:00	6dd9-C	4.6	6.6	94	125	11	MOLECULE:	SYNAPTONEMAL COMPLEX PROTEIN 3
83:00:00	5y5z-K	4.6	8.1	88	99	5	MOLECULE:	V-TYPE ATP SYNTHASE ALPHA CHAIN
84:00:00	5wjb-C	4.6	4.7	82	112	13	MOLECULE:	CAPSID ASSEMBLY SCAFFOLDING PROTEIN,MYOSIN-7
85:00:00	2xv5-B	4.6	1.7	57	57	9	MOLECULE:	LAMIN-A/C
86:00:00	3n4x-B	4.6	3.6	69	151	7	MOLECULE:	MONOPOLIN COMPLEX SUBUNIT CSM1
87:00:00	6dd9-A	4.6	3.3	86	128	3	MOLECULE:	SYNAPTONEMAL COMPLEX PROTEIN 3
88:00:00	1t2k-C	4.6	3	62	62	3	MOLECULE:	31-MER
89:00:00	5y60-I	4.6	4.6	87	99	6	MOLECULE:	V-TYPE ATP SYNTHASE ALPHA CHAIN
90:00:00	3rl0-Z	4.6	2.5	58	58	3	MOLECULE:	VESICLE-ASSOCIATED MEMBRANE PROTEIN 2
91:00:00	3rl0-B	4.6	2.7	59	59	3	MOLECULE:	VESICLE-ASSOCIATED MEMBRANE PROTEIN 2
92:00:00	3rk3-B	4.6	2.9	62	62	3	MOLECULE:	VAMP2
93:00:00	2fyz-E	4.6	2.3	62	62	8	MOLECULE:	FUSION GLYCOPROTEIN F0
94:00:00	1l4a-B	4.6	4	79	82	4	MOLECULE:	SYNAPTOBREVIN

95:00:00	3rl0-V	4.6	2.7	58	58	3	MOLECULE:	VESICLE-ASSOCIATED MEMBRANE PROTEIN 2
96:00:00	3j99-L	4.6	4	66	66	6	MOLECULE:	VESICLE-FUSING ATPASE
97:00:00	1fos-E	4.6	1.9	60	60	3	MOLECULE:	DNA (5'-
98:00:00	3u59-A	4.6	4.7	86	100	7	MOLECULE:	TROPOMYOSIN BETA CHAIN
99:00:00	3m0a-C	4.6	3.2	62	62	6	MOLECULE:	TNF RECEPTOR-ASSOCIATED FACTOR 2
0:00	3mqb-F	4.6	4.8	86	102	8	MOLECULE:	BONE MARROW STROMAL ANTIGEN 2
1:00	1z56-A	4.6	3.4	76	77	8	MOLECULE:	LIGASE INTERACTING FACTOR 1
2:00	3lt6-E	4.6	2.2	57	57	9	MOLECULE:	ADHESIN YADA
3:00	1nwq-A	4.6	2.2	60	60	10	MOLECULE:	5'-
4:00	2zxx-D	4.6	7.2	65	65	5	MOLECULE:	GEMININ
5:00	3u59-C	4.6	4.5	87	101	8	MOLECULE:	TROPOMYOSIN BETA CHAIN
6:00	3m06-B	4.6	2.9	62	62	6	MOLECULE:	TNF RECEPTOR-ASSOCIATED FACTOR 2
7:00	3vh7-E	4.6	1.9	56	56	9	MOLECULE:	ENVELOPE GLYCOPROTEIN GP160
8:00	3lt7-F	4.6	2.5	59	59	12	MOLECULE:	ADHESIN YADA
9:00	2xv5-A	4.6	2.7	57	57	9	MOLECULE:	LAMIN-A/C
10:00	3a7o-F	4.6	2.3	58	58	5	MOLECULE:	AUTOPHAGY PROTEIN 16
11:00	2guv-A	4.6	2.7	56	56	4	MOLECULE:	MAJOR OUTER MEMBRANE LIPOPROTEIN
12:00	3ipd-H	4.6	4.5	63	63	5	MOLECULE:	VESICLE-ASSOCIATED MEMBRANE PROTEIN 2
13:00	3lt7-C	4.6	2.5	59	59	12	MOLECULE:	ADHESIN YADA
14:00	7bv6-G	4.6	3.8	68	74	9	MOLECULE:	VESICLE-ASSOCIATED MEMBRANE PROTEIN 8
15:00	3bat-B	4.6	5	71	81	4	MOLECULE:	MYOSIN HEAVY CHAIN, STRIATED MUSCLE/GENERAL CONTR
16:00	8th8-S	4.6	43.4	94	187	3	MOLECULE:	DYNEIN REGULATORY COMPLEX PROTEIN 1/2 N-TERMINAL
17:00	1kmi-Z	4.5	23.4	109	177	6	MOLECULE:	CHEMOTAXIS PROTEIN CHEY
18:00	6znl-n	4.5	16.2	144	343	10	MOLECULE:	ARP1 ACTIN RELATED PROTEIN 1 HOMOLOG A
19:00	8gb3-F	4.5	3.3	79	90	9	MOLECULE:	CHAPERONE PROTEIN DNAK
20:00	7emf-D	4.5	6.7	86	158	9	MOLECULE:	MEDIATOR OF RNA POLYMERASE II TRANSCRIPTION SUBUN
21:00	8u95-A	4.5	98.5	103	1009	9	MOLECULE:	MYOSIN HEAVY CHAIN, ISOFORM U
22:00	4jle-B	4.5	4.5	64	159	2	MOLECULE:	PHIST
23:00	1m1j-B	4.5	8	101	402	8	MOLECULE:	FIBRINOGEN ALPHA SUBUNIT
24:00:00	8h36-G	4.5	6	103	379	9	MOLECULE:	E3 UBIQUITIN-PROTEIN LIGASE RBX1
25:00:00	8q3v-g	4.5	9.1	76	76	5	MOLECULE:	TETRAHYDROMETHANOPTERIN S-METHYLTRANSFERASE SUBUN
26:00:00	3zx6-B	4.5	13.8	116	303	4	MOLECULE:	HAMP, METHYL-ACCEPTING CHEMOTAXIS PROTEIN I
27:00:00	8q85-X	4.5	9.5	68	68	10	MOLECULE:	KINETOCHORE PROTEIN NDC80
28:00:00	2b9b-B	4.5	10	82	482	7	MOLECULE:	FUSION GLYCOPROTEIN F0
29:00:00	7eqc-G	4.5	6.3	89	107	2	MOLECULE:	CYTOKINESIS DEFECT
30:00:00	8p0v-L	4.5	20.6	96	416	9	MOLECULE:	COILED-COIL DOMAIN-CONTAINING PROTEIN 93
31:00:00	5wq4-E	4.5	3.6	66	69	3	MOLECULE:	UBIQUITIN
32:00:00	8cra-D	4.5	24.6	75	93	16	MOLECULE:	FLORAL HOMEOTIC PROTEIN AGAMOUS
33:00:00	5a5t-A	4.5	21.4	93	599	8	MOLECULE:	EUKARYOTIC TRANSLATION INITIATION FACTOR 3 SUBUNI
34:00:00	8izl-E	4.5	2.7	58	58	9	MOLECULE:	PHOSPHOPROTEIN
35:00:00	6jn2-B	4.5	9.8	67	67	12	MOLECULE:	PROTEIN AF-10
36:00:00	8q3v-G	4.5	5.9	73	74	8	MOLECULE:	TETRAHYDROMETHANOPTERIN S-METHYLTRANSFERASE SUBUN
37:00:00	8yxo-E	4.5	2.7	58	58	9	MOLECULE:	PHOSPHOPROTEIN
38:00:00	8k8a-B	4.5	2	57	59	11	MOLECULE:	NUCLEAR FACTOR INTERLEUKIN-3-REGULATED PROTEIN
39:00:00	8k8a-A	4.5	4.2	61	62	11	MOLECULE:	NUCLEAR FACTOR INTERLEUKIN-3-REGULATED PROTEIN
40:00:00	2ix7-C	4.5	1.7	58	59	2	MOLECULE:	MYOSIN-5A
41:00:00	5kc1-F	4.5	5.7	61	61	5	MOLECULE:	AUTOPHAGY-RELATED PROTEIN 38
42:00:00	8hpo-H	4.5	8.4	95	233	8	MOLECULE:	TRANSCRIPTIONAL REGULATORY PROTEIN UME1
43:00:00	8sq9-B	4.5	2.6	80	186	6	MOLECULE:	RNA-DIRECTED RNA POLYMERASE
44:00:00	3f6n-B	4.5	5.9	71	74	11	MOLECULE:	VIRION-ASSOCIATED PROTEIN
45:00:00	5ed9-C	4.5	2.2	63	63	11	MOLECULE:	SUN DOMAIN-CONTAINING PROTEIN 2
46:00:00	6fkh-b	4.5	6.6	92	161	2	MOLECULE:	ATP SYNTHASE SUBUNIT A, CHLOROPLASTIC
47:00:00	8i3e-C	4.5	2.3	56	56	11	MOLECULE:	ELKS/RAB6-INTERACTING/CAST FAMILY MEMBER 1
48:00:00	8aix-V	4.5	4.3	79	84	6	MOLECULE:	CRESCENTIN
49:00:00	5zuv-A	4.5	4.6	80	130	6	MOLECULE:	SPIKE GLYCOPROTEIN,INHIBITOR EK1
50:00:00	3uf1-A	4.5	6.3	88	104	5	MOLECULE:	VIMENTIN

51:00:00	6dd8-C	4.5	3.8	67	123	7	MOLECULE:	SYNAPTONEMAL COMPLEX PROTEIN 3
52:00:00	1sa1-E	4.5	6.8	95	134	4	MOLECULE:	TUBULIN ALPHA CHAIN
53:00:00	2zxx-E	4.5	5.9	69	78	6	MOLECULE:	GEMININ
54:00:00	1wyy-B	4.5	2.6	79	124	10	MOLECULE:	E2 GLYCOPROTEIN
55:00:00	3rl0-N	4.5	2.6	57	57	4	MOLECULE:	VESICLE-ASSOCIATED MEMBRANE PROTEIN 2
56:00:00	8afl-A	4.5	4.5	76	77	9	MOLECULE:	CRESCENTIN
57:00:00	3uux-B	4.5	9	91	132	12	MOLECULE:	MITOCHONDRIA FISSION 1 PROTEIN
58:00:00	4cjd-A	4.5	18.5	78	123	6	MOLECULE:	NADA
59:00:00	3m06-C	4.5	3.2	62	62	10	MOLECULE:	TNF RECEPTOR-ASSOCIATED FACTOR 2
60:00:00	4kht-A	4.5	3.6	66	66	6	MOLECULE:	GP41 HELIX
61:00:00	8b8b-A	4.5	5.2	84	99	7	MOLECULE:	MUNIA BORNAVIRUS 1 PHOSPHOPROTEIN
62:00:00	7oz3-A	4.5	7.1	82	202	2	MOLECULE:	GNTR FAMILY TRANSCRIPTIONAL REGULATOR
63:00:00	4pxj-C	4.5	2.6	60	61	8	MOLECULE:	C-JUN-AMINO-TERMINAL KINASE-INTERACTING PROTEIN 3
64:00:00	2ahm-G	4.5	4	86	191	5	MOLECULE:	REPLICASE POLYPROTEIN 1AB, LIGHT CHAIN
65:00:00	3a7o-D	4.5	2.6	58	58	12	MOLECULE:	AUTOPHAGY PROTEIN 16
66:00:00	8aia-L	4.5	4.3	79	84	6	MOLECULE:	CRESCENTIN
67:00:00	3htk-A	4.5	1.8	60	60	3	MOLECULE:	STRUCTURAL MAINTENANCE OF CHROMOSOMES PROTEIN 5
68:00:00	6j9r-A	4.5	6.8	89	125	9	MOLECULE:	BRAIN TUMOR PROTEIN
69:00:00	7aej-B	4.5	2.8	84	145	12	MOLECULE:	ENVELOPE GLYCOPROTEIN GP160,ENVELOPE GLYCOPROTEIN
70:00:00	7xyz-B	4.4	29.6	117	456	7	MOLECULE:	TRIPARTITE MOTIF-CONTAINING PROTEIN 72
71:00:00	5cws-D	4.4	26.5	99	180	4	MOLECULE:	SAB-158 FAB LIGHT CHAIN
72:00:00	6xp5-G	4.4	6.2	86	122	6	MOLECULE:	MEDIATOR OF RNA POLYMERASE II TRANSCRIPTION SUBUN
73:00:00	8tek-B	4.4	22.8	101	208	10	MOLECULE:	DYNEIN REGULATORY COMPLEX PROTEIN 1/2 N-TERMINAL
74:00:00	5d80-G	4.4	6.1	87	223	8	MOLECULE:	V-TYPE PROTON ATPASE CATALYTIC SUBUNIT A
75:00:00	4tql-B	4.4	3.2	100	235	6	MOLECULE:	THREE HELIX BUNDLE
76:00:00	8fef-B	4.4	17	93	335	4	MOLECULE:	VIRULENCE FACTOR MCE FAMILY PROTEIN
77:00:00	6zls-A	4.4	3.2	80	292	18	MOLECULE:	HISTIDINE KINASE
78:00:00	8ixk-E	4.4	3.2	81	109	5	MOLECULE:	ATTACHMENT PROTEIN G3P
79:00:00	8fed-F	4.4	18.7	101	399	9	MOLECULE:	VIRULENCE FACTOR MCE FAMILY PROTEIN
80:00:00	6h3a-A	4.4	46.1	84	266	12	MOLECULE:	SWI/SNF-RELATED MATRIX-ASSOCIATED ACTIN-DEPENDENT
81:00:00	3hnw-A	4.4	4.5	91	128	7	MOLECULE:	UNCHARACTERIZED PROTEIN
82:00:00	4f61-I	4.4	6.6	91	234	5	MOLECULE:	TUBULIN ALPHA CHAIN
83:00:00	4jpp-D	4.4	29.9	120	129	13	MOLECULE:	MINOR SPIKE PROTEIN H
84:00:00	8w6b-H	4.4	2.7	56	57	14	MOLECULE:	TAX1-BINDING PROTEIN 1
85:00:00	8pqw-F	4.4	6.4	89	150	3	MOLECULE:	CYTOPLASMIC DYNEIN 1 HEAVY CHAIN 1
86:00:00	7xho-Q	4.4	5.2	74	164	3	MOLECULE:	CENTROMERE PROTEIN C
87:00:00	5odw-D	4.4	2.7	64	198	9	MOLECULE:	FERRIPYOVERDINE RECEPTOR
88:00:00	7vc4-H	4.4	10.4	76	107	5	MOLECULE:	MITOCHONDRIAL IMPORT RECEPTOR SUBUNIT TOM6 HOMOLO
89:00:00	8rtd-B	4.4	3.7	66	197	11	MOLECULE:	TRWJ PROTEIN
90:00:00	8k86-A	4.4	4.5	61	63	11	MOLECULE:	NUCLEAR FACTOR INTERLEUKIN-3-REGULATED PROTEIN
91:00:00	8owi-B	4.4	2.1	54	54	6	MOLECULE:	CENTROMERE-ASSOCIATED PROTEIN E
92:00:00	8rtd-A	4.4	3.6	67	197	10	MOLECULE:	TRWJ PROTEIN
93:00:00	7say-A	4.4	2.4	56	56	11	MOLECULE:	GENERAL CONTROL TRANSCRIPTION FACTOR GCN4/M PROTE
94:00:00	8b8a-A	4.4	7.5	85	95	7	MOLECULE:	PHOSPHOPROTEIN
95:00:00	8w6b-C	4.4	3	57	57	14	MOLECULE:	TAX1-BINDING PROTEIN 1
96:00:00	6wbp-A	4.4	2.2	56	56	7	MOLECULE:	SEPTIN-6
97:00:00	6qle-Q	4.4	10.9	94	224	11	MOLECULE:	CENTRAL KINETOCHORE SUBUNIT MCM16,CENTRAL KINETOC
98:00:00	8aix-K	4.4	4.1	72	72	3	MOLECULE:	CRESCENTIN
99:00:00	7use-E	4.4	4.2	79	155	9	MOLECULE:	CYTOPLASMIC FMR1-INTERACTING PROTEIN 1
0:00	8ftk-A	4.4	30.1	103	1584	2	MOLECULE:	5'-3' RNA HELICASE-LIKE PROTEIN
1:00	7w35-W	4.4	13.4	108	142	8	MOLECULE:	NADH DEHYDROGENASE [UBIQUINONE] FLAVOPROTEIN 1,
2:00	7uo4-D	4.4	8.8	82	187	5	MOLECULE:	RNA-DIRECTED RNA POLYMERASE
3:00	6zr2-Z	4.4	13	90	141	2	MOLECULE:	NADH-UBIQUINONE OXIDOREDUCTASE CHAIN 3
4:00	7tkd-U	4.4	7.1	92	155	2	MOLECULE:	ATP SYNTHASE SUBUNIT 9, MITOCHONDRIAL
5:00	2fxo-B	4.4	9.5	100	125	10	MOLECULE:	MYOSIN HEAVY CHAIN, CARDIAC MUSCLE BETA ISOFORM
6:00	3hkb-E	4.4	4.6	86	124	6	MOLECULE:	TUBULIN ALPHA CHAIN

7:00	3j98-L	4.4	4.1	66	66	6	MOLECULE:	VESICLE-FUSING ATPASE
8:00	3j98-K	4.4	3.8	61	61	15	MOLECULE:	VESICLE-FUSING ATPASE
9:00	3j96-K	4.4	3.9	61	61	15	MOLECULE:	VESICLE-FUSING ATPASE
10:00	3j99-K	4.4	3.8	61	61	15	MOLECULE:	VESICLE-FUSING ATPASE
11:00	7lsy-F	4.4	4.6	92	201	7	MOLECULE:	X-RAY REPAIR CROSS-COMPLEMENTING PROTEIN 6
12:00	1fos-H	4.4	4	58	58	3	MOLECULE:	DNA (5'-
13:00	3hhm-B	4.4	5.1	100	247	6	MOLECULE:	PHOSPHATIDYLINOSITOL-4,5-BISPHOSPHATE 3-KINASE
14:00	7xhn-K	4.4	13.9	101	230	10	MOLECULE:	CENTROMERE PROTEIN O
15:00	4m3l-D	4.4	1.7	53	53	9	MOLECULE:	E3 UBIQUITIN-PROTEIN LIGASE TRIM63
16:00	8aix-L	4.4	4.3	79	84	6	MOLECULE:	CRESCENTIN
17:00	4g2k-A	4.4	21.6	87	110	9	MOLECULE:	GENERAL CONTROL PROTEIN GCN4, ENVELOPE GLYCOPROTE
18:00	2p22-A	4.3	6.8	79	168	6	MOLECULE:	SUPPRESSOR PROTEIN STP22 OF TEMPERATURE-SENSITIVE
19:00	5vpe-A	4.3	4.1	67	67	4	MOLECULE:	PROTEIN FOSB
20:00	8i03-G	4.3	7.4	111	166	5	MOLECULE:	PAIRED AMPHIPATHIC HELIX PROTEIN PST1
21:00	5oqm-h	4.3	7.9	79	131	5	MOLECULE:	DNA-DIRECTED RNA POLYMERASE II SUBUNIT RPB1
22:00	3e98-A	4.3	2.6	71	178	10	MOLECULE:	GAF DOMAIN OF UNKNOWN FUNCTION
23:00	7x5e-B	4.3	7.5	75	106	7	MOLECULE:	TRANSCRIPTION FACTOR MAFG
24:00:00	5j9u-G	4.3	25.8	91	297	7	MOLECULE:	HISTONE ACETYLTRANSFERASE ESA1
25:00:00	8q7e-J	4.3	6	71	895	6	MOLECULE:	CULLIN-9
26:00:00	4xa6-A	4.3	6.6	78	168	8	MOLECULE:	GP7-MYH7(1777-1855)-EB1 CHIMERA PROTEIN
27:00:00	3viq-B	4.3	10	74	85	11	MOLECULE:	SWI5-DEPENDENT RECOMBINATION DNA REPAIR PROTEIN 1
28:00:00	8aix-Q	4.3	6.4	95	183	7	MOLECULE:	CRESCENTIN
29:00:00	8b8d-D	4.3	4	85	103	8	MOLECULE:	PHOSPHOPROTEIN
30:00:00	8q0j-Z	4.3	11.3	99	142	11	MOLECULE:	NADH-UBIQUINONE OXIDOREDUCTASE CHAIN 3
31:00:00	6v85-C	4.3	4.2	68	74	10	MOLECULE:	RNA-DIRECTED RNA POLYMERASE L
32:00:00	8ovw-B	4.3	7.3	86	107	8	MOLECULE:	CENTROMERE-BINDING PROTEIN 1
33:00:00	7tmp-H	4.3	9.2	79	86	8	MOLECULE:	H(+)-TRANSPORTING TWO-SECTOR ATPASE
34:00:00	6j6g-c	4.3	7.9	89	436	8	MOLECULE:	PRE-MRNA-SPLICING FACTOR 8
35:00:00	2wss-T	4.3	9.1	79	86	1	MOLECULE:	ATP SYNTHASE SUBUNIT ALPHA, MITOCHONDRIAL
36:00:00	3hd7-D	4.3	3.4	58	63	2	MOLECULE:	VESICLE-ASSOCIATED MEMBRANE PROTEIN 2
37:00:00	8urb-B	4.3	3.6	78	184	9	MOLECULE:	NSP12
38:00:00	8gwb-D	4.3	8.3	80	186	6	MOLECULE:	RNA-DIRECTED RNA POLYMERASE
39:00:00	7uo7-D	4.3	9.3	84	186	6	MOLECULE:	RNA-DIRECTED RNA POLYMERASE
40:00:00	7r7c-J	4.3	8.5	69	96	6	MOLECULE:	25S RRNA
41:00:00	8t0p-B	4.3	2.1	70	107	9	MOLECULE:	INNER KINETOCHORE SUBUNIT AME1
42:00:00	8x01-E	4.3	2.5	54	54	9	MOLECULE:	RNA-DIRECTED RNA POLYMERASE L
43:00:00	7qog-A	4.3	9.9	97	657	6	MOLECULE:	PORTAL PROTEIN GP20
44:00:00	7uv8-U	4.3	6.5	93	161	8	MOLECULE:	UBIQUITIN-CONJUGATING ENZYME E2 2
45:00:00	8cra-F	4.3	13.6	68	93	16	MOLECULE:	FLORAL HOMEOTIC PROTEIN AGAMOUS
46:00:00	5gna-B	4.3	2.9	56	56	5	MOLECULE:	FLAGELLAR PROTEIN FLIT
47:00:00	3ghg-D	4.3	8.2	84	174	8	MOLECULE:	FIBRINOGEN ALPHA CHAIN
48:00:00	2x7r-C	4.3	3.7	58	58	7	MOLECULE:	TRANSMEMBRANE PROTEIN GP41
49:00:00	7w2l-W	4.3	13.2	107	142	8	MOLECULE:	NADH DEHYDROGENASE [UBIQUINONE] FLAVOPROTEIN 1,
50:00:00	6dd9-B	4.3	8.5	98	127	4	MOLECULE:	SYNAPTONEMAL COMPLEX PROTEIN 3
51:00:00	2ocy-B	4.3	6.5	91	149	8	MOLECULE:	RAB GUANINE NUCLEOTIDE EXCHANGE FACTOR SEC2
52:00:00	2fxo-C	4.3	5.6	91	127	10	MOLECULE:	MYOSIN HEAVY CHAIN, CARDIAC MUSCLE BETA ISOFORM
53:00:00	3hke-E	4.3	4.6	89	124	8	MOLECULE:	TUBULIN ALPHA CHAIN
54:00:00	5noj-H	4.3	6.9	91	136	0	MOLECULE:	ACTIN, ALPHA SKELETAL MUSCLE
55:00:00	6gak-C	4.3	6.4	95	131	16	MOLECULE:	OUTER CAPSID PROTEIN SIGMA-1
56:00:00	6gak-A	4.3	7.2	95	129	12	MOLECULE:	OUTER CAPSID PROTEIN SIGMA-1
57:00:00	2e7s-C	4.3	4	84	118	8	MOLECULE:	RAB GUANINE NUCLEOTIDE EXCHANGE FACTOR SEC2
58:00:00	8hhg-L	4.3	10.3	78	81	13	MOLECULE:	CELL DIVISION PROTEIN FTSQ
59:00:00	2wty-A	4.3	4.4	77	94	4	MOLECULE:	TRANSCRIPTION FACTOR MAFB
60:00:00	7vby-H	4.3	5.9	56	56	9	MOLECULE:	TRANSLOCASE OF THE OUTER MEMBRANE
61:00:00	4bhv-C	4.3	1.7	57	57	5	MOLECULE:	PHOSPHOPROTEIN
62:00:00	7w4d-W	4.3	14.7	112	142	9	MOLECULE:	NADH DEHYDROGENASE [UBIQUINONE] FLAVOPROTEIN 1,

63:00:00	3h01-A	4.3	1.3	54	54	6	MOLECULE:	ENVELOPE GLYCOPROTEIN GP160
64:00:00	8h9r-K	4.3	5.8	97	198	2	MOLECULE:	ATP SYNTHASE F(0) COMPLEX SUBUNIT B1, MITOCHONDRI
65:00:00	8afm-A	4.3	3	72	77	6	MOLECULE:	CRESCENTIN
66:00:00	6e95-B	4.3	19.3	94	229	5	MOLECULE:	STAPHYLOCOCCUS AUREUS AGRC HISTIDINE KINASE MODUL
67:00:00	6e52-B	4.3	20.1	94	229	6	MOLECULE:	STAPHYLOCOCCUS AUREUS AGRC HISTIDINE KINASE MODUL
68:00:00	6h9l-A	4.3	6.7	92	127	3	MOLECULE:	UNCHARACTERIZED PROTEIN
69:00:00	6ewy-A	4.2	26	104	201	9	MOLECULE:	PEPTIDOGLYCAN ENDOPEPTIDASE RIPA
70:00:00	6lk8-a	4.2	23.6	103	1272	8	MOLECULE:	MGC83295 PROTEIN
71:00:00	6b85-A	4.2	3.3	70	215	16	MOLECULE:	TMHC4_R
72:00:00	5yfp-C	4.2	5.7	84	790	10	MOLECULE:	EXOCYST COMPLEX COMPONENT SEC3
73:00:00	6wuc-K	4.2	18.5	105	235	14	MOLECULE:	INNER KINETOCHORE SUBUNIT MCM16
74:00:00	5wkq-A	4.2	4.8	92	166	10	MOLECULE:	INVASIN IPAB
75:00:00	8srm-B	4.2	10	90	235	8	MOLECULE:	RB1-INDUCIBLE COILED-COIL PROTEIN 1
76:00:00	8r1a-A	4.2	48.2	142	587	7	MOLECULE:	GUANYLATE BINDING PROTEIN 1
77:00:00	3hgf-A	4.2	3.7	82	98	9	MOLECULE:	RHOPTRY PROTEIN FRAGMENT
78:00:00	6exn-D	4.2	9.4	86	97	10	MOLECULE:	U2 SNRNA
79:00:00	4aj5-U	4.2	3.3	79	100	8	MOLECULE:	SPINDLE AND KINETOCHORE-ASSOCIATED PROTEIN 1
80:00:00	6id0-L	4.2	5.4	91	475	13	MOLECULE:	PRE-MRNA-PROCESSING-SPLICING FACTOR 8
81:00:00	2wz7-B	4.2	3.3	61	67	15	MOLECULE:	UNCHARACTERIZED PROTEIN YBGF
82:00:00	6wmz-A	4.2	3.6	97	299	4	MOLECULE:	SPLICING FACTOR, PROLINE- AND GLUTAMINE-RICH
83:00:00	5gmk-c	4.2	8.1	90	436	7	MOLECULE:	PRE-MRNA-SPLICING FACTOR 8
84:00:00	8ahl-C	4.2	71.9	152	234	7	MOLECULE:	CRESCENTIN
85:00:00	8x01-B	4.2	3.5	63	69	8	MOLECULE:	RNA-DIRECTED RNA POLYMERASE L
86:00:00	8afl-F	4.2	3.2	59	59	17	MOLECULE:	CRESCENTIN
87:00:00	8izm-B	4.2	5.8	68	69	1	MOLECULE:	PHOSPHOPROTEIN
88:00:00	8afm-F	4.2	3.2	59	59	17	MOLECULE:	CRESCENTIN
89:00:00	8ow0-U	4.2	5.5	75	184	8	MOLECULE:	C0N3 DNA
90:00:00	1eq7-A	4.2	2.5	56	56	13	MOLECULE:	OUTER MEMBRANE LIPOPROTEIN
91:00:00	8gwi-B	4.2	4.5	82	187	1	MOLECULE:	RNA-DIRECTED RNA POLYMERASE
92:00:00	6vq6-M	4.2	4.8	86	114	5	MOLECULE:	ATPASE H+-TRANSPORTING V1 SUBUNIT A
93:00:00	8tes-E	4.2	8	79	84	6	MOLECULE:	LARGE TEGUMENT PROTEIN DENEDDYLASE
94:00:00	6exn-O	4.2	17.4	82	320	7	MOLECULE:	U2 SNRNA
95:00:00	5dfz-D	4.2	6.3	96	341	11	MOLECULE:	VACUOLAR PROTEIN SORTING-ASSOCIATED PROTEIN 38
96:00:00	8yxr-E	4.2	2.5	54	54	9	MOLECULE:	PHOSPHOPROTEIN
97:00:00	8gwg-B	4.2	4.5	82	187	1	MOLECULE:	RNA-DIRECTED RNA POLYMERASE
98:00:00	7jtk-F	4.2	8.9	97	287	3	MOLECULE:	FLAGELLAR RADIAL SPOKE PROTEIN 1
99:00:00	8gwf-B	4.2	4.5	82	187	1	MOLECULE:	RNA-DIRECTED RNA POLYMERASE
0:00	8cra-E	4.2	3.7	63	96	6	MOLECULE:	FLORAL HOMEOTIC PROTEIN AGAMOUS
1:00	3a5t-B	4.2	2.3	68	93	4	MOLECULE:	TRANSCRIPTION FACTOR MAFG
2:00	8cra-B	4.2	2.9	61	91	7	MOLECULE:	FLORAL HOMEOTIC PROTEIN AGAMOUS
3:00	8afm-L	4.2	3.1	59	59	5	MOLECULE:	CRESCENTIN
4:00	2xnx-M	4.2	6.6	90	107	8	MOLECULE:	FIBRINOGEN ALPHA CHAIN
5:00	6j5i-b	4.2	9.4	99	209	6	MOLECULE:	ATP SYNTHASE SUBUNIT ALPHA, MITOCHONDRIAL
6:00	7nju-b	4.2	4.8	63	63	6	MOLECULE:	ATP SYNTHASE SUBUNIT C
7:00	7vpx-J	4.2	5.3	54	54	4	MOLECULE:	SPLICING FACTOR 3A SUBUNIT 2
8:00	4a7h-H	4.2	8.4	98	136	4	MOLECULE:	ACTIN, ALPHA SKELETAL MUSCLE
9:00	5noj-F	4.2	9.5	99	136	0	MOLECULE:	ACTIN, ALPHA SKELETAL MUSCLE
10:00	3j4k-G	4.2	8.4	98	136	0	MOLECULE:	ACTIN, ALPHA SKELETAL MUSCLE
11:00	6xby-N	4.2	3.4	82	108	2	MOLECULE:	V-TYPE PROTON ATPASE CATALYTIC SUBUNIT A
12:00	4a7f-H	4.2	8.4	98	136	4	MOLECULE:	ACTIN, ALPHA SKELETAL MUSCLE
13:00	4a7l-H	4.2	8.4	98	136	4	MOLECULE:	ACTIN, ALPHA SKELETON MUSCLE
14:00	4jol-A	4.2	2.5	57	60	7	MOLECULE:	PROTEIN CBFA2T1
15:00	8akr-v	4.2	4.9	79	196	11	MOLECULE:	CHLOROPLAST MEMBRANE-ASSOCIATED 30 KD PROTEIN
16:00	4bhv-A	4.2	1.7	57	57	5	MOLECULE:	PHOSPHOPROTEIN
17:00	8dd0-P	4.2	5.8	70	75	4	MOLECULE:	ACTIN, ALPHA CARDIAC MUSCLE 1
18:00	7znk-M	4.2	49.5	80	549	8	MOLECULE:	RNA

19:00	7znl-M	4.2	53.3	82	549	6	MOLECULE:	THO COMPLEX SUBUNIT 1
20:00	4xa1-B	4.2	30.7	115	155	8	MOLECULE:	GP7-MYH7(1173-1238)-EB1 CHIMERA PROTEIN
21:00	2yny-C	4.2	5.3	76	105	5	MOLECULE:	GENERAL CONTROL PROTEIN GCN4, PUTATIVE INNER MEMB
22:00	6cfz-C	4.1	6.4	76	79	7	MOLECULE:	ASK1
23:00	5y06-A	4.1	25.3	99	235	9	MOLECULE:	MSMEG_4306
24:00:00	3na7-A	4.1	24.8	106	237	7	MOLECULE:	HP0958
25:00:00	4jio-B	4.1	4.7	71	320	8	MOLECULE:	BRO1
26:00:00	5oqj-1	4.1	20.7	137	491	4	MOLECULE:	DNA-DIRECTED RNA POLYMERASE II SUBUNIT RPB1
27:00:00	7q83-D	4.1	3.9	64	156	11	MOLECULE:	EXOCYST COMPLEX COMPONENT SEC3
28:00:00	6zsi-D	4.1	4.3	79	133	8	MOLECULE:	RAS-RELATED PROTEIN RAB-8A
29:00:00	6b3o-A	4.1	8	89	344	9	MOLECULE:	SPIKE GLYCOPROTEIN
30:00:00	8rgr-Z	4.1	14.4	106	141	6	MOLECULE:	NADH DEHYDROGENASE [UBIQUINONE] IRON-SULFUR PROTE
31:00:00	8rgp-Z	4.1	14.4	108	141	6	MOLECULE:	NADH DEHYDROGENASE [UBIQUINONE] IRON-SULFUR PROTE
32:00:00	8jsn-D	4.1	3.8	63	68	10	MOLECULE:	RNA-DIRECTED RNA POLYMERASE L
33:00:00	6ucv-J	4.1	4.5	55	55	7	MOLECULE:	MITOCHONDRIAL IMPORT RECEPTOR SUBUNIT TOM40
34:00:00	8gwo-D	4.1	8.2	82	186	4	MOLECULE:	RNA-DIRECTED RNA POLYMERASE
35:00:00	7sxk-j	4.1	5.8	81	578	7	MOLECULE:	PORTAL PROTEIN
36:00:00	8ajb-I	4.1	5.5	90	120	8	MOLECULE:	CRESCENTIN
37:00:00	7w91-A	4.1	2.3	53	53	6	MOLECULE:	CENTROSOMAL PROTEIN OF 63 KDA
38:00:00	8i03-E	4.1	12.9	97	169	4	MOLECULE:	PAIRED AMPHIPATHIC HELIX PROTEIN PST1
39:00:00	8ahl-I	4.1	5.5	90	120	9	MOLECULE:	CRESCENTIN
40:00:00	7usc-C	4.1	3.7	78	190	8	MOLECULE:	CYTOPLASMIC FMR1-INTERACTING PROTEIN 1
41:00:00	8ajb-U	4.1	5.5	90	120	9	MOLECULE:	CRESCENTIN
42:00:00	6od2-A	4.1	1.8	53	53	9	MOLECULE:	SPINDLE POLE BODY COMPONENT SPC42
43:00:00	1kfm-A	4.1	2.2	50	50	8	MOLECULE:	MAJOR OUTER MEMBRANE LIPOPROTEIN
44:00:00	7w91-H	4.1	1.8	52	52	6	MOLECULE:	CENTROSOMAL PROTEIN OF 63 KDA
45:00:00	6f1t-M	4.1	14.2	146	587	0	MOLECULE:	ARP1 ACTIN RELATED PROTEIN 1 HOMOLOG A
46:00:00	5to7-A	4.1	9.7	99	141	10	MOLECULE:	NUCLEOPROTEIN TPR
47:00:00	4zxq-D	4.1	5.7	87	140	6	MOLECULE:	TAIL NEEDLE PROTEIN GP26
48:00:00	6gak-B	4.1	7.6	93	131	5	MOLECULE:	OUTER CAPSID PROTEIN SIGMA-1
49:00:00	6snz-C	4.1	6.5	92	146	4	MOLECULE:	PRELAMIN-A/C
50:00:00	5to6-A	4.1	6.7	96	141	8	MOLECULE:	NUCLEOPROTEIN TPR
51:00:00	3lt6-B	4.1	3.1	58	61	7	MOLECULE:	ADHESIN YADA
52:00:00	5tvb-B	4.1	7.9	98	140	9	MOLECULE:	NUCLEOPROTEIN TPR
53:00:00	8hhh-L	4.1	10.4	76	81	12	MOLECULE:	CELL DIVISION PROTEIN FTSQ
54:00:00	3rrr-H	4.1	5.6	89	359	7	MOLECULE:	FUSION GLYCOPROTEIN F0
55:00:00	5nol-G	4.1	7.3	90	135	0	MOLECULE:	CARDIAC MUSCLE ALPHA ACTIN 1
56:00:00	5nol-F	4.1	7.4	95	135	0	MOLECULE:	CARDIAC MUSCLE ALPHA ACTIN 1
57:00:00	5to7-D	4.1	5	86	141	10	MOLECULE:	NUCLEOPROTEIN TPR
58:00:00	6tda-L	4.1	7.4	86	596	9	MOLECULE:	HISTONE H3.2
59:00:00	3swf-B	4.1	3.3	56	58	5	MOLECULE:	CGMP-GATED CATION CHANNEL ALPHA-1
60:00:00	3j97-K	4.1	3.8	61	61	15	MOLECULE:	VESICLE-FUSING ATPASE
61:00:00	6o7x-J	4.1	2.8	79	105	4	MOLECULE:	V-TYPE PROTON ATPASE SUBUNIT C
62:00:00	1fe6-C	4.1	1.1	49	49	8	MOLECULE:	TETRABRACHION
63:00:00	8bef-u	4.1	5.5	55	55	4	MOLECULE:	NADH-UBIQUINONE OXIDOREDUCTASE CHAIN 3
64:00:00	2w6a-A	4.1	3.4	57	61	16	MOLECULE:	ARF GTPASE-ACTIVATING PROTEIN GIT1
65:00:00	7w91-G	4.1	2.3	53	53	6	MOLECULE:	CENTROSOMAL PROTEIN OF 63 KDA
66:00:00	1ik7-A	4.1	1.5	52	52	10	MOLECULE:	PROBABLE SERINE/THREONINE-PROTEIN KINASE PELLE
67:00:00	1ebo-E	4.1	19.2	89	111	6	MOLECULE:	EBOLA VIRUS ENVELOPE PROTEIN CHIMERA CONSISTING
68:00:00	1ebo-D	4.1	21	90	112	4	MOLECULE:	EBOLA VIRUS ENVELOPE PROTEIN CHIMERA CONSISTING
69:00:00	6pfp-D	4.1	41	116	156	7	MOLECULE:	MYOSIN-7 FUSED TO GP7 AND EB1
70:00:00	2yny-B	4.1	4.7	75	104	5	MOLECULE:	GENERAL CONTROL PROTEIN GCN4, PUTATIVE INNER MEMB
71:00:00	6a68-A	4	19.4	90	162	7	MOLECULE:	CALCIUM-DEPENDENT SECRETION ACTIVATOR 1
72:00:00	6y1y-B	4	4.5	79	128	4	MOLECULE:	CHEA
73:00:00	8ovw-U	4	6.9	78	184	8	MOLECULE:	CENTROMERE-BINDING PROTEIN 1
74:00:00	5twv-B	4	27.3	134	1375	9	MOLECULE:	ATP-SENSITIVE INWARD RECTIFIER POTASSIUM CHANNEL

75:00:00	1jad-A	4	3.2	109	242	6	MOLECULE:	PHOSPHOLIPASE C BETA
76:00:00	8pmq-9	4	4.2	79	412	14	MOLECULE:	E3 UBIQUITIN-PROTEIN LIGASE RMD5
77:00:00	7qoo-H	4	21.4	109	210	8	MOLECULE:	CENTROMERE PROTEIN C
78:00:00	4p1m-B	4	3.7	74	107	12	MOLECULE:	CELL DIVISION PROTEIN ZAPA
79:00:00	7mge-B	4	6.4	82	442	9	MOLECULE:	WD REPEAT-CONTAINING PROTEIN 41
80:00:00	8wpe-D	4	4.1	63	69	10	MOLECULE:	DNA POLYMERASE
81:00:00	8afz-B	4	9	104	376	9	MOLECULE:	SORTING NEXIN-1
82:00:00	6z6o-C	4	14.7	104	548	6	MOLECULE:	HISTONE DEACETYLASE HDA1
83:00:00	2xqh-A	4	8.7	101	258	4	MOLECULE:	IMMUNOGLOBULIN-BINDING PROTEIN EIBD
84:00:00	5gai-B	4	6.5	89	721	9	MOLECULE:	PORTAL PROTEIN
85:00:00	8aia-K	4	3.9	68	72	3	MOLECULE:	CRESCENTIN
86:00:00	8yxo-C	4	3.3	58	91	10	MOLECULE:	PHOSPHOPROTEIN
87:00:00	8aix-U	4	3.9	68	72	3	MOLECULE:	CRESCENTIN
88:00:00	3jb9-W	4	6.4	93	426	9	MOLECULE:	PRE-MRNA-SPLICING FACTOR SPP42
89:00:00	7z4f-F	4	7	68	68	10	MOLECULE:	PUTATIVE STRUCTURAL PROTEIN
90:00:00	8ajb-J	4	8.2	85	120	8	MOLECULE:	CRESCENTIN
91:00:00	7e9t-A	4	22.6	98	371	8	MOLECULE:	SPIKE PROTEIN S2
92:00:00	7w91-K	4	1.6	51	51	6	MOLECULE:	CENTROSOMAL PROTEIN OF 63 KDA
93:00:00	7w91-B	4	1.8	52	52	6	MOLECULE:	CENTROSOMAL PROTEIN OF 63 KDA
94:00:00	1jth-D	4	2.6	51	51	4	MOLECULE:	SNAP25
95:00:00	5jlf-F	4	7.5	96	135	0	MOLECULE:	ACTIN, ALPHA SKELETAL MUSCLE
96:00:00	6eb8-H	4	4.2	83	101	7	MOLECULE:	PHOSPHOPROTEIN
97:00:00	2fxm-A	4	6.7	95	126	4	MOLECULE:	MYOSIN HEAVY CHAIN, CARDIAC MUSCLE BETA ISOFORM
98:00:00	6id1-K	4	11.1	97	152	7	MOLECULE:	PRE-MRNA-PROCESSING-SPLICING FACTOR 8
99:00:00	4eot-B	4	4.4	67	92	4	MOLECULE:	TRANSCRIPTION FACTOR MAFA
0:00	5j3d-F	4	4	84	358	6	MOLECULE:	14N4 HEAVY CHAIN
1:00	6eup-A	4	4	82	147	4	MOLECULE:	ADHESIN A
2:00	3u1c-B	4	5.1	82	101	10	MOLECULE:	TROPOMYOSIN ALPHA-1 CHAIN
3:00	6ign-A	4	6.7	90	123	9	MOLECULE:	KINESIN-1 HEAVY CHAIN
4:00	3ii6-C	4	4.6	89	201	4	MOLECULE:	DNA REPAIR PROTEIN XRCC4
5:00	6gbp-J	4	4.4	63	66	14	MOLECULE:	POLYMERASE COFACTOR VP35
6:00	4c5q-B	4	2.9	55	55	7	MOLECULE:	PHOSPHOPROTEIN
7:00	6h9m-B	4	5.1	74	94	4	MOLECULE:	COILED-COIL DOMAIN-CONTAINING PROTEIN 90B, MITOCH
8:00	6h9m-C	4	4.9	73	94	12	MOLECULE:	COILED-COIL DOMAIN-CONTAINING PROTEIN 90B, MITOCH
9:00	7woo-I	3.9	10.2	100	187	9	MOLECULE:	NUCLEOPORIN NIC96
10:00	4c47-A	3.9	7.8	87	191	8	MOLECULE:	INNER MEMBRANE LIPOPROTEIN
11:00	3edv-A	3.9	6.9	108	322	6	MOLECULE:	SPECTRIN BETA CHAIN, BRAIN 1
12:00	1ezj-A	3.9	7.2	72	114	10	MOLECULE:	NUCLEOCAPSID PHOSPHOPROTEIN
13:00	8etc-F	3.9	4.7	53	218	2	MOLECULE:	RNA (2151-MER)
14:00	8pda-A	3.9	10.2	108	973	8	MOLECULE:	ERAD-ASSOCIATED E3 UBIQUITIN-PROTEIN LIGASE DOA10
15:00	6xby-a	3.9	21.8	89	494	2	MOLECULE:	V-TYPE PROTON ATPASE CATALYTIC SUBUNIT A
16:00	8hhf-B	3.9	4	72	89	7	MOLECULE:	CELL DIVISION PROTEIN FTSQ
17:00	7xzj-G	3.9	2.5	63	73	6	MOLECULE:	CTAP3
18:00	8auc-B	3.9	14.3	107	493	3	MOLECULE:	CELL WALL-ASSOCIATED HYDROLASES (INVASION-ASSOCIA
19:00	6gmh-Q	3.9	9.8	103	884	5	MOLECULE:	RPB1
20:00	5l4k-S	3.9	16.2	135	491	5	MOLECULE:	26S PROTEASOME NON-ATPASE REGULATORY SUBUNIT 4
21:00	8bd7-G	3.9	20.4	93	205	6	MOLECULE:	IFT88
22:00	4abx-A	3.9	8.8	95	167	7	MOLECULE:	DNA REPAIR PROTEIN RECN
23:00	8cr1-C	3.9	8.2	93	266	4	MOLECULE:	ATPASE ASNA1
24:00:00	4ilo-A	3.9	3.5	104	236	6	MOLECULE:	CT398
25:00:00	6u1s-A	3.9	7.7	127	310	7	MOLECULE:	DE NOVO DESIGNED 16-HELIX TRANSMEMBRANE NANOPORE,
26:00:00	8ajb-G	3.9	6.9	93	155	6	MOLECULE:	CRESCENTIN
27:00:00	2efr-A	3.9	8.7	97	155	6	MOLECULE:	GENERAL CONTROL PROTEIN GCN4 AND TROPOMYOSIN 1 AL
28:00:00	4n5b-C	3.9	6	69	100	9	MOLECULE:	PHOSPHOPROTEIN
29:00:00	2n64-A	3.9	3.6	72	75	7	MOLECULE:	SH3 DOMAIN-CONTAINING KINASE-BINDING PROTEIN 1
30:00:00	1urq-A	3.9	3.3	52	57	8	MOLECULE:	M-TOMOSYN ISOFORM

31:00:00	4cpc-G	3.9	7.3	90	152	10	MOLECULE:	SYNAPTONEMAL COMPLEX PROTEIN 3
32:00:00	4bhv-B	3.9	1.8	56	56	5	MOLECULE:	PHOSPHOPROTEIN
33:00:00	8ahl-G	3.9	16.6	105	155	9	MOLECULE:	CRESCENTIN
34:00:00	8ajb-S	3.9	16.2	103	155	8	MOLECULE:	CRESCENTIN
35:00:00	8qu5-q	3.9	7.7	86	128	6	MOLECULE:	39S RIBOSOMAL PROTEIN L32, MITOCHONDRIAL
36:00:00	6qp4-A	3.9	7.4	89	157	7	MOLECULE:	USPA1
37:00:00	6w1s-W	3.9	19.8	89	118	7	MOLECULE:	MEDIATOR OF RNA POLYMERASE II TRANSCRIPTION SUBUN
38:00:00	3h7x-E	3.9	2.3	53	53	4	MOLECULE:	ADHESIN YADA
39:00:00	2f8x-M	3.9	4.7	55	55	9	MOLECULE:	5'-
40:00:00	5wjb-D	3.9	6.1	88	121	7	MOLECULE:	CAPSID ASSEMBLY SCAFFOLDING PROTEIN,MYOSIN-7
41:00:00	3n4x-D	3.9	4.7	77	159	9	MOLECULE:	MONOPOLIN COMPLEX SUBUNIT CSM1
42:00:00	5vox-J	3.9	3.7	86	104	5	MOLECULE:	V-TYPE PROTON ATPASE CATALYTIC SUBUNIT A,V-TYPE P
43:00:00	7oad-C	3.9	6.9	78	80	5	MOLECULE:	GENERAL CONTROL TRANSCRIPTION FACTOR GCN4,CONSERV
44:00:00	5to6-B	3.9	6.5	97	139	6	MOLECULE:	NUCLEOPROTEIN TPR
45:00:00	1ysa-D	3.9	1.5	55	57	9	MOLECULE:	DNA (5'-
46:00:00	2efs-D	3.9	7.8	90	154	7	MOLECULE:	GENERAL CONTROL PROTEIN GCN4 AND TROPOMYOSIN 1 AL
47:00:00	2efr-D	3.9	7.6	89	155	4	MOLECULE:	GENERAL CONTROL PROTEIN GCN4 AND TROPOMYOSIN 1 AL
48:00:00	7oac-B	3.9	6.7	78	81	5	MOLECULE:	GENERAL CONTROL TRANSCRIPTION FACTOR GCN4,CONSERV
49:00:00	5t4o-I	3.9	5.5	92	155	4	MOLECULE:	ATP SYNTHASE SUBUNIT ALPHA
50:00:00	5lqy-G	3.9	3.7	73	267	10	MOLECULE:	ATP SYNTHASE SUBUNIT F
51:00:00	5n9j-A	3.9	11.2	76	566	8	MOLECULE:	MEDIATOR OF RNA POLYMERASE II TRANSCRIPTION SUBUN
52:00:00	6dd8-A	3.9	4.2	87	131	10	MOLECULE:	SYNAPTONEMAL COMPLEX PROTEIN 3
53:00:00	7apk-e	3.9	5.8	59	512	8	MOLECULE:	THO COMPLEX SUBUNIT 1
54:00:00	7aej-A	3.9	4.9	77	129	10	MOLECULE:	ENVELOPE GLYCOPROTEIN GP160,ENVELOPE GLYCOPROTEIN
55:00:00	2b9c-A	3.9	6.5	85	136	9	MOLECULE:	STRIATED-MUSCLE ALPHA TROPOMYOSIN
56:00:00	7aej-C	3.9	4.3	77	156	12	MOLECULE:	ENVELOPE GLYCOPROTEIN GP160,ENVELOPE GLYCOPROTEIN
57:00:00	3hh0-A	3.8	10.9	73	134	4	MOLECULE:	TRANSCRIPTIONAL REGULATOR, MERR FAMILY
58:00:00	8q85-G	3.8	53.1	87	178	9	MOLECULE:	KINETOCHORE PROTEIN NDC80
59:00:00	7zu0-C	3.8	3.6	53	842	0	MOLECULE:	E3 UBIQUITIN-PROTEIN LIGASE PEP5
60:00:00	8q7n-X	3.8	10.5	69	81	3	MOLECULE:	U5 SNRNA
61:00:00	5szj-B	3.8	3.3	63	145	5	MOLECULE:	RAS-RELATED PROTEIN RAB-10
62:00:00	3viq-A	3.8	11.3	84	122	2	MOLECULE:	SWI5-DEPENDENT RECOMBINATION DNA REPAIR PROTEIN 1
63:00:00	1owa-A	3.8	20.7	96	156	8	MOLECULE:	SPECTRIN ALPHA CHAIN, ERYTHROCYTE
64:00:00	7egm-H	3.8	11.5	88	388	9	MOLECULE:	TRANSCRIPTION REGULATORY PROTEIN SNF2
65:00:00	7tdq-A	3.8	11.7	76	85	7	MOLECULE:	TEGUMENT PROTEIN ORF52
66:00:00	7uh4-A	3.8	2.8	74	110	5	MOLECULE:	LXG-ASSOCIATED ALPHA-HELICAL PROTEIN D2
67:00:00	4n5b-B	3.8	5.5	67	100	6	MOLECULE:	PHOSPHOPROTEIN
68:00:00	4n5b-E	3.8	5.3	68	103	9	MOLECULE:	PHOSPHOPROTEIN
69:00:00	6wm2-K	3.8	4.8	86	114	5	MOLECULE:	V-TYPE PROTON ATPASE SUBUNIT E 1
70:00:00	7yv9-A	3.8	8.9	96	1067	7	MOLECULE:	UNCONVENTIONAL MYOSIN-VA
71:00:00	4wij-A	3.8	5.2	97	298	4	MOLECULE:	SPLICING FACTOR, PROLINE- AND GLUTAMINE-RICH
72:00:00	6xra-A	3.8	26.1	97	348	8	MOLECULE:	SPIKE GLYCOPROTEIN
73:00:00	1sfc-H	3.8	3.6	61	72	3	MOLECULE:	PROTEIN (SYNAPTOBREVIN 2)
74:00:00	1g2c-O	3.8	1.7	50	50	8	MOLECULE:	FUSION PROTEIN (F)
75:00:00	1ifk-A	3.8	2.9	51	51	14	MOLECULE:	INOVIRUS
76:00:00	1g2c-I	3.8	1.7	50	50	10	MOLECULE:	FUSION PROTEIN (F)
77:00:00	2d3e-B	3.8	4	82	130	4	MOLECULE:	GENERAL CONTROL PROTEIN GCN4 AND TROPOMYOSIN 1 AL
78:00:00	3j9v-J	3.8	3	71	105	10	MOLECULE:	V-TYPE PROTON ATPASE SUBUNIT A, VACUOLAR ISOFORM
79:00:00	6g2t-N	3.8	8.2	94	135	0	MOLECULE:	ACTIN, CYTOPLASMIC 2
80:00:00	7jh7-J	3.8	8	94	135	0	MOLECULE:	ACTIN, ALPHA CARDIAC MUSCLE 1
81:00:00	7tj7-T	3.8	8	93	135	0	MOLECULE:	CARDIAC ACTIN
82:00:00	7tj7-V	3.8	6.9	92	135	0	MOLECULE:	CARDIAC ACTIN
83:00:00	2efs-A	3.8	6.1	92	154	8	MOLECULE:	GENERAL CONTROL PROTEIN GCN4 AND TROPOMYOSIN 1 AL
84:00:00	5vr2-A	3.8	2.3	49	50	10	MOLECULE:	MYOCILIN
85:00:00	4w7y-B	3.8	3.2	57	64	4	MOLECULE:	B-CELL RECEPTOR-ASSOCIATED PROTEIN 29
86:00:00	6snz-A	3.8	6.8	90	160	9	MOLECULE:	PRELAMIN-A/C

87:00:00	2oto-C	3.8	8	96	138	7	MOLECULE:	M PROTEIN
88:00:00	2oto-A	3.8	6.7	91	137	8	MOLECULE:	M PROTEIN
89:00:00	3gjo-C	3.8	3.4	59	65	7	MOLECULE:	MICROTUBULE-ASSOCIATED PROTEIN RP/EB FAMILY MEMBE
90:00:00	6eun-B	3.8	2.9	75	145	4	MOLECULE:	ADHESIN
91:00:00	6eun-C	3.8	4	76	145	1	MOLECULE:	ADHESIN
92:00:00	7d7n-A	3.7	6.8	70	704	9	MOLECULE:	ATP-BINDING CASSETTE SUB-FAMILY B MEMBER 6, MITOC
93:00:00	3o1j-A	3.7	6.1	106	273	7	MOLECULE:	SENSOR PROTEIN TORS
94:00:00	8gel-A	3.7	23.6	93	498	2	MOLECULE:	SC4
95:00:00	8tvh-A	3.7	8.5	82	86	11	MOLECULE:	4G5 LIGHT CHAIN
96:00:00	6tmi-B	3.7	8.2	104	246	10	MOLECULE:	ATP SYNTHASE SUBUNIT ALPHA
97:00:00	6gvw-F	3.7	9.6	97	320	4	MOLECULE:	BRCA1-A COMPLEX SUBUNIT ABRAXAS 1
98:00:00	4dyl-A	3.7	2.7	82	376	12	MOLECULE:	TYROSINE-PROTEIN KINASE FES/FPS
99:00:00	3p8c-F	3.7	3.4	77	156	5	MOLECULE:	CYTOPLASMIC FMR1-INTERACTING PROTEIN 1
0:00	5vo5-A	3.7	5.2	90	179	6	MOLECULE:	COILED-COIL AND C2 DOMAIN-CONTAINING PROTEIN 1-LI
1:00	2fo1-D	3.7	6.1	60	63	10	MOLECULE:	5'-
2:00	5lc5-Z	3.7	13.4	104	138	3	MOLECULE:	NADH-UBIQUINONE OXIDOREDUCTASE CHAIN 3
3:00	4r8g-E	3.7	5	73	324	10	MOLECULE:	UNCONVENTIONAL MYOSIN-IC
4:00	5to5-A	3.7	39	112	141	6	MOLECULE:	NUCLEOPROTEIN TPR
5:00	4n5b-G	3.7	5.6	67	101	3	MOLECULE:	PHOSPHOPROTEIN
6:00	2v71-B	3.7	38.2	108	160	8	MOLECULE:	NUCLEAR DISTRIBUTION PROTEIN NUDE-LIKE 1
7:00	9b8q-I	3.7	6.9	94	148	2	MOLECULE:	V-TYPE PROTON ATPASE SUBUNIT C 1
8:00	8tek-M	3.7	3.9	69	164	3	MOLECULE:	DYNEIN REGULATORY COMPLEX PROTEIN 1/2 N-TERMINAL
9:00	1h88-B	3.7	3.8	60	71	5	MOLECULE:	CCAAT/ENHANCER BINDING PROTEIN BETA
10:00	7use-C	3.7	3.7	72	112	8	MOLECULE:	CYTOPLASMIC FMR1-INTERACTING PROTEIN 1
11:00	8t1i-I	3.7	9.3	80	1086	6	MOLECULE:	MEDIATOR OF RNA POLYMERASE II TRANSCRIPTION SUBUN
12:00	2z5h-A	3.7	2.8	49	49	2	MOLECULE:	GENERAL CONTROL PROTEIN GCN4 AND TROPOMYOSIN ALPH
13:00	8ovw-Y	3.7	7.9	94	223	5	MOLECULE:	CENTROMERE-BINDING PROTEIN 1
14:00	2v71-A	3.7	8.7	97	160	6	MOLECULE:	NUCLEAR DISTRIBUTION PROTEIN NUDE-LIKE 1
15:00	4c7n-B	3.7	2.3	51	51	6	MOLECULE:	MICROPHTHALMIA ASSOCIATED TRANSCRIPTION FACTOR
16:00	8q3v-A	3.7	4.6	56	61	16	MOLECULE:	TETRAHYDROMETHANOPTERIN S-METHYLTRANSFERASE SUBUN
17:00	5mqf-J	3.7	6.9	76	135	9	MOLECULE:	PRE-MRNA-PROCESSING-SPLICING FACTOR 8
18:00	6cxj-T	3.7	7.8	93	127	0	MOLECULE:	ACTIN, CYTOPLASMIC 2
19:00	6cxi-T	3.7	7.8	93	127	0	MOLECULE:	ACTIN, CYTOPLASMIC 2
20:00	6xbw-O	3.7	4.6	82	108	2	MOLECULE:	V-TYPE PROTON ATPASE CATALYTIC SUBUNIT A
21:00	4dl0-G	3.7	3.4	84	105	5	MOLECULE:	V-TYPE PROTON ATPASE SUBUNIT C
22:00	8q3v-Q	3.7	5	57	61	16	MOLECULE:	TETRAHYDROMETHANOPTERIN S-METHYLTRANSFERASE SUBUN
23:00	5jx1-A	3.7	2.3	55	64	4	MOLECULE:	CHIMERA PROTEIN OF KINESIN-LIKE PROTEIN KIF3A AND
24:00:00	2n1t-D	3.7	4	62	74	2	MOLECULE:	VESICLE-ASSOCIATED MEMBRANE PROTEIN 2
25:00:00	6mi3-A	3.7	6.7	80	122	8	MOLECULE:	NF-KB ESSENTIAL MODULATOR,NF-KAPPA-B ESSENTIAL MO
26:00:00	7oah-C	3.7	8	74	82	4	MOLECULE:	GENERAL CONTROL TRANSCRIPTION FACTOR GCN4,CONSERV
27:00:00	3wpr-C	3.7	2.8	54	218	6	MOLECULE:	TRIMERIC AUTOTRANSPORTER ADHESIN
28:00:00	4mvd-C	3.7	14.7	93	253	6	MOLECULE:	CHOLINE-PHOSPHATE CYTIDYLYLTRANSFERASE A
29:00:00	8es7-B	3.6	2.2	41	287	7	MOLECULE:	T-CELL SURFACE GLYCOPROTEIN CD3 ZETA CHAIN
30:00:00	3a6m-A	3.6	24.2	101	168	8	MOLECULE:	PROTEIN GRPE
31:00:00	8th8-B	3.6	50	86	276	3	MOLECULE:	DYNEIN REGULATORY COMPLEX PROTEIN 1/2 N-TERMINAL
32:00:00	7aw7-D	3.6	4.3	67	93	3	MOLECULE:	HAPB
33:00:00	6zz6-B	3.6	36.8	83	423	7	MOLECULE:	STRUCTURAL MAINTENANCE OF CHROMOSOMES PROTEIN 1,S
34:00:00	5t96-B	3.6	2.1	41	330	0	MOLECULE:	HE PROTEIN
35:00:00	5gox-A	3.6	6.8	95	181	11	MOLECULE:	DNA REPAIR PROTEIN RAD50
36:00:00	3ub0-D	3.6	12.5	83	194	6	MOLECULE:	NON-STRUCTURAL PROTEIN 6, NSP6,
37:00:00	6sp2-A	3.6	7.7	71	366	4	MOLECULE:	MEMBRANE PROTEIN TMS1D
38:00:00	8b9z-Z	3.6	14.4	97	146	4	MOLECULE:	NADH-UBIQUINONE OXIDOREDUCTASE CHAIN 3
39:00:00	5gw1-C	3.6	10.5	86	176	10	MOLECULE:	SORTING NEXIN-16
40:00:00	5nmo-A	3.6	6.4	84	162	6	MOLECULE:	CHROMOSOME PARTITION PROTEIN SMC,CHROMOSOME PARTI
41:00:00	6wcj-B	3.6	1.3	51	109	4	MOLECULE:	CLATHRIN HEAVY CHAIN 1
42:00:00	4m8m-B	3.6	5.5	84	574	5	MOLECULE:	GCN4 COILED-COIL FUSED ZEBRAFISH PLEXINC1

43:00:00	7zmg-n	3.6	12.1	75	136	9	MOLECULE:	NADH-UBIQUINONE OXIDOREDUCTASE CHAIN 1
44:00:00	8x5f-A	3.6	7.9	80	616	5	MOLECULE:	SOLUTE CARRIER FAMILY 53 MEMBER 1
45:00:00	5o31-Z	3.6	12.9	104	137	3	MOLECULE:	NADH-UBIQUINONE OXIDOREDUCTASE CHAIN 3
46:00:00	6ch2-E	3.6	4.2	62	147	8	MOLECULE:	FLAGELLAR BIOSYNTHESIS PROTEIN FLHA
47:00:00	7tit-N	3.6	55.7	119	135	0	MOLECULE:	CARDIAC ACTIN
48:00:00	7qoo-K	3.6	17.2	90	253	9	MOLECULE:	CENTROMERE PROTEIN C
49:00:00	8hhh-B	3.6	4.6	74	89	9	MOLECULE:	CELL DIVISION PROTEIN FTSQ
50:00:00	7x6g-D	3.6	4.8	80	152	14	MOLECULE:	QUORUM-SENSING REGULATOR PROTEIN G
51:00:00	7jg8-d	3.6	9.2	107	428	10	MOLECULE:	ATP SYNTHASE SUBUNIT ALPHA
52:00:00	2e7s-A	3.6	3.3	76	114	9	MOLECULE:	RAB GUANINE NUCLEOTIDE EXCHANGE FACTOR SEC2
53:00:00	3lj5-A	3.6	4	91	692	5	MOLECULE:	PORTAL PROTEIN
54:00:00	5jlh-K	3.6	7.2	91	135	0	MOLECULE:	ACTIN, CYTOPLASMIC 2
55:00:00	7pkq-w	3.6	7.7	88	155	7	MOLECULE:	MS35
56:00:00	3o0z-A	3.6	7.8	97	152	10	MOLECULE:	RHO-ASSOCIATED PROTEIN KINASE 1
57:00:00	4jgs-C	3.6	2.9	62	88	10	MOLECULE:	MLV-RELATED PROVIRAL ENV POLYPROTEIN
58:00:00	7oaf-A	3.6	6.8	72	81	6	MOLECULE:	GENERAL CONTROL TRANSCRIPTION FACTOR GCN4,CONSERV
59:00:00	3wpr-B	3.6	5.4	67	207	3	MOLECULE:	TRIMERIC AUTOTRANSPORTER ADHESIN
60:00:00	3gjo-A	3.6	3.3	60	66	7	MOLECULE:	MICROTUBULE-ASSOCIATED PROTEIN RP/EB FAMILY MEMBE
61:00:00	4egx-A	3.6	2	54	180	7	MOLECULE:	KINESIN-LIKE PROTEIN KIF1A
62:00:00	4mh6-A	3.5	20.8	92	159	4	MOLECULE:	PUTATIVE TYPE III SECRETION PROTEIN YSCO
63:00:00	8qfs-A	3.5	30.4	99	1183	4	MOLECULE:	ELONGATION FACTOR TU
64:00:00	5ncl-A	3.5	9.9	73	418	7	MOLECULE:	SERINE/THREONINE-PROTEIN KINASE CBK1
65:00:00	6em5-t	3.5	16	84	290	8	MOLECULE:	5.8S RIBOSOMAL RNA
66:00:00	6kn7-U	3.5	8.3	114	170	2	MOLECULE:	ACTIN, ALPHA SKELETAL MUSCLE
67:00:00	7yoj-A	3.5	3.1	66	867	3	MOLECULE:	CASPI
68:00:00	5n9j-E	3.5	16.1	98	201	6	MOLECULE:	MEDIATOR OF RNA POLYMERASE II TRANSCRIPTION SUBUN
69:00:00	6ted-R	3.5	11.2	81	244	9	MOLECULE:	DNA-DIRECTED RNA POLYMERASE SUBUNIT
70:00:00	5che-A	3.5	14.5	112	427	6	MOLECULE:	GLUTAMYL-TRNA REDUCTASE 1, CHLOROPLASTIC
71:00:00	5cd4-I	3.5	3.6	75	494	7	MOLECULE:	CRISPR SYSTEM CASCADE SUBUNIT CASE
72:00:00	6nyi-A	3.5	3.2	65	97	5	MOLECULE:	DESIGN CONSTRUCT XXA
73:00:00	6wuc-H	3.5	13.3	85	179	6	MOLECULE:	INNER KINETOCHORE SUBUNIT MCM16
74:00:00	5n9j-Z	3.5	3.6	58	119	7	MOLECULE:	MEDIATOR OF RNA POLYMERASE II TRANSCRIPTION SUBUN
75:00:00	7wkk-A	3.5	10.4	95	1684	8	MOLECULE:	MGC83295 PROTEIN
76:00:00	4abn-B	3.5	2.4	56	426	11	MOLECULE:	TETRATRICOPEPTIDE REPEAT PROTEIN 5
77:00:00	6ynw-H	3.5	1.8	60	75	10	MOLECULE:	SUBUNIT C
78:00:00	5yfp-H	3.5	21.4	75	518	11	MOLECULE:	EXOCYST COMPLEX COMPONENT SEC3
79:00:00	5vk5-B	3.5	6.8	79	282	10	MOLECULE:	POTASSIUM CHANNEL SUBFAMILY K MEMBER 2
80:00:00	7uus-Q	3.5	9.2	64	170	6	MOLECULE:	HYDROGENASE-2, LARGE SUBUNIT
81:00:00	6iac-E	3.5	11.9	98	146	6	MOLECULE:	PORTAL PROTEIN
82:00:00	7jw1-e	3.5	10.6	123	235	13	MOLECULE:	CAPSID PROTEINS
83:00:00	8w8q-C	3.5	8.2	74	287	5	MOLECULE:	SOLUBLE CYTOCHROME B562,PROBABLE G-PROTEIN COUPLE
84:00:00	8exh-a	3.5	3.9	60	69	5	MOLECULE:	PROTEIN VIRB2
85:00:00	3o0z-D	3.5	8	95	160	6	MOLECULE:	RHO-ASSOCIATED PROTEIN KINASE 1
86:00:00	8q4g-H	3.5	8.9	97	179	4	MOLECULE:	ACTIN, ALPHA CARDIAC MUSCLE 1
87:00:00	2oto-B	3.5	57	121	138	7	MOLECULE:	M PROTEIN
88:00:00	4tt1-B	3.5	7.6	95	129	3	MOLECULE:	DENEDDYLASE
89:00:00	8q4g-G	3.5	9.1	96	179	5	MOLECULE:	ACTIN, ALPHA CARDIAC MUSCLE 1
90:00:00	3eff-K	3.5	4.7	76	139	7	MOLECULE:	FAB
91:00:00	1czq-A	3.5	1.8	45	45	2	MOLECULE:	FUSION PROTEIN BETWEEN THE HYDROPHOBIC POCKET OF
92:00:00	3vmx-A	3.5	1.1	45	48	4	MOLECULE:	VOLTAGE-GATED HYDROGEN CHANNEL 1
93:00:00	5yr0-B	3.5	2	44	44	7	MOLECULE:	BECLIN-1
94:00:00	8gwe-B	3.5	4.3	79	190	6	MOLECULE:	RNA-DIRECTED RNA POLYMERASE
95:00:00	8t1i-W	3.5	20.8	85	118	12	MOLECULE:	MEDIATOR OF RNA POLYMERASE II TRANSCRIPTION SUBUN
96:00:00	5bvz-A	3.5	5	83	180	6	MOLECULE:	DNA STABILIZATION PROTEIN
97:00:00	5jlh-I	3.5	7.2	91	135	0	MOLECULE:	ACTIN, CYTOPLASMIC 2
98:00:00	5nog-G	3.5	7.2	91	135	0	MOLECULE:	CARDIAC MUSCLE ALPHA ACTIN 1

99:00:00	4a7l-B	3.5	7.2	91	136	10	MOLECULE:	ACTIN, ALPHA SKELETON MUSCLE
0:00	3j4k-F	3.5	7.2	91	136	0	MOLECULE:	ACTIN, ALPHA SKELETAL MUSCLE
1:00	4a7f-B	3.5	7.2	91	136	10	MOLECULE:	ACTIN, ALPHA SKELETAL MUSCLE
2:00	8q3v-a	3.5	4.8	58	61	2	MOLECULE:	TETRAHYDROMETHANOPTERIN S-METHYLTRANSFERASE SUBUN
3:00	4tko-B	3.5	9.4	105	326	8	MOLECULE:	EMRA
4:00	5nen-A	3.5	6.5	100	266	8	MOLECULE:	LIPASE C
5:00	7apk-M	3.5	59.2	93	549	8	MOLECULE:	THO COMPLEX SUBUNIT 1
6:00	7oaf-B	3.5	6.5	72	81	6	MOLECULE:	GENERAL CONTROL TRANSCRIPTION FACTOR GCN4,CONSERV
7:00	7oaf-C	3.5	6.3	74	81	3	MOLECULE:	GENERAL CONTROL TRANSCRIPTION FACTOR GCN4,CONSERV
8:00	6mi3-B	3.5	6.4	78	124	4	MOLECULE:	NF-KB ESSENTIAL MODULATOR,NF-KAPPA-B ESSENTIAL MO
9:00	7oac-C	3.5	7.9	72	81	4	MOLECULE:	GENERAL CONTROL TRANSCRIPTION FACTOR GCN4,CONSERV
10:00	2pms-C	3.4	21.1	88	109	10	MOLECULE:	LACTOTRANSFERRIN
11:00	8cra-A	3.4	15.8	76	96	7	MOLECULE:	FLORAL HOMEOTIC PROTEIN AGAMOUS
12:00	6w1s-Q	3.4	38	76	131	5	MOLECULE:	MEDIATOR OF RNA POLYMERASE II TRANSCRIPTION SUBUN
13:00	8vxq-A	3.4	17.7	79	155	5	MOLECULE:	GP72
14:00	6gap-A	3.4	61.3	121	218	5	MOLECULE:	OUTER CAPSID PROTEIN SIGMA-1
15:00	7qoo-Q	3.4	10.6	75	209	8	MOLECULE:	CENTROMERE PROTEIN C
16:00	7wkk-I	3.4	29.3	101	171	12	MOLECULE:	MGC83295 PROTEIN
17:00	7emf-G	3.4	7.5	88	161	7	MOLECULE:	MEDIATOR OF RNA POLYMERASE II TRANSCRIPTION SUBUN
18:00	4cq4-A	3.4	3.1	73	106	11	MOLECULE:	ENGINEERED VERSION OF TRANSMEMBRANE RECEPTOR AF15
19:00	4cgk-A	3.4	9.4	101	351	6	MOLECULE:	SECRETED 45 KDA PROTEIN
20:00	7c4j-F	3.4	4.5	75	383	9	MOLECULE:	TRANSCRIPTION REGULATORY PROTEIN SNF12
21:00	4whj-A	3.4	6.7	88	565	10	MOLECULE:	INTERFERON-INDUCED GTP-BINDING PROTEIN MX2
22:00	3nr7-A	3.4	4.9	64	81	9	MOLECULE:	DNA-BINDING PROTEIN H-NS
23:00	8hqo-x	3.4	8	75	80	7	MOLECULE:	PORTAL PROTEIN
24:00:00	8d8k-V	3.4	6	83	233	10	MOLECULE:	PROBABLE S-ADENOSYL-L-METHIONINE-DEPENDENT RNA
25:00:00	3k66-A	3.4	3.7	91	217	5	MOLECULE:	BETA-AMYLOID-LIKE PROTEIN
26:00:00	8cqn-B	3.4	7.3	97	187	13	MOLECULE:	LIPOPROTEIN, PUTATIVE
27:00:00	7vv6-R	3.4	5.8	78	271	5	MOLECULE:	MAS-RELATED G-PROTEIN COUPLED RECEPTOR MEMBER X2
28:00:00	2xeq-C	3.4	2.7	57	241	7	MOLECULE:	PAT1 HOMOLOG 1,
29:00:00	7v2w-I	3.4	41.9	124	239	7	MOLECULE:	THO COMPLEX SUBUNIT HPR1
30:00:00	9b8o-U	3.4	7.1	60	214	7	MOLECULE:	ATPASE H+-TRANSPORTING V1 SUBUNIT D
31:00:00	7sc0-A	3.4	13.1	75	129	3	MOLECULE:	CAVEOLIN-1
32:00:00	6qm5-A	3.4	17.7	100	673	4	MOLECULE:	PREDICTED PROTEIN
33:00:00	7zmb-3	3.4	20.9	79	130	8	MOLECULE:	NADH-UBIQUINONE OXIDOREDUCTASE CHAIN 1
34:00:00	3oja-A	3.4	11.7	98	482	4	MOLECULE:	LEUCINE-RICH IMMUNE MOLECULE 1
35:00:00	7w01-A	3.4	15.4	105	1598	8	MOLECULE:	PHOSPHOLIPID-TRANSPORTING ATPASE ABCA3
36:00:00	7m2w-F	3.4	2.7	55	674	9	MOLECULE:	TUBULIN GAMMA CHAIN
37:00:00	6c48-D	3.4	3.1	61	87	13	MOLECULE:	PROTEIN LIN-9 HOMOLOG
38:00:00	7cun-D	3.4	15.3	93	827	5	MOLECULE:	INTEGRATOR COMPLEX SUBUNIT 1
39:00:00	4n5b-F	3.4	6	70	101	4	MOLECULE:	PHOSPHOPROTEIN
40:00:00	6w4f-D	3.4	10.9	77	198	10	MOLECULE:	APOLIPOPROTEIN A-I
41:00:00	7fdb-G	3.4	7.2	92	225	4	MOLECULE:	YEAST VACUOLAR ATPASE A SUBUNIT
42:00:00	5uky-A	3.4	2.8	62	74	8	MOLECULE:	ATP-BINDING PROTEIN
43:00:00	7tit-P	3.4	55.7	117	135	0	MOLECULE:	CARDIAC ACTIN
44:00:00	6cfz-I	3.4	11.4	87	106	6	MOLECULE:	ASK1
45:00:00	3vyi-F	3.4	1.4	45	49	2	MOLECULE:	VOLTAGE-GATED HYDROGEN CHANNEL 1
46:00:00	1w2e-A	3.4	2.9	54	91	6	MOLECULE:	ZAPA
47:00:00	5nvu-A	3.4	6.6	99	3169	0	MOLECULE:	DYNEIN MOTOR DOMAIN
48:00:00	8aud-A	3.4	6	91	350	9	MOLECULE:	CELL WALL-ASSOCIATED HYDROLASES (INVASION-ASSOCIA
49:00:00	8fed-A	3.4	14.5	83	392	4	MOLECULE:	VIRULENCE FACTOR MCE FAMILY PROTEIN
50:00:00	1ebo-F	3.4	19.4	89	113	6	MOLECULE:	EBOLA VIRUS ENVELOPE PROTEIN CHIMERA CONSISTING
51:00:00	3wpo-A	3.4	26.9	96	193	6	MOLECULE:	TRIMERIC AUTOTRANSPORTER ADHESIN
52:00:00	4xa1-A	3.4	17.2	94	153	9	MOLECULE:	GP7-MYH7(1173-1238)-EB1 CHIMERA PROTEIN
53:00:00	4egx-C	3.4	2.6	55	174	4	MOLECULE:	KINESIN-LIKE PROTEIN KIF1A
54:00:00	6a70-B	3.3	12.9	73	704	3	MOLECULE:	POLYCYSTIN-2

55:00:00	5ijn-G	3.3	26.9	99	171	8	MOLECULE:	NUCLEAR PORE COMPLEX PROTEIN NUP155
56:00:00	8k9t-A	3.3	31.7	106	961	5	MOLECULE:	GPI INOSITOL-DEACYLASE,MCHERRY PROTEIN
57:00:00	7qo4-J	3.3	4.2	60	405	7	MOLECULE:	26S PROTEASOME REGULATORY SUBUNIT RPN1
58:00:00	8bjw-F	3.3	5	69	97	12	MOLECULE:	PROTEIN C
59:00:00	2m67-A	3.3	3.2	61	81	8	MOLECULE:	MERF
60:00:00	8tdl-A	3.3	10.1	76	364	11	MOLECULE:	MECHANOSENSITIVE ION CHANNEL PROTEIN 10
61:00:00	1t7s-A	3.3	3.2	63	129	13	MOLECULE:	BAG-1 COCHAPERONE
62:00:00	7xzi-A	3.3	46.6	118	1598	9	MOLECULE:	CTAP3
63:00:00	6c14-B	3.3	2.7	55	164	7	MOLECULE:	PROTOCADHERIN-15
64:00:00	7t6d-A	3.3	3.4	58	389	7	MOLECULE:	LIPOPOLYSACCHARIDE ASSEMBLY PROTEIN B
65:00:00	7v2c-d	3.3	9.7	66	175	5	MOLECULE:	NADH DEHYDROGENASE [UBIQUINONE] FLAVOPROTEIN 1,
66:00:00	4gcz-B	3.3	7.8	90	378	9	MOLECULE:	BLUE-LIGHT PHOTORECEPTOR, SENSOR PROTEIN FIXL
67:00:00	6xf1-A	3.3	4.8	96	230	6	MOLECULE:	NESPRIN-2
68:00:00	7zn7-D	3.3	3.1	58	128	9	MOLECULE:	DNA DAMAGE-BINDING PROTEIN 1
69:00:00	7kzn-B	3.3	15.6	106	683	6	MOLECULE:	HEAVY CHAIN ALPHA
70:00:00	4mer-D	3.3	5.2	68	97	10	MOLECULE:	STREPTOCOCCAL HISTIDINE-RICH GLYCOPROTEIN INTERAC
71:00:00	8qfc-C	3.3	4.9	101	380	13	MOLECULE:	60S RIBOSOMAL PROTEIN L10A
72:00:00	7pgg-A	3.3	7.6	81	147	9	MOLECULE:	ION TRANSPORT PROTEIN
73:00:00	8f7c-A	3.3	9.7	72	287	6	MOLECULE:	PANNEXIN-2, SOLUBLE CYTOCHROME B562 FUSION
74:00:00	7v2w-J	3.3	6.4	80	236	5	MOLECULE:	THO COMPLEX SUBUNIT HPR1
75:00:00	6hgc-A	3.3	10.5	78	304	1	MOLECULE:	UBIQUITIN CARBOXYL-TERMINAL HYDROLASE CALYPSO,UBI
76:00:00	6wg3-B	3.3	12.4	108	693	5	MOLECULE:	STRUCTURAL MAINTENANCE OF CHROMOSOMES PROTEIN 1A
77:00:00	6rlb-A	3.3	18	129	969	6	MOLECULE:	O6-ALKYLGUANINE-DNA ALKYLTRANSFERASE MUTANT,DYNC2
78:00:00	6ogd-E	3.3	13.7	121	1078	7	MOLECULE:	TOXIN SUBUNIT YENA1
79:00:00	8aia-G	3.3	6.3	95	183	7	MOLECULE:	CRESCENTIN
80:00:00	8c5v-I	3.3	53.2	107	516	7	MOLECULE:	CHEMOTAXIS PROTEIN CHEA
81:00:00	8ovw-Q	3.3	26.5	112	258	8	MOLECULE:	CENTROMERE-BINDING PROTEIN 1
82:00:00	7z4a-J	3.3	40.7	89	588	10	MOLECULE:	ADAPTOR PROTEIN
83:00:00	4mer-A	3.3	7.4	76	96	8	MOLECULE:	STREPTOCOCCAL HISTIDINE-RICH GLYCOPROTEIN INTERAC
84:00:00	8ovw-Z	3.3	24.2	101	151	10	MOLECULE:	CENTROMERE-BINDING PROTEIN 1
85:00:00	1gmj-D	3.3	1.8	52	56	10	MOLECULE:	ATPASE INHIBITOR
86:00:00	3oja-B	3.3	5.8	123	534	11	MOLECULE:	LEUCINE-RICH IMMUNE MOLECULE 1
87:00:00	5cws-E	3.3	26.6	111	241	7	MOLECULE:	SAB-158 FAB LIGHT CHAIN
88:00:00	2e7s-E	3.3	6.9	82	124	5	MOLECULE:	RAB GUANINE NUCLEOTIDE EXCHANGE FACTOR SEC2
89:00:00	5z7g-C	3.3	1.5	43	43	7	MOLECULE:	TAX1-BINDING PROTEIN 1
90:00:00	4nqi-D	3.3	4	87	232	5	MOLECULE:	SH3 DOMAIN-CONTAINING PROTEIN
91:00:00	7oaa-A	3.3	9.2	76	82	7	MOLECULE:	GENERAL CONTROL TRANSCRIPTION FACTOR GCN4,CONSERV
92:00:00	8th8-A	3.2	23.9	103	290	6	MOLECULE:	DYNEIN REGULATORY COMPLEX PROTEIN 1/2 N-TERMINAL
93:00:00	6jy0-A	3.2	25	109	410	6	MOLECULE:	FLAGELLIN
94:00:00	2d1l-A	3.2	4.7	93	249	6	MOLECULE:	METASTASIS SUPPRESSOR PROTEIN 1
95:00:00	6tpi-A	3.2	23.4	94	380	14	MOLECULE:	MUREIN HYDROLASE ACTIVATOR ENVC
96:00:00	6oqw-Y	3.2	52.1	90	155	8	MOLECULE:	ATP SYNTHASE SUBUNIT DELTA
97:00:00	6gh5-M	3.2	5.5	50	338	8	MOLECULE:	DNA-DIRECTED RNA POLYMERASE SUBUNIT ALPHA
98:00:00	8eja-A	3.2	3.1	65	113	8	MOLECULE:	ABAK
99:00:00	6xxv-C	3.2	3.2	62	111	8	MOLECULE:	ANTIBODY C57, HEAVY CHAIN
0:00	7edr-D	3.2	9.5	71	122	8	MOLECULE:	MOESIN/EZRIN/RADIXIN HOMOLOG 2
1:00	4wfc-B	3.2	3.9	57	118	4	MOLECULE:	EXOSOME COMPLEX EXONUCLEASE RRP6
2:00	6xdc-A	3.2	3.5	57	193	12	MOLECULE:	PROTEIN 3A
3:00	5td8-C	3.2	10.4	77	111	9	MOLECULE:	KINETOCHORE PROTEIN NDC80
4:00	5c21-A	3.2	3	83	267	7	MOLECULE:	CHROMOSOMAL HEMOLYSIN D
5:00	4gx2-B	3.2	2.1	56	548	9	MOLECULE:	TRKA DOMAIN PROTEIN
6:00	6gmh-M	3.2	6.4	68	991	7	MOLECULE:	RPB1
7:00	8oyy-A	3.2	11.9	67	233	1	MOLECULE:	DE NOVO DESIGNED SOLUBLE GPCR-LIKE PROTEIN
8:00	1s35-A	3.2	4.8	89	211	10	MOLECULE:	SPECTRIN BETA CHAIN, ERYTHROCYTE
9:00	6k7x-C	3.2	15.1	99	276	6	MOLECULE:	CALCIUM UNIPORTER PROTEIN, MITOCHONDRIAL
10:00	8hmy-B	3.2	31.1	88	264	5	MOLECULE:	TRNA-SPLICING ENDONUCLEASE SUBUNIT SEN2

11:00	4u6u-D	3.2	8.7	86	283	12	MOLECULE:	COG7
12:00	7cgp-C	3.2	12.3	95	223	11	MOLECULE:	MITOCHONDRIAL IMPORT INNER MEMBRANE TRANSLOCASE S
13:00	7khw-a	3.2	3.4	55	174	4	MOLECULE:	TRANSLOCON ESPA
14:00	5yfp-B	3.2	10.5	101	927	8	MOLECULE:	EXOCYST COMPLEX COMPONENT SEC3
15:00	7pv1-D	3.2	3.9	71	141	4	MOLECULE:	MICOS COMPLEX SUBUNIT MIC60 FUSED TO MIC19
16:00	4rp5-A	3.2	12.2	71	94	6	MOLECULE:	DISKS LARGE 1 TUMOR SUPPRESSOR PROTEIN
17:00	6yvu-A	3.2	15.3	118	1127	8	MOLECULE:	STRUCTURAL MAINTENANCE OF CHROMOSOMES PROTEIN 2,S
18:00	3cqx-D	3.2	2.8	62	84	6	MOLECULE:	HEAT SHOCK COGNATE 71 KDA PROTEIN
19:00	8ij1-D	3.2	6.7	68	627	3	MOLECULE:	CULLIN-2
20:00	6k9k-A	3.2	31	72	2501	8	MOLECULE:	SERINE-PROTEIN KINASE ATM
21:00	7pl9-A	3.2	16.5	117	994	9	MOLECULE:	RHODOPSIN
22:00	7z8b-C	3.2	8.7	106	1224	10	MOLECULE:	CULLIN-7
23:00	8smr-G	3.2	3.1	54	312	11	MOLECULE:	UBIQUINOL-CYTOCHROME C REDUCTASE IRON-SULFUR SUBU
24:00:00	8z9a-A	3.2	5.4	66	406	6	MOLECULE:	ODORANT RECEPTOR, APISORCO
25:00:00	6lqo-L	3.2	4.1	66	94	8	MOLECULE:	CYTOPLASMIC ENVELOPMENT PROTEIN 1
26:00:00	7vcf-C	3.2	4.7	80	217	8	MOLECULE:	TIC214
27:00:00	8rz0-E	3.2	3.3	66	113	5	MOLECULE:	RH5-34EM
28:00:00	6rd4-5	3.2	5.4	70	123	10	MOLECULE:	ASA-10: POLYTOMELLA F-ATP SYNTHASE ASSOCIATED SUB
29:00:00	8a3t-Q	3.2	11.6	86	623	10	MOLECULE:	ANAPHASE-PROMOTING COMPLEX SUBUNIT CDC27
30:00:00	6qaj-B	3.2	7.1	82	441	6	MOLECULE:	ENDOLYSIN,TRANSCRIPTION INTERMEDIARY FACTOR 1-BET
31:00:00	6z6o-D	3.2	9.1	95	542	7	MOLECULE:	HISTONE DEACETYLASE HDA1
32:00:00	1yig-B	3.2	4.4	51	61	6	MOLECULE:	MICROTUBULE-ASSOCIATED PROTEIN RP/EB FAMILY
33:00:00	1ifm-A	3.2	1.9	46	46	7	MOLECULE:	INOVIRUS
34:00:00	7oac-A	3.2	8.6	73	81	8	MOLECULE:	GENERAL CONTROL TRANSCRIPTION FACTOR GCN4,CONSERV
35:00:00	2efr-C	3.2	62.1	93	154	4	MOLECULE:	GENERAL CONTROL PROTEIN GCN4 AND TROPOMYOSIN 1 AL
36:00:00	6pfp-A	3.2	11	74	156	4	MOLECULE:	MYOSIN-7 FUSED TO GP7 AND EB1
37:00:00	6n6r-D	3.2	8.7	54	67	6	MOLECULE:	UBIQUITIN
38:00:00	5djo-A	3.2	2.8	59	158	0	MOLECULE:	KINESIN-LIKE PROTEIN
39:00:00	5j8v-A	3.1	25.4	102	3398	6	MOLECULE:	RYANODINE RECEPTOR 1
40:00:00	8t1l-W	3.1	16.5	76	119	4	MOLECULE:	MEDIATOR OF RNA POLYMERASE II TRANSCRIPTION SUBUN
41:00:00	7z4f-J	3.1	29.1	78	603	5	MOLECULE:	PUTATIVE STRUCTURAL PROTEIN
42:00:00	7vdv-A	3.1	21.2	77	809	6	MOLECULE:	HISTONE H4
43:00:00	7bw0-R	3.1	17.9	52	280	4	MOLECULE:	SOLUBLE CYTOCHROME B562,G-PROTEIN COUPLED BILE AC
44:00:00	6c0f-8	3.1	8.8	65	98	6	MOLECULE:	SACCHAROMYCES CEREVISIAE S288C 35S PRE-RIBOSOMAL
45:00:00	3zcj-A	3.1	22.9	95	205	8	MOLECULE:	CAGL
46:00:00	5ew5-A	3.1	22.2	116	490	3	MOLECULE:	COLICIN-E9
47:00:00	6r1j-J	3.1	9.1	102	264	8	MOLECULE:	UNCHARACTERIZED PROTEIN
48:00:00	7vc4-C	3.1	8.2	66	107	6	MOLECULE:	MITOCHONDRIAL IMPORT RECEPTOR SUBUNIT TOM6 HOMOLO
49:00:00	6m3q-F	3.1	3.5	88	301	8	MOLECULE:	ANKYRIN-2
50:00:00	6qeq-A	3.1	10.7	78	111	6	MOLECULE:	PCFF
51:00:00	6bog-A	3.1	30.1	101	967	7	MOLECULE:	RNA POLYMERASE-ASSOCIATED PROTEIN RAPA
52:00:00	7oqz-A	3.1	2.4	51	237	8	MOLECULE:	TRANSMEMBRANE PROTEIN 45A
53:00:00	8hvj-A	3.1	10.2	114	281	10	MOLECULE:	UPF0701 PROTEIN YICC
54:00:00	2kn8-A	3.1	10.8	66	68	8	MOLECULE:	DNA CLEAVAGE AND PACKAGING PROTEIN LARGE SUBUNIT,
55:00:00	6vls-D	3.1	19.8	93	963	10	MOLECULE:	MALTOSE/MALTODEXTRIN-BINDING PERIPLASMIC PROTEIN,
56:00:00	8th8-J	3.1	51.8	103	372	5	MOLECULE:	DYNEIN REGULATORY COMPLEX PROTEIN 1/2 N-TERMINAL
57:00:00	7e7i-A	3.1	7.4	71	2011	8	MOLECULE:	RETINAL-SPECIFIC PHOSPHOLIPID-TRANSPORTING ATPASE
58:00:00	6q0x-A	3.1	7.3	113	373	11	MOLECULE:	SORTING NEXIN MVP1
59:00:00	6cp3-7	3.1	14.3	114	171	8	MOLECULE:	ATP SYNTHASE SUBUNIT 9, MITOCHONDRIAL
60:00:00	3qwe-A	3.1	47.3	123	260	7	MOLECULE:	GEM-INTERACTING PROTEIN
61:00:00	7oqe-K	3.1	6.4	89	406	7	MOLECULE:	PROTEIN NAM8
62:00:00	7xdi-D	3.1	4.8	71	124	18	MOLECULE:	VP1
63:00:00	5vjx-J	3.1	4.2	59	64	10	MOLECULE:	CLOCK-INTERACTING PACEMAKER
64:00:00	6pl5-A	3.1	3.9	53	336	8	MOLECULE:	PEPTIDOGLYCAN GLYCOSYLTRANSFERASE RODA
65:00:00	7wkk-B	3.1	5.5	66	1482	6	MOLECULE:	MGC83295 PROTEIN
66:00:00	8ceg-B	3.1	3.2	76	214	7	MOLECULE:	CBY1-INTERACTING BAR DOMAIN-CONTAINING PROTEIN 1

67:00:00	6d5f-a	3.1	15	77	131	5	MOLECULE:	FIMBRIAL PROTEIN
68:00:00	7xc2-A	3.1	3.2	53	858	9	MOLECULE:	CNL9
69:00:00	4dci-B	3.1	3.8	72	148	10	MOLECULE:	UNCHARACTERIZED PROTEIN
70:00:00	8qfc-D	3.1	2.6	75	185	4	MOLECULE:	60S RIBOSOMAL PROTEIN L10A
71:00:00	3s4w-A	3.1	4.2	71	1206	3	MOLECULE:	FANCONI ANEMIA GROUP I PROTEIN HOMOLOG
72:00:00	7ty0-C	3.1	2.8	57	488	7	MOLECULE:	GLYCOPROTEIN G
73:00:00	6gy6-A	3.1	7.2	104	365	13	MOLECULE:	XAXA
74:00:00	3rip-A	3.1	3.1	54	568	6	MOLECULE:	GAMMA-TUBULIN COMPLEX COMPONENT 4
75:00:00	5mpd-U	3.1	24.5	113	298	6	MOLECULE:	26S PROTEASOME REGULATORY SUBUNIT RPN10
76:00:00	8tl7-A	3.1	3.4	74	694	0	MOLECULE:	COMPUTATIONALLY DESIGNED PROTEIN
77:00:00	6fze-A	3.1	11.2	71	209	7	MOLECULE:	PUTATIVE SURFACE PROTEIN
78:00:00	6nbx-C	3.1	10.3	68	100	10	MOLECULE:	NAD(P)H-QUINONE OXIDOREDUCTASE SUBUNIT 1
79:00:00	7v2c-j	3.1	21.4	74	115	16	MOLECULE:	NADH DEHYDROGENASE [UBIQUINONE] FLAVOPROTEIN 1,
80:00:00	5oxf-C	3.1	12.9	126	601	6	MOLECULE:	GTP-BINDING PROTEIN
81:00:00	5dlq-B	3.1	7.3	74	1052	11	MOLECULE:	EXPORTIN-4
82:00:00	6oqv-Y	3.1	5.5	78	156	5	MOLECULE:	ATP SYNTHASE SUBUNIT DELTA
83:00:00	4aj5-L	3.1	9.2	74	106	7	MOLECULE:	SPINDLE AND KINETOCHORE-ASSOCIATED PROTEIN 1
84:00:00	3jax-A	3.1	4.9	50	938	16	MOLECULE:	MYOSIN 2 HEAVY CHAIN
85:00:00	2efr-B	3.1	6.6	79	154	3	MOLECULE:	GENERAL CONTROL PROTEIN GCN4 AND TROPOMYOSIN 1 AL
86:00:00	7eao-C	3.1	3.9	46	62	9	MOLECULE:	POLYUBIQUITIN-C
87:00:00	3dtp-B	3.1	26.3	112	940	4	MOLECULE:	MYOSIN 2 HEAVY CHAIN CHIMERA OF SMOOTH AND CARDIA
88:00:00	5yma-A	3	6.5	85	179	2	MOLECULE:	PUTATIVE RRNA PROCESSING PROTEIN
89:00:00	3uux-D	3	4.5	73	135	7	MOLECULE:	MITOCHONDRIA FISSION 1 PROTEIN
90:00:00	8dt0-A	3	28.1	62	140	2	MOLECULE:	SCAFFOLDING PROTEIN FUNCTIONAL SITES
91:00:00	7wb4-E	3	31.9	92	1363	5	MOLECULE:	OUTER NUP133
92:00:00	5dfz-A	3	52	85	343	5	MOLECULE:	VACUOLAR PROTEIN SORTING-ASSOCIATED PROTEIN 38
93:00:00	7wkk-D	3	22.8	54	1115	9	MOLECULE:	MGC83295 PROTEIN
94:00:00	4mu6-A	3	5.9	100	274	11	MOLECULE:	KINECTIN 1 (KINESIN RECEPTOR)
95:00:00	7wji-A	3	4.6	57	1763	9	MOLECULE:	PROTEIN UNC-80 HOMOLOG
96:00:00	6jfk-A	3	11.8	111	428	5	MOLECULE:	MITOFUSIN-2,CDNA FLJ57997, HIGHLY SIMILAR TO TRAN
97:00:00	8ag8-A	3	2.9	60	106	7	MOLECULE:	FLUORESCENCE RECOVERY-LIKE PROTEIN
98:00:00	7o3x-B	3	28.4	80	215	10	MOLECULE:	PROTEIN SLL0617
99:00:00	7nyw-C	3	9.8	88	335	6	MOLECULE:	CHROMOSOME PARTITION PROTEIN MUKB
0:00	8bd7-L	3	31.3	97	303	7	MOLECULE:	IFT88
1:00	3plt-A	3	5.7	88	214	10	MOLECULE:	SPHINGOLIPID LONG CHAIN BASE-RESPONSIVE PROTEIN L
2:00	8h2j-A	3	6.4	67	92	7	MOLECULE:	P26
3:00	6r7l-E	3	17.1	78	98	6	MOLECULE:	SECG
4:00	4bk0-B	3	7.2	74	97	7	MOLECULE:	ATP-DEPENDENT DNA HELICASE Q5
5:00	1lj2-B	3	4.7	66	110	6	MOLECULE:	NONSTRUCTURAL RNA-BINDING PROTEIN 34
6:00	4lws-A	3	8.7	53	100	15	MOLECULE:	UNCHARACTERIZED PROTEIN
7:00	7loi-A	3	20.7	103	175	5	MOLECULE:	TRANSMEMBRANE PROTEIN GP41
8:00	8fhc-B	3	2.9	55	215	9	MOLECULE:	TRNA-(MS[2]IO[6]A)-HYDROXYLASE
9:00	7nh9-A	3	5.1	114	327	4	MOLECULE:	CMAX PROTEIN
10:00	7pkq-C	3	13.4	91	369	11	MOLECULE:	MS35
11:00	6arz-A	3	4.9	64	96	6	MOLECULE:	UNCHARACTERIZED PROTEIN
12:00	7zu0-F	3	5.9	63	112	13	MOLECULE:	E3 UBIQUITIN-PROTEIN LIGASE PEP5
13:00	8vc1-A	3	5.7	65	391	5	MOLECULE:	GUSTATORY RECEPTOR
14:00	8e1m-C	3	13.4	65	264	3	MOLECULE:	MITOCHONDRIAL IMPORT INNER MEMBRANE TRANSLOCASE S
15:00	8bhw-A	3	4.4	106	332	5	MOLECULE:	ECA POLYSACCHARIDE CHAIN LENGTH MODULATION PROTEI
16:00	5da9-A	3	5.1	90	433	3	MOLECULE:	PUTATIVE UNCHARACTERIZED PROTEIN,PUTATIVE UNCHARA
17:00	7p2y-a	3	11.9	75	277	17	MOLECULE:	ATP SYNTHASE SUBUNIT ALPHA
18:00	6tmh-H	3	2.2	55	71	11	MOLECULE:	INHIBITOR OF F1
19:00	7tch-A	3	13.8	73	645	3	MOLECULE:	BACITRACIN EXPORT PERMEASE PROTEIN BCEB
20:00	4zm1-B	3	5.1	91	238	10	MOLECULE:	CHAIN LENGTH DETERMINANT PROTEIN
21:00	4x0j-B	3	4.8	90	262	2	MOLECULE:	HAPTOGLOBIN-HEMOGLOBIN RECEPTOR
22:00	8ap7-c	3	4.7	57	64	5	MOLECULE:	ATP SYNTHASE SUBUNIT A

23:00	6jho-A	3	3.6	55	200	4	MOLECULE:	CAG PATHOGENICITY ISLAND PROTEIN (CAG6)
24:00:00	6ar7-C	3	2.8	57	207	5	MOLECULE:	UNCHARACTERIZED PROTEIN
25:00:00	8b9z-A	3	21.6	76	117	12	MOLECULE:	NADH-UBIQUINONE OXIDOREDUCTASE CHAIN 3
26:00:00	6qld-Y	3	27.3	99	223	12	MOLECULE:	INNER KINETOCHORE SUBUNIT MIF2
27:00:00	7jsv-a	3	3.6	57	66	5	MOLECULE:	PILIN
28:00:00	5n9j-D	3	14.6	84	135	7	MOLECULE:	MEDIATOR OF RNA POLYMERASE II TRANSCRIPTION SUBUN
29:00:00	8d2s-A	3	13.5	83	475	5	MOLECULE:	SODIUM-DEPENDENT LYSOPHOSPHATIDYLCHOLINE SYMPORTE
30:00:00	7pw9-A	3	30.9	69	1952	6	MOLECULE:	SERINE/THREONINE-PROTEIN KINASE SMG1,SERINE/THREO
31:00:00	3fey-A	3	7.6	91	761	3	MOLECULE:	NUCLEAR CAP-BINDING PROTEIN SUBUNIT 1
32:00:00	7woo-H	3	6.4	80	246	13	MOLECULE:	NUCLEOPORIN NIC96
33:00:00	8r5i-F	3	2.9	43	43	7	MOLECULE:	CORE PROTEIN A10
34:00:00	4aj5-M	3	10.7	75	109	7	MOLECULE:	SPINDLE AND KINETOCHORE-ASSOCIATED PROTEIN 1
35:00:00	4aj5-R	3	9.4	74	108	7	MOLECULE:	SPINDLE AND KINETOCHORE-ASSOCIATED PROTEIN 1
36:00:00	4aj5-O	3	9	74	109	7	MOLECULE:	SPINDLE AND KINETOCHORE-ASSOCIATED PROTEIN 1
37:00:00	4aj5-Q	3	9.4	73	102	5	MOLECULE:	SPINDLE AND KINETOCHORE-ASSOCIATED PROTEIN 1
38:00:00	7a48-B	3	1.9	40	40	8	MOLECULE:	NANOBODY 49
39:00:00	3kyp-A	3	3.4	55	171	5	MOLECULE:	NUCLEOSOME ASSEMBLY PROTEIN
40:00:00	5oqm-k	3	10.8	86	127	3	MOLECULE:	DNA-DIRECTED RNA POLYMERASE II SUBUNIT RPB1
41:00:00	4bk0-A	3	5.3	65	92	11	MOLECULE:	ATP-DEPENDENT DNA HELICASE Q5
42:00:00	6l5j-A	3	9.1	100	208	9	MOLECULE:	ROOTLETIN
43:00:00	2efl-A	3	3.6	95	281	4	MOLECULE:	FORMIN-BINDING PROTEIN 1
44:00:00	6eup-C	3	2.7	67	147	9	MOLECULE:	ADHESIN A
45:00:00	6eup-B	3	2.7	67	147	9	MOLECULE:	ADHESIN A
46:00:00	2yny-A	3	4.8	65	106	12	MOLECULE:	GENERAL CONTROL PROTEIN GCN4, PUTATIVE INNER MEMB
47:00:00	7y8r-P	2.9	40.7	78	385	8	MOLECULE:	HISTONE H3
48:00:00	5xg2-A	2.9	24	96	237	4	MOLECULE:	CHROMOSOME PARTITION PROTEIN SMC
49:00:00	7nna-A	2.9	30.1	92	194	1	MOLECULE:	KLEBICIN C ACTIVITY
50:00:00	3kkd-C	2.9	4.9	76	206	8	MOLECULE:	TRANSCRIPTIONAL REGULATOR
51:00:00	8ia3-E	2.9	7.7	78	111	6	MOLECULE:	UPSTREAM STIMULATORY FACTOR 2
52:00:00	8th8-L	2.9	13.8	89	862	4	MOLECULE:	DYNEIN REGULATORY COMPLEX PROTEIN 1/2 N-TERMINAL
53:00:00	7d8t-A	2.9	15.7	87	199	2	MOLECULE:	DNA (5'-
54:00:00	7yx4-A	2.9	30.3	95	650	8	MOLECULE:	PUTATIVE GLUCOSE-METHANOL-CHOLINE OXIDOREDUCTASE
55:00:00	8iw0-A	2.9	3.6	61	84	5	MOLECULE:	LIPRIN-BETA-1,KN MOTIF AND ANKYRIN REPEAT DOMAIN-
56:00:00	5v7p-A	2.9	3.1	53	281	0	MOLECULE:	PROTEIN-S-ISOPRENYLCYSTEINE O-METHYLTRANSFERASE
57:00:00	6yrf-A	2.9	4	56	777	13	MOLECULE:	VEGETATIVE INSECTICIDAL PROTEIN
58:00:00	6lo8-D	2.9	4.5	57	119	9	MOLECULE:	MITOCHONDRIAL IMPORT INNER MEMBRANE TRANSLOCASE S
59:00:00	6mdm-H	2.9	18.7	84	147	4	MOLECULE:	VESICLE-FUSING ATPASE
60:00:00	7pgs-N	2.9	5.6	65	1787	6	MOLECULE:	NEUROFIBROMIN
61:00:00	7emf-J	2.9	5.2	70	122	13	MOLECULE:	MEDIATOR OF RNA POLYMERASE II TRANSCRIPTION SUBUN
62:00:00	6ikn-A	2.9	5.2	88	305	7	MOLECULE:	GROWTH ARREST-SPECIFIC PROTEIN 7
63:00:00	8j8p-R	2.9	16.8	66	69	3	MOLECULE:	CTR9-LIKE PROTEIN
64:00:00	4wzi-B	2.9	9.3	71	424	8	MOLECULE:	CAMP-SPECIFIC 3',5'-CYCLIC PHOSPHODIESTERASE 4B
65:00:00	4cz8-A	2.9	8.6	74	422	8	MOLECULE:	NA+/H+ ANTIPORTER, PUTATIVE
66:00:00	8g3a-D	2.9	3.7	51	334	6	MOLECULE:	BACITRACIN EXPORT PERMEASE PROTEIN BCEB
67:00:00	8i8c-K	2.9	24.2	85	227	6	MOLECULE:	P40
68:00:00	2p4w-B	2.9	9.4	85	198	1	MOLECULE:	TRANSCRIPTIONAL REGULATORY PROTEIN ARSR FAMILY
69:00:00	3lvg-D	2.9	6.9	84	180	5	MOLECULE:	CLATHRIN HEAVY CHAIN 1
70:00:00	5j1i-A	2.9	7.3	128	346	6	MOLECULE:	PLECTIN
71:00:00	5sva-V	2.9	8.4	59	85	14	MOLECULE:	DNA-DIRECTED RNA POLYMERASE II SUBUNIT RPB1
72:00:00	8eki-C	2.9	8.8	101	701	8	MOLECULE:	PROTEIN TRANSPORT PROTEIN SEC20
73:00:00	2aze-A	2.9	5	66	149	6	MOLECULE:	TRANSCRIPTION FACTOR DP-1
74:00:00	8txr-C	2.9	31.8	93	431	8	MOLECULE:	EXODEOXYRIBONUCLEASE 7 LARGE SUBUNIT
75:00:00	5ztc-A	2.9	2.3	49	197	4	MOLECULE:	LMO2088 PROTEIN
76:00:00	1wgz-A	2.9	2.4	59	510	12	MOLECULE:	CARBOXYPEPTIDASE 1
77:00:00	7dpa-A	2.9	3.9	59	1642	10	MOLECULE:	DEDICATOR OF CYTOKINESIS PROTEIN 5
78:00:00	8soi-B	2.9	7.6	118	515	7	MOLECULE:	RB1-INDUCIBLE COILED-COIL PROTEIN 1

79:00:00	7lhe-A	2.9	12.1	89	2222	11	MOLECULE:	INOSITOL 1,4,5-TRISPHOSPHATE RECEPTOR TYPE 1
80:00:00	2wwb-B	2.9	10.9	60	68	2	MOLECULE:	PROTEIN TRANSPORT PROTEIN SEC61 SUBUNIT ALPHA ISO
81:00:00	7y7q-A	2.9	19.5	65	951	6	MOLECULE:	RNA-DEPENDENT RNA POLYMERASE
82:00:00	5xe3-E	2.9	12	64	81	6	MOLECULE:	ENDORIBONUCLEASE MAZF4
83:00:00	7z1n-O	2.9	14.2	85	570	7	MOLECULE:	DNA-DIRECTED RNA POLYMERASE III SUBUNIT RPC1
84:00:00	6ln2-A	2.9	13.6	98	437	9	MOLECULE:	GLUCAGON-LIKE PEPTIDE 1 RECEPTOR,RUBREDOXIN,GLUCA
85:00:00	7ab4-F	2.9	9.2	69	340	6	MOLECULE:	PREDICTED TRANSCRIPTIONAL REGULATOR, XRE FAMILY
86:00:00	4yxw-G	2.9	6.9	90	213	8	MOLECULE:	ATP SYNTHASE SUBUNIT ALPHA, MITOCHONDRIAL
87:00:00	6j72-A	2.9	6.2	112	561	6	MOLECULE:	ISONIAZID INDUCIBLE GENE PROTEIN INIA
88:00:00	5oqm-n	2.9	14.2	85	136	9	MOLECULE:	DNA-DIRECTED RNA POLYMERASE II SUBUNIT RPB1
89:00:00	6idp-A	2.9	8.9	85	438	9	MOLECULE:	MATE FAMILY EFFLUX TRANSPORTER
90:00:00	8dft-A	2.9	5.1	69	112	10	MOLECULE:	PILIN PROTEIN
91:00:00	8dkc-E	2.9	3.4	59	103	5	MOLECULE:	DNA-DIRECTED RNA POLYMERASE SUBUNIT ALPHA
92:00:00	3l9f-A	2.9	6	61	170	11	MOLECULE:	PUTATIVE UNCHARACTERIZED PROTEIN SMU.1604C
93:00:00	4pi0-A	2.9	15	88	390	6	MOLECULE:	UNKNOWN PEPTIDE
94:00:00	3zni-A	2.9	4.4	61	390	8	MOLECULE:	E3 UBIQUITIN-PROTEIN LIGASE CBL-B
95:00:00	8ih5-A	2.9	22.1	105	689	4	MOLECULE:	SYN-COPALYL DIPHOSPHATE SYNTHASE, CHLOROPLASTIC
96:00:00	3aei-A	2.9	3.7	63	94	11	MOLECULE:	PREFOLDIN BETA SUBUNIT 2
97:00:00	6ird-B	2.9	4.5	80	254	6	MOLECULE:	1-PHOSPHATIDYLINOSITOL 4,5-BISPHOSPHATE PHOSPHODI
98:00:00	4lin-A	2.9	7.1	87	289	9	MOLECULE:	TAIL NEEDLE PROTEIN GP26
99:00:00	8i8b-C	2.9	8.7	77	229	5	MOLECULE:	MAJOR VIRAL CAPSID PROTEIN
0:00	5apv-A	2.9	7.3	64	70	3	MOLECULE:	GENERAL CONTROL PROTEIN GCN4
1:00	8ajb-N	2.9	39.9	99	238	7	MOLECULE:	CRESCENTIN
2:00	4aj5-S	2.9	8.7	72	107	6	MOLECULE:	SPINDLE AND KINETOCHORE-ASSOCIATED PROTEIN 1
3:00	4aj5-K	2.9	8.4	72	108	8	MOLECULE:	SPINDLE AND KINETOCHORE-ASSOCIATED PROTEIN 1
4:00	7z4f-K	2.9	5.3	90	603	8	MOLECULE:	PUTATIVE STRUCTURAL PROTEIN
5:00	1qce-C	2.9	3.2	68	123	15	MOLECULE:	PROTEIN (GP41)
6:00	1qce-B	2.9	3.2	68	123	15	MOLECULE:	PROTEIN (GP41)
7:00	3uia-C	2.9	1.5	39	52	13	MOLECULE:	GLYCOPROTEIN 41
8:00	3wpo-C	2.9	3.6	50	193	10	MOLECULE:	TRIMERIC AUTOTRANSPORTER ADHESIN
9:00	6h9l-C	2.9	4.8	75	128	5	MOLECULE:	UNCHARACTERIZED PROTEIN
10:00	3wqa-C	2.9	4.9	53	201	4	MOLECULE:	TRIMERIC AUTOTRANSPORTER ADHESIN
11:00	3zmf-B	2.9	6.4	53	111	4	MOLECULE:	GENERAL CONTROL PROTEIN GCN4, PUTATIVE INNER MEMB
12:00	8pr4-X	2.8	5.2	74	128	7	MOLECULE:	ARP11
13:00	7p5j-A	2.8	31.2	83	399	6	MOLECULE:	PROTEIN TWEETY HOMOLOG 1
14:00	7rz4-A	2.8	11.9	71	993	6	MOLECULE:	GLUTAMATE RECEPTOR 2
15:00	5dfz-C	2.8	8	80	783	6	MOLECULE:	VACUOLAR PROTEIN SORTING-ASSOCIATED PROTEIN 38
16:00	7usd-A	2.8	15.2	85	1185	7	MOLECULE:	CYTOPLASMIC FMR1-INTERACTING PROTEIN 1
17:00	6zz6-C	2.8	30.5	63	83	6	MOLECULE:	STRUCTURAL MAINTENANCE OF CHROMOSOMES PROTEIN 1,S
18:00	8ip4-A	2.8	4.4	109	320	6	MOLECULE:	MAGNESIUM TRANSPORTER MRS2 HOMOLOG, MITOCHONDRIAL
19:00	2lck-A	2.8	17.1	102	296	1	MOLECULE:	MITOCHONDRIAL UNCOUPLING PROTEIN 2
20:00	4tsh-A	2.8	8.5	69	121	4	MOLECULE:	SURFACE PROTEIN ADHESIN
21:00	6o8b-B	2.8	4.2	105	657	9	MOLECULE:	STIMULATOR OF INTERFERON GENES PROTEIN
22:00	5tuu-B	2.8	4	49	106	6	MOLECULE:	TRANSCRIPTION FACTOR DP1
23:00	3b77-A	2.8	10.3	87	188	10	MOLECULE:	UNCHARACTERIZED PROTEIN
24:00:00	5xbt-A	2.8	10	84	271	10	MOLECULE:	PROBABLE TRANSCRIPTIONAL REGULATOR
25:00:00	5vj4-A	2.8	2.8	57	275	7	MOLECULE:	UNCHARACTERIZED PROTEIN
26:00:00	8t1l-Y	2.8	3.3	56	154	11	MOLECULE:	MEDIATOR OF RNA POLYMERASE II TRANSCRIPTION SUBUN
27:00:00	5nnv-D	2.8	6.2	93	252	10	MOLECULE:	CHROMOSOME PARTITION PROTEIN SMC,CHROMOSOME PARTI
28:00:00	4bpm-A	2.8	6	64	162	5	MOLECULE:	PROSTAGLANDIN E SYNTHASE, FUSION PEPTIDE
29:00:00	5t58-A	2.8	13	104	233	6	MOLECULE:	KLLA0F02343P
30:00:00	8teu-E	2.8	14.2	74	495	7	MOLECULE:	LARGE TEGUMENT PROTEIN DENEDDYLASE
31:00:00	8p62-D	2.8	4.9	61	245	11	MOLECULE:	DNA REPLICATION LICENSING FACTOR MCM2
32:00:00	8j22-C	2.8	13	83	285	6	MOLECULE:	GUANINE NUCLEOTIDE-BINDING PROTEIN G(I)/G(S)/G(T)
33:00:00	6sny-A	2.8	3.5	53	107	4	MOLECULE:	SYNTHETIC EPCR BINDING PROTEIN
34:00:00	8rt9-A	2.8	3.9	69	207	6	MOLECULE:	TRWJ PROTEIN

35:00:00	8ebt-K	2.8	9	78	172	3	MOLECULE:	GENERAL TRANSCRIPTION AND DNA REPAIR FACTOR IIH H
36:00:00	8ptx-A	2.8	31.1	103	1215	5	MOLECULE:	ELONGATOR COMPLEX PROTEIN 1
37:00:00	8hir-A	2.8	3.8	62	918	6	MOLECULE:	POTASSIUM CHANNEL SUBFAMILY T MEMBER 1
38:00:00	6ye4-A	2.8	5.4	82	236	4	MOLECULE:	BIOPOLYMER TRANSPORT PROTEIN EXBB
39:00:00	7kpx-C	2.8	4	70	1352	7	MOLECULE:	MEIOTIC MRNA STABILITY PROTEIN KINASE SSN3
40:00:00	6v35-A	2.8	7.5	79	914	11	MOLECULE:	CALCIUM-ACTIVATED POTASSIUM CHANNEL SUBUNIT ALPHA
41:00:00	1y2o-A	2.8	3.1	80	248	5	MOLECULE:	BAI1-ASSOCIATED PROTEIN 2 ISOFORM 1
42:00:00	6dlu-P	2.8	39.2	77	747	1	MOLECULE:	DYNAMIN-1
43:00:00	4uxv-A	2.8	3.1	61	522	5	MOLECULE:	SEPTATION RING FORMATION REGULATOR EZRA
44:00:00	7uxc-R	2.8	10.9	51	107	2	MOLECULE:	SERINE/THREONINE-PROTEIN KINASE MTOR
45:00:00	2e50-P	2.8	8.5	70	186	7	MOLECULE:	PROTEIN SET
46:00:00	7w76-D	2.8	7.2	78	168	5	MOLECULE:	UBIQUITIN-CONJUGATING ENZYME E2 2
47:00:00	6t5a-A	2.8	6	71	96	10	MOLECULE:	TEGUMENT PROTEIN UL51
48:00:00	1llm-C	2.8	4.1	55	87	7	MOLECULE:	5'-D(*TP*CP*CP*CP*AP*CP*GP*CP*GP*TP*GP*GP*G)-3'
49:00:00	6s3l-K	2.8	7.9	86	193	9	MOLECULE:	FLAGELLAR BIOSYNTHETIC PROTEIN FLIP
50:00:00	7xad-C	2.8	3	59	96	10	MOLECULE:	PROGRAMMED CELL DEATH 1 LIGAND 1
51:00:00	8b6j-i	2.8	12.5	85	117	7	MOLECULE:	PEPTIDASE M16 INACTIVE DOMAIN PROTEIN
52:00:00	8wcn-A	2.8	6.6	71	379	11	MOLECULE:	DIGUANYLATE CYCLASE
53:00:00	4uy3-A	2.8	4.6	80	194	9	MOLECULE:	SEPTATION RING FORMATION REGULATOR EZRA
54:00:00	8kde-G	2.8	8.2	67	145	10	MOLECULE:	PHOTOSYSTEM II CP47 REACTION CENTER PROTEIN
55:00:00	8fef-D	2.8	32.7	89	385	7	MOLECULE:	VIRULENCE FACTOR MCE FAMILY PROTEIN
56:00:00	6xxd-A	2.8	4.5	71	125	7	MOLECULE:	PILA
57:00:00	6b2z-M	2.8	10.5	85	249	5	MOLECULE:	ATP SYNTHASE SUBUNIT C, MITOCHONDRIAL
58:00:00	7wze-A	2.8	4.5	63	161	11	MOLECULE:	UNCHARACTERIZED HTH-TYPE TRANSCRIPTIONAL REGULATO
59:00:00	6em5-b	2.8	4.1	66	421	12	MOLECULE:	5.8S RIBOSOMAL RNA
60:00:00	8sgi-A	2.8	38.7	101	751	7	MOLECULE:	SODIUM/CALCIUM EXCHANGER 1
61:00:00	2kjy-A	2.8	4.2	56	60	4	MOLECULE:	PROTEIN PHOSPHATASE 1 REGULATORY SUBUNIT 12A
62:00:00	5j6f-A	2.8	4	55	352	7	MOLECULE:	3-DEOXY-D-ARABINO-HEPTULOSONATE 7-PHOSPHATE SYNTH
63:00:00	1u2m-C	2.8	2.3	63	143	5	MOLECULE:	HISTONE-LIKE PROTEIN HLP-1
64:00:00	3g9g-A	2.8	9.1	93	250	5	MOLECULE:	SUPPRESSOR OF YEAST PROFILIN DELETION
65:00:00	8sl3-A	2.8	12.6	87	578	9	MOLECULE:	ADENYLATE CYCLASE TYPE 5
66:00:00	3jc5-A	2.8	5.5	63	208	8	MOLECULE:	DNA REPLICATION LICENSING FACTOR MCM2
67:00:00	5ip0-D	2.8	7.1	69	108	7	MOLECULE:	PHA GRANULE-ASSOCIATED PROTEIN
68:00:00	8pm4-A	2.8	10.8	73	604	3	MOLECULE:	TRANSPOSASE
69:00:00	7etm-A	2.8	12.3	91	498	9	MOLECULE:	PORTAL PROTEIN
70:00:00	4tt0-B	2.8	10.6	79	131	9	MOLECULE:	DENEDDYLASE
71:00:00	8hnc-A	2.8	20.5	92	570	5	MOLECULE:	SOLUTE CARRIER ORGANIC ANION TRANSPORTER FAMILY M
72:00:00	7n15-A	2.8	13.5	73	520	3	MOLECULE:	CYCLIC NUCLEOTIDE-GATED CATION CHANNEL
73:00:00	4n5b-D	2.8	5.8	61	101	3	MOLECULE:	PHOSPHOPROTEIN
74:00:00	6jlb-C	2.8	8	93	256	3	MOLECULE:	LAMIN A/C
75:00:00	7yfv-B	2.8	4.1	47	50	9	MOLECULE:	PROTEIN FANTOM
76:00:00	1fze-A	2.8	2.9	54	81	7	MOLECULE:	FIBRINOGEN
77:00:00	8pew-a	2.8	3.2	64	187	3	MOLECULE:	TRANSCRIPTION TERMINATION FACTOR RHO
78:00:00	6tgz-F	2.8	8.2	128	352	5	MOLECULE:	55 KDA IMMEDIATE-EARLY PROTEIN 1
79:00:00	1fzb-A	2.8	3	54	81	7	MOLECULE:	FIBRINOGEN
80:00:00	8tek-N	2.8	7.4	96	187	8	MOLECULE:	DYNEIN REGULATORY COMPLEX PROTEIN 1/2 N-TERMINAL
81:00:00	5voy-K	2.8	8.8	96	216	6	MOLECULE:	V-TYPE PROTON ATPASE CATALYTIC SUBUNIT A,V-TYPE P
82:00:00	5tby-B	2.8	10.6	117	950	6	MOLECULE:	MYOSIN-7
83:00:00	8ek4-B	2.8	2.5	58	135	5	MOLECULE:	ICE-BINDING PROTEIN TIP-99A
84:00:00	3bat-D	2.8	5.2	50	80	4	MOLECULE:	MYOSIN HEAVY CHAIN, STRIATED MUSCLE/GENERAL CONTR
85:00:00	6pfp-B	2.8	6.7	75	156	8	MOLECULE:	MYOSIN-7 FUSED TO GP7 AND EB1
86:00:00	6mi4-A	2.8	6.8	72	123	6	MOLECULE:	NF-KB ESSENTIAL MODULATOR
87:00:00	5jvu-B	2.8	3.1	43	69	5	MOLECULE:	CHIMERA PROTEIN OF KINESIN HEAVY CHAIN AND MICROT
88:00:00	1am9-C	2.7	7	60	82	5	MOLECULE:	DNA (5'-
89:00:00	6wkr-C	2.7	32.8	77	607	9	MOLECULE:	UBIQUITIN
90:00:00	7rtn-A	2.7	11.1	76	270	5	MOLECULE:	OUTER CAPSID PROTEIN VP5

91:00:00	8fck-E	2.7	9.4	71	222	8	MOLECULE:	HAUS AUGMIN-LIKE COMPLEX SUBUNIT 1
92:00:00	7c4j-E	2.7	6.6	51	288	2	MOLECULE:	TRANSCRIPTION REGULATORY PROTEIN SNF12
93:00:00	4uig-A	2.7	4	59	91	7	MOLECULE:	COPPER SENSITIVE OPERON REPRESSOR
94:00:00	8jw0-b	2.7	27.9	128	617	8	MOLECULE:	PHOTOSYSTEM I PSAA
95:00:00	6ibl-A	2.7	16.3	95	397	8	MOLECULE:	THIOREDOXIN 1,BETA-1 ADRENERGIC RECEPTOR
96:00:00	6pwn-A	2.7	3.7	71	280	10	MOLECULE:	SMALL-CONDUCTANCE MECHANOSENSITIVE CHANNEL
97:00:00	8gyz-B	2.7	8.6	74	215	11	MOLECULE:	TRANSCRIPTION FACTOR TGA7
98:00:00	6qti-A	2.7	15.8	96	1038	6	MOLECULE:	NICOTINAMIDE NUCLEOTIDE TRANSHYDROGENASE
99:00:00	7tmw-R	2.7	11.8	60	499	7	MOLECULE:	RELAXIN RECEPTOR 1, GUANINE NUCLEOTIDE-BINDING PR
0:00	1na6-B	2.7	12.7	75	395	7	MOLECULE:	RESTRICTION ENDONUCLEASE ECORII
1:00	5yfp-G	2.7	20.4	70	615	13	MOLECULE:	EXOCYST COMPLEX COMPONENT SEC3
2:00	2odu-A	2.7	5.7	81	217	10	MOLECULE:	PLECTIN 1
3:00	8qq7-A	2.7	5.5	67	399	9	MOLECULE:	FAD-BINDING FR-TYPE DOMAIN-CONTAINING PROTEIN
4:00	6v3f-A	2.7	27.7	82	1234	10	MOLECULE:	NPC1-LIKE INTRACELLULAR CHOLESTEROL TRANSPORTER 1
5:00	6z16-d	2.7	4.1	57	489	7	MOLECULE:	MULTISUBUNIT NA+/H+ ANTIPORTER, A SUBUNIT
6:00	5td8-D	2.7	9.1	77	128	8	MOLECULE:	KINETOCHORE PROTEIN NDC80
7:00	8ik3-A	2.7	5.4	65	328	9	MOLECULE:	STIMULATOR OF INTERFERON GENES PROTEIN,IMMUNE PRO
8:00	5ziy-A	2.7	3.8	72	195	13	MOLECULE:	FLAGELLAR HOOK-ASSOCIATED PROTEIN 3
9:00	6djl-E	2.7	5.4	75	226	4	MOLECULE:	RAS-RELATED PROTEIN RAB-11A
10:00	8ap7-m	2.7	11.9	70	129	4	MOLECULE:	ATP SYNTHASE SUBUNIT A
11:00	8hub-B	2.7	17.2	88	545	5	MOLECULE:	AMP DEAMINASE 2
12:00	5tgz-A	2.7	8.9	97	439	1	MOLECULE:	CANNABINOID RECEPTOR 1,FLAVODOXIN,CANNABINOID REC
13:00	3mhh-C	2.7	8.3	51	93	8	MOLECULE:	UBIQUITIN CARBOXYL-TERMINAL HYDROLASE 8
14:00	2oeq-C	2.7	3.4	51	117	12	MOLECULE:	PROTEIN OF UNKNOWN FUNCTION, DUF964
15:00	3sjr-A	2.7	3.7	63	126	5	MOLECULE:	UNCHARACTERIZED PROTEIN
16:00	8ebw-H	2.7	4.3	89	532	8	MOLECULE:	TFIIH BASAL TRANSCRIPTION FACTOR COMPLEX HELICASE
17:00	2lhk-A	2.7	14.4	89	107	6	MOLECULE:	L0052
18:00	6zyx-C	2.7	16.5	107	278	8	MOLECULE:	DYNEIN HEAVY CHAIN, OUTER ARM PROTEIN
19:00	6ah0-w	2.7	33.4	99	443	5	MOLECULE:	U5SNRNA
20:00	5w82-B	2.7	9	67	101	4	MOLECULE:	PROTEIN DELTA
21:00	7o4i-W	2.7	5.4	73	312	8	MOLECULE:	GENERAL TRANSCRIPTION AND DNA REPAIR FACTOR IIH H
22:00	3pwx-A	2.7	4	85	224	9	MOLECULE:	PUTATIVE FLAGELLAR HOOK-ASSOCIATED PROTEIN
23:00	6weg-A	2.7	7.5	71	204	8	MOLECULE:	STRINGENT STARVATION PROTEIN A, REGULATOR OF TRAN
24:00:00	7b93-L	2.7	17.5	129	607	5	MOLECULE:	NADH-UBIQUINONE OXIDOREDUCTASE CHAIN 3
25:00:00	6zie-A	2.7	2.6	50	123	4	MOLECULE:	CMPX-383B
26:00:00	7p64-L	2.7	8.7	106	606	4	MOLECULE:	NADH-QUINONE OXIDOREDUCTASE SUBUNIT F
27:00:00	2ve7-D	2.7	5	76	242	5	MOLECULE:	KINETOCHORE PROTEIN HEC1, KINETOCHORE PROTEIN SPC
28:00:00	3cwz-B	2.7	3.4	54	310	9	MOLECULE:	RAS-RELATED PROTEIN RAB-6A
29:00:00	1few-A	2.7	4.9	70	173	4	MOLECULE:	SECOND MITOCHONDRIA-DERIVED ACTIVATOR OF CASPASES
30:00:00	6x1g-A	2.7	3.9	59	216	8	MOLECULE:	ULP_PROTEASE DOMAIN-CONTAINING PROTEIN
31:00:00	6ezn-B	2.7	4.3	56	110	13	MOLECULE:	DOLICHYL-DIPHOSPHOOLIGOSACCHARIDE--PROTEIN
32:00:00	5aj3-d	2.7	23.6	96	177	6	MOLECULE:	MITORIBOSOMAL 12S RRNA
33:00:00	7f3x-A	2.7	3.8	53	449	8	MOLECULE:	LPCAT3
34:00:00	8hfs-Z	2.7	8.7	73	303	5	MOLECULE:	MANNOSE-SPECIFIC PTS SYSTEM, IIC COMPONENT
35:00:00	2pih-A	2.7	11.4	62	123	3	MOLECULE:	PROTEIN YMCA
36:00:00	8eki-A	2.7	5	75	170	4	MOLECULE:	PROTEIN TRANSPORT PROTEIN SEC20
37:00:00	4j2c-A	2.7	4.2	61	108	3	MOLECULE:	SYNTAXIN-6
38:00:00	6ejq-B	2.7	14.6	83	141	6	MOLECULE:	TERMINASE SMALL SUBUNIT
39:00:00	8j0h-A	2.7	2.8	57	134	9	MOLECULE:	UNCHARACTERIZED PROTEIN C4H3.06
40:00:00	1e7p-C	2.7	3.4	62	254	6	MOLECULE:	FUMARATE REDUCTASE FLAVOPROTEIN SUBUNIT
41:00:00	5x5y-F	2.7	5.4	65	351	15	MOLECULE:	PROBABLE ATP-BINDING COMPONENT OF ABC TRANSPORTER
42:00:00	2pjw-H	2.7	5.6	63	88	8	MOLECULE:	UNCHARACTERIZED PROTEIN YHL002W
43:00:00	5dw7-A	2.7	3.1	70	309	3	MOLECULE:	GERMACRADIENOL/GEOSMIN SYNTHASE
44:00:00	6ybt-u	2.7	36	113	134	4	MOLECULE:	EUKARYOTIC TRANSLATION INITIATION FACTOR 3 SUBUNI
45:00:00	1fnt-c	2.7	3.8	61	198	5	MOLECULE:	PROTEASOME COMPONENT C7-ALPHA
46:00:00	7yfv-C	2.7	3.7	45	48	4	MOLECULE:	PROTEIN FANTOM

47:00:00	6id1-q	2.7	13.4	64	132	9 MOLECULE:	PRE-MRNA-PROCESSING-SPLICING FACTOR 8
48:00:00	2ezo-B	2.7	2.4	68	123	13 MOLECULE:	GP41
49:00:00	2ezp-B	2.7	2.4	68	123	12 MOLECULE:	GP41
50:00:00	2ezr-C	2.7	2.5	68	123	15 MOLECULE:	GP41
51:00:00	2ezq-C	2.7	2.5	68	123	13 MOLECULE:	GP41
52:00:00	2ezp-C	2.7	2.4	68	123	12 MOLECULE:	GP41
53:00:00	8dk2-C	2.7	7	85	723	7 MOLECULE:	JETA
54:00:00	2ezr-B	2.7	2.6	68	123	13 MOLECULE:	GP41
55:00:00	2ezp-A	2.7	2.4	68	123	12 MOLECULE:	GP41
56:00:00	2ezo-C	2.7	2.4	68	123	10 MOLECULE:	GP41
57:00:00	2q7c-C	2.7	2.3	36	45	3 MOLECULE:	FUSION PROTEIN BETWEEN YEAST VARIANT GCN4 AND HIV
58:00:00	3jax-B	2.7	26.3	112	940	4 MOLECULE:	MYOSIN 2 HEAVY CHAIN
59:00:00	8f3b-A	2.7	15.4	46	50	9 MOLECULE:	IQN22
60:00:00	7xe4-F	2.6	35	112	1503	6 MOLECULE:	1,3-BETA-GLUCAN SYNTHASE COMPONENT FKS1
61:00:00	8ulg-A	2.6	6.1	50	827	2 MOLECULE:	ROD CGMP-SPECIFIC 3',5'-CYCLIC PHOSPHODIESTERASE
62:00:00	8ozh-A	2.6	13.3	47	58	2 MOLECULE:	ENVELOPE GLYCOPROTEIN
63:00:00	6jni-A	2.6	10.3	68	145	7 MOLECULE:	CADR
64:00:00	7d60-B	2.6	34.4	83	288	11 MOLECULE:	CALCIUM HOMEOSTASIS MODULATOR PROTEIN 5
65:00:00	7a23-Z	2.6	6.2	77	140	3 MOLECULE:	51KDA
66:00:00	5n9j-U	2.6	7.6	102	198	7 MOLECULE:	MEDIATOR OF RNA POLYMERASE II TRANSCRIPTION SUBUN
67:00:00	5jdo-A	2.6	5.4	84	249	2 MOLECULE:	HAPTOGLOBIN-HAEMOGLOBIN RECEPTOR
68:00:00	8d8k-Q	2.6	8.5	71	205	8 MOLECULE:	PROBABLE S-ADENOSYL-L-METHIONINE-DEPENDENT RNA
69:00:00	1s7o-C	2.6	6	64	108	6 MOLECULE:	HYPOTHETICAL UPF0122 PROTEIN
70:00:00	6lo8-C	2.6	3.3	55	108	13 MOLECULE:	MITOCHONDRIAL IMPORT INNER MEMBRANE TRANSLOCASE S
71:00:00	5y5a-A	2.6	2.4	54	514	19 MOLECULE:	KLLA0F20702P
72:00:00	6en8-A	2.6	14.1	52	191	13 MOLECULE:	TRANSCRIPTIONAL REGULATOR TETR FAMILY
73:00:00	6tmi-A	2.6	5.9	67	109	1 MOLECULE:	ATP SYNTHASE SUBUNIT ALPHA
74:00:00	1gnc-A	2.6	4.9	67	175	9 MOLECULE:	GRANULOCYTE COLONY-STIMULATING FACTOR
75:00:00	4wxa-E	2.6	5	50	69	2 MOLECULE:	EKC/KEOPS COMPLEX SUBUNIT PCC1
76:00:00	8ttf-A	2.6	3.5	53	388	13 MOLECULE:	QUINOLONE RESISTANCE PROTEIN NORA
77:00:00	8q7h-A	2.6	8.6	104	1403	5 MOLECULE:	CULLIN-9
78:00:00	2v0o-A	2.6	6	84	273	5 MOLECULE:	FCH DOMAIN ONLY PROTEIN 2
79:00:00	7mbz-A	2.6	7.2	75	222	5 MOLECULE:	ABC TRANSPORTER, PERMEASE PROTEIN
80:00:00	7x0a-B	2.6	7.3	63	429	5 MOLECULE:	T-COMPLEX PROTEIN 1 SUBUNIT ALPHA
81:00:00	6hyd-A	2.6	24.9	62	1574	13 MOLECULE:	MIDASIN,MIDASIN,MIDASIN
82:00:00	8wjn-A	2.6	10.3	95	491	15 MOLECULE:	STRUCTURAL MAINTENANCE OF CHROMOSOMES PROTEIN 6
83:00:00	7yzp-C	2.6	3.7	79	448	9 MOLECULE:	DNA HAIRPIN (59-MER)
84:00:00	8bsb-B	2.6	2.8	56	162	4 MOLECULE:	METHYL-ACCEPTING CHEMOTAXIS PROTEIN
85:00:00	8eoi-K	2.6	12	85	1116	8 MOLECULE:	ER MEMBRANE PROTEIN COMPLEX SUBUNIT 1
86:00:00	7ozs-F	2.6	15.8	98	571	6 MOLECULE:	PUTATIVE RIBOSOMAL PROTEIN
87:00:00	5i6r-A	2.6	3.8	92	419	7 MOLECULE:	SLIT-ROBO RHO GTPASE-ACTIVATING PROTEIN 2
88:00:00	7oca-E	2.6	4.2	67	158	10 MOLECULE:	GLUTAMATE RECEPTOR 1
89:00:00	8qah-B	2.6	4.5	69	426	1 MOLECULE:	SC-CC-8-58
90:00:00	8imi-0	2.6	10.4	79	1045	9 MOLECULE:	APCE
91:00:00	5yfp-A	2.6	22.5	86	672	12 MOLECULE:	EXOCYST COMPLEX COMPONENT SEC3
92:00:00	7drt-B	2.6	12.6	70	496	3 MOLECULE:	PROTEIN WNT-3A
93:00:00	7jtk-C	2.6	5.7	84	427	5 MOLECULE:	FLAGELLAR RADIAL SPOKE PROTEIN 1
94:00:00	5n9j-F	2.6	9.3	58	79	12 MOLECULE:	MEDIATOR OF RNA POLYMERASE II TRANSCRIPTION SUBUN
95:00:00	7ura-A	2.6	12.8	87	432	11 MOLECULE:	ISOFORM 2 OF PROTEIN-SERINE O-PALMITOLEOYLTRANSFE
96:00:00	6s8g-G	2.6	5.4	66	244	6 MOLECULE:	LIPOPOLYSACCHARIDE ABC TRANSPORTER, ATP-BINDING P
97:00:00	3nbx-X	2.6	6	59	481	8 MOLECULE:	ATPASE RAVA
98:00:00	7yxx-A	2.6	6.2	67	1754	15 MOLECULE:	PROBABLE UBIQUITIN CARBOXYL-TERMINAL HYDROLASE FA
99:00:00	8tn1-A	2.6	3.7	60	147	12 MOLECULE:	DE NOVO DESIGNED 4 HELIX BUNDLES
0:00	7yp0-B	2.6	7.2	68	278	6 MOLECULE:	GLUTATHIONE S-TRANSFERASE
1:00	4n5c-D	2.6	5.9	62	757	5 MOLECULE:	CARGO-TRANSPORT PROTEIN YPP1
2:00	6kkk-A	2.6	4.5	54	380	9 MOLECULE:	SUGAR EFFLUX TRANSPORTER

3:00	5dfz-B	2.6	7.3	73	1224	8	MOLECULE:	VACUOLAR PROTEIN SORTING-ASSOCIATED PROTEIN 38
4:00	5img-A	2.6	19.7	92	467	2	MOLECULE:	DIPEPTIDASE
5:00	7byl-A	2.6	27	101	354	3	MOLECULE:	GREEN FLUORESCENT PROTEIN,POTASSIUM VOLTAGE-GATED
6:00	7emf-K	2.6	13.3	69	112	1	MOLECULE:	MEDIATOR OF RNA POLYMERASE II TRANSCRIPTION SUBUN
7:00	8oyv-A	2.6	10.6	68	182	9	MOLECULE:	DE NOVO DESIGNED SOLUBLE CLAUDIN
8:00	6v4a-C	2.6	7.7	95	593	13	MOLECULE:	NEUR_CHAN_LBD DOMAIN-CONTAINING PROTEIN
9:00	4xal-A	2.6	5.2	57	90	9	MOLECULE:	TEGUMENT PROTEIN VP22
10:00	5ijo-J	2.6	6.2	74	1256	8	MOLECULE:	NUCLEAR PORE COMPLEX PROTEIN NUP155
11:00	3oa7-A	2.6	8.9	85	193	15	MOLECULE:	HEAD MORPHOGENESIS PROTEIN, CHAOTIC NUCLEAR MIGRA
12:00	8a3t-C	2.6	27.1	74	1410	5	MOLECULE:	ANAPHASE-PROMOTING COMPLEX SUBUNIT CDC27
13:00	7elb-A	2.6	32.3	79	1937	9	MOLECULE:	RNA-DIRECTED RNA POLYMERASE L
14:00	5j9q-D	2.6	6.6	66	120	14	MOLECULE:	HISTONE ACETYLTRANSFERASE ESA1
15:00	6dql-A	2.6	4.8	48	227	6	MOLECULE:	REGULATOR OF PROTEINASE B ROPB
16:00	7jqe-A	2.6	3	70	357	7	MOLECULE:	ESAT-6/WXG100 SECRETION SYSTEM PROTEIN
17:00	7kak-E	2.6	26.5	67	172	6	MOLECULE:	PROTEIN TRANSPORT CHANNEL SEC61 COMPLEX, ALPHA SU
18:00	6v6d-A	2.6	5.9	74	209	8	MOLECULE:	PANNEXIN-1
19:00	5vgz-D	2.6	3.8	54	107	6	MOLECULE:	26S PROTEASOME REGULATORY SUBUNIT 7
20:00	3m62-A	2.6	5.6	57	955	14	MOLECULE:	UBIQUITIN CONJUGATION FACTOR E4
21:00	6rax-N	2.6	3.4	51	207	10	MOLECULE:	DNA REPLICATION LICENSING FACTOR MCM2
22:00	6yle-D	2.6	10.1	57	547	11	MOLECULE:	PRE-RRNA-PROCESSING PROTEIN IPI3
23:00	8b7b-E	2.6	51.5	93	123	6	MOLECULE:	TUBULIN ALPHA-1B CHAIN
24:00:00	7xqx-E	2.6	50.3	93	123	0	MOLECULE:	TUBULIN ALPHA-1B CHAIN
25:00:00	7xr1-E	2.6	50.2	93	123	0	MOLECULE:	TUBULIN ALPHA-1B CHAIN
26:00:00	6znm-x	2.6	45.8	81	142	9	MOLECULE:	ARP1 ACTIN RELATED PROTEIN 1 HOMOLOG A
27:00:00	2e7s-K	2.6	9	77	117	4	MOLECULE:	RAB GUANINE NUCLEOTIDE EXCHANGE FACTOR SEC2
28:00:00	6b3r-A	2.6	22.8	70	1502	4	MOLECULE:	PIEZO-TYPE MECHANOSENSITIVE ION CHANNEL COMPONENT
29:00:00	8pew-i	2.6	5.4	67	187	3	MOLECULE:	TRANSCRIPTION TERMINATION FACTOR RHO
30:00:00	2ezs-B	2.6	2.4	68	123	15	MOLECULE:	GP41
31:00:00	2yo1-A	2.6	8.7	83	273	6	MOLECULE:	GENERAL CONTROL PROTEIN GCN4, PUTATIVE INNER MEMB
32:00:00	2ezq-B	2.6	2.6	68	123	15	MOLECULE:	GP41
33:00:00	2ezq-A	2.6	2.6	68	123	15	MOLECULE:	GP41
34:00:00	2ezs-C	2.6	2.4	68	123	16	MOLECULE:	GP41
35:00:00	2d3e-A	2.6	13.8	74	130	8	MOLECULE:	GENERAL CONTROL PROTEIN GCN4 AND TROPOMYOSIN 1 AL
36:00:00	2wpr-B	2.6	9.7	62	97	13	MOLECULE:	TRIMERIC AUTOTRANSPORTER ADHESIN FRAGMENT
37:00:00	3mtu-A	2.6	4.4	47	75	6	MOLECULE:	TROPOMYOSIN ALPHA-1 CHAIN,MICROTUBULE-ASSOCIATED
38:00:00	4mvd-D	2.6	13.6	93	253	2	MOLECULE:	CHOLINE-PHOSPHATE CYTIDYLYLTRANSFERASE A
39:00:00	6bua-A	2.5	39.9	62	942	5	MOLECULE:	DICER-2, ISOFORM A
40:00:00	6xky-A	2.5	25.3	104	271	8	MOLECULE:	FLAGELLIN
41:00:00	6t9i-G	2.5	8.1	75	140	8	MOLECULE:	TRANSCRIPTION FACTOR SPT20
42:00:00	7xym-A	2.5	4.6	54	488	11	MOLECULE:	ENVELOPE GLYCOPROTEIN
43:00:00	8ewi-A	2.5	28.2	101	1775	5	MOLECULE:	E3 UBIQUITIN-PROTEIN LIGASE UBR5
44:00:00	6zh3-A	2.5	36.7	86	170	3	MOLECULE:	VACUOLAR PROTEIN-SORTING-ASSOCIATED PROTEIN 24
45:00:00	3jac-A	2.5	31.6	122	918	4	MOLECULE:	PIEZO-TYPE MECHANOSENSITIVE ION CHANNEL COMPONENT
46:00:00	8s7c-A	2.5	21.1	96	1030	7	MOLECULE:	VWFA AND CACHE DOMAIN-CONTAINING PROTEIN 1
47:00:00	6rjw-A	2.5	19.7	94	161	11	MOLECULE:	LYSM DOMAIN PROTEIN
48:00:00	1sc7-A	2.5	22.9	63	567	6	MOLECULE:	5'-D(*AP*AP*AP*AP*AP*GP*AP*CP*TP*T)-3'
49:00:00	8v46-A	2.5	10.5	74	412	5	MOLECULE:	ARIA ANTITOXIN
50:00:00	6xt9-A	2.5	37.6	60	567	5	MOLECULE:	BARDET-BIEDL SYNDROME 1 PROTEIN
51:00:00	4wid-A	2.5	3.3	96	353	7	MOLECULE:	RHUL123
52:00:00	7p3x-B	2.5	2.6	48	621	8	MOLECULE:	AP-3 COMPLEX SUBUNIT DELTA
53:00:00	7nyw-A	2.5	9.3	113	685	10	MOLECULE:	CHROMOSOME PARTITION PROTEIN MUKB
54:00:00	2ncg-A	2.5	4.3	58	118	7	MOLECULE:	RGA1E
55:00:00	8arl-A	2.5	8.1	87	232	5	MOLECULE:	TRYPTOPHAN-RICH ANTIGEN
56:00:00	7rmy-A	2.5	4.8	61	266	11	MOLECULE:	DE NOVO DESIGNED TUNABLE HOMODIMER, D_3-337
57:00:00	8w41-A	2.5	7.9	92	1162	10	MOLECULE:	CALMODULIN-1
58:00:00	5c1f-A	2.5	20.4	99	298	5	MOLECULE:	SEPTATION PROTEIN IMP2

59:00:00	8h6u-A	2.5	3.2	69	338	4	MOLECULE:	PRESILPHIPERFOLAN-8-BETA-OL SYNTHASE
60:00:00	8ap7-a	2.5	11	100	231	12	MOLECULE:	ATP SYNTHASE SUBUNIT A
61:00:00	7qlr-A	2.5	27.1	94	603	10	MOLECULE:	CDHS1_22 PUTATIVE TAIL FIBER PROTEIN
62:00:00	6ny2-Y	2.5	23.2	65	915	6	MOLECULE:	DNA TARGET STRAND
63:00:00	6its-A	2.5	3.6	59	147	2	MOLECULE:	METHYL-ACCEPTING CHEMOTAXIS SENSORY TRANSDUCER
64:00:00	1wa8-A	2.5	3.9	62	99	8	MOLECULE:	ESAT-6 LIKE PROTEIN ESXB
65:00:00	8jec-B	2.5	8.6	74	663	7	MOLECULE:	POTASSIUM CHANNEL SKOR
66:00:00	3vou-A	2.5	6.7	68	139	15	MOLECULE:	ION TRANSPORT 2 DOMAIN PROTEIN, VOLTAGE-GATED SOD
67:00:00	7nyw-B	2.5	10	113	858	7	MOLECULE:	CHROMOSOME PARTITION PROTEIN MUKB
68:00:00	6uo8-B	2.5	13.3	75	696	5	MOLECULE:	GAMMA-AMINOBUTYRIC ACID TYPE B RECEPTOR SUBUNIT 1
69:00:00	7uhb-K	2.5	2.8	49	75	2	MOLECULE:	SPIKE GLYCOPROTEIN
70:00:00	4myy-B	2.5	7.7	60	84	8	MOLECULE:	CURG, CURH FUSION PROTEIN
71:00:00	6wu0-B	2.5	8	91	854	8	MOLECULE:	HOPANOID BIOSYNTHESIS ASSOCIATED RND TRANSPORTER
72:00:00	3sae-A	2.5	7	65	780	8	MOLECULE:	ALPHA-BISABOLENE SYNTHASE
73:00:00	6e1k-A	2.5	9.3	88	536	9	MOLECULE:	TWO PORE CALCIUM CHANNEL PROTEIN 1
74:00:00	4gwp-A	2.5	7.1	54	112	9	MOLECULE:	MEDIATOR OF RNA POLYMERASE II TRANSCRIPTION SUBUN
75:00:00	8d8j-0	2.5	11.6	57	493	7	MOLECULE:	PROBABLE S-ADENOSYL-L-METHIONINE-DEPENDENT RNA
76:00:00	2p01-A	2.5	9.7	58	323	14	MOLECULE:	ALPHA-2-MACROGLOBULIN RECEPTOR-ASSOCIATED
77:00:00	2c5i-T	2.5	3	54	94	4	MOLECULE:	T-SNARE AFFECTING A LATE GOLGI COMPARTMENT
78:00:00	8hk0-B	2.5	12	69	379	4	MOLECULE:	DEHYDROGENASE
79:00:00	5b86-A	2.5	19.2	57	579	9	MOLECULE:	TUMOR NECROSIS FACTOR ALPHA-INDUCED PROTEIN 2
80:00:00	7ewp-A	2.5	32.6	68	577	7	MOLECULE:	PROBABLE G-PROTEIN COUPLED RECEPTOR 158
81:00:00	6yj4-o	2.5	4.3	53	83	4	MOLECULE:	NADH-UBIQUINONE OXIDOREDUCTASE CHAIN 3
82:00:00	8jwd-A	2.5	1.5	53	123	4	MOLECULE:	HISTIDINE KINASE
83:00:00	8ikg-R	2.5	9.3	66	275	9	MOLECULE:	GUANINE NUCLEOTIDE-BINDING PROTEIN G(I) SUBUNIT A
84:00:00	3f0c-A	2.5	3.4	50	193	18	MOLECULE:	TRANSCRIPTIONAL REGULATOR
85:00:00	3bm3-A	2.5	3.8	60	259	5	MOLECULE:	DNA (5'-D(*CP*AP*TP*CP*CP*AP*GP*GP*TP*AP*C)-3')
86:00:00	3lf9-A	2.5	3	66	120	8	MOLECULE:	4E10_D0_1IS1A_001_C (T161)
87:00:00	8hti-R	2.5	3.1	63	303	5	MOLECULE:	GUANINE NUCLEOTIDE-BINDING PROTEIN G(S) SUBUNIT A
88:00:00	4cem-B	2.5	15.6	114	309	5	MOLECULE:	REGULATOR OF NONSENSE TRANSCRIPTS 2
89:00:00	6uz3-A	2.5	6.1	54	1126	7	MOLECULE:	SODIUM CHANNEL PROTEIN TYPE 5 SUBUNIT ALPHA,GREEN
90:00:00	3kkb-B	2.5	2.2	57	128	12	MOLECULE:	SENSOR PROTEIN
91:00:00	8yxl-A	2.5	9.7	71	699	4	MOLECULE:	RNA-DIRECTED RNA POLYMERASE L
92:00:00	7oi8-q	2.5	9.8	91	164	7	MOLECULE:	39S RIBOSOMAL PROTEIN L2, MITOCHONDRIAL
93:00:00	8hc0-A	2.5	2.5	42	360	10	MOLECULE:	ADHESION G-PROTEIN COUPLED RECEPTOR F1
94:00:00	5wd8-A	2.5	4.5	53	90	9	MOLECULE:	UNCHARACTERIZED PROTEIN
95:00:00	8erc-A	2.5	15.9	88	441	3	MOLECULE:	LYSOPHOSPHOLIPID ACYLTRANSFERASE 7
96:00:00	2lor-A	2.5	7.8	70	108	9	MOLECULE:	TRANSMEMBRANE PROTEIN 141
97:00:00	3caz-B	2.5	4.9	80	210	6	MOLECULE:	BAR PROTEIN
98:00:00	7emf-I	2.5	5.4	58	73	5	MOLECULE:	MEDIATOR OF RNA POLYMERASE II TRANSCRIPTION SUBUN
99:00:00	8tek-E	2.5	5.9	64	219	3	MOLECULE:	DYNEIN REGULATORY COMPLEX PROTEIN 1/2 N-TERMINAL
0:00	7r5s-S	2.5	3	48	120	6	MOLECULE:	CENTROMERE PROTEIN H
1:00	3n1e-B	2.5	5.8	66	141	3	MOLECULE:	VACUOLAR PROTEIN SORTING-ASSOCIATED PROTEIN 54
2:00	2jmh-A	2.5	4.4	61	117	2	MOLECULE:	MITE ALLERGEN BLO T 5
3:00	7qoo-U	2.5	18.7	85	186	9	MOLECULE:	CENTROMERE PROTEIN C
4:00	3rx6-A	2.5	9.7	78	187	3	MOLECULE:	POLARITY SUPPRESSION PROTEIN
5:00	6ugm-X	2.5	3.1	54	73	6	MOLECULE:	HISTONE H3
6:00	6u9w-A	2.5	9.5	61	562	5	MOLECULE:	P2X PURINOCEPTOR 7
7:00	6xy6-C	2.5	3.4	45	138	13	MOLECULE:	ANTI-APOPTOTIC MEMBRANE PROTEIN
8:00	4wpe-A	2.5	20.1	100	275	3	MOLECULE:	CYTOKINESIS PROTEIN 2
9:00	8dd7-B	2.5	4.2	66	759	9	MOLECULE:	METHYLATED-DNA--PROTEIN-CYSTEINE METHYLTRANSFERAS
10:00	6cnn-A	2.5	8.5	83	360	7	MOLECULE:	INTERMEDIATE CONDUCTANCE CALCIUM-ACTIVATED POTASS
11:00	7pc1-A	2.5	4.2	50	72	10	MOLECULE:	STBA
12:00	8jjb-E	2.5	51.9	93	123	8	MOLECULE:	TUBULIN ALPHA-1B CHAIN
13:00	8clg-E	2.5	51.9	94	123	6	MOLECULE:	TUBULIN ALPHA-1B CHAIN
14:00	6ff7-K	2.5	32.7	94	213	7	MOLECULE:	RNA-BINDING MOTIF PROTEIN, X-LINKED 2

15:00	4n5b-H	2.5	5.6	68	103	4	MOLECULE:	PHOSPHOPROTEIN
16:00	8clc-E	2.5	51.8	93	123	6	MOLECULE:	TUBULIN ALPHA-1B CHAIN
17:00	7jfr-E	2.5	50.7	92	123	3	MOLECULE:	TUBULIN ALPHA-1B CHAIN
18:00	8jjc-E	2.5	52	94	123	4	MOLECULE:	TUBULIN ALPHA-1B CHAIN
19:00	5j2u-E	2.5	51	92	123	7	MOLECULE:	TUBULIN ALPHA-1B CHAIN
20:00	6sgz-H	2.5	9.7	87	442	6	MOLECULE:	ESX-3 SECRETION SYSTEM PROTEIN ECCD3
21:00	8c5v-K	2.5	55.2	134	516	5	MOLECULE:	CHEMOTAXIS PROTEIN CHEA
22:00	8q0f-p	2.5	10.1	75	174	7	MOLECULE:	NADH-UBIQUINONE OXIDOREDUCTASE CHAIN 3
23:00	2xnx-A	2.5	2.7	48	74	8	MOLECULE:	FIBRINOGEN ALPHA CHAIN
24:00:00	8cok-B	2.5	6	58	101	2	MOLECULE:	INHIBITOR OF GROWTH PROTEIN 3
25:00:00	8pew-k	2.5	5	69	187	4	MOLECULE:	TRANSCRIPTION TERMINATION FACTOR RHO
26:00:00	3c9i-B	2.5	8.1	84	242	7	MOLECULE:	TAIL NEEDLE PROTEIN GP26
27:00:00	4ckg-A	2.5	3.2	80	368	9	MOLECULE:	ARF-GAP WITH COILED-COIL, ANK REPEAT AND PH DOMAI
28:00:00	5m9e-C	2.5	1.4	40	68	3	MOLECULE:	MICROTUBULE INTEGRITY PROTEIN MAL3
29:00:00	6j5e-K	2.5	14.5	38	42	8	MOLECULE:	ENVELOPE GLYCOPROTEIN
30:00:00	4mbe-E	2.5	15.6	46	51	0	MOLECULE:	CELL DIVISION CONTROL PROTEIN 31
31:00:00	3zmf-C	2.5	8.7	64	111	8	MOLECULE:	GENERAL CONTROL PROTEIN GCN4, PUTATIVE INNER MEMB
32:00:00	1llm-D	2.5	6	59	85	7	MOLECULE:	5'-D(*TP*CP*CP*CP*AP*CP*GP*CP*GP*TP*GP*GP*G)-3'
33:00:00	7zet-A	2.4	18.2	96	377	6	MOLECULE:	CLUSTERIN
34:00:00	4tn3-B	2.4	11.8	128	362	5	MOLECULE:	TRIM5/CYCLOPHILIN A FUSION PROTEIN/T4 LYSOZYME CH
35:00:00	4gwp-B	2.4	35.7	95	492	7	MOLECULE:	MEDIATOR OF RNA POLYMERASE II TRANSCRIPTION SUBUN
36:00:00	8ifg-A	2.4	17.6	84	879	5	MOLECULE:	RBAP48-RELATED WD40 REPEAT-CONTAINING PROTEIN PRW
37:00:00	7qtt-Y	2.4	24.8	75	306	1	MOLECULE:	SPLICING FACTOR 3B SUBUNIT 3
38:00:00	8tie-c	2.4	23.8	55	610	2	MOLECULE:	NUCLEOPORIN NUP120
39:00:00	3tul-A	2.4	4.2	61	136	11	MOLECULE:	CELL INVASION PROTEIN SIPB
40:00:00	3wxx-B	2.4	22.3	73	183	4	MOLECULE:	ACRH
41:00:00	6yj4-g	2.4	12.6	78	198	5	MOLECULE:	NADH-UBIQUINONE OXIDOREDUCTASE CHAIN 3
42:00:00	8hpo-C	2.4	39.6	93	138	5	MOLECULE:	TRANSCRIPTIONAL REGULATORY PROTEIN UME1
43:00:00	7qhm-I	2.4	4.5	61	135	8	MOLECULE:	CYTOCHROME BC1 COMPLEX RIESKE IRON-SULFUR SUBUNIT
44:00:00	6fes-A	2.4	11.8	95	365	5	MOLECULE:	D12_BRIC2, A SYNTHETIC PROTEIN,D12_BRIC2, A SYNTH
45:00:00	6vz1-A	2.4	20.7	103	411	7	MOLECULE:	DIACYLGLYCEROL O-ACYLTRANSFERASE 1
46:00:00	7nb6-A	2.4	10.5	92	340	9	MOLECULE:	AI-2 TRANSPORT PROTEIN TQSA
47:00:00	3nrg-A	2.4	13.2	55	217	13	MOLECULE:	TETR FAMILY TRANSCRIPTIONAL REGULATOR
48:00:00	2qfc-A	2.4	4.5	54	284	4	MOLECULE:	PLCR PROTEIN
49:00:00	4fz4-A	2.4	3.6	53	154	11	MOLECULE:	UNCHARACTERIZED PROTEIN CONSERVED IN BACTERIA
50:00:00	2yfb-B	2.4	4.9	84	238	8	MOLECULE:	METHYL-ACCEPTING CHEMOTAXIS TRANSDUCER
51:00:00	7woo-F	2.4	8.4	79	1622	10	MOLECULE:	NUCLEOPORIN NIC96
52:00:00	5wjt-A	2.4	5.4	101	302	10	MOLECULE:	FLAGELLIN
53:00:00	7uwf-C	2.4	3.4	73	517	8	MOLECULE:	WD REPEAT-CONTAINING PROTEIN 18
54:00:00	6irr-A	2.4	4.6	59	133	8	MOLECULE:	DISRUPTED IN SCHIZOPHRENIA 1 HOMOLOG,CYCLIC AMP-D
55:00:00	7wiu-B	2.4	7.6	91	576	7	MOLECULE:	MYCOBACTIN IMPORT ATP-BINDING/PERMEASE PROTEIN IR
56:00:00	6lul-A	2.4	5.7	67	223	13	MOLECULE:	RESPIRATORY SUPERCOMPLEX FACTOR 2, MITOCHONDRIAL
57:00:00	7mx2-B	2.4	9.4	61	648	7	MOLECULE:	N-ALPHA-ACETYLTRANSFERASE 35, NATC AUXILIARY SUBU
58:00:00	8eup-3	2.4	8.2	68	194	7	MOLECULE:	RNA (1422-MER)
59:00:00	8dql-A	2.4	11.6	67	257	6	MOLECULE:	SECRETION SYSTEM PROTEIN
60:00:00	2a79-B	2.4	16.5	89	259	7	MOLECULE:	VOLTAGE-GATED POTASSIUM CHANNEL BETA-2 SUBUNIT
61:00:00	8jh7-D	2.4	14.3	96	431	4	MOLECULE:	ANTI-BRIL FAB HEAVY CHAIN
62:00:00	7qpg-W	2.4	6.4	69	779	9	MOLECULE:	PROTEIN ZWILCH HOMOLOG
63:00:00	3no6-A	2.4	3.5	58	235	5	MOLECULE:	TRANSCRIPTIONAL ACTIVATOR TENA
64:00:00	8b9z-K	2.4	2.9	53	96	6	MOLECULE:	NADH-UBIQUINONE OXIDOREDUCTASE CHAIN 3
65:00:00	6ayi-A	2.4	12	43	183	14	MOLECULE:	HTH-TYPE TRANSCRIPTIONAL REGULATOR UIDR
66:00:00	4wlp-B	2.4	15.7	72	114	6	MOLECULE:	UBIQUITIN CARBOXYL-TERMINAL HYDROLASE ISOZYME L5
67:00:00	5mnt-B	2.4	14.9	115	421	9	MOLECULE:	A2 MATURATION PROTEIN
68:00:00	7swj-A	2.4	8.2	76	300	14	MOLECULE:	INWARD RECTIFIER POTASSIUM CHANNEL
69:00:00	8buy-A	2.4	2.9	61	134	10	MOLECULE:	GRANULE ASSOCIATED RAC AND RHOG EFFECTOR PROTEIN
70:00:00	6vxm-A	2.4	3.1	57	277	5	MOLECULE:	MECHANOSENSITIVE ION CHANNEL PROTEIN 1, MITOCHOND

71:00:00	4xng-D	2.4	3.9	65	144	12	MOLECULE:	UNCHARACTERIZED PROTEIN MG218.1
72:00:00	6ajf-A	2.4	7.1	78	901	3	MOLECULE:	DRUG EXPORTERS OF THE RND SUPERFAMILY-LIKE PROTEI
73:00:00	8t53-A	2.4	7.9	63	398	8	MOLECULE:	UNDECAPRENYL-PHOSPHATE GALACTOSE PHOSPHOTRANSFERA
74:00:00	3p01-A	2.4	2.7	49	178	14	MOLECULE:	TWO-COMPONENT RESPONSE REGULATOR
75:00:00	7r5s-T	2.4	4.8	50	112	10	MOLECULE:	CENTROMERE PROTEIN H
76:00:00	3afl-A	2.4	7.5	66	766	9	MOLECULE:	OLIGO ALGINATE LYASE
77:00:00	3tix-A	2.4	6.2	60	153	10	MOLECULE:	UBIQUITIN-LIKE PROTEIN SMT3,RNA-INDUCED TRANSCRIP
78:00:00	5x3q-A	2.4	2.7	58	313	9	MOLECULE:	ENVELOPE GLYCOPROTEIN
79:00:00	5lbm-C	2.4	4	56	90	4	MOLECULE:	TRANSCRIPTIONAL REPRESSOR FRMR
80:00:00	2qup-A	2.4	2.3	53	119	9	MOLECULE:	BH1478 PROTEIN
81:00:00	7f9l-A	2.4	3.2	69	162	3	MOLECULE:	RIFIN
82:00:00	8hij-A	2.4	20.3	103	499	8	MOLECULE:	BRIL-SLC19A1 CHIMERA
83:00:00	5ijj-B	2.4	3.3	66	157	11	MOLECULE:	SPX DOMAIN
84:00:00	3lic-A	2.4	3	70	265	10	MOLECULE:	SENSOR PROTEIN
85:00:00	6zce-q	2.4	19.6	78	636	4	MOLECULE:	EUKARYOTIC TRANSLATION INITIATION FACTOR 3 SUBUNI
86:00:00	6o84-A	2.4	7.4	68	415	9	MOLECULE:	LOC100127796 PROTEIN,LOC100127796 PROTEIN,OTOP3,
87:00:00	4f52-E	2.4	6.5	78	523	6	MOLECULE:	CULLIN-1
88:00:00	8gxl-B	2.4	3.8	58	122	7	MOLECULE:	SURP AND G-PATCH DOMAIN-CONTAINING PROTEIN 1
89:00:00	6e67-B	2.4	8.9	72	476	8	MOLECULE:	BETA-2 ADRENERGIC RECEPTOR,ENDOLYSIN,GUANINE NUCL
90:00:00	8jhk-A	2.4	5.4	63	727	8	MOLECULE:	ENGULFMENT AND CELL MOTILITY PROTEIN 1
91:00:00	8gju-F	2.4	11.5	84	332	7	MOLECULE:	METHYLMALONIC ACIDURIA TYPE A PROTEIN, MITOCHONDR
92:00:00	4y66-C	2.4	21.3	94	197	7	MOLECULE:	MND1
93:00:00	5xtc-j	2.4	14.3	53	115	19	MOLECULE:	NADH DEHYDROGENASE [UBIQUINONE] IRON-SULFUR PROTE
94:00:00	8ebt-C	2.4	18.5	61	191	10	MOLECULE:	GENERAL TRANSCRIPTION AND DNA REPAIR FACTOR IIH H
95:00:00	2qqy-A	2.4	2.7	55	139	9	MOLECULE:	SIGMA B OPERON
96:00:00	7p5v-B	2.4	4.3	65	732	6	MOLECULE:	VOLUME-REGULATED ANION CHANNEL SUBUNIT LRRC8A
97:00:00	8dqk-A	2.4	5.2	65	733	5	MOLECULE:	CELLULOSE SYNTHASE-LIKE CSLF6
98:00:00	8ugc-A	2.4	15.3	72	384	3	MOLECULE:	FD15
99:00:00	3nmz-A	2.4	6.3	50	406	2	MOLECULE:	APC VARIANT PROTEIN
0:00	2py8-C	2.4	10.8	71	123	7	MOLECULE:	HYPOTHETICAL PROTEIN RBCX
1:00	6vtk-A	2.4	4.4	62	446	13	MOLECULE:	ACID-SENSING ION CHANNEL 1
2:00	6uak-A	2.4	7.6	59	298	3	MOLECULE:	SAM DEPENDENT METHYLTRANSFERASE LAHSB
3:00	3hj1-B	2.4	21	98	387	6	MOLECULE:	MINOR EDITOSOME-ASSOCIATED TUTASE
4:00	8z1e-A	2.4	7.8	64	278	6	MOLECULE:	UNCHARACTERIZED PROTEIN UL78
5:00	6wq0-A	2.4	14.5	75	131	11	MOLECULE:	DNA (301-MER)
6:00	1zbd-B	2.4	4.5	66	124	5	MOLECULE:	RABPHILIN-3A
7:00	6wqz-A	2.4	7.1	62	536	5	MOLECULE:	AUTOPHAGY-RELATED PROTEIN 9A
8:00	6poo-A	2.4	9.8	104	273	1	MOLECULE:	BIBA
9:00	4hkr-A	2.4	2.2	54	165	7	MOLECULE:	CALCIUM RELEASE-ACTIVATED CALCIUM CHANNEL PROTEIN
10:00	7pp2-A	2.4	4.3	56	442	9	MOLECULE:	EXOCYST SUBUNIT EXO70 FAMILY PROTEIN
11:00	7c79-L	2.4	6.7	60	131	7	MOLECULE:	RIBONUCLEASE MRP RNA SUBUNIT NME1
12:00	8tlq-A	2.4	7.5	70	1283	6	MOLECULE:	DNA POLYMERASE ZETA CATALYTIC SUBUNIT
13:00	7tuk-A	2.4	5.1	49	142	6	MOLECULE:	SAG PROTEIN
14:00	7zyw-E	2.4	49.8	88	119	0	MOLECULE:	TUBULIN ALPHA-1B CHAIN
15:00	8clf-E	2.4	50.8	91	121	8	MOLECULE:	TUBULIN ALPHA-1B CHAIN
16:00	8clb-E	2.4	50.8	91	121	7	MOLECULE:	TUBULIN ALPHA-1B CHAIN
17:00	8cld-E	2.4	50.6	91	121	8	MOLECULE:	DETYROSINATED TUBULIN ALPHA-1B CHAIN
18:00	8huh-E	2.4	50.6	91	121	8	MOLECULE:	TUBULIN ALPHA-1B CHAIN
19:00	6zkn-Z	2.4	9.7	75	171	4	MOLECULE:	NADH DEHYDROGENASE [UBIQUINONE] FLAVOPROTEIN 1,
20:00	6zkm-Z	2.4	9.7	75	171	4	MOLECULE:	NADH DEHYDROGENASE [UBIQUINONE] FLAVOPROTEIN 1,
21:00	5cw3-A	2.4	4.8	69	248	4	MOLECULE:	BRCA1/BRCA2-CONTAINING COMPLEX SUBUNIT 3
22:00	8cpz-B	2.4	7.1	99	2122	9	MOLECULE:	TCDA1
23:00	1kql-A	2.4	14.2	46	55	9	MOLECULE:	FUSION PROTEIN OF AND STRIATED MUSCLE ALPHA-TROPO
24:00:00	5h0n-K	2.4	12.3	37	43	16	MOLECULE:	HIV-1 GP41 NHR
25:00:00	2z5h-F	2.4	15.9	48	51	4	MOLECULE:	GENERAL CONTROL PROTEIN GCN4 AND TROPOMYOSIN ALPH
26:00:00	2r5b-C	2.4	16	42	45	2	MOLECULE:	GP41 N-PEPTIDE

27:00:00	6n5m-D	2.4	14.5	51	62	8	MOLECULE:	POLYUBIQUITIN-C
28:00:00	3vgy-C	2.4	15.3	44	49	9	MOLECULE:	ENVELOPE GLYCOPROTEIN GP160
29:00:00	5cmz-C	2.4	14.9	39	45	8	MOLECULE:	ENVELOPE GLYCOPROTEIN
30:00:00	5m9e-B	2.4	1.6	40	71	3	MOLECULE:	MICROTUBULE INTEGRITY PROTEIN MAL3
31:00:00	5jvp-F	2.4	9.6	58	84	5	MOLECULE:	CHIMERA PROTEIN OF CENTROMERE-ASSOCIATED PROTEIN
32:00:00	2q7c-B	2.4	15.4	42	45	10	MOLECULE:	FUSION PROTEIN BETWEEN YEAST VARIANT GCN4 AND HIV
33:00:00	7l8y-F	2.4	4.5	56	143	7	MOLECULE:	RH.33311 PABC-5 - HEAVY CHAIN
34:00:00	7l8t-D	2.4	4.5	56	144	7	MOLECULE:	BG505 SOSIP.V5.2 N241/N289 - GP120
35:00:00	7rai-D	2.4	4.1	45	128	13	MOLECULE:	ENVELOPE GLYCOPROTEIN GP160
36:00:00	6yvd-D	2.3	38.8	78	414	6	MOLECULE:	CONDENSIN COMPLEX SUBUNIT 2
37:00:00	8pbz-B	2.3	6.6	55	1380	4	MOLECULE:	MGP-OPERON PROTEIN 3
38:00:00	5ofb-B	2.3	17.9	85	541	2	MOLECULE:	MORC FAMILY CW-TYPE ZINC FINGER PROTEIN 2
39:00:00	8qae-A	2.3	29.6	68	211	6	MOLECULE:	SC-APCC-6-SLLA
40:00:00	7zr1-D	2.3	18	110	780	2	MOLECULE:	DOUBLE-STRAND BREAK REPAIR PROTEIN
41:00:00	3ter-B	2.3	5.3	68	121	7	MOLECULE:	MAMMALIAN STROMAL INTERACTION MOLECULE-1
42:00:00	8ipr-C	2.3	7.4	89	515	8	MOLECULE:	COMPONENT LINKED WITH THE ASSEMBLY OF CYTOCHROME'
43:00:00	6d5f-A	2.3	12	79	193	8	MOLECULE:	FIMBRIAL PROTEIN
44:00:00	5e5w-B	2.3	3.9	67	149	9	MOLECULE:	HEMAGGLUTININ-ESTERASE
45:00:00	7yfu-A	2.3	12.1	37	44	14	MOLECULE:	PROTEIN FANTOM
46:00:00	7y22-e	2.3	3.8	58	172	14	MOLECULE:	PHAGE CONNECTOR PROTEIN
47:00:00	7rvb-A	2.3	3.1	56	209	2	MOLECULE:	FERRITIN
48:00:00	6qg0-G	2.3	5.9	58	355	10	MOLECULE:	TRANSLATION INITIATION FACTOR EIF-2B SUBUNIT ALPH
49:00:00	1jfi-B	2.3	11.2	83	135	8	MOLECULE:	5'-
50:00:00	5tr1-A	2.3	23.3	65	606	3	MOLECULE:	CHLORIDE CHANNEL PROTEIN
51:00:00	7ykr-A	2.3	28.1	79	1035	6	MOLECULE:	TRANSIENT RECEPTOR POTENTIAL CATION CHANNEL SUBFA
52:00:00	8rhn-A	2.3	4.2	56	198	7	MOLECULE:	ATPASE FAMILY GENE 2 PROTEIN HOMOLOG A
53:00:00	2lqt-A	2.3	5.3	50	72	0	MOLECULE:	COILED-COIL-HELIX-COILED-COIL-HELIX DOMAIN-CONTAI
54:00:00	7b9f-D	2.3	15.1	78	485	5	MOLECULE:	ECCE5
55:00:00	8cqr-A	2.3	4.2	59	103	10	MOLECULE:	NINJURIN-1
56:00:00	2r44-A	2.3	3.4	51	330	4	MOLECULE:	UNCHARACTERIZED PROTEIN
57:00:00	6xp5-D	2.3	4.2	68	134	7	MOLECULE:	MEDIATOR OF RNA POLYMERASE II TRANSCRIPTION SUBUN
58:00:00	7d5p-B	2.3	14.7	77	413	3	MOLECULE:	DRUG TRANSPORTER, PUTATIVE
59:00:00	6vz0-B	2.3	3.1	56	76	11	MOLECULE:	PULMONARY SURFACTANT-ASSOCIATED PROTEIN B
60:00:00	7dgy-A	2.3	3.6	48	99	4	MOLECULE:	C2
61:00:00	2g8l-B	2.3	8	57	286	4	MOLECULE:	287AA LONG HYPOTHETICAL PROTEIN
62:00:00	7dwb-A	2.3	10.5	99	421	4	MOLECULE:	PANNEXIN-1
63:00:00	8b9z-h	2.3	17.5	78	145	8	MOLECULE:	NADH-UBIQUINONE OXIDOREDUCTASE CHAIN 3
64:00:00	6z5l-A	2.3	16.8	91	251	1	MOLECULE:	MATRIX PROTEIN 1
65:00:00	6icz-A	2.3	14.2	83	2253	5	MOLECULE:	PROTEIN MAGO NASHI HOMOLOG 2
66:00:00	7a6h-O	2.3	13.2	69	512	7	MOLECULE:	DNA-DIRECTED RNA POLYMERASE III SUBUNIT RPC1
67:00:00	2q83-A	2.3	18.8	70	332	9	MOLECULE:	YTAA PROTEIN
68:00:00	8z9a-D	2.3	7.1	88	357	5	MOLECULE:	ODORANT RECEPTOR, APISORCO
69:00:00	6q6e-A	2.3	9.5	88	221	8	MOLECULE:	CONDENSIN COMPLEX SUBUNIT 2,STRUCTURAL MAINTENANC
70:00:00	4gzu-A	2.3	2.6	56	436	7	MOLECULE:	FERM, RHOGEF AND PLECKSTRIN DOMAIN-CONTAINING PRO
71:00:00	6yj4-V	2.3	3.4	49	126	0	MOLECULE:	NADH-UBIQUINONE OXIDOREDUCTASE CHAIN 3
72:00:00	6bfi-B	2.3	4.1	80	806	5	MOLECULE:	VIN1
73:00:00	8bh8-A	2.3	3.7	55	523	7	MOLECULE:	PCIF1_WW DOMAIN-CONTAINING PROTEIN
74:00:00	8fbi-A	2.3	2.7	51	274	6	MOLECULE:	KWOCA_39
75:00:00	7cun-B	2.3	11.7	67	1058	7	MOLECULE:	INTEGRATOR COMPLEX SUBUNIT 1
76:00:00	4mk6-A	2.3	12.8	62	188	10	MOLECULE:	PROBABLE DIHYDROXYACETONE KINASE REGULATOR DHSK_R
77:00:00	5x11-E	2.3	7.1	63	181	8	MOLECULE:	DNA (28-MER)
78:00:00	8wlj-A	2.3	13.7	68	409	3	MOLECULE:	SYNAPTIC VESICULAR AMINE TRANSPORTER
79:00:00	8k0b-A	2.3	7.2	86	689	7	MOLECULE:	CALCIUM PERMEABLE STRESS-GATED CATION CHANNEL 1
80:00:00	6li2-A	2.3	8.4	73	347	8	MOLECULE:	CHIMERA OF G-PROTEIN COUPLED RECEPTOR 52 AND RUBR
81:00:00	6whp-A	2.3	12.1	80	428	6	MOLECULE:	CHOLINE KINASE
82:00:00	3wvo-A	2.3	5.4	81	543	4	MOLECULE:	CRISPR-ASSOCIATED PROTEIN, CSE1 FAMILY

83:00:00	8ftw-A	2.3	3.1	61	166	15	MOLECULE:	FLAGELLAR FLIT PROTEIN, FLAGELLAR FLIJ PROTEIN FU
84:00:00	3kwo-A	2.3	2.5	58	149	10	MOLECULE:	PUTATIVE BACTERIOFERRITIN
85:00:00	8ity-4	2.3	13.2	92	365	3	MOLECULE:	SNRNA-ACTIVATING PROTEIN COMPLEX SUBUNIT 1
86:00:00	6xss-A	2.3	18.2	56	260	9	MOLECULE:	C4_NAT_HFUSE-7900
87:00:00	7ux0-A	2.3	3.2	44	115	7	MOLECULE:	SPERM-EGG FUSION PROTEIN TMEM95
88:00:00	2d6y-B	2.3	14.9	57	188	7	MOLECULE:	PUTATIVE TETR FAMILY REGULATORY PROTEIN
89:00:00	7rcy-A	2.3	5.4	69	829	13	MOLECULE:	PENICILLIN-BINDING PROTEIN
90:00:00	8e9g-L	2.3	12.9	69	625	7	MOLECULE:	TWO-COMPONENT SYSTEM RESPONSE REGULATOR
91:00:00	1uus-A	2.3	3.4	66	465	8	MOLECULE:	STAT PROTEIN
92:00:00	8sl4-A	2.3	7.6	99	970	6	MOLECULE:	ADENYLATE CYCLASE TYPE 5
93:00:00	2qyw-A	2.3	3.4	54	96	7	MOLECULE:	VESICLE TRANSPORT THROUGH INTERACTION WITH T-SNAR
94:00:00	7woo-K	2.3	11.7	84	254	13	MOLECULE:	NUCLEOPORIN NIC96
95:00:00	1ku9-A	2.3	6.9	72	151	6	MOLECULE:	HYPOTHETICAL PROTEIN MJ223
96:00:00	4dlq-A	2.3	5.6	68	353	10	MOLECULE:	LATROPHILIN-1
97:00:00	7nad-8	2.3	8.7	58	58	10	MOLECULE:	25S RRNA
98:00:00	8hf2-A	2.3	2.7	52	120	10	MOLECULE:	PRA1 FAMILY PROTEIN
99:00:00	5n8k-C	2.3	4.8	58	80	10	MOLECULE:	GALACTOCEREBROSIDASE
0:00	7w5a-2	2.3	2.2	57	123	2	MOLECULE:	PRE-MRNA-PROCESSING-SPLICING FACTOR 8
1:00	8fck-H	2.3	5.7	68	218	12	MOLECULE:	HAUS AUGMIN-LIKE COMPLEX SUBUNIT 1
2:00	8q3v-b	2.3	3.4	56	70	9	MOLECULE:	TETRAHYDROMETHANOPTERIN S-METHYLTRANSFERASE SUBUN
3:00	7n84-b	2.3	3.2	60	675	10	MOLECULE:	NUCLEOPORIN NUP188
4:00	8jlf-A	2.3	3.8	71	592	8	MOLECULE:	SYNAPTIC VESICLE GLYCOPROTEIN 2A
5:00	8b9z-m	2.3	12.7	81	108	6	MOLECULE:	NADH-UBIQUINONE OXIDOREDUCTASE CHAIN 3
6:00	1xdo-A	2.3	3.7	53	687	11	MOLECULE:	POLYPHOSPHATE KINASE
7:00	8ap7-f	2.3	14.7	78	135	6	MOLECULE:	ATP SYNTHASE SUBUNIT A
8:00	6fj3-A	2.3	8.4	71	562	6	MOLECULE:	PARATHYROID HORMONE/PARATHYROID HORMONE-RELATED P
9:00	6f1y-f	2.3	3.6	46	280	7	MOLECULE:	CYTOPLASMIC DYNEIN 1 HEAVY CHAIN 1,DYNEIN HEAVY C
10:00	3c64-A	2.3	3.9	52	152	4	MOLECULE:	PFEMP1 VARIANT 2 OF STRAIN MC
11:00	1tjl-A	2.3	3.7	61	145	3	MOLECULE:	DNAK SUPPRESSOR PROTEIN
12:00	5ebz-A	2.3	8.9	115	655	5	MOLECULE:	INHIBITOR OF NUCLEAR FACTOR KAPPA-B KINASE SUBUNI
13:00	7qj2-G	2.3	31.7	70	640	4	MOLECULE:	GAMMA-TUBULIN COMPLEX COMPONENT 5
14:00	4p1n-B	2.3	6.3	56	207	11	MOLECULE:	ATG1 TMIT
15:00	6xz6-A	2.3	6	75	213	5	MOLECULE:	GARP DOMAIN-CONTAINING PROTEIN
16:00	8hf3-A	2.3	9.3	101	295	5	MOLECULE:	PALMITOYLTRANSFERASE ZDHHC9
17:00	5a7d-R	2.3	18.5	76	311	11	MOLECULE:	PINS
18:00	8ear-A	2.3	8.4	102	2389	7	MOLECULE:	INOSITOL 1,4,5-TRISPHOSPHATE RECEPTOR TYPE 1
19:00	8qa2-A	2.3	6.1	70	215	4	MOLECULE:	GAP JUNCTION BETA-2 PROTEIN
20:00	6lcn-F	2.3	8.6	68	309	10	MOLECULE:	SERINE O-ACETYLTRANSFERASE
21:00	8ikj-R	2.3	2.8	51	532	8	MOLECULE:	ADHESION G PROTEIN-COUPLED RECEPTOR E5,SOLUBLE CY
22:00	7w1m-B	2.3	11.8	117	528	5	MOLECULE:	STRUCTURAL MAINTENANCE OF CHROMOSOMES PROTEIN 1A
23:00	6br8-A	2.3	4.3	47	249	4	MOLECULE:	PROTEIN A6 HOMOLOG
24:00:00	6nsj-A	2.3	12.1	59	182	10	MOLECULE:	ACID-ACTIVATED UREA CHANNEL
25:00:00	7d2w-A	2.3	2.8	57	125	4	MOLECULE:	PRESAN DOMAIN-CONTAINING PROTEIN
26:00:00	2uxx-B	2.3	11.6	73	134	5	MOLECULE:	LYSINE-SPECIFIC HISTONE DEMETHYLASE 1
27:00:00	2oh3-A	2.3	3.5	55	149	5	MOLECULE:	COG1633: UNCHARACTERIZED CONSERVED PROTEIN
28:00:00	2fbq-A	2.3	10.4	61	213	7	MOLECULE:	PROBABLE TRANSCRIPTIONAL REGULATOR
29:00:00	8kih-A	2.3	8.3	67	291	1	MOLECULE:	DITERPENE SYNTHASE, PHMA
30:00:00	6e7e-A	2.3	5.1	65	169	12	MOLECULE:	INCLUSION MEMBRANE PROTEIN A
31:00:00	7wji-C	2.3	17.2	117	1394	6	MOLECULE:	PROTEIN UNC-80 HOMOLOG
32:00:00	3mxz-A	2.3	4.3	60	107	8	MOLECULE:	TUBULIN-SPECIFIC CHAPERONE A
33:00:00	8uc3-C	2.3	2.7	47	98	9	MOLECULE:	ALBONOURSIN SYNTHASE
34:00:00	6yxq-B	2.3	4.9	54	396	6	MOLECULE:	ACTIVATING SIGNAL COINTEGRATOR 1 COMPLEX SUBUNIT
35:00:00	8cle-E	2.3	50.1	90	120	8	MOLECULE:	TUBULIN ALPHA-1B CHAIN
36:00:00	7w2y-d	2.3	8.7	69	175	7	MOLECULE:	NADH DEHYDROGENASE [UBIQUINONE] FLAVOPROTEIN 1,
37:00:00	5kht-D	2.3	15.4	41	47	2	MOLECULE:	TROPOMYOSIN ALPHA-1 CHAIN,GENERAL CONTROL PROTEIN
38:00:00	1gzl-B	2.3	15.5	42	45	10	MOLECULE:	FUSION PROTEIN BETWEEN THE HYDROPHOBIC POCKET OF

39:00:00	5h0n-C	2.3	13	36	42	8	MOLECULE:	HIV-1 GP41 NHR
40:00:00	3jbh-H	2.3	26.8	104	964	2	MOLECULE:	MYOSIN 2 HEAVY CHAIN STRIATED MUSCLE
41:00:00	5jv3-C	2.3	14	45	64	0	MOLECULE:	CHIMERA PROTEIN OF KINESIN-LIKE PROTEIN KIF11 AND
42:00:00	2efs-C	2.3	11.4	72	154	8	MOLECULE:	GENERAL CONTROL PROTEIN GCN4 AND TROPOMYOSIN 1 AL
43:00:00	3cvf-B	2.3	9.5	55	70	4	MOLECULE:	HOMER PROTEIN HOMOLOG 3
44:00:00	3l35-C	2.3	16.5	43	45	9	MOLECULE:	GP41 N-PEPTIDE
45:00:00	8fis-B	2.3	10	47	125	6	MOLECULE:	ENVELOPE GLYCOPROTEIN GP41
46:00:00	7ur6-B	2.3	12.2	50	132	4	MOLECULE:	GP120
47:00:00	6vo3-B	2.3	4.1	43	124	7	MOLECULE:	PGV04 HEAVY CHAIN
48:00:00	2yo1-C	2.3	6.9	84	273	4	MOLECULE:	GENERAL CONTROL PROTEIN GCN4, PUTATIVE INNER MEMB
49:00:00	7rai-B	2.3	5.8	53	128	11	MOLECULE:	ENVELOPE GLYCOPROTEIN GP160
50:00:00	6vo3-F	2.3	4.1	43	124	7	MOLECULE:	PGV04 HEAVY CHAIN
51:00:00	6vo3-E	2.3	4.1	43	124	7	MOLECULE:	PGV04 HEAVY CHAIN
52:00:00	4avm-A	2.2	4.9	77	230	4	MOLECULE:	BRIDGING INTEGRATOR 2
53:00:00	7sn7-a	2.2	34.5	108	546	6	MOLECULE:	FLAGELLIN
54:00:00	6h2x-A	2.2	22	95	352	5	MOLECULE:	CHROMOSOME PARTITION PROTEIN MUKB,CHROMOSOME PART
55:00:00	5zb2-A	2.2	20.9	59	402	2	MOLECULE:	DNA REPAIR PROTEIN RAD7
56:00:00	5gao-E	2.2	5.1	48	338	0	MOLECULE:	SMALL NUCLEAR RIBONUCLEOPROTEIN-ASSOCIATED PROTEI
57:00:00	5y88-U	2.2	15.4	56	488	9	MOLECULE:	PRE-MRNA-SPLICING FACTOR 8
58:00:00	5lnc-B	2.2	13.6	94	367	6	MOLECULE:	VACUOLAR TRANSPORTER CHAPERONE 4,CORE HISTONE MAC
59:00:00	6z2w-E	2.2	31.5	90	2325	3	MOLECULE:	DNA DAMAGE CHECKPOINT PROTEIN LCD1
60:00:00	7yqh-A	2.2	12.4	97	1069	9	MOLECULE:	STRUCTURAL MAINTENANCE OF CHROMOSOMES PROTEIN 5
61:00:00	8tek-A	2.2	38.6	83	200	4	MOLECULE:	DYNEIN REGULATORY COMPLEX PROTEIN 1/2 N-TERMINAL
62:00:00	8j2f-A	2.2	2.8	44	384	5	MOLECULE:	SPHINGOMYELIN PHOSPHODIESTERASE 2
63:00:00	8g0l-B	2.2	28.5	68	925	4	MOLECULE:	N-ALPHA-ACETYLTRANSFERASE 20
64:00:00	5c8j-L	2.2	3.8	50	137	10	MOLECULE:	ANTIBODY FRAGMENT, HEAVY CHAIN
65:00:00	8xgr-R	2.2	14.4	67	305	7	MOLECULE:	GUANINE NUCLEOTIDE-BINDING PROTEIN G(I)/G(S)/G(O)
66:00:00	8k7t-B	2.2	3.6	60	160	10	MOLECULE:	HIGH AFFINITY IMMUNOGLOBULIN EPSILON RECEPTOR SUB
67:00:00	8uza-A	2.2	6.8	73	910	5	MOLECULE:	CRISPR-ASSOCIATED ENDONUCLEASE CAS9
68:00:00	5oqk-A	2.2	4.3	66	147	9	MOLECULE:	VOLTAGE-GATED HYDROGEN CHANNEL 1
69:00:00	3c98-B	2.2	12.8	85	230	11	MOLECULE:	SYNTAXIN-BINDING PROTEIN 1
70:00:00	6yj4-L	2.2	9.4	102	655	3	MOLECULE:	NADH-UBIQUINONE OXIDOREDUCTASE CHAIN 3
71:00:00	8bf9-G	2.2	9.4	62	158	6	MOLECULE:	RNA (1766)
72:00:00	8f72-B	2.2	10.3	58	595	12	MOLECULE:	TPR_REGION DOMAIN-CONTAINING PROTEIN
73:00:00	7oci-C	2.2	4.7	62	119	10	MOLECULE:	DOLICHYL-DIPHOSPHOOLIGOSACCHARIDE--PROTEIN
74:00:00	3k1s-A	2.2	4.6	58	109	3	MOLECULE:	PTS SYSTEM, CELLOBIOSE-SPECIFIC IIA COMPONENT
75:00:00	6c66-A	2.2	20.5	73	511	8	MOLECULE:	CRISPR-ASSOCIATED HELICASE, CAS3 FAMILY
76:00:00	2wzk-A	2.2	23.5	62	369	10	MOLECULE:	CULLIN-5
77:00:00	5tth-A	2.2	12.8	70	671	9	MOLECULE:	C-TERMINAL SPYCATCHER FUSION OF WILDTYPE ZEBRAFIS
78:00:00	8on7-A	2.2	4.1	57	525	9	MOLECULE:	FMRFAMIDE-GATED SODIUM CHANNEL 1 (FANAC1)
79:00:00	4h54-A	2.2	2.2	50	274	8	MOLECULE:	DIGUANYLATE CYCLASE YDEH
80:00:00	8i6v-D	2.2	29.5	76	678	8	MOLECULE:	VACUOLAR TRANSPORTER CHAPERONE COMPLEX SUBUNIT 1
81:00:00	6aay-A	2.2	21.1	107	1199	8	MOLECULE:	BERGEYELLA ZOOHELCUM CAS13B (R1177A) MUTANT
82:00:00	3o6q-A	2.2	4.1	51	145	14	MOLECULE:	STAGE II SPORULATION PROTEIN SA
83:00:00	4d6k-A	2.2	6.2	50	76	4	MOLECULE:	DEOXYNUCLEOTIDYLTRANSFERASE TERMINAL-INTERACTING
84:00:00	7e40-D	2.2	2.7	67	345	9	MOLECULE:	PROTEIN PHOSPHATE STARVATION RESPONSE 2
85:00:00	5f5p-A	2.2	5.2	86	181	10	MOLECULE:	PROTEIN SHROOM2
86:00:00	8r8q-A	2.2	11.4	77	391	8	MOLECULE:	MAJOR FACILITATOR SUPERFAMILY DOMAIN-CONTAINING P
87:00:00	4o8w-D	2.2	11.7	69	120	6	MOLECULE:	SPORE GERMINATION PROTEIN
88:00:00	6vrc-A	2.2	14.3	68	1007	10	MOLECULE:	CRISPR-ASSOCIATED ENDORIBONUCLEASE CAS13A
89:00:00	5kua-A	2.2	3.8	63	161	10	MOLECULE:	PILIN
90:00:00	2zet-C	2.2	4.7	70	141	6	MOLECULE:	RAS-RELATED PROTEIN RAB-27B
91:00:00	3aai-A	2.2	4.4	50	78	2	MOLECULE:	COPPER HOMEOSTASIS OPERON REGULATORY PROTEIN
92:00:00	7vrc-C	2.2	2.5	57	109	14	MOLECULE:	TRANSCRIPTION REGULATORY PROTEIN SNF11
93:00:00	6y92-A	2.2	9.5	67	178	12	MOLECULE:	B-LYMPHOCYTE ANTIGEN CD20
94:00:00	8d07-A	2.2	3	45	65	13	MOLECULE:	HALC3_109

95:00:00	7k10-A	2.2	3.4	44	1259	20	MOLECULE:	DNA-DEPENDENT PROTEIN KINASE CATALYTIC SUBUNIT
96:00:00	8fvt-A	2.2	3	53	96	8	MOLECULE:	3HB12
97:00:00	8pop-A	2.2	6.9	71	122	1	MOLECULE:	TERMINASE SMALL SUBUNIT
98:00:00	6rwb-A	2.2	11	102	1873	7	MOLECULE:	TOXIN,TOXIN COMPLEX SUBUNIT TCAB,PUTATIVE TOXIN S
99:00:00	7yim-A	2.2	13.8	77	592	8	MOLECULE:	ALPHA-FETOPROTEIN
0:00	3q9d-A	2.2	22.7	64	175	11	MOLECULE:	PROTEIN CPN_0803/CP_1068/CPJ0803/CPB0832
1:00	8wcs-A	2.2	10.9	66	1044	2	MOLECULE:	CAS13H1
2:00	4h8s-C	2.2	6.8	84	382	6	MOLECULE:	DCC-INTERACTING PROTEIN 13-BETA
3:00	7f4u-A	2.2	6.1	69	440	10	MOLECULE:	TELOMERE LENGTH REGULATION PROTEIN TEL2 HOMOLOG
4:00	2oap-1	2.2	14.3	64	498	5	MOLECULE:	TYPE II SECRETION SYSTEM PROTEIN
5:00	7eu3-D	2.2	3.6	54	499	11	MOLECULE:	NAD(P)H-QUINONE OXIDOREDUCTASE SUBUNIT 1, CHLOROP
6:00	7vcf-H	2.2	2.4	52	101	13	MOLECULE:	TIC214
7:00	8t1i-F	2.2	7.2	66	73	11	MOLECULE:	MEDIATOR OF RNA POLYMERASE II TRANSCRIPTION SUBUN
8:00	5wb2-A	2.2	9.1	68	421	7	MOLECULE:	ENVELOPE PROTEIN US28, NANOBODY 7 FUSION PROTEIN
9:00	5lc5-h	2.2	30.8	87	134	0	MOLECULE:	NADH-UBIQUINONE OXIDOREDUCTASE CHAIN 3
10:00	1vcs-A	2.2	7.2	64	102	8	MOLECULE:	VESICLE TRANSPORT THROUGH INTERACTION WITH T-
11:00	8jxo-A	2.2	8.6	55	262	5	MOLECULE:	BLCHR2
12:00	7bmh-A	2.2	5.4	78	247	8	MOLECULE:	OPSIN
13:00	6o1q-A	2.2	3.8	58	119	10	MOLECULE:	NEPHROCYSTIN-1
14:00	7zpo-M	2.2	7.9	74	438	8	MOLECULE:	KTR SYSTEM POTASSIUM UPTAKE PROTEIN A
15:00	2ewg-A	2.2	6.3	49	367	6	MOLECULE:	FARNESYL PYROPHOSPHATE SYNTHASE
16:00	1u4q-A	2.2	4.9	87	318	5	MOLECULE:	SPECTRIN ALPHA CHAIN, BRAIN
17:00	3h36-A	2.2	3.1	48	78	10	MOLECULE:	POLYRIBONUCLEOTIDE NUCLEOTIDYLTRANSFERASE
18:00	3f46-A	2.2	13.1	58	345	9	MOLECULE:	5,10-METHENYLTETRAHYDROMETHANOPTERIN HYDROGENASE
19:00	5ce3-B	2.2	18.6	81	598	7	MOLECULE:	ACTIN
20:00	4ad8-A	2.2	13.6	111	452	4	MOLECULE:	DNA REPAIR PROTEIN RECN
21:00	6ulg-N	2.2	11.9	59	483	3	MOLECULE:	FOLLICULIN
22:00	6eo1-A	2.2	6.2	61	355	10	MOLECULE:	CYCLIC NUCLEOTIDE-GATED POTASSIUM CHANNEL MLL3241
23:00	4y9j-B	2.2	17.2	79	593	9	MOLECULE:	PROTEIN ACDH-11, ISOFORM B
24:00:00	3j83-A	2.2	4.2	72	276	10	MOLECULE:	ESX-1 SECRETION-ASSOCIATED PROTEIN ESPB
25:00:00	4k2u-A	2.2	4.4	71	230	8	MOLECULE:	ERYTHROCYTE BINDING ANTIGEN 175
26:00:00	5loi-A	2.2	17.7	75	391	8	MOLECULE:	RAD26
27:00:00	3emo-A	2.2	5.4	68	126	7	MOLECULE:	HIA (ADHESIN)
28:00:00	7rps-A	2.2	3.2	52	154	8	MOLECULE:	FIBRONECTIN-BINDING LIPOPROTEIN FBPB
29:00:00	7lzh-A	2.2	11	84	799	5	MOLECULE:	GLUTAMATE RECEPTOR 3.4
30:00:00	7t71-A	2.2	15	68	348	6	MOLECULE:	MEVALONATE 3,5-BISPHOSPHATE DECARBOXYLASE
31:00:00	4aur-A	2.2	5.6	123	564	6	MOLECULE:	LEOA
32:00:00	5wg6-A	2.2	18.2	57	517	7	MOLECULE:	HISTONE-LYSINE N-METHYLTRANSFERASE EZH2,POLYCOMB
33:00:00	7jrg-G	2.2	7.6	61	70	8	MOLECULE:	MITOCHONDRIAL-PROCESSING PEPTIDASE SUBUNIT BETA,
34:00:00	6e9n-A	2.2	12	75	409	7	MOLECULE:	D-GALACTONATE TRANSPORT
35:00:00	7d5i-A	2.2	12.4	94	443	11	MOLECULE:	CYTOCHROME D UBIQUINOL OXIDASE SUBUNIT 1
36:00:00	6ka4-A	2.2	4	47	534	11	MOLECULE:	F22L4.1 PROTEIN
37:00:00	3zhe-A	2.2	2.7	51	399	8	MOLECULE:	NONSENSE-MEDIATED MRNA DECAY PROTEIN
38:00:00	5t58-N	2.2	12.1	77	196	8	MOLECULE:	KLLA0F02343P
39:00:00	7etw-A	2.2	5.8	60	197	10	MOLECULE:	INSULIN-INDUCED GENE 2 PROTEIN
40:00:00	6f1t-f	2.2	23.6	79	929	6	MOLECULE:	ARP1 ACTIN RELATED PROTEIN 1 HOMOLOG A
41:00:00	7xzj-A	2.2	18.5	77	423	8	MOLECULE:	CTAP3
42:00:00	7qe5-A	2.2	15.9	71	616	13	MOLECULE:	SIALIC ACID TRAP TRANSPORTER PERMEASE PROTEIN SIA
43:00:00	2xwu-B	2.2	5.6	63	910	13	MOLECULE:	SUMO-CONJUGATING ENZYME UBC9
44:00:00	2o8p-A	2.2	3.6	62	220	6	MOLECULE:	14-3-3 DOMAIN CONTAINING PROTEIN
45:00:00	6f2d-G	2.2	5.8	59	89	5	MOLECULE:	FLAGELLAR BIOSYNTHETIC PROTEIN FLIP
46:00:00	4n9n-A	2.2	4.7	54	428	9	MOLECULE:	STEROL UPTAKE CONTROL PROTEIN 2, LYSOZYME
47:00:00	3oop-A	2.2	5	64	139	6	MOLECULE:	LIN2960 PROTEIN
48:00:00	5mmj-n	2.2	3.5	50	99	0	MOLECULE:	50S RIBOSOMAL PROTEIN L31
49:00:00	4g6d-B	2.2	6.3	57	198	5	MOLECULE:	RNA POLYMERASE SIGMA FACTOR RPOD
50:00:00	3ut5-E	2.2	51	94	132	7	MOLECULE:	TUBULIN ALPHA CHAIN

51:00:00	8clh-E	2.2	44.6	90	123	6	MOLECULE:	TUBULIN ALPHA-1B CHAIN
52:00:00	7b9s-Z	2.2	12.5	86	462	7	MOLECULE:	ECCE5
53:00:00	8aud-B	2.2	6.4	78	346	8	MOLECULE:	CELL WALL-ASSOCIATED HYDROLASES (INVASION-ASSOCIA
54:00:00	6o7v-a	2.2	34	68	625	10	MOLECULE:	V-TYPE PROTON ATPASE SUBUNIT D
55:00:00	6zkh-q	2.2	9.4	68	139	6	MOLECULE:	NADH DEHYDROGENASE [UBIQUINONE] FLAVOPROTEIN 1,
56:00:00	7z4a-K	2.2	5.9	92	588	9	MOLECULE:	ADAPTOR PROTEIN
57:00:00	8t1l-G	2.2	8.9	75	122	7	MOLECULE:	MEDIATOR OF RNA POLYMERASE II TRANSCRIPTION SUBUN
58:00:00	3jac-B	2.2	3.8	73	918	0	MOLECULE:	PIEZO-TYPE MECHANOSENSITIVE ION CHANNEL COMPONENT
59:00:00	2z5i-B	2.2	16.9	46	49	4	MOLECULE:	GENERAL CONTROL PROTEIN GCN4 AND TROPOMYOSIN ALPH
60:00:00	3dtp-A	2.2	5.2	50	938	14	MOLECULE:	MYOSIN 2 HEAVY CHAIN CHIMERA OF SMOOTH AND CARDIA
61:00:00	3l35-A	2.2	16.6	43	45	2	MOLECULE:	GP41 N-PEPTIDE
62:00:00	2q5u-B	2.2	15.6	40	45	8	MOLECULE:	FUSION PROTEIN BETWEEN YEAST VARIANT GCN4 AND
63:00:00	2z5i-E	2.2	15.9	45	49	4	MOLECULE:	GENERAL CONTROL PROTEIN GCN4 AND TROPOMYOSIN ALPH
64:00:00	2z5i-A	2.2	15.9	45	49	4	MOLECULE:	GENERAL CONTROL PROTEIN GCN4 AND TROPOMYOSIN ALPH
65:00:00	8jtd-B	2.2	6.7	58	126	7	MOLECULE:	GP120 PROTEIN OF HIV ENVELOPE TRIMER
66:00:00	8tni-F	2.2	6	55	126	11	MOLECULE:	HIV-1 BG505 DS-SOSIP GP120
67:00:00	6x98-I	2.2	3.1	41	122	12	MOLECULE:	BG505 HIV-1 ENV GP120
68:00:00	6u59-B	2.2	3.8	43	120	7	MOLECULE:	SOSIP.664 GP120,SOSIP.664 GP120
69:00:00	6rw6-B	2.1	25.1	97	2485	3	MOLECULE:	TCDA1
70:00:00	8qrn-7	2.1	24.2	85	571	4	MOLECULE:	12S MITOCHONDRIAL RRNA
71:00:00	8yb7-C	2.1	24.1	101	455	9	MOLECULE:	PAPAIN-LIKE PROTEASE NSP3
72:00:00	7syf-A	2.1	24.8	92	1391	7	MOLECULE:	PHOSPHATIDYLINOSITOL 3,4,5-TRISPHOSPHATE-DEPENDEN
73:00:00	6qd6-G	2.1	40.5	89	469	6	MOLECULE:	MB-CHOPQ-NB207,OUTER MEMBRANE PROTEIN,MB-CHOPQ-NB
74:00:00	5z7b-B	2.1	32.3	55	197	2	MOLECULE:	PADR FAMILY TRANSCRIPTIONAL REGULATOR
75:00:00	6gyp-E	2.1	27.7	90	512	3	MOLECULE:	CENTROMERE DNA-BINDING PROTEIN COMPLEX CBF3 SUBUN
76:00:00	7qo4-M	2.1	15.7	49	421	8	MOLECULE:	26S PROTEASOME REGULATORY SUBUNIT RPN1
77:00:00	5ucg-A	2.1	24.7	75	345	5	MOLECULE:	STAGE II SPORULATION PROTEIN E
78:00:00	2oa5-B	2.1	12.4	78	97	13	MOLECULE:	HYPOTHETICAL PROTEIN BQLF2
79:00:00	7qj0-L	2.1	9.9	89	566	8	MOLECULE:	MITOTIC-SPINDLE ORGANIZING PROTEIN 1
80:00:00	6wq2-A	2.1	21.9	80	154	6	MOLECULE:	A-DNA
81:00:00	7v2y-B	2.1	35.5	117	1226	9	MOLECULE:	THO COMPLEX SUBUNIT HPR1
82:00:00	5b52-B	2.1	15.4	47	60	6	MOLECULE:	H-NS FAMILY PROTEIN MVAT
83:00:00	2qfa-B	2.1	18	55	62	11	MOLECULE:	BACULOVIRAL IAP REPEAT-CONTAINING PROTEIN 5
84:00:00	8d8j-d	2.1	10.8	69	660	14	MOLECULE:	PROBABLE S-ADENOSYL-L-METHIONINE-DEPENDENT RNA
85:00:00	8t1l-X	2.1	3.6	42	127	7	MOLECULE:	MEDIATOR OF RNA POLYMERASE II TRANSCRIPTION SUBUN
86:00:00	6lcc-A	2.1	18.1	74	375	7	MOLECULE:	AATPS
87:00:00	5h11-A	2.1	4.5	49	494	6	MOLECULE:	UNCHARACTERIZED PROTEIN
88:00:00	8yzk-A	2.1	12.6	75	264	5	MOLECULE:	SOLUBLE CYTOCHROME B562,G-PROTEIN COUPLED RECEPTO
89:00:00	5ouz-A	2.1	5.1	65	178	6	MOLECULE:	FERRITIN
90:00:00	8rc4-o	2.1	16.5	68	372	9	MOLECULE:	SERINE/THREONINE-PROTEIN PHOSPHATASE 2A 65 KDA RE
91:00:00	5b49-B	2.1	14.6	69	247	9	MOLECULE:	UDP-2,3-DIACYLGLUCOSAMINE HYDROLASE
92:00:00	3b09-A	2.1	3.8	44	65	5	MOLECULE:	PEPTIDYL-PROLYL CIS-TRANS ISOMERASE
93:00:00	5ctq-A	2.1	26.8	83	518	5	MOLECULE:	SQUAMOUS CELL CARCINOMA ANTIGEN RECOGNIZED BY T-C
94:00:00	8h1l-B	2.1	7.3	53	423	9	MOLECULE:	N-ACYLGLUCOSAMINE 2-EPIMERASE
95:00:00	7znk-b	2.1	31.3	115	919	5	MOLECULE:	RNA
96:00:00	6h95-A	2.1	6.1	55	225	5	MOLECULE:	ALBICIDIN RESISTANCE PROTEIN
97:00:00	1wrd-A	2.1	3.5	54	98	15	MOLECULE:	TARGET OF MYB PROTEIN 1
98:00:00	4um2-A	2.1	4.9	61	510	11	MOLECULE:	TELOMERASE-BINDING PROTEIN EST1A
99:00:00	8t1l-S	2.1	7.1	63	913	5	MOLECULE:	MEDIATOR OF RNA POLYMERASE II TRANSCRIPTION SUBUN
0:00	6tgb-A	2.1	8.3	62	1450	8	MOLECULE:	DEDICATOR OF CYTOKINESIS PROTEIN 2
1:00	4f23-A	2.1	10.9	126	509	10	MOLECULE:	HEMAGGLUTININ
2:00	6v9z-A	2.1	32.4	106	715	8	MOLECULE:	ABC-TYPE BACTERIOCIN TRANSPORTER
3:00	4pl0-B	2.1	9.5	108	576	6	MOLECULE:	MICROCIN-J25 EXPORT ATP-BINDING/PERMEASE PROTEIN
4:00	6h8q-A	2.1	5.5	73	879	4	MOLECULE:	COHESIN SUBUNIT SCC3
5:00	7ooc-S	2.1	3.3	56	77	7	MOLECULE:	30S RIBOSOMAL PROTEIN S3
6:00	3ont-A	2.1	8.2	74	112	3	MOLECULE:	SPOT 14 PROTEIN

7:00	1ox3-A	2.1	3.5	59	108	10 MOLECULE:	FIBRITIN
8:00	2zdi-C	2.1	4.9	65	148	3 MOLECULE:	PREFOLDIN SUBUNIT BETA
9:00	6rfl-C	2.1	3.5	54	304	4 MOLECULE:	DNA-DEPENDENT RNA POLYMERASE SUBUNIT RPO132
10:00	2oev-A	2.1	8	111	697	5 MOLECULE:	PROGRAMMED CELL DEATH 6-INTERACTING PROTEIN
11:00	6ds9-A	2.1	3.3	53	93	8 MOLECULE:	DE NOVO DESIGNED THREE HELIX BUNDLE GRA3D
12:00	7xzi-B	2.1	3.2	59	166	5 MOLECULE:	CTAP3
13:00	8ioi-A	2.1	11.2	72	190	6 MOLECULE:	PADR FAMILY TRANSCRIPTIONAL REGULATOR
14:00	2hxo-A	2.1	12.5	53	207	2 MOLECULE:	PUTATIVE TETR-FAMILY TRANSCRIPTIONAL REGULATOR
15:00	7etr-A	2.1	7.8	53	88	6 MOLECULE:	TRANSCRIPTIONAL REGULATOR COPG FAMILY
16:00	7t5p-B	2.1	4.6	67	419	7 MOLECULE:	SUMO-INTERACTING MOTIF-CONTAINING PROTEIN 1
17:00	2raj-A	2.1	5.6	94	382	10 MOLECULE:	SORTING NEXIN-9
18:00	8j0n-D	2.1	23.7	60	156	7 MOLECULE:	ER MEMBRANE PROTEIN COMPLEX SUBUNIT 1
19:00	8ppr-N	2.1	7.8	90	217	6 MOLECULE:	KINETOCHORE-ASSOCIATED PROTEIN DSN1 HOMOLOG
20:00	8i5f-A	2.1	12.7	74	433	9 MOLECULE:	DEDICATOR OF CYTOKINESIS PROTEIN 10
21:00	8fbj-A	2.1	3.5	82	307	6 MOLECULE:	KWOCA_60
22:00	7t1s-A	2.1	3.3	49	234	6 MOLECULE:	BSTC
23:00	6z0f-A	2.1	8.8	93	384	3 MOLECULE:	ESX SECRETION SYSTEM PROTEIN YUKC
24:00:00	6c5w-A	2.1	10.7	87	285	2 MOLECULE:	CALCIUM UNIPORTER
25:00:00	7lby-A	2.1	23.8	98	755	7 MOLECULE:	CELLULOSE SYNTHASE CATALYTIC SUBUNIT [UDP-FORMING
26:00:00	8djk-B	2.1	3.7	49	281	10 MOLECULE:	3-HYDROXY-3-METHYLGLUTARYL-COENZYME A REDUCTASE
27:00:00	6al9-B	2.1	4.4	55	91	7 MOLECULE:	CHORISMATE MUTASE
28:00:00	6t8d-X	2.1	4.8	81	355	16 MOLECULE:	MAKB
29:00:00	3owa-C	2.1	18.5	96	587	9 MOLECULE:	ACYL-COA DEHYDROGENASE
30:00:00	3fn2-A	2.1	4.1	50	97	6 MOLECULE:	PUTATIVE SENSOR HISTIDINE KINASE DOMAIN
31:00:00	6hty-B	2.1	5.7	60	302	8 MOLECULE:	NUCLEAR RECEPTOR SUBFAMILY 1 GROUP I MEMBER 2,NUC
32:00:00	6xu2-A	2.1	7.2	89	1091	9 MOLECULE:	IMPORTIN-5
33:00:00	6t1z-A	2.1	3	53	393	8 MOLECULE:	LMRP INTEGRAL MEMBRANE PROTEIN
34:00:00	6upn-A	2.1	2.8	52	245	8 MOLECULE:	ENDOPHILIN-B1
35:00:00	6zyy-C	2.1	50.9	110	2901	2 MOLECULE:	DYNEIN HEAVY CHAIN, OUTER ARM PROTEIN
36:00:00	7use-B	2.1	5.3	67	1099	6 MOLECULE:	CYTOPLASMIC FMR1-INTERACTING PROTEIN 1
37:00:00	7p34-A	2.1	4.1	49	391	4 MOLECULE:	PEPTIDE ANTIBIOTIC TRANSPORTER SBMA
38:00:00	8k32-C	2.1	32.1	80	333	10 MOLECULE:	KETOL-ACID REDUCTOISOMERASE (NADP(+))
39:00:00	4qn1-A	2.1	2.1	58	372	3 MOLECULE:	E3 UBIQUITIN-PROTEIN LIGASE SHPRH
40:00:00	8a43-A	2.1	8.8	68	1549	13 MOLECULE:	DNA-DIRECTED RNA POLYMERASE I SUBUNIT RPA1
41:00:00	5tpm-A	2.1	2.1	44	155	2 MOLECULE:	PYRUVATE DEHYDROGENASE COMPLEX REPRESSOR
42:00:00	5c73-A	2.1	5.4	79	564	6 MOLECULE:	PROTEIN GLYCOSYLATION K
43:00:00	6xrx-A	2.1	19.6	50	551	6 MOLECULE:	MALTOSE/MALTODEXTRIN-BINDING PERIPLASMIC PROTEIN,
44:00:00	2o6i-B	2.1	23.4	81	430	4 MOLECULE:	HD DOMAIN PROTEIN
45:00:00	9auc-E	2.1	2.5	42	117	5 MOLECULE:	GUANINE NUCLEOTIDE-BINDING PROTEIN G(S) SUBUNIT A
46:00:00	8u7i-A	2.1	28.7	70	626	4 MOLECULE:	ENDONUCLEASE GAJA
47:00:00	6tdv-f	2.1	12.4	115	274	8 MOLECULE:	ATPTB1
48:00:00	3bvo-A	2.1	19	62	197	6 MOLECULE:	CO-CHAPERONE PROTEIN HSCB, MITOCHONDRIAL PRECURSO
49:00:00	7ekp-A	2.1	7.5	76	396	5 MOLECULE:	NEURONAL ACETYLCHOLINE RECEPTOR SUBUNIT ALPHA-7
50:00:00	8xej-X	2.1	21.6	78	358	5 MOLECULE:	ISOFORM 2 OF BASIGIN
51:00:00	1lqs-L	2.1	3.8	50	142	6 MOLECULE:	INTERLEUKIN-10 RECEPTOR ALPHA CHAIN
52:00:00	8jhu-A	2.1	8.9	85	811	8 MOLECULE:	LEGIONELLA PNEUMOPHILA EFFECTOR PROTEIN SIDI
53:00:00	8ccr-A	2.1	3.3	50	105	6 MOLECULE:	4D2 (MUTANT T19D)
54:00:00	8b6l-M	2.1	3.5	54	110	6 MOLECULE:	PROTEIN TRANSPORT PROTEIN SEC61 SUBUNIT ALPHA ISO
55:00:00	3zjc-E	2.1	7.2	81	286	9 MOLECULE:	GTPASE IMAP FAMILY MEMBER 7
56:00:00	4acl-A	2.1	3.4	51	155	8 MOLECULE:	TSSL
57:00:00	1z2c-B	2.1	3	43	346	7 MOLECULE:	RHO-RELATED GTP-BINDING PROTEIN RHOC
58:00:00	5n77-A	2.1	7.5	74	257	9 MOLECULE:	MAGNESIUM TRANSPORT PROTEIN CORA
59:00:00	1d7m-A	2.1	39.4	83	101	7 MOLECULE:	CORTEXILLIN I
60:00:00	5jqz-B	2.1	2.8	51	75	8 MOLECULE:	DE NOVO DESIGNED HOMOTETRAMER
61:00:00	8at4-F	2.1	11.4	88	387	2 MOLECULE:	HAUS AUGMIN-LIKE COMPLEX SUBUNIT 1
62:00:00	2xnx-J	2.1	2.6	46	72	9 MOLECULE:	FIBRINOGEN ALPHA CHAIN

63:00:00	1ar1-B	2.1	5.5	55	252	11 MOLECULE:	CYTOCHROME C OXIDASE
64:00:00	2z5h-C	2.1	17.8	49	52	4 MOLECULE:	GENERAL CONTROL PROTEIN GCN4 AND TROPOMYOSIN ALPH
65:00:00	7bv6-O	2.1	15.6	53	73	11 MOLECULE:	VESICLE-ASSOCIATED MEMBRANE PROTEIN 8
66:00:00	2z5i-D	2.1	16.8	46	49	4 MOLECULE:	GENERAL CONTROL PROTEIN GCN4 AND TROPOMYOSIN ALPH
67:00:00	2z5i-G	2.1	19	47	50	2 MOLECULE:	GENERAL CONTROL PROTEIN GCN4 AND TROPOMYOSIN ALPH
68:00:00	3p7k-A	2.1	16.3	42	45	10 MOLECULE:	GP41 PEPTIDE
69:00:00	2z5i-C	2.1	19.8	47	50	2 MOLECULE:	GENERAL CONTROL PROTEIN GCN4 AND TROPOMYOSIN ALPH
70:00:00	6vy2-D	2.1	5.6	52	121	15 MOLECULE:	GLYCOPROTEIN 120
71:00:00	7txd-F	2.1	4	49	122	8 MOLECULE:	ENVELOPE GLYCOPROTEIN GP120
72:00:00	6vy2-F	2.1	5.9	54	121	11 MOLECULE:	GLYCOPROTEIN 120
73:00:00	7rai-F	2.1	5.7	48	128	13 MOLECULE:	ENVELOPE GLYCOPROTEIN GP160
74:00:00	6vy2-B	2.1	6.2	53	121	13 MOLECULE:	GLYCOPROTEIN 120
75:00:00	3o40-A	2.1	3.6	63	198	14 MOLECULE:	GP41-5
76:00:00	8tnh-F	2.1	6.7	62	126	11 MOLECULE:	HIV-1 BG505 DS-SOSIP GP120
77:00:00	3jbh-B	2.1	28.4	69	964	9 MOLECULE:	MYOSIN 2 HEAVY CHAIN STRIATED MUSCLE
78:00:00	3zmf-A	2.1	6.2	52	111	8 MOLECULE:	GENERAL CONTROL PROTEIN GCN4, PUTATIVE INNER MEMB
79:00:00	8jtm-B	2.1	6.1	56	126	11 MOLECULE:	GP120 PROTEIN OF HIV ENVELOPE TRIMER
80:00:00	6u0n-Z	2.1	4.2	58	124	5 MOLECULE:	ENVELOPE GLYCOPROTEIN GP120
81:00:00	3o43-A	2.1	10.6	80	198	9 MOLECULE:	GP41-5
82:00:00	8euv-B	2.1	5.6	54	130	13 MOLECULE:	ENVELOPE GLYCOPROTEIN GP120
83:00:00	7sq1-D	2.1	5.1	41	121	10 MOLECULE:	TRANSMEMBRANE PROTEIN GP41
84:00:00	8jtm-F	2.1	5.5	54	123	11 MOLECULE:	GP120 PROTEIN OF HIV ENVELOPE TRIMER
85:00:00	7l6o-f	2.1	4.2	44	126	7 MOLECULE:	CH848.3.D0949.10.17CHIM.6R.DS.SOSIP.664 - GP120
86:00:00	7qru-E	2	12.2	79	158	11 MOLECULE:	NA+/H+ ANTIPORTER SUBUNIT D
87:00:00	7roq-A	2	31.5	135	1831	10 MOLECULE:	PHOSPHOLIPID-TRANSPORTING ATPASE ABCA1
88:00:00	6vbu-2	2	4.3	43	659	2 MOLECULE:	BARDET-BIEDL SYNDROME 18 PROTEIN
89:00:00	6th1-R	2	34	92	360	4 MOLECULE:	IMMEDIATE EARLY PROTEIN 1
90:00:00	6uen-E	2	4	50	114	8 MOLECULE:	RNA-DIRECTED RNA POLYMERASE L
91:00:00	8t5e-A	2	3.2	48	130	8 MOLECULE:	BIM_FULLDIFF
92:00:00	7w7t-A	2	18.5	84	1021	10 MOLECULE:	SARCOPLASMIC/ENDOPLASMIC RETICULUM CALCIUM ATPASE
93:00:00	8kde-3	2	26.6	63	97	5 MOLECULE:	PHOTOSYSTEM II CP47 REACTION CENTER PROTEIN
94:00:00	6sz9-D	2	10.6	102	282	8 MOLECULE:	ICMO (DOTL)
95:00:00	2odm-B	2	2.3	48	83	6 MOLECULE:	UPF0358 PROTEIN MW0995
96:00:00	8csz-D	2	5.4	78	494	6 MOLECULE:	ISCB
97:00:00	1q6u-A	2	2.9	45	213	18 MOLECULE:	FKBP-TYPE PEPTIDYL-PROLYL CIS-TRANS ISOMERASE FKP
98:00:00	5ktf-A	2	8.3	58	73	14 MOLECULE:	SCAVENGER RECEPTOR CLASS B MEMBER 1
99:00:00	8ppr-D	2	17.9	86	248	6 MOLECULE:	KINETOCHORE-ASSOCIATED PROTEIN DSN1 HOMOLOG
0:00	8eki-B	2	4.7	49	85	0 MOLECULE:	PROTEIN TRANSPORT PROTEIN SEC20
1:00	7aqw-m	2	10.3	57	70	2 MOLECULE:	NADH-UBIQUINONE OXIDOREDUCTASE CHAIN 5
2:00	4m1p-A	2	4.2	54	96	4 MOLECULE:	COPPER-SENSITIVE OPERON REPRESSOR (CSOR)
3:00	8g7m-G	2	9.5	62	343	5 MOLECULE:	60 KDA HEAT SHOCK PROTEIN, MITOCHONDRIAL
4:00	7e2c-I	2	5.6	52	799	10 MOLECULE:	TRAPP-ASSOCIATED PROTEIN TCA17
5:00	1ypy-A	2	5.3	52	182	8 MOLECULE:	VIRION MEMBRANE PROTEIN
6:00	3lss-B	2	3.6	62	468	6 MOLECULE:	SERYL-TRNA SYNTHETASE
7:00	7kuw-A	2	3.2	47	62	11 MOLECULE:	SEQUENCE-BASED DESIGNED PROTEIN NMT_0994_GUIDED_0
8:00	3cit-A	2	2.9	48	155	13 MOLECULE:	SENSOR HISTIDINE KINASE
9:00	8qt5-A	2	3.2	60	248	5 MOLECULE:	14-3-3-LIKE PROTEIN G-BOX FACTOR 14 LAMBDA,PROTEI
10:00	7f1t-A	2	8.5	86	423	5 MOLECULE:	C-C MOTIF CHEMOKINE 3,C-C CHEMOKINE RECEPTOR TYPE
11:00	6s4m-A	2	2.6	56	426	11 MOLECULE:	MAJOR FACILITATOR SUPERFAMILY DOMAIN-CONTAINING P
12:00	7aqr-V	2	3.4	49	140	4 MOLECULE:	NADH DEHYDROGENASE [UBIQUINONE] IRON-SULFUR PROTE
13:00	5ndd-A	2	7	77	567	8 MOLECULE:	LYSOZYME,PROTEINASE-ACTIVATED RECEPTOR 2,SOLUBLE
14:00	6xm1-D	2	6.2	66	181	6 MOLECULE:	VPS45
15:00	7w6k-A	2	10.1	73	410	8 MOLECULE:	GMALMT12/QUAC1
16:00	7mvv-A	2	15.9	92	1542	7 MOLECULE:	NUCLEOPORIN NUP192
17:00	8pog-A	2	6.6	56	156	14 MOLECULE:	BCSD OF ENTEROBACTER SP. 638
18:00	3esl-B	2	3.7	49	202	6 MOLECULE:	CHECKPOINT SERINE/THREONINE-PROTEIN KINASE BUB1

19:00	7d3u-D	2	12.6	72	534	7	MOLECULE:	MONOVALENT NA+/H+ ANTIPORTER SUBUNIT D
20:00	8gji-A	2	3.7	65	167	8	MOLECULE:	GCG BINDER
21:00	6tpk-A	2	13.8	70	461	1	MOLECULE:	OXYTOCIN RECEPTOR
22:00	4r1i-A	2	9	59	507	8	MOLECULE:	AMINOBENZOYL-GLUTAMATE TRANSPORTER
23:00	6f2d-F	2	8.6	83	258	11	MOLECULE:	FLAGELLAR BIOSYNTHETIC PROTEIN FLIP
24:00:00	1qoy-A	2	11.1	92	303	5	MOLECULE:	HEMOLYSIN E
25:00:00	3kkz-A	2	4	59	257	7	MOLECULE:	UNCHARACTERIZED PROTEIN Q5LES9
26:00:00	5sy1-A	2	10	81	582	10	MOLECULE:	CALMODULIN
27:00:00	6sl2-A	2	5	103	619	5	MOLECULE:	CALPONIN HOMOLOGY DOMAIN PROTEIN PUTATIVE
28:00:00	5d2s-A	2	13.7	52	91	8	MOLECULE:	FIBROIN-MODULATOR-BINDING PROTEIN-1
29:00:00	7rsl-A	2	5.5	52	235	12	MOLECULE:	SEIPIN
30:00:00	7ahd-A	2	4.8	64	547	6	MOLECULE:	ABC-TYPE PROLINE/GLYCINE BETAINE TRANSPORT SYSTEM
31:00:00	1ej6-D	2	22.4	67	417	7	MOLECULE:	LAMBDA2
32:00:00	1y9b-A	2	9.6	59	81	10	MOLECULE:	CONSERVED HYPOTHETICAL PROTEIN
33:00:00	5ys9-A	2	10.7	87	692	5	MOLECULE:	ACYL-COENZYME A OXIDASE 3
34:00:00	7mi6-A	2	28.3	119	2419	4	MOLECULE:	FUSION PROTEIN OF DYNEIN AND ENDOLYSIN
35:00:00	6v4l-A	2	13	72	479	10	MOLECULE:	TRK SYSTEM POTASSIUM UPTAKE PROTEIN TRKH
36:00:00	8cwo-T	2	3.1	54	87	7	MOLECULE:	16S RIBOSOMAL RNA
37:00:00	6w2q-A	2	8	65	195	5	MOLECULE:	JUNCTION 34
38:00:00	6s8h-F	2	5.2	58	239	16	MOLECULE:	LIPOPOLYSACCHARIDE ABC TRANSPORTER, ATP-BINDING P
39:00:00	7xn9-A	2	7.2	70	476	1	MOLECULE:	SOMATOSTATIN RECEPTOR TYPE 2,ENDO-1,4-BETA-XYLANA
40:00:00	6d03-E	2	3.8	55	466	4	MOLECULE:	TRANSFERRIN RECEPTOR PROTEIN 1
41:00:00	7a5p-u	2	12.1	43	43	2	MOLECULE:	U2 SNRNA
42:00:00	7nyx-A	2	22.4	115	1467	3	MOLECULE:	CHROMOSOME PARTITION PROTEIN MUKB
43:00:00	1e1d-A	2	7.8	55	553	5	MOLECULE:	HYDROXYLAMINE REDUCTASE
44:00:00	7xzi-E	2	14.1	88	770	7	MOLECULE:	CTAP3
45:00:00	8q72-A	2	5.2	109	750	6	MOLECULE:	JETC
46:00:00	2z5i-F	2	17.2	46	49	4	MOLECULE:	GENERAL CONTROL PROTEIN GCN4 AND TROPOMYOSIN ALPH
47:00:00	6osy-A	2	8.3	59	132	12	MOLECULE:	BG505 GP120
48:00:00	5c0r-A	2	3.8	77	270	8	MOLECULE:	HEMAGGLUTININ, ENVELOPE GLYCOPROTEIN, FIBRITIN FU
49:00:00	4r61-A	2	2.7	56	140	14	MOLECULE:	GP41-BASED CONSTRUCT COVNHR3-ABC

DALI results: MdA-1 Wasp

No:	Chain	Z	rmsd	lali	nres	%id	PDB	Description
1:00	8puz-A	7.8	6.8	139	161	11	MOLECULE:	TROPOMYOSIN
2:00	6yvu-B	7.6	10	151	1191	5	MOLECULE:	STRUCTURAL MAINTENANCE OF CHROMOSOMES PROTEI
3:00	6fkf-p	7.3	8.8	142	143	4	MOLECULE:	ATP SYNTHASE SUBUNIT ALPHA, CHLOROPLASTIC
4:00	5app-B	7.3	8.9	128	128	5	MOLECULE:	GENERAL CONTROL PROTEIN GCN4, OUTER MEMBRANE
5:00	2ocy-A	7.1	9.2	141	149	9	MOLECULE:	RAB GUANINE NUCLEOTIDE EXCHANGE FACTOR SEC2
6:00	4cpc-G	7	8.5	144	152	6	MOLECULE:	SYNAPTONEMAL COMPLEX PROTEIN 3
7:00	4gkw-A	6.9	7.8	149	158	5	MOLECULE:	SPINDLE ASSEMBLY ABNORMAL PROTEIN 6
8:00	2efr-A	6.9	7.5	145	155	6	MOLECULE:	GENERAL CONTROL PROTEIN GCN4 AND TROPOMYOSIN
9:00	8bd7-M	6.8	19.8	134	164	7	MOLECULE:	IFT88
10:00	7znk-o	6.8	8.5	133	164	9	MOLECULE:	RNA
11:00	6z9l-A	6.8	6.9	142	744	10	MOLECULE:	PRGA
12:00	7pkq-w	6.7	7.4	127	155	5	MOLECULE:	MS35
13:00	3uf1-D	6.7	6.2	112	112	11	MOLECULE:	VIMENTIN
14:00	8pqw-F	6.7	12.9	146	150	9	MOLECULE:	CYTOPLASMIC DYNEIN 1 HEAVY CHAIN 1
15:00	2fxo-A	6.7	3.7	127	129	7	MOLECULE:	MYOSIN HEAVY CHAIN, CARDIAC MUSCLE BETA ISOF
16:00	5hmo-A	6.6	3.7	121	131	11	MOLECULE:	UNCONVENTIONAL MYOSIN-X
17:00	4ytd-B	6.6	4.2	99	99	7	MOLECULE:	PROTEIN BICAUDAL D HOMOLOG 1
18:00	1d7m-A	6.6	3.3	101	101	9	MOLECULE:	CORTEXILLIN I
19:00	8q85-F	6.6	8.6	149	230	4	MOLECULE:	KINETOCHORE PROTEIN NDC80
20:00	3o0z-D	6.6	7.5	145	160	10	MOLECULE:	RHO-ASSOCIATED PROTEIN KINASE 1
21:00	6yvu-A	6.6	10.7	154	1127	6	MOLECULE:	STRUCTURAL MAINTENANCE OF CHROMOSOMES PROTEI
22:00	8xi2-L	6.5	38.5	130	468	5	MOLECULE:	MPN DOMAIN-CONTAINING PROTEIN
23:00	4xa6-A	6.5	11.5	138	168	9	MOLECULE:	GP7-MYH7(1777-1855)-EB1 CHIMERA PROTEIN
24:00:00	3mqb-A	6.5	3	109	109	6	MOLECULE:	BONE MARROW STROMAL ANTIGEN 2
25:00:00	2qa7-C	6.4	2.9	102	102	7	MOLECULE:	HUNTINGTIN-INTERACTING PROTEIN 1
26:00:00	5nvu-A	6.3	10.1	144	3169	0	MOLECULE:	DYNEIN MOTOR DOMAIN
27:00:00	6qp4-A	6.3	5.8	137	157	8	MOLECULE:	USPA1
28:00:00	7bjg-A	6.2	2.6	98	98	6	MOLECULE:	SD21996P
29:00:00	6abo-A	6.2	10.8	111	213	11	MOLECULE:	DNA REPAIR PROTEIN XRCC4
30:00:00	8i03-E	6.2	5.1	113	169	8	MOLECULE:	PAIRED AMPHIPATHIC HELIX PROTEIN PST1
31:00:00	8q85-G	6.1	8.1	145	178	6	MOLECULE:	KINETOCHORE PROTEIN NDC80
32:00:00	4f61-I	6.1	6.3	148	234	5	MOLECULE:	TUBULIN ALPHA CHAIN
33:00:00	9mhh-D	6.1	14.6	143	313	6	MOLECULE:	PHOSPHOINOSITIDE 3-KINASE REGULATORY SUBUNIT
34:00:00	6eun-A	6	4.7	112	147	8	MOLECULE:	ADHESIN
35:00:00	7apk-m	6	5.8	126	549	7	MOLECULE:	THO COMPLEX SUBUNIT 1
36:00:00	7vf2-C	6	35.8	134	184	3	MOLECULE:	PROTEIN VIRILIZER HOMOLOG
37:00:00	8i03-G	6	5.7	116	166	14	MOLECULE:	PAIRED AMPHIPATHIC HELIX PROTEIN PST1
38:00:00	3oja-A	5.9	11.5	123	482	6	MOLECULE:	LEUCINE-RICH IMMUNE MOLECULE 1
39:00:00	1x79-B	5.9	4.7	90	90	9	MOLECULE:	ADP-RIBOSYLATION FACTOR BINDING PROTEIN GGA1
40:00:00	4nqj-A	5.9	5.8	124	177	12	MOLECULE:	E3 UBIQUITIN-PROTEIN LIGASE TRIM69
41:00:00	8th8-B	5.9	9.7	153	276	7	MOLECULE:	DYNEIN REGULATORY COMPLEX PROTEIN 1/2 N-TERM
42:00:00	5cws-C	5.9	20.9	96	169	8	MOLECULE:	SAB-158 FAB LIGHT CHAIN
43:00:00	8th8-J	5.8	43	149	372	5	MOLECULE:	DYNEIN REGULATORY COMPLEX PROTEIN 1/2 N-TERM
44:00:00	5aj3-d	5.8	21.3	149	177	4	MOLECULE:	MITORIBOSOMAL 12S RRNA
45:00:00	5cff-B	5.8	4	88	88	3	MOLECULE:	MIRANDA
46:00:00	5d80-G	5.8	2.6	98	223	14	MOLECULE:	V-TYPE PROTON ATPASE CATALYTIC SUBUNIT A
47:00:00	6ff7-K	5.8	36.3	132	213	7	MOLECULE:	RNA-BINDING MOTIF PROTEIN, X-LINKED 2
48:00:00	8r1a-A	5.8	5.1	134	587	9	MOLECULE:	GUANYLATE BINDING PROTEIN 1

49:00:00	3s4r-B	5.8	3.8	91	91	13	MOLECULE:	VIMENTIN
50:00:00	6iac-E	5.7	16.7	126	146	11	MOLECULE:	PORTAL PROTEIN
51:00:00	6j5i-b	5.7	14.4	143	209	1	MOLECULE:	ATP SYNTHASE SUBUNIT ALPHA, MITOCHONDRIAL
52:00:00	3a7p-A	5.7	3.4	88	88	8	MOLECULE:	AUTOPHAGY PROTEIN 16
53:00:00	8tek-P	5.6	4.9	119	138	7	MOLECULE:	DYNEIN REGULATORY COMPLEX PROTEIN 1/2 N-TERM
54:00:00	7qoo-H	5.6	22.1	125	210	10	MOLECULE:	CENTROMERE PROTEIN C
55:00:00	8txr-C	5.6	10.6	146	431	5	MOLECULE:	EXODEOXYRIBONUCLEASE 7 LARGE SUBUNIT
56:00:00	8fef-B	5.6	22.5	146	335	13	MOLECULE:	VIRULENCE FACTOR MCE FAMILY PROTEIN
57:00:00	6fia-D	5.6	9.3	104	104	7	MOLECULE:	LINE-1 RETROTRANSPOSABLE ELEMENT ORF1 PROTEI
58:00:00	7woo-I	5.6	36.3	131	187	8	MOLECULE:	NUCLEOPORIN NIC96
59:00:00	6h9l-B	5.6	7.6	116	129	6	MOLECULE:	UNCHARACTERIZED PROTEIN
60:00:00	8ovw-Z	5.6	8.9	123	151	6	MOLECULE:	CENTROMERE-BINDING PROTEIN 1
61:00:00	5dfz-D	5.6	11.9	136	341	10	MOLECULE:	VACUOLAR PROTEIN SORTING-ASSOCIATED PROTEIN
62:00:00	5bw9-g	5.5	2.9	87	141	2	MOLECULE:	V-TYPE PROTON ATPASE CATALYTIC SUBUNIT A
63:00:00	8th8-A	5.5	5.3	133	290	5	MOLECULE:	DYNEIN REGULATORY COMPLEX PROTEIN 1/2 N-TERM
64:00:00	3fwc-F	5.5	2.6	84	84	5	MOLECULE:	CELL DIVISION CONTROL PROTEIN 31
65:00:00	5mqf-L	5.5	36.3	118	336	3	MOLECULE:	PRE-MRNA-PROCESSING-SPLICING FACTOR 8
66:00:00	7e9t-A	5.5	23	133	371	9	MOLECULE:	SPIKE PROTEIN S2
67:00:00	6f1t-X	5.5	8	154	285	8	MOLECULE:	ARP1 ACTIN RELATED PROTEIN 1 HOMOLOG A
68:00:00	8a5a-G	5.5	6.8	105	110	7	MOLECULE:	CHROMATIN-REMODELING ATPASE INO80
69:00:00	8ovw-U	5.4	13.3	129	184	7	MOLECULE:	CENTROMERE-BINDING PROTEIN 1
70:00:00	2xnx-M	5.4	4.7	107	107	7	MOLECULE:	FIBRINOGEN ALPHA CHAIN
71:00:00	6exn-D	5.4	8.9	96	97	9	MOLECULE:	U2 SNRNA
72:00:00	4h22-B	5.4	5.1	83	83	7	MOLECULE:	LEUCINE-RICH REPEAT FLIGHTLESS-INTERACTING P
73:00:00	8p0v-L	5.4	32.7	152	416	5	MOLECULE:	COILED-COIL DOMAIN-CONTAINING PROTEIN 93
74:00:00	7puz-A	5.3	3.6	102	148	8	MOLECULE:	MICOS COMPLEX SUBUNIT MIC60
75:00:00	8fwj-A	5.3	8.7	114	552	5	MOLECULE:	CIRCADIAN CLOCK PROTEIN KAIC
76:00:00	5cws-D	5.3	5.1	90	180	7	MOLECULE:	SAB-158 FAB LIGHT CHAIN
77:00:00	6j9r-B	5.3	4.2	102	126	10	MOLECULE:	BRAIN TUMOR PROTEIN
78:00:00	8q85-d	5.3	19.1	104	108	3	MOLECULE:	KINETOCHORE PROTEIN NDC80
79:00:00	5ijn-H	5.3	3.1	94	169	7	MOLECULE:	NUCLEAR PORE COMPLEX PROTEIN NUP155
80:00:00	5hda-A	5.2	7.9	97	121	7	MOLECULE:	ZINC FINGER MYND DOMAIN-CONTAINING PROTEIN 1
81:00:00	8fed-F	5.2	20.9	153	399	9	MOLECULE:	VIRULENCE FACTOR MCE FAMILY PROTEIN
82:00:00	8bd7-L	5.2	31.1	155	303	8	MOLECULE:	IFT88
83:00:00	4lin-A	5.2	9.3	149	289	3	MOLECULE:	TAIL NEEDLE PROTEIN GP26
84:00:00	4y66-C	5.2	20.5	128	197	9	MOLECULE:	MND1
85:00:00	5gmk-c	5.2	38.6	134	436	7	MOLECULE:	PRE-MRNA-SPLICING FACTOR 8
86:00:00	8qbv-A	5.2	11.7	129	217	11	MOLECULE:	MEMBRANE-ASSOCIATED PROTEIN VIPP1
87:00:00	9dtr-D	5.2	9.9	104	108	11	MOLECULE:	U2 SNRNA
88:00:00	6j6g-c	5.2	47.2	143	436	9	MOLECULE:	PRE-MRNA-SPLICING FACTOR 8
89:00:00	5lxn-A	5.1	4.1	81	81	10	MOLECULE:	TRANSFORMING ACIDIC COILED-COIL-CONTAINING P
90:00:00	3vp8-B	5.1	2.5	80	80	6	MOLECULE:	GENERAL TRANSCRIPTIONAL COREPRESSOR TUP1
91:00:00	8hpo-D	5.1	10.2	118	165	6	MOLECULE:	TRANSCRIPTIONAL REGULATORY PROTEIN UME1
92:00:00	8cqn-B	5	8.4	123	187	7	MOLECULE:	LIPOPROTEIN, PUTATIVE
93:00:00	2xqh-A	5	10.1	125	258	7	MOLECULE:	IMMUNOGLOBULIN-BINDING PROTEIN EIBD
94:00:00	2p22-A	5	13.5	123	168	6	MOLECULE:	SUPPRESSOR PROTEIN STP22 OF TEMPERATURE-SENS
95:00:00	4mvd-B	5	6.2	116	253	6	MOLECULE:	CHOLINE-PHOSPHATE CYTIDYLYLTRANSFERASE A
96:00:00	3jb9-i	5	46.6	125	161	7	MOLECULE:	PRE-MRNA-SPLICING FACTOR SPP42
97:00:00	6yjd-A	5	3.2	87	104	7	MOLECULE:	CAPSID ASSEMBLY SCAFFOLDING PROTEIN,PRELAMIN
98:00:00	7fde-H	5	4.8	96	112	6	MOLECULE:	V-TYPE PROTON ATPASE SUBUNIT C

99:00:00	8ppr-P	5	7.8	124	176	5	MOLECULE:	KINETOCHORE-ASSOCIATED PROTEIN DSN1 HOMOLOG
0:00	3jb9-W	5	33.9	135	426	7	MOLECULE:	PRE-MRNA-SPLICING FACTOR SPP42
1:00	8tek-A	5	9.2	131	200	6	MOLECULE:	DYNEIN REGULATORY COMPLEX PROTEIN 1/2 N-TERM
2:00	3v6i-A	4.9	6.4	115	186	9	MOLECULE:	V-TYPE ATP SYNTHASE SUBUNIT E
3:00	6znl-n	4.9	11.1	113	343	7	MOLECULE:	ARP1 ACTIN RELATED PROTEIN 1 HOMOLOG A
4:00	6cfz-I	4.9	12	105	106	5	MOLECULE:	ASK1
5:00	4aj5-2	4.9	11	99	100	4	MOLECULE:	SPINDLE AND KINETOCHORE-ASSOCIATED PROTEIN 1
6:00	1nyh-A	4.9	3.8	76	76	9	MOLECULE:	REGULATORY PROTEIN SIR4
7:00	2jee-A	4.9	3	78	78	6	MOLECULE:	CELL DIVISION PROTEIN ZAPB
8:00	3iv1-A	4.9	2.9	77	78	10	MOLECULE:	TUMOR SUSCEPTIBILITY GENE 101 PROTEIN
9:00	8tek-B	4.8	15.5	121	208	7	MOLECULE:	DYNEIN REGULATORY COMPLEX PROTEIN 1/2 N-TERM
10:00	6oei-A	4.8	2.1	97	243	10	MOLECULE:	SPINDLE POLE BODY COMPONENT SPC42,SIGMA-54-D
11:00	9na8-E	4.8	4.7	84	244	10	MOLECULE:	AUGMIN SUBUNIT 1
12:00	3vem-C	4.8	2.8	79	83	9	MOLECULE:	HELICASE PROTEIN MOM1
13:00	8ppr-D	4.8	11.2	138	248	7	MOLECULE:	KINETOCHORE-ASSOCIATED PROTEIN DSN1 HOMOLOG
14:00	8amr-A	4.7	7.7	116	155	9	MOLECULE:	TRIPARTITE MOTIF-CONTAINING PROTEIN 3
15:00	5nnv-D	4.7	7.6	125	252	6	MOLECULE:	CHROMOSOME PARTITION PROTEIN SMC,CHROMOSOME
16:00	3b5n-C	4.7	3.7	70	70	6	MOLECULE:	SYNAPTOBREVIN HOMOLOG 1
17:00	3trt-A	4.7	2.9	75	75	3	MOLECULE:	VIMENTIN
18:00	3thf-A	4.7	3.8	85	175	8	MOLECULE:	PROTEIN SHROOM
19:00	6id0-L	4.7	22.8	125	475	6	MOLECULE:	PRE-MRNA-PROCESSING-SPLICING FACTOR 8
20:00	5mq4-C	4.6	6.2	90	117	3	MOLECULE:	PROTEIN KINASE C-BINDING PROTEIN 1
21:00	7jtk-F	4.6	7.2	130	287	8	MOLECULE:	FLAGELLAR RADIAL SPOKE PROTEIN 1
22:00	4dhx-A	4.6	2.2	72	72	4	MOLECULE:	80 KDA MCM3-ASSOCIATED PROTEIN
23:00	7qoo-U	4.6	4.5	110	186	6	MOLECULE:	CENTROMERE PROTEIN C
24:00:00	6wcj-B	4.5	8.5	81	109	4	MOLECULE:	CLATHRIN HEAVY CHAIN 1
25:00:00	8q85-V	4.5	9.3	93	117	9	MOLECULE:	KINETOCHORE PROTEIN NDC80
26:00:00	2ba2-A	4.5	5.8	81	81	12	MOLECULE:	HYPOTHETICAL UPF0134 PROTEIN MPN010
27:00:00	8wjo-B	4.5	7.8	129	283	8	MOLECULE:	STRUCTURAL MAINTENANCE OF CHROMOSOMES PROTEI
28:00:00	8a5p-G	4.5	5.8	89	99	9	MOLECULE:	INO80 ATPASE
29:00:00	6yvv-A	4.5	56.8	107	465	6	MOLECULE:	STRUCTURAL MAINTENANCE OF CHROMOSOMES PROTEI
30:00:00	7emf-D	4.4	9.7	107	158	14	MOLECULE:	MEDIATOR OF RNA POLYMERASE II TRANSCRIPTION
31:00:00	8ppr-M	4.4	18.3	124	205	10	MOLECULE:	KINETOCHORE-ASSOCIATED PROTEIN DSN1 HOMOLOG
32:00:00	3n7n-B	4.4	6.2	92	164	4	MOLECULE:	MONOPOLIN COMPLEX SUBUNIT CSM1
33:00:00	7jts-s	4.4	18.4	113	290	5	MOLECULE:	RADIAL SPOKE PROTEIN 3
34:00:00	5dfz-A	4.4	21.3	125	343	8	MOLECULE:	VACUOLAR PROTEIN SORTING-ASSOCIATED PROTEIN
35:00:00	7uw5-A	4.4	36.1	105	723	8	MOLECULE:	MECHANOSENSITIVE CHANNEL MSCK
36:00:00	4wpx-B	4.4	7.9	78	81	12	MOLECULE:	CELL DIVISION CONTROL PROTEIN 31-LIKE PROTEI
37:00:00	5kiu-A	4.4	5.9	76	76	5	MOLECULE:	SELENOPROTEIN S
38:00:00	5omb-C	4.4	9.7	88	98	9	MOLECULE:	REPLICATION FACTOR A PROTEIN 1
39:00:00	3qh9-A	4.4	3.1	66	66	9	MOLECULE:	LIPRIN-BETA-2
40:00:00	7vhp-G	4.4	13.4	132	299	10	MOLECULE:	ATP-DEPENDENT ZINC METALLOPROTEASE FTSH
41:00:00	1gmj-A	4.4	3.1	65	65	5	MOLECULE:	ATPASE INHIBITOR
42:00:00	2wz7-B	4.4	4.2	67	67	6	MOLECULE:	UNCHARACTERIZED PROTEIN YBGF
43:00:00	5wwl-N	4.4	5.6	73	155	4	MOLECULE:	CENTROMERE PROTEIN MIS12
44:00:00	8hpo-H	4.4	5.1	126	233	11	MOLECULE:	TRANSCRIPTIONAL REGULATORY PROTEIN UME1
45:00:00	7woo-G	4.3	3.8	91	200	13	MOLECULE:	NUCLEOPORIN NIC96
46:00:00	4n78-E	4.3	2.9	66	67	5	MOLECULE:	CYTOPLASMIC FMR1-INTERACTING PROTEIN 1
47:00:00	5c9n-A	4.3	1.7	64	64	9	MOLECULE:	GEMININ COILED-COIL DOMAIN-CONTAINING PROTEI
48:00:00	8fed-A	4.3	21.3	137	392	4	MOLECULE:	VIRULENCE FACTOR MCE FAMILY PROTEIN

49:00:00	5ed9-B	4.2	2.7	72	72	10	MOLECULE:	SUN DOMAIN-CONTAINING PROTEIN 2
50:00:00	8ppr-F	4.2	4.2	71	112	4	MOLECULE:	KINETOCHORE-ASSOCIATED PROTEIN DSN1 HOMOLOG
51:00:00	8p0v-K	4.2	26.4	129	304	13	MOLECULE:	COILED-COIL DOMAIN-CONTAINING PROTEIN 93
52:00:00	5t58-B	4.2	11	112	190	9	MOLECULE:	KLLA0F02343P
53:00:00	3b5n-D	4.2	3.6	64	64	9	MOLECULE:	SYNAPTOBREVIN HOMOLOG 1
54:00:00	4bry-B	4.2	4.7	69	69	6	MOLECULE:	GEMININ
55:00:00	6cfz-C	4.2	3.5	70	79	4	MOLECULE:	ASK1
56:00:00	4c47-A	4.1	8.6	81	191	7	MOLECULE:	INNER MEMBRANE LIPOPROTEIN
57:00:00	4r8g-E	4.1	15.8	109	324	6	MOLECULE:	UNCONVENTIONAL MYOSIN-IC
58:00:00	9cgi-C	4.1	11.8	107	201	4	MOLECULE:	RNA-DIRECTED RNA POLYMERASE L
59:00:00	7sqk-C	4.1	8.7	136	600	4	MOLECULE:	HAUS AUGMIN-LIKE COMPLEX SUBUNIT 1
60:00:00	4r3z-A	4.1	2	71	76	3	MOLECULE:	AMINOACYL TRNA SYNTHASE COMPLEX-INTERACTING
61:00:00	8tek-M	4.1	23.8	96	164	8	MOLECULE:	DYNEIN REGULATORY COMPLEX PROTEIN 1/2 N-TERM
62:00:00	8fed-E	4.1	21.2	136	358	6	MOLECULE:	VIRULENCE FACTOR MCE FAMILY PROTEIN
63:00:00	1s1c-Y	4.1	3.7	70	70	7	MOLECULE:	TRANSFORMING PROTEIN RHOA
64:00:00	3m0d-A	4.1	2.3	63	63	11	MOLECULE:	TNF RECEPTOR-ASSOCIATED FACTOR 2
65:00:00	3m0d-C	4.1	3.7	63	63	11	MOLECULE:	TNF RECEPTOR-ASSOCIATED FACTOR 2
66:00:00	3lt6-F	4.1	4.1	63	63	2	MOLECULE:	ADHESIN YADA
67:00:00	3zdo-F	4.1	3.7	65	65	9	MOLECULE:	PHOSPHOPROTEIN
68:00:00	5wlz-C	4.1	14.4	93	206	8	MOLECULE:	DNA REPAIR PROTEIN XRCC4,MYOSIN-7
69:00:00	5mq0-t	4.1	12.2	104	438	5	MOLECULE:	YEAST UBC4 GENE FOR UBIQUITIN-CONJUGATING EN
70:00:00	5dol-B	4.1	3.1	62	62	5	MOLECULE:	INITIATION-CONTROL PROTEIN YABA
71:00:00	8p1u-C	4.1	4.2	77	93	8	MOLECULE:	CELL DIVISION PROTEIN FTSL
72:00:00	9io5-G	4	5.2	89	260	6	MOLECULE:	G1-ATPASE SUBUNIT BETA
73:00:00	8q85-b	4	3.9	69	70	7	MOLECULE:	KINETOCHORE PROTEIN NDC80
74:00:00	8aud-A	4	3.8	94	350	10	MOLECULE:	CELL WALL-ASSOCIATED HYDROLASES (INVASION-AS
75:00:00	4orh-H	4	3	77	142	8	MOLECULE:	UBIQUITIN-CONJUGATING ENZYME E2 VARIANT 2
76:00:00	6wuc-K	4	23.1	115	235	4	MOLECULE:	INNER KINETOCHORE SUBUNIT MCM16
77:00:00	8srm-B	4	11.4	89	235	9	MOLECULE:	RB1-INDUCIBLE COILED-COIL PROTEIN 1
78:00:00	9d80-C	4	11.4	121	275	7	MOLECULE:	PORTAL PROTEIN
79:00:00	1m1j-B	4	13.6	119	402	4	MOLECULE:	FIBRINOGEN ALPHA SUBUNIT
80:00:00	2zxx-B	4	6.1	71	78	7	MOLECULE:	GEMININ
81:00:00	1gd2-E	4	3	65	65	6	MOLECULE:	DNA (5'-
82:00:00	1h88-B	4	5.8	70	71	13	MOLECULE:	CCAAT/ENHANCER BINDING PROTEIN BETA
83:00:00	3hrn-A	4	2.9	63	63	10	MOLECULE:	TRANSIENT RECEPTOR POTENTIAL (TRP) CHANNEL
84:00:00	5chx-A	4	13	110	210	6	MOLECULE:	XRCC4-MYH7-1590-1657
85:00:00	1aa0-A	4	4.4	84	113	5	MOLECULE:	FIBRITIN
86:00:00	6ian-A	3.9	34.7	100	306	4	MOLECULE:	INTRAFLAGELLAR TRANSPORT PROTEIN 74
87:00:00	8g0q-B	3.9	19.5	99	230	7	MOLECULE:	KINETOCHORE PROTEIN NDC80
88:00:00	5oqm-h	3.9	9.2	104	131	7	MOLECULE:	DNA-DIRECTED RNA POLYMERASE II SUBUNIT RPB1
89:00:00	3p8c-D	3.9	31.5	92	204	8	MOLECULE:	CYTOPLASMIC FMR1-INTERACTING PROTEIN 1
90:00:00	7eeb-C	3.9	20.5	88	278	6	MOLECULE:	ENHANCED GREEN FLUORESCENT PROTEIN,CATION CH
91:00:00	8q85-X	3.9	7.6	68	68	4	MOLECULE:	KINETOCHORE PROTEIN NDC80
92:00:00	4cjd-A	3.9	17.4	80	123	8	MOLECULE:	NADA
93:00:00	6kn7-T	3.9	14.7	67	138	6	MOLECULE:	ACTIN, ALPHA SKELETAL MUSCLE
94:00:00	5n9j-D	3.9	16.9	97	135	5	MOLECULE:	MEDIATOR OF RNA POLYMERASE II TRANSCRIPTION
95:00:00	6gmh-Q	3.9	18.8	116	884	9	MOLECULE:	RPB1
96:00:00	7x2e-A	3.9	9.4	97	163	8	MOLECULE:	HARMONIN
97:00:00	1z56-A	3.9	2	75	77	8	MOLECULE:	LIGASE INTERACTING FACTOR 1
98:00:00	3wmi-A	3.9	2.3	57	57	2	MOLECULE:	EIAV GP45 WILD TYPE

99:00:00	7sqk-F	3.9	28.5	128	385	5	MOLECULE:	HAUS AUGMIN-LIKE COMPLEX SUBUNIT 1
0:00	4ke2-A	3.9	3.1	94	196	4	MOLECULE:	TYPE I HYPERACTIVE ANTIFREEZE PROTEIN
1:00	7nad-5	3.9	16.1	73	123	12	MOLECULE:	25S RRNA
2:00	4njl-A	3.9	3.9	81	129	5	MOLECULE:	S PROTEIN
3:00	3b5n-A	3.9	2.4	60	61	5	MOLECULE:	SYNAPTOBREVIN HOMOLOG 1
4:00	5opt-P	3.9	9.4	98	249	8	MOLECULE:	ACTIVATED PROTEIN KINASE C RECEPTOR, PUTATIV
5:00	4om3-D	3.9	3.4	80	122	11	MOLECULE:	TRANSDUCIN-LIKE ENHANCER PROTEIN 1
6:00	2p2u-B	3.8	22.6	80	153	5	MOLECULE:	HOST-NUCLEASE INHIBITOR PROTEIN GAM, PUTATIV
7:00	2p22-C	3.8	8.1	108	186	7	MOLECULE:	SUPPRESSOR PROTEIN STP22 OF TEMPERATURE-SENS
8:00	8u95-A	3.8	8.9	154	1009	7	MOLECULE:	MYOSIN HEAVY CHAIN, ISOFORM U
9:00	6kn7-U	3.8	8.5	107	170	7	MOLECULE:	ACTIN, ALPHA SKELETAL MUSCLE
10:00	6gvw-F	3.8	32.5	110	320	5	MOLECULE:	BRCA1-A COMPLEX SUBUNIT ABRAXAS 1
11:00	5c1f-A	3.8	5.8	123	298	7	MOLECULE:	SEPTATION PROTEIN IMP2
12:00	6qld-Y	3.8	14	130	223	10	MOLECULE:	INNER KINETOCHORE SUBUNIT MIF2
13:00	8qyd-A	3.8	7.5	113	280	12	MOLECULE:	ANTI-PHAGE DEFENSE ZORAB SYSTEM ZORA
14:00	8d8k-V	3.8	17.7	119	233	10	MOLECULE:	PROBABLE S-ADENOSYL-L-METHIONINE-DEPENDENT R
15:00	8wge-A	3.8	1.4	57	348	2	MOLECULE:	ZAC
16:00	7zr1-D	3.8	14.3	133	780	7	MOLECULE:	DOUBLE-STRAND BREAK REPAIR PROTEIN
17:00	4i1l-A	3.8	2.3	62	62	8	MOLECULE:	FORKHEAD BOX PROTEIN P3
18:00	4aqr-D	3.8	2.4	57	57	4	MOLECULE:	CALMODULIN-7
19:00	4bwd-A	3.8	3.7	61	61	8	MOLECULE:	SHORT COILED-COIL PROTEIN
20:00	1joc-A	3.8	6.1	74	123	7	MOLECULE:	EARLY ENDOSOMAL AUTOANTIGEN 1
21:00	9cpo-B	3.7	10.8	95	195	8	MOLECULE:	RNA-DIRECTED RNA POLYMERASE NSP12
22:00	5y06-A	3.7	12.8	101	235	10	MOLECULE:	MSMEG_4306
23:00	4wpe-A	3.7	5.9	112	275	7	MOLECULE:	CYTOKINESIS PROTEIN 2
24:00:00	1urq-A	3.7	3.4	57	57	5	MOLECULE:	M-TOMOSYN ISOFORM
25:00:00	7aw7-D	3.7	18.6	74	93	4	MOLECULE:	HAPB
26:00:00	7kdf-A	3.7	10	114	270	4	MOLECULE:	NDC80 ISOFORM 1,NDC80 ISOFORM 1
27:00:00	8q3v-g	3.6	23.6	76	76	9	MOLECULE:	TETRAHYDROMETHANOPTERIN S-METHYLTRANSFERASE
28:00:00	5odw-D	3.6	2.5	66	198	8	MOLECULE:	FERRIPYOVERDINE RECEPTOR
29:00:00	2efl-A	3.6	4.7	121	281	3	MOLECULE:	FORMIN-BINDING PROTEIN 1
30:00:00	3cl3-D	3.6	2.2	59	59	0	MOLECULE:	ORF K13
31:00:00	3e7k-H	3.6	3.1	56	56	7	MOLECULE:	TRPM7 CHANNEL
32:00:00	4jol-A	3.6	3.7	60	60	8	MOLECULE:	PROTEIN CBFA2T1
33:00:00	7qog-A	3.5	3.8	91	657	8	MOLECULE:	PORTAL PROTEIN GP20
34:00:00	9f63-A	3.5	35.3	87	422	7	MOLECULE:	PROTEIN PNS1
35:00:00	5yfp-C	3.5	11.8	101	790	9	MOLECULE:	EXOCYST COMPLEX COMPONENT SEC3
36:00:00	2v0o-A	3.5	6.7	115	273	7	MOLECULE:	FCH DOMAIN ONLY PROTEIN 2
37:00:00	7w5a-X	3.5	8.4	77	87	3	MOLECULE:	PRE-MRNA-PROCESSING-SPLICING FACTOR 8
38:00:00	8v2q-A	3.5	15	103	184	6	MOLECULE:	CHARGED MULTIVESICULAR BODY PROTEIN 1B
39:00:00	7x5e-B	3.5	5.6	71	106	8	MOLECULE:	TRANSCRIPTION FACTOR MAFG
40:00:00	5mg8-A	3.5	20.8	85	282	8	MOLECULE:	STRUCTURAL MAINTENANCE OF CHROMOSOMES PROTEI
41:00:00	2x7r-C	3.5	2.8	58	58	3	MOLECULE:	TRANSMEMBRANE PROTEIN GP41
42:00:00	7sgs-A	3.5	1.2	53	53	8	MOLECULE:	TUBULIN ALPHA-1B CHAIN
43:00:00	5oqm-n	3.4	14.3	94	136	4	MOLECULE:	DNA-DIRECTED RNA POLYMERASE II SUBUNIT RPB1
44:00:00	5n9j-U	3.4	25.3	104	198	5	MOLECULE:	MEDIATOR OF RNA POLYMERASE II TRANSCRIPTION
45:00:00	8fck-F	3.4	14.8	122	393	8	MOLECULE:	HAUS AUGMIN-LIKE COMPLEX SUBUNIT 1
46:00:00	3p8c-F	3.4	10.4	106	156	7	MOLECULE:	CYTOPLASMIC FMR1-INTERACTING PROTEIN 1
47:00:00	7emf-G	3.4	3.8	79	161	3	MOLECULE:	MEDIATOR OF RNA POLYMERASE II TRANSCRIPTION
48:00:00	3opc-B	3.4	2.2	70	131	4	MOLECULE:	UNCHARACTERIZED PROTEIN

49:00:00	8afz-B	3.4	2.7	74	376	9	MOLECULE:	SORTING NEXIN-1
50:00:00	3eff-K	3.4	3.9	82	139	12	MOLECULE:	FAB
51:00:00	7emf-K	3.4	14.4	81	112	5	MOLECULE:	MEDIATOR OF RNA POLYMERASE II TRANSCRIPTION
52:00:00	6b85-A	3.4	10.3	78	215	10	MOLECULE:	TMHC4_R
53:00:00	3a5c-G	3.4	2.3	73	129	7	MOLECULE:	V-TYPE ATP SYNTHASE ALPHA CHAIN
54:00:00	6z6o-C	3.3	8.6	101	548	10	MOLECULE:	HISTONE DEACETYLASE HDA1
55:00:00	8qfc-D	3.3	13.1	98	185	10	MOLECULE:	60S RIBOSOMAL PROTEIN L10A
56:00:00	9c57-G	3.3	43.1	125	878	6	MOLECULE:	RUVB-LIKE 1
57:00:00	5jeq-A	3.3	4.8	86	227	7	MOLECULE:	NITRATE/NITRITE SENSOR PROTEIN NARQ
58:00:00	9na9-A	3.3	7.5	71	103	17	MOLECULE:	AUGMIN SUBUNIT 1
59:00:00	7vc4-C	3.3	16.5	85	107	9	MOLECULE:	MITOCHONDRIAL IMPORT RECEPTOR SUBUNIT TOM6 H
60:00:00	4tt0-B	3.3	4.6	84	131	7	MOLECULE:	DENEDDYLASE
61:00:00	7x6g-D	3.3	7.8	81	152	9	MOLECULE:	QUORUM-SENSING REGULATOR PROTEIN G
62:00:00	6ikn-A	3.3	5.5	115	305	10	MOLECULE:	GROWTH ARREST-SPECIFIC PROTEIN 7
63:00:00	7m2w-K	3.3	16.2	87	95	8	MOLECULE:	TUBULIN GAMMA CHAIN
64:00:00	5oxf-A	3.3	12.4	120	703	7	MOLECULE:	GTP-BINDING PROTEIN
65:00:00	4p1m-B	3.3	7.7	65	107	12	MOLECULE:	CELL DIVISION PROTEIN ZAPA
66:00:00	7f3t-A	3.3	4	70	330	13	MOLECULE:	TRANSMEMBRANE PROTEIN 120A
67:00:00	8q85-Z	3.3	10.8	83	94	8	MOLECULE:	KINETOCHORE PROTEIN NDC80
68:00:00	7sc0-A	3.2	24.4	101	129	5	MOLECULE:	CAVEOLIN-1
69:00:00	8hhf-B	3.2	3.8	67	89	9	MOLECULE:	CELL DIVISION PROTEIN FTSQ
70:00:00	7vdv-A	3.2	16	113	809	6	MOLECULE:	HISTONE H4
71:00:00	5ew5-A	3.2	23.7	109	490	5	MOLECULE:	COLICIN-E9
72:00:00	7v2w-J	3.2	15.2	115	236	5	MOLECULE:	THO COMPLEX SUBUNIT HPR1
73:00:00	7nvr-n	3.2	12.4	91	132	7	MOLECULE:	TFIIH BASAL TRANSCRIPTION FACTOR COMPLEX HEL
74:00:00	4h8s-C	3.2	11.1	116	382	5	MOLECULE:	DCC-INTERACTING PROTEIN 13-BETA
75:00:00	4jio-B	3.2	5.7	59	320	10	MOLECULE:	BRO1
76:00:00	1kmi-Z	3.2	11.3	99	177	5	MOLECULE:	CHEMOTAXIS PROTEIN CHEY
77:00:00	7wkk-I	3.2	3.6	88	171	10	MOLECULE:	MGC83295 PROTEIN
78:00:00	7mge-B	3.2	10.1	68	442	15	MOLECULE:	WD REPEAT-CONTAINING PROTEIN 41
79:00:00	5yfp-F	3.2	16.1	121	725	10	MOLECULE:	EXOCYST COMPLEX COMPONENT SEC3
80:00:00	6em5-t	3.2	15.5	78	290	3	MOLECULE:	5.8S RIBOSOMAL RNA
81:00:00	2d1l-A	3.2	40.5	105	249	3	MOLECULE:	METASTASIS SUPPRESSOR PROTEIN 1
82:00:00	4nad-A	3.2	6.1	92	133	4	MOLECULE:	REGULATION OF NUCLEAR PRE-MRNA DOMAIN-CONTAI
83:00:00	5wkq-A	3.1	3.2	83	166	8	MOLECULE:	INVASIN IPAB
84:00:00	4nqi-D	3.1	4.1	97	232	6	MOLECULE:	SH3 DOMAIN-CONTAINING PROTEIN
85:00:00	1ezj-A	3.1	7.3	71	114	7	MOLECULE:	NUCLEOCAPSID PHOSPHOPROTEIN
86:00:00	8tek-E	3.1	6	111	219	5	MOLECULE:	DYNEIN REGULATORY COMPLEX PROTEIN 1/2 N-TERM
87:00:00	6r7l-E	3.1	22	78	98	8	MOLECULE:	SECG
88:00:00	3hgf-A	3.1	4.6	69	98	3	MOLECULE:	RHOPTRY PROTEIN FRAGMENT
89:00:00	7v2w-I	3.1	18.1	123	239	9	MOLECULE:	THO COMPLEX SUBUNIT HPR1
90:00:00	7d7n-A	3.1	18.8	89	704	4	MOLECULE:	ATP-BINDING CASSETTE SUB-FAMILY B MEMBER 6,
91:00:00	6c0f-8	3.1	8.9	71	98	8	MOLECULE:	SACCHAROMYCES CEREVISIAE S288C 35S PRE-RIBOS
92:00:00	4ilo-A	3.1	9.5	95	236	4	MOLECULE:	CT398
93:00:00	6gmh-M	3.1	49.1	108	991	6	MOLECULE:	RPB1
94:00:00	8vvc-A	3	43.4	100	1390	8	MOLECULE:	MULTIDRUG RESISTANCE-ASSOCIATED PROTEIN 1
95:00:00	4m8m-B	3	16.5	85	574	6	MOLECULE:	GCN4 COILED-COIL FUSED ZEBRAFISH PLEXINC1
96:00:00	6oee-A	3	54.7	104	243	3	MOLECULE:	TYPE IV SECRETION SYSTEM APPARATUS PROTEIN C
97:00:00	4tko-B	3	11.6	111	326	11	MOLECULE:	EMRA
98:00:00	4mu6-A	3	5.5	99	274	9	MOLECULE:	KINECTIN 1 (KINESIN RECEPTOR)

99:00:00	7zmg-n	3	12.6	94	136	11	MOLECULE:	NADH-UBIQUINONE OXIDOREDUCTASE CHAIN 1
0:00	8x5f-A	3	36.1	94	616	6	MOLECULE:	SOLUTE CARRIER FAMILY 53 MEMBER 1
1:00	4cq4-A	3	3.7	77	106	6	MOLECULE:	ENGINEERED VERSION OF TRANSMEMBRANE RECEPTOR
2:00	6rd4-5	3	5.2	65	123	3	MOLECULE:	ASA-10: POLYTOMELLA F-ATP SYNTHASE ASSOCIATE
3:00	8xks-M	3	14.9	93	135	1	MOLECULE:	CTAP1
4:00	9eri-G	3	4.5	66	207	15	MOLECULE:	NA(+)-TRANSLOCATING FERREDOXIN:NAD(+) OXIDOR
5:00	1y2o-A	3	5.3	99	248	7	MOLECULE:	BAI1-ASSOCIATED PROTEIN 2 ISOFORM 1
6:00	7oi8-q	3	16.7	108	164	8	MOLECULE:	39S RIBOSOMAL PROTEIN L2, MITOCHONDRIAL
7:00	8fef-D	3	17.9	144	385	8	MOLECULE:	VIRULENCE FACTOR MCE FAMILY PROTEIN
8:00	2fo1-D	3	4.1	59	63	3	MOLECULE:	5'-
9:00	8cpz-B	3	10.1	158	2122	6	MOLECULE:	TCDA1
10:00	5vo5-A	3	2.9	76	179	4	MOLECULE:	COILED-COIL AND C2 DOMAIN-CONTAINING PROTEIN
11:00	8xva-K	3	38.6	81	145	10	MOLECULE:	MITOCHONDRIAL IMPORT RECEPTOR SUBUNIT TOM6 H
12:00	7egm-H	3	18.2	122	388	9	MOLECULE:	TRANSCRIPTION REGULATORY PROTEIN SNF2
13:00	4dyl-A	3	4.6	117	376	7	MOLECULE:	TYROSINE-PROTEIN KINASE FES/FPS
14:00	5n9j-E	2.9	11.9	97	201	7	MOLECULE:	MEDIATOR OF RNA POLYMERASE II TRANSCRIPTION
15:00	5mpd-U	2.9	29.3	102	298	6	MOLECULE:	26S PROTEASOME REGULATORY SUBUNIT RPN10
16:00	9na8-D	2.9	8.5	102	269	11	MOLECULE:	AUGMIN SUBUNIT 1
17:00	5nen-A	2.9	4.5	95	266	3	MOLECULE:	LIPASE C
18:00	6tdv-M	2.9	19.2	87	166	8	MOLECULE:	ATPTB1
19:00	3viq-A	2.9	16.6	75	122	11	MOLECULE:	SWI5-DEPENDENT RECOMBINATION DNA REPAIR PROT
20:00	5c21-A	2.9	3.9	94	267	5	MOLECULE:	CHROMOSOMAL HEMOLYSIN D
21:00	8hqo-x	2.9	7	77	80	4	MOLECULE:	PORTAL PROTEIN
22:00	6sl2-A	2.9	5.8	126	619	10	MOLECULE:	CALPONIN HOMOLOGY DOMAIN PROTEIN PUTATIVE
23:00	1jad-A	2.9	3.7	89	242	8	MOLECULE:	PHOSPHOLIPASE C BETA
24:00:00	6wuc-H	2.9	11.7	77	179	10	MOLECULE:	INNER KINETOCHORE SUBUNIT MCM16
25:00:00	7jtk-C	2.9	10.7	125	427	2	MOLECULE:	FLAGELLAR RADIAL SPOKE PROTEIN 1
26:00:00	8f7n-A	2.9	8.6	110	364	9	MOLECULE:	METHYL-ACCEPTING CHEMOTAXIS PROTEIN
27:00:00	3na7-A	2.9	13.2	99	237	8	MOLECULE:	HP0958
28:00:00	1lj2-B	2.8	7.3	66	110	6	MOLECULE:	NONSTRUCTURAL RNA-BINDING PROTEIN 34
29:00:00	7c4j-F	2.8	20.3	108	383	7	MOLECULE:	TRANSCRIPTION REGULATORY PROTEIN SNF12
30:00:00	6ted-R	2.8	11.7	77	244	5	MOLECULE:	DNA-DIRECTED RNA POLYMERASE SUBUNIT
31:00:00	8qby-L	2.8	32.3	108	660	6	MOLECULE:	NADH-QUINONE OXIDOREDUCTASE SUBUNIT K
32:00:00	7uus-Q	2.8	15.3	90	170	7	MOLECULE:	HYDROGENASE-2, LARGE SUBUNIT
33:00:00	8i3j-A	2.8	5	99	277	8	MOLECULE:	DYNEIN AXONEMAL HEAVY CHAIN 1
34:00:00	3wqa-A	2.8	7.3	106	201	4	MOLECULE:	TRIMERIC AUTOTRANSPORTER ADHESIN
35:00:00	8iw0-A	2.8	2.9	61	84	11	MOLECULE:	LIPRIN-BETA-1,KN MOTIF AND ANKYRIN REPEAT DO
36:00:00	3qwe-A	2.8	5.4	93	260	2	MOLECULE:	GEM-INTERACTING PROTEIN
37:00:00	8i4t-R	2.8	11.9	97	501	4	MOLECULE:	ENVELOPMENT POLYPROTEIN
38:00:00	7ty0-C	2.7	3.2	60	488	0	MOLECULE:	GLYCOPROTEIN G
39:00:00	7jw1-e	2.7	9.1	101	235	6	MOLECULE:	CAPSID PROTEINS
40:00:00	3lvg-D	2.7	4.1	88	180	3	MOLECULE:	CLATHRIN HEAVY CHAIN 1
41:00:00	6hgc-A	2.7	7.6	63	304	8	MOLECULE:	UBIQUITIN CARBOXYL-TERMINAL HYDROLASE CALYPS
42:00:00	6rw8-B	2.7	51.7	122	2337	11	MOLECULE:	A COMPONENT OF INSECTICIDAL TOXIN COMPLEX (T
43:00:00	9f5y-p	2.7	8.6	90	129	6	MOLECULE:	NADH:UBIQUINONE OXIDOREDUCTASE 24 KD SUBUNIT
44:00:00	7w9l-A	2.7	48.8	80	1422	4	MOLECULE:	SODIUM CHANNEL PROTEIN TYPE 9 SUBUNIT ALPHA
45:00:00	6tda-L	2.7	15.6	79	596	8	MOLECULE:	HISTONE H3.2
46:00:00	6tpi-A	2.7	6.7	103	380	4	MOLECULE:	MUREIN HYDROLASE ACTIVATOR ENVC
47:00:00	2ve7-D	2.7	15.1	103	242	6	MOLECULE:	KINETOCHORE PROTEIN HEC1, KINETOCHORE PROTEI
48:00:00	8ap7-c	2.7	4.2	59	64	7	MOLECULE:	ATP SYNTHASE SUBUNIT A

49:00:00	5lm2-B	2.7	14.1	112	340	4	MOLECULE:	TYROSINE-PROTEIN PHOSPHATASE NON-RECEPTOR TY
50:00:00	5lc5-Z	2.7	16.1	96	138	4	MOLECULE:	NADH-UBIQUINONE OXIDOREDUCTASE CHAIN 3
51:00:00	8ebw-H	2.7	26.1	94	532	12	MOLECULE:	TFIIH BASAL TRANSCRIPTION FACTOR COMPLEX HEL
52:00:00	6djl-E	2.7	27.7	99	226	8	MOLECULE:	RAS-RELATED PROTEIN RAB-11A
53:00:00	9na8-C	2.7	6.1	106	234	6	MOLECULE:	AUGMIN SUBUNIT 1
54:00:00	5knc-G	2.7	4.1	83	195	6	MOLECULE:	V-TYPE SODIUM ATPASE CATALYTIC SUBUNIT A
55:00:00	3w3a-G	2.7	2.9	93	210	6	MOLECULE:	V-TYPE ATP SYNTHASE ALPHA CHAIN
56:00:00	6r1j-J	2.6	4.2	90	264	12	MOLECULE:	UNCHARACTERIZED PROTEIN
57:00:00	8at3-A	2.6	42.2	68	286	12	MOLECULE:	HAUS AUGMIN-LIKE COMPLEX SUBUNIT 1
58:00:00	6ian-C	2.6	34.2	86	432	5	MOLECULE:	INTRAFLAGELLAR TRANSPORT PROTEIN 74
59:00:00	4u6u-D	2.6	9.7	84	283	13	MOLECULE:	COG7
60:00:00	8av6-H	2.6	8.2	76	98	11	MOLECULE:	RUVB-LIKE HELICASE
61:00:00	4abx-A	2.6	6.3	82	167	10	MOLECULE:	DNA REPAIR PROTEIN RECN
62:00:00	6xky-A	2.6	6.1	101	271	7	MOLECULE:	FLAGELLIN
63:00:00	7bqx-G	2.6	22.5	78	84	8	MOLECULE:	TRIPLEX CAPSID PROTEIN 1
64:00:00	5t58-A	2.6	23.3	113	233	9	MOLECULE:	KLLA0F02343P
65:00:00	8y7m-I	2.6	3.8	74	117	4	MOLECULE:	POLYMERASE ACIDIC PROTEIN
66:00:00	8hpo-C	2.6	14.2	83	138	5	MOLECULE:	TRANSCRIPTIONAL REGULATORY PROTEIN UME1
67:00:00	8hmy-B	2.6	14.5	77	264	14	MOLECULE:	TRNA-SPLICING ENDONUCLEASE SUBUNIT SEN2
68:00:00	8xku-A	2.6	37.3	81	730	11	MOLECULE:	PROBABLE INACTIVE ATP-DEPENDENT ZINC METALLO
69:00:00	5yfp-A	2.6	48.6	111	672	6	MOLECULE:	EXOCYST COMPLEX COMPONENT SEC3
70:00:00	4cgk-A	2.6	5	84	351	10	MOLECULE:	SECRETED 45 KDA PROTEIN
71:00:00	8fck-E	2.6	24	89	222	11	MOLECULE:	HAUS AUGMIN-LIKE COMPLEX SUBUNIT 1
72:00:00	7nyx-A	2.6	10.9	118	1467	8	MOLECULE:	CHROMOSOME PARTITION PROTEIN MUKB
73:00:00	9gk2-G	2.6	13.6	102	432	4	MOLECULE:	PS2
74:00:00	7zqy-B	2.5	5.3	80	177	15	MOLECULE:	DH DOMAIN-CONTAINING PROTEIN
75:00:00	3vou-A	2.5	5.2	68	139	7	MOLECULE:	ION TRANSPORT 2 DOMAIN PROTEIN, VOLTAGE-GATE
76:00:00	5td8-C	2.5	9.8	74	111	9	MOLECULE:	KINETOCHORE PROTEIN NDC80
77:00:00	8cr1-C	2.5	7.1	73	266	7	MOLECULE:	ATPASE ASNA1
78:00:00	4tql-B	2.5	3	82	235	11	MOLECULE:	THREE HELIX BUNDLE
79:00:00	1y9b-A	2.5	5.9	58	81	7	MOLECULE:	CONSERVED HYPOTHETICAL PROTEIN
80:00:00	8tmj-D	2.5	12.8	95	352	4	MOLECULE:	SAB C18 HEAVY CHAIN
81:00:00	6bpz-A	2.5	38.7	113	900	8	MOLECULE:	PIEZO-TYPE MECHANOSENSITIVE ION CHANNEL COMP
82:00:00	6ye4-A	2.5	3.7	83	236	4	MOLECULE:	BIOPOLYMER TRANSPORT PROTEIN EXBB
83:00:00	7qoo-Q	2.5	21	96	209	10	MOLECULE:	CENTROMERE PROTEIN C
84:00:00	7woo-K	2.5	11.2	95	254	6	MOLECULE:	NUCLEOPORIN NIC96
85:00:00	8tkp-A	2.5	35.2	84	614	6	MOLECULE:	TRANSMEMBRANE CHANNEL-LIKE PROTEIN 2
86:00:00	1few-A	2.5	4.3	75	173	9	MOLECULE:	SECOND MITOCHONDRIA-DERIVED ACTIVATOR OF CAS
87:00:00	8rhn-A	2.5	4.1	64	198	9	MOLECULE:	ATPASE FAMILY GENE 2 PROTEIN HOMOLOG A
88:00:00	5wjt-A	2.5	5.3	101	302	9	MOLECULE:	FLAGELLIN
89:00:00	6sp2-A	2.5	11.3	57	366	4	MOLECULE:	MEMBRANE PROTEIN TMS1D
90:00:00	7oqe-K	2.5	16.9	103	406	6	MOLECULE:	PROTEIN NAM8
91:00:00	7sn7-a	2.5	4.2	101	546	9	MOLECULE:	FLAGELLIN
92:00:00	8qfc-C	2.5	12.4	118	380	9	MOLECULE:	60S RIBOSOMAL PROTEIN L10A
93:00:00	8ceg-B	2.5	2.9	79	214	4	MOLECULE:	CBY1-INTERACTING BAR DOMAIN-CONTAINING PROTE
94:00:00	7n5d-A	2.5	48.4	110	386	5	MOLECULE:	MECHANOSENSITIVE ION CHANNEL FLYCATCHER1
95:00:00	3r6n-A	2.5	5.9	124	450	6	MOLECULE:	DESMOPLAKIN
96:00:00	5sva-V	2.5	8.6	62	85	5	MOLECULE:	DNA-DIRECTED RNA POLYMERASE II SUBUNIT RPB1
97:00:00	8wjn-A	2.4	4.2	92	491	11	MOLECULE:	STRUCTURAL MAINTENANCE OF CHROMOSOMES PROTEI
98:00:00	5gox-A	2.4	44.1	82	181	9	MOLECULE:	DNA REPAIR PROTEIN RAD50

99:00:00	6xp5-D	2.4	9.6	98	134	8	MOLECULE:	MEDIATOR OF RNA POLYMERASE II TRANSCRIPTION
0:00	7tch-A	2.4	11.3	78	645	4	MOLECULE:	BACITRACIN EXPORT PERMEASE PROTEIN BCEB
1:00	8b9z-m	2.4	18.8	82	108	10	MOLECULE:	NADH-UBIQUINONE OXIDOREDUCTASE CHAIN 3
2:00	5j1i-A	2.4	6.7	102	346	8	MOLECULE:	PLECTIN
3:00	4gcz-B	2.4	7.5	78	378	5	MOLECULE:	BLUE-LIGHT PHOTORECEPTOR, SENSOR PROTEIN FIX
4:00	4he8-D	2.4	59.2	86	160	7	MOLECULE:	NADH-QUINONE OXIDOREDUCTASE SUBUNIT 7
5:00	8ixk-E	2.4	3.4	62	109	5	MOLECULE:	ATTACHMENT PROTEIN G3P
6:00	6gy6-A	2.4	6.7	102	365	10	MOLECULE:	XAXA
7:00	5j6f-A	2.4	13.9	58	352	9	MOLECULE:	3-DEOXY-D-ARABINO-HEPTULOSONATE 7-PHOSPHATE
8:00	8e1m-C	2.4	17.4	66	264	11	MOLECULE:	MITOCHONDRIAL IMPORT INNER MEMBRANE TRANSLOC
9:00	7ctp-A	2.4	43.4	123	553	6	MOLECULE:	PROTEIN NIBAN 2
10:00	7uh4-A	2.4	3.8	63	110	6	MOLECULE:	LXG-ASSOCIATED ALPHA-HELICAL PROTEIN D2
11:00	8ymk-A	2.4	5.3	50	226	14	MOLECULE:	ISOFORM S OF LARGE ENVELOPE PROTEIN
12:00	6c5w-A	2.4	5.2	79	285	9	MOLECULE:	CALCIUM UNIPORTER
13:00	8qx8-A	2.4	21.5	106	969	4	MOLECULE:	VACUOLAR PROTEIN SORTING-ASSOCIATED PROTEIN
14:00	5lc5-h	2.4	20.7	92	134	1	MOLECULE:	NADH-UBIQUINONE OXIDOREDUCTASE CHAIN 3
15:00	4o8w-D	2.4	11.6	90	120	6	MOLECULE:	SPORE GERMINATION PROTEIN
16:00	2pjw-H	2.4	6.8	64	88	6	MOLECULE:	UNCHARACTERIZED PROTEIN YHL002W
17:00	6j6q-F	2.4	13.4	77	161	8	MOLECULE:	PRE-MRNA-SPLICING FACTOR 8
18:00	8rt9-A	2.4	10	97	207	11	MOLECULE:	TRWJ PROTEIN
19:00	6zyy-C	2.4	38.2	106	2901	9	MOLECULE:	DYNEIN HEAVY CHAIN, OUTER ARM PROTEIN
20:00	8k9c-A	2.4	6.9	82	207	5	MOLECULE:	CAPRIN-2
21:00	7c4j-C	2.4	20.2	83	154	12	MOLECULE:	TRANSCRIPTION REGULATORY PROTEIN SNF12
22:00	6tmi-B	2.4	6.9	88	246	5	MOLECULE:	ATP SYNTHASE SUBUNIT ALPHA
23:00	7zr2-A	2.4	6.2	84	227	8	MOLECULE:	SPIKE PROTEIN S2',CHIMERIC PROTEIN MIMIC OF
24:00:00	4wpc-B	2.4	8.6	114	288	11	MOLECULE:	RHO GTPASE-ACTIVATING PROTEIN RGD1
25:00:00	3on0-A	2.3	12.6	67	121	7	MOLECULE:	PROTEIN TRAM
26:00:00	5xe3-E	2.3	11.1	57	81	5	MOLECULE:	ENDORIBONUCLEASE MAZF4
27:00:00	8h36-G	2.3	63.9	96	379	5	MOLECULE:	E3 UBIQUITIN-PROTEIN LIGASE RBX1
28:00:00	6zbj-A	2.3	33.7	119	666	4	MOLECULE:	PRECURSOR OF THE MAJOR MEROZOITE SURFACE ANT
29:00:00	6cfw-I	2.3	36.3	93	114	8	MOLECULE:	MONOVALENT CATION/H+ ANTIPORTER SUBUNIT D
30:00:00	7zu0-F	2.3	8.3	58	112	5	MOLECULE:	E3 UBIQUITIN-PROTEIN LIGASE PEP5
31:00:00	8at3-D	2.3	70.4	116	666	8	MOLECULE:	HAUS AUGMIN-LIKE COMPLEX SUBUNIT 1
32:00:00	3bk6-A	2.3	10.5	72	170	3	MOLECULE:	PH STOMATIN
33:00:00	8b9z-h	2.3	20.8	90	145	8	MOLECULE:	NADH-UBIQUINONE OXIDOREDUCTASE CHAIN 3
34:00:00	2pih-A	2.3	31.9	68	123	9	MOLECULE:	PROTEIN YMCA
35:00:00	6gsi-J	2.3	28.9	75	89	7	MOLECULE:	CAPSID PROTEIN
36:00:00	8dft-A	2.3	2.4	46	112	4	MOLECULE:	PILIN PROTEIN
37:00:00	6tgz-F	2.3	3.4	93	352	3	MOLECULE:	55 KDA IMMEDIATE-EARLY PROTEIN 1
38:00:00	5o31-Z	2.3	5.7	70	137	9	MOLECULE:	NADH-UBIQUINONE OXIDOREDUCTASE CHAIN 3
39:00:00	8x2q-A	2.3	4.7	62	109	5	MOLECULE:	ADENOMATOUS POLYPOSIS COLI PROTEIN
40:00:00	7vxs-o	2.3	21.9	88	128	7	MOLECULE:	NADH DEHYDROGENASE [UBIQUINONE] IRON-SULFUR
41:00:00	8vb2-M	2.3	23.6	85	125	4	MOLECULE:	TETRAMERIC EJECTION PROTEIN (GP48)
42:00:00	6mdm-H	2.3	20.1	83	147	5	MOLECULE:	VESICLE-FUSING ATPASE
43:00:00	9bax-C	2.3	16.3	62	64	2	MOLECULE:	PROTEIN EFR3 HOMOLOG A
44:00:00	8xks-S	2.3	7.7	60	114	7	MOLECULE:	CTAP1
45:00:00	6z6o-D	2.3	33	111	542	7	MOLECULE:	HISTONE DEACETYLASE HDA1
46:00:00	5t58-N	2.2	12.7	79	196	8	MOLECULE:	KLLA0F02343P
47:00:00	4gwp-D	2.2	7.3	78	121	3	MOLECULE:	MEDIATOR OF RNA POLYMERASE II TRANSCRIPTION
48:00:00	3f6n-B	2.2	5.6	67	74	6	MOLECULE:	VIRION-ASSOCIATED PROTEIN

49:00:00	9ivk-A	2.2	3.8	71	278	11	MOLECULE:	HBC599 MEMBRANE PROTEIN BINDER
50:00:00	6h2x-A	2.2	8	105	352	7	MOLECULE:	CHROMOSOME PARTITION PROTEIN MUKB,CHROMOSOME
51:00:00	8e4c-C	2.2	7.7	79	142	4	MOLECULE:	ISOFORM 2 OF IMMUNOGLOBULIN HEAVY CONSTANT M
52:00:00	1u4q-A	2.2	5.8	103	318	9	MOLECULE:	SPECTRIN ALPHA CHAIN, BRAIN
53:00:00	7sgr-A	2.2	12.8	63	700	6	MOLECULE:	ALPHA-HEMOLYSIN TRANSLOCATION ATP-BINDING PR
54:00:00	8pmq-9	2.2	10.5	77	412	5	MOLECULE:	E3 UBIQUITIN-PROTEIN LIGASE RMD5
55:00:00	2odu-A	2.2	2.3	74	217	12	MOLECULE:	PLECTIN 1
56:00:00	8ip4-A	2.2	11.3	97	320	7	MOLECULE:	MAGNESIUM TRANSPORTER MRS2 HOMOLOG, MITOCHON
57:00:00	7b93-L	2.2	26.9	112	607	5	MOLECULE:	NADH-UBIQUINONE OXIDOREDUCTASE CHAIN 3
58:00:00	3edv-A	2.2	9.2	105	322	8	MOLECULE:	SPECTRIN BETA CHAIN, BRAIN 1
59:00:00	4lws-A	2.2	2.5	59	100	7	MOLECULE:	UNCHARACTERIZED PROTEIN
60:00:00	8bd7-G	2.2	35.6	89	205	11	MOLECULE:	IFT88
61:00:00	6zh3-A	2.2	16.9	91	170	7	MOLECULE:	VACUOLAR PROTEIN-SORTING-ASSOCIATED PROTEIN
62:00:00	5xbt-A	2.2	10	69	271	12	MOLECULE:	PROBABLE TRANSCRIPTIONAL REGULATOR
63:00:00	5vk5-B	2.2	3.9	61	282	10	MOLECULE:	POTASSIUM CHANNEL SUBFAMILY K MEMBER 2
64:00:00	1jfi-B	2.2	19.2	78	135	10	MOLECULE:	5'-
65:00:00	7uxc-R	2.2	18.3	60	107	5	MOLECULE:	SERINE/THREONINE-PROTEIN KINASE MTOR
66:00:00	8t9l-B	2.2	14.7	66	110	8	MOLECULE:	NUCLEOPORIN POM152
67:00:00	4y66-D	2.2	18.1	100	208	10	MOLECULE:	MND1
68:00:00	6e52-A	2.2	29.4	90	243	6	MOLECULE:	STAPHYLOCOCCUS AUREUS AGRC HISTIDINE KINASE
69:00:00	7xzj-G	2.2	2.9	52	73	2	MOLECULE:	CTAP3
70:00:00	2mi2-A	2.2	16.2	79	104	9	MOLECULE:	SEC-INDEPENDENT PROTEIN TRANSLOCASE PROTEIN
71:00:00	4aur-A	2.2	9.1	82	564	7	MOLECULE:	LEOA
72:00:00	5ik2-G	2.2	2.6	82	285	9	MOLECULE:	ATP SYNTHASE SUBUNIT ALPHA
73:00:00	6zsi-D	2.2	5.4	66	133	9	MOLECULE:	RAS-RELATED PROTEIN RAB-8A
74:00:00	6lk8-a	2.2	16.3	93	1272	12	MOLECULE:	MGC83295 PROTEIN
75:00:00	2p4w-B	2.1	21.8	73	198	8	MOLECULE:	TRANSCRIPTIONAL REGULATORY PROTEIN ARSR FAMI
76:00:00	5j9q-D	2.1	6.1	63	120	0	MOLECULE:	HISTONE ACETYLTRANSFERASE ESA1
77:00:00	3caz-B	2.1	6.4	82	210	5	MOLECULE:	BAR PROTEIN
78:00:00	7vcf-C	2.1	17.7	88	217	7	MOLECULE:	TIC214
79:00:00	4gnk-E	2.1	50.5	84	235	4	MOLECULE:	GUANINE NUCLEOTIDE-BINDING PROTEIN G(Q) SUBU
80:00:00	8q3v-b	2.1	3.2	55	70	11	MOLECULE:	TETRAHYDROMETHANOPTERIN S-METHYLTRANSFERASE
81:00:00	6lom-A	2.1	19.1	97	295	7	MOLECULE:	CALCIUM HOMEOSTASIS MODULATOR PROTEIN
82:00:00	8tsh-F	2.1	10.2	93	265	4	MOLECULE:	ABC TRANSPORTER ATP-BINDING PROTEIN
83:00:00	6j72-A	2.1	14.9	85	561	6	MOLECULE:	ISONIAZID INDUCIBLE GENE PROTEIN INIA
84:00:00	8cbk-F	2.1	27.6	79	344	5	MOLECULE:	3-HYDROXYACYL-COA DEHYDROGENASE TYPE-2
85:00:00	2m0q-A	2.1	41	85	123	4	MOLECULE:	POTASSIUM VOLTAGE-GATED CHANNEL SUBFAMILY E
86:00:00	6x0a-a	2.1	31.3	64	87	6	MOLECULE:	PLASMID STABILIZATION SYSTEM
87:00:00	2w0c-S	2.1	22.5	70	84	6	MOLECULE:	MAJOR CAPSID PROTEIN P2
88:00:00	2ehw-C	2.1	2.1	47	117	9	MOLECULE:	HYPOTHETICAL PROTEIN TTHB059
89:00:00	6cnn-A	2.1	37.4	73	360	11	MOLECULE:	INTERMEDIATE CONDUCTANCE CALCIUM-ACTIVATED P
90:00:00	8b9z-Z	2.1	8.4	70	146	9	MOLECULE:	NADH-UBIQUINONE OXIDOREDUCTASE CHAIN 3
91:00:00	7z7s-A	2.1	34.6	82	131	5	MOLECULE:	NADH-QUINONE OXIDOREDUCTASE SUBUNIT F
92:00:00	8kde-3	2.1	10.7	88	97	13	MOLECULE:	PHOTOSYSTEM II CP47 REACTION CENTER PROTEIN
93:00:00	8b6j-i	2.1	19.5	88	117	6	MOLECULE:	PEPTIDASE M16 INACTIVE DOMAIN PROTEIN
94:00:00	4ad8-A	2.1	10.3	110	452	6	MOLECULE:	DNA REPAIR PROTEIN RECN
95:00:00	7zu0-E	2.1	35.5	107	343	8	MOLECULE:	E3 UBIQUITIN-PROTEIN LIGASE PEP5
96:00:00	8eki-A	2.1	3.7	71	170	13	MOLECULE:	PROTEIN TRANSPORT PROTEIN SEC20
97:00:00	6zka-p	2.1	22.4	89	128	7	MOLECULE:	NADH-UBIQUINONE OXIDOREDUCTASE CHAIN 3
98:00:00	7nb6-A	2.1	31.8	75	340	9	MOLECULE:	AI-2 TRANSPORT PROTEIN TQSA

99:00:00	3mhh-C	2.1	10.2	64	93	14	MOLECULE:	UBIQUITIN CARBOXYL-TERMINAL HYDROLASE 8
0:00	1llm-C	2.1	3.9	55	87	13	MOLECULE:	5'-D(*TP*CP*CP*CP*AP*CP*GP*CP*GP*TP*GP*GP*G)
1:00	3fx7-B	2.1	45.7	62	87	5	MOLECULE:	PUTATIVE UNCHARACTERIZED PROTEIN
2:00	7mvv-A	2.1	20.1	102	1542	7	MOLECULE:	NUCLEOPORIN NUP192
3:00	2wwb-B	2	10.3	61	68	10	MOLECULE:	PROTEIN TRANSPORT PROTEIN SEC61 SUBUNIT ALPH
4:00	7yzp-C	2	8.1	94	448	9	MOLECULE:	DNA HAIRPIN (59-MER)
5:00	8z9y-F	2	15.2	77	95	6	MOLECULE:	PROTEIN TIC 214
6:00	7emf-J	2	6.3	68	122	6	MOLECULE:	MEDIATOR OF RNA POLYMERASE II TRANSCRIPTION
7:00	6thk-A	2	9	107	466	13	MOLECULE:	PYOCIN S5
8:00	6rwb-A	2	39.5	141	1873	10	MOLECULE:	TOXIN,TOXIN COMPLEX SUBUNIT TCAB,PUTATIVE TO
9:00	1u2m-C	2	5.4	63	143	11	MOLECULE:	HISTONE-LIKE PROTEIN HLP-1
10:00	6xp5-G	2	4.3	70	122	9	MOLECULE:	MEDIATOR OF RNA POLYMERASE II TRANSCRIPTION
11:00	8rrh-B	2	29.2	94	299	6	MOLECULE:	PROHIBITIN 1
12:00	8t1i-F	2	6.8	56	73	7	MOLECULE:	MEDIATOR OF RNA POLYMERASE II TRANSCRIPTION
13:00	9mli-B	2	50.3	118	2519	8	MOLECULE:	A COMPONENT OF INSECTICIDAL TOXIN COMPLEX (T
14:00	5n9j-A	2	26.1	118	566	5	MOLECULE:	MEDIATOR OF RNA POLYMERASE II TRANSCRIPTION
15:00	1i49-A	2	3.4	71	201	8	MOLECULE:	ARFAPTIN 2

DALI results: MdA-1 Cah

No:	Chain	Z	rmsd	lali	nres	%id	PDB Description			
1:00	6gao-B	2.8	71.6	162	225	7	MOLECULE:	OUTER CAPSID PROTEIN SIGMA-1		
2:00	6gao-A	2.8	68.3	156	223	5	MOLECULE:	OUTER CAPSID PROTEIN SIGMA-1		
3:00	8th8-B	2.7	64.4	154	276	3	MOLECULE:	DYNEIN REGULATORY COMPLEX PROTEIN 1/2	N-TERMINAL	
4:00	7dl2-C	2.7	85.9	168	224	7	MOLECULE:	HAMARTIN		
5:00	2v71-B	2.7	60.3	137	160	8	MOLECULE:	NUCLEAR DISTRIBUTION PROTEIN NUDE-LIK	E 1	
6:00	8at3-H	2.7	42.5	160	203	4	MOLECULE:	HAUS AUGMIN-LIKE COMPLEX SUBUNIT 1		
7:00	7dl2-D	2.6	89.5	173	226	9	MOLECULE:	HAMARTIN		
8:00	8tek-B	2.6	44.6	136	208	9	MOLECULE:	DYNEIN REGULATORY COMPLEX PROTEIN 1/2	N-TERMINAL	
9:00	8tek-P	2.6	40.9	114	138	8	MOLECULE:	DYNEIN REGULATORY COMPLEX PROTEIN 1/2	N-TERMINAL	
10:00	8th8-A	2.6	68.4	159	290	8	MOLECULE:	DYNEIN REGULATORY COMPLEX PROTEIN 1/2	N-TERMINAL	
11:00	5cws-J	2.6	22.4	130	180	6	MOLECULE:	SAB-158 FAB LIGHT CHAIN		
12:00	8sre-D	2.6	38.1	101	1377	6	MOLECULE:	TRPM2 CHANZYME		
13:00	8at4-H	2.6	42.1	161	203	5	MOLECULE:	HAUS AUGMIN-LIKE COMPLEX SUBUNIT 1		
14:00	6gak-A	2.6	50.2	119	129	1	MOLECULE:	OUTER CAPSID PROTEIN SIGMA-1		
15:00	8sri-C	2.6	36.4	94	1368	2	MOLECULE:	TRPM2 CHANZYME		
16:00	8sr7-A	2.6	37	100	1375	5	MOLECULE:	TRPM2 CHANZYME		
17:00	5mq4-D	2.6	5.1	83	111	7	MOLECULE:	PROTEIN KINASE C-BINDING PROTEIN 1		
18:00	4cpc-G	2.5	60.7	130	152	6	MOLECULE:	SYNAPTONEMAL COMPLEX PROTEIN 3		
19:00	2v71-A	2.5	61.1	133	160	5	MOLECULE:	NUCLEAR DISTRIBUTION PROTEIN NUDE-LIK	E 1	
20:00	8sre-A	2.5	38	98	1377	6	MOLECULE:	TRPM2 CHANZYME		
21:00	8fck-H	2.5	42.2	167	218	4	MOLECULE:	HAUS AUGMIN-LIKE COMPLEX SUBUNIT 1		
22:00	8p0v-L	2.5	51.3	174	416	7	MOLECULE:	COILED-COIL DOMAIN-CONTAINING PROTEIN		93
23:00	5cws-D	2.5	23	134	180	3	MOLECULE:	SAB-158 FAB LIGHT CHAIN		
24:00:00	3o0z-D	2.5	59.5	135	160	7	MOLECULE:	RHO-ASSOCIATED PROTEIN KINASE 1		
25:00:00	8th8-J	2.5	68.2	156	372	8	MOLECULE:	DYNEIN REGULATORY COMPLEX PROTEIN 1/2	N-TERMINAL	
26:00:00	8t9l-A	2.5	7.5	93	108	9	MOLECULE:	NUCLEOPORIN POM152		
27:00:00	8fwj-A	2.5	2.4	82	552	5	MOLECULE:	CIRCADIAN CLOCK PROTEIN KAIC		
28:00:00	3v6i-A	2.5	25.2	104	186	2	MOLECULE:	V-TYPE ATP SYNTHASE SUBUNIT E		
29:00:00	7qoo-H	2.5	26	107	210	8	MOLECULE:	CENTROMERE PROTEIN C		
30:00:00	5hda-A	2.5	5.6	85	121	5	MOLECULE:	ZINC FINGER MYND DOMAIN-CONTAINING PR	OTEIN 11	
31:00:00	7apk-m	2.5	44.1	100	549	7	MOLECULE:	THO COMPLEX SUBUNIT 1		
32:00:00	7x2e-A	2.5	2.3	75	163	8	MOLECULE:	HARMONIN		
33:00:00	4lin-L	2.5	84.9	181	289	9	MOLECULE:	TAIL NEEDLE PROTEIN GP26		
34:00:00	2ocy-B	2.5	59	136	149	6	MOLECULE:	RAB GUANINE NUCLEOTIDE EXCHANGE FACTO	R SEC2	
35:00:00	8sre-C	2.5	38.3	100	1377	8	MOLECULE:	TRPM2 CHANZYME		
36:00:00	4cpc-A	2.5	59.8	127	143	6	MOLECULE:	SYNAPTONEMAL COMPLEX PROTEIN 3		
37:00:00	8srf-B	2.5	38.4	107	1377	8	MOLECULE:	TRPM2 CHANZYME		
38:00:00	8sr7-C	2.5	38.2	103	1375	8	MOLECULE:	TRPM2 CHANZYME		
39:00:00	8sr7-D	2.5	37.5	98	1375	8	MOLECULE:	TRPM2 CHANZYME		
40:00:00	6xer-E	2.5	3	87	120	2	MOLECULE:	TUBULIN ALPHA-1B CHAIN		
41:00:00	6i5c-E	2.5	3.4	88	120	10	MOLECULE:	TUBULIN ALPHA-1B CHAIN		
42:00:00	7znk-O	2.5	39.9	102	164	7	MOLECULE:	RNA		
43:00:00	7l05-E	2.5	3.6	89	121	9	MOLECULE:	TUBULIN ALPHA-1B CHAIN		
44:00:00	8bdf-E	2.5	3.4	88	121	10	MOLECULE:	TUBULIN ALPHA-1B CHAIN		
45:00:00	4mvd-F	2.5	4.7	86	253	9	MOLECULE:	CHOLINE-PHOSPHATE CYTIDYLYLTRANSFERAS	E A	
46:00:00	7ttd-E	2.5	3.3	88	124	9	MOLECULE:	TUBULIN ALPHA-1B CHAIN		
47:00:00	4wbn-E	2.5	3.5	89	120	9	MOLECULE:	TUBULIN ALPHA-1B CHAIN		
48:00:00	5jcb-E	2.5	3.4	88	121	10	MOLECULE:	TUBULIN ALPHA-1B CHAIN		
49:00:00	8fwj-D	2.5	2.4	82	552	5	MOLECULE:	CIRCADIAN CLOCK PROTEIN KAIC		

50:00:00	5yl2-E	2.5	3.6	88	121	10	MOLECULE:	TUBULIN ALPHA-1B CHAIN	
51:00:00	6tde-E	2.5	3.5	91	131	8	MOLECULE:	TUBULIN ALPHA CHAIN	
52:00:00	7ttf-E	2.5	3.3	88	123	9	MOLECULE:	TUBULIN ALPHA-1B CHAIN	
53:00:00	6pc4-E	2.5	3.5	88	121	10	MOLECULE:	TUBULIN ALPHA-1B CHAIN	
54:00:00	7apk-O	2.5	43.9	108	155	6	MOLECULE:	THO COMPLEX SUBUNIT 1	
55:00:00	5o7a-E	2.5	3.5	88	118	10	MOLECULE:	TUBULIN ALPHA-1B CHAIN	
56:00:00	7znl-o	2.5	44.5	105	164	7	MOLECULE:	THO COMPLEX SUBUNIT 1	
57:00:00	5xkf-E	2.5	3.4	88	121	10	MOLECULE:	TUBULIN ALPHA-1B CHAIN	
58:00:00	5xke-E	2.5	3.5	88	121	10	MOLECULE:	TUBULIN ALPHA-1B CHAIN	
59:00:00	6r10-L	2.5	3.3	98	186	3	MOLECULE:	V-TYPE ATP SYNTHASE ALPHA CHAIN	
60:00:00	6th4-E	2.5	3.4	88	128	10	MOLECULE:	TUBULIN ALPHA CHAIN	
61:00:00	5lxs-E	2.5	4.9	89	123	4	MOLECULE:	TUBULIN ALPHA-1B CHAIN	
62:00:00	5lyj-E	2.5	3.7	90	123	10	MOLECULE:	TUBULIN ALPHA-1B CHAIN	
63:00:00	5hda-C	2.5	6.5	84	117	15	MOLECULE:	ZINC FINGER MYND DOMAIN-CONTAINING PR	OTEIN 11
64:00:00	3v6i-Y	2.5	26.4	104	185	5	MOLECULE:	V-TYPE ATP SYNTHASE SUBUNIT E	
65:00:00	7f1m-A	2.4	19.2	71	394	13	MOLECULE:	NUCLEOPROTEIN	
66:00:00	4f61-I	2.4	77.4	157	234	5	MOLECULE:	TUBULIN ALPHA CHAIN	
67:00:00	4hl8-A	2.4	50.9	136	782	9	MOLECULE:	MAJOR VAULT PROTEIN	
68:00:00	4gkw-A	2.4	59.3	133	158	8	MOLECULE:	SPINDLE ASSEMBLY ABNORMAL PROTEIN 6	
69:00:00	4ke2-A	2.4	24.4	120	196	7	MOLECULE:	TYPE I HYPERACTIVE ANTIFREEZE PROTEIN	
70:00:00	5mq4-C	2.4	6.2	85	117	7	MOLECULE:	PROTEIN KINASE C-BINDING PROTEIN 1	
71:00:00	8i03-G	2.4	14.8	112	166	7	MOLECULE:	PAIRED AMPHIPATHIC HELIX PROTEIN PST1	
72:00:00	7znk-o	2.4	43.7	106	164	7	MOLECULE:	RNA	
73:00:00	6oei-A	2.4	16.3	107	243	6	MOLECULE:	SPINDLE POLE BODY COMPONENT SPC42,SIG	MA-54-DEPEND
74:00:00	7woo-I	2.4	17.2	107	187	7	MOLECULE:	NUCLEOPORIN NIC96	
75:00:00	6abo-A	2.4	3.8	87	213	7	MOLECULE:	DNA REPAIR PROTEIN XRCC4	
76:00:00	4cpc-C	2.4	57.9	125	144	9	MOLECULE:	SYNAPTONEMAL COMPLEX PROTEIN 3	
77:00:00	7ypw-A	2.4	17	68	388	13	MOLECULE:	NUCLEOPROTEIN	
78:00:00	8srd-B	2.4	38.2	108	1368	5	MOLECULE:	TRPM2 CHANZYME	
79:00:00	2v66-B	2.4	39.7	104	111	12	MOLECULE:	NUCLEAR DISTRIBUTION PROTEIN NUDE-LIK	E 1
80:00:00	4cpc-E	2.4	57.6	129	143	7	MOLECULE:	SYNAPTONEMAL COMPLEX PROTEIN 3	
81:00:00	8sr7-B	2.4	38.1	103	1375	9	MOLECULE:	TRPM2 CHANZYME	
82:00:00	5xag-E	2.4	3.6	88	120	9	MOLECULE:	TUBULIN ALPHA-1B CHAIN	
83:00:00	5s5s-E	2.4	3.4	89	123	9	MOLECULE:	TUBULIN ALPHA-1B CHAIN	
84:00:00	5bmv-E	2.4	3.6	89	123	9	MOLECULE:	TUBULIN ALPHA-1B CHAIN	
85:00:00	6r0z-L	2.4	26.7	108	185	2	MOLECULE:	V-TYPE ATP SYNTHASE ALPHA CHAIN	
86:00:00	7z7d-E	2.4	3.5	86	122	9	MOLECULE:	TUBULIN ALPHA-1B CHAIN	
87:00:00	5s5u-E	2.4	3.5	89	123	9	MOLECULE:	TUBULIN ALPHA-1B CHAIN	
88:00:00	4i55-E	2.4	3.4	89	124	9	MOLECULE:	TUBULIN ALPHA-1B CHAIN	
89:00:00	6y4n-E	2.4	3.5	89	123	9	MOLECULE:	TUBULIN ALPHA-1B CHAIN	
90:00:00	7dp8-E	2.4	4.7	91	123	9	MOLECULE:	TUBULIN ALPHA-1B CHAIN	
91:00:00	5gon-E	2.4	22.5	89	121	9	MOLECULE:	TUBULIN ALPHA-1B CHAIN	
92:00:00	5s62-E	2.4	3.5	89	123	9	MOLECULE:	TUBULIN ALPHA-1B CHAIN	
93:00:00	7apk-o	2.4	41.6	107	155	7	MOLECULE:	THO COMPLEX SUBUNIT 1	
94:00:00	7cpd-E	2.4	3.6	88	121	10	MOLECULE:	TUBULIN ALPHA-1B CHAIN	
95:00:00	7db9-E	2.4	3.5	88	124	10	MOLECULE:	TUBULIN ALPHA-1B CHAIN	
96:00:00	7znk-G	2.4	38	100	161	6	MOLECULE:	RNA	
97:00:00	5sba-E	2.4	3.4	88	121	10	MOLECULE:	TUBULIN ALPHA-1B CHAIN	
98:00:00	5ezy-E	2.4	3.2	86	121	8	MOLECULE:	TUBULIN ALPHA-1B CHAIN	
99:00:00	5s5o-E	2.4	3.4	89	123	9	MOLECULE:	TUBULIN ALPHA-1B CHAIN	
0:00	7xr1-E	2.4	3.4	88	123	10	MOLECULE:	TUBULIN ALPHA-1B CHAIN	

DALI results: MdA-1 Macc

No:	Chain	Z	rmsd	lali	nres	%id	PDB Description	
1:00	5jdo-C	5.9	3.4	86	141	3	MOLECULE:	HAPTOGLOBIN
2:00	7kyr-A	5.7	3.8	90	151	11	MOLECULE:	MYOGLOBIN
3:00	6g5b-A	5.7	3.8	90	154	12	MOLECULE:	MYOGLOBIN
4:00	3e5o-A	5.6	3.9	88	153	13	MOLECULE:	MYOGLOBIN
5:00	7a44-A	5.6	4	93	154	11	MOLECULE:	MYOGLOBIN
6:00	7sph-A	5.6	3.9	92	154	11	MOLECULE:	MYOGLOBIN
7:00	5yzf-A	5.5	4	93	154	11	MOLECULE:	SPERM WHALE MY(
8:00	6f1a-A	5.4	3.8	90	154	11	MOLECULE:	MYOGLOBIN
9:00	5xkv-A	5.4	3.8	89	153	12	MOLECULE:	MYOGLOBIN
10:00	5b84-A	5.4	3.8	89	153	12	MOLECULE:	MYOGLOBIN
11:00	5hav-A	5.4	3.8	89	153	12	MOLECULE:	MYOGLOBIN
12:00	6e03-A	5.4	3.9	88	153	11	MOLECULE:	MYOGLOBIN
13:00	4tyx-A	5.4	3.8	89	153	12	MOLECULE:	MYOGLOBIN
14:00	2blh-A	5.3	4	90	153	13	MOLECULE:	MYOGLOBIN
15:00	8eko-A	5.3	3.9	88	153	11	MOLECULE:	MYOGLOBIN
16:00	7l3u-A	5.3	3.9	91	153	11	MOLECULE:	MYOGLOBIN
17:00	6krc-A	5.3	3.9	89	153	12	MOLECULE:	MYOGLOBIN
18:00	1ch9-A	5.3	4	92	154	12	MOLECULE:	PROTEIN (MY
19:00	7xcq-A	5.2	3.9	88	153	11	MOLECULE:	MYOGLOBIN
20:00	6az6-B	5.2	7.4	99	205	10	MOLECULE:	GNTR FAMILY
21:00	8j4k-A	5.2	3.9	91	153	11	MOLECULE:	MYOGLOBIN
22:00	7xc9-A	5.2	4	91	153	11	MOLECULE:	MYOGLOBIN
23:00	7l3y-A	5.2	3.8	90	153	11	MOLECULE:	MYOGLOBIN
24:00:00	2r4w-A	5.2	3.3	84	145	10	MOLECULE:	GLOBIN-1
25:00:00	1myt-A	5.2	3.8	85	145	8	MOLECULE:	MYOGLOBIN
26:00:00	3a59-B	5.2	3.6	85	146	8	MOLECULE:	HEMOGLOBIN
27:00:00	7spe-A	5.2	4	90	154	11	MOLECULE:	MYOGLOBIN
28:00:00	6az6-A	5.1	8	102	209	9	MOLECULE:	GNTR FAMILY
29:00:00	5azr-A	5.1	3.9	88	153	14	MOLECULE:	MYOGLOBIN
30:00:00	5zzf-A	5.1	4.1	90	153	11	MOLECULE:	MYOGLOBIN
31:00:00	3ecx-A	5.1	3.9	88	153	13	MOLECULE:	MYOGLOBIN
32:00:00	3w4u-E	5.1	3.1	85	138	4	MOLECULE:	HEMOGLOBIN
33:00:00	1dtm-A	5.1	3.8	89	152	11	MOLECULE:	RECOMBINANT
34:00:00	5yce-A	5.1	3.9	89	151	13	MOLECULE:	MYOGLOBIN
35:00:00	2bnl-C	5	4	84	134	8	MOLECULE:	MODULATOR P
36:00:00	5yci-B	5	3.9	94	157	14	MOLECULE:	ANCESTRAL M
37:00:00	7xzi-B	5	4.4	93	166	6	MOLECULE:	CTAP3
38:00:00	3qm9-A	5	4	85	144	8	MOLECULE:	MYOGLOBIN
39:00:00	3w4u-C	5	3.5	86	141	2	MOLECULE:	HEMOGLOBIN
40:00:00	5ycj-B	5	4	94	157	14	MOLECULE:	ANCESTRAL M
41:00:00	6ls8-I	5	3.8	91	153	13	MOLECULE:	MYOGLOBIN
42:00:00	3rgk-A	5	3.9	90	149	12	MOLECULE:	MYOGLOBIN

43:00:00	3w4u-A	5	3.6	86	141	2	MOLECULE:	HEMOGLOBIN
44:00:00	4nxa-A	5	3.8	89	153	12	MOLECULE:	MYOGLOBIN
45:00:00	1v75-A	5	3.5	87	141	6	MOLECULE:	HEMOGLOBIN
46:00:00	1nz3-A	5	4	90	153	14	MOLECULE:	MYOGLOBIN
47:00:00	7spg-A	5	4	92	152	12	MOLECULE:	MYOGLOBIN
48:00:00	3s1i-A	5	3.7	85	131	9	MOLECULE:	HEMOGLOBIN-
49:00:00	8oup-D	5	4.2	92	151	7	MOLECULE:	NERVE HEMOG
50:00:00	1u7s-A	4.9	3.9	91	153	11	MOLECULE:	MYOGLOBIN
51:00:00	1ebc-A	4.9	3.9	91	153	11	MOLECULE:	PROTEIN (MY
52:00:00	2frk-X	4.9	4	91	152	14	MOLECULE:	MYOGLOBIN
53:00:00	1wvp-A	4.9	3.9	89	151	12	MOLECULE:	MYOGLOBIN
54:00:00	3lr7-A	4.9	4	90	151	14	MOLECULE:	MYOGLOBIN
55:00:00	2frj-X	4.9	4	90	152	14	MOLECULE:	MYOGLOBIN
56:00:00	1lht-A	4.9	3.7	89	153	12	MOLECULE:	MYOGLOBIN
57:00:00	6q6p-B	4.9	4.2	91	157	3	MOLECULE:	CYTOGLOBIN
58:00:00	2nsr-A	4.9	4	90	152	14	MOLECULE:	MYOGLOBIN
59:00:00	1jeb-C	4.9	3.9	88	141	2	MOLECULE:	HEMOGLOBIN
60:00:00	5zzg-A	4.9	4.1	90	153	11	MOLECULE:	MYOGLOBIN
61:00:00	6ls8-G	4.9	3.9	90	153	14	MOLECULE:	MYOGLOBIN
62:00:00	4dc7-A	4.9	4	90	152	14	MOLECULE:	MYOGLOBIN
63:00:00	1lhs-A	4.9	3.7	89	153	12	MOLECULE:	MYOGLOBIN
64:00:00	6ls8-K	4.9	3.9	90	153	14	MOLECULE:	MYOGLOBIN
65:00:00	1nz4-A	4.9	4	90	153	14	MOLECULE:	MYOGLOBIN
66:00:00	4nxc-A	4.9	3.8	89	153	12	MOLECULE:	MYOGLOBIN
67:00:00	3qm7-A	4.9	3.9	85	144	8	MOLECULE:	MYOGLOBIN
68:00:00	1l2k-A	4.9	3.9	89	151	12	MOLECULE:	MYOGLOBIN
69:00:00	6ls8-C	4.9	3.9	91	153	14	MOLECULE:	MYOGLOBIN
70:00:00	1jeb-A	4.9	3.4	86	141	3	MOLECULE:	HEMOGLOBIN
71:00:00	5o41-A	4.9	4	93	154	11	MOLECULE:	MYOGLOBIN
72:00:00	8oup-B	4.9	3.8	91	151	8	MOLECULE:	NERVE HEMOG
73:00:00	8oup-A	4.9	3.9	89	150	6	MOLECULE:	NERVE HEMOG
74:00:00	3s1j-A	4.9	3.5	84	131	10	MOLECULE:	HEMOGLOBIN-
75:00:00	8oup-C	4.9	3.9	91	151	8	MOLECULE:	NERVE HEMOG
76:00:00	3ubc-D	4.9	3.7	84	131	7	MOLECULE:	HEMOGLOBIN-
77:00:00	3s1i-C	4.9	3.7	85	131	8	MOLECULE:	HEMOGLOBIN-
78:00:00	2ri4-C	4.9	3.7	86	138	7	MOLECULE:	HEMOGLOBIN
79:00:00	3pt7-B	4.8	4.2	89	152	4	MOLECULE:	HEMOGLOBIN
80:00:00	3ase-A	4.8	4	90	154	11	MOLECULE:	MYOGLOBIN
81:00:00	1hbr-C	4.8	3.5	87	140	6	MOLECULE:	PROTEIN (HE
82:00:00	1hbr-A	4.8	3.6	88	141	6	MOLECULE:	PROTEIN (HE
83:00:00	1do7-A	4.8	3.9	88	153	13	MOLECULE:	MYOGLOBIN
84:00:00	105m-A	4.8	3.9	90	153	11	MOLECULE:	MYOGLOBIN
85:00:00	2eb8-A	4.8	3.8	91	154	11	MOLECULE:	MYOGLOBIN
86:00:00	3qm5-A	4.8	3.9	84	145	8	MOLECULE:	MYOGLOBIN

87:00:00	4rol-A	4.8	3	85	141	4	MOLECULE:	HEMOGLOBIN
88:00:00	4pqb-A	4.8	4	90	153	12	MOLECULE:	MYOGLOBIN
89:00:00	1rse-A	4.8	4	90	153	14	MOLECULE:	HORSE HEART
90:00:00	7ddt-A	4.8	3.9	90	153	12	MOLECULE:	ANCESTRAL M
91:00:00	5cnc-A	4.8	3.9	90	152	14	MOLECULE:	MYOGLOBIN
92:00:00	1j3f-A	4.8	3.5	88	152	13	MOLECULE:	MYOGLOBIN
93:00:00	5mbn-A	4.8	4	91	153	11	MOLECULE:	MYOGLOBIN
94:00:00	6ls8-A	4.8	3.9	91	153	13	MOLECULE:	MYOGLOBIN
95:00:00	1iop-A	4.8	3.9	91	153	11	MOLECULE:	MYOGLOBIN
96:00:00	5cne-A	4.8	4	90	152	14	MOLECULE:	MYOGLOBIN
97:00:00	1nz5-A	4.8	4	90	153	14	MOLECULE:	MYOGLOBIN
98:00:00	1yhr-A	4.8	3.1	85	141	4	MOLECULE:	HEMOGLOBIN
99:00:00	4dc8-A	4.8	4	90	152	14	MOLECULE:	MYOGLOBIN
0:00	1yoi-A	4.8	3.9	89	153	12	MOLECULE:	MYOGLOBIN
1:00	5cnb-A	4.8	3.9	90	152	14	MOLECULE:	MYOGLOBIN
2:00	4fwy-A	4.8	3.8	90	153	11	MOLECULE:	MYOGLOBIN
3:00	2o5o-X	4.8	4	90	152	14	MOLECULE:	MYOGLOBIN
4:00	1swm-A	4.8	3.9	89	153	12	MOLECULE:	MYOGLOBIN
5:00	1dwr-A	4.8	4	91	152	14	MOLECULE:	MYOGLOBIN
6:00	2v1i-A	4.8	4	90	153	14	MOLECULE:	MYOGLOBIN
7:00	2bw9-M	4.8	3.9	90	153	12	MOLECULE:	MYOGLOBIN
8:00	1mbc-A	4.8	3.9	89	153	12	MOLECULE:	MYOGLOBIN
9:00	2nss-A	4.8	4	90	152	14	MOLECULE:	MYOGLOBIN
10:00	2o5b-X	4.8	4	90	152	14	MOLECULE:	MYOGLOBIN
11:00	1m6m-A	4.8	4	91	153	13	MOLECULE:	PROTEIN (MY
12:00	3vau-A	4.8	4	90	151	14	MOLECULE:	MYOGLOBIN
13:00	5utc-A	4.8	4	89	152	13	MOLECULE:	MYOGLOBIN
14:00	1bvc-A	4.8	3.9	90	153	11	MOLECULE:	APOMYOGLOBI
15:00	1wla-A	4.8	4	90	153	14	MOLECULE:	MYOGLOBIN
16:00	5cn5-A	4.8	4	90	152	14	MOLECULE:	MYOGLOBIN
17:00	4rol-C	4.8	3.4	86	141	3	MOLECULE:	HEMOGLOBIN
18:00	1npg-A	4.8	4	90	152	14	MOLECULE:	MYOGLOBIN
19:00	8a3t-T	4.8	16.6	97	645	8	MOLECULE:	ANAPHASE-PR
20:00	1vxf-A	4.8	3.9	89	153	12	MOLECULE:	MYOGLOBIN
21:00	1dwt-A	4.8	4	90	152	14	MOLECULE:	MYOGLOBIN
22:00	104m-A	4.8	3.9	89	153	12	MOLECULE:	MYOGLOBIN
23:00	1vxe-A	4.8	3.9	89	153	12	MOLECULE:	MYOGLOBIN
24:00:00	2v1h-A	4.8	4	90	153	14	MOLECULE:	MYOGLOBIN
25:00:00	1vxg-A	4.8	3.9	89	153	12	MOLECULE:	MYOGLOBIN
26:00:00	2myd-A	4.8	3.9	89	153	12	MOLECULE:	MYOGLOBIN (
27:00:00	5utd-A	4.8	3.9	90	151	12	MOLECULE:	MYOGLOBIN
28:00:00	4twv-A	4.8	4	90	153	14	MOLECULE:	MYOGLOBIN
29:00:00	2o5s-X	4.8	4	90	152	14	MOLECULE:	MYOGLOBIN
30:00:00	3lr9-A	4.8	4	90	152	14	MOLECULE:	MYOGLOBIN

31:00:00	3rj6-B	4.8	4	90	152	14	MOLECULE:	MYOGLOBIN
32:00:00	2frf-A	4.8	4.1	90	152	16	MOLECULE:	MYOGLOBIN
33:00:00	2vlz-A	4.8	4	91	153	14	MOLECULE:	MYOGLOBIN
34:00:00	1yog-A	4.8	3.9	89	153	12	MOLECULE:	MYOGLOBIN
35:00:00	4mbn-A	4.8	3.9	90	153	12	MOLECULE:	MYOGLOBIN
36:00:00	5cng-A	4.8	3.9	90	152	14	MOLECULE:	MYOGLOBIN
37:00:00	2myb-A	4.8	3.9	89	153	12	MOLECULE:	MYOGLOBIN (
38:00:00	2mb5-A	4.8	3.9	89	153	12	MOLECULE:	MYOGLOBIN
39:00:00	1emy-A	4.8	3.9	90	153	11	MOLECULE:	MYOGLOBIN
40:00:00	1bje-A	4.8	4	90	153	14	MOLECULE:	MYOGLOBIN
41:00:00	2o58-X	4.8	4	90	152	14	MOLECULE:	MYOGLOBIN
42:00:00	2mye-A	4.8	3.9	89	153	12	MOLECULE:	MYOGLOBIN (
43:00:00	1mbi-A	4.8	3.9	89	153	12	MOLECULE:	MYOGLOBIN
44:00:00	2fri-X	4.8	4	90	152	14	MOLECULE:	MYOGLOBIN
45:00:00	2o5m-X	4.8	4	90	152	14	MOLECULE:	MYOGLOBIN
46:00:00	6q6p-A	4.8	4.1	90	156	3	MOLECULE:	CYTOGLOBIN
47:00:00	3m3b-A	4.8	3.9	91	153	11	MOLECULE:	MYOGLOBIN
48:00:00	1bzp-A	4.8	3.9	89	153	12	MOLECULE:	PROTEIN (MY
49:00:00	5cn8-A	4.8	3.9	90	152	14	MOLECULE:	MYOGLOBIN
50:00:00	2vlx-A	4.8	4	90	153	14	MOLECULE:	MYOGLOBIN
51:00:00	2v1j-A	4.8	4	90	153	14	MOLECULE:	MYOGLOBIN
52:00:00	1vxh-A	4.8	3.9	89	153	12	MOLECULE:	MYOGLOBIN
53:00:00	5zze-A	4.8	4	91	152	14	MOLECULE:	MYOGLOBIN
54:00:00	2ekt-A	4.8	4	91	153	12	MOLECULE:	MYOGLOBIN
55:00:00	2o5t-X	4.8	4	90	152	14	MOLECULE:	MYOGLOBIN
56:00:00	1vxd-A	4.8	3.9	89	153	12	MOLECULE:	MYOGLOBIN
57:00:00	5cnd-A	4.8	4	90	152	14	MOLECULE:	MYOGLOBIN
58:00:00	1bz6-A	4.8	3.9	89	153	12	MOLECULE:	PROTEIN (MY
59:00:00	3at5-A	4.8	3.4	86	141	6	MOLECULE:	ALPHAA-GLOB
60:00:00	1yoh-A	4.8	3.9	89	153	12	MOLECULE:	MYOGLOBIN
61:00:00	5cmv-A	4.8	3.9	90	152	14	MOLECULE:	MYOGLOBIN
62:00:00	1gjn-A	4.8	4	90	153	14	MOLECULE:	MYOGLOBIN
63:00:00	5azq-A	4.8	3.9	90	153	14	MOLECULE:	MYOGLOBIN
64:00:00	5cn4-A	4.8	3.9	90	152	14	MOLECULE:	MYOGLOBIN
65:00:00	1npf-A	4.8	4	90	152	14	MOLECULE:	MYOGLOBIN
66:00:00	5vzn-A	4.8	3.9	91	151	11	MOLECULE:	MYOGLOBIN
67:00:00	5vzq-A	4.8	3.9	90	153	12	MOLECULE:	MYOGLOBIN
68:00:00	2v1g-A	4.8	4	90	153	14	MOLECULE:	MYOGLOBIN
69:00:00	5cn9-A	4.8	4	90	152	14	MOLECULE:	MYOGLOBIN
70:00:00	2v1k-A	4.8	4	90	153	14	MOLECULE:	MYOGLOBIN
71:00:00	2v1f-A	4.8	4	90	153	14	MOLECULE:	MYOGLOBIN
72:00:00	5z7f-A	4.8	4	90	152	13	MOLECULE:	MYOGLOBIN
73:00:00	6ls8-E	4.8	3.9	91	153	14	MOLECULE:	MYOGLOBIN
74:00:00	1jp6-A	4.8	3.9	89	152	12	MOLECULE:	MYOGLOBIN

75:00:00	5z7e-A	4.8	4	90	153	14	MOLECULE:	MYOGLOBIN
76:00:00	2vly-A	4.8	4	90	153	14	MOLECULE:	MYOGLOBIN
77:00:00	5cnf-A	4.8	4	90	152	14	MOLECULE:	MYOGLOBIN
78:00:00	8wf5-A	4.8	4	90	152	14	MOLECULE:	MYOGLOBIN
79:00:00	5yl3-A	4.8	4	90	151	14	MOLECULE:	MYOGLOBIN
80:00:00	1xch-A	4.8	4	90	153	14	MOLECULE:	MYOGLOBIN
81:00:00	1vxc-A	4.8	3.9	89	153	12	MOLECULE:	MYOGLOBIN
82:00:00	1cq2-A	4.8	3.9	89	153	12	MOLECULE:	MYOGLOBIN
83:00:00	2v1e-A	4.8	4	90	153	14	MOLECULE:	MYOGLOBIN
84:00:00	2o5q-X	4.8	4	90	152	14	MOLECULE:	MYOGLOBIN
85:00:00	1nz2-A	4.8	4	90	153	14	MOLECULE:	MYOGLOBIN
86:00:00	1bzr-A	4.8	3.9	90	153	12	MOLECULE:	PROTEIN (MY
87:00:00	2blj-M	4.8	3.9	90	153	12	MOLECULE:	MYOGLOBIN
88:00:00	1spe-A	4.8	4	89	153	13	MOLECULE:	MYOGLOBIN
89:00:00	1dws-A	4.8	4	91	152	14	MOLECULE:	MYOGLOBIN
90:00:00	1jp8-A	4.8	3.9	89	152	12	MOLECULE:	MYOGLOBIN
91:00:00	2o5l-X	4.8	4	90	152	14	MOLECULE:	MYOGLOBIN
92:00:00	3rj6-A	4.8	4	90	152	14	MOLECULE:	MYOGLOBIN
93:00:00	2vm0-A	4.8	4	90	153	14	MOLECULE:	MYOGLOBIN
94:00:00	2cmm-A	4.8	3.8	88	153	11	MOLECULE:	MYOGLOBIN
95:00:00	2mya-A	4.8	4	89	153	13	MOLECULE:	MYOGLOBIN (
96:00:00	6oty-B	4.8	4.2	88	152	5	MOLECULE:	HEMOGLOBIN
97:00:00	6hit-A	4.8	3.3	86	142	3	MOLECULE:	HEMOGLOBIN
98:00:00	1xz4-C	4.8	3	84	141	6	MOLECULE:	HEMOGLOBIN
99:00:00	3bom-C	4.8	3.4	86	142	8	MOLECULE:	HEMOGLOBIN
0:00	1mll-A	4.8	4	90	154	11	MOLECULE:	MYOGLOBIN
1:00	3ubv-D	4.8	3.7	85	131	9	MOLECULE:	HEMOGLOBIN-
2:00	1o1p-A	4.8	4.8	95	283	3	MOLECULE:	HEMOGLOBIN
3:00	1o1l-A	4.8	5	94	283	4	MOLECULE:	HEMOGLOBIN
4:00	3s1j-C	4.8	3.8	84	131	7	MOLECULE:	HEMOGLOBIN-
5:00	1ltw-A	4.8	4	91	154	12	MOLECULE:	MYOGLOBIN
6:00	6n03-A	4.8	3.8	89	154	12	MOLECULE:	MYOGLOBIN,
7:00	107m-A	4.8	3.9	92	154	11	MOLECULE:	MYOGLOBIN
8:00	1jw8-A	4.8	4.5	92	154	9	MOLECULE:	MYOGLOBIN
9:00	2r1h-A	4.8	3.4	86	142	8	MOLECULE:	HEMOGLOBIN
10:00	3ubc-G	4.8	3.8	84	131	8	MOLECULE:	HEMOGLOBIN-
11:00	106m-A	4.8	4	90	154	11	MOLECULE:	MYOGLOBIN
12:00	1jdo-A	4.8	4	91	154	12	MOLECULE:	MYOGLOBIN
13:00	5utb-A	4.8	3.9	90	152	11	MOLECULE:	MYOGLOBIN
14:00	2r1h-C	4.8	3.4	86	142	8	MOLECULE:	HEMOGLOBIN
15:00	3ogb-A	4.8	4	92	154	12	MOLECULE:	MYOGLOBIN
16:00	3h58-A	4.8	3.9	90	154	11	MOLECULE:	MYOGLOBIN
17:00	8f9i-B	4.8	4.1	92	154	12	MOLECULE:	MYOGLOBIN
18:00	1o1j-A	4.8	5.2	95	283	3	MOLECULE:	HEMOGLOBIN

19:00	111m-A	4.8	3.9	91	154	12	MOLECULE:	MYOGLOBIN
20:00	5m3s-A	4.8	4.5	93	154	8	MOLECULE:	MYOGLOBIN
21:00	1dti-A	4.8	4	92	154	11	MOLECULE:	PROTEIN (MY
22:00	1ofj-A	4.8	3.9	92	154	11	MOLECULE:	MYOGLOBIN
23:00	108m-A	4.8	3.9	91	154	12	MOLECULE:	MYOGLOBIN
24:00:00	3bom-A	4.8	2.9	84	142	8	MOLECULE:	HEMOGLOBIN
25:00:00	1c7c-A	4.8	4.8	95	283	3	MOLECULE:	PROTEIN (DE
26:00:00	6mv0-A	4.8	3.9	90	154	12	MOLECULE:	MYOGLOBIN
27:00:00	2g0r-A	4.8	4	91	154	12	MOLECULE:	MYOGLOBIN
28:00:00	1tes-A	4.8	3.9	91	154	12	MOLECULE:	MYOGLOBIN
29:00:00	101m-A	4.8	4	92	154	11	MOLECULE:	MYOGLOBIN
30:00:00	8a5y-T	4.7	17.7	101	650	8	MOLECULE:	ANAPHASE-PR
31:00:00	1abw-A	4.7	13.4	119	287	8	MOLECULE:	HEMOGLOBIN-
32:00:00	6otw-B	4.7	4.1	87	152	5	MOLECULE:	HEMOGLOBIN
33:00:00	3pt8-B	4.7	4.2	89	152	4	MOLECULE:	HEMOGLOBIN
34:00:00	6kye-A	4.7	3.6	88	140	2	MOLECULE:	HEMOGLOBIN
35:00:00	6kye-K	4.7	3.4	86	140	3	MOLECULE:	HEMOGLOBIN
36:00:00	6otx-B	4.7	4.2	90	152	6	MOLECULE:	HEMOGLOBIN
37:00:00	5ilm-A	4.7	4	88	153	13	MOLECULE:	MYOGLOBIN
38:00:00	4h07-A	4.7	4	90	154	11	MOLECULE:	MYOGLOBIN
39:00:00	1do4-A	4.7	3.8	87	153	11	MOLECULE:	MYOGLOBIN
40:00:00	3m3a-A	4.7	4	90	153	12	MOLECULE:	MYOGLOBIN
41:00:00	5kkk-A	4.7	4	90	154	12	MOLECULE:	MYOGLOBIN
42:00:00	1yen-A	4.7	3.6	87	138	2	MOLECULE:	HEMOGLOBIN
43:00:00	1do1-A	4.7	3.9	88	153	13	MOLECULE:	MYOGLOBIN
44:00:00	1dxc-A	4.7	3.9	88	154	11	MOLECULE:	MYOGLOBIN
45:00:00	1lfl-P	4.7	2.9	85	141	5	MOLECULE:	HEMOGLOBIN
46:00:00	3hxn-C	4.7	3.1	85	141	4	MOLECULE:	HEMOGLOBIN
47:00:00	1dxd-A	4.7	4	88	153	13	MOLECULE:	MYOGLOBIN
48:00:00	1yca-A	4.7	3.9	90	153	13	MOLECULE:	MYOGLOBIN
49:00:00	1sdk-A	4.7	3.5	87	141	2	MOLECULE:	HEMOGLOBIN
50:00:00	1aj9-A	4.7	3.5	87	141	2	MOLECULE:	HEMOGLOBIN
51:00:00	2w6w-A	4.7	4	89	153	11	MOLECULE:	MYOGLOBIN
52:00:00	7pcf-A	4.7	3.6	87	141	3	MOLECULE:	HEMOGLOBIN
53:00:00	3v2v-A	4.7	4	89	152	16	MOLECULE:	MYOGLOBIN
54:00:00	3u3e-A	4.7	3.9	88	153	13	MOLECULE:	MYOGLOBIN
55:00:00	4twu-A	4.7	3.8	88	153	15	MOLECULE:	MYOGLOBIN
56:00:00	4rom-A	4.7	3.1	85	141	4	MOLECULE:	HEMOGLOBIN
57:00:00	1do3-A	4.7	3.9	88	153	13	MOLECULE:	MYOGLOBIN
58:00:00	2bnl-F	4.7	3.9	79	129	8	MOLECULE:	MODULATOR P
59:00:00	6bmg-B	4.7	4	91	154	13	MOLECULE:	MYOGLOBIN
60:00:00	1hjt-A	4.7	4	90	153	12	MOLECULE:	MYOGLOBIN
61:00:00	8u5a-A	4.7	3.5	84	149	7	MOLECULE:	DESIGNED MY
62:00:00	1azi-A	4.7	4	90	153	14	MOLECULE:	MYOGLOBIN

63:00:00	1mnk-A	4.7	4.1	91	151	13	MOLECULE:	MYOGLOBIN
64:00:00	2zsn-A	4.7	3.9	88	153	11	MOLECULE:	MYOGLOBIN
65:00:00	3edb-A	4.7	3.9	88	153	11	MOLECULE:	MYOGLOBIN
66:00:00	2hbd-A	4.7	3.4	86	141	2	MOLECULE:	HEMOGLOBIN
67:00:00	1vwt-C	4.7	3.3	85	141	5	MOLECULE:	HEMOGLOBIN
68:00:00	6cf0-A	4.7	4	88	153	13	MOLECULE:	MYOGLOBIN
69:00:00	6f17-A	4.7	3.8	89	153	11	MOLECULE:	MYOGLOBIN
70:00:00	1myh-A	4.7	3.9	91	153	13	MOLECULE:	MYOGLOBIN
71:00:00	1sdl-C	4.7	3.4	86	141	3	MOLECULE:	HEMOGLOBIN
72:00:00	1y45-A	4.7	3	84	141	5	MOLECULE:	HEMOGLOBIN
73:00:00	5vrt-A	4.7	3.9	88	153	11	MOLECULE:	MYOGLOBIN
74:00:00	5ut9-A	4.7	4	88	153	13	MOLECULE:	MYOGLOBIN
75:00:00	8f9j-A	4.7	4	88	153	13	MOLECULE:	MYOGLOBIN
76:00:00	4m4b-A	4.7	3.4	85	140	5	MOLECULE:	HEMOGLOBIN
77:00:00	2jho-A	4.7	3.9	89	153	12	MOLECULE:	MYOGLOBIN
78:00:00	1rq3-C	4.7	3.1	85	141	4	MOLECULE:	HEMOGLOBIN
79:00:00	1yev-C	4.7	2.9	83	138	5	MOLECULE:	HEMOGLOBIN
80:00:00	1duo-A	4.7	3.8	90	152	11	MOLECULE:	SPERM WHALE
81:00:00	2zt2-A	4.7	3.9	89	153	12	MOLECULE:	MYOGLOBIN
82:00:00	1c7b-A	4.7	3	84	141	5	MOLECULE:	PROTEIN (DE
83:00:00	1xzv-C	4.7	3.1	85	141	4	MOLECULE:	HEMOGLOBIN
84:00:00	1y7g-C	4.7	3.1	85	141	4	MOLECULE:	HEMOGLOBIN
85:00:00	3sdn-A	4.7	3.9	89	152	12	MOLECULE:	MYOGLOBIN
86:00:00	2myc-A	4.7	3.9	89	153	12	MOLECULE:	MYOGLOBIN (
87:00:00	2bnl-A	4.7	4	79	129	8	MOLECULE:	MODULATOR P
88:00:00	1yeo-C	4.7	3.1	85	141	4	MOLECULE:	HEMOGLOBIN
89:00:00	5vnu-A	4.7	3.9	90	153	12	MOLECULE:	MYOGLOBIN
90:00:00	1y5j-C	4.7	3.1	85	141	4	MOLECULE:	HEMOGLOBIN
91:00:00	1dxv-A	4.7	3	84	141	5	MOLECULE:	HEMOGLOBIN
92:00:00	4of9-A	4.7	3.9	88	153	11	MOLECULE:	MYOGLOBIN
93:00:00	5ilr-A	4.7	3.9	88	151	11	MOLECULE:	MYOGLOBIN
94:00:00	5iks-A	4.7	3.9	89	151	12	MOLECULE:	MYOGLOBIN
95:00:00	1mwc-A	4.7	3.9	90	153	13	MOLECULE:	PROTEIN (MY
96:00:00	2hbf-A	4.7	3.4	86	141	2	MOLECULE:	HEMOGLOBIN
97:00:00	3hhb-A	4.7	3.5	86	141	3	MOLECULE:	HEMOGLOBIN
98:00:00	1ymc-A	4.7	4	90	153	14	MOLECULE:	CYANOMET-SU
99:00:00	3mn0-A	4.7	3.8	90	153	11	MOLECULE:	MYOGLOBIN
0:00	4it8-A	4.7	3.8	89	153	12	MOLECULE:	MYOGLOBIN
1:00	1lfl-C	4.7	3.1	85	141	4	MOLECULE:	HEMOGLOBIN
2:00	1a0z-C	4.7	3	85	141	6	MOLECULE:	HEMOGLOBIN
3:00	2dn2-A	4.7	3	84	141	5	MOLECULE:	HEMOGLOBIN
4:00	1y4f-C	4.7	3.1	85	141	4	MOLECULE:	HEMOGLOBIN
5:00	1y4v-C	4.7	3.5	87	141	2	MOLECULE:	HEMOGLOBIN
6:00	3wfu-A	4.7	4	90	152	14	MOLECULE:	MYOGLOBIN

7:00	1a6k-A	4.7	3.9	89	151	13	MOLECULE:	MYOGLOBIN
8:00	5ilp-A	4.7	3.9	88	151	11	MOLECULE:	MYOGLOBIN
9:00	1c7d-A	4.7	12.9	116	284	6	MOLECULE:	PROTEIN (DE
10:00	1hgc-C	4.7	3.1	85	141	4	MOLECULE:	HEMOGLOBIN
11:00	3k9z-A	4.7	3.9	91	153	11	MOLECULE:	MYOGLOBIN
12:00	1yh9-C	4.7	3	84	141	5	MOLECULE:	HEMOGLOBIN
13:00	1fsx-C	4.7	3.1	84	141	6	MOLECULE:	HEMOGLOBIN
14:00	1y4g-C	4.7	3.1	85	141	4	MOLECULE:	HEMOGLOBIN
15:00	1ye2-C	4.7	3	84	141	5	MOLECULE:	HEMOGLOBIN
16:00	1xzu-C	4.7	3.5	87	141	2	MOLECULE:	HEMOGLOBIN
17:00	1c7b-C	4.7	3	84	141	5	MOLECULE:	PROTEIN (DE
18:00	1xy0-C	4.7	3.1	85	141	4	MOLECULE:	HEMOGLOBIN
19:00	1y8w-C	4.7	3.4	86	141	3	MOLECULE:	HEMOGLOBIN
20:00	1gbv-A	4.7	3.3	85	141	5	MOLECULE:	HEMOGLOBIN
21:00	3d17-C	4.7	3.5	87	141	3	MOLECULE:	HEMOGLOBIN
22:00	1myj-A	4.7	4.1	90	153	14	MOLECULE:	MYOGLOBIN
23:00	1mno-A	4.7	3.8	90	153	13	MOLECULE:	PROTEIN (MY
24:00:00	2d6c-B	4.7	4	89	151	12	MOLECULE:	MYOGLOBIN
25:00:00	1gbv-C	4.7	3.4	86	141	3	MOLECULE:	HEMOGLOBIN
26:00:00	1myg-B	4.7	3.9	90	153	13	MOLECULE:	MYOGLOBIN
27:00:00	8fdj-A	4.7	3.9	89	151	12	MOLECULE:	MYOGLOBIN
28:00:00	5xl0-A	4.7	3.9	88	151	11	MOLECULE:	MYOGLOBIN
29:00:00	1ymb-A	4.7	4.2	90	153	14	MOLECULE:	METMYOGLOBI
30:00:00	5c6y-A	4.7	3.9	89	153	12	MOLECULE:	MYOGLOBIN
31:00:00	3wi8-A	4.7	4	90	151	14	MOLECULE:	MYOGLOBIN
32:00:00	1xz2-C	4.7	3	84	141	5	MOLECULE:	HEMOGLOBIN
33:00:00	1v4w-C	4.7	3.4	86	143	7	MOLECULE:	HEMOGLOBIN
34:00:00	1m6c-B	4.7	3.9	90	153	14	MOLECULE:	PROTEIN (MY
35:00:00	7sli-A	4.7	3.9	88	152	11	MOLECULE:	MYOGLOBIN
36:00:00	1myj-B	4.7	3.9	90	153	13	MOLECULE:	MYOGLOBIN
37:00:00	1rvw-A	4.7	3.8	88	141	2	MOLECULE:	HEMOGLOBIN
38:00:00	3e4n-A	4.7	3.9	88	153	13	MOLECULE:	MYOGLOBIN
39:00:00	3hhb-C	4.7	3.5	86	141	3	MOLECULE:	HEMOGLOBIN
40:00:00	1qsh-A	4.7	3	84	141	5	MOLECULE:	PROTEIN (HE
41:00:00	2zt4-A	4.7	4	88	153	13	MOLECULE:	MYOGLOBIN
42:00:00	1b86-C	4.7	3.6	87	141	2	MOLECULE:	PROTEIN (HE
43:00:00	2z6s-A	4.7	3.9	88	151	13	MOLECULE:	MYOGLOBIN
44:00:00	1mni-B	4.7	3.9	90	153	13	MOLECULE:	MYOGLOBIN
45:00:00	1ajh-A	4.7	3.9	88	153	11	MOLECULE:	MYOGLOBIN
46:00:00	4m4a-A	4.7	3.8	88	140	2	MOLECULE:	HEMOGLOBIN
47:00:00	1dxt-A	4.7	3	84	141	5	MOLECULE:	HEMOGLOBIN
48:00:00	2hbc-A	4.7	3.4	85	141	4	MOLECULE:	HEMOGLOBIN
49:00:00	1qsh-C	4.7	3.5	86	141	3	MOLECULE:	PROTEIN (HE
50:00:00	2hhe-C	4.7	2.9	84	141	6	MOLECULE:	HEMOGLOBIN

51:00:00	2zst-A	4.7	3.9	88	153	11	MOLECULE:	MYOGLOBIN
52:00:00	1myh-B	4.7	3.9	90	153	13	MOLECULE:	MYOGLOBIN
53:00:00	1j7w-A	4.7	3.5	86	141	3	MOLECULE:	HEMOGLOBIN
54:00:00	4pqc-A	4.7	3.8	89	153	12	MOLECULE:	MYOGLOBIN
55:00:00	1yev-A	4.7	3	84	138	4	MOLECULE:	HEMOGLOBIN
56:00:00	1vxa-A	4.7	3.9	89	153	12	MOLECULE:	MYOGLOBIN
57:00:00	1xz7-A	4.7	3.1	85	141	4	MOLECULE:	HEMOGLOBIN
58:00:00	3hc9-A	4.7	3.8	88	153	14	MOLECULE:	MYOGLOBIN
59:00:00	2bwh-A	4.7	3.9	90	153	12	MOLECULE:	MYOGLOBIN
60:00:00	1gli-C	4.7	3.5	86	141	3	MOLECULE:	DEOXYHEMOGL
61:00:00	1dxu-C	4.7	3.5	87	141	2	MOLECULE:	HEMOGLOBIN
62:00:00	4pq6-A	4.7	3.8	89	153	12	MOLECULE:	MYOGLOBIN
63:00:00	3e5i-A	4.7	3.9	88	153	11	MOLECULE:	MYOGLOBIN
64:00:00	2zsz-A	4.7	4	88	153	13	MOLECULE:	MYOGLOBIN
65:00:00	1duk-A	4.7	3.8	90	152	11	MOLECULE:	WILD-TYPE R
66:00:00	7xcf-A	4.7	3.9	90	153	11	MOLECULE:	MYOGLOBIN
67:00:00	2zss-A	4.7	4	88	153	13	MOLECULE:	MYOGLOBIN
68:00:00	2hhb-C	4.7	3.5	87	141	2	MOLECULE:	HEMOGLOBIN
69:00:00	2zt0-A	4.7	3.9	88	153	11	MOLECULE:	MYOGLOBIN
70:00:00	6d45-A	4.7	3.9	89	153	12	MOLECULE:	MYOGLOBIN
71:00:00	1dxv-C	4.7	3.5	86	141	3	MOLECULE:	HEMOGLOBIN
72:00:00	3onz-A	4.7	3.4	85	140	5	MOLECULE:	HEMOGLOBIN
73:00:00	2eku-A	4.7	3.9	88	153	13	MOLECULE:	MYOGLOBIN
74:00:00	6e02-A	4.7	4	88	153	13	MOLECULE:	MYOGLOBIN
75:00:00	2zt3-A	4.7	3.9	88	153	13	MOLECULE:	MYOGLOBIN
76:00:00	2w6y-A	4.7	3.9	88	153	11	MOLECULE:	MYOGLOBIN
77:00:00	1y0a-C	4.7	3	84	141	5	MOLECULE:	HEMOGLOBIN
78:00:00	1mnj-B	4.7	4	90	151	13	MOLECULE:	MYOGLOBIN
79:00:00	1a6g-A	4.7	3.9	88	151	11	MOLECULE:	MYOGLOBIN
80:00:00	3rjn-B	4.7	3.8	88	152	14	MOLECULE:	MYOGLOBIN
81:00:00	1mbn-A	4.7	4	91	153	11	MOLECULE:	MYOGLOBIN
82:00:00	3ecz-A	4.7	3.9	88	153	11	MOLECULE:	MYOGLOBIN
83:00:00	1myi-B	4.7	3.8	90	153	13	MOLECULE:	MYOGLOBIN
84:00:00	1y46-C	4.7	3.6	87	141	2	MOLECULE:	HEMOGLOBIN
85:00:00	2zso-A	4.7	3.9	88	153	11	MOLECULE:	MYOGLOBIN
86:00:00	1myg-A	4.7	4	91	153	13	MOLECULE:	MYOGLOBIN
87:00:00	3m39-A	4.7	3.9	89	153	11	MOLECULE:	MYOGLOBIN
88:00:00	1hga-C	4.7	3.1	85	141	4	MOLECULE:	HEMOGLOBIN
89:00:00	1gzx-C	4.7	3.1	85	141	4	MOLECULE:	HEMOGLOBIN
90:00:00	6nq5-A	4.7	3.2	86	140	3	MOLECULE:	HEMOGLOBIN
91:00:00	4qau-A	4.7	3.8	89	153	12	MOLECULE:	MYOGLOBIN
92:00:00	1hgb-A	4.7	3.1	85	141	4	MOLECULE:	HEMOGLOBIN
93:00:00	1nqp-A	4.7	3.4	86	141	2	MOLECULE:	HEMOGLOBIN
94:00:00	1y5f-C	4.7	3.5	87	141	2	MOLECULE:	HEMOGLOBIN

95:00:00	1hbb-C	4.7	3.1	85	141	4	MOLECULE:	HEMOGLOBIN
96:00:00	3e55-A	4.7	3.9	88	153	11	MOLECULE:	MYOGLOBIN
97:00:00	1yhe-C	4.7	3	84	141	5	MOLECULE:	HEMOGLOBIN
98:00:00	4n7n-K	4.7	3.8	88	141	2	MOLECULE:	HEMOGLOBIN
99:00:00	2zsx-A	4.7	3.9	88	153	11	MOLECULE:	MYOGLOBIN
0:00	1qsi-A	4.7	3.3	85	141	5	MOLECULE:	PROTEIN (HE
1:00	1yen-C	4.7	3	84	138	4	MOLECULE:	HEMOGLOBIN
2:00	1wmu-A	4.7	4.1	88	141	8	MOLECULE:	HEMOGLOBIN
3:00	1dxt-C	4.7	3.5	86	141	3	MOLECULE:	HEMOGLOBIN
4:00	1y22-C	4.7	3.1	85	141	4	MOLECULE:	HEMOGLOBIN
5:00	4mxk-A	4.7	3.8	88	153	11	MOLECULE:	MYOGLOBIN
6:00	1yca-B	4.7	4	90	153	14	MOLECULE:	MYOGLOBIN
7:00	1gli-A	4.7	3.5	86	141	3	MOLECULE:	DEOXYHEMOGL
8:00	2bli-A	4.7	4	90	153	13	MOLECULE:	MYOGLOBIN
9:00	8f9h-A	4.7	3.9	90	151	12	MOLECULE:	MYOGLOBIN
10:00	1y0w-C	4.7	3.1	85	141	4	MOLECULE:	HEMOGLOBIN
11:00	1xz4-A	4.7	3.5	87	141	3	MOLECULE:	HEMOGLOBIN
12:00	4ns2-A	4.7	3.8	88	153	14	MOLECULE:	MYOGLOBIN
13:00	1hdb-C	4.7	3.1	85	141	4	MOLECULE:	HEMOGLOBIN
14:00	5e6e-A	4.7	3.4	85	141	5	MOLECULE:	HEMOGLOBIN
15:00	4lpi-A	4.7	3.9	89	153	12	MOLECULE:	MYOGLOBIN
16:00	1v4w-A	4.7	3.4	86	143	7	MOLECULE:	HEMOGLOBIN
17:00	1k1k-A	4.7	3.4	86	141	3	MOLECULE:	HEMOGLOBIN
18:00	5wjk-A	4.7	3.9	90	153	12	MOLECULE:	MYOGLOBIN
19:00	5xkw-A	4.7	3.9	89	153	12	MOLECULE:	MYOGLOBIN
20:00	1a00-C	4.7	3	85	141	6	MOLECULE:	HEMOGLOBIN
21:00	3ecl-A	4.7	3.9	88	153	13	MOLECULE:	MYOGLOBIN
22:00	6ihx-A	4.7	3.6	87	140	5	MOLECULE:	HEMOGLOBIN
23:00	3whm-E	4.7	3.4	86	140	2	MOLECULE:	HEMOGLOBIN
24:00:00	1yma-A	4.7	4.2	90	153	14	MOLECULE:	MYOGLOBIN
25:00:00	1yih-C	4.7	3.4	86	141	3	MOLECULE:	HEMOGLOBIN
26:00:00	1y0t-C	4.7	3.5	87	141	2	MOLECULE:	HEMOGLOBIN
27:00:00	3ciu-A	4.7	3.4	86	140	5	MOLECULE:	HEMOGLOBIN
28:00:00	2hbs-C	4.7	3.1	85	141	6	MOLECULE:	HEMOGLOBIN
29:00:00	2zsy-A	4.7	3.9	88	153	11	MOLECULE:	MYOGLOBIN
30:00:00	1y7z-C	4.7	3.1	85	141	4	MOLECULE:	HEMOGLOBIN
31:00:00	1y0d-C	4.7	3.1	85	140	4	MOLECULE:	HEMOGLOBIN
32:00:00	1jpb-A	4.7	3.9	88	151	11	MOLECULE:	MYOGLOBIN
33:00:00	1g08-C	4.7	3.1	85	141	6	MOLECULE:	HEMOGLOBIN
34:00:00	1bzz-C	4.7	3.5	86	141	3	MOLECULE:	PROTEIN (HE
35:00:00	1yhr-C	4.7	3.1	85	141	4	MOLECULE:	HEMOGLOBIN
36:00:00	2hhd-C	4.7	3.3	85	141	5	MOLECULE:	HEMOGLOBIN
37:00:00	1y09-C	4.7	3.1	85	141	4	MOLECULE:	HEMOGLOBIN
38:00:00	1ydz-C	4.7	3.4	86	141	3	MOLECULE:	HEMOGLOBIN

39:00:00	5vzo-A	4.7	3.9	88	153	11	MOLECULE:	MYOGLOBIN
40:00:00	1j3z-C	4.7	3	84	141	5	MOLECULE:	HEMOGLOBIN
41:00:00	2z6t-A	4.7	3.9	88	151	11	MOLECULE:	MYOGLOBIN
42:00:00	1y4r-C	4.7	3.5	87	141	2	MOLECULE:	HEMOGLOBIN
43:00:00	1g9v-C	4.7	3.4	86	141	3	MOLECULE:	HEMOGLOBIN
44:00:00	2hhb-A	4.7	3.5	86	141	3	MOLECULE:	HEMOGLOBIN
45:00:00	1vwt-A	4.7	3	84	141	5	MOLECULE:	HEMOGLOBIN
46:00:00	1a0u-C	4.7	3.5	87	141	2	MOLECULE:	HEMOGLOBIN
47:00:00	1jp9-A	4.7	3.9	89	151	12	MOLECULE:	MYOGLOBIN
48:00:00	1a6n-A	4.7	3.9	88	151	11	MOLECULE:	MYOGLOBIN
49:00:00	5uta-A	4.7	4	88	153	13	MOLECULE:	MYOGLOBIN
50:00:00	1j3y-C	4.7	3	84	141	5	MOLECULE:	HEMOGLOBIN
51:00:00	1o1n-A	4.7	13.8	118	282	4	MOLECULE:	HEMOGLOBIN
52:00:00	1y35-C	4.7	3.5	87	141	2	MOLECULE:	HEMOGLOBIN
53:00:00	1pmb-B	4.7	3.9	90	153	13	MOLECULE:	MYOGLOBIN
54:00:00	2zsq-A	4.7	4	88	153	13	MOLECULE:	MYOGLOBIN
55:00:00	1y7d-C	4.7	3.6	87	141	2	MOLECULE:	HEMOGLOBIN
56:00:00	7cen-A	4.7	3.9	90	153	11	MOLECULE:	MYOGLOBIN
57:00:00	1dke-C	4.7	2.9	84	141	4	MOLECULE:	HEMOGLOBIN:
58:00:00	1y0d-A	4.7	3.1	85	140	4	MOLECULE:	HEMOGLOBIN
59:00:00	7slh-A	4.7	4	88	152	13	MOLECULE:	MYOGLOBIN
60:00:00	5kd1-A	4.7	4	88	153	13	MOLECULE:	MYOGLOBIN
61:00:00	1y7c-C	4.7	3.1	85	141	4	MOLECULE:	HEMOGLOBIN
62:00:00	7cez-A	4.7	3.9	90	153	12	MOLECULE:	MYOGLOBIN
63:00:00	1k0y-A	4.7	3	84	141	5	MOLECULE:	HEMOGLOBIN
64:00:00	1o1o-A	4.7	3.5	86	141	3	MOLECULE:	HEMOGLOBIN
65:00:00	1a6m-A	4.7	3.9	88	151	11	MOLECULE:	MYOGLOBIN
66:00:00	7vdn-A	4.7	3.9	88	151	11	MOLECULE:	MYOGLOBIN
67:00:00	1bbb-C	4.7	3.4	86	141	2	MOLECULE:	HEMOGLOBIN
68:00:00	1bbb-A	4.7	3.4	86	141	2	MOLECULE:	HEMOGLOBIN
69:00:00	3m38-A	4.7	4	91	153	11	MOLECULE:	MYOGLOBIN
70:00:00	1buw-A	4.7	3.4	86	141	2	MOLECULE:	PROTEIN (HE
71:00:00	1mbd-A	4.7	4	91	153	11	MOLECULE:	MYOGLOBIN
72:00:00	1mnj-A	4.7	4.1	91	153	13	MOLECULE:	MYOGLOBIN
73:00:00	6hit-E	4.7	3.4	86	142	3	MOLECULE:	HEMOGLOBIN
74:00:00	1bz0-C	4.7	3.5	87	141	2	MOLECULE:	PROTEIN (HE
75:00:00	1hsy-A	4.7	4	90	153	14	MOLECULE:	MYOGLOBIN
76:00:00	1coh-C	4.7	3.5	86	141	3	MOLECULE:	HEMOGLOBIN
77:00:00	4mxl-A	4.7	3.8	90	153	11	MOLECULE:	MYOGLOBIN
78:00:00	1myi-A	4.7	4	91	153	13	MOLECULE:	MYOGLOBIN
79:00:00	3ed9-A	4.7	3.9	88	153	13	MOLECULE:	MYOGLOBIN
80:00:00	3szk-D	4.7	3.1	85	140	2	MOLECULE:	HEMOGLOBIN
81:00:00	1y4q-C	4.7	3.5	87	141	2	MOLECULE:	HEMOGLOBIN
82:00:00	2zsp-A	4.7	3.9	88	153	11	MOLECULE:	MYOGLOBIN

83:00:00	3dut-A	4.7	3	84	141	5	MOLECULE:	HEMOGLOBIN
84:00:00	1y0c-C	4.7	3.1	85	141	4	MOLECULE:	HEMOGLOBIN
85:00:00	1ajg-A	4.7	3.9	88	153	11	MOLECULE:	MYOGLOBIN
86:00:00	6krf-A	4.7	3.8	90	153	11	MOLECULE:	MYOGLOBIN
87:00:00	8dov-G	4.7	3.4	86	140	3	MOLECULE:	HEMOGLOBIN
88:00:00	8fb0-A	4.7	3.9	88	153	11	MOLECULE:	MYOGLOBIN
89:00:00	5ut8-A	4.7	4	88	153	13	MOLECULE:	MYOGLOBIN
90:00:00	1bz0-A	4.7	3	84	141	5	MOLECULE:	PROTEIN (HE
91:00:00	1mnk-B	4.7	3.9	90	151	13	MOLECULE:	MYOGLOBIN
92:00:00	2hbs-E	4.7	3.5	87	141	2	MOLECULE:	HEMOGLOBIN
93:00:00	6nbd-A	4.7	3.4	86	140	3	MOLECULE:	HEMOGLOBIN
94:00:00	1y4p-C	4.7	3.1	85	141	4	MOLECULE:	HEMOGLOBIN
95:00:00	3oo5-A	4.7	3.4	85	140	5	MOLECULE:	HEMOGLOBIN
96:00:00	2zt1-A	4.7	3.9	88	153	11	MOLECULE:	MYOGLOBIN
97:00:00	1a01-C	4.7	3.5	86	141	3	MOLECULE:	HEMOGLOBIN
98:00:00	1hrm-A	4.7	4	90	153	14	MOLECULE:	MYOGLOBIN
99:00:00	1mbo-A	4.7	3.9	89	153	12	MOLECULE:	MYOGLOBIN
0:00	1k0y-C	4.7	3.5	87	141	2	MOLECULE:	HEMOGLOBIN
1:00	1qsi-C	4.7	3.4	85	141	5	MOLECULE:	PROTEIN (HE
2:00	1y83-C	4.7	3.5	87	141	2	MOLECULE:	HEMOGLOBIN
3:00	1bzz-A	4.7	3.5	86	141	3	MOLECULE:	PROTEIN (HE
4:00	1m6c-A	4.7	4	91	153	13	MOLECULE:	PROTEIN (MY
5:00	3eda-A	4.7	3.9	88	153	13	MOLECULE:	MYOGLOBIN
6:00	2zsr-A	4.7	3.9	88	153	13	MOLECULE:	MYOGLOBIN
7:00	6zmx-A	4.7	3.3	86	138	5	MOLECULE:	HEMOGLOBIN
8:00	3at6-A	4.7	2.9	84	141	7	MOLECULE:	ALPHAA-GLOB
9:00	1mlq-A	4.7	4	90	154	11	MOLECULE:	MYOGLOBIN
10:00	1obm-A	4.7	4	92	154	11	MOLECULE:	MYOGLOBIN
11:00	2mgi-A	4.7	4	92	154	11	MOLECULE:	MYOGLOBIN
12:00	2mga-A	4.7	4	91	154	12	MOLECULE:	MYOGLOBIN
13:00	2d5z-C	4.7	3	84	141	5	MOLECULE:	HEMOGLOBIN
14:00	3szk-A	4.7	3.4	85	140	4	MOLECULE:	HEMOGLOBIN
15:00	1abs-A	4.7	3.9	90	154	11	MOLECULE:	MYOGLOBIN
16:00	6n02-A	4.7	3.9	90	154	12	MOLECULE:	MYOGLOBIN,
17:00	8qbc-A	4.7	3.9	89	156	11	MOLECULE:	MYOGLOBIN
18:00	5jnz-A	4.7	3.1	85	142	4	MOLECULE:	ALPHA CHAIN
19:00	1moc-A	4.7	4	91	154	12	MOLECULE:	MYOGLOBIN
20:00	4n7o-K	4.7	3.4	86	141	3	MOLECULE:	HEMOGLOBIN
21:00	2mgg-A	4.7	4	90	154	11	MOLECULE:	MYOGLOBIN
22:00	8esu-A	4.7	4.1	90	154	12	MOLECULE:	MYOGLOBIN
23:00	2hbs-G	4.7	3	84	141	5	MOLECULE:	HEMOGLOBIN
24:00:00	1xy0-A	4.7	3	84	141	5	MOLECULE:	HEMOGLOBIN
25:00:00	4esa-C	4.7	3.3	85	142	6	MOLECULE:	HEMOGLOBIN
26:00:00	1g9v-A	4.7	3	84	141	5	MOLECULE:	HEMOGLOBIN

27:00:00	1mob-A	4.7	4	92	154	11	MOLECULE:	MYOGLOBIN
28:00:00	3ubv-G	4.7	3.7	85	131	11	MOLECULE:	HEMOGLOBIN-
29:00:00	4n7o-I	4.7	3.1	86	141	3	MOLECULE:	HEMOGLOBIN
30:00:00	1ufp-A	4.7	3.9	89	154	11	MOLECULE:	MYOGLOBIN
31:00:00	3qje-A	4.7	3.1	85	141	4	MOLECULE:	HEMOGLOBIN
32:00:00	1gbu-C	4.7	3.5	87	141	2	MOLECULE:	HEMOGLOBIN
33:00:00	1mgn-A	4.7	4	92	154	11	MOLECULE:	METMYOGLOBI
34:00:00	6kah-G	4.7	3.1	85	141	4	MOLECULE:	HEMOGLOBIN
35:00:00	1mtj-A	4.7	3.9	90	154	11	MOLECULE:	MYOGLOBIN
36:00:00	5ile-A	4.7	4.1	90	154	12	MOLECULE:	MYOGLOBIN
37:00:00	3ubc-A	4.7	3.8	84	131	8	MOLECULE:	HEMOGLOBIN-
38:00:00	5kdq-A	4.7	3	84	141	5	MOLECULE:	HEMOGLOBIN
39:00:00	7k4m-G	4.7	3.1	85	140	4	MOLECULE:	HEMOGLOBIN
40:00:00	1mlr-A	4.7	4	90	154	11	MOLECULE:	MYOGLOBIN
41:00:00	8qba-A	4.7	4	91	156	12	MOLECULE:	MYOGLOBIN
42:00:00	3ubv-A	4.7	3.8	84	131	8	MOLECULE:	HEMOGLOBIN-
43:00:00	3s1i-B	4.7	3.8	84	131	8	MOLECULE:	HEMOGLOBIN-
44:00:00	1moa-A	4.7	4	90	154	11	MOLECULE:	MYOGLOBIN
45:00:00	1o16-A	4.7	4	90	154	11	MOLECULE:	MYOGLOBIN
46:00:00	1j40-G	4.7	3.1	85	141	4	MOLECULE:	HEMOGLOBIN
47:00:00	1v4x-A	4.7	3.3	85	143	8	MOLECULE:	HEMOGLOBIN
48:00:00	6iyh-A	4.7	3.4	86	142	3	MOLECULE:	ALPHA CHAIN
49:00:00	1fcs-A	4.7	3.9	91	154	12	MOLECULE:	MYOGLOBIN
50:00:00	1ye0-A	4.7	2.9	83	141	5	MOLECULE:	HEMOGLOBIN
51:00:00	110m-A	4.7	3.8	89	154	12	MOLECULE:	MYOGLOBIN
52:00:00	2mbw-A	4.7	4	91	154	12	MOLECULE:	MYOGLOBIN
53:00:00	2mgm-A	4.7	4	90	154	11	MOLECULE:	MYOGLOBIN
54:00:00	1rps-C	4.7	3.1	85	141	4	MOLECULE:	HEMOGLOBIN
55:00:00	1bab-A	4.7	3	84	142	5	MOLECULE:	HEMOGLOBIN
56:00:00	1xz7-C	4.7	3	84	141	5	MOLECULE:	HEMOGLOBIN
57:00:00	4fwz-A	4.7	4	92	153	11	MOLECULE:	MYOGLOBIN
58:00:00	3bj1-C	4.7	3.4	86	142	6	MOLECULE:	HEMOGLOBIN
59:00:00	1mod-A	4.7	4	92	154	11	MOLECULE:	MYOGLOBIN
60:00:00	3bj1-A	4.7	3.4	86	142	6	MOLECULE:	HEMOGLOBIN
61:00:00	103m-A	4.7	4	91	154	12	MOLECULE:	MYOGLOBIN
62:00:00	8ess-A	4.7	3.9	90	154	11	MOLECULE:	MYOGLOBIN
63:00:00	6bwp-A	4.7	3	84	141	5	MOLECULE:	HEMOGLOBIN
64:00:00	3s1j-B	4.7	3.8	84	131	8	MOLECULE:	HEMOGLOBIN-
65:00:00	6m8f-A	4.7	4	90	154	11	MOLECULE:	MYOGLOBIN
66:00:00	1y5k-C	4.7	3.1	85	141	4	MOLECULE:	HEMOGLOBIN
67:00:00	3bj3-C	4.7	3.3	86	142	6	MOLECULE:	HEMOGLOBIN
68:00:00	2bnl-E	4.7	4	79	129	8	MOLECULE:	MODULATOR P
69:00:00	1mln-A	4.7	4	92	154	11	MOLECULE:	MYOGLOBIN
70:00:00	1xz2-A	4.7	3.1	85	141	4	MOLECULE:	HEMOGLOBIN

71:00:00	6iyi-A	4.7	3.1	85	142	4	MOLECULE:	ALPHA CHAIN
72:00:00	5wog-A	4.7	3	84	137	4	MOLECULE:	HEMOGLOBIN
73:00:00	1dxu-A	4.7	3	84	141	5	MOLECULE:	HEMOGLOBIN
74:00:00	2mgf-A	4.7	4	91	154	12	MOLECULE:	MYOGLOBIN
75:00:00	1mlj-A	4.7	4	90	154	11	MOLECULE:	MYOGLOBIN
76:00:00	8vyl-A	4.7	3.4	86	137	3	MOLECULE:	HEMOGLOBIN
77:00:00	1hba-C	4.7	3.1	85	141	4	MOLECULE:	HEMOGLOBIN
78:00:00	1bz1-C	4.7	3	84	142	5	MOLECULE:	PROTEIN (HE
79:00:00	1a3n-C	4.7	3	84	141	5	MOLECULE:	HEMOGLOBIN
80:00:00	1hbg-A	4.6	3.6	88	147	6	MOLECULE:	HEMOGLOBIN
81:00:00	6zca-H	4.6	16.6	122	771	6	MOLECULE:	PROBABLE DN
82:00:00	5x2u-G	4.6	3.5	87	140	2	MOLECULE:	HEMOGLOBIN
83:00:00	1rq4-A	4.6	3	84	141	5	MOLECULE:	HEMOGLOBIN
84:00:00	1y09-A	4.6	3.6	88	141	3	MOLECULE:	HEMOGLOBIN
85:00:00	1ut0-B	4.6	3.9	89	154	11	MOLECULE:	CYTOGLOBIN
86:00:00	1ut0-A	4.6	4.1	90	154	10	MOLECULE:	CYTOGLOBIN
87:00:00	7ddu-C	4.6	4	89	154	13	MOLECULE:	MYOGLOBIN
88:00:00	2bnl-B	4.6	4	78	128	8	MOLECULE:	MODULATOR P
89:00:00	2bnl-D	4.6	4	79	129	8	MOLECULE:	MODULATOR P
90:00:00	1kd2-A	4.6	3.1	85	141	4	MOLECULE:	HEMOGLOBIN
91:00:00	4mqi-A	4.6	3.4	85	140	5	MOLECULE:	HEMOGLOBIN
92:00:00	1xq5-C	4.6	3.4	86	142	8	MOLECULE:	HEMOGLOBIN
93:00:00	1lfv-A	4.6	3.2	85	141	4	MOLECULE:	HEMOGLOBIN
94:00:00	1urv-B	4.6	3.9	89	154	11	MOLECULE:	CYTOGLOBIN
95:00:00	1umo-B	4.6	4.2	90	154	10	MOLECULE:	CYTOGLOBIN
96:00:00	1urv-A	4.6	4.1	90	154	10	MOLECULE:	CYTOGLOBIN
97:00:00	3bj2-C	4.6	3.4	86	142	6	MOLECULE:	HEMOGLOBIN
98:00:00	8u5a-B	4.6	3.7	85	149	7	MOLECULE:	DESIGNED MY
99:00:00	1lft-A	4.6	3.5	86	141	3	MOLECULE:	HEMOGLOBIN
0:00	1fdh-A	4.6	3	85	141	4	MOLECULE:	HEMOGLOBIN
1:00	7k4m-C	4.6	3.4	86	140	2	MOLECULE:	HEMOGLOBIN
2:00	1spg-A	4.6	3.4	86	142	8	MOLECULE:	HEMOGLOBIN
3:00	1yie-A	4.6	3.4	85	141	5	MOLECULE:	HEMOGLOBIN
4:00	1y5k-A	4.6	3.1	85	141	4	MOLECULE:	HEMOGLOBIN
5:00	1hac-C	4.6	3.8	88	141	2	MOLECULE:	HEMOGLOBIN
6:00	3bcq-A	4.6	3.3	85	142	9	MOLECULE:	ALPHA-CHAIN
7:00	6nbc-C	4.6	3.4	85	140	4	MOLECULE:	HEMOGLOBIN
8:00	6lcx-G	4.6	3	85	141	5	MOLECULE:	HEMOGLOBIN
9:00	1nih-A	4.6	3.1	85	141	6	MOLECULE:	HEMOGLOBIN
10:00	1vxb-A	4.6	3.8	88	153	11	MOLECULE:	MYOGLOBIN
11:00	7uvb-A	4.6	3.4	86	141	2	MOLECULE:	HEMOGLOBIN
12:00	1gzx-A	4.6	3.1	85	141	4	MOLECULE:	HEMOGLOBIN
13:00	5e83-C	4.6	3.4	86	141	2	MOLECULE:	HEMOGLOBIN
14:00	2hbe-A	4.6	3	84	141	4	MOLECULE:	HEMOGLOBIN

15:00	2d5z-A	4.6	3.1	86	141	5	MOLECULE:	HEMOGLOBIN
16:00	3d17-A	4.6	3.5	86	141	3	MOLECULE:	HEMOGLOBIN
17:00	3cy5-A	4.6	3.5	87	141	3	MOLECULE:	HEMOGLOBIN
18:00	1j3y-G	4.6	2.9	84	141	6	MOLECULE:	HEMOGLOBIN
19:00	3qjb-A	4.6	3.4	85	141	5	MOLECULE:	HEMOGLOBIN
20:00	5urc-C	4.6	3.4	86	141	2	MOLECULE:	HEMOGLOBIN
21:00	6nbd-C	4.6	3.4	86	140	3	MOLECULE:	HEMOGLOBIN
22:00	1cls-A	4.6	3	84	141	5	MOLECULE:	HEMOGLOBIN
23:00	1y4f-A	4.6	3	84	141	5	MOLECULE:	HEMOGLOBIN
24:00:00	1hab-A	4.6	3.1	85	141	4	MOLECULE:	HEMOGLOBIN
25:00:00	2yrs-M	4.6	3.4	86	141	3	MOLECULE:	HEMOGLOBIN
26:00:00	4n7p-G	4.6	3.1	85	141	4	MOLECULE:	HEMOGLOBIN
27:00:00	1y4b-A	4.6	3.1	86	141	5	MOLECULE:	HEMOGLOBIN
28:00:00	6bwp-C	4.6	2.9	84	141	6	MOLECULE:	HEMOGLOBIN
29:00:00	6di4-C	4.6	3.4	86	141	2	MOLECULE:	HEMOGLOBIN
30:00:00	1kd2-C	4.6	3.1	85	141	6	MOLECULE:	HEMOGLOBIN
31:00:00	1rq4-C	4.6	3.6	87	141	2	MOLECULE:	HEMOGLOBIN
32:00:00	6ii1-C	4.6	3.5	86	138	6	MOLECULE:	HEMOGLOBIN
33:00:00	7uvb-C	4.6	3.4	85	141	4	MOLECULE:	HEMOGLOBIN
34:00:00	3ic0-A	4.6	3.4	86	141	2	MOLECULE:	HEMOGLOBIN
35:00:00	1ygd-A	4.6	3.1	85	141	4	MOLECULE:	HEMOGLOBIN
36:00:00	1g09-A	4.6	3.6	88	141	5	MOLECULE:	HEMOGLOBIN
37:00:00	1yeo-A	4.6	3.1	85	141	4	MOLECULE:	HEMOGLOBIN
38:00:00	7uf6-C	4.6	3.4	86	141	2	MOLECULE:	HEMOGLOBIN
39:00:00	6z4t-A	4.6	4	90	154	12	MOLECULE:	MYOGLOBIN
40:00:00	2dn2-C	4.6	2.9	84	141	6	MOLECULE:	HEMOGLOBIN
41:00:00	1a3o-C	4.6	3	85	141	6	MOLECULE:	HEMOGLOBIN
42:00:00	1j7y-A	4.6	2.9	84	141	6	MOLECULE:	HEMOGLOBIN
43:00:00	1yeq-C	4.6	3	84	141	4	MOLECULE:	HEMOGLOBIN
44:00:00	1rps-A	4.6	3.1	86	141	5	MOLECULE:	HEMOGLOBIN
45:00:00	7ehx-A	4.6	3.9	90	153	11	MOLECULE:	MYOGLOBIN
46:00:00	1g0a-A	4.6	3.4	86	141	5	MOLECULE:	HEMOGLOBIN
47:00:00	8dov-A	4.6	3.5	86	140	3	MOLECULE:	HEMOGLOBIN
48:00:00	1u7r-A	4.6	3.8	89	153	12	MOLECULE:	MYOGLOBIN
49:00:00	2hbs-A	4.6	3.1	85	141	4	MOLECULE:	HEMOGLOBIN
50:00:00	8fdm-C	4.6	3.4	85	139	5	MOLECULE:	HEMOGLOBIN
51:00:00	7jy3-C	4.6	3.4	85	141	4	MOLECULE:	HEMOGLOBIN
52:00:00	6lcw-C	4.6	3.1	85	141	4	MOLECULE:	HEMOGLOBIN
53:00:00	1y22-A	4.6	3.6	87	141	2	MOLECULE:	HEMOGLOBIN
54:00:00	2hco-A	4.6	3.3	85	141	5	MOLECULE:	HEMOGLOBIN
55:00:00	1xzv-A	4.6	2.9	83	141	5	MOLECULE:	HEMOGLOBIN
56:00:00	7pch-C	4.6	3.8	88	141	2	MOLECULE:	HEMOGLOBIN
57:00:00	7uf7-C	4.6	3.4	86	141	2	MOLECULE:	HEMOGLOBIN
58:00:00	6hbw-C	4.6	2.9	84	141	6	MOLECULE:	PROTEIN (HE

59:00:00	1y85-A	4.6	3	84	141	5	MOLECULE:	HEMOGLOBIN
60:00:00	1xxt-A	4.6	3.1	85	141	4	MOLECULE:	HEMOGLOBIN
61:00:00	3vrf-A	4.6	3.2	86	141	2	MOLECULE:	HEMOGLOBIN
62:00:00	5ufj-A	4.6	3.5	86	141	2	MOLECULE:	HEMOGLOBIN
63:00:00	7jy0-C	4.6	3.4	86	141	2	MOLECULE:	HEMOGLOBIN
64:00:00	3hen-A	4.6	3.9	89	153	16	MOLECULE:	MYOGLOBIN
65:00:00	1aby-A	4.6	9.5	110	283	5	MOLECULE:	HEMOGLOBIN
66:00:00	1mni-A	4.6	4	90	153	13	MOLECULE:	MYOGLOBIN
67:00:00	6hk2-A	4.6	3.4	85	141	5	MOLECULE:	HEMOGLOBIN
68:00:00	1yhe-A	4.6	3.6	87	141	2	MOLECULE:	HEMOGLOBIN
69:00:00	1xz5-A	4.6	3.1	85	141	4	MOLECULE:	HEMOGLOBIN
70:00:00	1yff-C	4.6	3.5	86	141	2	MOLECULE:	HEMOGLOBIN
71:00:00	1yvq-A	4.6	3	84	141	4	MOLECULE:	HEMOGLOBIN
72:00:00	2qss-C	4.6	3.4	85	141	6	MOLECULE:	HEMOGLOBIN
73:00:00	6ihx-C	4.6	3.4	86	140	6	MOLECULE:	HEMOGLOBIN
74:00:00	1yg5-A	4.6	3.6	87	141	2	MOLECULE:	HEMOGLOBIN
75:00:00	1r1y-A	4.6	3.4	87	141	5	MOLECULE:	HEMOGLOBIN
76:00:00	5zeo-A	4.6	4	89	153	11	MOLECULE:	MYOGLOBIN
77:00:00	1jy7-C	4.6	3.5	86	141	2	MOLECULE:	HEMOGLOBIN
78:00:00	2evk-A	4.6	3.9	89	153	12	MOLECULE:	MYOGLOBIN
79:00:00	3pi8-C	4.6	3.5	85	141	5	MOLECULE:	HEMOGLOBIN
80:00:00	1xxt-C	4.6	3.1	85	141	4	MOLECULE:	HEMOGLOBIN
81:00:00	6kao-A	4.6	3.6	87	141	2	MOLECULE:	HEMOGLOBIN
82:00:00	3v2z-A	4.6	4	88	152	15	MOLECULE:	MYOGLOBIN
83:00:00	1yie-C	4.6	3	84	141	4	MOLECULE:	HEMOGLOBIN
84:00:00	6xd9-A	4.6	3.5	86	141	2	MOLECULE:	HEMOGLOBIN
85:00:00	1ygd-C	4.6	3.1	85	141	4	MOLECULE:	HEMOGLOBIN
86:00:00	6kai-C	4.6	2.9	83	141	5	MOLECULE:	HEMOGLOBIN
87:00:00	1mdn-A	4.6	4	90	153	13	MOLECULE:	PROTEIN (MY
88:00:00	4mqh-A	4.6	3.1	85	139	4	MOLECULE:	HEMOGLOBIN
89:00:00	1y45-C	4.6	3.1	85	141	4	MOLECULE:	HEMOGLOBIN
90:00:00	1y5j-A	4.6	3.6	87	141	2	MOLECULE:	HEMOGLOBIN
91:00:00	1hda-C	4.6	3.1	85	141	6	MOLECULE:	HEMOGLOBIN
92:00:00	6kai-G	4.6	3.1	86	141	5	MOLECULE:	HEMOGLOBIN
93:00:00	6bnr-C	4.6	3.4	86	141	2	MOLECULE:	HEMOGLOBIN
94:00:00	1mwd-A	4.6	3.9	90	153	13	MOLECULE:	PROTEIN (MY
95:00:00	1y31-C	4.6	3.5	87	141	2	MOLECULE:	HEMOGLOBIN
96:00:00	5hlx-A	4.6	4	90	153	11	MOLECULE:	MYOGLOBIN
97:00:00	5oj9-A	4.6	3.9	89	154	11	MOLECULE:	MYOGLOBIN
98:00:00	1mwd-B	4.6	4	90	153	14	MOLECULE:	PROTEIN (MY
99:00:00	1y7g-A	4.6	3.1	85	141	4	MOLECULE:	HEMOGLOBIN
0:00	1a0z-A	4.6	2.9	84	141	6	MOLECULE:	HEMOGLOBIN
1:00	1r1x-A	4.6	3.8	87	141	3	MOLECULE:	HEMOGLOBIN
2:00	4mqk-C	4.6	3.5	86	141	3	MOLECULE:	HEMOGLOBIN

3:00	3nmm-A	4.6	3	84	141	5	MOLECULE:	HEMOGLOBIN
4:00	2ef2-A	4.6	3.5	88	153	11	MOLECULE:	MYOGLOBIN
5:00	4hhb-C	4.6	3.5	86	141	3	MOLECULE:	HEMOGLOBIN
6:00	6kah-C	4.6	3	84	141	4	MOLECULE:	HEMOGLOBIN
7:00	1y7d-A	4.6	3.6	87	141	2	MOLECULE:	HEMOGLOBIN
8:00	3heo-A	4.6	3.8	88	153	14	MOLECULE:	MYOGLOBIN
9:00	1j3z-G	4.6	2.9	84	141	6	MOLECULE:	HEMOGLOBIN
10:00	8fdl-A	4.6	2.9	84	141	6	MOLECULE:	HEMOGLOBIN
11:00	2yrs-C	4.6	3.3	86	141	6	MOLECULE:	HEMOGLOBIN
12:00	1j7w-C	4.6	3	84	141	5	MOLECULE:	HEMOGLOBIN
13:00	4mqg-A	4.6	3.9	88	141	2	MOLECULE:	HEMOGLOBIN
14:00	7ud7-A	4.6	3	85	141	5	MOLECULE:	HEMOGLOBIN
15:00	1j3z-E	4.6	3	84	141	5	MOLECULE:	HEMOGLOBIN
16:00	3nl7-A	4.6	3.6	87	141	2	MOLECULE:	HEMOGLOBIN
17:00	6lcx-C	4.6	3.1	86	141	5	MOLECULE:	HEMOGLOBIN
18:00	6kae-G	4.6	3	85	141	5	MOLECULE:	HEMOGLOBIN
19:00	6l5v-A	4.6	3.5	86	141	3	MOLECULE:	HEMOGLOBIN
20:00	1xz5-C	4.6	3.6	87	141	2	MOLECULE:	HEMOGLOBIN
21:00	5ucu-A	4.6	3.4	85	140	5	MOLECULE:	HEMOGLOBIN
22:00	7dy3-E	4.6	3	85	141	5	MOLECULE:	HEMOGLOBIN
23:00	3dut-C	4.6	3	84	141	5	MOLECULE:	HEMOGLOBIN
24:00:00	3s48-D	4.6	3.5	87	140	2	MOLECULE:	IRON-REGULA
25:00:00	3gdj-A	4.6	3.5	87	141	6	MOLECULE:	HEMOGLOBIN
26:00:00	3qje-C	4.6	3.1	85	141	6	MOLECULE:	HEMOGLOBIN
27:00:00	1yeu-A	4.6	3.1	85	141	4	MOLECULE:	HEMOGLOBIN
28:00:00	6hbw-A	4.6	3	84	141	5	MOLECULE:	PROTEIN (HE
29:00:00	6kat-A	4.6	3.4	86	141	2	MOLECULE:	HEMOGLOBIN
30:00:00	3p5q-A	4.6	3.4	86	139	6	MOLECULE:	HEMOGLOBIN
31:00:00	1r1y-C	4.6	3.3	85	141	5	MOLECULE:	HEMOGLOBIN
32:00:00	1rq3-A	4.6	3.5	86	141	3	MOLECULE:	HEMOGLOBIN
33:00:00	4mqc-A	4.6	3.1	84	141	5	MOLECULE:	HEMOGLOBIN
34:00:00	8f9n-A	4.6	4.1	88	153	13	MOLECULE:	MYOGLOBIN
35:00:00	5sw7-A	4.6	3.4	85	142	5	MOLECULE:	HEMOGLOBIN
36:00:00	6f18-A	4.6	3.8	88	154	11	MOLECULE:	MYOGLOBIN
37:00:00	1hgc-A	4.6	3.4	86	141	3	MOLECULE:	HEMOGLOBIN
38:00:00	1y83-A	4.6	3.1	85	141	4	MOLECULE:	HEMOGLOBIN
39:00:00	8fdk-A	4.6	3.6	88	141	3	MOLECULE:	HEMOGLOBIN
40:00:00	7jjq-C	4.6	3.6	86	139	3	MOLECULE:	HEMOGLOBIN
41:00:00	1ljw-A	4.6	3.5	87	141	2	MOLECULE:	HEMOGLOBIN
42:00:00	2z6n-A	4.6	4.1	88	141	8	MOLECULE:	HEMOGLOBIN
43:00:00	3vrg-A	4.6	3.2	85	141	2	MOLECULE:	HEMOGLOBIN
44:00:00	8fdk-C	4.6	3.6	86	141	3	MOLECULE:	HEMOGLOBIN
45:00:00	1y4r-A	4.6	3.6	87	141	2	MOLECULE:	HEMOGLOBIN
46:00:00	7cue-C	4.6	3.5	86	141	3	MOLECULE:	HEMOGLOBIN

47:00:00	1y31-A	4.6	3.6	87	141	2	MOLECULE:	HEMOGLOBIN
48:00:00	1bvd-A	4.6	4	88	153	11	MOLECULE:	APOMYOGLOBI
49:00:00	4hhb-A	4.6	3	84	141	5	MOLECULE:	HEMOGLOBIN
50:00:00	1j7s-A	4.6	3.1	85	141	4	MOLECULE:	HEMOGLOBIN
51:00:00	1y5f-A	4.6	3.6	87	141	2	MOLECULE:	HEMOGLOBIN
52:00:00	3gys-G	4.6	3	84	138	6	MOLECULE:	HEMOGLOBIN
53:00:00	1yvt-A	4.6	3.5	86	141	3	MOLECULE:	HEMOGLOBIN
54:00:00	6l5x-A	4.6	3.4	86	141	2	MOLECULE:	HEMOGLOBIN
55:00:00	6kae-C	4.6	3	84	141	5	MOLECULE:	HEMOGLOBIN
56:00:00	3qjd-A	4.6	2.9	84	141	6	MOLECULE:	HEMOGLOBIN
57:00:00	6kaq-A	4.6	3.5	87	141	2	MOLECULE:	HEMOGLOBIN
58:00:00	4fwx-A	4.6	3.9	90	153	11	MOLECULE:	MYOGLOBIN
59:00:00	1ygf-C	4.6	3.6	87	141	2	MOLECULE:	HEMOGLOBIN
60:00:00	1y2z-C	4.6	3.6	87	141	2	MOLECULE:	HEMOGLOBIN
61:00:00	2w72-C	4.6	2.9	84	141	4	MOLECULE:	HUMAN HEMOG
62:00:00	5vzp-A	4.6	3.9	88	153	11	MOLECULE:	MYOGLOBIN
63:00:00	3vre-C	4.6	3.1	85	141	2	MOLECULE:	HEMOGLOBIN
64:00:00	1xye-A	4.6	3.1	85	141	4	MOLECULE:	HEMOGLOBIN
65:00:00	6l5y-A	4.6	3.4	86	141	2	MOLECULE:	HEMOGLOBIN
66:00:00	8fdn-C	4.6	3.5	87	141	5	MOLECULE:	HEMOGLOBIN
67:00:00	5ufj-C	4.6	3.4	86	141	2	MOLECULE:	HEMOGLOBIN
68:00:00	4x0i-A	4.6	3	83	141	5	MOLECULE:	HEMOGLOBIN
69:00:00	1o1i-A	4.6	3.1	84	141	4	MOLECULE:	HEMOGLOBIN
70:00:00	5b85-A	4.6	3.9	88	153	11	MOLECULE:	MYOGLOBIN
71:00:00	1y4v-A	4.6	3.6	87	141	2	MOLECULE:	HEMOGLOBIN
72:00:00	3oo4-A	4.6	3.8	88	141	5	MOLECULE:	HEMOGLOBIN
73:00:00	1ycb-A	4.6	4.1	91	153	13	MOLECULE:	MYOGLOBIN
74:00:00	5x2t-G	4.6	3.9	88	140	2	MOLECULE:	HEMOGLOBIN
75:00:00	1j3y-E	4.6	3	84	141	5	MOLECULE:	HEMOGLOBIN
76:00:00	6f19-A	4.6	3.8	88	154	11	MOLECULE:	MYOGLOBIN
77:00:00	1mno-B	4.6	4	90	153	14	MOLECULE:	PROTEIN (MY
78:00:00	1cls-C	4.6	2.9	84	141	4	MOLECULE:	HEMOGLOBIN
79:00:00	1uiw-A	4.6	2.9	83	141	5	MOLECULE:	HEMOGLOBIN
80:00:00	1y7z-A	4.6	3.1	85	141	4	MOLECULE:	HEMOGLOBIN
81:00:00	6g5a-A	4.6	3.7	88	153	13	MOLECULE:	MYOGLOBIN
82:00:00	1a0u-A	4.6	3.1	85	141	4	MOLECULE:	HEMOGLOBIN
83:00:00	1hbb-A	4.6	3.6	87	141	2	MOLECULE:	HEMOGLOBIN
84:00:00	4ni0-A	4.6	3.6	87	141	2	MOLECULE:	HEMOGLOBIN
85:00:00	5ker-E	4.6	3.4	86	140	5	MOLECULE:	ALPHA-GLOBI
86:00:00	2hhe-A	4.6	3.1	86	141	5	MOLECULE:	HEMOGLOBIN
87:00:00	1y2z-A	4.6	3	84	141	4	MOLECULE:	HEMOGLOBIN
88:00:00	2qsp-C	4.6	3.6	87	141	5	MOLECULE:	HEMOGLOBIN
89:00:00	1ye2-A	4.6	3	84	141	5	MOLECULE:	HEMOGLOBIN
90:00:00	6kye-I	4.6	3.8	87	141	3	MOLECULE:	HEMOGLOBIN

91:00:00	6ka9-A	4.6	3.4	85	141	5	MOLECULE:	HEMOGLOBIN
92:00:00	1yeu-C	4.6	3	84	141	4	MOLECULE:	HEMOGLOBIN
93:00:00	1yff-G	4.6	3.4	86	141	2	MOLECULE:	HEMOGLOBIN
94:00:00	3nmm-C	4.6	2.9	84	141	6	MOLECULE:	HEMOGLOBIN
95:00:00	5jdo-E	4.6	3.4	86	140	3	MOLECULE:	HAPTOGLOBIN
96:00:00	1y4g-A	4.6	3	84	141	5	MOLECULE:	HEMOGLOBIN
97:00:00	8fdm-A	4.6	3.4	85	139	5	MOLECULE:	HEMOGLOBIN
98:00:00	1yih-A	4.6	3.4	85	141	5	MOLECULE:	HEMOGLOBIN
99:00:00	1lfl-A	4.6	3	85	141	5	MOLECULE:	HEMOGLOBIN
0:00	1ye1-C	4.6	3.5	87	141	2	MOLECULE:	HEMOGLOBIN
1:00	1yg5-C	4.6	3.1	85	141	4	MOLECULE:	HEMOGLOBIN
2:00	1y35-A	4.6	3.1	85	141	4	MOLECULE:	HEMOGLOBIN
3:00	8a61-T	4.6	23.1	112	650	12	MOLECULE:	ANAPHASE-PR
4:00	1sdk-C	4.6	3.4	85	141	5	MOLECULE:	HEMOGLOBIN
5:00	1m6m-B	4.6	4	90	153	14	MOLECULE:	PROTEIN (MY
6:00	6kav-A	4.6	3.4	86	141	2	MOLECULE:	HEMOGLOBIN
7:00	7pcq-C	4.6	3.9	88	141	2	MOLECULE:	HEMOGLOBIN
8:00	4n8t-A	4.6	3.4	85	140	5	MOLECULE:	HEMOGLOBIN
9:00	2dn3-A	4.6	3.6	87	141	2	MOLECULE:	HEMOGLOBIN
10:00	1nih-C	4.6	3.1	85	141	4	MOLECULE:	HEMOGLOBIN
11:00	4ni1-A	4.6	3.4	86	141	6	MOLECULE:	HEMOGLOBIN
12:00	1hab-C	4.6	3.4	86	141	3	MOLECULE:	HEMOGLOBIN
13:00	1xzu-A	4.6	3.1	85	141	4	MOLECULE:	HEMOGLOBIN
14:00	2w72-A	4.6	3.4	85	141	5	MOLECULE:	HUMAN HEMOG
15:00	1ycb-B	4.6	4	90	153	14	MOLECULE:	MYOGLOBIN
16:00	1hda-A	4.6	3.1	85	141	6	MOLECULE:	HEMOGLOBIN
17:00	1qi8-A	4.6	3.1	85	141	4	MOLECULE:	HEMOGLOBIN
18:00	1y85-C	4.6	3.6	87	141	2	MOLECULE:	HEMOGLOBIN
19:00	2d60-C	4.6	2.9	84	141	6	MOLECULE:	HEMOGLOBIN
20:00	1y0w-A	4.6	3	84	141	5	MOLECULE:	HEMOGLOBIN
21:00	6kye-E	4.6	3.4	86	140	3	MOLECULE:	HEMOGLOBIN
22:00	1y0c-A	4.6	3	85	141	5	MOLECULE:	HEMOGLOBIN
23:00	4l7y-C	4.6	3	84	141	5	MOLECULE:	HEMOGLOBIN
24:00:00	1fsx-A	4.6	3.1	85	141	6	MOLECULE:	HEMOGLOBIN
25:00:00	6e04-A	4.6	4	88	153	13	MOLECULE:	MYOGLOBIN
26:00:00	7dy4-E	4.6	3.1	84	141	5	MOLECULE:	HEMOGLOBIN
27:00:00	6kap-A	4.6	3.5	87	141	2	MOLECULE:	HEMOGLOBIN
28:00:00	6lcw-G	4.6	3	85	141	5	MOLECULE:	HEMOGLOBIN
29:00:00	7jy3-A	4.6	3.4	86	141	2	MOLECULE:	HEMOGLOBIN
30:00:00	1j41-C	4.6	2.9	83	141	5	MOLECULE:	HEMOGLOBIN
31:00:00	1fhj-C	4.6	3.5	86	141	5	MOLECULE:	HEMOGLOBIN
32:00:00	1fdh-B	4.6	3	85	141	4	MOLECULE:	HEMOGLOBIN
33:00:00	1yh9-A	4.6	3.1	85	141	4	MOLECULE:	HEMOGLOBIN
34:00:00	2yrs-I	4.6	3	85	141	5	MOLECULE:	HEMOGLOBIN

35:00:00	2w6v-A	4.6	2.9	84	141	6	MOLECULE:	HEMOGLOBIN
36:00:00	1yeq-A	4.6	3	84	141	4	MOLECULE:	HEMOGLOBIN
37:00:00	1hdb-A	4.6	3.5	86	141	3	MOLECULE:	HEMOGLOBIN
38:00:00	5kdq-C	4.6	3.1	85	141	6	MOLECULE:	HEMOGLOBIN
39:00:00	7pch-A	4.6	3.9	88	141	2	MOLECULE:	HEMOGLOBIN
40:00:00	1y0a-A	4.6	2.9	83	141	5	MOLECULE:	HEMOGLOBIN
41:00:00	2d6c-A	4.6	4	88	153	13	MOLECULE:	MYOGLOBIN
42:00:00	1mwc-B	4.6	3.9	90	153	13	MOLECULE:	PROTEIN (MY
43:00:00	6ka9-C	4.6	3.3	85	141	5	MOLECULE:	HEMOGLOBIN
44:00:00	6kye-C	4.6	3.6	87	140	2	MOLECULE:	HEMOGLOBIN
45:00:00	1pmb-A	4.6	3.9	90	153	13	MOLECULE:	MYOGLOBIN
46:00:00	7dy4-C	4.6	3.5	86	141	3	MOLECULE:	HEMOGLOBIN
47:00:00	1rqa-C	4.6	3.4	86	141	2	MOLECULE:	HEMOGLOBIN
48:00:00	1mdn-B	4.6	4	90	153	14	MOLECULE:	PROTEIN (MY
49:00:00	1m9p-A	4.6	3.4	85	141	4	MOLECULE:	HEMOGLOBIN
50:00:00	7vde-C	4.6	3.5	87	141	2	MOLECULE:	HEMOGLOBIN
51:00:00	5hu6-A	4.6	3	83	141	5	MOLECULE:	HEMOGLOBIN
52:00:00	3o89-A	4.6	4	89	153	12	MOLECULE:	MYOGLOBIN
53:00:00	6rp5-A	4.6	3.1	84	142	6	MOLECULE:	HEMOGLOBIN
54:00:00	1ye1-A	4.6	3.6	87	141	2	MOLECULE:	HEMOGLOBIN
55:00:00	1j7y-C	4.6	2.9	84	141	6	MOLECULE:	HEMOGLOBIN
56:00:00	1j41-G	4.6	3	85	141	6	MOLECULE:	HEMOGLOBIN
57:00:00	1y7c-A	4.6	3.1	85	141	4	MOLECULE:	HEMOGLOBIN
58:00:00	1y4p-A	4.6	3.1	85	141	4	MOLECULE:	HEMOGLOBIN
59:00:00	6bwu-A	4.6	3.6	87	140	5	MOLECULE:	HEMOGLOBIN
60:00:00	6kau-A	4.6	3.4	86	141	2	MOLECULE:	HEMOGLOBIN
61:00:00	1y4q-A	4.6	3.1	85	141	4	MOLECULE:	HEMOGLOBIN
62:00:00	6kas-A	4.6	3.4	86	141	2	MOLECULE:	HEMOGLOBIN
63:00:00	1g08-A	4.6	3.5	86	141	5	MOLECULE:	HEMOGLOBIN
64:00:00	3ba2-A	4.6	4	88	152	15	MOLECULE:	MYOGLOBIN
65:00:00	5u3i-A	4.6	3.4	86	141	2	MOLECULE:	HEMOGLOBIN
66:00:00	3hep-A	4.6	3.8	88	153	14	MOLECULE:	MYOGLOBIN
67:00:00	8fdn-A	4.6	3.1	84	141	5	MOLECULE:	HEMOGLOBIN
68:00:00	6kae-A	4.6	3	84	141	5	MOLECULE:	HEMOGLOBIN
69:00:00	1y46-A	4.6	3.6	87	141	2	MOLECULE:	HEMOGLOBIN
70:00:00	3d7o-A	4.6	3.6	86	141	3	MOLECULE:	HEMOGLOBIN
71:00:00	1gbu-A	4.6	3	84	141	5	MOLECULE:	HEMOGLOBIN
72:00:00	8j4l-A	4.6	3.9	88	153	11	MOLECULE:	MYOGLOBIN
73:00:00	1y0t-A	4.6	3.1	85	141	4	MOLECULE:	HEMOGLOBIN
74:00:00	1hba-A	4.6	3.1	85	141	4	MOLECULE:	HEMOGLOBIN
75:00:00	2evp-A	4.6	3.8	90	153	11	MOLECULE:	MYOGLOBIN
76:00:00	1j7s-C	4.6	3	84	141	5	MOLECULE:	HEMOGLOBIN
77:00:00	2hhd-A	4.6	2.9	84	141	6	MOLECULE:	HEMOGLOBIN
78:00:00	1irc-A	4.6	3.8	90	152	11	MOLECULE:	MYOGLOBIN (

79:00:00	8dov-C	4.6	3.5	87	140	5	MOLECULE:	HEMOGLOBIN
80:00:00	1hgb-C	4.6	3.1	86	141	5	MOLECULE:	HEMOGLOBIN
81:00:00	1xq5-A	4.6	3.4	86	142	8	MOLECULE:	HEMOGLOBIN
82:00:00	7jxz-A	4.6	3.4	86	141	2	MOLECULE:	HEMOGLOBIN
83:00:00	6zmy-A	4.6	3.1	85	139	5	MOLECULE:	HEMOGLOBIN
84:00:00	1umo-A	4.6	4.2	89	154	10	MOLECULE:	CYTOGLOBIN
85:00:00	3a59-C	4.6	3.4	86	141	6	MOLECULE:	HEMOGLOBIN
86:00:00	7k4m-A	4.6	3.1	85	141	4	MOLECULE:	HEMOGLOBIN
87:00:00	3odq-C	4.6	3.4	86	141	3	MOLECULE:	HEMOGLOBIN
88:00:00	4rom-C	4.6	3	84	141	5	MOLECULE:	HEMOGLOBIN
89:00:00	1qxd-A	4.6	3	84	141	4	MOLECULE:	HEMOGLOBIN
90:00:00	3hxn-A	4.6	3.4	86	141	3	MOLECULE:	HEMOGLOBIN
91:00:00	7ddu-A	4.6	3.8	89	154	12	MOLECULE:	MYOGLOBIN
92:00:00	6xdt-C	4.6	3	84	141	4	MOLECULE:	HEMOGLOBIN
93:00:00	3wtg-A	4.6	3	85	138	11	MOLECULE:	HEMOGLOBIN
94:00:00	3bj3-A	4.6	3.3	86	142	6	MOLECULE:	HEMOGLOBIN
95:00:00	4ij2-A	4.6	3.5	86	138	3	MOLECULE:	HEMOGLOBIN
96:00:00	5ojb-A	4.6	3.9	91	154	13	MOLECULE:	MYOGLOBIN
97:00:00	7vw4-A	4.6	4	89	152	12	MOLECULE:	MYOGLOBIN
98:00:00	2aa1-C	4.6	3.3	85	142	7	MOLECULE:	HEMOGLOBIN
99:00:00	5oja-A	4.6	4.1	92	152	12	MOLECULE:	MYOGLOBIN
0:00	8dov-E	4.6	3.5	86	138	3	MOLECULE:	HEMOGLOBIN
1:00	2yrs-A	4.6	3.4	86	141	3	MOLECULE:	HEMOGLOBIN
2:00	1hga-A	4.6	3.4	86	141	3	MOLECULE:	HEMOGLOBIN
3:00	1yzi-A	4.6	3.5	87	141	2	MOLECULE:	HEMOGLOBIN
4:00	6l5w-A	4.6	3.5	86	141	3	MOLECULE:	HEMOGLOBIN
5:00	4esa-A	4.6	3.3	85	142	6	MOLECULE:	HEMOGLOBIN
6:00	2w6v-C	4.6	3.5	87	141	5	MOLECULE:	HEMOGLOBIN
7:00	5jgg-A	4.6	3.4	86	142	3	MOLECULE:	ALPHA CHAIN
8:00	2aa1-A	4.6	3.3	85	142	7	MOLECULE:	HEMOGLOBIN
9:00	3ic2-A	4.6	3	84	141	4	MOLECULE:	HEMOGLOBIN
10:00	8fdl-C	4.6	3.1	85	141	6	MOLECULE:	HEMOGLOBIN
11:00	3r5i-A	4.6	3	84	141	4	MOLECULE:	HEMOGLOBIN
12:00	1hac-A	4.6	3.4	86	141	3	MOLECULE:	HEMOGLOBIN
13:00	5ee4-E	4.6	3	84	141	4	MOLECULE:	HPUA
14:00	7pcq-A	4.6	3.5	87	141	2	MOLECULE:	HEMOGLOBIN
15:00	4ij2-C	4.6	3.6	87	138	2	MOLECULE:	HEMOGLOBIN
16:00	6hit-G	4.6	3.1	84	142	5	MOLECULE:	HEMOGLOBIN
17:00	1rqa-A	4.6	2.9	83	141	5	MOLECULE:	HEMOGLOBIN
18:00	5yci-A	4.6	3.9	89	154	16	MOLECULE:	ANCESTRAL M
19:00	6ii1-A	4.6	3.2	85	137	6	MOLECULE:	HEMOGLOBIN
20:00	2ri4-I	4.6	3	84	137	5	MOLECULE:	HEMOGLOBIN
21:00	4l7y-A	4.6	3.1	85	141	4	MOLECULE:	HEMOGLOBIN
22:00	3s66-A	4.6	3.5	86	139	3	MOLECULE:	HEMOGLOBIN

23:00	6l5y-C	4.6	3	84	141	4	MOLECULE:	HEMOGLOBIN
24:00:00	5x2s-G	4.6	3.5	86	140	3	MOLECULE:	HEMOGLOBIN
25:00:00	7dy3-G	4.6	3	84	141	4	MOLECULE:	HEMOGLOBIN
26:00:00	5woh-A	4.6	3	84	137	4	MOLECULE:	HEMOGLOBIN
27:00:00	1qxe-A	4.6	3	84	141	4	MOLECULE:	HEMOGLOBIN
28:00:00	6hit-C	4.6	3.4	86	142	3	MOLECULE:	HEMOGLOBIN
29:00:00	4b3w-A	4.6	4.2	90	154	10	MOLECULE:	CYTOGLOBIN
30:00:00	5e29-A	4.6	3.1	84	141	5	MOLECULE:	HEMOGLOBIN
31:00:00	6xe7-A	4.6	3.5	86	141	2	MOLECULE:	HEMOGLOBIN
32:00:00	1a01-A	4.6	3	85	141	6	MOLECULE:	HEMOGLOBIN
33:00:00	3bcq-C	4.6	3.3	85	142	9	MOLECULE:	ALPHA-CHAIN
34:00:00	1v4u-A	4.6	3.3	85	143	8	MOLECULE:	HEMOGLOBIN
35:00:00	6kae-E	4.6	3.1	85	141	4	MOLECULE:	HEMOGLOBIN
36:00:00	1uiw-C	4.6	2.9	83	141	5	MOLECULE:	HEMOGLOBIN
37:00:00	6kas-C	4.6	3.2	86	141	2	MOLECULE:	HEMOGLOBIN
38:00:00	1lfl-R	4.6	3.4	86	141	3	MOLECULE:	HEMOGLOBIN
39:00:00	3d1k-A	4.6	3.4	85	142	7	MOLECULE:	HEMOGLOBIN
40:00:00	6bnr-A	4.6	3	84	141	4	MOLECULE:	HEMOGLOBIN
41:00:00	7uf6-A	4.6	3	84	141	4	MOLECULE:	HEMOGLOBIN
42:00:00	6ka9-E	4.6	3.4	85	141	5	MOLECULE:	HEMOGLOBIN
43:00:00	5x2s-K	4.6	3.5	86	140	3	MOLECULE:	HEMOGLOBIN
44:00:00	7dy4-G	4.6	3.1	85	141	4	MOLECULE:	HEMOGLOBIN
45:00:00	6kau-C	4.6	3	84	141	4	MOLECULE:	HEMOGLOBIN
46:00:00	1j40-C	4.6	3	84	141	5	MOLECULE:	HEMOGLOBIN
47:00:00	4n7n-G	4.6	3.4	87	141	5	MOLECULE:	HEMOGLOBIN
48:00:00	1bz1-A	4.6	3	84	142	5	MOLECULE:	PROTEIN (HE
49:00:00	1fhj-A	4.6	3.4	86	141	6	MOLECULE:	HEMOGLOBIN
50:00:00	6jp1-A	4.6	3.9	89	151	12	MOLECULE:	MYOGLOBIN
51:00:00	1o1k-C	4.6	3.6	87	140	2	MOLECULE:	HEMOGLOBIN
52:00:00	3qjc-A	4.6	3.5	86	141	3	MOLECULE:	HEMOGLOBIN
53:00:00	1bab-C	4.6	3.3	85	142	5	MOLECULE:	HEMOGLOBIN
54:00:00	5x2r-G	4.6	3.5	86	140	3	MOLECULE:	HEMOGLOBIN
55:00:00	2vwa-A	4.5	2.8	71	99	13	MOLECULE:	PUTATIVE UN
56:00:00	1d8u-A	4.5	5.5	95	165	9	MOLECULE:	NON-SYMBIOT
57:00:00	5ziq-A	4.5	4	89	155	7	MOLECULE:	GLOBIN PROT
58:00:00	1yhu-U	4.5	3.3	84	145	11	MOLECULE:	HEMOGLOBIN
59:00:00	4mqj-D	4.5	3.4	85	145	7	MOLECULE:	HEMOGLOBIN
60:00:00	3gou-C	4.5	3.8	88	141	5	MOLECULE:	HEMOGLOBIN
61:00:00	1iwh-A	4.5	3.2	85	141	4	MOLECULE:	HEMOGLOBIN
62:00:00	1qpw-A	4.5	3.1	85	141	5	MOLECULE:	PORCINE HEM
63:00:00	5x2s-I	4.5	3.6	87	140	2	MOLECULE:	HEMOGLOBIN
64:00:00	1ux9-A	4.5	4.1	89	154	10	MOLECULE:	CYTOGLOBIN
65:00:00	1i3d-A	4.5	3.4	85	146	7	MOLECULE:	HEMOGLOBIN
66:00:00	3gqr-E	4.5	3.6	87	141	5	MOLECULE:	HEMOGLOBIN

67:00:00	1hbr-B	4.5	3.5	85	143	9	MOLECULE:	PROTEIN (HE
68:00:00	3mkb-A	4.5	3.4	86	140	6	MOLECULE:	HEMOGLOBIN
69:00:00	1jy7-W	4.5	3.5	86	141	2	MOLECULE:	HEMOGLOBIN
70:00:00	4mqk-B	4.5	3.5	86	145	6	MOLECULE:	HEMOGLOBIN
71:00:00	1ury-A	4.5	4.1	89	154	10	MOLECULE:	CYTOGLOBIN
72:00:00	1jy7-U	4.5	3.3	86	141	2	MOLECULE:	HEMOGLOBIN
73:00:00	1j40-E	4.5	3.1	86	141	5	MOLECULE:	HEMOGLOBIN
74:00:00	1lfq-A	4.5	3.1	84	141	5	MOLECULE:	HEMOGLOBIN
75:00:00	1nej-A	4.5	3.6	86	141	3	MOLECULE:	HEMOGLOBIN
76:00:00	3pi9-A	4.5	3.5	86	141	5	MOLECULE:	HEMOGLOBIN
77:00:00	1lfy-A	4.5	3.6	88	141	3	MOLECULE:	HEMOGLOBIN
78:00:00	2zlu-A	4.5	3.6	87	140	2	MOLECULE:	HEMOGLOBIN
79:00:00	2qls-A	4.5	3.5	87	141	6	MOLECULE:	HEMOGLOBIN
80:00:00	2mhb-A	4.5	3.4	86	141	3	MOLECULE:	HEMOGLOBIN
81:00:00	1hco-A	4.5	3.1	86	141	5	MOLECULE:	HEMOGLOBIN
82:00:00	1cmy-A	4.5	3.5	86	141	3	MOLECULE:	HEMOGLOBIN
83:00:00	1hho-A	4.5	3.6	87	141	2	MOLECULE:	HEMOGLOBIN
84:00:00	6hk2-C	4.5	4	89	141	2	MOLECULE:	HEMOGLOBIN
85:00:00	6lcw-A	4.5	3.1	85	141	6	MOLECULE:	HEMOGLOBIN
86:00:00	1ns9-A	4.5	3.6	87	141	2	MOLECULE:	HEMOGLOBIN
87:00:00	5e29-C	4.5	3.1	85	141	6	MOLECULE:	HEMOGLOBIN
88:00:00	7jy1-A	4.5	3	85	141	5	MOLECULE:	HEMOGLOBIN
89:00:00	1g0b-A	4.5	3.2	85	141	4	MOLECULE:	HEMOGLOBIN
90:00:00	6hal-A	4.5	3.6	87	139	2	MOLECULE:	HEMOGLOBIN
91:00:00	3pia-C	4.5	3.5	85	141	5	MOLECULE:	HEMOGLOBIN
92:00:00	7jy0-A	4.5	3.4	86	141	2	MOLECULE:	HEMOGLOBIN
93:00:00	1ns6-A	4.5	3.2	86	141	3	MOLECULE:	HEMOGLOBIN
94:00:00	3a0g-A	4.5	3.5	86	139	6	MOLECULE:	HEMOGLOBIN
95:00:00	1uiw-G	4.5	3.1	85	141	6	MOLECULE:	HEMOGLOBIN
96:00:00	6lcx-A	4.5	3.1	85	141	6	MOLECULE:	HEMOGLOBIN
97:00:00	1uiw-E	4.5	2.9	83	141	5	MOLECULE:	HEMOGLOBIN
98:00:00	2qsp-A	4.5	3.5	85	141	5	MOLECULE:	HEMOGLOBIN
99:00:00	1j41-A	4.5	3.2	86	141	5	MOLECULE:	HEMOGLOBIN
0:00	3d1a-C	4.5	3.9	88	141	3	MOLECULE:	HEMOGLOBIN
1:00	3pel-A	4.5	3.5	87	141	5	MOLECULE:	HEMOGLOBIN
2:00	1mko-C	4.5	3.1	85	141	4	MOLECULE:	HEMOGLOBIN
3:00	1a9w-C	4.5	3.1	85	141	2	MOLECULE:	HEMOGLOBIN
4:00	6r2o-C	4.5	3.4	86	141	2	MOLECULE:	HEMOGLOBIN
5:00	5c6e-A	4.5	3.2	85	141	4	MOLECULE:	HEMOGLOBIN
6:00	5ee4-C	4.5	3	85	141	5	MOLECULE:	HPUA
7:00	3vre-A	4.5	3.5	88	141	2	MOLECULE:	HEMOGLOBIN
8:00	1out-A	4.5	3	84	142	10	MOLECULE:	HEMOGLOBIN
9:00	3d1a-A	4.5	4	89	141	3	MOLECULE:	HEMOGLOBIN
10:00	5ksi-C	4.5	2.9	83	141	5	MOLECULE:	HEMOGLOBIN

11:00	3gys-C	4.5	3.5	87	139	6	MOLECULE:	HEMOGLOBIN
12:00	3whm-A	4.5	3	84	140	4	MOLECULE:	HEMOGLOBIN
13:00	3pi8-A	4.5	3.4	85	141	5	MOLECULE:	HEMOGLOBIN
14:00	2qls-C	4.5	3.5	87	141	5	MOLECULE:	HEMOGLOBIN
15:00	6kai-A	4.5	3.1	86	141	5	MOLECULE:	HEMOGLOBIN
16:00	2qss-A	4.5	3.4	85	141	5	MOLECULE:	HEMOGLOBIN
17:00	1fn3-A	4.5	3.6	88	141	2	MOLECULE:	HEMOGLOBIN
18:00	1m9p-C	4.5	3.5	87	141	3	MOLECULE:	HEMOGLOBIN
19:00	3wcp-A	4.5	3.2	86	141	5	MOLECULE:	HEMOGLOBIN
20:00	5ksj-C	4.5	3	84	141	4	MOLECULE:	HEMOGLOBIN
21:00	3cy5-C	4.5	3.5	87	141	5	MOLECULE:	HEMOGLOBIN
22:00	4mqj-C	4.5	3.5	86	141	3	MOLECULE:	HEMOGLOBIN
23:00	4mqj-A	4.5	3.5	86	141	3	MOLECULE:	HEMOGLOBIN
24:00:00	1b86-A	4.5	3.4	86	141	6	MOLECULE:	PROTEIN (HE
25:00:00	1cmy-C	4.5	3.1	84	141	5	MOLECULE:	HEMOGLOBIN
26:00:00	3eu1-C	4.5	3.5	87	141	5	MOLECULE:	HEMOGLOBIN
27:00:00	3hyu-A	4.5	3.5	86	141	6	MOLECULE:	HEMOGLOBIN
28:00:00	6kye-G	4.5	3.8	88	140	3	MOLECULE:	HEMOGLOBIN
29:00:00	4f4o-G	4.5	3.4	86	141	5	MOLECULE:	HEMOGLOBIN
30:00:00	7vde-A	4.5	3.5	87	141	2	MOLECULE:	HEMOGLOBIN
31:00:00	3r5i-C	4.5	3.5	85	141	4	MOLECULE:	HEMOGLOBIN
32:00:00	2in4-A	4.5	3.9	88	153	14	MOLECULE:	MYOGLOBIN
33:00:00	3s65-C	4.5	3.4	86	141	2	MOLECULE:	HEMOGLOBIN
34:00:00	4mqk-E	4.5	3.6	87	138	3	MOLECULE:	HEMOGLOBIN
35:00:00	7jjq-A	4.5	3.4	85	140	5	MOLECULE:	HEMOGLOBIN
36:00:00	1g09-C	4.5	3.5	85	141	5	MOLECULE:	HEMOGLOBIN
37:00:00	1ouu-A	4.5	3.4	85	142	9	MOLECULE:	HEMOGLOBIN
38:00:00	1ibe-A	4.5	3.4	86	141	5	MOLECULE:	HEMOGLOBIN
39:00:00	1sdl-A	4.5	3.6	88	141	3	MOLECULE:	HEMOGLOBIN
40:00:00	1dke-A	4.5	3	84	141	6	MOLECULE:	HEMOGLOBIN:
41:00:00	5e83-A	4.5	3.5	86	141	2	MOLECULE:	HEMOGLOBIN
42:00:00	5x2u-K	4.5	2.9	84	140	5	MOLECULE:	HEMOGLOBIN
43:00:00	1a3n-A	4.5	2.9	84	141	6	MOLECULE:	HEMOGLOBIN
44:00:00	3pi9-C	4.5	3.5	85	141	5	MOLECULE:	HEMOGLOBIN
45:00:00	7dy4-A	4.5	3.1	85	141	6	MOLECULE:	HEMOGLOBIN
46:00:00	6tb2-A	4.5	3.4	85	141	5	MOLECULE:	HEMOGLOBIN
47:00:00	2rao-A	4.5	3.5	85	141	5	MOLECULE:	HEMOGLOBIN
48:00:00	1ydz-A	4.5	3.4	86	141	3	MOLECULE:	HEMOGLOBIN
49:00:00	4wjg-Z	4.5	3.5	86	141	2	MOLECULE:	HEMOGLOBIN
50:00:00	6ka9-G	4.5	2.9	84	141	6	MOLECULE:	HEMOGLOBIN
51:00:00	4f4o-A	4.5	3.5	86	141	3	MOLECULE:	HEMOGLOBIN
52:00:00	5ksj-A	4.5	3.1	85	141	4	MOLECULE:	HEMOGLOBIN
53:00:00	4wjg-U	4.5	3.6	87	141	2	MOLECULE:	HEMOGLOBIN
54:00:00	1shr-C	4.5	3.5	86	141	2	MOLECULE:	HEMOGLOBIN

55:00:00	1j3y-A	4.5	2.9	84	141	6	MOLECULE:	HEMOGLOBIN
56:00:00	1fn3-C	4.5	3.6	88	141	2	MOLECULE:	HEMOGLOBIN
57:00:00	1g0a-C	4.5	3.5	86	141	5	MOLECULE:	HEMOGLOBIN
58:00:00	3dhr-E	4.5	3.5	86	141	6	MOLECULE:	HEMOGLOBIN
59:00:00	7ud8-C	4.5	3.5	85	141	4	MOLECULE:	HEMOGLOBIN
60:00:00	7ud7-C	4.5	3.1	85	141	6	MOLECULE:	HEMOGLOBIN
61:00:00	7dy3-C	4.5	3	84	141	6	MOLECULE:	HEMOGLOBIN
62:00:00	4mqk-A	4.5	3.5	86	141	3	MOLECULE:	HEMOGLOBIN
63:00:00	2qu0-C	4.5	3.6	87	141	3	MOLECULE:	HEMOGLOBIN
64:00:00	1thb-A	4.5	3.1	85	141	6	MOLECULE:	HEMOGLOBIN
65:00:00	1y4b-C	4.5	3.4	87	141	3	MOLECULE:	HEMOGLOBIN
66:00:00	4wjg-K	4.5	3.6	87	141	2	MOLECULE:	HEMOGLOBIN
67:00:00	5ni1-C	4.5	3.5	87	141	2	MOLECULE:	HEMOGLOBIN
68:00:00	3gqp-C	4.5	3.5	87	141	6	MOLECULE:	HEMOGLOBIN
69:00:00	1y8k-C	4.5	3	84	141	4	MOLECULE:	HEMOGLOBIN
70:00:00	1v4x-C	4.5	3.3	85	143	8	MOLECULE:	HEMOGLOBIN
71:00:00	7k4m-I	4.5	3	84	140	4	MOLECULE:	HEMOGLOBIN
72:00:00	4wjg-F	4.5	3.6	87	141	2	MOLECULE:	HEMOGLOBIN
73:00:00	1j3z-A	4.5	2.9	84	141	6	MOLECULE:	HEMOGLOBIN
74:00:00	1yff-A	4.5	3.5	87	141	2	MOLECULE:	HEMOGLOBIN
75:00:00	7jxz-C	4.5	3.1	85	141	2	MOLECULE:	HEMOGLOBIN
76:00:00	1a9w-A	4.5	3.5	86	141	2	MOLECULE:	HEMOGLOBIN
77:00:00	2d60-A	4.5	2.9	84	141	6	MOLECULE:	HEMOGLOBIN
78:00:00	1si4-A	4.5	3.4	86	141	2	MOLECULE:	HEMOGLOBIN
79:00:00	4f4o-J	4.5	3.5	86	141	3	MOLECULE:	HEMOGLOBIN
80:00:00	4yu4-A	4.5	3.5	86	141	5	MOLECULE:	HEMOGLOBIN
81:00:00	3wcp-C	4.5	3.2	86	141	5	MOLECULE:	HEMOGLOBIN
82:00:00	3gys-E	4.5	3	84	138	6	MOLECULE:	HEMOGLOBIN
83:00:00	6hal-C	4.5	3.7	87	139	2	MOLECULE:	HEMOGLOBIN
84:00:00	4wjg-A	4.5	3.6	87	141	2	MOLECULE:	HEMOGLOBIN
85:00:00	6lcx-E	4.5	3.1	86	141	5	MOLECULE:	HEMOGLOBIN
86:00:00	1j40-A	4.5	3.2	86	141	5	MOLECULE:	HEMOGLOBIN
87:00:00	8pur-A	4.5	3.6	87	140	3	MOLECULE:	HEMOGLOBIN,
88:00:00	6kah-A	4.5	3.1	86	141	5	MOLECULE:	HEMOGLOBIN
89:00:00	6zmx-C	4.5	3.4	85	138	7	MOLECULE:	HEMOGLOBIN
90:00:00	4f4o-D	4.5	3.4	86	141	5	MOLECULE:	HEMOGLOBIN
91:00:00	1jy7-R	4.5	3.5	86	141	2	MOLECULE:	HEMOGLOBIN
92:00:00	1thb-C	4.5	3.6	88	141	3	MOLECULE:	HEMOGLOBIN
93:00:00	1ygf-A	4.5	3.1	86	141	5	MOLECULE:	HEMOGLOBIN
94:00:00	4fc3-A	4.5	3.4	86	140	2	MOLECULE:	HEMOGLOBIN
95:00:00	1y8i-C	4.5	3	84	141	4	MOLECULE:	HEMOGLOBIN
96:00:00	5ksi-A	4.5	3	84	141	6	MOLECULE:	HEMOGLOBIN
97:00:00	7jy1-C	4.5	3	84	141	6	MOLECULE:	HEMOGLOBIN
98:00:00	6z4r-A	4.5	4	89	154	11	MOLECULE:	MYOGLOBIN

99:00:00	3odq-A	4.5	3.4	86	141	3	MOLECULE:	HEMOGLOBIN
0:00	1jy7-A	4.5	3	83	141	5	MOLECULE:	HEMOGLOBIN
1:00	3b75-S	4.5	3.5	86	141	2	MOLECULE:	HEMOGLOBIN
2:00	4n7o-E	4.5	3.5	86	141	3	MOLECULE:	HEMOGLOBIN
3:00	6nbc-A	4.5	3	83	140	5	MOLECULE:	HEMOGLOBIN
4:00	3qjd-C	4.5	3.5	87	141	5	MOLECULE:	HEMOGLOBIN
5:00	2eb9-A	4.5	3.4	84	150	13	MOLECULE:	MYOGLOBIN
6:00	2h8f-A	4.5	2.8	83	142	7	MOLECULE:	HEMOGLOBIN
7:00	1v4u-C	4.5	3.1	85	143	7	MOLECULE:	HEMOGLOBIN
8:00	1y01-B	4.5	3.2	85	126	4	MOLECULE:	ALPHA-HEMOG
9:00	7qu4-B	4.5	3	83	141	5	MOLECULE:	HEMOGLOBIN
10:00	4odc-A	4.5	3.4	85	142	7	MOLECULE:	HEMOGLOBIN
11:00	1nej-C	4.5	3	83	141	5	MOLECULE:	HEMOGLOBIN
12:00	3nfe-A	4.5	3	84	142	7	MOLECULE:	HEMOGLOBIN
13:00	1hbh-C	4.5	2.8	83	142	7	MOLECULE:	HEMOGLOBIN
14:00	1mko-A	4.5	3.5	86	141	3	MOLECULE:	HEMOGLOBIN
15:00	3gkv-A	4.5	3.4	85	142	7	MOLECULE:	HEMOGLOBIN
16:00	2peg-A	4.5	3.4	85	142	7	MOLECULE:	HEMOGLOBIN
17:00	4n7p-E	4.5	3.4	86	141	3	MOLECULE:	HEMOGLOBIN
18:00	2h8d-A	4.5	3.3	85	142	7	MOLECULE:	HEMOGLOBIN
19:00	3gqg-A	4.5	3.3	85	142	7	MOLECULE:	HEMOGLOBIN
20:00	5ni1-A	4.5	3.5	87	141	2	MOLECULE:	HEMOGLOBIN
21:00	5lfg-A	4.5	3.4	86	142	6	MOLECULE:	HEMOGLOBIN
22:00	7qu4-A	4.5	3	84	142	4	MOLECULE:	HEMOGLOBIN
23:00	5woh-C	4.5	3.4	86	137	3	MOLECULE:	HEMOGLOBIN
24:00:00	5x2t-K	4.5	3.2	85	140	4	MOLECULE:	HEMOGLOBIN
25:00:00	2dn1-A	4.5	3.5	85	140	5	MOLECULE:	HEMOGLOBIN
26:00:00	1la6-A	4.5	3.4	85	142	7	MOLECULE:	HEMOGLOBIN
27:00:00	1t1n-A	4.5	3.3	85	142	7	MOLECULE:	PROTEIN (HE
28:00:00	3s48-C	4.5	3.4	86	138	3	MOLECULE:	IRON-REGULA
29:00:00	3gqg-C	4.5	3.3	85	142	7	MOLECULE:	HEMOGLOBIN
30:00:00	2h8d-C	4.5	3.3	85	142	7	MOLECULE:	HEMOGLOBIN
31:00:00	1pbx-A	4.5	3.3	85	142	7	MOLECULE:	HEMOGLOBIN
32:00:00	1hbh-A	4.5	2.8	83	142	7	MOLECULE:	HEMOGLOBIN
33:00:00	1o1o-C	4.5	3.5	86	141	5	MOLECULE:	HEMOGLOBIN
34:00:00	1ouu-C	4.5	3.4	86	142	9	MOLECULE:	HEMOGLOBIN
35:00:00	1o1k-A	4.5	3	84	140	6	MOLECULE:	HEMOGLOBIN
36:00:00	4mqk-H	4.5	3.4	85	145	7	MOLECULE:	HEMOGLOBIN
37:00:00	4mqk-D	4.5	3.5	86	145	6	MOLECULE:	HEMOGLOBIN
38:00:00	3ng6-C	4.5	2.8	83	142	7	MOLECULE:	HEMOGLOBIN
39:00:00	5x2t-I	4.5	3.2	85	140	4	MOLECULE:	HEMOGLOBIN
40:00:00	5x2u-C	4.5	3.5	86	140	3	MOLECULE:	HEMOGLOBIN
41:00:00	5wog-B	4.5	3.4	85	137	5	MOLECULE:	HEMOGLOBIN
42:00:00	3ng6-A	4.5	3.3	85	142	7	MOLECULE:	HEMOGLOBIN

43:00:00	1tu9-A	4.4	4.2	83	131	8	MOLECULE:	HYPOTHETICA
44:00:00	5hv1-A	4.4	4.4	93	847	8	MOLECULE:	PHOSPHOENOL
45:00:00	4hrr-A	4.4	3.5	86	149	3	MOLECULE:	GLOBIN-2 A
46:00:00	6p5b-A	4.4	4.7	93	388	2	MOLECULE:	MAVC
47:00:00	6bb5-A	4.4	3.4	86	139	5	MOLECULE:	HEMOGLOBIN
48:00:00	3pt8-A	4.4	3.8	85	151	6	MOLECULE:	HEMOGLOBIN
49:00:00	3mkb-C	4.4	3.4	85	140	6	MOLECULE:	HEMOGLOBIN
50:00:00	2olp-B	4.4	4	85	151	5	MOLECULE:	HEMOGLOBIN
51:00:00	3b75-G	4.4	4	89	141	2	MOLECULE:	HEMOGLOBIN
52:00:00	1y8h-C	4.4	3.5	85	141	4	MOLECULE:	HEMOGLOBIN
53:00:00	1hbr-D	4.4	3.5	85	146	9	MOLECULE:	PROTEIN (HE
54:00:00	4iro-C	4.4	3.3	85	142	7	MOLECULE:	HEMOGLOBIN
55:00:00	1lfz-A	4.4	3.4	86	141	3	MOLECULE:	HEMOGLOBIN
56:00:00	2dhb-A	4.4	3	83	141	4	MOLECULE:	HEMOGLOBIN
57:00:00	3s65-A	4.4	3.5	85	141	4	MOLECULE:	HEMOGLOBIN
58:00:00	7qu4-G	4.4	3.5	84	143	7	MOLECULE:	HEMOGLOBIN
59:00:00	4mqj-F	4.4	3.5	86	146	7	MOLECULE:	HEMOGLOBIN
60:00:00	5vmm-C	4.4	3.6	88	141	2	MOLECULE:	HEMOGLOBIN
61:00:00	4mqk-G	4.4	4	89	140	2	MOLECULE:	HEMOGLOBIN
62:00:00	3hf4-E	4.4	3.4	86	141	6	MOLECULE:	HEMOGLOBIN
63:00:00	1o1p-D	4.4	3.6	86	146	6	MOLECULE:	HEMOGLOBIN
64:00:00	1yev-B	4.4	3.5	85	146	7	MOLECULE:	HEMOGLOBIN
65:00:00	1a4f-A	4.4	3.7	86	141	8	MOLECULE:	HEMOGLOBIN
66:00:00	1yeo-B	4.4	3.5	86	146	7	MOLECULE:	HEMOGLOBIN
67:00:00	5x2r-C	4.4	3	84	140	4	MOLECULE:	HEMOGLOBIN
68:00:00	1i3e-A	4.4	3.5	86	146	7	MOLECULE:	HEMOGLOBIN
69:00:00	2d5x-A	4.4	3.5	86	141	3	MOLECULE:	HEMOGLOBIN
70:00:00	3wtg-B	4.4	3.4	83	146	10	MOLECULE:	HEMOGLOBIN
71:00:00	3b75-E	4.4	3.8	88	141	3	MOLECULE:	HEMOGLOBIN
72:00:00	3gqr-G	4.4	3.1	85	141	7	MOLECULE:	HEMOGLOBIN
73:00:00	3gou-A	4.4	3.7	88	141	5	MOLECULE:	HEMOGLOBIN
74:00:00	1y8i-A	4.4	3	84	141	4	MOLECULE:	HEMOGLOBIN
75:00:00	4yu3-A	4.4	3.5	86	141	5	MOLECULE:	HEMOGLOBIN
76:00:00	3hf4-A	4.4	3.4	86	141	6	MOLECULE:	HEMOGLOBIN
77:00:00	4wjg-P	4.4	3.5	86	141	2	MOLECULE:	HEMOGLOBIN
78:00:00	5ker-C	4.4	3.5	86	140	5	MOLECULE:	ALPHA-GLOBI
79:00:00	3gqr-C	4.4	3.3	87	141	6	MOLECULE:	HEMOGLOBIN
80:00:00	4x0l-A	4.4	3.5	86	141	3	MOLECULE:	HEMOGLOBIN
81:00:00	1qpw-C	4.4	3.6	87	141	3	MOLECULE:	PORCINE HEM
82:00:00	1v9q-A	4.4	4.8	80	154	11	MOLECULE:	MYOGLOBIN
83:00:00	2b7h-A	4.4	3.5	87	141	7	MOLECULE:	HEMOGLOBIN
84:00:00	3eok-A	4.4	3	84	141	6	MOLECULE:	HEMOGLOBIN
85:00:00	8puq-A	4.4	3.5	86	139	2	MOLECULE:	HEMOGLOBIN
86:00:00	1a3o-A	4.4	2.9	83	141	6	MOLECULE:	HEMOGLOBIN

87:00:00	6kah-E	4.4	3.1	83	141	4	MOLECULE:	HEMOGLOBIN
88:00:00	6sva-A	4.4	3.2	85	139	4	MOLECULE:	HEMOGLOBIN
89:00:00	1y8k-A	4.4	3	84	141	4	MOLECULE:	HEMOGLOBIN
90:00:00	2zlv-A	4.4	3.1	84	141	4	MOLECULE:	HEMOGLOBIN
91:00:00	3wr1-A	4.4	3.5	87	141	9	MOLECULE:	HEMOGLOBIN
92:00:00	4mqj-E	4.4	3.9	87	141	2	MOLECULE:	HEMOGLOBIN
93:00:00	1s0h-A	4.4	3.4	86	141	5	MOLECULE:	HEMOGLOBIN
94:00:00	7cue-A	4.4	3.6	86	141	2	MOLECULE:	HEMOGLOBIN
95:00:00	4mqj-G	4.4	3.5	86	141	3	MOLECULE:	HEMOGLOBIN
96:00:00	2rao-C	4.4	3.5	86	141	5	MOLECULE:	HEMOGLOBIN
97:00:00	1qxe-C	4.4	3.4	85	141	4	MOLECULE:	HEMOGLOBIN
98:00:00	1ird-A	4.4	3.6	88	141	3	MOLECULE:	HEMOGLOBIN
99:00:00	2qmb-A	4.4	4.4	88	141	7	MOLECULE:	HEMOGLOBIN
0:00	2qu0-A	4.4	3.6	88	141	3	MOLECULE:	HEMOGLOBIN
1:00	3ic0-C	4.4	3.5	85	141	4	MOLECULE:	HEMOGLOBIN
2:00	6r2o-A	4.4	3	84	141	4	MOLECULE:	HEMOGLOBIN
3:00	4yu4-C	4.4	3.5	86	141	5	MOLECULE:	HEMOGLOBIN
4:00	1hv4-G	4.4	3	83	141	6	MOLECULE:	HEMOGLOBIN
5:00	2zlt-A	4.4	3.5	86	141	2	MOLECULE:	HEMOGLOBIN
6:00	3mjp-A	4.4	3.4	86	141	5	MOLECULE:	HEMOGLOBIN
7:00	3ciu-C	4.4	3.5	85	141	5	MOLECULE:	HEMOGLOBIN
8:00	1hbs-E	4.4	2.9	84	141	4	MOLECULE:	HEMOGLOBIN
9:00	3d4x-A	4.4	3.1	85	141	6	MOLECULE:	HEMOGLOBIN
10:00	1shr-A	4.4	3.5	86	141	2	MOLECULE:	HEMOGLOBIN
11:00	3ic2-C	4.4	3.5	85	141	4	MOLECULE:	HEMOGLOBIN
12:00	3a59-G	4.4	3.6	87	141	5	MOLECULE:	HEMOGLOBIN
13:00	1qxd-C	4.4	3	83	141	5	MOLECULE:	HEMOGLOBIN
14:00	2b7h-C	4.4	3.5	87	141	7	MOLECULE:	HEMOGLOBIN
15:00	3d4x-C	4.4	3.1	84	141	6	MOLECULE:	HEMOGLOBIN
16:00	7dy3-A	4.4	3	84	141	6	MOLECULE:	HEMOGLOBIN
17:00	3fh9-A	4.4	3.6	87	141	5	MOLECULE:	HEMOGLOBIN
18:00	1c40-A	4.4	4.1	87	141	8	MOLECULE:	PROTEIN (HE
19:00	5u3i-C	4.4	3.4	84	140	4	MOLECULE:	HEMOGLOBIN
20:00	3gys-A	4.4	3.6	87	138	5	MOLECULE:	HEMOGLOBIN
21:00	4h2l-A	4.4	3.5	87	141	8	MOLECULE:	ALPHA-GLOBI
22:00	3kmf-A	4.4	3.1	85	141	6	MOLECULE:	HEMOGLOBIN
23:00	2ohb-A	4.4	4.5	93	154	8	MOLECULE:	MYOGLOBIN
24:00:00	2mgj-A	4.4	4.5	93	154	8	MOLECULE:	MYOGLOBIN
25:00:00	3pi1-B	4.4	3.9	86	151	5	MOLECULE:	HEMOGLOBIN
26:00:00	2h8f-C	4.4	3.3	85	142	7	MOLECULE:	HEMOGLOBIN
27:00:00	1ouu-B	4.4	3.6	85	146	7	MOLECULE:	HEMOGLOBIN
28:00:00	3nfe-C	4.4	3	84	142	7	MOLECULE:	HEMOGLOBIN
29:00:00	4g51-A	4.4	3.3	85	142	7	MOLECULE:	HEMOGLOBIN
30:00:00	6zmy-D	4.4	3.5	85	146	9	MOLECULE:	HEMOGLOBIN

31:00:00	7qu4-H	4.4	3.5	85	144	6	MOLECULE:	HEMOGLOBIN
32:00:00	6zmx-D	4.4	3.5	85	146	9	MOLECULE:	HEMOGLOBIN
33:00:00	1j7s-D	4.4	3.3	84	146	7	MOLECULE:	HEMOGLOBIN
34:00:00	6zmy-C	4.4	3.3	84	137	6	MOLECULE:	HEMOGLOBIN
35:00:00	2g0s-A	4.4	4.5	93	154	8	MOLECULE:	MYOGLOBIN
36:00:00	5x2t-E	4.4	3.6	86	140	3	MOLECULE:	HEMOGLOBIN
37:00:00	1mz0-A	4.4	4.5	93	154	8	MOLECULE:	MYOGLOBIN
38:00:00	2dc3-B	4.4	4.2	98	168	9	MOLECULE:	CYTOGLOBIN
39:00:00	1faw-D	4.4	3.5	85	146	11	MOLECULE:	HEMOGLOBIN
40:00:00	1s5x-A	4.4	3.5	85	142	7	MOLECULE:	HEMOGLOBIN
41:00:00	1cio-A	4.4	4.5	93	154	8	MOLECULE:	PROTEIN (MY
42:00:00	1ye2-D	4.4	3.5	86	146	7	MOLECULE:	HEMOGLOBIN
43:00:00	6zmy-B	4.4	3.5	85	146	9	MOLECULE:	HEMOGLOBIN
44:00:00	1y4b-D	4.4	3.2	85	146	7	MOLECULE:	HEMOGLOBIN
45:00:00	1s5y-A	4.4	3.5	85	142	7	MOLECULE:	HEMOGLOBIN
46:00:00	1c40-B	4.4	3.5	85	146	11	MOLECULE:	PROTEIN (HE
47:00:00	5vmm-A	4.4	3.6	87	139	3	MOLECULE:	HEMOGLOBIN
48:00:00	1yeu-B	4.4	3.4	84	146	7	MOLECULE:	HEMOGLOBIN
49:00:00	1out-B	4.4	3.8	87	146	7	MOLECULE:	HEMOGLOBIN
50:00:00	7ud7-D	4.4	3.5	86	146	7	MOLECULE:	HEMOGLOBIN
51:00:00	6zmx-B	4.4	3.5	85	146	9	MOLECULE:	HEMOGLOBIN
52:00:00	5lfg-C	4.4	3.4	87	142	6	MOLECULE:	HEMOGLOBIN
53:00:00	1j7s-B	4.4	3.3	84	146	7	MOLECULE:	HEMOGLOBIN
54:00:00	1ouu-D	4.4	3.6	85	146	7	MOLECULE:	HEMOGLOBIN
55:00:00	5x2u-E	4.4	3.2	85	140	4	MOLECULE:	HEMOGLOBIN
56:00:00	5x2u-I	4.4	3.1	85	140	5	MOLECULE:	HEMOGLOBIN
57:00:00	1a01-B	4.4	3.6	86	146	7	MOLECULE:	HEMOGLOBIN
58:00:00	1rqa-B	4.4	3.5	86	146	7	MOLECULE:	HEMOGLOBIN
59:00:00	4hrt-E	4.4	3.4	85	149	4	MOLECULE:	GLOBIN-2 A
60:00:00	1faw-B	4.4	3.5	85	146	11	MOLECULE:	HEMOGLOBIN
61:00:00	1myz-A	4.4	4.5	93	154	8	MOLECULE:	MYOGLOBIN
62:00:00	1y8w-A	4.4	4.3	87	141	3	MOLECULE:	HEMOGLOBIN
63:00:00	1ch7-A	4.4	4.4	93	154	8	MOLECULE:	PROTEIN (MY
64:00:00	1o1p-B	4.4	3.3	84	146	7	MOLECULE:	HEMOGLOBIN
65:00:00	1dxu-B	4.4	3.3	84	146	7	MOLECULE:	HEMOGLOBIN
66:00:00	1a00-D	4.4	3.2	84	146	7	MOLECULE:	HEMOGLOBIN
67:00:00	1cik-A	4.4	4.4	91	154	11	MOLECULE:	PROTEIN (MY
68:00:00	5x2r-A	4.4	3.6	87	140	2	MOLECULE:	HEMOGLOBIN
69:00:00	1y4g-B	4.4	3.5	86	146	7	MOLECULE:	HEMOGLOBIN
70:00:00	8fef-I	4.3	6.6	101	253	9	MOLECULE:	VIRULENCE F
71:00:00	6myo-D	4.3	8.1	83	102	10	MOLECULE:	SUCCINATE D
72:00:00	3sxk-A	4.3	3.9	96	140	5	MOLECULE:	TRANSCRIPTI
73:00:00	7tn2-W	4.3	19.7	95	895	6	MOLECULE:	HISTONE H3
74:00:00	6vxm-A	4.3	10.4	88	277	6	MOLECULE:	MECHANOSENS

75:00:00	1o1n-D	4.3	3.3	85	146	6	MOLECULE:	HEMOGLOBIN
76:00:00	3qm8-A	4.3	4.2	84	145	7	MOLECULE:	MYOGLOBIN
77:00:00	4n7n-I	4.3	3.6	88	141	3	MOLECULE:	HEMOGLOBIN
78:00:00	2hbs-B	4.3	3.4	84	146	6	MOLECULE:	HEMOGLOBIN
79:00:00	1y8h-A	4.3	3.5	85	141	4	MOLECULE:	HEMOGLOBIN
80:00:00	5me2-A	4.3	3.5	85	140	4	MOLECULE:	HEMOGLOBIN
81:00:00	2hbs-F	4.3	4.2	86	146	6	MOLECULE:	HEMOGLOBIN
82:00:00	1yeu-D	4.3	3.7	87	146	6	MOLECULE:	HEMOGLOBIN
83:00:00	1a9w-F	4.3	3.5	85	145	7	MOLECULE:	HEMOGLOBIN
84:00:00	2olp-A	4.3	3.6	84	151	5	MOLECULE:	HEMOGLOBIN
85:00:00	2zlx-C	4.3	3.1	84	139	4	MOLECULE:	HEMOGLOBIN
86:00:00	1hba-B	4.3	3.4	84	146	6	MOLECULE:	HEMOGLOBIN
87:00:00	2zlw-C	4.3	3.5	86	141	2	MOLECULE:	HEMOGLOBIN
88:00:00	2r80-A	4.3	3.6	87	141	6	MOLECULE:	HEMOGLOBIN
89:00:00	1hv4-H	4.3	3.6	85	146	11	MOLECULE:	HEMOGLOBIN
90:00:00	1yih-B	4.3	4.1	86	146	6	MOLECULE:	HEMOGLOBIN
91:00:00	3eu1-A	4.3	4	89	141	3	MOLECULE:	HEMOGLOBIN
92:00:00	1o1l-B	4.3	3.3	85	146	6	MOLECULE:	HEMOGLOBIN
93:00:00	3fs4-A	4.3	3.7	86	141	8	MOLECULE:	HEMOGLOBIN
94:00:00	7pcf-B	4.3	3.3	84	146	10	MOLECULE:	HEMOGLOBIN
95:00:00	1hv4-F	4.3	3.6	85	146	11	MOLECULE:	HEMOGLOBIN
96:00:00	2r80-B	4.3	3.8	86	146	8	MOLECULE:	HEMOGLOBIN
97:00:00	4hrt-B	4.3	3.8	89	146	9	MOLECULE:	GLOBIN-2 A
98:00:00	1hv4-B	4.3	3.6	85	146	11	MOLECULE:	HEMOGLOBIN
99:00:00	4hrt-H	4.3	4.1	90	148	8	MOLECULE:	GLOBIN-2 A
0:00	3a59-F	4.3	3.6	85	146	8	MOLECULE:	HEMOGLOBIN
1:00	1hv4-D	4.3	3.6	85	146	11	MOLECULE:	HEMOGLOBIN
2:00	1yie-B	4.3	3.3	84	146	6	MOLECULE:	HEMOGLOBIN
3:00	4n7p-C	4.3	3.1	84	141	5	MOLECULE:	HEMOGLOBIN
4:00	2dc3-A	4.3	4.8	95	172	11	MOLECULE:	CYTOGLOBIN
5:00	1flp-A	4.3	3.4	78	142	5	MOLECULE:	HEMOGLOBIN
6:00	4xs0-A	4.3	3.3	83	141	5	MOLECULE:	HEMOGLOBIN
7:00	3hrw-A	4.3	3.5	87	141	6	MOLECULE:	HEMOGLOBIN
8:00	2ri4-A	4.3	3	84	139	5	MOLECULE:	HEMOGLOBIN
9:00	4n7p-A	4.3	3.5	84	141	4	MOLECULE:	HEMOGLOBIN
10:00	5ker-A	4.3	3.8	87	140	5	MOLECULE:	ALPHA-GLOBI
11:00	1hbs-C	4.3	3.5	87	141	2	MOLECULE:	HEMOGLOBIN
12:00	3mju-A	4.3	3.4	86	141	7	MOLECULE:	HEMOGLOBIN
13:00	6kai-E	4.3	3	83	141	5	MOLECULE:	HEMOGLOBIN
14:00	3gdj-C	4.3	3.5	86	141	6	MOLECULE:	HEMOGLOBIN
15:00	1hbs-A	4.3	3.1	85	141	4	MOLECULE:	HEMOGLOBIN
16:00	2zfb-A	4.3	3.6	87	141	6	MOLECULE:	HEMOGLOBIN
17:00	1o1m-A	4.3	13.8	117	282	6	MOLECULE:	HEMOGLOBIN
18:00	3gqr-A	4.3	4	89	141	6	MOLECULE:	HEMOGLOBIN

19:00	2pgh-C	4.3	3.1	84	141	6	MOLECULE:	HEMOGLOBIN
20:00	1hbs-H	4.3	3.4	86	146	7	MOLECULE:	HEMOGLOBIN
21:00	3wtg-C	4.3	3.4	85	138	8	MOLECULE:	HEMOGLOBIN
22:00	2zlx-A	4.3	3.1	84	140	4	MOLECULE:	HEMOGLOBIN
23:00	5ker-G	4.3	3.4	86	140	6	MOLECULE:	ALPHA-GLOBI
24:00:00	3gqp-A	4.3	3.2	86	141	7	MOLECULE:	HEMOGLOBIN
25:00:00	5jgg-B	4.3	4.1	87	144	6	MOLECULE:	ALPHA CHAIN
26:00:00	3k8b-D	4.3	3.5	85	146	9	MOLECULE:	HEMOGLOBIN
27:00:00	3pi3-A	4.3	3.7	83	151	4	MOLECULE:	HEMOGLOBIN
28:00:00	1yvq-C	4.3	4	86	141	3	MOLECULE:	HEMOGLOBIN
29:00:00	3dhr-C	4.3	3.1	84	141	7	MOLECULE:	HEMOGLOBIN
30:00:00	1o1n-B	4.3	3.3	84	146	7	MOLECULE:	HEMOGLOBIN
31:00:00	1mtk-A	4.3	4.5	93	154	8	MOLECULE:	MYOGLOBIN
32:00:00	1ch2-A	4.3	4.5	93	154	8	MOLECULE:	PROTEIN (MY
33:00:00	1ye2-B	4.3	3.5	86	146	7	MOLECULE:	HEMOGLOBIN
34:00:00	2oh8-A	4.3	4.5	93	154	8	MOLECULE:	MYOGLOBIN
35:00:00	1y4g-D	4.3	3.5	85	146	7	MOLECULE:	HEMOGLOBIN
36:00:00	1yie-D	4.3	3.5	85	146	7	MOLECULE:	HEMOGLOBIN
37:00:00	1y4v-B	4.3	3.4	84	146	7	MOLECULE:	HEMOGLOBIN
38:00:00	2oha-A	4.3	4.4	93	154	8	MOLECULE:	MYOGLOBIN
39:00:00	2r80-D	4.3	3.5	85	146	9	MOLECULE:	HEMOGLOBIN
40:00:00	1mlo-A	4.3	4.4	92	154	8	MOLECULE:	MYOGLOBIN
41:00:00	1yev-D	4.3	3.6	86	146	7	MOLECULE:	HEMOGLOBIN
42:00:00	5ut7-A	4.3	4.5	92	154	9	MOLECULE:	MYOGLOBIN
43:00:00	1mls-A	4.3	4.5	93	154	6	MOLECULE:	MYOGLOBIN
44:00:00	2qmb-B	4.3	3.5	85	146	9	MOLECULE:	HEMOGLOBIN
45:00:00	1a9w-E	4.3	3.5	85	145	7	MOLECULE:	HEMOGLOBIN
46:00:00	2mgl-A	4.3	4.5	93	154	8	MOLECULE:	MYOGLOBIN
47:00:00	1ygd-B	4.3	3.5	85	146	7	MOLECULE:	HEMOGLOBIN
48:00:00	1xye-C	4.3	3.6	85	141	6	MOLECULE:	HEMOGLOBIN
49:00:00	1ofk-A	4.3	4.5	93	154	8	MOLECULE:	MYOGLOBIN
50:00:00	1co8-A	4.3	4.5	93	154	8	MOLECULE:	PROTEIN (MY
51:00:00	1y46-D	4.3	3.2	84	146	7	MOLECULE:	HEMOGLOBIN
52:00:00	3pi2-B	4.3	3.9	86	151	5	MOLECULE:	HEMOGLOBIN
53:00:00	1h1x-A	4.3	4.5	92	154	9	MOLECULE:	MYOGLOBIN
54:00:00	1mcy-A	4.3	4.1	89	154	10	MOLECULE:	MYOGLOBIN (
55:00:00	4hrt-G	4.3	3.5	86	149	3	MOLECULE:	GLOBIN-2 A
56:00:00	3gqp-D	4.3	3.6	85	145	7	MOLECULE:	HEMOGLOBIN
57:00:00	3dhr-G	4.3	3	82	141	6	MOLECULE:	HEMOGLOBIN
58:00:00	1buw-C	4.3	4	86	141	6	MOLECULE:	PROTEIN (HE
59:00:00	6kav-C	4.3	3.9	86	141	6	MOLECULE:	HEMOGLOBIN
60:00:00	4hrt-C	4.3	3.4	85	149	4	MOLECULE:	GLOBIN-2 A
61:00:00	3k8b-C	4.3	3.5	87	141	3	MOLECULE:	HEMOGLOBIN
62:00:00	1mlu-A	4.3	4.5	93	154	8	MOLECULE:	MYOGLOBIN

63:00:00	3pi1-A	4.3	3.8	85	151	4	MOLECULE:	HEMOGLOBIN
64:00:00	2mgb-A	4.3	4.5	93	154	8	MOLECULE:	MYOGLOBIN
65:00:00	1ye0-C	4.3	4.2	86	141	5	MOLECULE:	HEMOGLOBIN
66:00:00	3dhr-B	4.3	3.6	85	145	9	MOLECULE:	HEMOGLOBIN
67:00:00	5hlq-A	4.3	4.4	91	152	8	MOLECULE:	MYOGLOBIN
68:00:00	1n9h-A	4.3	4.5	92	154	9	MOLECULE:	MYOGLOBIN
69:00:00	1yeo-D	4.3	3.6	86	146	7	MOLECULE:	HEMOGLOBIN
70:00:00	102m-A	4.3	4.5	93	154	8	MOLECULE:	MYOGLOBIN
71:00:00	1mym-A	4.3	4.4	93	154	8	MOLECULE:	MYOGLOBIN
72:00:00	1yg5-D	4.3	3.5	86	146	7	MOLECULE:	HEMOGLOBIN
73:00:00	1ufj-A	4.3	4.1	91	154	8	MOLECULE:	MYOGLOBIN
74:00:00	2mgc-A	4.3	4.5	93	154	8	MOLECULE:	MYOGLOBIN
75:00:00	3pi3-B	4.3	3.8	84	151	5	MOLECULE:	HEMOGLOBIN
76:00:00	1rqa-D	4.3	3.5	86	146	7	MOLECULE:	HEMOGLOBIN
77:00:00	1y46-B	4.3	3.1	83	146	7	MOLECULE:	HEMOGLOBIN
78:00:00	1co9-A	4.3	4.5	93	154	8	MOLECULE:	PROTEIN (MY
79:00:00	2mgh-A	4.3	4.5	93	154	8	MOLECULE:	MYOGLOBIN
80:00:00	1ch3-A	4.3	4.5	93	154	6	MOLECULE:	PROTEIN (MY
81:00:00	4n7n-A	4.3	3.1	84	141	6	MOLECULE:	HEMOGLOBIN
82:00:00	1mlk-A	4.3	4.5	93	154	8	MOLECULE:	MYOGLOBIN
83:00:00	1qi8-C	4.3	3.4	85	141	5	MOLECULE:	HEMOGLOBIN
84:00:00	1y0t-B	4.3	3.4	84	146	7	MOLECULE:	HEMOGLOBIN
85:00:00	2pgh-A	4.3	3.1	84	141	5	MOLECULE:	HEMOGLOBIN
86:00:00	2mge-A	4.3	4.5	93	154	6	MOLECULE:	MYOGLOBIN
87:00:00	2spl-A	4.3	4.5	93	154	6	MOLECULE:	MYOGLOBIN
88:00:00	1f63-A	4.3	4.4	93	154	6	MOLECULE:	MYOGLOBIN
89:00:00	4iro-A	4.3	3.3	85	142	7	MOLECULE:	HEMOGLOBIN
90:00:00	1y4f-B	4.3	3.4	84	146	7	MOLECULE:	HEMOGLOBIN
91:00:00	1j41-E	4.3	3.3	86	141	3	MOLECULE:	HEMOGLOBIN
92:00:00	6u8y-b	4.2	6	69	79	7	MOLECULE:	MONOVALENT
93:00:00	1cqx-A	4.2	4.9	104	403	10	MOLECULE:	FLAVOHEMOPR
94:00:00	7ol3-B	4.2	11.6	92	425	7	MOLECULE:	ATLASTIN-1
95:00:00	7d3u-F	4.2	6.9	75	85	8	MOLECULE:	MONOVALENT
96:00:00	6wb9-6	4.2	4.7	79	98	4	MOLECULE:	ENDOPLASMIC
97:00:00	2qmb-D	4.2	3.6	85	146	9	MOLECULE:	HEMOGLOBIN
98:00:00	3bcq-B	4.2	3.6	86	146	9	MOLECULE:	ALPHA-CHAIN
99:00:00	1myf-A	4.2	3.8	87	153	10	MOLECULE:	MYOGLOBIN
0:00	5x2r-K	4.2	3.9	87	140	6	MOLECULE:	HEMOGLOBIN
1:00	3gqp-B	4.2	3.7	85	145	6	MOLECULE:	HEMOGLOBIN
2:00	7uf7-A	4.2	3.4	85	141	7	MOLECULE:	HEMOGLOBIN
3:00	3pi4-B	4.2	4.1	87	151	7	MOLECULE:	HEMOGLOBIN
4:00	1i3e-B	4.2	3.6	86	146	5	MOLECULE:	HEMOGLOBIN
5:00	7pch-B	4.2	3.6	85	146	6	MOLECULE:	HEMOGLOBIN
6:00	1hds-A	4.2	3.4	86	141	6	MOLECULE:	HEMOGLOBIN

7:00	2r1h-B	4.2	3.6	86	147	9	MOLECULE:	HEMOGLOBIN
8:00	1a0u-D	4.2	3.4	84	146	6	MOLECULE:	HEMOGLOBIN
9:00	3vrg-B	4.2	3.6	85	146	6	MOLECULE:	HEMOGLOBIN
10:00	2qmb-C	4.2	3.7	86	141	7	MOLECULE:	HEMOGLOBIN
11:00	3pt7-A	4.2	3.8	84	151	5	MOLECULE:	HEMOGLOBIN
12:00	3bom-B	4.2	3.6	86	147	9	MOLECULE:	HEMOGLOBIN
13:00	5x2s-E	4.2	3.3	85	140	4	MOLECULE:	HEMOGLOBIN
14:00	3mju-B	4.2	4	87	146	8	MOLECULE:	HEMOGLOBIN
15:00	1y0t-D	4.2	3.3	85	146	6	MOLECULE:	HEMOGLOBIN
16:00	7ylk-A	4.2	4.2	91	153	8	MOLECULE:	MYOGLOBIN
17:00	3k8b-A	4.2	4	87	141	7	MOLECULE:	HEMOGLOBIN
18:00	3bcq-D	4.2	3.5	85	146	9	MOLECULE:	ALPHA-CHAIN
19:00	3eok-B	4.2	3.6	86	146	9	MOLECULE:	HEMOGLOBIN
20:00	1hv4-E	4.2	4.3	87	141	8	MOLECULE:	HEMOGLOBIN
21:00	1coh-A	4.2	3.4	84	141	5	MOLECULE:	HEMOGLOBIN
22:00	1a3o-B	4.2	3.5	84	145	7	MOLECULE:	HEMOGLOBIN
23:00	1hv4-A	4.2	4.3	87	141	8	MOLECULE:	HEMOGLOBIN
24:00:00	3lqd-C	4.2	3.2	86	141	6	MOLECULE:	HEMOGLOBIN
25:00:00	3fs4-C	4.2	4.4	88	141	7	MOLECULE:	HEMOGLOBIN
26:00:00	1faw-C	4.2	3.7	86	141	8	MOLECULE:	HEMOGLOBIN
27:00:00	8egi-A	4.2	3.4	84	141	6	MOLECULE:	HEMOGLOBIN
28:00:00	1a00-A	4.2	3.6	84	141	6	MOLECULE:	HEMOGLOBIN
29:00:00	5urc-A	4.2	3.7	86	141	7	MOLECULE:	HEMOGLOBIN
30:00:00	1b0b-A	4.2	3.4	78	142	4	MOLECULE:	HEMOGLOBIN
31:00:00	1faw-A	4.2	4.1	87	141	8	MOLECULE:	HEMOGLOBIN
32:00:00	4hrt-D	4.2	4.1	90	151	8	MOLECULE:	GLOBIN-2 A
33:00:00	6xdt-A	4.2	3.3	84	141	6	MOLECULE:	HEMOGLOBIN
34:00:00	1hv4-C	4.2	4.2	87	141	8	MOLECULE:	HEMOGLOBIN
35:00:00	2zlw-A	4.2	3.5	86	141	2	MOLECULE:	HEMOGLOBIN
36:00:00	6di4-A	4.2	3.3	84	141	6	MOLECULE:	HEMOGLOBIN
37:00:00	3lqd-A	4.2	3.5	86	141	5	MOLECULE:	HEMOGLOBIN
38:00:00	7xgy-C	4.2	3.6	84	141	6	MOLECULE:	HEMOGLOBIN
39:00:00	1hbs-G	4.2	3.6	87	141	2	MOLECULE:	HEMOGLOBIN
40:00:00	4hrt-F	4.2	4.1	90	151	8	MOLECULE:	GLOBIN-2 A
41:00:00	7xgy-A	4.2	3.6	85	141	5	MOLECULE:	HEMOGLOBIN
42:00:00	4n7p-K	4.2	3.9	87	141	5	MOLECULE:	HEMOGLOBIN
43:00:00	3qm6-A	4.2	4.3	84	145	7	MOLECULE:	MYOGLOBIN
44:00:00	5x2u-A	4.2	4	86	140	6	MOLECULE:	HEMOGLOBIN
45:00:00	1nqp-C	4.2	4	86	141	6	MOLECULE:	HEMOGLOBIN
46:00:00	1dxu-D	4.2	3.8	86	146	5	MOLECULE:	HEMOGLOBIN
47:00:00	2ri4-K	4.2	3	84	138	5	MOLECULE:	HEMOGLOBIN
48:00:00	4mqk-F	4.2	4.1	86	145	6	MOLECULE:	HEMOGLOBIN
49:00:00	1j52-A	4.2	4.2	91	154	10	MOLECULE:	MYOGLOBIN
50:00:00	1fhj-D	4.2	3.5	84	146	6	MOLECULE:	HEMOGLOBIN

51:00:00	6g5t-A	4.2	4.5	93	155	6	MOLECULE:	MYOGLOBIN
52:00:00	109m-A	4.2	4.5	93	154	6	MOLECULE:	MYOGLOBIN
53:00:00	8egi-D	4.2	3.6	85	146	7	MOLECULE:	HEMOGLOBIN
54:00:00	1ch1-A	4.2	4.5	93	154	6	MOLECULE:	PROTEIN (MY
55:00:00	3ovu-C	4.2	3.7	87	139	2	MOLECULE:	ALPHA-HEMOG
56:00:00	2mgk-A	4.2	4.5	93	154	6	MOLECULE:	MYOGLOBIN
57:00:00	3dhr-D	4.2	3.6	85	145	9	MOLECULE:	HEMOGLOBIN
58:00:00	1gbu-B	4.2	3.6	84	146	6	MOLECULE:	HEMOGLOBIN
59:00:00	1mlm-A	4.2	4.4	92	154	8	MOLECULE:	MYOGLOBIN
60:00:00	2oh9-A	4.2	4.5	93	154	6	MOLECULE:	MYOGLOBIN
61:00:00	3k8b-B	4.2	3.5	85	146	9	MOLECULE:	HEMOGLOBIN
62:00:00	6otw-A	4.2	3.7	84	151	5	MOLECULE:	HEMOGLOBIN
63:00:00	1lue-A	4.2	4.5	93	154	6	MOLECULE:	MYOGLOBIN
64:00:00	2mgd-A	4.2	4.5	93	154	6	MOLECULE:	MYOGLOBIN
65:00:00	1cp0-A	4.2	4.4	93	154	6	MOLECULE:	PROTEIN (MY
66:00:00	2zfb-B	4.2	3.6	85	146	9	MOLECULE:	HEMOGLOBIN
67:00:00	2dn1-B	4.2	3.4	84	145	7	MOLECULE:	HEMOGLOBIN
68:00:00	1fhj-B	4.2	3.5	84	146	6	MOLECULE:	HEMOGLOBIN
69:00:00	7jjq-B	4.2	3.6	85	145	6	MOLECULE:	HEMOGLOBIN
70:00:00	1cpw-A	4.2	4.5	93	154	6	MOLECULE:	PROTEIN (MY
71:00:00	2spn-A	4.2	4.5	93	154	6	MOLECULE:	MYOGLOBIN
72:00:00	2dxm-A	4.2	3.7	85	141	6	MOLECULE:	HEMOGLOBIN
73:00:00	5e83-D	4.2	3.4	84	146	7	MOLECULE:	HEMOGLOBIN
74:00:00	112m-A	4.2	4.5	93	154	6	MOLECULE:	MYOGLOBIN
75:00:00	4hrr-C	4.2	3.5	86	149	3	MOLECULE:	GLOBIN-2 A
76:00:00	2spo-A	4.2	4.4	93	154	6	MOLECULE:	MYOGLOBIN
77:00:00	1gli-D	4.2	3.1	83	146	7	MOLECULE:	DEOXYHEMOGL
78:00:00	1yeq-B	4.2	3.5	85	146	7	MOLECULE:	HEMOGLOBIN
79:00:00	1jy7-P	4.2	4.6	88	141	6	MOLECULE:	HEMOGLOBIN
80:00:00	3pi4-A	4.2	3.9	84	151	5	MOLECULE:	HEMOGLOBIN
81:00:00	1gli-B	4.2	3.6	84	146	6	MOLECULE:	DEOXYHEMOGL
82:00:00	1ebt-A	4.2	3.4	78	142	5	MOLECULE:	HEMOGLOBIN
83:00:00	7pcq-B	4.2	3.6	85	146	6	MOLECULE:	HEMOGLOBIN
84:00:00	4hrr-E	4.2	3.9	89	149	6	MOLECULE:	GLOBIN-2 A
85:00:00	2dxm-C	4.2	3.7	86	141	6	MOLECULE:	HEMOGLOBIN
86:00:00	1cp5-A	4.2	4.2	91	154	10	MOLECULE:	PROTEIN (MY
87:00:00	6oty-A	4.2	4	85	151	5	MOLECULE:	HEMOGLOBIN
88:00:00	5e29-D	4.2	3.1	83	145	7	MOLECULE:	HEMOGLOBIN
89:00:00	1mlf-A	4.2	4.6	93	154	8	MOLECULE:	MYOGLOBIN
90:00:00	2spm-A	4.2	4.5	93	154	6	MOLECULE:	MYOGLOBIN
91:00:00	3at6-B	4.2	4	89	146	9	MOLECULE:	ALPHAA-GLOB
92:00:00	1hlb-A	4.1	3.9	91	157	9	MOLECULE:	HEMOGLOBIN
93:00:00	6m5r-B	4.1	17.2	101	689	6	MOLECULE:	TRIPARTITE
94:00:00	4zvb-A	4.1	4.1	89	150	3	MOLECULE:	DIGUANYLATE

95:00:00	2peg-B	4.1	3.8	84	139	7	MOLECULE:	HEMOGLOBIN
96:00:00	4j19-A	4.1	3.2	72	77	6	MOLECULE:	HOMEOBOX-CO
97:00:00	1dm1-A	4.1	4.3	84	146	11	MOLECULE:	MYOGLOBIN
98:00:00	3gys-H	4.1	3.6	85	145	7	MOLECULE:	HEMOGLOBIN
99:00:00	3uhk-D	4.1	3.3	84	145	11	MOLECULE:	GLOBIN-1
0:00	4sdh-A	4.1	3.3	83	145	8	MOLECULE:	HEMOGLOBIN
1:00	3fs4-B	4.1	3.8	86	146	6	MOLECULE:	HEMOGLOBIN
2:00	3dht-A	4.1	3.4	84	139	8	MOLECULE:	HEMOGLOBIN
3:00	7xgy-B	4.1	3.5	84	146	8	MOLECULE:	HEMOGLOBIN
4:00	4n7o-C	4.1	3	82	141	4	MOLECULE:	HEMOGLOBIN
5:00	7pcq-D	4.1	3.6	85	146	6	MOLECULE:	HEMOGLOBIN
6:00	2r1h-D	4.1	3.6	86	147	9	MOLECULE:	HEMOGLOBIN
7:00	1y35-B	4.1	3.6	82	146	7	MOLECULE:	HEMOGLOBIN
8:00	6hbi-B	4.1	3.3	84	145	10	MOLECULE:	HEMOGLOBIN
9:00	3uhw-B	4.1	3.8	86	145	10	MOLECULE:	GLOBIN-1
10:00	5me2-C	4.1	4	86	140	6	MOLECULE:	HEMOGLOBIN
11:00	3b75-C	4.1	3.4	85	141	7	MOLECULE:	HEMOGLOBIN
12:00	1si4-C	4.1	3.4	85	141	7	MOLECULE:	HEMOGLOBIN
13:00	5x2t-J	4.1	3.5	84	145	6	MOLECULE:	HEMOGLOBIN
14:00	3bom-D	4.1	3.7	86	147	7	MOLECULE:	HEMOGLOBIN
15:00	1y2z-B	4.1	3.6	83	146	7	MOLECULE:	HEMOGLOBIN
16:00	7xgy-D	4.1	3.4	84	146	8	MOLECULE:	HEMOGLOBIN
17:00	4mqj-B	4.1	4.1	86	146	5	MOLECULE:	HEMOGLOBIN
18:00	3a59-E	4.1	3.7	86	141	8	MOLECULE:	HEMOGLOBIN
19:00	1nxf-B	4.1	3.5	85	145	9	MOLECULE:	GLOBIN I
20:00	3g4w-A	4.1	3.4	85	145	9	MOLECULE:	GLOBIN-1
21:00	3uhn-B	4.1	3.4	85	145	9	MOLECULE:	GLOBIN-1
22:00	3g4v-A	4.1	3.5	86	145	9	MOLECULE:	GLOBIN-1
23:00	8egi-C	4.1	3.5	85	141	7	MOLECULE:	HEMOGLOBIN
24:00:00	5hy8-S	4.1	3.3	84	141	6	MOLECULE:	HEMOGLOBIN
25:00:00	3mjp-C	4.1	3.8	88	141	3	MOLECULE:	HEMOGLOBIN
26:00:00	1yff-E	4.1	3.4	84	141	6	MOLECULE:	HEMOGLOBIN
27:00:00	5hy8-C	4.1	3.4	85	141	7	MOLECULE:	HEMOGLOBIN
28:00:00	8vyl-C	4.1	3.9	86	140	6	MOLECULE:	HEMOGLOBIN
29:00:00	1bij-A	4.1	3.4	83	141	5	MOLECULE:	HEMOGLOBIN
30:00:00	6kat-C	4.1	3.6	85	141	6	MOLECULE:	HEMOGLOBIN
31:00:00	6l5x-C	4.1	3.9	86	141	6	MOLECULE:	HEMOGLOBIN
32:00:00	6kar-A	4.1	4.1	87	141	7	MOLECULE:	HEMOGLOBIN
33:00:00	3g52-A	4.1	3.5	86	145	9	MOLECULE:	GLOBIN-1
34:00:00	6lcw-E	4.1	4	88	141	6	MOLECULE:	HEMOGLOBIN
35:00:00	4n7p-I	4.1	3.4	85	141	2	MOLECULE:	HEMOGLOBIN
36:00:00	7ud8-A	4.1	3.3	84	141	6	MOLECULE:	HEMOGLOBIN
37:00:00	5hy8-A	4.1	3.6	85	141	6	MOLECULE:	HEMOGLOBIN
38:00:00	4sdh-B	4.1	3.3	84	145	8	MOLECULE:	HEMOGLOBIN

39:00:00	5u3i-D	4.1	3.5	84	145	7	MOLECULE:	HEMOGLOBIN
40:00:00	5hy8-G	4.1	4.1	85	138	6	MOLECULE:	HEMOGLOBIN
41:00:00	1nwi-A	4.1	3.3	85	145	9	MOLECULE:	GLOBIN I
42:00:00	6xd9-C	4.1	3.4	86	141	6	MOLECULE:	HEMOGLOBIN
43:00:00	4n7n-E	4.1	3.8	86	141	7	MOLECULE:	HEMOGLOBIN
44:00:00	4n7o-A	4.1	4.1	88	141	6	MOLECULE:	HEMOGLOBIN
45:00:00	3hrw-C	4.1	3.2	85	141	7	MOLECULE:	HEMOGLOBIN
46:00:00	3g4y-A	4.1	3.5	86	145	9	MOLECULE:	GLOBIN-1
47:00:00	4n7o-G	4.1	4.1	87	141	6	MOLECULE:	HEMOGLOBIN
48:00:00	7vcf-W	4.1	4.5	96	164	6	MOLECULE:	TIC214
49:00:00	6xe7-C	4.1	3.6	85	141	6	MOLECULE:	HEMOGLOBIN
50:00:00	3dhr-A	4.1	4.1	87	141	8	MOLECULE:	HEMOGLOBIN
51:00:00	3uhk-A	4.1	3.2	83	145	8	MOLECULE:	GLOBIN-1
52:00:00	3uhs-B	4.1	3.3	84	145	10	MOLECULE:	GLOBIN-1
53:00:00	5x2t-C	4.1	4	85	140	6	MOLECULE:	HEMOGLOBIN
54:00:00	1a01-D	4.1	3.8	85	146	7	MOLECULE:	HEMOGLOBIN
55:00:00	3b75-A	4.1	4	87	141	7	MOLECULE:	HEMOGLOBIN
56:00:00	1jzk-B	4.1	3.5	84	145	13	MOLECULE:	GLOBIN I -
57:00:00	3g4u-A	4.1	3.5	86	145	10	MOLECULE:	GLOBIN-1
58:00:00	1yg5-B	4.1	3.7	84	146	7	MOLECULE:	HEMOGLOBIN
59:00:00	1y4b-B	4.1	3.6	83	146	7	MOLECULE:	HEMOGLOBIN
60:00:00	5x2r-I	4.1	4.1	86	140	6	MOLECULE:	HEMOGLOBIN
61:00:00	5u3i-B	4.1	3.5	84	146	7	MOLECULE:	HEMOGLOBIN
62:00:00	1y85-B	4.1	3.6	83	145	7	MOLECULE:	HEMOGLOBIN
63:00:00	7pch-D	4.1	3.6	84	146	6	MOLECULE:	HEMOGLOBIN
64:00:00	4hrr-B	4.1	4.1	90	151	8	MOLECULE:	GLOBIN-2 A
65:00:00	1a3n-B	4.1	3.6	83	145	7	MOLECULE:	HEMOGLOBIN
66:00:00	3uhx-B	4.1	3.3	84	145	10	MOLECULE:	GLOBIN-1
67:00:00	1nwi-D	4.1	3.3	83	145	10	MOLECULE:	GLOBIN I
68:00:00	3gys-D	4.1	3.6	85	145	7	MOLECULE:	HEMOGLOBIN
69:00:00	6jp1-B	4.1	4.4	92	151	7	MOLECULE:	MYOGLOBIN
70:00:00	2qls-B	4.1	3.5	84	146	6	MOLECULE:	HEMOGLOBIN
71:00:00	4hrt-A	4.1	4	89	149	4	MOLECULE:	GLOBIN-2 A
72:00:00	1qpw-D	4.1	3.5	84	146	8	MOLECULE:	PORCINE HEM
73:00:00	8vyl-D	4.1	3.7	84	146	6	MOLECULE:	HEMOGLOBIN
74:00:00	8fdm-B	4.1	3.6	84	146	6	MOLECULE:	HEMOGLOBIN
75:00:00	3wcp-B	4.1	3	82	145	7	MOLECULE:	HEMOGLOBIN
76:00:00	2hbs-D	4.1	4.2	85	146	7	MOLECULE:	HEMOGLOBIN
77:00:00	3pel-B	4.1	3.8	86	146	5	MOLECULE:	HEMOGLOBIN
78:00:00	3a59-H	4.1	3.6	85	146	8	MOLECULE:	HEMOGLOBIN
79:00:00	7dds-A	4.1	4.3	90	154	11	MOLECULE:	ANCESTRAL M
80:00:00	8fdm-D	4.1	3.5	84	146	7	MOLECULE:	HEMOGLOBIN
81:00:00	3pia-A	4.1	4	85	141	8	MOLECULE:	HEMOGLOBIN
82:00:00	3g4r-A	4.1	3.6	86	145	10	MOLECULE:	GLOBIN-1

83:00:00	4hrr-G	4.1	4	90	149	6	MOLECULE:	GLOBIN-2 A
84:00:00	3fh9-B	4.1	3.7	86	146	7	MOLECULE:	HEMOGLOBIN
85:00:00	2r80-C	4.1	3.7	86	140	7	MOLECULE:	HEMOGLOBIN
86:00:00	7uf6-D	4.1	3.6	84	146	6	MOLECULE:	HEMOGLOBIN
87:00:00	2b7h-D	4.1	3.5	85	145	6	MOLECULE:	HEMOGLOBIN
88:00:00	8dov-B	4.1	3.6	84	146	6	MOLECULE:	HEMOGLOBIN
89:00:00	5x2s-B	4.1	3.5	84	145	7	MOLECULE:	HEMOGLOBIN
90:00:00	7ud8-D	4.1	3.6	85	146	6	MOLECULE:	HEMOGLOBIN
91:00:00	5x2s-A	4.1	4	87	140	6	MOLECULE:	HEMOGLOBIN
92:00:00	1hbs-B	4.1	3.6	85	146	6	MOLECULE:	HEMOGLOBIN
93:00:00	1jzk-A	4.1	3.5	85	145	11	MOLECULE:	GLOBIN I -
94:00:00	8dov-D	4.1	3.6	85	145	6	MOLECULE:	HEMOGLOBIN
95:00:00	5xwp-A	4	18	86	1125	3	MOLECULE:	UNCHARACTER
96:00:00	8cr1-C	4	3.8	77	266	5	MOLECULE:	ATPASE ASNA
97:00:00	5m0n-A	4	6.3	131	423	2	MOLECULE:	TERMINAL OL
98:00:00	6wig-A	4	4.3	74	84	5	MOLECULE:	STENOFOLIA
99:00:00	4u8u-G	4	3.6	86	151	5	MOLECULE:	GLOBIN A CH
0:00	5tsz-A	4	6.8	86	130	9	MOLECULE:	PV CELL-TRA
1:00	8rjl-C	4	19.8	94	273	5	MOLECULE:	CITRATE SYN
2:00	7wb4-b	4	15.6	97	636	5	MOLECULE:	OUTER NUP13
3:00	7xk3-E	4	7.6	85	198	7	MOLECULE:	NA(+)-TRANS
4:00	1y0w-D	4	3.6	83	146	7	MOLECULE:	HEMOGLOBIN
5:00	4hbi-B	4	3.4	84	145	8	MOLECULE:	HEMOGLOBIN
6:00	2z85-A	4	3.4	84	145	11	MOLECULE:	GLOBIN-1
7:00	6hal-D	4	3.8	85	145	7	MOLECULE:	HEMOGLOBIN
8:00	1hba-D	4	3.6	83	146	7	MOLECULE:	HEMOGLOBIN
9:00	1y7c-B	4	3.6	83	146	7	MOLECULE:	HEMOGLOBIN
10:00	3at5-B	4	4.3	86	146	7	MOLECULE:	ALPHAA-GLOB
11:00	1jzm-B	4	3.5	84	145	8	MOLECULE:	GLOBIN I -
12:00	1nwi-B	4	3.3	84	145	11	MOLECULE:	GLOBIN I
13:00	1y4v-D	4	3.6	83	146	7	MOLECULE:	HEMOGLOBIN
14:00	3vre-D	4	3.7	83	146	7	MOLECULE:	HEMOGLOBIN
15:00	3uhd-A	4	3.4	84	145	11	MOLECULE:	GLOBIN-1
16:00	1jzk-C	4	3.3	83	145	10	MOLECULE:	GLOBIN I -
17:00	3wr1-B	4	4.3	87	146	10	MOLECULE:	HEMOGLOBIN
18:00	5x2s-L	4	3.2	83	145	6	MOLECULE:	HEMOGLOBIN
19:00	1y7c-D	4	3.6	82	146	7	MOLECULE:	HEMOGLOBIN
20:00	3a59-A	4	3.7	86	141	7	MOLECULE:	HEMOGLOBIN
21:00	2hhe-D	4	3.6	83	145	7	MOLECULE:	HEMOGLOBIN
22:00	1nxf-A	4	3.3	82	145	12	MOLECULE:	GLOBIN I
23:00	1gbu-D	4	3.6	83	146	7	MOLECULE:	HEMOGLOBIN
24:00:00	3a0g-B	4	3.5	85	144	8	MOLECULE:	HEMOGLOBIN
25:00:00	3uhc-A	4	3.3	84	145	11	MOLECULE:	GLOBIN-1
26:00:00	3sdh-A	4	3.3	84	145	11	MOLECULE:	HEMOGLOBIN

27:00:00	7ud7-B	4	3.8	86	146	6	MOLECULE:	HEMOGLOBIN
28:00:00	2r4x-B	4	3.5	86	145	9	MOLECULE:	GLOBIN-1
29:00:00	4f4o-B	4	3.7	85	146	7	MOLECULE:	HEMOGLOBIN
30:00:00	3gys-F	4	3.7	85	145	7	MOLECULE:	HEMOGLOBIN
31:00:00	1a3n-D	4	3.6	82	145	7	MOLECULE:	HEMOGLOBIN
32:00:00	2av3-B	4	3.5	84	145	8	MOLECULE:	GLOBIN I
33:00:00	3dhr-H	4	4.2	85	145	9	MOLECULE:	HEMOGLOBIN
34:00:00	1y0w-B	4	3.6	83	146	7	MOLECULE:	HEMOGLOBIN
35:00:00	2d5z-D	4	3.6	82	145	7	MOLECULE:	HEMOGLOBIN
36:00:00	1ygd-D	4	4.1	87	146	6	MOLECULE:	HEMOGLOBIN
37:00:00	3qob-A	4	3.4	85	145	9	MOLECULE:	GLOBIN-1
38:00:00	1o1l-D	4	3.9	86	146	6	MOLECULE:	HEMOGLOBIN
39:00:00	4l7y-D	4	3.6	82	145	7	MOLECULE:	HEMOGLOBIN
40:00:00	1yih-D	4	3.8	86	146	6	MOLECULE:	HEMOGLOBIN
41:00:00	2auq-A	4	3.4	84	145	11	MOLECULE:	GLOBIN I
42:00:00	3uhk-B	4	3.3	82	145	12	MOLECULE:	GLOBIN-1
43:00:00	1jwn-B	4	3.5	83	145	12	MOLECULE:	GLOBIN I -
44:00:00	1fdh-H	4	3.8	86	146	5	MOLECULE:	HEMOGLOBIN
45:00:00	1v5h-A	4	4.1	86	151	10	MOLECULE:	CYTOGLOBIN
46:00:00	2aur-B	4	3.5	85	145	8	MOLECULE:	GLOBIN I
47:00:00	2d60-B	4	3.7	83	145	7	MOLECULE:	HEMOGLOBIN
48:00:00	1y4f-D	4	3.6	83	146	7	MOLECULE:	HEMOGLOBIN
49:00:00	1a0u-B	4	3.7	85	146	6	MOLECULE:	HEMOGLOBIN
50:00:00	1y85-D	4	3.6	82	145	7	MOLECULE:	HEMOGLOBIN
51:00:00	3uht-B	4	3.5	85	145	11	MOLECULE:	GLOBIN-1
52:00:00	1fdh-G	4	3.8	86	146	5	MOLECULE:	HEMOGLOBIN
53:00:00	3uhe-A	4	3.3	84	145	10	MOLECULE:	GLOBIN-1
54:00:00	1a0z-D	4	3.6	82	146	7	MOLECULE:	HEMOGLOBIN
55:00:00	1y2z-D	4	3.6	82	146	7	MOLECULE:	HEMOGLOBIN
56:00:00	1jzl-A	4	3.3	84	145	11	MOLECULE:	GLOBIN I -
57:00:00	3ui0-B	4	3.5	85	145	8	MOLECULE:	GLOBIN-1
58:00:00	3uhz-A	4	3.3	84	145	11	MOLECULE:	GLOBIN-1
59:00:00	5x2r-F	4	3.6	84	145	7	MOLECULE:	HEMOGLOBIN
60:00:00	3uhg-A	4	3.4	84	145	11	MOLECULE:	GLOBIN-1
61:00:00	3uht-A	4	3.3	83	145	10	MOLECULE:	GLOBIN-1
62:00:00	2z85-B	4	3.3	83	145	11	MOLECULE:	GLOBIN-1
63:00:00	1y4p-D	4	3.8	86	146	6	MOLECULE:	HEMOGLOBIN
64:00:00	3uhz-B	4	3.5	84	145	8	MOLECULE:	GLOBIN-1
65:00:00	2d60-D	4	3.6	82	145	7	MOLECULE:	HEMOGLOBIN
66:00:00	1a00-B	4	3.7	85	146	6	MOLECULE:	HEMOGLOBIN
67:00:00	3uhi-A	4	3.4	84	145	8	MOLECULE:	GLOBIN-1
68:00:00	3vre-B	4	3.3	83	146	6	MOLECULE:	HEMOGLOBIN
69:00:00	3uhy-B	4	3.5	84	145	8	MOLECULE:	GLOBIN-1
70:00:00	3uhk-C	4	3.3	84	145	10	MOLECULE:	GLOBIN-1

71:00:00	1nwi-C	4	3.4	85	145	9	MOLECULE:	GLOBIN I
72:00:00	3g4q-A	4	3.5	86	145	9	MOLECULE:	GLOBIN-1
73:00:00	4n7n-C	4	4.1	86	141	6	MOLECULE:	HEMOGLOBIN
74:00:00	3g4w-B	4	3.5	84	145	8	MOLECULE:	GLOBIN-1
75:00:00	3g53-A	4	3.3	84	145	10	MOLECULE:	GLOBIN-1
76:00:00	7vuc-A	4	4.9	77	153	12	MOLECULE:	MYOGLOBIN
77:00:00	7hbi-A	4	3.3	83	145	8	MOLECULE:	HEMOGLOBIN
78:00:00	3gys-B	4	3.7	85	145	7	MOLECULE:	HEMOGLOBIN
79:00:00	3uhx-A	4	3.3	84	145	10	MOLECULE:	GLOBIN-1
80:00:00	3ugy-A	4	3.3	84	145	10	MOLECULE:	GLOBIN-1
81:00:00	3uhw-A	4	3.3	84	145	10	MOLECULE:	GLOBIN-1
82:00:00	3uh7-A	4	3.3	84	145	10	MOLECULE:	GLOBIN-1
83:00:00	3uhr-B	4	3.3	83	145	8	MOLECULE:	GLOBIN-1
84:00:00	1jzk-D	4	3.3	84	145	11	MOLECULE:	GLOBIN I -
85:00:00	3ag0-A	4	4	89	158	10	MOLECULE:	CYTOGLOBIN
86:00:00	1jwn-D	4	3.3	84	145	10	MOLECULE:	GLOBIN I -
87:00:00	3gqr-F	4	3.5	84	145	8	MOLECULE:	HEMOGLOBIN
88:00:00	2hhe-B	4	4.2	85	145	7	MOLECULE:	HEMOGLOBIN
89:00:00	5jdo-D	4	3.5	84	145	6	MOLECULE:	HAPTOGLOBIN
90:00:00	1y35-D	4	3.6	83	146	7	MOLECULE:	HEMOGLOBIN
91:00:00	1shr-B	4	3.6	84	146	5	MOLECULE:	HEMOGLOBIN
92:00:00	1a3o-D	4	3.7	83	145	7	MOLECULE:	HEMOGLOBIN
93:00:00	5e83-B	4	3.7	84	146	6	MOLECULE:	HEMOGLOBIN
94:00:00	5hbi-A	4	3.4	84	145	10	MOLECULE:	HEMOGLOBIN
95:00:00	3gou-D	4	3.6	85	146	5	MOLECULE:	HEMOGLOBIN
96:00:00	3uhu-A	4	3.3	83	145	8	MOLECULE:	GLOBIN-1
97:00:00	3uhv-A	4	3.3	84	145	10	MOLECULE:	GLOBIN-1
98:00:00	3uhy-A	4	3.3	82	146	12	MOLECULE:	GLOBIN-1
99:00:00	3uhv-B	4	3.5	86	145	10	MOLECULE:	GLOBIN-1
0:00	3uh6-A	4	3.4	85	145	11	MOLECULE:	GLOBIN-1
1:00	8dov-F	4	4.2	85	144	7	MOLECULE:	HEMOGLOBIN
2:00	5x2r-D	4	3.7	85	145	6	MOLECULE:	HEMOGLOBIN
3:00	3uh3-A	4	3.3	83	145	11	MOLECULE:	GLOBIN-1
4:00	1ch4-D	4	3.5	84	146	5	MOLECULE:	MODULE-SUBS
5:00	2aup-B	4	3.5	84	145	12	MOLECULE:	GLOBIN I
6:00	3gqr-D	4	3.7	84	145	7	MOLECULE:	HEMOGLOBIN
7:00	3g46-A	4	3.5	85	146	8	MOLECULE:	GLOBIN-1
8:00	1nej-D	4	3.6	85	146	7	MOLECULE:	HEMOGLOBIN
9:00	2grf-A	4	3.3	83	145	8	MOLECULE:	GLOBIN-1
10:00	7uf6-B	4	3.6	84	146	7	MOLECULE:	HEMOGLOBIN
11:00	5x2t-L	4	3.4	84	145	7	MOLECULE:	HEMOGLOBIN
12:00	3uhu-B	4	3.8	83	145	11	MOLECULE:	GLOBIN-1
13:00	3uhr-A	4	3.3	83	145	12	MOLECULE:	GLOBIN-1
14:00	3uhn-A	4	3.3	83	145	8	MOLECULE:	GLOBIN-1

15:00	6hbi-A	4	3.6	85	145	9	MOLECULE:	HEMOGLOBIN
16:00	3uhs-A	4	3.4	84	145	10	MOLECULE:	GLOBIN-1
17:00	3ugz-A	4	3.4	84	145	11	MOLECULE:	GLOBIN-1
18:00	3ui0-A	4	3.4	84	145	10	MOLECULE:	GLOBIN-1
19:00	3gqr-H	4	3.6	84	145	7	MOLECULE:	HEMOGLOBIN
20:00	1ch4-C	4	3.5	84	146	5	MOLECULE:	MODULE-SUBS
21:00	3uhq-A	4	3.3	83	145	8	MOLECULE:	GLOBIN-1
22:00	6wg7-E	4	8.3	102	228	8	MOLECULE:	DNA (35-MER
23:00	8qfs-A	3.9	13	100	1183	12	MOLECULE:	ELONGATION
24:00:00	5y0r-A	3.9	14.2	112	423	4	MOLECULE:	TMTMCAL(SEM
25:00:00	7m8i-A	3.9	9.7	151	572	6	MOLECULE:	ADRENODOXIN
26:00:00	6m9a-C	3.9	4.3	87	157	13	MOLECULE:	SIGNALING P
27:00:00	8q4h-C	3.9	4.3	70	89	7	MOLECULE:	TETRACHLORO
28:00:00	3bhn-A	3.9	5.5	81	214	9	MOLECULE:	THIJ/PFPI D
29:00:00	8q6o-2	3.9	6.5	92	267	9	MOLECULE:	DNA REPLICA
30:00:00	1or4-A	3.9	5.5	106	169	8	MOLECULE:	HEME-BASED
31:00:00	6z16-d	3.9	9.2	104	489	8	MOLECULE:	MULTISUBUNI
32:00:00	2p6v-A	3.9	3.3	77	97	10	MOLECULE:	TRANSCRIPTI
33:00:00	2hvr-A	3.9	11.5	90	319	6	MOLECULE:	5'-
34:00:00	7ewp-A	3.9	9.7	106	577	6	MOLECULE:	PROBABLE G-
35:00:00	3eqm-A	3.9	4.5	147	452	4	MOLECULE:	CYTOCHROME
36:00:00	6zka-K	3.9	8	81	98	7	MOLECULE:	NADH-UBIQUI
37:00:00	3mjp-B	3.9	3.6	85	146	9	MOLECULE:	HEMOGLOBIN
38:00:00	4l7y-B	3.9	3.6	83	145	7	MOLECULE:	HEMOGLOBIN
39:00:00	6hal-B	3.9	3.9	85	145	7	MOLECULE:	HEMOGLOBIN
40:00:00	4f4o-E	3.9	3.7	85	146	7	MOLECULE:	HEMOGLOBIN
41:00:00	3uhi-B	3.9	3.4	83	145	10	MOLECULE:	GLOBIN-1
42:00:00	5x2s-J	3.9	3.8	83	145	7	MOLECULE:	HEMOGLOBIN
43:00:00	3uhq-B	3.9	3.6	83	145	12	MOLECULE:	GLOBIN-1
44:00:00	5e29-B	3.9	3.6	82	145	7	MOLECULE:	HEMOGLOBIN
45:00:00	5x2s-C	3.9	4.2	86	140	6	MOLECULE:	HEMOGLOBIN
46:00:00	5x2r-E	3.9	4.1	86	140	6	MOLECULE:	HEMOGLOBIN
47:00:00	3ugy-B	3.9	3.3	81	145	11	MOLECULE:	GLOBIN-1
48:00:00	4f4o-H	3.9	3.7	85	146	7	MOLECULE:	HEMOGLOBIN
49:00:00	2aup-A	3.9	3.4	84	145	11	MOLECULE:	GLOBIN I
50:00:00	1a0z-B	3.9	3.7	85	146	6	MOLECULE:	HEMOGLOBIN
51:00:00	7k4m-E	3.9	3.9	86	142	6	MOLECULE:	HEMOGLOBIN
52:00:00	3sdh-B	3.9	3.4	83	145	8	MOLECULE:	HEMOGLOBIN
53:00:00	3uhc-B	3.9	3.4	81	145	12	MOLECULE:	GLOBIN-1
54:00:00	3g4y-B	3.9	3.4	82	145	12	MOLECULE:	GLOBIN-1
55:00:00	3uh7-B	3.9	3.4	83	145	8	MOLECULE:	GLOBIN-1
56:00:00	3uhe-B	3.9	3.4	83	145	8	MOLECULE:	GLOBIN-1
57:00:00	3g52-B	3.9	3.4	82	145	12	MOLECULE:	GLOBIN-1
58:00:00	3uhi-C	3.9	3.3	84	145	10	MOLECULE:	GLOBIN-1

59:00:00	3g4v-B	3.9	3.4	82	145	12	MOLECULE:	GLOBIN-1
60:00:00	1jwn-C	3.9	3.4	84	145	11	MOLECULE:	GLOBIN I -
61:00:00	1bij-C	3.9	3.8	86	141	7	MOLECULE:	HEMOGLOBIN
62:00:00	2aur-A	3.9	3.3	82	145	12	MOLECULE:	GLOBIN I
63:00:00	3kmf-E	3.9	3.8	86	141	7	MOLECULE:	HEMOGLOBIN
64:00:00	3uhb-B	3.9	3.4	83	145	8	MOLECULE:	GLOBIN-1
65:00:00	3uhh-B	3.9	3.6	83	145	12	MOLECULE:	GLOBIN-1
66:00:00	2auo-B	3.9	3.4	82	145	12	MOLECULE:	GLOBIN I
67:00:00	2m6z-A	3.9	3.2	85	141	5	MOLECULE:	HEMOGLOBIN
68:00:00	1hds-C	3.9	3.6	86	141	6	MOLECULE:	HEMOGLOBIN
69:00:00	3g4q-B	3.9	3.4	83	145	8	MOLECULE:	GLOBIN-1
70:00:00	2grh-B	3.9	3.5	84	145	8	MOLECULE:	GLOBIN-1
71:00:00	7hbi-B	3.9	3.4	82	145	12	MOLECULE:	HEMOGLOBIN
72:00:00	3uh6-B	3.9	3.4	83	145	8	MOLECULE:	GLOBIN-1
73:00:00	3uhg-B	3.9	3.4	82	145	12	MOLECULE:	GLOBIN-1
74:00:00	5x2u-J	3.9	3.7	83	145	7	MOLECULE:	HEMOGLOBIN
75:00:00	3d4x-B	3.9	4.1	85	145	7	MOLECULE:	HEMOGLOBIN
76:00:00	8vyl-B	3.9	4.1	84	145	7	MOLECULE:	HEMOGLOBIN
77:00:00	3hyu-B	3.9	3.7	86	146	8	MOLECULE:	HEMOGLOBIN
78:00:00	3d4x-D	3.9	3.8	83	145	7	MOLECULE:	HEMOGLOBIN
79:00:00	3mjp-D	3.9	3.6	85	146	9	MOLECULE:	HEMOGLOBIN
80:00:00	4h2l-B	3.9	3.7	85	146	7	MOLECULE:	ALPHA-GLOBI
81:00:00	5x2u-L	3.9	4.2	85	145	6	MOLECULE:	HEMOGLOBIN
82:00:00	2m6z-C	3.9	3.2	85	141	5	MOLECULE:	HEMOGLOBIN
83:00:00	1jzm-A	3.9	3.4	82	145	10	MOLECULE:	GLOBIN I -
84:00:00	7k4m-D	3.9	3.5	84	147	7	MOLECULE:	HEMOGLOBIN
85:00:00	1jwn-A	3.9	3.4	82	145	11	MOLECULE:	GLOBIN I -
86:00:00	5x2t-A	3.9	4.1	87	140	7	MOLECULE:	HEMOGLOBIN
87:00:00	5x2s-H	3.9	3.5	83	145	6	MOLECULE:	HEMOGLOBIN
88:00:00	3gqr-B	3.9	3.7	85	145	7	MOLECULE:	HEMOGLOBIN
89:00:00	3gou-B	3.9	4.2	85	146	6	MOLECULE:	HEMOGLOBIN
90:00:00	2r4y-B	3.9	3.5	82	145	10	MOLECULE:	GLOBIN-1
91:00:00	5ufj-B	3.9	3.7	84	146	6	MOLECULE:	HEMOGLOBIN
92:00:00	7jjq-D	3.9	4.1	85	144	6	MOLECULE:	HEMOGLOBIN
93:00:00	5ker-H	3.9	3.7	86	146	8	MOLECULE:	ALPHA-GLOBI
94:00:00	1x3k-A	3.8	3.8	81	152	9	MOLECULE:	HEMOGLOBIN
95:00:00	4bja-A	3.8	5.3	99	182	7	MOLECULE:	PROTEIN GLB
96:00:00	8h17-A	3.8	5	88	157	7	MOLECULE:	TLR1989 PRO
97:00:00	4m8m-B	3.8	6.4	118	574	3	MOLECULE:	GCN4 COILED
98:00:00	2q0y-A	3.8	3.8	57	153	4	MOLECULE:	GCN5-RELATE
99:00:00	5o5e-A	3.8	10.7	129	381	1	MOLECULE:	UDP-N-ACETY
0:00	7xdi-D	3.8	5.8	79	124	6	MOLECULE:	VP1
1:00	2da6-A	3.8	5	76	102	5	MOLECULE:	HEPATOCYTE
2:00	7w0y-k	3.8	6.3	81	98	6	MOLECULE:	NADH DEHYDR

3:00	5ex8-A	3.8	5.9	143	414	8	MOLECULE:	CYTOCHROME
4:00	7uw5-A	3.8	14	99	723	9	MOLECULE:	MECHANOSENS
5:00	3wft-A	3.8	4	81	153	14	MOLECULE:	MYOGLOBIN
6:00	1shr-D	3.8	4.8	85	146	4	MOLECULE:	HEMOGLOBIN
7:00	2d5z-B	3.8	3.9	84	145	5	MOLECULE:	HEMOGLOBIN
8:00	5x2t-F	3.8	3.7	84	145	6	MOLECULE:	HEMOGLOBIN
9:00	7spf-A	3.8	3.8	84	152	11	MOLECULE:	MYOGLOBIN
10:00	6bmg-A	3.8	4	85	154	9	MOLECULE:	MYOGLOBIN
11:00	3g4u-B	3.8	4.1	84	145	15	MOLECULE:	GLOBIN-1
12:00	2r4w-B	3.8	3.6	83	145	11	MOLECULE:	GLOBIN-1
13:00	3uh3-B	3.8	3.6	82	145	13	MOLECULE:	GLOBIN-1
14:00	6on4-A	3.8	3.9	91	217	8	MOLECULE:	HTH-TYPE TR
15:00	3uh5-B	3.8	3.5	84	145	8	MOLECULE:	GLOBIN-1
16:00	2b7h-B	3.8	4.6	86	144	6	MOLECULE:	HEMOGLOBIN
17:00	5ufj-D	3.8	4.1	86	146	6	MOLECULE:	HEMOGLOBIN
18:00	7ud8-B	3.8	4.1	85	146	6	MOLECULE:	HEMOGLOBIN
19:00	3g4r-B	3.8	4	84	145	15	MOLECULE:	GLOBIN-1
20:00	3uhd-B	3.8	3.6	85	144	9	MOLECULE:	GLOBIN-1
21:00	1nej-B	3.8	3.6	84	146	7	MOLECULE:	HEMOGLOBIN
22:00	5x2u-B	3.8	4.2	86	145	6	MOLECULE:	HEMOGLOBIN
23:00	5x2r-L	3.8	4.2	86	145	7	MOLECULE:	HEMOGLOBIN
24:00:00	5x2r-J	3.8	4.1	88	145	6	MOLECULE:	HEMOGLOBIN
25:00:00	5x2t-B	3.8	4.2	86	145	6	MOLECULE:	HEMOGLOBIN
26:00:00	5x2u-F	3.8	4.1	84	145	7	MOLECULE:	HEMOGLOBIN
27:00:00	5jdo-F	3.8	4	84	144	7	MOLECULE:	HAPTOGLOBIN
28:00:00	1nwn-B	3.8	3.9	84	145	12	MOLECULE:	GLOBIN I
29:00:00	6wfq-C	3.8	8.1	107	228	7	MOLECULE:	HTH-TYPE TR
30:00:00	4g51-C	3.8	3.7	86	142	6	MOLECULE:	HEMOGLOBIN
31:00:00	2pgh-D	3.8	4	84	146	8	MOLECULE:	HEMOGLOBIN
32:00:00	5v7p-A	3.7	6.2	101	281	7	MOLECULE:	PROTEIN-S-I
33:00:00	4noo-A	3.7	3.7	77	200	6	MOLECULE:	VGRG PROTEI
34:00:00	7dpd-B	3.7	4.3	80	273	4	MOLECULE:	DNA HELICAS
35:00:00	7lt2-A	3.7	17.1	101	387	10	MOLECULE:	MAB-21 DOMA
36:00:00	1x46-A	3.7	4	80	150	6	MOLECULE:	HEMOGLOBIN
37:00:00	8b6l-M	3.7	6.4	74	110	3	MOLECULE:	PROTEIN TRA
38:00:00	5ca9-A	3.7	25.6	116	656	7	MOLECULE:	PROTEIN SEY
39:00:00	7sjr-A	3.7	7.7	99	911	10	MOLECULE:	DNA HELICAS
40:00:00	6ezn-B	3.7	7.5	77	110	4	MOLECULE:	DOLICHYL-DI
41:00:00	8pi9-B	3.7	10.3	92	177	8	MOLECULE:	CHAINS: E
42:00:00	2zfo-D	3.7	3.8	83	145	8	MOLECULE:	EXTRACELLUL
43:00:00	8jem-B	3.7	9	97	419	6	MOLECULE:	TEICHOIC AC
44:00:00	7rb4-A	3.7	18.8	122	603	7	MOLECULE:	DIPHTHERIA
45:00:00	7k4m-B	3.7	4.2	86	146	5	MOLECULE:	HEMOGLOBIN
46:00:00	3uhi-D	3.7	3.8	83	145	12	MOLECULE:	GLOBIN-1

47:00:00	1hbi-A	3.7	3.9	84	145	12	MOLECULE:	HEMOGLOBIN
48:00:00	5jom-A	3.7	3.7	84	154	11	MOLECULE:	MYOGLOBIN
49:00:00	1ch4-B	3.7	3.9	84	146	8	MOLECULE:	MODULE-SUBS
50:00:00	2r4z-A	3.7	3.8	82	145	13	MOLECULE:	GLOBIN-1
51:00:00	5ych-A	3.7	3.8	84	153	12	MOLECULE:	ANCESTRAL M
52:00:00	2grh-A	3.7	3.9	84	145	12	MOLECULE:	GLOBIN-1
53:00:00	5x2t-H	3.7	3.8	83	145	7	MOLECULE:	HEMOGLOBIN
54:00:00	1ch4-A	3.7	3.9	84	146	8	MOLECULE:	MODULE-SUBS
55:00:00	4f4o-K	3.7	4.3	85	146	8	MOLECULE:	HEMOGLOBIN
56:00:00	2grf-B	3.7	3.9	85	145	12	MOLECULE:	GLOBIN-1
57:00:00	1jzl-B	3.7	4	84	145	15	MOLECULE:	GLOBIN I -
58:00:00	5m3r-A	3.7	3.7	84	154	11	MOLECULE:	MYOGLOBIN
59:00:00	5hbi-B	3.7	4	84	145	15	MOLECULE:	HEMOGLOBIN
60:00:00	7a45-A	3.7	3.7	84	154	11	MOLECULE:	MYOGLOBIN
61:00:00	3uhh-A	3.7	3.9	84	145	14	MOLECULE:	GLOBIN-1
62:00:00	2auq-B	3.7	4	84	145	15	MOLECULE:	GLOBIN I
63:00:00	4hbi-A	3.7	3.8	83	145	12	MOLECULE:	HEMOGLOBIN
64:00:00	3g53-B	3.7	4.1	84	145	15	MOLECULE:	GLOBIN-1
65:00:00	2av0-A	3.7	3.9	84	145	12	MOLECULE:	GLOBIN I
66:00:00	2pgh-B	3.7	3.7	84	146	6	MOLECULE:	HEMOGLOBIN
67:00:00	3qob-B	3.7	4	84	145	14	MOLECULE:	GLOBIN-1
68:00:00	5x2u-H	3.7	3.8	84	145	6	MOLECULE:	HEMOGLOBIN
69:00:00	6bb5-B	3.7	3.8	83	145	6	MOLECULE:	HEMOGLOBIN
70:00:00	1mbs-A	3.7	3.9	91	153	11	MOLECULE:	MYOGLOBIN
71:00:00	2av0-B	3.7	4	84	145	15	MOLECULE:	GLOBIN I
72:00:00	1si4-D	3.7	4.2	84	146	6	MOLECULE:	HEMOGLOBIN
73:00:00	6kye-H	3.7	4.3	86	145	7	MOLECULE:	HEMOGLOBIN
74:00:00	7uvb-D	3.7	4.1	84	145	7	MOLECULE:	HEMOGLOBIN
75:00:00	3ugz-B	3.7	4.1	84	145	15	MOLECULE:	GLOBIN-1
76:00:00	4ood-A	3.7	3.5	80	154	11	MOLECULE:	MYOGLOBIN
77:00:00	2r4y-A	3.7	3.9	84	145	14	MOLECULE:	GLOBIN-1
78:00:00	3g46-B	3.7	3.7	82	146	13	MOLECULE:	GLOBIN-1
79:00:00	4x0l-B	3.7	4.2	85	144	5	MOLECULE:	HEMOGLOBIN
80:00:00	2z8a-A	3.7	3.9	85	145	11	MOLECULE:	GLOBIN-1
81:00:00	5x2s-D	3.7	4.2	85	145	6	MOLECULE:	HEMOGLOBIN
82:00:00	3hf4-B	3.7	3.8	85	145	6	MOLECULE:	HEMOGLOBIN
83:00:00	5ker-D	3.7	4.3	85	146	8	MOLECULE:	ALPHA-GLOBI
84:00:00	2auo-A	3.7	3.9	85	145	12	MOLECULE:	GLOBIN I
85:00:00	3uh5-A	3.7	3.9	85	145	12	MOLECULE:	GLOBIN-1
86:00:00	6wg7-C	3.7	8.1	107	228	7	MOLECULE:	DNA (35-MER
87:00:00	6wg7-F	3.7	8.2	102	228	7	MOLECULE:	DNA (35-MER
88:00:00	3uhb-A	3.7	3.9	85	145	12	MOLECULE:	GLOBIN-1
89:00:00	3vw4-A	3.6	18.9	85	122	7	MOLECULE:	REP
90:00:00	4ktp-A	3.6	16.3	100	767	9	MOLECULE:	GLYCOSIDE H

91:00:00	1us7-B	3.6	20.6	100	193	2	MOLECULE:	HEAT SHOCK
92:00:00	6rd4-4	3.6	14	92	290	8	MOLECULE:	ASA-10: POL
93:00:00	4u6u-A	3.6	4.5	59	67	7	MOLECULE:	COG7
94:00:00	3bes-R	3.6	11.8	88	250	9	MOLECULE:	INTERFERON
95:00:00	8slm-A	3.6	8	96	248	10	MOLECULE:	ZN DEPENDEN
96:00:00	3v8d-A	3.6	4.9	142	479	3	MOLECULE:	CHOLESTEROL
97:00:00	1lqs-L	3.6	11.7	99	142	10	MOLECULE:	INTERLEUKIN
98:00:00	3zjh-B	3.6	7.2	99	193	10	MOLECULE:	PROTOGLOBIN
99:00:00	1puf-A	3.6	4.5	67	77	3	MOLECULE:	5'-
0:00	8w20-A	3.6	31.8	65	732	9	MOLECULE:	SECRETED PR
1:00	7etw-B	3.6	22	108	483	6	MOLECULE:	INSULIN-IND
2:00	6s8h-F	3.6	5	85	239	7	MOLECULE:	LIPOPOLYSAC
3:00	5eik-A	3.6	9.3	87	234	6	MOLECULE:	UNCHARACTER
4:00	6iuy-A	3.6	10.4	89	585	2	MOLECULE:	GLYCEROL-3-
5:00	6ygb-B	3.6	19.2	106	725	5	MOLECULE:	N-ALPHA-ACE
6:00	7z8b-C	3.6	26.3	131	1224	9	MOLECULE:	CULLIN-7
7:00	1nek-D	3.6	9.1	84	113	6	MOLECULE:	SUCCINATE D
8:00	1yvl-B	3.6	13.9	107	653	7	MOLECULE:	SIGNAL TRAN
9:00	8hmc-B	3.6	27.3	131	1195	4	MOLECULE:	INTRAFLAGEL
10:00	7uic-e	3.6	23.8	92	729	12	MOLECULE:	MEDIATOR OF
11:00	7yug-A	3.6	2.9	63	117	3	MOLECULE:	PROTEIN BAN
12:00	8fib-A	3.6	5.2	134	418	10	MOLECULE:	CYTOCHROME
13:00	2z8a-B	3.6	4.1	84	146	14	MOLECULE:	GLOBIN-1
14:00	2av3-A	3.6	3.9	84	145	12	MOLECULE:	GLOBIN I
15:00	1hbs-F	3.6	3.7	84	146	6	MOLECULE:	HEMOGLOBIN
16:00	2r4z-B	3.6	4	84	146	14	MOLECULE:	GLOBIN-1
17:00	1hbi-B	3.6	4.1	84	145	15	MOLECULE:	HEMOGLOBIN
18:00	5ycj-A	3.6	4	81	154	12	MOLECULE:	ANCESTRAL M
19:00	8f9i-A	3.6	3.7	80	154	11	MOLECULE:	MYOGLOBIN
20:00	1mnh-A	3.6	3.7	80	153	15	MOLECULE:	MYOGLOBIN
21:00	7uvb-B	3.6	4.3	86	146	7	MOLECULE:	HEMOGLOBIN
22:00	5x2r-H	3.6	4.2	88	145	8	MOLECULE:	HEMOGLOBIN
23:00	1it3-B	3.6	4	80	146	6	MOLECULE:	HEMOGLOBIN
24:00:00	7ddr-A	3.6	4.6	90	154	10	MOLECULE:	ANCESTRAL M
25:00:00	1nwn-A	3.6	3.9	84	145	12	MOLECULE:	GLOBIN I
26:00:00	2r4x-A	3.6	3.9	84	145	12	MOLECULE:	GLOBIN-1
27:00:00	1z8u-B	3.6	3.6	81	135	7	MOLECULE:	ALPHA-HEMOG
28:00:00	1it3-D	3.6	3.2	76	146	7	MOLECULE:	HEMOGLOBIN
29:00:00	1z8u-D	3.6	3.7	81	135	7	MOLECULE:	ALPHA-HEMOG
30:00:00	3al0-B	3.5	13.3	79	482	10	MOLECULE:	GLUTAMYL-TR
31:00:00	6rfl-I	3.5	20.7	119	773	9	MOLECULE:	DNA-DEPENDE
32:00:00	4zi2-C	3.5	4.1	80	131	6	MOLECULE:	ADP-RIBOSYL
33:00:00	7kzn-C	3.5	34.3	121	965	7	MOLECULE:	HEAVY CHAIN
34:00:00	5o1l-A	3.5	4.3	109	375	6	MOLECULE:	RUBBER OXYG

35:00:00	8wu3-B	3.5	24	121	592	7	MOLECULE:	RNA POLYMER
36:00:00	8ce1-c	3.5	8.7	79	241	8	MOLECULE:	CYTOCHROME
37:00:00	1w9r-A	3.5	12.3	88	119	2	MOLECULE:	CHOLINE BIN
38:00:00	5t8v-A	3.5	11	103	1315	5	MOLECULE:	PUTATIVE UN
39:00:00	3l9w-B	3.5	6.6	97	357	2	MOLECULE:	GLUTATHIONE
40:00:00	7obq-u	3.5	15.6	76	441	3	MOLECULE:	SRP RNA
41:00:00	5tpm-A	3.5	9	94	155	5	MOLECULE:	PYRUVATE DE
42:00:00	6csm-D	3.5	11.4	129	277	8	MOLECULE:	GTACR1
43:00:00	8xh6-A	3.5	6.5	72	161	8	MOLECULE:	LATENT MEMB
44:00:00	7pkq-o	3.5	7.1	76	163	3	MOLECULE:	MS35
45:00:00	8p50-A	3.5	24.6	95	1574	5	MOLECULE:	TOXIN PROTE
46:00:00	7qru-F	3.5	7.9	80	91	6	MOLECULE:	NA+/H+ ANTI
47:00:00	7pik-C	3.5	21.3	90	557	4	MOLECULE:	TRANSPOSON
48:00:00	6wg3-E	3.5	10.2	97	1226	4	MOLECULE:	STRUCTURAL
49:00:00	1n9i-A	3.5	3.8	83	154	10	MOLECULE:	MYOGLOBIN
50:00:00	3qma-A	3.5	4.1	78	145	5	MOLECULE:	MYOGLOBIN
51:00:00	5ycg-A	3.5	3.7	80	154	14	MOLECULE:	ANCESTRAL M
52:00:00	5hlu-A	3.5	4	80	152	10	MOLECULE:	MYOGLOBIN
53:00:00	2w6x-A	3.5	4.1	84	154	8	MOLECULE:	MYOGLOBIN
54:00:00	3h57-A	3.5	3.8	83	154	10	MOLECULE:	MYOGLOBIN
55:00:00	5ojc-A	3.5	3.8	84	154	10	MOLECULE:	MYOGLOBIN
56:00:00	3nml-A	3.5	3.6	80	154	11	MOLECULE:	MYOGLOBIN
57:00:00	3hf4-F	3.5	4.2	84	146	7	MOLECULE:	HEMOGLOBIN
58:00:00	1f65-A	3.5	4.1	84	154	10	MOLECULE:	MYOGLOBIN
59:00:00	1n9x-A	3.5	4.1	84	154	10	MOLECULE:	MYOGLOBIN
60:00:00	3dht-B	3.5	4.2	84	145	7	MOLECULE:	HEMOGLOBIN
61:00:00	3a2g-A	3.5	4.1	84	154	11	MOLECULE:	MYOGLOBIN
62:00:00	1mlh-A	3.5	3.9	84	154	11	MOLECULE:	MYOGLOBIN
63:00:00	2e2y-A	3.5	4.1	84	154	10	MOLECULE:	MYOGLOBIN
64:00:00	1n9f-A	3.5	4.1	84	154	10	MOLECULE:	MYOGLOBIN
65:00:00	1naz-A	3.5	3.7	79	154	10	MOLECULE:	MYOGLOBIN
66:00:00	2nx0-A	3.5	3.9	78	146	5	MOLECULE:	MYOGLOBIN
67:00:00	3ia3-B	3.5	3	79	135	8	MOLECULE:	ALPHA-HEMOG
68:00:00	6c94-A	3.4	8.5	109	482	5	MOLECULE:	CYTOCHROME
69:00:00	8j5t-C	3.4	4.1	100	288	12	MOLECULE:	UNCHARACTER
70:00:00	6z3w-F	3.4	7.6	78	110	1	MOLECULE:	ER MEMBRANE
71:00:00	8iic-B	3.4	6.5	128	425	5	MOLECULE:	POLYMERASE
72:00:00	7wji-A	3.4	24.5	100	1763	11	MOLECULE:	PROTEIN UNC
73:00:00	4p96-A	3.4	4.5	103	278	8	MOLECULE:	FATTY ACID
74:00:00	5zle-A	3.4	5.8	98	225	7	MOLECULE:	CYTOCHROME
75:00:00	5yo8-A	3.4	6.4	91	349	5	MOLECULE:	TETRAPRENYL
76:00:00	2mjm-A	3.4	3.6	67	101	4	MOLECULE:	PROTEIN NLR
77:00:00	7v9g-D	3.4	10.5	77	112	9	MOLECULE:	BEN DOMAIN-
78:00:00	7tt9-A	3.4	4.7	71	116	8	MOLECULE:	GROUP 1 TRU

79:00:00	6g2j-K	3.4	6.1	82	98	6	MOLECULE:	NADH-UBIQUI
80:00:00	2pke-A	3.4	5.2	82	233	5	MOLECULE:	HALOACID DE
81:00:00	7w5g-A	3.4	16.2	69	355	1	MOLECULE:	TERPENE CYC
82:00:00	3jc6-5	3.4	4.3	81	254	5	MOLECULE:	DNA REPLICA
83:00:00	4q9v-B	3.4	6.7	94	186	16	MOLECULE:	TUMOR NECRO
84:00:00	6uz3-A	3.4	24.7	108	1126	6	MOLECULE:	SODIUM CHAN
85:00:00	4jle-B	3.4	10.3	73	159	3	MOLECULE:	PHIST
86:00:00	6e8c-A	3.4	5.6	65	135	6	MOLECULE:	DOUBLE HOME
87:00:00	2py8-C	3.4	7.8	79	123	5	MOLECULE:	HYPOTHETICA
88:00:00	8e9g-L	3.4	21.2	113	625	10	MOLECULE:	TWO-COMPONE
89:00:00	4h0b-A	3.4	4.1	84	154	10	MOLECULE:	MYOGLOBIN
90:00:00	3hrw-B	3.4	4	81	146	6	MOLECULE:	HEMOGLOBIN
91:00:00	3bj2-A	3.4	3.5	80	142	10	MOLECULE:	HEMOGLOBIN
92:00:00	5eui-A	3.4	3.2	76	134	7	MOLECULE:	HBA PROTEIN
93:00:00	1ury-B	3.4	4.5	86	154	5	MOLECULE:	CYTOGLOBIN
94:00:00	4pnj-A	3.4	4	84	154	10	MOLECULE:	MYOGLOBIN
95:00:00	3a59-D	3.4	4.1	82	146	7	MOLECULE:	HEMOGLOBIN
96:00:00	6on4-B	3.4	4	89	214	8	MOLECULE:	HTH-TYPE TR
97:00:00	5hy8-E	3.4	3.7	80	137	6	MOLECULE:	HEMOGLOBIN
98:00:00	1f5o-E	3.4	3.5	77	149	6	MOLECULE:	HEMOGLOBIN
99:00:00	1jeb-B	3.4	4	82	143	7	MOLECULE:	HEMOGLOBIN
0:00	3lhb-A	3.4	3.1	76	149	5	MOLECULE:	PROTEIN (HE
1:00	3lhb-F	3.4	3.6	78	149	6	MOLECULE:	PROTEIN (HE
2:00	1f5p-F	3.4	3.6	78	149	5	MOLECULE:	HEMOGLOBIN
3:00	3lhb-D	3.4	3.6	78	149	6	MOLECULE:	PROTEIN (HE
4:00	1uc3-K	3.4	3.6	78	149	5	MOLECULE:	GLOBIN
5:00	3lhb-H	3.4	3.1	76	149	5	MOLECULE:	PROTEIN (HE
6:00	2h35-C	3.4	3.4	82	141	6	MOLECULE:	HEMOGLOBIN
7:00	3lhb-G	3.4	3.5	77	149	6	MOLECULE:	PROTEIN (HE
8:00	3lhb-K	3.4	3.1	76	149	5	MOLECULE:	PROTEIN (HE
9:00	1f5p-A	3.4	3.6	78	149	5	MOLECULE:	HEMOGLOBIN
10:00	3lhb-J	3.4	3.6	78	149	6	MOLECULE:	PROTEIN (HE
11:00	3lhb-I	3.4	3.1	76	149	5	MOLECULE:	PROTEIN (HE
12:00	3lhb-B	3.4	3.6	78	149	6	MOLECULE:	PROTEIN (HE
13:00	1f5p-D	3.4	3.6	78	149	5	MOLECULE:	HEMOGLOBIN
14:00	1f5p-C	3.4	3.6	78	149	5	MOLECULE:	HEMOGLOBIN
15:00	1f5p-B	3.4	3.6	78	149	5	MOLECULE:	HEMOGLOBIN
16:00	3lhb-L	3.4	3.5	77	149	6	MOLECULE:	PROTEIN (HE
17:00	7yim-A	3.3	15.7	84	592	4	MOLECULE:	ALPHA-FETOP
18:00	5nvu-A	3.3	27.7	130	3169	0	MOLECULE:	DYNEIN MOTO
19:00	7uy5-K	3.3	4	58	157	5	MOLECULE:	TELOMERASE
20:00	5w65-P	3.3	7	78	481	10	MOLECULE:	DNA-DIRECTE
21:00	4pi0-A	3.3	18.6	85	390	15	MOLECULE:	UNKNOWN PEP
22:00	6ewz-A	3.3	6.6	71	202	3	MOLECULE:	GTP PYROPHO

23:00	7exx-A	3.3	12.1	74	440	4	MOLECULE:	DNA PHOSPHO
24:00:00	6vls-D	3.3	32.5	131	963	8	MOLECULE:	MALTOSE/MAL
25:00:00	8arl-A	3.3	14.2	84	232	8	MOLECULE:	TRYPTOPHAN-
26:00:00	7uic-b	3.3	9.6	56	65	9	MOLECULE:	MEDIATOR OF
27:00:00	3g06-A	3.3	20.7	87	601	7	MOLECULE:	SSPH2 (LEUC
28:00:00	8bpz-A	3.3	16.3	68	322	9	MOLECULE:	SPBETA PROP
29:00:00	5gzb-A	3.3	3.6	66	91	6	MOLECULE:	TRANSCRIPTI
30:00:00	6br8-A	3.3	8.3	81	249	7	MOLECULE:	PROTEIN A6
31:00:00	2f4q-A	3.3	4.2	72	309	3	MOLECULE:	TYPE I TOPO
32:00:00	7dgu-A	3.3	4.7	71	97	6	MOLECULE:	A1
33:00:00	3p3o-A	3.3	5.8	150	416	7	MOLECULE:	CYTOCHROME
34:00:00	6wq0-A	3.3	7.4	73	131	3	MOLECULE:	DNA (301-ME
35:00:00	6xdc-A	3.3	16.3	98	193	7	MOLECULE:	PROTEIN 3A
36:00:00	3ua3-A	3.3	17.6	124	663	3	MOLECULE:	PROTEIN ARG
37:00:00	7bc6-A	3.3	7	95	432	5	MOLECULE:	PROTON-COUP
38:00:00	7oqz-A	3.3	12.6	67	237	4	MOLECULE:	TRANSMEMBRA
39:00:00	6zgd-A	3.3	6.2	76	288	12	MOLECULE:	PROTON-GATE
40:00:00	8k49-T	3.3	18.9	106	627	7	MOLECULE:	VP2
41:00:00	7dgy-A	3.3	3.8	70	99	3	MOLECULE:	C2
42:00:00	3h36-A	3.3	4.1	68	78	6	MOLECULE:	POLYRIBONUC
43:00:00	8b9z-K	3.3	7.8	79	96	11	MOLECULE:	NADH-UBIQUI
44:00:00	7qe7-A	3.3	17.9	135	1648	5	MOLECULE:	ANAPHASE-PR
45:00:00	7ar9-J	3.3	8.6	87	145	3	MOLECULE:	ND3
46:00:00	7mhz-A	3.3	8.7	102	490	4	MOLECULE:	SONIC HEDGE
47:00:00	7mi6-A	3.3	42.1	127	2419	7	MOLECULE:	FUSION PROT
48:00:00	2gb7-B	3.3	19.1	79	295	4	MOLECULE:	DNA STRAND
49:00:00	1ux9-B	3.3	4.5	86	154	5	MOLECULE:	CYTOGLOBIN
50:00:00	2h35-A	3.3	3.3	80	141	5	MOLECULE:	HEMOGLOBIN
51:00:00	1f5o-C	3.3	3.1	76	149	5	MOLECULE:	HEMOGLOBIN
52:00:00	1jeb-D	3.3	4.6	84	146	7	MOLECULE:	HEMOGLOBIN
53:00:00	1f5o-A	3.3	3.1	76	149	5	MOLECULE:	HEMOGLOBIN
54:00:00	1f5o-B	3.3	3.1	76	149	5	MOLECULE:	HEMOGLOBIN
55:00:00	1uc3-I	3.3	3.1	76	149	5	MOLECULE:	GLOBIN
56:00:00	1f5o-D	3.3	3.5	77	149	6	MOLECULE:	HEMOGLOBIN
57:00:00	1uc3-H	3.3	3.6	78	149	5	MOLECULE:	GLOBIN
58:00:00	3lhb-C	3.3	3.6	78	149	6	MOLECULE:	PROTEIN (HE
59:00:00	5lcw-N	3.3	17.4	90	703	11	MOLECULE:	ANAPHASE-PR
60:00:00	2nrm-A	3.3	4	76	146	5	MOLECULE:	MYOGLOBIN
61:00:00	3k3u-B	3.2	3.7	74	137	9	MOLECULE:	DEHALOPEROX
62:00:00	7a7c-A	3.2	16.6	100	560	8	MOLECULE:	CATALASE-PE
63:00:00	1zs4-A	3.2	3	45	82	7	MOLECULE:	DNA - 27MER
64:00:00	5oxf-C	3.2	22.1	106	601	10	MOLECULE:	GTP-BINDING
65:00:00	6al9-B	3.2	3.1	42	91	2	MOLECULE:	CHORISMATE
66:00:00	5k94-A	3.2	13.8	99	506	6	MOLECULE:	MALTOSE-BIN

67:00:00	6fwr-A	3.2	21.8	89	699	3	MOLECULE:	ATP-DEPENDE
68:00:00	1kw2-A	3.2	7.4	76	455	12	MOLECULE:	VITAMIN D-B
69:00:00	8v46-D	3.2	3.6	87	305	6	MOLECULE:	ARIA ANTITO
70:00:00	8ieu-B	3.2	14.9	75	336	9	MOLECULE:	DUF2891 DOM
71:00:00	2hs5-A	3.2	4.7	97	207	7	MOLECULE:	PUTATIVE TR
72:00:00	8v3b-a	3.2	12.3	79	416	3	MOLECULE:	O43_129_+4
73:00:00	6w2r-B	3.2	6.5	73	221	8	MOLECULE:	JUNCTION 19
74:00:00	6ebu-A	3.2	4.4	88	165	8	MOLECULE:	LPXE
75:00:00	1q2h-A	3.2	4.3	57	63	7	MOLECULE:	ADAPTOR PRO
76:00:00	3a6m-A	3.2	6.1	68	168	4	MOLECULE:	PROTEIN GRP
77:00:00	7ctp-A	3.2	5	102	553	12	MOLECULE:	PROTEIN NIB
78:00:00	5z1n-A	3.2	3.9	83	166	11	MOLECULE:	G-PROTEIN I
79:00:00	5jm8-A	3.2	20.6	68	556	3	MOLECULE:	AEROBACTIN
80:00:00	5j6f-A	3.2	16.6	59	352	7	MOLECULE:	3-DEOXY-D-A
81:00:00	1r5i-D	3.2	10	87	214	3	MOLECULE:	HLA CLASS I
82:00:00	6zyd-A	3.2	21.9	121	323	5	MOLECULE:	LOW CONDUCT
83:00:00	4gr6-B	3.2	3.9	71	105	7	MOLECULE:	ATRBCX2
84:00:00	5cd4-I	3.2	10	82	494	9	MOLECULE:	CRISPR SYST
85:00:00	3ed5-A	3.2	6.1	96	232	7	MOLECULE:	YFNB
86:00:00	8j3r-B	3.2	25.7	115	387	3	MOLECULE:	TRANSPOSASE
87:00:00	8uw3-A	3.2	7.2	109	1265	6	MOLECULE:	LINE-1 RETR
88:00:00	4zsf-A	3.2	17.6	99	272	5	MOLECULE:	BSAWI ENDON
89:00:00	3bzk-A	3.2	13.4	92	728	7	MOLECULE:	TEX
90:00:00	3bni-A	3.2	5.2	67	174	9	MOLECULE:	PUTATIVE TE
91:00:00	3bm3-A	3.2	4.8	90	259	4	MOLECULE:	DNA (5'-D(*
92:00:00	8a3t-C	3.2	26.7	134	1410	10	MOLECULE:	ANAPHASE-PR
93:00:00	5d2m-C	3.2	4.6	83	158	17	MOLECULE:	SUMO-CONJUG
94:00:00	3dan-A	3.2	6.7	106	473	2	MOLECULE:	CYTOCHROME
95:00:00	6dlu-P	3.2	8.3	95	747	5	MOLECULE:	DYNAMIN-1
96:00:00	6zyy-C	3.2	8.4	90	2901	6	MOLECULE:	DYNEIN HEAV
97:00:00	4hkr-A	3.2	8.8	89	165	2	MOLECULE:	CALCIUM REL
98:00:00	3vr8-D	3.2	8.5	84	129	7	MOLECULE:	FLAVOPROTEI
99:00:00	8ity-V	3.2	13.8	80	361	9	MOLECULE:	SNRNA-ACTIV
0:00	8ovw-Z	3.2	21.5	93	151	8	MOLECULE:	CENTROMERE-
1:00	7ryq-B	3.2	15.6	93	527	6	MOLECULE:	KIF-BINDING
2:00	7kfu-C	3.2	19.3	137	909	7	MOLECULE:	CAS2
3:00	6khj-E	3.2	7.2	80	101	9	MOLECULE:	NAD(P)H-QUI
4:00	3w6j-A	3.2	5.3	101	150	8	MOLECULE:	SCPA
5:00	5lbm-C	3.2	5.7	70	90	7	MOLECULE:	TRANSCRIPTI
6:00	1uc3-J	3.2	3.5	76	149	5	MOLECULE:	GLOBIN
7:00	3ia3-D	3.2	3.6	82	135	5	MOLECULE:	ALPHA-HEMOG
8:00	6otx-A	3.2	4.2	78	151	4	MOLECULE:	HEMOGLOBIN
9:00	4hrr-F	3.2	4.1	87	151	7	MOLECULE:	GLOBIN-2 A
10:00	1s5y-C	3.2	4.2	81	142	7	MOLECULE:	HEMOGLOBIN

11:00	1uc3-D	3.2	4.2	81	149	7	MOLECULE:	GLOBIN
12:00	3lhb-E	3.2	4.1	80	149	8	MOLECULE:	PROTEIN (HE
13:00	6tlj-N	3.2	19.8	99	703	7	MOLECULE:	ANAPHASE-PR
14:00	1f5o-F	3.2	4.1	80	149	8	MOLECULE:	HEMOGLOBIN
15:00	1uc3-G	3.2	3.6	78	149	6	MOLECULE:	GLOBIN
16:00	7z0o-G	3.1	2.3	53	84	8	MOLECULE:	HISTONE H3
17:00	7vvy-K	3.1	31.3	90	237	8	MOLECULE:	CHROMATIN M
18:00	8pnj-A	3.1	5.2	58	390	9	MOLECULE:	CHORISMATE
19:00	6zpa-A	3.1	4.1	72	173	10	MOLECULE:	DATZ
20:00	7xdi-F	3.1	6.6	69	456	6	MOLECULE:	VP1
21:00	8ea3-W	3.1	20.6	101	514	7	MOLECULE:	TARGET_LE
22:00	4fcy-B	3.1	24.5	100	476	2	MOLECULE:	TRANSPOSASE
23:00	1ysm-A	3.1	4.5	43	55	9	MOLECULE:	CALCYCLIN-B
24:00:00	3f1i-H	3.1	19.9	77	98	13	MOLECULE:	HEPATOCYTE
25:00:00	8fuq-A	3.1	4.1	40	51	10	MOLECULE:	PHOSPHOPROT
26:00:00	1m12-A	3.1	2.9	57	84	7	MOLECULE:	SAPOSIN C
27:00:00	7ro2-A	3.1	10.4	70	94	6	MOLECULE:	MAJOR CAPSI
28:00:00	1xdo-A	3.1	20.3	103	687	8	MOLECULE:	POLYPHOSPHA
29:00:00	5szj-B	3.1	9.5	85	145	5	MOLECULE:	RAS-RELATED
30:00:00	6o3s-A	3.1	5.8	44	47	5	MOLECULE:	RIBOSOME-IN
31:00:00	7s00-c	3.1	14.3	67	484	9	MOLECULE:	DNA-DIRECTE
32:00:00	7ymd-C	3.1	11.4	78	285	9	MOLECULE:	NON-STRUCTU
33:00:00	7pq1-A	3.1	5.8	130	395	8	MOLECULE:	CYTOCHROME
34:00:00	7pkq-C	3.1	25.3	71	369	6	MOLECULE:	MS35
35:00:00	6gys-B	3.1	10.3	105	579	10	MOLECULE:	CENTROMERE
36:00:00	7emf-J	3.1	16.2	67	122	6	MOLECULE:	MEDIATOR OF
37:00:00	1knz-A	3.1	9.6	56	154	7	MOLECULE:	5'-R(*UP*GP
38:00:00	6wc3-B	3.1	8.3	67	94	15	MOLECULE:	PROTEIN TRA
39:00:00	7wst-A	3.1	11.1	110	601	2	MOLECULE:	IRON-PHYTOS
40:00:00	7lma-A	3.1	23.2	145	1012	6	MOLECULE:	TELOMERASE
41:00:00	8j2x-A	3.1	4.1	77	337	10	MOLECULE:	COBALAMIN-B
42:00:00	6lum-D	3.1	10.4	91	146	8	MOLECULE:	SUCCINATE D
43:00:00	5yhf-A	3.1	5.6	107	734	9	MOLECULE:	PROTEIN TRA
44:00:00	7zmb-6	3.1	10.5	102	190	10	MOLECULE:	NADH-UBIQUI
45:00:00	7wuq-R	3.1	9.7	84	278	4	MOLECULE:	GUANINE NUC
46:00:00	5e6g-B	3.1	10.4	75	114	9	MOLECULE:	DE NOVO DES
47:00:00	7ot9-A	3.1	11.9	84	221	7	MOLECULE:	AI-2E MEMBE
48:00:00	6k5s-A	3.1	11	70	220	7	MOLECULE:	TELOMERIC D
49:00:00	7op8-A	3.1	14.1	88	1118	3	MOLECULE:	CATION-TRAN
50:00:00	7mxb-A	3.1	8.1	62	672	8	MOLECULE:	SERINE/THRE
51:00:00	6ysg-A	3.1	17.8	126	1264	4	MOLECULE:	MG-CHELATAS
52:00:00	8uc3-C	3.1	7.4	70	98	3	MOLECULE:	ALBONOURSIN
53:00:00	6zvh-R	3.1	14.8	78	135	6	MOLECULE:	18S RRNA
54:00:00	5iy7-M	3.1	11.1	76	310	9	MOLECULE:	DNA-DIRECTE

55:00:00	5w7g-B	3.1	9.5	66	126	8	MOLECULE:	ORF140
56:00:00	5ys9-A	3.1	23.5	132	692	5	MOLECULE:	ACYL-COENZY
57:00:00	6d5f-A	3.1	10.4	82	193	10	MOLECULE:	FIMBRIAL PR
58:00:00	7d3u-G	3.1	8.8	87	111	7	MOLECULE:	MONOVALENT
59:00:00	6rao-I	3.1	19.6	91	443	4	MOLECULE:	AFP1
60:00:00	4y9j-B	3.1	13.7	108	593	3	MOLECULE:	PROTEIN ACD
61:00:00	5w7g-A	3.1	7.7	67	131	4	MOLECULE:	ORF140
62:00:00	3waq-A	3.1	6.9	78	134	8	MOLECULE:	HEMERYTHRIN
63:00:00	2wus-R	3.1	2.9	60	87	5	MOLECULE:	PUTATIVE UN
64:00:00	8c3y-A	3.1	5.7	105	929	2	MOLECULE:	PFEMP1
65:00:00	2rld-C	3.1	5	78	116	4	MOLECULE:	UNCHARACTER
66:00:00	3hc1-A	3.1	4.9	80	298	10	MOLECULE:	UNCHARACTER
67:00:00	7vw0-B	3.1	8.6	76	94	11	MOLECULE:	DUF305 DOMA
68:00:00	3pi2-A	3.1	4.6	78	151	5	MOLECULE:	HEMOGLOBIN
69:00:00	2nrl-A	3.1	4.3	76	145	5	MOLECULE:	MYOGLOBIN
70:00:00	1f6h-A	3.1	4.1	82	153	11	MOLECULE:	MYOGLOBIN (
71:00:00	7vde-D	3.1	4.6	81	146	7	MOLECULE:	HEMOGLOBIN
72:00:00	7vde-B	3.1	4.6	81	146	7	MOLECULE:	HEMOGLOBIN
73:00:00	1uc3-L	3.1	3.6	78	149	6	MOLECULE:	GLOBIN
74:00:00	1yeq-D	3.1	3.5	84	146	10	MOLECULE:	HEMOGLOBIN
75:00:00	8w6c-A	3	17	92	530	11	MOLECULE:	SOLUTE CARR
76:00:00	6hqa-J	3	3.3	45	51	4	MOLECULE:	TAF2
77:00:00	5dw7-A	3	4.1	79	309	8	MOLECULE:	GERMACRADIE
78:00:00	4wfc-B	3	12.9	81	118	2	MOLECULE:	EXOSOME COM
79:00:00	2e87-A	3	12.2	91	356	5	MOLECULE:	HYPOTHETICA
80:00:00	2btv-B	3	25.2	86	885	8	MOLECULE:	PROTEIN (VP
81:00:00	6vz1-A	3	11.1	90	411	13	MOLECULE:	DIACYLGLYCE
82:00:00	6kg7-A	3	15.7	130	1817	5	MOLECULE:	PIEZO-TYPE
83:00:00	7u5d-A	3	18.2	70	630	6	MOLECULE:	CRRNA
84:00:00	7x07-A	3	11.9	122	633	5	MOLECULE:	ATP-BINDING
85:00:00	7x2q-A	3	5.7	153	467	4	MOLECULE:	SUGIOL SYNT
86:00:00	6v4a-C	3	14.4	63	593	5	MOLECULE:	NEUR_CHAN_L
87:00:00	4hts-A	3	5.1	89	228	9	MOLECULE:	SEC-INDEPEN
88:00:00	8eja-A	3	5.4	63	113	6	MOLECULE:	ABAK
89:00:00	8djk-A	3	9.1	108	245	4	MOLECULE:	3-HYDROXY-3
90:00:00	8tr4-A	3	13.8	60	276	5	MOLECULE:	POLYKETIDE
91:00:00	7ojn-L	3	30.8	143	2010	7	MOLECULE:	RNA-DIRECTE
92:00:00	3tix-A	3	6.3	84	153	8	MOLECULE:	UBIQUITIN-L
93:00:00	3mlg-A	3	5.9	76	161	9	MOLECULE:	2X CHIMERA
94:00:00	7n8x-A	3	10.5	77	691	5	MOLECULE:	CHONDROITIN
95:00:00	7xr3-Z	3	13.4	114	1320	5	MOLECULE:	VP3
96:00:00	2ouw-B	3	4.6	60	136	12	MOLECULE:	ALKYLHYDROP
97:00:00	8q85-a	3	21.8	94	234	5	MOLECULE:	KINETOCHORE
98:00:00	8bou-A	3	19.1	100	807	4	MOLECULE:	N,N'-DIACET

99:00:00	2r6i-A	3	16.4	66	268	8	MOLECULE:	UNCHARACTER
0:00	7lhf-A	3	22.1	98	2123	4	MOLECULE:	INOSITOL 1,
1:00	6pvp-A	3	17	78	661	6	MOLECULE:	TRANSIENT R
2:00	6cfw-A	3	10.4	69	165	9	MOLECULE:	MONOVALENT
3:00	6hsy-A	3	4.4	55	190	2	MOLECULE:	TOLUENE TOL
4:00	5e2q-A	3	12.4	98	725	7	MOLECULE:	DIPEPTIDYL
5:00	1g8x-A	3	18.5	103	1009	3	MOLECULE:	MYOSIN II H
6:00	5f3o-A	3	6.3	82	194	9	MOLECULE:	PUTATIVE UN
7:00	5ean-A	3	20.1	110	1051	7	MOLECULE:	DNA REPLICA
8:00	3dp7-A	3	7.9	76	351	8	MOLECULE:	SAM-DEPENDE
9:00	8jpj-A	3	14.7	125	733	7	MOLECULE:	H(+)/CL(-)
10:00	7lzh-A	3	35.9	104	799	4	MOLECULE:	GLUTAMATE R
11:00	3i4m-D	3	7	76	187	7	MOLECULE:	DNA-DIRECTE
12:00	7w6k-A	3	28.3	119	410	4	MOLECULE:	GMALMT12/QU
13:00	6uak-A	3	3.8	74	298	3	MOLECULE:	SAM DEPENDE
14:00	7vu7-A	3	3.9	64	89	3	MOLECULE:	FLAGELLIFOR
15:00	7m5w-A	3	9.5	69	138	12	MOLECULE:	PROTEIN CAP
16:00	8ifg-A	3	16.3	90	879	11	MOLECULE:	RBAP48-RELA
17:00	3f7c-A	3	4.3	64	199	8	MOLECULE:	PROTEIN OF
18:00	6cfw-G	3	8.2	79	113	8	MOLECULE:	MONOVALENT
19:00	5a31-N	3	12	72	703	13	MOLECULE:	ANAPHASE-PR
20:00	2q5z-B	3	5.8	65	94	6	MOLECULE:	HYPOTHETICA
21:00	2qup-A	3	8	79	119	5	MOLECULE:	BH1478 PROT
22:00	5lnk-J	3	10.3	79	175	6	MOLECULE:	MITOCHONDRI
23:00	6xgx-A	3	20.6	103	402	8	MOLECULE:	MUTATOR FAM
24:00:00	3mzy-A	3	2.5	51	123	16	MOLECULE:	RNA POLYMER
25:00:00	7m2w-G	3	10.4	89	712	7	MOLECULE:	TUBULIN GAM
26:00:00	1jog-D	3	7.6	77	136	6	MOLECULE:	HYPOTHETICA
27:00:00	1gzs-B	3	3.3	88	165	10	MOLECULE:	SOPE
28:00:00	8qq7-A	3	10.8	100	399	9	MOLECULE:	FAD-BINDING
29:00:00	6r6h-O	3	20.8	124	744	6	MOLECULE:	COP9 SIGNAL
30:00:00	8pm6-A	3	15.5	88	872	8	MOLECULE:	BILE SALT E
31:00:00	2cqn-A	3	3.6	62	77	0	MOLECULE:	FORMIN-BIND
32:00:00	7tmw-R	3	11.4	113	499	9	MOLECULE:	RELAXIN REC
33:00:00	7woo-Z	3	16.7	81	746	2	MOLECULE:	NUCLEOPORIN
34:00:00	1mti-A	3	4.9	78	154	10	MOLECULE:	MYOGLOBIN
35:00:00	1mlg-A	3	5	78	154	12	MOLECULE:	MYOGLOBIN
36:00:00	3wyo-D	3	10.6	72	153	11	MOLECULE:	MYOGLOBIN
37:00:00	1i3d-B	3	4.4	80	146	10	MOLECULE:	HEMOGLOBIN
38:00:00	3wtg-D	3	4.7	83	146	13	MOLECULE:	HEMOGLOBIN
39:00:00	1ch5-A	3	4.8	76	154	11	MOLECULE:	PROTEIN (MY
40:00:00	5g04-N	3	28.1	113	631	5	MOLECULE:	ANAPHASE-PR
41:00:00	4hrr-D	3	3.7	85	151	9	MOLECULE:	GLOBIN-2 A
42:00:00	2di3-B	3	6.8	99	230	7	MOLECULE:	BACTERIAL R

43:00:00	6q6g-N	3	32	122	656	6	MOLECULE:	CELL DIVISI
44:00:00	7dgn-B	3	10.1	72	153	7	MOLECULE:	MYOGLOBIN
45:00:00	7dgl-A	3	13.3	60	153	7	MOLECULE:	MYOGLOBIN
46:00:00	3wyo-A	3	7.1	54	153	2	MOLECULE:	MYOGLOBIN
47:00:00	1a4f-B	3	3.6	85	146	11	MOLECULE:	HEMOGLOBIN
48:00:00	6wg7-G	3	12.2	94	219	2	MOLECULE:	DNA (35-MER
49:00:00	7k4m-F	3	3.7	85	146	8	MOLECULE:	HEMOGLOBIN
50:00:00	5x2r-B	3	3.3	79	145	10	MOLECULE:	HEMOGLOBIN
51:00:00	7uf7-B	3	3.5	84	146	8	MOLECULE:	HEMOGLOBIN
52:00:00	2vyw-A	2.9	3.9	78	148	4	MOLECULE:	HEMOGLOBIN
53:00:00	5flv-M	2.9	7.1	68	229	7	MOLECULE:	HOMEOBOX PR
54:00:00	8a5a-U	2.9	19.8	88	690	6	MOLECULE:	CHROMATIN-R
55:00:00	6nd4-L	2.9	13.5	91	473	4	MOLECULE:	ETS RRNA
56:00:00	7b1s-D	2.9	3.7	93	593	3	MOLECULE:	ETHYL-COENZ
57:00:00	6h4c-D	2.9	4.4	65	148	0	MOLECULE:	DUTPASE
58:00:00	4kp3-D	2.9	12.8	52	82	10	MOLECULE:	UNCONVENTIO
59:00:00	3nmz-A	2.9	23	89	406	7	MOLECULE:	APC VARIANT
60:00:00	5zr1-E	2.9	26.5	70	460	6	MOLECULE:	ORIGIN RECO
61:00:00	4geh-D	2.9	3.6	48	69	4	MOLECULE:	PROGRAMMED
62:00:00	7kpv-B	2.9	9.3	80	290	10	MOLECULE:	MEIOTIC MRN
63:00:00	4wid-A	2.9	7.3	66	353	5	MOLECULE:	RHUL123
64:00:00	6nr8-6	2.9	3.6	41	102	15	MOLECULE:	PREFOLDIN S
65:00:00	8qrl-1	2.9	12	75	278	7	MOLECULE:	12S MITOCHO
66:00:00	5wwl-N	2.9	15.8	61	155	3	MOLECULE:	CENTROMERE
67:00:00	6ec8-A	2.9	11.2	65	803	8	MOLECULE:	LANTIBIOTIC
68:00:00	1x8z-A	2.9	5.3	81	151	9	MOLECULE:	INVERTASE/P
69:00:00	3lsg-A	2.9	3.6	57	103	7	MOLECULE:	TWO-COMPONE
70:00:00	3hmj-A	2.9	10	103	1750	10	MOLECULE:	FATTY ACID
71:00:00	6z6o-D	2.9	19.9	73	542	4	MOLECULE:	HISTONE DEA
72:00:00	2d1l-A	2.9	21.9	101	249	5	MOLECULE:	METASTASIS
73:00:00	6oap-A	2.9	13.2	105	305	12	MOLECULE:	DUAL SENSOR
74:00:00	1whu-A	2.9	6	72	104	10	MOLECULE:	POLYNUCLEOT
75:00:00	7o4i-1	2.9	12.9	68	522	6	MOLECULE:	GENERAL TRA
76:00:00	5j0l-A	2.9	9.3	64	130	6	MOLECULE:	DESIGNED PR
77:00:00	7w7c-B	2.9	4.9	73	159	10	MOLECULE:	PUTATIVE AB
78:00:00	7ml0-1	2.9	16.9	64	367	5	MOLECULE:	DNA-DIRECTE
79:00:00	8r33-A	2.9	6.1	111	489	5	MOLECULE:	PHO90 ISOFO
80:00:00	6g18-y	2.9	14.9	70	325	7	MOLECULE:	PRE-18S RIB
81:00:00	6e11-E	2.9	11.3	103	210	5	MOLECULE:	UNKNOWN (CL
82:00:00	8hdu-B	2.9	5.5	55	162	11	MOLECULE:	DE NOVO DES
83:00:00	7lt1-A	2.9	12.6	100	403	4	MOLECULE:	PROTEIN MB2
84:00:00	4v2o-B	2.9	7.4	51	81	8	MOLECULE:	SAPOSIN-B
85:00:00	8th8-H	2.9	12.1	61	820	5	MOLECULE:	DYNEIN REGU
86:00:00	1q1v-A	2.9	3.8	63	70	3	MOLECULE:	DEK PROTEIN

87:00:00	7elb-A	2.9	29.3	121	1937	7	MOLECULE:	RNA-DIRECTE
88:00:00	8j22-C	2.9	6.6	88	285	6	MOLECULE:	GUANINE NUC
89:00:00	8kee-C	2.9	8.4	104	505	4	MOLECULE:	SHEATH
90:00:00	7kmt-B	2.9	11.7	87	475	5	MOLECULE:	TRAFFICKING
91:00:00	7aqq-N	2.9	12.6	92	488	3	MOLECULE:	NADH-UBIQUI
92:00:00	6nps-A	2.9	18.9	102	968	5	MOLECULE:	AXYAGU115A
93:00:00	6csv-A	2.9	11.9	74	90	5	MOLECULE:	CENTROSOMAL
94:00:00	6s6b-B	2.9	3.6	76	154	7	MOLECULE:	CRISPR-ASSO
95:00:00	6ud5-D	2.9	10.5	77	355	6	MOLECULE:	TRYPTOPHAN
96:00:00	5hzi-A	2.9	9.8	96	476	5	MOLECULE:	INTERSECTIN
97:00:00	5fhp-C	2.9	13.1	67	209	9	MOLECULE:	NICR
98:00:00	8d4y-B	2.9	14.9	80	217	11	MOLECULE:	CHROMODOMAI
99:00:00	2qwt-A	2.9	8.1	65	167	6	MOLECULE:	TRANSCRIPTI
0:00	8wt9-A	2.9	11.2	86	319	9	MOLECULE:	IS621 TRANS
1:00	5oqj-W	2.9	21.5	83	258	5	MOLECULE:	DNA-DIRECTE
2:00	7eeb-K	2.9	15.9	91	143	5	MOLECULE:	ENHANCED GR
3:00	5c8j-L	2.9	8.7	71	137	8	MOLECULE:	ANTIBODY FR
4:00	6ejq-B	2.9	14.9	72	141	8	MOLECULE:	TERMINASE S
5:00	8ap7-j	2.9	11	78	168	4	MOLECULE:	ATP SYNTHAS
6:00	8ttf-A	2.9	12.6	89	388	8	MOLECULE:	QUINOLONE R
7:00	2dh4-A	2.9	11.8	92	326	1	MOLECULE:	YPL069C
8:00	7oca-E	2.9	10.8	93	158	6	MOLECULE:	GLUTAMATE R
9:00	7ody-C	2.9	5.1	69	100	13	MOLECULE:	MAZG-LIKE P
10:00	6yxq-A	2.9	17.6	64	188	6	MOLECULE:	ACTIVATING
11:00	8fed-J	2.9	3.5	89	263	1	MOLECULE:	VIRULENCE F
12:00	7x3t-V	2.9	14.6	93	792	8	MOLECULE:	HISTONE H3
13:00	6w6x-A	2.9	7.8	77	126	8	MOLECULE:	DE NOVO DES
14:00	6kex-A	2.9	3.6	59	349	5	MOLECULE:	PHOSPHORIBU
15:00	5ajk-A	2.9	5.7	72	148	4	MOLECULE:	HOMOLOG OF
16:00	6bhp-A	2.9	4.7	78	170	8	MOLECULE:	MEMBRANE PR
17:00	7t7t-A	2.9	10.4	84	466	4	MOLECULE:	PROTEIN TON
18:00	6wtw-A	2.9	12.3	95	489	5	MOLECULE:	DASS FAMILY
19:00	8axa-A	2.9	22	118	465	1	MOLECULE:	CAS12K
20:00	6swy-9	2.9	5.5	59	157	12	MOLECULE:	VACUOLAR IM
21:00	8p0w-A	2.9	19.1	73	190	12	MOLECULE:	COMM DOMAIN
22:00	8ucq-A	2.9	23.9	105	1232	9	MOLECULE:	SEC7 DOMAIN
23:00	7wb4-I	2.9	33.6	118	796	3	MOLECULE:	OUTER NUP13
24:00:00	4raf-A	2.9	8.9	85	362	7	MOLECULE:	PROTEIN PHO
25:00:00	6elu-A	2.9	5.5	74	193	12	MOLECULE:	SERUM RESIS
26:00:00	5z58-1	2.9	20.5	108	1038	6	MOLECULE:	PRE-MRNA-PR
27:00:00	5unk-A	2.9	3.6	53	62	9	MOLECULE:	SLEEPING BE
28:00:00	5v2z-A	2.9	10.3	64	350	2	MOLECULE:	2-OXOGLUTAR
29:00:00	8hf3-A	2.9	12.9	106	295	7	MOLECULE:	PALMITOYLTR
30:00:00	2z3r-E	2.9	4.5	75	119	4	MOLECULE:	INTERLEUKIN

31:00:00	4kha-A	2.9	12.1	78	360	8	MOLECULE:	SPT16M-HIST
32:00:00	7jr7-A	2.9	5.5	86	588	7	MOLECULE:	ATP-BINDING
33:00:00	4f9k-B	2.9	3.2	46	58	2	MOLECULE:	CAMP-DEPEND
34:00:00	2hjm-A	2.9	5.5	69	91	4	MOLECULE:	HYPOTHETICA
35:00:00	5jhf-D	2.9	7.9	61	82	7	MOLECULE:	KLTH0D11660
36:00:00	5do7-D	2.9	4.5	74	571	9	MOLECULE:	ATP-BINDING
37:00:00	7bmh-A	2.9	8.6	104	247	11	MOLECULE:	OPSIN
38:00:00	6whg-A	2.9	19.5	121	523	7	MOLECULE:	CALCIUM CHA
39:00:00	7dgj-A	2.9	8.3	55	153	4	MOLECULE:	MYOGLOBIN
40:00:00	7dgm-B	2.9	8.1	57	153	4	MOLECULE:	MYOGLOBIN
41:00:00	3vm9-A	2.9	11.5	57	153	5	MOLECULE:	MYOGLOBIN
42:00:00	4hrr-H	2.9	4.1	75	150	8	MOLECULE:	GLOBIN-2 A
43:00:00	3dhr-F	2.9	4.9	79	145	11	MOLECULE:	HEMOGLOBIN
44:00:00	7dgo-A	2.9	8.3	54	153	2	MOLECULE:	MYOGLOBIN
45:00:00	4mqj-H	2.9	4.8	83	146	10	MOLECULE:	HEMOGLOBIN
46:00:00	7v5q-A	2.9	10.1	71	153	6	MOLECULE:	MYOGLOBIN
47:00:00	7dgm-A	2.9	8.1	55	153	4	MOLECULE:	MYOGLOBIN
48:00:00	3fs4-D	2.9	4.6	78	146	15	MOLECULE:	HEMOGLOBIN
49:00:00	7dgk-A	2.9	10.5	65	153	3	MOLECULE:	MYOGLOBIN
50:00:00	7dgj-B	2.9	9.9	72	153	6	MOLECULE:	MYOGLOBIN
51:00:00	3wyo-C	2.9	11.4	56	153	2	MOLECULE:	MYOGLOBIN
52:00:00	6ltm-A	2.9	8	56	153	4	MOLECULE:	MYOGLOBIN
53:00:00	7dgl-B	2.9	10.1	72	153	7	MOLECULE:	MYOGLOBIN
54:00:00	7v5r-A	2.9	9.6	63	153	5	MOLECULE:	MYOGLOBIN
55:00:00	2hbs-H	2.9	4.6	82	146	10	MOLECULE:	HEMOGLOBIN
56:00:00	6ltl-A	2.9	11.6	57	153	4	MOLECULE:	MYOGLOBIN
57:00:00	1y4p-B	2.9	4.4	79	146	10	MOLECULE:	HEMOGLOBIN
58:00:00	8dov-H	2.9	3.6	81	145	7	MOLECULE:	HEMOGLOBIN
59:00:00	7b1s-B	2.8	10.1	105	466	10	MOLECULE:	ETHYL-COENZ
60:00:00	6zu0-C	2.8	17.2	87	426	8	MOLECULE:	CITRATE SYN
61:00:00	6cgh-A	2.8	4.2	47	89	6	MOLECULE:	DNAJ HOMOLO
62:00:00	2y7c-A	2.8	3.8	63	464	10	MOLECULE:	TYPE-1 REST
63:00:00	7zgi-B	2.8	6.1	58	329	7	MOLECULE:	PEPTIDYLPRO
64:00:00	8snl-B	2.8	16.3	75	491	8	MOLECULE:	DISINTEGRIN
65:00:00	4f4h-A	2.8	16.7	102	540	9	MOLECULE:	GLUTAMINE D
66:00:00	6j36-A	2.8	4.7	55	459	4	MOLECULE:	ENOLASE
67:00:00	4jvy-A	2.8	10.5	58	191	14	MOLECULE:	FEMALE GERM
68:00:00	6gyt-B	2.8	6.2	52	168	8	MOLECULE:	HISTONE ACE
69:00:00	4cej-A	2.8	22.4	129	1177	9	MOLECULE:	ATP-DEPENDE
70:00:00	6ffv-A	2.8	13	59	194	3	MOLECULE:	BTUM
71:00:00	6gkw-A	2.8	3.7	73	351	7	MOLECULE:	PUTATIVE PH
72:00:00	2yev-B	2.8	17.3	92	319	4	MOLECULE:	CYTOCHROME
73:00:00	4nqj-A	2.8	7.5	64	177	8	MOLECULE:	E3 UBIQUITI
74:00:00	5vp3-A	2.8	11	72	195	6	MOLECULE:	MNEMIOPSIN

75:00:00	6rfs-6	2.8	9.8	78	138	8	MOLECULE:	SUBUNIT NUA
76:00:00	5jhf-F	2.8	24.9	95	405	12	MOLECULE:	KLTH0D11660
77:00:00	7yoj-A	2.8	28.3	103	867	5	MOLECULE:	CASPI
78:00:00	7asv-A	2.8	10	71	155	7	MOLECULE:	DNA-DIRECTE
79:00:00	8vjn-A	2.8	4.7	43	52	9	MOLECULE:	ENCAPSULIN
80:00:00	3stq-A	2.8	6.4	51	86	6	MOLECULE:	PUTATIVE UN
81:00:00	6ql4-A	2.8	10.6	114	660	6	MOLECULE:	PUTATIVE MI
82:00:00	7sbe-A	2.8	6.3	96	228	8	MOLECULE:	TELOMERASE
83:00:00	4he8-D	2.8	7.2	60	160	7	MOLECULE:	NADH-QUINON
84:00:00	2wtg-A	2.8	4.3	76	158	5	MOLECULE:	GLOBIN-LIKE
85:00:00	6p18-Q	2.8	7.2	73	156	8	MOLECULE:	DNA (67-MER
86:00:00	3wfw-A	2.8	10.7	57	138	7	MOLECULE:	HEMOGLOBIN-
87:00:00	8izu-A	2.8	4.5	79	137	5	MOLECULE:	VIRION MORP
88:00:00	6swg-B	2.8	4.7	54	74	2	MOLECULE:	PERIPHILIN-
89:00:00	7ao3-A	2.8	9.5	57	303	4	MOLECULE:	CYCLOOCTAT-
90:00:00	6jo0-A	2.8	5.3	69	224	3	MOLECULE:	VIRRDTS
91:00:00	2wj9-B	2.8	6.1	79	145	5	MOLECULE:	INTERGENIC-
92:00:00	1w0b-A	2.8	6.2	72	102	10	MOLECULE:	ALPHA-HEMOG
93:00:00	7s5l-A	2.8	4.5	87	378	9	MOLECULE:	CEMBRENE A
94:00:00	6jal-A	2.8	11.6	58	416	10	MOLECULE:	ABC TRANSPO
95:00:00	6lum-G	2.8	5.6	61	123	2	MOLECULE:	SUCCINATE D
96:00:00	3n1e-B	2.8	7.8	83	141	2	MOLECULE:	VACUOLAR PR
97:00:00	7emf-P	2.8	16	75	766	8	MOLECULE:	MEDIATOR OF
98:00:00	6rwy-g	2.8	12.1	73	86	4	MOLECULE:	INNER ROD P
99:00:00	1it2-A	2.8	3.9	76	146	12	MOLECULE:	HEMOGLOBIN
0:00	6idp-A	2.8	13	103	438	7	MOLECULE:	MATE FAMILY
1:00	1akh-B	2.8	3.6	66	78	14	MOLECULE:	DNA (5'-
2:00	6sum-A	2.8	7.5	95	335	4	MOLECULE:	AMICOUMACIN
3:00	3cit-A	2.8	9	64	155	6	MOLECULE:	SENSOR HIST
4:00	2r5z-A	2.8	5.9	67	75	1	MOLECULE:	HOMEOTIC PR
5:00	6sz9-D	2.8	19.3	69	282	4	MOLECULE:	ICMO (DOTL)
6:00	6wql-A	2.8	5.4	44	49	11	MOLECULE:	SEED PEPTID
7:00	8ppr-P	2.8	14.7	56	176	4	MOLECULE:	KINETOCHORE
8:00	3fx7-B	2.8	13.1	73	87	3	MOLECULE:	PUTATIVE UN
9:00	4j41-B	2.8	9.7	65	86	3	MOLECULE:	SECRETED PR
10:00	7eu3-G	2.8	8.5	80	176	9	MOLECULE:	NAD(P)H-QUI
11:00	6ybw-y	2.8	18.4	83	162	11	MOLECULE:	40S RIBOSOM
12:00	2ilk-A	2.8	17.1	85	155	9	MOLECULE:	INTERLEUKIN
13:00	4u7b-A	2.8	19.4	75	342	3	MOLECULE:	DNA (25-MER
14:00	8if8-B	2.8	14.2	97	620	5	MOLECULE:	GALACTAN 5-
15:00	7os0-A	2.8	13.2	95	1130	5	MOLECULE:	CAS13A
16:00	6awl-A	2.8	18	84	217	8	MOLECULE:	UBIQUINONE
17:00	2yvx-A	2.8	7.1	99	442	8	MOLECULE:	MG2+ TRANSP
18:00	4gfq-A	2.8	4.6	76	186	9	MOLECULE:	RIBOSOME-RE

19:00	5abb-A	2.8	9.2	90	443	8	MOLECULE:	PROTEIN TRA
20:00	7eqf-A	2.8	5.8	77	184	12	MOLECULE:	TETR/ACRR F
21:00	6oqr-W	2.8	8.7	89	173	7	MOLECULE:	ATP SYNTHAS
22:00	6k7l-A	2.8	21.9	96	992	5	MOLECULE:	PHOSPHOLIPI
23:00	5o5j-O	2.8	5.9	63	88	14	MOLECULE:	16S RRNA
24:00:00	7ar9-K	2.8	9.2	82	104	7	MOLECULE:	ND3
25:00:00	6u8y-d	2.8	6.3	67	89	1	MOLECULE:	MONOVALENT
26:00:00	3utk-A	2.8	3.4	60	95	7	MOLECULE:	LIPOPROTEIN
27:00:00	1uzc-A	2.8	2.8	55	69	7	MOLECULE:	HYPOTHETICA
28:00:00	7nhr-A	2.8	20.8	85	663	4	MOLECULE:	PUTATIVE TR
29:00:00	2p6j-A	2.8	2.3	44	52	0	MOLECULE:	DESIGNED EN
30:00:00	8j56-C	2.8	3.9	65	160	6	MOLECULE:	FLAGELLAR T
31:00:00	8eau-e	2.8	5.3	61	72	7	MOLECULE:	V-TYPE PROT
32:00:00	7lsv-B	2.8	6.1	85	158	4	MOLECULE:	CALMODULIN-
33:00:00	5dn6-H	2.8	4.4	69	110	3	MOLECULE:	CHAIN A
34:00:00	6z5s-W	2.8	4	73	94	3	MOLECULE:	LIGHT HARVE
35:00:00	8ovt-A	2.8	7.1	71	288	4	MOLECULE:	YEGT GLYCOS
36:00:00	5ch6-A	2.8	8.3	81	286	6	MOLECULE:	FRIGIDA
37:00:00	6wxv-A	2.8	17.3	121	1091	10	MOLECULE:	DUAL OXIDAS
38:00:00	3wvo-A	2.8	4.6	76	543	12	MOLECULE:	CRISPR-ASSO
39:00:00	6wg3-C	2.8	23.6	81	248	10	MOLECULE:	STRUCTURAL
40:00:00	4p9f-A	2.8	7.8	102	212	8	MOLECULE:	HTH-TYPE TR
41:00:00	2k85-A	2.8	3.2	58	65	9	MOLECULE:	GLUCOCORTIC
42:00:00	3pux-G	2.8	5.2	88	293	5	MOLECULE:	MALTOSE-BIN
43:00:00	8eew-A	2.8	4.7	112	766	3	MOLECULE:	DYNAMIN-LIK
44:00:00	6ekr-A	2.8	6.6	89	303	10	MOLECULE:	TYPE II SIT
45:00:00	8q7e-J	2.8	21.4	126	895	8	MOLECULE:	CULLIN-9
46:00:00	7z1n-O	2.8	22.7	96	570	5	MOLECULE:	DNA-DIRECTE
47:00:00	7yh5-B	2.8	8.8	85	177	18	MOLECULE:	NUCLEOSIDE
48:00:00	5ero-A	2.8	14.1	96	301	4	MOLECULE:	FUSICOCCADI
49:00:00	8hsb-A	2.8	19.6	102	374	5	MOLECULE:	CDNG
50:00:00	1hbs-D	2.8	3.4	77	146	8	MOLECULE:	HEMOGLOBIN
51:00:00	7v5p-B	2.8	8.4	55	153	4	MOLECULE:	MYOGLOBIN
52:00:00	7dgk-B	2.8	10.2	66	153	8	MOLECULE:	MYOGLOBIN
53:00:00	5x2s-F	2.8	4.1	80	145	13	MOLECULE:	HEMOGLOBIN
54:00:00	6q6h-N	2.8	30.9	120	653	4	MOLECULE:	ANAPHASE-PR
55:00:00	1qpw-B	2.8	4.6	79	146	10	MOLECULE:	PORCINE HEM
56:00:00	7v5q-B	2.8	10.1	65	153	5	MOLECULE:	MYOGLOBIN
57:00:00	7dgo-B	2.8	10.1	66	153	3	MOLECULE:	MYOGLOBIN
58:00:00	3vrf-B	2.8	4.8	79	146	8	MOLECULE:	HEMOGLOBIN
59:00:00	3wyo-B	2.8	7.2	55	153	7	MOLECULE:	MYOGLOBIN
60:00:00	3wcp-D	2.8	4.5	79	145	10	MOLECULE:	HEMOGLOBIN
61:00:00	7v5p-A	2.8	11.3	56	153	5	MOLECULE:	MYOGLOBIN
62:00:00	1moh-A	2.8	4.4	74	142	5	MOLECULE:	MONOMERIC H

63:00:00	3w4u-B	2.8	4.4	81	145	11	MOLECULE:	HEMOGLOBIN
64:00:00	5gko-A	2.7	4.2	79	650	3	MOLECULE:	MACROLIDE E
65:00:00	6n7p-X	2.7	12.2	83	826	2	MOLECULE:	U1 SMALL NU
66:00:00	6u4b-A	2.7	11.5	122	570	4	MOLECULE:	WBBM PROTEI
67:00:00	6id0-L	2.7	14	81	475	4	MOLECULE:	PRE-MRNA-PR
68:00:00	3ucq-A	2.7	19.9	82	651	4	MOLECULE:	AMYLOSUCRAS
69:00:00	2rdc-B	2.7	11.3	90	135	9	MOLECULE:	UNCHARACTER
70:00:00	6m36-I	2.7	4.2	45	104	4	MOLECULE:	SERINE-PROT
71:00:00	6pij-A	2.7	4.3	68	351	6	MOLECULE:	CAS7 TYPE I
72:00:00	3d1n-I	2.7	13.7	67	149	7	MOLECULE:	5'-
73:00:00	6xiv-A	2.7	4.6	84	257	4	MOLECULE:	REGULATORY
74:00:00	7f52-A	2.7	29.7	124	584	5	MOLECULE:	NON-STRUCTU
75:00:00	3etz-B	2.7	14.9	76	115	3	MOLECULE:	ADHESIN A
76:00:00	3oov-A	2.7	2.9	39	164	10	MOLECULE:	METHYL-ACCE
77:00:00	5gm2-B	2.7	11	75	283	13	MOLECULE:	O-METHYLRAN
78:00:00	8bf9-G	2.7	7.6	60	158	3	MOLECULE:	RNA (1766)
79:00:00	4j7o-A	2.7	13.4	91	329	3	MOLECULE:	PUTATIVE SU
80:00:00	5w7d-A	2.7	16.1	84	528	4	MOLECULE:	ACYLOXYACYL
81:00:00	6kp3-B	2.7	9.5	42	112	2	MOLECULE:	PROGRAMMED
82:00:00	8bd7-A	2.7	22.5	105	539	10	MOLECULE:	IFT88
83:00:00	7z36-B	2.7	9.5	62	446	5	MOLECULE:	ENDOLYSIN,T
84:00:00	6vmb-g	2.7	4.5	42	323	10	MOLECULE:	ATP SYNTHAS
85:00:00	2ebi-A	2.7	3.1	61	86	8	MOLECULE:	DNA BINDING
86:00:00	2pmq-B	2.7	3.6	46	374	0	MOLECULE:	MANDELATE R
87:00:00	2g8l-B	2.7	2.7	49	286	4	MOLECULE:	287AA LONG
88:00:00	5yfp-B	2.7	17.9	77	927	5	MOLECULE:	EXOCYST COM
89:00:00	8k2g-A	2.7	20.7	75	505	8	MOLECULE:	OLIGOSACCHA
90:00:00	7e8k-B	2.7	9.4	67	205	7	MOLECULE:	RNA-FREE RI
91:00:00	8sun-B	2.7	16.3	116	743	4	MOLECULE:	ANOCTAMIN-6
92:00:00	7bhq-B	2.7	15	87	204	8	MOLECULE:	BASAL-BODY
93:00:00	2jvg-A	2.7	4	64	72	11	MOLECULE:	IGG-BINDING
94:00:00	8ek4-B	2.7	9.4	70	135	14	MOLECULE:	ICE-BINDING
95:00:00	7sbb-I	2.7	7	111	680	6	MOLECULE:	CAS7D
96:00:00	8gwe-A	2.7	24.7	121	931	5	MOLECULE:	RNA-DIRECTE
97:00:00	5k8o-B	2.7	10.4	73	425	8	MOLECULE:	5-NITROANTH
98:00:00	8cjf-B	2.7	8.2	90	639	3	MOLECULE:	AETF
99:00:00	7ml7-A	2.7	17.7	121	1216	5	MOLECULE:	TOXIN B
0:00	7tb3-A	2.7	15.8	78	243	5	MOLECULE:	ISOFORM 2 O
1:00	5w10-A	2.7	3.4	39	173	8	MOLECULE:	CGMP-SPECIF
2:00	3ps5-A	2.7	5.2	90	529	8	MOLECULE:	TYROSINE-PR
3:00	1n93-X	2.7	19	84	335	2	MOLECULE:	P40 NUCLEOP
4:00	7nza-A	2.7	9.1	65	141	9	MOLECULE:	ODORANT BIN
5:00	5csk-A	2.7	34.2	124	1996	7	MOLECULE:	ACETYL-COA
6:00	5d50-G	2.7	9.7	55	77	9	MOLECULE:	REPRESSOR

7:00	5im3-A	2.7	15.8	77	874	5	MOLECULE:	RIBONUCLEOS
8:00	6wuc-H	2.7	7.8	69	179	9	MOLECULE:	INNER KINET
9:00	8j5q-B	2.7	2.8	42	325	10	MOLECULE:	UNCHARACTER
10:00	6idf-B	2.7	8.2	98	312	9	MOLECULE:	NICASTRIN
11:00	2kjf-A	2.7	2.6	50	60	14	MOLECULE:	CARNOCYCLIN
12:00	5t58-A	2.7	13.2	71	233	4	MOLECULE:	KLLA0F02343
13:00	4y2f-A	2.7	9.1	61	143	10	MOLECULE:	SENSOR PROT
14:00	8wzb-A	2.7	3	42	46	10	MOLECULE:	DPY30 DOMAI
15:00	8ear-A	2.7	20.5	116	2389	6	MOLECULE:	INOSITOL 1,
16:00	7lvf-A	2.7	6.4	48	57	4	MOLECULE:	VICILIN JUG
17:00	7zm7-2	2.7	15.7	100	558	4	MOLECULE:	NADH-UBIQUI
18:00	8h4p-A	2.7	5.6	81	326	7	MOLECULE:	LONGIBORNEO
19:00	8fo9-F	2.7	31.2	108	2289	6	MOLECULE:	LEUCINE-RIC
20:00	6r6b-F	2.7	8.4	66	245	6	MOLECULE:	SURFACE PRE
21:00	3kdw-A	2.7	5.3	76	206	8	MOLECULE:	PUTATIVE SU
22:00	6z70-A	2.7	9.1	88	444	9	MOLECULE:	AQ128
23:00	4n5a-A	2.7	25.5	99	535	6	MOLECULE:	PROTEIN EFR
24:00:00	8tdl-A	2.7	24.8	83	364	5	MOLECULE:	MECHANOSENS
25:00:00	4yo2-A	2.7	4.7	67	186	9	MOLECULE:	TRANSCRIPTI
26:00:00	7kzn-B	2.7	18.5	80	683	10	MOLECULE:	HEAVY CHAIN
27:00:00	6o3q-A	2.7	3.7	46	49	7	MOLECULE:	VICILIN
28:00:00	2fbq-A	2.7	11.7	70	213	7	MOLECULE:	PROBABLE TR
29:00:00	5n8k-C	2.7	5.5	51	80	10	MOLECULE:	GALACTOCERE
30:00:00	3f0c-A	2.7	6.7	74	193	4	MOLECULE:	TRANSCRIPTI
31:00:00	6bhc-A	2.7	4	80	371	4	MOLECULE:	PSEUDOPODIU
32:00:00	2xco-A	2.7	14.6	80	636	8	MOLECULE:	DNA GYRASE
33:00:00	2hcf-A	2.7	6.3	63	225	8	MOLECULE:	HYDROLASE,
34:00:00	5mmi-Z	2.7	10.8	77	101	3	MOLECULE:	50S RIBOSOM
35:00:00	7zke-E	2.7	22.4	132	1623	7	MOLECULE:	DNA (36-MER
36:00:00	2m2e-A	2.7	3.7	56	70	11	MOLECULE:	DNAJ HOMOLO
37:00:00	4ejo-A	2.7	4.3	53	112	13	MOLECULE:	TRANSCRIPTI
38:00:00	5xw4-B	2.7	9.9	87	369	9	MOLECULE:	TYROSINE-PR
39:00:00	6ny2-Y	2.7	29.7	137	915	7	MOLECULE:	DNA TARGET
40:00:00	7e40-A	2.7	6.7	74	106	5	MOLECULE:	PROTEIN PHO
41:00:00	3lml-A	2.7	3.9	77	440	9	MOLECULE:	LIN1278 PRO
42:00:00	6vrb-C	2.7	7.3	64	229	5	MOLECULE:	RNA (52-MER
43:00:00	2ly9-A	2.7	5.6	64	74	8	MOLECULE:	ZINC FINGER
44:00:00	8agg-A	2.7	7.8	64	489	3	MOLECULE:	PHOSPHOCHOL
45:00:00	1a7d-A	2.7	6.4	70	118	4	MOLECULE:	MYOHEMERYTH
46:00:00	6yxp-B	2.7	11	76	272	4	MOLECULE:	SWI/SNF COM
47:00:00	2ijd-1	2.7	16.2	111	644	7	MOLECULE:	PICORNAIN 3
48:00:00	5iz5-A	2.7	9.2	113	757	4	MOLECULE:	CYTOSOLIC P
49:00:00	7way-A	2.7	9	101	960	5	MOLECULE:	DNA (33-MER
50:00:00	4qvg-C	2.7	7.6	104	349	8	MOLECULE:	SIBL

51:00:00	5udb-4	2.7	8.6	111	751	9	MOLECULE:	DNA REPLICA
52:00:00	3nnq-A	2.7	4.4	65	95	3	MOLECULE:	N-TERMINAL
53:00:00	2js9-A	2.7	3.1	54	81	6	MOLECULE:	SAPOSIN-LIK
54:00:00	7bw0-R	2.7	17.8	110	280	10	MOLECULE:	SOLUBLE CYT
55:00:00	8p62-D	2.7	19	80	245	6	MOLECULE:	DNA REPLICA
56:00:00	5h72-A	2.7	2.7	58	67	7	MOLECULE:	FLAGELLAR B
57:00:00	8r6y-A	2.7	18.9	135	1991	7	MOLECULE:	RNA-DIRECTE
58:00:00	7c7e-A	2.7	8.5	88	142	6	MOLECULE:	PUTATIVE DN
59:00:00	6l30-A	2.7	13.2	81	518	9	MOLECULE:	PROTEIN ECT
60:00:00	8rf0-A	2.7	16.4	111	2780	5	MOLECULE:	CYCLIC BETA
61:00:00	3trc-A	2.7	4.7	74	168	9	MOLECULE:	PHOSPHOENOL
62:00:00	7yyg-A	2.7	16.3	91	275	7	MOLECULE:	PUTATIVE OU
63:00:00	6gmh-M	2.7	22.5	111	991	5	MOLECULE:	RPB1
64:00:00	2be4-A	2.7	7.1	93	270	5	MOLECULE:	HYPOTHETICA
65:00:00	3egw-C	2.7	10.3	107	225	11	MOLECULE:	RESPIRATORY
66:00:00	5nzz-A	2.7	15.2	101	378	11	MOLECULE:	TGF-BETA-AC
67:00:00	8iq4-R	2.7	13.3	99	274	2	MOLECULE:	PROSTAGLAND
68:00:00	7jgr-D	2.7	21.4	89	441	8	MOLECULE:	ORIGIN RECO
69:00:00	5zib-A	2.7	26.6	107	579	5	MOLECULE:	ALPHA-1,6-M
70:00:00	3pde-B	2.7	7.8	84	287	2	MOLECULE:	FARNESYL-DI
71:00:00	1hlv-A	2.7	6.8	78	131	10	MOLECULE:	CENP-B BOX
72:00:00	7whm-A	2.7	33.4	100	1214	6	MOLECULE:	PPPDE DOMAI
73:00:00	7aoa-A	2.7	31.7	94	343	12	MOLECULE:	METHYL-CPG-
74:00:00	5zb3-A	2.7	13.6	105	280	9	MOLECULE:	ORF57
75:00:00	6thh-C	2.7	13.5	79	816	6	MOLECULE:	SIRV2 ACRID
76:00:00	7p4a-A	2.7	26.7	94	246	6	MOLECULE:	STL
77:00:00	5e6e-B	2.7	4.5	75	146	11	MOLECULE:	HEMOGLOBIN
78:00:00	5x2u-D	2.7	4.6	78	145	12	MOLECULE:	HEMOGLOBIN
79:00:00	1si4-B	2.7	4.6	78	146	12	MOLECULE:	HEMOGLOBIN
80:00:00	3w4u-D	2.7	4.5	79	145	10	MOLECULE:	HEMOGLOBIN
81:00:00	7dgn-A	2.7	8.3	55	153	4	MOLECULE:	MYOGLOBIN
82:00:00	3vm9-B	2.7	8.3	56	153	4	MOLECULE:	MYOGLOBIN
83:00:00	6ltl-B	2.7	9.7	73	153	5	MOLECULE:	MYOGLOBIN
84:00:00	5ker-B	2.7	4.5	79	146	10	MOLECULE:	ALPHA-GLOBI
85:00:00	6ltm-B	2.7	8	57	153	4	MOLECULE:	MYOGLOBIN
86:00:00	7v5r-B	2.7	8.1	57	153	4	MOLECULE:	MYOGLOBIN
87:00:00	2qls-D	2.7	4.9	79	146	9	MOLECULE:	HEMOGLOBIN
88:00:00	7uf7-D	2.7	4.6	76	146	11	MOLECULE:	HEMOGLOBIN
89:00:00	6tm5-N	2.7	22.3	109	676	6	MOLECULE:	ANAPHASE-PR
90:00:00	8pkp-N	2.7	8.5	121	652	5	MOLECULE:	ANAPHASE-PR
91:00:00	5hdi-A	2.6	4.7	98	397	6	MOLECULE:	CYTOCHROME
92:00:00	7ubn-Q	2.6	5.2	74	207	8	MOLECULE:	DNA (53-MER
93:00:00	2pyw-A	2.6	7.9	77	417	1	MOLECULE:	UNCHARACTER
94:00:00	6em5-b	2.6	16	88	421	6	MOLECULE:	5.8S RIBOSO

95:00:00	7cbb-A	2.6	5.6	100	576	6	MOLECULE:	IUCA/IUCC F
96:00:00	7tjl-A	2.6	5.4	58	85	5	MOLECULE:	DE NOVO DES
97:00:00	3zjc-E	2.6	13.8	88	286	6	MOLECULE:	GTPASE IMAP
98:00:00	8c5s-A	2.6	4.4	78	924	4	MOLECULE:	MITOCHONDRI
99:00:00	8t53-A	2.6	15.2	106	398	6	MOLECULE:	UNDECAPRENY
0:00	5x3x-M	2.6	6.9	66	207	6	MOLECULE:	COBALT ABC
1:00	3ut2-A	2.6	18.9	108	735	6	MOLECULE:	CATALASE-PE
2:00	8sgi-A	2.6	4	62	751	5	MOLECULE:	SODIUM/CALC
3:00	8es4-C	2.6	4.9	74	181	5	MOLECULE:	GP35
4:00	8hwa-A	2.6	27.9	105	700	7	MOLECULE:	PRIMASE D5
5:00	8wue-A	2.6	12.7	70	226	7	MOLECULE:	SIGMA NON-O
6:00	7eld-A	2.6	25.5	99	1137	8	MOLECULE:	ENDORIBONUC
7:00	8ahx-E	2.6	9.2	76	213	9	MOLECULE:	ION-TRANSLO
8:00	8cws-A	2.6	16.8	130	451	12	MOLECULE:	F4132-2 CHA
9:00	7e2d-H	2.6	6.7	62	147	8	MOLECULE:	TRAPP-ASSOC
10:00	7pv1-D	2.6	18.4	61	141	8	MOLECULE:	MICOS COMPL
11:00	6lk8-J	2.6	19.3	83	1026	10	MOLECULE:	MGC83295 PR
12:00	5ve9-B	2.6	3.7	67	91	7	MOLECULE:	MICROTUBULE
13:00	7ccl-C	2.6	9.5	74	147	8	MOLECULE:	BCL-2-LIKE
14:00	8v46-A	2.6	15.2	84	412	8	MOLECULE:	ARIA ANTITO
15:00	6dlm-B	2.6	6	50	72	6	MOLECULE:	DHD127_A
16:00	7uy7-B	2.6	16.3	82	291	9	MOLECULE:	TELOMERASE-
17:00	7xad-C	2.6	6.9	55	96	5	MOLECULE:	PROGRAMMED
18:00	8tl7-A	2.6	7.1	104	694	7	MOLECULE:	COMPUTATION
19:00	6ofb-A	2.6	12.7	87	696	3	MOLECULE:	GLUTAMINE-D
20:00	6s3l-K	2.6	7.2	80	193	5	MOLECULE:	FLAGELLAR B
21:00	8dmg-B	2.6	10.5	144	1033	4	MOLECULE:	BIFUNCTIONA
22:00	7jts-s	2.6	24.6	85	290	7	MOLECULE:	RADIAL SPOK
23:00	1dce-A	2.6	11.1	110	567	6	MOLECULE:	PROTEIN (RA
24:00:00	2l7x-A	2.6	2.8	47	77	2	MOLECULE:	ENVELOPE GL
25:00:00	5j8v-A	2.6	19.4	76	3398	9	MOLECULE:	RYANODINE R
26:00:00	4qn1-A	2.6	10.2	100	372	10	MOLECULE:	E3 UBIQUITI
27:00:00	7qoo-Q	2.6	13.7	71	209	4	MOLECULE:	CENTROMERE
28:00:00	7wmp-A	2.6	7.4	53	263	6	MOLECULE:	PORTAL PROT
29:00:00	8fm9-M	2.6	4.8	94	333	7	MOLECULE:	RNA-DIRECTE
30:00:00	4hga-A	2.6	20.5	104	207	5	MOLECULE:	DEATH DOMAI
31:00:00	3fey-A	2.6	5.7	79	761	5	MOLECULE:	NUCLEAR CAP
32:00:00	7obq-y	2.6	8.8	80	454	8	MOLECULE:	SRP RNA
33:00:00	8ovw-Q	2.6	24.2	74	258	4	MOLECULE:	CENTROMERE-
34:00:00	8fed-K	2.6	4.4	57	141	5	MOLECULE:	VIRULENCE F
35:00:00	2qf9-A	2.6	6.7	75	162	8	MOLECULE:	PUTATIVE SE
36:00:00	3w5m-A	2.6	17.6	103	1030	6	MOLECULE:	PUTATIVE RH
37:00:00	7ykr-A	2.6	28.5	97	1035	12	MOLECULE:	TRANSIENT R
38:00:00	8d08-D	2.6	6.6	58	67	7	MOLECULE:	HALC4_135

39:00:00	8h38-C	2.6	22.7	117	407	5	MOLECULE:	COP9 SIGNAL
40:00:00	7t5p-B	2.6	17.2	85	419	9	MOLECULE:	SUMO-INTERA
41:00:00	6yiz-A	2.6	2.7	43	211	12	MOLECULE:	TRANSCRIPTI
42:00:00	6gyf-B	2.6	11.8	89	366	7	MOLECULE:	NICOTINAMID
43:00:00	5izs-D	2.6	10.2	56	82	4	MOLECULE:	DESIGNED PR
44:00:00	5m4s-A	2.6	3.5	56	209	7	MOLECULE:	TRANSCRIPTI
45:00:00	6vjy-B	2.6	10.6	63	280	5	MOLECULE:	ERAD-ASSOCI
46:00:00	8xj6-D	2.6	19.1	95	589	9	MOLECULE:	MONKEYPOX V
47:00:00	8erc-A	2.6	16.4	97	441	5	MOLECULE:	LYSOPHOSPHO
48:00:00	7zr6-C	2.6	4.7	83	277	7	MOLECULE:	HEAT SHOCK
49:00:00	5t58-B	2.6	11.1	55	190	5	MOLECULE:	KLLA0F02343
50:00:00	2fiy-A	2.6	8.2	106	285	16	MOLECULE:	PROTEIN FDH
51:00:00	6p8r-B	2.6	6.5	64	168	5	MOLECULE:	HORMA DOMAI
52:00:00	6fkg-C	2.6	4.8	50	111	10	MOLECULE:	RV1989C (MB
53:00:00	2maj-A	2.6	9.7	61	82	11	MOLECULE:	STROMAL INT
54:00:00	8d8j-O	2.6	12.3	60	210	5	MOLECULE:	PROBABLE S-
55:00:00	4mlb-B	2.6	9.5	101	454	5	MOLECULE:	PF0708
56:00:00	5vvh-D	2.6	2.9	40	210	10	MOLECULE:	OCTOPINE CA
57:00:00	7f5v-A	2.6	21.8	113	689	8	MOLECULE:	DELTA-1-PYR
58:00:00	5anz-A	2.6	10.9	99	375	3	MOLECULE:	SOLUBLE LYT
59:00:00	7vcf-C	2.6	12.2	74	217	8	MOLECULE:	TIC214
60:00:00	7nur-A	2.6	13.4	104	1044	12	MOLECULE:	PROTEIN KIN
61:00:00	7qd4-A	2.6	14.4	108	908	6	MOLECULE:	TRANSPOSASE
62:00:00	7sp5-A	2.6	9.9	122	433	11	MOLECULE:	PHOSPHATE T
63:00:00	4hzu-S	2.6	5.5	91	164	5	MOLECULE:	ENERGY-COUP
64:00:00	2odm-B	2.6	6.5	72	83	6	MOLECULE:	UPF0358 PRO
65:00:00	4mk6-A	2.6	7.9	70	188	3	MOLECULE:	PROBABLE DI
66:00:00	7d3u-C	2.6	7.6	88	129	9	MOLECULE:	MONOVALENT
67:00:00	6q6b-A	2.6	6.7	76	132	8	MOLECULE:	CYTOSOLIC C
68:00:00	8d2z-A	2.6	13.1	77	306	6	MOLECULE:	METALLO-BET
69:00:00	2khm-A	2.6	11.8	89	140	11	MOLECULE:	FIBROIN-3
70:00:00	4wbd-A	2.6	21.2	63	540	8	MOLECULE:	BSHC
71:00:00	4avm-A	2.6	14	112	230	7	MOLECULE:	BRIDGING IN
72:00:00	7yh3-D	2.6	4.7	72	157	7	MOLECULE:	TRAPPC3 FRO
73:00:00	3dby-C	2.6	12.6	91	267	7	MOLECULE:	UNCHARACTER
74:00:00	4x2c-A	2.6	8.1	65	207	6	MOLECULE:	FIC FAMILY
75:00:00	3nrk-A	2.6	9.1	89	312	10	MOLECULE:	LIC12922
76:00:00	1e9s-A	2.6	15.3	104	427	9	MOLECULE:	CONJUGAL TR
77:00:00	4uig-A	2.6	7	67	91	7	MOLECULE:	COPPER SENS
78:00:00	3nrg-A	2.6	8.4	76	217	5	MOLECULE:	TETR FAMILY
79:00:00	7emf-H	2.6	7.9	75	181	9	MOLECULE:	MEDIATOR OF
80:00:00	2xl4-A	2.6	9.4	81	147	6	MOLECULE:	LMO0438 PRO
81:00:00	8j0n-D	2.6	35.8	95	156	4	MOLECULE:	ER MEMBRANE
82:00:00	3u8p-A	2.6	16.2	97	336	10	MOLECULE:	CYTOCHROME

83:00:00	3bey-D	2.6	3	55	94	11	MOLECULE:	CONSERVED P
84:00:00	8twj-A	2.6	5	90	303	7	MOLECULE:	AVIRULENCE
85:00:00	6nd4-M	2.6	11.8	107	431	9	MOLECULE:	ETS RRNA
86:00:00	2w4m-A	2.6	7.1	84	250	11	MOLECULE:	N-ACYLNEURA
87:00:00	5a5t-L	2.6	23.9	87	372	6	MOLECULE:	EUKARYOTIC
88:00:00	7zol-B	2.6	20.8	119	1702	7	MOLECULE:	CAS7-11
89:00:00	8urw-Z	2.6	8.2	122	1235	10	MOLECULE:	DNA-DIRECTE
90:00:00	5yfp-C	2.6	15.7	101	790	7	MOLECULE:	EXOCYST COM
91:00:00	8vej-B	2.6	3.1	53	242	4	MOLECULE:	CHD_BUTTRES
92:00:00	6hux-A	2.6	14.2	85	369	7	MOLECULE:	H(2)-FORMIN
93:00:00	8ca1-A	2.6	20.9	127	589	6	MOLECULE:	VERY LONG-C
94:00:00	3hug-A	2.6	11.4	58	80	10	MOLECULE:	RNA POLYMER
95:00:00	1tjf-B	2.6	8.9	86	186	3	MOLECULE:	ADENYLYL CY
96:00:00	6w2j-B	2.6	21.3	96	1364	5	MOLECULE:	CARBAMOYL-P
97:00:00	3esw-A	2.6	6.6	98	333	6	MOLECULE:	PEPTIDE-N(4
98:00:00	5v5t-A	2.6	7.5	107	306	7	MOLECULE:	CONSERVED D
99:00:00	7yca-O	2.6	5	65	96	3	MOLECULE:	LHCA1
0:00	6c66-I	2.6	8.9	67	177	10	MOLECULE:	CRISPR-ASSO
1:00	6nsj-A	2.6	9.4	86	182	6	MOLECULE:	ACID-ACTIVA
2:00	7v99-A	2.6	23.8	152	991	9	MOLECULE:	TELOMERASE
3:00	6t58-A	2.6	6.6	84	524	5	MOLECULE:	CELLULAR TU
4:00	8hmd-C	2.6	10.2	81	1334	11	MOLECULE:	INTRAFLAGEL
5:00	6es9-A	2.6	8.9	92	545	4	MOLECULE:	ACYL-COA DE
6:00	7jtk-A	2.6	15.2	75	749	9	MOLECULE:	FLAGELLAR R
7:00	8vc8-A	2.6	4	68	197	7	MOLECULE:	HEM_3.C9
8:00	6qm5-A	2.6	9.3	79	673	4	MOLECULE:	PREDICTED P
9:00	5eqw-B	2.6	12.1	73	123	4	MOLECULE:	PUTATIVE MA
10:00	8foc-B	2.6	11.2	81	450	7	MOLECULE:	DNA PRIMASE
11:00	8cqr-A	2.6	6.4	71	103	3	MOLECULE:	NINJURIN-1
12:00	8uq9-a	2.6	14.8	83	433	7	MOLECULE:	E3 UBIQUITI
13:00	6cfw-C	2.6	9.1	89	114	6	MOLECULE:	MONOVALENT
14:00	3c3w-A	2.6	14.4	70	211	7	MOLECULE:	TWO COMPONE
15:00	8egi-B	2.6	4.5	76	146	11	MOLECULE:	HEMOGLOBIN
16:00	5ker-F	2.6	4.8	79	146	9	MOLECULE:	ALPHA-GLOBI
17:00	4ui9-N	2.6	29.2	116	631	8	MOLECULE:	ANAPHASE-PR
18:00	3w4u-F	2.6	4.4	78	145	10	MOLECULE:	HEMOGLOBIN
19:00	5x2t-D	2.6	4.7	81	145	11	MOLECULE:	HEMOGLOBIN
20:00	8hki-a	2.5	4.5	82	291	2	MOLECULE:	T-COMPLEX P
21:00	8t9f-K	2.5	17.1	82	265	7	MOLECULE:	HISTONE-LYS
22:00	4uis-B	2.5	20	90	241	1	MOLECULE:	GAMMA-SECRE
23:00	6psk-R	2.5	3.3	46	72	2	MOLECULE:	ANTIHOLIN
24:00:00	8tkp-A	2.5	26.7	125	614	8	MOLECULE:	TRANSMEMBRA
25:00:00	4hlq-C	2.5	13.4	70	281	7	MOLECULE:	TBC1 DOMAIN
26:00:00	7qog-M	2.5	4.1	50	126	6	MOLECULE:	PORTAL PROT

27:00:00	2kob-A	2.5	10	50	108	16	MOLECULE:	UNCHARACTER
28:00:00	2z0q-A	2.5	12.8	104	321	10	MOLECULE:	RHO GUANINE
29:00:00	6qzh-A	2.5	5	84	744	7	MOLECULE:	HUMAN CHEMO
30:00:00	5t58-N	2.5	13.7	74	196	7	MOLECULE:	KLLA0F02343
31:00:00	8ip4-A	2.5	28.5	103	320	10	MOLECULE:	MAGNESIUM T
32:00:00	1wa8-A	2.5	9.8	59	99	8	MOLECULE:	ESAT-6 LIKE
33:00:00	4q9u-A	2.5	22.6	121	303	8	MOLECULE:	RAB5 GDP/GT
34:00:00	1vzs-A	2.5	6.1	57	76	5	MOLECULE:	ATP SYNTHAS
35:00:00	7xpc-A	2.5	13.3	66	337	5	MOLECULE:	D-GLYCERATE
36:00:00	4pfr-B	2.5	4.1	63	229	5	MOLECULE:	TRAP DICARB
37:00:00	1y71-B	2.5	8.1	53	112	8	MOLECULE:	KINASE-ASSO
38:00:00	2f9i-A	2.5	11.8	64	309	3	MOLECULE:	ACETYL-COEN
39:00:00	7cun-G	2.5	22	100	895	7	MOLECULE:	INTEGRATOR
40:00:00	8g3a-D	2.5	17.4	66	334	3	MOLECULE:	BACITRACIN
41:00:00	2o57-A	2.5	11.4	76	282	3	MOLECULE:	PUTATIVE SA
42:00:00	6ahp-A	2.5	3.9	70	110	6	MOLECULE:	FLAGELLAR P
43:00:00	3bo0-A	2.5	6.9	103	442	5	MOLECULE:	23S RIBOSOM
44:00:00	4q2g-B	2.5	6	96	269	11	MOLECULE:	PHOSPHATIDA
45:00:00	8iqi-C	2.5	11.8	109	916	3	MOLECULE:	PUTATIVE PR
46:00:00	6ozu-A	2.5	11.2	65	235	5	MOLECULE:	EUKARYOTIC
47:00:00	6hty-B	2.5	4.7	71	302	11	MOLECULE:	NUCLEAR REC
48:00:00	2qqy-A	2.5	6.6	73	139	3	MOLECULE:	SIGMA B OPE
49:00:00	6eqo-A	2.5	16.4	127	1804	4	MOLECULE:	ACETYL-COEN
50:00:00	5che-A	2.5	4.2	60	427	3	MOLECULE:	GLUTAMYL-TR
51:00:00	8pzp-A	2.5	13.2	61	470	18	MOLECULE:	NUCLEOPROTE
52:00:00	7qh5-A	2.5	8.3	81	313	11	MOLECULE:	RNA POLYMER
53:00:00	6ye4-A	2.5	17.1	51	236	10	MOLECULE:	BIOPOLYMER
54:00:00	5way-A	2.5	22.2	81	482	4	MOLECULE:	TRANSCRIPTI
55:00:00	7ywq-A	2.5	2.8	51	79	14	MOLECULE:	GENOME POLY
56:00:00	7qcd-D	2.5	12.9	94	326	9	MOLECULE:	STRUCTURAL
57:00:00	5x41-F	2.5	10.2	82	244	6	MOLECULE:	COBALT ABC
58:00:00	4rp5-A	2.5	4.7	57	94	11	MOLECULE:	DISKS LARGE
59:00:00	4knf-C	2.5	4.9	81	232	5	MOLECULE:	BLUE-LIGHT
60:00:00	6xqk-A	2.5	4.1	46	47	4	MOLECULE:	CAMP-DEPEND
61:00:00	8syn-A	2.5	12.4	71	801	8	MOLECULE:	VPS35 ENDOS
62:00:00	7jv7-B	2.5	6.4	80	310	10	MOLECULE:	CTD KINASE
63:00:00	8ity-W	2.5	9.6	64	111	6	MOLECULE:	SNRNA-ACTIV
64:00:00	8cih-A	2.5	6.4	63	188	6	MOLECULE:	CYCLIN-DEPE
65:00:00	6vzd-E	2.5	8.1	61	85	5	MOLECULE:	PULMONARY S
66:00:00	8hfs-Y	2.5	8.3	75	249	3	MOLECULE:	MANNOSE-SPE
67:00:00	8ts6-A	2.5	17.4	66	500	5	MOLECULE:	PORTAL PROT
68:00:00	7xgr-A	2.5	26.7	96	546	1	MOLECULE:	GEM-ASSOCIA
69:00:00	5xmw-A	2.5	15.8	87	270	16	MOLECULE:	ZEARALENONE
70:00:00	5ip0-D	2.5	10.3	65	108	11	MOLECULE:	PHA GRANULE

71:00:00	6zu9-Q	2.5	14.2	74	226	8	MOLECULE:	18S RIBOSOM
72:00:00	7e5c-A	2.5	16.1	78	439	8	MOLECULE:	XAA-PRO DIP
73:00:00	4zs9-B	2.5	11	57	378	9	MOLECULE:	SUGAR BINDI
74:00:00	3r2x-C	2.5	2.8	55	82	9	MOLECULE:	HEMAGGLUTIN
75:00:00	5yde-A	2.5	5.4	56	111	9	MOLECULE:	PARAFIBROMI
76:00:00	8by2-A	2.5	14.1	74	548	8	MOLECULE:	GLUTATHIONE
77:00:00	1q8c-A	2.5	4	69	132	4	MOLECULE:	HYPOTHETICA
78:00:00	7y22-M	2.5	8.3	81	215	4	MOLECULE:	PHAGE CONNE
79:00:00	6nd4-J	2.5	23.8	83	493	11	MOLECULE:	ETS RRNA
80:00:00	1wpk-A	2.5	11.7	74	146	7	MOLECULE:	ADA REGULAT
81:00:00	6giq-Q	2.5	2.9	50	75	4	MOLECULE:	CYTOCHROME
82:00:00	7p2y-a	2.5	10.7	82	277	6	MOLECULE:	ATP SYNTHAS
83:00:00	6mur-B	2.5	9.6	56	162	9	MOLECULE:	UNCHARACTER
84:00:00	1ypy-A	2.5	7.9	78	182	9	MOLECULE:	VIRION MEMB
85:00:00	4xig-M	2.5	5.4	95	288	6	MOLECULE:	ALGM1
86:00:00	7s0r-A	2.5	2.8	45	85	7	MOLECULE:	C PROTEIN B
87:00:00	4kqt-A	2.5	6	59	169	7	MOLECULE:	PUTATIVE OU
88:00:00	3cjh-I	2.5	3.4	42	54	7	MOLECULE:	MITOCHONDRI
89:00:00	3ilk-A	2.5	12	78	239	3	MOLECULE:	UNCHARACTER
90:00:00	3kfw-X	2.5	8.7	74	234	3	MOLECULE:	UNCHARACTER
91:00:00	2owy-A	2.5	20	82	306	6	MOLECULE:	RECOMBINATI
92:00:00	8hp7-A	2.5	4.1	91	241	5	MOLECULE:	(S)-2-HALOA
93:00:00	8tsh-F	2.5	4.1	46	265	4	MOLECULE:	ABC TRANSPO
94:00:00	8ppr-D	2.5	15.5	80	248	9	MOLECULE:	KINETOCHORE
95:00:00	5kzb-A	2.5	8.8	58	104	3	MOLECULE:	VIRUS MATRI
96:00:00	7ogp-D	2.5	17.9	77	510	10	MOLECULE:	PHIKZ055,PH
97:00:00	2ehw-C	2.5	17.5	61	117	16	MOLECULE:	HYPOTHETICA
98:00:00	8ijq-A	2.5	7.6	105	264	4	MOLECULE:	SPHINGOMYEL
99:00:00	7jz2-A	2.5	14.6	91	481	11	MOLECULE:	SUCCINATE D
0:00	5ojl-A	2.5	6.2	78	284	9	MOLECULE:	IMINE REDUC
1:00	6wjv-A	2.5	22.4	113	1951	4	MOLECULE:	DNA POLYMER
2:00	6swy-1	2.5	14.6	80	542	0	MOLECULE:	VACUOLAR IM
3:00	7udk-A	2.5	12	62	172	8	MOLECULE:	DESIGNED HE
4:00	1zl8-B	2.5	3.1	41	54	5	MOLECULE:	LIN-7
5:00	7uv1-A	2.5	3.1	49	56	8	MOLECULE:	VICILIN-LIK
6:00	7jrg-H	2.5	2.9	50	65	2	MOLECULE:	MITOCHONDRI
7:00	4bzj-C	2.5	12.6	91	693	12	MOLECULE:	PROTEIN TRA
8:00	6tqe-A	2.5	17.9	103	634	9	MOLECULE:	ABC TRANSPO
9:00	5iwz-A	2.5	6.9	78	372	8	MOLECULE:	SYNAPTONEMA
10:00	8pm4-A	2.5	7.1	90	604	6	MOLECULE:	TRANSPOSASE
11:00	6zyx-K	2.5	3.2	48	95	13	MOLECULE:	DYNEIN HEAV
12:00	5z62-B	2.5	18.9	79	227	5	MOLECULE:	CYTOCHROME
13:00	7dl2-A	2.5	24.3	98	1127	8	MOLECULE:	HAMARTIN
14:00	7nnh-X	2.5	18.8	88	637	3	MOLECULE:	ERYTHROCYTE

15:00	7bin-L	2.5	10.6	57	75	11	MOLECULE:	FLAGELLAR B
16:00	6d5f-a	2.5	9.8	81	131	11	MOLECULE:	FIMBRIAL PR
17:00	3k66-A	2.5	19.2	85	217	5	MOLECULE:	BETA-AMYLOI
18:00	5b86-A	2.5	30.9	102	579	6	MOLECULE:	TUMOR NECRO
19:00	1yo7-A	2.5	5.2	46	120	0	MOLECULE:	REGULATORY
20:00	6jl7-A	2.5	21.6	90	444	4	MOLECULE:	TBC1 DOMAIN
21:00	5jki-A	2.5	3.8	82	201	7	MOLECULE:	PUTATIVE LI
22:00	1zxj-D	2.5	8.2	56	193	9	MOLECULE:	HYPOTHETICA
23:00	5kbw-B	2.5	8.7	59	171	7	MOLECULE:	RIBOFLAVIN
24:00:00	5h69-A	2.5	6.3	49	252	8	MOLECULE:	CHROMOSOME
25:00:00	7xdi-A	2.5	4.7	51	69	16	MOLECULE:	VP1
26:00:00	6k9k-A	2.5	11.9	83	2501	5	MOLECULE:	SERINE-PROT
27:00:00	2l3y-A	2.5	4.6	79	184	5	MOLECULE:	INTERLEUKIN
28:00:00	1l9l-A	2.5	6	41	74	2	MOLECULE:	GRANULYSIN
29:00:00	2mxx-A	2.5	16.6	104	179	10	MOLECULE:	AMYLASE-BIN
30:00:00	6gdj-A	2.5	5.5	47	71	11	MOLECULE:	MTO2
31:00:00	4x5m-B	2.5	10.2	58	93	10	MOLECULE:	UNCHARACTER
32:00:00	2ieq-B	2.5	11.4	60	89	13	MOLECULE:	SPIKE GLYCO
33:00:00	5b0l-B	2.5	8.2	102	334	8	MOLECULE:	MOEN5,DNA-B
34:00:00	7pkn-R	2.5	5.7	60	80	7	MOLECULE:	CENTROMERE
35:00:00	5ir2-A	2.5	4.6	58	222	5	MOLECULE:	CELLULASE
36:00:00	3cp8-A	2.5	6.6	94	611	4	MOLECULE:	TRNA URIDIN
37:00:00	1sf9-A	2.5	10.3	77	118	1	MOLECULE:	YFHH HYPOTH
38:00:00	7w0y-i	2.5	8.6	95	347	6	MOLECULE:	NADH DEHYDR
39:00:00	8k9z-A	2.5	17.8	122	405	5	MOLECULE:	RDTND-RID C
40:00:00	8psn-B	2.5	10.9	117	515	9	MOLECULE:	POLYMERASE
41:00:00	3rip-A	2.5	23.9	104	568	9	MOLECULE:	GAMMA-TUBUL
42:00:00	8y7g-B	2.5	9.5	74	543	1	MOLECULE:	CRISPR-ASSO
43:00:00	7x1i-A	2.5	10.8	94	678	5	MOLECULE:	ISOFORM 1 O
44:00:00	8hyj-D	2.5	11.1	58	117	2	MOLECULE:	DNA-DIRECTE
45:00:00	3njc-A	2.5	4.2	81	153	5	MOLECULE:	YSLB PROTEI
46:00:00	5eom-C	2.5	6.2	83	353	7	MOLECULE:	PROTEIN MAB
47:00:00	4c0n-A	2.5	4.9	78	150	6	MOLECULE:	2-ON-2 HEMO
48:00:00	7f3t-A	2.5	11	103	330	5	MOLECULE:	TRANSMEMBRA
49:00:00	7xsq-A	2.5	11.2	108	1289	6	MOLECULE:	RAMP SUPERF
50:00:00	3nlc-A	2.5	7.8	95	534	7	MOLECULE:	UNCHARACTER
51:00:00	5dy0-A	2.5	3.2	57	220	4	MOLECULE:	TETR FAMILY
52:00:00	6zvh-y	2.5	2.7	57	72	12	MOLECULE:	18S RRNA
53:00:00	1fyz-F	2.5	15.9	97	168	9	MOLECULE:	METHANE MON
54:00:00	7o3d-A	2.5	16.1	120	709	10	MOLECULE:	TRANSCRIPTI
55:00:00	8xat-A	2.5	4.7	70	187	4	MOLECULE:	TWO-COMPONE
56:00:00	8otx-A	2.5	18.6	131	1054	10	MOLECULE:	SPERM-SPECI
57:00:00	8pmq-9	2.5	27.5	103	412	4	MOLECULE:	E3 UBIQUITI
58:00:00	7wwb-A	2.5	6.4	86	333	9	MOLECULE:	CHOLINE TRA

59:00:00	1qgi-A	2.5	6.7	77	259	4	MOLECULE:	PROTEIN (CH
60:00:00	6h3w-A	2.5	5.3	87	246	2	MOLECULE:	ENVELOPMENT
61:00:00	7e38-B	2.5	9.8	92	305	1	MOLECULE:	DEOXYPODOPH
62:00:00	7k5c-A	2.5	19.4	111	650	6	MOLECULE:	INTERNAL VI
63:00:00	3wa8-A	2.5	6.4	63	179	6	MOLECULE:	CRISPR-ASSO
64:00:00	8e4g-0	2.5	7.5	71	196	7	MOLECULE:	PORTAL PROT
65:00:00	6zmq-A	2.5	9.4	97	589	5	MOLECULE:	CYTOCHROME
66:00:00	8aw5-A	2.5	10.4	96	266	9	MOLECULE:	HEME O OXYG
67:00:00	6ppl-B	2.5	20.7	102	665	9	MOLECULE:	N-ALPHA-ACE
68:00:00	4ap2-B	2.5	11.2	84	359	10	MOLECULE:	KELCH-LIKE
69:00:00	7awt-D	2.5	7.5	105	589	9	MOLECULE:	NADH-QUINON
70:00:00	4jbw-F	2.5	22.6	110	492	10	MOLECULE:	MALTOSE TRA
71:00:00	8xje-B	2.5	4.2	59	149	5	MOLECULE:	YQEY
72:00:00	6wgv-A	2.5	11.6	91	173	4	MOLECULE:	CORRINOID A
73:00:00	6qfd-B	2.5	2.9	46	116	11	MOLECULE:	DNA-BINDING
74:00:00	6fkf-d	2.5	8.1	86	179	5	MOLECULE:	ATP SYNTHAS
75:00:00	7z0s-G	2.5	9.3	72	250	13	MOLECULE:	FORMATE HYD
76:00:00	7bss-A	2.5	27.4	115	816	7	MOLECULE:	ATP11C
77:00:00	5xf9-D	2.5	8	99	455	9	MOLECULE:	NAD-REDUCIN
78:00:00	3jac-A	2.5	7	98	918	3	MOLECULE:	PIEZO-TYPE
79:00:00	8b17-A	2.5	9.7	65	451	9	MOLECULE:	DIPEPTIDE A
80:00:00	7pds-A	2.5	22.7	100	457	5	MOLECULE:	SIMILAR TO
81:00:00	8f2a-E	2.5	9	76	117	4	MOLECULE:	RECEPTOR AC
82:00:00	7oci-C	2.5	7.5	70	119	10	MOLECULE:	DOLICHYL-DI
83:00:00	3syk-A	2.5	6	73	290	3	MOLECULE:	PROTEIN CBB
84:00:00	4n4w-A	2.5	24.3	109	457	6	MOLECULE:	CYTOCHROME
85:00:00	3lss-B	2.5	19.3	85	468	9	MOLECULE:	SERYL-TRNA
86:00:00	6w2q-A	2.5	4.2	69	195	4	MOLECULE:	JUNCTION 34
87:00:00	7arc-E	2.5	4.9	87	235	9	MOLECULE:	PSST
88:00:00	4i1s-A	2.5	7.5	71	238	8	MOLECULE:	MELANOMA DI
89:00:00	3s6j-A	2.5	12.7	60	216	15	MOLECULE:	HYDROLASE,
90:00:00	6w38-A	2.5	6.2	90	331	4	MOLECULE:	TERMINAL NU
91:00:00	2be3-A	2.5	10.8	68	203	4	MOLECULE:	GTP PYROPHO
92:00:00	8gkh-P	2.5	11.3	96	600	7	MOLECULE:	DNA (5'-D(P
93:00:00	3jyy-B	2.5	7.5	90	266	4	MOLECULE:	LINCOSAMIDE
94:00:00	8odu-C	2.5	21.3	114	232	7	MOLECULE:	ATPASE GET3
95:00:00	4iwb-A	2.5	7.8	81	165	4	MOLECULE:	FLIC, FLIS
96:00:00	8c81-B	2.5	24.5	93	539	12	MOLECULE:	PROTEIN ORM
97:00:00	7qe5-A	2.5	10.2	83	616	5	MOLECULE:	SIALIC ACID
98:00:00	2gtt-K	2.5	15.2	92	420	7	MOLECULE:	NUCLEOPROTE
99:00:00	7jsr-A	2.5	7.6	78	1496	5	MOLECULE:	NAD-SPECIFI
0:00	1jr5-A	2.5	4.3	61	90	7	MOLECULE:	10 KDA ANTI
1:00	2qeu-B	2.5	4.9	58	133	7	MOLECULE:	PUTATIVE CA
2:00	7m5t-A	2.5	7.4	66	100	3	MOLECULE:	DE NOVO DES

3:00	1zp2-A	2.5	3.9	74	227	5	MOLECULE:	RNA POLYMER
4:00	6ulo-A	2.5	15.6	94	317	5	MOLECULE:	UNCHARACTER
5:00	4rng-C	2.5	3.9	64	83	5	MOLECULE:	MTN3/SALIVA
6:00	2d7d-A	2.5	17.5	87	621	3	MOLECULE:	5'-D(P*TP*T
7:00	5kqj-A	2.5	7.8	90	185	13	MOLECULE:	2''-AMINOGL
8:00	7zkq-C	2.5	22.9	97	416	9	MOLECULE:	NADH DEHYDR
9:00	3hrw-D	2.5	4.6	79	146	9	MOLECULE:	HEMOGLOBIN
10:00	2nup-C	2.4	4.2	52	137	10	MOLECULE:	PROTEIN TRA
11:00	7uv4-A	2.4	3.5	44	60	7	MOLECULE:	VICILIN PIS
12:00	6n63-A	2.4	9.9	77	141	9	MOLECULE:	ENCAPSULIN
13:00	8ebt-C	2.4	19.4	107	191	6	MOLECULE:	GENERAL TRA
14:00	7kzn-F	2.4	3.3	44	100	7	MOLECULE:	HEAVY CHAIN
15:00	7jqe-A	2.4	18.9	88	357	9	MOLECULE:	ESAT-6/WXG1
16:00	3c2g-A	2.4	26.4	102	618	4	MOLECULE:	SYS-1 PROTE
17:00	5ztc-A	2.4	16	63	197	2	MOLECULE:	LMO2088 PRO
18:00	8hel-A	2.4	2.8	42	68	5	MOLECULE:	ACRIE3
19:00	3sjb-D	2.4	10	65	72	8	MOLECULE:	ATPASE GET3
20:00	4i0x-F	2.4	9.9	59	85	12	MOLECULE:	ESAT-6-LIKE
21:00	1uj8-A	2.4	2.8	53	73	4	MOLECULE:	HYPOTHETICA
22:00	8env-A	2.4	6.5	92	504	7	MOLECULE:	SHEATH PROT
23:00	1dek-A	2.4	18.6	77	241	10	MOLECULE:	DEOXYNUCLEO
24:00:00	1bp3-A	2.4	4.1	80	186	4	MOLECULE:	PROTEIN (GR
25:00:00	6b2z-M	2.4	3.9	58	249	3	MOLECULE:	ATP SYNTHAS
26:00:00	8rc4-o	2.4	17.7	73	372	5	MOLECULE:	SERINE/THRE
27:00:00	7r5s-T	2.4	13.5	58	112	5	MOLECULE:	CENTROMERE
28:00:00	8eki-B	2.4	6.7	48	85	8	MOLECULE:	PROTEIN TRA
29:00:00	8e4y-A	2.4	9.4	89	651	8	MOLECULE:	GLYCEROL-3-
30:00:00	2fm8-C	2.4	15.7	88	220	8	MOLECULE:	SURFACE PRE
31:00:00	6lpf-B	2.4	22.7	82	1007	10	MOLECULE:	LEUCINE--TR
32:00:00	7dkh-I	2.4	10.2	64	914	6	MOLECULE:	RNA POLYMER
33:00:00	6lo8-F	2.4	3.7	55	84	9	MOLECULE:	MITOCHONDRI
34:00:00	2ew2-B	2.4	6.2	82	314	7	MOLECULE:	2-DEHYDROPA
35:00:00	5tgq-A	2.4	9.1	102	226	6	MOLECULE:	R.SWAI PROT
36:00:00	5a9j-A	2.4	15.6	96	805	7	MOLECULE:	DNA POLYMER
37:00:00	3t4r-A	2.4	3.3	49	72	10	MOLECULE:	PHOSPHOPROT
38:00:00	3f8f-A	2.4	10.5	57	114	11	MOLECULE:	TRANSCRIPTI
39:00:00	7evp-C	2.4	4.6	36	43	17	MOLECULE:	BETA SLIDIN
40:00:00	5i1u-B	2.4	10.6	80	294	8	MOLECULE:	GERMACRADIE
41:00:00	4fl2-A	2.4	5.9	70	555	6	MOLECULE:	TYROSINE-PR
42:00:00	7l48-A	2.4	11.5	93	522	9	MOLECULE:	CAS12F
43:00:00	6ulg-N	2.4	13	66	483	3	MOLECULE:	FOLLICULIN
44:00:00	3pmd-A	2.4	7.6	92	153	5	MOLECULE:	CONSERVED D
45:00:00	7wff-F	2.4	7.4	105	677	9	MOLECULE:	NAD(P)H-QUI
46:00:00	7p2p-C	2.4	20.6	84	174	6	MOLECULE:	SIGNAL PEPT

47:00:00	2fna-A	2.4	10.4	77	352	8	MOLECULE:	CONSERVED H
48:00:00	7l7v-A	2.4	7.7	56	121	7	MOLECULE:	PROBABLE DI
49:00:00	6ero-A	2.4	16.7	88	296	6	MOLECULE:	DIMETHYLADE
50:00:00	5eyb-A	2.4	12.3	81	340	2	MOLECULE:	DNA-BINDING
51:00:00	7r3e-A	2.4	18.3	96	545	3	MOLECULE:	2-AMINOBENZ
52:00:00	2nb2-A	2.4	1.9	34	38	12	MOLECULE:	NIGELLIN-1.
53:00:00	3aq0-C	2.4	8	63	327	2	MOLECULE:	GERANYL DIP
54:00:00	1upt-B	2.4	5.6	42	58	5	MOLECULE:	ADP-RIBOSYL
55:00:00	6vld-B	2.4	5.4	57	470	7	MOLECULE:	ALPHA-(1,6)
56:00:00	7cgp-J	2.4	13.3	69	83	7	MOLECULE:	MITOCHONDRI
57:00:00	6u05-A	2.4	22.6	84	413	4	MOLECULE:	TRNA LIGASE
58:00:00	3jbr-F	2.4	11.9	118	872	2	MOLECULE:	VOLTAGE-DEP
59:00:00	4ux3-B	2.4	4.2	55	71	9	MOLECULE:	STRUCTURAL
60:00:00	5vvr-B	2.4	28.4	95	1207	5	MOLECULE:	DNA-DIRECTE
61:00:00	2m7a-A	2.4	4.2	63	86	3	MOLECULE:	UNCHARACTER
62:00:00	8etc-h	2.4	18.7	80	121	5	MOLECULE:	RNA (2151-M
63:00:00	6f7s-C	2.4	9.9	65	319	8	MOLECULE:	SERRATE RNA
64:00:00	7zcm-B	2.4	6.6	55	66	9	MOLECULE:	SENSORY RHO
65:00:00	5gox-A	2.4	7.9	58	181	12	MOLECULE:	DNA REPAIR
66:00:00	6zyx-L	2.4	4.3	47	97	6	MOLECULE:	DYNEIN HEAV
67:00:00	8tdj-A	2.4	32.2	109	467	9	MOLECULE:	MECHANOSENS
68:00:00	6xi6-A	2.4	17.2	81	269	11	MOLECULE:	HELICAL FUS
69:00:00	7aqq-d	2.4	5.3	54	75	15	MOLECULE:	NADH-UBIQUI
70:00:00	2khq-A	2.4	15.7	50	110	6	MOLECULE:	INTEGRASE
71:00:00	7eu9-A	2.4	21.6	117	1078	7	MOLECULE:	CAS12I1 D64
72:00:00	4evv-A	2.4	5.5	66	145	8	MOLECULE:	METHYL-CPG-
73:00:00	7ahd-A	2.4	17.5	79	547	6	MOLECULE:	ABC-TYPE PR
74:00:00	7sn7-a	2.4	15.5	83	546	4	MOLECULE:	FLAGELLIN
75:00:00	7o4i-W	2.4	19.8	86	312	6	MOLECULE:	GENERAL TRA
76:00:00	7x29-B	2.4	14.6	87	1190	7	MOLECULE:	SPIKE GLYCO
77:00:00	7mrw-C	2.4	8.5	113	706	4	MOLECULE:	CYTOADHEREN
78:00:00	8k3h-A	2.4	11.2	91	480	10	MOLECULE:	PSEP
79:00:00	4bk0-B	2.4	11.4	75	97	9	MOLECULE:	ATP-DEPENDE
80:00:00	8ab6-Q	2.4	2.9	49	71	0	MOLECULE:	CYTOCHROME
81:00:00	8bdk-A	2.4	3.2	46	96	7	MOLECULE:	DNA-BINDING
82:00:00	4cc9-B	2.4	6.4	48	98	8	MOLECULE:	PROTEIN VPR
83:00:00	4dvy-P	2.4	16.6	110	656	4	MOLECULE:	CYTOTOXICIT
84:00:00	3eo8-A	2.4	16.6	68	219	4	MOLECULE:	BLUB-LIKE F
85:00:00	3bqp-B	2.4	6.7	42	80	7	MOLECULE:	PROACTIVATO
86:00:00	6nwo-D	2.4	5.1	63	165	5	MOLECULE:	TRANSCRIPTI
87:00:00	4dlq-A	2.4	9.3	74	353	8	MOLECULE:	LATROPHILIN
88:00:00	7np8-B	2.4	13.6	77	620	8	MOLECULE:	COENZYME F4
89:00:00	3zcj-A	2.4	9.3	90	205	6	MOLECULE:	CAGL
90:00:00	6nbu-A	2.4	4.5	73	126	8	MOLECULE:	CRISPR-ASSO

91:00:00	5yfp-E	2.4	30	119	776	7	MOLECULE:	EXOCYST COM
92:00:00	7mnn-A	2.4	14.6	93	750	11	MOLECULE:	E3 SUMO-PRO
93:00:00	5l09-B	2.4	2.4	38	164	3	MOLECULE:	QUORUM-SENS
94:00:00	3odn-A	2.4	8.8	71	366	6	MOLECULE:	DALLY-LIKE
95:00:00	2ksr-A	2.4	4.2	60	140	13	MOLECULE:	NEURONAL AC
96:00:00	4mt4-B	2.4	4.4	99	473	7	MOLECULE:	CMEC
97:00:00	4djg-A	2.4	5.5	40	49	5	MOLECULE:	PLECTIN-REL
98:00:00	7jzw-A	2.4	15.1	61	427	3	MOLECULE:	CRISPR TYPE
99:00:00	1p8c-C	2.4	6.7	63	119	8	MOLECULE:	CONSERVED H
0:00	6jly-G	2.4	4.5	75	363	9	MOLECULE:	TRANSLATION
1:00	6ygu-C	2.4	8.4	44	215	9	MOLECULE:	ATP DEPENDE
2:00	5muu-A	2.4	6.1	118	766	6	MOLECULE:	MAJOR INNER
3:00	8idb-D	2.4	6.6	84	295	13	MOLECULE:	CELL DIVISI
4:00	1f02-T	2.4	2.8	42	66	7	MOLECULE:	INTIMIN
5:00	1yns-A	2.4	4.5	83	254	11	MOLECULE:	E-1 ENZYME
6:00	2px0-A	2.4	10.9	50	258	8	MOLECULE:	FLAGELLAR B
7:00	6ynw-H	2.4	4.5	50	75	6	MOLECULE:	SUBUNIT C
8:00	8a9b-B	2.4	22.8	95	809	9	MOLECULE:	LIPID BINDI
9:00	4lnb-A	2.4	14.5	85	339	5	MOLECULE:	CAAX FARNES
10:00	7d3e-A	2.4	17.5	80	1380	4	MOLECULE:	DUAL OXIDAS
11:00	4ql6-B	2.4	3.4	66	509	11	MOLECULE:	CARBOXY-TER
12:00	1lj2-B	2.4	15.6	64	110	6	MOLECULE:	NONSTRUCTUR
13:00	6g7c-C	2.4	13	63	239	5	MOLECULE:	IMPA-RELATE
14:00	2kmd-A	2.4	4.2	71	111	0	MOLECULE:	PROTEIN C-E
15:00	6gw6-B	2.4	4.8	67	149	9	MOLECULE:	RES TOXIN
16:00	3kkb-B	2.4	5.7	71	128	6	MOLECULE:	SENSOR PROT
17:00	8wm8-B	2.4	6.1	91	265	9	MOLECULE:	NITRATE TRA
18:00	4djb-A	2.4	3.9	76	118	11	MOLECULE:	E4-ORF3
19:00	4kyw-A	2.4	7.9	76	254	9	MOLECULE:	TYPE-2 REST
20:00	2a2f-X	2.4	14.1	93	296	4	MOLECULE:	EXOCYST COM
21:00	7yzi-A	2.4	12	103	378	8	MOLECULE:	ADENYLATE C
22:00	8i24-F	2.4	12.3	68	233	10	MOLECULE:	DNA-DIRECTE
23:00	6wq2-A	2.4	7.3	69	154	3	MOLECULE:	A-DNA
24:00:00	6vr4-A	2.4	13.3	112	2166	5	MOLECULE:	DNA-DEPENDE
25:00:00	8gy3-A	2.4	11.2	83	440	6	MOLECULE:	CYTOCHROME
26:00:00	6v6d-A	2.4	20.6	82	209	11	MOLECULE:	PANNEXIN-1
27:00:00	5dpl-A	2.4	14.5	99	521	10	MOLECULE:	PROTEIN LYS
28:00:00	8f2r-E	2.4	9.1	84	205	8	MOLECULE:	COMM DOMAIN
29:00:00	8t1l-I	2.4	18.8	85	1111	13	MOLECULE:	MEDIATOR OF
30:00:00	2gta-A	2.4	5.8	68	98	3	MOLECULE:	HYPOTHETICA
31:00:00	7eew-A	2.4	20.9	112	611	8	MOLECULE:	TYPE I REST
32:00:00	7nj1-A	2.4	20.2	99	1448	12	MOLECULE:	SEPARIN
33:00:00	2qby-B	2.4	5.9	75	368	3	MOLECULE:	CELL DIVISI
34:00:00	6xss-A	2.4	6.8	60	260	7	MOLECULE:	C4_NAT_HFUS

35:00:00	2qv2-A	2.4	15.2	90	305	10	MOLECULE:	INOSITOL PO
36:00:00	1i5p-A	2.4	13.6	103	633	4	MOLECULE:	PESTICIDIAL
37:00:00	7oqe-K	2.4	17.2	87	406	0	MOLECULE:	PROTEIN NAM
38:00:00	5f8c-B	2.4	17	85	363	4	MOLECULE:	METHYLTRANS
39:00:00	5hiu-A	2.4	15.5	108	407	7	MOLECULE:	GTPASE ACTI
40:00:00	2e0w-B	2.4	12.1	103	500	9	MOLECULE:	GAMMA-GLUTA
41:00:00	4fhn-B	2.4	9.1	66	1022	6	MOLECULE:	NUCLEOPORIN
42:00:00	6eud-A	2.4	19	87	808	3	MOLECULE:	ATP-DEPENDE
43:00:00	6rjy-A	2.4	7.2	101	415	11	MOLECULE:	ARABINO-OLI
44:00:00	8g01-A	2.4	11.5	96	360	7	MOLECULE:	PHOSPHO-N-A
45:00:00	3qt2-C	2.4	11.4	83	110	4	MOLECULE:	INTERLEUKIN
46:00:00	3sjr-A	2.4	8.8	73	126	5	MOLECULE:	UNCHARACTER
47:00:00	4r29-B	2.4	10.6	87	204	3	MOLECULE:	UNCHARACTER
48:00:00	8w15-A	2.4	24.6	100	2431	5	MOLECULE:	HUNTINGTIN
49:00:00	6ibl-A	2.4	13.5	102	397	6	MOLECULE:	THIOREDOXIN
50:00:00	7s37-P	2.4	27	124	1033	10	MOLECULE:	CRISPR-ASSO
51:00:00	8adl-A	2.4	20.4	111	633	6	MOLECULE:	MTC5 ISOFOR
52:00:00	7pp4-f	2.4	8.9	57	142	7	MOLECULE:	DNA-DIRECTE
53:00:00	7vv6-R	2.4	15.1	89	271	6	MOLECULE:	MAS-RELATED
54:00:00	6s7t-H	2.4	8.3	73	112	4	MOLECULE:	DOLICHYL-DI
55:00:00	5yqz-R	2.4	16.8	140	558	9	MOLECULE:	GLUCAGON RE
56:00:00	8qkd-A	2.4	5.6	80	281	6	MOLECULE:	SC-CC-5-24
57:00:00	4tq4-D	2.4	6.7	91	290	3	MOLECULE:	PRENYLTRANS
58:00:00	4dwl-B	2.4	4	66	110	11	MOLECULE:	BBP7
59:00:00	7w72-U	2.4	9	97	420	8	MOLECULE:	PHOSPHATIDY
60:00:00	3d5l-A	2.4	5.3	68	203	3	MOLECULE:	REGULATORY
61:00:00	6yrf-A	2.4	20.4	114	777	9	MOLECULE:	VEGETATIVE
62:00:00	5bs1-D	2.4	5.2	76	121	1	MOLECULE:	CRRBCX-IIA
63:00:00	5xaz-A	2.4	4.1	74	223	3	MOLECULE:	GAMMA-BUTYR
64:00:00	3h9p-A	2.4	7.6	79	226	3	MOLECULE:	PUTATIVE TR
65:00:00	7nhr-C	2.4	24.7	93	565	6	MOLECULE:	PUTATIVE TR
66:00:00	6ty9-A	2.4	5.9	119	1208	8	MOLECULE:	RNA-DEPENDE
67:00:00	1sqf-A	2.4	7.3	83	425	2	MOLECULE:	SUN PROTEIN
68:00:00	8age-H	2.4	7.8	72	133	10	MOLECULE:	DOLICHYL-DI
69:00:00	7ljn-A	2.4	6.8	74	342	8	MOLECULE:	CD-NTASE
70:00:00	8ojy-C	2.4	5.4	106	952	4	MOLECULE:	PHOSPHOENOL
71:00:00	1knv-B	2.4	8.9	92	291	9	MOLECULE:	BSE634I RES
72:00:00	6hqa-A	2.4	19	108	887	3	MOLECULE:	TAF2
73:00:00	7pp2-A	2.4	21	100	442	4	MOLECULE:	EXOCYST SUB
74:00:00	8vc1-A	2.4	7.5	101	391	8	MOLECULE:	GUSTATORY R
75:00:00	4qvz-B	2.4	4	55	204	7	MOLECULE:	FRAGILE X M
76:00:00	2mab-A	2.4	3.7	78	109	8	MOLECULE:	ACINIFORM S
77:00:00	9b8o-e	2.4	6.2	59	80	5	MOLECULE:	ATPASE H+-T
78:00:00	5v8f-D	2.4	5.2	75	456	9	MOLECULE:	DNA REPLICA

79:00:00	7ye1-G	2.4	13.2	60	103	5	MOLECULE:	DNA-DIRECTE
80:00:00	3k1z-A	2.4	6.8	96	241	5	MOLECULE:	HALOACID DE
81:00:00	5b7j-A	2.4	3.6	71	110	8	MOLECULE:	SWITCH-ACTI
82:00:00	7xr2-a	2.4	7.5	62	274	6	MOLECULE:	VP3
83:00:00	5hea-A	2.4	10.7	57	276	5	MOLECULE:	PUTATIVE GL
84:00:00	4tkr-B	2.4	4.2	79	186	5	MOLECULE:	THIAMINE TR
85:00:00	2gsc-B	2.4	6.8	76	117	7	MOLECULE:	PUTATIVE UN
86:00:00	2v5d-A	2.4	11.2	82	722	5	MOLECULE:	O-GLCNACASE
87:00:00	2huj-A	2.4	10.5	78	125	10	MOLECULE:	LIN2004 PRO
88:00:00	4an8-A	2.4	4.8	73	468	8	MOLECULE:	CSE1
89:00:00	8tar-N	2.4	13.6	123	682	4	MOLECULE:	ANAPHASE-PR
90:00:00	6tnt-N	2.4	26.1	110	672	7	MOLECULE:	ANAPHASE-PR
91:00:00	8oqs-B	2.3	5.9	83	734	4	MOLECULE:	3-HYDROXYAC
92:00:00	1i2l-A	2.3	14.4	63	270	6	MOLECULE:	4-AMINO-4-D
93:00:00	5jw9-B	2.3	9.2	67	115	6	MOLECULE:	AF4/FMR2 FA
94:00:00	3h0g-A	2.3	25.7	117	1496	6	MOLECULE:	DNA-DIRECTE
95:00:00	7opl-A	2.3	18.5	119	1070	8	MOLECULE:	DNA POLYMER
96:00:00	6lo8-D	2.3	7.9	51	119	4	MOLECULE:	MITOCHONDRI
97:00:00	2iw3-B	2.3	12.5	96	980	1	MOLECULE:	ELONGATION
98:00:00	8t05-A	2.3	5	71	201	13	MOLECULE:	MYOMAKER
99:00:00	8dfu-0	2.3	6.7	54	84	7	MOLECULE:	PILIN PROTE
0:00	3dkq-A	2.3	5.5	57	230	5	MOLECULE:	PKHD-TYPE H
1:00	3ni7-A	2.3	4.7	72	174	11	MOLECULE:	BACTERIAL R
2:00	7bst-C	2.3	15.2	102	992	8	MOLECULE:	TYPE I REST
3:00	7wji-B	2.3	36.6	116	1663	4	MOLECULE:	PROTEIN UNC
4:00	6h08-B	2.3	9.9	84	297	5	MOLECULE:	CYTOCHROME
5:00	7lve-A	2.3	6.1	43	44	2	MOLECULE:	VICILIN JUG
6:00	5wcn-A	2.3	14.3	89	382	4	MOLECULE:	SIAD
7:00	7auf-A	2.3	3.1	47	89	6	MOLECULE:	SIMILAR TO
8:00	3f46-A	2.3	7.9	86	345	3	MOLECULE:	5,10-METHEN
9:00	1qdm-A	2.3	19.4	88	430	10	MOLECULE:	PROPHYTEPSI
10:00	6ayi-A	2.3	9.3	44	183	11	MOLECULE:	HTH-TYPE TR
11:00	7cun-D	2.3	17.1	96	827	5	MOLECULE:	INTEGRATOR
12:00	4i0x-K	2.3	8	59	77	10	MOLECULE:	ESAT-6-LIKE
13:00	7ag9-B	2.3	16.7	80	384	5	MOLECULE:	KAR9
14:00	3hjz-A	2.3	8.8	91	333	7	MOLECULE:	TRANSALDOLA
15:00	7mq1-B	2.3	9.8	59	83	8	MOLECULE:	COPPER-SENS
16:00	5n9j-D	2.3	12.4	74	135	5	MOLECULE:	MEDIATOR OF
17:00	2e62-A	2.3	3.4	46	61	9	MOLECULE:	PROTEIN AT5
18:00	6uxu-A	2.3	8.5	81	300	10	MOLECULE:	CHLOROTHALO
19:00	8jhk-A	2.3	24.1	110	727	7	MOLECULE:	ENGULFMENT
20:00	1s56-B	2.3	4.1	68	135	12	MOLECULE:	HEMOGLOBIN-
21:00	8tzk-E	2.3	14.4	59	101	2	MOLECULE:	CELL DIVISI
22:00	7ppo-A	2.3	7.7	111	500	6	MOLECULE:	UBIQUITINAT

23:00	3ogi-B	2.3	13.5	69	89	6	MOLECULE:	PUTATIVE ES
24:00:00	8c83-x	2.3	17	55	259	9	MOLECULE:	OTU DOMAIN-
25:00:00	6fvb-A	2.3	14.4	102	1001	8	MOLECULE:	IMPORTIN BE
26:00:00	7ab4-F	2.3	8	90	340	6	MOLECULE:	PREDICTED T
27:00:00	5fgn-A	2.3	10	98	536	9	MOLECULE:	LIPOOLIGOSA
28:00:00	7tjk-F	2.3	24	60	262	5	MOLECULE:	ORIGIN RECO
29:00:00	3hr0-A	2.3	11.6	74	250	4	MOLECULE:	COG4
30:00:00	2m6u-A	2.3	3.2	46	82	2	MOLECULE:	CHOLINE BIN
31:00:00	5xj5-A	2.3	6.7	88	200	7	MOLECULE:	GLYCEROL-3-
32:00:00	8kg9-E	2.3	19.1	72	568	6	MOLECULE:	DNA REPLICA
33:00:00	3wz4-G	2.3	8.1	45	140	2	MOLECULE:	DOTI
34:00:00	2h7f-X	2.3	11.2	79	314	5	MOLECULE:	5'-D(*TP*TP
35:00:00	4ayb-Q	2.3	3.5	40	50	10	MOLECULE:	DNA-DIRECTE
36:00:00	6mc9-A	2.3	17.2	80	150	6	MOLECULE:	SODIUM CHAN
37:00:00	4cpg-A	2.3	7.4	51	69	10	MOLECULE:	SMALL GLUTA
38:00:00	8ae6-W	2.3	14.5	75	941	4	MOLECULE:	NITROGEN PE
39:00:00	6jhm-A	2.3	13.2	82	494	11	MOLECULE:	CHLOROPHENO
40:00:00	2bsk-D	2.3	3.8	48	90	4	MOLECULE:	MITOCHONDRI
41:00:00	2oc6-A	2.3	9.3	45	124	2	MOLECULE:	YDHG PROTEI
42:00:00	2fzt-B	2.3	7.5	63	79	14	MOLECULE:	HYPOTHETICA
43:00:00	8k0b-A	2.3	16.7	100	689	3	MOLECULE:	CALCIUM PER
44:00:00	6juv-A	2.3	15.9	66	192	15	MOLECULE:	SEGREGATION
45:00:00	8w8q-C	2.3	8	98	287	9	MOLECULE:	SOLUBLE CYT
46:00:00	1urf-A	2.3	12.4	69	81	10	MOLECULE:	PROTEIN KIN
47:00:00	5ham-A	2.3	13.9	80	260	6	MOLECULE:	RICKCE
48:00:00	7b93-n	2.3	8.6	83	178	0	MOLECULE:	NADH-UBIQUI
49:00:00	5k7l-A	2.3	29.7	99	701	5	MOLECULE:	POTASSIUM V
50:00:00	8a3t-Q	2.3	27.2	93	623	3	MOLECULE:	ANAPHASE-PR
51:00:00	5ydc-B	2.3	6.6	65	122	5	MOLECULE:	UNCHARACTER
52:00:00	8ovw-U	2.3	17.2	78	184	5	MOLECULE:	CENTROMERE-
53:00:00	3iuk-A	2.3	20	88	552	3	MOLECULE:	UNCHARACTER
54:00:00	7k15-A	2.3	4.6	96	455	2	MOLECULE:	LEUKOTRIENE
55:00:00	7el9-A	2.3	33.4	132	1731	10	MOLECULE:	RNA-DIRECTE
56:00:00	5x3q-A	2.3	20	75	313	3	MOLECULE:	ENVELOPE GL
57:00:00	6iy8-A	2.3	17.7	86	469	3	MOLECULE:	POSITIVE RE
58:00:00	6c66-A	2.3	16.2	82	511	4	MOLECULE:	CRISPR-ASSO
59:00:00	7d7n-A	2.3	15.9	122	704	5	MOLECULE:	ATP-BINDING
60:00:00	2okx-A	2.3	15.8	107	954	5	MOLECULE:	RHAMNOSIDAS
61:00:00	5wb2-A	2.3	14.7	104	421	9	MOLECULE:	ENVELOPE PR
62:00:00	5nx9-D	2.3	15.2	104	477	5	MOLECULE:	ADENYLOSUCC
63:00:00	8g8i-A	2.3	21.8	78	465	5	MOLECULE:	C3HR3_9R_SH
64:00:00	1mje-A	2.3	26.4	108	600	9	MOLECULE:	5'-D(P*TP*T
65:00:00	3cfo-A	2.3	10.1	73	906	8	MOLECULE:	DNA POLYMER
66:00:00	6xp5-D	2.3	19.9	74	134	3	MOLECULE:	MEDIATOR OF

67:00:00	8adb-A	2.3	10.6	59	209	7	MOLECULE:	WC-VDT1
68:00:00	1uuj-D	2.3	5.2	64	79	5	MOLECULE:	PLATELET-AC
69:00:00	3b77-A	2.3	10.6	55	188	9	MOLECULE:	UNCHARACTER
70:00:00	7ef9-A	2.3	17.7	81	414	11	MOLECULE:	ADENINE DNA
71:00:00	8ghn-B	2.3	11.7	95	800	8	MOLECULE:	PROTEIN HIR
72:00:00	1ej6-D	2.3	13.2	106	417	8	MOLECULE:	LAMBDA2
73:00:00	7amy-A	2.3	23.4	77	353	3	MOLECULE:	FLAGELLAR B
74:00:00	7mbz-A	2.3	5.7	87	222	13	MOLECULE:	ABC TRANSPO
75:00:00	7krw-A	2.3	9	90	608	6	MOLECULE:	CHAPERONE P
76:00:00	6z2k-D	2.3	18.8	87	157	9	MOLECULE:	HISTONE DEA
77:00:00	7rfq-A	2.3	8.6	57	223	4	MOLECULE:	BETA CELL E
78:00:00	6ivh-A	2.3	3	48	94	15	MOLECULE:	SEGREGATION
79:00:00	8e4v-A	2.3	3.1	53	84	15	MOLECULE:	ISOFORM 3 O
80:00:00	7jmn-O	2.3	13.5	66	164	6	MOLECULE:	MEDIATOR OF
81:00:00	1k32-A	2.3	29.7	92	1023	7	MOLECULE:	TRICORN PRO
82:00:00	2ou3-A	2.3	6.2	76	160	9	MOLECULE:	TELLURITE R
83:00:00	8tef-A	2.3	27.7	99	516	4	MOLECULE:	RESPONSE RE
84:00:00	3v53-B	2.3	3.4	55	112	16	MOLECULE:	RNA-BINDING
85:00:00	2uy1-A	2.3	15.3	90	438	7	MOLECULE:	CLEAVAGE ST
86:00:00	7xuh-A	2.3	25	88	707	5	MOLECULE:	CHLORIDE AN
87:00:00	8hvj-A	2.3	5.5	66	281	9	MOLECULE:	UPF0701 PRO
88:00:00	6c6r-A	2.3	10	76	451	4	MOLECULE:	SQUALENE MO
89:00:00	7khw-a	2.3	9.6	52	174	2	MOLECULE:	TRANSLOCON
90:00:00	6mj1-A	2.3	4.2	79	200	4	MOLECULE:	PROBABLE HT
91:00:00	3hhc-B	2.3	11.3	60	157	5	MOLECULE:	INTERLEUKIN
92:00:00	8b9z-Y	2.3	16.5	90	167	3	MOLECULE:	NADH-UBIQUI
93:00:00	7pp6-B	2.3	23.3	101	1148	2	MOLECULE:	MUCIN-2
94:00:00	5xfs-A	2.3	4.8	47	78	4	MOLECULE:	PE FAMILY P
95:00:00	6c0f-w	2.3	6.9	51	70	10	MOLECULE:	SACCHAROMYC
96:00:00	2khv-A	2.3	3.9	48	106	0	MOLECULE:	PHAGE INTEG
97:00:00	8fcj-I	2.3	13.2	97	494	7	MOLECULE:	TYPE I-B CR
98:00:00	8cqv-A	2.3	16.1	76	202	3	MOLECULE:	NITROREDUCT
99:00:00	8ikj-R	2.3	15.7	84	532	14	MOLECULE:	ADHESION G
0:00	7qoj-E	2.3	3.6	49	238	12	MOLECULE:	PORTAL PROT
1:00	6ihr-A	2.3	7.2	70	257	7	MOLECULE:	PHOSPHORYLA
2:00	6wlz-Y	2.3	12.7	45	267	9	MOLECULE:	V-TYPE PROT
3:00	4xp7-A	2.3	9.2	72	324	13	MOLECULE:	TRNA-DIHYDR
4:00	5l4k-S	2.3	21.4	77	491	12	MOLECULE:	26S PROTEAS
5:00	7ugc-A	2.3	6.1	62	191	8	MOLECULE:	PROTEIN VIA
6:00	7cy6-A	2.3	4.8	72	872	6	MOLECULE:	MALTODEXTRI
7:00	6urt-A	2.3	17.5	91	331	4	MOLECULE:	LOW CONDUCT
8:00	7zmb-X	2.3	13.6	65	187	5	MOLECULE:	NADH-UBIQUI
9:00	8ewi-A	2.3	18.7	97	1775	10	MOLECULE:	E3 UBIQUITI
10:00	4bt9-B	2.3	21.8	85	238	2	MOLECULE:	PROLYL 4-HY

11:00	2fgy-A	2.3	9.1	108	471	8	MOLECULE:	CARBOXYSOME
12:00	8cli-A	2.3	12.4	109	542	6	MOLECULE:	GENERAL TRA
13:00	8btl-A	2.3	9.7	78	353	6	MOLECULE:	CDNA FLJ125
14:00	7upq-F	2.3	10.6	59	77	5	MOLECULE:	DHT03 PROTE
15:00	8rhf-A	2.3	16.7	97	327	6	MOLECULE:	LYSM PEPTID
16:00	6ird-B	2.3	23.2	66	254	5	MOLECULE:	1-PHOSPHATI
17:00	4oph-A	2.3	14.1	77	202	6	MOLECULE:	NONSTRUCTUR
18:00	5oc0-A	2.3	6.7	61	174	8	MOLECULE:	CYTOCHROME
19:00	8t8k-A	2.3	4.2	61	183	2	MOLECULE:	DUF507 FAMI
20:00	7apd-B	2.3	20.9	72	289	24	MOLECULE:	REPLICATION
21:00	5da8-K	2.3	6.4	86	433	8	MOLECULE:	60 KDA CHAP
22:00	3dee-A	2.3	9	64	200	5	MOLECULE:	PUTATIVE RE
23:00	6l80-C	2.3	10.1	74	107	3	MOLECULE:	GAMMA-TUBUL
24:00:00	7t62-A	2.3	16.4	79	561	3	MOLECULE:	GLYPICAN-2
25:00:00	4gmq-A	2.3	6.3	47	92	4	MOLECULE:	PUTATIVE RI
26:00:00	3kfu-G	2.3	7.9	48	86	4	MOLECULE:	NON-DISCRIM
27:00:00	8uz8-B	2.3	4.8	52	60	8	MOLECULE:	2-OXOGLUTAR
28:00:00	5cm8-A	2.3	17	70	419	9	MOLECULE:	RAL GUANINE
29:00:00	6wqz-A	2.3	10.2	112	536	4	MOLECULE:	AUTOPHAGY-R
30:00:00	2ixp-B	2.3	9	90	316	7	MOLECULE:	SERINE/THRE
31:00:00	3wqy-A	2.3	27.4	132	906	8	MOLECULE:	ALANINE--TR
32:00:00	3x38-B	2.3	3.2	39	79	13	MOLECULE:	MITOCHONDRI
33:00:00	5xa5-A	2.3	12	66	248	3	MOLECULE:	ALPHA-CATEN
34:00:00	5xsv-A	2.3	25.8	81	486	6	MOLECULE:	CHITINASE
35:00:00	4wrp-A	2.3	10.3	80	121	1	MOLECULE:	UNCHARACTER
36:00:00	6uek-A	2.3	10.1	100	660	8	MOLECULE:	UROCANATE H
37:00:00	6gw5-A	2.3	9	72	393	1	MOLECULE:	PUTATIVE OU
38:00:00	2oeb-A	2.3	3.8	77	152	5	MOLECULE:	HYPOTHETICA
39:00:00	7zaw-A	2.3	5.3	94	361	9	MOLECULE:	GLYPICAN-3
40:00:00	2h1n-A	2.3	17.6	109	566	4	MOLECULE:	OLIGOENDOPE
41:00:00	7vrc-C	2.3	7.8	72	109	6	MOLECULE:	TRANSCRIPTI
42:00:00	2euf-A	2.3	3	64	244	3	MOLECULE:	VIRAL CYCLI
43:00:00	3svi-A	2.3	3.2	58	79	10	MOLECULE:	TYPE III EF
44:00:00	6qyi-B	2.3	18.9	122	519	8	MOLECULE:	4-HYDROXYPH
45:00:00	4z7f-B	2.3	4.3	86	169	5	MOLECULE:	FOLATE ECF
46:00:00	6oiu-C	2.3	20.1	119	900	7	MOLECULE:	AMINOPEPTID
47:00:00	7vmb-A	2.3	18.3	99	359	6	MOLECULE:	IQ MOTIF AN
48:00:00	8yb7-C	2.3	10.2	96	455	4	MOLECULE:	PAPAIN-LIKE
49:00:00	8egr-G	2.3	5.8	59	313	7	MOLECULE:	GP15, RECEP
50:00:00	4hm9-A	2.3	19.1	112	511	2	MOLECULE:	BETA-CATENI
51:00:00	2k9a-A	2.3	8.9	95	136	7	MOLECULE:	ADP-RIBOSYL
52:00:00	5zi2-A	2.3	6.6	77	339	6	MOLECULE:	MALATE DEHY
53:00:00	7d7c-F	2.3	3.2	67	137	6	MOLECULE:	DNA-DIRECTE
54:00:00	8u53-A	2.3	9.1	110	574	7	MOLECULE:	CSC1-LIKE P

55:00:00	8emh-B	2.3	20.3	105	676	11	MOLECULE:	PROTEASE LO
56:00:00	6x58-F	2.3	12.3	70	135	3	MOLECULE:	10E8V4 FAB
57:00:00	8evm-C	2.3	10	71	237	8	MOLECULE:	CHLOROPHYLL
58:00:00	5yet-A	2.3	10.1	87	397	3	MOLECULE:	UNCHARACTER
59:00:00	5tr1-A	2.3	11.5	78	606	4	MOLECULE:	CHLORIDE CH
60:00:00	6xu2-A	2.3	23.9	109	1091	9	MOLECULE:	IMPORTIN-5
61:00:00	8qgy-B	2.3	9.2	73	809	10	MOLECULE:	MITOGEN-ACT
62:00:00	4l3u-A	2.3	6	71	123	8	MOLECULE:	UNCHARACTER
63:00:00	7e4n-A	2.3	6.3	79	290	6	MOLECULE:	SAT1646
64:00:00	4ysm-A	2.3	7	78	475	8	MOLECULE:	CALMODULIN-
65:00:00	8bsb-B	2.3	13.2	89	162	8	MOLECULE:	METHYL-ACCE
66:00:00	7njn-C	2.3	18.8	89	536	10	MOLECULE:	ATP SYNTHAS
67:00:00	7y3f-1	2.3	11.8	77	466	8	MOLECULE:	PHOTOSYSTEM
68:00:00	6f42-W	2.3	13.2	76	165	5	MOLECULE:	DNA-DIRECTE
69:00:00	1irx-B	2.3	14.1	84	508	7	MOLECULE:	LYSYL-TRNA
70:00:00	7v9x-A	2.3	15.1	74	314	11	MOLECULE:	RNA-DIRECTE
71:00:00	8xr6-q	2.3	7.8	58	143	2	MOLECULE:	PHOTOSYSTEM
72:00:00	7kpo-A	2.3	3.4	64	107	8	MOLECULE:	RESPONSE RE
73:00:00	8imi-0	2.3	27.3	121	1045	7	MOLECULE:	APCE
74:00:00	7x34-C	2.3	3.1	56	100	5	MOLECULE:	ZUOTIN
75:00:00	6lb8-C	2.3	11.9	94	483	1	MOLECULE:	ENDOLYSIN,C
76:00:00	2f07-A	2.3	8.4	73	197	7	MOLECULE:	YVDT
77:00:00	8hwi-B	2.3	4.5	67	177	12	MOLECULE:	CD-NTASE-AS
78:00:00	7wb4-g	2.3	6.8	65	672	3	MOLECULE:	OUTER NUP13
79:00:00	5oqm-n	2.3	21.8	74	136	7	MOLECULE:	DNA-DIRECTE
80:00:00	6xwi-A	2.3	3.5	57	66	7	MOLECULE:	S0_2.126
81:00:00	3pxp-A	2.3	10.4	74	292	11	MOLECULE:	HELIX-TURN-
82:00:00	5w99-A	2.3	7.9	84	319	4	MOLECULE:	PBTD
83:00:00	6h02-A	2.3	9.1	114	1334	8	MOLECULE:	MEDIATOR OF
84:00:00	7cgp-A	2.3	2.6	61	147	10	MOLECULE:	MITOCHONDRI
85:00:00	8gxl-B	2.3	9.9	64	122	6	MOLECULE:	SURP AND G-
86:00:00	7jmn-E	2.3	8.1	79	584	5	MOLECULE:	MEDIATOR OF
87:00:00	2w96-A	2.3	9.1	75	249	4	MOLECULE:	CELL DIVISI
88:00:00	6nf4-A	2.3	13.3	90	421	4	MOLECULE:	OTOPETRIN1
89:00:00	7k3z-A	2.3	9.5	85	564	7	MOLECULE:	60 KDA CHAP
90:00:00	2eul-A	2.3	7.2	79	156	5	MOLECULE:	ANTI-CLEAVA
91:00:00	5u30-A	2.3	18.1	81	1085	6	MOLECULE:	CRISPR-ASSO
92:00:00	4oe8-C	2.3	8.8	74	87	15	MOLECULE:	INTERLEUKIN
93:00:00	7woo-F	2.3	11.1	106	1622	8	MOLECULE:	NUCLEOPORIN
94:00:00	6qti-A	2.3	19.3	105	1038	8	MOLECULE:	NICOTINAMID
95:00:00	5v8f-6	2.3	9.2	117	692	8	MOLECULE:	DNA REPLICA
96:00:00	6aqk-A	2.3	14.3	69	216	7	MOLECULE:	TOXIN PROTE
97:00:00	1bmt-A	2.3	11	97	246	6	MOLECULE:	METHIONINE
98:00:00	8wtc-B	2.3	3.8	48	100	2	MOLECULE:	PROTEIN-ARG

99:00:00	1b48-A	2.3	5.1	79	221	11	MOLECULE:	PROTEIN (GL
0:00	6wb9-2	2.3	13.2	80	292	11	MOLECULE:	ENDOPLASMIC
1:00	4a01-A	2.3	7	106	740	7	MOLECULE:	PROTON PYRO
2:00	6mit-G	2.3	10.2	78	355	5	MOLECULE:	LIPOPOLYSAC
3:00	8dol-A	2.3	19.3	106	540	2	MOLECULE:	CAG PATHOGE
4:00	3kwo-A	2.3	7.5	77	149	5	MOLECULE:	PUTATIVE BA
5:00	8d00-A	2.3	13.2	92	299	7	MOLECULE:	MICROTUBULE
6:00	6cp6-Y	2.3	9.2	83	188	7	MOLECULE:	ATP SYNTHAS
7:00	8g1e-A	2.3	6.5	102	1037	10	MOLECULE:	ATP-CITRATE
8:00	6hcy-A	2.3	5.4	84	436	6	MOLECULE:	METALLOREDU
9:00	5y78-A	2.3	11.3	89	305	8	MOLECULE:	PUTATIVE HE
10:00	8kem-A	2.3	4.8	60	425	13	MOLECULE:	SDR FAMILY
11:00	6btm-F	2.3	17.4	113	402	6	MOLECULE:	ALTERNATIVE
12:00	8p2m-A	2.3	5.4	98	659	7	MOLECULE:	NAD(+) HYDR
13:00	6kn5-A	2.3	8.2	78	225	9	MOLECULE:	AF4/FMR2 FA
14:00	2lp4-A	2.3	20.1	110	225	5	MOLECULE:	CHEMOTAXIS
15:00	7k10-A	2.3	15.2	121	1259	6	MOLECULE:	DNA-DEPENDE
16:00	2g7l-A	2.3	7.4	68	201	9	MOLECULE:	TETR-FAMILY
17:00	6uhd-A	2.3	4	86	322	3	MOLECULE:	HISTONE ACE
18:00	2gtv-X	2.3	3.9	60	104	3	MOLECULE:	CHORISMATE
19:00	6cfz-J	2.3	22.3	80	134	5	MOLECULE:	ASK1
20:00	3mka-J	2.3	13	65	252	6	MOLECULE:	PROTEASOME
21:00	8h77-F	2.3	10.7	82	310	5	MOLECULE:	HEAT SHOCK
22:00	8cqn-B	2.3	28.3	102	187	7	MOLECULE:	LIPOPROTEIN
23:00	6vek-A	2.3	6.1	71	321	7	MOLECULE:	CONTACT-DEP
24:00:00	5dku-B	2.3	16.4	88	581	8	MOLECULE:	PREX DNA PO
25:00:00	5wtk-A	2.3	20.5	105	1215	7	MOLECULE:	CRISPR-ASSO
26:00:00	6wg7-H	2.3	8.2	72	219	1	MOLECULE:	DNA (35-MER
27:00:00	6wg7-D	2.3	8.2	72	219	1	MOLECULE:	DNA (35-MER
28:00:00	5wgg-A	2.2	14.9	85	441	2	MOLECULE:	RADICAL SAM
29:00:00	3c2b-A	2.2	13.6	63	200	6	MOLECULE:	TRANSCRIPTI
30:00:00	3euh-B	2.2	8.9	104	321	6	MOLECULE:	CHROMOSOME
31:00:00	7thw-A	2.2	8.6	72	158	11	MOLECULE:	PUTATIVE OM
32:00:00	6qeq-A	2.2	8.9	67	111	7	MOLECULE:	PCFF
33:00:00	7y6t-A	2.2	26.8	107	1124	8	MOLECULE:	SPIKE GLYCO
34:00:00	3cjh-B	2.2	3.8	49	59	2	MOLECULE:	MITOCHONDRI
35:00:00	7z0o-D	2.2	19.8	77	296	5	MOLECULE:	HISTONE H3
36:00:00	6cc4-A	2.2	12.8	75	608	7	MOLECULE:	SOLUBLE CYT
37:00:00	6hua-B	2.2	4.3	57	229	9	MOLECULE:	UNCHARACTER
38:00:00	4x86-A	2.2	3.9	38	51	8	MOLECULE:	UBIQUITIN-L
39:00:00	1jjo-A	2.2	3.1	35	40	6	MOLECULE:	NEUROSERPIN
40:00:00	7bst-A	2.2	3.7	48	384	8	MOLECULE:	TYPE I REST
41:00:00	2dsy-D	2.2	3.5	49	81	4	MOLECULE:	HYPOTHETICA
42:00:00	2fdr-A	2.2	6.5	54	222	11	MOLECULE:	CONSERVED H

43:00:00	1snl-A	2.2	12.1	61	99	3	MOLECULE:	NUCLEOBINDI
44:00:00	5chi-A	2.2	9.3	69	230	7	MOLECULE:	UNCHARACTER
45:00:00	6wg3-A	2.2	11.3	67	561	7	MOLECULE:	STRUCTURAL
46:00:00	7lqt-A	2.2	15.8	64	173	8	MOLECULE:	SERINE/THRE
47:00:00	8eau-p	2.2	4.3	40	56	3	MOLECULE:	V-TYPE PROT
48:00:00	5dfz-B	2.2	15.4	90	1224	2	MOLECULE:	VACUOLAR PR
49:00:00	7oq4-Z	2.2	4.4	52	118	0	MOLECULE:	DNA-DIRECTE
50:00:00	3rmi-A	2.2	13.2	48	105	10	MOLECULE:	CHORISMATE
51:00:00	7qru-G	2.2	10.2	85	113	7	MOLECULE:	NA+/H+ ANTI
52:00:00	5ncl-A	2.2	10.3	69	418	7	MOLECULE:	SERINE/THRE
53:00:00	5b3f-A	2.2	14.9	83	306	5	MOLECULE:	PHOSPHORIBU
54:00:00	2qq8-A	2.2	8.8	72	288	7	MOLECULE:	TBC1 DOMAIN
55:00:00	2hh6-A	2.2	9.1	50	112	8	MOLECULE:	BH3980 PROT
56:00:00	3cih-A	2.2	19.2	102	714	5	MOLECULE:	PUTATIVE AL
57:00:00	3u2r-A	2.2	3.6	44	135	2	MOLECULE:	REGULATORY
58:00:00	7xe4-F	2.2	19.2	79	1503	5	MOLECULE:	1,3-BETA-GL
59:00:00	7sox-A	2.2	16.6	93	514	8	MOLECULE:	EH DOMAIN-C
60:00:00	6bs3-A	2.2	13	61	330	8	MOLECULE:	PUTATIVE AT
61:00:00	8fvx-A	2.2	2.7	42	63	10	MOLECULE:	CBFD_NFYB_H
62:00:00	3fay-A	2.2	10	125	379	6	MOLECULE:	RAS GTPASE-
63:00:00	6xxv-C	2.2	9	54	111	6	MOLECULE:	ANTIBODY C5
64:00:00	3oao-A	2.2	10.2	66	140	11	MOLECULE:	UNCHARACTER
65:00:00	4eba-A	2.2	22.3	95	585	4	MOLECULE:	MRNA 3'-END
66:00:00	2azj-A	2.2	3.7	55	276	11	MOLECULE:	GERANYLGERA
67:00:00	7x5e-B	2.2	20	73	106	10	MOLECULE:	TRANSCRIPTI
68:00:00	3ufe-A	2.2	13.7	51	109	4	MOLECULE:	TRANSCRIPTI
69:00:00	1ufi-A	2.2	4.5	42	48	10	MOLECULE:	MAJOR CENTR
70:00:00	7t92-B	2.2	9.4	83	359	7	MOLECULE:	PEROXIN-12
71:00:00	1h0a-A	2.2	7.1	70	158	6	MOLECULE:	EPSIN
72:00:00	7ocn-A	2.2	11.2	98	690	4	MOLECULE:	HAD HYDROLA
73:00:00	5gmk-I	2.2	5.8	61	102	3	MOLECULE:	PRE-MRNA-SP
74:00:00	2d3o-1	2.2	4.3	59	100	3	MOLECULE:	23S RIBOSOM
75:00:00	6pc0-B	2.2	15.7	73	484	1	MOLECULE:	GUANOSINE P
76:00:00	8pjn-b	2.2	20.3	106	296	7	MOLECULE:	E3 UBIQUITI
77:00:00	5x62-B	2.2	20.5	98	388	2	MOLECULE:	CARNOSINE N
78:00:00	8jzr-B	2.2	11.5	108	456	10	MOLECULE:	LYSOSOMAL T
79:00:00	6rwt-A	2.2	5.4	71	181	7	MOLECULE:	UBIQUINOL-C
80:00:00	8r8q-A	2.2	10.1	80	391	6	MOLECULE:	MAJOR FACIL
81:00:00	9b8q-J	2.2	11.5	66	149	0	MOLECULE:	V-TYPE PROT
82:00:00	8th8-P	2.2	8.1	96	305	5	MOLECULE:	DYNEIN REGU
83:00:00	1kx5-A	2.2	14	73	135	10	MOLECULE:	DNA
84:00:00	7uuy-A	2.2	4.6	89	503	4	MOLECULE:	SODIUM/IODI
85:00:00	5mps-R	2.2	6.9	56	108	7	MOLECULE:	YEAST UBC4
86:00:00	6mdw-A	2.2	14.8	80	185	3	MOLECULE:	SPRT-LIKE D

87:00:00	5x55-A	2.2	7.8	66	276	6	MOLECULE:	PROBABLE UR
88:00:00	6btm-D	2.2	9.4	63	172	5	MOLECULE:	ALTERNATIVE
89:00:00	5e8b-A	2.2	4.3	40	54	3	MOLECULE:	RNA POLYMER
90:00:00	3edv-A	2.2	24.1	86	322	9	MOLECULE:	SPECTRIN BE
91:00:00	5w0p-C	2.2	24	117	850	3	MOLECULE:	ENDOLYSIN,R
92:00:00	7te2-A	2.2	9.4	62	205	10	MOLECULE:	AERR
93:00:00	2n72-A	2.2	6.8	53	69	6	MOLECULE:	GOLGI RESID
94:00:00	7d6v-C	2.2	4.8	57	264	5	MOLECULE:	SUCCINATE D
95:00:00	2lk2-A	2.2	4.3	59	89	5	MOLECULE:	HOMEOBOX PR
96:00:00	7qy5-B	2.2	10.7	67	320	3	MOLECULE:	NURS COMPLE
97:00:00	1ukf-A	2.2	4.8	61	188	13	MOLECULE:	AVIRULENCE
98:00:00	3hj1-B	2.2	16.7	71	387	11	MOLECULE:	MINOR EDITO
99:00:00	7zge-A	2.2	12.6	67	199	9	MOLECULE:	BRXA, A BRE
0:00	6xf1-A	2.2	23.2	88	230	9	MOLECULE:	NESPRIN-2
1:00	7dtl-A	2.2	6.8	53	61	6	MOLECULE:	PSK
2:00	8iqg-A	2.2	14	76	213	11	MOLECULE:	CHROMATIN A
3:00	5mbv-A	2.2	11.7	66	98	3	MOLECULE:	RECBCD ENZY
4:00	7wmv-A	2.2	18.9	104	602	6	MOLECULE:	SODIUM/GLUC
5:00	4psn-C	2.2	20.2	62	221	8	MOLECULE:	SSDNA BINDI
6:00	8teu-E	2.2	34.4	73	495	10	MOLECULE:	LARGE TEGUM
7:00	2rq5-A	2.2	5	75	121	13	MOLECULE:	PROTEIN JUM
8:00	8sl4-A	2.2	28.4	89	970	7	MOLECULE:	ADENYLATE C
9:00	4abn-B	2.2	18	89	426	7	MOLECULE:	TETRATRICOP
10:00	6y4b-A	2.2	10.9	69	255	6	MOLECULE:	RNA (77-MER
11:00	7bin-A	2.2	9	71	209	1	MOLECULE:	FLAGELLAR B
12:00	5htf-B	2.2	5.3	45	260	13	MOLECULE:	FOLDASE PRO
13:00	8hnp-A	2.2	6.9	62	141	10	MOLECULE:	ARCHAEAL TR
14:00	8ibw-C	2.2	12.3	86	922	3	MOLECULE:	DNA (60-MER
15:00	6n04-B	2.2	4.9	69	366	9	MOLECULE:	ABSH3
16:00	8eki-A	2.2	12.2	84	170	4	MOLECULE:	PROTEIN TRA
17:00	7kyp-F	2.2	12.5	89	290	8	MOLECULE:	MANGANESE A
18:00	5yti-A	2.2	5.4	42	330	2	MOLECULE:	FLAGELLAR H
19:00	2bf0-X	2.2	4	67	131	12	MOLECULE:	PCF11
20:00	4ily-B	2.2	4.7	71	250	8	MOLECULE:	CHITOSANASE
21:00	3lph-A	2.2	4.4	42	62	2	MOLECULE:	PROTEIN REV
22:00	7dm1-A	2.2	3.3	66	334	6	MOLECULE:	PHOSPHATE-B
23:00	3wee-A	2.2	23.1	81	428	5	MOLECULE:	ACTIN-LIKE
24:00:00	6iuq-B	2.2	15	77	296	6	MOLECULE:	PROLYL 4-HY
25:00:00	6nd4-I	2.2	29.8	85	487	5	MOLECULE:	ETS RRNA
26:00:00	1d2t-A	2.2	5.2	89	222	8	MOLECULE:	ACID PHOSPH
27:00:00	8ppr-M	2.2	15.2	63	205	5	MOLECULE:	KINETOCHORE
28:00:00	6tdw-C	2.2	10.3	70	157	11	MOLECULE:	ATPTB1
29:00:00	3veb-B	2.2	13	58	150	16	MOLECULE:	MACRODOMAIN
30:00:00	2np5-A	2.2	13.3	69	187	10	MOLECULE:	TRANSCRIPTI

31:00:00	7vxs-m	2.2	5.2	57	175	4	MOLECULE:	NADH DEHYDR
32:00:00	8p23-B	2.2	12.1	110	715	6	MOLECULE:	ANAEROBIC R
33:00:00	1hs7-A	2.2	5.3	58	97	5	MOLECULE:	SYNTAXIN VA
34:00:00	5mu4-A	2.2	4.9	68	196	4	MOLECULE:	TAIL TUBULA
35:00:00	5ujc-A	2.2	8	88	267	8	MOLECULE:	MMACHC-LIKE
36:00:00	6v4m-A	2.2	4.6	58	163	7	MOLECULE:	BCL-2
37:00:00	4okv-F	2.2	3.1	43	66	12	MOLECULE:	HEAVY CHAIN
38:00:00	8w5z-A	2.2	13.8	77	333	1	MOLECULE:	PROTEIN-TYR
39:00:00	1qhh-C	2.2	10.9	57	102	7	MOLECULE:	PROTEIN (PC
40:00:00	1dj3-A	2.2	13.2	78	432	6	MOLECULE:	ADENYLOSUCC
41:00:00	7kpx-C	2.2	12.6	103	1352	3	MOLECULE:	MEIOTIC MRN
42:00:00	3c1d-B	2.2	14.8	69	154	12	MOLECULE:	REGULATORY
43:00:00	5i0n-A	2.2	10.8	86	489	13	MOLECULE:	PHOSPHATIDY
44:00:00	7qqe-A	2.2	3.3	66	163	3	MOLECULE:	NUCLEAR FAC
45:00:00	6dkm-D	2.2	9.5	59	79	3	MOLECULE:	DHD131_A
46:00:00	5gpa-A	2.2	13.9	72	189	8	MOLECULE:	TRANSCRIPTI
47:00:00	5fqf-A	2.2	5.5	48	583	2	MOLECULE:	BETA-N-ACET
48:00:00	5v8z-C	2.2	8	65	98	11	MOLECULE:	ENDOPLASMIC
49:00:00	7alw-A	2.2	15.6	71	344	4	MOLECULE:	LOW CALCIUM
50:00:00	2es4-E	2.2	15.8	95	278	6	MOLECULE:	LIPASE
51:00:00	3bfm-A	2.2	3.4	60	230	8	MOLECULE:	BIOTIN PROT
52:00:00	3d9d-A	2.2	9.8	102	432	10	MOLECULE:	NITROALKANE
53:00:00	7amv-K	2.2	19.5	80	708	8	MOLECULE:	DNA-DIRECTE
54:00:00	8jgw-A	2.2	18.2	76	500	7	MOLECULE:	EXOPOLYPHOS
55:00:00	1nl3-A	2.2	20.9	102	839	4	MOLECULE:	PREPROTEIN
56:00:00	5oqq-B	2.2	24	111	824	3	MOLECULE:	CONDENSIN C
57:00:00	5j10-A	2.2	9.3	53	70	11	MOLECULE:	PEPTIDE DES
58:00:00	2n8p-A	2.2	7	41	53	15	MOLECULE:	LACTICIN Q
59:00:00	3jb9-r	2.2	23.3	74	573	3	MOLECULE:	PRE-MRNA-SP
60:00:00	3g3z-A	2.2	13.6	76	142	5	MOLECULE:	TRANSCRIPTI
61:00:00	7rsl-A	2.2	3.3	46	235	9	MOLECULE:	SEIPIN
62:00:00	4hw5-A	2.2	3.3	39	77	8	MOLECULE:	COMPLEMENT
63:00:00	5ndd-A	2.2	9.1	103	567	9	MOLECULE:	LYSOZYME,PR
64:00:00	2g49-A	2.2	17.3	108	970	7	MOLECULE:	INSULIN-DEG
65:00:00	5j2l-A	2.2	9.3	59	76	7	MOLECULE:	PROTEIN DES
66:00:00	7upo-A	2.2	8.2	57	74	2	MOLECULE:	DHT03 PROTE
67:00:00	6zyx-C	2.2	23.4	103	278	5	MOLECULE:	DYNEIN HEAV
68:00:00	8ssk-D	2.2	11.1	61	306	5	MOLECULE:	CONTACT-DEP
69:00:00	3txs-C	2.2	7.9	53	91	4	MOLECULE:	TERMINASE D
70:00:00	6s8g-G	2.2	9.3	92	244	14	MOLECULE:	LIPOPOLYSAC
71:00:00	3tek-A	2.2	2.8	50	139	8	MOLECULE:	THERMODBP-S
72:00:00	2hi3-A	2.2	4.1	59	73	2	MOLECULE:	HOMEODOMAIN
73:00:00	8bmw-E	2.2	3.2	57	122	11	MOLECULE:	CRISPR-ASSO
74:00:00	7msk-A	2.2	21.9	107	421	8	MOLECULE:	GLYCO_TRANS

75:00:00	2gmf-A	2.2	4.6	72	121	4	MOLECULE:	GRANULOCYTE
76:00:00	6zfb-d	2.2	17	78	139	3	MOLECULE:	DNA-DIRECTE
77:00:00	7cth-E	2.2	5.1	70	75	6	MOLECULE:	CORE PROTEI
78:00:00	6o0y-A	2.2	6.6	105	1146	3	MOLECULE:	CRISPR-ASSO
79:00:00	6nvz-A	2.2	2.7	47	60	6	MOLECULE:	MYB FAMILY
80:00:00	5nug-A	2.2	9.6	92	2920	5	MOLECULE:	CYTOPLASMIC
81:00:00	3ajb-A	2.2	6.4	82	293	6	MOLECULE:	PEROXISOMAL
82:00:00	8rd2-B	2.2	3.7	59	400	8	MOLECULE:	INVARIANT S
83:00:00	8bb6-A	2.2	6.3	69	476	4	MOLECULE:	SUCROSE TRA
84:00:00	7unk-A	2.2	27.5	150	1047	7	MOLECULE:	IMPORTIN-4
85:00:00	5a2v-B	2.2	11.1	69	472	4	MOLECULE:	MITOCHONDRI
86:00:00	7qpg-B	2.2	21.7	111	591	5	MOLECULE:	PROTEIN ZWI
87:00:00	8e1u-A	2.2	7.9	85	1126	6	MOLECULE:	PPI-DEPENDE
88:00:00	7yxx-A	2.2	27.1	125	1754	6	MOLECULE:	PROBABLE UB
89:00:00	7nj0-C	2.2	13	69	270	3	MOLECULE:	SECURIN,SEP
90:00:00	3llw-D	2.2	8.6	73	289	3	MOLECULE:	GERANYLTRAN
91:00:00	2ewg-A	2.2	13.8	90	367	8	MOLECULE:	FARNESYL PY
92:00:00	3pvl-A	2.2	26.4	89	597	10	MOLECULE:	MYOSIN VIIA
93:00:00	8h2h-D	2.2	14.2	78	599	1	MOLECULE:	LTRB
94:00:00	7xn2-A	2.2	3.3	69	148	7	MOLECULE:	ALR3614 PRO
95:00:00	2r44-A	2.2	7.1	67	330	9	MOLECULE:	UNCHARACTER
96:00:00	7qba-D	2.2	4.7	85	275	5	MOLECULE:	PROBABLE AB
97:00:00	8umd-B	2.2	13.8	68	331	10	MOLECULE:	FLAGELLAR M
98:00:00	8d8l-8	2.2	5.8	86	452	2	MOLECULE:	PROBABLE S-
99:00:00	1l1l-A	2.2	16.1	84	717	7	MOLECULE:	RIBONUCLEOS
0:00	8jo3-A	2.2	7.2	85	350	5	MOLECULE:	SUBSTRATE O
1:00	3bve-A	2.2	8.2	81	173	5	MOLECULE:	FERRITIN
2:00	3lpz-A	2.2	8.8	95	314	3	MOLECULE:	UNCHARACTER
3:00	6p8v-G	2.2	7.3	65	320	5	MOLECULE:	ATPASE, AAA
4:00	4w8j-A	2.2	20.7	100	1017	8	MOLECULE:	PESTICIDAL
5:00	2rbd-A	2.2	10.4	83	159	2	MOLECULE:	BH2358 PROT
6:00	2qkm-B	2.2	6.1	84	266	4	MOLECULE:	SPBC3B9.21
7:00	5z7c-A	2.2	11.7	100	414	6	MOLECULE:	3'3'-CGAMP-
8:00	2mn2-A	2.2	9.3	77	122	4	MOLECULE:	YMOB
9:00	7ux8-A	2.2	11.3	83	380	11	MOLECULE:	MFNG
10:00	4v19-2	2.2	13.8	89	178	6	MOLECULE:	MITORIBOSOM
11:00	2k3q-A	2.2	4	68	118	12	MOLECULE:	TUSP1
12:00	2nn4-A	2.2	3.5	53	62	9	MOLECULE:	HYPOTHETICA
13:00	4i9e-A	2.2	18.3	100	383	6	MOLECULE:	RESPONSE RE
14:00	3o77-A	2.2	3.2	62	388	3	MOLECULE:	MYOSIN LIGH
15:00	3l9f-A	2.2	6.6	67	170	4	MOLECULE:	PUTATIVE UN
16:00	6vrc-A	2.2	12.4	103	1007	10	MOLECULE:	CRISPR-ASSO
17:00	4kis-A	2.2	7.1	67	315	6	MOLECULE:	PUTATIVE IN
18:00	4uxv-A	2.2	13.8	92	522	8	MOLECULE:	SEPTATION R

19:00	5b4s-B	2.2	5.1	75	310	3	MOLECULE:	CHITOSANASE
20:00	5lxf-B	2.2	5.3	83	149	6	MOLECULE:	MACROPHAGE
21:00	2l6f-A	2.2	8.6	92	176	5	MOLECULE:	FOCAL ADHES
22:00	7yzb-A	2.2	3.6	56	125	7	MOLECULE:	FORKHEAD BO
23:00	7nyw-C	2.2	11.2	68	335	10	MOLECULE:	CHROMOSOME
24:00:00	2hi4-A	2.2	5	123	480	5	MOLECULE:	CYTOCHROME
25:00:00	2kmf-A	2.2	9.1	68	115	4	MOLECULE:	PHOTOSYSTEM
26:00:00	2hxo-A	2.2	5.2	57	207	11	MOLECULE:	PUTATIVE TE
27:00:00	6hgc-A	2.2	12.6	81	304	6	MOLECULE:	UBIQUITIN C
28:00:00	6nmi-B	2.2	9.3	101	760	8	MOLECULE:	GENERAL TRA
29:00:00	1mwk-A	2.2	10.2	88	320	3	MOLECULE:	PARM
30:00:00	3ljl-A	2.2	7.4	59	149	7	MOLECULE:	TRANSCRIPTI
31:00:00	5awf-A	2.2	5.9	70	385	0	MOLECULE:	FES CLUSTER
32:00:00	7w7p-D	2.2	17.7	96	284	6	MOLECULE:	DNA HELICAS
33:00:00	5vyl-A	2.2	16.2	92	502	5	MOLECULE:	INNER TEGUM
34:00:00	6i3m-E	2.2	7.3	88	354	8	MOLECULE:	TRANSLATION
35:00:00	2fdc-A	2.2	17.4	89	505	4	MOLECULE:	5'-D(P*CP*G
36:00:00	4cz8-A	2.2	16.8	122	422	6	MOLECULE:	NA+/H+ ANTI
37:00:00	3fmt-A	2.2	4.5	72	162	7	MOLECULE:	PROTEIN SEQ
38:00:00	7wvz-A	2.2	12.9	110	1719	11	MOLECULE:	BETA-KETOAC
39:00:00	8hra-C	2.2	6.9	76	852	12	MOLECULE:	ARCHAEAL AT
40:00:00	7etm-A	2.2	22.4	92	498	5	MOLECULE:	PORTAL PROT
41:00:00	3h3m-A	2.2	6.8	70	89	4	MOLECULE:	FLAGELLAR P
42:00:00	2js1-A	2.2	4.3	63	80	8	MOLECULE:	UNCHARACTER
43:00:00	8q9t-E	2.2	16.5	82	1060	7	MOLECULE:	ANTIVIRAL H
44:00:00	4bm5-A	2.2	14.5	94	268	6	MOLECULE:	SIMILAR TO
45:00:00	6s6b-K	2.2	13.9	102	1024	2	MOLECULE:	CRISPR-ASSO
46:00:00	4buj-A	2.2	24	85	903	11	MOLECULE:	SUPERKILLER
47:00:00	1sc7-A	2.2	7.2	88	567	5	MOLECULE:	5'-D(*AP*AP
48:00:00	8thc-A	2.2	4.4	55	479	9	MOLECULE:	ELG1 ISOFOR
49:00:00	8kde-D	2.2	17.1	110	351	5	MOLECULE:	PHOTOSYSTEM
50:00:00	6ef3-A	2.2	8.3	84	247	6	MOLECULE:	PROTEASOME
51:00:00	7vt4-A	2.2	9.1	82	438	9	MOLECULE:	ENDOGLUCANA
52:00:00	5aqd-M	2.2	7	83	184	6	MOLECULE:	PHYCOERYTHR
53:00:00	4otn-A	2.2	11.9	54	123	11	MOLECULE:	EUKARYOTIC
54:00:00	8iwh-q	2.2	8.1	58	156	9	MOLECULE:	PHOTOSYSTEM
55:00:00	7mi8-A	2.2	12	97	868	5	MOLECULE:	FUSION PROT
56:00:00	8wr4-B	2.2	31.2	103	1442	5	MOLECULE:	CBCAS9 EFFE
57:00:00	4ng2-E	2.2	8.9	62	94	6	MOLECULE:	TRANSCRIPTI
58:00:00	5aor-A	2.2	7.1	112	1009	6	MOLECULE:	DOSAGE COMP
59:00:00	5utg-A	2.2	5.2	73	134	3	MOLECULE:	EGG-LYSIN
60:00:00	6jc4-A	2.2	3.9	68	200	6	MOLECULE:	UREASE ACCE
61:00:00	7c6g-A	2.2	3.8	55	420	11	MOLECULE:	SUGAR ABC T
62:00:00	6vqx-A	2.2	3.6	65	97	11	MOLECULE:	ACRF6

63:00:00	6xpd-A	2.2	18.6	106	303	5	MOLECULE:	ZINC TRANSP
64:00:00	2ia0-A	2.2	20	59	156	8	MOLECULE:	PUTATIVE HT
65:00:00	6m9t-A	2.2	12.2	77	444	6	MOLECULE:	PROSTAGLAND
66:00:00	8hrc-A	2.2	21.8	102	776	6	MOLECULE:	ADENOSINE D
67:00:00	4hh0-A	2.2	14.8	89	383	7	MOLECULE:	APPA PROTEI
68:00:00	7z12-A	2.2	5.1	122	1853	10	MOLECULE:	PAM1.4, HEA
69:00:00	5jpw-A	2.2	6.1	78	143	8	MOLECULE:	DYNEIN LIGH
70:00:00	8p0v-M	2.2	14.7	92	357	5	MOLECULE:	COILED-COIL
71:00:00	5w5y-O	2.2	10.4	102	640	7	MOLECULE:	DNA-DIRECTE
72:00:00	8hhe-B	2.2	8.4	103	647	7	MOLECULE:	CRYSTALINE
73:00:00	6e6y-A	2.2	9.9	98	268	8	MOLECULE:	DIECKMANN C
74:00:00	7wiu-B	2.2	18.2	95	576	7	MOLECULE:	MYCOBACTIN
75:00:00	5b2o-A	2.2	20	123	1455	7	MOLECULE:	CRISPR-ASSO
76:00:00	3cra-A	2.2	7.6	94	239	12	MOLECULE:	PROTEIN MAZ
77:00:00	5kkl-B	2.2	24.8	107	838	3	MOLECULE:	PUTATIVE PO
78:00:00	8j98-A	2.2	4.1	75	184	7	MOLECULE:	NEAR-INFRAR
79:00:00	2wzk-A	2.2	6	80	369	9	MOLECULE:	CULLIN-5
80:00:00	5lq4-B	2.2	22.2	89	465	10	MOLECULE:	CYAGOX
81:00:00	5oqj-1	2.2	28.2	99	491	6	MOLECULE:	DNA-DIRECTE
82:00:00	6upn-A	2.2	6.7	78	245	4	MOLECULE:	ENDOPHILIN-
83:00:00	7zcv-B	2.2	14.5	73	285	3	MOLECULE:	TRANSCRIPTI
84:00:00	8pda-A	2.2	7	94	973	3	MOLECULE:	ERAD-ASSOCI
85:00:00	7sfj-A	2.2	8.8	92	284	9	MOLECULE:	CHRMINE
86:00:00	7fbo-A	2.2	10.3	82	283	9	MOLECULE:	BEZA
87:00:00	2q83-A	2.2	6.8	65	332	6	MOLECULE:	YTAA PROTEI
88:00:00	1cf7-B	2.2	4	69	82	4	MOLECULE:	DNA (5'-
89:00:00	7emf-G	2.2	20.4	87	161	6	MOLECULE:	MEDIATOR OF
90:00:00	5lo9-A	2.2	3.7	81	471	4	MOLECULE:	CYTOCHROME
91:00:00	6b85-A	2.2	21.8	104	215	8	MOLECULE:	TMHC4_R
92:00:00	8p0w-J	2.2	17.1	68	202	12	MOLECULE:	COMM DOMAIN
93:00:00	7jw3-C	2.2	10.1	94	244	9	MOLECULE:	EXONUCLEASE
94:00:00	4lev-A	2.2	4.2	72	369	4	MOLECULE:	CYCLIC GMP-
95:00:00	6lg3-A	2.2	3.2	57	74	7	MOLECULE:	PHIRV1 PHAG
96:00:00	6wfq-D	2.2	8.2	72	219	1	MOLECULE:	HTH-TYPE TR
97:00:00	8f2r-D	2.1	17	60	199	7	MOLECULE:	COMM DOMAIN
98:00:00	6twr-A	2.1	7.6	74	169	9	MOLECULE:	BETA-GLUCOS
99:00:00	8utk-A	2.1	3.8	37	55	14	MOLECULE:	23R-B04DSLF
0:00	1kr7-A	2.1	4.2	61	110	13	MOLECULE:	NEURAL GLOB
1:00	6m3q-F	2.1	20.6	108	301	10	MOLECULE:	ANKYRIN-2
2:00	6lk8-a	2.1	24.8	120	1272	8	MOLECULE:	MGC83295 PR
3:00	6s3f-A	2.1	2.9	49	65	6	MOLECULE:	2S ALBUMIN
4:00	4ryk-A	2.1	15.7	71	295	3	MOLECULE:	LMO0325 PRO
5:00	4rvq-A	2.1	5.7	69	122	14	MOLECULE:	PRE-MRNA SP
6:00	8a2n-B	2.1	4.3	82	299	9	MOLECULE:	CGND

7:00	3qnk-C	2.1	25.3	84	511	6	MOLECULE:	PUTATIVE LI
8:00	6f2d-F	2.1	14.9	65	258	2	MOLECULE:	FLAGELLAR B
9:00	7yul-A	2.1	3.6	55	92	5	MOLECULE:	BEN DOMAIN-
10:00	6gmh-Q	2.1	29.4	84	884	10	MOLECULE:	RPB1
11:00	8d8j-5	2.1	7.7	76	270	7	MOLECULE:	PROBABLE S-
12:00	1gnc-A	2.1	15.5	63	175	14	MOLECULE:	GRANULOCYTE
13:00	7nyw-B	2.1	32.7	92	858	4	MOLECULE:	CHROMOSOME
14:00	6zbj-B	2.1	26	124	501	2	MOLECULE:	PRECURSOR O
15:00	5lb7-B	2.1	5.8	53	78	6	MOLECULE:	KATANIN P80
16:00	8oix-b	2.1	3.9	62	208	5	MOLECULE:	FAMILY T1,
17:00	7uqj-A	2.1	10	62	585	6	MOLECULE:	ATPASE HIST
18:00	7v2c-p	2.1	6.5	76	178	3	MOLECULE:	NADH DEHYDR
19:00	5fia-B	2.1	4.9	79	397	8	MOLECULE:	LPIR1
20:00	8th8-A	2.1	16.4	71	290	10	MOLECULE:	DYNEIN REGU
21:00	6efn-A	2.1	9.9	62	384	8	MOLECULE:	SPORULATION
22:00	8bw9-A	2.1	3.6	54	88	4	MOLECULE:	PROTEIN AVE
23:00	1qoy-A	2.1	7	77	303	5	MOLECULE:	HEMOLYSIN E
24:00:00	1rso-B	2.1	3.1	40	56	3	MOLECULE:	PRESYNAPTIC
25:00:00	4k6j-A	2.1	15.8	85	497	12	MOLECULE:	WINGS APART
26:00:00	7jqq-A	2.1	12.4	59	327	5	MOLECULE:	DNA PACKAGI
27:00:00	5mpd-Z	2.1	12.5	82	906	9	MOLECULE:	26S PROTEAS
28:00:00	1nkl-A	2.1	6.8	42	78	5	MOLECULE:	NK-LYSIN
29:00:00	8jar-B	2.1	20	101	573	4	MOLECULE:	AMYLOID PRO
30:00:00	3mse-B	2.1	7	56	168	2	MOLECULE:	CALCIUM-DEP
31:00:00	7qru-E	2.1	7	58	158	14	MOLECULE:	NA+/H+ ANTI
32:00:00	7but-A	2.1	7.7	59	142	7	MOLECULE:	ACINIFORM S
33:00:00	4z7k-A	2.1	5	82	218	10	MOLECULE:	UNCHARACTER
34:00:00	3vej-B	2.1	2.7	37	41	3	MOLECULE:	UBIQUITIN-L
35:00:00	3jcm-I	2.1	21.6	92	416	7	MOLECULE:	PRE-MRNA-SP
36:00:00	1xnx-A	2.1	4.5	78	232	14	MOLECULE:	CONSTITUTIV
37:00:00	7p37-A	2.1	9.7	63	147	8	MOLECULE:	TRANSCRIPTI
38:00:00	7p2y-J	2.1	9.5	54	80	13	MOLECULE:	ATP SYNTHAS
39:00:00	6f0x-B	2.1	3.5	74	398	9	MOLECULE:	PACHYTENE C
40:00:00	8rz0-E	2.1	5.6	63	113	3	MOLECULE:	RH5-34EM
41:00:00	7eaw-A	2.1	15	83	560	10	MOLECULE:	TREHALASE
42:00:00	7ar9-d	2.1	5.7	51	80	4	MOLECULE:	ND3
43:00:00	7dkk-A	2.1	2.4	42	89	5	MOLECULE:	DE NOVO DES
44:00:00	8osp-A	2.1	6.9	62	174	6	MOLECULE:	N-ACETYLTRA
45:00:00	6k61-I	2.1	5.1	32	34	3	MOLECULE:	PHOTOSYSTEM
46:00:00	4gd3-A	2.1	12.9	67	179	4	MOLECULE:	HYDROGENASE
47:00:00	8ixk-E	2.1	21.7	65	109	5	MOLECULE:	ATTACHMENT
48:00:00	7yov-E	2.1	10.5	49	158	10	MOLECULE:	NDV P PROTE
49:00:00	3rh2-A	2.1	11.7	78	210	4	MOLECULE:	HYPOTHETICA
50:00:00	4z8u-A	2.1	13.6	112	332	4	MOLECULE:	AVRRXO1-ORF

51:00:00	7et0-C	2.1	16.9	113	684	3	MOLECULE:	BACTERIA FA
52:00:00	1xsx-A	2.1	3.6	59	95	8	MOLECULE:	SSO10A
53:00:00	1g4a-E	2.1	16	74	356	7	MOLECULE:	ATP-DEPENDE
54:00:00	7x5e-E	2.1	21.9	70	101	6	MOLECULE:	TRANSCRIPTI
55:00:00	5jk9-A	2.1	21.9	71	241	8	MOLECULE:	IZUMO SPERM
56:00:00	8dl7-A	2.1	6.2	82	467	7	MOLECULE:	SOLUTE CARR
57:00:00	6jho-A	2.1	7.8	74	200	8	MOLECULE:	CAG PATHOGE
58:00:00	2yml-A	2.1	7	107	228	9	MOLECULE:	L-HALOACID
59:00:00	5iuy-C	2.1	4.9	92	451	10	MOLECULE:	MULTIDRUG E
60:00:00	3eaf-A	2.1	4.9	52	384	4	MOLECULE:	ABC TRANSPO
61:00:00	6jcc-A	2.1	4.1	65	97	8	MOLECULE:	COMPUTATION
62:00:00	5hyc-A	2.1	4.5	79	130	4	MOLECULE:	UNCHARACTER
63:00:00	6poo-A	2.1	19.3	59	273	12	MOLECULE:	BIBA
64:00:00	1wa8-B	2.1	7.5	54	95	7	MOLECULE:	ESAT-6 LIKE
65:00:00	5ea1-B	2.1	3.8	49	128	2	MOLECULE:	TRANSCRIPTI
66:00:00	5dlq-B	2.1	29.1	101	1052	9	MOLECULE:	EXPORTIN-4
67:00:00	4gdf-A	2.1	9.2	98	497	6	MOLECULE:	LARGE T ANT
68:00:00	5j4i-A	2.1	10.5	103	437	6	MOLECULE:	ARGININE/AG
69:00:00	5y9o-A	2.1	23.8	79	401	5	MOLECULE:	WIPA
70:00:00	4eyw-B	2.1	11.8	104	634	9	MOLECULE:	CARNITINE O
71:00:00	6a5n-A	2.1	4.6	77	502	3	MOLECULE:	HISTONE-LYS
72:00:00	3boy-A	2.1	14.9	54	150	7	MOLECULE:	5'-R(*UP*UP
73:00:00	5mz6-1	2.1	16.4	109	1018	6	MOLECULE:	SEPARASE
74:00:00	3s63-B	2.1	7	44	90	5	MOLECULE:	SAPOSIN-LIK
75:00:00	6qld-Y	2.1	21.1	68	223	4	MOLECULE:	INNER KINET
76:00:00	4ocu-A	2.1	16.5	68	356	7	MOLECULE:	N-ACETYLHEX
77:00:00	7w1m-D	2.1	19.1	107	967	10	MOLECULE:	STRUCTURAL
78:00:00	7nyw-E	2.1	8.3	80	212	8	MOLECULE:	CHROMOSOME
79:00:00	5of3-C	2.1	6.6	49	107	6	MOLECULE:	DNA PRIMASE
80:00:00	6s6v-A	2.1	15.3	65	386	3	MOLECULE:	NUCLEASE SB
81:00:00	2mrl-A	2.1	3.9	44	72	2	MOLECULE:	UNCHARACTER
82:00:00	5ek8-A	2.1	7.4	103	668	7	MOLECULE:	LIPOXYGENAS
83:00:00	4hqo-B	2.1	24.6	83	260	11	MOLECULE:	SPOROZOITE
84:00:00	5uju-A	2.1	9.1	110	558	3	MOLECULE:	NAD-DEPENDE
85:00:00	5i6r-A	2.1	25.2	78	419	6	MOLECULE:	SLIT-ROBO R
86:00:00	8hm3-B	2.1	9.6	66	427	8	MOLECULE:	PUTATIVE UB
87:00:00	3mem-A	2.1	5.3	68	453	10	MOLECULE:	PUTATIVE SI
88:00:00	2z3x-A	2.1	4.3	48	56	10	MOLECULE:	SMALL, ACID
89:00:00	7b0p-A	2.1	6.8	52	224	8	MOLECULE:	HYPOTHETICA
90:00:00	3ck9-B	2.1	23.4	87	515	7	MOLECULE:	SUSD
91:00:00	7wo9-A	2.1	30.5	99	1581	6	MOLECULE:	NUCLEOPORIN
92:00:00	4nwp-D	2.1	10.6	54	149	9	MOLECULE:	PUTATIVE UN
93:00:00	7y88-A	2.1	9.8	60	354	2	MOLECULE:	PUTATIVE GL
94:00:00	4nsm-A	2.1	5.8	44	71	0	MOLECULE:	COLLAGEN-LI

95:00:00	1uyv-A	2.1	17.2	83	571	12	MOLECULE:	ACETYL-COA
96:00:00	7wkk-A	2.1	28.9	103	1684	6	MOLECULE:	MGC83295 PR
97:00:00	6wq2-a	2.1	7.5	73	202	15	MOLECULE:	A-DNA
98:00:00	6nr8-3	2.1	3.1	46	132	4	MOLECULE:	PREFOLDIN S
99:00:00	7rmy-A	2.1	14.5	67	266	7	MOLECULE:	DE NOVO DES
0:00	5cqq-B	2.1	3.2	53	75	6	MOLECULE:	REGULATORY
1:00	7p3x-A	2.1	13.9	74	576	11	MOLECULE:	AP-3 COMPLE
2:00	5c4y-A	2.1	3.2	55	136	11	MOLECULE:	PUTATIVE TR
3:00	8d0b-F	2.1	9.7	69	915	3	MOLECULE:	CST COMPLEX
4:00	4wd9-A	2.1	19.7	81	965	4	MOLECULE:	NISIN BIOSY
5:00	1nzb-A	2.1	17.4	76	332	9	MOLECULE:	LOXP DNA
6:00	6yxu-H	2.1	17.3	84	691	13	MOLECULE:	DNA-DIRECTE
7:00	8ga7-A	2.1	15.9	107	503	7	MOLECULE:	THR5
8:00	2iho-A	2.1	4	83	292	8	MOLECULE:	LECTIN
9:00	2grm-A	2.1	14.2	73	315	8	MOLECULE:	PRGX
10:00	1ku9-A	2.1	7.9	72	151	8	MOLECULE:	HYPOTHETICA
11:00	8h8e-D	2.1	4.5	41	293	7	MOLECULE:	PROTON-ACTI
12:00	1ung-D	2.1	4	69	149	9	MOLECULE:	CELL DIVISI
13:00	7rpk-A	2.1	18.9	123	953	11	MOLECULE:	PROTEIN DIS
14:00	2jps-A	2.1	4.6	60	105	12	MOLECULE:	NUCLEAR POL
15:00	4bbp-A	2.1	9.3	61	266	10	MOLECULE:	ZINC ABC TR
16:00	8cmk-A	2.1	28.5	118	913	13	MOLECULE:	TRANSPORTIN
17:00	4akk-A	2.1	16.7	76	368	7	MOLECULE:	NITRATE REG
18:00	4itr-A	2.1	16.3	78	299	5	MOLECULE:	ADENOSINE M
19:00	8f2r-C	2.1	24.6	78	195	8	MOLECULE:	COMM DOMAIN
20:00	8ibh-A	2.1	8.9	55	75	9	MOLECULE:	CENTROSOMAL
21:00	7ek2-A	2.1	16.5	95	341	7	MOLECULE:	BESTROPHIN-
22:00	5od9-A	2.1	6.5	52	95	8	MOLECULE:	MID1SC9
23:00	7yqh-E	2.1	16.3	88	527	6	MOLECULE:	STRUCTURAL
24:00:00	7jqd-B	2.1	3.5	35	38	3	MOLECULE:	PITUITARY A
25:00:00	8hqs-B	2.1	21.5	80	408	8	MOLECULE:	STRUCTURAL
26:00:00	6ahf-C	2.1	25.5	98	865	6	MOLECULE:	HEAT SHOCK
27:00:00	8f0x-A	2.1	10.2	90	928	3	MOLECULE:	IMPORTIN SU
28:00:00	6dft-A	2.1	6.5	113	384	3	MOLECULE:	DEOXYHYPUSI
29:00:00	7lhe-A	2.1	22.5	101	2222	8	MOLECULE:	INOSITOL 1,
30:00:00	6n7p-H	2.1	20.2	66	105	3	MOLECULE:	U1 SMALL NU
31:00:00	4kky-X	2.1	5.4	114	411	11	MOLECULE:	CAMPHOR 5-M
32:00:00	5nf8-A	2.1	13.8	73	158	4	MOLECULE:	RESPIRATORY
33:00:00	6f1t-f	2.1	35.4	124	929	5	MOLECULE:	ARP1 ACTIN
34:00:00	6jo5-K	2.1	2.9	39	45	10	MOLECULE:	PHOTOSYSTEM
35:00:00	6fht-B	2.1	22.9	113	735	12	MOLECULE:	BACTERIOPHY
36:00:00	1xkp-A	2.1	11.9	84	230	5	MOLECULE:	PUTATIVE ME
37:00:00	4rpu-A	2.1	10	116	988	12	MOLECULE:	PRESEQUENCE
38:00:00	2p5g-B	2.1	20.9	107	756	8	MOLECULE:	TEMPLATE DN

39:00:00	3pf6-A	2.1	3.9	44	60	11	MOLECULE:	HYPOTHETICA
40:00:00	7xcd-A	2.1	5.5	52	178	2	MOLECULE:	DISEASE SPE
41:00:00	2c5z-A	2.1	4.7	54	93	2	MOLECULE:	SET DOMAIN
42:00:00	6n7p-D	2.1	25.8	111	544	5	MOLECULE:	U1 SMALL NU
43:00:00	2xe4-A	2.1	13.9	60	721	5	MOLECULE:	OLIGOPEPTID
44:00:00	6k3b-B	2.1	4.2	67	106	9	MOLECULE:	LPG2147
45:00:00	4c2e-A	2.1	18.3	69	435	12	MOLECULE:	CARBOXY-TER
46:00:00	7dbe-B	2.1	4.6	64	332	6	MOLECULE:	BRANCHED-CH
47:00:00	6r6u-A	2.1	7.8	114	458	11	MOLECULE:	CIS-ACONITA
48:00:00	3t6p-A	2.1	15.1	62	330	8	MOLECULE:	BACULOVIRAL
49:00:00	7x8u-A	2.1	9.2	73	227	7	MOLECULE:	TOSPOVIRUS
50:00:00	8t1l-V	2.1	20.1	63	278	6	MOLECULE:	MEDIATOR OF
51:00:00	4qsz-A	2.1	17.9	94	686	2	MOLECULE:	MALTOSE-BIN
52:00:00	7t5p-A	2.1	15.6	82	403	6	MOLECULE:	SUMO-INTERA
53:00:00	5jfm-B	2.1	9.4	114	452	10	MOLECULE:	ALDEHYDE DE
54:00:00	5w60-A	2.1	10.7	55	179	5	MOLECULE:	APOPTOSIS R
55:00:00	8iaz-A	2.1	18	77	384	3	MOLECULE:	TRANSPOSASE
56:00:00	6j3i-A	2.1	4.4	84	583	12	MOLECULE:	GAMMA-GLUTA
57:00:00	1rw5-A	2.1	5.1	89	199	4	MOLECULE:	PROLACTIN
58:00:00	6x1g-A	2.1	6	65	216	12	MOLECULE:	ULP_PROTEAS
59:00:00	2b9z-A	2.1	4.1	44	74	7	MOLECULE:	B2 PROTEIN
60:00:00	2r9i-A	2.1	10.9	62	72	15	MOLECULE:	PUTATIVE PH
61:00:00	2zop-A	2.1	3.9	64	113	8	MOLECULE:	PUTATIVE UN
62:00:00	8ke8-A	2.1	11.7	60	201	3	MOLECULE:	NALC
63:00:00	2lck-A	2.1	8.4	105	296	5	MOLECULE:	MITOCHONDRI
64:00:00	6xf9-D	2.1	3.5	67	426	10	MOLECULE:	PACKAGING P
65:00:00	2o5h-A	2.1	4.6	67	128	12	MOLECULE:	HYPOTHETICA
66:00:00	7ecd-A	2.1	7	82	272	7	MOLECULE:	PHOSPHATIDA
67:00:00	5ool-v	2.1	7.5	50	69	2	MOLECULE:	16S RIBOSOM
68:00:00	8ui7-C	2.1	8.3	76	339	11	MOLECULE:	ATPASE FAMI
69:00:00	5an6-A	2.1	5.2	52	123	12	MOLECULE:	CRISPR-ASSO
70:00:00	6li2-A	2.1	8.5	103	347	4	MOLECULE:	CHIMERA OF
71:00:00	8e9h-J	2.1	24.2	74	231	9	MOLECULE:	TWO-COMPONE
72:00:00	4z7x-A	2.1	6.8	82	211	16	MOLECULE:	MDBA
73:00:00	4mi2-B	2.1	6.3	51	261	14	MOLECULE:	PUTATIVE EN
74:00:00	7z4w-a	2.1	5.5	67	102	6	MOLECULE:	PORTAL PROT
75:00:00	6w8p-B	2.1	9.3	108	426	5	MOLECULE:	ENDOSOMAL/L
76:00:00	7b4m-A	2.1	13.4	109	459	6	MOLECULE:	SODIUM/HYDR
77:00:00	1fou-A	2.1	8.5	75	257	5	MOLECULE:	UPPER COLLA
78:00:00	1d8b-A	2.1	3.6	63	81	2	MOLECULE:	SGS1 RECQ H
79:00:00	3cuz-A	2.1	7.4	66	529	6	MOLECULE:	MALATE SYNT
80:00:00	4ymt-B	2.1	4.1	69	216	6	MOLECULE:	ABC-TYPE PO
81:00:00	6iqc-A	2.1	6.4	70	103	11	MOLECULE:	DNA-BINDING
82:00:00	5ejq-A	2.1	17.9	89	501	7	MOLECULE:	MYOSIN-I HE

83:00:00	6n1c-A	2.1	6.4	65	176	2	MOLECULE:	INORGANIC P
84:00:00	2juc-A	2.1	3.2	49	55	2	MOLECULE:	PRE-MRNA-SP
85:00:00	7eeb-C	2.1	13.8	91	278	4	MOLECULE:	ENHANCED GR
86:00:00	2kn6-A	2.1	9.7	78	195	6	MOLECULE:	APOPTOSIS-A
87:00:00	7z6q-c	2.1	20.7	110	121	6	MOLECULE:	PHOTOSYSTEM
88:00:00	7fap-A	2.1	11.9	113	1236	9	MOLECULE:	ERYTHROCYTE
89:00:00	7ldk-B	2.1	4.3	72	106	10	MOLECULE:	NON-STRUCTU
90:00:00	4zzl-A	2.1	11.2	62	146	11	MOLECULE:	MULTIDRUG R
91:00:00	5m1m-A	2.1	9.3	53	154	6	MOLECULE:	MATRIX PROT
92:00:00	6yj4-V	2.1	12.4	97	126	3	MOLECULE:	NADH-UBIQUI
93:00:00	7qia-A	2.1	5	89	401	12	MOLECULE:	DIVALENT ME
94:00:00	2nvu-A	2.1	13.7	74	530	7	MOLECULE:	NEDD8-ACTIV
95:00:00	7dsq-B	2.1	13.8	96	465	6	MOLECULE:	4F2 CELL-SU
96:00:00	7y38-X	2.1	16.8	129	611	5	MOLECULE:	MRNA-CAPPIN
97:00:00	5cx6-B	2.1	18.4	139	665	7	MOLECULE:	RNA-DEPENDE
98:00:00	7f3x-A	2.1	18.4	76	449	3	MOLECULE:	LPCAT3
99:00:00	3taz-B	2.1	9.9	104	433	5	MOLECULE:	DNA DOUBLE-
0:00	7kfk-C	2.1	4.4	79	132	8	MOLECULE:	ISOFORM 2 O
1:00	8rc4-e	2.1	14.1	103	873	4	MOLECULE:	SERINE/THRE
2:00	2p06-B	2.1	5	72	88	4	MOLECULE:	HYPOTHETICA
3:00	8gi1-A	2.1	6.8	72	144	3	MOLECULE:	ACCESSORY P
4:00	8j8j-A	2.1	6.4	80	290	8	MOLECULE:	DECAPRENYL-
5:00	8bsh-C	2.1	23.4	109	794	6	MOLECULE:	PROTEIN TRA
6:00	5dzt-A	2.1	18.6	104	886	6	MOLECULE:	CYLM
7:00	3kd4-A	2.1	17	103	505	8	MOLECULE:	PUTATIVE PR
8:00	4gp2-A	2.1	8.4	89	342	7	MOLECULE:	POLYPRENYL
9:00	7k36-I	2.1	17.3	102	599	5	MOLECULE:	SERINE/THRE
10:00	6mpb-A	2.1	6.8	100	447	9	MOLECULE:	NEUTRAL AMI
11:00	4zht-A	2.1	8.3	77	384	10	MOLECULE:	BIFUNCTIONA
12:00	7lma-H	2.1	18.1	86	199	6	MOLECULE:	TELOMERASE
13:00	3zqg-A	2.1	12.4	73	208	10	MOLECULE:	TETRACYCLIN
14:00	7pc1-A	2.1	4.1	61	72	7	MOLECULE:	STBA
15:00	7roa-A	2.1	3.3	69	117	3	MOLECULE:	ENTV
16:00	8d8j-0	2.1	9.8	78	493	4	MOLECULE:	PROBABLE S-
17:00	2c9e-A	2.1	17.5	89	317	6	MOLECULE:	PERIDININ-C
18:00	3faj-A	2.1	7	70	101	4	MOLECULE:	PUTATIVE UN
19:00	5x5y-F	2.1	10.5	81	351	14	MOLECULE:	PROBABLE AT
20:00	6v3z-A	2.1	10.2	76	178	7	MOLECULE:	SEN1395
21:00	7qsd-N	2.1	8.4	93	347	5	MOLECULE:	NADH-UBIQUI
22:00	6vzz-A	2.1	5.4	85	374	9	MOLECULE:	BAMAA.19900
23:00	6jkg-A	2.1	7.2	79	216	4	MOLECULE:	STEROL-4-AL
24:00:00	7xpt-A	2.1	7.6	79	375	11	MOLECULE:	TRANSGLYCOS
25:00:00	2aja-B	2.1	18.2	83	345	4	MOLECULE:	ANKYRIN REP
26:00:00	8tfo-A	2.1	13.5	89	390	9	MOLECULE:	MEVALONATE

27:00:00	2dmn-A	2.1	5.9	72	83	4	MOLECULE:	HOMEOBOX PR
28:00:00	5xn7-B	2.1	9.3	103	551	7	MOLECULE:	PUTATIVE RT
29:00:00	5l7s-A	2.1	17	83	267	12	MOLECULE:	SECRETED RX
30:00:00	5n9j-U	2.1	11.1	94	198	5	MOLECULE:	MEDIATOR OF
31:00:00	5y7q-A	2.1	11.7	105	546	10	MOLECULE:	FANCONI-ASS
32:00:00	2m64-A	2.1	12.4	88	231	6	MOLECULE:	PHLP5
33:00:00	6t1z-A	2.1	15.6	82	393	5	MOLECULE:	LMRP INTEGR
34:00:00	2voh-A	2.1	5	81	155	10	MOLECULE:	BCL-2-RELAT
35:00:00	8sfj-A	2.1	19.1	116	1001	3	MOLECULE:	CRISPR-ASSO
36:00:00	4afi-A	2.1	7.9	76	155	11	MOLECULE:	AP-3 COMPLE
37:00:00	2apl-A	2.1	8	63	149	5	MOLECULE:	HYPOTHETICA
38:00:00	8fzb-A	2.1	22.1	87	319	11	MOLECULE:	PROTEIN-LYS
39:00:00	7w78-B	2.1	10.5	104	344	3	MOLECULE:	PUTATIVE AB
40:00:00	5y36-A	2.1	25.8	156	1368	6	MOLECULE:	CRISPR-ASSO
41:00:00	6jql-A	2.1	18.1	108	678	14	MOLECULE:	BIFUNCTIONA
42:00:00	6zfw-A	2.1	4.8	54	70	4	MOLECULE:	PEROXIN-14
43:00:00	3f75-P	2.1	5.3	60	75	13	MOLECULE:	CATHEPSIN L
44:00:00	2ccy-A	2.1	4.7	66	127	14	MOLECULE:	CYTOCHROME
45:00:00	7zkq-T	2.1	20	89	351	6	MOLECULE:	NADH DEHYDR
46:00:00	2vqg-B	2.1	4.2	65	77	9	MOLECULE:	UNCHARACTER
47:00:00	7t7r-A	2.1	18.8	118	1132	9	MOLECULE:	PROTEIN UNC
48:00:00	7y7q-A	2.1	13.1	108	951	6	MOLECULE:	RNA-DEPENDE
49:00:00	7mgq-A	2.1	16	80	602	3	MOLECULE:	5-AMINOIMID
50:00:00	3uit-D	2.1	12.3	85	257	6	MOLECULE:	INAD-LIKE P
51:00:00	2ly1-A	2.1	17.5	83	180	5	MOLECULE:	TUDOR DOMAI
52:00:00	5dcq-E	2.1	7.4	59	200	5	MOLECULE:	ARTIFICIAL
53:00:00	7w01-A	2.1	15.2	107	1598	8	MOLECULE:	PHOSPHOLIPI
54:00:00	7akx-A	2.1	8.8	91	224	7	MOLECULE:	VIRAL RHODO
55:00:00	8tlt-E	2.1	11.5	68	232	4	MOLECULE:	DNA POLYMER
56:00:00	6qum-N	2.1	17.3	113	649	4	MOLECULE:	V-TYPE ATP
57:00:00	3iee-A	2.1	10.4	79	256	4	MOLECULE:	PUTATIVE EX
58:00:00	3maj-A	2.1	6.7	57	353	9	MOLECULE:	DNA PROCESS
59:00:00	6j8l-A	2.1	3.6	72	132	13	MOLECULE:	AVH240
60:00:00	8ap7-o	2.1	11.2	78	96	6	MOLECULE:	ATP SYNTHAS
61:00:00	7rvb-A	2.1	16.5	97	209	2	MOLECULE:	FERRITIN
62:00:00	7wj0-A	2.1	9.1	42	49	12	MOLECULE:	ANTITOXIN M
63:00:00	8et6-A	2.1	18.9	115	532	10	MOLECULE:	1-Oct
64:00:00	7bu0-A	2.1	5	77	407	10	MOLECULE:	UNCHARACTER
65:00:00	8tcw-A	2.1	8.4	77	282	5	MOLECULE:	PYRROLINE-5
66:00:00	7f4l-B	2.1	5.5	75	130	11	MOLECULE:	TRANSMEMBRA
67:00:00	1jud-A	2.1	6.1	102	220	9	MOLECULE:	L-2-HALOACI
68:00:00	3p8c-F	2.1	28.3	99	156	4	MOLECULE:	CYTOPLASMIC
69:00:00	5wat-A	2.1	6	76	310	8	MOLECULE:	HOMOSERINE
70:00:00	5usf-A	2.1	23.2	96	683	9	MOLECULE:	TYROSYL-TRN

71:00:00	4k2u-A	2.1	8.4	65	230	6	MOLECULE:	ERYTHROCYTE
72:00:00	4elj-A	2.1	8.5	86	590	6	MOLECULE:	RETINOBLAST
73:00:00	1ntc-A	2.1	7.1	61	91	8	MOLECULE:	PROTEIN (NI
74:00:00	5tk6-A	2.1	5.8	70	191	13	MOLECULE:	OXSA PROTEI
75:00:00	8t1l-S	2.1	12.1	111	913	5	MOLECULE:	MEDIATOR OF
76:00:00	6wkr-A	2.1	8.8	66	439	8	MOLECULE:	UBIQUITIN
77:00:00	1n81-A	2.1	11	72	186	4	MOLECULE:	PLASMODIUM
78:00:00	6wge-C	2.1	17.4	78	174	9	MOLECULE:	STRUCTURAL
79:00:00	6zie-A	2.1	5	66	123	8	MOLECULE:	CMPX-383B
80:00:00	8izd-A	2.1	12.5	95	315	6	MOLECULE:	CERAMIDE SY
81:00:00	7aft-F	2.1	11	76	190	4	MOLECULE:	PROTEIN TRA
82:00:00	4acl-A	2.1	8.2	73	155	7	MOLECULE:	TSSL
83:00:00	5uld-A	2.1	5.5	83	445	5	MOLECULE:	TRANSPORTER
84:00:00	6tdy-M	2.1	7.6	93	243	13	MOLECULE:	ATP SYNTHAS
85:00:00	8bhw-A	2.1	2.9	58	332	3	MOLECULE:	ECA POLYSAC
86:00:00	8ck1-E	2.1	4.6	71	206	4	MOLECULE:	TAIL NOZZLE
87:00:00	8xgr-R	2.1	15.2	101	305	5	MOLECULE:	GUANINE NUC
88:00:00	7pw9-A	2.1	19.6	113	1952	10	MOLECULE:	SERINE/THRE
89:00:00	6uzt-A	2.1	25.2	113	587	6	MOLECULE:	RECEPTOR-TY
90:00:00	8hgu-A	2.1	4.6	98	295	10	MOLECULE:	ALPHA/BETA
91:00:00	6icz-A	2.1	20.3	134	2253	10	MOLECULE:	PROTEIN MAG
92:00:00	5lpc-A	2.1	4.7	76	624	7	MOLECULE:	VANADIUM-DE
93:00:00	6xp5-H	2.1	11.4	87	146	5	MOLECULE:	MEDIATOR OF
94:00:00	6m31-A	2.1	14.2	95	280	5	MOLECULE:	DIGERANYLGE
95:00:00	3no4-A	2.1	11	77	264	12	MOLECULE:	CREATININE
96:00:00	3fgg-A	2.1	8.1	70	147	4	MOLECULE:	UNCHARACTER
97:00:00	3wfr-H	2.1	20	109	463	3	MOLECULE:	RNA (74-MER
98:00:00	6rfl-C	2.1	3.7	71	304	11	MOLECULE:	DNA-DEPENDE
99:00:00	6l1k-A	2.1	8.3	68	384	12	MOLECULE:	NADH-DEPEND
0:00	6vk0-C	2.1	6.7	93	180	4	MOLECULE:	U1 SNP1-ASS
1:00	6iu4-A	2.1	5.7	77	225	5	MOLECULE:	VIT1
2:00	8dgc-E	2.1	20.5	98	538	9	MOLECULE:	SEAVS3
3:00	5jb3-Q	2.1	9.1	60	158	8	MOLECULE:	16S RIBOSOM
4:00	6ofs-A	2.1	8.5	73	905	8	MOLECULE:	PROBABLE ZI
5:00	8qrk-R	2.1	12.8	82	295	4	MOLECULE:	28S RIBOSOM
6:00	1x3w-B	2.1	3.5	49	57	12	MOLECULE:	PEPTIDE:N-G
7:00	8osg-B	2.1	7.7	75	323	8	MOLECULE:	MAGNESIUM-C
8:00	7k5c-B	2.1	17.5	83	228	2	MOLECULE:	INTERNAL VI
9:00	5wue-A	2.1	4.8	71	203	4	MOLECULE:	UNCHARACTER
10:00	2c5u-A	2.1	14.3	77	375	4	MOLECULE:	RNA LIGASE
11:00	8jnt-A	2.1	3.5	57	77	5	MOLECULE:	RXLR19781
12:00	7z8v-D	2.1	24.9	124	1158	7	MOLECULE:	CULLIN-1
13:00	7jpp-E	2	27.1	89	406	7	MOLECULE:	ORIGIN RECO
14:00	8ic6-B	2	22.1	99	855	9	MOLECULE:	EXO-BETA-D-

15:00	2lon-A	2	15.2	74	99	5	MOLECULE:	HIG1 DOMAIN
16:00	7lxt-J	2	3.8	63	215	14	MOLECULE:	20S PROTEAS
17:00	1gk7-A	2	3.3	39	39	8	MOLECULE:	VIMENTIN
18:00	6ugm-X	2	10.1	58	73	12	MOLECULE:	HISTONE H3
19:00	2a01-A	2	13.3	74	243	7	MOLECULE:	APOLIPOPROT
20:00	3cs3-A	2	8.1	68	266	4	MOLECULE:	SUGAR-BINDI
21:00	6mag-A	2	10.4	82	282	13	MOLECULE:	BBVCI ENDON
22:00	5wvc-B	2	3.9	57	103	7	MOLECULE:	APOPTOTIC P
23:00	2bdt-A	2	3.6	48	172	13	MOLECULE:	BH3686
24:00:00	3a1y-A	2	2.6	44	58	9	MOLECULE:	50S RIBOSOM
25:00:00	5y6r-A	2	15.4	116	667	9	MOLECULE:	GENOME POLY
26:00:00	6jta-A	2	17.5	81	1299	4	MOLECULE:	PHOSPHORIBO
27:00:00	8jhu-A	2	20.2	99	811	4	MOLECULE:	LEGIONELLA
28:00:00	1zvr-A	2	7.6	85	514	6	MOLECULE:	MYOTUBULARI
29:00:00	7twd-B	2	3.2	41	45	15	MOLECULE:	ALPHA- AND
30:00:00	6cnb-R	2	17.7	107	522	4	MOLECULE:	DNA-DIRECTE
31:00:00	8hr5-A	2	28.2	94	466	9	MOLECULE:	TRANSPOSASE
32:00:00	1u4q-A	2	38	122	318	8	MOLECULE:	SPECTRIN AL
33:00:00	6wut-C	2	18.5	75	348	5	MOLECULE:	SAM35
34:00:00	8sre-A	2	25.9	103	1377	6	MOLECULE:	TRPM2 CHANZ
35:00:00	8q3v-e	2	10.1	109	294	6	MOLECULE:	TETRAHYDROM
36:00:00	1u2m-C	2	12.4	57	143	14	MOLECULE:	HISTONE-LIK
37:00:00	7px2-C	2	3	50	85	6	MOLECULE:	CONOTOXIN M
38:00:00	4rs7-B	2	5.1	58	88	5	MOLECULE:	PARB-C
39:00:00	4imj-A	2	11.6	87	333	6	MOLECULE:	SYMPLEKIN
40:00:00	8h5s-A	2	4.6	83	247	6	MOLECULE:	BETA-PHOSPH
41:00:00	1lsh-A	2	22	98	955	3	MOLECULE:	LIPOVITELLI
42:00:00	7orl-A	2	12.4	121	2183	7	MOLECULE:	RNA (5'-D(*
43:00:00	1hrk-A	2	16.2	73	359	1	MOLECULE:	FERROCHELAT
44:00:00	7dsj-A	2	6	56	363	11	MOLECULE:	ANTHRANILAT
45:00:00	6jxc-A	2	22.2	100	2345	7	MOLECULE:	SERINE/THRE
46:00:00	8dwj-A	2	7	64	339	8	MOLECULE:	RNA(5'-PPP-
47:00:00	6bzf-A	2	19.1	62	197	5	MOLECULE:	SPORULATION
48:00:00	6p7r-A	2	6	82	277	5	MOLECULE:	TOXIN CO-RE
49:00:00	5j08-A	2	9	71	163	13	MOLECULE:	EPSIN-5
50:00:00	7msw-A	2	17.3	87	635	6	MOLECULE:	NON-STRUCTU
51:00:00	7qe7-O	2	32.7	81	707	9	MOLECULE:	ANAPHASE-PR
52:00:00	6fes-A	2	13.9	84	365	11	MOLECULE:	D12_BRIC2,
53:00:00	5erg-A	2	8.5	69	340	6	MOLECULE:	TRNA (ADENI
54:00:00	5m48-A	2	20.1	73	114	3	MOLECULE:	REGULATOR O
55:00:00	6lo8-E	2	6	54	80	11	MOLECULE:	MITOCHONDRI
56:00:00	2o7g-A	2	3.4	55	88	5	MOLECULE:	PROBABLE RN
57:00:00	8bgo-C	2	9.2	64	271	8	MOLECULE:	DIACETYLCHI
58:00:00	4v1a-a	2	20.1	92	393	7	MOLECULE:	MITORIBOSOM

59:00:00	8kde-G	2	8.8	62	145	10	MOLECULE:	PHOTOSYSTEM
60:00:00	7r07-F	2	7.7	108	600	7	MOLECULE:	ABIK
61:00:00	6l1q-A	2	14.1	70	267	7	MOLECULE:	CBBQ PROTEI
62:00:00	4pas-B	2	4.7	39	39	3	MOLECULE:	GAMMA-AMINO
63:00:00	3wrn-A	2	3.9	92	248	5	MOLECULE:	NON-CAPSID
64:00:00	1n1c-A	2	14.4	92	199	8	MOLECULE:	TORA SPECIF
65:00:00	8hpo-C	2	9.6	60	138	2	MOLECULE:	TRANSCRIPTI
66:00:00	3on2-B	2	4.7	68	195	6	MOLECULE:	PROBABLE TR
67:00:00	6vej-A	2	28.6	138	1022	3	MOLECULE:	PROBABLE RE
68:00:00	1zhc-A	2	8.3	51	76	8	MOLECULE:	HYPOTHETICA
69:00:00	3dxj-D	2	19.2	75	1504	3	MOLECULE:	DNA-DIRECTE
70:00:00	3n2o-A	2	6.3	76	630	1	MOLECULE:	BIOSYNTHETI
71:00:00	1na6-B	2	17.7	102	395	4	MOLECULE:	RESTRICTION
72:00:00	2jdi-H	2	8.4	43	88	14	MOLECULE:	ATP SYNTHAS
73:00:00	3r6n-A	2	37	112	450	6	MOLECULE:	DESMOPLAKIN
74:00:00	7pkq-N	2	9.7	51	195	2	MOLECULE:	MS35
75:00:00	1oks-A	2	3.3	37	53	8	MOLECULE:	RNA POLYMER
76:00:00	8c1s-D	2	10.9	61	155	7	MOLECULE:	GLUTAMATE R
77:00:00	3cwz-B	2	16.1	59	310	5	MOLECULE:	RAS-RELATED
78:00:00	2xus-A	2	3.3	41	41	10	MOLECULE:	BREAST CANC
79:00:00	6c8c-A	2	5.1	71	119	8	MOLECULE:	CHIMERIC PR
80:00:00	3otv-C	2	15.6	63	257	8	MOLECULE:	PROBABLE CO
81:00:00	8ozh-D	2	21.5	84	405	7	MOLECULE:	ENVELOPE GL
82:00:00	5g49-A	2	6.4	52	97	6	MOLECULE:	NUCLEAR TRA
83:00:00	1nxh-A	2	9	66	124	8	MOLECULE:	MTH396 PROT
84:00:00	6its-A	2	6	56	147	4	MOLECULE:	METHYL-ACCE
85:00:00	2n5n-A	2	7.4	48	86	4	MOLECULE:	CHROMODOMAI
86:00:00	5jbr-A	2	5.4	56	149	5	MOLECULE:	UNCHARACTER
87:00:00	6u1s-A	2	13.8	73	310	7	MOLECULE:	DE NOVO DES
88:00:00	5a9e-A	2	15.5	91	254	11	MOLECULE:	DELTAMBD GA
89:00:00	1f3m-A	2	3.5	43	70	0	MOLECULE:	SERINE/THRE
90:00:00	4xig-N	2	10.3	79	290	6	MOLECULE:	ALGM1
91:00:00	5f0n-A	2	13.3	125	1038	5	MOLECULE:	COHESIN SUB
92:00:00	6vwb-A	2	4.5	52	90	15	MOLECULE:	CROSSOVER J
93:00:00	7lxk-A	2	3.6	47	59	4	MOLECULE:	ALLERGEN AR
94:00:00	4dci-B	2	25.1	80	148	11	MOLECULE:	UNCHARACTER
95:00:00	6sny-A	2	4.7	61	107	2	MOLECULE:	SYNTHETIC E
96:00:00	5l1a-A	2	6.6	48	108	8	MOLECULE:	UNCHARACTER
97:00:00	8a51-B	2	3.6	39	39	8	MOLECULE:	HEAT SHOCK
98:00:00	4q2d-A	2	5.2	128	927	4	MOLECULE:	CRISPR-ASSO
99:00:00	5ncn-B	2	7.5	60	76	7	MOLECULE:	DBF2 KINASE
0:00	3ezh-A	2	8.7	57	114	7	MOLECULE:	NITRATE/NIT
1:00	5b6b-G	2	11	64	74	2	MOLECULE:	MOB KINASE
2:00	7yh8-A	2	6.9	43	62	2	MOLECULE:	L-19437

3:00	7ktr-A	2	22	90	3042	6	MOLECULE:	TRANSFORMAT
4:00	6wi5-A	2	4.1	53	90	8	MOLECULE:	DE NOVO DES
5:00	6s18-A	2	11.8	84	143	7	MOLECULE:	AROMATIC AC
6:00	1jwy-A	2	18.3	117	1039	9	MOLECULE:	MYOSIN-2 HE
7:00	6o3c-A	2	8.8	81	484	9	MOLECULE:	SMOOTHENED
8:00	6pqx-A	2	8.7	82	480	4	MOLECULE:	DNA-MEDIATE
9:00	1tuz-A	2	4.6	73	118	4	MOLECULE:	DIACYLGLYCE
10:00	4l6v-6	2	6.9	71	125	3	MOLECULE:	PHOTOSYSTEM
11:00	6lu0-A	2	14.2	89	937	3	MOLECULE:	CAS12I2
12:00	1puo-B	2	7.3	54	142	2	MOLECULE:	MAJOR ALLER
13:00	6zyv-A	2	14.8	77	238	4	MOLECULE:	CIR PROTEIN
14:00	8iue-P	2	18.3	73	303	5	MOLECULE:	DNA-DIRECTE
15:00	6ei6-A	2	4.2	59	242	8	MOLECULE:	COILED-COIL
16:00	5img-A	2	14.9	75	467	4	MOLECULE:	DIPEPTIDASE
17:00	2guz-B	2	5.4	53	65	8	MOLECULE:	MITOCHONDRI
18:00	8bpd-A	2	11.7	75	370	8	MOLECULE:	GLYCOSYL HY
19:00	8irl-A	2	19.2	106	540	6	MOLECULE:	ADENINE/GUA
20:00	4mi8-B	2	6.7	71	136	7	MOLECULE:	BCL-2 HOMOL
21:00	2jes-A	2	4.8	55	370	7	MOLECULE:	PORTAL PROT
22:00	4dra-H	2	2.7	49	76	14	MOLECULE:	CENTROMERE
23:00	2oyy-A	2	3.4	57	71	4	MOLECULE:	HEXAMERIC C
24:00:00	4gwp-C	2	8.8	83	184	6	MOLECULE:	MEDIATOR OF
25:00:00	3f41-A	2	19.4	95	590	11	MOLECULE:	PHYTASE
26:00:00	2mb9-A	2	4.3	68	106	10	MOLECULE:	B-CELL LYMP
27:00:00	3sqn-A	2	17.2	117	468	6	MOLECULE:	CONSERVED D
28:00:00	6kkk-A	2	14.3	95	380	4	MOLECULE:	SUGAR EFFLU
29:00:00	3h20-A	2	9.9	68	288	13	MOLECULE:	REPLICATION
30:00:00	5yfp-H	2	10.2	66	518	8	MOLECULE:	EXOCYST COM
31:00:00	5okc-B	2	5.9	99	377	5	MOLECULE:	SISTER CHRO
32:00:00	4nk2-A	2	5.7	81	164	7	MOLECULE:	HEMOGLOBIN-
33:00:00	7ura-A	2	16.6	98	432	6	MOLECULE:	ISOFORM 2 O
34:00:00	5k2a-A	2	11.6	73	398	4	MOLECULE:	ADENOSINE R
35:00:00	7wb4-E	2	10.3	120	1363	7	MOLECULE:	OUTER NUP13
36:00:00	2dla-A	2	5.2	83	222	7	MOLECULE:	397AA LONG
37:00:00	8eg3-A	2	27.6	94	554	6	MOLECULE:	STERYL-SULF
38:00:00	7n1j-B	2	4.6	59	64	5	MOLECULE:	FIBROBLAST
39:00:00	5y77-A	2	7.8	72	456	4	MOLECULE:	KYNURENINE
40:00:00	5mnt-B	2	26.8	86	421	5	MOLECULE:	A2 MATURATI
41:00:00	7uqi-G	2	13.3	83	197	4	MOLECULE:	ATPASE HIST
42:00:00	6l7r-A	2	15	59	105	2	MOLECULE:	PUTATIVE SP
43:00:00	6lyy-A	2	16.3	94	382	6	MOLECULE:	MONOCARBOXY
44:00:00	3icq-T	2	13.8	98	949	7	MOLECULE:	EXPORTIN-T
45:00:00	6p5a-B	2	4.7	74	118	4	MOLECULE:	TRANSPOSABL
46:00:00	7cww-A	2	10.2	77	350	9	MOLECULE:	TSRE

47:00:00	8exh-a	2	11.4	63	69	5	MOLECULE:	PROTEIN VIR
48:00:00	6xtt-B	2	2.9	59	116	2	MOLECULE:	NTTA
49:00:00	6exn-O	2	18.8	88	320	6	MOLECULE:	U2 SNRNA
50:00:00	1yoz-B	2	6.1	61	116	5	MOLECULE:	HYPOTHETICA
51:00:00	8jhq-A	2	14.7	98	446	4	MOLECULE:	SPHINGOSINE
52:00:00	6f2d-G	2	7.6	65	89	8	MOLECULE:	FLAGELLAR B
53:00:00	3hc7-A	2	15.8	67	252	9	MOLECULE:	GENE 12 PRO
54:00:00	6drh-E	2	5.1	79	366	6	MOLECULE:	ADP-RIBOSYL
55:00:00	8b4h-C	2	19.9	86	362	8	MOLECULE:	PUTATIVE TR
56:00:00	7xno-B	2	5.9	62	106	10	MOLECULE:	BACTERIOCIN
57:00:00	5w6l-A	2	20.7	99	494	3	MOLECULE:	RTX REPEAT-
58:00:00	5t9f-B	2	9.7	75	254	5	MOLECULE:	PREPHENATE
59:00:00	6rj9-C	2	25	86	836	7	MOLECULE:	ACRIIA6
60:00:00	7f47-A	2	6.7	117	830	7	MOLECULE:	HYPOTHETICA
61:00:00	7dve-B	2	3.7	71	299	6	MOLECULE:	6'''-HYDROX
62:00:00	8e5t-6	2	18.9	80	514	8	MOLECULE:	RIBOSOME BI
63:00:00	7vf2-A	2	20	108	1196	7	MOLECULE:	PROTEIN VIR
64:00:00	8e9b-G	2	9.1	83	151	6	MOLECULE:	ACTIN-RELAT
65:00:00	4u7u-B	2	5.9	64	157	8	MOLECULE:	CRISPR SYST
66:00:00	6p4o-F	2	17.1	82	481	10	MOLECULE:	DNA-DEPENDE
67:00:00	7d60-B	2	13.9	103	288	6	MOLECULE:	CALCIUM HOM
68:00:00	4y7k-A	2	23.6	90	250	4	MOLECULE:	LARGE CONDU
69:00:00	7jhj-R	2	10.6	79	277	5	MOLECULE:	GUANINE NUC
70:00:00	8h7g-C	2	24.7	100	3261	6	MOLECULE:	TRANSFORMAT
71:00:00	4g33-A	2	6.9	86	661	12	MOLECULE:	15S-LIPOXYG
72:00:00	7y6b-A	2	7.1	56	72	2	MOLECULE:	ESCE/YSCE/S
73:00:00	1g4u-S	2	12.1	73	360	11	MOLECULE:	PROTEIN TYR
74:00:00	5tj5-A	2	15.2	80	570	8	MOLECULE:	V-TYPE PROT
75:00:00	7ctq-A	2	9.9	93	429	10	MOLECULE:	PEPTIDYL TR
76:00:00	2nnu-A	2	8.1	88	201	13	MOLECULE:	REGULATORY
77:00:00	3ksy-A	2	21.8	102	1007	3	MOLECULE:	SON OF SEVE
78:00:00	8eki-D	2	12.6	96	684	8	MOLECULE:	PROTEIN TRA
79:00:00	8k4a-H	2	4.4	74	301	8	MOLECULE:	VP2
80:00:00	8s4g-N	2	31	138	682	7	MOLECULE:	ANAPHASE-PR

Query : s001 A

DALI results: MdA-1 Penton

No:	Chain	Z	rmsd	lali	nres	%id	PDB Description	
1:00	6z7q-E	12	3.8	224	416	13	MOLECULE:	PENTON PROTEIN
2:00	6z7q-B	11.9	3.9	225	416	13	MOLECULE:	PENTON PROTEIN
3:00	2c6s-C	11.8	3.9	224	443	14	MOLECULE:	ADENOVIRUS 2,12
4:00	2c6s-I	11.8	3.8	223	443	13	MOLECULE:	ADENOVIRUS 2,12
5:00	2c6s-M	11.8	3.9	224	443	14	MOLECULE:	ADENOVIRUS 2,12
6:00	2c6s-L	11.8	3.8	223	443	13	MOLECULE:	ADENOVIRUS 2,12
7:00	6z7q-D	11.8	3.9	225	416	13	MOLECULE:	PENTON PROTEIN
8:00	6z7q-A	11.8	3.9	225	416	13	MOLECULE:	PENTON PROTEIN
9:00	2c6s-G	11.8	3.8	223	443	14	MOLECULE:	ADENOVIRUS 2,12
10:00	6z7q-C	11.8	3.9	224	416	13	MOLECULE:	PENTON PROTEIN
11:00	2c6s-H	11.8	3.9	224	443	14	MOLECULE:	ADENOVIRUS 2,12
12:00	2c6s-O	11.8	3.8	224	443	13	MOLECULE:	ADENOVIRUS 2,12
13:00	2c6s-E	11.8	3.9	224	443	14	MOLECULE:	ADENOVIRUS 2,12
14:00	2c6s-D	11.8	3.8	223	443	13	MOLECULE:	ADENOVIRUS 2,12
15:00	2c6s-F	11.8	3.8	223	443	13	MOLECULE:	ADENOVIRUS 2,12
16:00	1x9p-A	11.7	3.9	226	460	14	MOLECULE:	PENTON PROTEIN
17:00	2c6s-K	11.7	3.8	223	443	13	MOLECULE:	ADENOVIRUS 2,12
18:00	2c6s-A	11.7	3.8	223	443	14	MOLECULE:	ADENOVIRUS 2,12
19:00	2c6s-J	11.7	3.8	223	443	13	MOLECULE:	ADENOVIRUS 2,12
20:00	2c9g-B	11.7	3.9	226	447	14	MOLECULE:	PENTON PROTEIN
21:00	2c6s-B	11.7	3.8	224	443	14	MOLECULE:	ADENOVIRUS 2,12
22:00	2c6s-N	11.7	3.8	223	443	13	MOLECULE:	ADENOVIRUS 2,12
23:00	2c9g-C	11.7	3.9	222	447	14	MOLECULE:	PENTON PROTEIN
24:00:00	2c9g-E	11.7	3.9	225	447	14	MOLECULE:	PENTON PROTEIN
25:00:00	2c9g-D	11.7	3.9	222	447	14	MOLECULE:	PENTON PROTEIN
26:00:00	2c9g-A	11.7	4	225	447	15	MOLECULE:	PENTON PROTEIN
27:00:00	1x9t-A	11.6	3.9	222	440	14	MOLECULE:	PENTON PROTEIN
28:00:00	2c9f-A	11.6	3.9	222	440	14	MOLECULE:	PENTON PROTEIN
29:00:00	2c9f-B	11.6	3.9	222	440	13	MOLECULE:	PENTON PROTEIN
30:00:00	2c9f-D	11.6	3.9	222	440	14	MOLECULE:	PENTON PROTEIN
31:00:00	2c9f-C	11.6	3.9	222	440	14	MOLECULE:	PENTON PROTEIN
32:00:00	2c9f-E	11.6	3.9	222	440	14	MOLECULE:	PENTON PROTEIN
33:00:00	4ar2-A	11.4	3.9	226	455	13	MOLECULE:	L2 PROTEIN III
34:00:00	3izo-D	11.4	3.9	227	455	13	MOLECULE:	PENTON PROTEIN
35:00:00	3izo-C	11.4	3.9	226	455	13	MOLECULE:	PENTON PROTEIN
36:00:00	3izo-A	11.4	3.9	227	455	13	MOLECULE:	PENTON PROTEIN
37:00:00	3izo-E	11.4	3.9	226	455	13	MOLECULE:	PENTON PROTEIN
38:00:00	3izo-B	11.4	3.9	227	455	13	MOLECULE:	PENTON PROTEIN
39:00:00	4aqq-A	11.3	3.9	226	462	13	MOLECULE:	L2 PROTEIN III
40:00:00	6g41-F	11.2	3.3	159	301	6	MOLECULE:	MINOR CAPSID PR
41:00:00	6g41-I	11	3.3	154	301	6	MOLECULE:	MINOR CAPSID PR

42:00:00	6g42-D	11	3.3	155	302	6	MOLECULE:	MINOR CAPSID PR
43:00:00	6g42-E	10.9	3.2	158	301	6	MOLECULE:	MINOR CAPSID PR
44:00:00	6g41-J	10.8	3.1	153	301	6	MOLECULE:	MINOR CAPSID PR
45:00:00	6g41-C	10.8	3.2	156	301	6	MOLECULE:	MINOR CAPSID PR
46:00:00	6g42-A	10.8	3.4	161	301	6	MOLECULE:	MINOR CAPSID PR
47:00:00	6g42-C	10.8	3.3	159	302	6	MOLECULE:	MINOR CAPSID PR
48:00:00	6g42-B	10.8	3.6	162	301	6	MOLECULE:	MINOR CAPSID PR
49:00:00	6g41-A	10.8	3.1	155	297	6	MOLECULE:	MINOR CAPSID PR
50:00:00	6g41-E	10.7	3	150	301	6	MOLECULE:	MINOR CAPSID PR
51:00:00	6g41-B	10.7	3.2	154	295	6	MOLECULE:	MINOR CAPSID PR
52:00:00	6g41-H	10.7	3.5	162	301	6	MOLECULE:	MINOR CAPSID PR
53:00:00	6g41-D	10.7	3.2	155	291	6	MOLECULE:	MINOR CAPSID PR
54:00:00	8rbt-S	9.3	3.3	209	663	11	MOLECULE:	MAJOR CAPSID PR
55:00:00	8coi-I	8.9	2.4	99	249	11	MOLECULE:	ADDOBODY
56:00:00	8coi-A	8.4	2.3	95	245	11	MOLECULE:	ADDOBODY
57:00:00	8coi-J	8.4	2.4	94	243	11	MOLECULE:	ADDOBODY
58:00:00	8coi-Q	8.4	2.4	96	232	11	MOLECULE:	ADDOBODY
59:00:00	8coi-P	8.4	2.3	95	243	12	MOLECULE:	ADDOBODY
60:00:00	3j26-N	8.2	3.8	166	362	7	MOLECULE:	CAPSID PROTEIN
61:00:00	8coi-C	8.2	2.5	99	249	11	MOLECULE:	ADDOBODY
62:00:00	8coi-E	8.2	2.3	94	228	11	MOLECULE:	ADDOBODY
63:00:00	6g41-G	8	3.3	156	287	6	MOLECULE:	MINOR CAPSID PR
64:00:00	8coi-N	7.9	2.3	92	232	12	MOLECULE:	ADDOBODY
65:00:00	8coi-H	7.7	2.3	96	243	11	MOLECULE:	ADDOBODY
66:00:00	8coi-F	7.6	2.3	98	240	11	MOLECULE:	ADDOBODY
67:00:00	8coi-R	7.1	2.3	93	227	11	MOLECULE:	ADDOBODY
68:00:00	8coi-D	7.1	2.3	96	244	11	MOLECULE:	ADDOBODY
69:00:00	8coi-G	7	2.3	96	243	11	MOLECULE:	ADDOBODY
70:00:00	8coi-M	7	2.4	96	237	11	MOLECULE:	ADDOBODY
71:00:00	8coi-L	7	2.4	95	244	12	MOLECULE:	ADDOBODY
72:00:00	8qb3-E	6.9	2.7	89	233	10	MOLECULE:	ADDOBODY
73:00:00	8coi-S	6.8	2.3	94	230	11	MOLECULE:	ADDOBODY
74:00:00	8coi-B	6.7	2.3	93	246	12	MOLECULE:	ADDOBODY
75:00:00	8coi-O	6.7	2.4	92	229	10	MOLECULE:	ADDOBODY
76:00:00	8coi-K	6.6	2.3	92	250	12	MOLECULE:	ADDOBODY
77:00:00	8coi-T	6.6	2.4	84	212	11	MOLECULE:	ADDOBODY
78:00:00	7cz6-C	6.2	3.1	99	126	8	MOLECULE:	CAPSID PROTEIN
79:00:00	6ds5-E	6.2	3.8	107	160	13	MOLECULE:	SEIPIN
80:00:00	6ds5-K	6.2	3.8	107	160	13	MOLECULE:	SEIPIN
81:00:00	6ds5-B	6.2	3.8	107	160	13	MOLECULE:	SEIPIN
82:00:00	6ds5-J	6.2	3.8	107	160	13	MOLECULE:	SEIPIN
83:00:00	6ds5-D	6.2	3.8	107	160	13	MOLECULE:	SEIPIN
84:00:00	6ds5-C	6.2	3.8	107	160	13	MOLECULE:	SEIPIN
85:00:00	6ds5-G	6.1	3.6	104	160	13	MOLECULE:	SEIPIN

86:00:00	6ds5-H	6.1	3.8	105	160	13	MOLECULE:	SEIPIN
87:00:00	6wpc-C	6	3.4	113	588	9	MOLECULE:	CRY1A.2
88:00:00	6ds5-A	6	3.6	105	160	12	MOLECULE:	SEIPIN
89:00:00	6ds5-F	6	3.7	104	160	13	MOLECULE:	SEIPIN
90:00:00	6ds5-I	6	3.7	104	160	13	MOLECULE:	SEIPIN
91:00:00	6wpc-B	5.9	3.4	109	588	11	MOLECULE:	CRY1A.2
92:00:00	6wpc-A	5.9	3.2	114	588	11	MOLECULE:	CRY1A.2
93:00:00	8qb3-C	5.9	2.6	89	216	10	MOLECULE:	ADDOBODY
94:00:00	6owk-A	5.9	3.2	116	582	6	MOLECULE:	PESTICIDAL CRYS
95:00:00	7rsl-B	5.7	3.3	108	235	11	MOLECULE:	SEIPIN
96:00:00	7rsl-D	5.7	3.3	108	235	11	MOLECULE:	SEIPIN
97:00:00	7rsl-F	5.7	3.4	109	235	11	MOLECULE:	SEIPIN
98:00:00	7rsl-H	5.7	3.2	107	235	11	MOLECULE:	SEIPIN
99:00:00	1ciy-A	5.7	3.9	115	577	8	MOLECULE:	CRYIA(A)
0:00	7rsl-J	5.7	3.3	108	235	11	MOLECULE:	SEIPIN
1:00	9buv-A	5.6	3.9	110	573	7	MOLECULE:	PESTICIDAL CRYS
2:00	8qb3-D	5.6	2.5	84	206	11	MOLECULE:	ADDOBODY
3:00	7rsl-E	5.5	5.2	111	235	13	MOLECULE:	SEIPIN
4:00	7rsl-G	5.5	5.2	110	235	13	MOLECULE:	SEIPIN
5:00	7rsl-A	5.5	5.2	110	235	13	MOLECULE:	SEIPIN
6:00	7rsl-I	5.5	5.2	110	235	13	MOLECULE:	SEIPIN
7:00	7rsl-C	5.5	5.2	112	235	13	MOLECULE:	SEIPIN
8:00	6mlu-A	5.5	4.1	105	153	5	MOLECULE:	SEIPIN
9:00	6mlu-B	5.5	4.1	105	153	5	MOLECULE:	SEIPIN
10:00	6za2-A	5.5	3.7	104	1081	11	MOLECULE:	POR SECRETION S
11:00	8qb3-A	5.5	2.4	85	233	11	MOLECULE:	ADDOBODY
12:00	3eb7-A	5.4	3.8	109	589	8	MOLECULE:	INSECTICIDAL DE
13:00	3eb7-C	5.4	3.3	105	588	9	MOLECULE:	INSECTICIDAL DE
14:00	1dlc-A	5.4	4	114	584	4	MOLECULE:	DELTA-ENDOTOXIN
15:00	8qb3-B	5.4	2.8	89	233	10	MOLECULE:	ADDOBODY
16:00	6dj4-A	5.3	3.3	110	571	5	MOLECULE:	CRY1A.105
17:00	6ovb-A	5.3	4.1	110	566	11	MOLECULE:	ACTIVE CORE CRY
18:00	4qx3-A	5.3	4	115	584	5	MOLECULE:	PESTICIDAL CRYS
19:00	4qx0-A	5.3	4	115	584	5	MOLECULE:	PESTICIDAL CRYS
20:00	1ji6-A	5.2	3.9	105	589	6	MOLECULE:	PESTICIDIAL CRY
21:00	4qx1-A	5.2	4	116	584	4	MOLECULE:	PESTICIDAL CRYS
22:00	4qx2-A	5.1	4	112	584	7	MOLECULE:	PESTICIDAL CRYS
23:00	7ear-A	5.1	3.9	110	584	7	MOLECULE:	CRYSTALINE ENTO
24:00:00	3iii-A	5	3.4	114	556	4	MOLECULE:	COCE/NOND FAMIL
25:00:00	4moa-A	5	4	117	602	11	MOLECULE:	PESTICIDAL CRYS
26:00:00	6za2-B	4.9	3.7	111	1083	10	MOLECULE:	POR SECRETION S
27:00:00	5hlz-A	4.9	4.4	103	204	7	MOLECULE:	INHIBIN BETA A
28:00:00	4ary-B	4.8	4.5	106	580	8	MOLECULE:	PESTICIDAL CRYS
29:00:00	4arx-C	4.8	4.5	104	577	9	MOLECULE:	PESTICIDAL CRYS

30:00:00	5hlz-C	4.8	4.3	104	204	7	MOLECULE:	INHIBIN BETA A
31:00:00	5hly-A	4.8	4.4	105	299	7	MOLECULE:	INHIBIN BETA A
32:00:00	4w8j-A	4.7	4.3	103	1017	9	MOLECULE:	PESTICIDAL CRYS
33:00:00	4arx-B	4.7	4.1	106	578	7	MOLECULE:	PESTICIDAL CRYS
34:00:00	5zi1-A	4.7	4	115	590	7	MOLECULE:	INSECTICIDAL CR
35:00:00	4ary-C	4.7	4.5	105	577	7	MOLECULE:	PESTICIDAL CRYS
36:00:00	5cvz-A	4.6	3.6	104	141	6	MOLECULE:	COAT PROTEIN
37:00:00	3eb7-B	4.6	4.5	97	589	7	MOLECULE:	INSECTICIDAL DE
38:00:00	5zi1-B	4.6	3.5	115	590	7	MOLECULE:	INSECTICIDAL CR
39:00:00	3qd6-D	4.6	3.7	100	135	11	MOLECULE:	CD40 LIGAND
40:00:00	1w99-A	4.6	4.2	98	558	10	MOLECULE:	PESTICIDIAL CRY
41:00:00	1stm-A	4.5	3.4	99	141	5	MOLECULE:	SATELLITE PANIC
42:00:00	1stm-C	4.5	3.5	100	141	5	MOLECULE:	SATELLITE PANIC
43:00:00	1stm-B	4.5	3.5	101	141	5	MOLECULE:	SATELLITE PANIC
44:00:00	4arx-D	4.5	4.2	104	576	8	MOLECULE:	PESTICIDAL CRYS
45:00:00	1stm-D	4.5	3.6	102	141	5	MOLECULE:	SATELLITE PANIC
46:00:00	1stm-E	4.5	3.5	100	141	5	MOLECULE:	SATELLITE PANIC
47:00:00	4ary-D	4.5	4.5	100	577	8	MOLECULE:	PESTICIDAL CRYS
48:00:00	3c7g-A	4.4	3.7	101	488	7	MOLECULE:	ENDO-1,4-BETA-X
49:00:00	3ib3-B	4.4	3.3	114	552	4	MOLECULE:	COCE/NOND FAMIL
50:00:00	1aly-A	4.4	3.6	106	146	9	MOLECULE:	CD40 LIGAND
51:00:00	7r1m-A	4.3	3.4	102	156	8	MOLECULE:	EXOSPORIUM PROT
52:00:00	8j3y-A	4.3	4.2	98	392	5	MOLECULE:	PUTATIVE POLYSA
53:00:00	3ib3-A	4.3	3.5	115	551	9	MOLECULE:	COCE/NOND FAMIL
54:00:00	6lfp-A	4.3	4.5	95	582	6	MOLECULE:	CRY3AA PROTEIN
55:00:00	2c9k-A	4.3	5	98	598	6	MOLECULE:	PESTICIDAL CRYS
56:00:00	4ind-A	4.2	5.2	96	377	9	MOLECULE:	C381 TURRET PRO
57:00:00	3qd6-E	4.2	3.8	95	135	11	MOLECULE:	CD40 LIGAND
58:00:00	8w7n-A	4.2	5	96	1011	10	MOLECULE:	PESTICIDAL CRYS
59:00:00	4lr4-C	4.1	3.7	96	346	6	MOLECULE:	HYPOTHETICAL PR
60:00:00	4qpw-A	4.1	3	86	142	8	MOLECULE:	GLYCOSYL HYDROL
61:00:00	1i9r-A	4.1	3.9	101	143	10	MOLECULE:	CD40 LIGAND
62:00:00	3qd6-B	4.1	3.8	95	135	11	MOLECULE:	CD40 LIGAND
63:00:00	3qd6-F	4.1	3.5	95	135	8	MOLECULE:	CD40 LIGAND
64:00:00	2rjl-A	4.1	4	99	141	11	MOLECULE:	TNF SUPERFAMILY
65:00:00	1i9r-C	4.1	3.8	99	143	9	MOLECULE:	CD40 LIGAND
66:00:00	6w9g-B	4.1	3.5	95	137	9	MOLECULE:	CD40 LIGAND
67:00:00	3qd6-A	4.1	3.3	99	135	8	MOLECULE:	CD40 LIGAND
68:00:00	4ary-A	4.1	5.1	92	578	7	MOLECULE:	PESTICIDAL CRYS
69:00:00	4qhw-A	4	3.2	91	400	3	MOLECULE:	UNCHARACTERIZED
70:00:00	6w9g-A	4	3.6	93	137	11	MOLECULE:	CD40 LIGAND
71:00:00	4arx-A	4	3.6	107	578	9	MOLECULE:	PESTICIDAL CRYS
72:00:00	7sgm-C	4	3.7	96	137	9	MOLECULE:	CD40 LIGAND
73:00:00	3qd6-C	4	3.5	100	135	11	MOLECULE:	CD40 LIGAND

74:00:00	1i9r-B	4	3.9	100	143	8	MOLECULE:	CD40 LIGAND
75:00:00	2rjk-A	4	4.1	96	141	9	MOLECULE:	TNF SUPERFAMILY
76:00:00	5cw0-A	4	3.8	100	141	6	MOLECULE:	COAT PROTEIN
77:00:00	7vt4-A	3.9	4.3	86	438	7	MOLECULE:	ENDOGLUCANASE H
78:00:00	7kx0-A	3.9	3.8	95	140	5	MOLECULE:	CD70 ANTIGEN
79:00:00	5jsd-A	3.9	3.2	85	548	7	MOLECULE:	PHIAB6 TAILSPIK
80:00:00	8xcg-J	3.9	6.8	106	598	5	MOLECULE:	TAIL TIP PROTEI
81:00:00	4mtm-A	3.9	4.2	81	137	10	MOLECULE:	PUTATIVE TAIL F
82:00:00	6wpc-D	3.9	3.3	114	588	11	MOLECULE:	CRY1A.2
83:00:00	5hlz-G	3.9	4.5	97	204	6	MOLECULE:	INHIBIN BETA A
84:00:00	3lkj-A	3.9	3.8	97	133	11	MOLECULE:	CD40 LIGAND
85:00:00	6w9g-C	3.9	3.5	98	139	8	MOLECULE:	CD40 LIGAND
86:00:00	4e4d-X	3.9	3.9	96	154	3	MOLECULE:	TUMOR NECROSIS
87:00:00	2wys-B	3.8	3.3	90	516	8	MOLECULE:	ENDO-1,4-BETA-X
88:00:00	6sih-A	3.8	18.2	98	488	8	MOLECULE:	FLAGELLAR HOOK-
89:00:00	2hey-F	3.8	3.8	87	135	10	MOLECULE:	TUMOR NECROSIS
90:00:00	2qe3-A	3.8	4	96	140	10	MOLECULE:	TNF SUPERFAMILY
91:00:00	3lkj-C	3.8	3.7	91	129	2	MOLECULE:	CD40 LIGAND
92:00:00	1jtz-X	3.8	3.8	97	155	3	MOLECULE:	TUMOR NECROSIS
93:00:00	7dn2-1	3.7	3.2	89	126	8	MOLECULE:	MAJOR STRUCTURA
94:00:00	8des-A	3.7	3.7	102	475	7	MOLECULE:	MAJOR CAPSID PR
95:00:00	4z11-A	3.7	4.3	97	513	2	MOLECULE:	AURONE SYNTHASE
96:00:00	5hlz-E	3.7	4.4	104	204	5	MOLECULE:	INHIBIN BETA A
97:00:00	4msv-A	3.7	4.1	100	139	4	MOLECULE:	TUMOR NECROSIS
98:00:00	3wqb-A	3.6	3.8	104	597	13	MOLECULE:	EXTRACELLULAR S
99:00:00	8ond-A	3.6	3.5	103	344	5	MOLECULE:	BD2133
0:00	4ht1-T	3.6	3.8	87	131	5	MOLECULE:	TUMOR NECROSIS
1:00	6iwy-A	3.6	2.6	75	366	12	MOLECULE:	FLAGELLAR HOOK-
2:00	5zru-A	3.6	6.8	105	576	7	MOLECULE:	ALPHA-1,3-GLUCA
3:00	5cir-D	3.6	4.3	102	150	8	MOLECULE:	TUMOR NECROSIS
4:00	5l19-A	3.6	4.7	100	150	5	MOLECULE:	TUMOR NECROSIS
5:00	7ta6-B	3.6	4.4	96	129	7	MOLECULE:	TUMOR NECROSIS
6:00	7sgm-A	3.6	3.9	95	137	8	MOLECULE:	CD40 LIGAND
7:00	3lkj-B	3.6	3.8	97	132	6	MOLECULE:	CD40 LIGAND
8:00	5cir-B	3.6	4.1	101	151	9	MOLECULE:	TUMOR NECROSIS
9:00	4mxw-D	3.6	4.4	104	135	6	MOLECULE:	TUMOR NECROSIS
10:00	5cir-A	3.5	4.2	102	149	8	MOLECULE:	TUMOR NECROSIS
11:00	7ta6-A	3.5	3.9	109	141	5	MOLECULE:	TUMOR NECROSIS
12:00	7ta6-H	3.5	3.4	98	131	7	MOLECULE:	TUMOR NECROSIS
13:00	6w98-A	3.4	9.3	141	1312	6	MOLECULE:	F5/8 TYPE C DOM
14:00	2vzs-A	3.4	3.5	92	857	9	MOLECULE:	EXO-BETA-D-GLUC
15:00	1a6c-A	3.4	7.1	96	513	4	MOLECULE:	TOBACCO RINGSPO
16:00	2gh8-A	3.4	6.8	103	544	4	MOLECULE:	CAPSID PROTEIN
17:00	2wz8-A	3.4	3.3	82	135	11	MOLECULE:	CELLULOSOME PRO

18:00	7ta6-F	3.4	3.8	105	138	8	MOLECULE:	TUMOR NECROSIS
19:00	7jra-B	3.4	4.7	98	146	7	MOLECULE:	TUMOR NECROSIS
20:00	6x86-F	3.4	3.8	103	144	7	MOLECULE:	TUMOR NECROSIS
21:00	6x85-F	3.4	3.7	104	144	7	MOLECULE:	TUMOR NECROSIS
22:00	7sgm-B	3.4	3.8	100	140	3	MOLECULE:	CD40 LIGAND
23:00	7ta6-G	3.4	4.4	97	138	6	MOLECULE:	TUMOR NECROSIS
24:00:00	3k51-A	3.4	3.7	97	143	3	MOLECULE:	TUMOR NECROSIS
25:00:00	7kp9-B	3.4	3.9	97	139	10	MOLECULE:	TUMOR NECROSIS
26:00:00	7w63-B	3.3	4.1	94	395	7	MOLECULE:	TOXIN-COREGULAT
27:00:00	4jdm-C	3.3	4.2	102	269	7	MOLECULE:	VIRULENCE PLASM
28:00:00	7ta6-E	3.3	3.9	102	136	7	MOLECULE:	TUMOR NECROSIS
29:00:00	2tun-D	3.3	3.6	105	151	6	MOLECULE:	TUMOR NECROSIS
30:00:00	2tun-F	3.3	3.9	103	151	10	MOLECULE:	TUMOR NECROSIS
31:00:00	2tun-A	3.3	4	108	151	7	MOLECULE:	TUMOR NECROSIS
32:00:00	6ooy-B	3.3	3.9	103	140	10	MOLECULE:	TUMOR NECROSIS
33:00:00	6ooy-A	3.3	4	102	140	7	MOLECULE:	TUMOR NECROSIS
34:00:00	6ooz-C	3.3	4	96	126	8	MOLECULE:	TUMOR NECROSIS
35:00:00	2zpx-B	3.3	4.3	99	142	7	MOLECULE:	TUMOR NECROSIS
36:00:00	6rmj-C	3.3	3.8	108	140	6	MOLECULE:	TUMOR NECROSIS
37:00:00	5mu8-f	3.3	3.8	99	143	8	MOLECULE:	TUMOR NECROSIS
38:00:00	6ooz-B	3.3	3.8	95	142	8	MOLECULE:	TUMOR NECROSIS
39:00:00	2o0o-B	3.3	3.9	104	153	8	MOLECULE:	TNF SUPERFAMILY
40:00:00	6brb-A	3.3	3.6	97	142	2	MOLECULE:	CD40 LIGAND
41:00:00	5mu8-L	3.3	4	103	142	8	MOLECULE:	TUMOR NECROSIS
42:00:00	6x82-F	3.3	3.6	95	140	6	MOLECULE:	TUMOR NECROSIS
43:00:00	2re9-A	3.3	3.8	114	164	5	MOLECULE:	TNF SUPERFAMILY
44:00:00	8env-A	3.2	15	87	504	9	MOLECULE:	SHEATH PROTEIN
45:00:00	8gld-A	3.2	3.6	84	365	6	MOLECULE:	SGNH HYDROLASE-
46:00:00	1dg6-A	3.2	4.2	95	149	9	MOLECULE:	APO2L/TNF-RELAT
47:00:00	5uui-A	3.2	4.2	90	134	11	MOLECULE:	TUMOR NECROSIS
48:00:00	2zjc-A	3.2	3.8	103	142	11	MOLECULE:	TUMOR NECROSIS
49:00:00	7ta6-D	3.2	3.7	104	145	6	MOLECULE:	TUMOR NECROSIS
50:00:00	4mxw-Y	3.2	3.5	95	131	8	MOLECULE:	TUMOR NECROSIS
51:00:00	1tnf-A	3.2	3.8	102	152	8	MOLECULE:	TUMOR NECROSIS
52:00:00	2az5-B	3.2	3.7	97	139	9	MOLECULE:	TUMOR NECROSIS
53:00:00	6x85-C	3.2	3.9	100	144	7	MOLECULE:	TUMOR NECROSIS
54:00:00	7ta6-C	3.2	4	102	138	8	MOLECULE:	TUMOR NECROSIS
55:00:00	3it8-G	3.2	4	99	152	8	MOLECULE:	TUMOR NECROSIS
56:00:00	3it8-I	3.2	4	107	152	5	MOLECULE:	TUMOR NECROSIS
57:00:00	6op0-B	3.2	3.9	104	141	7	MOLECULE:	TUMOR NECROSIS
58:00:00	3it8-H	3.2	4	107	152	5	MOLECULE:	TUMOR NECROSIS
59:00:00	5mu8-H	3.2	4	101	150	7	MOLECULE:	TUMOR NECROSIS
60:00:00	5yoy-B	3.2	3.8	98	154	9	MOLECULE:	TUMOR NECROSIS
61:00:00	1s55-A	3.2	3.7	98	156	3	MOLECULE:	TUMOR NECROSIS

62:00:00	7kp9-C	3.2	3.6	100	131	7	MOLECULE:	TUMOR NECROSIS
63:00:00	6ooz-A	3.2	4	101	147	7	MOLECULE:	TUMOR NECROSIS
64:00:00	3it8-C	3.2	3.7	99	152	9	MOLECULE:	TUMOR NECROSIS
65:00:00	5yoy-C	3.2	3.6	100	154	7	MOLECULE:	TUMOR NECROSIS
66:00:00	6x86-B	3.2	3.7	95	130	6	MOLECULE:	TUMOR NECROSIS
67:00:00	5m2m-M	3.2	3.8	102	148	8	MOLECULE:	TUMOR NECROSIS
68:00:00	1iqa-A	3.2	3.8	99	156	4	MOLECULE:	RECEPTOR ACTIVA
69:00:00	1tnf-B	3.2	3.8	102	152	9	MOLECULE:	TUMOR NECROSIS
70:00:00	6x85-A	3.2	3.9	98	148	8	MOLECULE:	TUMOR NECROSIS
71:00:00	1tnf-C	3.2	3.8	101	152	10	MOLECULE:	TUMOR NECROSIS
72:00:00	5yoy-L	3.2	4.1	106	153	7	MOLECULE:	TUMOR NECROSIS
73:00:00	5mu8-b	3.2	3.8	96	144	8	MOLECULE:	TUMOR NECROSIS
74:00:00	7kpa-B	3.2	3.9	100	146	10	MOLECULE:	TUMOR NECROSIS
75:00:00	2zpx-C	3.2	4.2	102	142	6	MOLECULE:	TUMOR NECROSIS
76:00:00	3wd5-A	3.2	3.8	110	152	5	MOLECULE:	TUMOR NECROSIS
77:00:00	1a8m-B	3.2	3.5	101	152	7	MOLECULE:	TUMOR NECROSIS
78:00:00	5l36-A	3.2	3.7	90	138	6	MOLECULE:	TUMOR NECROSIS
79:00:00	6x83-E	3.2	4.2	97	130	10	MOLECULE:	TUMOR NECROSIS
80:00:00	3mi8-A	3.2	3.7	89	144	4	MOLECULE:	TUMOR NECROSIS
81:00:00	3it8-A	3.2	4	107	152	5	MOLECULE:	TUMOR NECROSIS
82:00:00	6q5u-R	3.1	3.4	81	125	12	MOLECULE:	MAJOR CAPSID PR
83:00:00	6x85-B	3.1	3.9	98	129	8	MOLECULE:	TUMOR NECROSIS
84:00:00	3it8-B	3.1	4.3	96	152	4	MOLECULE:	TUMOR NECROSIS
85:00:00	7kp8-C	3.1	3.9	92	137	11	MOLECULE:	TUMOR NECROSIS
86:00:00	7kpb-C	3.1	3.9	97	138	9	MOLECULE:	TUMOR NECROSIS
87:00:00	5yoy-A	3.1	3.9	106	154	8	MOLECULE:	TUMOR NECROSIS
88:00:00	1iqa-C	3.1	4.5	98	156	4	MOLECULE:	RECEPTOR ACTIVA
89:00:00	3alq-E	3.1	4.2	96	141	9	MOLECULE:	TUMOR NECROSIS
90:00:00	1a8m-A	3.1	3.9	98	152	8	MOLECULE:	TUMOR NECROSIS
91:00:00	5mu8-C	3.1	4	100	146	9	MOLECULE:	TUMOR NECROSIS
92:00:00	7kp9-A	3.1	4.1	105	148	9	MOLECULE:	TUMOR NECROSIS
93:00:00	3urf-A	3.1	4.1	99	156	3	MOLECULE:	TUMOR NECROSIS
94:00:00	6x83-F	3.1	4.1	101	138	9	MOLECULE:	TUMOR NECROSIS
95:00:00	5wux-G	3.1	4.1	98	141	7	MOLECULE:	HEAVY
96:00:00	1s55-B	3.1	4	95	156	4	MOLECULE:	TUMOR NECROSIS
97:00:00	5mu8-X	3.1	3.8	101	150	6	MOLECULE:	TUMOR NECROSIS
98:00:00	5m2m-C	3.1	4	98	150	6	MOLECULE:	TUMOR NECROSIS
99:00:00	6op0-C	3.1	3.9	92	119	10	MOLECULE:	TUMOR NECROSIS
0:00	5mu8-I	3.1	4	100	141	6	MOLECULE:	TUMOR NECROSIS
1:00	2tnf-B	3.1	3.6	102	148	8	MOLECULE:	PROTEIN (TUMOR
2:00	7kpa-A	3.1	4.2	102	147	8	MOLECULE:	TUMOR NECROSIS
3:00	1d0g-B	3.1	4	105	151	4	MOLECULE:	DEATH RECEPTOR-
4:00	5mu8-d	3.1	4.1	99	138	9	MOLECULE:	TUMOR NECROSIS
5:00	7kp8-A	3.1	4	96	140	8	MOLECULE:	TUMOR NECROSIS

6:00	1a8m-C	3.1	4.1	101	152	7	MOLECULE:	TUMOR NECROSIS
7:00	2az5-A	3.1	3.9	99	131	8	MOLECULE:	TUMOR NECROSIS
8:00	7kp7-B	3.1	4	98	148	7	MOLECULE:	TUMOR NECROSIS
9:00	7kpb-B	3.1	4	99	143	7	MOLECULE:	TUMOR NECROSIS
10:00	6x82-C	3.1	3.3	98	143	7	MOLECULE:	TUMOR NECROSIS
11:00	6x83-C	3.1	3.7	93	135	6	MOLECULE:	TUMOR NECROSIS
12:00	5m2m-G	3.1	4	98	150	6	MOLECULE:	TUMOR NECROSIS
13:00	6x83-B	3.1	4.1	99	138	10	MOLECULE:	TUMOR NECROSIS
14:00	2o0o-A	3.1	4.2	102	153	4	MOLECULE:	TNF SUPERFAMILY
15:00	5mu8-i	3.1	4.1	98	145	9	MOLECULE:	TUMOR NECROSIS
16:00	2e7a-B	3.1	3.8	100	142	9	MOLECULE:	TUMOR NECROSIS
17:00	6x81-F	3.1	3.8	102	144	7	MOLECULE:	TUMOR NECROSIS
18:00	4mxw-B	3.1	3.5	87	126	6	MOLECULE:	TUMOR NECROSIS
19:00	6ooy-C	3.1	4.1	91	134	5	MOLECULE:	TUMOR NECROSIS
20:00	7kpa-C	3.1	3.9	97	133	7	MOLECULE:	TUMOR NECROSIS
21:00	5mu8-g	3.1	4	102	145	9	MOLECULE:	TUMOR NECROSIS
22:00	4twt-D	3.1	3.9	95	130	6	MOLECULE:	TUMOR NECROSIS
23:00	5m2m-I	3.1	3.9	101	150	7	MOLECULE:	TUMOR NECROSIS
24:00:00	5yoy-K	3.1	4	105	153	7	MOLECULE:	TUMOR NECROSIS
25:00:00	3qbq-A	3.1	4.4	98	155	4	MOLECULE:	TUMOR NECROSIS
26:00:00	2zpx-A	3.1	3.9	100	142	9	MOLECULE:	TUMOR NECROSIS
27:00:00	2az5-C	3.1	3.6	96	128	8	MOLECULE:	TUMOR NECROSIS
28:00:00	7kp7-C	3.1	3.8	102	148	10	MOLECULE:	TUMOR NECROSIS
29:00:00	2e7a-A	3.1	3.7	103	150	6	MOLECULE:	TUMOR NECROSIS
30:00:00	6x81-D	3.1	3.9	99	141	10	MOLECULE:	TUMOR NECROSIS
31:00:00	6x83-D	3.1	3.9	98	127	11	MOLECULE:	TUMOR NECROSIS
32:00:00	5yoy-J	3.1	3.7	108	153	6	MOLECULE:	TUMOR NECROSIS
33:00:00	2o14-A	3	2.9	83	354	4	MOLECULE:	HYPOTHETICAL PR
34:00:00	1d2q-B	3	3.5	84	134	6	MOLECULE:	TNF-RELATED APO
35:00:00	6x82-D	3	4.1	95	142	9	MOLECULE:	TUMOR NECROSIS
36:00:00	5wux-F	3	4.1	101	141	8	MOLECULE:	HEAVY
37:00:00	3alq-A	3	4.3	97	141	11	MOLECULE:	TUMOR NECROSIS
38:00:00	6rmj-B	3	4.1	100	147	6	MOLECULE:	TUMOR NECROSIS
39:00:00	5mu8-R	3	4.1	101	138	7	MOLECULE:	TUMOR NECROSIS
40:00:00	5mu8-r	3	3.9	100	144	9	MOLECULE:	TUMOR NECROSIS
41:00:00	8kb6-A	3	3.9	98	148	6	MOLECULE:	TUMOR NECROSIS
42:00:00	6x86-D	3	3.8	100	139	7	MOLECULE:	TUMOR NECROSIS
43:00:00	5mu8-j	3	3.9	99	150	8	MOLECULE:	TUMOR NECROSIS
44:00:00	5mu8-k	3	3.9	100	142	7	MOLECULE:	TUMOR NECROSIS
45:00:00	6x81-B	3	4.4	95	146	6	MOLECULE:	TUMOR NECROSIS
46:00:00	6x86-A	3	3.9	104	151	7	MOLECULE:	TUMOR NECROSIS
47:00:00	6x85-E	3	4.1	98	135	7	MOLECULE:	TUMOR NECROSIS
48:00:00	1iqa-B	3	4.6	103	156	5	MOLECULE:	RECEPTOR ACTIVA
49:00:00	5mu8-l	3	3.7	97	143	8	MOLECULE:	TUMOR NECROSIS

50:00:00	2o0o-C	3	3.9	101	152	8	MOLECULE:	TNF SUPERFAMILY
51:00:00	6x82-B	3	4.2	99	139	10	MOLECULE:	TUMOR NECROSIS
52:00:00	5mu8-q	3	3.8	102	149	9	MOLECULE:	TUMOR NECROSIS
53:00:00	5mu8-F	3	3.9	98	140	8	MOLECULE:	TUMOR NECROSIS
54:00:00	5m2i-C	3	3.9	100	148	7	MOLECULE:	TUMOR NECROSIS
55:00:00	7kp7-A	3	3.9	97	148	6	MOLECULE:	TUMOR NECROSIS
56:00:00	5m2m-B	3	4.1	102	151	10	MOLECULE:	TUMOR NECROSIS
57:00:00	5mu8-P	3	3.7	97	145	7	MOLECULE:	TUMOR NECROSIS
58:00:00	3alq-D	3	3.7	101	141	6	MOLECULE:	TUMOR NECROSIS
59:00:00	7kpb-A	3	3.8	104	153	9	MOLECULE:	TUMOR NECROSIS
60:00:00	5m2j-A	3	3.8	104	139	7	MOLECULE:	TUMOR NECROSIS
61:00:00	2zjc-C	3	4.1	100	142	11	MOLECULE:	TUMOR NECROSIS
62:00:00	5mu8-Z	3	3.8	103	143	6	MOLECULE:	TUMOR NECROSIS
63:00:00	5mu8-M	3	3.9	104	149	8	MOLECULE:	TUMOR NECROSIS
64:00:00	5mu8-e	3	3.7	100	149	6	MOLECULE:	TUMOR NECROSIS
65:00:00	7jra-A	3	3.9	102	153	9	MOLECULE:	TUMOR NECROSIS
66:00:00	5mu8-m	3	4.1	96	147	8	MOLECULE:	TUMOR NECROSIS
67:00:00	5mu8-n	3	3.7	97	147	8	MOLECULE:	TUMOR NECROSIS
68:00:00	3alq-B	3	4.1	100	141	9	MOLECULE:	TUMOR NECROSIS
69:00:00	5m2m-A	3	3.7	99	150	7	MOLECULE:	TUMOR NECROSIS
70:00:00	5m2i-F	3	3.9	100	148	7	MOLECULE:	TUMOR NECROSIS
71:00:00	1d0g-D	3	3.8	98	151	4	MOLECULE:	DEATH RECEPTOR-
72:00:00	5mu8-S	3	4	101	149	7	MOLECULE:	TUMOR NECROSIS
73:00:00	6x81-E	3	3.8	97	132	5	MOLECULE:	TUMOR NECROSIS
74:00:00	7kp8-B	3	4	100	140	13	MOLECULE:	TUMOR NECROSIS
75:00:00	1s55-C	3	4.4	100	156	4	MOLECULE:	TUMOR NECROSIS
76:00:00	3qbq-C	3	4.6	98	155	3	MOLECULE:	TUMOR NECROSIS
77:00:00	5mu8-J	3	4.1	97	138	9	MOLECULE:	TUMOR NECROSIS
78:00:00	1jtz-Z	2.9	4.5	99	155	3	MOLECULE:	TUMOR NECROSIS
79:00:00	5bnq-A	2.9	4.4	99	156	4	MOLECULE:	TUMOR NECROSIS
80:00:00	1jtz-Y	2.9	4.1	98	155	3	MOLECULE:	TUMOR NECROSIS
81:00:00	4giq-A	2.9	4.3	102	155	5	MOLECULE:	TUMOR NECROSIS
82:00:00	5wux-E	2.9	4.5	102	136	9	MOLECULE:	HEAVY
83:00:00	4n90-B	2.9	4.7	103	155	3	MOLECULE:	TUMOR NECROSIS
84:00:00	5mu8-a	2.9	3.9	101	149	9	MOLECULE:	TUMOR NECROSIS
85:00:00	2tun-C	2.9	4	103	151	10	MOLECULE:	TUMOR NECROSIS
86:00:00	1du3-L	2.9	4.4	104	152	3	MOLECULE:	DEATH RECEPTOR
87:00:00	7ta3-D	2.9	4.1	100	149	7	MOLECULE:	ALPHA-PEPTIDE-3
88:00:00	1du3-E	2.9	4.4	105	152	3	MOLECULE:	DEATH RECEPTOR
89:00:00	6x81-C	2.9	3.7	97	144	6	MOLECULE:	TUMOR NECROSIS
90:00:00	2az5-D	2.9	3.8	94	140	6	MOLECULE:	TUMOR NECROSIS
91:00:00	7jra-C	2.9	4	99	142	6	MOLECULE:	TUMOR NECROSIS
92:00:00	5mu8-v	2.9	3.8	97	144	9	MOLECULE:	TUMOR NECROSIS
93:00:00	5mu8-B	2.9	3.9	94	145	10	MOLECULE:	TUMOR NECROSIS

94:00:00	5mu8-s	2.9	3.7	96	149	10	MOLECULE:	TUMOR NECROSIS
95:00:00	5m2i-A	2.9	4.1	103	148	7	MOLECULE:	TUMOR NECROSIS
96:00:00	5mu8-G	2.9	3.7	100	149	6	MOLECULE:	TUMOR NECROSIS
97:00:00	1du3-K	2.9	4.2	100	152	4	MOLECULE:	DEATH RECEPTOR
98:00:00	4twt-A	2.9	3.8	98	149	9	MOLECULE:	TUMOR NECROSIS
99:00:00	5mu8-V	2.9	3.9	102	138	10	MOLECULE:	TUMOR NECROSIS
0:00	5mu8-O	2.9	4.2	99	145	9	MOLECULE:	TUMOR NECROSIS
1:00	2tnf-C	2.9	4.1	98	148	8	MOLECULE:	PROTEIN (TUMOR
2:00	5mu8-Q	2.9	3.9	106	149	6	MOLECULE:	TUMOR NECROSIS
3:00	5mu8-u	2.9	3.7	93	145	5	MOLECULE:	TUMOR NECROSIS
4:00	2zjc-B	2.9	3.9	100	151	10	MOLECULE:	TUMOR NECROSIS
5:00	5mu8-h	2.9	4.2	98	137	6	MOLECULE:	TUMOR NECROSIS
6:00	6x82-E	2.9	4	100	136	9	MOLECULE:	TUMOR NECROSIS
7:00	6rmj-A	2.9	4.2	99	149	7	MOLECULE:	TUMOR NECROSIS
8:00	1d2q-A	2.9	3.4	85	134	5	MOLECULE:	TNF-RELATED APO
9:00	5mu8-U	2.9	4.1	98	149	9	MOLECULE:	TUMOR NECROSIS
10:00	5mu8-o	2.9	4	98	147	9	MOLECULE:	TUMOR NECROSIS
11:00	5m2i-B	2.8	4.2	96	148	8	MOLECULE:	TUMOR NECROSIS
12:00	2e7a-C	2.8	3.9	97	143	6	MOLECULE:	TUMOR NECROSIS
13:00	6x86-E	2.8	4.1	98	135	8	MOLECULE:	TUMOR NECROSIS
14:00	5tsw-E	2.8	4.3	95	148	9	MOLECULE:	PROTEIN (TUMOR
15:00	3l9j-T	2.8	3.7	99	149	5	MOLECULE:	TNFALPHA
16:00	3me2-A	2.8	4.5	99	156	4	MOLECULE:	TUMOR NECROSIS
17:00	1du3-J	2.8	4	99	152	4	MOLECULE:	DEATH RECEPTOR
18:00	1du3-F	2.8	4.5	105	152	3	MOLECULE:	DEATH RECEPTOR
19:00	2tun-E	2.8	4.4	93	151	6	MOLECULE:	TUMOR NECROSIS
20:00	2tnf-A	2.8	3.8	95	148	9	MOLECULE:	PROTEIN (TUMOR
21:00	5mu8-w	2.8	3.9	98	145	6	MOLECULE:	TUMOR NECROSIS
22:00	1du3-D	2.8	4.4	101	152	4	MOLECULE:	DEATH RECEPTOR
23:00	3alq-F	2.8	4.2	100	141	10	MOLECULE:	TUMOR NECROSIS
24:00:00	5mu8-Y	2.8	3.8	99	149	10	MOLECULE:	TUMOR NECROSIS
25:00:00	5mu8-A	2.8	4.3	98	141	8	MOLECULE:	TUMOR NECROSIS
26:00:00	4mxw-Z	2.8	3.8	92	134	9	MOLECULE:	TUMOR NECROSIS
27:00:00	5m2i-D	2.8	4.1	94	148	6	MOLECULE:	TUMOR NECROSIS
28:00:00	6x83-A	2.8	3.8	94	141	9	MOLECULE:	TUMOR NECROSIS
29:00:00	5mu8-W	2.8	4	98	143	7	MOLECULE:	TUMOR NECROSIS
30:00:00	1d4v-B	2.8	4.5	104	163	4	MOLECULE:	TNF-RELATED APO
31:00:00	4twt-C	2.8	4.1	99	142	7	MOLECULE:	TUMOR NECROSIS
32:00:00	1d0g-A	2.8	4	102	151	5	MOLECULE:	DEATH RECEPTOR-
33:00:00	5tsw-B	2.8	4.2	101	148	11	MOLECULE:	PROTEIN (TUMOR
34:00:00	5mu8-t	2.8	4.1	96	143	8	MOLECULE:	TUMOR NECROSIS
35:00:00	5tsw-C	2.8	4	99	148	7	MOLECULE:	PROTEIN (TUMOR
36:00:00	5mu8-c	2.8	3.9	93	145	9	MOLECULE:	TUMOR NECROSIS
37:00:00	5ubu-B	2.7	4.8	116	331	7	MOLECULE:	PUTATIVE ACETAM

38:00:00	6x81-A	2.7	4.1	101	145	7	MOLECULE:	TUMOR NECROSIS
39:00:00	5mu8-N	2.7	3.9	96	143	8	MOLECULE:	TUMOR NECROSIS
40:00:00	5mu8-T	2.7	4.2	97	146	10	MOLECULE:	TUMOR NECROSIS
41:00:00	5mu8-D	2.7	4	95	149	12	MOLECULE:	TUMOR NECROSIS
42:00:00	6x82-A	2.6	4	101	143	4	MOLECULE:	TUMOR NECROSIS
43:00:00	4n90-A	2.6	4.2	96	155	3	MOLECULE:	TUMOR NECROSIS
44:00:00	4n90-C	2.6	3.9	98	154	7	MOLECULE:	TUMOR NECROSIS
45:00:00	5tsw-D	2.6	3.7	102	148	9	MOLECULE:	PROTEIN (TUMOR
46:00:00	5tsw-A	2.5	4	98	148	9	MOLECULE:	PROTEIN (TUMOR
47:00:00	5tsw-F	2.5	4	98	148	7	MOLECULE:	PROTEIN (TUMOR
48:00:00	4g3y-C	2.5	4	99	147	4	MOLECULE:	INFLIXIMAB FAB
49:00:00	3alq-C	2.5	3.8	91	141	11	MOLECULE:	TUMOR NECROSIS
50:00:00	6kty-A	2.4	2.7	63	176	6	MOLECULE:	FLAGELLAR HOOK-
51:00:00	6x80-C	2.4	16.9	80	574	11	MOLECULE:	FLAGELLIN A
52:00:00	8slr-D	2.2	4.1	89	155	4	MOLECULE:	TUMOR NECROSIS

DALI results: MdA-1 Hexon

No:	Chain	Z	rmsd	lali	nres	%id	PDB Description	
1:00	8rbt-A	14.9	3.9	333	481	10	MOLECULE:	MAJOR CAPSID PROTEIN
2:00	6g43-A	14.4	4.3	353	515	7	MOLECULE:	PUTATIVE MAJOR CAPSID PROTEIN
3:00	5j7o-F	14.2	4.3	347	622	10	MOLECULE:	MAJOR CAPSID PROTEIN
4:00	3j26-A	13.8	4.5	370	508	10	MOLECULE:	CAPSID PROTEIN V20
5:00	5tip-A	13.8	3.6	322	436	9	MOLECULE:	MAJOR CAPSID PROTEIN
6:00	3sam-B	12.4	4	316	544	8	MOLECULE:	RIFAMPICIN RESISTANCE PROTEIN
7:00	7yjl-A	11.4	4	335	459	6	MOLECULE:	MAJOR CAPSID PROTEIN
8:00	6l2t-C	10.6	4.5	353	633	7	MOLECULE:	B646L,MAJOR CAPSID PROTEIN
9:00	2iny-A	10.4	4.3	400	941	8	MOLECULE:	HEXON PROTEIN
10:00	6ojn-A	10.4	4.2	313	423	7	MOLECULE:	MAJOR CAPSID PROTEIN
11:00	6q5u-C	9.6	4.9	291	394	6	MOLECULE:	MAJOR CAPSID PROTEIN (P3)
12:00	9cls-K	8.9	5.4	401	935	6	MOLECULE:	HEXON PROTEIN
13:00	2vvf-A	7.5	4.2	207	269	7	MOLECULE:	MAJOR CAPSID PROTEIN P2
14:00	3j31-A	6.3	4.2	215	344	8	MOLECULE:	A223 PENTON BASE
15:00	5oac-A	5.4	4.9	186	310	9	MOLECULE:	MAJOR CAPSID PROTEIN
16:00	7zzz-A	4.4	4.2	172	239	7	MOLECULE:	MAJOR CAPSID PROTEIN P5
17:00	7v1v-A	3	4.7	205	447	4	MOLECULE:	DIFRUCTOSE DIANHYDRIDE I SYNTHASE/HYDROLASE

DALI results: MdA-1 Adenain

No:	Chain	Z	rmsd	lali	nres	%id	PDB Description	
1:00	4pis-A	13.4	2.8	148	204	14	MOLECULE:	PROTEASE
2:00	4wx4-A	13.3	2.7	146	201	14	MOLECULE:	PROTEASE
3:00	4piq-A	13.3	2.7	146	204	12	MOLECULE:	PROTEASE
4:00	1nln-A	13.2	2.9	148	203	16	MOLECULE:	ADENAIN
5:00	4wx7-C	13.2	2.7	147	204	14	MOLECULE:	PROTEASE
6:00	4pie-A	13.2	2.7	147	202	14	MOLECULE:	PROTEASE
7:00	4wx6-A	13.2	2.7	147	204	14	MOLECULE:	PROTEASE
8:00	4wx6-C	13.1	2.7	147	204	14	MOLECULE:	PROTEASE
9:00	4pid-A	13.1	2.8	147	204	15	MOLECULE:	PROTEASE
10:00	4wx7-A	13.1	2.8	147	204	12	MOLECULE:	PROTEASE
11:00	4ekf-A	13.1	2.7	143	192	14	MOLECULE:	ADENAIN
12:00	5fgy-A	12.9	2.8	146	203	14	MOLECULE:	PROTEASE
13:00	1avp-A	12.8	2.8	146	204	14	MOLECULE:	ADENOVIRAL PROTEINASE
14:00	7fiu-A	11.9	4.5	166	270	13	MOLECULE:	ULP_PROTEASE DOMAIN-CONTAINING
15:00	6ups-A	11.6	3.6	147	245	15	MOLECULE:	ULP_PROTEASE DOMAIN-CONTAINING
16:00	6upu-E	11.2	3.6	147	258	14	MOLECULE:	ULP_PROTEASE DOMAIN-CONTAINING
17:00	8efx-A	11.1	3.9	150	259	13	MOLECULE:	OTDUB
18:00	6upu-M	11	3.6	149	258	14	MOLECULE:	ULP_PROTEASE DOMAIN-CONTAINING
19:00	6upu-I	10.8	3.5	148	258	14	MOLECULE:	ULP_PROTEASE DOMAIN-CONTAINING
20:00	5haf-A	10.8	6.6	166	312	10	MOLECULE:	DEUBIQUITINASE SSEL
21:00	5ubw-B	10.8	3.1	145	173	10	MOLECULE:	DEUBIQUITINASE SSEL
22:00	5ubw-A	10.7	3.2	146	175	12	MOLECULE:	DEUBIQUITINASE SSEL
23:00	6upu-A	10.7	3.4	146	259	14	MOLECULE:	ULP_PROTEASE DOMAIN-CONTAINING
24:00:00	6wtg-A	10.6	3.6	147	169	10	MOLECULE:	UBIQUITINATING/DEUBIQUITINATIN
25:00:00	5cra-A	10.4	3.5	143	172	10	MOLECULE:	SDEA
26:00:00	5ham-B	10.3	2.9	136	260	13	MOLECULE:	RICKCE
27:00:00	1xt9-A	10.3	3.4	143	208	13	MOLECULE:	SENTRIN-SPECIFIC PROTEASE 8
28:00:00	5b5q-B	10.3	3.5	140	244	16	MOLECULE:	MEMBRANE THIOL PROTEASE
29:00:00	5cra-B	10.3	3.5	143	172	10	MOLECULE:	SDEA
30:00:00	5ham-A	10.2	2.9	135	260	13	MOLECULE:	RICKCE
31:00:00	5haf-B	10.1	9.3	169	327	10	MOLECULE:	DEUBIQUITINASE SSEL
32:00:00	2bkq-A	10.1	3.5	146	210	12	MOLECULE:	SENTRIN-SPECIFIC PROTEASE 8
33:00:00	2bkr-A	10	3.4	142	211	12	MOLECULE:	SENTRIN-SPECIFIC PROTEASE 8
34:00:00	6be0-A	10	3.5	150	266	14	MOLECULE:	AVRA
35:00:00	2bkq-C	9.9	3.6	142	207	13	MOLECULE:	SENTRIN-SPECIFIC PROTEASE 8
36:00:00	5crb-B	9.8	3.8	151	191	11	MOLECULE:	SDEA
37:00:00	6lj9-A	9.8	6	139	272	14	MOLECULE:	CYSTEINE PROTEASE S273R
38:00:00	2bkq-B	9.8	3.6	143	208	13	MOLECULE:	SENTRIN-SPECIFIC PROTEASE 8
39:00:00	8efw-B	9.7	4	153	183	12	MOLECULE:	SDEA
40:00:00	6fdu-A	9.6	3.5	139	240	16	MOLECULE:	DEUBIQUITINASE AND DENEDDYLASE
41:00:00	6fdk-A	9.6	3.5	139	242	16	MOLECULE:	DEUBIQUITINASE AND DENEDDYLASE
42:00:00	5crb-A	9.5	3.7	148	192	10	MOLECULE:	SDEA

43:00:00	5b5q-A	9.5	3.4	139	243	14	MOLECULE:	MEMBRANE THIOL PROTEASE
44:00:00	6oam-B	9.5	3.3	141	239	14	MOLECULE:	DEUBIQUITINASE AND DENEDDYLASE
45:00:00	6fdu-B	9.5	3.4	137	240	13	MOLECULE:	DEUBIQUITINASE AND DENEDDYLASE
46:00:00	6fdq-A	9.5	3.4	138	239	14	MOLECULE:	DEUBIQUITINASE AND DENEDDYLASE
47:00:00	6ljb-A	9.5	6.1	138	267	14	MOLECULE:	CYSTEINE PROTEASE S273R
48:00:00	8oi3-A	9.3	2.9	123	183	10	MOLECULE:	TYPE III EFFECTOR
49:00:00	6lj9-B	9.3	6.1	130	259	15	MOLECULE:	CYSTEINE PROTEASE S273R
50:00:00	6fdq-B	9.3	3.4	140	247	16	MOLECULE:	DEUBIQUITINASE AND DENEDDYLASE
51:00:00	5jp3-E	9.3	3	133	193	11	MOLECULE:	XANTHOMONAS OUTER PROTEIN D
52:00:00	5klq-B	9.2	3.8	153	320	12	MOLECULE:	ORF34
53:00:00	5klq-C	9.2	3.7	152	320	11	MOLECULE:	ORF34
54:00:00	6oam-A	9.2	3.3	141	239	14	MOLECULE:	DEUBIQUITINASE AND DENEDDYLASE
55:00:00	5klp-A	9.2	4	153	309	10	MOLECULE:	ORF34
56:00:00	2bkq-D	9.1	3.6	138	209	12	MOLECULE:	SENTRIN-SPECIFIC PROTEASE 8
57:00:00	6gzs-A	9.1	3.5	142	250	13	MOLECULE:	DEUBIQUITINASE AND DENEDDYLASE
58:00:00	5klq-A	9.1	3.8	153	324	12	MOLECULE:	ORF34
59:00:00	5jp3-C	9.1	3	130	196	11	MOLECULE:	XANTHOMONAS OUTER PROTEIN D
60:00:00	6mrn-A	9.1	3.4	139	240	14	MOLECULE:	DEUBIQUITINASE AND DENEDDYLASE
61:00:00	5klp-B	9.1	4	152	310	11	MOLECULE:	ORF34
62:00:00	6gzt-A	9	8.1	152	267	13	MOLECULE:	DEUBIQUITINASE AND DENEDDYLASE
63:00:00	6gzu-A	9	2.9	130	241	14	MOLECULE:	CONSERVED MEMBRANE PROTEIN
64:00:00	5jp1-A	9	3.1	129	199	12	MOLECULE:	XANTHOMONAS OUTER PROTEIN D
65:00:00	5hag-A	9	7.5	150	266	13	MOLECULE:	DEUBIQUITINASE AND DENEDDYLASE
66:00:00	5klp-C	9	4	155	315	10	MOLECULE:	ORF34
67:00:00	8oi3-B	9	3	125	183	10	MOLECULE:	TYPE III EFFECTOR
68:00:00	2oix-A	9	2.8	122	178	11	MOLECULE:	XANTHOMONAS OUTER PROTEIN D
69:00:00	5aek-A	8.9	3.5	129	223	10	MOLECULE:	SENTRIN-SPECIFIC PROTEASE 2
70:00:00	5jp3-G	8.9	3	129	196	12	MOLECULE:	XANTHOMONAS OUTER PROTEIN D
71:00:00	5jp3-A	8.7	3	129	205	11	MOLECULE:	XANTHOMONAS OUTER PROTEIN D
72:00:00	7r2e-B	8.7	4.5	144	243	12	MOLECULE:	SENTRIN-SPECIFIC PROTEASE 7
73:00:00	2g4d-C	8.7	3.3	127	205	8	MOLECULE:	SENP1 PROTEIN
74:00:00	5dgg-B	8.7	3.3	125	176	15	MOLECULE:	UNCHARACTERIZED PROTEIN
75:00:00	7r2e-A	8.7	4.5	148	244	11	MOLECULE:	SENTRIN-SPECIFIC PROTEASE 7
76:00:00	5aek-M	8.7	3.5	129	222	11	MOLECULE:	SENTRIN-SPECIFIC PROTEASE 2
77:00:00	2g4d-A	8.7	3.3	128	205	8	MOLECULE:	SENP1 PROTEIN
78:00:00	3eay-A	8.6	4.4	146	247	12	MOLECULE:	SENTRIN-SPECIFIC PROTEASE 7
79:00:00	1th0-B	8.6	3.4	129	223	12	MOLECULE:	SENTRIN-SPECIFIC PROTEASE 2
80:00:00	2hl9-A	8.6	3.4	133	218	11	MOLECULE:	UBIQUITIN-LIKE-SPECIFIC PROTEA
81:00:00	2hkp-A	8.6	3.3	133	219	11	MOLECULE:	UBIQUITIN-LIKE-SPECIFIC PROTEA
82:00:00	2hl8-A	8.5	3.4	128	219	12	MOLECULE:	UBIQUITIN-LIKE-SPECIFIC PROTEA
83:00:00	1euv-A	8.5	3.4	133	221	13	MOLECULE:	ULP1 PROTEASE
84:00:00	5aek-K	8.4	3.2	127	223	9	MOLECULE:	SENTRIN-SPECIFIC PROTEASE 2
85:00:00	5aek-U	8.4	3.3	126	223	10	MOLECULE:	SENTRIN-SPECIFIC PROTEASE 2
86:00:00	5lnb-B	8.4	4.7	152	259	9	MOLECULE:	UBIQUITIN-LIKE-SPECIFIC PROTEA

87:00:00	5dgg-A	8.4	2.8	119	176	15	MOLECULE:	UNCHARACTERIZED PROTEIN
88:00:00	2iy1-A	8.4	3.3	128	226	8	MOLECULE:	SENTRIN-SPECIFIC PROTEASE 1
89:00:00	2xph-A	8.4	3.5	130	227	9	MOLECULE:	SENTRIN-SPECIFIC PROTEASE 1
90:00:00	6nnq-A	8.4	3.3	128	224	9	MOLECULE:	SENTRIN-SPECIFIC PROTEASE 1
91:00:00	5w40-D	8.3	3.9	145	327	12	MOLECULE:	POPP2 PROTEIN
92:00:00	5aek-C	8.3	3.5	129	224	10	MOLECULE:	SENTRIN-SPECIFIC PROTEASE 2
93:00:00	2ckh-A	8.3	3.5	128	225	9	MOLECULE:	SENTRIN-SPECIFIC PROTEASE 1
94:00:00	2iyc-A	8.3	3.4	128	226	9	MOLECULE:	SENTRIN-SPECIFIC PROTEASE 1
95:00:00	5w3y-C	8.3	3.8	143	335	12	MOLECULE:	POPP2 PROTEIN
96:00:00	2ckg-B	8.3	3.5	130	225	9	MOLECULE:	SENTRIN-SPECIFIC PROTEASE 1
97:00:00	2ckg-A	8.3	3.4	128	225	9	MOLECULE:	SENTRIN-SPECIFIC PROTEASE 1
98:00:00	2iy0-A	8.3	3.4	128	226	8	MOLECULE:	SENTRIN-SPECIFIC PROTEASE 1
99:00:00	2iyc-B	8.3	3.4	128	226	9	MOLECULE:	SENTRIN-SPECIFIC PROTEASE 1
0:00	5aek-G	8.3	3.3	128	223	9	MOLECULE:	SENTRIN-SPECIFIC PROTEASE 2
1:00	5aek-S	8.3	3.3	128	224	9	MOLECULE:	SENTRIN-SPECIFIC PROTEASE 2
2:00	1th0-A	8.2	3.4	129	226	12	MOLECULE:	SENTRIN-SPECIFIC PROTEASE 2
3:00	1tgz-A	8.2	3.5	129	224	11	MOLECULE:	SENTRIN-SPECIFIC PROTEASE 2
4:00	5aek-W	8.2	3.5	131	222	11	MOLECULE:	SENTRIN-SPECIFIC PROTEASE 2
5:00	5aek-I	8.2	3.4	128	223	9	MOLECULE:	SENTRIN-SPECIFIC PROTEASE 2
6:00	2iyd-A	8.2	3.5	128	226	9	MOLECULE:	SENTRIN-SPECIFIC PROTEASE 1
7:00	5w3t-B	8.2	3.7	141	333	13	MOLECULE:	POPP2 PROTEIN
8:00	5aek-O	8.2	3.4	128	223	11	MOLECULE:	SENTRIN-SPECIFIC PROTEASE 2
9:00	2iy1-C	8.2	3.4	125	226	7	MOLECULE:	SENTRIN-SPECIFIC PROTEASE 1
10:00	5w40-B	8.2	3.8	142	333	12	MOLECULE:	POPP2 PROTEIN
11:00	5aek-E	8.2	3.5	131	224	11	MOLECULE:	SENTRIN-SPECIFIC PROTEASE 2
12:00	2oiv-A	8.2	2.8	116	170	11	MOLECULE:	XANTHOMONAS OUTER PROTEIN D
13:00	2xre-A	8.2	3.4	128	227	9	MOLECULE:	SENTRIN-SPECIFIC PROTEASE 1
14:00	2xph-B	8.1	3.6	128	226	9	MOLECULE:	SENTRIN-SPECIFIC PROTEASE 1
15:00	2io1-A	8.1	3.3	128	223	9	MOLECULE:	SENTRIN-SPECIFIC PROTEASE 2
16:00	2xre-B	8	3.6	128	225	9	MOLECULE:	SENTRIN-SPECIFIC PROTEASE 1
17:00	2io1-C	8	3.5	127	224	11	MOLECULE:	SENTRIN-SPECIFIC PROTEASE 2
18:00	6dg4-A	8	3.5	131	253	11	MOLECULE:	ULP1-LIKE SUMO PROTEASE
19:00	2io1-E	8	3.4	131	223	11	MOLECULE:	SENTRIN-SPECIFIC PROTEASE 2
20:00	5ms8-A	7.9	6.3	165	377	13	MOLECULE:	LEGIONELLA PNEUMOPHILA EFFECTO
21:00	2io2-A	7.9	3.3	127	225	9	MOLECULE:	SENTRIN-SPECIFIC PROTEASE 2
22:00	2io3-A	7.9	3.3	129	224	9	MOLECULE:	SENTRIN-SPECIFIC PROTEASE 2
23:00	3zo5-A	7.9	3.4	131	230	10	MOLECULE:	SENTRIN-SPECIFIC PROTEASE 2
24:00:00	5ms2-A	7.8	7	160	405	14	MOLECULE:	LEGIONELLA PNEUMOPHILA EFFECTO
25:00:00	5io3-A	7.8	7.1	158	385	11	MOLECULE:	UNCHARACTERIZED PROTEIN RAVZ
26:00:00	5w3y-B	7.8	3.9	147	327	10	MOLECULE:	POPP2 PROTEIN
27:00:00	5aek-Q	7.8	3.3	127	224	9	MOLECULE:	SENTRIN-SPECIFIC PROTEASE 2
28:00:00	5izv-A	7.8	6.8	165	376	12	MOLECULE:	UNCHARACTERIZED PROTEIN RAVZ
29:00:00	5w3y-D	7.8	3.9	145	334	12	MOLECULE:	POPP2 PROTEIN
30:00:00	5w40-A	7.8	3.9	146	336	12	MOLECULE:	POPP2 PROTEIN

31:00:00	5w3x-A	7.7	4	146	335	13	MOLECULE:	POPP2 PROTEIN
32:00:00	5w3t-D	7.7	3.7	142	333	13	MOLECULE:	POPP2 PROTEIN
33:00:00	5w3t-A	7.7	3.9	141	332	13	MOLECULE:	POPP2 PROTEIN
34:00:00	5w3t-C	7.7	3.6	141	332	13	MOLECULE:	POPP2 PROTEIN
35:00:00	5w40-C	7.7	3.8	140	333	12	MOLECULE:	POPP2 PROTEIN
36:00:00	5w3x-C	7.7	3.9	144	342	13	MOLECULE:	POPP2 PROTEIN
37:00:00	5izv-B	7.6	6.6	165	369	13	MOLECULE:	UNCHARACTERIZED PROTEIN RAVZ
38:00:00	5w3y-A	7.6	3.9	141	336	12	MOLECULE:	POPP2 PROTEIN
39:00:00	2io0-A	7.6	3.4	127	226	9	MOLECULE:	SENTRIN-SPECIFIC PROTEASE 2
40:00:00	5hzy-A	7.4	6.5	159	380	13	MOLECULE:	UNCHARACTERIZED PROTEIN RAVZ
41:00:00	5cqc-A	7.4	6.3	150	352	12	MOLECULE:	PUTATIVE RAVZ PROTEIN
42:00:00	5ms7-A	7.3	6.8	160	370	13	MOLECULE:	LEGIONELLA PNEUMOPHILA EFFECTO
43:00:00	7f3n-A	6.8	3.8	125	247	14	MOLECULE:	TYPE III EFFECTOR PROTEIN POPP
44:00:00	5crc-A	5.4	6.1	99	152	10	MOLECULE:	SDEA
45:00:00	5crc-B	5.4 1	0.2	106	155	9	MOLECULE:	SDEA
46:00:00	6w9s-A	5.3	8.1	106	178	8	MOLECULE:	OTU DOMAIN-CONTAINING PROTEIN
47:00:00	4g38-A	5	5	103	449	8	MOLECULE:	SULFITE REDUCTASE [NADPH] HEMO
48:00:00	5gep-A	4.8 1	2.3	107	456	7	MOLECULE:	SULFITE REDUCTASE HEMOPROTEIN
49:00:00	6gep-A	4.8 1	2.3	107	456	7	MOLECULE:	SULFITE REDUCTASE HEMOPROTEIN
50:00:00	4gep-A	4.7 1	2.2	108	456	6	MOLECULE:	SULFITE REDUCTASE HEMOPROTEIN
51:00:00	6c3z-A	4.7 1	2.3	107	462	7	MOLECULE:	SULFITE REDUCTASE [NADPH] HEMO
52:00:00	3aop-A	4.6 1	2.3	107	453	7	MOLECULE:	SULFITE REDUCTASE HEMOPROTEIN
53:00:00	7gep-A	4.6 1	2.2	108	469	6	MOLECULE:	SULFITE REDUCTASE HEMOPROTEIN
54:00:00	4htr-A	4.6	5	104	441	8	MOLECULE:	SULFITE REDUCTASE [NADPH] HEMO
55:00:00	5aop-A	4.6	4.9	101	456	6	MOLECULE:	SULFITE REDUCTASE HEMOPROTEIN
56:00:00	6c3x-A	4.5 1	2.2	108	464	6	MOLECULE:	SULFITE REDUCTASE [NADPH] HEMO
57:00:00	8gep-A	4.5	5.1	104	456	8	MOLECULE:	SULFITE REDUCTASE HEMOPROTEIN
58:00:00	4aop-A	4.4 1	2.2	108	453	6	MOLECULE:	SULFITE REDUCTASE HEMOPROTEIN
59:00:00	5h92-A	4.4 1	3.7	105	567	11	MOLECULE:	SULFITE REDUCTASE [FERREDOXIN]
60:00:00	3geo-A	4.4 1	2.2	107	455	7	MOLECULE:	SULFITE REDUCTASE HEMOPROTEIN
61:00:00	2aop-A	4.4 1	2.3	107	456	7	MOLECULE:	SULFITE REDUCTASE HEMOPROTEIN
62:00:00	4g39-A	4.4 1	2.2	108	465	5	MOLECULE:	SULFITE REDUCTASE [NADPH] HEMO
63:00:00	5h92-B	4.3 1	3.8	106	572	11	MOLECULE:	SULFITE REDUCTASE [FERREDOXIN]
64:00:00	6c3m-A	4.3 1	2.3	107	465	7	MOLECULE:	SULFITE REDUCTASE [NADPH] HEMO
65:00:00	5h8y-C	4.3 1	4.6	107	563	11	MOLECULE:	SULFITE REDUCTASE [FERREDOXIN]
66:00:00	2gep-A	4.2 1	2.2	107	472	5	MOLECULE:	SULFITE REDUCTASE HEMOPROTEIN
67:00:00	5h8v-B	4.1 1	3.6	106	560	11	MOLECULE:	SULFITE REDUCTASE [FERREDOXIN]
68:00:00	1zj8-B	4.1	5.2	103	546	4	MOLECULE:	PROBABLE FERREDOXIN-DEPENDENT
69:00:00	5h8v-A	4.1 1	3.7	109	564	10	MOLECULE:	SULFITE REDUCTASE [FERREDOXIN]
70:00:00	1aop-A	4.1	5	103	456	6	MOLECULE:	SULFITE REDUCTASE HEMOPROTEIN
71:00:00	7xql-A	4	5.3	159	444	11	MOLECULE:	ANKYRIN REPEAT-CONTAINING PROT
72:00:00	5h8y-D	4	5.2	105	554	12	MOLECULE:	SULFITE REDUCTASE [FERREDOXIN]
73:00:00	5h8y-A	4.0 1	3.7	109	567	8	MOLECULE:	SULFITE REDUCTASE [FERREDOXIN]
74:00:00	1zj9-A	3.8 1	0.4	110	546	5	MOLECULE:	PROBABLE FERREDOXIN-DEPENDENT

75:00:00	1zj9-B	3.8	1	0.3	110	546	5	MOLECULE:	PROBABLE FERREDOXIN-DEPENDENT
76:00:00	3b0n-A	3.7	1	3.6	106	538	8	MOLECULE:	NITRITE REDUCTASE
77:00:00	3vm0-A	3.7	1	3.7	107	538	7	MOLECULE:	NITRITE REDUCTASE
78:00:00	6mca-A	3.6	1	1.8	148	460	12	MOLECULE:	ANKYRIN REPEAT DOMAIN PROTEIN
79:00:00	3vkr-A	3.6	1	1.7	107	538	10	MOLECULE:	NITRITE REDUCTASE
80:00:00	3vks-A	3.6	1	4.1	112	538	9	MOLECULE:	NITRITE REDUCTASE
81:00:00	3b0m-A	3.6	1	2.9	107	538	8	MOLECULE:	NITRITE REDUCTASE
82:00:00	3vlz-A	3.6	1	2.9	107	538	9	MOLECULE:	NITRITE REDUCTASE
83:00:00	5h8y-B	3.5	1	3.1	110	565	12	MOLECULE:	SULFITE REDUCTASE [FERREDOXIN]
84:00:00	3b0l-A	3.3	1	2.5	106	538	10	MOLECULE:	NITRITE REDUCTASE
85:00:00	4kqe-A	3.2	1	7.2	93	597	5	MOLECULE:	GLYCINE--TRNA LIGASE
86:00:00	3vkt-A	3.1	1	6.9	98	538	5	MOLECULE:	NITRITE REDUCTASE
87:00:00	2akj-A	3.1	1	2.5	97	535	6	MOLECULE:	FERREDOXIN--NITRITE REDUCTASE,
88:00:00	5e6m-A	3.0	1	7.1	95	520	6	MOLECULE:	GLYCINE--TRNA LIGASE
89:00:00	3b0j-A	3.0	1	1.7	107	538	9	MOLECULE:	NITRITE REDUCTASE
90:00:00	3vkp-A	3.0	1	3.7	107	538	7	MOLECULE:	NITRITE REDUCTASE
91:00:00	3b0h-A	3.0	1	3.6	95	536	6	MOLECULE:	NITRITE REDUCTASE
92:00:00	3b0g-A	3.0	1	4.8	94	538	5	MOLECULE:	NITRITE REDUCTASE
93:00:00	3vlx-A	3.0	1	2.5	95	538	6	MOLECULE:	NITRITE REDUCTASE
94:00:00	3b0h-B	3.0	1	3.1	83	535	7	MOLECULE:	NITRITE REDUCTASE
95:00:00	3vly-A	3.0	1	2.5	95	538	6	MOLECULE:	NITRITE REDUCTASE
96:00:00	3vkq-A	3.0	1	2.7	93	538	6	MOLECULE:	NITRITE REDUCTASE
97:00:00	1zj8-A	2.9		5.2	103	546	4	MOLECULE:	PROBABLE FERREDOXIN-DEPENDENT
98:00:00	2zt7-A	2.9	1	3.3	91	531	5	MOLECULE:	GLYCYL-TRNA SYNTHETASE
99:00:00	3vm1-A	2.9	1	4.4	95	538	5	MOLECULE:	NITRITE REDUCTASE
0:00	6c3y-A	2.9	1	1.9	107	464	7	MOLECULE:	SULFITE REDUCTASE [NADPH] HEMO
1:00	5uto-A	2.8		3.6	95	288	4	MOLECULE:	EDD DOMAIN PROTEIN, DEGV FAMIL
2:00	2eo6-A	2.6		3.7	85	141	7	MOLECULE:	B-CELL LINKER PROTEIN
3:00	7xrm-B	2.6		3.7	100	253	8	MOLECULE:	ETHANOLAMINE AMMONIA-LYASE LAR
4:00	2j3l-B	2.6	1	8.4	82	571	13	MOLECULE:	PROLYL-TRNA SYNTHETASE

DALI results: MdA-1 Prim

No:	Chain	Z	rmsd l	al	i nres	%id	PDB Description
1:00	5iok-A	2.9	3.5	61	142	10	MOLECULE: TRANSCRIPTION
2:00	6miq-A	2.5	3.4	61	141	10	MOLECULE: TRANSCRIPTION
3:00	5d7e-A	2.5	3.5	61	140	10	MOLECULE: TRANSCRIPTION
4:00	7egm-H	2.4	5.6	66	388	9	MOLECULE: TRANSCRIPTION
5:00	6mip-A	2.3	4.2	62	139	10	MOLECULE: TRANSCRIPTION
6:00	6min-A	2.3	3.6	61	139	10	MOLECULE: TRANSCRIPTION
7:00	2hcz-X	2.2	4.3	66	242	3	MOLECULE: BETA-EXPANSIN
8:00	7f5m-A	2.1	3.5	61	139	11	MOLECULE: SOMETHING ABO
9:00	6ans-B	2.0	4.5	54	369	6	MOLECULE: UNCHARACTERIZ
10:00	6mio-A	2.0	3.4	56	142	7	MOLECULE: TRANSCRIPTION

DALI results: MdA-1 E1

No:	Chain	Z	rmsd	lali	nres	%id	PDB	Description
1:00	7apd-B	19.8	3.4	268	289	21	MOLECULE:	REPLICATION PROTEIN E1
2:00	1tue-K	17	2.4	185	192	22	MOLECULE:	REPLICATION PROTEIN E1
3:00	4gdf-A	16.5	5.7	334	497	16	MOLECULE:	LARGE T ANTIGEN
4:00	9bc5-F	11.1	4.3	157	480	19	MOLECULE:	PROTEIN REP68
5:00	7apd-G	10.9	3.9	140	152	9	MOLECULE:	REPLICATION PROTEIN E1
6:00	6k9c-A	10.7	6.7	230	416	14	MOLECULE:	PRIMASE
7:00	4fb3-A	10.3	2.6	112	115	9	MOLECULE:	ORI DNA OLIGONUCLEOTIDE-CRICK ST
8:00	1r9w-A	9.5	3.4	124	138	11	MOLECULE:	REPLICATION PROTEIN E1
9:00	7ola-A	9.4	6.7	217	441	13	MOLECULE:	DNA PRIMASE
10:00	8wgy-E	9.3	6	259	769	8	MOLECULE:	UNCOATING FACTOR OPG117
11:00	8iqi-C	9	4.3	262	916	11	MOLECULE:	PUTATIVE PRIMASE C962R
12:00	3dkx-A	8	10.3	119	202	8	MOLECULE:	REPLICATION PROTEIN REPB
13:00	7mi8-A	6.8	4.4	191	868	8	MOLECULE:	FUSION PROTEIN OF DYNEIN AND END
14:00	7tts-D	6.8	13.6	177	463	11	MOLECULE:	CASEINOLYTIC PEPTIDASE B PROTEIN
15:00	4p6q-A	6.6	10.9	121	286	6	MOLECULE:	MSX2-INTERACTING PROTEIN
16:00	5nvu-A	6.4	16.3	197	3169	0	MOLECULE:	DYNEIN MOTOR DOMAIN
17:00	3bos-B	6.4	3.6	136	231	13	MOLECULE:	PUTATIVE DNA REPLICATION FACTOR
18:00	7fd4-A	6.3	15	235	779	10	MOLECULE:	LON PROTEASE
19:00	3j3r-A	6.2	11.5	216	798	11	MOLECULE:	ADAPTER PROTEIN MECA 1
20:00	5udb-4	6	8.3	227	751	12	MOLECULE:	DNA REPLICATION LICENSING FACTOR
21:00	5axk-B	5.9	3.9	107	239	6	MOLECULE:	TRNA(HIS)-5'-GUANYLYLTRANSFERASE
22:00	3nbx-X	5.8	4.1	173	481	9	MOLECULE:	ATPASE RAVA
23:00	8umy-A	5.8	21.9	161	546	14	MOLECULE:	CHROMOSOME TRANSMISSION FIDELITY
24:00:00	7w42-B	5.7	16.2	171	402	15	MOLECULE:	UNCHARACTERIZED ATPASE YJOB
25:00:00	8c0v-B	5.7	12.2	213	1030	8	MOLECULE:	PEROXISOMAL ATPASE PEX1
26:00:00	5nug-A	5.7	22.2	214	2920	8	MOLECULE:	CYTOPLASMIC DYNEIN 1 HEAVY CHAIN
27:00:00	8xku-B	5.7	12.3	186	845	13	MOLECULE:	PROBABLE INACTIVE ATP-DEPENDENT
28:00:00	8osg-B	5.7	4.4	171	323	9	MOLECULE:	MAGNESIUM-CHELATASE SUBUNIT CHLI
29:00:00	4rh7-A	5.6	22.9	222	3005	11	MOLECULE:	GREEN FLUORESCENT PROTEIN/CYTOPL
30:00:00	2hiy-D	5.6	3	82	183	11	MOLECULE:	HYPOTHETICAL PROTEIN
31:00:00	3u5z-B	5.6	11	138	320	17	MOLECULE:	DNA POLYMERASE ACCESSORY PROTEIN
32:00:00	8dr5-A	5.5	12	154	646	11	MOLECULE:	REPLICATION FACTOR C SUBUNIT 1
33:00:00	6f74-A	5.5	14.6	132	572	4	MOLECULE:	ALCOHOL OXIDASE
34:00:00	5m7o-A	5.5	14.5	162	448	12	MOLECULE:	NITROGEN ASSIMILATION REGULATORY
35:00:00	7b8s-C	5.5	5.4	104	589	3	MOLECULE:	MULTIDRUG EFFLUX PUMP SUBUNIT AC
36:00:00	5dgk-A	5.5	7.1	201	519	9	MOLECULE:	ACTIVE HELICASE
37:00:00	7uqj-A	5.4	5.8	157	585	11	MOLECULE:	ATPASE HISTONE CHAPERONE YTA7
38:00:00	7vcs-A	5.4	9.1	210	766	11	MOLECULE:	TRANSITIONAL ENDOPLASMIC RETICUL
39:00:00	8k8v-A	5.3	14.3	155	719	12	MOLECULE:	ENDOPEPTIDASE LA
40:00:00	7qo4-M	5.3	10.4	183	421	13	MOLECULE:	26S PROTEASOME REGULATORY SUBUNI
41:00:00	5udb-7	5.1	5.8	203	726	12	MOLECULE:	DNA REPLICATION LICENSING FACTOR
42:00:00	6fhs-C	5.1	15.4	149	459	12	MOLECULE:	RUVB-LIKE HELICASE

43:00:00	3tht-B	5.1	15.3	118	312	5	MOLECULE:	ALKYLATED DNA REPAIR PROTEIN ALK
44:00:00	7ykk-A	5.1	9.2	199	735	10	MOLECULE:	ATPASE FAMILY GENE 2 PROTEIN
45:00:00	6uen-A	5	16.2	195	1392	6	MOLECULE:	RNA-DIRECTED RNA POLYMERASE L
46:00:00	3vf0-B	5	5.3	105	283	4	MOLECULE:	VINCULIN
47:00:00	2qby-B	5	7	137	368	9	MOLECULE:	CELL DIVISION CONTROL PROTEIN 6
48:00:00	3f8t-A	4.9	5.5	185	459	9	MOLECULE:	PREDICTED ATPASE INVOLVED IN REP
49:00:00	8xks-B	4.9	34.3	144	603	13	MOLECULE:	CTAP1
50:00:00	6dgd-A	4.8	10.7	153	704	12	MOLECULE:	PRIMOSOMAL PROTEIN N'
51:00:00	3wbz-E	4.8	4.5	115	268	4	MOLECULE:	LIKELY HISTIDYL TRNA-SPECIFIC GU
52:00:00	5yww-A	4.8	13.7	175	485	11	MOLECULE:	NUCLEOTIDE BINDING PROTEIN PINC
53:00:00	5zr1-A	4.8	17.9	168	494	8	MOLECULE:	ORIGIN RECOGNITION COMPLEX SUBUN
54:00:00	7crc-B	4.8	13.1	186	1112	10	MOLECULE:	NAD+ HYDROLASE (NADASE)
55:00:00	7mjy-A	4.7	15.9	121	449	10	MOLECULE:	TRNA-2-METHYLTHIO-N(6)-DIMETHYLA
56:00:00	8fwj-A	4.7	10.1	140	552	11	MOLECULE:	CIRCADIAN CLOCK PROTEIN KAIC
57:00:00	3k1j-A	4.7	12.2	177	566	8	MOLECULE:	ATP-DEPENDENT PROTEASE LON
58:00:00	8c0v-A	4.7	11.9	186	823	6	MOLECULE:	PEROXISOMAL ATPASE PEX1
59:00:00	8jx6-B	4.6	11.1	122	451	9	MOLECULE:	DEEP-SEA HELICASE 9
60:00:00	7qh2-C	4.5	12.2	137	467	12	MOLECULE:	LACTATE DEHYDROGENASE (NAD(+),FE
61:00:00	8sk1-A	4.5	3.3	91	162	11	MOLECULE:	2-AMINO-4-HYDROXY-6-HYDROXYMETHY
62:00:00	6dhs-B	4.5	6.8	93	176	4	MOLECULE:	HETEROGENEOUS NUCLEAR RIBONUCLEO
63:00:00	6fjw-A	4.5	3.4	84	239	10	MOLECULE:	CAS6 PROTEIN
64:00:00	6f7s-C	4.4	13.1	131	319	3	MOLECULE:	SERRATE RNA EFFECTOR MOLECULE HO
65:00:00	7dex-A	4.4	9.2	80	460	4	MOLECULE:	ANTHOCYANIN 5-AROMATIC ACYLTRANS
66:00:00	7t8b-A	4.4	8	170	518	9	MOLECULE:	TWINKLE MTDNA HELICASE
67:00:00	5e7p-A	4.4	18.6	208	719	11	MOLECULE:	CELL DIVISION CONTROL PROTEIN CD
68:00:00	5aor-A	4.4	18.1	167	1009	12	MOLECULE:	DOSAGE COMPENSATION REGULATOR
69:00:00	4kw3-A	4.4	3.5	105	266	5	MOLECULE:	NS1
70:00:00	8emh-B	4.4	4.8	198	676	8	MOLECULE:	PROTEASE LON-RELATED BREX SYSTEM
71:00:00	4gwp-F	4.3	3.5	81	209	4	MOLECULE:	MEDIATOR OF RNA POLYMERASE II TR
72:00:00	6v55-A	4.3	21.7	156	746	6	MOLECULE:	ADHESION G-PROTEIN COUPLED RECEP
73:00:00	5che-A	4.3	23.6	120	427	5	MOLECULE:	GLUTAMYL-TRNA REDUCTASE 1, CHLOR
74:00:00	5jpq-i	4.3	7.2	120	659	13	MOLECULE:	WD40 DOMAIN PROTEINS
75:00:00	8tdl-A	4.3	23.5	118	364	8	MOLECULE:	MECHANOSENSITIVE ION CHANNEL PRO
76:00:00	4ksr-A	4.3	16.5	153	521	12	MOLECULE:	TYPE II SECRETION SYSTEM PROTEIN
77:00:00	8tzk-C	4.3	22.4	118	304	6	MOLECULE:	CELL DIVISION ATP-BINDING PROTEI
78:00:00	8xku-D	4.3	23.8	216	805	9	MOLECULE:	PROBABLE INACTIVE ATP-DEPENDENT
79:00:00	8fcv-R	4.3	8.2	142	343	8	MOLECULE:	DNA (60-MER)
80:00:00	5wx8-A	4.2	3.8	89	165	8	MOLECULE:	IMMEDIATE-EARLY PROTEIN 2
81:00:00	1vr6-A	4.2	14.7	109	343	7	MOLECULE:	PHOSPHO-2-DEHYDRO-3-DEOXYHEPTONA
82:00:00	1m1g-C	4.2	7.6	101	244	10	MOLECULE:	TRANSCRIPTION ANTITERMINATION PR
83:00:00	6o16-A	4.2	15.7	140	798	11	MOLECULE:	DEAH (ASP-GLU-ALA-HIS) BOX POLYP
84:00:00	8k4i-A	4.1	2.8	68	163	1	MOLECULE:	YAJQ
85:00:00	8y6p-Q	4.1	13.6	143	1228	11	MOLECULE:	APAF-1 RELATED KILLER DARK
86:00:00	4lgt-A	4.1	16.6	98	251	5	MOLECULE:	RIBOSOMAL LARGE SUBUNIT PSEUDOUR

87:00:00	4lya-A	4.1	4.7	132	521	8	MOLECULE:	UNCHARACTERIZED PROTEIN
88:00:00	3cmu-A	4.1	10.4	143	1937	13	MOLECULE:	DNA (5'-
89:00:00	6ted-Z	4	5.4	131	510	5	MOLECULE:	DNA-DIRECTED RNA POLYMERASE SUBU
90:00:00	6hyp-A	4	7.9	171	2272	8	MOLECULE:	MIDASIN,MIDASIN
91:00:00	3c19-A	4	12	99	177	7	MOLECULE:	UNCHARACTERIZED PROTEIN MK0293
92:00:00	2qv6-A	4	11.3	89	253	7	MOLECULE:	GTP CYCLOHYDROLASE III
93:00:00	4m9s-B	4	14.3	154	511	12	MOLECULE:	CELL DEATH PROTEIN 4
94:00:00	8yha-B	4	3.5	84	268	7	MOLECULE:	CRISPR SYSTEM CASCADE SUBUNIT CA
95:00:00	6mfv-A	4	7.7	125	641	11	MOLECULE:	TETRATRICOPEPTIDE REPEAT SENSOR
96:00:00	3mtj-A	4	9.7	113	431	12	MOLECULE:	HOMOSERINE DEHYDROGENASE
97:00:00	9esh-P	3.9	16.6	110	271	6	MOLECULE:	PRE-MRNA
98:00:00	7v9x-A	3.9	14.7	124	314	10	MOLECULE:	RNA-DIRECTED DNA POLYMERASE FROM
99:00:00	8th8-L	3.9	11.7	174	862	8	MOLECULE:	DYNEIN REGULATORY COMPLEX PROTEI
0:00	1ahu-A	3.9	13.8	127	555	12	MOLECULE:	VANILLYL-ALCOHOL OXIDASE
1:00	5o6b-A	3.9	3.8	119	528	13	MOLECULE:	ATP-DEPENDENT DNA HELICASE PIF1
2:00	3d5l-A	3.8	4.1	58	203	12	MOLECULE:	REGULATORY PROTEIN RECX
3:00	7oky-Z	3.8	7.8	145	374	7	MOLECULE:	DNA-DIRECTED RNA POLYMERASE II S
4:00	7epu-B	3.8	16	134	748	10	MOLECULE:	NON-IMMUNIZED HUMAN SCFV
5:00	6d7y-B	3.7	2.7	79	155	9	MOLECULE:	HEMAGGLUTININ
6:00	4nh0-A	3.7	4.2	134	861	10	MOLECULE:	CELL DIVISIONFTSK/SPOIIIE
7:00	4znm-B	3.7	12.6	91	444	10	MOLECULE:	C-DOMAIN TYPE II PEPTIDE SYNTHET
8:00	8peu-A	3.7	9	164	419	12	MOLECULE:	TRANSCRIPTION TERMINATION FACTOR
9:00	5k8o-B	3.7	12.2	104	425	11	MOLECULE:	5-NITROANTHRANILIC ACID AMINOHYD
10:00	6pwn-A	3.7	18.7	113	280	4	MOLECULE:	SMALL-CONDUCTANCE MECHANOSENSITI
11:00	8x5i-A	3.7	13.4	146	477	12	MOLECULE:	ENDONUCLEASE GAJA
12:00	3zqj-F	3.7	16.5	126	607	20	MOLECULE:	UVRABC SYSTEM PROTEIN A
13:00	3crv-A	3.7	9.2	126	551	13	MOLECULE:	XPD/RAD3 RELATED DNA HELICASE
14:00	4d2i-A	3.6	8.7	130	464	8	MOLECULE:	HERA
15:00	3gp8-A	3.6	18.3	129	551	14	MOLECULE:	EXODEOXYRIBONUCLEASE V, SUBUNIT
16:00	2iai-A	3.6	3.5	71	200	7	MOLECULE:	PUTATIVE TRANSCRIPTIONAL REGULAT
17:00	7sgr-A	3.6	13.8	168	700	11	MOLECULE:	ALPHA-HEMOLYSIN TRANSLOCATION AT
18:00	7t5q-I	3.6	22.3	90	283	9	MOLECULE:	ACTIN-RELATED PROTEIN 3
19:00	9fzy-F	3.6	13.4	121	442	5	MOLECULE:	CORRINOID IRON-SULFUR PROTEIN LA
20:00	2nyi-A	3.6	10.8	92	171	7	MOLECULE:	UNKNOWN PROTEIN
21:00	3al0-B	3.6	20.2	115	482	10	MOLECULE:	GLUTAMYL-TRNA(GLN) AMIDOTRANSFER
22:00	2pfd-A	3.6	23.5	119	540	3	MOLECULE:	FORMIMIDOYLTRANSFERASE-CYCLODEAM
23:00	8oz7-A	3.6	13	151	597	6	MOLECULE:	ABIA
24:00:00	8dgf-A	3.6	12.8	188	1541	8	MOLECULE:	ATP-BINDING PROTEIN AVS4
25:00:00	7t5q-J	3.6	30.4	106	291	7	MOLECULE:	ACTIN-RELATED PROTEIN 3
26:00:00	4a15-A	3.6	10.7	118	596	12	MOLECULE:	ATP-DEPENDENT DNA HELICASE TA005
27:00:00	8xks-E	3.6	10.9	198	1458	10	MOLECULE:	CTAP1
28:00:00	7cww-A	3.6	6.8	116	350	10	MOLECULE:	TSRE
29:00:00	7pkq-g	3.5	3.4	70	132	7	MOLECULE:	MS35
30:00:00	5n9j-Y	3.5	4.5	89	185	7	MOLECULE:	MEDIATOR OF RNA POLYMERASE II TR

31:00:00	8uy1-A	3.5	4.8	115	598	2	MOLECULE:	METHYLENETETRAHYDROFOLATE REDUCT
32:00:00	5ks8-F	3.5	11.9	137	482	8	MOLECULE:	PYRUVATE CARBOXYLASE SUBUNIT ALP
33:00:00	5ijn-F	3.5	17.8	118	335	8	MOLECULE:	NUCLEAR PORE COMPLEX PROTEIN NUP
34:00:00	2cg8-C	3.5	8.5	96	248	11	MOLECULE:	DIHYDRONEOPTERIN ALDOLASE 6-HYDR
35:00:00	6fon-A	3.5	7.2	78	252	5	MOLECULE:	COPPER CHAPERONE FOR SUPEROXIDE
36:00:00	6zyd-A	3.5	12.6	99	323	11	MOLECULE:	LOW CONDUCTANCE MECHANOSENSITIVE
37:00:00	3tuz-C	3.5	5.5	121	345	10	MOLECULE:	D-METHIONINE TRANSPORT SYSTEM PE
38:00:00	8fvu-A	3.5	10.6	181	1361	10	MOLECULE:	BACULOVIRAL IAP REPEAT-CONTAININ
39:00:00	6bwo-A	3.5	5.2	98	144	3	MOLECULE:	PYRIDINIUM-3,5-BISTHIOCARBOXYLIC
40:00:00	7wrx-C	3.5	11.5	139	594	9	MOLECULE:	HERA
41:00:00	1wp9-A	3.4	14.4	131	479	13	MOLECULE:	ATP-DEPENDENT RNA HELICASE, PUTA
42:00:00	6pif-G	3.4	16.7	108	521	6	MOLECULE:	CAS7, TYPE I-F CRISPR-ASSOCIATED
43:00:00	7njn-C	3.4	12.9	186	536	6	MOLECULE:	ATP SYNTHASE SUBUNIT ALPHA
44:00:00	9io5-J	3.4	6.9	186	597	7	MOLECULE:	G1-ATPASE SUBUNIT BETA
45:00:00	8vx9-B	3.4	20.4	170	1174	10	MOLECULE:	HAMA
46:00:00	6pe4-A	3.4	5.8	88	758	3	MOLECULE:	V-TYPE PROTON ATPASE SUBUNIT A,
47:00:00	5gko-A	3.4	10.9	122	650	11	MOLECULE:	MACROLIDE EXPORT ATP-BINDING/PER
48:00:00	6umm-A	3.4	16.2	121	285	4	MOLECULE:	ESX-3 SECRETION SYSTEM PROTEIN E
49:00:00	7elb-A	3.4	5.1	171	1937	3	MOLECULE:	RNA-DIRECTED RNA POLYMERASE L
50:00:00	6rfl-Y	3.4	4.2	131	600	11	MOLECULE:	DNA-DEPENDENT RNA POLYMERASE SUB
51:00:00	8qvt-A	3.4	12	127	514	11	MOLECULE:	CYTOKININ DEHYDROGENASE
52:00:00	2xgj-B	3.3	8.5	126	773	6	MOLECULE:	ATP-DEPENDENT RNA HELICASE DOB1
53:00:00	6ekk-B	3.3	6.5	137	381	12	MOLECULE:	DENN DOMAIN-CONTAINING PROTEIN 1
54:00:00	5okc-B	3.3	14.4	105	377	6	MOLECULE:	SISTER CHROMATID COHESION PROTEI
55:00:00	3pfo-A	3.3	14.2	115	426	10	MOLECULE:	PUTATIVE ACETYLORNITHINE DEACETY
56:00:00	6ner-E	3.3	3.8	89	186	6	MOLECULE:	BMC-H TANDEM FUSION PROTEIN
57:00:00	5m86-A	3.3	6.7	111	321	6	MOLECULE:	TA1207
58:00:00	5a9j-A	3.3	4.4	133	805	14	MOLECULE:	DNA POLYMERASE THETA
59:00:00	7r9x-A	3.3	12.5	82	418	12	MOLECULE:	AMBE
60:00:00	3onq-B	3.2	16.5	104	255	8	MOLECULE:	REGULATOR OF POLYKETIDE SYNTHASE
61:00:00	8hra-C	3.2	14	141	852	9	MOLECULE:	ARCHAEAL ATPASE
62:00:00	7ou2-B	3.2	12.4	170	650	8	MOLECULE:	DNA MISMATCH REPAIR PROTEIN MUTS
63:00:00	2oca-A	3.2	9.5	134	494	6	MOLECULE:	ATP-DEPENDENT DNA HELICASE UVSW
64:00:00	6jyt-A	3.2	21.2	143	597	10	MOLECULE:	HELICASE
65:00:00	3c6k-D	3.2	14.3	92	348	13	MOLECULE:	SPERMINE SYNTHASE
66:00:00	9go3-A	3.2	19.2	116	253	7	MOLECULE:	MECHANOSENSITIVE CHANNEL PROTEIN
67:00:00	7mi1-A	3.2	16.1	107	2628	7	MOLECULE:	CHIMERA PROTEIN OF DYNEIN AND EN
68:00:00	5zxd-B	3.2	13.3	116	491	16	MOLECULE:	ATP-BINDING CASSETTE SUB-FAMILY
69:00:00	6d9m-A	3.2	13	103	326	6	MOLECULE:	FUSION PROTEIN OF ENDOLYSIN,RESP
70:00:00	1yoz-B	3.2	2.8	55	116	4	MOLECULE:	HYPOTHETICAL PROTEIN AF0941
71:00:00	9cvc-A	3.2	16.8	98	570	8	MOLECULE:	CODANIN-1
72:00:00	8k0b-A	3.1	19.5	143	689	2	MOLECULE:	CALCIUM PERMEABLE STRESS-GATED C
73:00:00	6nmi-A	3.1	19.2	145	653	10	MOLECULE:	GENERAL TRANSCRIPTION AND DNA RE
74:00:00	6njy-A	3.1	7.4	85	237	4	MOLECULE:	TYPE IV CRISPR ASSOCIATED CAS6 R

75:00:00	8jqb-E	3.1	5.7	114	499	9	MOLECULE:	ENDONUCLEASE GAJA
76:00:00	5z79-C	3.1	11	96	599	10	MOLECULE:	HYDROXYMETHYLDIHYDROPTERIN PYROP
77:00:00	7tmr-F	3.1	6.7	151	478	9	MOLECULE:	H(+)-TRANSPORTING TWO-SECTOR ATP
78:00:00	6iy8-A	3.1	10.8	96	469	7	MOLECULE:	POSITIVE REGULATOR CAPR
79:00:00	6xf9-D	3.1	10	94	426	6	MOLECULE:	PACKAGING PROTEIN UL32
80:00:00	3pyb-B	3.1	4.9	97	689	7	MOLECULE:	ENT-COPALYL DIPHOSPHATE SYNTHASE
81:00:00	3dew-A	3.1	3.5	77	192	3	MOLECULE:	TRANSCRIPTIONAL REGULATOR, TETR
82:00:00	4wd9-A	3.1	16.5	85	965	7	MOLECULE:	NISIN BIOSYNTHESIS PROTEIN NISB
83:00:00	4qbu-A	3.1	6	107	436	8	MOLECULE:	ZMAA
84:00:00	2pgc-A	3	7.6	102	206	12	MOLECULE:	UNCHARACTERIZED PROTEIN
85:00:00	8wtk-A	3	11.9	118	496	14	MOLECULE:	CRISPR-ASSOCIATED HELICASE CAS3
86:00:00	7rgd-A	3	10.2	142	519	9	MOLECULE:	ISOFORM 5 OF INOSINE-5'-MONOPHOS
87:00:00	6urt-A	3	13.8	99	331	8	MOLECULE:	LOW CONDUCTANCE MECHANOSENSITIVE
88:00:00	1lfp-A	3	4.9	94	243	6	MOLECULE:	HYPOTHETICAL PROTEIN AQ_1575
89:00:00	1yb3-A	3	5.5	85	166	8	MOLECULE:	HYPOTHETICAL PROTEIN
90:00:00	3a1y-G	3	19.4	101	211	10	MOLECULE:	50S RIBOSOMAL PROTEIN P1 (L12P)
91:00:00	4j8s-A	3	11.3	66	180	9	MOLECULE:	CCR4-NOT TRANSCRIPTION COMPLEX S
92:00:00	8xj6-D	3	15.9	132	589	11	MOLECULE:	MONKEYPOX VIRUS E5
93:00:00	4cej-B	2.9	15.7	146	1156	8	MOLECULE:	ATP-DEPENDENT HELICASE/NUCLEASE
94:00:00	8phk-P	2.9	4.2	85	162	7	MOLECULE:	TRANSCRIPTION ANTITERMINATION PR
95:00:00	9gm7-C	2.9	8.5	50	440	2	MOLECULE:	CHROMOSOME PARTITION PROTEIN MUK
96:00:00	5egf-C	2.9	16.3	92	444	10	MOLECULE:	TQAA
97:00:00	8s7v-K	2.9	14.6	119	521	15	MOLECULE:	METHYL-COENZYME M REDUCTASE SUBU
98:00:00	7z3h-A	2.9	11.7	91	372	4	MOLECULE:	TRANSFERASE-LIKE PROTEIN
99:00:00	3on2-B	2.9	3.5	76	195	7	MOLECULE:	PROBABLE TRANSCRIPTIONAL REGULAT
0:00	3gmg-A	2.8	7	84	143	2	MOLECULE:	UNCHARACTERIZED PROTEIN RV1825/M
1:00	5dy0-A	2.8	3.1	73	220	3	MOLECULE:	TETR FAMILY TRANSCRIPTIONAL REGU
2:00	6zca-H	2.8	17.9	153	771	9	MOLECULE:	PROBABLE DNA-DIRECTED RNA POLYME
3:00	8j0s-d	2.8	13.3	74	445	8	MOLECULE:	ATP SYNTHASE SUBUNIT A
4:00	2opl-B	2.7	8.7	75	182	3	MOLECULE:	HYPOTHETICAL PROTEIN
5:00	5jb3-H	2.7	3.2	69	214	9	MOLECULE:	16S RIBOSOMAL RNA
6:00	7c0q-A	2.7	3.2	57	279	7	MOLECULE:	EFFECTOR LPG2505
7:00	7yqh-A	2.6	4.1	125	1069	11	MOLECULE:	STRUCTURAL MAINTENANCE OF CHROMO
8:00	8xku-A	2.6	9.8	112	730	12	MOLECULE:	PROBABLE INACTIVE ATP-DEPENDENT
9:00	9gm6-B	2.6	4	111	1148	10	MOLECULE:	CHROMOSOME PARTITION PROTEIN MUK
10:00	7f52-A	2.6	19.5	78	584	13	MOLECULE:	NON-STRUCTURAL PROTEIN 2
11:00	2k5s-A	2.6	4.3	45	73	7	MOLECULE:	MODULATING PROTEIN YMOA
12:00	7e7i-A	2.6	13	147	2011	9	MOLECULE:	RETINAL-SPECIFIC PHOSPHOLIPID-TR
13:00	6w2r-B	2.6	9	58	221	3	MOLECULE:	JUNCTION 19 DHR54-DHR79
14:00	4x4r-A	2.5	13.3	100	443	9	MOLECULE:	CCA-ADDING ENZYME
15:00	3j5s-D	2.5	13.7	117	554	9	MOLECULE:	16S RIBOSOMAL RNA
16:00	7roq-A	2.5	12.9	154	1831	10	MOLECULE:	PHOSPHOLIPID-TRANSPORTING ATPASE
17:00	8wcn-A	2.5	11.8	82	379	5	MOLECULE:	DIGUANYLATE CYCLASE
18:00	7pde-A	2.5	21.9	122	846	6	MOLECULE:	ADENYLATE CYCLASE 9

19:00	7nyw-B	2.5	3.9	110	858	10	MOLECULE:	CHROMOSOME PARTITION PROTEIN MUK
20:00	8s36-M	2.5	12	124	519	10	MOLECULE:	CRISPR TYPE AFERR-ASSOCIATED PRO
21:00	1k8w-A	2.5	3.5	81	303	10	MOLECULE:	5'-R(*GP*GP*CP*AP*AP*CP*GP*GP*UP
22:00	7b0c-A	2.4	3.4	57	144	7	MOLECULE:	HTH-TYPE TRANSCRIPTIONAL REPRESS
23:00	7sjr-A	2.4	17.4	136	911	13	MOLECULE:	DNA HELICASE
24:00:00	4krr-A	2.4	3	65	194	6	MOLECULE:	WNT INHIBITOR OF DORSAL PROTEIN
25:00:00	2hxi-B	2.4	2.4	59	198	10	MOLECULE:	PUTATIVE TRANSCRIPTIONAL REGULAT
26:00:00	8f4x-0	2.4	3.2	43	67	19	MOLECULE:	RC_I_1-H11
27:00:00	7q5b-B	2.4	9.1	101	482	6	MOLECULE:	DNA (56-MER)
28:00:00	6r4p-A	2.4	13.7	119	478	5	MOLECULE:	ADENYLATE CYCLASE 9
29:00:00	5opt-h	2.4	10.8	74	173	8	MOLECULE:	ACTIVATED PROTEIN KINASE C RECEP
30:00:00	2lc2-A	2.3	3.2	47	108	9	MOLECULE:	AVR3A4
31:00:00	7lcc-A	2.2	25.2	110	1369	10	MOLECULE:	HELRAISER K1068Q
32:00:00	8xku-C	2.1	16.6	105	921	13	MOLECULE:	PROBABLE INACTIVE ATP-DEPENDENT

DALI results: MdA-1 RepE1

No:	Chain	Z	rmsd	lali	nres	%id	PDB	Description
1:00	2gxa-I	16.9	4.1	261	270	21	MOLECULE:	5'-D(*TP*TP*TP*TP*TP*TP*TP*TP*TP*TP*TP*TP*T)-3'
2:00	2v9p-D	16.9	3.8	254	274	21	MOLECULE:	REPLICATION PROTEIN E1
3:00	5a9k-D	16.8	3.8	254	274	21	MOLECULE:	REPLICATION PROTEIN E1
4:00	2gxa-G	16.8	3.8	251	270	21	MOLECULE:	5'-D(*TP*TP*TP*TP*TP*TP*TP*TP*TP*TP*TP*TP*T)-3'
5:00	2gxa-D	16.8	3.9	261	270	20	MOLECULE:	5'-D(*TP*TP*TP*TP*TP*TP*TP*TP*TP*TP*TP*TP*T)-3'
6:00	2v9p-J	16.7	3.9	256	272	21	MOLECULE:	REPLICATION PROTEIN E1
7:00	2v9p-I	16.7	3.5	238	270	21	MOLECULE:	REPLICATION PROTEIN E1
8:00	2gxa-B	16.7	4	258	270	21	MOLECULE:	5'-D(*TP*TP*TP*TP*TP*TP*TP*TP*TP*TP*TP*TP*T)-3'
9:00	2v9p-H	16.7	3.5	237	268	22	MOLECULE:	REPLICATION PROTEIN E1
10:00	2gxa-C	16.6	3.8	245	270	21	MOLECULE:	5'-D(*TP*TP*TP*TP*TP*TP*TP*TP*TP*TP*TP*TP*T)-3'
11:00	2gxa-E	16.6	4.2	262	274	21	MOLECULE:	5'-D(*TP*TP*TP*TP*TP*TP*TP*TP*TP*TP*TP*TP*T)-3'
12:00	2gxa-J	16.6	4.1	249	270	20	MOLECULE:	5'-D(*TP*TP*TP*TP*TP*TP*TP*TP*TP*TP*TP*TP*T)-3'
13:00	2v9p-B	16.6	3.5	237	268	22	MOLECULE:	REPLICATION PROTEIN E1
14:00	2gxa-F	16.5	4.2	257	270	21	MOLECULE:	5'-D(*TP*TP*TP*TP*TP*TP*TP*TP*TP*TP*TP*TP*T)-3'
15:00	2gxa-H	16.5	4.1	248	274	20	MOLECULE:	5'-D(*TP*TP*TP*TP*TP*TP*TP*TP*TP*TP*TP*TP*T)-3'
16:00	5a9k-B	16.5	3.5	236	268	21	MOLECULE:	REPLICATION PROTEIN E1
17:00	5a9k-C	16.5	3.5	236	267	22	MOLECULE:	REPLICATION PROTEIN E1
18:00	2v9p-C	16.5	3.5	236	267	22	MOLECULE:	REPLICATION PROTEIN E1
19:00	2v9p-G	16.4	3.9	254	270	21	MOLECULE:	REPLICATION PROTEIN E1
20:00	2v9p-E	16.3	3.7	255	270	22	MOLECULE:	REPLICATION PROTEIN E1
21:00	7apd-E	16.3	4.2	268	287	20	MOLECULE:	REPLICATION PROTEIN E1
22:00	5a9k-E	16.3	3.7	255	270	22	MOLECULE:	REPLICATION PROTEIN E1
23:00	2gxa-K	16.3	4.2	266	273	21	MOLECULE:	5'-D(*TP*TP*TP*TP*TP*TP*TP*TP*TP*TP*TP*TP*T)-3'
24:00:00	5a9k-A	16.3	4	256	272	21	MOLECULE:	REPLICATION PROTEIN E1
25:00:00	2v9p-A	16.3	3.9	240	272	22	MOLECULE:	REPLICATION PROTEIN E1
26:00:00	2v9p-K	16.2	3.6	253	269	22	MOLECULE:	REPLICATION PROTEIN E1
27:00:00	2h1l-K	16.1	5.4	272	362	17	MOLECULE:	LARGE T ANTIGEN
28:00:00	2gxa-A	16	4.6	261	270	20	MOLECULE:	5'-D(*TP*TP*TP*TP*TP*TP*TP*TP*TP*TP*TP*TP*T)-3'
29:00:00	1svm-F	16	5.4	275	363	17	MOLECULE:	LARGE T ANTIGEN
30:00:00	2v9p-F	15.9	4	253	269	21	MOLECULE:	REPLICATION PROTEIN E1
31:00:00	2h1l-E	15.9	5.2	269	362	17	MOLECULE:	LARGE T ANTIGEN
32:00:00	1svm-E	15.9	5.1	268	362	17	MOLECULE:	LARGE T ANTIGEN
33:00:00	2h1l-L	15.9	5.5	273	363	17	MOLECULE:	LARGE T ANTIGEN
34:00:00	1svm-C	15.9	5.4	274	363	17	MOLECULE:	LARGE T ANTIGEN
35:00:00	2h1l-C	15.9	5.5	274	363	17	MOLECULE:	LARGE T ANTIGEN
36:00:00	1svm-B	15.8	5.2	268	362	17	MOLECULE:	LARGE T ANTIGEN
37:00:00	2h1l-F	15.8	5.4	273	363	17	MOLECULE:	LARGE T ANTIGEN
38:00:00	1svm-A	15.7	5.4	273	362	17	MOLECULE:	LARGE T ANTIGEN
39:00:00	2h1l-D	15.7	5.4	270	362	17	MOLECULE:	LARGE T ANTIGEN
40:00:00	2h1l-B	15.7	5.5	272	362	17	MOLECULE:	LARGE T ANTIGEN
41:00:00	5j4y-A	15.7	4.8	258	363	16	MOLECULE:	LARGE T ANTIGEN
42:00:00	7apd-B	15.6	3.4	270	289	21	MOLECULE:	REPLICATION PROTEIN E1
43:00:00	2h1l-J	15.6	5.1	266	362	17	MOLECULE:	LARGE T ANTIGEN
44:00:00	2v9p-L	15.6	4.1	251	267	21	MOLECULE:	REPLICATION PROTEIN E1
45:00:00	2h1l-H	15.6	5.4	273	362	17	MOLECULE:	LARGE T ANTIGEN
46:00:00	7apd-D	15.6	3.9	270	287	20	MOLECULE:	REPLICATION PROTEIN E1
47:00:00	4e2i-J	15.5	5.3	270	362	17	MOLECULE:	LARGE T ANTIGEN
48:00:00	4e2i-H	15.5	5.3	270	362	17	MOLECULE:	LARGE T ANTIGEN
49:00:00	7apd-C	15.5	3.8	271	287	20	MOLECULE:	REPLICATION PROTEIN E1
50:00:00	4gdf-E	15.5	7.2	308	497	16	MOLECULE:	LARGE T ANTIGEN
51:00:00	4e2i-A	15.5	5.3	270	362	17	MOLECULE:	LARGE T ANTIGEN

52:00:00	2h1l-G	15.5	5.3	268	362	18	MOLECULE:	LARGE T ANTIGEN
53:00:00	2h1l-I	15.5	5.4	270	363	17	MOLECULE:	LARGE T ANTIGEN
54:00:00	1svm-D	15.5	5.2	269	362	17	MOLECULE:	LARGE T ANTIGEN
55:00:00	4e2i-E	15.5	5.3	270	362	17	MOLECULE:	LARGE T ANTIGEN
56:00:00	4e2i-G	15.5	5.3	270	362	17	MOLECULE:	LARGE T ANTIGEN
57:00:00	4e2i-D	15.5	5.3	270	362	17	MOLECULE:	LARGE T ANTIGEN
58:00:00	4e2i-K	15.5	5.3	270	362	17	MOLECULE:	LARGE T ANTIGEN
59:00:00	4e2i-I	15.5	5.3	270	362	17	MOLECULE:	LARGE T ANTIGEN
60:00:00	4e2i-L	15.5	5.3	270	362	17	MOLECULE:	LARGE T ANTIGEN
61:00:00	4e2i-F	15.5	5.3	270	362	17	MOLECULE:	LARGE T ANTIGEN
62:00:00	2h1l-A	15.5	5.2	270	362	17	MOLECULE:	LARGE T ANTIGEN
63:00:00	5tct-B	15.5	5.7	277	362	17	MOLECULE:	LARGE T ANTIGEN
64:00:00	7apd-F	15.4	4.5	271	286	20	MOLECULE:	REPLICATION PROTEIN E1
65:00:00	4gdf-A	15.4	7.2	307	497	16	MOLECULE:	LARGE T ANTIGEN
66:00:00	1svo-A	15.4	4.9	253	362	19	MOLECULE:	LARGE T ANTIGEN
67:00:00	1n25-B	15.4	4.8	257	362	18	MOLECULE:	LARGE T ANTIGEN
68:00:00	1svl-C	15.3	5.7	280	362	16	MOLECULE:	LARGE T ANTIGEN
69:00:00	1svl-B	15.3	4.4	252	362	18	MOLECULE:	LARGE T ANTIGEN
70:00:00	1n25-A	15.3	4.7	250	362	18	MOLECULE:	LARGE T ANTIGEN
71:00:00	1svl-A	15.3	5.2	253	363	17	MOLECULE:	LARGE T ANTIGEN
72:00:00	5a9k-F	15.3	4	253	269	21	MOLECULE:	REPLICATION PROTEIN E1
73:00:00	1svo-B	15.3	4.5	252	362	19	MOLECULE:	LARGE T ANTIGEN
74:00:00	5j40-A	15.3	4.7	251	358	16	MOLECULE:	LARGE T ANTIGEN
75:00:00	5tct-C	15.2	5.2	260	362	18	MOLECULE:	LARGE T ANTIGEN
76:00:00	5tct-F	15.2	4.7	255	362	19	MOLECULE:	LARGE T ANTIGEN
77:00:00	4e2i-C	15.2	5.3	270	362	17	MOLECULE:	LARGE T ANTIGEN
78:00:00	4ij8-B	15.2	2.3	124	151	18	MOLECULE:	N-LYSINE METHYLTRANSFERASE SETD8
79:00:00	4e2i-B	15.1	5.3	270	362	17	MOLECULE:	LARGE T ANTIGEN
80:00:00	5tct-D	15.1	5	261	362	17	MOLECULE:	LARGE T ANTIGEN
81:00:00	1tue-A	15.1	2.7	192	201	21	MOLECULE:	REPLICATION PROTEIN E1
82:00:00	1tue-K	15	2.4	184	192	22	MOLECULE:	REPLICATION PROTEIN E1
83:00:00	3f9z-A	15	2.6	125	157	18	MOLECULE:	HISTONE-LYSINE N-METHYLTRANSFERASE SETD8
84:00:00	3f9y-A	14.9	2.6	124	160	18	MOLECULE:	HISTONE-LYSINE N-METHYLTRANSFERASE SETD8
85:00:00	5teg-B	14.9	2.4	123	159	18	MOLECULE:	N-LYSINE METHYLTRANSFERASE KMT5A
86:00:00	5w1y-B	14.9	2.2	120	151	18	MOLECULE:	N-LYSINE METHYLTRANSFERASE KMT5A
87:00:00	3f9w-B	14.9	2.5	124	161	18	MOLECULE:	HISTONE-LYSINE N-METHYLTRANSFERASE SETD8
88:00:00	5j47-A	14.9	5	258	360	16	MOLECULE:	LARGE T ANTIGEN
89:00:00	5j4v-A	14.9	5.6	264	358	16	MOLECULE:	LARGE T ANTIGEN
90:00:00	3f9y-B	14.9	2.5	123	161	18	MOLECULE:	HISTONE-LYSINE N-METHYLTRANSFERASE SETD8
91:00:00	5tct-A	14.8	5.4	265	362	17	MOLECULE:	LARGE T ANTIGEN
92:00:00	1tue-D	14.8	2.5	191	202	22	MOLECULE:	REPLICATION PROTEIN E1
93:00:00	5tct-E	14.8	5.2	260	362	18	MOLECULE:	LARGE T ANTIGEN
94:00:00	1zkk-D	14.8	2.5	124	162	18	MOLECULE:	HISTONE-LYSINE N-METHYLTRANSFERASE, H4 LYSINE-20
95:00:00	1zkk-B	14.8	2.5	124	162	18	MOLECULE:	HISTONE-LYSINE N-METHYLTRANSFERASE, H4 LYSINE-20
96:00:00	3f9w-D	14.8	2.5	124	162	18	MOLECULE:	HISTONE-LYSINE N-METHYLTRANSFERASE SETD8
97:00:00	3f9z-B	14.8	2.5	123	162	19	MOLECULE:	HISTONE-LYSINE N-METHYLTRANSFERASE SETD8
98:00:00	3f9z-D	14.8	2.5	124	162	19	MOLECULE:	HISTONE-LYSINE N-METHYLTRANSFERASE SETD8
99:00:00	3f9w-A	14.8	2.5	123	161	18	MOLECULE:	HISTONE-LYSINE N-METHYLTRANSFERASE SETD8
0:00	7apd-A	14.7	4.7	271	284	20	MOLECULE:	REPLICATION PROTEIN E1
1:00	1zkk-A	14.7	2.5	123	161	18	MOLECULE:	HISTONE-LYSINE N-METHYLTRANSFERASE, H4 LYSINE-20
2:00	5teg-A	14.7	2.5	123	161	18	MOLECULE:	N-LYSINE METHYLTRANSFERASE KMT5A
3:00	3f9x-B	14.7	2.5	123	161	18	MOLECULE:	HISTONE-LYSINE N-METHYLTRANSFERASE SETD8
4:00	3f9x-D	14.7	2.5	124	162	18	MOLECULE:	HISTONE-LYSINE N-METHYLTRANSFERASE SETD8

5:00	2bqz-A	14.7	2.4	123	162	18	MOLECULE:	SET8 PROTEIN
6:00	3f9x-C	14.7	2.5	123	161	18	MOLECULE:	HISTONE-LYSINE N-METHYLTRANSFERASE SETD8
7:00	1tue-M	14.7	2.5	187	196	21	MOLECULE:	REPLICATION PROTEIN E1
8:00	1tue-H	14.6	2.5	182	191	23	MOLECULE:	REPLICATION PROTEIN E1
9:00	2bqz-E	14.6	2.5	123	162	18	MOLECULE:	SET8 PROTEIN
10:00	3f9w-C	14.6	2.6	124	161	18	MOLECULE:	HISTONE-LYSINE N-METHYLTRANSFERASE SETD8
11:00	5w1y-A	14.6	3	125	162	18	MOLECULE:	N-LYSINE METHYLTRANSFERASE KMT5A
12:00	3f9x-A	14.6	2.5	123	161	18	MOLECULE:	HISTONE-LYSINE N-METHYLTRANSFERASE SETD8
13:00	1zkk-C	14.5	2.6	124	161	18	MOLECULE:	HISTONE-LYSINE N-METHYLTRANSFERASE, H4 LYSINE-20
14:00	3f9z-C	14.5	2.6	124	161	19	MOLECULE:	HISTONE-LYSINE N-METHYLTRANSFERASE SETD8
15:00	5hq2-M	14.4	2.4	123	160	18	MOLECULE:	HISTONE H3.2
16:00	4ij8-A	14.2	2.4	124	162	18	MOLECULE:	N-LYSINE METHYLTRANSFERASE SETD8
17:00	6boz-B	14.1	2.3	116	134	19	MOLECULE:	N-LYSINE METHYLTRANSFERASE KMT5A
18:00	2gxa-L	14	3.7	225	270	20	MOLECULE:	5'-D(*TP*TP*TP*TP*TP*TP*TP*TP*TP*TP*TP*TP*T)-3'
19:00	7d20-K	14	2.5	124	166	18	MOLECULE:	HISTONE H3-LIKE CENTROMERIC PROTEIN A
20:00	5t5g-A	14	2.4	118	143	19	MOLECULE:	N-LYSINE METHYLTRANSFERASE KMT5A
21:00	7u5v-A	14	2.4	111	125	16	MOLECULE:	HISTONE-LYSINE N-METHYLTRANSFERASE 2A
22:00	1tue-F	14	2.4	178	188	22	MOLECULE:	REPLICATION PROTEIN E1
23:00	4ldg-A	14	2	126	216	17	MOLECULE:	PROTEIN WITH A SET DOMAIN WITHIN CARBOXY REGION
24:00:00	4z4p-A	13.9	2.2	119	162	24	MOLECULE:	HISTONE-LYSINE N-METHYLTRANSFERASE 2D
25:00:00	5v2n-A	13.9	2.3	123	161	18	MOLECULE:	N-LYSINE METHYLTRANSFERASE KMT5A
26:00:00	6boz-A	13.9	2.8	120	139	18	MOLECULE:	N-LYSINE METHYLTRANSFERASE KMT5A
27:00:00	6u9m-A	13.8	2.3	118	148	17	MOLECULE:	HISTONE-LYSINE N-METHYLTRANSFERASE
28:00:00	6u9r-B	13.8	2.3	118	148	17	MOLECULE:	HISTONE-LYSINE N-METHYLTRANSFERASE
29:00:00	6u9n-B	13.8	2.3	118	147	17	MOLECULE:	HISTONE-LYSINE N-METHYLTRANSFERASE
30:00:00	6u9r-A	13.8	2.4	119	149	17	MOLECULE:	HISTONE-LYSINE N-METHYLTRANSFERASE
31:00:00	6u9k-B	13.7	2.3	118	149	17	MOLECULE:	HISTONE-LYSINE N-METHYLTRANSFERASE
32:00:00	7w6l-E	13.7	2.3	119	152	20	MOLECULE:	SET1/ASH2 HISTONE METHYLTRANSFERASE COMPLEX SUBUN
33:00:00	6u9n-A	13.7	2.3	117	147	17	MOLECULE:	HISTONE-LYSINE N-METHYLTRANSFERASE
34:00:00	5f6k-C	13.7	2.2	116	151	20	MOLECULE:	SET1/ASH2 HISTONE METHYLTRANSFERASE COMPLEX SUBUN
35:00:00	5f59-A	13.7	2.5	118	149	19	MOLECULE:	HISTONE-LYSINE N-METHYLTRANSFERASE 2C
36:00:00	7w67-C	13.7	4.1	124	158	17	MOLECULE:	SET1/ASH2 HISTONE METHYLTRANSFERASE COMPLEX SUBUN
37:00:00	5f5e-A	13.6	2.3	117	149	17	MOLECULE:	HISTONE-LYSINE N-METHYLTRANSFERASE 2A
38:00:00	6pww-C	13.6	2.3	120	154	17	MOLECULE:	RETINOBLASTOMA-BINDING PROTEIN 5
39:00:00	6u9k-A	13.6	2.5	118	149	18	MOLECULE:	HISTONE-LYSINE N-METHYLTRANSFERASE
40:00:00	7w6i-C	13.5	4.1	122	160	17	MOLECULE:	SET1/ASH2 HISTONE METHYLTRANSFERASE COMPLEX SUBUN
41:00:00	5f6k-E	13.5	2.1	116	149	21	MOLECULE:	SET1/ASH2 HISTONE METHYLTRANSFERASE COMPLEX SUBUN
42:00:00	7w6j-C	13.4	4.1	122	159	17	MOLECULE:	SET1/ASH2 HISTONE METHYLTRANSFERASE COMPLEX SUBUN
43:00:00	6u9m-B	13.4	2.5	117	149	19	MOLECULE:	HISTONE-LYSINE N-METHYLTRANSFERASE
44:00:00	6w5n-C	13.4	2.4	119	153	17	MOLECULE:	RETINOBLASTOMA-BINDING PROTEIN 5
45:00:00	7xpx-K	13.3	2.6	124	168	18	MOLECULE:	HISTONE H3
46:00:00	5th7-B	13.3	2.3	118	144	19	MOLECULE:	N-LYSINE METHYLTRANSFERASE KMT5A
47:00:00	6pwv-C	13.3	2.4	119	155	17	MOLECULE:	RETINOBLASTOMA-BINDING PROTEIN 5
48:00:00	7w6l-C	13.3	3.2	124	159	19	MOLECULE:	SET1/ASH2 HISTONE METHYLTRANSFERASE COMPLEX SUBUN
49:00:00	6w5i-C	13.3	2.3	116	152	17	MOLECULE:	RETINOBLASTOMA-BINDING PROTEIN 5
50:00:00	7mbn-C	13.3	2.4	119	153	17	MOLECULE:	RETINOBLASTOMA-BINDING PROTEIN 5
51:00:00	6w5m-C	13.3	2.2	117	148	17	MOLECULE:	RETINOBLASTOMA-BINDING PROTEIN 5
52:00:00	7w6a-C	13.3	2.4	121	160	17	MOLECULE:	SET1/ASH2 HISTONE METHYLTRANSFERASE COMPLEX SUBUN
53:00:00	5th7-A	13.3	2.3	117	141	19	MOLECULE:	N-LYSINE METHYLTRANSFERASE KMT5A
54:00:00	5f6l-A	13.2	2.5	120	154	17	MOLECULE:	RETINOBLASTOMA-BINDING PROTEIN 5
55:00:00	7d1z-K	13.1	2.5	124	166	18	MOLECULE:	HISTONE H3.1
56:00:00	1s9h-A	13.1	5.2	206	268	16	MOLECULE:	REP 40 PROTEIN
57:00:00	2w5y-A	13	2.2	118	171	17	MOLECULE:	HISTONE-LYSINE N-METHYLTRANSFERASE HRX

58:00:00	7bre-B	13	2.4	121	164	15	MOLECULE:	SET1/ASH2 HISTONE METHYLTRANSFERASE COMPLEX SUBUN
59:00:00	5ht6-A	12.9	2.6	110	123	19	MOLECULE:	HISTONE-LYSINE N-METHYLTRANSFERASE 2E
60:00:00	1s9h-B	12.9	4.3	203	267	16	MOLECULE:	REP 40 PROTEIN
61:00:00	6chg-C	12.8	2.1	114	144	18	MOLECULE:	KLLA0E24487P
62:00:00	7bre-E	12.8	2.4	120	164	15	MOLECULE:	SET1/ASH2 HISTONE METHYLTRANSFERASE COMPLEX SUBUN
63:00:00	4gdf-B	12.6	12.6	208	497	17	MOLECULE:	LARGE T ANTIGEN
64:00:00	2w5z-A	12.6	2.3	120	181	17	MOLECULE:	HISTONE-LYSINE N-METHYLTRANSFERASE HRX
65:00:00	1u0j-A	12.6	5.1	204	261	14	MOLECULE:	DNA REPLICATION PROTEIN
66:00:00	4gdf-F	12.2	11.7	210	497	18	MOLECULE:	LARGE T ANTIGEN
67:00:00	5ht6-B	12.2	2.5	104	109	20	MOLECULE:	HISTONE-LYSINE N-METHYLTRANSFERASE 2E
68:00:00	6uh5-M	12.1	2.4	114	222	18	MOLECULE:	HISTONE H3
69:00:00	5hyn-Q	12.1	2.3	118	566	19	MOLECULE:	HISTONE-LYSINE N-METHYLTRANSFERASE EZH2
70:00:00	6kqq-B	12	2.5	118	220	20	MOLECULE:	HISTONE-LYSINE N-METHYLTRANSFERASE, H3 LYSINE-36
71:00:00	5ij8-A	12	2.1	116	464	20	MOLECULE:	ENHANCER OF ZESTE HOMOLOG 2 (EZH2),HISTONE-LYSINE
72:00:00	6c24-K	12	2.6	119	325	19	MOLECULE:	POLYCOMB PROTEIN SUZ12
73:00:00	6ven-N	11.9	2.2	112	219	18	MOLECULE:	HISTONE H3.2
74:00:00	7jsg-F	11.9	3.6	221	272	14	MOLECULE:	PROTEIN REP68
75:00:00	6kqp-A	11.7	2.1	114	221	20	MOLECULE:	HISTONE-LYSINE N-METHYLTRANSFERASE, H3 LYSINE-36
76:00:00	4w2r-B	11.7	2.6	120	465	19	MOLECULE:	ENHANCER OF ZESTE 2 POLYCOMB REPRESSIVE COMPLEX 2
77:00:00	7td5-A	11.7	2.3	118	498	19	MOLECULE:	HISTONE-LYSINE N-METHYLTRANSFERASE EZH1
78:00:00	5ij8-B	11.7	2.5	120	466	19	MOLECULE:	ENHANCER OF ZESTE HOMOLOG 2 (EZH2),HISTONE-LYSINE
79:00:00	6b3w-B	11.6	2.2	116	465	20	MOLECULE:	ENHANCER OF ZESTE 2 POLYCOMB REPRESSIVE COMPLEX 2
80:00:00	5ij7-A	11.6	2.1	116	465	20	MOLECULE:	ENHANCER OF ZESTE HOMOLOG 2 (EZH2),HISTONE-LYSINE
81:00:00	7jsi-E	11.5	3.6	226	276	14	MOLECULE:	PROTEIN REP68
82:00:00	5kkl-B	11.5	9	155	838	15	MOLECULE:	PUTATIVE POLYCOMB PROTEIN EED
83:00:00	6cen-A	11.4	2	114	227	19	MOLECULE:	HISTONE-LYSINE N-METHYLTRANSFERASE NSD3
84:00:00	4j7i-A	11.4	2.6	114	244	18	MOLECULE:	HISTONE-LYSINE N-METHYLTRANSFERASE SETD7
85:00:00	1mt6-A	11.4	2.7	116	281	18	MOLECULE:	SET9
86:00:00	5lt8-A	11.4	1.9	113	228	19	MOLECULE:	HISTONE-LYSINE N-METHYLTRANSFERASE SETD2
87:00:00	7td5-F	11.4	2.3	118	448	19	MOLECULE:	HISTONE-LYSINE N-METHYLTRANSFERASE EZH1
88:00:00	7jsf-E	11.4	5.2	217	276	14	MOLECULE:	PROTEIN REP68
89:00:00	7e8d-K	11.4	3.1	119	219	19	MOLECULE:	HISTONE H3.1
90:00:00	1h3i-A	11.3	2.8	116	293	16	MOLECULE:	HISTONE H3 LYSINE 4 SPECIFIC METHYLTRANSFERASE
91:00:00	5m5g-B	11.3	6.7	156	796	18	MOLECULE:	PUTATIVE POLYCOMB PROTEIN EED
92:00:00	7jsg-E	11.3	4	223	272	13	MOLECULE:	PROTEIN REP68
93:00:00	6b3w-A	11.3	2.6	120	463	19	MOLECULE:	ENHANCER OF ZESTE 2 POLYCOMB REPRESSIVE COMPLEX 2
94:00:00	1muf-A	11.3	2.7	116	257	16	MOLECULE:	SET9
95:00:00	7jsh-B	11.3	3.3	224	272	14	MOLECULE:	PROTEIN REP68
96:00:00	5tqr-B	11.2	6.8	160	808	16	MOLECULE:	POLYCOMB PROTEIN EED
97:00:00	5hyn-F	11.2	2.4	118	569	19	MOLECULE:	HISTONE-LYSINE N-METHYLTRANSFERASE EZH2
98:00:00	4ynp-B	11.2	2.2	116	194	17	MOLECULE:	HISTONE-LYSINE N-METHYLTRANSFERASE ASH1L
99:00:00	7jsf-A	11.2	5	213	276	14	MOLECULE:	PROTEIN REP68
0:00	7jsf-C	11.2	5	213	276	14	MOLECULE:	PROTEIN REP68
1:00	7jsf-G	11.2	5	211	276	14	MOLECULE:	PROTEIN REP68
2:00	7jsf-F	11.2	5	213	276	14	MOLECULE:	PROTEIN REP68
3:00	7jsf-B	11.2	5	213	276	14	MOLECULE:	PROTEIN REP68
4:00	7jsg-C	11.2	3.4	214	272	14	MOLECULE:	PROTEIN REP68
5:00	1n6a-A	11.1	2.8	116	235	18	MOLECULE:	SET DOMAIN-CONTAINING PROTEIN 7
6:00	4yz8-A	11.1	2	113	210	19	MOLECULE:	HISTONE-LYSINE N-METHYLTRANSFERASE NSD3
7:00	4ynm-B	11.1	2.2	116	197	17	MOLECULE:	HISTONE-LYSINE N-METHYLTRANSFERASE ASH1L
8:00	4ypu-B	11.1	2.3	116	191	17	MOLECULE:	HISTONE-LYSINE N-METHYLTRANSFERASE ASH1L
9:00	7jsf-D	11.1	5.1	215	276	14	MOLECULE:	PROTEIN REP68
10:00	7jsi-C	11.1	3.5	228	276	14	MOLECULE:	PROTEIN REP68

11:00	1h3i-B	11	2.9	117	293	16	MOLECULE:	HISTONE H3 LYSINE 4 SPECIFIC METHYLTRANSFERASE
12:00	3vuz-A	11	2.5	113	247	19	MOLECULE:	HISTONE-LYSINE N-METHYLTRANSFERASE SETD7
13:00	3m56-A	11	2.7	115	245	18	MOLECULE:	HISTONE-LYSINE N-METHYLTRANSFERASE SETD7
14:00	3cbp-A	11	3	120	240	17	MOLECULE:	HISTONE-LYSINE N-METHYLTRANSFERASE SETD7
15:00	6kqq-A	11	2.5	118	224	20	MOLECULE:	HISTONE-LYSINE N-METHYLTRANSFERASE, H3 LYSINE-36
16:00	5lsu-A	11	2.1	115	232	20	MOLECULE:	HISTONE-LYSINE N-METHYLTRANSFERASE NSD2
17:00	5upd-A	11	2	113	212	19	MOLECULE:	HISTONE-LYSINE N-METHYLTRANSFERASE NSD3
18:00	3ope-B	11	2.2	116	194	17	MOLECULE:	PROBABLE HISTONE-LYSINE N-METHYLTRANSFERASE ASH1L
19:00	6kiz-K	11	3	121	180	17	MOLECULE:	HISTONE H3
20:00	7lzb-A	11	2.8	117	236	20	MOLECULE:	HISTONE-LYSINE N-METHYLTRANSFERASE SETD2
21:00	5wfd-B	11	7.6	156	772	14	MOLECULE:	POLYCOMB PROTEIN EED
22:00	7jsi-B	11	3.5	226	276	14	MOLECULE:	PROTEIN REP68
23:00	3m55-A	10.9	3	120	245	17	MOLECULE:	HISTONE-LYSINE N-METHYLTRANSFERASE SETD7
24:00:00	5ch2-B	10.9	5.4	165	755	16	MOLECULE:	PUTATIVE POLYCOMB PROTEIN EED
25:00:00	4j83-A	10.9	2.6	113	243	18	MOLECULE:	HISTONE-LYSINE N-METHYLTRANSFERASE SETD7
26:00:00	3m54-A	10.9	3	120	246	17	MOLECULE:	HISTONE-LYSINE N-METHYLTRANSFERASE SETD7
27:00:00	3m57-A	10.9	2.6	114	245	18	MOLECULE:	HISTONE-LYSINE N-METHYLTRANSFERASE SETD7
28:00:00	4jds-B	10.9	2.8	117	245	18	MOLECULE:	HISTONE-LYSINE N-METHYLTRANSFERASE SETD7
29:00:00	4j7f-A	10.9	3	119	242	17	MOLECULE:	HISTONE-LYSINE N-METHYLTRANSFERASE SETD7
30:00:00	3m53-A	10.9	2.6	113	247	18	MOLECULE:	HISTONE-LYSINE N-METHYLTRANSFERASE SETD7
31:00:00	3cbo-A	10.9	2.9	118	240	16	MOLECULE:	HISTONE-LYSINE N-METHYLTRANSFERASE SETD7
32:00:00	5ls6-D	10.9	2.2	115	570	20	MOLECULE:	HISTONE-LYSINE N-METHYLTRANSFERASE EZH2,HISTONE-L
33:00:00	5ylt-A	10.9	3.7	123	248	18	MOLECULE:	HISTONE-LYSINE N-METHYLTRANSFERASE SETD7
34:00:00	3cbm-A	10.9	3	120	244	17	MOLECULE:	HISTONE-LYSINE N-METHYLTRANSFERASE SETD7
35:00:00	5wf7-B	10.9	7.5	163	764	15	MOLECULE:	POLYCOMB PROTEIN EED
36:00:00	4ype-B	10.9	2.2	115	203	17	MOLECULE:	HISTONE-LYSINE N-METHYLTRANSFERASE ASH1L
37:00:00	5lsu-B	10.9	2.1	115	223	20	MOLECULE:	HISTONE-LYSINE N-METHYLTRANSFERASE NSD2
38:00:00	5eg2-A	10.9	3	120	244	17	MOLECULE:	HISTONE-LYSINE N-METHYLTRANSFERASE SETD7
39:00:00	4e47-A	10.9	2.9	118	247	18	MOLECULE:	HISTONE-LYSINE N-METHYLTRANSFERASE SETD7
40:00:00	7jsi-D	10.9	3.6	226	276	15	MOLECULE:	PROTEIN REP68
41:00:00	5wg6-A	10.8	6	158	517	16	MOLECULE:	HISTONE-LYSINE N-METHYLTRANSFERASE EZH2,POLYCOMB
42:00:00	8sr6-A	10.8	23.3	159	452	16	MOLECULE:	EUKARYOTIC HUNTINGTIN INTERACTING PROTEIN B
43:00:00	5jlb-A	10.8	2.7	118	249	19	MOLECULE:	HISTONE-LYSINE N-METHYLTRANSFERASE SETD2
44:00:00	4j8o-A	10.8	2.9	118	246	17	MOLECULE:	HISTONE-LYSINE N-METHYLTRANSFERASE SETD7
45:00:00	2f69-A	10.8	3	119	245	17	MOLECULE:	HISTONE-LYSINE N-METHYLTRANSFERASE, H3 LYSINE-4
46:00:00	3m5a-A	10.8	3.1	120	247	17	MOLECULE:	HISTONE-LYSINE N-METHYLTRANSFERASE SETD7
47:00:00	4mi5-A	10.8	2.3	112	208	21	MOLECULE:	HISTONE-LYSINE N-METHYLTRANSFERASE EZH2
48:00:00	4jds-A	10.8	2.4	112	247	19	MOLECULE:	HISTONE-LYSINE N-METHYLTRANSFERASE SETD7
49:00:00	3m59-A	10.8	2.6	114	246	18	MOLECULE:	HISTONE-LYSINE N-METHYLTRANSFERASE SETD7
50:00:00	8fbg-A	10.8	2.1	114	218	20	MOLECULE:	HISTONE-LYSINE N-METHYLTRANSFERASE, H3 LYSINE-36
51:00:00	6ine-A	10.8	2.1	114	248	18	MOLECULE:	HISTONE-LYSINE N-METHYLTRANSFERASE ASH1L
52:00:00	5wfc-B	10.8	7.2	150	768	16	MOLECULE:	POLYCOMB PROTEIN EED
53:00:00	8fyh-G	10.8	2.1	116	514	20	MOLECULE:	POLYCOMB PROTEIN SUZ12
54:00:00	4e47-B	10.8	2.4	112	246	19	MOLECULE:	HISTONE-LYSINE N-METHYLTRANSFERASE SETD7
55:00:00	5ayf-A	10.8	2.8	117	245	16	MOLECULE:	HISTONE-LYSINE N-METHYLTRANSFERASE SETD7
56:00:00	6ago-A	10.8	2.1	116	243	17	MOLECULE:	HISTONE-LYSINE N-METHYLTRANSFERASE ASH1L
57:00:00	3os5-A	10.7	4.1	124	250	18	MOLECULE:	HISTONE-LYSINE N-METHYLTRANSFERASE SETD7
58:00:00	3m58-A	10.7	2.6	114	254	18	MOLECULE:	HISTONE-LYSINE N-METHYLTRANSFERASE SETD7
59:00:00	5ch1-B	10.7	4.3	160	796	16	MOLECULE:	PUTATIVE POLYCOMB PROTEIN EED
60:00:00	5bjs-B	10.7	7.2	156	799	16	MOLECULE:	POLYCOMB PROTEIN EED
61:00:00	4jds-C	10.7	2.4	112	247	20	MOLECULE:	HISTONE-LYSINE N-METHYLTRANSFERASE SETD7
62:00:00	4ypa-B	10.7	2.2	116	213	17	MOLECULE:	HISTONE-LYSINE N-METHYLTRANSFERASE ASH1L
63:00:00	4ypa-D	10.7	2.3	116	212	17	MOLECULE:	HISTONE-LYSINE N-METHYLTRANSFERASE ASH1L

64:00:00	8fbg-B	10.7	2.3	115	219	20	MOLECULE:	HISTONE-LYSINE N-METHYLTRANSFERASE, H3 LYSINE-36
65:00:00	5lsz-A	10.7	2.3	116	243	19	MOLECULE:	HISTONE-LYSINE N-METHYLTRANSFERASE SETD2
66:00:00	6c23-K	10.7	2.5	119	325	19	MOLECULE:	POLYCOMB PROTEIN SUZ12
67:00:00	4e47-C	10.7	2.7	115	246	18	MOLECULE:	HISTONE-LYSINE N-METHYLTRANSFERASE SETD7
68:00:00	7jsh-G	10.7	3.8	224	272	14	MOLECULE:	PROTEIN REP68
69:00:00	1xqh-E	10.6	4	118	251	18	MOLECULE:	HISTONE-LYSINE N-METHYLTRANSFERASE, H3 LYSINE-4
70:00:00	1xqh-A	10.6	4	119	251	19	MOLECULE:	HISTONE-LYSINE N-METHYLTRANSFERASE, H3 LYSINE-4
71:00:00	1o9s-A	10.6	4.2	124	251	18	MOLECULE:	HISTONE-LYSINE N-METHYLTRANSFERASE, H3 LYSINE-4
72:00:00	5kji-B	10.6	6.7	155	762	17	MOLECULE:	PUTATIVE POLYCOMB PROTEIN EED
73:00:00	3ooi-A	10.6	2.3	114	233	20	MOLECULE:	HISTONE-LYSINE N-METHYLTRANSFERASE, H3 LYSINE-36
74:00:00	4jds-D	10.6	2.6	113	246	18	MOLECULE:	HISTONE-LYSINE N-METHYLTRANSFERASE SETD7
75:00:00	4ynm-A	10.6	2.2	116	216	17	MOLECULE:	HISTONE-LYSINE N-METHYLTRANSFERASE ASH1L
76:00:00	3ope-A	10.6	2.2	116	214	17	MOLECULE:	PROBABLE HISTONE-LYSINE N-METHYLTRANSFERASE ASH1L
77:00:00	7jsi-F	10.6	3.6	227	276	13	MOLECULE:	PROTEIN REP68
78:00:00	7jsg-B	10.6	3.7	217	272	15	MOLECULE:	PROTEIN REP68
79:00:00	6kiv-K	10.6	2.5	119	180	18	MOLECULE:	HISTONE H3
80:00:00	1o9s-B	10.6	3.3	121	251	17	MOLECULE:	HISTONE-LYSINE N-METHYLTRANSFERASE, H3 LYSINE-4
81:00:00	6x0p-D	10.6	2.2	117	221	17	MOLECULE:	HISTONE-LYSINE N-METHYLTRANSFERASE ASH1L
82:00:00	6x0p-B	10.6	2.2	117	221	17	MOLECULE:	HISTONE-LYSINE N-METHYLTRANSFERASE ASH1L
83:00:00	1peg-B	10.5	2.7	119	210	18	MOLECULE:	HISTONE H3 METHYLTRANSFERASE DIM-5
84:00:00	4jlg-A	10.5	3.6	121	249	16	MOLECULE:	HISTONE-LYSINE N-METHYLTRANSFERASE SETD7
85:00:00	5h6z-A	10.5	2.7	111	123	13	MOLECULE:	SET DOMAIN-CONTAINING PROTEIN 7
86:00:00	4jlg-B	10.5	3.6	117	248	19	MOLECULE:	HISTONE-LYSINE N-METHYLTRANSFERASE SETD7
87:00:00	4ypa-C	10.5	2.2	116	218	17	MOLECULE:	HISTONE-LYSINE N-METHYLTRANSFERASE ASH1L
88:00:00	4h12-A	10.5	1.9	114	234	19	MOLECULE:	HISTONE-LYSINE N-METHYLTRANSFERASE SETD2
89:00:00	4ynp-A	10.5	2.1	115	214	17	MOLECULE:	HISTONE-LYSINE N-METHYLTRANSFERASE ASH1L
90:00:00	4ypu-A	10.5	2.2	116	215	17	MOLECULE:	HISTONE-LYSINE N-METHYLTRANSFERASE ASH1L
91:00:00	5ww0-A	10.5	2.6	111	123	13	MOLECULE:	SET DOMAIN-CONTAINING PROTEIN 7
92:00:00	6x0p-A	10.5	2.2	116	220	17	MOLECULE:	HISTONE-LYSINE N-METHYLTRANSFERASE ASH1L
93:00:00	7at8-A	10.5	2.4	118	312	19	MOLECULE:	ISOFORM 2 OF HISTONE-LYSINE N-METHYLTRANSFERASE E
94:00:00	4ypa-A	10.5	2.2	116	219	17	MOLECULE:	HISTONE-LYSINE N-METHYLTRANSFERASE ASH1L
95:00:00	4e47-D	10.5	3.1	120	251	17	MOLECULE:	HISTONE-LYSINE N-METHYLTRANSFERASE SETD7
96:00:00	6x0p-C	10.5	2.2	116	221	17	MOLECULE:	HISTONE-LYSINE N-METHYLTRANSFERASE ASH1L
97:00:00	6wzw-A	10.5	2.5	118	222	17	MOLECULE:	HISTONE-LYSINE N-METHYLTRANSFERASE ASH1L
98:00:00	8fbh-A	10.4	2.3	114	233	20	MOLECULE:	HISTONE-LYSINE N-METHYLTRANSFERASE, H3 LYSINE-36
99:00:00	6kiu-K	10.4	2.5	116	182	17	MOLECULE:	HISTONE H3
0:00	7lzd-A	10.4	2.1	116	238	18	MOLECULE:	HISTONE-LYSINE N-METHYLTRANSFERASE SETD2
1:00	5lsx-A	10.4	2	114	231	19	MOLECULE:	HISTONE-LYSINE N-METHYLTRANSFERASE SETD2
2:00	5jle-A	10.4	2.9	118	233	19	MOLECULE:	HISTONE-LYSINE N-METHYLTRANSFERASE SETD2
3:00	4ype-A	10.4	2.2	115	218	17	MOLECULE:	HISTONE-LYSINE N-METHYLTRANSFERASE ASH1L
4:00	5vah-A	10.4	3.1	123	214	15	MOLECULE:	PROBABLE HISTONE-LYSINE N-METHYLTRANSFERASE ATXR5
5:00	5vsc-A	10.4	5.4	120	270	17	MOLECULE:	HISTONE-LYSINE N-METHYLTRANSFERASE EHMT2
6:00	7jsg-G	10.4	4	215	272	13	MOLECULE:	PROTEIN REP68
7:00	5vbc-B	10.4	3.1	123	216	15	MOLECULE:	PROBABLE HISTONE-LYSINE N-METHYLTRANSFERASE ATXR5
8:00	1n6c-A	10.4	2.9	120	284	18	MOLECULE:	SET DOMAIN-CONTAINING PROTEIN 7
9:00	7lzf-A	10.3	2.5	118	240	19	MOLECULE:	HISTONE-LYSINE N-METHYLTRANSFERASE SETD2
10:00	6vdb-A	10.3	2.7	118	245	19	MOLECULE:	HISTONE-LYSINE N-METHYLTRANSFERASE SETD2
11:00	4rz0-B	10.3	2.3	99	110	12	MOLECULE:	PFL0690C
12:00	7ty3-A	10.3	2.3	117	239	19	MOLECULE:	HISTONE-LYSINE N-METHYLTRANSFERASE SETD2
13:00	5va6-B	10.3	3.1	123	216	15	MOLECULE:	PROBABLE HISTONE-LYSINE N-METHYLTRANSFERASE ATXR5
14:00	5vah-B	10.3	3.1	123	215	15	MOLECULE:	PROBABLE HISTONE-LYSINE N-METHYLTRANSFERASE ATXR5
15:00	7ty2-A	10.3	2.3	117	235	18	MOLECULE:	HISTONE-LYSINE N-METHYLTRANSFERASE SETD2
16:00	5vbc-A	10.3	3.1	123	217	15	MOLECULE:	PROBABLE HISTONE-LYSINE N-METHYLTRANSFERASE ATXR5

17:00	4o30-B	10.3	3.1	123	217	15	MOLECULE:	HISTONE-LYSINE N-METHYLTRANSFERASE ATXR6, PUTATIV
18:00	4mi0-A	10.3	2.5	117	208	20	MOLECULE:	HISTONE-LYSINE N-METHYLTRANSFERASE EZH2
19:00	5jjy-A	10.2	2.7	118	248	19	MOLECULE:	HISTONE-LYSINE N-METHYLTRANSFERASE SETD2
20:00	4o30-A	10.2	3.1	124	218	15	MOLECULE:	HISTONE-LYSINE N-METHYLTRANSFERASE ATXR6, PUTATIV
21:00	5lsy-A	10.2	2.3	116	246	19	MOLECULE:	HISTONE-LYSINE N-METHYLTRANSFERASE SETD2
22:00	6j9j-A	10.2	2.7	118	248	19	MOLECULE:	HISTONE-LYSINE N-METHYLTRANSFERASE SETD2
23:00	5v21-A	10.2	2.7	118	246	19	MOLECULE:	HISTONE-LYSINE N-METHYLTRANSFERASE SETD2
24:00:00	5lss-A	10.2	2.6	117	238	19	MOLECULE:	HISTONE-LYSINE N-METHYLTRANSFERASE SETD2
25:00:00	8q5p-A	10.2	2.8	118	246	19	MOLECULE:	HISTONE-LYSINE N-METHYLTRANSFERASE SETD2
26:00:00	5lt7-A	10.2	2.3	116	243	19	MOLECULE:	HISTONE-LYSINE N-METHYLTRANSFERASE SETD2
27:00:00	5vac-A	10.2	3.1	123	216	15	MOLECULE:	PROBABLE HISTONE-LYSINE N-METHYLTRANSFERASE ATXR5
28:00:00	4fmu-A	10.2	2.8	118	236	19	MOLECULE:	HISTONE-LYSINE N-METHYLTRANSFERASE SETD2
29:00:00	5va6-A	10.2	3.1	123	217	15	MOLECULE:	PROBABLE HISTONE-LYSINE N-METHYLTRANSFERASE ATXR5
30:00:00	7xud-A	10.2	2.5	121	269	17	MOLECULE:	HISTONE-LYSINE N-METHYLTRANSFERASE EHMT2
31:00:00	3bo5-A	10.2	3.1	128	270	20	MOLECULE:	HISTONE-LYSINE N-METHYLTRANSFERASE SETMAR
32:00:00	7jsi-A	10.2	3.8	228	276	14	MOLECULE:	PROTEIN REP68
33:00:00	7jsh-E	10.2	3.5	224	272	14	MOLECULE:	PROTEIN REP68
34:00:00	7jsg-D	10.2	3.9	215	272	13	MOLECULE:	PROTEIN REP68
35:00:00	5v22-A	10.1	2.7	118	246	19	MOLECULE:	HISTONE-LYSINE N-METHYLTRANSFERASE SETD2
36:00:00	5kjh-B	10.1	6.6	155	806	17	MOLECULE:	PUTATIVE POLYCOMB PROTEIN EED
37:00:00	6ugm-M	10.1	2.4	112	189	18	MOLECULE:	HISTONE H3
38:00:00	6a5n-A	10.1	2.9	122	502	16	MOLECULE:	HISTONE-LYSINE N-METHYLTRANSFERASE, H3 LYSINE-9 S
39:00:00	5ls6-G	10	2.2	115	572	20	MOLECULE:	HISTONE-LYSINE N-METHYLTRANSFERASE EZH2,HISTONE-L
40:00:00	4w2r-A	10	2.2	116	463	20	MOLECULE:	ENHANCER OF ZESTE 2 POLYCOMB REPRESSIVE COMPLEX 2
41:00:00	5vsc-B	10	5.6	121	273	18	MOLECULE:	HISTONE-LYSINE N-METHYLTRANSFERASE EHMT2
42:00:00	7xub-A	10	2.5	120	271	18	MOLECULE:	HISTONE-LYSINE N-METHYLTRANSFERASE EHMT2
43:00:00	7om0-B	10	7.2	216	439	14	MOLECULE:	DNA PRIMASE
44:00:00	7ola-B	10	7.7	228	441	13	MOLECULE:	DNA PRIMASE
45:00:00	7mbm-C	10	2.3	115	179	19	MOLECULE:	RETINOBLASTOMA-BINDING PROTEIN 5
46:00:00	6kix-K	10	2.4	116	182	19	MOLECULE:	HISTONE H3
47:00:00	5czy-A	9.9	23.4	151	450	19	MOLECULE:	LEGIONELLA EFFECTOR LEGAS4
48:00:00	4rz0-A	9.9	2.7	100	112	12	MOLECULE:	PFL0690C
49:00:00	3mo0-B	9.9	2.2	117	243	19	MOLECULE:	HISTONE-LYSINE N-METHYLTRANSFERASE, H3 LYSINE-9 S
50:00:00	5ij7-B	9.9	2.2	116	469	20	MOLECULE:	ENHANCER OF ZESTE HOMOLOG 2 (EZH2),HISTONE-LYSINE
51:00:00	5vk3-B	9.9	7.6	152	792	16	MOLECULE:	POLYCOMB PROTEIN EED
52:00:00	5lt6-B	9.9	2.3	113	227	19	MOLECULE:	HISTONE-LYSINE N-METHYLTRANSFERASE SETD2
53:00:00	6ago-B	9.9	2.1	112	224	18	MOLECULE:	HISTONE-LYSINE N-METHYLTRANSFERASE ASH1L
54:00:00	5tuz-B	9.9	2.4	116	258	18	MOLECULE:	HISTONE-LYSINE N-METHYLTRANSFERASE EHMT1
55:00:00	7jsg-A	9.9	3.8	214	272	14	MOLECULE:	PROTEIN REP68
56:00:00	8uh7-E	9.9	4.4	127	314	20	MOLECULE:	SLIDING-CLAMP-LOADER SMALL SUBUNIT
57:00:00	7t7m-A	9.9	2.8	121	260	19	MOLECULE:	HISTONE-LYSINE N-METHYLTRANSFERASE EHMT1
58:00:00	7x73-A	9.9	2.5	121	275	17	MOLECULE:	HISTONE-LYSINE N-METHYLTRANSFERASE EHMT2
59:00:00	8xpt-C	9.9	2.5	118	256	18	MOLECULE:	HISTONE-LYSINE N-METHYLTRANSFERASE EHMT1
60:00:00	6wkr-C	9.8	3.1	120	607	21	MOLECULE:	UBIQUITIN
61:00:00	5h6z-B	9.8	2.6	102	120	14	MOLECULE:	SET DOMAIN-CONTAINING PROTEIN 7
62:00:00	3swc-A	9.8	2.5	121	262	18	MOLECULE:	HISTONE-LYSINE N-METHYLTRANSFERASE EHMT1
63:00:00	5lt6-A	9.8	2.3	113	234	19	MOLECULE:	HISTONE-LYSINE N-METHYLTRANSFERASE SETD2
64:00:00	3kmt-C	9.8	2.5	97	120	16	MOLECULE:	A612L PROTEIN
65:00:00	5vsf-A	9.8	2.7	122	262	18	MOLECULE:	HISTONE-LYSINE N-METHYLTRANSFERASE EHMT1
66:00:00	5v9j-B	9.8	2.4	119	260	19	MOLECULE:	HISTONE-LYSINE N-METHYLTRANSFERASE EHMT1
67:00:00	7t7m-C	9.8	2.7	120	260	18	MOLECULE:	HISTONE-LYSINE N-METHYLTRANSFERASE EHMT1
68:00:00	5ttf-B	9.8	2.4	117	268	16	MOLECULE:	HISTONE-LYSINE N-METHYLTRANSFERASE EHMT2
69:00:00	5ttf-D	9.8	2.5	117	262	17	MOLECULE:	HISTONE-LYSINE N-METHYLTRANSFERASE EHMT2

70:00:00	8unh-E	9.8	3.8	132	319	20	MOLECULE:	SLIDING-CLAMP-LOADER LARGE SUBUNIT
71:00:00	6mbp-A	9.8	2.8	122	262	18	MOLECULE:	HISTONE-LYSINE N-METHYLTRANSFERASE EHMT1
72:00:00	3kmt-A	9.7	2.6	97	120	16	MOLECULE:	A612L PROTEIN
73:00:00	5hyn-A	9.7	2.4	118	582	19	MOLECULE:	HISTONE-LYSINE N-METHYLTRANSFERASE EZH2
74:00:00	8swi-A	9.7	23.7	154	452	14	MOLECULE:	EUKARYOTIC HUNTINGTIN INTERACTING PROTEIN B
75:00:00	3kmt-B	9.7	2.5	97	120	16	MOLECULE:	A612L PROTEIN
76:00:00	5ttf-A	9.7	2.5	117	266	17	MOLECULE:	HISTONE-LYSINE N-METHYLTRANSFERASE EHMT2
77:00:00	7x73-B	9.7	2.4	121	274	17	MOLECULE:	HISTONE-LYSINE N-METHYLTRANSFERASE EHMT2
78:00:00	7xud-B	9.7	2.5	121	271	17	MOLECULE:	HISTONE-LYSINE N-METHYLTRANSFERASE EHMT2
79:00:00	7jsh-A	9.7	3.7	221	272	15	MOLECULE:	PROTEIN REP68
80:00:00	7jsh-C	9.7	3.9	216	272	13	MOLECULE:	PROTEIN REP68
81:00:00	7jsh-F	9.7	3.6	228	272	14	MOLECULE:	PROTEIN REP68
82:00:00	3u61-C	9.7	9.5	147	319	18	MOLECULE:	DNA POLYMERASE ACCESSORY PROTEIN 44
83:00:00	8uh7-B	9.7	9.6	139	319	18	MOLECULE:	SLIDING-CLAMP-LOADER SMALL SUBUNIT
84:00:00	3swc-B	9.6	2.7	121	262	18	MOLECULE:	HISTONE-LYSINE N-METHYLTRANSFERASE EHMT1
85:00:00	3sw9-A	9.6	2.7	122	262	18	MOLECULE:	HISTONE-LYSINE N-METHYLTRANSFERASE EHMT1
86:00:00	6a5m-A	9.6	2.9	126	489	15	MOLECULE:	HISTONE-LYSINE N-METHYLTRANSFERASE, H3 LYSINE-9 S
87:00:00	5tuz-A	9.6	2.5	119	260	18	MOLECULE:	HISTONE-LYSINE N-METHYLTRANSFERASE EHMT1
88:00:00	7dcf-A	9.6	2.3	118	261	17	MOLECULE:	HISTONE-LYSINE N-METHYLTRANSFERASE EHMT2
89:00:00	5v9i-A	9.6	2.3	115	266	17	MOLECULE:	HISTONE-LYSINE N-METHYLTRANSFERASE EHMT2
90:00:00	7xuc-B	9.6	2.5	121	270	17	MOLECULE:	HISTONE-LYSINE N-METHYLTRANSFERASE EHMT2
91:00:00	5v9i-B	9.6	2.3	115	268	17	MOLECULE:	HISTONE-LYSINE N-METHYLTRANSFERASE EHMT2
92:00:00	6mbo-A	9.6	2.7	121	262	18	MOLECULE:	HISTONE-LYSINE N-METHYLTRANSFERASE EHMT1
93:00:00	6k9c-A	9.6	5.6	229	416	16	MOLECULE:	PRIMASE
94:00:00	1peg-A	9.6	2.7	120	249	18	MOLECULE:	HISTONE H3 METHYLTRANSFERASE DIM-5
95:00:00	6mbp-B	9.6	2.8	122	261	18	MOLECULE:	HISTONE-LYSINE N-METHYLTRANSFERASE EHMT1
96:00:00	3mo0-A	9.6	2.8	121	252	18	MOLECULE:	HISTONE-LYSINE N-METHYLTRANSFERASE, H3 LYSINE-9 S
97:00:00	5v9j-A	9.6	2.4	117	258	19	MOLECULE:	HISTONE-LYSINE N-METHYLTRANSFERASE EHMT1
98:00:00	5tuy-A	9.6	2.5	121	268	17	MOLECULE:	HISTONE-LYSINE N-METHYLTRANSFERASE EHMT2
99:00:00	8iqi-C	9.5	5.1	246	916	10	MOLECULE:	PUTATIVE PRIMASE C962R
0:00	3kma-A	9.5	2.3	93	105	17	MOLECULE:	A612L PROTEIN
1:00	7kso-A	9.5	2.5	117	395	19	MOLECULE:	HISTONE-LYSINE N-METHYLTRANSFERASE EZH1
2:00	4i51-A	9.5	2.8	120	262	18	MOLECULE:	HISTONE-LYSINE N-METHYLTRANSFERASE EHMT1
3:00	3sw9-B	9.5	2.7	121	262	19	MOLECULE:	HISTONE-LYSINE N-METHYLTRANSFERASE EHMT1
4:00	1mvx-A	9.5	2.9	124	269	15	MOLECULE:	CRYPTIC LOCI REGULATOR 4
5:00	2rfi-A	9.5	2.8	120	263	18	MOLECULE:	HISTONE-LYSINE N-METHYLTRANSFERASE, H3 LYSINE-9
6:00	3mo2-A	9.5	2.5	118	255	19	MOLECULE:	HISTONE-LYSINE N-METHYLTRANSFERASE, H3 LYSINE-9 S
7:00	2igq-B	9.5	2.5	117	255	18	MOLECULE:	EUCHROMATIC HISTONE METHYLTRANSFERASE 1
8:00	3hna-B	9.5	2.8	120	261	18	MOLECULE:	HISTONE-LYSINE N-METHYLTRANSFERASE, H3 LYSINE-9 S
9:00	2o8j-A	9.5	2.6	118	255	17	MOLECULE:	HISTONE-LYSINE N-METHYLTRANSFERASE, H3 LYSINE-9
10:00	5ww0-B	9.5	2.6	102	120	14	MOLECULE:	SET DOMAIN-CONTAINING PROTEIN 7
11:00	3kma-B	9.5	2.3	93	105	17	MOLECULE:	A612L PROTEIN
12:00	6bx3-E	9.5	2.6	115	192	17	MOLECULE:	HISTONE-LYSINE N-METHYLTRANSFERASE, H3 LYSINE-4 S
13:00	6xaz-B	9.5	2.5	101	125	21	MOLECULE:	PR DOMAIN ZINC FINGER PROTEIN 5
14:00	5ttg-A	9.5	2.6	118	261	19	MOLECULE:	HISTONE-LYSINE N-METHYLTRANSFERASE EHMT1
15:00	5ls6-J	9.5	2.5	117	570	20	MOLECULE:	HISTONE-LYSINE N-METHYLTRANSFERASE EZH2,HISTONE-L
16:00	8xpt-B	9.5	2.6	119	251	18	MOLECULE:	HISTONE-LYSINE N-METHYLTRANSFERASE EHMT1
17:00	5ttg-B	9.5	2.4	117	259	19	MOLECULE:	HISTONE-LYSINE N-METHYLTRANSFERASE EHMT1
18:00	3kmj-A	9.5	2.5	96	119	17	MOLECULE:	A612L PROTEIN
19:00	5vsd-A	9.5	2.3	116	258	18	MOLECULE:	HISTONE-LYSINE N-METHYLTRANSFERASE EHMT1
20:00	5vse-A	9.5	5.5	121	270	17	MOLECULE:	HISTONE-LYSINE N-METHYLTRANSFERASE EHMT2
21:00	7xuc-A	9.5	2.5	121	273	17	MOLECULE:	HISTONE-LYSINE N-METHYLTRANSFERASE EHMT2
22:00	7jsh-D	9.5	3.7	221	272	13	MOLECULE:	PROTEIN REP68

23:00	8umy-A	9.5	28.6	164	546	16	MOLECULE:	CHROMOSOME TRANSMISSION FIDELITY PROTEIN 18 HOMOL
24:00:00	5vsf-B	9.5	2.6	118	259	19	MOLECULE:	HISTONE-LYSINE N-METHYLTRANSFERASE EHMT1
25:00:00	5vsd-B	9.5	2.6	118	259	19	MOLECULE:	HISTONE-LYSINE N-METHYLTRANSFERASE EHMT1
26:00:00	3mo5-C	9.5	2.6	118	259	19	MOLECULE:	HISTONE-LYSINE N-METHYLTRANSFERASE, H3 LYSINE-9 S
27:00:00	3mo5-B	9.5	2.5	118	258	19	MOLECULE:	HISTONE-LYSINE N-METHYLTRANSFERASE, H3 LYSINE-9 S
28:00:00	8fyh-A	9.5	2.1	116	514	20	MOLECULE:	POLYCOMB PROTEIN SUZ12
29:00:00	6mbo-B	9.5	2.6	119	259	18	MOLECULE:	HISTONE-LYSINE N-METHYLTRANSFERASE EHMT1
30:00:00	3mo5-D	9.4	2.5	117	257	18	MOLECULE:	HISTONE-LYSINE N-METHYLTRANSFERASE, H3 LYSINE-9 S
31:00:00	2o8j-D	9.4	2.6	116	249	16	MOLECULE:	HISTONE-LYSINE N-METHYLTRANSFERASE, H3 LYSINE-9
32:00:00	1mvh-A	9.4	2.8	123	269	15	MOLECULE:	CRYPTIC LOCI REGULATOR 4
33:00:00	3fpd-B	9.4	2.8	121	261	18	MOLECULE:	HISTONE-LYSINE N-METHYLTRANSFERASE, H3 LYSINE-9
34:00:00	5ls6-A	9.4	2.2	115	570	20	MOLECULE:	HISTONE-LYSINE N-METHYLTRANSFERASE EZH2,HISTONE-L
35:00:00	2o8j-B	9.4	2.6	121	272	17	MOLECULE:	HISTONE-LYSINE N-METHYLTRANSFERASE, H3 LYSINE-9
36:00:00	3mo5-A	9.4	2.5	118	255	19	MOLECULE:	HISTONE-LYSINE N-METHYLTRANSFERASE, H3 LYSINE-9 S
37:00:00	3rjw-A	9.4	2.6	121	269	17	MOLECULE:	HISTONE-LYSINE N-METHYLTRANSFERASE EHMT2
38:00:00	3fpd-A	9.4	2.8	122	262	18	MOLECULE:	HISTONE-LYSINE N-METHYLTRANSFERASE, H3 LYSINE-9
39:00:00	6bp4-A	9.4	2.7	120	269	15	MOLECULE:	HISTONE-LYSINE N-METHYLTRANSFERASE, H3 LYSINE-9 S
40:00:00	5t0k-B	9.4	5.1	125	266	17	MOLECULE:	HISTONE-LYSINE N-METHYLTRANSFERASE EHMT2
41:00:00	7xua-A	9.4	2.6	121	274	17	MOLECULE:	HISTONE-LYSINE N-METHYLTRANSFERASE EHMT2
42:00:00	7xub-B	9.4	2.4	117	267	17	MOLECULE:	HISTONE-LYSINE N-METHYLTRANSFERASE EHMT2
43:00:00	1ml9-A	9.4	2.7	121	250	17	MOLECULE:	HISTONE H3 METHYLTRANSFERASE DIM-5
44:00:00	6p0r-B	9.4	2.4	114	230	20	MOLECULE:	HISTONE-LYSINE N-METHYLTRANSFERASE SUV39H2
45:00:00	6k9e-F	9.4	5.3	215	404	16	MOLECULE:	PRIMASE
46:00:00	6k9e-B	9.4	5.5	222	404	18	MOLECULE:	PRIMASE
47:00:00	6oax-D	9.4	11.2	191	581	13	MOLECULE:	HYPERACTIVE DISAGGREGASE CLPB
48:00:00	3u61-D	9.4	10.4	143	320	18	MOLECULE:	DNA POLYMERASE ACCESSORY PROTEIN 44
49:00:00	8uk9-K	9.4	10.4	135	319	19	MOLECULE:	SLIDING-CLAMP-LOADER SMALL SUBUNIT
50:00:00	8uh7-D	9.4	9.2	135	319	20	MOLECULE:	SLIDING-CLAMP-LOADER SMALL SUBUNIT
51:00:00	6rn4-C	9.4	10.6	189	689	13	MOLECULE:	CHAPERONE PROTEIN CLPB
52:00:00	3mo2-B	9.4	2.5	118	258	18	MOLECULE:	HISTONE-LYSINE N-METHYLTRANSFERASE, H3 LYSINE-9 S
53:00:00	3mo2-C	9.4	2.6	118	259	19	MOLECULE:	HISTONE-LYSINE N-METHYLTRANSFERASE, H3 LYSINE-9 S
54:00:00	5ecj-A	9.3	7	125	263	14	MOLECULE:	PR DOMAIN ZINC FINGER PROTEIN 14,PROTEIN CBFA2T2
55:00:00	6nzo-S	9.3	2.4	117	233	20	MOLECULE:	HISTONE H3
56:00:00	5wg6-C	9.3	3.5	154	504	16	MOLECULE:	HISTONE-LYSINE N-METHYLTRANSFERASE EZH2,POLYCOMB
57:00:00	4i51-B	9.3	2.3	115	259	17	MOLECULE:	HISTONE-LYSINE N-METHYLTRANSFERASE EHMT1
58:00:00	2igq-A	9.3	2.5	115	252	17	MOLECULE:	EUCHROMATIC HISTONE METHYLTRANSFERASE 1
59:00:00	3vv0-A	9.3	2.7	116	236	16	MOLECULE:	HISTONE-LYSINE N-METHYLTRANSFERASE SETD7
60:00:00	3k5k-B	9.3	5.6	122	270	16	MOLECULE:	HISTONE-LYSINE N-METHYLTRANSFERASE, H3 LYSINE-9 S
61:00:00	3mo2-D	9.3	2.5	117	257	18	MOLECULE:	HISTONE-LYSINE N-METHYLTRANSFERASE, H3 LYSINE-9 S
62:00:00	7dcf-B	9.3	2.1	114	260	18	MOLECULE:	HISTONE-LYSINE N-METHYLTRANSFERASE EHMT2
63:00:00	6a5k-A	9.3	2.6	119	467	17	MOLECULE:	HISTONE-LYSINE N-METHYLTRANSFERASE, H3 LYSINE-9 S
64:00:00	6px3-S	9.3	2.4	117	233	20	MOLECULE:	HISTONE H3
65:00:00	7t7l-C	9.3	2.5	119	267	18	MOLECULE:	HISTONE-LYSINE N-METHYLTRANSFERASE EHMT2
66:00:00	7t7l-B	9.3	2.5	118	265	18	MOLECULE:	HISTONE-LYSINE N-METHYLTRANSFERASE EHMT2
67:00:00	5t0m-A	9.3	2.4	116	261	17	MOLECULE:	HISTONE-LYSINE N-METHYLTRANSFERASE EHMT2
68:00:00	7btv-A	9.3	2.5	120	272	18	MOLECULE:	HISTONE-LYSINE N-METHYLTRANSFERASE EHMT2
69:00:00	8xpt-D	9.3	2.4	115	248	19	MOLECULE:	HISTONE-LYSINE N-METHYLTRANSFERASE EHMT1
70:00:00	5ttf-C	9.3	2.4	117	265	16	MOLECULE:	HISTONE-LYSINE N-METHYLTRANSFERASE EHMT2
71:00:00	7xua-B	9.3	5.9	123	275	17	MOLECULE:	HISTONE-LYSINE N-METHYLTRANSFERASE EHMT2
72:00:00	7buc-A	9.3	5.7	125	274	17	MOLECULE:	HISTONE-LYSINE N-METHYLTRANSFERASE EHMT2
73:00:00	3ray-A	9.3	2.7	109	159	19	MOLECULE:	PR DOMAIN-CONTAINING PROTEIN 11
74:00:00	7tts-B	9.3	8.6	182	440	13	MOLECULE:	CASEINOLYTIC PEPTIDASE B PROTEIN HOMOLOG
75:00:00	3u61-B	9.3	4.5	131	305	18	MOLECULE:	DNA POLYMERASE ACCESSORY PROTEIN 44

76:00:00	8unh-B	9.3	4.1	128	319	20	MOLECULE:	SLIDING-CLAMP-LOADER LARGE SUBUNIT
77:00:00	8unf-B	9.3	10.6	140	319	19	MOLECULE:	PRIMER DNA
78:00:00	8umu-A	9.3	24	167	515	14	MOLECULE:	CHROMOSOME TRANSMISSION FIDELITY PROTEIN 18 HOMOL
79:00:00	2rfi-B	9.2	2.3	114	252	18	MOLECULE:	HISTONE-LYSINE N-METHYLTRANSFERASE, H3 LYSINE-9
80:00:00	4nvq-B	9.2	5.6	118	264	16	MOLECULE:	HISTONE-LYSINE N-METHYLTRANSFERASE EHMT2
81:00:00	2o8j-C	9.2	2.6	117	263	17	MOLECULE:	HISTONE-LYSINE N-METHYLTRANSFERASE, H3 LYSINE-9
82:00:00	3k5k-A	9.2	2.5	119	273	17	MOLECULE:	HISTONE-LYSINE N-METHYLTRANSFERASE, H3 LYSINE-9 S
83:00:00	8xpt-A	9.2	3.1	122	258	18	MOLECULE:	HISTONE-LYSINE N-METHYLTRANSFERASE EHMT1
84:00:00	5jhn-A	9.2	2.8	120	274	18	MOLECULE:	HISTONE-LYSINE N-METHYLTRANSFERASE EHMT2
85:00:00	5jj0-A	9.2	2.8	120	274	18	MOLECULE:	HISTONE-LYSINE N-METHYLTRANSFERASE EHMT2
86:00:00	5jin-A	9.2	2.8	120	274	18	MOLECULE:	HISTONE-LYSINE N-METHYLTRANSFERASE EHMT2
87:00:00	5jhn-B	9.2	5.5	120	268	18	MOLECULE:	HISTONE-LYSINE N-METHYLTRANSFERASE EHMT2
88:00:00	5t0k-A	9.2	2.4	116	265	17	MOLECULE:	HISTONE-LYSINE N-METHYLTRANSFERASE EHMT2
89:00:00	5v9i-C	9.2	2.3	115	265	17	MOLECULE:	HISTONE-LYSINE N-METHYLTRANSFERASE EHMT2
90:00:00	7t7l-A	9.2	2.7	120	267	16	MOLECULE:	HISTONE-LYSINE N-METHYLTRANSFERASE EHMT2
91:00:00	5t0m-B	9.2	2.8	120	272	18	MOLECULE:	HISTONE-LYSINE N-METHYLTRANSFERASE EHMT2
92:00:00	5v9i-D	9.2	2.3	115	265	17	MOLECULE:	HISTONE-LYSINE N-METHYLTRANSFERASE EHMT2
93:00:00	6k9e-D	9.2	5.7	222	404	16	MOLECULE:	PRIMASE
94:00:00	3u5z-O	9.2	4.4	135	305	19	MOLECULE:	DNA POLYMERASE ACCESSORY PROTEIN 44
95:00:00	3u5z-C	9.2	10.9	139	320	19	MOLECULE:	DNA POLYMERASE ACCESSORY PROTEIN 44
96:00:00	8uh7-C	9.2	9.2	139	320	19	MOLECULE:	SLIDING-CLAMP-LOADER SMALL SUBUNIT
97:00:00	8unf-C	9.2	10.5	140	319	19	MOLECULE:	PRIMER DNA
98:00:00	7ttr-D	9.2	12.2	163	334	12	MOLECULE:	CASEINOLYTIC PEPTIDASE B PROTEIN HOMOLOG
99:00:00	7t7m-D	9.2	2.5	117	262	19	MOLECULE:	HISTONE-LYSINE N-METHYLTRANSFERASE EHMT1
0:00	5jiy-A	9.1	2.8	120	274	18	MOLECULE:	HISTONE-LYSINE N-METHYLTRANSFERASE EHMT2
1:00	3hna-A	9.1	2.8	120	281	18	MOLECULE:	HISTONE-LYSINE N-METHYLTRANSFERASE, H3 LYSINE-9 S
2:00	4nvq-A	9.1	2.6	117	268	18	MOLECULE:	HISTONE-LYSINE N-METHYLTRANSFERASE EHMT2
3:00	3rjw-B	9.1	11.4	122	271	16	MOLECULE:	HISTONE-LYSINE N-METHYLTRANSFERASE EHMT2
4:00	6box-B	9.1	2.6	118	268	15	MOLECULE:	HISTONE-LYSINE N-METHYLTRANSFERASE, H3 LYSINE-9 S
5:00	5tuy-B	9.1	2.6	119	264	18	MOLECULE:	HISTONE-LYSINE N-METHYLTRANSFERASE EHMT2
6:00	5jin-B	9.1	5.5	120	268	18	MOLECULE:	HISTONE-LYSINE N-METHYLTRANSFERASE EHMT2
7:00	7btv-B	9.1	2.5	119	273	18	MOLECULE:	HISTONE-LYSINE N-METHYLTRANSFERASE EHMT2
8:00	5jj0-B	9.1	5.5	120	268	18	MOLECULE:	HISTONE-LYSINE N-METHYLTRANSFERASE EHMT2
9:00	7ea5-K	9.1	2.9	118	229	18	MOLECULE:	HISTONE H3
10:00	7t7l-D	9.1	2.5	118	270	17	MOLECULE:	HISTONE-LYSINE N-METHYLTRANSFERASE EHMT2
11:00	5jiy-B	9.1	5.4	120	268	18	MOLECULE:	HISTONE-LYSINE N-METHYLTRANSFERASE EHMT2
12:00	5vse-B	9.1	5.5	120	272	17	MOLECULE:	HISTONE-LYSINE N-METHYLTRANSFERASE EHMT2
13:00	7buc-B	9.1	2.4	116	270	17	MOLECULE:	HISTONE-LYSINE N-METHYLTRANSFERASE EHMT2
14:00	7qnr-A	9.1	3.8	121	427	14	MOLECULE:	HISTONE-LYSINE N-METHYLTRANSFERASE SMYD3
15:00	6z2a-A	9.1	2.8	122	284	15	MOLECULE:	HISTONE-LYSINE N-METHYLTRANSFERASE, H3 LYSINE-9 S
16:00	6z2a-B	9.1	2.7	122	277	15	MOLECULE:	HISTONE-LYSINE N-METHYLTRANSFERASE, H3 LYSINE-9 S
17:00	6p0r-A	9.1	2.8	122	254	20	MOLECULE:	HISTONE-LYSINE N-METHYLTRANSFERASE SUV39H2
18:00	8dr5-A	9.1	17.5	174	646	10	MOLECULE:	REPLICATION FACTOR C SUBUNIT 1
19:00	7u1a-A	9.1	13.8	166	617	12	MOLECULE:	REPLICATION FACTOR C SUBUNIT 1
20:00	3u60-D	9.1	8.6	135	319	19	MOLECULE:	DNA POLYMERASE ACCESSORY PROTEIN 44
21:00	3u60-C	9.1	9	138	320	19	MOLECULE:	DNA POLYMERASE ACCESSORY PROTEIN 44
22:00	3u60-E	9.1	4.2	130	305	19	MOLECULE:	DNA POLYMERASE ACCESSORY PROTEIN 44
23:00	8uk9-B	9.1	10	134	319	19	MOLECULE:	SLIDING-CLAMP-LOADER SMALL SUBUNIT
24:00:00	8unf-D	9.1	10.8	140	319	19	MOLECULE:	PRIMER DNA
25:00:00	6rn3-E	9.1	9.6	185	684	14	MOLECULE:	CHAPERONE PROTEIN CLPB
26:00:00	7t7m-B	9.1	2.6	119	259	18	MOLECULE:	HISTONE-LYSINE N-METHYLTRANSFERASE EHMT1
27:00:00	7ksr-A	9	2	114	395	19	MOLECULE:	HISTONE-LYSINE N-METHYLTRANSFERASE EZH1
28:00:00	5arg-A	9	3.7	119	427	11	MOLECULE:	N-LYSINE METHYLTRANSFERASE SMYD2

29:00:00	5arf-A	9	3.6	118	426	12	MOLECULE:	N-LYSINE METHYLTRANSFERASE SMYD2
30:00:00	6xaz-A	9	2.3	94	118	21	MOLECULE:	PR DOMAIN ZINC FINGER PROTEIN 5
31:00:00	7pds-D	9	4.4	237	454	11	MOLECULE:	SIMILAR TO D. NODOSUS VAPE
32:00:00	7pds-C	9	4.4	239	451	11	MOLECULE:	SIMILAR TO D. NODOSUS VAPE
33:00:00	7tib-A	9	11.1	151	495	14	MOLECULE:	REPLICATION FACTOR C SUBUNIT 1
34:00:00	8dr1-A	9	11.1	149	493	13	MOLECULE:	REPLICATION FACTOR C SUBUNIT 1
35:00:00	8efy-N	9	11.7	148	310	12	MOLECULE:	HOLLIDAY JUNCTION ATP-DEPENDENT DNA HELICASE RUVB
36:00:00	3u5z-D	9	10.1	138	320	20	MOLECULE:	DNA POLYMERASE ACCESSORY PROTEIN 44
37:00:00	8uk9-M	9	9.4	135	317	18	MOLECULE:	SLIDING-CLAMP-LOADER SMALL SUBUNIT
38:00:00	9emc-F	9	8.2	144	337	13	MOLECULE:	RUVB-LIKE 1
39:00:00	7ktp-A	9	1.9	114	395	19	MOLECULE:	HISTONE-LYSINE N-METHYLTRANSFERASE EZH1
40:00:00	2r3a-A	9	4.2	124	272	19	MOLECULE:	HISTONE-LYSINE N-METHYLTRANSFERASE SUV39H2
41:00:00	6xau-A	8.9	2.4	100	119	13	MOLECULE:	HISTONE-LYSINE N-METHYLTRANSFERASE MECOM
42:00:00	6kiw-K	8.9	2.7	117	156	20	MOLECULE:	HISTONE H3
43:00:00	7pds-B	8.9	4	234	448	12	MOLECULE:	SIMILAR TO D. NODOSUS VAPE
44:00:00	7xc2-E	8.9	26.7	172	858	13	MOLECULE:	CNL9
45:00:00	8iqi-B	8.9	6.4	254	915	10	MOLECULE:	PUTATIVE PRIMASE C962R
46:00:00	3u5z-B	8.9	10.9	134	320	19	MOLECULE:	DNA POLYMERASE ACCESSORY PROTEIN 44
47:00:00	3u5z-N	8.9	10.2	138	320	20	MOLECULE:	DNA POLYMERASE ACCESSORY PROTEIN 44
48:00:00	3u5z-L	8.9	8.4	133	320	19	MOLECULE:	DNA POLYMERASE ACCESSORY PROTEIN 44
49:00:00	3u5z-M	8.9	9.4	137	320	19	MOLECULE:	DNA POLYMERASE ACCESSORY PROTEIN 44
50:00:00	9emc-D	8.9	4.7	141	337	13	MOLECULE:	RUVB-LIKE 1
51:00:00	5hi7-A	8.8	3.7	121	426	14	MOLECULE:	HISTONE-LYSINE N-METHYLTRANSFERASE SMYD3
52:00:00	8iqi-D	8.8	5	243	916	10	MOLECULE:	PUTATIVE PRIMASE C962R
53:00:00	3u61-E	8.8	4	130	294	22	MOLECULE:	DNA POLYMERASE ACCESSORY PROTEIN 44
54:00:00	3u60-B	8.8	8.6	134	319	19	MOLECULE:	DNA POLYMERASE ACCESSORY PROTEIN 44
55:00:00	8uk9-N	8.8	4.5	127	305	19	MOLECULE:	SLIDING-CLAMP-LOADER SMALL SUBUNIT
56:00:00	8uk9-C	8.8	11	141	320	18	MOLECULE:	SLIDING-CLAMP-LOADER SMALL SUBUNIT
57:00:00	8ui8-A	8.8	17.8	168	643	11	MOLECULE:	ATPASE FAMILY AAA DOMAIN-CONTAINING PROTEIN 5
58:00:00	8un0-A	8.8	26.2	160	546	15	MOLECULE:	CHROMOSOME TRANSMISSION FIDELITY PROTEIN 18 HOMOL
59:00:00	7xc2-A	8.7	25.5	163	858	13	MOLECULE:	CNL9
60:00:00	7mi8-A	8.7	4.9	190	868	8	MOLECULE:	FUSION PROTEIN OF DYNEIN AND ENDOLYSIN
61:00:00	6zrb-A	8.7	3.8	121	426	14	MOLECULE:	HISTONE-LYSINE N-METHYLTRANSFERASE SMYD3
62:00:00	7xc2-C	8.7	24.5	171	858	13	MOLECULE:	CNL9
63:00:00	7ttr-A	8.7	8.8	147	304	12	MOLECULE:	CASEINOLYTIC PEPTIDASE B PROTEIN HOMOLOG
64:00:00	2qpw-A	8.7	3.1	105	147	16	MOLECULE:	PR DOMAIN ZINC FINGER PROTEIN 2
65:00:00	7om0-D	8.7	6.5	217	438	12	MOLECULE:	DNA PRIMASE
66:00:00	7u1p-A	8.7	13.3	161	617	12	MOLECULE:	REPLICATION FACTOR C SUBUNIT 1
67:00:00	8iqi-A	8.7	6.4	249	915	10	MOLECULE:	PUTATIVE PRIMASE C962R
68:00:00	7xc2-I	8.7	26.4	172	858	13	MOLECULE:	CNL9
69:00:00	8unf-E	8.7	4.9	129	319	19	MOLECULE:	PRIMER DNA
70:00:00	8uk9-L	8.7	9.2	137	320	18	MOLECULE:	SLIDING-CLAMP-LOADER SMALL SUBUNIT
71:00:00	6qs6-E	8.7	15.2	222	577	11	MOLECULE:	CHAPERONE PROTEIN CLPB
72:00:00	6oax-B	8.7	10.8	191	581	14	MOLECULE:	HYPERACTIVE DISAGGREGASE CLPB
73:00:00	4qep-A	8.7	3.2	118	468	19	MOLECULE:	HISTONE-LYSINE N-METHYLTRANSFERASE, H3 LYSINE-9 S
74:00:00	3ep0-A	8.6	2.2	89	113	15	MOLECULE:	PR DOMAIN ZINC FINGER PROTEIN 12
75:00:00	7o2c-A	8.6	3.8	121	428	14	MOLECULE:	HISTONE-LYSINE N-METHYLTRANSFERASE SMYD3
76:00:00	4qeo-A	8.6	3.4	120	473	18	MOLECULE:	HISTONE-LYSINE N-METHYLTRANSFERASE, H3 LYSINE-9 S
77:00:00	3ep0-B	8.6	2.5	92	113	13	MOLECULE:	PR DOMAIN ZINC FINGER PROTEIN 12
78:00:00	4qen-A	8.6	3.3	120	473	18	MOLECULE:	HISTONE-LYSINE N-METHYLTRANSFERASE, H3 LYSINE-9 S
79:00:00	7ola-A	8.6	6.9	216	441	13	MOLECULE:	DNA PRIMASE
80:00:00	8dr7-A	8.6	13.5	149	497	13	MOLECULE:	REPLICATION FACTOR C SUBUNIT 1
81:00:00	6oax-A	8.6	14.6	189	581	14	MOLECULE:	HYPERACTIVE DISAGGREGASE CLPB

82:00:00	6mfv-B	8.6	8.8	131	641	11	MOLECULE:	TETRATRICOPEPTIDE REPEAT SENSOR PH0952
83:00:00	8uk9-D	8.6	10.4	138	318	19	MOLECULE:	SLIDING-CLAMP-LOADER SMALL SUBUNIT
84:00:00	3uk6-E	8.6	3.1	139	290	12	MOLECULE:	RUVB-LIKE 2
85:00:00	5fm7-B	8.6	11.4	146	422	12	MOLECULE:	RVB1
86:00:00	7crr-I	8.5	3.2	116	223	21	MOLECULE:	HISTONE H3
87:00:00	7o2b-A	8.5	3.8	121	428	14	MOLECULE:	HISTONE-LYSINE N-METHYLTRANSFERASE SMYD3
88:00:00	6paf-A	8.5	3.7	121	424	15	MOLECULE:	HISTONE-LYSINE N-METHYLTRANSFERASE SMYD3
89:00:00	3dal-B	8.5	3.2	111	166	14	MOLECULE:	PR DOMAIN ZINC FINGER PROTEIN 1
90:00:00	4au7-B	8.5	3.1	98	209	10	MOLECULE:	HISTONE-LYSINE N-METHYLTRANSFERASE SUV420H2
91:00:00	7qnu-A	8.5	3.8	121	427	15	MOLECULE:	HISTONE-LYSINE N-METHYLTRANSFERASE SMYD3
92:00:00	7om0-E	8.5	6.2	215	439	13	MOLECULE:	DNA PRIMASE
93:00:00	7om0-A	8.5	6.2	215	439	13	MOLECULE:	DNA PRIMASE
94:00:00	7om0-C	8.5	6.5	225	439	13	MOLECULE:	DNA PRIMASE
95:00:00	7om0-F	8.5	7.1	242	439	13	MOLECULE:	DNA PRIMASE
96:00:00	3u5z-E	8.5	4.3	131	305	19	MOLECULE:	DNA POLYMERASE ACCESSORY PROTEIN 44
97:00:00	3uk6-L	8.5	8.6	128	232	12	MOLECULE:	RUVB-LIKE 2
98:00:00	1s9h-C	8.5	4.6	157	199	14	MOLECULE:	REP 40 PROTEIN
99:00:00	6o9o-A	8.4	3.8	121	427	14	MOLECULE:	HISTONE-LYSINE N-METHYLTRANSFERASE SMYD3
0:00	6z2r-A	8.4	3.8	121	427	14	MOLECULE:	HISTONE-LYSINE N-METHYLTRANSFERASE SMYD3
1:00	6p6g-A	8.4	3.8	121	427	14	MOLECULE:	HISTONE-LYSINE N-METHYLTRANSFERASE SMYD3
2:00	7las-A	8.4	5.7	134	183	16	MOLECULE:	ATP-DEPENDENT HELICASE REP
3:00	5n7r-A	8.4	14.9	143	380	10	MOLECULE:	RUVB-LIKE 2
4:00	6rn2-D	8.4	11.4	189	684	12	MOLECULE:	CHAPERONE PROTEIN CLPB
5:00	6vvo-A	8.4	6.6	156	448	9	MOLECULE:	REPLICATION FACTOR C SUBUNIT 1
6:00	3uk6-F	8.4	11.8	144	301	13	MOLECULE:	RUVB-LIKE 2
7:00	7qv9-a	8.4	7.3	149	259	9	MOLECULE:	DNA-DIRECTED RNA POLYMERASE SUBUNIT ALPHA
8:00	9emc-E	8.4	8.6	145	337	13	MOLECULE:	RUVB-LIKE 1
9:00	7ud5-K	8.3	2.7	114	176	18	MOLECULE:	HISTONE H3
10:00	6bp4-B	8.3	4.4	124	283	15	MOLECULE:	HISTONE-LYSINE N-METHYLTRANSFERASE, H3 LYSINE-9 S
11:00	7ln2-F	8.3	14.6	185	700	9	MOLECULE:	TRANSITIONAL ENDOPLASMIC RETICULUM ATPASE
12:00	4bup-A	8.3	3.2	105	233	10	MOLECULE:	HISTONE-LYSINE N-METHYLTRANSFERASE SUV420H1
13:00	5v3h-A	8.3	4.2	121	439	12	MOLECULE:	N-LYSINE METHYLTRANSFERASE SMYD2
14:00	6oax-E	8.3	10.5	184	579	14	MOLECULE:	HYPERACTIVE DISAGGREGASE CLPB
15:00	7ola-E	8.3	4.3	198	344	14	MOLECULE:	DNA PRIMASE
16:00	8apl-D	8.3	6.1	214	449	13	MOLECULE:	PRIMASE D5
17:00	8hwe-D	8.3	5	215	379	9	MOLECULE:	PRIMASE D5
18:00	6u1y-F	8.3	2.9	127	244	18	MOLECULE:	MITOCHONDRIAL CHAPERONE BCS1
19:00	6mat-C	8.3	12.4	159	578	11	MOLECULE:	RIX7 MUTANT
20:00	7jlv-A	8.3	15.6	144	437	15	MOLECULE:	DISEASE RESISTANCE PROTEIN ROQ1
21:00	7jlv-G	8.3	13	136	437	15	MOLECULE:	DISEASE RESISTANCE PROTEIN ROQ1
22:00	5oaf-D	8.3	9.3	144	411	11	MOLECULE:	RUVB-LIKE 1
23:00	5z6r-A	8.3	11.5	143	273	11	MOLECULE:	SPASTIN
24:00:00	6mat-D	8.3	18.9	188	578	9	MOLECULE:	RIX7 MUTANT
25:00:00	6og1-A	8.3	14.2	220	581	15	MOLECULE:	HYPERACTIVE DISAGGREGASE CLPB
26:00:00	6box-A	8.2	2.7	114	271	16	MOLECULE:	HISTONE-LYSINE N-METHYLTRANSFERASE, H3 LYSINE-9 S
27:00:00	4bup-B	8.2	3.2	105	238	10	MOLECULE:	HISTONE-LYSINE N-METHYLTRANSFERASE SUV420H1
28:00:00	3db5-B	8.2	2.9	99	134	14	MOLECULE:	PR DOMAIN ZINC FINGER PROTEIN 4
29:00:00	3ihx-C	8.2	3	99	124	13	MOLECULE:	PR DOMAIN ZINC FINGER PROTEIN 10
30:00:00	3db5-A	8.2	2.5	93	124	14	MOLECULE:	PR DOMAIN ZINC FINGER PROTEIN 4
31:00:00	8apl-C	8.2	6.2	221	449	12	MOLECULE:	PRIMASE D5
32:00:00	7lar-E	8.2	5.8	136	183	16	MOLECULE:	ATP-DEPENDENT HELICASE REP
33:00:00	8apl-E	8.2	6.1	217	449	12	MOLECULE:	PRIMASE D5
34:00:00	8apl-B	8.2	6.8	225	449	12	MOLECULE:	PRIMASE D5

35:00:00	8apm-D	8.2	6.4	220	449	12	MOLECULE:	PRIMASE D5
36:00:00	7las-E	8.2	5.4	136	183	16	MOLECULE:	ATP-DEPENDENT HELICASE REP
37:00:00	7qo4-M	8.2	11.4	178	421	11	MOLECULE:	26S PROTEASOME REGULATORY SUBUNIT RPN1
38:00:00	7las-C	8.2	5.9	142	183	15	MOLECULE:	ATP-DEPENDENT HELICASE REP
39:00:00	8apl-F	8.2	6.4	207	449	12	MOLECULE:	PRIMASE D5
40:00:00	8apl-A	8.2	6.2	214	449	12	MOLECULE:	PRIMASE D5
41:00:00	5oaf-F	8.2	8.7	147	407	12	MOLECULE:	RUVB-LIKE 1
42:00:00	7jlv-B	8.2	10.6	141	437	16	MOLECULE:	DISEASE RESISTANCE PROTEIN ROQ1
43:00:00	3uk6-B	8.2	3.4	139	300	13	MOLECULE:	RUVB-LIKE 2
44:00:00	3uk6-J	8.2	3.2	145	300	12	MOLECULE:	RUVB-LIKE 2
45:00:00	3uk6-A	8.2	3.1	139	298	12	MOLECULE:	RUVB-LIKE 2
46:00:00	7ttr-C	8.2	10.4	155	327	14	MOLECULE:	CASEINOLYTIC PEPTIDASE B PROTEIN HOMOLOG
47:00:00	5vy9-A	8.2	10.2	211	849	9	MOLECULE:	HEAT SHOCK PROTEIN 104
48:00:00	5z6q-A	8.2	13.2	145	272	10	MOLECULE:	SPASTIN
49:00:00	3dal-A	8.1	3.2	110	179	19	MOLECULE:	PR DOMAIN ZINC FINGER PROTEIN 1
50:00:00	3s8p-B	8.1	3.2	105	239	10	MOLECULE:	HISTONE-LYSINE N-METHYLTRANSFERASE SUV420H1
51:00:00	7xc2-G	8.1	26.8	172	858	13	MOLECULE:	CNL9
52:00:00	7lar-C	8.1	5.2	134	183	16	MOLECULE:	ATP-DEPENDENT HELICASE REP
53:00:00	8apm-E	8.1	6	223	449	12	MOLECULE:	PRIMASE D5
54:00:00	8hwf-C	8.1	4.1	206	378	9	MOLECULE:	PRIMASE D5
55:00:00	8hwd-B	8.1	4.9	206	378	10	MOLECULE:	PRIMASE D5
56:00:00	8xj7-B	8.1	7	212	452	13	MOLECULE:	MONKEYPOX VIRUS E5
57:00:00	8hwh-C	8.1	4.2	204	361	9	MOLECULE:	PRIMASE D5
58:00:00	7u19-A	8.1	14.4	163	617	13	MOLECULE:	REPLICATION FACTOR C SUBUNIT 1
59:00:00	6h7x-A	8.1	13.9	144	371	11	MOLECULE:	RUVB-LIKE 2
60:00:00	6e10-6	8.1	11.1	187	718	17	MOLECULE:	HEAT SHOCK PROTEIN 101
61:00:00	5fm6-A	8.1	3.4	145	426	16	MOLECULE:	RVB1
62:00:00	5fm7-A	8.1	3.5	146	418	13	MOLECULE:	RVB1
63:00:00	6u1y-G	8.1	3	128	244	18	MOLECULE:	MITOCHONDRIAL CHAPERONE BCS1
64:00:00	7ttr-E	8.1	11.5	164	332	12	MOLECULE:	CASEINOLYTIC PEPTIDASE B PROTEIN HOMOLOG
65:00:00	6rn2-E	8.1	10.5	186	684	12	MOLECULE:	CHAPERONE PROTEIN CLPB
66:00:00	6qi9-E	8.1	9.1	150	335	11	MOLECULE:	RUVB-LIKE 1
67:00:00	7ea8-L	8.1	3.1	119	246	18	MOLECULE:	HISTONE-LYSINE N-METHYLTRANSFERASE SETD2
68:00:00	8y6p-Q	8	8.5	152	1228	11	MOLECULE:	APAF-1 RELATED KILLER DARK
69:00:00	6hyp-A	8	18.3	194	2272	9	MOLECULE:	MIDASIN,MIDASIN
70:00:00	7crp-I	8	2.8	114	223	21	MOLECULE:	HISTONE H3
71:00:00	4nj5-A	8	2.6	114	482	16	MOLECULE:	PROBABLE HISTONE-LYSINE N-METHYLTRANSFERASE, H3 L
72:00:00	6p7z-A	8	3.8	121	426	14	MOLECULE:	HISTONE-LYSINE N-METHYLTRANSFERASE SMYD3
73:00:00	5ecj-B	8	8.1	128	263	13	MOLECULE:	PR DOMAIN ZINC FINGER PROTEIN 14,PROTEIN CBFA2T2
74:00:00	5wbv-A	8	3.3	106	234	9	MOLECULE:	HISTONE-LYSINE N-METHYLTRANSFERASE KMT5B
75:00:00	6hz9-J	8	3.7	137	284	11	MOLECULE:	5-METHYLCYTOSINE-SPECIFIC RESTRICTION ENZYME B
76:00:00	8xj7-C	8	5.6	207	454	10	MOLECULE:	MONKEYPOX VIRUS E5
77:00:00	8hwf-A	8	4.4	214	378	11	MOLECULE:	PRIMASE D5
78:00:00	8hwf-B	8	4.4	207	378	10	MOLECULE:	PRIMASE D5
79:00:00	8hwa-B	8	4.1	210	379	10	MOLECULE:	PRIMASE D5
80:00:00	8xj7-A	8	4.4	212	377	11	MOLECULE:	MONKEYPOX VIRUS E5
81:00:00	6e10-1	8	5.6	184	719	15	MOLECULE:	HEAT SHOCK PROTEIN 101
82:00:00	7tts-D	8	11.4	177	463	12	MOLECULE:	CASEINOLYTIC PEPTIDASE B PROTEIN HOMOLOG
83:00:00	3uk6-I	8	3.1	137	294	12	MOLECULE:	RUVB-LIKE 2
84:00:00	3uk6-C	8	3.2	146	321	12	MOLECULE:	RUVB-LIKE 2
85:00:00	7xbk-H	8	3.2	146	345	12	MOLECULE:	ISOFORM 2 OF CASEINOLYTIC PEPTIDASE B PROTEIN HOM
86:00:00	3s8p-A	7.9	3.3	106	234	10	MOLECULE:	HISTONE-LYSINE N-METHYLTRANSFERASE SUV420H1
87:00:00	5hq8-A	7.9	3.8	121	427	13	MOLECULE:	HISTONE-LYSINE N-METHYLTRANSFERASE SMYD3

88:00:00	3rq4-A	7.9	3.9	111	241	10	MOLECULE:	HISTONE-LYSINE N-METHYLTRANSFERASE SUV420H2
89:00:00	4c1q-A	7.9	2.6	107	174	16	MOLECULE:	HISTONE-LYSINE N-METHYLTRANSFERASE PRDM9
90:00:00	7t0v-C	7.9	11.4	155	570	11	MOLECULE:	RIX7
91:00:00	6hz9-D	7.9	3.7	137	284	11	MOLECULE:	5-METHYLCYTOSINE-SPECIFIC RESTRICTION ENZYME B
92:00:00	7ola-F	7.9	5.3	199	345	14	MOLECULE:	DNA PRIMASE
93:00:00	3syk-A	7.9	11.3	146	290	11	MOLECULE:	PROTEIN CBBX
94:00:00	1g4a-E	7.9	9.7	152	356	13	MOLECULE:	ATP-DEPENDENT HSL PROTEASE ATP-BINDING SUBUNIT
95:00:00	8hwf-D	7.9	4.2	202	378	10	MOLECULE:	PRIMASE D5
96:00:00	8hwe-C	7.9	4.3	208	379	9	MOLECULE:	PRIMASE D5
97:00:00	6po1-B	7.9	12.5	164	352	14	MOLECULE:	ATP-DEPENDENT CLP PROTEASE ATP-BINDING SUBUNIT CL
98:00:00	8hwh-B	7.9	4.6	209	357	11	MOLECULE:	PRIMASE D5
99:00:00	8hwe-B	7.9	4.4	209	379	10	MOLECULE:	PRIMASE D5
0:00	6p07-D	7.9	12.1	136	303	13	MOLECULE:	SPASTIN
1:00	6oay-D	7.9	14.1	151	570	10	MOLECULE:	HYPERACTIVE DISAGGREGASE CLPB
2:00	3uk6-K	7.9	3.1	136	283	12	MOLECULE:	RUVB-LIKE 2
3:00	3uk6-H	7.9	8.9	138	277	15	MOLECULE:	RUVB-LIKE 2
4:00	3zuh-C	7.9	12.2	143	286	11	MOLECULE:	PROTEIN CBBX
5:00	6e11-4	7.9	9.1	192	717	15	MOLECULE:	UNKNOWN (CLAW)
6:00	7crq-L	7.8	2.8	115	223	21	MOLECULE:	HISTONE H3
7:00	4au7-A	7.8	3.8	109	244	12	MOLECULE:	HISTONE-LYSINE N-METHYLTRANSFERASE SUV420H2
8:00	6fnd-C	7.8	5.8	109	420	13	MOLECULE:	APICAL COMPLEX LYSINE METHYLTRANSFERASE
9:00	6mat-F	7.8	9.8	160	568	12	MOLECULE:	RIX7 MUTANT
10:00	8umt-A	7.8	24	162	537	15	MOLECULE:	CHROMOSOME TRANSMISSION FIDELITY PROTEIN 18 HOMOL
11:00	5wbv-B	7.8	3.6	105	228	11	MOLECULE:	HISTONE-LYSINE N-METHYLTRANSFERASE KMT5B
12:00	5yww-A	7.8	14.4	149	485	11	MOLECULE:	NUCLEOTIDE BINDING PROTEIN PINC
13:00	8hwb-B	7.8	4.4	213	379	10	MOLECULE:	PRIMASE D5
14:00	8hwb-C	7.8	4.6	211	379	8	MOLECULE:	PRIMASE D5
15:00	8hwb-A	7.8	3.9	206	379	10	MOLECULE:	PRIMASE D5
16:00	7xe0-C	7.8	22.9	162	838	14	MOLECULE:	CNL9
17:00	8xj8-C	7.8	4.2	204	379	9	MOLECULE:	DNA (70-MER)
18:00	2qz4-A	7.8	3.3	134	223	14	MOLECULE:	PARAPLEGIN
19:00	8umy-D	7.8	15.1	147	329	12	MOLECULE:	CHROMOSOME TRANSMISSION FIDELITY PROTEIN 18 HOMOL
20:00	7crq-I	7.8	2.8	116	223	20	MOLECULE:	HISTONE H3
21:00	7fd4-A	7.7	18.1	244	779	10	MOLECULE:	LON PROTEASE
22:00	8c0v-B	7.7	11.9	204	1030	10	MOLECULE:	PEROXISOMAL ATPASE PEX1
23:00	8hr8-H	7.7	14.6	158	834	7	MOLECULE:	ARCHAEAL ATPASE
24:00:00	5hyn-K	7.7	2.6	119	563	19	MOLECULE:	HISTONE-LYSINE N-METHYLTRANSFERASE EZH2
25:00:00	5cpr-B	7.7	4.4	107	233	11	MOLECULE:	HISTONE-LYSINE N-METHYLTRANSFERASE SUV420H1
26:00:00	7yrd-K	7.7	5.9	117	260	9	MOLECULE:	HISTONE H3.2
27:00:00	7t3i-B	7.7	13.1	165	578	10	MOLECULE:	RIX7
28:00:00	3oxf-B	7.7	3.8	121	426	15	MOLECULE:	SET AND MYND DOMAIN-CONTAINING PROTEIN 3
29:00:00	7z11-D	7.7	9.7	189	734	8	MOLECULE:	ATPASE FAMILY GENE 2 PROTEIN
30:00:00	7lar-F	7.7	5.2	128	183	17	MOLECULE:	ATP-DEPENDENT HELICASE REP
31:00:00	7las-F	7.7	5.2	124	183	16	MOLECULE:	ATP-DEPENDENT HELICASE REP
32:00:00	8apm-B	7.7	6.2	221	449	12	MOLECULE:	PRIMASE D5
33:00:00	8hwa-D	7.7	4.2	202	379	9	MOLECULE:	PRIMASE D5
34:00:00	8hwh-D	7.7	4.6	204	363	11	MOLECULE:	PRIMASE D5
35:00:00	8hwb-E	7.7	4.7	223	700	11	MOLECULE:	PRIMASE D5
36:00:00	8hwg-D	7.7	4.8	213	378	9	MOLECULE:	PRIMASE D5
37:00:00	8dr4-A	7.7	10.2	144	497	14	MOLECULE:	REPLICATION FACTOR C SUBUNIT 1
38:00:00	6fnd-B	7.7	6.2	111	418	13	MOLECULE:	APICAL COMPLEX LYSINE METHYLTRANSFERASE
39:00:00	8xj8-A	7.7	6.2	214	422	10	MOLECULE:	DNA (70-MER)
40:00:00	6opc-B	7.7	9.4	166	741	10	MOLECULE:	CELL DIVISION CONTROL PROTEIN 48

41:00:00	6mat-B	7.7	12.9	170	578	12	MOLECULE:	RIX7 MUTANT
42:00:00	3jc7-7	7.7	8.9	211	657	10	MOLECULE:	DNA REPLICATION LICENSING FACTOR MCM2
43:00:00	2chq-A	7.7	14.7	144	313	13	MOLECULE:	REPLICATION FACTOR C SMALL SUBUNIT
44:00:00	6z1f-2	7.7	11.3	145	281	11	MOLECULE:	RIBULOSE BISPHOSPHATE CARBOXYLASE/OXYGENASE ACTIV
45:00:00	7jlv-D	7.7	12.4	131	437	17	MOLECULE:	DISEASE RESISTANCE PROTEIN ROQ1
46:00:00	6igm-D	7.7	4.6	135	396	13	MOLECULE:	RUVB-LIKE 1
47:00:00	5fm6-B	7.7	11.2	147	419	14	MOLECULE:	RVB1
48:00:00	6epc-L	7.7	19.5	159	389	10	MOLECULE:	PROTEASOME SUBUNIT ALPHA TYPE-6
49:00:00	7xbk-A	7.7	3.6	147	367	14	MOLECULE:	ISOFORM 2 OF CASEINOLYTIC PEPTIDASE B PROTEIN HOM
50:00:00	6oa9-B	7.7	8.6	168	493	7	MOLECULE:	CELL DIVISION CONTROL PROTEIN 48
51:00:00	5ji3-F	7.7	9.6	154	353	14	MOLECULE:	ATP-DEPENDENT PROTEASE SUBUNIT HSLV
52:00:00	2fuf-A	7.7	3.3	104	126	7	MOLECULE:	LARGE T ANTIGEN
53:00:00	2ipr-B	7.7	3.3	105	124	7	MOLECULE:	LARGE T ANTIGEN
54:00:00	8hwa-A	7.6	4.2	226	700	10	MOLECULE:	PRIMASE D5
55:00:00	4ijd-A	7.6	4.8	118	215	14	MOLECULE:	HISTONE-LYSINE N-METHYLTRANSFERASE PRDM9
56:00:00	7cro-I	7.6	2.6	113	220	22	MOLECULE:	HISTONE H3
57:00:00	2h2e-C	7.6	22.7	123	440	13	MOLECULE:	RIBULOSE-1,5 BISPHOSPHATE CARBOXYLASE/OXYGENASE L
58:00:00	8hr9-K	7.6	15.3	150	836	10	MOLECULE:	ARCHAEAL ATPASE
59:00:00	3ihx-A	7.6	2.7	96	126	15	MOLECULE:	PR DOMAIN ZINC FINGER PROTEIN 10
60:00:00	2jv0-A	7.6	3.4	108	163	16	MOLECULE:	PR DOMAIN ZINC FINGER PROTEIN 2
61:00:00	6hz9-L	7.6	3.7	137	285	11	MOLECULE:	5-METHYLCYTOSINE-SPECIFIC RESTRICTION ENZYME B
62:00:00	8hwa-E	7.6	6.2	208	379	10	MOLECULE:	PRIMASE D5
63:00:00	7w46-A	7.6	6.8	176	396	10	MOLECULE:	UNCHARACTERIZED ATPASE YJOB
64:00:00	7xe0-A	7.6	24.6	165	838	15	MOLECULE:	CNL9
65:00:00	6hz5-J	7.6	3.7	138	284	12	MOLECULE:	5-METHYLCYTOSINE-SPECIFIC RESTRICTION ENZYME B
66:00:00	7xe0-G	7.6	22.8	163	838	15	MOLECULE:	CNL9
67:00:00	4rh7-A	7.6	18.3	217	3005	8	MOLECULE:	GREEN FLUORESCENT PROTEIN/CYTOPLASMIC DYNEIN 2 HE
68:00:00	5bk4-3	7.6	7.8	244	604	7	MOLECULE:	DNA REPLICATION LICENSING FACTOR MCM2
69:00:00	7xe0-I	7.6	24.5	165	838	15	MOLECULE:	CNL9
70:00:00	6rn2-B	7.6	5	187	689	16	MOLECULE:	CHAPERONE PROTEIN CLPB
71:00:00	8btg-B	7.6	12.7	140	335	12	MOLECULE:	CHROMOSOMAL REPLICATION INITIATOR PROTEIN DNAA
72:00:00	3m6a-A	7.6	10.6	166	496	14	MOLECULE:	ATP-DEPENDENT PROTEASE LA 1
73:00:00	6qi9-A	7.6	3.4	142	317	11	MOLECULE:	RUVB-LIKE 1
74:00:00	6hz9-F	7.6	3.7	137	285	11	MOLECULE:	5-METHYLCYTOSINE-SPECIFIC RESTRICTION ENZYME B
75:00:00	2nl8-A	7.6	3.2	99	117	8	MOLECULE:	18-NT PEN ELEMENT OF THE SV40 DNA ORIGIN
76:00:00	3qn2-A	7.6	3.3	105	126	9	MOLECULE:	LARGE T ANTIGEN
77:00:00	4fb3-B	7.6	3.1	100	114	11	MOLECULE:	ORI DNA OLIGONUCLEOTIDE-CRICK STRAND
78:00:00	7crc-B	7.5	8.9	177	1112	10	MOLECULE:	NAD+ HYDROLASE (NADASE)
79:00:00	2g46-A	7.5	3	97	120	16	MOLECULE:	PBCV-1 HISTONE H3-LYS 27 METHYLTRANSFERASE
80:00:00	8v48-A	7.5	3.5	109	412	15	MOLECULE:	ARIA ANTITOXIN
81:00:00	5v8f-6	7.5	5.9	231	692	10	MOLECULE:	DNA REPLICATION LICENSING FACTOR MCM2
82:00:00	6uks-G	7.5	4.3	153	362	15	MOLECULE:	MITOCHONDRIAL CHAPERONE BCS1
83:00:00	6rn4-E	7.5	10.5	190	684	13	MOLECULE:	CHAPERONE PROTEIN CLPB
84:00:00	6uks-B	7.5	3.9	149	362	16	MOLECULE:	MITOCHONDRIAL CHAPERONE BCS1
85:00:00	5udb-7	7.5	6.7	236	726	10	MOLECULE:	DNA REPLICATION LICENSING FACTOR MCM2
86:00:00	7tfh-A	7.5	19.1	181	628	9	MOLECULE:	REPLICATION FACTOR C SUBUNIT 1
87:00:00	8hwa-F	7.5	5	272	700	9	MOLECULE:	PRIMASE D5
88:00:00	8hwb-D	7.5	10.5	263	700	10	MOLECULE:	PRIMASE D5
89:00:00	7w42-A	7.5	7.2	182	397	13	MOLECULE:	UNCHARACTERIZED ATPASE YJOB
90:00:00	5grb-B	7.5	4	128	212	8	MOLECULE:	EV71 2C ATPASE
91:00:00	7xe0-E	7.5	24.5	167	838	14	MOLECULE:	CNL9
92:00:00	8v45-F	7.5	3.2	107	378	14	MOLECULE:	ARIA ANTITOXIN
93:00:00	6hz4-A	7.5	3.8	137	290	12	MOLECULE:	5-METHYLCYTOSINE-SPECIFIC RESTRICTION ENZYME B

94:00:00	8ui7-A	7.5	17.9	167	643	10 MOLECULE:	ATPASE FAMILY AAA DOMAIN-CONTAINING PROTEIN 5
95:00:00	6rn2-A	7.5	4.4	191	684	14 MOLECULE:	CHAPERONE PROTEIN CLPB
96:00:00	8dqx-A	7.5	10.6	144	497	13 MOLECULE:	REPLICATION FACTOR C SUBUNIT 1
97:00:00	1ixs-B	7.5	10.1	146	315	13 MOLECULE:	HOLLIDAY JUNCTION DNA HELICASE RUVA
98:00:00	2xsz-D	7.5	3.3	138	310	12 MOLECULE:	RUVB-LIKE 1
99:00:00	2xsz-F	7.5	3.2	139	311	14 MOLECULE:	RUVB-LIKE 1
0:00	3uk6-G	7.5	3.7	148	330	12 MOLECULE:	RUVB-LIKE 2
1:00	3glf-D	7.5	12.4	144	362	12 MOLECULE:	DNA POLYMERASE III SUBUNIT DELTA
2:00	3gli-I	7.5	12.4	152	362	12 MOLECULE:	DNA POLYMERASE III SUBUNIT DELTA
3:00	6hz4-D	7.5	3.7	138	284	12 MOLECULE:	5-METHYLCYTOSINE-SPECIFIC RESTRICTION ENZYME B
4:00	6etx-B	7.5	8.7	142	429	13 MOLECULE:	RUVB-LIKE 1
5:00	6pek-A	7.5	6.9	132	283	9 MOLECULE:	SPASTIN
6:00	6etx-F	7.5	8.8	143	434	12 MOLECULE:	RUVB-LIKE 1
7:00	8ui9-A	7.5	23.7	169	643	9 MOLECULE:	ATPASE FAMILY AAA DOMAIN-CONTAINING PROTEIN 5
8:00	6pen-A	7.5	6.9	132	283	9 MOLECULE:	SPASTIN
9:00	6qs8-F	7.5	15.3	213	573	12 MOLECULE:	CHAPERONE PROTEIN CLPB
10:00	2ntc-A	7.5	3.4	106	127	8 MOLECULE:	21-NT PEN ELEMENT OF THE SV40 DNA ORIGIN
11:00	4fb3-A	7.5	3	97	115	11 MOLECULE:	ORI DNA OLIGONUCLEOTIDE-CRICK STRAND
12:00	2itl-A	7.5	3.3	101	120	8 MOLECULE:	24-NT PEN ELEMENT OF THE SV40 DNA ORIGIN
13:00	2ipr-A	7.5	3.3	103	127	8 MOLECULE:	LARGE T ANTIGEN
14:00	4nbp-A	7.5	3.6	108	129	9 MOLECULE:	LARGE T ANTIGEN
15:00	4fb3-E	7.5	3.2	99	114	11 MOLECULE:	ORI DNA OLIGONUCLEOTIDE-CRICK STRAND
16:00	4lmd-B	7.5	3.7	108	130	10 MOLECULE:	LARGE T ANTIGEN
17:00	4lmd-A	7.5	3.7	110	128	9 MOLECULE:	LARGE T ANTIGEN
18:00	6fnd-A	7.4	5.3	109	423	13 MOLECULE:	APICAL COMPLEX LYSINE METHYLTRANSFERASE
19:00	8hr9-E	7.4	10.3	148	833	8 MOLECULE:	ARCHAEAL ATPASE
20:00	7wbb-A	7.4	13.1	188	726	7 MOLECULE:	AFG2 ISOFORM 1
21:00	3ihx-B	7.4	3	100	129	14 MOLECULE:	PR DOMAIN ZINC FINGER PROTEIN 10
22:00	8hr9-D	7.4	15	146	836	8 MOLECULE:	ARCHAEAL ATPASE
23:00	7m9b-D	7.4	9.2	135	257	10 MOLECULE:	TNSC
24:00:00	4ijd-B	7.4	5.4	121	215	14 MOLECULE:	HISTONE-LYSINE N-METHYLTRANSFERASE PRDM9
25:00:00	3tg4-A	7.4	3.6	118	428	12 MOLECULE:	N-LYSINE METHYLTRANSFERASE SMYD2
26:00:00	6hz7-F	7.4	3.8	137	285	11 MOLECULE:	5-METHYLCYTOSINE-SPECIFIC RESTRICTION ENZYME B
27:00:00	7swl-A	7.4	8.9	159	553	11 MOLECULE:	RIX7
28:00:00	6l1q-A	7.4	6.7	138	267	11 MOLECULE:	CBBQ PROTEIN
29:00:00	5m7o-A	7.4	13.3	162	448	9 MOLECULE:	NITROGEN ASSIMILATION REGULATORY PROTEIN
30:00:00	3pvs-D	7.4	11.2	139	424	12 MOLECULE:	REPLICATION-ASSOCIATED RECOMBINATION PROTEIN A
31:00:00	8dr3-A	7.4	15.5	168	643	11 MOLECULE:	REPLICATION FACTOR C SUBUNIT 1
32:00:00	8hwh-A	7.4	4.1	198	353	12 MOLECULE:	PRIMASE D5
33:00:00	2uz3-B	7.4	9.8	111	207	9 MOLECULE:	THYMIDINE KINASE
34:00:00	8hwe-E	7.4	4	200	372	10 MOLECULE:	PRIMASE D5
35:00:00	5vqa-A	7.4	4.2	156	368	13 MOLECULE:	PACHYTENE CHECKPOINT PROTEIN 2 HOMOLOG
36:00:00	6hz7-L	7.4	3.7	135	285	12 MOLECULE:	5-METHYLCYTOSINE-SPECIFIC RESTRICTION ENZYME B
37:00:00	1sxj-B	7.4	13.3	148	316	12 MOLECULE:	ACTIVATOR 1 95 KDA SUBUNIT
38:00:00	6rn2-C	7.4	5.6	180	689	14 MOLECULE:	CHAPERONE PROTEIN CLPB
39:00:00	5bk4-7	7.4	5.5	219	689	10 MOLECULE:	DNA REPLICATION LICENSING FACTOR MCM2
40:00:00	1iqp-A	7.4	16	157	326	13 MOLECULE:	RFCS
41:00:00	4xgu-D	7.4	4.5	168	377	13 MOLECULE:	PUTATIVE PACHYTENE CHECKPOINT PROTEIN 2
42:00:00	2xsz-E	7.4	3.2	139	315	14 MOLECULE:	RUVB-LIKE 1
43:00:00	3gli-D	7.4	9.8	150	362	11 MOLECULE:	DNA POLYMERASE III SUBUNIT DELTA
44:00:00	2x8a-A	7.4	6.9	133	237	14 MOLECULE:	NUCLEAR VALOSIN-CONTAINING PROTEIN-LIKE
45:00:00	6hz5-L	7.4	3.7	137	285	11 MOLECULE:	5-METHYLCYTOSINE-SPECIFIC RESTRICTION ENZYME B
46:00:00	3bos-B	7.4	3.5	133	231	13 MOLECULE:	PUTATIVE DNA REPLICATION FACTOR

47:00:00	4fgn-B	7.4	3.7	109	124	7	MOLECULE:	LARGE T ANTIGEN
48:00:00	5d9i-B	7.4	3.4	105	132	7	MOLECULE:	LARGE T ANTIGEN
49:00:00	5cyn-A	7.4	3.4	106	127	8	MOLECULE:	LARGE T ANTIGEN
50:00:00	2ntc-B	7.4	3.4	106	126	8	MOLECULE:	21-NT PEN ELEMENT OF THE SV40 DNA ORIGIN
51:00:00	2if9-A	7.4	3.4	106	132	8	MOLECULE:	LARGE T ANTIGEN
52:00:00	5d9i-A	7.4	3.5	106	133	8	MOLECULE:	LARGE T ANTIGEN
53:00:00	3qk2-A	7.4	3.5	106	132	8	MOLECULE:	LARGE T ANTIGEN
54:00:00	6hz5-C	7.3	3.7	139	290	11	MOLECULE:	5-METHYLCYTOSINE-SPECIFIC RESTRICTION ENZYME B
55:00:00	7t0v-D	7.3	9.5	143	570	13	MOLECULE:	RIX7
56:00:00	6sh3-D	7.3	14.6	170	376	11	MOLECULE:	MITOCHONDRIAL CHAPERONE BCS1
57:00:00	6uks-C	7.3	4.1	154	362	16	MOLECULE:	MITOCHONDRIAL CHAPERONE BCS1
58:00:00	7upr-A	7.3	9.4	134	235	13	MOLECULE:	OUTER MITOCHONDRIAL TRANSMEMBRANE HELIX TRANSLOCA
59:00:00	6hz4-F	7.3	3.8	137	285	11	MOLECULE:	5-METHYLCYTOSINE-SPECIFIC RESTRICTION ENZYME B
60:00:00	5j1s-A	7.3	3.1	142	277	14	MOLECULE:	TORSIN-1A
61:00:00	6pod-A	7.3	10.8	150	329	15	MOLECULE:	ATP-DEPENDENT CLP PROTEASE ATP-BINDING SUBUNIT CL
62:00:00	8hwb-F	7.3	4.4	205	379	11	MOLECULE:	PRIMASE D5
63:00:00	6hz6-J	7.3	3.7	137	284	11	MOLECULE:	5-METHYLCYTOSINE-SPECIFIC RESTRICTION ENZYME B
64:00:00	8hwc-A	7.3	6.8	207	378	12	MOLECULE:	PRIMASE D5
65:00:00	6rn3-C	7.3	4.1	189	689	14	MOLECULE:	CHAPERONE PROTEIN CLPB
66:00:00	6l8d-A	7.3	4.3	148	267	13	MOLECULE:	MAGNESIUM-CHELATASE SUBUNIT CHLI
67:00:00	6oay-C	7.3	13.9	154	570	9	MOLECULE:	HYPERACTIVE DISAGGREGASE CLPB
68:00:00	4xgu-A	7.3	5.7	162	376	14	MOLECULE:	PUTATIVE PACHYTENE CHECKPOINT PROTEIN 2
69:00:00	5ifw-B	7.3	17.5	175	702	9	MOLECULE:	TETHER CONTAINING UBX DOMAIN FOR GLUT4
70:00:00	2rko-A	7.3	14	144	280	13	MOLECULE:	VACUOLAR PROTEIN SORTING-ASSOCIATED PROTEIN 4
71:00:00	3glg-C	7.3	12	141	365	11	MOLECULE:	DNA POLYMERASE III SUBUNIT DELTA
72:00:00	3glf-I	7.3	12	142	362	12	MOLECULE:	DNA POLYMERASE III SUBUNIT DELTA
73:00:00	3glg-I	7.3	9.4	145	362	11	MOLECULE:	DNA POLYMERASE III SUBUNIT DELTA
74:00:00	6vvo-E	7.3	3.2	127	346	9	MOLECULE:	REPLICATION FACTOR C SUBUNIT 1
75:00:00	3glf-H	7.3	9.8	140	365	11	MOLECULE:	DNA POLYMERASE III SUBUNIT DELTA
76:00:00	3gli-C	7.3	11.8	143	365	11	MOLECULE:	DNA POLYMERASE III SUBUNIT DELTA
77:00:00	3gli-H	7.3	11.6	143	365	12	MOLECULE:	DNA POLYMERASE III SUBUNIT DELTA
78:00:00	3glg-H	7.3	11.8	140	365	12	MOLECULE:	DNA POLYMERASE III SUBUNIT DELTA
79:00:00	3glg-D	7.3	12.7	141	362	11	MOLECULE:	DNA POLYMERASE III SUBUNIT DELTA
80:00:00	3glf-C	7.3	11.6	141	365	12	MOLECULE:	DNA POLYMERASE III SUBUNIT DELTA
81:00:00	1sxj-A	7.3	4.1	142	441	13	MOLECULE:	ACTIVATOR 1 95 KDA SUBUNIT
82:00:00	1w4r-A	7.3	3.5	98	174	10	MOLECULE:	THYMIDINE KINASE
83:00:00	6pen-C	7.3	10.1	137	288	11	MOLECULE:	SPASTIN
84:00:00	6pek-C	7.3	10.1	137	288	11	MOLECULE:	SPASTIN
85:00:00	6pen-F	7.3	11.8	140	272	11	MOLECULE:	SPASTIN
86:00:00	6p8v-F	7.3	3.6	138	270	15	MOLECULE:	ATPASE, AAA FAMILY
87:00:00	6hz6-D	7.3	3.7	137	284	11	MOLECULE:	5-METHYLCYTOSINE-SPECIFIC RESTRICTION ENZYME B
88:00:00	4lif-A	7.3	3.6	107	127	7	MOLECULE:	LARGE T ANTIGEN
89:00:00	2if9-B	7.3	3.7	107	131	7	MOLECULE:	LARGE T ANTIGEN
90:00:00	2itj-A	7.3	3.3	105	128	8	MOLECULE:	LARGE T ANTIGEN
91:00:00	2itl-B	7.3	3.3	100	115	8	MOLECULE:	24-NT PEN ELEMENT OF THE SV40 DNA ORIGIN
92:00:00	3qfq-A	7.3	3.5	102	119	7	MOLECULE:	LARGE T ANTIGEN
93:00:00	7vcs-A	7.2	7	180	766	8	MOLECULE:	TRANSITIONAL ENDOPLASMIC RETICULUM ATPASE
94:00:00	2h21-B	7.2	16.4	123	441	12	MOLECULE:	RIBULOSE-1,5 BISPHOSPHATE CARBOXYLASE/OXYGENASE
95:00:00	8hr8-A	7.2	15.6	149	836	9	MOLECULE:	ARCHAEAL ATPASE
96:00:00	6j6i-O	7.2	21.9	150	790	11	MOLECULE:	PROBABLE SERINE/THREONINE-PROTEIN KINASE PBL2
97:00:00	8hrb-B	7.2	15.5	144	835	8	MOLECULE:	ARCHAEAL ATPASE
98:00:00	6hz6-F	7.2	3.7	137	285	11	MOLECULE:	5-METHYLCYTOSINE-SPECIFIC RESTRICTION ENZYME B
99:00:00	6sh4-F	7.2	6.3	141	376	14	MOLECULE:	MITOCHONDRIAL CHAPERONE BCS1

0:00	5e7p-B	7.2	16	165	704	11	MOLECULE:	CELL DIVISION CONTROL PROTEIN CDC48
1:00	5bq5-B	7.2	3.5	126	187	14	MOLECULE:	INSERTION SEQUENCE IS5376 PUTATIVE ATP-BINDING PR
2:00	7lar-A	7.2	4.9	127	183	17	MOLECULE:	ATP-DEPENDENT HELICASE REP
3:00	5ex0-A	7.2	4.4	125	430	14	MOLECULE:	HISTONE-LYSINE N-METHYLTRANSFERASE SMYD3
4:00	6rn3-A	7.2	12.7	153	684	8	MOLECULE:	CHAPERONE PROTEIN CLPB
5:00	8q6p-2	7.2	4.9	176	431	9	MOLECULE:	DNA REPLICATION LICENSING FACTOR MCM2
6:00	1sxj-D	7.2	14.5	140	328	11	MOLECULE:	ACTIVATOR 1 95 KDA SUBUNIT
7:00	3glg-B	7.2	14.8	141	364	14	MOLECULE:	DNA POLYMERASE III SUBUNIT DELTA
8:00	8jx6-A	7.2	6.8	128	447	10	MOLECULE:	DEEP-SEA HELICASE 9
9:00	5ji3-E	7.2	11.4	159	375	13	MOLECULE:	ATP-DEPENDENT PROTEASE SUBUNIT HSLV
10:00	6hz6-L	7.2	3.7	137	285	11	MOLECULE:	5-METHYLCYTOSINE-SPECIFIC RESTRICTION ENZYME B
11:00	6hz4-C	7.2	3.8	139	290	11	MOLECULE:	5-METHYLCYTOSINE-SPECIFIC RESTRICTION ENZYME B
12:00	6hz7-J	7.2	3.7	137	284	11	MOLECULE:	5-METHYLCYTOSINE-SPECIFIC RESTRICTION ENZYME B
13:00	7y53-C	7.2	7.8	187	741	11	MOLECULE:	DERLIN-1
14:00	7qv9-d	7.2	7.3	148	256	9	MOLECULE:	DNA-DIRECTED RNA POLYMERASE SUBUNIT ALPHA
15:00	3zuh-D	7.2	12.3	143	286	10	MOLECULE:	PROTEIN CBBX
16:00	4xgc-E	7.2	8.6	136	402	10	MOLECULE:	ORIGIN RECOGNITION COMPLEX SUBUNIT 2
17:00	6qs8-A	7.2	7.9	210	568	14	MOLECULE:	CHAPERONE PROTEIN CLPB
18:00	3qfq-E	7.2	3.3	100	117	6	MOLECULE:	LARGE T ANTIGEN
19:00	3qfq-B	7.2	3.5	106	120	8	MOLECULE:	LARGE T ANTIGEN
20:00	7sh2-A	7.1	11.3	163	405	12	MOLECULE:	CHECKPOINT PROTEIN RAD24
21:00	8hr9-J	7.1	15.7	147	835	7	MOLECULE:	ARCHAEAL ATPASE
22:00	7t3i-F	7.1	9.5	167	552	12	MOLECULE:	RIX7
23:00	8hr8-F	7.1	15.6	145	835	10	MOLECULE:	ARCHAEAL ATPASE
24:00:00	8fcw-V	7.1	7.9	140	343	9	MOLECULE:	TNSC
25:00:00	8fcx-U	7.1	8.1	147	343	7	MOLECULE:	TNSC
26:00:00	7bj1-A	7.1	3.8	121	427	14	MOLECULE:	HISTONE-LYSINE N-METHYLTRANSFERASE SMYD3
27:00:00	6rn3-B	7.1	9.1	195	689	12	MOLECULE:	CHAPERONE PROTEIN CLPB
28:00:00	3glf-G	7.1	16.3	158	378	13	MOLECULE:	DNA POLYMERASE III SUBUNIT DELTA
29:00:00	7st9-E	7.1	3.2	129	354	11	MOLECULE:	CHECKPOINT PROTEIN RAD24
30:00:00	8xj7-D	7.1	6.5	218	452	10	MOLECULE:	MONKEYPOX VIRUS E5
31:00:00	6rn4-A	7.1	12.9	151	675	11	MOLECULE:	CHAPERONE PROTEIN CLPB
32:00:00	6opc-F	7.1	14.6	187	681	9	MOLECULE:	CELL DIVISION CONTROL PROTEIN 48
33:00:00	5vca-P	7.1	10.4	177	543	8	MOLECULE:	VCP-LIKE ATPASE
34:00:00	3glh-G	7.1	11.1	145	364	10	MOLECULE:	DNA POLYMERASE III SUBUNIT DELTA
35:00:00	3glf-B	7.1	13.6	146	364	12	MOLECULE:	DNA POLYMERASE III SUBUNIT DELTA
36:00:00	3glg-G	7.1	16.4	159	378	13	MOLECULE:	DNA POLYMERASE III SUBUNIT DELTA
37:00:00	6pen-D	7.1	9.2	140	293	11	MOLECULE:	SPASTIN
38:00:00	4w5w-A	7.1	12.7	147	269	12	MOLECULE:	RIBULOSE BISPHOSPHATE CARBOXYLASE/OXYGENASE ACTIV
39:00:00	6pek-D	7.1	9.2	140	293	11	MOLECULE:	SPASTIN
40:00:00	6igm-F	7.1	4.7	141	366	13	MOLECULE:	RUVB-LIKE 1
41:00:00	5bk4-5	7.1	5.6	199	661	9	MOLECULE:	DNA REPLICATION LICENSING FACTOR MCM2
42:00:00	6hz5-D	7.1	3.7	138	284	12	MOLECULE:	5-METHYLCYTOSINE-SPECIFIC RESTRICTION ENZYME B
43:00:00	3zuh-B	7.1	11.3	138	286	10	MOLECULE:	PROTEIN CBBX
44:00:00	2itj-B	7.1	3.2	96	118	8	MOLECULE:	LARGE T ANTIGEN
45:00:00	3j3r-A	7	19.1	227	798	13	MOLECULE:	ADAPTER PROTEIN MECA 1
46:00:00	7stb-A	7	7.6	153	515	15	MOLECULE:	CHECKPOINT PROTEIN RAD24
47:00:00	8fcw-S	7	6.5	141	343	9	MOLECULE:	TNSC
48:00:00	3ihx-D	7	2.9	89	116	12	MOLECULE:	PR DOMAIN ZINC FINGER PROTEIN 10
49:00:00	7y4w-C	7	8	191	744	11	MOLECULE:	DERLIN-1
50:00:00	6f0x-B	7	3.9	155	398	13	MOLECULE:	PACHYTENE CHECKPOINT PROTEIN 2 HOMOLOG
51:00:00	6qi8-E	7	8.7	150	335	11	MOLECULE:	RUVB-LIKE 1
52:00:00	4ypl-C	7	8.7	189	538	13	MOLECULE:	LON PROTEASE

53:00:00	8osg-A	7	4.8	162	283	10	MOLECULE:	MAGNESIUM-CHELATASE SUBUNIT CHLI
54:00:00	8fcv-R	7	7.9	149	343	8	MOLECULE:	DNA (60-MER)
55:00:00	6j6i-F	7	23.2	156	790	8	MOLECULE:	PROBABLE SERINE/THREONINE-PROTEIN KINASE PBL2
56:00:00	6j5t-G	7	24.8	140	808	9	MOLECULE:	PROBABLE SERINE/THREONINE-PROTEIN KINASE PBL2
57:00:00	6oaa-D	7	5.8	156	537	12	MOLECULE:	CELL DIVISION CONTROL PROTEIN 48
58:00:00	6j6i-G	7	23.9	155	790	8	MOLECULE:	PROBABLE SERINE/THREONINE-PROTEIN KINASE PBL2
59:00:00	5vc7-F	7	9.6	179	544	10	MOLECULE:	VCP-LIKE ATPASE
60:00:00	1sxj-C	7	13	143	322	10	MOLECULE:	ACTIVATOR 1 95 KDA SUBUNIT
61:00:00	6oay-B	7	11.7	134	570	10	MOLECULE:	HYPERACTIVE DISAGGREGASE CLPB
62:00:00	5vc7-C	7	9.6	179	544	10	MOLECULE:	VCP-LIKE ATPASE
63:00:00	5vc7-D	7	9.5	176	544	10	MOLECULE:	VCP-LIKE ATPASE
64:00:00	3cmw-A	7	15.3	145	1609	12	MOLECULE:	DNA (5'-
65:00:00	3gli-B	7	14.8	147	359	13	MOLECULE:	DNA POLYMERASE III SUBUNIT DELTA
66:00:00	3glh-M	7	13.5	150	364	11	MOLECULE:	DNA POLYMERASE III SUBUNIT DELTA
67:00:00	1mo4-A	7	10.4	147	324	12	MOLECULE:	RECA
68:00:00	6pen-E	7	3.5	127	288	9	MOLECULE:	SPASTIN
69:00:00	6pxk-K	7	11.4	159	429	12	MOLECULE:	ATP-DEPENDENT PROTEASE ATPASE SUBUNIT HSLU
70:00:00	6pek-E	7	3.6	129	243	9	MOLECULE:	SPASTIN
71:00:00	3vfd-A	7	11.1	135	266	12	MOLECULE:	SPASTIN
72:00:00	6qs8-B	7	6.1	192	582	12	MOLECULE:	CHAPERONE PROTEIN CLPB
73:00:00	4fgn-A	7	3.4	102	126	8	MOLECULE:	LARGE T ANTIGEN
74:00:00	6dgd-A	6.9	21.2	166	704	10	MOLECULE:	PRIMOSOMAL PROTEIN N'
75:00:00	7m9c-G	6.9	9.7	135	257	9	MOLECULE:	TNSC
76:00:00	8hr9-F	6.9	14.9	144	835	8	MOLECULE:	ARCHAEAL ATPASE
77:00:00	7m9a-G	6.9	9.7	136	257	9	MOLECULE:	TNSC
78:00:00	7m9a-H	6.9	9.5	138	257	9	MOLECULE:	TNSC
79:00:00	8fcv-T	6.9	8.6	150	343	9	MOLECULE:	DNA (60-MER)
80:00:00	7m9c-P	6.9	9.7	135	257	9	MOLECULE:	TNSC
81:00:00	8hr8-E	6.9	10	147	833	8	MOLECULE:	ARCHAEAL ATPASE
82:00:00	8hr8-G	6.9	14.8	151	830	9	MOLECULE:	ARCHAEAL ATPASE
83:00:00	7m9b-G	6.9	9.6	138	257	9	MOLECULE:	TNSC
84:00:00	7ykl-F	6.9	9.4	207	735	9	MOLECULE:	ATPASE FAMILY GENE 2 PROTEIN
85:00:00	8owo-A	6.9	3.8	121	427	14	MOLECULE:	HISTONE-LYSINE N-METHYLTRANSFERASE SMYD3
86:00:00	6nyy-C	6.9	3.4	128	491	12	MOLECULE:	AFG3-LIKE PROTEIN 2
87:00:00	8apm-C	6.9	6.2	221	449	11	MOLECULE:	PRIMASE D5
88:00:00	6sh3-A	6.9	14.8	164	376	10	MOLECULE:	MITOCHONDRIAL CHAPERONE BCS1
89:00:00	6zyy-C	6.9	20.5	216	2901	6	MOLECULE:	DYNEIN HEAVY CHAIN, OUTER ARM PROTEIN
90:00:00	3n70-A	6.9	3.2	117	142	9	MOLECULE:	TRANSPORT ACTIVATOR
91:00:00	6sh3-F	6.9	5.9	146	376	14	MOLECULE:	MITOCHONDRIAL CHAPERONE BCS1
92:00:00	6hz8-F	6.9	3.7	136	285	11	MOLECULE:	5-METHYLCYTOSINE-SPECIFIC RESTRICTION ENZYME B
93:00:00	2is6-A	6.9	16.6	136	654	6	MOLECULE:	5'-D(*CP*GP*AP*GP*CP*AP*CP*TP*GP*CP*AP*GP*TP*GP*C
94:00:00	6rn3-D	6.9	4.3	178	684	16	MOLECULE:	CHAPERONE PROTEIN CLPB
95:00:00	6og2-A	6.9	7.3	192	548	13	MOLECULE:	HYPERACTIVE DISAGGREGASE CLPB
96:00:00	6j6i-L	6.9	24	158	790	8	MOLECULE:	PROBABLE SERINE/THREONINE-PROTEIN KINASE PBL2
97:00:00	6j5t-O	6.9	24.5	134	808	9	MOLECULE:	PROBABLE SERINE/THREONINE-PROTEIN KINASE PBL2
98:00:00	6j6i-C	6.9	21.7	162	790	9	MOLECULE:	PROBABLE SERINE/THREONINE-PROTEIN KINASE PBL2
99:00:00	6j5t-C	6.9	24.5	137	808	9	MOLECULE:	PROBABLE SERINE/THREONINE-PROTEIN KINASE PBL2
0:00	3j96-A	6.9	17.6	183	678	11	MOLECULE:	VESICLE-FUSING ATPASE
1:00	5w0t-A	6.9	5.5	144	298	15	MOLECULE:	PROTEIN MSP1
2:00	3glh-I	6.9	13.8	148	363	11	MOLECULE:	DNA POLYMERASE III SUBUNIT DELTA
3:00	3glh-H	6.9	13.3	137	364	12	MOLECULE:	DNA POLYMERASE III SUBUNIT DELTA
4:00	4wvy-B	6.9	11.1	144	408	12	MOLECULE:	RUVB-LIKE 1
5:00	4hse-A	6.9	11	148	369	11	MOLECULE:	CHAPERONE PROTEIN CLPB

6:00	2c9o-A	6.9	3.1	139	398	11	MOLECULE:	RUVB-LIKE 1
7:00	1e94-E	6.9	11.7	157	408	14	MOLECULE:	HEAT SHOCK PROTEIN HSLV
8:00	5oaf-B	6.9	8.2	143	418	13	MOLECULE:	RUVB-LIKE 1
9:00	6uks-E	6.9	4.2	154	362	15	MOLECULE:	MITOCHONDRIAL CHAPERONE BCS1
10:00	8t9f-K	6.8	6.3	117	265	11	MOLECULE:	HISTONE-LYSINE N-METHYLTRANSFERASE KMT5B
11:00	3n71-A	6.8	20.9	143	469	16	MOLECULE:	HISTONE LYSINE METHYLTRANSFERASE SMYD1
12:00	6p6k-A	6.8	3.8	121	425	15	MOLECULE:	HISTONE-LYSINE N-METHYLTRANSFERASE SMYD3
13:00	7w42-B	6.8	23.3	176	402	16	MOLECULE:	UNCHARACTERIZED ATPASE YJOB
14:00	7m9c-H	6.8	8.9	133	257	11	MOLECULE:	TNSC
15:00	8osg-B	6.8	4.3	167	323	10	MOLECULE:	MAGNESIUM-CHELATASE SUBUNIT CHLI
16:00	7ufi-4	6.8	10.5	149	311	7	MOLECULE:	DNA (5'-
17:00	7m9b-H	6.8	9.5	138	257	9	MOLECULE:	TNSC
18:00	8fcx-R	6.8	8	148	343	8	MOLECULE:	TNSC
19:00	7ufi-1	6.8	10.5	150	311	8	MOLECULE:	DNA (5'-
20:00	5hq8-B	6.8	3.9	121	426	14	MOLECULE:	HISTONE-LYSINE N-METHYLTRANSFERASE SMYD3
21:00	6fnd-D	6.8	6.3	112	422	13	MOLECULE:	APICAL COMPLEX LYSINE METHYLTRANSFERASE
22:00	6p8v-C	6.8	3.7	140	303	16	MOLECULE:	ATPASE, AAA FAMILY
23:00	7tts-C	6.8	12.1	170	456	14	MOLECULE:	CASEINOLYTIC PEPTIDASE B PROTEIN HOMOLOG
24:00:00	6oab-D	6.8	8.6	140	537	11	MOLECULE:	CELL DIVISION CONTROL PROTEIN 48
25:00:00	7z6h-D	6.8	14.7	136	324	10	MOLECULE:	CELL CYCLE CHECKPOINT CONTROL PROTEIN RAD9A
26:00:00	3j3t-F	6.8	10.1	193	798	13	MOLECULE:	ADAPTER PROTEIN MECA 1
27:00:00	5ftn-A	6.8	11.6	192	737	12	MOLECULE:	TRANSITIONAL ENDOPLASMIC RETICULUM ATPASE
28:00:00	8hwg-C	6.8	4.1	203	372	9	MOLECULE:	PRIMASE D5
29:00:00	1sxj-E	6.8	11.7	134	317	9	MOLECULE:	ACTIVATOR 1 95 KDA SUBUNIT
30:00:00	7tjj-I	6.8	16.6	144	380	14	MOLECULE:	ORIGIN RECOGNITION COMPLEX SUBUNIT 1
31:00:00	6j5t-L	6.8	25.2	130	808	9	MOLECULE:	PROBABLE SERINE/THREONINE-PROTEIN KINASE PBL2
32:00:00	8jx6-B	6.8	6.7	124	451	10	MOLECULE:	DEEP-SEA HELICASE 9
33:00:00	3b9p-A	6.8	9	132	268	11	MOLECULE:	CG5977-PA, ISOFORM A
34:00:00	5ln3-J	6.8	12.2	174	373	8	MOLECULE:	26S PROTEASOME NON-ATPASE REGULATORY SUBUNIT 2
35:00:00	3glh-C	6.8	12.5	140	364	11	MOLECULE:	DNA POLYMERASE III SUBUNIT DELTA
36:00:00	2xsz-B	6.8	3.3	142	319	11	MOLECULE:	RUVB-LIKE 1
37:00:00	3glh-B	6.8	14.8	144	364	10	MOLECULE:	DNA POLYMERASE III SUBUNIT DELTA
38:00:00	6p8v-A	6.8	3.6	140	299	15	MOLECULE:	ATPASE, AAA FAMILY
39:00:00	3t15-A	6.8	12.4	135	263	16	MOLECULE:	RIBULOSE BISPHOSPHATE CARBOXYLASE/OXYGENASE ACTIV
40:00:00	3s7d-A	6.8	3.8	118	430	11	MOLECULE:	N-LYSINE METHYLTRANSFERASE SMYD2
41:00:00	7ufi-5	6.7	10.2	150	311	8	MOLECULE:	DNA (5'-
42:00:00	3qwv-A	6.7	3.6	119	431	9	MOLECULE:	SET AND MYND DOMAIN-CONTAINING PROTEIN 2
43:00:00	8dgf-B	6.7	15.5	165	1534	7	MOLECULE:	ATP-BINDING PROTEIN AVS4
44:00:00	7mbw-C	6.7	13.6	147	461	11	MOLECULE:	TRANSPOSON TN7 TRANSPOSITION PROTEIN TNSC
45:00:00	8fcx-S	6.7	6.6	145	343	8	MOLECULE:	TNSC
46:00:00	8ff4-S	6.7	6.6	145	343	8	MOLECULE:	TYPE I-B CRISPR-ASSOCIATED PROTEIN CAS5
47:00:00	6oa9-D	6.7	8	166	489	8	MOLECULE:	CELL DIVISION CONTROL PROTEIN 48
48:00:00	1mlv-A	6.7	16.3	116	425	13	MOLECULE:	RIBULOSE-1,5 BIPHOSPHATE CARBOXYLASE/OXYGENASE LA
49:00:00	2qby-B	6.7	13.3	142	368	8	MOLECULE:	CELL DIVISION CONTROL PROTEIN 6 HOMOLOG 1
50:00:00	5udb-4	6.7	6.9	243	751	12	MOLECULE:	DNA REPLICATION LICENSING FACTOR MCM2
51:00:00	1g41-A	6.7	9.9	154	334	15	MOLECULE:	HEAT SHOCK PROTEIN HSLU
52:00:00	6sh5-D	6.7	4.5	148	334	13	MOLECULE:	MITOCHONDRIAL CHAPERONE BCS1
53:00:00	6j5t-F	6.7	25.9	132	808	11	MOLECULE:	PROBABLE SERINE/THREONINE-PROTEIN KINASE PBL2
54:00:00	6sh5-B	6.7	8.8	147	334	13	MOLECULE:	MITOCHONDRIAL CHAPERONE BCS1
55:00:00	6sh5-C	6.7	4.5	148	334	13	MOLECULE:	MITOCHONDRIAL CHAPERONE BCS1
56:00:00	6sh5-A	6.7	5.6	152	334	13	MOLECULE:	MITOCHONDRIAL CHAPERONE BCS1
57:00:00	4d80-F	6.7	10.1	139	274	13	MOLECULE:	AAA ATPASE, CENTRAL DOMAIN PROTEIN
58:00:00	4ww4-B	6.7	11.9	148	435	11	MOLECULE:	RUVB-LIKE 1

59:00:00	2xsz-A	6.7	3.3	143	318	11	MOLECULE:	RUVB-LIKE 1
60:00:00	3ecc-A	6.7	3.6	122	182	14	MOLECULE:	DNA REPLICATION PROTEIN DNAC
61:00:00	5xmi-A	6.7	12.9	137	319	12	MOLECULE:	VACUOLAR PROTEIN SORTING-ASSOCIATED PROTEIN 4
62:00:00	3glh-N	6.7	8.4	147	363	12	MOLECULE:	DNA POLYMERASE III SUBUNIT DELTA
63:00:00	3gli-G	6.7	16.4	158	373	12	MOLECULE:	DNA POLYMERASE III SUBUNIT DELTA
64:00:00	6p07-A	6.7	12.1	131	270	12	MOLECULE:	SPASTIN
65:00:00	2awn-D	6.7	8.6	119	301	15	MOLECULE:	MALTOSE/MALTODEXTRIN IMPORT ATP-BINDING PROTEIN M
66:00:00	3s7b-A	6.7	3.9	119	430	10	MOLECULE:	N-LYSINE METHYLTRANSFERASE SMYD2
67:00:00	7m9c-D	6.6	8.5	132	257	10	MOLECULE:	TNSC
68:00:00	8hrb-P	6.6	12	133	834	11	MOLECULE:	ARCHAEAL ATPASE
69:00:00	7ufi-2	6.6	10.1	150	311	8	MOLECULE:	DNA (5'-
70:00:00	8hr9-L	6.6	14.5	143	833	8	MOLECULE:	ARCHAEAL ATPASE
71:00:00	8fcx-T	6.6	7.9	148	343	9	MOLECULE:	TNSC
72:00:00	7m9a-F	6.6	8.3	129	257	10	MOLECULE:	TNSC
73:00:00	7m9c-F	6.6	9.4	138	257	9	MOLECULE:	TNSC
74:00:00	7m9a-I	6.6	9.2	134	257	10	MOLECULE:	TNSC
75:00:00	7m99-C	6.6	8.7	130	257	8	MOLECULE:	TNSC
76:00:00	7ufm-H	6.6	9.2	143	311	8	MOLECULE:	VCHTNSC
77:00:00	7m9b-I	6.6	9.2	134	257	10	MOLECULE:	TNSC
78:00:00	8fnt-G	6.6	14.6	132	785	8	MOLECULE:	ARCHAEAL ATPASE
79:00:00	8ff4-T	6.6	6.2	140	343	9	MOLECULE:	TYPE I-B CRISPR-ASSOCIATED PROTEIN CAS5
80:00:00	2h21-A	6.6	28.6	132	425	14	MOLECULE:	RIBULOSE-1,5 BISPHOSPHATE CARBOXYLASE/OXYGENASE
81:00:00	8fnt-F	6.6	15	150	785	8	MOLECULE:	ARCHAEAL ATPASE
82:00:00	6oaa-C	6.6	8.4	141	532	13	MOLECULE:	CELL DIVISION CONTROL PROTEIN 48
83:00:00	3oxg-A	6.6	3.8	121	425	13	MOLECULE:	SET AND MYND DOMAIN-CONTAINING PROTEIN 3
84:00:00	5nvu-A	6.6	17.1	221	3169	0	MOLECULE:	DYNEIN MOTOR DOMAIN
85:00:00	7ufi-6	6.6	10.5	149	311	8	MOLECULE:	DNA (5'-
86:00:00	8hwf-F	6.6	4.4	202	371	11	MOLECULE:	PRIMASE D5
87:00:00	5zr1-A	6.6	19.8	169	494	8	MOLECULE:	ORIGIN RECOGNITION COMPLEX SUBUNIT 1
88:00:00	5zr1-E	6.6	12.9	143	460	8	MOLECULE:	ORIGIN RECOGNITION COMPLEX SUBUNIT 1
89:00:00	6oa9-E	6.6	8.5	166	496	8	MOLECULE:	CELL DIVISION CONTROL PROTEIN 48
90:00:00	5mp9-H	6.6	14.7	160	390	11	MOLECULE:	PROTEASOME SUBUNIT ALPHA TYPE-1
91:00:00	4xgu-B	6.6	6.9	165	382	13	MOLECULE:	PUTATIVE PACHYTENE CHECKPOINT PROTEIN 2
92:00:00	6sh5-F	6.6	5.6	152	334	13	MOLECULE:	MITOCHONDRIAL CHAPERONE BCS1
93:00:00	6sh5-E	6.6	4.5	149	334	13	MOLECULE:	MITOCHONDRIAL CHAPERONE BCS1
94:00:00	5wc0-A	6.6	10.9	137	275	12	MOLECULE:	MEIOTIC SPINDLE FORMATION PROTEIN MEI-1
95:00:00	3glh-L	6.6	12.9	150	364	11	MOLECULE:	DNA POLYMERASE III SUBUNIT DELTA
96:00:00	6etx-E	6.6	4.5	139	443	12	MOLECULE:	RUVB-LIKE 1
97:00:00	6rn4-B	6.6	10.5	206	689	11	MOLECULE:	CHAPERONE PROTEIN CLPB
98:00:00	5jpq-i	6.6	28.9	133	659	11	MOLECULE:	WD40 DOMAIN PROTEINS
99:00:00	2h23-C	6.6	17.2	127	440	13	MOLECULE:	RIBULOSE-1,5 BISPHOSPHATE CARBOXYLASE/OXYGENASE L
0:00	3s7f-A	6.6	4.4	121	430	12	MOLECULE:	N-LYSINE METHYLTRANSFERASE SMYD2
1:00	8val-E	6.6	13.7	139	337	11	MOLECULE:	DNA POLYMERASE III SUBUNIT DELTA
2:00	6etx-D	6.6	5.5	140	434	14	MOLECULE:	RUVB-LIKE 1
3:00	7t3i-C	6.6	13.2	176	578	10	MOLECULE:	RIX7
4:00	1z1d-B	6.6	4	107	131	8	MOLECULE:	REPLICATION PROTEIN A 32 KDA SUBUNIT
5:00	8fcv-V	6.5	8	147	343	9	MOLECULE:	DNA (60-MER)
6:00	7m9c-O	6.5	9.1	133	257	10	MOLECULE:	TNSC
7:00	7m9a-A	6.5	9.2	136	257	10	MOLECULE:	TNSC
8:00	8hra-B	6.5	10.1	138	835	9	MOLECULE:	ARCHAEAL ATPASE
9:00	7st9-A	6.5	11	168	522	14	MOLECULE:	CHECKPOINT PROTEIN RAD24
10:00	8fcx-V	6.5	8.5	153	343	9	MOLECULE:	TNSC
11:00	8fcw-T	6.5	8.4	152	343	9	MOLECULE:	TNSC

12:00	7m9b-A	6.5	9.2	135	257	10	MOLECULE:	TNSC
13:00	8fnt-A	6.5	14.5	137	785	9	MOLECULE:	ARCHAEAL ATPASE
14:00	7m9c-A	6.5	9.1	135	257	10	MOLECULE:	TNSC
15:00	8c0v-C	6.5	12	170	821	8	MOLECULE:	PEROXISOMAL ATPASE PEX1
16:00	7ttr-B	6.5	10.1	159	311	13	MOLECULE:	CASEINOLYTIC PEPTIDASE B PROTEIN HOMOLOG
17:00	6oa9-F	6.5	7.2	168	533	10	MOLECULE:	CELL DIVISION CONTROL PROTEIN 48
18:00	7ufi-3	6.5	10.1	149	311	8	MOLECULE:	DNA (5'-
19:00	7ykk-F	6.5	7.9	208	735	9	MOLECULE:	ATPASE FAMILY GENE 2 PROTEIN
20:00	8hwa-C	6.5	3.9	200	379	9	MOLECULE:	PRIMASE D5
21:00	6epc-H	6.5	12.7	158	396	8	MOLECULE:	PROTEASOME SUBUNIT ALPHA TYPE-6
22:00	8osf-C	6.5	4.4	168	321	11	MOLECULE:	MAGNESIUM-CHELATASE SUBUNIT CHLI
23:00	6sh3-C	6.5	6	146	376	14	MOLECULE:	MITOCHONDRIAL CHAPERONE BCS1
24:00:00	1lv7-A	6.5	3.3	134	251	9	MOLECULE:	FTSH
25:00:00	1g64-B	6.5	8.9	113	190	15	MOLECULE:	COB(I)ALAMIN ADENOSYLTRANSFERASE
26:00:00	4hut-A	6.5	9.1	111	191	14	MOLECULE:	COB(I)YRINIC ACID A,C-DIAMIDE ADENOSYLTRANSFERASE
27:00:00	3glh-D	6.5	11.3	138	363	12	MOLECULE:	DNA POLYMERASE III SUBUNIT DELTA
28:00:00	7yup-E	6.5	11	156	773	12	MOLECULE:	LON PROTEASE
29:00:00	6pen-B	6.5	10.8	138	288	10	MOLECULE:	SPASTIN
30:00:00	6qs6-F	6.5	16.2	210	577	12	MOLECULE:	CHAPERONE PROTEIN CLPB
31:00:00	6pek-B	6.5	10.8	138	288	10	MOLECULE:	SPASTIN
32:00:00	8emc-F	6.5	6.8	188	666	9	MOLECULE:	PROTEASE LON-RELATED BREX SYSTEM PROTEIN BRXL
33:00:00	6xji-C	6.5	7.2	130	290	12	MOLECULE:	PHENOL-SOLUBLE MODULIN EXPORT ABC TRANSPORTER PER
34:00:00	7mi1-A	6.5	20.5	119	2628	8	MOLECULE:	CHIMERA PROTEIN OF DYNEIN AND ENDOLYSIN
35:00:00	6qs7-F	6.5	12.6	199	573	11	MOLECULE:	CHAPERONE PROTEIN CLPB
36:00:00	6qs6-B	6.5	8.7	193	582	11	MOLECULE:	CHAPERONE PROTEIN CLPB
37:00:00	1tbd-A	6.5	4.2	111	134	9	MOLECULE:	SV40 T-ANTIGEN
38:00:00	6orb-A	6.4	24.6	170	3352	9	MOLECULE:	MIDASIN
39:00:00	8fcv-Q	6.4	7.5	142	310	9	MOLECULE:	DNA (60-MER)
40:00:00	8ff4-V	6.4	8.2	148	343	9	MOLECULE:	TYPE I-B CRISPR-ASSOCIATED PROTEIN CAS5
41:00:00	7n6i-C	6.4	8.7	131	257	8	MOLECULE:	TNIQ (HOMOLOGY MODEL)
42:00:00	7n6i-E	6.4	8.7	129	257	9	MOLECULE:	TNIQ (HOMOLOGY MODEL)
43:00:00	7m9b-F	6.4	9.3	135	257	10	MOLECULE:	TNSC
44:00:00	8hra-E	6.4	15.7	143	834	10	MOLECULE:	ARCHAEAL ATPASE
45:00:00	8fcu-Q	6.4	7.5	142	310	9	MOLECULE:	TYPE I-B CRISPR-ASSOCIATED PROTEIN CAS5
46:00:00	8ff4-R	6.4	7.9	149	343	8	MOLECULE:	TYPE I-B CRISPR-ASSOCIATED PROTEIN CAS5
47:00:00	7m9a-J	6.4	9.2	133	257	10	MOLECULE:	TNSC
48:00:00	8fcv-S	6.4	6.7	143	343	9	MOLECULE:	DNA (60-MER)
49:00:00	8fnt-B	6.4	15	150	785	9	MOLECULE:	ARCHAEAL ATPASE
50:00:00	2h2j-A	6.4	16.2	116	425	13	MOLECULE:	RIBULOSE-1,5 BISPHOSPHATE CARBOXYLASE/OXYGENASE L
51:00:00	8fnt-C	6.4	13.1	139	785	9	MOLECULE:	ARCHAEAL ATPASE
52:00:00	6oaa-E	6.4	5.9	174	442	9	MOLECULE:	CELL DIVISION CONTROL PROTEIN 48
53:00:00	8bob-C	6.4	8	125	412	17	MOLECULE:	PROTEIN MALY
54:00:00	6u1y-B	6.4	3.2	131	245	18	MOLECULE:	MITOCHONDRIAL CHAPERONE BCS1
55:00:00	4fw9-A	6.4	14.4	156	602	9	MOLECULE:	TTC1975 PEPTIDASE
56:00:00	7tts-A	6.4	9.9	156	433	13	MOLECULE:	CASEINOLYTIC PEPTIDASE B PROTEIN HOMOLOG
57:00:00	6oab-E	6.4	8	136	442	10	MOLECULE:	CELL DIVISION CONTROL PROTEIN 48
58:00:00	7jpp-E	6.4	16.2	134	406	8	MOLECULE:	ORIGIN RECOGNITION COMPLEX SUBUNIT 1
59:00:00	6p8v-D	6.4	3.6	138	303	17	MOLECULE:	ATPASE, AAA FAMILY
60:00:00	6oay-E	6.4	14.2	142	570	10	MOLECULE:	HYPERACTIVE DISAGGREGASE CLPB
61:00:00	6edo-A	6.4	3.8	157	2031	15	MOLECULE:	MIDASIN
62:00:00	5c3c-B	6.4	6.3	139	235	12	MOLECULE:	CBBQ/NIRQ/NORQ DOMAIN PROTEIN
63:00:00	5vca-O	6.4	9.5	148	543	11	MOLECULE:	VCP-LIKE ATPASE
64:00:00	5vhm-F	6.4	8.3	136	219	16	MOLECULE:	26S PROTEASOME NON-ATPASE REGULATORY SUBUNIT 10

65:00:00	5vc7-A	6.4	9.6	179	544	10	MOLECULE:	VCP-LIKE ATPASE
66:00:00	1g64-A	6.4	3.4	103	169	15	MOLECULE:	COB(I)ALAMIN ADENOSYLTRANSFERASE
67:00:00	6p07-F	6.4	11.6	139	300	13	MOLECULE:	SPASTIN
68:00:00	8osg-E	6.4	4.5	167	323	10	MOLECULE:	MAGNESIUM-CHELATASE SUBUNIT CHLI
69:00:00	2tbd-A	6.4	3.6	103	134	6	MOLECULE:	SV40 T ANTIGEN
70:00:00	6mdm-D	6.3	10.5	162	713	13	MOLECULE:	VESICLE-FUSING ATPASE
71:00:00	7m9c-J	6.3	9.2	133	257	10	MOLECULE:	TNSC
72:00:00	7n6i-J	6.3	9.3	131	257	10	MOLECULE:	TNIQ (HOMOLOGY MODEL)
73:00:00	7n6i-I	6.3	10.1	132	257	10	MOLECULE:	TNIQ (HOMOLOGY MODEL)
74:00:00	8fcw-R	6.3	8.2	153	343	8	MOLECULE:	TNSC
75:00:00	7ufm-A	6.3	9.2	143	311	8	MOLECULE:	VCHTNSC
76:00:00	7m9b-E	6.3	8.2	132	257	8	MOLECULE:	TNSC
77:00:00	7m9b-L	6.3	9.1	134	257	10	MOLECULE:	TNSC
78:00:00	8hrb-F	6.3	15	150	851	9	MOLECULE:	ARCHAEAL ATPASE
79:00:00	7m9b-C	6.3	9.4	133	257	10	MOLECULE:	TNSC
80:00:00	7m9a-L	6.3	9.2	135	257	9	MOLECULE:	TNSC
81:00:00	8ff4-Q	6.3	7.5	142	310	9	MOLECULE:	TYPE I-B CRISPR-ASSOCIATED PROTEIN CAS5
82:00:00	7m9a-B	6.3	9	132	257	11	MOLECULE:	TNSC
83:00:00	7m9c-I	6.3	9.2	134	257	10	MOLECULE:	TNSC
84:00:00	7m9a-K	6.3	9.1	132	257	11	MOLECULE:	TNSC
85:00:00	7m9c-B	6.3	9.1	133	257	10	MOLECULE:	TNSC
86:00:00	8thb-A	6.3	18.4	174	468	11	MOLECULE:	ELG1 ISOFORM 1
87:00:00	7m9b-B	6.3	9.1	133	257	10	MOLECULE:	TNSC
88:00:00	7m9c-L	6.3	9.1	133	257	11	MOLECULE:	TNSC
89:00:00	7m9b-J	6.3	9.2	133	257	10	MOLECULE:	TNSC
90:00:00	8hra-D	6.3	15.5	141	848	10	MOLECULE:	ARCHAEAL ATPASE
91:00:00	7ufm-C	6.3	10.1	146	311	8	MOLECULE:	VCHTNSC
92:00:00	7ufm-J	6.3	10.3	150	311	8	MOLECULE:	VCHTNSC
93:00:00	7m9a-E	6.3	8.2	131	257	9	MOLECULE:	TNSC
94:00:00	7m9a-C	6.3	9.4	134	257	10	MOLECULE:	TNSC
95:00:00	7mcs-A	6.3	3.7	129	249	12	MOLECULE:	TRANSPOSON TN7 TRANSPOSITION PROTEIN TNSC
96:00:00	7t3i-A	6.3	9.4	142	577	15	MOLECULE:	RIX7
97:00:00	1l8q-A	6.3	15.9	137	321	9	MOLECULE:	CHROMOSOMAL REPLICATION INITIATOR PROTEIN DNAA
98:00:00	7rzy-7	6.3	10.5	147	311	8	MOLECULE:	TN6677 VIBRIO CHOLERAE TRANSPOSON TNSC (VCHTNSC)
99:00:00	6mat-A	6.3	9	148	578	11	MOLECULE:	RIX7 MUTANT
0:00	6sh3-G	6.3	14.1	161	376	10	MOLECULE:	MITOCHONDRIAL CHAPERONE BCS1
1:00	6sh3-B	6.3	12.2	153	376	10	MOLECULE:	MITOCHONDRIAL CHAPERONE BCS1
2:00	6sh3-E	6.3	12.2	154	376	10	MOLECULE:	MITOCHONDRIAL CHAPERONE BCS1
3:00	5nug-A	6.3	28.9	245	2920	8	MOLECULE:	CYTOPLASMIC DYNEIN 1 HEAVY CHAIN 1
4:00	2xsz-C	6.3	3.4	142	320	11	MOLECULE:	RUVB-LIKE 1
5:00	2z4r-A	6.3	7.9	127	240	14	MOLECULE:	CHROMOSOMAL REPLICATION INITIATOR PROTEIN DNAA
6:00	4ww4-A	6.3	3.7	143	416	14	MOLECULE:	RUVB-LIKE 1
7:00	8c0w-C	6.3	18.2	211	1030	8	MOLECULE:	PEROXISOMAL ATPASE PEX6
8:00	6qs6-A	6.3	4.4	184	577	12	MOLECULE:	CHAPERONE PROTEIN CLPB
9:00	6u1y-A	6.3	4	136	244	17	MOLECULE:	MITOCHONDRIAL CHAPERONE BCS1
10:00	1w5s-B	6.3	14.8	158	396	9	MOLECULE:	ORIGIN RECOGNITION COMPLEX SUBUNIT 2 ORC2
11:00	1ng9-A	6.2	16.1	183	794	7	MOLECULE:	5'-D(*AP*GP*CP*TP*GP*CP*CP*AP*GP*GP*CP*AP*CP*CP*A
12:00	8hra-G	6.2	15.9	140	835	10	MOLECULE:	ARCHAEAL ATPASE
13:00	7ufm-M	6.2	9.2	143	311	8	MOLECULE:	VCHTNSC
14:00	7ufm-F	6.2	9.2	143	311	8	MOLECULE:	VCHTNSC
15:00	7m9b-K	6.2	9.3	131	257	10	MOLECULE:	TNSC
16:00	7m99-H	6.2	8.7	129	257	9	MOLECULE:	TNSC
17:00	7ufi-7	6.2	9.9	148	311	7	MOLECULE:	DNA (5'-

18:00	8hra-C	6.2	9.8	143	852	9	MOLECULE:	ARCHAEAL ATPASE
19:00	8fcv-W	6.2	8.2	148	343	9	MOLECULE:	DNA (60-MER)
20:00	7ufm-G	6.2	9.1	143	311	8	MOLECULE:	VCHTNSC
21:00	7m9c-K	6.2	9.3	132	257	10	MOLECULE:	TNSC
22:00	8hra-H	6.2	14.6	136	834	10	MOLECULE:	ARCHAEAL ATPASE
23:00	8uii-A	6.2	28.5	170	641	9	MOLECULE:	ATPASE FAMILY AAA DOMAIN-CONTAINING PROTEIN 5
24:00:00	8hrb-Q	6.2	15.1	144	835	9	MOLECULE:	ARCHAEAL ATPASE
25:00:00	7ufm-D	6.2	9.1	143	311	8	MOLECULE:	VCHTNSC
26:00:00	7m9c-C	6.2	9.4	133	257	10	MOLECULE:	TNSC
27:00:00	5ex3-A	6.2	12.8	126	429	13	MOLECULE:	HISTONE-LYSINE N-METHYLTRANSFERASE SMYD3
28:00:00	2r44-A	6.2	4.8	165	330	8	MOLECULE:	UNCHARACTERIZED PROTEIN
29:00:00	1jr3-E	6.2	14.7	129	334	11	MOLECULE:	DNA POLYMERASE III SUBUNIT GAMMA
30:00:00	7qo4-J	6.2	13.2	181	405	9	MOLECULE:	26S PROTEASOME REGULATORY SUBUNIT RPN1
31:00:00	5vca-R	6.2	8.8	143	543	12	MOLECULE:	VCP-LIKE ATPASE
32:00:00	8d8k-W	6.2	27.6	137	395	9	MOLECULE:	PROBABLE S-ADENOSYL-L-METHIONINE-DEPENDENT RNA
33:00:00	6ef2-J	6.2	3.5	128	262	12	MOLECULE:	PROTEASOME SUBUNIT ALPHA TYPE-1
34:00:00	5g4f-B	6.2	8.5	179	726	10	MOLECULE:	VCP-LIKE ATPASE
35:00:00	4kfu-B	6.2	3.2	104	196	13	MOLECULE:	GENOME PACKAGING NTPASE B204
36:00:00	5t0g-C	6.2	11.3	164	384	12	MOLECULE:	PROTEASOME SUBUNIT ALPHA TYPE-6
37:00:00	4wvy-A	6.2	8.4	150	427	13	MOLECULE:	RUVB-LIKE 1
38:00:00	8uk9-E	6.2	4.4	130	318	18	MOLECULE:	SLIDING-CLAMP-LOADER SMALL SUBUNIT
39:00:00	1jbk-A	6.2	3.8	129	189	12	MOLECULE:	CLPB PROTEIN
40:00:00	5irm-C	6.2	11	159	770	8	MOLECULE:	UNCHARACTERIZED PROTEIN
41:00:00	5bk4-6	6.2	6.2	200	615	8	MOLECULE:	DNA REPLICATION LICENSING FACTOR MCM2
42:00:00	8ag6-B	6.1	14.4	161	975	9	MOLECULE:	DNA MISMATCH REPAIR PROTEIN MSH2
43:00:00	3mek-A	6.1	3.8	121	424	14	MOLECULE:	SET AND MYND DOMAIN-CONTAINING PROTEIN 3
44:00:00	7m9c-E	6.1	9.7	136	257	10	MOLECULE:	TNSC
45:00:00	7m9a-D	6.1	9.4	139	257	9	MOLECULE:	TNSC
46:00:00	3qww-A	6.1	3.6	120	431	9	MOLECULE:	SET AND MYND DOMAIN-CONTAINING PROTEIN 2
47:00:00	8c0v-E	6.1	9.2	172	821	9	MOLECULE:	PEROXISOMAL ATPASE PEX1
48:00:00	8emc-K	6.1	5.5	206	665	9	MOLECULE:	PROTEASE LON-RELATED BREX SYSTEM PROTEIN BRXL
49:00:00	8thd-A	6.1	22.3	170	474	12	MOLECULE:	ELG1 ISOFORM 1
50:00:00	8osh-A	6.1	4.5	163	290	9	MOLECULE:	MAGNESIUM-CHELATASE SUBUNIT CHLI
51:00:00	7jgr-D	6.1	17.4	152	441	9	MOLECULE:	ORIGIN RECOGNITION COMPLEX SUBUNIT 2
52:00:00	5g4f-C	6.1	8.9	180	726	11	MOLECULE:	VCP-LIKE ATPASE
53:00:00	6rn3-F	6.1	8.1	192	675	14	MOLECULE:	CHAPERONE PROTEIN CLPB
54:00:00	6qem-L	6.1	5.6	148	239	12	MOLECULE:	REPLICATIVE DNA HELICASE
55:00:00	4kfu-D	6.1	3.7	109	195	14	MOLECULE:	GENOME PACKAGING NTPASE B204
56:00:00	5wcb-A	6.1	13.3	138	277	11	MOLECULE:	MEIOTIC SPINDLE FORMATION PROTEIN MEI-1
57:00:00	4kft-C	6.1	3.2	108	204	12	MOLECULE:	GENOME PACKAGING NTPASE B204
58:00:00	5udb-A	6.1	15.6	141	372	6	MOLECULE:	DNA REPLICATION LICENSING FACTOR MCM2
59:00:00	7wrx-E	6.1	9.7	137	590	7	MOLECULE:	HERA
60:00:00	2dhr-A	6.1	9.1	153	458	12	MOLECULE:	FTSH
61:00:00	7m99-E	6	8.7	131	257	8	MOLECULE:	TNSC
62:00:00	8ff4-W	6	6.7	140	343	9	MOLECULE:	TYPE I-B CRISPR-ASSOCIATED PROTEIN CAS5
63:00:00	7ufm-N	6	9.1	144	311	8	MOLECULE:	VCHTNSC
64:00:00	7n6i-G	6	9.4	133	257	10	MOLECULE:	TNIQ (HOMOLOGY MODEL)
65:00:00	8hr9-I	6	15.5	143	836	10	MOLECULE:	ARCHAEAL ATPASE
66:00:00	8fnt-D	6	15	150	784	9	MOLECULE:	ARCHAEAL ATPASE
67:00:00	7m99-D	6	8.7	131	257	8	MOLECULE:	TNSC
68:00:00	7n6i-F	6	8.7	129	257	9	MOLECULE:	TNIQ (HOMOLOGY MODEL)
69:00:00	7ufm-E	6	9.1	141	311	9	MOLECULE:	VCHTNSC
70:00:00	8osh-B	6	4.4	167	317	12	MOLECULE:	MAGNESIUM-CHELATASE SUBUNIT CHLI

71:00:00	7plh-A	6	8.5	128	260	10	MOLECULE:	SHTNSC
72:00:00	3zh9-B	6	15	126	339	10	MOLECULE:	DELTA
73:00:00	5zr1-B	6	12.5	133	374	11	MOLECULE:	ORIGIN RECOGNITION COMPLEX SUBUNIT 1
74:00:00	4wia-A	6	3.5	120	229	11	MOLECULE:	PUTATIVE FLAGELLA-RELATED PROTEIN H
75:00:00	4ai6-A	6	15.8	102	2650	10	MOLECULE:	GLUTATHIONE S-TRANSFERASE CLASS-MU 26 KDA ISOZYME
76:00:00	2h23-A	6	23.5	130	426	13	MOLECULE:	RIBULOSE-1,5 BISPHOSPHATE CARBOXYLASE/OXYGENASE L
77:00:00	6qi9-D	6	10.6	140	268	9	MOLECULE:	RUVB-LIKE 1
78:00:00	7rzy-1	6	7.1	142	311	8	MOLECULE:	TN6677 VIBRIO CHOLERAE TRANSPOSON TNSC (VCHTNSC)
79:00:00	6uks-D	6	4.2	154	362	15	MOLECULE:	MITOCHONDRIAL CHAPERONE BCS1
80:00:00	7ttr-F	6	10.8	149	304	15	MOLECULE:	CASEINOLYTIC PEPTIDASE B PROTEIN HOMOLOG
81:00:00	5v8f-D	6	14.9	148	456	11	MOLECULE:	DNA REPLICATION LICENSING FACTOR MCM2
82:00:00	5vhr-B	6	3.5	125	233	10	MOLECULE:	26S PROTEASOME NON-ATPASE REGULATORY SUBUNIT 10
83:00:00	4kfu-A	6	3.5	105	205	13	MOLECULE:	GENOME PACKAGING NTPASE B204
84:00:00	6oab-C	6	8.5	139	532	12	MOLECULE:	CELL DIVISION CONTROL PROTEIN 48
85:00:00	3co5-A	6	4.2	109	134	7	MOLECULE:	PUTATIVE TWO-COMPONENT SYSTEM TRANSCRIPTIONAL RES
86:00:00	4kft-D	6	3.6	109	194	14	MOLECULE:	GENOME PACKAGING NTPASE B204
87:00:00	4kft-B	6	3.6	110	197	14	MOLECULE:	GENOME PACKAGING NTPASE B204
88:00:00	5x71-A	5.9	6.1	129	796	6	MOLECULE:	MRNA CAPPING ENZYME P5
89:00:00	8thc-A	5.9	18.9	171	479	11	MOLECULE:	ELG1 ISOFORM 1
90:00:00	8ff4-U	5.9	8.5	152	343	9	MOLECULE:	TYPE I-B CRISPR-ASSOCIATED PROTEIN CAS5
91:00:00	8hr9-G	5.9	15.1	146	830	10	MOLECULE:	ARCHAEAL ATPASE
92:00:00	7f02-E	5.9	3.3	100	202	8	MOLECULE:	CYTOCHROME C BIOGENESIS ATP-BINDING EXPORT PROTEI
93:00:00	7mcs-E	5.9	4.2	149	402	12	MOLECULE:	TRANSPOSON TN7 TRANSPOSITION PROTEIN TNSC
94:00:00	7ufm-K	5.9	9.1	142	311	8	MOLECULE:	VCHTNSC
95:00:00	8fcv-U	5.9	8	149	343	8	MOLECULE:	DNA (60-MER)
96:00:00	8fcw-U	5.9	8	145	343	9	MOLECULE:	TNSC
97:00:00	7mcs-F	5.9	3.9	140	401	10	MOLECULE:	TRANSPOSON TN7 TRANSPOSITION PROTEIN TNSC
98:00:00	3k1j-A	5.9	11.6	151	566	13	MOLECULE:	ATP-DEPENDENT PROTEASE LON
99:00:00	8p49-A	5.9	4.9	148	377	7	MOLECULE:	Q8U0N8 PROTEIN
0:00	5ln3-M	5.9	11.8	159	389	9	MOLECULE:	26S PROTEASOME NON-ATPASE REGULATORY SUBUNIT 2
1:00	7mbw-B	5.9	10.6	155	469	12	MOLECULE:	TRANSPOSON TN7 TRANSPOSITION PROTEIN TNSC
2:00	6n8v-F	5.9	10.9	235	710	9	MOLECULE:	HEAT SHOCK PROTEIN 104
3:00	3cmw-C	5.9	15.2	143	1608	12	MOLECULE:	DNA (5'-
4:00	4kft-A	5.9	3.5	107	195	13	MOLECULE:	GENOME PACKAGING NTPASE B204
5:00	2ce7-D	5.9	8.6	145	413	12	MOLECULE:	CELL DIVISION PROTEIN FTSH
6:00	1xp8-A	5.9	10.8	139	298	12	MOLECULE:	RECA PROTEIN
7:00	4kfs-A	5.9	3.6	110	202	14	MOLECULE:	GENOME PACKAGING NTPASE B204
8:00	7qh2-C	5.8	12.5	138	467	9	MOLECULE:	LACTATE DEHYDROGENASE (NAD(+),FERREDOXIN) SUBUNIT
9:00	4cej-B	5.8	15.6	136	1156	8	MOLECULE:	ATP-DEPENDENT HELICASE/NUCLEASE SUBUNIT A
10:00	7n6i-D	5.8	8.7	129	257	9	MOLECULE:	TNIQ (HOMOLOGY MODEL)
11:00	8emh-G	5.8	7.1	181	671	9	MOLECULE:	PROTEASE LON-RELATED BREX SYSTEM PROTEIN BRXL
12:00	8emh-I	5.8	5.6	200	672	9	MOLECULE:	PROTEASE LON-RELATED BREX SYSTEM PROTEIN BRXL
13:00	8c0w-F	5.8	11.4	187	823	9	MOLECULE:	PEROXISOMAL ATPASE PEX6
14:00	7m99-B	5.8	8.7	129	257	9	MOLECULE:	TNSC
15:00	1g8p-A	5.8	4.4	176	321	11	MOLECULE:	MAGNESIUM-CHELATASE 38 KDA SUBUNIT
16:00	8th8-L	5.8	11.9	206	862	11	MOLECULE:	DYNEIN REGULATORY COMPLEX PROTEIN 1/2 N-TERMINAL
17:00	7rzy-5	5.8	10.6	148	311	8	MOLECULE:	TN6677 VIBRIO CHOLERAE TRANSPOSON TNSC (VCHTNSC)
18:00	3b85-A	5.8	3.4	114	187	10	MOLECULE:	PHOSPHATE STARVATION-INDUCIBLE PROTEIN
19:00	8iqh-J	5.8	9	237	881	8	MOLECULE:	PUTATIVE PRIMASE C962R
20:00	4fwv-A	5.8	3.2	115	340	10	MOLECULE:	TTC1975 PEPTIDASE
21:00	1xwi-A	5.8	12.9	135	322	16	MOLECULE:	SKD1 PROTEIN
22:00	8gja-F	5.8	5	143	342	5	MOLECULE:	RAD51C
23:00	4kfr-B	5.8	3.5	112	208	13	MOLECULE:	GENOME PACKAGING NTPASE B204

24:00:00	5g4f-E	5.8	8.9	197	726	11	MOLECULE:	VCP-LIKE ATPASE
25:00:00	5vhj-C	5.8	9.1	133	213	13	MOLECULE:	26S PROTEASOME NON-ATPASE REGULATORY SUBUNIT 10
26:00:00	4kfr-A	5.8	3.3	107	204	13	MOLECULE:	GENOME PACKAGING NTPASE B204
27:00:00	6oab-B	5.8	8.6	139	509	12	MOLECULE:	CELL DIVISION CONTROL PROTEIN 48
28:00:00	3uk6-D	5.8	8.5	143	299	10	MOLECULE:	RUVB-LIKE 2
29:00:00	5og1-B	5.8	9.4	174	572	11	MOLECULE:	CHAPERONE PROTEIN CLPB,ATP-DEPENDENT CLP PROTEASE
30:00:00	4kfs-B	5.8	3.7	110	199	13	MOLECULE:	GENOME PACKAGING NTPASE B204
31:00:00	6uks-F	5.8	3.9	150	362	15	MOLECULE:	MITOCHONDRIAL CHAPERONE BCS1
32:00:00	2oca-A	5.8	6.1	122	494	7	MOLECULE:	ATP-DEPENDENT DNA HELICASE UVSW
33:00:00	8dgf-A	5.7	10.2	168	1541	7	MOLECULE:	ATP-BINDING PROTEIN AVS4
34:00:00	6o16-A	5.7	10.7	138	798	11	MOLECULE:	DEAH (ASP-GLU-ALA-HIS) BOX POLYPEPTIDE 37
35:00:00	7rzy-2	5.7	10	148	311	7	MOLECULE:	TN6677 VIBRIO CHOLERAE TRANSPOSON TNSC (VCHTNSC)
36:00:00	8c0w-B	5.7	11.4	183	821	8	MOLECULE:	PEROXISOMAL ATPASE PEX6
37:00:00	7m99-F	5.7	8.7	129	257	9	MOLECULE:	TNSC
38:00:00	8fnt-E	5.7	15.5	137	781	9	MOLECULE:	ARCHAEAL ATPASE
39:00:00	8c0w-D	5.7	9.9	176	821	9	MOLECULE:	PEROXISOMAL ATPASE PEX6
40:00:00	8hrb-K	5.7	12.9	133	835	10	MOLECULE:	ARCHAEAL ATPASE
41:00:00	7n6i-H	5.7	8.7	129	257	9	MOLECULE:	TNIQ (HOMOLOGY MODEL)
42:00:00	3rib-B	5.7	3.8	120	419	10	MOLECULE:	N-LYSINE METHYLTRANSFERASE SMYD2
43:00:00	6oaa-B	5.7	12.1	147	509	13	MOLECULE:	CELL DIVISION CONTROL PROTEIN 48
44:00:00	3f8t-A	5.7	5.2	212	459	10	MOLECULE:	PREDICTED ATPASE INVOLVED IN REPLICATION CONTROL,
45:00:00	1jr3-D	5.7	16.8	141	338	9	MOLECULE:	DNA POLYMERASE III SUBUNIT GAMMA
46:00:00	6eud-A	5.7	14.1	138	808	14	MOLECULE:	ATP-DEPENDENT RNA HELICASE HRPB
47:00:00	7mcs-B	5.7	4.1	144	402	11	MOLECULE:	TRANSPOSON TN7 TRANSPOSITION PROTEIN TNSC
48:00:00	5g4f-P	5.7	8.7	181	726	10	MOLECULE:	VCP-LIKE ATPASE
49:00:00	2qgz-A	5.7	3.9	124	183	14	MOLECULE:	PUTATIVE PRIMOSOME COMPONENT
50:00:00	5vhr-D	5.7	6.9	128	236	15	MOLECULE:	26S PROTEASOME NON-ATPASE REGULATORY SUBUNIT 10
51:00:00	5vhn-D	5.7	8.1	131	237	14	MOLECULE:	26S PROTEASOME NON-ATPASE REGULATORY SUBUNIT 10
52:00:00	5vhq-B	5.7	8.8	131	233	11	MOLECULE:	26S PROTEASOME NON-ATPASE REGULATORY SUBUNIT 10
53:00:00	6jpu-A	5.7	3.7	143	577	10	MOLECULE:	UNCHARACTERIZED AAA DOMAIN-CONTAINING PROTEIN C31
54:00:00	5d4w-C	5.7	4.7	179	688	12	MOLECULE:	PUTATIVE HEAT SHOCK PROTEIN
55:00:00	7ykk-A	5.7	8.7	205	735	9	MOLECULE:	ATPASE FAMILY GENE 2 PROTEIN
56:00:00	4a15-A	5.6	9.7	115	596	12	MOLECULE:	ATP-DEPENDENT DNA HELICASE TA0057
57:00:00	8d8l-W	5.6	5.5	127	395	10	MOLECULE:	PROBABLE S-ADENOSYL-L-METHIONINE-DEPENDENT RNA
58:00:00	8dgc-B	5.6	15.4	165	2004	11	MOLECULE:	SEAVS3
59:00:00	8fnu-B	5.6	15.2	133	796	5	MOLECULE:	KAP NTPASE DOMAIN-CONTAINING PROTEIN
60:00:00	7yut-F	5.6	21.8	191	773	13	MOLECULE:	LON PROTEASE
61:00:00	5a5b-M	5.6	4	162	367	14	MOLECULE:	PROTEASOME COMPONENT PRE3
62:00:00	7rzy-3	5.6	10.6	148	311	9	MOLECULE:	TN6677 VIBRIO CHOLERAE TRANSPOSON TNSC (VCHTNSC)
63:00:00	6og2-F	5.6	10.5	133	558	11	MOLECULE:	HYPERACTIVE DISAGGREGASE CLPB
64:00:00	5g4f-D	5.6	13.5	188	726	10	MOLECULE:	VCP-LIKE ATPASE
65:00:00	6mfv-D	5.6	8.6	127	641	11	MOLECULE:	TETRATRICOPEPTIDE REPEAT SENSOR PH0952
66:00:00	5vhm-D	5.6	3.3	126	220	16	MOLECULE:	26S PROTEASOME NON-ATPASE REGULATORY SUBUNIT 10
67:00:00	4kfu-C	5.6	3.7	109	195	14	MOLECULE:	GENOME PACKAGING NTPASE B204
68:00:00	5og1-C	5.6	9.8	187	624	10	MOLECULE:	CHAPERONE PROTEIN CLPB,ATP-DEPENDENT CLP PROTEASE
69:00:00	6qs7-B	5.6	6.5	178	582	11	MOLECULE:	CHAPERONE PROTEIN CLPB
70:00:00	6ees-A	5.6	7.5	157	2023	13	MOLECULE:	MIDASIN
71:00:00	6qs7-A	5.6	8	213	568	14	MOLECULE:	CHAPERONE PROTEIN CLPB
72:00:00	8fnu-A	5.5	13.6	137	796	7	MOLECULE:	KAP NTPASE DOMAIN-CONTAINING PROTEIN
73:00:00	7ufm-L	5.5	9.3	142	311	8	MOLECULE:	VCHTNSC
74:00:00	8hrb-M	5.5	15	136	849	11	MOLECULE:	ARCHAEAL ATPASE
75:00:00	7ufm-I	5.5	9	143	311	8	MOLECULE:	VCHTNSC
76:00:00	3qxy-A	5.5	18.5	129	421	12	MOLECULE:	N-LYSINE METHYLTRANSFERASE SETD6

77:00:00	8fnu-C	5.5	13.7	139	796	5	MOLECULE:	KAP NTPASE DOMAIN-CONTAINING PROTEIN
78:00:00	6n9x-B	5.5	21.6	174	500	9	MOLECULE:	DNA PRIMASE/HELICASE
79:00:00	6fhs-C	5.5	9.6	141	459	15	MOLECULE:	RUVB-LIKE HELICASE
80:00:00	5vhf-D	5.5	13.4	163	344	12	MOLECULE:	26S PROTEASOME NON-ATPASE REGULATORY SUBUNIT 10
81:00:00	3gp8-A	5.5	14.3	141	551	14	MOLECULE:	EXODEOXYRIBONUCLEASE V, SUBUNIT RECD, PUTATIVE
82:00:00	7jgs-C	5.5	18.9	171	614	9	MOLECULE:	ORIGIN RECOGNITION COMPLEX SUBUNIT 2
83:00:00	5aor-A	5.5	27.8	200	1009	10	MOLECULE:	DOSAGE COMPENSATION REGULATOR
84:00:00	5t0j-B	5.5	4.2	144	348	11	MOLECULE:	26S PROTEASOME NON-ATPASE REGULATORY SUBUNIT 2
85:00:00	6igm-B	5.5	4.8	141	398	11	MOLECULE:	RUVB-LIKE 1
86:00:00	6oab-A	5.5	14.3	180	493	12	MOLECULE:	CELL DIVISION CONTROL PROTEIN 48
87:00:00	6fo1-D	5.5	9.4	142	310	13	MOLECULE:	RUVB-LIKE 1
88:00:00	6ppj-B	5.5	15.6	119	848	13	MOLECULE:	UVRD/REP HELICASE
89:00:00	6qs4-A	5.5	11.6	192	568	13	MOLECULE:	CHAPERONE PROTEIN CLPB
90:00:00	6ox1-B	5.4	30	138	485	10	MOLECULE:	ACTIN, CYTOPLASMIC 1
91:00:00	5e7p-A	5.4	15.9	199	719	11	MOLECULE:	CELL DIVISION CONTROL PROTEIN CDC48
92:00:00	2fna-A	5.4	12.1	136	352	10	MOLECULE:	CONSERVED HYPOTHETICAL PROTEIN
93:00:00	6hz8-L	5.4	3.7	136	285	11	MOLECULE:	5-METHYLCYTOSINE-SPECIFIC RESTRICTION ENZYME B
94:00:00	7otj-B	5.4	4.3	123	436	10	MOLECULE:	ATP-DEPENDENT DNA HELICASE PIF1
95:00:00	3nbx-X	5.4	5.5	175	481	10	MOLECULE:	ATPASE RAVA
96:00:00	5dgk-A	5.4	4.8	202	519	9	MOLECULE:	ACTIVE HELICASE
97:00:00	7rzy-6	5.4	6.7	138	311	7	MOLECULE:	TN6677 VIBRIO CHOLERAE TRANSPOSON TNSC (VCHTNSC)
98:00:00	6ef1-M	5.4	10.4	134	262	15	MOLECULE:	PROTEASOME SUBUNIT ALPHA TYPE-1
99:00:00	5vhf-E	5.4	10.2	151	345	10	MOLECULE:	26S PROTEASOME NON-ATPASE REGULATORY SUBUNIT 10
0:00	4tvs-A	5.4	3.6	147	224	10	MOLECULE:	TORSIN-1A-INTERACTING PROTEIN 1
1:00	5kne-F	5.4	10.1	196	607	10	MOLECULE:	HEAT SHOCK PROTEIN 104
2:00	5nss-N	5.4	3.6	138	251	7	MOLECULE:	DNA-DIRECTED RNA POLYMERASE SUBUNIT ALPHA
3:00	3j16-B	5.3	5.2	126	608	11	MOLECULE:	DOM34P
4:00	6nmi-A	5.3	16.3	144	653	9	MOLECULE:	GENERAL TRANSCRIPTION AND DNA REPAIR FACTOR IIH H
5:00	7rzy-4	5.3	7.3	142	311	8	MOLECULE:	TN6677 VIBRIO CHOLERAE TRANSPOSON TNSC (VCHTNSC)
6:00	7pkq-x	5.3	3.8	117	368	7	MOLECULE:	MS35
7:00	8hr9-H	5.3	15.4	139	834	8	MOLECULE:	ARCHAEAL ATPASE
8:00	7uqj-A	5.3	9.2	202	585	13	MOLECULE:	ATPASE HISTONE CHAPERONE YTA7
9:00	6oa9-C	5.3	8.7	166	492	9	MOLECULE:	CELL DIVISION CONTROL PROTEIN 48
10:00	7qy7-A	5.3	15.8	160	342	8	MOLECULE:	26S PROTEASOME NON-ATPASE REGULATORY SUBUNIT 13
11:00	7mcs-C	5.3	4	143	404	11	MOLECULE:	TRANSPOSON TN7 TRANSPOSITION PROTEIN TNSC
12:00	8giy-A	5.3	14	130	343	11	MOLECULE:	DNA POLYMERASE III SUBUNIT DELTA
13:00	3io5-B	5.3	4.3	142	272	8	MOLECULE:	RECOMBINATION AND REPAIR PROTEIN
14:00	5vhn-E	5.3	6.4	121	241	12	MOLECULE:	26S PROTEASOME NON-ATPASE REGULATORY SUBUNIT 10
15:00	5vhh-E	5.3	10.6	152	345	12	MOLECULE:	26S PROTEASOME REGULATORY SUBUNIT 7
16:00	6ahf-C	5.3	15.5	215	865	10	MOLECULE:	HEAT SHOCK PROTEIN 104
17:00	2xgj-B	5.2	7.4	128	773	5	MOLECULE:	ATP-DEPENDENT RNA HELICASE DOB1
18:00	8fnu-G	5.2	15	142	796	6	MOLECULE:	KAP NTPASE DOMAIN-CONTAINING PROTEIN
19:00	8fnu-D	5.2	15.7	129	796	6	MOLECULE:	KAP NTPASE DOMAIN-CONTAINING PROTEIN
20:00	5o6b-A	5.2	4	121	528	12	MOLECULE:	ATP-DEPENDENT DNA HELICASE PIF1
21:00	7z6h-K	5.2	9.4	138	439	11	MOLECULE:	CELL CYCLE CHECKPOINT CONTROL PROTEIN RAD9A
22:00	8fnu-E	5.2	13.5	137	796	5	MOLECULE:	KAP NTPASE DOMAIN-CONTAINING PROTEIN
23:00	7wi4-B	5.2	11.6	132	410	11	MOLECULE:	ATP-DEPENDENT ZINC METALLOPROTEASE FTSH
24:00:00	6ef0-J	5.2	8	139	272	12	MOLECULE:	PROTEASOME SUBUNIT ALPHA TYPE-1
25:00:00	2f1h-A	5.2	6.6	141	313	9	MOLECULE:	DNA REPAIR AND RECOMBINATION PROTEIN RADA
26:00:00	5vft-C	5.2	7.8	151	384	12	MOLECULE:	26S PROTEASOME NON-ATPASE REGULATORY SUBUNIT 1
27:00:00	5udb-D	5.2	11.6	143	447	10	MOLECULE:	DNA REPLICATION LICENSING FACTOR MCM2
28:00:00	8emc-B	5.2	6	177	671	10	MOLECULE:	PROTEASE LON-RELATED BREX SYSTEM PROTEIN BRXL
29:00:00	6acx-A	5.1	16.7	173	1175	10	MOLECULE:	MYCOBACTERIUM SMEGMATIS MFD

30:00:00	7ypj-E	5.1	23.6	202	773	13	MOLECULE:	LON PROTEASE
31:00:00	7amv-W	5.1	8.7	142	637	12	MOLECULE:	DNA-DIRECTED RNA POLYMERASE 147 KDA POLYPEPTIDE
32:00:00	8fnu-F	5.1	13.6	139	796	6	MOLECULE:	KAP NTPASE DOMAIN-CONTAINING PROTEIN
33:00:00	2mpl-A	5.1	7.2	91	127	5	MOLECULE:	ZINC FINGER PROTEIN ZFPM1
34:00:00	4xgc-C	5.1	12.4	157	567	9	MOLECULE:	ORIGIN RECOGNITION COMPLEX SUBUNIT 2
35:00:00	6qem-I	5.1	6.8	142	239	14	MOLECULE:	REPLICATIVE DNA HELICASE
36:00:00	3crv-A	5.1	14.9	126	551	12	MOLECULE:	XPD/RAD3 RELATED DNA HELICASE
37:00:00	8faz-X	5.1	3.4	111	236	9	MOLECULE:	DNA REPAIR PROTEIN RAD51 HOMOLOG 2
38:00:00	8jqb-E	5.1	5.7	112	499	9	MOLECULE:	ENDONUCLEASE GAJA
39:00:00	5vhi-B	5.1	9.5	162	298	10	MOLECULE:	26S PROTEASOME REGULATORY SUBUNIT 7
40:00:00	6fhs-E	5.1	14.3	146	441	11	MOLECULE:	RUVB-LIKE HELICASE
41:00:00	4fwh-A	5.1	11.6	152	580	9	MOLECULE:	TTC1975 PEPTIDASE
42:00:00	7t1q-A	5	5.8	105	377	4	MOLECULE:	SUCCINYL-DIAMINOPIMELATE DESUCCINYLASE
43:00:00	8fvu-A	5	9.5	179	1361	10	MOLECULE:	BACULOVIRAL IAP REPEAT-CONTAINING PROTEIN 1
44:00:00	7mqj-A	5	16.1	140	773	10	MOLECULE:	PROBABLE ATP-DEPENDENT RNA HELICASE DHR1
45:00:00	8hr8-D	5	14.8	146	836	9	MOLECULE:	ARCHAEAL ATPASE
46:00:00	8osf-B	5	4.3	166	321	10	MOLECULE:	MAGNESIUM-CHELATASE SUBUNIT CHLI
47:00:00	6sh5-G	5	5.8	136	334	13	MOLECULE:	MITOCHONDRIAL CHAPERONE BCS1
48:00:00	5vca-N	5	9.6	147	543	9	MOLECULE:	VCP-LIKE ATPASE
49:00:00	8gbj-X	5	3.5	116	232	9	MOLECULE:	DNA REPAIR PROTEIN RAD51 HOMOLOG 2
50:00:00	5d4w-A	5	13.5	191	688	10	MOLECULE:	PUTATIVE HEAT SHOCK PROTEIN
51:00:00	7ou2-B	4.9	17	172	650	8	MOLECULE:	DNA MISMATCH REPAIR PROTEIN MUTS
52:00:00	4q2d-A	4.9	11.8	163	927	7	MOLECULE:	CRISPR-ASSOCIATED HELICASE CAS3
53:00:00	8c0v-A	4.9	14.6	205	823	13	MOLECULE:	PEROXISOMAL ATPASE PEX1
54:00:00	4cej-A	4.9	11.8	119	1177	9	MOLECULE:	ATP-DEPENDENT HELICASE/NUCLEASE SUBUNIT A
55:00:00	3cmu-A	4.9	10.8	140	1937	14	MOLECULE:	DNA (5'-
56:00:00	8dgc-A	4.9	22	175	2028	9	MOLECULE:	SEAVS3
57:00:00	7mbw-D	4.9	10.3	154	467	11	MOLECULE:	TRANSPOSON TN7 TRANSPOSITION PROTEIN TNSC
58:00:00	7qy7-D	4.9	5.6	169	378	9	MOLECULE:	26S PROTEASOME NON-ATPASE REGULATORY SUBUNIT 13
59:00:00	6npy-A	4.9	9.8	147	798	12	MOLECULE:	NACHT, LRR AND PYD DOMAINS-CONTAINING PROTEIN 3
60:00:00	8osf-D	4.9	4.2	165	319	12	MOLECULE:	MAGNESIUM-CHELATASE SUBUNIT CHLI
61:00:00	7mcs-D	4.9	4.1	146	403	11	MOLECULE:	TRANSPOSON TN7 TRANSPOSITION PROTEIN TNSC
62:00:00	6qem-K	4.9	4.7	141	239	14	MOLECULE:	REPLICATIVE DNA HELICASE
63:00:00	6qem-H	4.9	5.1	138	239	14	MOLECULE:	REPLICATIVE DNA HELICASE
64:00:00	5wcb-B	4.9	10.3	121	277	12	MOLECULE:	MEIOTIC SPINDLE FORMATION PROTEIN MEI-1
65:00:00	7jk5-A	4.9	17.2	169	390	7	MOLECULE:	ORIGIN RECOGNITION COMPLEX SUBUNIT 2
66:00:00	4xgc-A	4.9	13.5	142	310	8	MOLECULE:	ORIGIN RECOGNITION COMPLEX SUBUNIT 2
67:00:00	8ouz-D	4.9	3.5	117	234	9	MOLECULE:	DNA REPAIR PROTEIN RAD51 HOMOLOG 2
68:00:00	6qem-G	4.9	15.3	153	239	10	MOLECULE:	REPLICATIVE DNA HELICASE
69:00:00	6rfl-Y	4.8	4.3	131	600	11	MOLECULE:	DNA-DEPENDENT RNA POLYMERASE SUBUNIT RPO132
70:00:00	6qem-J	4.8	8.3	143	239	13	MOLECULE:	REPLICATIVE DNA HELICASE
71:00:00	6ef0-I	4.8	10.4	137	271	14	MOLECULE:	PROTEASOME SUBUNIT ALPHA TYPE-1
72:00:00	6mfv-C	4.8	8.5	123	641	11	MOLECULE:	TETRATRICOPEPTIDE REPEAT SENSOR PH0952
73:00:00	4ag5-B	4.8	4	117	374	9	MOLECULE:	TYPE IV SECRETORY PATHWAY VIRB4 COMPONENTS-LIKE P
74:00:00	8odp-A	4.7	4.5	110	505	5	MOLECULE:	RNA-SPLICING LIGASE RTCB HOMOLOG
75:00:00	7sjr-A	4.7	16.9	140	911	11	MOLECULE:	DNA HELICASE
76:00:00	2wjy-A	4.7	24.2	182	773	4	MOLECULE:	REGULATOR OF NONSENSE TRANSCRIPTS 1
77:00:00	7mbw-A	4.7	11.5	152	475	11	MOLECULE:	TRANSPOSON TN7 TRANSPOSITION PROTEIN TNSC
78:00:00	6ef1-J	4.7	3.4	122	273	13	MOLECULE:	PROTEASOME SUBUNIT ALPHA TYPE-1
79:00:00	2d7d-A	4.7	16.7	138	621	9	MOLECULE:	5'-D(P*TP*TP*T)-3'
80:00:00	4ag5-A	4.7	3.8	116	374	11	MOLECULE:	TYPE IV SECRETORY PATHWAY VIRB4 COMPONENTS-LIKE P
81:00:00	4ag6-A	4.7	4	118	373	8	MOLECULE:	TYPE IV SECRETORY PATHWAY VIRB4 COMPONENTS-LIKE P
82:00:00	4ag6-B	4.7	3.8	117	376	9	MOLECULE:	TYPE IV SECRETORY PATHWAY VIRB4 COMPONENTS-LIKE P

83:00:00	5vhp-D	4.7	3.7	123	237	13	MOLECULE:	26S PROTEASOME NON-ATPASE REGULATORY SUBUNIT 10
84:00:00	7yux-B	4.7	25.2	174	773	10	MOLECULE:	LON PROTEASE
85:00:00	7lcc-A	4.7	26.6	121	1369	12	MOLECULE:	HELRAISER K1068Q
86:00:00	4zcf-C	4.6	6.4	143	627	7	MOLECULE:	RESTRICTION ENDONUCLEASE ECOP15I, MODIFICATION SU
87:00:00	7lgp-B	4.6	7.1	102	377	8	MOLECULE:	SUCCINYL-DIAMINOPIMELATE DESUCCINYLASE
88:00:00	2fdc-A	4.6	4.3	123	505	10	MOLECULE:	5'-D(P*CP*GP*GP*CP*TP*CP*CP*AP*TP*CP*TP*CP*TP*AP*
89:00:00	5vhn-A	4.6	8.7	129	258	9	MOLECULE:	26S PROTEASOME NON-ATPASE REGULATORY SUBUNIT 10
90:00:00	4m9s-B	4.6	12.3	153	511	10	MOLECULE:	CELL DEATH PROTEIN 4
91:00:00	6qs4-B	4.6	7	181	582	13	MOLECULE:	CHAPERONE PROTEIN CLPB
92:00:00	1gm5-A	4.5	14.3	139	729	11	MOLECULE:	RECG
93:00:00	8b1r-B	4.5	12.9	127	1170	9	MOLECULE:	RECBCD ENZYME SUBUNIT RECB
94:00:00	4ksr-A	4.5	18.9	135	521	9	MOLECULE:	TYPE II SECRETION SYSTEM PROTEIN E, HEMOLYSIN-COR
95:00:00	7bpa-A	4.5	17.7	200	742	10	MOLECULE:	TRANSITIONAL ENDOPLASMIC RETICULUM ATPASE
96:00:00	5vhq-A	4.5	3.2	122	261	11	MOLECULE:	26S PROTEASOME NON-ATPASE REGULATORY SUBUNIT 10
97:00:00	6ef1-I	4.5	6.2	128	271	13	MOLECULE:	PROTEASOME SUBUNIT ALPHA TYPE-1
98:00:00	7yph-E	4.5	13.6	74	773	22	MOLECULE:	LON PROTEASE
99:00:00	7mi6-A	4.4	24	95	2419	14	MOLECULE:	FUSION PROTEIN OF DYNEIN AND ENDOLYSIN
0:00	7nyx-A	4.4	3.7	112	1467	11	MOLECULE:	CHROMOSOME PARTITION PROTEIN MUKB
1:00	6fwr-A	4.4	15.4	134	699	10	MOLECULE:	ATP-DEPENDENT DNA HELICASE DING
2:00	5j1s-B	4.4	3.7	145	235	11	MOLECULE:	TORSIN-1A
3:00	5udb-9	4.4	16.7	147	376	7	MOLECULE:	DNA REPLICATION LICENSING FACTOR MCM2
4:00	6sh4-B	4.4	7.1	144	376	13	MOLECULE:	MITOCHONDRIAL CHAPERONE BCS1
5:00	7mcs-G	4.4	4.2	142	358	13	MOLECULE:	TRANSPOSON TN7 TRANSPOSITION PROTEIN TNSC
6:00	5t0i-B	4.4	9.5	149	348	12	MOLECULE:	26S PROTEASE REGULATORY SUBUNIT 7
7:00	7b8s-C	4.3	5	96	589	4	MOLECULE:	MULTIDRUG EFFLUX PUMP SUBUNIT ACRB,MULTIDRUG EFFL
8:00	3al0-B	4.3	21.1	119	482	4	MOLECULE:	GLUTAMYL-TRNA(GLN) AMIDOTRANSFERASE SUBUNIT A
9:00	8t5s-A	4.3	4.5	121	693	7	MOLECULE:	DICER-RELATED HELICASE
10:00	6slf-A	4.3	12.8	111	398	4	MOLECULE:	N-ALPHA-ACYL-GLUTAMINE AMINOACYLASE
11:00	7w46-B	4.3	7.8	156	402	13	MOLECULE:	UNCHARACTERIZED ATPASE YJOB
12:00	6sh4-A	4.3	6.7	162	376	10	MOLECULE:	MITOCHONDRIAL CHAPERONE BCS1
13:00	7wi4-C	4.3	11.4	127	410	13	MOLECULE:	ATP-DEPENDENT ZINC METALLOPROTEASE FTSH
14:00	6sh4-G	4.3	7.9	152	376	13	MOLECULE:	MITOCHONDRIAL CHAPERONE BCS1
15:00	4m4w-O	4.3	4.1	121	250	13	MOLECULE:	REPLICATIVE HELICASE
16:00	8oz7-A	4.2	22.6	142	597	9	MOLECULE:	ABIA
17:00	8q1b-B	4.2	7.4	124	406	10	MOLECULE:	PROBABLE MITOCHONDRIAL-PROCESSING PEPTIDASE SUBUN
18:00	6jpq-A	4.2	8.2	196	577	11	MOLECULE:	UNCHARACTERIZED AAA DOMAIN-CONTAINING PROTEIN C31
19:00	8osf-F	4.2	4.2	115	226	10	MOLECULE:	MAGNESIUM-CHELATASE SUBUNIT CHLI
20:00	7wi4-A	4.2	11.7	128	410	12	MOLECULE:	ATP-DEPENDENT ZINC METALLOPROTEASE FTSH
21:00	5vhn-B	4.2	3.4	135	257	13	MOLECULE:	26S PROTEASOME NON-ATPASE REGULATORY SUBUNIT 10
22:00	6em8-B	4.2	5.4	181	744	15	MOLECULE:	ATP-DEPENDENT CLP PROTEASE ATP-BINDING SUBUNIT CL
23:00	6bog-A	4.1	5.1	174	967	10	MOLECULE:	RNA POLYMERASE-ASSOCIATED PROTEIN RAPA
24:00:00	4f4c-A	4.1	18.7	97	1250	10	MOLECULE:	MULTIDRUG RESISTANCE PROTEIN PGP-1
25:00:00	7ufm-B	4.1	8.1	145	311	8	MOLECULE:	VCHTNSC
26:00:00	7wi4-F	4.1	11.7	134	410	8	MOLECULE:	ATP-DEPENDENT ZINC METALLOPROTEASE FTSH
27:00:00	6ef0-M	4.1	12.1	129	258	15	MOLECULE:	PROTEASOME SUBUNIT ALPHA TYPE-1
28:00:00	4cr4-J	4.1	10.5	164	373	10	MOLECULE:	PROTEASOME COMPONENT PRE3
29:00:00	1w36-C	4	17.8	127	1121	9	MOLECULE:	EXODEOXYRIBONUCLEASE V BETA CHAIN
30:00:00	8osh-D	4	4.4	145	230	7	MOLECULE:	MAGNESIUM-CHELATASE SUBUNIT CHLI
31:00:00	4xgc-D	4	15.6	143	440	8	MOLECULE:	ORIGIN RECOGNITION COMPLEX SUBUNIT 2
32:00:00	6epc-K	4	16.7	87	391	17	MOLECULE:	PROTEASOME SUBUNIT ALPHA TYPE-6
33:00:00	3jb9-X	3.9	27	199	1195	12	MOLECULE:	PRE-MRNA-SPLICING FACTOR SPP42
34:00:00	3tuz-C	3.9	7	110	345	7	MOLECULE:	D-METHIONINE TRANSPORT SYSTEM PERMEASE PROTEIN ME
35:00:00	6mfv-A	3.9	8	123	641	11	MOLECULE:	TETRATRICOPEPTIDE REPEAT SENSOR PH0952

36:00:00	8iqh-C	3.9	6.3	203	891	7	MOLECULE:	PUTATIVE PRIMASE C962R
37:00:00	5g4f-A	3.9	7.5	173	726	9	MOLECULE:	VCP-LIKE ATPASE
38:00:00	8yax-A	3.8	4.7	111	1314	9	MOLECULE:	PAPAIN-LIKE PROTEASE NSP3
39:00:00	5cio-A	3.8	5.6	176	770	7	MOLECULE:	PYRROLOQUINOLINE QUINONE BIOSYNTHESIS PROTEIN PQQ
40:00:00	8vx9-B	3.8	9	165	1174	9	MOLECULE:	HAMA
41:00:00	4nh0-A	3.8	4.2	130	861	12	MOLECULE:	CELL DIVISIONFTSK/SPOIIIE
42:00:00	8hr8-C	3.8	15.2	143	835	10	MOLECULE:	ARCHAEAL ATPASE
43:00:00	8iqh-A	3.8	5.4	202	882	8	MOLECULE:	PUTATIVE PRIMASE C962R
44:00:00	5kzf-K	3.8	10.5	168	498	12	MOLECULE:	PROTEASOME-ASSOCIATED ATPASE
45:00:00	5wcb-E	3.8	10.3	125	277	11	MOLECULE:	MEIOTIC SPINDLE FORMATION PROTEIN MEI-1
46:00:00	5vho-A	3.8	3.4	122	255	11	MOLECULE:	26S PROTEASOME NON-ATPASE REGULATORY SUBUNIT 10
47:00:00	7y8u-F	3.7	8.4	96	347	10	MOLECULE:	ALBE HOMOLOG
48:00:00	7sr6-A	3.7	12.7	166	566	5	MOLECULE:	POLYMERASE
49:00:00	6og1-F	3.7	10.7	140	571	9	MOLECULE:	HYPERACTIVE DISAGGREGASE CLPB
50:00:00	2g49-A	3.6	16	189	970	6	MOLECULE:	INSULIN-DEGRADING ENZYME
51:00:00	3c6k-D	3.6	9.8	108	348	14	MOLECULE:	SPERMINE SYNTHASE
52:00:00	7yv9-A	3.5	12.7	144	1067	9	MOLECULE:	UNCONVENTIONAL MYOSIN-VA
53:00:00	5b7i-A	3.5	13.3	149	1044	11	MOLECULE:	CRISPR-ASSOCIATED NUCLEASE/HELICASE CAS3 SUBTYPE
54:00:00	4ke4-A	3.5	3.5	86	437	5	MOLECULE:	HYDROXYCINNAMOYL-COA:SHIKIMATE HYDROXYCINNAMOYL
55:00:00	6jqh-B	3.5	9.7	107	513	9	MOLECULE:	MADA
56:00:00	8iqh-E	3.5	5.4	199	880	8	MOLECULE:	PUTATIVE PRIMASE C962R
57:00:00	7wi4-E	3.5	10.3	134	410	10	MOLECULE:	ATP-DEPENDENT ZINC METALLOPROTEASE FTSH
58:00:00	8csp-X	3.5	4.3	131	352	11	MOLECULE:	28S RIBOSOMAL PROTEIN S34, MITOCHONDRIAL
59:00:00	4bj6-A	3.5	6	132	293	5	MOLECULE:	RAP1-INTERACTING FACTOR 2
60:00:00	7v2b-D	3.5	7.5	107	376	9	MOLECULE:	VPSR
61:00:00	6ted-Z	3.4	14.8	150	510	5	MOLECULE:	DNA-DIRECTED RNA POLYMERASE SUBUNIT
62:00:00	3ife-A	3.4	10	92	416	7	MOLECULE:	PEPTIDASE T
63:00:00	7w01-A	3.4	7.6	147	1598	12	MOLECULE:	PHOSPHOLIPID-TRANSPORTING ATPASE ABCA3
64:00:00	3qmz-B	3.4	21.8	87	2136	7	MOLECULE:	CYTOPLASMIC DYNEIN HEAVY CHAIN
65:00:00	7nfy-A	3.4	18.8	101	776	17	MOLECULE:	LON PROTEASE HOMOLOG, MITOCHONDRIAL
66:00:00	8iqh-B	3.4	6.7	202	892	6	MOLECULE:	PUTATIVE PRIMASE C962R
67:00:00	6sh4-D	3.4	8.1	144	376	11	MOLECULE:	MITOCHONDRIAL CHAPERONE BCS1
68:00:00	6sh4-E	3.4	7.9	144	376	11	MOLECULE:	MITOCHONDRIAL CHAPERONE BCS1
69:00:00	6sh4-C	3.4	6.8	142	376	11	MOLECULE:	MITOCHONDRIAL CHAPERONE BCS1
70:00:00	7oky-Z	3.3	7.9	114	374	5	MOLECULE:	DNA-DIRECTED RNA POLYMERASE II SUBUNIT RPB1
71:00:00	3fp0-A	3.3	6.5	96	510	7	MOLECULE:	15-O-ACETYLTRANSFERASE
72:00:00	6vh5-D	3.3	4.6	88	283	7	MOLECULE:	PREPHENATE DEHYDRATASE:AMINO ACID-BINDING ACT
73:00:00	4pj3-A	3.3	22.9	196	1335	8	MOLECULE:	INTRON-BINDING PROTEIN AQUARIUS
74:00:00	1g8x-A	3.3	4.7	131	1009	4	MOLECULE:	MYOSIN II HEAVY CHAIN FUSED TO ALPHA-ACTININ 3
75:00:00	7mdm-B	3.3	8.1	101	701	12	MOLECULE:	TRANSITIONAL ENDOPLASMIC RETICULUM ATPASE
76:00:00	8iqh-K	3.3	5.5	203	866	8	MOLECULE:	PUTATIVE PRIMASE C962R
77:00:00	8iqh-G	3.3	5.7	209	863	7	MOLECULE:	PUTATIVE PRIMASE C962R
78:00:00	8iqh-D	3.3	5.7	199	891	8	MOLECULE:	PUTATIVE PRIMASE C962R
79:00:00	4w8f-B	3.3	26	138	2609	7	MOLECULE:	DYNEIN HEAVY CHAIN LYSOZYME CHIMERA
80:00:00	7wae-A	3.2	16.9	136	877	6	MOLECULE:	CYANOPHYCIN SYNTHASE
81:00:00	8e5t-5	3.2	18.8	81	530	10	MOLECULE:	RIBOSOME BIOGENESIS PROTEIN MAK21
82:00:00	5yvu-B	3.2	21.7	129	601	6	MOLECULE:	GENOME POLYPROTEIN
83:00:00	7nhr-C	3.2	18.2	115	565	7	MOLECULE:	PUTATIVE TRANSMEMBRANE PROTEIN WZC
84:00:00	7r9x-A	3.2	14.3	91	418	9	MOLECULE:	AMBE
85:00:00	5ofo-A	3.2	11.9	180	686	8	MOLECULE:	CHAPERONE PROTEIN CLPB,ATP-DEPENDENT CLP PROTEASE
86:00:00	7n7s-B	3.1	18.3	123	441	8	MOLECULE:	HYDROXYMETHYLGLUTARYL-COA REDUCTASE
87:00:00	5k8o-B	3.1	6.8	118	425	4	MOLECULE:	5-NITROANTHRANILIC ACID AMINOHYDROLASE
88:00:00	7t5q-J	3.1	21.9	87	291	7	MOLECULE:	ACTIN-RELATED PROTEIN 3

89:00:00	6c7n-D	3.1	24.2	73	389	10	MOLECULE:	MALIC ENZYME
90:00:00	4ch7-A	3.1	19.4	111	309	5	MOLECULE:	NIRD-LIKE PROTEIN
91:00:00	4qbu-A	3.1	4.4	103	436	10	MOLECULE:	ZMAA
92:00:00	8iqh-F	3.1	6.5	204	880	7	MOLECULE:	PUTATIVE PRIMASE C962R
93:00:00	8iqi-F	3.1	5.5	201	910	6	MOLECULE:	PUTATIVE PRIMASE C962R
94:00:00	6s6b-J	3	12.4	89	475	8	MOLECULE:	CRISPR-ASSOCIATED PROTEIN, CMR5 FAMILY
95:00:00	8dew-A	3	23.1	150	1058	8	MOLECULE:	EFFLUX PUMP MEMBRANE TRANSPORTER
96:00:00	5yhf-A	3	19.4	123	734	7	MOLECULE:	PROTEIN TRANSLOCASE SUBUNIT SECDF
97:00:00	6fsk-A	3	3.6	85	484	6	MOLECULE:	DYP-TYPE PEROXIDASE
98:00:00	6mur-E	3	3.5	88	286	6	MOLECULE:	UNCHARACTERIZED PROTEIN
99:00:00	8iqh-H	3	5.4	201	872	9	MOLECULE:	PUTATIVE PRIMASE C962R
0:00	6qel-J	3	4.4	86	237	16	MOLECULE:	REPLICATIVE DNA HELICASE
1:00	7uoi-A	2.9	7	96	383	3	MOLECULE:	SUCCINYL-DIAMINOPIMELATE DESUCCINYLASE
2:00	8sg4-A	2.9	21.1	90	1469	11	MOLECULE:	METAL RESISTANCE PROTEIN YCF1
3:00	6cse-M	2.9	13.5	98	432	7	MOLECULE:	MONOCLONAL ANTIBODY FAB HEAVY CHAIN
4:00	6em8-E	2.9	7.3	216	664	8	MOLECULE:	ATP-DEPENDENT CLP PROTEASE ATP-BINDING SUBUNIT CL
5:00	6pwy-B	2.8	8.8	125	513	7	MOLECULE:	ZK177.8
6:00	6pns-A	2.8	24.7	169	1291	6	MOLECULE:	RNA-DIRECTED RNA POLYMERASE
7:00	7nhr-A	2.8	17.8	121	663	7	MOLECULE:	PUTATIVE TRANSMEMBRANE PROTEIN WZC
8:00	7r07-F	2.8	16.9	142	600	8	MOLECULE:	ABIK
9:00	5mgu-A	2.7	14.8	102	408	3	MOLECULE:	PHENYLALANINE--TRNA LIGASE, MITOCHONDRIAL
10:00	3nlc-A	2.7	11.4	127	534	6	MOLECULE:	UNCHARACTERIZED PROTEIN VP0956
11:00	4tx3-B	2.7	15.8	108	457	13	MOLECULE:	OXYB PROTEIN
12:00	4yut-A	2.7	14.9	91	351	9	MOLECULE:	FAMILY 3 ADENYLATE CYCLASE
13:00	6neq-g	2.7	4.5	123	351	12	MOLECULE:	28S RIBOSOMAL RNA, MITOCHONDRIAL
14:00	6qel-L	2.7	4.4	78	237	17	MOLECULE:	REPLICATIVE DNA HELICASE
15:00	4xea-A	2.6	4.6	119	423	8	MOLECULE:	PEPTIDASE M16 DOMAIN PROTEIN
16:00	6qel-H	2.6	4.1	73	237	16	MOLECULE:	REPLICATIVE DNA HELICASE
17:00	7p04-A	2.5	19.3	87	1353	14	MOLECULE:	PLEIOTROPIC ABC EFFLUX TRANSPORTER OF MULTIPLE DR
18:00	1q9j-B	2.5	13.6	89	399	2	MOLECULE:	POLYKETIDE SYNTHASE ASSOCIATED PROTEIN 5
19:00	8hwd-E	2.5	4.7	139	247	10	MOLECULE:	PRIMASE D5
20:00	6qel-I	2.5	4.2	73	237	16	MOLECULE:	REPLICATIVE DNA HELICASE
21:00	5vlj-A	2.5	25.6	86	2376	8	MOLECULE:	DYNEIN HEAVY CHAIN, CYTOPLASMIC
22:00	3d3y-A	2.4	6.6	110	425	7	MOLECULE:	UNCHARACTERIZED PROTEIN
23:00	6qel-K	2.4	4.4	68	237	15	MOLECULE:	REPLICATIVE DNA HELICASE
24:00:00	4ap2-B	2.3	21.8	89	359	4	MOLECULE:	KELCH-LIKE PROTEIN 11
25:00:00	5n8o-A	2.3	19.2	80	1432	11	MOLECULE:	DNA HELICASE I
26:00:00	6ahf-F	2.3	8.3	169	574	9	MOLECULE:	HEAT SHOCK PROTEIN 104
27:00:00	3s4w-A	2.2	11.6	84	1206	6	MOLECULE:	FANCONI ANEMIA GROUP I PROTEIN HOMOLOG
28:00:00	7c0q-A	2.2	3.2	59	279	7	MOLECULE:	EFFECTOR LPG2505
29:00:00	6qel-G	2.2	4.3	87	229	18	MOLECULE:	REPLICATIVE DNA HELICASE
30:00:00	5t0j-C	2.2	11.3	154	392	8	MOLECULE:	26S PROTEASOME NON-ATPASE REGULATORY SUBUNIT 2
31:00:00	5twv-B	2.1	19.6	75	1375	11	MOLECULE:	ATP-SENSITIVE INWARD RECTIFIER POTASSIUM CHANNEL
32:00:00	8iqh-I	2.1	7	199	866	7	MOLECULE:	PUTATIVE PRIMASE C962R
33:00:00	7jtk-A	2.1	24.5	124	749	1	MOLECULE:	FLAGELLAR RADIAL SPOKE PROTEIN 1

DALI results: MdA-1 SET

No:	Chain	Z	rmsd	lali	nres	%id	PDB	Description
1:00	4ldg-A	13.8	2.1	125	216	17	MOLECULE:	PROTEIN WITH A SET DOMAIN WITHIN CARBOXY REGION
2:00	7xpx-K	13.7	2.3	124	168	18	MOLECULE:	HISTONE H3
3:00	6uh5-M	12.1	3	116	222	18	MOLECULE:	HISTONE H3
4:00	1h3i-A	11.6	2.7	118	293	19	MOLECULE:	HISTONE H3 LYSINE 4 SPECIFIC METHYLTRANSFERASE
5:00	5kkl-B	11.5	5.1	123	838	20	MOLECULE:	PUTATIVE POLYCOMB PROTEIN EED
6:00	8sr6-A	10.8	2.4	110	452	18	MOLECULE:	EUKARYOTIC HUNTINGTIN INTERACTING PROTEIN B
7:00	8rzu-A	10.7	2.3	121	251	17	MOLECULE:	HISTONE-LYSINE N-METHYLTRANSFERASE SETD2
8:00	5wg6-A	10.7	2.4	118	517	19	MOLECULE:	HISTONE-LYSINE N-METHYLTRANSFERASE EZH2,POLYCOMB
9:00	5h6z-A	10.3	2.6	108	123	13	MOLECULE:	SET DOMAIN-CONTAINING PROTEIN 7
10:00	6wkr-C	10.1	2.7	122	607	19	MOLECULE:	UBIQUITIN
11:00	4o30-A	10.1	3.2	129	218	16	MOLECULE:	HISTONE-LYSINE N-METHYLTRANSFERASE ATXR6, PUTATIV
12:00	6a5n-A	10	2.7	125	502	17	MOLECULE:	HISTONE-LYSINE N-METHYLTRANSFERASE, H3 LYSINE-9 S
13:00	3kma-A	9.8	2.3	94	105	17	MOLECULE:	A612L PROTEIN
14:00	5ecj-A	9.1	3.1	117	263	15	MOLECULE:	PR DOMAIN ZINC FINGER PROTEIN 14,PROTEIN CBFA2T2
15:00	4ijd-A	7.8	2.7	108	215	17	MOLECULE:	HISTONE-LYSINE N-METHYLTRANSFERASE PRDM9
16:00	3n71-A	6.6	2.9	118	469	16	MOLECULE:	HISTONE LYSINE METHYLTRANSFERASE SMYD1
17:00	8t9f-K	6.4	3	106	265	11	MOLECULE:	HISTONE-LYSINE N-METHYLTRANSFERASE KMT5B
18:00	8xzv-I	6.4	3.3	114	413	14	MOLECULE:	DNA-DIRECTED RNA POLYMERASE SUBUNIT ALPHA
19:00	6fnd-A	6	2.9	98	423	13	MOLECULE:	APICAL COMPLEX LYSINE METHYLTRANSFERASE
20:00	6ox1-B	5.5	3.7	112	485	12	MOLECULE:	ACTIN, CYTOPLASMIC 1
21:00	2mpl-A	4.2	3.9	77	127	8	MOLECULE:	ZINC FINGER PROTEIN ZFPM1
22:00	2n1i-A	3.8	3.2	87	176	9	MOLECULE:	PR DOMAIN ZINC FINGER PROTEIN 16

DALI results: MnA-1 Zifi

No:	Chain	Z	rmsd	lali	nres	%id	PDB Description			
1:00	6k7e-A	5.6	3.1	74	498	9	MOLECULE:	MALTOSE	/MALTOD	EXTRIN-BINDING PERIPLASMIC PROTEIN,
2:00	2ig7-B	5.5	2.5	67	342	10	MOLECULE:	CHOLINE	/ETHANO	LAMINE KINASE
3:00	5eqp-A	5.4	2.5	67	352	7	MOLECULE:	CHOLINE	KINASE	ALPHA
4:00	4r80-A	5.4	2.8	68	76	9	MOLECULE:	OR486		
5:00	8bi6-A	5.4	2.5	67	355	7	MOLECULE:	CHOLINE	KINASE	ALPHA
6:00	3g15-A	5.4	2.5	67	354	7	MOLECULE:	CHOLINE	KINASE	ALPHA
7:00	8bi6-B	5.4	2.5	67	354	7	MOLECULE:	CHOLINE	KINASE	ALPHA
8:00	5afv-B	5.3	2.5	67	354	7	MOLECULE:	CHOLINE	KINASE	ALPHA
9:00	5afv-A	5.3	2.5	67	354	7	MOLECULE:	CHOLINE	KINASE	ALPHA
10:00	3f2r-A	5.3	2.5	66	348	8	MOLECULE:	CHOLINE	KINASE	ALPHA
11:00	5eqe-A	5.3	2.5	68	353	7	MOLECULE:	CHOLINE	KINASE	ALPHA
12:00	3zm9-A	5.3	2.9	69	354	9	MOLECULE:	CHOLINE	KINASE	ALPHA
13:00	4r80-B	5.3	2.8	68	76	9	MOLECULE:	OR486		
14:00	7a04-B	5.2	2.8	68	348	9	MOLECULE:	CHOLINE	KINASE	ALPHA
15:00	7sh3-B	5.2	3.2	70	125	7	MOLECULE:	SYNTHET	IC VIRB	8 MINIPROTEIN BINDER
16:00	7a04-A	5.2	2.9	68	348	9	MOLECULE:	CHOLINE	KINASE	ALPHA
17:00	8bi5-B	5.2	2.6	67	354	6	MOLECULE:	CHOLINE	KINASE	ALPHA
18:00	8bi5-A	5.2	2.8	70	355	7	MOLECULE:	CHOLINE	KINASE	ALPHA
19:00	4cga-A	5.2	2.8	69	354	9	MOLECULE:	CHOLINE	KINASE	ALPHA
20:00	4cg9-A	5.2	2.9	68	353	7	MOLECULE:	CHOLINE	KINASE	ALPHA
21:00	3wz4-C	5.2	3.5	73	139	10	MOLECULE:	DOTI		
22:00	7a06-A	5.2	3	70	354	9	MOLECULE:	CHOLINE	KINASE	ALPHA
23:00	5fut-A	5.1	2.9	68	355	9	MOLECULE:	CHOLINE	KINASE	ALPHA
24:00:00	5w6o-A	5.1	2.9	68	352	9	MOLECULE:	CHOLINE	KINASE	ALPHA
25:00:00	5kuy-G	5.1	3.8	70	84	10	MOLECULE:	HEMAGGL	UTININ	HA1
26:00:00	3wz4-D	5.1	3	69	136	9	MOLECULE:	DOTI		
27:00:00	4da5-A	5.1	2.5	65	352	8	MOLECULE:	CHOLINE	KINASE	ALPHA
28:00:00	5eqy-B	5.1	2.5	68	351	6	MOLECULE:	CHOLINE	KINASE	ALPHA
29:00:00	6k7f-A	5	3.2	78	499	9	MOLECULE:	MALTOSE	/MALTOD	EXTRIN-BINDING PERIPLASMIC PROTEIN,
30:00:00	5ftg-A	5	2.9	68	353	9	MOLECULE:	CHOLINE	KINASE	ALPHA
31:00:00	5kuy-H	5	3.9	71	85	10	MOLECULE:	HEMAGGL	UTININ	HA1
32:00:00	5w6o-B	5	2.9	68	353	9	MOLECULE:	CHOLINE	KINASE	ALPHA
33:00:00	6k7d-A	5	3.4	78	499	9	MOLECULE:	MALTOSE	/MALTOD	EXTRIN-BINDING PERIPLASMIC PROTEIN,
34:00:00	4lzi-A	4.9	3.8	77	277	4	MOLECULE:	MULTICY	STATIN	
35:00:00	5eqp-B	4.9	2.5	67	349	6	MOLECULE:	CHOLINE	KINASE	ALPHA
36:00:00	3fi8-A	4.9	2.6	66	339	3	MOLECULE:	CHOLINE	KINASE	
37:00:00	5eqy-A	4.9	2.8	69	352	7	MOLECULE:	CHOLINE	KINASE	ALPHA
38:00:00	3wz4-H	4.9	3.6	73	139	10	MOLECULE:	DOTI		
39:00:00	3h4z-C	4.9	3	75	552	5	MOLECULE:	MALTOSE	#NAME?	G PERIPLASMIC PROTEIN FUSED WITH AL
40:00:00	6i1m-A	4.8	3	69	93	9	MOLECULE:	CYSTATI	N	
41:00:00	3wz4-G	4.8	3.5	73	140	10	MOLECULE:	DOTI		
42:00:00	5kph-A	4.8	2.7	68	85	12	MOLECULE:	DE NOVO	BETA S	HEET DESIGN PROTEIN OR485
43:00:00	6l77-A	4.8	4.3	75	214	8	MOLECULE:	MS5A		
44:00:00	2w9p-G	4.8	3.3	66	87	5	MOLECULE:	MULTICY	STATIN	
45:00:00	4n6t-A	4.8	3.9	68	79	6	MOLECULE:	ADHIRON		
46:00:00	3feg-A	4.8	2.5	62	336	10	MOLECULE:	CHOLINE	/ETHANO	LAMINE KINASE
47:00:00	5kpe-A	4.7	3.8	71	120	7	MOLECULE:	DE NOVO	BETA S	HEET DESIGN PROTEIN OR664
48:00:00	2ig7-A	4.7	2.7	68	335	10	MOLECULE:	CHOLINE	/ETHANO	LAMINE KINASE
49:00:00	4it7-C	4.7	3.4	72	107	7	MOLECULE:	CPI		
50:00:00	6qoz-C	4.7	3.8	68	79	6	MOLECULE:	RNA2 PO	LYPROTE	IN
51:00:00	4it7-D	4.7	3.3	72	107	8	MOLECULE:	CPI		
52:00:00	3zm9-B	4.7	2.6	64	346	8	MOLECULE:	CHOLINE	KINASE	ALPHA

53:00:00	2w9p-F	4.7	3.7	66	87	5	MOLECULE:	MULTICY	STATIN	
54:00:00	2w9p-M	4.7	3.5	66	87	5	MOLECULE:	MULTICY	STATIN	
55:00:00	5eqe-B	4.7	2.5	63	344	8	MOLECULE:	CHOLINE	KINASE	ALPHA
56:00:00	4it7-B	4.7	3.3	72	107	8	MOLECULE:	CPI		
57:00:00	4o3v-A	4.7	3.3	69	138	9	MOLECULE:	VIRB8-L	IKE PRO	TEIN OF TYPE IV SECRETION SYSTEM
58:00:00	6rpv-A	4.7	3.4	74	120	7	MOLECULE:	CYSTATI	N-C	
59:00:00	6w3g-B	4.7	3	68	107	4	MOLECULE:	RD1NTF2	_04	
60:00:00	2w9p-I	4.7	3.5	66	87	6	MOLECULE:	MULTICY	STATIN	
61:00:00	2w9p-K	4.7	3.4	67	87	4	MOLECULE:	MULTICY	STATIN	
62:00:00	4r1k-B	4.6	3.5	80	136	4	MOLECULE:	UNCHARA	CTERIZE	D PROTEIN
63:00:00	4it7-A	4.6	3.3	71	107	7	MOLECULE:	CPI		
64:00:00	2ckp-A	4.6	2.8	64	316	8	MOLECULE:	CHOLINE	KINASE	ALPHA
65:00:00	2uug-D	4.6	2.8	61	82	11	MOLECULE:	URACIL-	DNA GLY	COSYLASE
66:00:00	4n6v-6	4.6	3.3	68	91	9	MOLECULE:	CYSTATI	N-B	
67:00:00	4n6v-1	4.6	3.3	68	91	9	MOLECULE:	CYSTATI	N-B	
68:00:00	4n6u-A	4.6	3.9	67	79	6	MOLECULE:	ADHIRON		
69:00:00	3dc2-B	4.6	3.2	75	524	11	MOLECULE:	D-3-PHO	SPHOGLY	CERATE DEHYDROGENASE
70:00:00	4n6v-7	4.6	3.3	68	91	9	MOLECULE:	CYSTATI	N-B	
71:00:00	4n6v-4	4.6	3.3	68	91	9	MOLECULE:	CYSTATI	N-B	
72:00:00	5ohm-J	4.6	3.7	69	90	4	MOLECULE:	POLYUBI	QUITIN-	C
73:00:00	4br3-A	4.6	2.8	69	354	9	MOLECULE:	CHOLINE	KINASE	ALPHA
74:00:00	2ugi-A	4.6	2.6	60	83	10	MOLECULE:	URACIL-	DNA GLY	COSYLASE INHIBITOR
75:00:00	4n6v-3	4.6	3.3	68	91	9	MOLECULE:	CYSTATI	N-B	
76:00:00	4n6v-9	4.6	3.2	68	91	9	MOLECULE:	CYSTATI	N-B	
77:00:00	2w9p-A	4.6	3.9	69	87	6	MOLECULE:	MULTICY	STATIN	
78:00:00	2w9p-E	4.6	3.8	67	87	4	MOLECULE:	MULTICY	STATIN	
79:00:00	2w9p-D	4.6	3.7	68	87	6	MOLECULE:	MULTICY	STATIN	
80:00:00	6hjl-D	4.6	3.7	68	90	4	MOLECULE:	BCL-2-L	IKE PRO	TEIN 1
81:00:00	2w9p-H	4.6	3.6	67	87	4	MOLECULE:	MULTICY	STATIN	
82:00:00	5ohv-B	4.6	3.5	68	89	4	MOLECULE:	UBIQUIT	IN	
83:00:00	3mes-A	4.6	2.6	62	357	5	MOLECULE:	CHOLINE	KINASE	
84:00:00	2w9p-C	4.6	3.7	68	87	6	MOLECULE:	MULTICY	STATIN	
85:00:00	4lyl-H	4.5	2.9	61	82	11	MOLECULE:	URACIL-	DNA GLY	COSYLASE
86:00:00	2cko-B	4.5	2.5	62	346	8	MOLECULE:	CHOLINE	KINASE	ALPHA
87:00:00	6k8q-A	4.5	3	72	115	7	MOLECULE:	MITOCHO	NDRIAL	IMPORT INNER MEMBRANE TRANSLOCASE S
88:00:00	2ckq-A	4.5	2.7	65	346	8	MOLECULE:	CHOLINE	KINASE	ALPHA
89:00:00	4n6v-8	4.5	3.3	68	91	9	MOLECULE:	CYSTATI	N-B	
90:00:00	1ugh-I	4.5	2.3	56	82	11	MOLECULE:	PROTEIN	(URACI	L-DNA GLYCOSYLASE)
91:00:00	1roa-A	4.5	2.8	65	111	2	MOLECULE:	CYSTATI	N D	
92:00:00	2owp-A	4.5	3.4	76	129	7	MOLECULE:	HYPOTHE	TICAL P	ROTEIN BXE_B1374
93:00:00	6hjl-H	4.5	3.8	67	90	4	MOLECULE:	BCL-2-L	IKE PRO	TEIN 1
94:00:00	4n6v-2	4.5	3.3	68	91	9	MOLECULE:	CYSTATI	N-B	
95:00:00	5ohm-F	4.5	3.7	67	91	4	MOLECULE:	POLYUBI	QUITIN-	C
96:00:00	1ugi-A	4.5	2.8	62	83	11	MOLECULE:	URACIL-	DNA GLY	COSYLASE INHIBITOR
97:00:00	3wz4-A	4.5	3	69	137	9	MOLECULE:	DOTI		
98:00:00	2w9p-B	4.5	3.8	68	87	6	MOLECULE:	MULTICY	STATIN	
99:00:00	2w9p-J	4.5	3.8	69	87	6	MOLECULE:	MULTICY	STATIN	
0:00	2mzv-A	4.5	3.3	73	199	4	MOLECULE:	CYSTATI	N	
1:00	2w9p-N	4.5	3.4	65	87	5	MOLECULE:	MULTICY	STATIN	
2:00	3dc2-A	4.5	3.3	79	527	9	MOLECULE:	D-3-PHO	SPHOGLY	CERATE DEHYDROGENASE
3:00	3ima-D	4.5	3.6	68	85	6	MOLECULE:	PAPAIN		
4:00	1ugi-G	4.5	2.7	61	83	11	MOLECULE:	URACIL-	DNA GLY	COSYLASE INHIBITOR
5:00	2q83-A	4.4	2.7	57	332	7	MOLECULE:	YTAA PR	OTEIN	
6:00	1ylx-A	4.4	3.3	68	100	6	MOLECULE:	HYPOTHE	TICAL P	ROTEIN APC35702

7:00	4ec6-A	4.4	3	71	109	6	MOLECULE:	PUTATIV	E UNCHA	RACTERIZED PROTEIN
8:00	1rn7-A	4.4	3	64	112	2	MOLECULE:	CYSTATI	N D	
9:00	6lg3-A	4.4	2.8	60	74	10	MOLECULE:	PHIRV1	PHAGE P	ROTEIN
10:00	2q83-B	4.4	2.6	57	332	7	MOLECULE:	YTAA PR	OTEIN	
11:00	4cg8-A	4.4	2.9	68	353	9	MOLECULE:	CHOLINE	KINASE	ALPHA
12:00	5elj-A	4.4	3.8	69	90	4	MOLECULE:	SUMO-AD	HIRON-S	2D5
13:00	1ugi-H	4.4	3	62	83	11	MOLECULE:	URACIL-	DNA GLY	COSYLASE INHIBITOR
14:00	4ec6-C	4.4	2.9	69	109	6	MOLECULE:	PUTATIV	E UNCHA	RACTERIZED PROTEIN
15:00	2ciu-A	4.4	3.7	71	123	8	MOLECULE:	IMPORT	INNER M	EMBRANE TRANSLOCASE SUBUNIT TIM21
16:00	6yxs-A	4.4	2.4	64	359	3	MOLECULE:	CHOLINE	KINASE	
17:00	3wz5-D	4.4	3	69	135	9	MOLECULE:	DOTI		
18:00	4ec6-F	4.4	3	71	109	7	MOLECULE:	PUTATIV	E UNCHA	RACTERIZED PROTEIN
19:00	4ec6-D	4.4	2.9	69	109	4	MOLECULE:	PUTATIV	E UNCHA	RACTERIZED PROTEIN
20:00	5ohv-D	4.4	3.6	67	90	4	MOLECULE:	UBIQUIT	IN	
21:00	6stj-F	4.4	3.6	65	90	5	MOLECULE:	INDUCED	MYELOI	D LEUKEMIA CELL DIFFERENTIATION PRO
22:00	7ds3-A	4.4	3.3	71	266	4	MOLECULE:	F-ACTIN	#NAME?	G PROTEIN SUBUNIT ALPHA-1
23:00	3h3h-A	4.4	3.1	71	118	4	MOLECULE:	UNCHARA	CTERIZE	D SNOAL-LIKE PROTEIN
24:00:00	6pai-C	4.4	3.3	74	401	7	MOLECULE:	DNA DAM	AGE-BIN	DING PROTEIN 1
25:00:00	1nw1-A	4.4	3.1	72	365	8	MOLECULE:	CHOLINE	KINASE	(49.2 KD)
26:00:00	2rcd-B	4.4	3.4	74	128	11	MOLECULE:	UNCHARA	CTERIZE	D PROTEIN
27:00:00	2w9q-A	4.4	3.7	68	87	6	MOLECULE:	MULTICY	STATIN	
28:00:00	8aim-G	4.4	2.8	59	86	7	MOLECULE:	URACIL-	DNA GLY	COSYLASE INHIBITOR
29:00:00	3wz4-F	4.4	3	69	136	9	MOLECULE:	DOTI		
30:00:00	5ohm-B	4.4	3.2	65	89	6	MOLECULE:	POLYUBI	QUITIN-	C
31:00:00	3ddn-A	4.4	3.4	79	527	9	MOLECULE:	D-3-PHO	SPHOGLY	CERATE DEHYDROGENASE
32:00:00	5ohm-H	4.4	3.6	66	89	5	MOLECULE:	POLYUBI	QUITIN-	C
33:00:00	8aim-B	4.3	2.9	62	86	6	MOLECULE:	URACIL-	DNA GLY	COSYLASE INHIBITOR
34:00:00	4ccv-A	4.3	3.4	70	115	7	MOLECULE:	HISTIDI	NE-RICH	GLYCOPROTEIN
35:00:00	3f14-A	4.3	3.6	72	112	4	MOLECULE:	UNCHARA	CTERIZE	D NTF2-LIKE PROTEIN
36:00:00	1ygy-A	4.3	3.3	79	527	9	MOLECULE:	D-3-PHO	SPHOGLY	CERATE DEHYDROGENASE
37:00:00	7eeb-H	4.3	3.5	74	916	5	MOLECULE:	ENHANCE	D GREEN	FLUORESCENT PROTEIN,CATION CHANNEL
38:00:00	4br3-B	4.3	2.9	67	349	7	MOLECULE:	CHOLINE	KINASE	ALPHA
39:00:00	5uxd-B	4.3	2.5	57	299	7	MOLECULE:	MACROLI	DE 2'-P	HOSPHOTRANSFERASE MPHH
40:00:00	3fka-C	4.3	3.5	70	119	7	MOLECULE:	UNCHARA	CTERIZE	D NTF-2 LIKE PROTEIN
41:00:00	2ckq-B	4.3	2.8	68	348	9	MOLECULE:	CHOLINE	KINASE	ALPHA
42:00:00	5mn2-D	4.3	3.7	65	90	5	MOLECULE:	LOW AFF	INITY I	MMUNOGLOBULIN GAMMA FC REGION RECEP
43:00:00	6q0w-C	4.3	3	67	192	7	MOLECULE:	DNA DAM	AGE-BIN	DING PROTEIN 1
44:00:00	7z8i-K	4.3	3.5	74	278	3	MOLECULE:	ARP1 AC	TIN REL	ATED PROTEIN 1 HOMOLOG A
45:00:00	7ccc-C	4.3	3.8	74	269	3	MOLECULE:	ACTIN,	ALPHA S	KELETAL MUSCLE
46:00:00	8ixi-B	4.3	2.5	57	295	5	MOLECULE:	ALDEHYD	E DEHYD	ROGENASE PROTEIN
47:00:00	5uxb-B	4.3	2.5	57	297	7	MOLECULE:	MACROLI	DE 2'-P	HOSPHOTRANSFERASE MPHH
48:00:00	4ec6-E	4.3	2.8	69	109	4	MOLECULE:	PUTATIV	E UNCHA	RACTERIZED PROTEIN
49:00:00	6z0o-E	4.3	3.6	67	90	4	MOLECULE:	NUCLEOC	APSID	
50:00:00	2cko-A	4.3	2.6	63	344	10	MOLECULE:	CHOLINE	KINASE	ALPHA
51:00:00	5ohm-D	4.3	3.5	64	89	5	MOLECULE:	POLYUBI	QUITIN-	C
52:00:00	6stj-G	4.3	3.6	63	88	5	MOLECULE:	INDUCED	MYELOI	D LEUKEMIA CELL DIFFERENTIATION PRO
53:00:00	6stj-E	4.3	3.6	64	89	5	MOLECULE:	INDUCED	MYELOI	D LEUKEMIA CELL DIFFERENTIATION PRO
54:00:00	3c5i-B	4.3	3.4	67	345	4	MOLECULE:	CHOLINE	KINASE	
55:00:00	7ds4-A	4.3	3.5	74	270	5	MOLECULE:	F-ACTIN	#NAME?	G PROTEIN SUBUNIT ALPHA-1
56:00:00	3h4z-A	4.3	2.9	75	568	5	MOLECULE:	MALTOSE	#NAME?	G PERIPLASMIC PROTEIN FUSED WITH AL
57:00:00	6gki-A	4.3	3	68	185	1	MOLECULE:	PROBABL	E PHOSP	HOLIPID-BINDING PROTEIN MLAC
58:00:00	5igw-A	4.3	2.5	56	296	9	MOLECULE:	MACROLI	DE 2'-P	HOSPHOTRANSFERASE II
59:00:00	1ylx-B	4.3	3.2	70	100	6	MOLECULE:	HYPOTHE	TICAL P	ROTEIN APC35702
60:00:00	3gax-A	4.3	2.9	69	107	3	MOLECULE:	CYSTATI	N-C	

61:00:00	4r1k-A	4.3	2.8	72	135	7	MOLECULE:	UNCHARA	CTERIZE	D PROTEIN	
62:00:00	3wz5-B	4.3	2.9	69	135	9	MOLECULE:	DOTI			
63:00:00	9b8o-P	4.3	2.9	76	204	16	MOLECULE:	ATPASE	H+-TRAN	SPORTING V1 SUBUNIT D	
64:00:00	3mwz-A	4.3	4	72	115	6	MOLECULE:	SIALOST	ATIN L2		
65:00:00	3nx0-A	4.3	3.6	72	109	7	MOLECULE:	CYSTATI	N-C		
66:00:00	6roa-B	4.3	3.6	72	109	7	MOLECULE:	CYSTATI	N-C		
67:00:00	5ml9-B	4.3	4	71	103	4	MOLECULE:	LOW AFF	INITY I	MMUNOGLOBULIN GAMMA FC REGION RECEP	
68:00:00	3ima-B	4.3	3.6	67	84	6	MOLECULE:	PAPAIN			
69:00:00	6roa-A	4.3	3.5	71	109	7	MOLECULE:	CYSTATI	N-C		
70:00:00	2bo9-D	4.3	4.1	75	217	7	MOLECULE:	CARBOXY	PEPTIDA	SE A4	
71:00:00	6hjl-C	4.3	3.8	69	90	6	MOLECULE:	BCL-2-L	IKE PRO	TEIN 1	
72:00:00	2zhx-N	4.3	2.6	58	82	12	MOLECULE:	URACIL-	DNA GLY	COSYLASE	
73:00:00	5igh-A	4.2	2.7	59	301	14	MOLECULE:	MACROLI	DE 2'-P	HOSPHOTRANSFERASE	
74:00:00	3fka-A	4.2	3.2	68	119	7	MOLECULE:	UNCHARA	CTERIZE	D NTF-2 LIKE PROTEIN	
75:00:00	5uxb-A	4.2	2.3	55	290	7	MOLECULE:	MACROLI	DE 2'-P	HOSPHOTRANSFERASE MPHH	
76:00:00	2zhx-B	4.2	3	59	82	10	MOLECULE:	URACIL-	DNA GLY	COSYLASE	
77:00:00	6yr8-B	4.2	3.6	64	89	5	MOLECULE:	GTPASE	KRAS		
78:00:00	5n9j-W	4.2	4.1	77	463	9	MOLECULE:	MEDIATO	R OF RN	A POLYMERASE II TRANSCRIPTION SUBUN	
79:00:00	5ohm-L	4.2	3.9	66	89	5	MOLECULE:	POLYUBI	QUITIN-	C	
80:00:00	5iwu-A	4.2	2.6	58	299	9	MOLECULE:	MACROLI	DE 2'-P	HOSPHOTRANSFERASE II	
81:00:00	5u0s-Q	4.2	4.2	76	500	9	MOLECULE:	MEDIATO	R COMPL	EX SUBUNIT 6	
82:00:00	3c5i-D	4.2	3.2	68	355	6	MOLECULE:	CHOLINE	KINASE		
83:00:00	8ixi-A	4.2	2.4	58	297	7	MOLECULE:	ALDEHYD	E DEHYD	ROGENASE PROTEIN	
84:00:00	6swt-B	4.2	3.2	64	88	5	MOLECULE:	ALPHA-A	CTININ-		2
85:00:00	6stj-H	4.2	3.6	63	88	5	MOLECULE:	INDUCED	MYELOI	D LEUKEMIA CELL DIFFERENTIATION PRO	
86:00:00	5igv-A	4.2	2.5	57	298	9	MOLECULE:	MACROLI	DE 2'-P	HOSPHOTRANSFERASE II	
87:00:00	2pun-B	4.2	2.6	58	378	3	MOLECULE:	METHYLT	HIORIBO	SE KINASE	
88:00:00	5l33-A	4.2	3.3	69	106	12	MOLECULE:	DENOVO	NTF2		
89:00:00	5elu-A	4.2	3.6	65	91	5	MOLECULE:	SUMO-AD	HIRON-S	2B3	
90:00:00	3c5i-C	4.2	3.5	69	353	6	MOLECULE:	CHOLINE	KINASE		
91:00:00	6q0r-C	4.2	3.5	72	183	7	MOLECULE:	DNA DAM	AGE-BIN	DING PROTEIN 1	
92:00:00	5ih0-A	4.2	2.6	58	299	9	MOLECULE:	MACROLI	DE 2'-P	HOSPHOTRANSFERASE II	
93:00:00	5igy-A	4.2	2.3	55	295	9	MOLECULE:	MACROLI	DE 2'-P	HOSPHOTRANSFERASE II	
94:00:00	6uio-C	4.2	2.9	69	112	7	MOLECULE:	CYSTATI	N-8		
95:00:00	6uio-B	4.2	3	69	110	7	MOLECULE:	CYSTATI	N-8		
96:00:00	3wz4-B	4.2	3.4	68	137	10	MOLECULE:	DOTI			
97:00:00	3c5i-A	4.2	3.3	66	343	5	MOLECULE:	CHOLINE	KINASE		
98:00:00	4ec6-B	4.2	3.1	68	109	7	MOLECULE:	PUTATIV	E UNCHA	RACTERIZED PROTEIN	
99:00:00	8pk3-I	4.2	3.2	66	87	6	MOLECULE:	HEMAGGL	UTININ	HA1 CHAIN	
0:00	1nb3-I	4.2	2.9	66	98	5	MOLECULE:	CATHEPS	IN H		
1:00	1kwi-A	4.2	2.7	61	85	3	MOLECULE:	PROTEGR	IN-3 PR	ECURSOR	
2:00	1stf-I	4.2	3.9	70	98	7	MOLECULE:	PAPAIN			
3:00	3k9m-D	4.2	4.2	69	98	7	MOLECULE:	CATHEPS	IN B		
4:00	1nb3-J	4.2	4	71	98	8	MOLECULE:	CATHEPS	IN H		
5:00	2mf7-A	4.2	4.4	78	127	8	MOLECULE:	MITOCHO	NDRIAL	IMPORT INNER MEMBRANE TRANSLOCASE S	
6:00	1ygy-B	4.2	3.1	73	527	11	MOLECULE:	D-3-PHO	SPHOGLY	CERATE DEHYDROGENASE	
7:00	6sj7-A	4.2	3.2	74	355	8	MOLECULE:	DDB1- A	ND CUL4	-ASSOCIATED FACTOR 15	
8:00	3fka-B	4.1	3.2	69	120	7	MOLECULE:	UNCHARA	CTERIZE	D NTF-2 LIKE PROTEIN	
9:00	6bjt-B	4.1	3.1	71	130	6	MOLECULE:	DUF4440	DOMAIN	-CONTAINING PROTEIN	
10:00	1nnv-A	4.1	3.8	67	107	3	MOLECULE:	HYPOTHE	TICAL P	ROTEIN HI1450	
11:00	8gqq-A	4.1	3.1	66	99	3	MOLECULE:	CYSTATI	N-A2		
12:00	4i4k-A	4.1	3.2	72	138	8	MOLECULE:	UNCHARA	CTERIZE	D PROTEIN SGCJ	
13:00	7pkw-A	4.1	3.4	70	240	9	MOLECULE:	PUTATIV	E TRANS	FER PROTEIN	
14:00	4n6l-A	4.1	2.8	67	114	3	MOLECULE:	CYSTATI	N-M		

15:00	3lh4-A	4.1	4.3	73	115	7	MOLECULE:	SECRETE	D CYSTA	TIN
16:00	3b8l-A	4.1	4.1	71	147	6	MOLECULE:	UNCHARA	CTERIZE	D PROTEIN
17:00	4pw1-B	4.1	3.3	73	215	8	MOLECULE:	UNCHARA	CTERIZE	D PROTEIN
18:00	3fka-D	4.1	3.2	68	119	7	MOLECULE:	UNCHARA	CTERIZE	D NTF-2 LIKE PROTEIN
19:00	8gt7-D	4.1	3.7	70	98	9	MOLECULE:	CYSTEIN	E PROTE	INASE FALCIPAIN 2A
20:00	5a0o-B	4.1	3.1	64	93	5	MOLECULE:	ADHIRON		
21:00	6w3d-A	4.1	2.8	69	114	7	MOLECULE:	RD1NTF2	_05	
22:00	5mn2-C	4.1	3.6	63	88	5	MOLECULE:	LOW AFF	INITY I	MMUNOGLOBULIN GAMMA FC REGION RECEP
23:00	1spu-B	4.1	3.3	67	720	6	MOLECULE:	COPPER	AMINE O	XIDASE
24:00:00	1nb3-K	4.1	2.9	65	98	5	MOLECULE:	CATHEPS	IN H	
25:00:00	3kfq-D	4.1	3	66	98	8	MOLECULE:	CATHEPS	IN L2	
26:00:00	5zc1-C	4.1	3.5	70	100	6	MOLECULE:	CYSTATI	N-1	
27:00:00	4qvh-B	4.1	3.1	74	595	1	MOLECULE:	MALTOSE	#NAME?	G PERIPLASMIC PROTEIN, 4'-PHOSPHOPA
28:00:00	3buu-B	4.1	4	76	220	8	MOLECULE:	UNCHARA	CTERIZE	D LOLA SUPERFAMILY PROTEIN NE2245
29:00:00	2olc-A	4.1	1.8	51	376	4	MOLECULE:	METHYLT	HIORIBO	SE KINASE
30:00:00	5uxd-A	4.1	2.5	57	300	7	MOLECULE:	MACROLI	DE 2'-P	HOSPHOTRANSFERASE MPHH
31:00:00	3ub1-A	4.1	2.8	69	248	0	MOLECULE:	ORF13-L	IKE PRO	TEIN
32:00:00	7pk4-B	4.1	3.2	58	81	5	MOLECULE:	CATHEPS	IN L2	
33:00:00	5zc1-F	4.1	3.4	67	98	4	MOLECULE:	CYSTATI	N-1	
34:00:00	6uio-D	4.1	2.8	67	109	7	MOLECULE:	CYSTATI	N-8	
35:00:00	5uxa-A	4.1	2.3	55	301	11	MOLECULE:	MACROLI	DE 2'-P	HOSPHOTRANSFERASE II
36:00:00	6yxw-D	4.1	3.4	62	88	5	MOLECULE:	GTPASE	KRAS	
37:00:00	6qb2-A	4.1	2.9	65	95	5	MOLECULE:	MONOMEI	OF SQT	-1C
38:00:00	3gax-B	4.1	3.5	70	107	4	MOLECULE:	CYSTATI	N-C	
39:00:00	3wz5-A	4.1	2.9	69	135	9	MOLECULE:	DOTI		
40:00:00	3nx0-B	4.1	3.6	68	109	6	MOLECULE:	CYSTATI	N-C	
41:00:00	1nb5-I	4.1	4.1	69	98	7	MOLECULE:	CATHEPS	IN H	
42:00:00	3blz-A	4.1	3.5	68	124	9	MOLECULE:	NTF2-LI	KE PROT	EIN OF UNKNOWN FUNCTION
43:00:00	7w1a-A	4.1	2.6	56	287	5	MOLECULE:	MACROLI	DE 2'-P	HOSPHOTRANSFERASE
44:00:00	8pk3-G	4.1	3.2	67	87	6	MOLECULE:	HEMAGGL	UTININ	HA1 CHAIN
45:00:00	7ny8-C	4.1	4.2	69	91	4	MOLECULE:	GTPASE	KRAS	
46:00:00	6st2-C	4.1	3.7	66	87	5	MOLECULE:	BCL-2-L	IKE PRO	TEIN 1
47:00:00	1nb5-J	4.1	3.1	67	98	4	MOLECULE:	CATHEPS	IN H	
48:00:00	6st2-D	4.1	3.7	64	87	5	MOLECULE:	BCL-2-L	IKE PRO	TEIN 1
49:00:00	6zgn-A	4.1	3.2	63	101	5	MOLECULE:	PUTATIV	E TRANS	FER PROTEIN
50:00:00	3blz-H	4.1	3.4	70	123	10	MOLECULE:	NTF2-LI	KE PROT	EIN OF UNKNOWN FUNCTION
51:00:00	5zc1-E	4.1	3.2	70	98	7	MOLECULE:	CYSTATI	N-1	
52:00:00	3blz-C	4.1	3.3	68	124	9	MOLECULE:	NTF2-LI	KE PROT	EIN OF UNKNOWN FUNCTION
53:00:00	5o46-B	4.1	4	70	114	3	MOLECULE:	IRISTAT	IN	
54:00:00	1nb5-K	4.1	3	66	98	6	MOLECULE:	CATHEPS	IN H	
55:00:00	1n5h-A	4	3	66	105	3	MOLECULE:	PROTEGR	INS	
56:00:00	7rag-A	4	3.3	61	170	5	MOLECULE:	LIPOPRO	TEIN	
57:00:00	5ts4-B	4	3.1	71	106	6	MOLECULE:	DENOVO	NTF2	
58:00:00	3mes-B	4	2.6	62	358	5	MOLECULE:	CHOLINE	KINASE	
59:00:00	1wnh-A	4	3.7	68	220	4	MOLECULE:	LATEXIN		
60:00:00	5ih1-A	4	2.5	56	297	9	MOLECULE:	MACROLI	DE 2'-P	HOSPHOTRANSFERASE II
61:00:00	6yxw-B	4	3.1	63	89	5	MOLECULE:	GTPASE	KRAS	
62:00:00	4n6m-B	4	2.8	66	116	3	MOLECULE:	CYSTATI	N-M	
63:00:00	3kfq-C	4	3.2	68	98	4	MOLECULE:	CATHEPS	IN L2	
64:00:00	8f8q-G	4	3.9	75	278	3	MOLECULE:	ACTIN,	ALPHA S	KELETAL MUSCLE
65:00:00	3kse-F	4	3.6	72	98	4	MOLECULE:	CATHEPS	IN L1	
66:00:00	5cnl-A	4	3.3	72	139	13	MOLECULE:	ICML-LI	KE	
67:00:00	4akr-A	4	3.2	75	269	4	MOLECULE:	F-ACTIN	#NAME?	G PROTEIN SUBUNIT ALPHA
68:00:00	3lq3-A	4	2.5	63	339	10	MOLECULE:	CHOLINE	/ETHANO	LAMINE KINASE

69:00:00	3wz4-E	4	3.6	73	139	10	MOLECULE:	DOTI		
70:00:00	2j8x-B	4	2.5	58	83	12	MOLECULE:	URACIL-	DNA GLY	COSYLASE
71:00:00	8pk3-H	4	3	64	87	5	MOLECULE:	HEMAGGL	UTININ	HA1 CHAIN
72:00:00	5a0o-S	4	3.9	71	93	6	MOLECULE:	ADHIRON		
73:00:00	7dsb-A	4	3.5	73	264	5	MOLECULE:	F-ACTIN	#NAME?	G PROTEIN SUBUNIT ALPHA-1
74:00:00	7ds8-A	4	3.3	74	274	5	MOLECULE:	F-ACTIN	#NAME?	G PROTEIN SUBUNIT ALPHA-1
75:00:00	5jou-A	4	3.3	66	945	11	MOLECULE:	ALPHA-X	YLOSIDA	SE BOGH31A
76:00:00	1lqm-D	4	2.5	59	83	14	MOLECULE:	URACIL-	DNA GLY	COSYLASE
77:00:00	3h4z-B	4	3	74	563	5	MOLECULE:	MALTOSE	#NAME?	G PERIPLASMIC PROTEIN FUSED WITH AL
78:00:00	3blz-B	4	3.1	66	124	9	MOLECULE:	NTF2-LI	KE PROT	EIN OF UNKNOWN FUNCTION
79:00:00	1cew-I	4	3.6	69	108	4	MOLECULE:	CYSTATI	N	
80:00:00	1ugi-E	4	2.8	60	83	13	MOLECULE:	URACIL-	DNA GLY	COSYLASE INHIBITOR
81:00:00	1nb3-L	4	3	66	98	8	MOLECULE:	CATHEPS	IN H	
82:00:00	3ub1-E	4	3	71	252	1	MOLECULE:	ORF13-L	IKE PRO	TEIN
83:00:00	7w1a-B	4	2.6	56	287	5	MOLECULE:	MACROLI	DE 2'-P	HOSPHOTRANSFERASE
84:00:00	3kse-D	4	3.2	69	98	4	MOLECULE:	CATHEPS	IN L1	
85:00:00	5igp-A	4	3.1	59	300	17	MOLECULE:	MACROLI	DE 2'-P	HOSPHOTRANSFERASE
86:00:00	5zc1-B	4	3.4	68	101	9	MOLECULE:	CYSTATI	N-1	
87:00:00	3ub1-F	4	2.9	68	251	0	MOLECULE:	ORF13-L	IKE PRO	TEIN
88:00:00	7knc-A	4	2.9	58	919	7	MOLECULE:	ALPHA-X	YLOSIDA	SE
89:00:00	8hbn-B	3.9	3.2	71	165	11	MOLECULE:	MRNA EX	PORT FA	CTOR MEX67
90:00:00	2lyx-A	3.9	3.1	63	87	3	MOLECULE:	UNCHARA	CTERIZE	D PROTEIN YQZG
91:00:00	3ub1-D	3.9	3.8	70	253	14	MOLECULE:	ORF13-L	IKE PRO	TEIN
92:00:00	6ud7-A	3.9	3.4	73	426	5	MOLECULE:	DDB1- A	ND CUL4	-ASSOCIATED FACTOR 15
93:00:00	3buu-A	3.9	3.7	75	224	7	MOLECULE:	UNCHARA	CTERIZE	D LOLA SUPERFAMILY PROTEIN NE2245
94:00:00	6wlw-U	3.9	3	76	205	12	MOLECULE:	V-TYPE	PROTON	ATPASE 21 KDA PROTEOLIPID SUBUNIT
95:00:00	3en8-A	3.9	3.3	73	128	8	MOLECULE:	UNCHARA	CTERIZE	D NTF-2 LIKE PROTEIN
96:00:00	6w3g-A	3.9	3.1	69	109	4	MOLECULE:	RD1NTF2	_04	
97:00:00	4k90-B	3.9	3	71	207	6	MOLECULE:	EXTRACE	LLULAR	METALLOPROTEINASE MEP
98:00:00	7w15-A	3.9	2.6	56	294	5	MOLECULE:	MACROLI	DE 2'-P	HOSPHOTRANSFERASE
99:00:00	4n6m-A	3.9	2.8	66	117	3	MOLECULE:	CYSTATI	N-M	
0:00	5uxc-A	3.9	2.5	57	296	7	MOLECULE:	PREDICT	ED AMIN	OGLYCOSIDE PHOSPHOTRANSFERASE
1:00	7w15-B	3.9	2.5	56	293	5	MOLECULE:	MACROLI	DE 2'-P	HOSPHOTRANSFERASE
2:00	3ub1-C	3.9	3.7	69	251	14	MOLECULE:	ORF13-L	IKE PRO	TEIN
3:00	6ihj-D	3.9	3.7	74	122	3	MOLECULE:	NUCLEAR	RNA EX	PORT FACTOR 1
4:00	6e5u-H	3.9	2.6	69	138	12	MOLECULE:	NUCLEAR	RNA EX	PORT FACTOR 1
5:00	3aa6-A	3.9	3.3	73	270	5	MOLECULE:	F-ACTIN	#NAME?	G PROTEIN SUBUNIT ALPHA-1
6:00	1lvn-B	3.9	3.3	67	720	6	MOLECULE:	COPPER	AMINE O	XIDASE
7:00	1ugi-B	3.9	2.7	60	83	13	MOLECULE:	URACIL-	DNA GLY	COSYLASE INHIBITOR
8:00	6ta7-D	3.9	3.1	70	124	6	MOLECULE:	RAS GTP	ASE-ACT	IVATING PROTEIN-BINDING PROTEIN 1
9:00	6q0v-C	3.9	3.1	69	192	7	MOLECULE:	DNA DAM	AGE-BIN	DING PROTEIN 1
10:00	4n4x-A	3.9	3	71	535	10	MOLECULE:	MALTOSE	#NAME?	G PERIPLASMIC/PALATE LUNG AND NASAL
11:00	2r4i-B	3.9	3.3	69	121	3	MOLECULE:	UNCHARA	CTERIZE	D PROTEIN
12:00	1ugi-C	3.9	2.6	59	83	14	MOLECULE:	URACIL-	DNA GLY	COSYLASE INHIBITOR
13:00	2olc-B	3.9	2.1	55	376	4	MOLECULE:	METHYLT	HIORIBO	SE KINASE
14:00	1udi-I	3.9	2.7	59	83	14	MOLECULE:	URACIL-	DNA GLY	COSYLASE
15:00	3blz-G	3.9	3.3	70	124	10	MOLECULE:	NTF2-LI	KE PROT	EIN OF UNKNOWN FUNCTION
16:00	1lqm-F	3.9	2.8	59	82	14	MOLECULE:	URACIL-	DNA GLY	COSYLASE
17:00	5igr-A	3.9	3.4	59	300	15	MOLECULE:	MACROLI	DE 2'-P	HOSPHOTRANSFERASE
18:00	3blz-K	3.9	3	64	123	9	MOLECULE:	NTF2-LI	KE PROT	EIN OF UNKNOWN FUNCTION
19:00	5o46-A	3.9	3.8	69	114	3	MOLECULE:	IRISTAT	IN	
20:00	4lyl-J	3.9	2.5	58	82	14	MOLECULE:	URACIL-	DNA GLY	COSYLASE
21:00	5igj-A	3.9	3.4	59	300	15	MOLECULE:	MACROLI	DE 2'-P	HOSPHOTRANSFERASE
22:00	7ny8-D	3.9	3.3	62	97	5	MOLECULE:	GTPASE	KRAS	

23:00	3blz-L	3.9	3.4	70	123	10	MOLECULE:	NTF2-LI	KE PROT	EIN OF UNKNOWN FUNCTION
24:00:00	2w9p-L	3.9	3.8	70	87	7	MOLECULE:	MULTICY	STATIN	
25:00:00	5igs-A	3.9	3.3	59	300	15	MOLECULE:	MACROLI	DE 2'-P	HOSPHOTRANSFERASE
26:00:00	1ugi-F	3.9	2.9	61	83	13	MOLECULE:	URACIL-	DNA GLY	COSYLASE INHIBITOR
27:00:00	1lvn-A	3.9	3.2	63	718	6	MOLECULE:	COPPER	AMINE O	XIDASE
28:00:00	5zc1-A	3.9	3.4	67	101	9	MOLECULE:	CYSTATI	N-1	
29:00:00	3blz-F	3.9	3.5	70	123	10	MOLECULE:	NTF2-LI	KE PROT	EIN OF UNKNOWN FUNCTION
30:00:00	5cnl-B	3.9	3.3	71	139	13	MOLECULE:	ICML-LI	KE	
31:00:00	3kse-E	3.9	3.6	71	98	4	MOLECULE:	CATHEPS	IN L1	
32:00:00	2rgq-B	3.8	3.3	72	134	10	MOLECULE:	DOMAIN	OF UNKN	OWN FUNCTION WITH A CYSTATIN-LIKE F
33:00:00	8cgm-A	3.8	3.2	70	190	7	MOLECULE:	OUTER M	EMBRANE	LIPOPROTEIN CARRIER PROTEIN LOLA
34:00:00	1jkg-A	3.8	2.9	71	139	11	MOLECULE:	P15		
35:00:00	2qiy-A	3.8	2.9	67	134	10	MOLECULE:	UBP3-AS	SOCIATE	D PROTEIN BRE5
36:00:00	3f7s-A	3.8	3.5	68	142	7	MOLECULE:	UNCHARA	CTERIZE	D NTF2-LIKE PROTEIN
37:00:00	5uwa-A	3.8	3.1	66	185	0	MOLECULE:	PROBABL	E PHOSP	HOLIPID-BINDING PROTEIN MLAC
38:00:00	7mhu-A	3.8	3.4	61	374	11	MOLECULE:	EXO-ALP	HA-SIAL	IDASE
39:00:00	2gu3-A	3.8	2.9	59	128	3	MOLECULE:	YPMB PR	OTEIN	
40:00:00	1lqm-H	3.8	2.7	59	84	14	MOLECULE:	URACIL-	DNA GLY	COSYLASE
41:00:00	4k61-B	3.8	2.8	61	138	8	MOLECULE:	UNCHARA	CTERIZE	D PROTEIN
42:00:00	2rgq-A	3.8	3.3	72	133	10	MOLECULE:	DOMAIN	OF UNKN	OWN FUNCTION WITH A CYSTATIN-LIKE F
43:00:00	5ts4-A	3.8	3.1	69	101	6	MOLECULE:	DENOVO	NTF2	
44:00:00	4lyl-D	3.8	3.1	62	82	13	MOLECULE:	URACIL-	DNA GLY	COSYLASE
45:00:00	5igz-A	3.8	2.6	57	299	9	MOLECULE:	MACROLI	DE 2'-P	HOSPHOTRANSFERASE II
46:00:00	5tgn-C	3.8	3.7	71	107	7	MOLECULE:	UNCHARA	CTERIZE	D PROTEIN
47:00:00	1nb5-L	3.8	3	64	98	5	MOLECULE:	CATHEPS	IN H	
48:00:00	1yvb-I	3.8	3.4	65	111	6	MOLECULE:	FALCIPA	IN 2	
49:00:00	3ub1-B	3.8	3.7	70	252	14	MOLECULE:	ORF13-L	IKE PRO	TEIN
50:00:00	1dvc-A	3.8	3.2	62	98	5	MOLECULE:	STEFIN	A	
51:00:00	6hjl-G	3.8	3.6	63	88	5	MOLECULE:	BCL-2-L	IKE PRO	TEIN 1
52:00:00	4h63-Q	3.8	4	67	410	7	MOLECULE:	MEDIATO	R OF RN	A POLYMERASE II TRANSCRIPTION SUBUN
53:00:00	3nv0-B	3.8	3.2	72	136	3	MOLECULE:	NUCLEAR	RNA EX	PORT FACTOR 2
54:00:00	6yxt-A	3.8	2.6	63	359	5	MOLECULE:	CHOLINE	KINASE	
55:00:00	7ds2-A	3.8	3.3	73	272	5	MOLECULE:	F-ACTIN	#NAME?	G PROTEIN SUBUNIT ALPHA-1
56:00:00	7ds6-A	3.8	3.4	74	275	5	MOLECULE:	F-ACTIN	#NAME?	G PROTEIN SUBUNIT ALPHA-1
57:00:00	6ue5-A	3.8	3.3	73	424	7	MOLECULE:	DDB1- A	ND CUL4	-ASSOCIATED FACTOR 15
58:00:00	6znl-K	3.8	3.9	73	278	4	MOLECULE:	ARP1 AC	TIN REL	ATED PROTEIN 1 HOMOLOG A
59:00:00	2wo0-B	3.8	3.3	67	720	7	MOLECULE:	PRIMARY	AMINE	OXIDASE
60:00:00	5uwa-B	3.8	3.1	68	185	0	MOLECULE:	PROBABL	E PHOSP	HOLIPID-BINDING PROTEIN MLAC
61:00:00	1a67-A	3.8	3.4	73	107	5	MOLECULE:	CYSTATI	N	
62:00:00	5ts4-C	3.8	3.3	70	104	6	MOLECULE:	DENOVO	NTF2	
63:00:00	7kmp-A	3.8	2.6	57	920	7	MOLECULE:	ALPHA-X	YLOSIDA	SE
64:00:00	1lqm-B	3.8	2.5	58	82	14	MOLECULE:	URACIL-	DNA GLY	COSYLASE
65:00:00	1eqk-A	3.8	3.6	69	102	9	MOLECULE:	ORYZACY	STATIN-	I
66:00:00	6grr-A	3.8	3.4	69	718	10	MOLECULE:	AMINE O	XIDASE	
67:00:00	8cgm-B	3.8	3.6	71	190	8	MOLECULE:	OUTER M	EMBRANE	LIPOPROTEIN CARRIER PROTEIN LOLA
68:00:00	4lyl-F	3.8	2.8	60	82	13	MOLECULE:	URACIL-	DNA GLY	COSYLASE
69:00:00	6e5u-F	3.8	2.6	69	138	12	MOLECULE:	NUCLEAR	RNA EX	PORT FACTOR 1
70:00:00	3wz3-A	3.8	3.1	69	137	4	MOLECULE:	TRAM PR	OTEIN	
71:00:00	4z48-B	3.8	4.4	73	240	5	MOLECULE:	UNCHARA	CTERIZE	D PROTEIN
72:00:00	5tgn-B	3.8	3.5	71	108	7	MOLECULE:	UNCHARA	CTERIZE	D PROTEIN
73:00:00	4lyl-L	3.8	2.7	60	82	13	MOLECULE:	URACIL-	DNA GLY	COSYLASE
74:00:00	4ebg-A	3.8	3.4	71	97	4	MOLECULE:	UNCHARA	CTERIZE	D PROTEIN
75:00:00	5tgn-D	3.8	3.7	69	109	9	MOLECULE:	UNCHARA	CTERIZE	D PROTEIN
76:00:00	4lyl-N	3.8	2.6	58	82	14	MOLECULE:	URACIL-	DNA GLY	COSYLASE

77:00:00	1qal-A	3.8	3.1	64	718	11	MOLECULE:	COPPER	AMINE O	XIDASE
78:00:00	6uio-A	3.8	3.4	62	112	8	MOLECULE:	CYSTATI	N-8	
79:00:00	4wyk-D	3.8	2.6	69	138	12	MOLECULE:	NUCLEAR	RNA EX	PORT FACTOR 1
80:00:00	3blz-D	3.8	3.3	73	124	11	MOLECULE:	NTF2-LI	KE PROT	EIN OF UNKNOWN FUNCTION
81:00:00	2bo9-B	3.8	3.1	66	217	9	MOLECULE:	CARBOXY	PEPTIDA	SE A4
82:00:00	1jrq-A	3.8	3	64	718	11	MOLECULE:	COPPER	AMINE O	XIDASE
83:00:00	1ugi-D	3.8	2.9	61	82	13	MOLECULE:	URACIL-	DNA GLY	COSYLASE INHIBITOR
84:00:00	6ezz-A	3.8	3.1	66	718	9	MOLECULE:	PRIMARY	AMINE	OXIDASE
85:00:00	3blz-I	3.8	3.3	67	123	9	MOLECULE:	NTF2-LI	KE PROT	EIN OF UNKNOWN FUNCTION
86:00:00	2m7o-A	3.7	3.2	60	70	7	MOLECULE:	UNCHARA	CTERIZE	D PROTEIN
87:00:00	1oac-B	3.7	3.2	66	723	8	MOLECULE:	COPPER	AMINE O	XIDASE
88:00:00	7zhu-B	3.7	4.5	65	543	3	MOLECULE:	GLUCOSE	#NAME?	PHATE 1-DEHYDROGENASE
89:00:00	5tgn-A	3.7	3.3	66	109	8	MOLECULE:	UNCHARA	CTERIZE	D PROTEIN
90:00:00	1nkg-A	3.7	3	47	508	6	MOLECULE:	RHAMNOG	ALACTUR	ONASE B
91:00:00	3ke7-B	3.7	3.9	73	132	7	MOLECULE:	PUTATIV	E KETOS	TEROID ISOMERASE
92:00:00	5jov-A	3.7	2.9	65	949	12	MOLECULE:	ALPHA-X	YLOSIDA	SE BOGH31A
93:00:00	2pyw-A	3.7	2.4	62	417	5	MOLECULE:	UNCHARA	CTERIZE	D PROTEIN
94:00:00	8wzb-G	3.7	4.2	70	387	3	MOLECULE:	DPY30 D	OMAIN C	ONTAINING 2
95:00:00	4exr-A	3.7	3.4	59	152	12	MOLECULE:	PUTATIV	E LIPOP	ROTEIN
96:00:00	6lyd-I	3.7	2.8	60	83	10	MOLECULE:	PROBABL	E URACI	L-DNA GLYCOSYLASE
97:00:00	3b8l-F	3.7	4	70	143	9	MOLECULE:	UNCHARA	CTERIZE	D PROTEIN
98:00:00	4k61-A	3.7	2.8	61	140	8	MOLECULE:	UNCHARA	CTERIZE	D PROTEIN
99:00:00	1dvd-A	3.7	3.5	66	98	5	MOLECULE:	STEFIN	A	
0:00	5tph-A	3.7	4.2	69	120	6	MOLECULE:	DE NOVO	NTF2 H	OMODIMER
1:00	8gt0-D	3.7	3.6	71	98	3	MOLECULE:	CYSTEIN	E PROTE	ASE FALCIPAIN-2
2:00	4wwu-I	3.7	3.3	73	161	11	MOLECULE:	MRNA EX	PORT FA	CTOR MEX67
3:00	4wwu-L	3.7	3.3	73	162	11	MOLECULE:	MRNA EX	PORT FA	CTOR MEX67
4:00	2uug-C	3.7	2.5	58	82	14	MOLECULE:	URACIL-	DNA GLY	COSYLASE
5:00	2r4i-C	3.7	3.5	69	120	3	MOLECULE:	UNCHARA	CTERIZE	D PROTEIN
6:00	1d6u-B	3.7	2.8	63	720	8	MOLECULE:	COPPER	AMINE O	XIDASE
7:00	6z0o-F	3.7	3.9	66	90	5	MOLECULE:	NUCLEOC	APSID	
8:00	2zhx-J	3.7	2.6	59	82	12	MOLECULE:	URACIL-	DNA GLY	COSYLASE
9:00	3b8l-C	3.7	3.8	71	144	8	MOLECULE:	UNCHARA	CTERIZE	D PROTEIN
10:00	3b8l-E	3.7	3.9	71	144	8	MOLECULE:	UNCHARA	CTERIZE	D PROTEIN
11:00	3b8l-B	3.7	3.6	71	144	8	MOLECULE:	UNCHARA	CTERIZE	D PROTEIN
12:00	3b8l-D	3.7	3.8	71	144	8	MOLECULE:	UNCHARA	CTERIZE	D PROTEIN
13:00	2wyh-B	3.7	3.1	76	891	5	MOLECULE:	ALPHA-M	ANNOSID	ASE
14:00	1ask-B	3.7	3.3	70	120	10	MOLECULE:	NUCLEAR	TRANSP	ORT FACTOR 2
15:00	4jf8-A	3.7	3	63	144	6	MOLECULE:	TRWG CO	MPONENT	OF TYPE IV SECRETION SYSTEM
16:00	5zc1-D	3.7	3.4	70	98	4	MOLECULE:	CYSTATI	N-1	
17:00	9bry-c	3.7	3.1	77	203	12	MOLECULE:	V-TYPE	PROTON	ATPASE SUBUNIT S1
18:00	2vw2-A	3.7	3.4	63	658	11	MOLECULE:	SIALIDA	SE B	
19:00	4ttg-B	3.7	3	51	1015	4	MOLECULE:	BETA-GA	LACTOSI	DASE
20:00	2zhx-H	3.7	2.8	60	82	12	MOLECULE:	URACIL-	DNA GLY	COSYLASE
21:00	2pul-B	3.7	1.9	50	369	4	MOLECULE:	METHYLT	HIORIBO	SE KINASE
22:00	4fq4-A	3.7	3.5	62	658	8	MOLECULE:	SIALIDA	SE B	
23:00	3k9m-C	3.7	3	66	98	5	MOLECULE:	CATHEPS	IN B	
24:00:00	2qiy-B	3.7	3.2	67	133	12	MOLECULE:	UBP3-AS	SOCIATE	D PROTEIN BRE5
25:00:00	1uug-B	3.7	2.6	58	82	12	MOLECULE:	URACIL-	DNA GLY	COSYLASE
26:00:00	1zx2-A	3.7	3.5	73	130	12	MOLECULE:	UBP3-AS	SOCIATE	D PROTEIN BRE5
27:00:00	6ihj-B	3.7	3.8	73	128	3	MOLECULE:	NUCLEAR	RNA EX	PORT FACTOR 1
28:00:00	6wm3-U	3.7	3.1	78	204	10	MOLECULE:	V-TYPE	PROTON	ATPASE 116 KDA SUBUNIT A ISOFORM 1
29:00:00	1qaf-A	3.7	3.1	63	718	11	MOLECULE:	PROTEIN	(COPPE	R AMINE OXIDASE)
30:00:00	2pun-A	3.7	2	51	380	4	MOLECULE:	METHYLT	HIORIBO	SE KINASE

31:00:00	6grr-B	3.7	3.2	65	720	8	MOLECULE:	AMINE O	XIDASE	
32:00:00	2ugi-B	3.7	2.8	57	83	11	MOLECULE:	URACIL-	DNA GLY	COSYLASE INHIBITOR
33:00:00	3blz-J	3.7	3.5	67	124	9	MOLECULE:	NTF2-LI	KE PROT	EIN OF UNKNOWN FUNCTION
34:00:00	5eql-A	3.7	3.3	61	82	3	MOLECULE:	SUMO-AD	HIRON-S	2D5
35:00:00	6mrk-U	3.7	3.4	70	132	10	MOLECULE:	NUCLEAR	RNA EX	PORT FACTOR 2
36:00:00	4wyk-B	3.7	2.7	69	138	12	MOLECULE:	NUCLEAR	RNA EX	PORT FACTOR 1
37:00:00	6w90-A	3.6	3.3	68	130	13	MOLECULE:	NTF2 FO	LD PROT	EIN LOOP-HELIX-LOOP DESIGN NT-9
38:00:00	4hbr-A	3.6	3.1	62	140	5	MOLECULE:	PUTATIV	E PERIP	LASMIC PROTEIN
39:00:00	4o3v-B	3.6	3.2	69	140	9	MOLECULE:	VIRB8-L	IKE PRO	TEIN OF TYPE IV SECRETION SYSTEM
40:00:00	3u1w-C	3.6	3.2	60	250	5	MOLECULE:	HYPOTHE	TICAL P	ERIPLASMIC PROTEIN
41:00:00	2l4v-A	3.6	3.7	74	135	5	MOLECULE:	CYSTATI	N	
42:00:00	5u0p-Q	3.6	4.1	75	508	9	MOLECULE:	MEDIATO	R COMPL	EX SUBUNIT 14
43:00:00	4z48-A	3.6	4.5	77	240	3	MOLECULE:	UNCHARA	CTERIZE	D PROTEIN
44:00:00	7a0h-A	3.6	3.4	76	285	5	MOLECULE:	F-ACTIN	#NAME?	G PROTEIN SUBUNIT ALPHA
45:00:00	2r4i-A	3.6	3.7	69	123	3	MOLECULE:	UNCHARA	CTERIZE	D PROTEIN
46:00:00	4ebg-B	3.6	3.4	70	99	6	MOLECULE:	UNCHARA	CTERIZE	D PROTEIN
47:00:00	6myv-C	3.6	3.8	64	526	6	MOLECULE:	SIALIDA	SE26	
48:00:00	2wyh-A	3.6	3.1	76	905	5	MOLECULE:	ALPHA-M	ANNOSID	ASE
49:00:00	3cu3-A	3.6	3.4	71	162	3	MOLECULE:	DOMAIN	OF UNKN	OWN FUNCTION WITH A CYSTATIN-LIKE F
50:00:00	6s6z-A	3.6	3.5	75	1083	4	MOLECULE:	BETA-GA	LACTOSI	DASE
51:00:00	8ppr-K	3.6	4.2	63	246	8	MOLECULE:	KINETOC	HORE-AS	SOCIATED PROTEIN DSN1 HOMOLOG
52:00:00	3h3h-B	3.6	3.1	71	120	4	MOLECULE:	UNCHARA	CTERIZE	D SNOAL-LIKE PROTEIN
53:00:00	7to3-A	3.6	4.5	75	568	9	MOLECULE:	CAP2		
54:00:00	3bcy-A	3.6	3.3	72	146	8	MOLECULE:	PROTEIN	YER067	W
55:00:00	3dxq-A	3.6	3.5	57	294	2	MOLECULE:	CHOLINE	/ETHANO	LAMINE KINASE FAMILY PROTEIN
56:00:00	3db7-A	3.6	2.7	50	127	12	MOLECULE:	PUTATIV	E CALCI	UM-REGULATED PERIPLASMIC PROTEIN
57:00:00	3d00-A	3.6	3.3	65	184	11	MOLECULE:	TUNGSTE	N FORMY	LMETHANOFURAN DEHYDROGENASE SUBUNIT
58:00:00	7e8h-I	3.6	4.1	64	760	11	MOLECULE:	DIPEPTI	DYL AMI	NOPEPTIDASE-LIKE PROTEIN 6
59:00:00	4hbr-D	3.6	3.2	60	139	3	MOLECULE:	PUTATIV	E PERIP	LASMIC PROTEIN
60:00:00	3aae-A	3.6	3.6	74	270	5	MOLECULE:	F-ACTIN	#NAME?	G PROTEIN SUBUNIT ALPHA-1
61:00:00	4wwu-F	3.6	2.9	70	164	9	MOLECULE:	MRNA EX	PORT FA	CTOR MEX67
62:00:00	5u35-A	3.6	4.2	73	124	5	MOLECULE:	DE NOVO	NTF2 W	ITH LARGE CAVITY
63:00:00	1zx2-B	3.6	3.4	72	130	11	MOLECULE:	UBP3-AS	SOCIATE	D PROTEIN BRE5
64:00:00	6bjt-A	3.6	3.2	72	128	7	MOLECULE:	DUF4440	DOMAIN	-CONTAINING PROTEIN
65:00:00	3lk4-M	3.6	3.3	73	269	5	MOLECULE:	F-ACTIN	#NAME?	G PROTEIN SUBUNIT ALPHA-1
66:00:00	3lk4-P	3.6	3.3	73	269	5	MOLECULE:	F-ACTIN	#NAME?	G PROTEIN SUBUNIT ALPHA-1
67:00:00	3lk4-G	3.6	3.3	73	269	5	MOLECULE:	F-ACTIN	#NAME?	G PROTEIN SUBUNIT ALPHA-1
68:00:00	2rcd-A	3.6	3.3	73	128	4	MOLECULE:	UNCHARA	CTERIZE	D PROTEIN
69:00:00	6ta7-B	3.6	3.4	67	129	6	MOLECULE:	RAS GTP	ASE-ACT	IVATING PROTEIN-BINDING PROTEIN 1
70:00:00	2rcd-C	3.6	3.3	73	128	4	MOLECULE:	UNCHARA	CTERIZE	D PROTEIN
71:00:00	6e5u-D	3.6	3.1	73	138	5	MOLECULE:	NUCLEAR	RNA EX	PORT FACTOR 1
72:00:00	3lk4-1	3.6	3.3	73	269	5	MOLECULE:	F-ACTIN	#NAME?	G PROTEIN SUBUNIT ALPHA-1
73:00:00	1gy6-A	3.6	3.5	72	125	11	MOLECULE:	NUCLEAR	TRANSP	ORT FACTOR 2
74:00:00	1oun-A	3.6	3.4	71	125	11	MOLECULE:	NUCLEAR	TRANSP	ORT FACTOR 2
75:00:00	3lk4-A	3.6	3.3	73	269	5	MOLECULE:	F-ACTIN	#NAME?	G PROTEIN SUBUNIT ALPHA-1
76:00:00	3ke7-A	3.6	4	72	131	7	MOLECULE:	PUTATIV	E KETOS	TEROID ISOMERASE
77:00:00	3lk4-Y	3.6	3.3	73	269	5	MOLECULE:	F-ACTIN	#NAME?	G PROTEIN SUBUNIT ALPHA-1
78:00:00	3lk4-J	3.6	3.3	73	269	5	MOLECULE:	F-ACTIN	#NAME?	G PROTEIN SUBUNIT ALPHA-1
79:00:00	6e5u-B	3.6	3.2	73	138	5	MOLECULE:	NUCLEAR	RNA EX	PORT FACTOR 1
80:00:00	1of5-B	3.6	3.2	67	128	9	MOLECULE:	MRNA EX	PORT FA	CTOR MEX67
81:00:00	2rcd-D	3.6	3.3	73	128	4	MOLECULE:	UNCHARA	CTERIZE	D PROTEIN
82:00:00	3lk4-D	3.6	3.3	73	269	5	MOLECULE:	F-ACTIN	#NAME?	G PROTEIN SUBUNIT ALPHA-1
83:00:00	3lk4-V	3.6	3.3	73	269	5	MOLECULE:	F-ACTIN	#NAME?	G PROTEIN SUBUNIT ALPHA-1
84:00:00	2owp-B	3.6	3.5	76	129	11	MOLECULE:	HYPOTHE	TICAL P	ROTEIN BXE_B1374

85:00:00	4ipb-A	3.6	3.1	63	141	3	MOLECULE:	UNCHARA	CTERIZE	D PROTEIN
86:00:00	3lk4-7	3.6	3.3	73	269	5	MOLECULE:	F-ACTIN	#NAME?	G PROTEIN SUBUNIT ALPHA-1
87:00:00	1ask-A	3.6	3.4	72	125	11	MOLECULE:	NUCLEAR	TRANSP	ORT FACTOR 2
88:00:00	1ar0-A	3.6	3.4	71	125	11	MOLECULE:	NUCLEAR	TRANSP	ORT FACTOR 2
89:00:00	1uug-D	3.6	2.8	58	82	10	MOLECULE:	URACIL-	DNA GLY	COSYLASE
90:00:00	3lk4-S	3.6	3.3	73	269	5	MOLECULE:	F-ACTIN	#NAME?	G PROTEIN SUBUNIT ALPHA-1
91:00:00	2zhx-D	3.6	2.6	59	82	12	MOLECULE:	URACIL-	DNA GLY	COSYLASE
92:00:00	3blz-E	3.6	3	65	124	9	MOLECULE:	NTF2-LI	KE PROT	EIN OF UNKNOWN FUNCTION
93:00:00	2wyi-A	3.6	3.1	76	896	5	MOLECULE:	ALPHA-M	ANNOSID	ASE
94:00:00	6wm4-U	3.6	3	78	204	12	MOLECULE:	V-TYPE	PROTON	ATPASE 116 KDA SUBUNIT A ISOFORM 1
95:00:00	4fpf-A	3.6	3.4	62	658	8	MOLECULE:	SIALIDA	SE B	
96:00:00	7to3-B	3.6	4.5	75	568	9	MOLECULE:	CAP2		
97:00:00	2j8x-D	3.6	2.5	57	81	14	MOLECULE:	URACIL-	DNA GLY	COSYLASE
98:00:00	6s6z-B	3.6	3.6	71	1083	4	MOLECULE:	BETA-GA	LACTOSI	DASE
99:00:00	5bxq-A	3.6	3.3	70	124	10	MOLECULE:	NUCLEAR	TRANSP	ORT FACTOR 2
0:00	1jz7-B	3.6	3	51	1011	4	MOLECULE:	BETA-GA	LACTOSI	DASE
1:00	4lyl-P	3.6	2.6	59	82	12	MOLECULE:	URACIL-	DNA GLY	COSYLASE
2:00	1oun-B	3.6	3.3	70	121	10	MOLECULE:	NUCLEAR	TRANSP	ORT FACTOR 2
3:00	1ar0-B	3.6	3.3	70	121	10	MOLECULE:	NUCLEAR	TRANSP	ORT FACTOR 2
4:00	5bxq-B	3.6	3.3	70	124	10	MOLECULE:	NUCLEAR	TRANSP	ORT FACTOR 2
5:00	1a2k-A	3.6	3.3	70	124	10	MOLECULE:	NUCLEAR	TRANSP	ORT FACTOR 2
6:00	6drv-B	3.6	2.8	48	1024	4	MOLECULE:	BETA-GA	LACTOSI	DASE
7:00	2cc3-B	3.6	2.5	65	144	9	MOLECULE:	PROTEIN	VIRB8	
8:00	6wm2-U	3.6	3	76	205	12	MOLECULE:	V-TYPE	PROTON	ATPASE SUBUNIT E 1
9:00	4lyl-B	3.6	2.7	59	82	12	MOLECULE:	URACIL-	DNA GLY	COSYLASE
10:00	1jb4-A	3.6	3.4	70	123	9	MOLECULE:	NUCLEAR	TRANSP	ORT FACTOR 2
11:00	5trv-A	3.6	3.2	68	118	6	MOLECULE:	DENOVO	NTF2	
12:00	6bju-C	3.6	3	71	125	6	MOLECULE:	ATZH		
13:00	7tqd-B	3.6	4.5	74	513	11	MOLECULE:	CAP2		
14:00	6bju-A	3.6	3.1	72	125	6	MOLECULE:	ATZH		
15:00	4fpy-A	3.6	3.5	62	658	6	MOLECULE:	SIALIDA	SE B	
16:00	8hbn-A	3.5	3.3	72	371	3	MOLECULE:	MRNA EX	PORT FA	CTOR MEX67
17:00	3w0s-A	3.5	2.6	54	298	6	MOLECULE:	HYGROMY	CIN-B 4	-O-KINASE
18:00	4gbf-B	3.5	3.3	73	370	8	MOLECULE:	PHIKZ13	1	
19:00	3a76-B	3.5	3	69	153	9	MOLECULE:	GAMMA-H	EXACHLO	ROCYCLOHEXANE DEHYDROCHLORINASE
20:00	7zhm-C	3.5	3.3	60	87	5	MOLECULE:	RHS1 PR	OTEIN	
21:00	2kxg-A	3.5	3.7	64	95	6	MOLECULE:	ASPARTI	C PROTE	ASE INHIBITOR
22:00	7cd7-A	3.5	3.3	64	99	6	MOLECULE:	GREEN F	LUORESC	ENT PROTEIN
23:00	8v1k-A	3.5	3.2	65	185	5	MOLECULE:	OUTER-M	EMBRANE	LIPOPROTEIN CARRIER PROTEIN
24:00:00	7abw-B	3.5	3.3	60	365	5	MOLECULE:	PEPSY D	OMAIN-C	ONTAINING PROTEIN
25:00:00	7kd9-A	3.5	3.7	77	231	3	MOLECULE:	GALLATE	DECARB	OXYLASE
26:00:00	7bcz-A	3.5	4	72	242	6	MOLECULE:	4'-PHOS	PHOPANT	ETHEINYL TRANSFERASE ENTD
27:00:00	5iqc-A	3.5	3.4	62	301	10	MOLECULE:	BIFUNCT	IONAL A	AC/APH
28:00:00	6ekt-A	3.5	4.2	77	419	5	MOLECULE:	P-47 PR	OTEIN	
29:00:00	3cxj-A	3.5	3.3	60	144	2	MOLECULE:	UNCHARA	CTERIZE	D PROTEIN
30:00:00	7sjy-A	3.5	3.8	67	313	6	MOLECULE:	ANTI-SI	GMA-I F	ACTOR RSGI9
31:00:00	3qv0-A	3.5	3.3	59	179	10	MOLECULE:	MITOCHO	NDRIAL	ACIDIC PROTEIN MAM33
32:00:00	1u5o-A	3.5	3.5	73	125	11	MOLECULE:	NUCLEAR	TRANSP	ORT FACTOR 2
33:00:00	7prq-B	3.5	2.3	63	316	2	MOLECULE:	PROBABL	E CHEMO	TAXIS TRANSDUCER
34:00:00	3k0z-A	3.5	3.6	75	149	9	MOLECULE:	PUTATIV	E POLYK	ETIDE CYCLASE
35:00:00	4std-A	3.5	3	65	164	5	MOLECULE:	SCYTALO	NE DEHY	DRATASE
36:00:00	6ya7-A	3.5	3.8	61	350	5	MOLECULE:	CELL DI	VISION	CYCLE 7-RELATED PROTEIN KINASE,CELL
37:00:00	3g16-A	3.5	4.2	72	156	3	MOLECULE:	UNCHARA	CTERIZE	D PROTEIN WITH CYSTATIN-LIKE FOLD
38:00:00	8hsb-B	3.5	3.7	69	159	17	MOLECULE:	CDNG		

39:00:00	5cd6-A	3.5	3	75	575	8	MOLECULE:	TPR-DOM	AIN CON	TAINING PROTEIN	
40:00:00	3ecf-A	3.5	3.3	66	128	3	MOLECULE:	NTF2-LI	KE PROT	EIN	
41:00:00	2k54-A	3.5	3.8	73	123	7	MOLECULE:	PROTEIN	ATU074		2
42:00:00	3f7w-A	3.5	2.2	52	288	4	MOLECULE:	PUTATIV	E FRUCT	OSAMINE-3-KINASE	
43:00:00	5aiw-A	3.5	4	71	127	7	MOLECULE:	TRAH			
44:00:00	2cc3-A	3.5	2.3	61	144	10	MOLECULE:	PROTEIN	VIRB8		
45:00:00	5evh-A	3.5	3.6	66	121	11	MOLECULE:	UNCHARA	CTERIZE	D PROTEIN	
46:00:00	3tdw-A	3.5	2.1	53	302	6	MOLECULE:	GENTAMI	CIN RES	ISTANCE PROTEIN	
47:00:00	6w3f-A	3.5	3.4	67	115	7	MOLECULE:	RD1NTF2	_05_I64	F_A80G_T94P_D101K_L106W	
48:00:00	4wwu-C	3.5	2.9	70	165	9	MOLECULE:	MRNA EX	PORT FA	CTOR MEX67	
49:00:00	1qma-A	3.5	3.5	73	126	11	MOLECULE:	NUCLEAR	TRANSP	ORT FACTOR 2	
50:00:00	3ddn-B	3.5	3.2	77	525	9	MOLECULE:	D-3-PHO	SPHOGLY	CERATE DEHYDROGENASE	
51:00:00	1gy6-B	3.5	3.5	72	123	11	MOLECULE:	NUCLEAR	TRANSP	ORT FACTOR 2	
52:00:00	5nw4-Y	3.5	3.6	74	275	4	MOLECULE:	DYNEIN	HEAVY C	HAIN	
53:00:00	5adx-K	3.5	3.6	74	275	4	MOLECULE:	ACTIN R	ELATED	PROTEIN 1	
54:00:00	4eyc-B	3.5	3.5	63	99	5	MOLECULE:	CATHELI	CIDIN A	NTIMICROBIAL PEPTIDE	
55:00:00	1qak-A	3.5	3.3	67	719	6	MOLECULE:	COPPER	AMINE O	XIDASE	
56:00:00	1d6y-B	3.5	3.3	67	720	7	MOLECULE:	COPPER	AMINE O	XIDASE	
57:00:00	1n5p-A	3.5	3.2	64	105	3	MOLECULE:	PROTEGR	INS		
58:00:00	6w3f-B	3.5	3.4	66	114	8	MOLECULE:	RD1NTF2	_05_I64	F_A80G_T94P_D101K_L106W	
59:00:00	1qma-C	3.5	3.5	73	126	11	MOLECULE:	NUCLEAR	TRANSP	ORT FACTOR 2	
60:00:00	4hbr-B	3.5	3.4	60	140	3	MOLECULE:	PUTATIV	E PERIP	LASMIC PROTEIN	
61:00:00	4ovm-F	3.5	3	70	129	9	MOLECULE:	UNCHARA	CTERIZE	D PROTEIN SGCJ	
62:00:00	8wzb-S	3.5	4.3	69	392	7	MOLECULE:	DPY30 D	OMAIN C	ONTAINING 2	
63:00:00	1u5o-B	3.5	3.4	70	121	10	MOLECULE:	NUCLEAR	TRANSP	ORT FACTOR 2	
64:00:00	4pw1-A	3.5	3.3	71	214	8	MOLECULE:	UNCHARA	CTERIZE	D PROTEIN	
65:00:00	8u2w-A	3.5	5.1	69	482	3	MOLECULE:	ACETYL-	COENZYM	E A SYNTHETASE	
66:00:00	6ztk-A	3.5	3.2	62	108	5	MOLECULE:	MIALOST	ATIN		
67:00:00	1jb2-A	3.5	3.4	70	123	10	MOLECULE:	NUCLEAR	TRANSP	ORT FACTOR 2	
68:00:00	5u35-B	3.5	3.3	69	121	1	MOLECULE:	DE NOVO	NTF2 W	ITH LARGE CAVITY	
69:00:00	3l0r-B	3.5	3.7	64	108	3	MOLECULE:	CYSTATI	N-2		
70:00:00	5kbp-B	3.5	3.4	80	884	8	MOLECULE:	GLYCOSY	L HYDRO	LASE, FAMILY 38	
71:00:00	2w0q-B	3.5	3.3	66	719	8	MOLECULE:	COPPER	AMINE O	XIDASE	
72:00:00	1gy5-A	3.5	3.1	66	123	11	MOLECULE:	NUCLEAR	TRANSP	ORT FACTOR 2	
73:00:00	1jb5-A	3.5	3.3	67	123	9	MOLECULE:	NUCLEAR	TRANSP	ORT FACTOR 2	
74:00:00	1qma-D	3.5	3.4	70	124	10	MOLECULE:	NUCLEAR	TRANSP	ORT FACTOR 2	
75:00:00	6ztk-B	3.5	3.5	65	110	5	MOLECULE:	MIALOST	ATIN		
76:00:00	4ttg-C	3.5	3.1	51	1015	4	MOLECULE:	BETA-GA	LACTOSI	DASE	
77:00:00	1a2k-B	3.5	3.3	70	124	10	MOLECULE:	NUCLEAR	TRANSP	ORT FACTOR 2	
78:00:00	3vd9-A	3.5	3	50	1015	6	MOLECULE:	BETA-GA	LACTOSI	DASE	
79:00:00	3l0r-A	3.5	3.6	64	108	3	MOLECULE:	CYSTATI	N-2		
80:00:00	2xvl-A	3.5	2.7	63	944	11	MOLECULE:	ALPHA-X	YLOSIDA	SE, PUTATIVE, XYL31A	
81:00:00	3vd9-D	3.5	3.1	51	1015	4	MOLECULE:	BETA-GA	LACTOSI	DASE	
82:00:00	1qma-B	3.5	3.4	70	124	10	MOLECULE:	NUCLEAR	TRANSP	ORT FACTOR 2	
83:00:00	9brz-c	3.5	3.1	76	203	11	MOLECULE:	V-TYPE	PROTON	ATPASE SUBUNIT S1	
84:00:00	2zhx-L	3.5	2.9	62	82	11	MOLECULE:	URACIL-	DNA GLY	COSYLASE	
85:00:00	5igt-A	3.5	3.4	59	300	15	MOLECULE:	MACROLI	DE 2'-P	HOSPHOTRANSFERASE	
86:00:00	4uvq-A	3.4	3.2	75	263	12	MOLECULE:	THIAZOL	INE OXI	DASE/SUBTILISIN-LIKE PROTEASE	
87:00:00	2xf3-B	3.4	3.1	68	428	6	MOLECULE:	ORF12			
88:00:00	6p7l-A	3.4	3.5	64	140	6	MOLECULE:	ALN2			
89:00:00	3pgb-A	3.4	3.7	83	740	4	MOLECULE:	PUTATIV	E UNCHA	RACTERIZED PROTEIN	
90:00:00	6e8a-A	3.4	3.8	65	148	9	MOLECULE:	DUF1795	DOMAIN	-CONTAINING PROTEIN	
91:00:00	4nlm-A	3.4	4.5	65	326	3	MOLECULE:	LMO1340	PROTEI	N	
92:00:00	8gm7-C	3.4	3	61	170	7	MOLECULE:	TK0353			

93:00:00	3ef8-A	3.4	3.2	67	149	9	MOLECULE:	PUTATIV	E SCYAL	ONE DEHYDRATASE
94:00:00	3nct-D	3.4	3.7	73	140	7	MOLECULE:	PROTEIN	PSIB	
95:00:00	4cdb-A	3.4	3.4	71	488	3	MOLECULE:	LISTERI	OLYSIN	O
96:00:00	5u6y-A	3.4	3.7	69	459	1	MOLECULE:	CALCIUM	/CALMOD	ULIN-DEPENDENT PROTEIN KINASE TYPE
97:00:00	6e5u-A	3.4	3.3	73	339	3	MOLECULE:	NUCLEAR	RNA EX	PORT FACTOR 1
98:00:00	2ags-A	3.4	3.9	67	634	6	MOLECULE:	SIALIDA	SE	
99:00:00	3d9r-B	3.4	3.9	73	134	7	MOLECULE:	KETOSTE	ROID IS	OMERASE-LIKE PROTEIN
0:00	6w3w-A	3.4	3.3	67	107	15	MOLECULE:	DENOVO	NTF2	
1:00	4lmg-B	3.4	2.9	59	121	5	MOLECULE:	IRON-RE	GULATED	TRANSCRIPTIONAL ACTIVATOR AFT2
2:00	7vnx-A	3.4	2.7	58	216	7	MOLECULE:	TKARKI		
3:00	3os7-A	3.4	3.5	76	338	8	MOLECULE:	GALACTO	SE MUTA	ROTASE-LIKE PROTEIN
4:00	4fr9-A	3.4	3.7	66	141	11	MOLECULE:	UNCHARA	CTERIZE	D PROTEIN
5:00	1gyb-A	3.4	3.5	74	122	5	MOLECULE:	NUCLEAR	TRANSP	ORT FACTOR 2
6:00	4n6n-B	3.4	3.4	62	117	3	MOLECULE:	LEGUMAI	N	
7:00	6mrk-V	3.4	3.7	72	126	3	MOLECULE:	NUCLEAR	RNA EX	PORT FACTOR 2
8:00	6d63-B	3.4	3.2	72	128	13	MOLECULE:	ATZH		
9:00	5tpj-A	3.4	3.4	68	122	13	MOLECULE:	DENOVO	NTF2	
10:00	1jn5-A	3.4	2.8	67	136	12	MOLECULE:	P15		
11:00	8v1l-A	3.4	3.8	71	126	8	MOLECULE:	RAS GTP	ASE-ACT	IVATING PROTEIN-BINDING PROTEIN 1
12:00	6d63-I	3.4	3.3	68	123	6	MOLECULE:	ATZH		
13:00	5tph-B	3.4	4.4	71	121	6	MOLECULE:	DE NOVO	NTF2 H	OMODIMER
14:00	6ta7-E	3.4	3.6	72	125	7	MOLECULE:	RAS GTP	ASE-ACT	IVATING PROTEIN-BINDING PROTEIN 1
15:00	6s5l-B	3.4	3	61	124	10	MOLECULE:	ALL4940	PROTEI	N
16:00	4n6o-B	3.4	3.4	62	115	3	MOLECULE:	LEGUMAI	N	
17:00	6xby-s	3.4	3.2	77	205	10	MOLECULE:	V-TYPE	PROTON	ATPASE CATALYTIC SUBUNIT A
18:00	6lye-I	3.4	2.6	57	82	11	MOLECULE:	PROBABL	E URACI	L-DNA GLYCOSYLASE
19:00	6bju-B	3.4	3.2	68	121	7	MOLECULE:	ATZH		
20:00	4hbr-C	3.4	2.9	62	140	2	MOLECULE:	PUTATIV	E PERIP	LASMIC PROTEIN
21:00	6d63-A	3.4	2.9	70	127	6	MOLECULE:	ATZH		
22:00	3njv-A	3.4	3	47	508	6	MOLECULE:	RHAMNOG	ALACTUR	ONASE B
23:00	7toe-D	3.4	4.1	65	467	3	MOLECULE:	GLUCOSE	#NAME?	PHATE 1-DEHYDROGENASE
24:00:00	4j9t-A	3.4	3.2	63	355	16	MOLECULE:	DESIGNE	D UNNAT	URAL AMINO ACID DEPENDENT METALLOPR
25:00:00	7dmp-B	3.4	3.5	71	336	3	MOLECULE:	RADIAL	SPOKE H	EAD PROTEIN 4 HOMOLOG A
26:00:00	1jb2-B	3.4	3.5	70	123	10	MOLECULE:	NUCLEAR	TRANSP	ORT FACTOR 2
27:00:00	1wcq-C	3.4	3.4	66	599	15	MOLECULE:	SIALIDA	SE	
28:00:00	2zhx-F	3.4	2.7	59	82	12	MOLECULE:	URACIL-	DNA GLY	COSYLASE
29:00:00	8feb-A	3.4	3.3	65	501	5	MOLECULE:	SIALIDA	SE	
30:00:00	3njx-A	3.4	3	47	508	6	MOLECULE:	RHAMNOG	ALACTUR	ONASE B
31:00:00	4ttg-D	3.4	3	50	1015	6	MOLECULE:	BETA-GA	LACTOSI	DASE
32:00:00	6d63-G	3.4	3.1	65	115	8	MOLECULE:	ATZH		
33:00:00	1jb4-B	3.4	3.4	70	123	10	MOLECULE:	NUCLEAR	TRANSP	ORT FACTOR 2
34:00:00	1gy5-B	3.4	3.4	70	124	10	MOLECULE:	NUCLEAR	TRANSP	ORT FACTOR 2
35:00:00	4fpk-A	3.4	3.4	60	658	10	MOLECULE:	SIALIDA	SE B	
36:00:00	4dux-C	3.4	3.1	50	1015	6	MOLECULE:	BETA-GA	LACTOSI	DASE
37:00:00	2wof-A	3.4	3	64	718	11	MOLECULE:	PRIMARY	AMINE	OXIDASE
38:00:00	7ual-D	3.4	4.3	69	484	3	MOLECULE:	GLUCOSE	#NAME?	PHATE 1-DEHYDROGENASE
39:00:00	2pu8-A	3.4	2.5	52	373	2	MOLECULE:	METHYLT	HIORIBO	SE KINASE
40:00:00	4orl-A	3.3	3.2	66	110	11	MOLECULE:	UNCHARA	CTERIZE	D PROTEIN
41:00:00	4q51-B	3.3	3.1	64	265	11	MOLECULE:	UNCHARA	CTERIZE	D PROTEIN
42:00:00	8t1l-V	3.3	3.1	66	278	11	MOLECULE:	MEDIATO	R OF RN	A POLYMERASE II TRANSCRIPTION SUBUN
43:00:00	6mrk-B	3.3	3.5	73	188	4	MOLECULE:	NUCLEAR	RNA EX	PORT FACTOR 2
44:00:00	3flj-A	3.3	3.5	71	141	8	MOLECULE:	UNCHARA	CTERIZE	D PROTEIN CONSERVED IN BACTERIA WIT
45:00:00	8frs-A	3.3	3.1	66	317	8	MOLECULE:	MAJOR S	TRUCTUR	AL PROTEIN
46:00:00	4qvh-A	3.3	3.2	69	596	4	MOLECULE:	MALTOSE	#NAME?	G PERIPLASMIC PROTEIN, 4'-PHOSPHOPA

47:00:00	4kt3-B	3.3	4	74	128	8	MOLECULE:	UNCHARA	CTERIZE	D PROTEIN
48:00:00	3h51-A	3.3	3.6	67	142	9	MOLECULE:	PUTATIV	E CALCI	UM/CALMODULIN DEPENDENT PROTEIN KIN
49:00:00	2mc8-A	3.3	2.7	59	114	10	MOLECULE:	UNCHARA	CTERIZE	D PROTEIN
50:00:00	3en1-B	3.3	4.1	69	180	4	MOLECULE:	BENZENE	1,2-DI	OXYGENASE SUBUNIT ALPHA
51:00:00	5k21-B	3.3	3.3	58	123	3	MOLECULE:	PYOCYAN	IN DEME	THYLASE
52:00:00	3f8x-B	3.3	3.1	61	132	10	MOLECULE:	PUTATIV	E DELTA	-5-3-KETOSTEROID ISOMERASE
53:00:00	6jzf-A	3.3	4	68	153	4	MOLECULE:	PLASTID	DIVISI	ON PROTEIN CDP1, CHLOROPLASTIC
54:00:00	6w40-A	3.3	3.7	69	120	13	MOLECULE:	DENOVO	NTF2	
55:00:00	6e0a-B	3.3	3	65	262	5	MOLECULE:	METHYL-	ACCEPTI	NG CHEMOTAXIS PROTEIN TLPA
56:00:00	5efv-B	3.3	3.6	69	635	7	MOLECULE:	PHI ETA	ORF 56	-LIKE PROTEIN
57:00:00	3aki-A	3.3	3.2	68	448	7	MOLECULE:	PUTATIV	E SECRE	TED ALPHA L-ARABINOFURANOSIDASE II
58:00:00	5boi-A	3.3	3.6	67	226	6	MOLECULE:	GERMINA	TION PR	OTEIN YPEB
59:00:00	2du7-A	3.3	2.9	61	539	0	MOLECULE:	O-PHOSP	HOSERYL	-TRNA SYNTHETASE
60:00:00	2r41-C	3.3	2.6	58	106	16	MOLECULE:	UNCHARA	CTERIZE	D PROTEIN
61:00:00	8t5t-A	3.3	4.2	75	195	3	MOLECULE:	OUTER M	EMBRANE	LIPOPROTEIN CARRIER PROTEIN (LOLA)
62:00:00	3u1w-B	3.3	3.2	60	248	5	MOLECULE:	HYPOTHE	TICAL P	ERIPLASMIC PROTEIN
63:00:00	6bju-D	3.3	3.1	68	123	6	MOLECULE:	ATZH		
64:00:00	6d63-L	3.3	3.5	69	121	13	MOLECULE:	ATZH		
65:00:00	7pdz-F	3.3	3.6	73	272	4	MOLECULE:	ISOFORM	2 OF F	#NAME?
66:00:00	1dyu-B	3.3	3.3	67	720	7	MOLECULE:	COPPER	AMINE O	XIDASE
67:00:00	6d63-C	3.3	3.2	68	125	7	MOLECULE:	ATZH		
68:00:00	2wof-B	3.3	3.3	67	720	7	MOLECULE:	PRIMARY	AMINE	OXIDASE
69:00:00	1jrq-B	3.3	3.2	64	721	6	MOLECULE:	COPPER	AMINE O	XIDASE
70:00:00	8v1l-D	3.3	3.2	69	128	7	MOLECULE:	RAS GTP	ASE-ACT	IVATING PROTEIN-BINDING PROTEIN 1
71:00:00	7a0h-B	3.3	3.3	73	255	3	MOLECULE:	F-ACTIN	#NAME?	G PROTEIN SUBUNIT ALPHA
72:00:00	2woh-B	3.3	3.4	65	720	8	MOLECULE:	PRIMARY	AMINE	OXIDASE
73:00:00	4nfa-A	3.3	4.2	63	204	11	MOLECULE:	PROTEIN	CASC5	
74:00:00	7c03-A	3.3	3.1	66	312	5	MOLECULE:	POLARIS	ACT(T57	S)
75:00:00	4eyc-A	3.3	3.3	61	103	5	MOLECULE:	CATHELI	CIDIN A	NTIMICROBIAL PEPTIDE
76:00:00	1d6z-B	3.3	3.4	68	720	6	MOLECULE:	COPPER	AMINE O	XIDASE
77:00:00	2rgq-C	3.3	3.5	65	134	5	MOLECULE:	DOMAIN	OF UNKN	OWN FUNCTION WITH A CYSTATIN-LIKE F
78:00:00	2pul-A	3.3	2.4	54	381	4	MOLECULE:	METHYLT	HIORIBO	SE KINASE
79:00:00	7zhw-B	3.3	4.3	68	535	3	MOLECULE:	GLUCOSE	#NAME?	PHATE 1-DEHYDROGENASE
80:00:00	6drv-D	3.3	2.6	46	1024	4	MOLECULE:	BETA-GA	LACTOSI	DASE
81:00:00	6ezz-B	3.3	3.3	66	720	8	MOLECULE:	PRIMARY	AMINE	OXIDASE
82:00:00	4dux-B	3.3	3.1	50	1015	6	MOLECULE:	BETA-GA	LACTOSI	DASE
83:00:00	2wyi-B	3.3	3.4	71	896	15	MOLECULE:	ALPHA-M	ANNOSID	ASE
84:00:00	7zvd-N	3.3	4.1	64	484	3	MOLECULE:	GLUCOSE	#NAME?	PHATE 1-DEHYDROGENASE
85:00:00	5igi-A	3.3	3.4	59	300	15	MOLECULE:	MACROLI	DE 2'-P	HOSPHOTRANSFERASE
86:00:00	1ms1-A	3.3	3.9	67	622	7	MOLECULE:	TRANS-S	IALIDAS	E
87:00:00	1jb5-B	3.3	3.5	69	123	10	MOLECULE:	NUCLEAR	TRANSP	ORT FACTOR 2
88:00:00	7zhv-A	3.3	5	67	533	3	MOLECULE:	GLUCOSE	#NAME?	PHATE 1-DEHYDROGENASE
89:00:00	3elg-A	3.3	4.3	56	127	5	MOLECULE:	UNCHARA	CTERIZE	D PERIPLASMIC PROTEIN
90:00:00	1jz7-A	3.3	3.2	51	1011	4	MOLECULE:	BETA-GA	LACTOSI	DASE
91:00:00	3due-A	3.3	3.5	54	127	9	MOLECULE:	PUTATIV	E PERIP	LASMIC PROTEIN
92:00:00	1dyu-A	3.3	3	64	718	11	MOLECULE:	COPPER	AMINE O	XIDASE
93:00:00	6d63-K	3.3	3.3	68	120	7	MOLECULE:	ATZH		
94:00:00	1spu-A	3.3	3.2	64	718	11	MOLECULE:	COPPER	AMINE O	XIDASE
95:00:00	4lv8-A	3.2	3.4	66	360	11	MOLECULE:	RHOPTRY	PROTEI	N 5 C
96:00:00	3dmc-A	3.2	3.7	71	134	11	MOLECULE:	NTF2-LI	KE PROT	EIN
97:00:00	5hx0-A	3.2	3.7	60	365	5	MOLECULE:	UNCHARA	CTERIZE	D PROTEIN DFER_1899
98:00:00	2iut-A	3.2	3.5	59	408	8	MOLECULE:	DNA TRA	NSLOCAS	E FTSK
99:00:00	6gvw-C	3.2	3.7	69	383	4	MOLECULE:	BRCA1-A	COMPLE	X SUBUNIT ABRAXAS 1
0:00	6qbe-A	3.2	3.2	74	215	9	MOLECULE:	NEP1-LI	KE PROT	EIN

1:00	6d8v-A	3.2	2.8	60	270	7	MOLECULE:	PROBABL	E CHEMO	RECEPTOR (METHYL-ACCEPTING CHEMOTAX
2:00	8u50-A	3.2	3.1	51	267	12	MOLECULE:	KLEBSIE	LLA PNE	UMONIAE FAMILY 1 ENCAPSULIN SHELL
3:00	7n34-A	3.2	3.1	64	108	2	MOLECULE:	CAP15		
4:00	2d7d-A	3.2	2.8	58	621	12	MOLECULE:	5'-D(P*	TP*TP*T	)-3'
5:00	2h1t-A	3.2	3.9	54	186	7	MOLECULE:	HYPOTHE	TICAL P	ROTEIN
6:00	6y2p-A	3.2	2.7	54	354	9	MOLECULE:	MRNA EN	DORIBON	UCLEASE TOXIN LS
7:00	7f13-A	3.2	3.6	71	154	6	MOLECULE:	DCR3		
8:00	4epa-A	3.2	3.3	69	632	3	MOLECULE:	PESTICI	N RECEP	TOR
9:00	5hal-A	3.2	3.4	65	103	9	MOLECULE:	UNCHARA	CTERIZE	D PROTEIN
10:00	1sa8-A	3.2	3.5	60	106	8	MOLECULE:	FATTY A	CID-BIN	DING PROTEIN, INTESTINAL
11:00	4mjd-A	3.2	3.8	66	113	12	MOLECULE:	KETOSTE	ROID IS	OMERASE FOLD PROTEIN HMUK_0747
12:00	6s50-A	3.2	3.7	53	248	4	MOLECULE:	STREPTA	VIDIN	
13:00	7fhr-A	3.2	3.5	74	439	5	MOLECULE:	PUTATIV	E PHTHA	LATE 4,5-DIOXYGENASE, SUBUNIT ALPHA
14:00	7woo-D	3.2	3.2	59	1398	3	MOLECULE:	NUCLEOP	ORIN NI	C96
15:00	1jki-A	3.2	4.1	59	525	10	MOLECULE:	MYO-INO	SITOL-1	-PHOSPHATE SYNTHASE
16:00	8dml-B	3.2	2.9	54	133	2	MOLECULE:	VTRA		
17:00	7yls-A	3.2	3.5	67	158	4	MOLECULE:	AROMATI	C-RING-	HYDROXYLATING DIOXYGENASE BETA SUBU
18:00	7z6e-C	3.2	2.9	55	534	11	MOLECULE:	SERINE/	THREONI	NE-PROTEIN KINASE MRCK-1
19:00	6whp-A	3.2	3.3	70	428	9	MOLECULE:	CHOLINE	KINASE	
20:00	6fhm-A	3.2	3.7	59	193	5	MOLECULE:	OUTER-M	EMBRANE	LIPOPROTEIN CARRIER PROTEIN
21:00	7agv-F	3.2	2.6	51	163	6	MOLECULE:	K(+)/H(	+) ANTI	PORTER SUBUNIT KHTT
22:00	8gt0-B	3.2	3.5	71	98	4	MOLECULE:	CYSTEIN	E PROTE	ASE FALCIPAIN-2
23:00	1gyb-D	3.2	3.2	66	120	9	MOLECULE:	NUCLEAR	TRANSP	ORT FACTOR 2
24:00:00	1gy7-D	3.2	3.4	68	120	9	MOLECULE:	NUCLEAR	TRANSP	ORT FACTOR 2
25:00:00	5drv-A	3.2	3.7	72	131	7	MOLECULE:	RAS GTP	ASE-ACT	IVATING PROTEIN-BINDING PROTEIN 2
26:00:00	8th5-D	3.2	3.7	71	129	7	MOLECULE:	RAS GTP	ASE-ACT	IVATING PROTEIN-BINDING PROTEIN 1
27:00:00	7xhf-A	3.2	3.4	69	124	6	MOLECULE:	RAS GTP	ASE-ACT	IVATING PROTEIN-BINDING PROTEIN 1
28:00:00	3fsd-A	3.2	3.2	65	121	5	MOLECULE:	NTF2-LI	KE PROT	EIN OF UNKNOWN FUNCTION IN NUTRIENT
29:00:00	4nf9-A	3.2	4.3	65	216	11	MOLECULE:	PROTEIN	CASC5	
30:00:00	1izn-A	3.2	3.6	71	275	7	MOLECULE:	CAPZ AL	PHA-1 S	UBUNIT
31:00:00	6d63-E	3.2	3.7	68	119	7	MOLECULE:	ATZH		
32:00:00	4ipb-B	3.2	3	63	141	3	MOLECULE:	UNCHARA	CTERIZE	D PROTEIN
33:00:00	6d63-J	3.2	3.3	68	129	7	MOLECULE:	ATZH		
34:00:00	1gyb-C	3.2	3.3	68	120	9	MOLECULE:	NUCLEAR	TRANSP	ORT FACTOR 2
35:00:00	7qz3-B	3.2	2.5	54	519	6	MOLECULE:	BNR/ASP	#NAME?	PEAT PROTEIN
36:00:00	4i4k-B	3.2	3	70	131	10	MOLECULE:	UNCHARA	CTERIZE	D PROTEIN SGCJ
37:00:00	2w0q-A	3.2	3.3	66	718	8	MOLECULE:	COPPER	AMINE O	XIDASE
38:00:00	1d6u-A	3.2	3.1	65	718	8	MOLECULE:	COPPER	AMINE O	XIDASE
39:00:00	3u1w-A	3.2	3.4	62	249	8	MOLECULE:	HYPOTHE	TICAL P	ERIPLASMIC PROTEIN
40:00:00	6d63-D	3.2	3.1	66	115	8	MOLECULE:	ATZH		
41:00:00	6s6z-G	3.2	2.7	49	1083	10	MOLECULE:	BETA-GA	LACTOSI	DASE
42:00:00	1ms4-A	3.2	3.2	62	623	6	MOLECULE:	TRANS-S	IALIDAS	E
43:00:00	1q42-A	3.2	3.5	71	159	13	MOLECULE:	MRNA TR	ANSPORT	REGULATOR MTR2
44:00:00	1a90-A	3.2	3.3	62	107	5	MOLECULE:	CYSTATI	N	
45:00:00	7jr9-C	3.2	4.3	69	280	4	MOLECULE:	RADIAL	SPOKE P	ROTEIN 9
46:00:00	8agy-A	3.1	3	55	346	7	MOLECULE:	CORRAMY	CIN PHO	SPHOTRANSFERASE
47:00:00	6szw-C	3.1	2.8	53	186	8	MOLECULE:	COMPLEM	ENT COM	PONENT 1 Q SUBCOMPONENT-BINDING PRO
48:00:00	5u4z-A	3.1	3.1	73	181	5	MOLECULE:	REPRESS	OR OF R	NA POLYMERASE III TRANSCRIPTION
49:00:00	4e72-A	3.1	3.6	70	218	4	MOLECULE:	UNCHARA	CTERIZE	D PROTEIN
50:00:00	1vr7-A	3.1	3.4	62	120	0	MOLECULE:	S-ADENO	SYLMETH	IONINE DECARBOXYLASE PROENZYME
51:00:00	2p4o-A	3.1	3.1	61	302	13	MOLECULE:	HYPOTHE	TICAL P	ROTEIN
52:00:00	3ats-A	3.1	3.4	62	352	5	MOLECULE:	PUTATIV	E UNCHA	RACTERIZED PROTEIN
53:00:00	3k1l-A	3.1	3.8	61	376	5	MOLECULE:	FANCL		
54:00:00	6iqt-A	3.1	3.3	64	135	9	MOLECULE:	CAG PAT	HOGENIC	ITY ISLAND PROTEIN (CAG10)

55:00:00	6sy9-A	3.1	4.1	61	307	10	MOLECULE:	RESPONS	E REGUL	ATOR		
56:00:00	3lid-A	3.1	3.2	62	279	8	MOLECULE:	PUTATIV	E SENSO	RY BOX/GGDEF FAMILY PROTEIN		
57:00:00	5yvf-A	3.1	3.9	71	322	8	MOLECULE:	BFA1				
58:00:00	3elz-B	3.1	3.9	54	135	7	MOLECULE:	ILEAL B	ILE ACI	D-BINDING PROTEIN		
59:00:00	6vxk-B	3.1	3.8	73	747	5	MOLECULE:	SEMAPHO	RIN-LIK	E PROTEIN 139		
60:00:00	1wzn-A	3.1	5.9	49	245	6	MOLECULE:	SAM-DEP	ENDENT	METHYLTRANSFERASE		
61:00:00	1sgo-A	3.1	4	69	139	7	MOLECULE:	PROTEIN	C14ORF			129
62:00:00	3cwy-A	3.1	3.6	66	130	9	MOLECULE:	PROTEIN	CAGD			
63:00:00	6ck1-A	3.1	3	59	375	7	MOLECULE:	A1B2F4	PROTEIN			
64:00:00	7vud-A	3.1	3.5	59	535	7	MOLECULE:	CAROTEN	OID CLE	AVAGE DIOXYGENASE 1		
65:00:00	7vwk-A	3.1	2.9	54	260	6	MOLECULE:	POLYKET	IDE SYN	THASE		
66:00:00	6h3i-A	3.1	4.5	70	2124	4	MOLECULE:	PROTEIN	INVOLV	ED IN GLIDING MOTILITY SPRA		
67:00:00	2h36-X	3.1	3	55	108	4	MOLECULE:	HYPOTHE	TICAL P	ROTEIN SIFV0014		
68:00:00	3eps-A	3.1	4.5	79	566	3	MOLECULE:	ISOCITR	ATE DEH	YDROGENASE KINASE/PHOSPHATASE		
69:00:00	6zce-p	3.1	3.8	67	646	4	MOLECULE:	EUKARYO	TIC TRA	NSLATION INITIATION FACTOR 3 SUBUNI		
70:00:00	3ff2-A	3.1	4.1	65	117	8	MOLECULE:	UNCHARA	CTERIZE	D CYSTATIN FOLD PROTEIN (YP_497570.		
71:00:00	4pqg-A	3.1	2.7	54	506	11	MOLECULE:	GLYCOSY	LTRANSF	ERASE GTF1		
72:00:00	8ovw-N	3.1	3.7	69	391	4	MOLECULE:	CENTROM	ERE-BIN	DING PROTEIN 1		
73:00:00	7qh3-B	3.1	4.5	63	148	5	MOLECULE:	RSFG				
74:00:00	4tqj-A	3.1	3.5	66	403	5	MOLECULE:	LECTIN	2			
75:00:00	8wxf-A	3.1	3.9	54	730	13	MOLECULE:	BIFUNCT	IONAL G	UANOSINE PENTAPHOSPHATE		
76:00:00	3nqh-A	3.1	4.3	57	439	4	MOLECULE:	GLYCOSY	L HYDRO	LASE		
77:00:00	3s25-A	3.1	3.6	58	295	14	MOLECULE:	HYPOTHE	TICAL 7		#NAME?	
78:00:00	2wgo-A	3.1	4	74	98	4	MOLECULE:	RANASPU	MIN-2			
79:00:00	7jrj-C	3.1	4.3	72	359	10	MOLECULE:	RADIAL	SPOKE P	ROTEIN 10		
80:00:00	8th1-D	3.1	3.5	69	137	6	MOLECULE:	RAS GTP	ASE-ACT	IVATING PROTEIN-BINDING PROTEIN 1		
81:00:00	4fcj-A	3.1	3.2	66	128	6	MOLECULE:	RAS GTP	ASE-ACT	IVATING PROTEIN-BINDING PROTEIN 1		
82:00:00	8th5-A	3.1	3.7	71	123	6	MOLECULE:	RAS GTP	ASE-ACT	IVATING PROTEIN-BINDING PROTEIN 1		
83:00:00	7suo-B	3.1	3.8	72	137	7	MOLECULE:	RAS GTP	ASE-ACT	IVATING PROTEIN-BINDING PROTEIN 1		
84:00:00	1zo2-A	3.1	3.2	67	124	10	MOLECULE:	NUCLEAR	TRANSP	ORT FACTOR 2		
85:00:00	8x2u-G	3.1	4.2	70	387	3	MOLECULE:	DPY30 D	OMAIN C	ONTAINING 2		
86:00:00	8th1-A	3.1	3.4	68	137	6	MOLECULE:	RAS GTP	ASE-ACT	IVATING PROTEIN-BINDING PROTEIN 1		
87:00:00	2pup-B	3.1	2.5	54	370	4	MOLECULE:	METHYLT	HIORIBO	SE KINASE		
88:00:00	6f38-K	3.1	3.4	73	278	4	MOLECULE:	ARP1 AC	TIN REL	ATED PROTEIN 1 HOMOLOG A		
89:00:00	6fej-A	3.1	3.3	68	124	10	MOLECULE:	ALL4940	PROTEI	N		
90:00:00	4nf9-B	3.1	4.2	65	207	11	MOLECULE:	PROTEIN	CASC5			
91:00:00	3ob8-D	3.1	4.3	50	1024	8	MOLECULE:	BETA-GA	LACTOSI	DASE		
92:00:00	4xj9-A	3.1	3.4	60	658	7	MOLECULE:	SIALIDA	SE B			
93:00:00	4ovm-C	3.1	3.1	71	129	10	MOLECULE:	UNCHARA	CTERIZE	D PROTEIN SGCJ		
94:00:00	2bzd-B	3.1	3.1	64	601	16	MOLECULE:	BACTERI	AL SIAL	IDASE		
95:00:00	5fw5-B	3.1	3.2	67	131	6	MOLECULE:	RAS GTP	ASE-ACT	IVATING PROTEIN-BINDING PROTEIN 1		
96:00:00	3wz5-C	3.1	3.9	70	136	7	MOLECULE:	DOTI				
97:00:00	1w0p-A	3.1	3.3	73	753	7	MOLECULE:	SIALIDA	SE			
98:00:00	7zhz-B	3.1	4.6	70	527	3	MOLECULE:	GLUCOSE	#NAME?	PHATE 1-DEHYDROGENASE		
99:00:00	7w19-A	3.1	2.5	55	293	5	MOLECULE:	MACROLI	DE 2'-P	HOSPHOTRANSFERASE		
0:00	1d6z-A	3.1	3.1	65	718	8	MOLECULE:	COPPER	AMINE O	XIDASE		
1:00	3ujm-A	3.1	3.1	64	117	5	MOLECULE:	RASPUTI	N			
2:00	6s6z-F	3.1	3.4	74	1083	4	MOLECULE:	BETA-GA	LACTOSI	DASE		
3:00	4fp3-A	3.1	2.9	57	658	2	MOLECULE:	SIALIDA	SE B			
4:00	4dux-D	3.1	3	50	1015	6	MOLECULE:	BETA-GA	LACTOSI	DASE		
5:00	3lk3-A	3.1	3.3	68	266	6	MOLECULE:	F-ACTIN	#NAME?	G PROTEIN SUBUNIT ALPHA-1		
6:00	2ber-A	3.1	3.3	65	601	15	MOLECULE:	BACTERI	AL SIAL	IDASE		
7:00	6s6z-H	3.1	2.7	50	1083	10	MOLECULE:	BETA-GA	LACTOSI	DASE		
8:00	3aa1-A	3.1	3.5	72	271	7	MOLECULE:	F-ACTIN	#NAME?	G PROTEIN SUBUNIT ALPHA-1		

9:00	3dxq-B	3.1	2.5	49	292	6	MOLECULE:	CHOLINE	/ETHANO	LAMINE KINASE FAMILY PROTEIN
10:00	1jz7-C	3.1	3.1	51	1011	4	MOLECULE:	BETA-GA	LACTOSI	DASE
11:00	6m1u-A	3	3.5	63	122	3	MOLECULE:	RNA N6-	ADENOSI	NE-METHYLTRANSFERASE METTL16,RNA N6
12:00	5wlz-C	3	2.8	52	206	12	MOLECULE:	DNA REP	AIR PRO	TEIN XRCC4,MYOSIN-7
13:00	8e1m-C	3	3.5	69	264	6	MOLECULE:	MITOCHO	NDRIAL	IMPORT INNER MEMBRANE TRANSLOCASE S
14:00	4zsv-A	3	3.6	60	294	5	MOLECULE:	UNCHARA	CTERIZE	D PROTEIN
15:00	8ju7-A	3	3.8	60	104	8	MOLECULE:	HISTIDI	NE KINA	SE
16:00	2chc-A	3	3.5	70	169	4	MOLECULE:	PROTEIN	RV3472	
17:00	3duk-A	3	3.4	70	125	9	MOLECULE:	NTF2-LI	KE PROT	EIN OF UNKNOWN FUNCTION
18:00	2glz-A	3	3.8	70	149	3	MOLECULE:	SIMILAR	TO FOR	MYLMETHANOFURAN DEHYDROGENASE SUBUN
19:00	8pjn-2	3	3.8	64	156	6	MOLECULE:	E3 UBIQ	UITIN-P	ROTEIN TRANSFERASE RMND5A
20:00	4pj2-B	3	2.4	61	123	10	MOLECULE:	PUTATIV	E EXPOR	TED PROTEIN
21:00	2rfr-A	3	3.4	68	154	7	MOLECULE:	UNCHARA	CTERIZE	D PROTEIN
22:00	5fw5-A	3	3.6	70	139	6	MOLECULE:	RAS GTP	ASE-ACT	IVATING PROTEIN-BINDING PROTEIN 1
23:00	4zr7-D	3	2.5	56	120	16	MOLECULE:	SENSOR	HISTIDI	NE KINASE RESE
24:00:00	6x1i-B	3	3.6	72	146	7	MOLECULE:	COB_ADE	NO_TRAN	S DOMAIN-CONTAINING PROTEIN PH0671
25:00:00	3lml-A	3	4.1	68	440	4	MOLECULE:	LIN1278	PROTEI	N
26:00:00	3soy-A	3	3.2	67	142	13	MOLECULE:	NTF2-LI	KE SUPE	RFAMILY PROTEIN
27:00:00	6mni-A	3	2.7	59	259	7	MOLECULE:	METHYL-	ACCEPTI	NG CHEMOTAXIS PROTEIN
28:00:00	5d16-A	3	3.3	66	161	8	MOLECULE:	TRANSPO	SON TN7	TRANSPOSITION PROTEIN TNSE
29:00:00	3k7c-A	3	3.5	62	108	6	MOLECULE:	PUTATIV	E NTF2-	LIKE TRANSPEPTIDASE
30:00:00	2f98-C	3	3.3	70	143	7	MOLECULE:	AKLANON	IC ACID	METHYL ESTER CYCLASE, AKNH
31:00:00	4lmi-B	3	3.7	71	136	10	MOLECULE:	UNCHARA	CTERIZE	D PROTEIN
32:00:00	3h6j-A	3	3.3	64	438	8	MOLECULE:	NEURAMI	NIDASE	
33:00:00	3i2n-A	3	3.8	67	343	7	MOLECULE:	WD REPE	AT-CONT	AINING PROTEIN 92
34:00:00	2gxf-A	3	2.9	60	119	12	MOLECULE:	HYPOTHE	TICAL P	ROTEIN YYBH
35:00:00	6nd4-H	3	3.9	59	834	5	MOLECULE:	ETS RRN	A	
36:00:00	5xq3-A	3	2.8	52	901	8	MOLECULE:	PCRGLX	PROTEIN	
37:00:00	3g0k-A	3	3.6	69	129	9	MOLECULE:	PUTATIV	E MEMBR	ANE PROTEIN
38:00:00	7fds-B	3	3.7	54	118	4	MOLECULE:	LIPOPRO	TEIN LP	QH
39:00:00	2lqv-A	3	3.5	64	99	9	MOLECULE:	PROTEIN	YEBF	
40:00:00	6p2k-B	3	3.9	73	778	7	MOLECULE:	FIBRONE	CTIN TY	PE III DOMAIN-CONTAINING PROTEIN
41:00:00	8shj-A	3	3.6	64	334	13	MOLECULE:	WD REPE	AT-CONT	AINING PROTEIN 91
42:00:00	1pre-B	3	3.7	75	451	7	MOLECULE:	PROAERO	LYSIN	
43:00:00	6qvi-A	3	6.2	57	518	7	MOLECULE:	COMZ		
44:00:00	1l0q-A	3	3.4	60	391	12	MOLECULE:	SURFACE	LAYER	PROTEIN
45:00:00	6ht9-B	3	3.9	67	316	4	MOLECULE:	ASTACIN		
46:00:00	7pge-B	3	4	60	198	13	MOLECULE:	COPPER	RESISTA	NCE PROTEIN B
47:00:00	3fh1-A	3	4.1	70	122	7	MOLECULE:	UNCHARA	CTERIZE	D NTF2-LIKE PROTEIN
48:00:00	7aqc-R	3	3.9	71	558	4	MOLECULE:	23S RIB	OSOMAL	RNA
49:00:00	6igb-A	3	3.5	67	363	9	MOLECULE:	PERIPLA	SMIC GL	UCONOLACTONASE, PPGL
50:00:00	2ch9-A	3	4.4	68	126	10	MOLECULE:	CYSTATI	N F	
51:00:00	6b62-A	3	3.5	64	256	6	MOLECULE:	FRUCTOS	E-1,6-B	ISPHOSPHATASE/INOSITOL-1-MONOPHOSPH
52:00:00	3f9s-A	3	3.5	70	143	10	MOLECULE:	PUTATIV	E POLYK	ETIDE CYCLASE
53:00:00	6jp6-A	3	3.3	66	998	5	MOLECULE:	TRNA (G	UANOSIN	E(34)-2'-O)-METHYLTRANSFERASE NON-C
54:00:00	4agi-A	3	2.7	46	314	9	MOLECULE:	FUCOSE-	SPECIFI	C LECTIN FLEA
55:00:00	6z37-A	3	7.1	69	418	1	MOLECULE:	TODX		
56:00:00	7kbj-I	3	3.1	61	153	5	MOLECULE:	NEUTRAL	ALPHA-	GLUCOSIDASE AB TRYPSIN-CLEAVED FRAG
57:00:00	6iy9-A	3	2.7	58	325	7	MOLECULE:	HYGROMY	CIN-B 7	'-O-KINASE
58:00:00	6mso-A	3	3	63	540	3	MOLECULE:	FUMARAT	E HYDRA	TASE
59:00:00	7c5x-A	3	3.6	71	506	1	MOLECULE:	IOTA-CA	RBONIC	ANHYDRASE
60:00:00	6mly-C	3	3.7	71	774	10	MOLECULE:	BIFUNCT	IONAL G	H43-CE PROTEIN
61:00:00	8veh-C	3	3.5	73	194	4	MOLECULE:	OUTER M	EMBRANE	LIPOPROTEIN-SORTING PROTEIN LOLA
62:00:00	1q40-A	3	3.6	71	165	11	MOLECULE:	MRNA TR	ANSPORT	REGULATOR MTR2

63:00:00	3lyg-A	3	3.2	64	120	13	MOLECULE:	NTF2-LI	KE PROT	EIN OF UNKNOWN FUNCTION
64:00:00	3ejv-A	3	3.5	69	159	4	MOLECULE:	UNCHARA	CTERIZE	D PROTEIN WITH CYSTATIN-LIKE FOLD
65:00:00	4u13-A	3	3.1	63	109	10	MOLECULE:	PUTATIV	E POLYK	ETIDE CYCLASE SMA1630
66:00:00	4ghb-B	3	3	53	257	2	MOLECULE:	HYPOTHE	TICAL P	ROTEIN
67:00:00	8th7-B	3	3.9	70	139	6	MOLECULE:	RAS GTP	ASE-ACT	IVATING PROTEIN-BINDING PROTEIN 1
68:00:00	4xju-A	3	3.2	63	658	2	MOLECULE:	SIALIDA	SE B	
69:00:00	6f3a-K	3	3.4	74	278	3	MOLECULE:	ARP1 AC	TIN REL	ATED PROTEIN 1 HOMOLOG A
70:00:00	4akr-C	3	3.2	75	271	4	MOLECULE:	F-ACTIN	#NAME?	G PROTEIN SUBUNIT ALPHA
71:00:00	7suo-A	3	3.7	71	136	6	MOLECULE:	RAS GTP	ASE-ACT	IVATING PROTEIN-BINDING PROTEIN 1
72:00:00	3sil-A	3	3.1	63	379	5	MOLECULE:	SIALIDA	SE	
73:00:00	7yth-B	3	3.5	70	315	10	MOLECULE:	KETOSTE	ROID IS	OMERASE-RELATED PROTEIN
74:00:00	4fcm-B	3	3.8	67	135	6	MOLECULE:	RAS GTP	ASE-ACT	IVATING PROTEIN-BINDING PROTEIN 1
75:00:00	4xma-A	3	3.3	63	658	2	MOLECULE:	SIALIDA	SE B	
76:00:00	8th6-B	3	3.9	71	139	8	MOLECULE:	RAS GTP	ASE-ACT	IVATING PROTEIN-BINDING PROTEIN 1
77:00:00	8th6-A	3	3.5	72	138	7	MOLECULE:	RAS GTP	ASE-ACT	IVATING PROTEIN-BINDING PROTEIN 1
78:00:00	6ta7-A	3	3.9	71	131	7	MOLECULE:	RAS GTP	ASE-ACT	IVATING PROTEIN-BINDING PROTEIN 1
79:00:00	3q90-B	3	3.5	68	130	6	MOLECULE:	RAS GTP	ASE-ACT	IVATING PROTEIN-BINDING PROTEIN 1
80:00:00	1qaf-B	3	3.4	65	721	8	MOLECULE:	PROTEIN	(COPPE	R AMINE OXIDASE)
81:00:00	7xhg-C	3	3.6	70	126	6	MOLECULE:	RAS GTP	ASE-ACT	IVATING PROTEIN-BINDING PROTEIN 1
82:00:00	7vr6-A	3	3.2	67	185	0	MOLECULE:	INTERME	MBRANE	PHOSPHOLIPID TRANSPORT SYSTEM BINDI
83:00:00	1zo2-B	3	3.5	68	124	15	MOLECULE:	NUCLEAR	TRANSP	ORT FACTOR 2
84:00:00	1mr5-A	3	3.1	59	621	7	MOLECULE:	TRANS-S	IALIDAS	E
85:00:00	8v1l-C	3	3.3	68	127	6	MOLECULE:	RAS GTP	ASE-ACT	IVATING PROTEIN-BINDING PROTEIN 1
86:00:00	3ujm-B	3	3	62	117	5	MOLECULE:	RASPUTI	N	
87:00:00	7uc2-C	3	4.4	70	475	3	MOLECULE:	GLUCOSE	#NAME?	PHATE 1-DEHYDROGENASE
88:00:00	8th1-C	3	3.9	70	133	7	MOLECULE:	RAS GTP	ASE-ACT	IVATING PROTEIN-BINDING PROTEIN 1
89:00:00	6s6z-C	3	2.7	50	1083	10	MOLECULE:	BETA-GA	LACTOSI	DASE
90:00:00	6d63-H	3	3.1	66	121	8	MOLECULE:	ATZH		
91:00:00	2ya7-B	3	3.7	64	468	9	MOLECULE:	NEURAMI	NIDASE	A
92:00:00	1oac-A	3	3.5	69	720	10	MOLECULE:	COPPER	AMINE O	XIDASE
93:00:00	1gy7-C	3	3.2	62	119	6	MOLECULE:	NUCLEAR	TRANSP	ORT FACTOR 2
94:00:00	1gy7-A	3	3.3	63	121	8	MOLECULE:	NUCLEAR	TRANSP	ORT FACTOR 2
95:00:00	1ms3-B	3	3.1	62	623	6	MOLECULE:	TRANS-S	IALIDAS	E
96:00:00	4xjz-A	3	3.3	63	658	2	MOLECULE:	SIALIDA	SE B	
97:00:00	6d63-F	3	3.2	68	126	6	MOLECULE:	ATZH		
98:00:00	7zhw-A	3	4.4	67	534	3	MOLECULE:	GLUCOSE	#NAME?	PHATE 1-DEHYDROGENASE
99:00:00	7xhg-A	3	3.2	67	133	4	MOLECULE:	RAS GTP	ASE-ACT	IVATING PROTEIN-BINDING PROTEIN 1
0:00	8th5-C	3	3.5	69	125	6	MOLECULE:	RAS GTP	ASE-ACT	IVATING PROTEIN-BINDING PROTEIN 1
1:00	1q40-C	3	3.6	71	164	11	MOLECULE:	MRNA TR	ANSPORT	REGULATOR MTR2
2:00	1ms1-B	3	3.7	61	622	7	MOLECULE:	TRANS-S	IALIDAS	E
3:00	4ovm-A	3	3.1	69	128	9	MOLECULE:	UNCHARA	CTERIZE	D PROTEIN SGCJ
4:00	4bbw-A	3	3	59	520	5	MOLECULE:	SIALIDA	SE (NEU	RAMINIDASE)
5:00	6ta7-C	3	3.1	67	128	6	MOLECULE:	RAS GTP	ASE-ACT	IVATING PROTEIN-BINDING PROTEIN 1
6:00	8t26-A	3	3.3	65	504	5	MOLECULE:	SIALIDA	SE, PUT	ATIVE
7:00	2jkb-A	3	3.4	60	661	8	MOLECULE:	SIALIDA	SE B	
8:00	6s6z-D	3	2.7	50	1083	10	MOLECULE:	BETA-GA	LACTOSI	DASE
9:00	6baq-F	2.9	2.8	72	211	10	MOLECULE:	BPI FOL	D-CONTA	INING FAMILY A MEMBER 1
10:00	5d06-A	2.9	3.7	71	1526	3	MOLECULE:	UNCHARA	CTERIZE	D PROTEIN
11:00	8rtd-S	2.9	3.8	70	223	10	MOLECULE:	TRWJ PR	OTEIN	
12:00	3obu-A	2.9	3.6	68	142	9	MOLECULE:	TUMOR S	USCEPTI	BILITY GENE 101 PROTEIN
13:00	3kkg-A	2.9	4.2	72	144	10	MOLECULE:	PUTATIV	E SNOAL	-LIKE POLYKETIDE CYCLASE
14:00	3bb9-B	2.9	3.3	64	125	8	MOLECULE:	PUTATIV	E ORPHA	N PROTEIN
15:00	3e29-B	2.9	3.8	69	135	6	MOLECULE:	UNCHARA	CTERIZE	D PROTEIN Q7WE92_BORBR
16:00	1s5a-B	2.9	3.5	65	143	5	MOLECULE:	HYPOTHE	TICAL P	ROTEIN YESE

17:00	1tu1-A	2.9	4.3	67	144	6	MOLECULE:	HYPOTHE	TICAL P	ROTEIN PA0094
18:00	8a9b-B	2.9	4	78	809	1	MOLECULE:	LIPID B	INDING	PROTEIN P116 (MPN213)
19:00	8jd7-A	2.9	4.1	74	510	4	MOLECULE:	ENDO-1,	3-FUCAN	ASE
20:00	3k5j-A	2.9	3.8	70	182	13	MOLECULE:	SUPPRES	SOR OF	FUSED FAMILY PROTEIN
21:00	4ywz-A	2.9	3	61	147	7	MOLECULE:	SENSOR	PROTEIN	KINASE WALK
22:00	4wp5-A	2.9	3.2	73	199	4	MOLECULE:	MRNA EX	PORT PR	OTEIN
23:00	2k3d-A	2.9	3.1	57	87	7	MOLECULE:	LIN0334	PROTEI	N
24:00:00	7zei-A	2.9	3.4	59	310	8	MOLECULE:	CH_GAF1	59A	
25:00:00	4gl6-B	2.9	3.5	59	241	2	MOLECULE:	HYPOTHE	TICAL P	ROTEIN
26:00:00	1mdc-A	2.9	3.6	60	131	5	MOLECULE:	INSECT	FATTY A	CID BINDING PROTEIN
27:00:00	2w5n-A	2.9	3.9	67	365	12	MOLECULE:	ALPHA-L	#NAME?	OFURANOSIDASE
28:00:00	3ksp-A	2.9	3.6	68	129	9	MOLECULE:	CALCIUM	/CALMOD	ULIN-DEPENDENT KINASE II ASSOCIATIO
29:00:00	5jk2-A	2.9	3.3	71	128	6	MOLECULE:	TP0751		
30:00:00	4k17-A	2.9	3.3	55	660	9	MOLECULE:	LEUCINE	-RICH R	EPEAT-CONTAINING PROTEIN 16A
31:00:00	8hpo-K	2.9	3.2	55	413	15	MOLECULE:	TRANSCR	IPTIONA	L REGULATORY PROTEIN UME1
32:00:00	1jp4-A	2.9	5.3	72	302	3	MOLECULE:	3'(2'),	5'-BISP	HOSPHATE NUCLEOTIDASE
33:00:00	5uac-C	2.9	6.2	51	1342	4	MOLECULE:	DNA-DIR	ECTED R	NA POLYMERASE SUBUNIT ALPHA
34:00:00	8ain-B	2.9	3.2	59	107	7	MOLECULE:	URACIL-	DNA GLY	COSYLASE
35:00:00	6n8s-A	2.9	3.5	72	834	4	MOLECULE:	LETHAL(	2) GIAN	T LARVAE PROTEIN HOMOLOG 2
36:00:00	5chs-B	2.9	3.4	54	315	7	MOLECULE:	RNA-DIR	ECTED R	NA POLYMERASE L
37:00:00	7e85-A	2.9	2.9	63	114	8	MOLECULE:	SNOAL-L	IKE DOM	AIN-CONTAINING PROTEIN
38:00:00	6pon-A	2.9	4	71	255	1	MOLECULE:	ADHEREN	CE AND	VIRULENCE PROTEIN A
39:00:00	3eu7-A	2.9	3.1	56	313	5	MOLECULE:	PARTNER	AND LO	CALIZER OF BRCA2
40:00:00	8of7-A	2.9	4.8	61	143	5	MOLECULE:	RHS FAM	ILY PRO	TEIN
41:00:00	2yzy-A	2.9	3.5	59	163	8	MOLECULE:	PUTATIV	E UNCHA	RACTERIZED PROTEIN TTHA1012
42:00:00	2ppq-A	2.9	2.7	53	310	6	MOLECULE:	HOMOSER	INE KIN	ASE
43:00:00	4pr7-A	2.9	3.5	52	171	12	MOLECULE:	OLIGOGA	LACTURO	NATE-SPECIFIC PORIN KDGM
44:00:00	4gfc-A	2.9	3	55	165	4	MOLECULE:	SPINDLE	ASSEMB	LY ABNORMAL PROTEIN 6
45:00:00	7r2x-A	2.9	4.2	69	762	6	MOLECULE:	MANNURC	NIC ACI	D SPECIFIC LYASE
46:00:00	7apk-F	2.9	3.3	58	337	16	MOLECULE:	THO COM	PLEX SU	BUNIT 1
47:00:00	5hax-A	2.9	3.3	60	698	10	MOLECULE:	NUCLEOP	ORIN NU	P170
48:00:00	6vk3-B	2.9	3.6	67	350	9	MOLECULE:	HRD3		
49:00:00	6sg8-B	2.9	3.2	61	381	7	MOLECULE:	HEMAGGL	UTININ-	NEURAMINIDASE
50:00:00	7jj9-A	2.9	3.3	72	461	6	MOLECULE:	ZINC-BI	NDING L	IPOPROTEIN ADCA
51:00:00	3i0y-A	2.9	4.1	67	138	9	MOLECULE:	PUTATIV	E POLYK	ETIDE CYCLASE
52:00:00	3msw-A	2.9	3.8	61	139	5	MOLECULE:	UNCHARA	CTERIZE	D PROTEIN
53:00:00	4gb5-A	2.9	3.2	63	148	10	MOLECULE:	UNCHARA	CTERIZE	D PROTEIN
54:00:00	8cli-B	2.9	3.1	65	689	0	MOLECULE:	GENERAL	TRANSC	RIPTION FACTOR 3C POLYPEPTIDE 1
55:00:00	5yx4-A	2.9	3.8	70	232	9	MOLECULE:	CHALCON	E-FLAVO	NONE ISOMERASE FAMILY PROTEIN
56:00:00	4keg-A	2.9	3	71	535	10	MOLECULE:	MALTOSE	#NAME?	G PERIPLASMIC/PALATE LUNG AND NASAL
57:00:00	1qak-B	2.9	3.4	64	722	8	MOLECULE:	COPPER	AMINE O	XIDASE
58:00:00	8qx7-A	2.9	3	62	124	10	MOLECULE:	ALL4940	PROTEI	N
59:00:00	6myv-B	2.9	3.7	64	522	6	MOLECULE:	SIALIDA	SE26	
60:00:00	8qx7-B	2.9	3.1	62	126	10	MOLECULE:	ALL4940	PROTEI	N
61:00:00	8v1l-E	2.9	3.3	70	134	6	MOLECULE:	RAS GTP	ASE-ACT	IVATING PROTEIN-BINDING PROTEIN 1
62:00:00	7jr9-D	2.9	3.1	66	339	6	MOLECULE:	RADIAL	SPOKE P	ROTEIN 9
63:00:00	4xja-A	2.9	3.2	63	658	2	MOLECULE:	SIALIDA	SE B	
64:00:00	6eku-A	2.9	3.1	71	755	7	MOLECULE:	SIALIDA	SE	
65:00:00	2pui-B	2.9	1.9	49	367	4	MOLECULE:	METHYLT	HIORIBO	SE KINASE
66:00:00	1eui-D	2.9	2.6	48	70	8	MOLECULE:	URACIL-	DNA GLY	COSYLASE
67:00:00	2r4i-D	2.9	3.7	63	117	10	MOLECULE:	UNCHARA	CTERIZE	D PROTEIN
68:00:00	7w19-B	2.9	2.4	55	291	5	MOLECULE:	MACROLI	DE 2'-P	HOSPHOTRANSFERASE
69:00:00	4ovm-B	2.9	2.9	65	126	12	MOLECULE:	UNCHARA	CTERIZE	D PROTEIN SGCJ
70:00:00	3ob8-A	2.9	3.4	48	1024	6	MOLECULE:	BETA-GA	LACTOSI	DASE

71:00:00	7qy9-A	2.9	3.8	63	519	5	MOLECULE:	BNR/ASP	#NAME?	PEAT PROTEIN
72:00:00	7xhg-B	2.9	3.1	65	133	6	MOLECULE:	RAS GTP	ASE-ACT	IVATING PROTEIN-BINDING PROTEIN 1
73:00:00	1eui-C	2.9	2.6	48	70	8	MOLECULE:	URACIL-	DNA GLY	COSYLASE
74:00:00	4xyx-A	2.9	2.9	55	660	0	MOLECULE:	SIALIDA	SE B	
75:00:00	7zht-D	2.9	4.2	65	485	3	MOLECULE:	GLUCOSE	#NAME?	PHATE 1-DEHYDROGENASE
76:00:00	6sd0-C	2.9	2.7	50	1083	10	MOLECULE:	BETA-GA	LACTOSI	DASE
77:00:00	1gd4-A	2.9	3.7	66	98	5	MOLECULE:	CYSTATI	N A	
78:00:00	2chc-C	2.9	3.4	63	159	10	MOLECULE:	PROTEIN	RV3472	
79:00:00	5ohl-F	2.9	3.7	58	88	5	MOLECULE:	K6-SPEC	IFIC AF	FIMER
80:00:00	2wgq-A	2.9	3.1	65	720	11	MOLECULE:	AMINE O	XIDASE	
81:00:00	2sim-A	2.9	2.9	62	381	5	MOLECULE:	SIALIDA	SE	
82:00:00	7zht-B	2.9	4.3	65	525	3	MOLECULE:	GLUCOSE	#NAME?	PHATE 1-DEHYDROGENASE
83:00:00	3gzr-B	2.9	3.7	68	142	9	MOLECULE:	UNCHARA	CTERIZE	D PROTEIN WITH A NTF2-LIKE FOLD
84:00:00	1lqg-D	2.9	2.6	48	70	8	MOLECULE:	URACIL-	DNA GLY	COSYLASE
85:00:00	7a5x-B	2.9	3.3	64	472	6	MOLECULE:	SIALIDA	SE A	
86:00:00	1gy7-B	2.9	3.6	68	122	7	MOLECULE:	NUCLEAR	TRANSP	ORT FACTOR 2
87:00:00	4fp2-A	2.9	3.1	58	658	0	MOLECULE:	SIALIDA	SE B	
88:00:00	7zhy-B	2.9	4.4	66	533	3	MOLECULE:	GLUCOSE	#NAME?	PHATE 1-DEHYDROGENASE
89:00:00	1izn-C	2.9	3.6	73	273	5	MOLECULE:	CAPZ AL	PHA-1 S	UBUNIT
90:00:00	2imj-A	2.8	3.1	71	158	4	MOLECULE:	HYPOTHE	TICAL P	ROTEIN DUF1348
91:00:00	7k5n-A	2.8	2.8	59	238	10	MOLECULE:	SENSOR	DOMAIN-	CONTAINING DIGUANYLATE CYCLASE
92:00:00	4i5q-A	2.8	3.2	51	220	4	MOLECULE:	THIOL:D	ISULFID	E INTERCHANGE PROTEIN DSBC
93:00:00	4xzv-B	2.8	3.9	64	142	5	MOLECULE:	MALTOSE	#NAME?	G PERIPLASMIC PROTEIN,TP53-REGULATE
94:00:00	3rob-A	2.8	4.1	69	131	3	MOLECULE:	UNCHARA	CTERIZE	D CONSERVED PROTEIN
95:00:00	3bws-A	2.8	3.3	61	407	15	MOLECULE:	PROTEIN	LP49	
96:00:00	1vi8-B	2.8	3.9	68	146	9	MOLECULE:	HYPOTHE	TICAL P	ROTEIN YDII
97:00:00	3hzp-A	2.8	3.3	64	128	6	MOLECULE:	NTF2-LI	KE PROT	EIN OF UNKNOWN FUNCTION
98:00:00	8ghn-A	2.8	3.6	59	667	5	MOLECULE:	PROTEIN	HIR1	
99:00:00	2gu1-A	2.8	3.7	62	324	11	MOLECULE:	ZINC PE	PTIDASE	
0:00	2ia7-A	2.8	3.2	62	111	6	MOLECULE:	TAIL LY	SOZYME,	PUTATIVE
1:00	2gvi-A	2.8	3.5	65	201	9	MOLECULE:	CONSERV	ED HYPO	THETICAL PROTEIN
2:00	2r5x-B	2.8	3.2	55	118	5	MOLECULE:	UNCHARA	CTERIZE	D CONSERVED PROTEIN
3:00	7t5q-J	2.8	3.6	72	291	3	MOLECULE:	ACTIN-R	ELATED	PROTEIN 3
4:00	7xfk-C	2.8	3.7	65	132	14	MOLECULE:	SORDARI	N/HYPOX	YSORDARIN BIOSYNTHESIS CLUSTER PROT
5:00	7aed-B	2.8	3	58	138	5	MOLECULE:	PRGL		
6:00	4hz9-B	2.8	2.5	61	123	11	MOLECULE:	PUTATIV	E CYTOP	LASMIC PROTEIN
7:00	6xrb-A	2.8	4.3	67	150	6	MOLECULE:	SCIW		
8:00	8p32-D	2.8	3.4	65	125	3	MOLECULE:	BB0238		
9:00	3put-B	2.8	3.9	66	155	5	MOLECULE:	HYPOTHE	TICAL C	ONSERVED PROTEIN
10:00	4l8p-A	2.8	3.3	73	168	3	MOLECULE:	BILE AC	ID 7A-D	EHYDRATASE, BAIE
11:00	6khi-V	2.8	3	63	109	5	MOLECULE:	NAD(P)H	#NAME?	E OXIDOREDUCTASE SUBUNIT 1
12:00	3by9-A	2.8	2.7	54	259	7	MOLECULE:	SENSOR	PROTEIN	
13:00	8a9y-E	2.8	3.5	64	497	3	MOLECULE:	GLYCOSI	DE HYDR	OLASE FAMILY 32
14:00	2iv9-A	2.8	3.1	57	236	0	MOLECULE:	AP-2 CO	MPLEX S	UBUNIT BETA-2
15:00	5njb-B	2.8	2.9	62	445	5	MOLECULE:	METALLO	PROTEAS	E TLDD
16:00	6moc-A	2.8	2.8	57	363	5	MOLECULE:	PEPTIDA	SE M23	
17:00	1nyc-A	2.8	3.8	67	111	9	MOLECULE:	CYSTEIN	E PROTE	ASE INHIBITOR
18:00	5iw9-A	2.8	3.2	60	126	10	MOLECULE:	BASEPLA	TE WEDG	E PROTEIN GP25
19:00	8jxe-A	2.8	3	62	1469	10	MOLECULE:	LDL REC	EPTOR R	ELATED PROTEIN 2
20:00	3v7d-B	2.8	2.5	46	450	11	MOLECULE:	SUPPRES	SOR OF	KINETOCHORE PROTEIN 1
21:00	8t5j-A	2.8	3.1	48	154	8	MOLECULE:	OUTER M	EMBRANE	LIPOCARRIER LOLA FAMILY PROTEIN
22:00	3ehc-B	2.8	3.5	70	128	11	MOLECULE:	SNOAL-L	IKE POL	YKETIDE CYCLASE
23:00	3hk4-A	2.8	3.5	67	118	12	MOLECULE:	MLR7391	PROTEI	N
24:00:00	5edf-A	2.8	3	54	224	4	MOLECULE:	FRPC OP	ERON PR	OTEIN

25:00:00	6pny-A	2.8	4	66	116	12	MOLECULE:	FLPP3		
26:00:00	7erl-A	2.8	3.4	63	545	8	MOLECULE:	BETA-XY	LANASE	
27:00:00	2gey-A	2.8	3.5	70	157	6	MOLECULE:	ACLR PR	OTEIN	
28:00:00	6h3i-F	2.8	3.7	66	345	8	MOLECULE:	PROTEIN	INVOLV	ED IN GLIDING MOTILITY SPRA
29:00:00	3gzr-A	2.8	3.6	64	143	9	MOLECULE:	UNCHARA	CTERIZE	D PROTEIN WITH A NTF2-LIKE FOLD
30:00:00	3f40-A	2.8	3.8	62	112	10	MOLECULE:	UNCHARA	CTERIZE	D NTF2-LIKE PROTEIN
31:00:00	6i7s-G	2.8	5.1	72	863	4	MOLECULE:	PROTEIN	DISULF	IDE-ISOMERASE
32:00:00	3vsf-C	2.8	3.3	63	482	6	MOLECULE:	RICIN B	LECTIN	
33:00:00	6zlt-B	2.8	7.1	65	933	3	MOLECULE:	SUSD HO	MOLOG	
34:00:00	8gex-A	2.8	3.6	59	411	7	MOLECULE:	DUF4374	DOMAIN	-CONTAINING PROTEIN
35:00:00	8ye0-C	2.8	3.4	70	290	6	MOLECULE:	LYNF/TR	UF/PATF	FAMILY PEPTIDE O-PRENYLTRANSFERASE
36:00:00	3uv1-A	2.8	3.2	71	190	6	MOLECULE:	DER F 7	ALLERG	EN
37:00:00	7akv-A	2.8	2.5	59	375	7	MOLECULE:	PLASMA	PROTEAS	E C1 INHIBITOR
38:00:00	8dwj-A	2.8	4	58	339	10	MOLECULE:	RNA(5'-	PPP-GCC	GA-3')
39:00:00	6nhi-A	2.8	4.2	64	448	6	MOLECULE:	HISTIDI	NE--TRN	A LIGASE
40:00:00	3df6-B	2.8	3	56	99	13	MOLECULE:	ORF99		
41:00:00	3s5t-A	2.8	3.7	69	253	7	MOLECULE:	DUF3298	FAMILY	PROTEIN
42:00:00	4i0o-A	2.8	2.6	47	463	11	MOLECULE:	PROTEIN	ELYS	
43:00:00	6yai-E	2.8	3.8	66	726	5	MOLECULE:	CLATHRI	N HEAVY	CHAIN
44:00:00	6s9u-A	2.8	3.9	69	520	9	MOLECULE:	PUTATIV	E SUCRO	SE PHOSPHORYLASE
45:00:00	5hi8-A	2.8	3.4	73	136	5	MOLECULE:	ANTENNA	PROTEI	N
46:00:00	4v1a-i	2.8	3.6	74	242	8	MOLECULE:	MITORIB	OSOMAL	PROTEIN ML37, MRPL37
47:00:00	8bbg-B	2.8	3.3	58	1426	9	MOLECULE:	WD REPE	AT-CONT	AINING PROTEIN 19
48:00:00	7qpg-R	2.8	3	55	2208	7	MOLECULE:	PROTEIN	ZWILCH	HOMOLOG
49:00:00	5hlb-A	2.8	5	58	733	12	MOLECULE:	PENICIL	LIN-BIN	DING PROTEIN 1B
50:00:00	4ocu-A	2.8	2.3	52	356	6	MOLECULE:	N-ACETY	LHEXOSA	MINE 1-PHOSPHATE KINASE
51:00:00	4irt-A	2.8	3	57	398	7	MOLECULE:	UNCHARA	CTERIZE	D PROTEIN
52:00:00	6m36-I	2.8	4.1	55	104	11	MOLECULE:	SERINE-	PROTEIN	KINASE RSBW
53:00:00	3b7c-A	2.8	3.6	69	121	10	MOLECULE:	UNCHARA	CTERIZE	D PROTEIN
54:00:00	7eeb-F	2.8	2.7	54	1042	7	MOLECULE:	ENHANCE	D GREEN	FLUORESCENT PROTEIN,CATION CHANNEL
55:00:00	5h2d-A	2.8	7	62	418	8	MOLECULE:	KLLA0C0	4147P	
56:00:00	8rf0-A	2.8	3	50	2780	12	MOLECULE:	CYCLIC	BETA-(1	,2)-GLUCAN SYNTHASE NDVB
57:00:00	5ogs-A	2.8	3.3	59	403	7	MOLECULE:	WD REPE	AT AND	HMG-BOX DNA-BINDING PROTEIN 1
58:00:00	3aae-G	2.8	3.6	73	270	5	MOLECULE:	F-ACTIN	#NAME?	G PROTEIN SUBUNIT ALPHA-1
59:00:00	4fpc-A	2.8	3.3	63	658	2	MOLECULE:	SIALIDA	SE B	
60:00:00	6fej-B	2.8	2.7	57	118	9	MOLECULE:	ALL4940	PROTEI	N
61:00:00	7yth-C	2.8	3.1	63	314	8	MOLECULE:	KETOSTE	ROID IS	OMERASE-RELATED PROTEIN
62:00:00	4fcm-A	2.8	3	64	132	6	MOLECULE:	RAS GTP	ASE-ACT	IVATING PROTEIN-BINDING PROTEIN 1
63:00:00	6f1u-K	2.8	3.6	74	278	7	MOLECULE:	ARP1 AC	TIN REL	ATED PROTEIN 1 HOMOLOG A
64:00:00	2pup-A	2.8	2.4	52	371	4	MOLECULE:	METHYLT	HIORIBO	SE KINASE
65:00:00	6w40-B	2.8	3.2	62	115	16	MOLECULE:	DENOVO	NTF2	
66:00:00	8th5-F	2.8	3.7	69	130	7	MOLECULE:	RAS GTP	ASE-ACT	IVATING PROTEIN-BINDING PROTEIN 1
67:00:00	2ya6-A	2.8	3.1	60	470	10	MOLECULE:	NEURAMI	NIDASE	A
68:00:00	2woh-A	2.8	3.3	68	718	10	MOLECULE:	PRIMARY	AMINE	OXIDASE
69:00:00	5ohl-E	2.8	3.7	58	89	5	MOLECULE:	K6-SPEC	IFIC AF	FIMER
70:00:00	7zhu-A	2.8	4.6	65	534	3	MOLECULE:	GLUCOSE	#NAME?	PHATE 1-DEHYDROGENASE
71:00:00	5kky-B	2.8	3.6	71	470	4	MOLECULE:	SIALIDA	SE A	
72:00:00	7xhg-D	2.8	3.5	65	133	6	MOLECULE:	RAS GTP	ASE-ACT	IVATING PROTEIN-BINDING PROTEIN 1
73:00:00	6d24-A	2.8	4.6	73	502	4	MOLECULE:	GLUCOSE	#NAME?	PHATE 1-DEHYDROGENASE
74:00:00	3pjq-A	2.8	3.1	61	626	7	MOLECULE:	TRANS-S	IALIDAS	E
75:00:00	4xio-A	2.8	3.4	60	658	7	MOLECULE:	SIALIDA	SE B	
76:00:00	1d6y-A	2.8	3.1	65	718	8	MOLECULE:	COPPER	AMINE O	XIDASE
77:00:00	2xhn-A	2.8	3	47	508	6	MOLECULE:	RHAMNOG	ALACTUR	ONASE B
78:00:00	6f1t-K	2.8	3.6	74	278	7	MOLECULE:	ARP1 AC	TIN REL	ATED PROTEIN 1 HOMOLOG A

79:00:00	1cyu-A	2.8	3	59	98	5	MOLECULE:	CYSTATI	N A	
80:00:00	2xgl-A	2.7	2.8	60	90	0	MOLECULE:	COLICIN	#NAME?	NITY PROTEIN
81:00:00	6sum-A	2.7	3	57	335	2	MOLECULE:	AMICOUM	ACIN KI	NASE
82:00:00	7jsr-A	2.7	3.8	78	1496	5	MOLECULE:	NAD-SPE	CIFIC G	LUTAMATE DEHYDROGENASE
83:00:00	2o3o-I	2.7	3.2	59	244	5	MOLECULE:	YYCI PR	OTEIN	
84:00:00	4fvs-A	2.7	3.7	81	214	6	MOLECULE:	PUTATIV	E LIPOP	ROTEIN
85:00:00	6zxf-z	2.7	3.7	66	373	5	MOLECULE:	PRE-18S	RIBOSO	MAL RNA
86:00:00	3w9k-A	2.7	3.8	61	135	7	MOLECULE:	FATTY A	CID-BIN	DING PROTEIN
87:00:00	1eq6-A	2.7	3.4	72	189	8	MOLECULE:	MOG1P		
88:00:00	6gn5-A	2.7	3.8	67	177	6	MOLECULE:	GRAM DO	MAIN-CO	NTAINING PROTEIN 1C
89:00:00	4plp-A	2.7	3	74	475	7	MOLECULE:	HOMOSPE	RMIDINE	SYNTHASE
90:00:00	4l9h-A	2.7	4	69	155	4	MOLECULE:	F-BOX O	NLY PRO	TEIN 7
91:00:00	6kac-P	2.7	3.6	71	188	7	MOLECULE:	PHOTOSY	STEM II	PROTEIN D1
92:00:00	3ub9-B	2.7	2.5	57	165	7	MOLECULE:	CHEMORE	CEPTOR	TLPB
93:00:00	8qc6-A	2.7	3.5	62	366	5	MOLECULE:	OXIDORE	DUCTASE	
94:00:00	1aom-B	2.7	3.4	64	559	9	MOLECULE:	NITRITE	REDUCT	ASE
95:00:00	3g8z-A	2.7	3.8	64	129	11	MOLECULE:	PROTEIN	OF UNK	NOWN FUNCTION WITH CYSTATIN-LIKE FO
96:00:00	3i0o-A	2.7	3	58	329	9	MOLECULE:	SPECTIN	OMYCIN	PHOSPHOTRANSFERASE
97:00:00	2bmo-B	2.7	3.5	70	194	10	MOLECULE:	OXYGENA	SE-ALPH	A NBDO
98:00:00	5g4y-A	2.7	2.1	56	147	5	MOLECULE:	CHEMOTA	XIS PRO	TEIN
99:00:00	7qh5-A	2.7	3.9	73	313	7	MOLECULE:	RNA POL	YMERASE	SIGMA FACTOR
0:00	2xlq-A	2.7	3.7	67	316	3	MOLECULE:	CLOQ		
1:00	6tlb-A	2.7	4.1	69	185	13	MOLECULE:	SERINE/	THREONI	NE PROTEIN KINASE
2:00	3byv-A	2.7	3.9	63	345	8	MOLECULE:	RHOPTRY	KINASE	
3:00	3hw2-A	2.7	4.8	56	308	9	MOLECULE:	PROTEIN	SIFA	
4:00	8bo9-A	2.7	3.2	58	174	9	MOLECULE:	NON STR	UCTURAL	POLYPROTEIN
5:00	6eus-A	2.7	3.9	56	346	5	MOLECULE:	DCAP-LI	KE PROT	EIN
6:00	6qp8-A	2.7	3.6	66	622	3	MOLECULE:	IP13724	P	
7:00	7q4p-C	2.7	3	61	878	7	MOLECULE:	SPLICIN	G FACTO	R 3A SUBUNIT 2
8:00	4r5o-A	2.7	3.6	64	426	6	MOLECULE:	QUINONP	ROTEIN	ALCOHOL DEHYDROGENASE-LIKE PROTEIN
9:00	8hmc-B	2.7	3.8	58	1195	5	MOLECULE:	INTRAFL	AGELLAR	TRANSPORT PROTEIN 122 HOMOLOG
10:00	6nd4-Q	2.7	3.3	59	862	12	MOLECULE:	ETS RRN	A	
11:00	8wid-w	2.7	3.4	60	126	10	MOLECULE:	16S RRN	A	
12:00	3mlq-A	2.7	4	60	186	5	MOLECULE:	DNA-DIR	ECTED R	NA POLYMERASE SUBUNIT BETA
13:00	6b7l-A	2.7	8.4	51	290	4	MOLECULE:	IMMUNE	MODULAT	OR A
14:00	8alo-B	2.7	3.9	60	148	12	MOLECULE:	CHOLERA	TOXIN	TRANSCRIPTIONAL ACTIVATOR
15:00	7jta-A	2.7	3.2	48	56	2	MOLECULE:	NTF2-LI	KE NUCL	EASE/ANTI-CRISPR
16:00	3jr1-A	2.7	2.1	53	296	8	MOLECULE:	PUTATIV	E FRUCT	OSAMINE-3-KINASE
17:00	8vwp-A	2.7	4.1	65	502	9	MOLECULE:	LANGYA	VIRUS A	TTACHMENT (G) PROTEIN
18:00	6xp5-N	2.7	3.4	61	544	2	MOLECULE:	MEDIATO	R OF RN	A POLYMERASE II TRANSCRIPTION SUBUN
19:00	3tkn-A	2.7	4.2	58	451	9	MOLECULE:	NUCLEOP	ORIN NU	P82
20:00	7nie-C	2.7	5.3	61	294	10	MOLECULE:	GLYCERO	L KINAS	E
21:00	6m76-A	2.7	3.3	64	923	5	MOLECULE:	LPXTG-M	OTIF CE	LL WALL ANCHOR DOMAIN PROTEIN
22:00	6f37-A	2.7	2.7	55	113	5	MOLECULE:	NANO3,F	UCOSE-B	INDING LECTIN PROTEIN
23:00	5c9p-A	2.7	3	59	354	10	MOLECULE:	PLL LEC	TIN	
24:00:00	4hxg-F	2.7	4.1	62	614	5	MOLECULE:	PUTATIV	E UNCHA	RACTERIZED PROTEIN PH0594
25:00:00	7n3v-A	2.7	2.8	48	266	4	MOLECULE:	LMCA		
26:00:00	2fkj-A	2.7	4.4	63	361	5	MOLECULE:	OUTER S	URFACE	PROTEIN A
27:00:00	5x7o-A	2.7	3.1	56	1247	7	MOLECULE:	GLYCOSI	DE HYDR	OLASE FAMILY 31 ALPHA-GLUCOSIDASE
28:00:00	6exp-F	2.7	3.2	60	103	2	MOLECULE:	SIRV3 A	CRID1 (	GP02) ANTI-CRISPR PROTEIN
29:00:00	5yje-B	2.7	3.5	57	318	7	MOLECULE:	PROTEIN	HIRA	
30:00:00	6hxv-A	2.7	1.5	46	163	4	MOLECULE:	COILED-	COIL DO	MAIN-CONTAINING PROTEIN 61
31:00:00	6eu4-A	2.7	4.5	57	587	7	MOLECULE:	TAIL SP	IKE PRO	TEIN
32:00:00	2p9w-A	2.7	3.4	59	333	7	MOLECULE:	MAL S 1	ALLERG	ENIC PROTEIN

33:00:00	7ep9-A	2.7	3.7	57	660	12	MOLECULE:	S9 FAMI	LY PEPT	IDASE
34:00:00	4ww9-A	2.7	3.4	52	238	6	MOLECULE:	EKC/KEO	PS COMP	LEX SUBUNIT BUD32
35:00:00	7r78-A	2.7	5.7	65	1881	3	MOLECULE:	DNA REP	AIR PRO	TEIN RAD8
36:00:00	6jdp-A	2.7	4.5	70	241	6	MOLECULE:	IMM52 F	AMILY P	ROTEIN
37:00:00	2h0h-B	2.7	3.1	54	230	9	MOLECULE:	THIOL:D	ISULFID	E INTERCHANGE PROTEIN DSBG
38:00:00	8uzl-A	2.7	5.9	62	143	10	MOLECULE:	DESIGNE	D TRANS	MEMBRANE BETA-BARREL TMB10_163
39:00:00	3ci0-K	2.7	3.4	60	280	5	MOLECULE:	PSEUDOP	ILIN GS	PI
40:00:00	5gva-A	2.7	3.1	56	305	5	MOLECULE:	WD REPE	AT AND	HMG-BOX DNA-BINDING PROTEIN 1
41:00:00	4xmi-A	2.7	3.3	63	657	2	MOLECULE:	SIALIDA	SE B	
42:00:00	2chc-B	2.7	3.2	62	161	5	MOLECULE:	PROTEIN	RV3472	
43:00:00	7mhu-B	2.7	3.6	62	374	3	MOLECULE:	EXO-ALP	HA-SIAL	IDASE
44:00:00	6q0v-B	2.7	3.9	66	208	11	MOLECULE:	DNA DAM	AGE-BIN	DING PROTEIN 1
45:00:00	7dmp-b	2.7	3.3	63	336	8	MOLECULE:	RADIAL	SPOKE H	EAD PROTEIN 4 HOMOLOG A
46:00:00	1gd3-A	2.7	3.7	67	98	4	MOLECULE:	CYSTATI	N A	
47:00:00	4yw4-A	2.7	3.3	56	659	2	MOLECULE:	NEURAMI	NIDASE	C
48:00:00	6eks-A	2.7	2.6	66	749	8	MOLECULE:	SIALIDA	SE	
49:00:00	7yth-D	2.7	3.5	68	311	10	MOLECULE:	KETOSTE	ROID IS	OMERASE-RELATED PROTEIN
50:00:00	2wo0-A	2.7	3.3	67	718	9	MOLECULE:	PRIMARY	AMINE	OXIDASE
51:00:00	3vd9-B	2.7	3.5	71	1016	8	MOLECULE:	BETA-GA	LACTOSI	DASE
52:00:00	8v1l-B	2.7	3.2	66	133	6	MOLECULE:	RAS GTP	ASE-ACT	IVATING PROTEIN-BINDING PROTEIN 1
53:00:00	7zht-A	2.7	4.5	67	530	3	MOLECULE:	GLUCOSE	#NAME?	PHATE 1-DEHYDROGENASE
54:00:00	4ovm-D	2.7	2.9	64	125	13	MOLECULE:	UNCHARA	CTERIZE	D PROTEIN SGCJ
55:00:00	5ohl-G	2.7	3.8	56	89	5	MOLECULE:	K6-SPEC	IFIC AF	FIMER
56:00:00	5ohl-D	2.7	3.8	58	89	5	MOLECULE:	K6-SPEC	IFIC AF	FIMER
57:00:00	4dux-A	2.7	3.6	69	1015	9	MOLECULE:	BETA-GA	LACTOSI	DASE
58:00:00	2vw1-A	2.7	3	57	658	0	MOLECULE:	SIALIDA	SE B	
59:00:00	2ya7-A	2.7	3.5	67	469	6	MOLECULE:	NEURAMI	NIDASE	A
60:00:00	2wgq-B	2.7	3.5	58	723	9	MOLECULE:	AMINE O	XIDASE	
61:00:00	8th1-B	2.7	3.3	66	133	6	MOLECULE:	RAS GTP	ASE-ACT	IVATING PROTEIN-BINDING PROTEIN 1
62:00:00	7xhf-B	2.7	3.4	67	134	6	MOLECULE:	RAS GTP	ASE-ACT	IVATING PROTEIN-BINDING PROTEIN 1
63:00:00	3opz-C	2.7	3.1	59	625	7	MOLECULE:	TRANS-S	IALIDAS	E
64:00:00	4ovm-H	2.7	3	64	126	13	MOLECULE:	UNCHARA	CTERIZE	D PROTEIN SGCJ
65:00:00	4xhb-A	2.7	2.9	55	658	0	MOLECULE:	SIALIDA	SE B	
66:00:00	5f9t-B	2.7	3.3	56	659	2	MOLECULE:	NEURAMI	NIDASE	C
67:00:00	4yw5-A	2.7	2.7	46	659	2	MOLECULE:	NEURAMI	NIDASE	C
68:00:00	3ob8-C	2.7	4.3	51	1024	8	MOLECULE:	BETA-GA	LACTOSI	DASE
69:00:00	1eur-A	2.7	3.3	65	361	15	MOLECULE:	SIALIDA	SE	
70:00:00	3elg-B	2.7	4.1	55	126	5	MOLECULE:	UNCHARA	CTERIZE	D PERIPLASMIC PROTEIN
71:00:00	4xil-A	2.7	3	56	658	0	MOLECULE:	SIALIDA	SE B	
72:00:00	1gyb-B	2.7	3.6	64	122	8	MOLECULE:	NUCLEAR	TRANSP	ORT FACTOR 2
73:00:00	4yw4-B	2.7	2.9	54	659	2	MOLECULE:	NEURAMI	NIDASE	C
74:00:00	4yz2-B	2.7	3.3	56	656	2	MOLECULE:	SIALIDA	SE NANC	
75:00:00	3vd9-C	2.7	3.1	64	1015	6	MOLECULE:	BETA-GA	LACTOSI	DASE
76:00:00	4fpo-B	2.7	3.1	58	658	0	MOLECULE:	SIALIDA	SE B	
77:00:00	8p26-J	2.6	2.4	51	147	12	MOLECULE:	U2 SMAL	L NUCLE	AR RIBONUCLEOPROTEIN AUXILIARY FACT
78:00:00	5ezu-A	2.6	2.8	50	76	14	MOLECULE:	PROTEIN	A46	
79:00:00	8abv-A	2.6	3.3	71	244	7	MOLECULE:	SNOAL-L	IKE DOM	AIN-CONTAINING PROTEIN
80:00:00	3cnx-C	2.6	3.1	61	148	3	MOLECULE:	UNCHARA	CTERIZE	D PROTEIN
81:00:00	2obd-A	2.6	4	74	472	7	MOLECULE:	CHOLEST	ERYL ES	TER TRANSFER PROTEIN
82:00:00	4x2m-A	2.6	3.3	67	177	3	MOLECULE:	MTR2		
83:00:00	2lnj-A	2.6	4.9	74	170	1	MOLECULE:	PUTATIV	E UNCHA	RACTERIZED PROTEIN SLL1418
84:00:00	6kaw-A	2.6	3.6	70	381	6	MOLECULE:	CGHA		
85:00:00	6hsy-A	2.6	3.4	67	190	6	MOLECULE:	TOLUENE	TOLERA	NCE PROTEIN TTG2D
86:00:00	4y4v-A	2.6	3	65	318	3	MOLECULE:	CONSERV	ED HYPO	THETICAL SECRETED PROTEIN

87:00:00	5kku-B	2.6	4	61	289	0	MOLECULE:	POLYKET	IDE SYN	THASE TYPE I
88:00:00	7w0w-A	2.6	3.4	56	131	9	MOLECULE:	METHYL-	ACCEPTI	NG CHEMOTAXIS PROTEIN
89:00:00	8gqe-A	2.6	3.5	59	348	8	MOLECULE:	WD REPE	AT-CONT	AINING PROTEIN RUP2
90:00:00	2y3u-A	2.6	3.9	53	679	2	MOLECULE:	COLLAGE	NASE	
91:00:00	7apk-m	2.6	3.6	66	549	8	MOLECULE:	THO COM	PLEX SU	BUNIT 1
92:00:00	4h3u-A	2.6	3.3	65	131	6	MOLECULE:	HYPOTHE	TICAL P	ROTEIN
93:00:00	7yzi-A	2.6	3.6	65	378	6	MOLECULE:	ADENYLA	TE CYCL	ASE
94:00:00	1s18-A	2.6	3.2	64	317	6	MOLECULE:	APYRASE		
95:00:00	2a8e-A	2.6	3.9	77	210	8	MOLECULE:	HYPOTHE	TICAL P	ROTEIN YKTB
96:00:00	3vu4-A	2.6	3.4	59	319	7	MOLECULE:	KMHSV2		
97:00:00	7pab-A	2.6	3.8	62	415	6	MOLECULE:	NUCLEAR	EGRESS	PROTEIN 2,NUCLEAR EGRESS PROTEIN 1
98:00:00	1k1x-A	2.6	3.3	71	636	6	MOLECULE:	4-ALPHA	#NAME?	OTRANSFERASE
99:00:00	2kcd-A	2.6	3.4	69	120	6	MOLECULE:	UNCHARA	CTERIZE	D PROTEIN SSP0047
0:00	5h3z-A	2.6	6.7	55	1113	2	MOLECULE:	UNCHARA	CTERIZE	D PROTEIN
1:00	6kml-C	2.6	3.7	52	105	4	MOLECULE:	MRNA IN	TERFERA	SE TOXIN HIGB
2:00	8t1l-L	2.6	3.4	66	559	6	MOLECULE:	MEDIATO	R OF RN	A POLYMERASE II TRANSCRIPTION SUBUN
3:00	3dkq-A	2.6	3.2	60	230	7	MOLECULE:	PKHD-TY	PE HYDR	OXYLASE SBAL_3634
4:00	1pu1-A	2.6	3.7	59	91	10	MOLECULE:	HYPOTHE	TICAL P	ROTEIN MTH677
5:00	1r5m-A	2.6	3.4	57	351	12	MOLECULE:	SIR4-IN	TERACTI	NG PROTEIN SIF2
6:00	6nd4-O	2.6	4.3	60	832	12	MOLECULE:	ETS RRN	A	
7:00	7uhy-C	2.6	3.1	57	578	7	MOLECULE:	GATOR C	OMPLEX	PROTEIN MIOS
8:00	4o9d-B	2.6	3.1	57	392	7	MOLECULE:	RIK1-AS	SOCIATE	D FACTOR 1
9:00	2rk0-A	2.6	3.5	51	135	2	MOLECULE:	GLYOXAL	ASE/BLE	OMYCIN RESISTANCE PROTEIN/DIOXYGENA
10:00	7zty-A	2.6	3.6	54	345	4	MOLECULE:	CNH DOM	AIN-CON	TAINING PROTEIN
11:00	5mc1-B	2.6	3.9	73	487	12	MOLECULE:	XAA-PRO	DIPEPT	IDASE
12:00	3cyg-A	2.6	3.9	64	215	5	MOLECULE:	UNCHARA	CTERIZE	D PROTEIN
13:00	6rwb-A	2.6	3.4	69	1873	7	MOLECULE:	TOXIN,T	OXIN CO	MPLEX SUBUNIT TCAB,PUTATIVE TOXIN S
14:00	4qtq-A	2.6	5	54	209	4	MOLECULE:	XAC2610	PROTEI	N
15:00	1fwx-A	2.6	4.2	67	591	6	MOLECULE:	NITROUS	OXIDE	REDUCTASE
16:00	6mv2-A	2.6	4.4	59	356	8	MOLECULE:	CYTOCHR	OME B5	REDUCTASE 4
17:00	6icl-A	2.6	3.1	59	338	5	MOLECULE:	METHYLX	ANTHINE	N3-DEMETHYLASE NDMB
18:00	8q7n-F	2.6	3.1	60	431	7	MOLECULE:	U5 SNRN	A	
19:00	8ih8-D	2.6	4.4	60	130	3	MOLECULE:	ANTI-SI	GMA-F F	ACTOR ANTAGONIST RSFB
20:00	8r54-A	2.6	4.6	63	1982	5	MOLECULE:	TENEURI	N-3	
21:00	6e14-D	2.6	7.3	64	805	6	MOLECULE:	TYPE 1	FIMBRIN	D-MANNOSE SPECIFIC ADHESIN
22:00	5chx-A	2.6	2.5	49	210	8	MOLECULE:	XRCC4-M	YH7-159	0-1657
23:00	7btx-L	2.6	6.1	59	314	7	MOLECULE:	MITOCHO	NDRIAL	OUTER MEMBRANE BETA-BARREL PROTEIN
24:00:00	6f3a-h	2.6	3.8	70	401	10	MOLECULE:	ARP1 AC	TIN REL	ATED PROTEIN 1 HOMOLOG A
25:00:00	5ho2-A	2.6	3.3	66	803	3	MOLECULE:	EXTRACE	LLULAR	ARABINANASE
26:00:00	7obm-A	2.6	2.8	57	624	4	MOLECULE:	PROLYL	ENDOPEP	TIDASE-LIKE
27:00:00	3ldk-A	2.6	3.5	56	634	5	MOLECULE:	FRUCTOS	YLTRANS	FERASE
28:00:00	7bkb-k	2.6	3	47	386	13	MOLECULE:	COB--CO	M HETER	ODISULFIDE REDUCTASE IRON-SULFUR SU
29:00:00	7upn-C	2.6	3.1	47	208	4	MOLECULE:	PEPTIDY	L-PROLY	L CIS-TRANS ISOMERASE A
30:00:00	6jq9-B	2.6	2.6	58	483	10	MOLECULE:	SHORT U	LVAN LY	ASE
31:00:00	3lic-A	2.6	2.8	59	265	12	MOLECULE:	SENSOR	PROTEIN	
32:00:00	3dcz-A	2.6	3.6	67	170	12	MOLECULE:	PUTATIV	E RNFG	SUBUNIT OF ELECTRON TRANSPORT COMPL
33:00:00	7uea-U	2.6	3.8	55	364	5	MOLECULE:	PHOTOSY	STEM P8	40 REACTION CENTER, LARGE SUBUNIT
34:00:00	2eyq-A	2.6	3.1	57	1146	4	MOLECULE:	TRANSCR	IPTION-	REPAIR COUPLING FACTOR
35:00:00	5bw0-F	2.6	7.4	49	91	4	MOLECULE:	TYPE II	SECRET	ION SYSTEM PROTEIN J
36:00:00	6k4e-B	2.6	3.7	66	246	9	MOLECULE:	HAMP DO	MAIN-CO	NTAINING PROTEIN
37:00:00	7p3l-A	2.6	2.9	66	332	8	MOLECULE:	ISOPENI	CILLIN	N SYNTHASE
38:00:00	3csl-A	2.6	6.2	67	753	4	MOLECULE:	HASR PR	OTEIN	
39:00:00	1eut-A	2.6	3.5	58	601	7	MOLECULE:	SIALIDA	SE	
40:00:00	7uic-n	2.6	3.5	63	220	3	MOLECULE:	MEDIATO	R OF RN	A POLYMERASE II TRANSCRIPTION SUBUN

41:00:00	5k1c-C	2.6	2.8	53	407	8	MOLECULE:	UBIQUIT	IN CARB	OXYL-TERMINAL HYDROLASE 12
42:00:00	6stx-C	2.6	3.1	58	663	3	MOLECULE:	KELCH D	OMAIN-C	ONTAINING PROTEIN
43:00:00	8t8s-B	2.6	3	57	664	4	MOLECULE:	SORTILI	N	
44:00:00	1cyv-A	2.6	2.9	58	98	3	MOLECULE:	CYSTATI	N A	
45:00:00	2xhn-B	2.6	2.9	67	508	4	MOLECULE:	RHAMNOG	ALACTUR	ONASE B
46:00:00	4bl9-C	2.6	4.6	73	724	7	MOLECULE:	MALTOSE	#NAME?	G PERIPLASMIC PROTEIN, SUPPRESSOR O
47:00:00	6mnj-B	2.6	2.9	58	511	7	MOLECULE:	HZ136		
48:00:00	1lqg-C	2.6	3.1	52	73	8	MOLECULE:	URACIL-	DNA GLY	COSYLASE
49:00:00	2pu8-B	2.6	2.4	48	364	4	MOLECULE:	METHYLT	HIORIBO	SE KINASE
50:00:00	7e8h-K	2.6	4.3	72	760	14	MOLECULE:	DIPEPTI	DYL AMI	NOPEPTIDASE-LIKE PROTEIN 6
51:00:00	4fpe-A	2.6	3	56	658	0	MOLECULE:	SIALIDA	SE B	
52:00:00	2ya4-B	2.6	3.2	61	471	8	MOLECULE:	NEURAMI	NIDASE	A
53:00:00	4ovm-J	2.6	3.2	66	124	11	MOLECULE:	UNCHARA	CTERIZE	D PROTEIN SGCJ
54:00:00	4ovm-E	2.6	3.1	67	126	9	MOLECULE:	UNCHARA	CTERIZE	D PROTEIN SGCJ
55:00:00	5ohl-B	2.6	3.7	55	89	5	MOLECULE:	K6-SPEC	IFIC AF	FIMER
56:00:00	4fph-A	2.6	2.9	56	658	0	MOLECULE:	SIALIDA	SE B	
57:00:00	2vvz-A	2.6	4.1	73	470	4	MOLECULE:	SIALIDA	SE A	
58:00:00	4xa2-A	2.6	3.7	62	490	5	MOLECULE:	MALTOSE	#NAME?	G PERIPLASMIC PROTEIN,MBP-PILA: C
59:00:00	7a5x-A	2.6	3.1	60	472	7	MOLECULE:	SIALIDA	SE A	
60:00:00	3lvt-A	2.6	3	70	833	4	MOLECULE:	GLYCOSY	L HYDRO	LASE, FAMILY 38
61:00:00	3oba-B	2.6	3.8	50	1024	8	MOLECULE:	BETA-GA	LACTOSI	DASE
62:00:00	4q0p-A	2.5	3.6	68	249	4	MOLECULE:	L-RIBOS	E ISOME	RASE
63:00:00	2e3m-A	2.5	4.3	73	237	10	MOLECULE:	LIPID-T	RANSFER	PROTEIN CERT
64:00:00	3kzt-A	2.5	3.2	64	132	14	MOLECULE:	UNCHARA	CTERIZE	D PROTEIN
65:00:00	2bas-A	2.5	2.7	60	402	10	MOLECULE:	YKUI PR	OTEIN	
66:00:00	2xu8-A	2.5	5.1	52	116	10	MOLECULE:	PA1645		
67:00:00	5h7z-B	2.5	5.4	57	302	9	MOLECULE:	UNCHARA	CTERIZE	D PROTEIN
68:00:00	2y32-C	2.5	3.5	65	138	3	MOLECULE:	BLR5658	PROTEI	N
69:00:00	5ku5-A	2.5	2.9	60	148	7	MOLECULE:	SENSOR	KINASE	CUSS
70:00:00	8g1r-A	2.5	3.5	55	331	5	MOLECULE:	MAJOR H	EAD PRO	TEIN
71:00:00	5lkh-A	2.5	3.6	62	948	8	MOLECULE:	TCDA1		
72:00:00	2qhk-A	2.5	2.6	56	148	11	MOLECULE:	METHYL-	ACCEPTI	NG CHEMOTAXIS PROTEIN
73:00:00	3te8-B	2.5	3.3	57	125	14	MOLECULE:	SENSOR	HISTIDI	NE KINASE RISS
74:00:00	6p59-B	2.5	4.5	63	196	13	MOLECULE:	CORE-BI	NDING F	ACTOR SUBUNIT BETA
75:00:00	7t5q-I	2.5	3.6	76	283	1	MOLECULE:	ACTIN-R	ELATED	PROTEIN 3
76:00:00	4eg9-A	2.5	3.9	83	231	2	MOLECULE:	UNCHARA	CTERIZE	D PROTEIN SAOUHSC_02783
77:00:00	7d9b-A	2.5	3.6	65	588	5	MOLECULE:	ALPHA-G	LYCOSID	ASE
78:00:00	1oh1-A	2.5	3	58	109	0	MOLECULE:	STAPHOS	TATIN A	
79:00:00	6z9u-D	2.5	4.1	57	134	5	MOLECULE:	TRNA-SP	LICING	ENDONUCLEASE SUBUNIT SEN34
80:00:00	1vl4-A	2.5	3.7	65	428	8	MOLECULE:	PMBA-RE	LATED P	ROTEIN
81:00:00	8fg6-B	2.5	3.7	54	151	4	MOLECULE:	AMYLOID	OGENIC	PEPTIDE
82:00:00	5nj5-A	2.5	3.8	62	480	10	MOLECULE:	METALLO	PROTEAS	E TLDD
83:00:00	3nqz-A	2.5	3.6	66	175	6	MOLECULE:	SECRETE	D METAL	LOPROTEASE MCP02
84:00:00	5ul2-A	2.5	3.1	59	731	3	MOLECULE:	OXSB PR	OTEIN	
85:00:00	6lk8-J	2.5	3.7	84	1026	7	MOLECULE:	MGC8329	5 PROTE	IN
86:00:00	4lgq-A	2.5	3.8	63	134	5	MOLECULE:	PUTATIV	E POLYK	ETIDE CYCLASE
87:00:00	4ouh-A	2.5	3.4	62	149	6	MOLECULE:	PROTEAS	OME INH	IBITOR PI31 SUBUNIT
88:00:00	1pvj-A	2.5	3.3	65	339	8	MOLECULE:	PYROGEN	IC EXOT	OXIN B
89:00:00	4ci8-A	2.5	4.7	65	640	8	MOLECULE:	ECHINOD	ERM MIC	ROTUBULE-ASSOCIATED PROTEIN-LIKE 1
90:00:00	1su3-B	2.5	2.7	49	416	4	MOLECULE:	INTERST	ITIAL C	OLLAGENASE
91:00:00	6gxs-B	2.5	3.3	56	363	11	MOLECULE:	CV39L L	ECTIN	
92:00:00	2x2h-A	2.5	3.3	63	1025	6	MOLECULE:	ALPHA-1	,4-GLUC	AN LYASE ISOZYME 1
93:00:00	5txc-A	2.5	3.7	56	653	9	MOLECULE:	ATXE2		
94:00:00	3e99-A	2.5	3.8	69	149	6	MOLECULE:	BENZOAT	E 1,2-D	IOXYGENASE BETA SUBUNIT

95:00:00	8i4w-A	2.5	4.7	67	442	6	MOLECULE:	STRUCTU	RAL MAI	NTENANCE OF CHROMOSOMES PROTEIN 5	
96:00:00	7esn-A	2.5	3.7	64	435	8	MOLECULE:	L-RHAMN	OSE-ALP	HA-1,4-D-GLUCURONATE LYASE	
97:00:00	2dso-C	2.5	3	57	323	4	MOLECULE:	DRP35			
98:00:00	7epn-B	2.5	3.5	61	137	15	MOLECULE:	SNOAL-L	IKE DOM	AIN-CONTAINING PROTEIN	
99:00:00	2n19-A	2.5	4.3	53	87	6	MOLECULE:	SERINE/	THREONI	NE-PROTEIN KINASE PLK4	
0:00	3zpm-A	2.5	3.3	71	211	8	MOLECULE:	LATHERI	N		
1:00	6d7k-H	2.5	2.4	48	68	8	MOLECULE:	METHANE	MONOOX	YGENASE HYDROXYLASE, MMOX1	
2:00	6cl5-A	2.5	3.3	54	374	11	MOLECULE:	TAIL FI	BER PRO	TEIN	
3:00	5iri-A	2.5	3.3	58	105	10	MOLECULE:	SERINE/	THREONI	NE-PROTEIN KINASE BRSK1	
4:00	6xlf-C	2.5	4.2	68	316	3	MOLECULE:	ATP-DEP	ENDENT	MOLECULAR CHAPERONE HSC82	
5:00	7bkb-e	2.5	2.6	43	411	9	MOLECULE:	COB--CO	M HETER	ODISULFIDE REDUCTASE IRON-SULFUR SU	
6:00	8qca-C	2.5	3.4	64	384	11	MOLECULE:	ANTIVIR	AL HELI	CASE SKI2	
7:00	7pi3-A	2.5	4	67	328	4	MOLECULE:	CYSTEIN	E-RICH	PROTECTIVE ANTIGEN	
8:00	2pk8-A	2.5	3.7	49	94	8	MOLECULE:	UNCHARA	CTERIZE	D PROTEIN PF0899	
9:00	7azn-A	2.5	4.6	68	177	3	MOLECULE:	PROTEIN	ASTER-	C	
10:00	5mqr-A	2.5	3.8	64	1082	11	MOLECULE:	BETA-L-	ARABINO	BIOSIDASE	
11:00	2ich-B	2.5	4	63	323	3	MOLECULE:	PUTATIV	E ATTH		
12:00	7zb1-B	2.5	2.8	51	725	4	MOLECULE:	PROLYL	ENDOPEP	TIDASE	
13:00	8hhv-A	2.5	3.5	62	479	6	MOLECULE:	ENDO-AL	PHA-D-A	RABINANASE	
14:00	3dzm-A	2.5	5.1	62	208	5	MOLECULE:	HYPOTHE	TICAL C	ONSERVED PROTEIN	
15:00	8ovo-A	2.5	4.6	58	504	5	MOLECULE:	PUTATIV	E PERIP	LASMIC BINDING TRANSPORT PROTEIN,GR	
16:00	4gk9-A	2.5	3.5	50	279	4	MOLECULE:	AGGLUTI	NIN (BO	A)	
17:00	2xe4-A	2.5	3.6	63	721	13	MOLECULE:	OLIGOPE	PTIDASE	B	
18:00	6urg-A	2.5	3.4	61	1202	7	MOLECULE:	CLEAVAG	E AND P	OLYADENYLATION SPECIFICITY FACTOR S	
19:00	1sli-A	2.5	4.2	71	679	11	MOLECULE:	INTRAMO	LECULAR	TRANS-SIALIDASE	
20:00	1no3-A	2.5	3.8	75	851	5	MOLECULE:	LIPOXYG	ENASE-3		
21:00	8tie-a	2.5	3.5	65	1012	8	MOLECULE:	NUCLEOP	ORIN NU	P120	
22:00	4wy9-A	2.5	3.3	60	285	7	MOLECULE:	PUTATIV	E MCP-T	YPE SIGNAL TRANSDUCTION PROTEIN	
23:00	7kra-A	2.5	4.3	65	694	8	MOLECULE:	ER MEMB	RANE PR	OTEIN COMPLEX SUBUNIT 1	
24:00:00	7qe7-A	2.5	3.9	63	1648	8	MOLECULE:	ANAPHAS	E-PROMO	TING COMPLEX SUBUNIT 10	
25:00:00	6h6g-A	2.5	2.7	56	2139	9	MOLECULE:	TCDB2,T	CCC3		
26:00:00	5l8s-A	2.5	3.5	55	604	5	MOLECULE:	AMINO A	CYL PEP	TIDASE	
27:00:00	5mmj-f	2.5	3.2	50	113	6	MOLECULE:	50S RIB	OSOMAL	PROTEIN L31	
28:00:00	8asw-A	2.5	3.3	64	1255	11	MOLECULE:	ELONGAT	OR COMP	LEX PROTEIN 1	
29:00:00	4h5j-B	2.5	3.3	57	347	11	MOLECULE:	GUANINE	NUCLEO	TIDE-EXCHANGE FACTOR SEC12	
30:00:00	5f75-C	2.5	3.6	61	475	11	MOLECULE:	THIOCYA	NATE DE	HYDROGENASE	
31:00:00	5uam-A	2.5	3.9	61	440	5	MOLECULE:	ULVAN L	YASE-PL		25
32:00:00	3n7n-B	2.5	3.3	51	164	8	MOLECULE:	MONOPOL	IN COMP	LEX SUBUNIT CSM1	
33:00:00	7zkh-A	2.5	6.4	51	267	6	MOLECULE:	METHYLT	RANSFER	ASE	
34:00:00	5ien-A	2.5	3.7	63	129	6	MOLECULE:	D30H_F1	4		
35:00:00	6kyb-B	2.5	3.1	60	330	3	MOLECULE:	AUTOPHA	GY-RELA	TED PROTEIN 18	
36:00:00	4x49-A	2.5	3	65	489	9	MOLECULE:	ANHYDRO	SIALIDA	SE	
37:00:00	7yth-A	2.5	3.1	65	314	8	MOLECULE:	KETOSTE	ROID IS	OMERASE-RELATED PROTEIN	
38:00:00	8x2u-J	2.5	3.9	69	392	9	MOLECULE:	DPY30 D	OMAIN C	ONTAINING 2	
39:00:00	4fov-A	2.5	3.2	63	658	5	MOLECULE:	SIALIDA	SE B		
40:00:00	4bl9-A	2.5	4.8	78	722	6	MOLECULE:	MALTOSE	#NAME?	G PERIPLASMIC PROTEIN, SUPPRESSOR O	
41:00:00	7brs-D	2.5	3.2	73	1015	3	MOLECULE:	BETA-GA	LACTOSI	DASE	
42:00:00	4yz5-A	2.5	3.3	56	656	2	MOLECULE:	PUTATIV	E NEURA	MINIDASE	
43:00:00	6s5l-F	2.5	3.6	64	129	11	MOLECULE:	ALL4940	PROTEI	N	
44:00:00	4iia-A	2.5	3.5	68	131	4	MOLECULE:	RAS GTP	ASE-ACT	IVATING PROTEIN-BINDING PROTEIN 1	
45:00:00	2sli-A	2.5	3.5	62	679	6	MOLECULE:	INTRAMO	LECULAR	TRANS-SIALIDASE	
46:00:00	1qal-B	2.5	3.5	58	721	9	MOLECULE:	COPPER	AMINE O	XIDASE	
47:00:00	4yw2-B	2.5	3.3	56	659	2	MOLECULE:	NEURAMI	NIDASE	C	
48:00:00	4yz2-A	2.5	3.3	57	655	2	MOLECULE:	SIALIDA	SE NANC		

49:00:00	1ms0-B	2.5	3.2	62	623	6	MOLECULE:	TRANS-S	IALIDAS	E
50:00:00	1jz7-D	2.5	3.6	69	1011	7	MOLECULE:	BETA-GA	LACTOSI	DASE
51:00:00	4xjw-A	2.5	3.6	66	658	6	MOLECULE:	SIALIDA	SE B	
52:00:00	4yw5-B	2.5	3.2	58	659	2	MOLECULE:	NEURAMI	NIDASE	C
53:00:00	3dec-A	2.5	3.7	59	983	5	MOLECULE:	BETA-GA	LACTOSI	DASE
54:00:00	4yz1-B	2.5	3.3	57	656	2	MOLECULE:	PUTATIV	E NEURA	MINIDASE
55:00:00	4xe9-A	2.5	3.6	65	658	6	MOLECULE:	SIALIDA	SE B	
56:00:00	4ovm-G	2.5	2.8	64	122	9	MOLECULE:	UNCHARA	CTERIZE	D PROTEIN SGCJ
57:00:00	1sll-A	2.5	3.8	62	679	5	MOLECULE:	SIALIDA	SE L	
58:00:00	5tsp-A	2.5	4	68	448	9	MOLECULE:	SIALIDA	SE	
59:00:00	1wcq-B	2.5	3.5	68	601	4	MOLECULE:	SIALIDA	SE	
60:00:00	1eus-A	2.5	4	70	358	4	MOLECULE:	SIALIDA	SE	
61:00:00	7lbu-A	2.5	3.9	69	380	4	MOLECULE:	EXO-ALP	HA-SIAL	IDASE
62:00:00	2bzd-A	2.5	3.8	68	601	13	MOLECULE:	BACTERI	AL SIAL	IDASE
63:00:00	7a54-B	2.5	3.3	57	472	7	MOLECULE:	SIALIDA	SE A	
64:00:00	7bgh-A	2.4	6	63	177	5	MOLECULE:	OUTER E	NVELOPE	PORE PROTEIN 21, CHLOROPLASTIC
65:00:00	6rw6-B	2.4	3.4	65	2485	6	MOLECULE:	TCDA1		
66:00:00	4p07-A	2.4	3.7	69	569	4	MOLECULE:	ARYLSUL	FATE SU	LFOTRANSFERASE ASST
67:00:00	7k5c-A	2.4	3.2	52	650	8	MOLECULE:	INTERNA	L VIRIO	N PROTEIN GP15
68:00:00	7fde-P	2.4	4.2	72	181	10	MOLECULE:	V-TYPE	PROTON	ATPASE SUBUNIT C
69:00:00	6gcd-A	2.4	4.6	63	159	5	MOLECULE:	5-METHY	LCYTOSI	NE-SPECIFIC RESTRICTION ENZYME B
70:00:00	8ok9-C	2.4	3.8	71	236	6	MOLECULE:	PIWI PR	OTEIN A	F_1318
71:00:00	1twu-A	2.4	2.8	50	137	10	MOLECULE:	HYPOTHE	TICAL P	ROTEIN YYCE
72:00:00	4x4r-A	2.4	4.7	67	443	4	MOLECULE:	CCA-ADD	ING ENZ	YME
73:00:00	2ooj-B	2.4	4	58	134	5	MOLECULE:	HYPOTHE	TICAL P	ROTEIN
74:00:00	8t0b-A	2.4	4.7	62	121	10	MOLECULE:	DUF1842	DOMAIN	-CONTAINING PROTEIN
75:00:00	8c47-B	2.4	3.1	52	151	4	MOLECULE:	PROFILI	N	
76:00:00	8h8b-A	2.4	3.4	57	1027	4	MOLECULE:	PUTATIV	E RHS-F	AMILY PROTEIN
77:00:00	5oa3-0	2.4	2.9	51	479	10	MOLECULE:	EUKARYO	TIC TRA	NSLATION INITIATION FACTOR 2D
78:00:00	8t2v-B	2.4	3.6	63	718	3	MOLECULE:	INTEGRI	N ALPHA	-IIB
79:00:00	5mwv-A	2.4	4.7	43	281	14	MOLECULE:	OUTER M	EMBRANE	PROTEIN G
80:00:00	6itw-A	2.4	3.5	51	221	2	MOLECULE:	TYPE VI	IMMUNI	TY PROTEIN ATU4351
81:00:00	7px8-A	2.4	3.6	50	700	8	MOLECULE:	ACYLAMI	NO-ACID	-RELEASING ENZYME
82:00:00	1lsh-B	2.4	3.1	50	174	6	MOLECULE:	LIPOVIT	ELLIN (	LV-1N, LV-1C)
83:00:00	6rfl-E	2.4	3.5	67	184	4	MOLECULE:	DNA-DEP	ENDENT	RNA POLYMERASE SUBUNIT RPO132
84:00:00	7wb4-c	2.4	3.1	59	346	5	MOLECULE:	OUTER N	UP133	
85:00:00	6v04-A	2.4	6.5	53	268	6	MOLECULE:	UNCHARA	CTERIZE	D SRPBCC DOMAIN-CONTAINING PROTEIN
86:00:00	7vcf-I	2.4	5	61	363	7	MOLECULE:	TIC214		
87:00:00	4ffg-A	2.4	3	63	480	5	MOLECULE:	LEVAN F	RUCTOTR	ANSFERASE
88:00:00	3j1w-A	2.4	3.1	52	106	12	MOLECULE:	PROTEIN	PRGH	
89:00:00	6kme-A	2.4	5.1	61	284	8	MOLECULE:	PHYTOCH	ROMOBIL	IN SYNTHASE
90:00:00	8feh-D	2.4	5.9	59	268	7	MOLECULE:	MINOR C	APSID P	ROTEIN A1 FUSION
91:00:00	5jen-A	2.4	3.4	62	210	8	MOLECULE:	ANTI-SI	GMA-V F	ACTOR RSIV
92:00:00	5o8o-A	2.4	4.5	66	332	6	MOLECULE:	MITOCHO	NDRIAL	IMPORT RECEPTOR SUBUNIT TOM40
93:00:00	2rjz-A	2.4	5.1	62	135	15	MOLECULE:	PILO PR	OTEIN	
94:00:00	3c6k-D	2.4	3.6	59	348	3	MOLECULE:	SPERMIN	E SYNTH	ASE
95:00:00	4hh4-C	2.4	3.3	48	257	4	MOLECULE:	CCBJ		
96:00:00	1mg2-A	2.4	3.2	61	382	8	MOLECULE:	METHYLA	MINE DE	HYDROGENASE, HEAVY CHAIN
97:00:00	8orn-B	2.4	3.4	54	180	11	MOLECULE:	OUTER-M	EMBRANE	LIPOPROTEIN CARRIER PROTEIN
98:00:00	6cd2-C	2.4	7.1	62	727	2	MOLECULE:	CHAPERO	NE PROT	EIN PAPD
99:00:00	8wkl-G	2.4	3.2	68	307	9	MOLECULE:	STRICTO	SIDINE	SYNTHASE
0:00	3lif-A	2.4	3.2	56	243	13	MOLECULE:	PUTATIV	E DIGUA	NYLATE CYCLASE (GGDEF) WITH PAS/PAC
1:00	8fo9-F	2.4	3.6	50	2289	12	MOLECULE:	LEUCINE	-RICH R	EPEAT SERINE/THREONINE-PROTEIN KINA
2:00	8eti-A	2.4	4.9	62	254	5	MOLECULE:	RNA (15	64-MER)	

3:00	5img-A	2.4	3.2	57	467	7	MOLECULE:	DIPEPTI	DASE	
4:00	7ty0-C	2.4	3.6	66	488	8	MOLECULE:	GLYCOPR	OTEIN G	
5:00	4dvy-P	2.4	4.5	51	656	4	MOLECULE:	CYTOTOX	ICITY-A	SSOCIATED IMMUNODOMINANT ANTIGEN
6:00	8xpg-B	2.4	3.4	62	224	8	MOLECULE:	SERINE/	THREONI	NE-PROTEIN KINASE PLK4
7:00	7eeb-G	2.4	3	56	714	7	MOLECULE:	ENHANCE	D GREEN	FLUORESCENT PROTEIN,CATION CHANNEL
8:00	1jnw-A	2.4	4.3	67	214	4	MOLECULE:	PYRIDOX	INE 5'-	PHOSPHATE OXIDASE
9:00	2i51-A	2.4	4.3	65	194	8	MOLECULE:	UNCHARA	CTERIZE	D CONSERVED PROTEIN OF COG5135
10:00	3qqz-A	2.4	2.9	57	243	4	MOLECULE:	PUTATIV	E UNCHA	RACTERIZED PROTEIN YJIK
11:00	5m11-A	2.4	3.2	60	748	10	MOLECULE:	IMMUNOR	EACTIVE	84KD ANTIGEN PG93
12:00	8u1o-o	2.4	49.6	74	663	9	MOLECULE:	TAIL SP	IKE PRO	TEIN
13:00	7v2w-H	2.4	3.3	63	379	8	MOLECULE:	THO COM	PLEX SU	BUNIT HPR1
14:00	4uw8-A	2.4	4.5	60	401	5	MOLECULE:	L-SHAPE	D TAIL	FIBER PROTEIN
15:00	8ptx-B	2.4	3.3	58	800	7	MOLECULE:	ELONGAT	OR COMP	LEX PROTEIN 1
16:00	8glo-A	2.4	4.2	57	255	11	MOLECULE:	HEMOPHI	LIN	
17:00	7dl2-A	2.4	4.7	63	1127	6	MOLECULE:	HAMARTI	N	
18:00	8chv-B	2.4	2.8	47	147	6	MOLECULE:	TRANSCR	IPTIONA	L ACTIVATOR PROTEIN PUR-ALPHA
19:00	4u8u-M	2.4	3	57	222	0	MOLECULE:	GLOBIN	A CHAIN	
20:00	3w7s-A	2.4	3.8	60	760	8	MOLECULE:	UNCHARA	CTERIZE	D PROTEIN YGJK
21:00	8a3t-B	2.4	2.8	52	440	10	MOLECULE:	ANAPHAS	E-PROMO	TING COMPLEX SUBUNIT CDC27
22:00	8kg3-A	2.4	3.5	63	311	5	MOLECULE:	OS06G06	23700 P	ROTEIN
23:00	4fhn-B	2.4	3.3	62	1022	8	MOLECULE:	NUCLEOP	ORIN NU	P37
24:00:00	5yyl-A	2.4	4	63	405	10	MOLECULE:	MAJOR R	OYAL JE	LLY PROTEIN 1
25:00:00	4d4o-A	2.4	4.1	56	414	7	MOLECULE:	PROTEIN	ATS1,	DIPHTHAMIDE BIOSYNTHESIS PROTEIN 3
26:00:00	4ui9-A	2.4	3.1	58	1441	9	MOLECULE:	ANAPHAS	E-PROMO	TING COMPLEX SUBUNIT 1
27:00:00	6s5l-A	2.4	3.2	59	125	8	MOLECULE:	ALL4940	PROTEI	N
28:00:00	3aaa-A	2.4	3.5	74	267	5	MOLECULE:	F-ACTIN	#NAME?	G PROTEIN SUBUNIT ALPHA-1
29:00:00	6mrx-A	2.4	3.9	62	522	5	MOLECULE:	SIALIDA	SE26	
30:00:00	3lk4-4	2.4	3.3	73	269	5	MOLECULE:	F-ACTIN	#NAME?	G PROTEIN SUBUNIT ALPHA-1
31:00:00	3lk2-A	2.4	3.5	74	270	5	MOLECULE:	F-ACTIN	#NAME?	G PROTEIN SUBUNIT ALPHA-1
32:00:00	4ovm-I	2.4	3.1	64	120	11	MOLECULE:	UNCHARA	CTERIZE	D PROTEIN SGCJ
33:00:00	4poi-B	2.4	3.3	57	120	4	MOLECULE:	PUTATIV	E PERIP	LASMIC PROTEIN
34:00:00	3opz-A	2.4	2.8	60	625	5	MOLECULE:	TRANS-S	IALIDAS	E
35:00:00	8x2u-R	2.4	4.3	66	392	5	MOLECULE:	DPY30 D	OMAIN C	ONTAINING 2
36:00:00	6d24-D	2.4	4.5	70	494	4	MOLECULE:	GLUCOSE	#NAME?	PHATE 1-DEHYDROGENASE
37:00:00	4yz3-A	2.4	3.2	56	654	2	MOLECULE:	NEURAMI	NIDASE	
38:00:00	6q0w-B	2.4	3.7	66	208	6	MOLECULE:	DNA DAM	AGE-BIN	DING PROTEIN 1
39:00:00	4yw1-B	2.4	3.2	56	659	2	MOLECULE:	NEURAMI	NIDASE	C
40:00:00	6sd0-D	2.4	2.7	50	1083	10	MOLECULE:	BETA-GA	LACTOSI	DASE
41:00:00	1ms5-A	2.4	3.8	69	623	9	MOLECULE:	TRANS-S	IALIDAS	E
42:00:00	5ts4-D	2.4	2.9	58	89	7	MOLECULE:	DENOVO	NTF2	
43:00:00	4yw1-A	2.4	3.3	59	659	2	MOLECULE:	NEURAMI	NIDASE	C
44:00:00	4yz3-B	2.4	2.8	53	656	2	MOLECULE:	NEURAMI	NIDASE	
45:00:00	3b69-A	2.4	3.6	65	626	8	MOLECULE:	TRANS-S	IALIDAS	E
46:00:00	4poi-A	2.4	3.4	57	116	4	MOLECULE:	PUTATIV	E PERIP	LASMIC PROTEIN
47:00:00	4yw3-B	2.4	3.3	56	659	2	MOLECULE:	NEURAMI	NIDASE	C
48:00:00	3sli-A	2.4	3.3	61	679	8	MOLECULE:	INTRAMO	LECULAR	TRANS-SIALIDASE
49:00:00	3opz-B	2.4	3.2	65	625	8	MOLECULE:	TRANS-S	IALIDAS	E
50:00:00	7brs-B	2.4	3.6	69	1012	7	MOLECULE:	BETA-GA	LACTOSI	DASE
51:00:00	2lf2-A	2.3	4	72	175	3	MOLECULE:	UNCHARA	CTERIZE	D PROTEIN
52:00:00	8d8l-C	2.3	3.1	56	240	4	MOLECULE:	PROBABL	E S-ADE	NOSYL-L-METHIONINE-DEPENDENT RNA
53:00:00	7c8z-B	2.3	3.6	70	161	11	MOLECULE:	SALICYL	ATE 5-H	YDROXYLASE, LARGE OXYGENASE COMPONE
54:00:00	6am0-F	2.3	3.6	62	177	3	MOLECULE:	KLLA0F2	3980P	
55:00:00	7ofn-A	2.3	4.2	68	163	3	MOLECULE:	LIPOPRO	TEIN	
56:00:00	2kd2-A	2.3	3.3	56	94	5	MOLECULE:	FAS APO	PTOTIC	INHIBITORY MOLECULE 1

57:00:00	1ojg-A	2.3	3.2	61	135	10	MOLECULE:	SENSOR	PROTEIN	DCUS
58:00:00	2y2l-B	2.3	3.5	57	476	4	MOLECULE:	PENICIL	LIN-BIN	DING PROTEIN 1B
59:00:00	8x6f-C	2.3	3.8	63	1153	11	MOLECULE:	DNA-DIR	ECTED R	NA POLYMERASE SUBUNIT ALPHA
60:00:00	7mp9-A	2.3	4.9	65	414	5	MOLECULE:	SERINE/	THREONI	NE-PROTEIN KINASE PINK1, MITOCHONDR
61:00:00	5dyv-A	2.3	4.1	57	135	5	MOLECULE:	YD REPE	AT-CONT	AINING PROTEIN
62:00:00	3o2u-B	2.3	4	71	185	1	MOLECULE:	NEDD8-C	ONJUGAT	ING ENZYME UBC12
63:00:00	5x9j-B	2.3	4.1	68	152	9	MOLECULE:	PRHC		
64:00:00	3nqn-A	2.3	2.9	60	151	2	MOLECULE:	UNCHARA	CTERIZE	D PROTEIN
65:00:00	3c7g-A	2.3	3.4	59	488	5	MOLECULE:	ENDO-1,	4-BETA-	XYLANASE
66:00:00	6o38-L	2.3	4.3	63	130	2	MOLECULE:	ACINETO	BACTER	SECRETED PROTEASE CPAA
67:00:00	6r82-A	2.3	4.4	73	181	3	MOLECULE:	GTPASE-	ACTIVAT	ING PROTEIN SKYWALKER
68:00:00	6ipv-A	2.3	3.4	63	221	10	MOLECULE:	CQSB2		
69:00:00	4rny-A	2.3	3.4	60	359	3	MOLECULE:	CONSERV	ED HYPO	THETICAL SECRETED PROTEIN
70:00:00	3kk7-A	2.3	3.2	63	502	5	MOLECULE:	PUTATIV	E CELL	INVASION PROTEIN WITH MAC/PERFORIN
71:00:00	6jql-A	2.3	3.2	58	678	3	MOLECULE:	BIFUNCT	IONAL P	ROTEIN PAAZ
72:00:00	4ped-A	2.3	3.7	72	387	1	MOLECULE:	CHAPERO	NE ACTI	VITY OF BC1 COMPLEX-LIKE, MITOCHOND
73:00:00	7pd7-B	2.3	9.3	57	251	9	MOLECULE:	METHYLT	RANSFER	ASE
74:00:00	6ns4-A	2.3	4.9	68	685	4	MOLECULE:	LIPOXYG	ENASE	
75:00:00	7p2p-C	2.3	3.6	61	174	5	MOLECULE:	SIGNAL	PEPTIDA	SE COMPLEX CATALYTIC SUBUNIT SEC11A
76:00:00	5yk1-A	2.3	3.9	66	401	5	MOLECULE:	PROBABL	E CONSE	RVED ATP-BINDING PROTEIN ABC TRANSP
77:00:00	8ajm-B	2.3	3.7	56	395	11	MOLECULE:	DNA DAM	AGE-BIN	DING PROTEIN 1
78:00:00	7eu3-7	2.3	3.5	69	317	9	MOLECULE:	NAD(P)H	#NAME?	E OXIDOREDUCTASE SUBUNIT 1, CHLOROP
79:00:00	2o5p-A	2.3	4.1	66	772	6	MOLECULE:	FERRIPY	OVERDIN	E RECEPTOR
80:00:00	7c3c-B	2.3	3	58	346	7	MOLECULE:	AOFLEA		
81:00:00	3zwl-D	2.3	3.1	52	352	13	MOLECULE:	EUKARYO	TIC TRA	NSLATION INITIATION FACTOR 3 SUBUNI
82:00:00	7wb4-E	2.3	3.8	71	1363	11	MOLECULE:	OUTER N	UP133	
83:00:00	2gia-G	2.3	3.7	52	155	13	MOLECULE:	MITOCHO	NDRIAL	RNA-BINDING PROTEIN 2
84:00:00	2joz-A	2.3	3.5	56	135	4	MOLECULE:	HYPOTHE	TICAL P	ROTEIN YXEF
85:00:00	1k32-A	2.3	3.1	55	1023	7	MOLECULE:	TRICORN	PROTEA	SE
86:00:00	7oc9-A	2.3	3.3	60	133	12	MOLECULE:	BD0675		
87:00:00	7r5z-B	2.3	4.3	62	154	5	MOLECULE:	DIRIGEN	T PROTE	IN
88:00:00	4fnv-A	2.3	3.8	69	659	9	MOLECULE:	HEPARIN	ASE III	PROTEIN, HEPARITIN SULFATE LYASE
89:00:00	6hzn-A	2.3	3.3	59	742	7	MOLECULE:	DERMATA	N-SULFA	TE EPIMERASE
90:00:00	2qml-A	2.3	4.9	47	193	11	MOLECULE:	BH2621	PROTEIN	
91:00:00	3hi2-B	2.3	3.2	62	97	2	MOLECULE:	HTH-TYP	E TRANS	CRIPTIONAL REGULATOR MQSA(YGIT)
92:00:00	4asc-A	2.3	3	48	308	4	MOLECULE:	KELCH R	EPEAT A	ND BTB DOMAIN-CONTAINING PROTEIN 5
93:00:00	8a3t-Q	2.3	3.6	60	623	7	MOLECULE:	ANAPHAS	E-PROMO	TING COMPLEX SUBUNIT CDC27
94:00:00	5h68-B	2.3	3.6	61	332	11	MOLECULE:	CHROMOS	OME PAR	TITION PROTEIN SMC
95:00:00	7dtr-A	2.3	2.5	46	163	11	MOLECULE:	ACRIF24		
96:00:00	6y86-A	2.3	3.6	48	425	6	MOLECULE:	MEMBRAN	E PROTE	IN INSERTASE YIDC
97:00:00	1qjs-A	2.3	2.5	43	408	2	MOLECULE:	HEMOPEX	IN	
98:00:00	8giu-A	2.3	2.8	48	381	10	MOLECULE:	GP_4 (C	APSID A	CCESSORY PROTEIN)
99:00:00	3af5-A	2.3	10.3	65	638	5	MOLECULE:	PUTATIV	E UNCHA	RACTERIZED PROTEIN PH1404
0:00	5hl8-A	2.3	3.6	52	81	12	MOLECULE:	TYPE II	SECRET	ION SYSTEM PROTEIN L
1:00	8beq-A	2.3	3.3	61	534	7	MOLECULE:	FRUCTOF	URANOSI	DASE FROM RHODOTORULA DAIRENENSIS
2:00	6lnh-C	2.3	3.4	60	228	7	MOLECULE:	L-ISOLE	UCINE-4	-HYDROXYLASE
3:00	2crf-A	2.3	3.4	59	150	5	MOLECULE:	RAN BIN	DING PR	OTEIN 3
4:00	2pff-B	2.3	7.1	50	2006	0	MOLECULE:	FATTY A	CID SYN	THASE SUBUNIT ALPHA
5:00	7aak-A	2.3	3	59	345	5	MOLECULE:	PORPHOB	ILINOGE	N DEAMINASE
6:00	4doo-A	2.3	3.1	64	203	2	MOLECULE:	CHALCON	E-FLAVA	NONE ISOMERASE FAMILY PROTEIN
7:00	6zrd-A	2.3	2.9	51	411	10	MOLECULE:	HISTONE	#NAME?	G PROTEIN RBBP4
8:00	8tar-R	2.3	3.8	55	440	5	MOLECULE:	ANAPHAS	E-PROMO	TING COMPLEX SUBUNIT 1
9:00	8wqr-B	2.3	4.8	55	356	11	MOLECULE:	ACTIVAT	ING MOL	ECULE IN BECN1-REGULATED AUTOPHAGY
10:00	3dsb-A	2.3	4.5	62	153	5	MOLECULE:	PUTATIV	E ACETY	LTRANSFERASE

11:00	6n2g-A	2.3	4.6	64	285	5	MOLECULE:	NUCLEOS	OME ASS	EMBLY PROTEIN
12:00	6dzk-Y	2.3	3.2	57	103	4	MOLECULE:	16S RRN	A	
13:00	8j0n-A	2.3	3.7	62	949	3	MOLECULE:	ER MEMB	RANE PR	OTEIN COMPLEX SUBUNIT 1
14:00	8ond-A	2.3	4.6	55	344	5	MOLECULE:	BD2133		
15:00	6acx-A	2.3	3	52	1175	8	MOLECULE:	MYCOBAC	TERIUM	SMEGMATIS MFD
16:00	5yvq-A	2.3	7.3	54	358	2	MOLECULE:	TAIL FI	BER PRO	TEIN S
17:00	8bys-A	2.3	3.7	59	323	3	MOLECULE:	S-LAYER	HOMOLO	GY DOMAIN-CONTAINING PROTEIN
18:00	6dn4-A	2.3	10.1	60	275	7	MOLECULE:	BETA-LA	CTAMASE	
19:00	3e5d-A	2.3	4	50	126	6	MOLECULE:	PUTATIV	E GLYOX	ALASE I
20:00	6zym-p	2.3	3.9	62	325	5	MOLECULE:	U2 SNRN	A	
21:00	7mge-A	2.3	2.9	52	349	8	MOLECULE:	WD REPE	AT-CONT	AINING PROTEIN 41
22:00	8q66-A	2.3	3.8	60	271	3	MOLECULE:	EXONUCL	EASE MU	T-7
23:00	4aip-B	2.3	8.1	65	665	12	MOLECULE:	FE-REGU	LATED P	ROTEIN B
24:00:00	2hhi-A	2.3	3.1	67	204	6	MOLECULE:	IMMUNOG	ENIC PR	OTEIN MPT64
25:00:00	8f0v-A	2.3	3.9	57	155	5	MOLECULE:	MILK PR	OTEIN	
26:00:00	6vs7-A	2.3	2.9	48	409	2	MOLECULE:	ADHESIN		
27:00:00	7t3j-J	2.3	2.5	46	228	11	MOLECULE:	CRISPR-	ASSOCIA	TED PROTEIN CSY1
28:00:00	8chw-A	2.3	2.9	44	67	7	MOLECULE:	TRANSCR	IPTIONA	L ACTIVATOR PROTEIN PUR-ALPHA
29:00:00	4ktp-A	2.3	4.5	60	767	7	MOLECULE:	GLYCOSI	DE HYDR	OLASE FAMILY 65 CENTRAL CATALYTIC
30:00:00	8wo8-A	2.3	3.7	68	463	3	MOLECULE:	PROBABL	E RIBON	UCLEASE FAU-1
31:00:00	4e54-B	2.3	2.9	51	402	14	MOLECULE:	DNA DAM	AGE-BIN	DING PROTEIN 1
32:00:00	7svm-A	2.3	3.4	51	852	10	MOLECULE:	DIPEPTI	DYL PEP	TIDASE 8
33:00:00	1fj1-E	2.3	5.5	58	251	3	MOLECULE:	HYBRIDO	MA ANTI	BODY LA2 (LIGHT CHAIN)
34:00:00	3aae-E	2.3	3.5	75	270	5	MOLECULE:	F-ACTIN	#NAME?	G PROTEIN SUBUNIT ALPHA-1
35:00:00	4x47-A	2.3	3.4	69	489	7	MOLECULE:	ANHYDRO	SIALIDA	SE
36:00:00	4dsd-B	2.3	4	58	126	10	MOLECULE:	PUTATIV	E PERIP	LASMIC PROTEIN
37:00:00	6q0r-B	2.3	3.3	68	207	9	MOLECULE:	DNA DAM	AGE-BIN	DING PROTEIN 1
38:00:00	4yw3-A	2.3	3.4	60	659	2	MOLECULE:	NEURAMI	NIDASE	C
39:00:00	6drv-A	2.3	2.9	62	1024	5	MOLECULE:	BETA-GA	LACTOSI	DASE
40:00:00	7e8h-J	2.3	3	53	756	0	MOLECULE:	DIPEPTI	DYL AMI	NOPEPTIDASE-LIKE PROTEIN 6
41:00:00	1dil-A	2.3	4.1	70	381	6	MOLECULE:	SIALIDA	SE	
42:00:00	3oba-C	2.3	3.6	73	1024	7	MOLECULE:	BETA-GA	LACTOSI	DASE
43:00:00	2vvz-B	2.3	4.1	73	470	5	MOLECULE:	SIALIDA	SE A	
44:00:00	2ya8-B	2.3	3.2	57	470	7	MOLECULE:	NEURAMI	NIDASE	A
45:00:00	2ya5-B	2.3	3.2	57	470	7	MOLECULE:	NEURAMI	NIDASE	A
46:00:00	5kbp-A	2.3	3.5	72	886	6	MOLECULE:	GLYCOSY	L HYDRO	LASE, FAMILY 38
47:00:00	4foy-A	2.3	2.9	55	658	0	MOLECULE:	SIALIDA	SE B	
48:00:00	8gn6-C	2.3	3.5	62	494	10	MOLECULE:	SIALIDA	SE	
49:00:00	8gn6-B	2.3	3.6	64	496	11	MOLECULE:	SIALIDA	SE	
50:00:00	4fj6-C	2.3	3	69	519	10	MOLECULE:	GLYCOSI	DE HYDR	OLASE FAMILY 33, CANDIDATE SIALIDAS
51:00:00	4xog-A	2.3	3.6	63	660	6	MOLECULE:	SIALIDA	SE B	
52:00:00	4q6k-D	2.3	3.3	71	523	11	MOLECULE:	BNR/ASP	#NAME?	PEAT PROTEIN
53:00:00	5vg5-W	2.3	4.4	64	486	5	MOLECULE:	GLUCOSE	#NAME?	PHATE 1-DEHYDROGENASE
54:00:00	5ohl-A	2.3	2.9	50	89	6	MOLECULE:	K6-SPEC	IFIC AF	FIMER
55:00:00	7ual-A	2.3	4.3	59	484	8	MOLECULE:	GLUCOSE	#NAME?	PHATE 1-DEHYDROGENASE
56:00:00	7a54-A	2.3	3.5	70	472	4	MOLECULE:	SIALIDA	SE A	
57:00:00	4qa8-A	2.2	3.8	73	210	10	MOLECULE:	PUTATIV	E LIPOP	ROTEIN LPRF
58:00:00	3cwf-A	2.2	3.4	56	108	9	MOLECULE:	ALKALIN	E PHOSP	HATASE SYNTHESIS SENSOR PROTEIN PHO
59:00:00	1n3g-A	2.2	3.7	60	113	5	MOLECULE:	PROTEIN	YFIA	
60:00:00	6pif-G	2.2	3.2	61	521	5	MOLECULE:	CAS7, T	YPE I-F	CRISPR-ASSOCIATED PROTEIN
61:00:00	7e9d-A	2.2	3.3	55	443	9	MOLECULE:	IRG1		
62:00:00	1tuh-A	2.2	3.4	63	131	16	MOLECULE:	HYPOTHE	TICAL P	ROTEIN EGC068
63:00:00	6hdv-A	2.2	4.1	54	136	6	MOLECULE:	AFIFAVI	DIN	
64:00:00	7pkq-f	2.2	4.2	57	103	7	MOLECULE:	MS35		

65:00:00	7t69-A	2.2	3.9	51	209	12	MOLECULE:	AVR3 (S	IX1), S	ECRETED IN XYLEM 1
66:00:00	7s00-c	2.2	3.3	60	484	5	MOLECULE:	DNA-DIR	ECTED R	NA POLYMERASE BETA SUBUNIT
67:00:00	6vqv-D	2.2	3.9	65	306	2	MOLECULE:	ACRF9		
68:00:00	8f3h-A	2.2	3.1	61	643	8	MOLECULE:	PENICIL	LIN BIN	DING PROTEIN 5
69:00:00	3fo5-A	2.2	3.4	71	239	10	MOLECULE:	THIOEST	ERASE,	ADIPOSE ASSOCIATED, ISOFORM BFIT2
70:00:00	5zi2-A	2.2	3.5	68	339	7	MOLECULE:	MALATE	DEHYDRO	GENASE
71:00:00	4i60-A	2.2	4.7	56	128	5	MOLECULE:	AVIDIN		
72:00:00	8ixw-A	2.2	3	52	168	10	MOLECULE:	CHAINS:	A,B	
73:00:00	1zwy-B	2.2	3.1	57	180	5	MOLECULE:	HYPOTHE	TICAL U	PF0244 PROTEIN VC0702
74:00:00	8a0h-A	2.2	3.2	63	324	5	MOLECULE:	OCP N-T	ERMINAL	DOMAIN-CONTAINING PROTEIN
75:00:00	8ttb-B	2.2	3.8	66	426	9	MOLECULE:	SERINE/	THREONI	NE-PROTEIN PHOSPHATASE 2A 65 KDA RE
76:00:00	6r1g-A	2.2	3.4	65	148	8	MOLECULE:	OUTER S	URFACE	22 KDA LIPOPROTEIN
77:00:00	8tl6-B	2.2	3.7	52	353	8	MOLECULE:	DNA DAM	AGE-BIN	DING PROTEIN 1
78:00:00	3c30-A	2.2	3.5	61	219	8	MOLECULE:	AUTOIND	UCER 2	SENSOR KINASE/PHOSPHATASE LUXQ
79:00:00	7vma-A	2.2	3.7	62	620	5	MOLECULE:	AMYLO-A	LPHA-1,	6-GLUCOSIDASE, PUTATIVE ARCHAEAL TY
80:00:00	7czf-B	2.2	3	53	644	9	MOLECULE:	EPHRIN	TYPE-A	RECEPTOR 2
81:00:00	6rte-A	2.2	3.6	57	464	9	MOLECULE:	CYTOCHR	OME C	
82:00:00	5dky-A	2.2	3.4	63	928	0	MOLECULE:	ALPHA G	LUCOSID	ASE-LIKE PROTEIN
83:00:00	5n9j-A	2.2	4.7	64	566	5	MOLECULE:	MEDIATO	R OF RN	A POLYMERASE II TRANSCRIPTION SUBUN
84:00:00	1jc5-B	2.2	4.6	54	147	9	MOLECULE:	METHYLM	ALONYL-	COA EPIMERASE
85:00:00	3b77-A	2.2	3.4	52	188	13	MOLECULE:	UNCHARA	CTERIZE	D PROTEIN
86:00:00	7syf-A	2.2	5	63	1391	10	MOLECULE:	PHOSPHA	TIDYLIN	OSITOL 3,4,5-TRISPHOSPHATE-DEPENDEN
87:00:00	7kpr-A	2.2	4.8	65	403	8	MOLECULE:	PROTEIN	PHOSPH	ATASE 1H
88:00:00	2wjq-A	2.2	3.6	52	205	4	MOLECULE:	PROBABL	E N-ACE	TYLNEURAMINIC ACID OUTER MEMBRANE C
89:00:00	3v89-A	2.2	4.3	63	853	6	MOLECULE:	TRANSFE	RRIN-BI	NDING PROTEIN A
90:00:00	4hvt-A	2.2	3.4	61	691	10	MOLECULE:	POST-PR	OLINE C	LEAVING ENZYME
91:00:00	6dm4-A	2.2	3.4	60	119	2	MOLECULE:	RAVO		
92:00:00	7st9-H	2.2	4.1	53	316	4	MOLECULE:	CHECKPO	INT PRO	TEIN RAD24
93:00:00	5fq6-M	2.2	6.2	63	948	3	MOLECULE:	PUTATIV	E LIPOP	ROTEIN
94:00:00	7krw-A	2.2	2.9	52	608	2	MOLECULE:	CHAPERO	NE PROT	EIN DNAK FUSED WITH SUBSTRATE PEPTI
95:00:00	4jpd-A	2.2	4	60	109	8	MOLECULE:	PROTEIN	CYAY	
96:00:00	6z3b-A	2.2	3.8	62	371	10	MOLECULE:	GFO/IDH	/MOCA F	AMILY OXIDOREDUCTASE
97:00:00	5if6-A	2.2	4.2	60	134	10	MOLECULE:	OHP9_1C		
98:00:00	3wpv-A	2.2	3.7	70	538	6	MOLECULE:	BETA-FR	UCTOFUR	ANOSIDASE
99:00:00	5gzl-A	2.2	4.8	63	358	6	MOLECULE:	LYSINE	CYCLODE	AMINASE
0:00	6nd4-T	2.2	3.3	49	812	8	MOLECULE:	ETS RRN	A	
1:00	5a1u-E	2.2	3.2	54	822	11	MOLECULE:	ADP-RIB	OSYLATI	ON FACTOR 1
2:00	6abo-A	2.2	2.7	43	213	5	MOLECULE:	DNA REP	AIR PRO	TEIN XRCC4
3:00	5cvo-D	2.2	3.8	61	612	7	MOLECULE:	WD REPE	AT-CONT	AINING PROTEIN 48
4:00	8bmx-C	2.2	4	68	633	1	MOLECULE:	PUTATIV	E TONB-	LINKED OUTER MEMBRANE RECEPTOR
5:00	7z8b-F	2.2	3	57	457	2	MOLECULE:	CULLIN-	7	
6:00	8dgt-A	2.2	4.6	54	431	7	MOLECULE:	SERINE/	THREONI	NE-PROTEIN KINASE B-RAF
7:00	7zgr-A	2.2	3.6	74	1253	1	MOLECULE:	PROTEIN	CFT1	
8:00	7wkk-D	2.2	4.2	53	1115	2	MOLECULE:	MGC8329	5 PROTE	IN
9:00	1jmx-B	2.2	3.5	59	339	5	MOLECULE:	AMINE D	EHYDROG	ENASE
10:00	3u4y-A	2.2	2.9	52	319	12	MOLECULE:	UNCHARA	CTERIZE	D PROTEIN
11:00	6r2q-B	2.2	4.4	57	649	2	MOLECULE:	CYSTATH	IONINE	BETA-SYNTHASE
12:00	8s1p-V	2.2	3.3	54	249	6	MOLECULE:	50S RIB	OSOMAL	PROTEIN L35
13:00	8an0-A	2.2	3.4	54	282	7	MOLECULE:	PUTATIV	E SECRE	TED PROTEIN
14:00	3alx-B	2.2	5.5	69	526	7	MOLECULE:	HEMAGGL	UTININ,	LINKER,CDW150
15:00	7jrd-A	2.2	7.3	63	566	2	MOLECULE:	LACTOFE	RRIN-BI	NDING PROTEIN B
16:00	8c7g-A	2.2	3.8	65	607	14	MOLECULE:	MIC1 DO	MAIN-CO	NTAINING PROTEIN
17:00	7eeb-E	2.2	3.1	50	1053	4	MOLECULE:	ENHANCE	D GREEN	FLUORESCENT PROTEIN,CATION CHANNEL
18:00	4pdy-A	2.2	3.2	56	333	4	MOLECULE:	AMINOGL	YCOSIDE	PHOSPHOTRANSFERASE

19:00	2prv-A	2.2	4.9	52	153	4	MOLECULE:	UNCHARA	CTERIZE	D PROTEIN YOBK
20:00	7zet-A	2.2	3.2	60	377	8	MOLECULE:	CLUSTER	IN	
21:00	3c5m-B	2.2	3.8	66	378	11	MOLECULE:	OLIGOGA	LACTURO	NATE LYASE
22:00	7z4f-G	2.2	3.9	60	922	3	MOLECULE:	PUTATIV	E STRUC	TURAL PROTEIN
23:00	8h93-B	2.2	3	54	367	7	MOLECULE:	NACHT,	LRR AND	PYD DOMAINS-CONTAINING PROTEIN 5
24:00:00	2qkd-A	2.2	3.8	50	384	8	MOLECULE:	ZINC FI	NGER PR	OTEIN ZPR1
25:00:00	7np8-B	2.2	2.7	42	620	10	MOLECULE:	COENZYM	E F420-	DEPENDENT SULFITE REDUCTASE
26:00:00	8ptx-A	2.2	2.8	58	1215	7	MOLECULE:	ELONGAT	OR COMP	LEX PROTEIN 1
27:00:00	8ew9-A	2.2	2.9	62	241	2	MOLECULE:	ALTERED	INHERI	TANCE OF MITOCHONDRIA PROTEIN 46,
28:00:00	3oan-A	2.2	3	47	116	0	MOLECULE:	ABR034W	P	
29:00:00	6y9c-A	2.2	3.8	66	365	9	MOLECULE:	CARNITI	NE MONO	OXYGENASE OXYGENASE SUBUNIT
30:00:00	6imj-A	2.2	3.1	56	412	5	MOLECULE:	DNA LIG	ASE	
31:00:00	3wyb-A	2.2	3.9	53	327	6	MOLECULE:	MESO-DI	AMINOPI	MELATE D-DEHYDROGENASE
32:00:00	3ong-A	2.2	3.6	55	114	9	MOLECULE:	UBIQUIT	IN-ACTI	VATING ENZYME E1-LIKE
33:00:00	2l3a-A	2.2	4.1	48	82	6	MOLECULE:	UNCHARA	CTERIZE	D PROTEIN
34:00:00	8gxj-A	2.2	3.4	49	189	6	MOLECULE:	N-ACETY	LTRANSF	ERASE DOMAIN-CONTAINING PROTEIN
35:00:00	2mhg-A	2.2	4.6	50	75	8	MOLECULE:	UNCHARA	CTERIZE	D PROTEIN
36:00:00	1q9u-A	2.2	3.2	58	128	12	MOLECULE:	UNCHARA	CTERIZE	D PROTEIN APC35924
37:00:00	6wl5-A	2.2	3.5	52	158	6	MOLECULE:	ECMRR T	RANSCRI	PTIONAL REGULATOR
38:00:00	3a15-B	2.2	5.3	67	362	9	MOLECULE:	ALDOXIM	E DEHYD	RATASE
39:00:00	7uic-p	2.2	2.6	62	903	10	MOLECULE:	MEDIATO	R OF RN	A POLYMERASE II TRANSCRIPTION SUBUN
40:00:00	4zn4-B	2.2	3.4	57	411	7	MOLECULE:	SQT1		
41:00:00	7t6a-A	2.2	2.8	46	185	13	MOLECULE:	AVR1 (S	IX4), A	VIRULENCE PROTEIN 1
42:00:00	3was-A	2.2	3.6	59	389	5	MOLECULE:	4-O-BET	A-D-MAN	NOSYL-D-GLUCOSE PHOSPHORYLASE
43:00:00	3ty1-A	2.2	5	56	384	7	MOLECULE:	HYPOTHE	TICAL A	LDOSE 1-EPIMERASE
44:00:00	3hdp-A	2.2	3.7	49	132	6	MOLECULE:	GLYOXAL	ASE-I	
45:00:00	4wvm-B	2.2	4.8	51	616	2	MOLECULE:	STONUST	OXIN SU	BUNIT ALPHA
46:00:00	3d4e-A	2.2	2.9	54	162	4	MOLECULE:	PUTATIV	E BETA-	LACTAMASE INHIBITOR PROTEIN
47:00:00	5nxh-A	2.2	3.6	60	546	5	MOLECULE:	LONG-TA	IL FIBE	R PROXIMAL SUBUNIT
48:00:00	5tf2-A	2.2	3.3	57	338	7	MOLECULE:	PROLACT	IN REGU	LATORY ELEMENT-BINDING PROTEIN
49:00:00	6q50-A	2.2	3.7	63	318	6	MOLECULE:	MPT-4		
50:00:00	4ddt-A	2.2	4.2	56	1102	4	MOLECULE:	REVERSE	GYRASE	
51:00:00	8jwu-C	2.2	3.8	63	412	2	MOLECULE:	PHD FIN	GER PRO	TEIN 7,UBIQUITIN-CONJUGATING ENZYME
52:00:00	8tum-a	2.2	3.1	52	143	0	MOLECULE:	TYPE IV	MAJOR	PILIN PROTEIN PILA
53:00:00	6zls-A	2.2	3.7	60	292	12	MOLECULE:	HISTIDI	NE KINA	SE
54:00:00	4bla-A	2.2	4.4	69	730	3	MOLECULE:	MALTOSE	#NAME?	G PERIPLASMIC PROTEIN, SUPPRESSOR O
55:00:00	4bla-C	2.2	5.4	72	724	4	MOLECULE:	MALTOSE	#NAME?	G PERIPLASMIC PROTEIN, SUPPRESSOR O
56:00:00	4q6k-A	2.2	3.9	62	523	5	MOLECULE:	BNR/ASP	#NAME?	PEAT PROTEIN
57:00:00	6mnj-A	2.2	2.8	58	511	7	MOLECULE:	HZ136		
58:00:00	4dsd-A	2.2	3.8	58	126	12	MOLECULE:	PUTATIV	E PERIP	LASMIC PROTEIN
59:00:00	2ya6-B	2.2	3.8	70	470	3	MOLECULE:	NEURAMI	NIDASE	A
60:00:00	2ya7-D	2.2	4.1	72	470	4	MOLECULE:	NEURAMI	NIDASE	A
61:00:00	1ms4-B	2.2	3.1	65	623	8	MOLECULE:	TRANS-S	IALIDAS	E
62:00:00	1w8n-A	2.2	4	67	601	4	MOLECULE:	BACTERI	AL SIAL	IDASE
63:00:00	2ah2-A	2.2	3.2	65	624	8	MOLECULE:	TRANS-S	IALIDAS	E
64:00:00	4bl9-B	2.2	4.8	71	729	6	MOLECULE:	MALTOSE	#NAME?	G PERIPLASMIC PROTEIN, SUPPRESSOR O
65:00:00	7lbv-A	2.2	3.6	65	379	11	MOLECULE:	EXO-ALP	HA-SIAL	IDASE
66:00:00	8t1z-A	2.2	3.7	62	502	10	MOLECULE:	SIALIDA	SE	
67:00:00	3ob8-B	2.2	3.5	71	1024	7	MOLECULE:	BETA-GA	LACTOSI	DASE
68:00:00	5ihj-A	2.2	3.9	59	484	2	MOLECULE:	MALTOSE	#NAME?	G PERIPLASMIC PROTEIN,FIMBRIAL PROT
69:00:00	6d23-B	2.2	4.7	71	490	3	MOLECULE:	GLUCOSE	#NAME?	PHATE 1-DEHYDROGENASE
70:00:00	6mrx-D	2.2	3.3	60	521	7	MOLECULE:	SIALIDA	SE26	
71:00:00	5o6c-A	2.1	3.4	71	252	7	MOLECULE:	E3 UBIQ	UITIN-P	ROTEIN LIGASE MYCBP2
72:00:00	6s62-A	2.1	3.7	54	485	11	MOLECULE:	PROPION	ATE CAT	ABOLIC PROTEIN PRPD

73:00:00	6j6a-A	2.1	3.8	68	442	7	MOLECULE:	ZINC-DE	PENDENT	PROTEASE, TLDD/PMBA FAMILY
74:00:00	5cl2-A	2.1	3.8	74	246	7	MOLECULE:	SPORULA	TION-CO	NTROL PROTEIN SPO0M
75:00:00	4ebr-A	2.1	3.5	58	157	9	MOLECULE:	UBIQUIT	IN-LIKE	-CONJUGATING ENZYME ATG10
76:00:00	6t3x-A	2.1	4.1	65	212	5	MOLECULE:	NUCLEAR	EGRESS	PROTEIN 2,NUCLEAR EGRESS PROTEIN 1
77:00:00	4h5b-A	2.1	3.3	61	152	10	MOLECULE:	DR_1245	PROTEI	N
78:00:00	7mh2-B	2.1	2.7	52	279	13	MOLECULE:	T4GALA	ENGINEE	RED PROTEIN NANOCAGE
79:00:00	5xta-A	2.1	3.7	61	120	13	MOLECULE:	VIRK PR	OTEIN	
80:00:00	7e5c-A	2.1	4.4	72	439	7	MOLECULE:	XAA-PRO	DIPEPT	IDASE
81:00:00	6bqi-A	2.1	4.1	63	258	8	MOLECULE:	PROTEIN	IMPACT	HOMOLOG
82:00:00	3t8n-B	2.1	4.5	70	130	7	MOLECULE:	STEROID	DELTA-	ISOMERASE
83:00:00	8w7p-A	2.1	2.8	60	250	0	MOLECULE:	EXTRACE	LLULAR	DOMAIN OF A SENSOR HISTIDINE KINASE
84:00:00	8e16-A	2.1	5.4	53	272	9	MOLECULE:	MAJOR C	APSID P	ROTEIN, GP6
85:00:00	1x9y-A	2.1	3.5	73	346	4	MOLECULE:	CYSTEIN	E PROTE	INASE
86:00:00	3wqy-A	2.1	3.7	60	906	5	MOLECULE:	ALANINE	#NAME?	LIGASE
87:00:00	5npy-A	2.1	5.7	73	472	3	MOLECULE:	FLAGELL	AR BASA	L BODY PROTEIN
88:00:00	4tq2-A	2.1	3.5	64	177	2	MOLECULE:	PUTATIV	E PHYCO	ERYTHRIN LYASE
89:00:00	3nlc-A	2.1	2.7	48	534	8	MOLECULE:	UNCHARA	CTERIZE	D PROTEIN VP0956
90:00:00	1vjh-A	2.1	4.1	60	120	10	MOLECULE:	BET V I	ALLERG	EN FAMILY
91:00:00	2gjv-A	2.1	3.2	62	136	3	MOLECULE:	PUTATIV	E CYTOP	LASMIC PROTEIN
92:00:00	3uau-A	2.1	4	77	344	6	MOLECULE:	SURFACE	#NAME?	D LIPOPROTEIN
93:00:00	4jgo-B	2.1	3.5	61	210	2	MOLECULE:	SPORULA	TION KI	NASE D
94:00:00	4onw-A	2.1	4.7	66	265	6	MOLECULE:	SUCCINY	L-DIAMI	NOPIMELATE DESUCCINYLASE
95:00:00	4o4s-A	2.1	3.8	70	199	9	MOLECULE:	PHYCOCY	ANOBILI	N LYASE CPCT
96:00:00	4r9k-C	2.1	4.8	68	160	4	MOLECULE:	LIMONEN	E-1,2-E	POXIDE HYDROLASE
97:00:00	2i9i-A	2.1	3.8	65	221	8	MOLECULE:	HYPOTHE	TICAL P	ROTEIN
98:00:00	6xgq-A	2.1	4.5	55	353	7	MOLECULE:	YSD1_17		
99:00:00	7x80-A	2.1	3.5	56	148	7	MOLECULE:	PLOI4		
0:00	4xkz-A	2.1	3.7	50	251	12	MOLECULE:	VALINE-	TRNA LI	GASE
1:00	5mmj-c	2.1	3.4	59	216	7	MOLECULE:	50S RIB	OSOMAL	PROTEIN L31
2:00	5ux0-D	2.1	4.9	69	639	1	MOLECULE:	ARGONAU	TE PROT	EIN
3:00	4rzm-B	2.1	4.7	72	283	11	MOLECULE:	EPOXIDE	HYDROL	ASE LASB
4:00	8gmm-A	2.1	3.8	51	232	8	MOLECULE:	HEMOPHI	LIN	
5:00	2k6p-A	2.1	2.8	53	84	4	MOLECULE:	UNCHARA	CTERIZE	D PROTEIN HP_1423
6:00	2h9f-A	2.1	4.4	58	391	5	MOLECULE:	HYPOTHE	TICAL P	ROTEIN
7:00	7cqy-D	2.1	3.2	50	464	6	MOLECULE:	TETRATH	IONATE	HYDROLASE
8:00	7cud-A	2.1	3.6	56	116	4	MOLECULE:	AMINO A	CID ABC	TRANSPORTER SUBSTRATE-BINDING PROT
9:00	3u0s-A	2.1	3.2	58	327	7	MOLECULE:	DIISOPR	OPYL-FL	UOROPHOSPHATASE
10:00	6rw8-B	2.1	3.4	50	2337	2	MOLECULE:	A COMPO	NENT OF	INSECTICIDAL TOXIN COMPLEX (TC)
11:00	8xcj-F	2.1	7.6	55	420	4	MOLECULE:	MALTOPO	RIN	
12:00	6mfi-A	2.1	7.9	54	264	6	MOLECULE:	METALLO	#NAME?	ACTAMASE
13:00	1jyh-A	2.1	4	54	155	2	MOLECULE:	DNA GYR	ASE INH	IBITORY PROTEIN
14:00	6e8e-A	2.1	3.6	44	468	11	MOLECULE:	BETA SL	IDING C	LAMP,DNA MISMATCH REPAIR PROTEIN MU
15:00	8sy3-A	2.1	3.6	58	209	3	MOLECULE:	BURP DO	MAIN-CO	NTAINING PROTEIN
16:00	6trq-C	2.1	3.9	55	336	4	MOLECULE:	M7GPPPX	DIPHOS	PHATASE
17:00	8c8g-B	2.1	3	55	1195	2	MOLECULE:	PUTATIV	E BOTUL	INUM-LIKE TOXIN WO
18:00	6goc-A	2.1	3.2	49	444	8	MOLECULE:	DUF3826	DOMAIN	-CONTAINING PROTEIN
19:00	5dl5-A	2.1	5.3	59	416	7	MOLECULE:	MEMBRAN	E PROTE	IN
20:00	4ut1-A	2.1	5	57	551	4	MOLECULE:	FLAGELL	AR HOOK	-ASSOCIATED PROTEIN
21:00	8iha-A	2.1	4	58	129	10	MOLECULE:	POLYKET	IDE CYC	LASE / DEHYDRASE AND LIPID TRANSPOR
22:00	8y7e-3	2.1	6.6	60	1193	3	MOLECULE:	PRE-MRN	A	
23:00	7qe7-I	2.1	5.8	57	742	7	MOLECULE:	ANAPHAS	E-PROMO	TING COMPLEX SUBUNIT 10
24:00:00	8rt6-A	2.1	3.5	58	221	3	MOLECULE:	TRWE PR	OTEIN	
25:00:00	6nd4-J	2.1	3.3	55	493	7	MOLECULE:	ETS RRN	A	
26:00:00	7rtn-A	2.1	4.4	63	270	8	MOLECULE:	OUTER C	APSID P	ROTEIN VP5

27:00:00	8jxc-A	2.1	3.3	49	1337	14	MOLECULE:	LDL REC	EPTOR R	ELATED PROTEIN 2
28:00:00	1ugj-A	2.1	3.7	56	141	2	MOLECULE:	RIKEN C	DNA 231	0057J16 PROTEIN
29:00:00	8dit-C	2.1	3.1	66	736	5	MOLECULE:	VACUOLA	R PROTE	IN SORTING-ASSOCIATED PROTEIN 33
30:00:00	8a0j-A	2.1	8	55	223	7	MOLECULE:	UNCHARA	CTERIZE	D PROTEIN TCIL3000_11_11110
31:00:00	7fh6-A	2.1	3.5	62	653	5	MOLECULE:	CYLK		
32:00:00	8fs6-G	2.1	3.2	44	298	9	MOLECULE:	CHECKPO	INT PRO	TEIN RAD24
33:00:00	8ovw-O	2.1	5.8	55	241	13	MOLECULE:	CENTROM	ERE-BIN	DING PROTEIN 1
34:00:00	7pkq-N	2.1	2.9	58	195	3	MOLECULE:	MS35		
35:00:00	7sg7-S	2.1	3.3	56	471	4	MOLECULE:	GENE 14	PROTEI	N
36:00:00	6mit-G	2.1	4.6	58	355	3	MOLECULE:	LIPOPOL	YSACCHA	RIDE EXPORT SYSTEM ATP-BINDING PROT
37:00:00	7pkn-O	2.1	4.1	62	210	6	MOLECULE:	CENTROM	ERE PRO	TEIN H
38:00:00	3no2-A	2.1	3	57	274	2	MOLECULE:	UNCHARA	CTERIZE	D PROTEIN
39:00:00	1y8c-A	2.1	8.7	58	246	0	MOLECULE:	S-ADENO	SYLMETH	IONINE-DEPENDENT METHYLTRANSFERASE
40:00:00	6tdy-M	2.1	4.4	51	243	6	MOLECULE:	ATP SYN	THASE S	UBUNIT ALPHA
41:00:00	4jf7-A	2.1	3.9	69	501	6	MOLECULE:	HEMAGGL	UTININ-	NEURAMINIDASE
42:00:00	6nd4-L	2.1	3	43	473	5	MOLECULE:	ETS RRN	A	
43:00:00	5yjl-D	2.1	4.1	64	253	6	MOLECULE:	GLUTAMY	L-TRNA	REDUCTASE 1
44:00:00	7lc1-B	2.1	4.1	56	218	7	MOLECULE:	ISOFORM	2B OF	GTPASE KRAS
45:00:00	7bjt-A	2.1	3.7	58	727	7	MOLECULE:	ALGINAT	E LYASE	, FAMILY PL17
46:00:00	1vrq-D	2.1	3.9	55	91	5	MOLECULE:	SARCOSI	NE OXID	ASE ALPHA SUBUNIT
47:00:00	6nd4-W	2.1	2.7	51	385	14	MOLECULE:	ETS RRN	A	
48:00:00	8xcj-A	2.1	4.4	64	422	9	MOLECULE:	MALTOPO	RIN	
49:00:00	6rh5-A	2.1	2.9	51	138	6	MOLECULE:	ADAPTIN	EAR-BI	NDING COAT-ASSOCIATED PROTEIN 1
50:00:00	3wy4-B	2.1	2.9	59	421	8	MOLECULE:	ALPHA-G	LUCOSID	ASE
51:00:00	1bp1-A	2.1	3.2	57	456	5	MOLECULE:	BACTERI	CIDAL/P	ERMEABILITY-INCREASING PROTEIN
52:00:00	5vf3-A	2.1	2.4	52	456	8	MOLECULE:	CAPSID	VERTEX	PROTEIN GP24
53:00:00	8k9z-A	2.1	3.2	54	405	9	MOLECULE:	RDTND-R	ID CBD	
54:00:00	6yle-B	2.1	2.8	53	415	6	MOLECULE:	PRE-RRN	A-PROCE	SSING PROTEIN IPI3
55:00:00	7qf6-B	2.1	4.1	61	440	5	MOLECULE:	N(5)-HY	DROXYOR	NITHINE:CIS-ANHYDROMEVALONYL COENZY
56:00:00	3csv-A	2.1	3.2	53	322	6	MOLECULE:	AMINOGL	YCOSIDE	PHOSPHOTRANSFERASE
57:00:00	1g6o-A	2.1	2.6	41	323	7	MOLECULE:	CAG-ALP	HA	
58:00:00	2ojh-A	2.1	4.2	58	277	7	MOLECULE:	UNCHARA	CTERIZE	D PROTEIN ATU1656/AGR_C_3050
59:00:00	4yhc-A	2.1	3.7	56	439	9	MOLECULE:	STEROL	REGULAT	ORY ELEMENT-BINDING PROTEIN CLEAVAG
60:00:00	3lib-C	2.1	3.1	56	268	5	MOLECULE:	HYPOTHE	TICAL S	ENSORY TRANSDUCTION HISTIDINE KINAS
61:00:00	7t4e-A	2.1	3.7	51	307	10	MOLECULE:	EPX1		
62:00:00	4rqo-B	2.1	2.9	57	448	7	MOLECULE:	L-SERIN	E DEHYD	RATASE
63:00:00	6em5-R	2.1	3.3	52	120	8	MOLECULE:	5.8S RI	BOSOMAL	RNA
64:00:00	8e2g-A	2.1	3.8	67	523	7	MOLECULE:	BACULOV	IRAL IA	P REPEAT-CONTAINING PROTEIN 6
65:00:00	7c06-A	2.1	2.6	52	193	6	MOLECULE:	SPLICIN	G FACTO	R U2AF 23 KDA SUBUNIT
66:00:00	2nyk-A	2.1	3.7	52	237	2	MOLECULE:	M157		
67:00:00	6nd4-U	2.1	3.6	56	407	9	MOLECULE:	ETS RRN	A	
68:00:00	8bou-A	2.1	3.2	69	807	10	MOLECULE:	N,N'-DI	ACETYLC	HITOBIOSE PHOSPHORYLASE
69:00:00	5wd6-A	2.1	3.4	54	195	7	MOLECULE:	SHORT P	ALATE,	LUNG AND NASAL EPITHELIUM CARCINOMA
70:00:00	4upl-A	2.1	6	55	555	7	MOLECULE:	SULFATA	SE FAMI	LY PROTEIN
71:00:00	1a87-A	2.1	3.1	48	297	10	MOLECULE:	COLICIN	N	
72:00:00	5hzd-A	2.1	2.6	46	476	4	MOLECULE:	3' TERM	INAL UR	IDYLYL TRANSFERASE
73:00:00	8onx-A	2.1	4.5	64	371	6	MOLECULE:	METHION	INE AMI	NOPEPTIDASE 2
74:00:00	8adl-A	2.1	3.1	51	633	8	MOLECULE:	MTC5 IS	OFORM 1	
75:00:00	5hcc-C	2.1	3.3	53	148	8	MOLECULE:	COMPLEM	ENT C5	
76:00:00	8jxg-A	2.1	3.3	61	1114	7	MOLECULE:	RAT RAP		
77:00:00	4pk1-A	2.1	2.9	56	494	9	MOLECULE:	CHIMERA	PROTEI	N OF OUTER MEMBRANE PROTEIN ASSEMBL
78:00:00	6k96-B	2.1	4	55	343	9	MOLECULE:	FIVE-ME	MBERED-	CYCLITOL-PHOSPHATE SYNTHASE
79:00:00	8y6p-Q	2.1	3	52	1228	8	MOLECULE:	APAF-1	RELATED	KILLER DARK
80:00:00	5e4m-A	2.1	4.4	63	177	5	MOLECULE:	HYDROXY	NITRILE	LYASE

81:00:00	8kei-D	2.1	3.8	50	164	16	MOLECULE:	CYTOCHR	OME B-2	45 LIGHT CHAIN
82:00:00	2y3w-A	2.1	2.8	53	172	9	MOLECULE:	SPINDLE	ASSEMB	LY ABNORMAL PROTEIN 6 HOMOLOG
83:00:00	5c0p-A	2.1	3.5	62	284	8	MOLECULE:	ENDO-AR	ABINASE	
84:00:00	8ovw-P	2.1	3.6	56	257	4	MOLECULE:	CENTROM	ERE-BIN	DING PROTEIN 1
85:00:00	4ntq-A	2.1	2.9	49	76	2	MOLECULE:	CONTACT	#NAME?	ENT INHIBITOR A
86:00:00	2fgt-A	2.1	4.4	52	373	12	MOLECULE:	TWO-COM	PONENT	SYSTEM YYCF/YYCG REGULATORY PROTEIN
87:00:00	8uci-A	2.1	3.3	62	381	8	MOLECULE:	ATP DEP	ENDENT	DNA LIGASE
88:00:00	8du5-B	2.1	3.6	63	366	3	MOLECULE:	SIALIDA	SE-1	
89:00:00	5afu-K	2.1	3.6	74	275	4	MOLECULE:	DYNEIN	TAIL	
90:00:00	3aae-C	2.1	3.6	74	270	5	MOLECULE:	F-ACTIN	#NAME?	G PROTEIN SUBUNIT ALPHA-1
91:00:00	1qki-A	2.1	4.4	75	487	1	MOLECULE:	GLUCOSE	#NAME?	PHATE 1-DEHYDROGENASE
92:00:00	7qyp-A	2.1	2.9	67	519	12	MOLECULE:	BNR/ASP	#NAME?	PEAT PROTEIN
93:00:00	2kxp-A	2.1	3.8	74	275	5	MOLECULE:	F-ACTIN	#NAME?	G PROTEIN SUBUNIT ALPHA-1
94:00:00	5tsp-B	2.1	3.3	65	448	5	MOLECULE:	SIALIDA	SE	
95:00:00	8du5-A	2.1	3.5	61	366	3	MOLECULE:	SIALIDA	SE-1	
96:00:00	7qyj-A	2.1	2.9	66	519	12	MOLECULE:	BNR/ASP	#NAME?	PEAT PROTEIN
97:00:00	1ms0-A	2.1	2.9	64	623	11	MOLECULE:	TRANS-S	IALIDAS	E
98:00:00	4yz1-A	2.1	3.2	59	656	3	MOLECULE:	PUTATIV	E NEURA	MINIDASE
99:00:00	6myv-D	2.1	3.3	57	522	5	MOLECULE:	SIALIDA	SE26	
0:00	7xhl-F	2.1	4.1	75	462	7	MOLECULE:	GLUCOSE	6-PHOS	PHATE DEHYDROGENASE
1:00	5cfv-A	2.1	6.6	59	484	8	MOLECULE:	MALTOSE	#NAME?	G PERIPLASMIC PROTEIN,MALTOSE-BINDI
2:00	4xa2-B	2.1	3.5	56	488	4	MOLECULE:	MALTOSE	#NAME?	G PERIPLASMIC PROTEIN,MBP-PILA: C
3:00	1w8o-A	2.1	2.9	54	601	4	MOLECULE:	BACTERI	AL SIAL	IDASE
4:00	2vw0-A	2.1	3.1	57	657	11	MOLECULE:	SIALIDA	SE B	
5:00	7qy8-B	2.1	2.8	58	519	5	MOLECULE:	BNR/ASP	#NAME?	PEAT PROTEIN
6:00	2ya8-A	2.1	3.2	64	470	6	MOLECULE:	NEURAMI	NIDASE	A
7:00	4q6k-C	2.1	3.9	69	523	12	MOLECULE:	BNR/ASP	#NAME?	PEAT PROTEIN
8:00	4fow-A	2.1	3.4	61	658	11	MOLECULE:	SIALIDA	SE B	
9:00	5u0p-F	2	3.8	58	195	2	MOLECULE:	MEDIATO	R COMPL	EX SUBUNIT 14
10:00	8e0f-B	2	4	60	454	10	MOLECULE:	DOUBLE-	STRANDE	D RNA-SPECIFIC EDITASE 1
11:00	6wub-f	2	4.2	53	97	6	MOLECULE:	16S RRN	A	
12:00	3mqz-A	2	4	75	205	5	MOLECULE:	UNCHARA	CTERIZE	D CONSERVED PROTEIN DUF1054
13:00	8bgm-D	2	4.2	74	476	7	MOLECULE:	TOXIN		
14:00	5dcx-A	2	3.8	60	209	2	MOLECULE:	PROTEIN	REP68	
15:00	7lzh-A	2	4.8	78	799	8	MOLECULE:	GLUTAMA	TE RECE	PTOR 3.4
16:00	3n72-B	2	3.7	67	152	3	MOLECULE:	PUTATIV	E ACTIV	ATOR OF HSP90
17:00	5z4g-A	2	3.2	59	145	3	MOLECULE:	SAHS4		
18:00	7cn3-C	2	4	77	349	4	MOLECULE:	2,5-DIH	YDROXYP	YRIDINE 5,6-DIOXYGENASE
19:00	7b3a-A	2	3.4	63	276	6	MOLECULE:	PUTATIV	E ACYL-	TRANSFERASE DOMAIN PROTEIN
20:00	5lal-B	2	4.5	61	158	2	MOLECULE:	DIRIGEN	T PROTE	IN 6
21:00	6tyd-V	2	3.9	59	219	3	MOLECULE:	LIM DOM	AIN-BIN	DING PROTEIN 1
22:00	7sn8-I	2	2.6	47	634	11	MOLECULE:	INTEGRA	TOR COM	PLEX SUBUNIT 4
23:00	1xko-B	2	2.8	57	159	12	MOLECULE:	CHEMOTA	XIS PRO	TEIN CHEX
24:00:00	1dbf-A	2	4.8	60	127	12	MOLECULE:	PROTEIN	(CHORI | SMATE MUTASE)
25:00:00	6lix-B	2	3.6	74	196	7	MOLECULE:	CHROMOF	HORE LY	ASE CRL, CHLOROPLASTIC
26:00:00	2p2s-A	2	3.6	67	333	9	MOLECULE:	PUTATIV	E OXIDO	REDUCTASE
27:00:00	7el9-A	2	3.9	55	1731	5	MOLECULE:	RNA-DIR	ECTED R	NA POLYMERASE L
28:00:00	1ss4-A	2	3.2	50	149	6	MOLECULE:	GLYOXAL	ASE FAM	ILY PROTEIN
29:00:00	3w15-A	2	3.9	60	336	5	MOLECULE:	PEROXIS	OMAL TA	RGETING SIGNAL 2 RECEPTOR
30:00:00	7rw4-A	2	4	45	321	0	MOLECULE:	JUNCTOP	HILIN-1	
31:00:00	2hye-A	2	3.6	55	1140	2	MOLECULE:	DNA DAM	AGE-BIN	DING PROTEIN 1
32:00:00	5o51-A	2	3	54	309	6	MOLECULE:	RHO GUA	NYL NUC	LEOTIDE EXCHANGE FACTOR (ROM2), PUT
33:00:00	4rki-A	2	3.7	50	374	4	MOLECULE:	DNA POL	YMERASE	III SUBUNIT BETA
34:00:00	4yy8-B	2	2.2	47	377	4	MOLECULE:	KELCH P	ROTEIN	

35:00:00	2opd-A	2	3.3	59	121	5	MOLECULE:	PILX			
36:00:00	7st9-G	2	3.6	53	340	6	MOLECULE:	CHECKPO	INT PRO	TEIN RAD24	
37:00:00	3lp9-A	2	3.1	49	227	6	MOLECULE:	LS-24			
38:00:00	6l7x-A	2	3.1	53	203	11	MOLECULE:	MRNA_TR	IPASE D	OMAIN-CONTAINING PROTEIN	
39:00:00	8suc-A	2	2.6	52	282	8	MOLECULE:	NHL (RI	NG FING	ER B-BOX COILED COIL) DOMAIN CONTAI	
40:00:00	3r75-B	2	4.8	60	622	7	MOLECULE:	ANTHRAN	ILATE/P	ARA-AMINOBENZOATE SYNTHASES COMPONE	
41:00:00	6fpt-A	2	3.1	55	391	11	MOLECULE:	E3 UBIQ	UITIN-P	ROTEIN LIGASE TRIM71	
42:00:00	2g7j-A	2	3.1	55	112	5	MOLECULE:	PUTATIV	E CYTOP	LASMIC PROTEIN	
43:00:00	2ntk-A	2	3.8	53	206	8	MOLECULE:	IMP CYC	LOHYDRO	LASE	
44:00:00	7l5m-A	2	8.1	51	87	4	MOLECULE:	LIPOCAL	IN FAMI	LY PROTEIN	
45:00:00	6a5g-B	2	3.9	66	157	9	MOLECULE:	[4+2] A	ND [4+6	] CYCLASE STMD	
46:00:00	5jrk-A	2	3.2	57	695	12	MOLECULE:	DIPEPTI	DYL AMI	NOPEPTIDASES/ACYLAMINOACYL-PEPTIDAS	
47:00:00	6j6q-F	2	3.8	56	161	2	MOLECULE:	PRE-MRN	A-SPLIC	ING FACTOR 8	
48:00:00	3pqi-A	2	3.7	47	202	2	MOLECULE:	GENE PR	ODUCT 1		38
49:00:00	5mq0-t	2	3	48	438	8	MOLECULE:	YEAST U	BC4 GEN	E FOR UBIQUITIN-CONJUGATING ENZYME	
50:00:00	6gie-A	2	4.8	60	218	5	MOLECULE:	33-36 K	DA OUTE	R MEMBRANE PROTEIN	
51:00:00	7zao-A	2	3.4	69	429	6	MOLECULE:	SIALIDA	SE (NEU	RAMINIDASE) FAMILY PROTEIN-LIKE PRO	
52:00:00	8ck1-A	2	3.2	53	826	2	MOLECULE:	TAIL NO	ZZLE		
53:00:00	8eec-A	2	4.3	53	317	8	MOLECULE:	ISOFORM	2 OF M	ITOGEN-ACTIVATED PROTEIN KINASE KIN	
54:00:00	3to3-B	2	3.5	53	595	6	MOLECULE:	PETROBA	CTIN BI	OSYNTHESIS PROTEIN ASBB	
55:00:00	2fug-7	2	4.4	60	127	3	MOLECULE:	NADH-QU	INONE O	XIDOREDUCTASE CHAIN 1	
56:00:00	1r0u-A	2	4.6	50	142	8	MOLECULE:	PROTEIN	YWIB		
57:00:00	8bux-A	2	3.4	66	289	9	MOLECULE:	GRANULE	ASSOCI	ATED RAC AND RHOG EFFECTOR PROTEIN	
58:00:00	5vh9-B	2	2.9	50	354	6	MOLECULE:	DYNEIN	HEAVY C	HAIN, CYTOPLASMIC	
59:00:00	5nqz-A	2	3.7	52	267	2	MOLECULE:	FACTOR	H BINDI	NG PROTEIN,MAJOR OUTER MEMBRANE PRO	
60:00:00	5wru-E	2	4.7	50	311	2	MOLECULE:	PROBABL	E INORG	ANIC PYROPHOSPHATASE	
61:00:00	6qam-A	2	3.6	55	211	9	MOLECULE:	OUTER M	EMBRANE	PROTEIN ALKL	
62:00:00	3t4k-A	2	3.8	54	268	11	MOLECULE:	HISTIDI	NE KINA	SE 4	
63:00:00	1qqg-B	2	4.3	51	210	4	MOLECULE:	INSULIN	RECEPT	OR SUBSTRATE 1	
64:00:00	3kya-A	2	3.6	61	471	10	MOLECULE:	PUTATIV	E PHOSP	HATASE	
65:00:00	6m48-A	2	3.6	65	813	11	MOLECULE:	SPAC			
66:00:00	7wj9-A	2	3.5	54	733	6	MOLECULE:	ALPHA-X	YLOSIDA	SE	
67:00:00	7snh-C	2	3.6	62	476	3	MOLECULE:	GLUCOSE	#NAME?	PHATE 1-DEHYDROGENASE	
68:00:00	3aae-I	2	3.5	75	270	5	MOLECULE:	F-ACTIN	#NAME?	G PROTEIN SUBUNIT ALPHA-1	
69:00:00	2pyw-B	2	2.4	55	411	5	MOLECULE:	UNCHARA	CTERIZE	D PROTEIN	
70:00:00	7qy9-B	2	3.3	68	519	12	MOLECULE:	BNR/ASP	#NAME?	PEAT PROTEIN	
71:00:00	1ms3-A	2	3.2	69	623	10	MOLECULE:	TRANS-S	IALIDAS	E	
72:00:00	5aq1-C	2	4.3	69	484	4	MOLECULE:	GLUCOSE	#NAME?	PHATE DEHYDROGENASE	
73:00:00	4yz4-B	2	3.6	59	656	7	MOLECULE:	SIALIDA	SE, NAN	C	
74:00:00	7sng-B	2	3.4	71	467	7	MOLECULE:	GLUCOSE	#NAME?	PHATE 1-DEHYDROGENASE	
75:00:00	4yz5-B	2	3.7	54	654	6	MOLECULE:	PUTATIV	E NEURA	MINIDASE	
76:00:00	7qyj-B	2	3.2	67	519	12	MOLECULE:	BNR/ASP	#NAME?	PEAT PROTEIN	
77:00:00	3bk5-A	2	3.2	53	235	2	MOLECULE:	PUTATIV	E OUTER	MEMBRANE LIPOPROTEIN-SORTING PROTE	

DALI results: MnA-1 Col

No:	Chain	Z	rmsd	lali	nres	%id	PDB	Description
1:00	8th8-J	5.6	9	178	372	6	MOLECULE:	DYNEIN REGULATORY COMPLEX PROTEIN 1/2 N-TERMINAL
2:00	3o0z-D	5.1	5.5	144	160	4	MOLECULE:	RHO-ASSOCIATED PROTEIN KINASE 1
3:00	8q85-G	5	6.9	152	178	8	MOLECULE:	KINETOCHORE PROTEIN NDC80
4:00	4jpp-D	5	9.4	128	129	3	MOLECULE:	MINOR SPIKE PROTEIN H
5:00	2fxo-A	5	4.9	126	129	10	MOLECULE:	MYOSIN HEAVY CHAIN, CARDIAC MUSCLE BETA ISOFORM
6:00	7jg5-b	4.9	8.1	136	145	2	MOLECULE:	ATP SYNTHASE SUBUNIT ALPHA
7:00	8txr-C	4.8	3.9	167	431	5	MOLECULE:	EXODEOXYRIBONUCLEASE 7 LARGE SUBUNIT
8:00	4f61-I	4.8	7.7	173	234	9	MOLECULE:	TUBULIN ALPHA CHAIN
9:00	9bw6-A	4.8	16.6	159	783	9	MOLECULE:	MAJOR VAULT PROTEIN
10:00	8i3e-B	4.8	7	128	135	8	MOLECULE:	ELKS/RAB6-INTERACTING/CAST FAMILY MEMBER 1
11:00	2ocy-A	4.5	9	145	149	6	MOLECULE:	RAB GUANINE NUCLEOTIDE EXCHANGE FACTOR SEC2
12:00	6j5i-b	4.4	12.3	154	209	4	MOLECULE:	ATP SYNTHASE SUBUNIT ALPHA, MITOCHONDRIAL
13:00	7znk-o	4.4	8.7	134	164	9	MOLECULE:	RNA
14:00	5gai-B	4.4	7.9	131	721	8	MOLECULE:	PORTAL PROTEIN
15:00	6fkf-p	4.3	12.1	135	143	5	MOLECULE:	ATP SYNTHASE SUBUNIT ALPHA, CHLOROPLASTIC
16:00	8th8-A	4.3	7.7	158	290	6	MOLECULE:	DYNEIN REGULATORY COMPLEX PROTEIN 1/2 N-TERMINAL
17:00	4xa6-A	4.3	11.4	131	168	6	MOLECULE:	GP7-MYH7(1777-1855)-EB1 CHIMERA PROTEIN
18:00	3uf1-D	4.3	3.4	112	112	10	MOLECULE:	VIMENTIN
19:00	6e2j-B	4.3	3.1	103	104	2	MOLECULE:	KERATIN, TYPE II CYTOSKELETAL 1
20:00	6fkh-b	4.3	6.1	131	161	3	MOLECULE:	ATP SYNTHASE SUBUNIT A, CHLOROPLASTIC
21:00	7apk-m	4.2	8	128	549	6	MOLECULE:	THO COMPLEX SUBUNIT 1
22:00	8fef-B	4.2	22.9	159	335	3	MOLECULE:	VIRULENCE FACTOR MCE FAMILY PROTEIN
23:00	8th8-D	4.2	10.3	166	292	7	MOLECULE:	DYNEIN REGULATORY COMPLEX PROTEIN 1/2 N-TERMINAL
24:00:00	7fde-H	4.2	10.8	112	112	5	MOLECULE:	V-TYPE PROTON ATPASE SUBUNIT C
25:00:00	6wm2-K	4.1	10.9	114	114	9	MOLECULE:	V-TYPE PROTON ATPASE SUBUNIT E 1
26:00:00	2no2-A	4.1	3.4	102	102	8	MOLECULE:	HUNTINGTIN-INTERACTING PROTEIN 1
27:00:00	6bl7-A	4.1	2.8	99	99	6	MOLECULE:	PROTEIN STU2
28:00:00	2oto-B	4.1	6.5	134	138	8	MOLECULE:	M PROTEIN
29:00:00	8bd7-X	4.1	6.7	103	106	7	MOLECULE:	IFT88
30:00:00	5to5-A	4.1	6.6	126	141	10	MOLECULE:	NUCLEOPROTEIN TPR
31:00:00	6ys4-A	4.1	4.1	100	103	4	MOLECULE:	SPINDLE ASSEMBLY ABNORMAL PROTEIN 6 HOMOLOG
32:00:00	6iac-E	4	27.4	131	146	3	MOLECULE:	PORTAL PROTEIN
33:00:00	4lin-A	4	10.3	179	289	4	MOLECULE:	TAIL NEEDLE PROTEIN GP26
34:00:00	1d7m-A	4	4.4	99	101	10	MOLECULE:	CORTEXILLIN I
35:00:00	6uui-C	4	6.1	107	107	6	MOLECULE:	KERATIN, TYPE II CYTOSKELETAL 1
36:00:00	9ce3-C	4	11.3	183	616	4	MOLECULE:	ISOFORM 4 OF TUBERIN
37:00:00	8tek-P	3.9	8.4	128	138	5	MOLECULE:	DYNEIN REGULATORY COMPLEX PROTEIN 1/2 N-TERMINAL
38:00:00	8bd7-L	3.9	41.1	151	303	7	MOLECULE:	IFT88
39:00:00	5d80-G	3.9	5	101	223	5	MOLECULE:	V-TYPE PROTON ATPASE CATALYTIC SUBUNIT A
40:00:00	8bd7-M	3.9	22.9	137	164	2	MOLECULE:	IFT88
41:00:00	8r1a-A	3.9	5.2	167	587	5	MOLECULE:	GUANYLATE BINDING PROTEIN 1
42:00:00	6eun-A	3.9	3.2	112	147	3	MOLECULE:	ADHESIN
43:00:00	8tek-N	3.9	5.6	158	187	5	MOLECULE:	DYNEIN REGULATORY COMPLEX PROTEIN 1/2 N-TERMINAL
44:00:00	6ff7-K	3.9	33.4	123	213	3	MOLECULE:	RNA-BINDING MOTIF PROTEIN, X-LINKED 2
45:00:00	8arf-B	3.9	3.2	96	97	9	MOLECULE:	PROTEIN SPINDLY
46:00:00	8pqw-F	3.9	13.9	139	150	9	MOLECULE:	CYTOPLASMIC DYNEIN 1 HEAVY CHAIN 1
47:00:00	8i03-G	3.9	4.1	112	166	13	MOLECULE:	PAIRED AMPHIPATHIC HELIX PROTEIN PST1
48:00:00	4yto-B	3.9	5.7	96	97	4	MOLECULE:	SYNAPTONEMAL COMPLEX PROTEIN 1
49:00:00	6vq6-M	3.9	9.8	111	114	7	MOLECULE:	ATPASE H+TRANSPORTING V1 SUBUNIT A
50:00:00	8p0v-L	3.8	44.8	139	416	5	MOLECULE:	COILED-COIL DOMAIN-CONTAINING PROTEIN 93

51:00:00	8ppr-D	3.8	13.9	152	248	5	MOLECULE:	KINETOCHORE-ASSOCIATED PROTEIN DSN1 HOMOLOG
52:00:00	8i03-E	3.8	12.4	129	169	4	MOLECULE:	PAIRED AMPHIPATHIC HELIX PROTEIN PST1
53:00:00	3oja-A	3.8	32.1	146	482	7	MOLECULE:	LEUCINE-RICH IMMUNE MOLECULE 1
54:00:00	3ghg-J	3.8	8.7	129	186	3	MOLECULE:	FIBRINOGEN ALPHA CHAIN
55:00:00	3s4r-B	3.8	3.9	91	91	3	MOLECULE:	VIMENTIN
56:00:00	6h9l-B	3.8	7.5	114	129	11	MOLECULE:	UNCHARACTERIZED PROTEIN
57:00:00	8tek-B	3.8	10	147	208	2	MOLECULE:	DYNEIN REGULATORY COMPLEX PROTEIN 1/2 N-TERMINAL
58:00:00	6e2j-A	3.8	5.1	105	107	7	MOLECULE:	KERATIN, TYPE II CYTOSKELETAL 1
59:00:00	7bjg-A	3.8	5	97	98	6	MOLECULE:	SD21996P
60:00:00	8tek-A	3.7	11.6	149	200	3	MOLECULE:	DYNEIN REGULATORY COMPLEX PROTEIN 1/2 N-TERMINAL
61:00:00	5hmo-A	3.7	5.1	123	131	12	MOLECULE:	UNCONVENTIONAL MYOSIN-X
62:00:00	8fed-A	3.7	25.9	152	392	5	MOLECULE:	VIRULENCE FACTOR MCE FAMILY PROTEIN
63:00:00	6r17-B	3.7	3.9	91	94	2	MOLECULE:	SYNAPTONEMAL COMPLEX CENTRAL ELEMENT PROTEIN 2
64:00:00	9b0z-A	3.7	3.3	89	89	8	MOLECULE:	OPTINEURIN
65:00:00	6fia-D	3.7	9.2	102	104	10	MOLECULE:	LINE-1 RETROTRANSPOSABLE ELEMENT ORF1 PROTEIN
66:00:00	8ovw-U	3.6	15	128	184	2	MOLECULE:	CENTROMERE-BINDING PROTEIN 1
67:00:00	9fn1-U	3.6	7.9	120	158	8	MOLECULE:	VPS20/32/60-LIKE PROTEIN (ESCRT-III)
68:00:00	7woo-I	3.6	25.4	109	187	4	MOLECULE:	NUCLEOPORIN NIC96
69:00:00	3ni0-A	3.6	5.2	92	95	4	MOLECULE:	BONE MARROW STROMAL ANTIGEN 2
70:00:00	3oja-B	3.6	6.6	109	534	10	MOLECULE:	LEUCINE-RICH IMMUNE MOLECULE 1
71:00:00	5nvu-A	3.6	33.4	163	3169	0	MOLECULE:	DYNEIN MOTOR DOMAIN
72:00:00	6u0t-A	3.6	4.4	117	136	6	MOLECULE:	TUBULIN ALPHA CHAIN
73:00:00	6vzf-A	3.6	3.2	86	86	7	MOLECULE:	AUTOPHAGY-RELATED PROTEIN 11
74:00:00	8z22-C	3.6	3.5	90	93	7	MOLECULE:	REGULATING SYNAPTIC MEMBRANE EXOCYTOSIS 1
75:00:00	8ims-A	3.6	3.7	92	93	3	MOLECULE:	E3 UBIQUITIN-PROTEIN LIGASE TRAF7
76:00:00	8ppr-N	3.5	15.2	148	217	7	MOLECULE:	KINETOCHORE-ASSOCIATED PROTEIN DSN1 HOMOLOG
77:00:00	8ovw-Z	3.5	8.9	121	151	5	MOLECULE:	CENTROMERE-BINDING PROTEIN 1
78:00:00	5mqf-L	3.5	34.7	116	336	9	MOLECULE:	PRE-MRNA-PROCESSING-SPLICING FACTOR 8
79:00:00	4zqa-A	3.5	4.7	84	86	5	MOLECULE:	SIN3 HISTONE DEACETYLASE COREPRESSOR COMPLEX COMP
80:00:00	1x79-B	3.5	3.7	88	90	6	MOLECULE:	ADP-RIBOSYLATION FACTOR BINDING PROTEIN GGA1
81:00:00	6oqa-G	3.5	2.7	87	87	6	MOLECULE:	PEPTIDYL-PROLYL CIS-TRANS ISOMERASE FKBP1A
82:00:00	6rd4-5	3.4	6.2	115	123	12	MOLECULE:	ASA-10: POLYTOMELLA F-ATP SYNTHASE ASSOCIATED SUB
83:00:00	2p22-A	3.4	14	121	168	3	MOLECULE:	SUPPRESSOR PROTEIN STP22 OF TEMPERATURE-SENSITIVE
84:00:00	7qoo-H	3.4	31.3	130	210	5	MOLECULE:	CENTROMERE PROTEIN C
85:00:00	2xnx-M	3.4	18.6	106	107	6	MOLECULE:	FIBRINOGEN ALPHA CHAIN
86:00:00	6cfz-I	3.4	13.2	104	106	6	MOLECULE:	ASK1
87:00:00	9mhg-C	3.4	10.4	124	304	2	MOLECULE:	PHOSPHOINOSITIDE 3-KINASE REGULATORY SUBUNIT 4
88:00:00	8ppr-P	3.4	9	124	176	8	MOLECULE:	KINETOCHORE-ASSOCIATED PROTEIN DSN1 HOMOLOG
89:00:00	4y66-C	3.4	19.3	127	197	6	MOLECULE:	MND1
90:00:00	6exn-D	3.4	8.6	95	97	11	MOLECULE:	U2 SNRNA
91:00:00	5ijn-H	3.4	20.9	98	169	6	MOLECULE:	NUCLEAR PORE COMPLEX PROTEIN NUP155
92:00:00	6l5j-A	3.4	7.3	150	208	7	MOLECULE:	ROOTLETIN
93:00:00	7yqh-A	3.4	8.4	183	1069	4	MOLECULE:	STRUCTURAL MAINTENANCE OF CHROMOSOMES PROTEIN 5
94:00:00	5bw9-g	3.3	4.5	85	141	7	MOLECULE:	V-TYPE PROTON ATPASE CATALYTIC SUBUNIT A
95:00:00	5dfz-A	3.3	21.9	157	343	4	MOLECULE:	VACUOLAR PROTEIN SORTING-ASSOCIATED PROTEIN 38
96:00:00	6znl-n	3.3	48.5	112	343	5	MOLECULE:	ARP1 ACTIN RELATED PROTEIN 1 HOMOLOG A
97:00:00	8th8-B	3.3	30.2	166	276	8	MOLECULE:	DYNEIN REGULATORY COMPLEX PROTEIN 1/2 N-TERMINAL
98:00:00	3vp8-B	3.3	4.5	79	80	5	MOLECULE:	GENERAL TRANSCRIPTIONAL COREPRESSOR TUP1
99:00:00	5lxn-A	3.3	3.6	81	81	9	MOLECULE:	TRANSFORMING ACIDIC COILED-COIL-CONTAINING PROTEI
0:00	8amr-A	3.3	4.8	106	155	3	MOLECULE:	TRIPARTITE MOTIF-CONTAINING PROTEIN 3
1:00	4h22-B	3.3	4.1	82	83	6	MOLECULE:	LEUCINE-RICH REPEAT FLIGHTLESS-INTERACTING PROTEI
2:00	6fln-B	3.3	47.9	127	367	7	MOLECULE:	E3 UBIQUITIN/ISG15 LIGASE TRIM25

3:00	7fde-G	3.3	8.9	131	231	7	MOLECULE:	V-TYPE PROTON ATPASE SUBUNIT C
4:00	6b8h-b	3.3	25.4	135	200	7	MOLECULE:	ATP SYNTHASE SUBUNIT 9, MITOCHONDRIAL
5:00	8fed-F	3.2	21.3	153	399	5	MOLECULE:	VIRULENCE FACTOR MCE FAMILY PROTEIN
6:00	7puz-A	3.2	4.5	101	148	9	MOLECULE:	MICOS COMPLEX SUBUNIT MIC60
7:00	9na8-E	3.2	4.9	84	244	7	MOLECULE:	AUGMIN SUBUNIT 1
8:00	5nnv-D	3.2	5.1	127	252	9	MOLECULE:	CHROMOSOME PARTITION PROTEIN SMC,CHROMOSOME PARTI
9:00	5hda-A	3.2	8	97	121	8	MOLECULE:	ZINC FINGER MYND DOMAIN-CONTAINING PROTEIN 11
10:00	4xa1-D	3.2	7.3	103	122	9	MOLECULE:	GP7-MYH7(1173-1238)-EB1 CHIMERA PROTEIN
11:00	9bq2-A	3.2	9.3	148	402	10	MOLECULE:	FLOTILLIN-2
12:00	8xi2-K	3.2	50.6	148	210	3	MOLECULE:	MPN DOMAIN-CONTAINING PROTEIN
13:00	6vq6-I	3.2	4.6	101	225	6	MOLECULE:	ATPASE H+TRANSPORTING V1 SUBUNIT A
14:00	6y09-C	3.2	5.1	83	86	7	MOLECULE:	RAS-RELATED PROTEIN RAB-33B
15:00	8wjo-B	3.1	41.9	88	283	5	MOLECULE:	STRUCTURAL MAINTENANCE OF CHROMOSOMES PROTEIN 5
16:00	5dfz-D	3.1	13.4	135	341	7	MOLECULE:	VACUOLAR PROTEIN SORTING-ASSOCIATED PROTEIN 38
17:00	7zr1-D	3.1	7.9	146	780	8	MOLECULE:	DOUBLE-STRAND BREAK REPAIR PROTEIN
18:00	9dtr-D	3.1	10.9	100	108	3	MOLECULE:	U2 SNRNA
19:00	8hpo-D	3.1	11.6	109	165	4	MOLECULE:	TRANSCRIPTIONAL REGULATORY PROTEIN UME1
20:00	1gk4-A	3.1	2.9	78	79	6	MOLECULE:	VIMENTIN
21:00	2ch7-A	3.1	6.2	150	309	5	MOLECULE:	METHYL-ACCEPTING CHEMOTAXIS PROTEIN
22:00	3iv1-A	3.1	2.6	78	78	8	MOLECULE:	TUMOR SUSCEPTIBILITY GENE 101 PROTEIN
23:00	6lth-O	3.1	16.9	111	325	8	MOLECULE:	TRANSCRIPTION ACTIVATOR BRG1
24:00:00	3bas-B	3.1	3.9	82	87	10	MOLECULE:	MYOSIN HEAVY CHAIN, STRIATED MUSCLE/GENERAL CONTR
25:00:00	1fav-A	3.1	2.9	78	78	9	MOLECULE:	HIV-1 ENVELOPE PROTEIN CHIMERA
26:00:00	2xqh-A	3	9.5	130	258	5	MOLECULE:	IMMUNOGLOBULIN-BINDING PROTEIN EIBD
27:00:00	8tek-M	3	13	111	164	5	MOLECULE:	DYNEIN REGULATORY COMPLEX PROTEIN 1/2 N-TERMINAL
28:00:00	6oqr-X	3	7.1	120	156	7	MOLECULE:	ATP SYNTHASE SUBUNIT DELTA
29:00:00	5h07-D	3	3.9	78	78	8	MOLECULE:	POLYUBIQUITIN-C
30:00:00	8ppr-M	3	9.3	129	205	7	MOLECULE:	KINETOCHORE-ASSOCIATED PROTEIN DSN1 HOMOLOG
31:00:00	8g0p-A	3	6.8	101	143	10	MOLECULE:	KINETOCHORE PROTEIN NDC80 HOMOLOG
32:00:00	2jee-A	3	4.2	77	78	6	MOLECULE:	CELL DIVISION PROTEIN ZAPB
33:00:00	7qoo-U	3	4.4	110	186	4	MOLECULE:	CENTROMERE PROTEIN C
34:00:00	3v6i-A	3	6.4	113	186	12	MOLECULE:	V-TYPE ATP SYNTHASE SUBUNIT E
35:00:00	6z6o-D	3	65.4	162	542	7	MOLECULE:	HISTONE DEACETYLASE HDA1
36:00:00	9bq2-B	3	15.4	139	422	9	MOLECULE:	FLOTILLIN-2
37:00:00	7vdv-A	2.9	22.2	125	809	5	MOLECULE:	HISTONE H4
38:00:00	9ftl-a	2.9	29.5	116	170	6	MOLECULE:	HEIMDALLARCHAEOTA ARCHAEON AB_125 ESCRT-IIIB
39:00:00	8cqn-B	2.9	7.7	122	187	6	MOLECULE:	LIPOPROTEIN, PUTATIVE
40:00:00	8u95-A	2.9	9.8	180	1009	6	MOLECULE:	MYOSIN HEAVY CHAIN, ISOFORM U
41:00:00	9i8m-U	2.9	5.6	74	75	3	MOLECULE:	GAMMA-TUBULIN COMPLEX COMPONENT
42:00:00	3trt-A	2.9	2.8	75	75	5	MOLECULE:	VIMENTIN
43:00:00	3vem-C	2.9	3.2	79	83	9	MOLECULE:	HELICASE PROTEIN MOM1
44:00:00	2doq-D	2.9	3.6	81	83	4	MOLECULE:	CELL DIVISION CONTROL PROTEIN 31
45:00:00	4dt0-A	2.9	7.9	98	181	8	MOLECULE:	V-TYPE ATP SYNTHASE SUBUNIT E
46:00:00	7sqk-E	2.9	10.7	144	610	6	MOLECULE:	HAUS AUGMIN-LIKE COMPLEX SUBUNIT 1
47:00:00	7jtk-F	2.9	8.3	151	287	5	MOLECULE:	FLAGELLAR RADIAL SPOKE PROTEIN 1
48:00:00	9kns-B	2.9	5.1	74	75	9	MOLECULE:	PROTEIN HOOK HOMOLOG 3
49:00:00	4wpe-A	2.9	72.5	162	275	2	MOLECULE:	CYTOKINESIS PROTEIN 2
50:00:00	8ovw-B	2.9	5.8	90	107	7	MOLECULE:	CENTROMERE-BINDING PROTEIN 1
51:00:00	8q85-U	2.8	29.4	94	95	4	MOLECULE:	KINETOCHORE PROTEIN NDC80
52:00:00	7sc0-A	2.8	26.2	102	129	5	MOLECULE:	CAVEOLIN-1
53:00:00	6wuc-K	2.8	21.7	109	235	5	MOLECULE:	INNER KINETOCHORE SUBUNIT MCM16
54:00:00	8d8k-V	2.8	17.3	122	233	4	MOLECULE:	PROBABLE S-ADENOSYL-L-METHIONINE-DEPENDENT RNA

55:00:00	4wpx-B	2.8	8.8	77	81	9	MOLECULE:	CELL DIVISION CONTROL PROTEIN 31-LIKE PROTEIN
56:00:00	7sqk-H	2.8	25.7	130	201	5	MOLECULE:	HAUS AUGMIN-LIKE COMPLEX SUBUNIT 1
57:00:00	1m1j-B	2.8	7.4	129	402	3	MOLECULE:	FIBRINOGEN ALPHA SUBUNIT
58:00:00	4mvd-B	2.8	17.4	118	253	7	MOLECULE:	CHOLINE-PHOSPHATE CYTIDYLYLTRANSFERASE A
59:00:00	1m1j-D	2.8	9.6	132	194	5	MOLECULE:	FIBRINOGEN ALPHA SUBUNIT
60:00:00	9esh-W	2.8	33.6	144	526	6	MOLECULE:	PRE-MRNA
61:00:00	4aj5-B	2.8	12	85	87	7	MOLECULE:	SPINDLE AND KINETOCHORE-ASSOCIATED PROTEIN 1
62:00:00	8qbv-A	2.8	12.9	128	217	8	MOLECULE:	MEMBRANE-ASSOCIATED PROTEIN VIPP1
63:00:00	5wlq-A	2.8	4.6	86	104	5	MOLECULE:	CAPSID ASSEMBLY SCAFFOLDING PROTEIN,MYOSIN-7,MICR
64:00:00	7x2e-A	2.8	7.4	90	163	4	MOLECULE:	HARMONIN
65:00:00	3n7n-B	2.7	2.6	84	164	2	MOLECULE:	MONOPOLIN COMPLEX SUBUNIT CSM1
66:00:00	9cgi-C	2.7	13.4	113	201	4	MOLECULE:	RNA-DIRECTED RNA POLYMERASE L
67:00:00	3wqa-A	2.7	9.6	132	201	6	MOLECULE:	TRIMERIC AUTOTRANSPORTER ADHESIN
68:00:00	6oei-A	2.7	7.4	94	243	10	MOLECULE:	SPINDLE POLE BODY COMPONENT SPC42,SIGMA-54-DEPEND
69:00:00	2ba2-A	2.7	4.8	81	81	6	MOLECULE:	HYPOTHETICAL UPF0134 PROTEIN MPN010
70:00:00	4dhx-A	2.7	2.9	72	72	8	MOLECULE:	80 KDA MCM3-ASSOCIATED PROTEIN
71:00:00	5wst-A	2.7	2.1	71	71	10	MOLECULE:	UNCONVENTIONAL MYOSIN-VIIA
72:00:00	5eof-A	2.7	6.6	74	74	11	MOLECULE:	OPTINEURIN
73:00:00	3k29-A	2.7	5	98	162	6	MOLECULE:	PUTATIVE UNCHARACTERIZED PROTEIN
74:00:00	6djl-E	2.7	56.2	106	226	8	MOLECULE:	RAS-RELATED PROTEIN RAB-11A
75:00:00	7v2w-J	2.7	18.1	144	236	2	MOLECULE:	THO COMPLEX SUBUNIT HPR1
76:00:00	7oac-A	2.7	6.2	81	81	5	MOLECULE:	GENERAL CONTROL TRANSCRIPTION FACTOR GCN4,CONSERV
77:00:00	6n6s-D	2.7	3.2	68	69	10	MOLECULE:	TNFAIP3-INTERACTING PROTEIN 1
78:00:00	7yqh-B	2.7	9.8	187	1066	6	MOLECULE:	STRUCTURAL MAINTENANCE OF CHROMOSOMES PROTEIN 5
79:00:00	8q85-V	2.7	9.6	92	117	2	MOLECULE:	KINETOCHORE PROTEIN NDC80
80:00:00	5mq4-C	2.7	8.1	92	117	7	MOLECULE:	PROTEIN KINASE C-BINDING PROTEIN 1
81:00:00	5t58-B	2.6	10.9	110	190	5	MOLECULE:	KLLA0F02343P
82:00:00	8q85-b	2.6	6.2	68	70	4	MOLECULE:	KINETOCHORE PROTEIN NDC80
83:00:00	9io5-G	2.6	5.3	89	260	8	MOLECULE:	G1-ATPASE SUBUNIT BETA
84:00:00	6wcj-B	2.6	12.4	87	109	7	MOLECULE:	CLATHRIN HEAVY CHAIN 1
85:00:00	8qyd-A	2.6	6.1	118	280	4	MOLECULE:	ANTI-PHAGE DEFENSE ZORAB SYSTEM ZORA
86:00:00	9cpo-B	2.6	12.4	92	195	2	MOLECULE:	RNA-DIRECTED RNA POLYMERASE NSP12
87:00:00	8qfc-D	2.6	21.7	120	185	13	MOLECULE:	60S RIBOSOMAL PROTEIN L10A
88:00:00	7emf-D	2.6	11.5	109	158	6	MOLECULE:	MEDIATOR OF RNA POLYMERASE II TRANSCRIPTION SUBUN
89:00:00	6kn7-U	2.6	9.4	108	170	6	MOLECULE:	ACTIN, ALPHA SKELETAL MUSCLE
90:00:00	8at3-B	2.6	10.3	153	597	6	MOLECULE:	HAUS AUGMIN-LIKE COMPLEX SUBUNIT 1
91:00:00	2nps-B	2.6	3.8	66	68	2	MOLECULE:	VESICLE-ASSOCIATED MEMBRANE PROTEIN 4
92:00:00	3zdo-F	2.6	2.3	65	65	6	MOLECULE:	PHOSPHOPROTEIN
93:00:00	3b5n-C	2.6	3.3	69	70	1	MOLECULE:	SYNAPTOBREVIN HOMOLOG 1
94:00:00	5opt-P	2.6	13.6	94	249	2	MOLECULE:	ACTIVATED PROTEIN KINASE C RECEPTOR, PUTATIVE
95:00:00	6b3o-A	2.6	32	127	344	2	MOLECULE:	SPIKE GLYCOPROTEIN
96:00:00	5mq0-t	2.6	17.8	96	438	6	MOLECULE:	YEAST UBC4 GENE FOR UBIQUITIN-CONJUGATING ENZYME
97:00:00	5toh-B	2.6	5.3	69	70	1	MOLECULE:	POLYMERASE COFACTOR VP35
98:00:00	4n21-C	2.6	4	84	124	4	MOLECULE:	GP2 ECTODOMAIN
99:00:00	5ed9-B	2.6	3.9	72	72	8	MOLECULE:	SUN DOMAIN-CONTAINING PROTEIN 2
0:00	5kiu-A	2.6	7.1	75	76	3	MOLECULE:	SELENOPROTEIN S
1:00	8a5p-G	2.6	9.8	93	99	4	MOLECULE:	INO80 ATPASE
2:00	5oqm-h	2.6	9.9	102	131	6	MOLECULE:	DNA-DIRECTED RNA POLYMERASE II SUBUNIT RPB1
3:00	8j0s-d	2.6	23.3	161	445	4	MOLECULE:	ATP SYNTHASE SUBUNIT A
4:00	2gv5-C	2.6	2.4	69	73	4	MOLECULE:	CELL DIVISION CONTROL PROTEIN 31
5:00	5zuv-A	2.6	3.5	86	130	0	MOLECULE:	SPIKE GLYCOPROTEIN,INHIBITOR EK1
6:00	9lc0-K	2.5	12.2	134	298	4	MOLECULE:	60 KDA PROTEIN

7:00	8fed-E	2.5	21.4	141	358	7	MOLECULE:	VIRULENCE FACTOR MCE FAMILY PROTEIN
8:00	6ird-B	2.5	5.1	112	254	4	MOLECULE:	1-PHOSPHATIDYLINOSITOL 4,5-BISPHOSPHATE PHOSPHODI
9:00	3jb9-i	2.5	34.7	112	161	5	MOLECULE:	PRE-MRNA-SPLICING FACTOR SPP42
10:00	4r8g-E	2.5	20.2	118	324	3	MOLECULE:	UNCONVENTIONAL MYOSIN-IC
11:00	1joc-A	2.5	7	75	123	0	MOLECULE:	EARLY ENDOSOMAL AUTOANTIGEN 1
12:00	8ppr-F	2.5	2.8	66	112	14	MOLECULE:	KINETOCHORE-ASSOCIATED PROTEIN DSN1 HOMOLOG
13:00	7woo-G	2.5	6.3	91	200	3	MOLECULE:	NUCLEOPORIN NIC96
14:00	6yvu-A	2.5	61.7	146	1127	7	MOLECULE:	STRUCTURAL MAINTENANCE OF CHROMOSOMES PROTEIN 2,S
15:00	1gmj-A	2.5	2.7	65	65	8	MOLECULE:	ATPASE INHIBITOR
16:00	4l2w-D	2.5	3.7	67	68	10	MOLECULE:	RHO-ASSOCIATED PROTEIN KINASE 1
17:00	5vpc-B	2.5	3.9	68	68	4	MOLECULE:	PROTEIN FOSB
18:00	4e61-A	2.5	2.5	68	91	9	MOLECULE:	PROTEIN BIM1
19:00	4cg4-A	2.5	30.3	95	376	12	MOLECULE:	PYRIN
20:00	2wz7-B	2.5	3	67	67	3	MOLECULE:	UNCHARACTERIZED PROTEIN YBGF
21:00	3qh9-A	2.5	3.8	66	66	9	MOLECULE:	LIPRIN-BETA-2
22:00	5jhf-F	2.5	48.7	160	405	1	MOLECULE:	KLTH0D11660P
23:00	5c9n-A	2.5	2.3	64	64	6	MOLECULE:	GEMININ COILED-COIL DOMAIN-CONTAINING PROTEIN 1
24:00:00	4aj5-T	2.5	8.7	90	110	7	MOLECULE:	SPINDLE AND KINETOCHORE-ASSOCIATED PROTEIN 1
25:00:00	4nad-A	2.5	5.8	88	133	6	MOLECULE:	REGULATION OF NUCLEAR PRE-MRNA DOMAIN-CONTAINING
26:00:00	5apz-A	2.5	6.5	104	112	4	MOLECULE:	GENERAL CONTROL PROTEIN GCN4, NOR1 TCAR0761, GENE
27:00:00	6otn-B	2.5	3	66	67	5	MOLECULE:	TROPOMYOSIN ALPHA-3 CHAIN
28:00:00	8c5v-I	2.4	5.9	154	516	3	MOLECULE:	CHEMOTAXIS PROTEIN CHEA
29:00:00	7jts-s	2.4	20.8	103	290	4	MOLECULE:	RADIAL SPOKE PROTEIN 3
30:00:00	2p22-C	2.4	9.5	103	186	8	MOLECULE:	SUPPRESSOR PROTEIN STP22 OF TEMPERATURE-SENSITIVE
31:00:00	4r3z-A	2.4	4	69	76	7	MOLECULE:	AMINOACYL TRNA SYNTHASE COMPLEX-INTERACTING MULTI
32:00:00	4orh-H	2.4	2.8	74	142	8	MOLECULE:	UBIQUITIN-CONJUGATING ENZYME E2 VARIANT 2
33:00:00	1aa0-A	2.4	7.4	87	113	2	MOLECULE:	FIBRITIN
34:00:00	7bv6-B	2.4	2.2	61	61	5	MOLECULE:	VESICLE-ASSOCIATED MEMBRANE PROTEIN 8
35:00:00	3m0d-A	2.4	3.6	63	63	8	MOLECULE:	TNF RECEPTOR-ASSOCIATED FACTOR 2
36:00:00	4n78-E	2.4	3.8	67	67	4	MOLECULE:	CYTOPLASMIC FMR1-INTERACTING PROTEIN 1
37:00:00	3b5n-D	2.4	3.5	64	64	6	MOLECULE:	SYNAPTOBREVIN HOMOLOG 1
38:00:00	3lt6-F	2.4	3	63	63	5	MOLECULE:	ADHESIN YADA
39:00:00	4c47-A	2.4	6.2	84	191	5	MOLECULE:	INNER MEMBRANE LIPOPROTEIN
40:00:00	6w1s-Q	2.4	28.6	101	131	8	MOLECULE:	MEDIATOR OF RNA POLYMERASE II TRANSCRIPTION SUBUN
41:00:00	7d2e-D	2.4	2.5	63	63	6	MOLECULE:	LIPRIN-ALPHA-2
42:00:00	8ia3-E	2.4	7.4	87	111	1	MOLECULE:	UPSTREAM STIMULATORY FACTOR 2
43:00:00	8p1u-C	2.4	10.9	89	93	6	MOLECULE:	CELL DIVISION PROTEIN FTSL
44:00:00	5jvp-B	2.4	6	68	90	12	MOLECULE:	CHIMERA PROTEIN OF CENTROMERE-ASSOCIATED PROTEIN
45:00:00	4pxj-C	2.4	2.3	59	61	8	MOLECULE:	C-JUN-AMINO-TERMINAL KINASE-INTERACTING PROTEIN 3
46:00:00	8q85-d	2.4	17.8	87	108	7	MOLECULE:	KINETOCHORE PROTEIN NDC80
47:00:00	2nps-A	2.4	3.9	62	63	10	MOLECULE:	VESICLE-ASSOCIATED MEMBRANE PROTEIN 4
48:00:00	5oqm-n	2.4	16.1	97	136	6	MOLECULE:	DNA-DIRECTED RNA POLYMERASE II SUBUNIT RPB1
49:00:00	7eeb-C	2.3	4.1	80	278	6	MOLECULE:	ENHANCED GREEN FLUORESCENT PROTEIN,CATION CHANNEL
50:00:00	8q3v-g	2.3	14.5	73	76	7	MOLECULE:	TETRAHYDROMETHANOPTERIN S-METHYLTRANSFERASE SUBUN
51:00:00	4y66-D	2.3	47.7	115	208	11	MOLECULE:	MND1
52:00:00	1go4-G	2.3	16.1	88	100	9	MOLECULE:	MITOTIC SPINDLE ASSEMBLY CHECKPOINT PROTEIN MAD2A
53:00:00	5c1f-A	2.3	43.2	129	298	5	MOLECULE:	SEPTATION PROTEIN IMP2
54:00:00	8soi-B	2.3	16.8	145	515	0	MOLECULE:	RB1-INDUCIBLE COILED-COIL PROTEIN 1
55:00:00	3p8c-F	2.3	11	105	156	3	MOLECULE:	CYTOPLASMIC FMR1-INTERACTING PROTEIN 1
56:00:00	4cjd-A	2.3	17.9	79	123	6	MOLECULE:	NADA
57:00:00	1z56-A	2.3	4.8	75	77	4	MOLECULE:	LIGASE INTERACTING FACTOR 1
58:00:00	5odw-D	2.3	2.7	64	198	9	MOLECULE:	FERRIPYOVERDINE RECEPTOR

59:00:00	1t2k-D	2.3	2.8	60	61	3	MOLECULE:	31-MER
60:00:00	6kn7-T	2.3	15.7	82	138	10	MOLECULE:	ACTIN, ALPHA SKELETAL MUSCLE
61:00:00	3m0d-C	2.3	2.6	63	63	5	MOLECULE:	TNF RECEPTOR-ASSOCIATED FACTOR 2
62:00:00	2yo2-A	2.3	5.8	106	160	3	MOLECULE:	GENERAL CONTROL PROTEIN GCN4, PUTATIVE INNER MEMB
63:00:00	1s1c-Y	2.3	3.9	70	70	7	MOLECULE:	TRANSFORMING PROTEIN RHOA
64:00:00	2n64-A	2.3	6	71	75	8	MOLECULE:	SH3 DOMAIN-CONTAINING KINASE-BINDING PROTEIN 1
65:00:00	1h88-B	2.3	5.3	69	71	4	MOLECULE:	CCAAT/ENHANCER BINDING PROTEIN BETA
66:00:00	7sqk-C	2.3	32.2	164	600	5	MOLECULE:	HAUS AUGMIN-LIKE COMPLEX SUBUNIT 1
67:00:00	4ke2-A	2.3	38.6	107	196	5	MOLECULE:	TYPE I HYPERACTIVE ANTIFREEZE PROTEIN
68:00:00	3hrn-A	2.3	3.6	63	63	8	MOLECULE:	TRANSIENT RECEPTOR POTENTIAL (TRP) CHANNEL
69:00:00	8at3-C	2.3	19.8	144	353	10	MOLECULE:	HAUS AUGMIN-LIKE COMPLEX SUBUNIT 1
70:00:00	7w5a-X	2.3	11.3	86	87	6	MOLECULE:	PRE-MRNA-PROCESSING-SPLICING FACTOR 8
71:00:00	7v2w-I	2.3	17.5	137	239	9	MOLECULE:	THO COMPLEX SUBUNIT HPR1
72:00:00	8t1l-I	2.3	25.7	117	1111	3	MOLECULE:	MEDIATOR OF RNA POLYMERASE II TRANSCRIPTION SUBUN
73:00:00	5n9j-D	2.3	16.9	95	135	7	MOLECULE:	MEDIATOR OF RNA POLYMERASE II TRANSCRIPTION SUBUN
74:00:00	8teu-E	2.3	14.6	101	495	8	MOLECULE:	LARGE TEGUMENT PROTEIN DENEDDYLASE
75:00:00	7d2h-D	2.3	3	59	60	15	MOLECULE:	LIPRIN-ALPHA-2
76:00:00	7mge-B	2.2	10.9	67	442	9	MOLECULE:	WD REPEAT-CONTAINING PROTEIN 41
77:00:00	8hqo-x	2.2	8.7	77	80	3	MOLECULE:	PORTAL PROTEIN
78:00:00	6em5-t	2.2	15.7	77	290	9	MOLECULE:	5.8S RIBOSOMAL RNA
79:00:00	6gmh-Q	2.2	17.7	109	884	8	MOLECULE:	RPB1
80:00:00	2m0q-A	2.2	59.6	98	123	3	MOLECULE:	POTASSIUM VOLTAGE-GATED CHANNEL SUBFAMILY E MEMBE
81:00:00	4i1l-A	2.2	3.7	61	62	3	MOLECULE:	FORKHEAD BOX PROTEIN P3
82:00:00	7zcg-B	2.2	12.5	104	147	5	MOLECULE:	CHARGED MULTIVESICULAR BODY PROTEIN 2A
83:00:00	4om3-D	2.2	4.1	80	122	10	MOLECULE:	TRANSDUCIN-LIKE ENHANCER PROTEIN 1
84:00:00	8idc-E	2.2	42.3	106	300	4	MOLECULE:	CELL DIVISION ATP-BINDING PROTEIN FTSE
85:00:00	6gvw-F	2.2	31	101	320	5	MOLECULE:	BRCA1-A COMPLEX SUBUNIT ABRAXAS 1
86:00:00	7z47-D	2.2	3.6	71	87	4	MOLECULE:	ADAPTOR PROTEIN
87:00:00	8q85-Y	2.2	5.6	66	72	6	MOLECULE:	KINETOCHORE PROTEIN NDC80
88:00:00	7zm8-W	2.2	6.9	72	101	7	MOLECULE:	NADH-UBIQUINONE OXIDOREDUCTASE CHAIN 1
89:00:00	9na9-A	2.1	7.7	68	103	9	MOLECULE:	AUGMIN SUBUNIT 1
90:00:00	5n9j-U	2.1	25.2	100	198	9	MOLECULE:	MEDIATOR OF RNA POLYMERASE II TRANSCRIPTION SUBUN
91:00:00	2v0o-A	2.1	54.5	122	273	4	MOLECULE:	FCH DOMAIN ONLY PROTEIN 2
92:00:00	3hgf-A	2.1	3.7	70	98	6	MOLECULE:	RHOPTRY PROTEIN FRAGMENT
93:00:00	7emf-G	2.1	5.3	77	161	13	MOLECULE:	MEDIATOR OF RNA POLYMERASE II TRANSCRIPTION SUBUN
94:00:00	9f63-A	2.1	42.5	88	422	1	MOLECULE:	PROTEIN PNS1
95:00:00	4h8s-C	2.1	14.1	122	382	8	MOLECULE:	DCC-INTERACTING PROTEIN 13-BETA
96:00:00	5yfp-C	2.1	41.6	124	790	7	MOLECULE:	EXOCYST COMPLEX COMPONENT SEC3
97:00:00	8ap7-c	2.1	5.5	63	64	10	MOLECULE:	ATP SYNTHASE SUBUNIT A
98:00:00	8p0v-K	2.1	29.9	145	304	5	MOLECULE:	COILED-COIL DOMAIN-CONTAINING PROTEIN 93
99:00:00	5yfp-F	2.1	15.3	165	725	4	MOLECULE:	EXOCYST COMPLEX COMPONENT SEC3
0:00	2p2u-B	2.1	23.4	80	153	6	MOLECULE:	HOST-NUCLEASE INHIBITOR PROTEIN GAM, PUTATIVE
1:00	1gl2-C	2.1	3.3	58	59	3	MOLECULE:	ENDOBREVIN
2:00	2ahm-G	2.1	9.1	80	191	3	MOLECULE:	REPLICASE POLYPROTEIN 1AB, LIGHT CHAIN
3:00	4bwd-A	2.1	4.5	60	61	8	MOLECULE:	SHORT COILED-COIL PROTEIN
4:00	5gna-B	2.1	3.9	55	56	5	MOLECULE:	FLAGELLAR PROTEIN FLIT
5:00	4njl-A	2.1	2.7	81	129	5	MOLECULE:	S PROTEIN
6:00	8izl-C	2.1	6.9	74	91	4	MOLECULE:	PHOSPHOPROTEIN
7:00	8cra-E	2.1	13	77	96	5	MOLECULE:	FLORAL HOMEOTIC PROTEIN AGAMOUS
8:00	8q85-c	2.1	4.7	62	64	3	MOLECULE:	KINETOCHORE PROTEIN NDC80
9:00	8iss-D	2	12	45	289	7	MOLECULE:	TRNA-SPLICING ENDONUCLEASE SUBUNIT SEN15
10:00	6oee-A	2	68.6	119	243	8	MOLECULE:	TYPE IV SECRETION SYSTEM APPARATUS PROTEIN CAGT

11:00	5jeq-A	2	5.1	88	227	1	MOLECULE:	NITRATE/NITRITE SENSOR PROTEIN NARQ
12:00	7zmg-n	2	13.2	91	136	10	MOLECULE:	NADH-UBIQUINONE OXIDOREDUCTASE CHAIN 1
13:00	8qby-L	2	27.6	104	660	7	MOLECULE:	NADH-QUINONE OXIDOREDUCTASE SUBUNIT K
14:00	6od2-A	2	2.2	53	53	6	MOLECULE:	SPINDLE POLE BODY COMPONENT SPC42
15:00	8x5f-A	2	30.8	89	616	4	MOLECULE:	SOLUTE CARRIER FAMILY 53 MEMBER 1
16:00	7xyz-B	2	17.5	115	456	7	MOLECULE:	TRIPARTITE MOTIF-CONTAINING PROTEIN 72

DALI results: MnA-1 Colalt

No:	Chain	Z	rmsd	lali	nres	%id	PDB Description	
1:00	8cra-A	2.7	14	77	96	8	MOLECULE:	FLORAL HOMEOTIC PROTEIN AGAMOUS
2:00	6y09-C	2.7	5.7	66	86	9	MOLECULE:	RAS-RELATED PROTEIN RAB-33B
3:00	3f1i-H	2.7	4.7	75	98	7	MOLECULE:	HEPATOCYTE GROWTH FACTOR-REGULATED
4:00	5vpc-B	2.5	3.1	56	68	9	MOLECULE:	PROTEIN FOSB
5:00	5i50-B	2.4	14.4	60	96	8	MOLECULE:	MYC PROTO-ONCOGENE PROTEIN
6:00	6c0f-8	2.4	4.5	66	98	6	MOLECULE:	SACCHAROMYCES CEREVISIAE S288C 35S
7:00	7x5e-E	2.3	5.1	60	101	8	MOLECULE:	TRANSCRIPTION FACTOR MAFG
8:00	8pqw-F	2.3	25.7	62	150	8	MOLECULE:	CYTOPLASMIC DYNEIN 1 HEAVY CHAIN 1

DALI results: MnA-1 Wasp

No:	Chain	Z	rmsd	lali	nres	%id	PDB	Description		
1:00	2fzt-B	5.9	4.6	62	79	10	MOLECULE:	HYPOTHETICAL PROTEIN TM0693		
2:00	6cp3-7	5.3	34.7	111	171	7	MOLECULE:	ATP SYNTHASE SUBUNIT 9, MITOCHONDRIAL		
3:00	9b8o-U	5.3	7.4	88	214	6	MOLECULE:	ATPASE H+-TRANSPORTING V1 SUBUNIT D		
4:00	6lk8-a	5.1	22.3	115	1272	4	MOLECULE:	MGC83295 PROTEIN		
5:00	2maj-A	5.1	4.8	64	82	6	MOLECULE:	STROMAL INTERACTION MOLECULE 1		
6:00	9bpg-N	5.1	2.8	57	219	2	MOLECULE:	ATP SYNTHASE SUBUNIT C		
7:00	8hmc-B	5	7.4	71	1195	10	MOLECULE:	INTRAFLAGELLAR TRANSPORT PROTEIN 122 HOMOLO	G	
8:00	7xzi-B	5	8.9	57	166	7	MOLECULE:	CTAP3		
9:00	7uic-b	5	3.1	58	65	5	MOLECULE:	MEDIATOR OF RNA POLYMERASE II TRANSCRIPTION	SUBUN	
10:00	7ryq-B	4.9	21	100	527	4	MOLECULE:	KIF-BINDING PROTEIN		
11:00	8xij-A	4.9	2.2	49	469	6	MOLECULE:	GPI-ANCHORED WALL TRANSFER PROTEIN 1		
12:00	7bln-A	4.9	13	85	769	5	MOLECULE:	VACUOLAR PROTEIN SORTING-ASSOCIATED PROTEIN		29
13:00	6xp5-G	4.9	13.1	91	122	9	MOLECULE:	MEDIATOR OF RNA POLYMERASE II TRANSCRIPTION	SUBUN	
14:00	5wlq-A	4.9	17.4	66	104	6	MOLECULE:	CAPSID ASSEMBLY SCAFFOLDING PROTEIN,MYOSIN-	7,MICR	
15:00	6z0l-A	4.9	2.7	47	48	0	MOLECULE:	POSITIVE STRAND		
16:00	8qkd-A	4.8	6.7	67	281	12	MOLECULE:	SC-CC-5-24		
17:00	5an6-A	4.8	9.3	77	123	10	MOLECULE:	CRISPR-ASSOCIATED PROTEIN, CSM2 FAMILY		
18:00	4pi0-A	4.8	26.9	94	390	5	MOLECULE:	UNKNOWN PEPTIDE		
19:00	5b49-B	4.8	15.1	69	247	7	MOLECULE:	UDP-2,3-DIACYLGLUCOSAMINE HYDROLASE		
20:00	5j8v-A	4.8	10.8	65	3398	9	MOLECULE:	RYANODINE RECEPTOR 1		
21:00	4i0x-F	4.8	4.8	61	85	8	MOLECULE:	ESAT-6-LIKE PROTEIN MAB_3112		
22:00	5n9j-D	4.7	14.7	95	135	7	MOLECULE:	MEDIATOR OF RNA POLYMERASE II TRANSCRIPTION	SUBUN	
23:00	1lj2-B	4.7	6.4	60	110	2	MOLECULE:	NONSTRUCTURAL RNA-BINDING PROTEIN 34		
24:00:00	6w8p-B	4.7	17.2	86	426	3	MOLECULE:	ENDOSOMAL/LYSOSOMAL POTASSIUM CHANNEL TMEM1		75
25:00:00	6hgc-A	4.7	4.7	97	304	1	MOLECULE:	UBIQUITIN CARBOXYL-TERMINAL HYDROLASE CALYP	SO,UBI	
26:00:00	4ut1-A	4.7	12.2	110	551	5	MOLECULE:	FLAGELLAR HOOK-ASSOCIATED PROTEIN		
27:00:00	3oa7-A	4.7	29.8	104	193	5	MOLECULE:	HEAD MORPHOGENESIS PROTEIN, CHAOTIC NUCLEAR	MIGRA	
28:00:00	6rie-A	4.7	14.4	118	1285	7	MOLECULE:	DNA-DEPENDENT RNA POLYMERASE SUBUNIT RPO147		
29:00:00	2spc-A	4.7	5.4	70	107	4	MOLECULE:	SPECTRIN		
30:00:00	5izs-D	4.7	2.4	62	82	6	MOLECULE:	DESIGNED PROTEIN 5L6HC3_1		
31:00:00	4kqt-A	4.7	7.4	82	169	4	MOLECULE:	PUTATIVE OUTER MEMBRANE CHAPERONE (OMPH-LIK	E)	
32:00:00	8ap9-G	4.7	3.8	61	279	0	MOLECULE:	ATP SYNTHASE GAMMA SUBUNIT		
33:00:00	2a01-A	4.6	18.8	93	243	5	MOLECULE:	APOLIPOPROTEIN A-I		
34:00:00	8rsq-A	4.6	17.7	104	389	8	MOLECULE:	MICROBIAL RHODOPSIN - GTPASE CHIMERA,ADP-RI	BOSYLA	
35:00:00	8hc0-A	4.6	7.3	60	360	13	MOLECULE:	ADHESION G-PROTEIN COUPLED RECEPTOR F1		
36:00:00	3g80-A	4.6	4	61	73	10	MOLECULE:	PROTEIN B2		
37:00:00	8qfs-A	4.6	22.7	75	1183	7	MOLECULE:	ELONGATION FACTOR TU		
38:00:00	6wg3-C	4.6	33.2	99	248	7	MOLECULE:	STRUCTURAL MAINTENANCE OF CHROMOSOMES PROTE	IN 1A	
39:00:00	7zw6-A	4.6	17.3	109	793	3	MOLECULE:	SLR0869 PROTEIN		
40:00:00	7cbc-A	4.5	26.9	123	319	8	MOLECULE:	DE NOVO DESIGNED SWITCH PROTEIN CAGING A HE	MAGGLU	
41:00:00	8x5f-A	4.5	22.8	103	616	10	MOLECULE:	SOLUTE CARRIER FAMILY 53 MEMBER 1		
42:00:00	2r6i-A	4.5	2.9	47	268	0	MOLECULE:	UNCHARACTERIZED PROTEIN ATU1473		
43:00:00	7lzh-A	4.5	19	99	799	5	MOLECULE:	GLUTAMATE RECEPTOR 3.4		
44:00:00	6zyx-C	4.5	8.8	55	278	4	MOLECULE:	DYNEIN HEAVY CHAIN, OUTER ARM PROTEIN		
45:00:00	6zyv-A	4.5	3	48	238	4	MOLECULE:	CIR PROTEIN		
46:00:00	2v0p-A	4.5	13.4	85	219	5	MOLECULE:	TYPE 2A PHOSPHATASE-ASSOCIATED PROTEIN 42		
47:00:00	6nd4-T	4.5	6.6	59	812	5	MOLECULE:	ETS RRNA		
48:00:00	7wkk-A	4.5	7	54	1684	6	MOLECULE:	MGC83295 PROTEIN		
49:00:00	7pmk-X	4.5	13.3	76	665	0	MOLECULE:	DNA REPLICATION LICENSING FACTOR MCM2		
50:00:00	7tvz-A	4.5	3.4	63	850	5	MOLECULE:	BAND 3 ANION TRANSPORT PROTEIN		
51:00:00	6upn-A	4.5	25	113	245	8	MOLECULE:	ENDOPHILIN-B1		
52:00:00	5xbt-A	4.5	9	81	271	2	MOLECULE:	PROBABLE TRANSCRIPTIONAL REGULATOR		
53:00:00	5wwl-N	4.5	7.7	73	155	11	MOLECULE:	CENTROMERE PROTEIN MIS12		
54:00:00	6wc3-B	4.5	3.3	57	94	2	MOLECULE:	PROTEIN TRANSPORT PROTEIN TIP20		
55:00:00	8xku-C	4.5	26.1	110	921	6	MOLECULE:	PROBABLE INACTIVE ATP-DEPENDENT ZINC METALL	OPROTE	
56:00:00	2r9i-A	4.5	4.3	61	72	13	MOLECULE:	PUTATIVE PHAGE CAPSID PROTEIN		
57:00:00	6irr-A	4.4	33.9	103	133	6	MOLECULE:	DISRUPTED IN SCHIZOPHRENIA 1 HOMOLOG,CYCLIC	AMP-D	
58:00:00	8y7m-I	4.4	30.2	108	117	7	MOLECULE:	POLYMERASE ACIDIC PROTEIN		
59:00:00	6icz-A	4.4	25.8	124	2253	4	MOLECULE:	PROTEIN MAGO NASHI HOMOLOG 2		
60:00:00	4h54-A	4.4	7.6	89	274	6	MOLECULE:	DIGUANYLATE CYCLASE YDEH		
61:00:00	5vj4-A	4.4	9.5	80	275	10	MOLECULE:	UNCHARACTERIZED PROTEIN		
62:00:00	7wji-B	4.4	12.7	83	1663	5	MOLECULE:	PROTEIN UNC-80 HOMOLOG		
63:00:00	8wjn-B	4.4	29.1	140	481	6	MOLECULE:	STRUCTURAL MAINTENANCE OF CHROMOSOMES PROTE	IN 6	
64:00:00	7w01-A	4.4	28.2	112	1598	6	MOLECULE:	PHOSPHOLIPID-TRANSPORTING ATPASE ABCA3		
65:00:00	6dfp-A	4.4	8.2	68	361	10	MOLECULE:	VCA0883		
66:00:00	7vgr-A	4.4	16.4	91	198	8	MOLECULE:	YN7756_1 FAB LIGHT CHAIN		
67:00:00	6uxe-B	4.4	4	53	85	4	MOLECULE:	CYSTEINE DESULFURASE, MITOCHONDRIAL		
68:00:00	6h8q-A	4.4	15.2	87	879	5	MOLECULE:	COHESIN SUBUNIT SCC3		
69:00:00	4kpk-A	4.4	4.1	50	265	0	MOLECULE:	ENOYL-COA HYDRATASE/ISOMERASE		
70:00:00	3rfw-A	4.4	20.4	118	252	4	MOLECULE:	CELL-BINDING FACTOR 2		
71:00:00	5j2l-A	4.4	3.7	58	76	7	MOLECULE:	PROTEIN DESIGN 2L4HC2_11		
72:00:00	6zyd-A	4.4	28.4	119	323	2	MOLECULE:	LOW CONDUCTANCE MECHANOSENSITIVE CHANNEL YN	AI,LOW	
73:00:00	4ux3-B	4.4	2.7	54	71	9	MOLECULE:	STRUCTURAL MAINTENANCE OF CHROMOSOMES PROTE	IN 3	
74:00:00	6ql4-A	4.4	3.4	49	660	12	MOLECULE:	PUTATIVE MITOCHONDRIAL DYNAMIN PROTEIN		
75:00:00	4lws-A	4.4	3.5	68	100	7	MOLECULE:	UNCHARACTERIZED PROTEIN		
76:00:00	3fx7-B	4.4	3.5	60	87	3	MOLECULE:	PUTATIVE UNCHARACTERIZED PROTEIN		
77:00:00	6gdj-A	4.4	3	50	71	0	MOLECULE:	MTO2		
78:00:00	5h11-A	4.3	28.4	102	494	7	MOLECULE:	UNCHARACTERIZED PROTEIN		
79:00:00	7f3x-A	4.3	7.7	90	449	6	MOLECULE:	LPCAT3		
80:00:00	3u84-A	4.3	12.1	72	506	7	MOLECULE:	MENIN		
81:00:00	5img-A	4.3	3	63	467	13	MOLECULE:	DIPEPTIDASE		
82:00:00	6rax-N	4.3	12.7	82	207	2	MOLECULE:	DNA REPLICATION LICENSING FACTOR MCM2		
83:00:00	8ovw-Z	4.3	29.4	99	151	8	MOLECULE:	CENTROMERE-BINDING PROTEIN 1		
84:00:00	6cfz-J	4.3	3.2	60	134	8	MOLECULE:	ASK1		
85:00:00	4gwp-D	4.3	5.9	91	121	4	MOLECULE:	MEDIATOR OF RNA POLYMERASE II TRANSCRIPTION	SUBUN	
86:00:00	8g3a-D	4.3	21.4	95	334	6	MOLECULE:	BACITRACIN EXPORT PERMEASE PROTEIN BCEB		
87:00:00	6arz-A	4.3	5.6	68	96	4	MOLECULE:	UNCHARACTERIZED PROTEIN		
88:00:00	1kf6-C	4.3	8.3	78	130	6	MOLECULE:	FUMARATE REDUCTASE FLAVOPROTEIN		
89:00:00	7wb4-b	4.3	26.6	154	636	9	MOLECULE:	OUTER NUP133		
90:00:00	1au1-A	4.3	4.4	53	166	11	MOLECULE:	INTERFERON-BETA		
91:00:00	6c96-A	4.2	25.1	140	723	4	MOLECULE:	TWO PORE CALCIUM CHANNEL PROTEIN 1		
92:00:00	8pm6-A	4.2	31.6	192	872	5	MOLECULE:	BILE SALT EXPORT PUMP		
93:00:00	7qoo-Q	4.2	38.3	101	209	6	MOLECULE:	CENTROMERE PROTEIN C		
94:00:00	7mvv-A	4.2	25.4	161	1542	7	MOLECULE:	NUCLEOPORIN NUP192		
95:00:00	5yfp-B	4.2	19.3	77	927	8	MOLECULE:	EXOCYST COMPLEX COMPONENT SEC3		
96:00:00	1e7p-C	4.2	7.9	78	254	8	MOLECULE:	FUMARATE REDUCTASE FLAVOPROTEIN SUBUNIT		
97:00:00	1xdo-A	4.2	28	88	687	8	MOLECULE:	POLYPHOSPHATE KINASE		
98:00:00	5mbv-A	4.2	12.8	87	98	10	MOLECULE:	RECBCD ENZYME SUBUNIT RECB		
99:00:00	5v5t-A	4.2	2.5	47	306	15	MOLECULE:	CONSERVED DOMAIN PROTEIN		

0:00	8tef-A	4.2	8.1	78	516	5	MOLECULE:	RESPONSE REGULATOR RECEIVER PROTEIN		
1:00	5sy1-A	4.2	14.1	83	582	5	MOLECULE:	CALMODULIN		
2:00	3zc0-B	4.2	11.9	85	191	7	MOLECULE:	AFTRAX		
3:00	8d8j-0	4.2	17.5	102	493	8	MOLECULE:	PROBABLE S-ADENOSYL-L-METHIONINE-DEPENDENT	RNA	
4:00	8eki-C	4.2	6.8	97	701	7	MOLECULE:	PROTEIN TRANSPORT PROTEIN SEC20		
5:00	5c8j-L	4.2	9.7	61	137	5	MOLECULE:	ANTIBODY FRAGMENT, HEAVY CHAIN		
6:00	5oqj-1	4.2	22.6	100	491	2	MOLECULE:	DNA-DIRECTED RNA POLYMERASE II SUBUNIT RPB1		
7:00	7xjz-A	4.2	24.3	81	983	4	MOLECULE:	GLYCINE--TRNA LIGASE		
8:00	4gx2-B	4.2	25.6	91	548	7	MOLECULE:	TRKA DOMAIN PROTEIN		
9:00	3uum-B	4.2	3.7	66	123	5	MOLECULE:	UTROPHIN		
10:00	7qpg-W	4.2	31.8	96	779	5	MOLECULE:	PROTEIN ZWILCH HOMOLOG		
11:00	3he4-A	4.2	1.6	42	45	10	MOLECULE:	SYNZIP6		
12:00	4j41-B	4.2	3.3	62	86	10	MOLECULE:	SECRETED PROTEIN ESXB		
13:00	2n72-A	4.2	4.8	56	69	7	MOLECULE:	GOLGI RESIDENT PROTEIN GCP60		
14:00	3vop-C	4.2	2.9	43	43	19	MOLECULE:	PROTEIN A27		
15:00	6zgd-A	4.1	12.1	65	288	14	MOLECULE:	PROTON-GATED ION CHANNEL		
16:00	8qqm-B	4.1	28.8	107	180	7	MOLECULE:	ACETYLCHOLINE RECEPTOR SUBUNIT ALPHA		
17:00	6vxm-A	4.1	20.8	108	277	6	MOLECULE:	MECHANOSENSITIVE ION CHANNEL PROTEIN 1, MIT	OCHOND	
18:00	7egd-D	4.1	17.3	91	197	8	MOLECULE:	TRANSCRIPTION INITIATION FACTOR TFIID SUBUN	IT 1	
19:00	5n9j-V	4.1	11.7	83	99	7	MOLECULE:	MEDIATOR OF RNA POLYMERASE II TRANSCRIPTION	SUBUN	
20:00	5dfz-B	4.1	27	112	1224	6	MOLECULE:	VACUOLAR PROTEIN SORTING-ASSOCIATED PROTEIN		38
21:00	6vjy-B	4.1	9.1	94	280	9	MOLECULE:	ERAD-ASSOCIATED E3 UBIQUITIN-PROTEIN LIGASE	HRD1	
22:00	8otx-A	4.1	29.6	129	1054	5	MOLECULE:	SPERM-SPECIFIC SODIUM PROTON EXCHANGER		
23:00	5j43-B	4.1	3.2	57	101	5	MOLECULE:	CYSTEINE SYNTHASE A		
24:00:00	4y7k-A	4.1	18	111	250	7	MOLECULE:	LARGE CONDUCTANCE MECHANOSENSITIVE CHANNEL	PROTEI	
25:00:00	3rv0-B	4.1	23.2	91	304	8	MOLECULE:	K. POLYSPORUS DCR1		
26:00:00	8h17-A	4.1	2	49	157	2	MOLECULE:	TLR1989 PROTEIN		
27:00:00	8tcw-A	4.1	2.5	56	282	0	MOLECULE:	PYRROLINE-5-CARBOXYLATE REDUCTASE 1, MITOCH	ONDRIA	
28:00:00	8dfv-A	4.1	27.6	118	1664	4	MOLECULE:	ENDORIBONUCLEASE DCR-1		
29:00:00	5ibw-C	4.1	3.2	43	43	7	MOLECULE:	CALCIUM-BINDING EF-HAND DOMAIN-CONTAINING P	ROTEIN	
30:00:00	7ekn-F	4.1	1.3	40	42	5	MOLECULE:	IPEP		
31:00:00	2yev-B	4.1	15.5	92	319	7	MOLECULE:	CYTOCHROME C OXIDASE POLYPEPTIDE I+III		
32:00:00	7uw5-A	4.1	22.8	107	723	6	MOLECULE:	MECHANOSENSITIVE CHANNEL MSCK		
33:00:00	6al9-B	4.1	3.2	49	91	10	MOLECULE:	CHORISMATE MUTASE		
34:00:00	1q2h-A	4.1	4.8	53	63	0	MOLECULE:	ADAPTOR PROTEIN WITH PLECKSTRIN HOMOLOGY AN	D SRC	
35:00:00	6b87-D	4.1	3.8	63	102	10	MOLECULE:	TMHC2_E		
36:00:00	7cgp-J	4.1	4.1	58	83	5	MOLECULE:	MITOCHONDRIAL IMPORT INNER MEMBRANE TRANSLO	CASE S	
37:00:00	2kg7-A	4.1	5.1	57	97	4	MOLECULE:	UNCHARACTERIZED PROTEIN ESXG (PE FAMILY PRO	TEIN)	
38:00:00	6cfw-G	4.1	2.5	51	113	2	MOLECULE:	MONOVALENT CATION/H+ ANTIPORTER SUBUNIT D		
39:00:00	6tj1-C	4.1	3.3	49	74	8	MOLECULE:	DE NOVO DESIGNED WSHC6		
40:00:00	7drt-B	4.1	7.5	121	496	5	MOLECULE:	PROTEIN WNT-3A		
41:00:00	7cm3-A	4	27.1	190	1278	7	MOLECULE:	SODIUM LEAK CHANNEL NON-SELECTIVE PROTEIN		
42:00:00	5t58-N	4	46.8	104	196	5	MOLECULE:	KLLA0F02343P		
43:00:00	7wji-A	4	31.7	129	1763	5	MOLECULE:	PROTEIN UNC-80 HOMOLOG		
44:00:00	8ipr-C	4	28.5	156	515	3	MOLECULE:	COMPONENT LINKED WITH THE ASSEMBLY OF CYTOC	HROME'	
45:00:00	7rmy-A	4	29.1	97	266	6	MOLECULE:	DE NOVO DESIGNED TUNABLE HOMODIMER, D_3-337		
46:00:00	8t1l-I	4	58.6	138	1111	5	MOLECULE:	MEDIATOR OF RNA POLYMERASE II TRANSCRIPTION	SUBUN	
47:00:00	5tgz-A	4	8.7	127	439	6	MOLECULE:	CANNABINOID RECEPTOR 1,FLAVODOXIN,CANNABINO	ID REC	
48:00:00	6u8y-x	4	28.4	108	603	4	MOLECULE:	MONOVALENT CATION/H+ ANTIPORTER SUBUNIT E		
49:00:00	4hw5-A	4	2.5	49	77	2	MOLECULE:	COMPLEMENT C3		
50:00:00	7xpt-A	4	3.8	59	375	10	MOLECULE:	TRANSGLYCOSYLSE		
51:00:00	8b6l-J	4	6.9	90	121	4	MOLECULE:	PROTEIN TRANSPORT PROTEIN SEC61 SUBUNIT ALP	HA ISO	
52:00:00	7r5s-S	4	11.4	76	120	5	MOLECULE:	CENTROMERE PROTEIN H		
53:00:00	6gv8-A	4	10.5	60	157	12	MOLECULE:	HYPEROSMOLARITY RESISTANCE PROTEIN EMB		
54:00:00	6djs-A	4	8.8	99	490	0	MOLECULE:	SHORT TRANSIENT RECEPTOR POTENTIAL CHANNEL		3
55:00:00	7v2w-I	4	28.5	128	239	10	MOLECULE:	THO COMPLEX SUBUNIT HPR1		
56:00:00	8j22-C	4	5.4	77	285	6	MOLECULE:	GUANINE NUCLEOTIDE-BINDING PROTEIN G(I)/G(S	)/G(T)	
57:00:00	2be3-A	4	5.6	59	203	8	MOLECULE:	GTP PYROPHOSPHOKINASE		
58:00:00	8w5z-A	4	14.4	75	333	8	MOLECULE:	PROTEIN-TYROSINE SULFOTRANSFERASE (FRAGMENT	)	
59:00:00	3axj-B	4	11.8	76	240	4	MOLECULE:	GM27569P		
60:00:00	7w0b-A	4	17.8	126	1586	5	MOLECULE:	DICER-2, ISOFORM A		
61:00:00	5al6-A	4	2.5	42	44	5	MOLECULE:	ANASTRAL SPINDLE 2		
62:00:00	3g9r-A	4	1.8	40	42	13	MOLECULE:	FUSION COMPLEX OF HIV-1 ENVELOPE GLYCOPROTE	IN AND	
63:00:00	3mud-C	4	2.2	40	41	0	MOLECULE:	DNA REPAIR PROTEIN XRCC4,TROPOMYOSIN ALPHA-	1 CHAI	
64:00:00	2oqq-A	4	1.9	41	42	5	MOLECULE:	TRANSCRIPTION FACTOR HY5		
65:00:00	6jxr-n	4	3.7	42	287	12	MOLECULE:	T-CELL SURFACE GLYCOPROTEIN CD3 ZETA CHAIN		
66:00:00	3he4-B	4	1.3	40	44	10	MOLECULE:	SYNZIP6		
67:00:00	2dnx-A	4	5.4	66	130	12	MOLECULE:	SYNTAXIN-12		
68:00:00	6c2j-A	4	11.2	60	265	8	MOLECULE:	RNAP1		
69:00:00	3opc-B	4	4	58	131	3	MOLECULE:	UNCHARACTERIZED PROTEIN		
70:00:00	7dxb-A	4	22.5	99	738	4	MOLECULE:	SHORT TRANSIENT RECEPTOR POTENTIAL CHANNEL		3
71:00:00	6dkm-D	4	4	57	79	4	MOLECULE:	DHD131_A		
72:00:00	4kxr-A	4	2.9	59	85	7	MOLECULE:	PE25		
73:00:00	3whj-A	4	5.3	61	111	5	MOLECULE:	PROBABLE 26S PROTEASOME REGULATORY SUBUNIT	P27	
74:00:00	5vyk-C	4	4.1	53	203	4	MOLECULE:	CHIMERA PROTEIN OF BRS DOMAIN OF BRAF AND C	C-SAM	
75:00:00	4p96-A	4	7.2	56	278	4	MOLECULE:	FATTY ACID METABOLISM REGULATOR PROTEIN		
76:00:00	4y2f-A	4	3.7	49	143	4	MOLECULE:	SENSOR PROTEIN KDPD		
77:00:00	8gxl-B	4	6.4	52	122	8	MOLECULE:	SURP AND G-PATCH DOMAIN-CONTAINING PROTEIN		1
78:00:00	6oit-F	4	3.9	62	342	8	MOLECULE:	PROTEIN RDM1		
79:00:00	6u1s-A	4	2.9	62	310	3	MOLECULE:	DE NOVO DESIGNED 16-HELIX TRANSMEMBRANE NAN	OPORE,	
80:00:00	8rz0-E	4	2.8	57	113	14	MOLECULE:	RH5-34EM		
81:00:00	3h3m-A	4	4	52	89	2	MOLECULE:	FLAGELLAR PROTEIN FLIT		
82:00:00	5i9e-H	4	2.9	44	44	11	MOLECULE:	ACTIN-RELATED PROTEIN 4		
83:00:00	7kzn-B	3.9	28.1	148	683	5	MOLECULE:	HEAVY CHAIN ALPHA		
84:00:00	8ppr-P	3.9	25.7	114	176	4	MOLECULE:	KINETOCHORE-ASSOCIATED PROTEIN DSN1 HOMOLOG		
85:00:00	5yi4-A	3.9	19.5	73	145	4	MOLECULE:	DISRUPTED IN SCHIZOPHRENIA 1 HOMOLOG,NUCLEA	R DIST	
86:00:00	7jtk-A	3.9	30.2	90	749	11	MOLECULE:	FLAGELLAR RADIAL SPOKE PROTEIN 1		
87:00:00	5wb2-A	3.9	13.7	121	421	3	MOLECULE:	ENVELOPE PROTEIN US28, NANOBODY 7 FUSION PR	OTEIN	
88:00:00	7oqz-A	3.9	6.4	94	237	6	MOLECULE:	TRANSMEMBRANE PROTEIN 45A		
89:00:00	7tch-A	3.9	14.2	108	645	4	MOLECULE:	BACITRACIN EXPORT PERMEASE PROTEIN BCEB		
90:00:00	4xng-D	3.9	3.8	61	144	5	MOLECULE:	UNCHARACTERIZED PROTEIN MG218.1		
91:00:00	5x62-B	3.9	17.1	93	388	11	MOLECULE:	CARNOSINE N-METHYLTRANSFERASE		
92:00:00	8odu-C	3.9	15.3	98	232	6	MOLECULE:	ATPASE GET3		
93:00:00	7w72-A	3.9	11	85	588	6	MOLECULE:	PHOSPHATIDYLINOSITOL GLYCAN ANCHOR BIOSYNTH	ESIS C	
94:00:00	3bhn-A	3.9	2.7	49	214	0	MOLECULE:	THIJ/PFPI DOMAIN PROTEIN		
95:00:00	2p7n-A	3.9	5.3	62	335	11	MOLECULE:	PATHOGENICITY ISLAND 1 EFFECTOR PROTEIN		
96:00:00	6its-A	3.9	2.5	61	147	20	MOLECULE:	METHYL-ACCEPTING CHEMOTAXIS SENSORY TRANSDU	CER	
97:00:00	5x5y-F	3.9	2.4	51	351	0	MOLECULE:	PROBABLE ATP-BINDING COMPONENT OF ABC TRANS	PORTER	
98:00:00	3p01-A	3.9	6.8	70	178	3	MOLECULE:	TWO-COMPONENT RESPONSE REGULATOR		
99:00:00	6xf1-A	3.9	16.4	91	230	7	MOLECULE:	NESPRIN-2		
0:00	3he5-A	3.9	3.2	44	47	9	MOLECULE:	SYNZIP1		

1:00	7edp-B	3.9	2.8	40	40	15	MOLECULE:	HISTONE-LYSINE N-METHYLTRANSFERASE, H3 LYSI	NE-79
2:00	5yr0-B	3.9	2.6	42	44	10	MOLECULE:	BECLIN-1	
3:00	1jek-A	3.9	3	40	40	8	MOLECULE:	ENV POLYPROTEIN	
4:00	2xus-A	3.9	1.7	40	41	5	MOLECULE:	BREAST CANCER METASTASIS-SUPPRESSOR 1	
5:00	6iva-B	3.9	2.9	41	42	0	MOLECULE:	OXALOACETATE DECARBOXYLASE BETA CHAIN	
6:00	6pl5-A	3.9	3.5	52	336	0	MOLECULE:	PEPTIDOGLYCAN GLYCOSYLTRANSFERASE RODA	
7:00	1usd-A	3.9	3.6	40	41	10	MOLECULE:	VASODILATOR-STIMULATED PHOSPHOPROTEIN	
8:00	6mit-G	3.9	3.5	52	355	6	MOLECULE:	LIPOPOLYSACCHARIDE EXPORT SYSTEM ATP-BINDIN	G PROT
9:00	8eoi-K	3.9	19.7	139	1116	3	MOLECULE:	ER MEMBRANE PROTEIN COMPLEX SUBUNIT 1	
10:00	4gif-A	3.9	2.7	42	45	7	MOLECULE:	POLYCYSTIC KIDNEY DISEASE 2-LIKE 1 PROTEIN	
11:00	6zbj-B	3.8	24.3	136	501	7	MOLECULE:	PRECURSOR OF THE MAJOR MEROZOITE SURFACE AN	TIGENS
12:00	5oqm-n	3.8	16.9	93	136	8	MOLECULE:	DNA-DIRECTED RNA POLYMERASE II SUBUNIT RPB1	
13:00	7zkq-T	3.8	5.2	77	351	6	MOLECULE:	NADH DEHYDROGENASE SUBUNIT 2	
14:00	7xn9-A	3.8	5.1	60	476	3	MOLECULE:	SOMATOSTATIN RECEPTOR TYPE 2,ENDO-1,4-BETA-	XYLANA
15:00	8vej-B	3.8	12	62	242	5	MOLECULE:	CHD_BUTTRESS	
16:00	4kis-A	3.8	10.1	69	315	4	MOLECULE:	PUTATIVE INTEGRASE [BACTERIOPHAGE A118]	
17:00	3tul-A	3.8	3.9	86	136	6	MOLECULE:	CELL INVASION PROTEIN SIPB	
18:00	5yud-C	3.8	5.6	67	75	7	MOLECULE:	BACULOVIRAL IAP REPEAT-CONTAINING PROTEIN 1	E
19:00	8kfh-A	3.8	2.5	49	160	16	MOLECULE:	MYOGLOBIN	
20:00	8ulg-A	3.8	11.4	86	827	7	MOLECULE:	ROD CGMP-SPECIFIC 3',5'-CYCLIC PHOSPHODIEST	ERASE
21:00	3bo0-A	3.8	12.7	95	442	4	MOLECULE:	23S RIBOSOMAL RNA	
22:00	6gmh-M	3.8	32.2	130	991	9	MOLECULE:	RPB1	
23:00	5j6f-A	3.8	12.8	73	352	10	MOLECULE:	3-DEOXY-D-ARABINO-HEPTULOSONATE 7-PHOSPHATE	SYNTH
24:00:00	6s8h-F	3.8	2.2	48	239	4	MOLECULE:	LIPOPOLYSACCHARIDE ABC TRANSPORTER, ATP-BIN	DING P
25:00:00	5xsv-A	3.8	2.3	46	486	7	MOLECULE:	CHITINASE	
26:00:00	7k10-A	3.8	24.3	77	1259	5	MOLECULE:	DNA-DEPENDENT PROTEIN KINASE CATALYTIC SUBU	NIT
27:00:00	8ug4-A	3.8	29.6	102	486	6	MOLECULE:	OTOPETRIN-2	
28:00:00	6sqw-A	3.8	10.4	60	114	7	MOLECULE:	DCTP PYROPHOSPHATASE 1	
29:00:00	2p2u-B	3.8	17.9	96	153	3	MOLECULE:	HOST-NUCLEASE INHIBITOR PROTEIN GAM, PUTATI	VE
30:00:00	7t71-A	3.8	8.7	57	348	4	MOLECULE:	MEVALONATE 3,5-BISPHOSPHATE DECARBOXYLASE	
31:00:00	4u6u-D	3.8	19.8	110	283	8	MOLECULE:	COG7	
32:00:00	4cgk-A	3.8	15.3	153	351	4	MOLECULE:	SECRETED 45 KDA PROTEIN	
33:00:00	2cwo-A	3.8	2.9	58	165	2	MOLECULE:	RNA SILENCING SUPPRESSOR	
34:00:00	5yfp-H	3.8	7.6	70	518	7	MOLECULE:	EXOCYST COMPLEX COMPONENT SEC3	
35:00:00	8op7-A	3.8	20.6	128	1162	6	MOLECULE:	CATION-TRANSPORTING ATPASE-LIKE PROTEIN	
36:00:00	3oov-A	3.8	2.8	47	164	11	MOLECULE:	METHYL-ACCEPTING CHEMOTAXIS PROTEIN, PUTATI	VE
37:00:00	5gw1-C	3.8	2.8	43	176	12	MOLECULE:	SORTING NEXIN-16	
38:00:00	4cgb-B	3.8	2.5	42	45	2	MOLECULE:	ECHINODERM MICROTUBULE-ASSOCIATED PROTEIN-L	IKE 2
39:00:00	3m9b-A	3.8	4.6	64	186	9	MOLECULE:	PROTEASOME-ASSOCIATED ATPASE	
40:00:00	4r0r-A	3.8	2.9	44	48	11	MOLECULE:	EBOIZN21	
41:00:00	1n2d-C	3.8	2.5	43	48	5	MOLECULE:	MYOSIN LIGHT CHAIN	
42:00:00	2z5h-A	3.8	4.1	47	49	11	MOLECULE:	GENERAL CONTROL PROTEIN GCN4 AND TROPOMYOSI	N ALPH
43:00:00	7n15-A	3.8	10.3	70	520	6	MOLECULE:	CYCLIC NUCLEOTIDE-GATED CATION CHANNEL	
44:00:00	8e4g-a	3.8	3.2	65	127	3	MOLECULE:	PORTAL PROTEIN	
45:00:00	6ep3-B	3.8	3.4	49	218	6	MOLECULE:	LMO0651 PROTEIN	
46:00:00	5fhp-C	3.8	5.3	51	209	6	MOLECULE:	NICR	
47:00:00	7x29-A	3.8	4.1	64	991	8	MOLECULE:	SPIKE GLYCOPROTEIN	
48:00:00	8zue-A	3.7	25.1	95	531	3	MOLECULE:	ANTI-BACTERIOPHAGE PROTEIN A	
49:00:00	7vf2-A	3.7	22.7	94	1196	9	MOLECULE:	PROTEIN VIRILIZER HOMOLOG	
50:00:00	7kmt-B	3.7	25	111	475	8	MOLECULE:	TRAFFICKING PROTEIN PARTICLE COMPLEX SUBUNI	T 23
51:00:00	8ek4-B	3.7	13.8	69	135	7	MOLECULE:	ICE-BINDING PROTEIN TIP-99A	
52:00:00	5uz5-E	3.7	18.9	95	583	6	MOLECULE:	U1 SMALL NUCLEAR RIBONUCLEOPROTEIN 70 KDA H	OMOLOG
53:00:00	9c59-d	3.7	20.2	76	605	8	MOLECULE:	AP-3 COMPLEX SUBUNIT DELTA-1	
54:00:00	8bf9-G	3.7	9.3	74	158	7	MOLECULE:	RNA (1766)	
55:00:00	6s8g-G	3.7	6.2	80	244	5	MOLECULE:	LIPOPOLYSACCHARIDE ABC TRANSPORTER, ATP-BIN	DING P
56:00:00	8ahx-E	3.7	3.5	56	213	2	MOLECULE:	ION-TRANSLOCATING OXIDOREDUCTASE COMPLEX SU	BUNIT
57:00:00	6lo8-C	3.7	2.9	56	108	5	MOLECULE:	MITOCHONDRIAL IMPORT INNER MEMBRANE TRANSLO	CASE S
58:00:00	3mhh-C	3.7	9.1	64	93	5	MOLECULE:	UBIQUITIN CARBOXYL-TERMINAL HYDROLASE 8	
59:00:00	8etc-F	3.7	7.7	64	218	3	MOLECULE:	RNA (2151-MER)	
60:00:00	8jtv-A	3.7	8.3	87	437	6	MOLECULE:	SOLUTE CARRIER FAMILY 22 MEMBER 1	
61:00:00	3wfw-A	3.7	2.6	49	138	10	MOLECULE:	HEMOGLOBIN-LIKE FLAVOPROTEIN FUSED TO ROADB	LOCK/L
62:00:00	5gox-A	3.7	9	85	181	14	MOLECULE:	DNA REPAIR PROTEIN RAD50	
63:00:00	7wkk-B	3.7	7.6	83	1482	4	MOLECULE:	MGC83295 PROTEIN	
64:00:00	3dhi-A	3.7	7.4	68	498	10	MOLECULE:	TOLUENE 4-MONOOXYGENASE HYDROXYLASE ALPHA S	UBUNIT
65:00:00	5e5w-B	3.7	6.6	69	149	1	MOLECULE:	HEMAGGLUTININ-ESTERASE	
66:00:00	6q82-A	3.7	26.9	108	1027	5	MOLECULE:	IMPORTIN BETA-LIKE PROTEIN KAP122	
67:00:00	6w1s-Q	3.7	10.3	118	131	6	MOLECULE:	MEDIATOR OF RNA POLYMERASE II TRANSCRIPTION	SUBUN
68:00:00	5vjx-H	3.7	2.3	45	51	7	MOLECULE:	CLOCK-INTERACTING PACEMAKER	
69:00:00	1go4-G	3.7	30.7	68	100	4	MOLECULE:	MITOTIC SPINDLE ASSEMBLY CHECKPOINT PROTEIN	MAD2A
70:00:00	3fga-D	3.7	2.3	43	47	9	MOLECULE:	SERINE/THREONINE-PROTEIN PHOSPHATASE 2A 65	KDA RE
71:00:00	7bhq-B	3.7	7.9	86	204	7	MOLECULE:	BASAL-BODY ROD MODIFICATION PROTEIN FLGD	
72:00:00	5i50-B	3.7	2.3	48	96	8	MOLECULE:	MYC PROTO-ONCOGENE PROTEIN	
73:00:00	5vr2-A	3.7	3.3	44	50	2	MOLECULE:	MYOCILIN	
74:00:00	5y2h-A	3.7	3	43	48	7	MOLECULE:	NONSTRUCTURAL PROTEIN 4	
75:00:00	5hud-E	3.7	4.5	54	84	6	MOLECULE:	3-DEOXY-D-ARABINO-HEPTULOSONATE 7-PHOSPHATE	(DAHP
76:00:00	6dnq-E	3.7	3.4	43	54	7	MOLECULE:	BZIP FACTOR	
77:00:00	3vn0-B	3.7	3.8	42	49	7	MOLECULE:	VOLTAGE-GATED HYDROGEN CHANNEL 1	
78:00:00	6ugm-X	3.7	3.2	55	73	5	MOLECULE:	HISTONE H3	
79:00:00	3ci9-B	3.7	2.5	42	45	10	MOLECULE:	HEAT SHOCK FACTOR-BINDING PROTEIN 1	
80:00:00	7exx-A	3.7	4	57	440	4	MOLECULE:	DNA PHOSPHOROTHIOATION-DEPENDENT RESTRICTIO	N PROT
81:00:00	6akm-B	3.7	3.7	44	46	5	MOLECULE:	SUPPRESSOR OF IKBKE 1	
82:00:00	3txs-C	3.7	3.3	54	91	9	MOLECULE:	TERMINASE DNA PACKAGING ENZYME SMALL SUBUNI	T
83:00:00	8qbv-A	3.7	31	90	217	13	MOLECULE:	MEMBRANE-ASSOCIATED PROTEIN VIPP1	
84:00:00	2v4h-A	3.7	32.4	80	98	4	MOLECULE:	NF-KAPPA-B ESSENTIAL MODULATOR	
85:00:00	3ayf-A	3.7	4.5	75	754	9	MOLECULE:	NITRIC OXIDE REDUCTASE	
86:00:00	6z6o-D	3.7	28.4	102	542	7	MOLECULE:	HISTONE DEACETYLASE HDA1	
87:00:00	6cv0-A	3.7	3.9	63	993	8	MOLECULE:	SPIKE GLYCOPROTEIN	
88:00:00	5x41-F	3.7	21.5	79	244	0	MOLECULE:	COBALT ABC TRANSPORTER ATP-BINDING PROTEIN	
89:00:00	3rmi-A	3.7	2.2	50	105	10	MOLECULE:	CHORISMATE MUTASE PROTEIN	
90:00:00	3o1j-A	3.6	27.9	93	273	8	MOLECULE:	SENSOR PROTEIN TORS	
91:00:00	6th1-R	3.6	33	118	360	3	MOLECULE:	IMMEDIATE EARLY PROTEIN 1	
92:00:00	6wuc-H	3.6	17	95	179	6	MOLECULE:	INNER KINETOCHORE SUBUNIT MCM16	
93:00:00	7sgr-A	3.6	27.7	165	700	6	MOLECULE:	ALPHA-HEMOLYSIN TRANSLOCATION ATP-BINDING P	ROTEIN
94:00:00	6zie-A	3.6	31.6	83	123	12	MOLECULE:	CMPX-383B	
95:00:00	8teu-A	3.6	23.7	132	722	3	MOLECULE:	LARGE TEGUMENT PROTEIN DENEDDYLASE	
96:00:00	8yad-B	3.6	85.7	171	1396	6	MOLECULE:	SPATACSIN	
97:00:00	6myo-D	3.6	5.3	84	102	4	MOLECULE:	SUCCINATE DEHYDROGENASE [UBIQUINONE] FLAVOP	ROTEIN
98:00:00	8xr6-q	3.6	11	71	143	6	MOLECULE:	PHOTOSYSTEM II PROTEIN D1	
99:00:00	7a1g-z	3.6	12.2	75	120	8	MOLECULE:	18S RIBOSOMAL RNA	
0:00	7uxc-R	3.6	7.3	65	107	3	MOLECULE:	SERINE/THREONINE-PROTEIN KINASE MTOR	
1:00	6wqz-A	3.6	26.9	116	536	7	MOLECULE:	AUTOPHAGY-RELATED PROTEIN 9A	

2:00	6swy-1	3.6	3.4	80	542	5	MOLECULE:	VACUOLAR IMPORT AND DEGRADATION PROTEIN 28	
3:00	7etm-A	3.6	30	131	498	7	MOLECULE:	PORTAL PROTEIN	
4:00	6kkk-A	3.6	4.9	65	380	5	MOLECULE:	SUGAR EFFLUX TRANSPORTER	
5:00	6a70-B	3.6	6.2	128	704	4	MOLECULE:	POLYCYSTIN-2	
6:00	4wzx-A	3.6	2.5	53	87	8	MOLECULE:	SERINE/THREONINE-PROTEIN KINASE ULK3	
7:00	8xks-S	3.6	5	57	114	4	MOLECULE:	CTAP1	
8:00	1ej6-D	3.6	5.3	60	417	13	MOLECULE:	LAMBDA2	
9:00	7y9n-A	3.6	4.3	73	212	4	MOLECULE:	SPIKE PROTEIN S2',5HB-H2	
10:00	2ycd-A	3.6	4.8	71	213	3	MOLECULE:	GLUTATHIONE S-TRANSFERASE	
11:00	6wg3-B	3.6	12.6	98	693	9	MOLECULE:	STRUCTURAL MAINTENANCE OF CHROMOSOMES PROTE	IN 1A
12:00	7xdi-D	3.6	11.6	91	124	3	MOLECULE:	VP1	
13:00	6uo8-B	3.6	8.1	78	696	4	MOLECULE:	GAMMA-AMINOBUTYRIC ACID TYPE B RECEPTOR SUB	UNIT 1
14:00	1o7d-C	3.6	9.8	62	150	6	MOLECULE:	LYSOSOMAL ALPHA-MANNOSIDASE	
15:00	8ca1-A	3.6	13.3	137	589	6	MOLECULE:	VERY LONG-CHAIN SPECIFIC ACYL-COA DEHYDROGE	NASE,
16:00	6ye4-A	3.6	22.5	108	236	7	MOLECULE:	BIOPOLYMER TRANSPORT PROTEIN EXBB	
17:00	6dg6-A	3.6	3.7	70	100	1	MOLECULE:	NEOLEUKIN-2/15	
18:00	2lon-A	3.6	5.6	66	99	3	MOLECULE:	HIG1 DOMAIN FAMILY MEMBER 1B	
19:00	9mny-A	3.6	3.4	59	108	7	MOLECULE:	MITOCHONDRIAL PYRUVATE CARRIER 1	
20:00	9cpo-B	3.6	7.7	93	195	6	MOLECULE:	RNA-DIRECTED RNA POLYMERASE NSP12	
21:00	7bst-A	3.6	2.2	60	384	3	MOLECULE:	TYPE I RESTRICTION ENZYME R PROTEIN	
22:00	6y1y-B	3.6	4.4	64	128	3	MOLECULE:	CHEA	
23:00	3nmd-A	3.6	3.1	46	53	11	MOLECULE:	CGMP DEPENDENT PROTEIN KINASE	
24:00:00	4r4l-C	3.6	2.3	44	49	11	MOLECULE:	CGMP-DEPENDENT PROTEIN KINASE 1	
25:00:00	6akl-A	3.6	2.3	41	52	5	MOLECULE:	SUPPRESSOR OF IKBKE 1	
26:00:00	4x01-C	3.6	2.6	45	50	9	MOLECULE:	DNA BINDING CTP1	
27:00:00	6ku5-B	3.6	12.7	66	207	5	MOLECULE:	TRAF5	
28:00:00	4gkg-A	3.6	3.6	45	52	2	MOLECULE:	C4-DICARBOXYLATE TRANSPORT SENSOR PROTEIN D	CTB
29:00:00	3he5-B	3.6	2.2	41	48	12	MOLECULE:	SYNZIP1	
30:00:00	3efg-A	3.6	4.1	44	51	5	MOLECULE:	PROTEIN SLYX HOMOLOG	
31:00:00	6j6q-F	3.6	3.2	42	161	2	MOLECULE:	PRE-MRNA-SPLICING FACTOR 8	
32:00:00	7tpr-A	3.6	13.7	77	1145	4	MOLECULE:	SPIKE GLYCOPROTEIN	
33:00:00	2m67-A	3.6	3.6	59	81	3	MOLECULE:	MERF	
34:00:00	5yij-A	3.6	3	57	959	7	MOLECULE:	SDEA	
35:00:00	3zid-A	3.6	2.8	46	360	9	MOLECULE:	TUBULIN/FTSZ, GTPASE	
36:00:00	7dkh-I	3.6	2.8	65	914	8	MOLECULE:	RNA POLYMERASE-ASSOCIATED PROTEIN CTR9	
37:00:00	7yvb-A	3.6	4.9	49	471	0	MOLECULE:	FMRFAMIDE-GATED NA+ CHANNEL	
38:00:00	6hd5-t	3.6	15	90	838	9	MOLECULE:	N-TERMINAL ACETYLTRANSFERASE A COMPLEX SUBU	NIT NA
39:00:00	6xp5-H	3.6	7.5	72	146	6	MOLECULE:	MEDIATOR OF RNA POLYMERASE II TRANSCRIPTION	SUBUN
40:00:00	3u2r-A	3.6	2.7	53	135	11	MOLECULE:	REGULATORY PROTEIN MARR	
41:00:00	7bu0-A	3.6	23.4	91	407	3	MOLECULE:	UNCHARACTERIZED PROTEIN	
42:00:00	3vp8-B	3.6	3.1	49	80	6	MOLECULE:	GENERAL TRANSCRIPTIONAL COREPRESSOR TUP1	
43:00:00	7znk-o	3.5	41.4	108	164	8	MOLECULE:	RNA	
44:00:00	6f1t-f	3.5	24.4	125	929	5	MOLECULE:	ARP1 ACTIN RELATED PROTEIN 1 HOMOLOG A	
45:00:00	7emf-K	3.5	23	89	112	4	MOLECULE:	MEDIATOR OF RNA POLYMERASE II TRANSCRIPTION	SUBUN
46:00:00	6ird-B	3.5	26.7	97	254	6	MOLECULE:	1-PHOSPHATIDYLINOSITOL 4,5-BISPHOSPHATE PHO	SPHODI
47:00:00	7woo-D	3.5	25.9	125	1398	6	MOLECULE:	NUCLEOPORIN NIC96	
48:00:00	5yfp-C	3.5	41	177	790	7	MOLECULE:	EXOCYST COMPLEX COMPONENT SEC3	
49:00:00	8kg9-B	3.5	6.5	58	193	10	MOLECULE:	DNA REPLICATION LICENSING FACTOR MCM2	
50:00:00	8ikj-R	3.5	29.4	120	532	8	MOLECULE:	ADHESION G PROTEIN-COUPLED RECEPTOR E5,SOLU	BLE CY
51:00:00	5eqz-A	3.5	6.4	57	138	7	MOLECULE:	REV PROTEIN	
52:00:00	8wrx-A	3.5	4.7	50	86	4	MOLECULE:	ANTI-CRISPR PROTEIN ACRIIA28	
53:00:00	8vc1-A	3.5	7	101	391	5	MOLECULE:	GUSTATORY RECEPTOR	
54:00:00	6soy-A	3.5	5.5	105	323	10	MOLECULE:	ESAG6, SUBUNIT OF HETERODIMERIC TRANSFERRIN	RECEP
55:00:00	7xe4-F	3.5	6.1	68	1503	3	MOLECULE:	1,3-BETA-GLUCAN SYNTHASE COMPONENT FKS1	
56:00:00	8ave-A	3.5	3.9	44	146	7	MOLECULE:	LEPTIN	
57:00:00	1flc-B	3.5	11.2	92	162	5	MOLECULE:	HAEMAGGLUTININ-ESTERASE-FUSION GLYCOPROTEIN	
58:00:00	8hsi-A	3.5	7.2	104	405	6	MOLECULE:	TRANSMEMBRANE PROTEIN 87A	
59:00:00	6d03-E	3.5	28.6	79	466	6	MOLECULE:	TRANSFERRIN RECEPTOR PROTEIN 1	
60:00:00	6r7x-A	3.5	16.7	76	629	4	MOLECULE:	ANOCTAMIN-10	
61:00:00	8eg3-A	3.5	23.3	86	554	2	MOLECULE:	STERYL-SULFATASE	
62:00:00	6xpf-B	3.5	7.3	68	285	1	MOLECULE:	ZINC TRANSPORTER 8	
63:00:00	8ip4-A	3.5	17.3	139	320	4	MOLECULE:	MAGNESIUM TRANSPORTER MRS2 HOMOLOG, MITOCHO	NDRIAL
64:00:00	5a42-A	3.5	20.2	91	1597	7	MOLECULE:	UNCHARACTERIZED LIPOPROTEIN YFHM	
65:00:00	5t58-B	3.5	24.4	105	190	9	MOLECULE:	KLLA0F02343P	
66:00:00	5a1v-K	3.5	17.5	87	1125	5	MOLECULE:	ADP-RIBOSYLATION FACTOR 1	
67:00:00	2pms-C	3.5	5.1	85	109	12	MOLECULE:	LACTOTRANSFERRIN	
68:00:00	8pht-A	3.5	3.6	64	111	2	MOLECULE:	SCAFFOLD PROTEIN	
69:00:00	3anw-A	3.5	9.4	70	171	6	MOLECULE:	PUTATIVE UNCHARACTERIZED PROTEIN	
70:00:00	8q85-d	3.5	37.9	104	108	3	MOLECULE:	KINETOCHORE PROTEIN NDC80	
71:00:00	7deg-A	3.5	7.8	63	581	6	MOLECULE:	CYTOCHROME C OXIDASE SUBUNIT I	
72:00:00	6wuh-A	3.5	12.7	83	308	10	MOLECULE:	SAM35	
73:00:00	4mer-D	3.5	4.7	50	97	4	MOLECULE:	STREPTOCOCCAL HISTIDINE-RICH GLYCOPROTEIN I	NTERAC
74:00:00	3bey-D	3.5	2.8	47	94	2	MOLECULE:	CONSERVED PROTEIN O27018	
75:00:00	7wff-F	3.5	27.6	105	677	4	MOLECULE:	NAD(P)H-QUINONE OXIDOREDUCTASE SUBUNIT 1, C	HLOROP
76:00:00	1sc7-A	3.5	19.9	88	567	9	MOLECULE:	5'-D(*AP*AP*AP*AP*AP*GP*AP*CP*TP*T)-3'	
77:00:00	8eaw-a	3.5	8.2	72	287	13	MOLECULE:	CAPSID PROTEIN	
78:00:00	8y5f-B	3.5	2.1	52	273	4	MOLECULE:	SPERMIDINE/PUTRESCINE IMPORT ATP-BINDING PR	OTEIN
79:00:00	8t05-A	3.5	5.1	55	201	2	MOLECULE:	MYOMAKER	
80:00:00	8g52-A	3.5	18.6	68	279	4	MOLECULE:	TPR_REGION DOMAIN-CONTAINING PROTEIN	
81:00:00	4nnh-A	3.5	3.1	48	164	10	MOLECULE:	30S RIBOSOMAL PROTEIN S1	
82:00:00	7egm-H	3.5	11.9	99	388	9	MOLECULE:	TRANSCRIPTION REGULATORY PROTEIN SNF2	
83:00:00	1dvo-A	3.5	11	81	152	5	MOLECULE:	FERTILITY INHIBITION PROTEIN O	
84:00:00	6gmh-Q	3.5	16.7	80	884	10	MOLECULE:	RPB1	
85:00:00	3syk-A	3.5	7	78	290	1	MOLECULE:	PROTEIN CBBX	
86:00:00	8th8-K	3.5	26.6	118	434	5	MOLECULE:	DYNEIN REGULATORY COMPLEX PROTEIN 1/2 N-TER	MINAL
87:00:00	3n1e-B	3.5	5.8	54	141	11	MOLECULE:	VACUOLAR PROTEIN SORTING-ASSOCIATED PROTEIN	54
88:00:00	4um2-A	3.5	24	85	510	4	MOLECULE:	TELOMERASE-BINDING PROTEIN EST1A	
89:00:00	8vyb-A	3.5	9.9	80	464	8	MOLECULE:	ISOFORM 2 OF RAB3 GTPASE-ACTIVATING PROTEIN	CATAL
90:00:00	8fhc-B	3.5	4	51	215	0	MOLECULE:	TRNA-(MS[2]IO[6]A)-HYDROXYLASE	
91:00:00	6xwx-E	3.5	7.6	84	566	8	MOLECULE:	UNCHARACTERIZED PROTEIN,UNCHARACTERIZED PRO	TEIN
92:00:00	1u2m-C	3.5	5.2	81	143	9	MOLECULE:	HISTONE-LIKE PROTEIN HLP-1	
93:00:00	8p0v-M	3.5	3.2	48	357	8	MOLECULE:	COILED-COIL DOMAIN-CONTAINING PROTEIN 93	
94:00:00	7sqk-C	3.5	36.9	138	600	6	MOLECULE:	HAUS AUGMIN-LIKE COMPLEX SUBUNIT 1	
95:00:00	5a31-N	3.5	27.3	92	703	4	MOLECULE:	ANAPHASE-PROMOTING COMPLEX SUBUNIT 1	
96:00:00	5cx2-A	3.5	2.9	47	55	11	MOLECULE:	CORONIN	
97:00:00	1gl2-A	3.5	3.6	43	53	9	MOLECULE:	ENDOBREVIN	
98:00:00	2kes-A	3.5	2.5	44	48	5	MOLECULE:	SYNPHILIN-1	
99:00:00	4b86-B	3.5	3.8	46	51	7	MOLECULE:	MALE-SPECIFIC LETHAL 1 HOMOLOG	
0:00	1deb-A	3.5	2.1	44	54	11	MOLECULE:	ADENOMATOUS POLYPOSIS COLI PROTEIN	
1:00	6cko-C	3.5	4.7	44	50	16	MOLECULE:	PROTEIN AF-10	
2:00	1ifm-A	3.5	3.4	43	46	5	MOLECULE:	INOVIRUS	

3:00	6od2-A	3.5	3	43	53	5	MOLECULE:	SPINDLE POLE BODY COMPONENT SPC42	
4:00	2bsk-D	3.5	6.3	64	90	13	MOLECULE:	MITOCHONDRIAL IMPORT INNER MEMBRANE TRANSLO	CASE
5:00	3dkq-A	3.5	3.5	43	230	5	MOLECULE:	PKHD-TYPE HYDROXYLASE SBAL_3634	
6:00	1ezj-A	3.5	3.3	44	114	11	MOLECULE:	NUCLEOCAPSID PHOSPHOPROTEIN	
7:00	6s7t-A	3.5	26.4	104	706	6	MOLECULE:	DOLICHYL-DIPHOSPHOOLIGOSACCHARIDE--PROTEIN	
8:00	3zta-A	3.5	2.4	62	139	6	MOLECULE:	ANTI-SIGMA-FACTOR ANTAGONIST (STAS) DOMAIN	PROTEI
9:00	6lo8-E	3.5	3.7	55	80	9	MOLECULE:	MITOCHONDRIAL IMPORT INNER MEMBRANE TRANSLO	CASE S
10:00	3bm3-A	3.5	3.4	77	259	10	MOLECULE:	DNA (5'-D(*CP*AP*TP*CP*CP*AP*GP*GP*TP*AP*C)	-3')
11:00	5tj5-A	3.5	27.6	109	570	2	MOLECULE:	V-TYPE PROTON ATPASE SUBUNIT A	
12:00	6hs5-A	3.5	2.9	46	249	4	MOLECULE:	TSSA	
13:00	8th8-L	3.5	14.2	108	862	8	MOLECULE:	DYNEIN REGULATORY COMPLEX PROTEIN 1/2 N-TER	MINAL
14:00	8b6l-P	3.5	3.5	50	602	4	MOLECULE:	PROTEIN TRANSPORT PROTEIN SEC61 SUBUNIT ALP	HA ISO
15:00	6v4l-A	3.4	27.9	99	479	7	MOLECULE:	TRK SYSTEM POTASSIUM UPTAKE PROTEIN TRKH	
16:00	3j9e-D	3.4	19.2	112	520	13	MOLECULE:	VP5	
17:00	8z9y-A	3.4	25.3	125	781	3	MOLECULE:	PROTEIN TIC 214	
18:00	8ppr-D	3.4	69.6	147	248	13	MOLECULE:	KINETOCHORE-ASSOCIATED PROTEIN DSN1 HOMOLOG	
19:00	8sfo-A	3.4	37.8	153	1240	7	MOLECULE:	CRISPR-ASSOCIATED ENDONUCLEASE CAS12A	
20:00	5j0l-A	3.4	30.6	79	130	10	MOLECULE:	DESIGNED PROTEIN 3L6HC2_2	
21:00	4fhn-B	3.4	29.1	140	1022	9	MOLECULE:	NUCLEOPORIN NUP37	
22:00	8gix-E	3.4	22.7	102	991	6	MOLECULE:	HISTONE TRANSCRIPTION REGULATOR 3 HOMOLOG	
23:00	2o8p-A	3.4	6.6	97	220	3	MOLECULE:	14-3-3 DOMAIN CONTAINING PROTEIN	
24:00:00	5l1a-A	3.4	29.5	67	108	10	MOLECULE:	UNCHARACTERIZED PROTEIN	
25:00:00	8ttf-A	3.4	30.6	94	388	10	MOLECULE:	QUINOLONE RESISTANCE PROTEIN NORA	
26:00:00	8gb3-F	3.4	2.8	39	90	3	MOLECULE:	CHAPERONE PROTEIN DNAK	
27:00:00	8bd7-M	3.4	24	127	164	6	MOLECULE:	IFT88	
28:00:00	8whn-A	3.4	4.3	95	142	5	MOLECULE:	RIBOPHORIN 1 SUPERFAMILY PROTEIN	
29:00:00	3add-A	3.4	2.5	45	251	11	MOLECULE:	L-SERYL-TRNA(SEC) KINASE	
30:00:00	2d6y-B	3.4	2.3	43	188	5	MOLECULE:	PUTATIVE TETR FAMILY REGULATORY PROTEIN	
31:00:00	7z0l-B	3.4	18.8	72	370	4	MOLECULE:	INTERLEUKIN-6 RECEPTOR SUBUNIT BETA	
32:00:00	7tmw-R	3.4	17	113	499	4	MOLECULE:	RELAXIN RECEPTOR 1, GUANINE NUCLEOTIDE-BIND	ING PR
33:00:00	1llm-C	3.4	2.5	45	87	7	MOLECULE:	5'-D(*TP*CP*CP*CP*AP*CP*GP*CP*GP*TP*GP*GP*G	)-3'
34:00:00	7yzp-C	3.4	27.7	115	448	5	MOLECULE:	DNA HAIRPIN (59-MER)	
35:00:00	4g6d-B	3.4	3.4	48	198	10	MOLECULE:	RNA POLYMERASE SIGMA FACTOR RPOD	
36:00:00	8ij1-D	3.4	20	75	627	11	MOLECULE:	CULLIN-2	
37:00:00	7eu9-A	3.4	4.9	72	1078	8	MOLECULE:	CAS12I1 D647A MUTANT	
38:00:00	7ewp-A	3.4	14.5	110	577	2	MOLECULE:	PROBABLE G-PROTEIN COUPLED RECEPTOR 158	
39:00:00	3j83-A	3.4	4.3	60	276	2	MOLECULE:	ESX-1 SECRETION-ASSOCIATED PROTEIN ESPB	
40:00:00	9ivk-A	3.4	4.1	67	278	7	MOLECULE:	HBC599 MEMBRANE PROTEIN BINDER	
41:00:00	4jio-B	3.4	16.6	101	320	4	MOLECULE:	BRO1	
42:00:00	7sjr-A	3.4	10.2	88	911	2	MOLECULE:	DNA HELICASE	
43:00:00	4rp5-A	3.4	6.5	67	94	10	MOLECULE:	DISKS LARGE 1 TUMOR SUPPRESSOR PROTEIN	
44:00:00	6zsi-D	3.4	5.6	100	133	4	MOLECULE:	RAS-RELATED PROTEIN RAB-8A	
45:00:00	8d8k-V	3.4	25.1	90	233	12	MOLECULE:	PROBABLE S-ADENOSYL-L-METHIONINE-DEPENDENT	RNA
46:00:00	8dqk-A	3.4	25.8	111	733	5	MOLECULE:	CELLULOSE SYNTHASE-LIKE CSLF6	
47:00:00	7e7i-A	3.4	20.1	105	2011	7	MOLECULE:	RETINAL-SPECIFIC PHOSPHOLIPID-TRANSPORTING	ATPASE
48:00:00	6gy6-A	3.4	12.4	90	365	6	MOLECULE:	XAXA	
49:00:00	7etw-A	3.4	4.4	51	197	8	MOLECULE:	INSULIN-INDUCED GENE 2 PROTEIN	
50:00:00	8bh8-A	3.4	10	93	523	3	MOLECULE:	PCIF1_WW DOMAIN-CONTAINING PROTEIN	
51:00:00	6wge-C	3.4	32.8	76	174	13	MOLECULE:	STRUCTURAL MAINTENANCE OF CHROMOSOMES PROTE	IN 1A
52:00:00	4wpx-B	3.4	10.2	56	81	14	MOLECULE:	CELL DIVISION CONTROL PROTEIN 31-LIKE PROTE	IN
53:00:00	6yvd-D	3.4	21.8	89	414	6	MOLECULE:	CONDENSIN COMPLEX SUBUNIT 2	
54:00:00	5bw9-g	3.4	22.6	87	141	8	MOLECULE:	V-TYPE PROTON ATPASE CATALYTIC SUBUNIT A	
55:00:00	6xp5-D	3.4	25.4	97	134	8	MOLECULE:	MEDIATOR OF RNA POLYMERASE II TRANSCRIPTION	SUBUN
56:00:00	5wah-A	3.4	2.6	56	99	4	MOLECULE:	IGA FC RECEPTOR	
57:00:00	8j3r-B	3.4	6.1	78	387	9	MOLECULE:	TRANSPOSASE IS605 ORFB C-TERMINAL DOMAIN-CO	NTAINI
58:00:00	6e1k-A	3.4	4.5	66	536	5	MOLECULE:	TWO PORE CALCIUM CHANNEL PROTEIN 1	
59:00:00	8wjg-A	3.4	10.6	72	533	7	MOLECULE:	SOLUTE CARRIER FAMILY 22 MEMBER 12	
60:00:00	7x07-A	3.4	14.5	143	633	7	MOLECULE:	ATP-BINDING CASSETTE SUB-FAMILY D MEMBER 1	
61:00:00	3h7x-E	3.4	3.5	46	53	4	MOLECULE:	ADHESIN YADA	
62:00:00	6wbp-A	3.4	2.7	47	56	9	MOLECULE:	SEPTIN-6	
63:00:00	6hk5-B	3.4	3.1	44	66	0	MOLECULE:	COOJ	
64:00:00	5mw9-H	3.4	3.2	48	57	4	MOLECULE:	CENTROSOMIN	
65:00:00	6fpr-B	3.4	3.4	49	58	4	MOLECULE:	SIGNAL RECOGNITION PARTICLE RECEPTOR FTSY	
66:00:00	1n73-C	3.4	13	56	322	4	MOLECULE:	FIBRIN ALPHA-1 CHAIN	
67:00:00	6wkr-C	3.4	21.7	85	607	9	MOLECULE:	UBIQUITIN	
68:00:00	1ifk-A	3.4	3	44	51	7	MOLECULE:	INOVIRUS	
69:00:00	4yv4-C	3.4	3.2	48	57	4	MOLECULE:	SPINDLE ASSEMBLY ABNORMAL PROTEIN 5	
70:00:00	1gl2-D	3.4	3.1	45	54	4	MOLECULE:	ENDOBREVIN	
71:00:00	3rk3-E	3.4	2.2	41	50	10	MOLECULE:	VAMP2	
72:00:00	3wmi-A	3.4	3.2	50	57	4	MOLECULE:	EIAV GP45 WILD TYPE	
73:00:00	7a9w-A	3.4	15.8	70	513	4	MOLECULE:	PROTEIN RMD9, MITOCHONDRIAL	
74:00:00	6lth-Q	3.4	2.7	49	105	4	MOLECULE:	TRANSCRIPTION ACTIVATOR BRG1	
75:00:00	3jb9-r	3.4	2.4	40	573	0	MOLECULE:	PRE-MRNA-SPLICING FACTOR SPP42	
76:00:00	3bhp-C	3.4	5.7	50	54	8	MOLECULE:	UPF0291 PROTEIN YNZC	
77:00:00	4v1a-o	3.4	3.6	49	94	8	MOLECULE:	MITORIBOSOMAL PROTEIN ML37, MRPL37	
78:00:00	7asm-W	3.4	4	51	66	12	MOLECULE:	50S RIBOSOMAL PROTEIN L19	
79:00:00	6nd4-Q	3.4	6.6	57	862	11	MOLECULE:	ETS RRNA	
80:00:00	1eq7-A	3.4	3.5	47	56	4	MOLECULE:	OUTER MEMBRANE LIPOPROTEIN	
81:00:00	7vv6-R	3.3	24.7	71	271	6	MOLECULE:	MAS-RELATED G-PROTEIN COUPLED RECEPTOR MEMB	ER X2
82:00:00	6ted-R	3.3	23.7	88	244	6	MOLECULE:	DNA-DIRECTED RNA POLYMERASE SUBUNIT	
83:00:00	8ga7-A	3.3	30.3	137	503	9	MOLECULE:	THR5	
84:00:00	5j1f-A	3.3	32.9	92	188	12	MOLECULE:	PLECTIN,PLECTIN	
85:00:00	8dl7-A	3.3	29.4	129	467	7	MOLECULE:	SOLUTE CARRIER FAMILY 40 MEMBER 1	
86:00:00	6m6z-A	3.3	31.4	103	203	5	MOLECULE:	TMH4C4	
87:00:00	7rz4-A	3.3	18.7	98	993	3	MOLECULE:	GLUTAMATE RECEPTOR 2	
88:00:00	6i3m-E	3.3	29	102	354	4	MOLECULE:	TRANSLATION INITIATION FACTOR EIF-2B SUBUNI	T ALPH
89:00:00	4gd3-A	3.3	6	106	179	5	MOLECULE:	HYDROGENASE-1 SMALL CHAIN	
90:00:00	1n1c-A	3.3	7.7	79	199	8	MOLECULE:	TORA SPECIFIC CHAPERONE	
91:00:00	8th8-B	3.3	37.2	126	276	10	MOLECULE:	DYNEIN REGULATORY COMPLEX PROTEIN 1/2 N-TER	MINAL
92:00:00	1oks-A	3.3	1.7	39	53	8	MOLECULE:	RNA POLYMERASE ALPHA SUBUNIT	
93:00:00	8pnl-C	3.3	9.7	80	486	5	MOLECULE:	PUTATIVE TRIACYLGLYCERIDE TRANSPORTER	
94:00:00	7nsb-7	3.3	17	117	531	9	MOLECULE:	VACUOLAR IMPORT AND DEGRADATION PROTEIN 30	
95:00:00	5lbm-C	3.3	6.1	77	90	10	MOLECULE:	TRANSCRIPTIONAL REPRESSOR FRMR	
96:00:00	9c7u-C	3.3	7.5	70	205	4	MOLECULE:	NICALIN	
97:00:00	2oar-A	3.3	18.2	86	125	6	MOLECULE:	LARGE-CONDUCTANCE MECHANOSENSITIVE CHANNEL	
98:00:00	8xh6-A	3.3	4.7	75	161	1	MOLECULE:	LATENT MEMBRANE PROTEIN 1	
99:00:00	6cfz-I	3.3	30.3	86	106	2	MOLECULE:	ASK1	
0:00	8rot-A	3.3	29.4	86	897	1	MOLECULE:	CHAT DOMAIN-CONTAINING PROTEIN	
1:00	2n12-A	3.3	4.3	57	82	5	MOLECULE:	UNCONVENTIONAL MYOSIN-VI	
2:00	1ux5-A	3.3	21	105	411	3	MOLECULE:	BNI1 PROTEIN	
3:00	7byl-A	3.3	29	114	354	8	MOLECULE:	GREEN FLUORESCENT PROTEIN,POTASSIUM VOLTAGE	#NAME?

4:00	8b6l-M	3.3	6	86	110	6	MOLECULE:	PROTEIN TRANSPORT PROTEIN SEC61 SUBUNIT ALP	HA ISO
5:00	2p4w-B	3.3	8.5	64	198	8	MOLECULE:	TRANSCRIPTIONAL REGULATORY PROTEIN ARSR FAM	ILY
6:00	6z2w-E	3.3	24.7	87	2325	1	MOLECULE:	DNA DAMAGE CHECKPOINT PROTEIN LCD1	
7:00	8ftk-A	3.3	24.5	122	1584	4	MOLECULE:	5'-3' RNA HELICASE-LIKE PROTEIN	
8:00	7u8g-A	3.3	5.6	86	260	5	MOLECULE:	EGFP, CYTOCHROME B-245 HEAVY CHAIN CHIMERA	
9:00	3trc-A	3.3	4.2	48	168	2	MOLECULE:	PHOSPHOENOLPYRUVATE-PROTEIN PHOSPHOTRANSFEF	ASE
10:00	3hef-B	3.3	8	77	130	12	MOLECULE:	GENE 1 PROTEIN	
11:00	7mgq-A	3.3	16.9	99	602	6	MOLECULE:	5-AMINOIMIDAZOLE-4-CARBOXAMIDE RIBONUCLEOTI	DE
12:00	6b85-A	3.3	7.4	101	215	5	MOLECULE:	TMHC4_R	
13:00	6xns-B	3.3	9.1	85	333	8	MOLECULE:	C3_CROWN-05	
14:00	3p8c-F	3.3	9.5	104	156	3	MOLECULE:	CYTOPLASMIC FMR1-INTERACTING PROTEIN 1	
15:00	5k47-A	3.3	21.1	81	484	7	MOLECULE:	POLYCYSTIN-2	
16:00	8smr-G	3.3	20.3	133	312	5	MOLECULE:	UBIQUINOL-CYTOCHROME C REDUCTASE IRON-SULFU	R SUBU
17:00	5da9-B	3.3	9.3	92	400	5	MOLECULE:	PUTATIVE UNCHARACTERIZED PROTEIN,PUTATIVE U	NCHARA
18:00	6mpb-A	3.3	13.8	81	447	7	MOLECULE:	NEUTRAL AMINO ACID TRANSPORTER B(0)	
19:00	7zu0-F	3.3	4.5	56	112	7	MOLECULE:	E3 UBIQUITIN-PROTEIN LIGASE PEP5	
20:00	6nd4-P	3.3	22.5	79	359	6	MOLECULE:	ETS RRNA	
21:00	1gnc-A	3.3	5.5	68	175	6	MOLECULE:	GRANULOCYTE COLONY-STIMULATING FACTOR	
22:00	6whb-A	3.3	10.8	71	284	8	MOLECULE:	MITOGEN-ACTIVATED PROTEIN KINASE KINASE KIN	ASE 1
23:00	7d2h-D	3.3	2.4	49	60	6	MOLECULE:	LIPRIN-ALPHA-2	
24:00:00	6cfz-G	3.3	2.9	45	56	4	MOLECULE:	ASK1	
25:00:00	2x7r-C	3.3	3.5	48	58	6	MOLECULE:	TRANSMEMBRANE PROTEIN GP41	
26:00:00	3e7k-H	3.3	3.8	46	56	2	MOLECULE:	TRPM7 CHANNEL	
27:00:00	3uf1-D	3.3	3.7	48	112	10	MOLECULE:	VIMENTIN	
28:00:00	6fia-D	3.3	4	50	104	12	MOLECULE:	LINE-1 RETROTRANSPOSABLE ELEMENT ORF1 PROTE	IN
29:00:00	4i1l-A	3.3	3	51	62	2	MOLECULE:	FORKHEAD BOX PROTEIN P3	
30:00:00	5oxf-A	3.3	3.8	57	703	12	MOLECULE:	GTP-BINDING PROTEIN	
31:00:00	3swf-B	3.3	3.5	48	58	4	MOLECULE:	CGMP-GATED CATION CHANNEL ALPHA-1	
32:00:00	7bb3-A	3.3	3.4	48	54	8	MOLECULE:	SURVIVAL MOTOR NEURON-LIKE PROTEIN 1,SURVIV	AL MOT
33:00:00	5xfs-A	3.3	2.8	49	78	4	MOLECULE:	PE FAMILY PROTEIN PE8	
34:00:00	1urq-A	3.3	4.2	44	57	5	MOLECULE:	M-TOMOSYN ISOFORM	
35:00:00	4m3l-A	3.3	2.5	44	60	9	MOLECULE:	E3 UBIQUITIN-PROTEIN LIGASE TRIM63	
36:00:00	5mz6-1	3.3	9.7	71	1018	6	MOLECULE:	SEPARASE	
37:00:00	7xad-C	3.3	2.6	53	96	15	MOLECULE:	PROGRAMMED CELL DEATH 1 LIGAND 1	
38:00:00	7v2w-J	3.3	41.3	113	236	4	MOLECULE:	THO COMPLEX SUBUNIT HPR1	
39:00:00	3bvo-A	3.3	4.1	48	197	19	MOLECULE:	CO-CHAPERONE PROTEIN HSCB, MITOCHONDRIAL PR	ECURSO
40:00:00	6jzt-A	3.3	4	57	402	9	MOLECULE:	FLAGELLAR HOOK PROTEIN FLGE	
41:00:00	4tko-B	3.3	26.9	98	326	13	MOLECULE:	EMRA	
42:00:00	2c5i-T	3.3	3.3	51	94	16	MOLECULE:	T-SNARE AFFECTING A LATE GOLGI COMPARTMENT	
43:00:00	6kg7-A	3.3	15.4	66	1817	2	MOLECULE:	PIEZO-TYPE MECHANOSENSITIVE ION CHANNEL COM	PONENT
44:00:00	6nr8-4	3.3	3.5	61	104	8	MOLECULE:	PREFOLDIN SUBUNIT 1	
45:00:00	7z6q-c	3.3	8.3	77	121	1	MOLECULE:	PHOTOSYSTEM P840 REACTION CENTER, LARGE SUB	UNIT
46:00:00	8hpo-H	3.2	52.3	110	233	9	MOLECULE:	TRANSCRIPTIONAL REGULATORY PROTEIN UME1	
47:00:00	9gm9-A	3.2	48	180	999	4	MOLECULE:	CHROMOSOME PARTITION PROTEIN MUKB	
48:00:00	6h95-A	3.2	30	91	225	4	MOLECULE:	ALBICIDIN RESISTANCE PROTEIN	
49:00:00	5v7p-A	3.2	21.4	74	281	5	MOLECULE:	PROTEIN-S-ISOPRENYLCYSTEINE O-METHYLTRANSFE	RASE
50:00:00	5lnc-B	3.2	20.6	102	367	9	MOLECULE:	VACUOLAR TRANSPORTER CHAPERONE 4,CORE HISTO	NE MAC
51:00:00	1e1d-A	3.2	30	99	553	2	MOLECULE:	HYDROXYLAMINE REDUCTASE	
52:00:00	5n9j-A	3.2	27.8	108	566	10	MOLECULE:	MEDIATOR OF RNA POLYMERASE II TRANSCRIPTION	SUBUN
53:00:00	8eki-D	3.2	23.4	114	684	4	MOLECULE:	PROTEIN TRANSPORT PROTEIN SEC20	
54:00:00	5n9j-Z	3.2	22.6	111	119	5	MOLECULE:	MEDIATOR OF RNA POLYMERASE II TRANSCRIPTION	SUBUN
55:00:00	8qz7-A	3.2	11.6	92	333	5	MOLECULE:	ISOFORM 2 OF CERAMIDE SYNTHASE 6	
56:00:00	3hr0-A	3.2	6.9	101	250	6	MOLECULE:	COG4	
57:00:00	3nym-A	3.2	4.2	69	124	3	MOLECULE:	UNCHARACTERIZED PROTEIN	
58:00:00	8h38-C	3.2	19.9	72	407	8	MOLECULE:	COP9 SIGNALOSOME COMPLEX SUBUNIT 1	
59:00:00	7tmr-F	3.2	9	87	478	5	MOLECULE:	H(+)-TRANSPORTING TWO-SECTOR ATPASE	
60:00:00	3qz0-B	3.2	1.3	41	79	2	MOLECULE:	FACTOR H BINDING PROTEIN	
61:00:00	8d0b-F	3.2	32.8	111	915	3	MOLECULE:	CST COMPLEX SUBUNIT CTC1	
62:00:00	7yjm-C	3.2	3.4	41	48	5	MOLECULE:	ATLCB1	
63:00:00	6r7n-B	3.2	15.7	83	443	12	MOLECULE:	COP9 SIGNALOSOME COMPLEX SUBUNIT 1	
64:00:00	1un8-A	3.2	20.5	61	542	13	MOLECULE:	DIHYDROXYACETONE KINASE	
65:00:00	2xqh-A	3.2	24.9	65	258	12	MOLECULE:	IMMUNOGLOBULIN-BINDING PROTEIN EIBD	
66:00:00	8iqg-A	3.2	6.7	64	213	6	MOLECULE:	CHROMATIN ASSEMBLY FACTOR 1 SUBUNIT A	
67:00:00	4tsh-A	3.2	4.4	57	121	12	MOLECULE:	SURFACE PROTEIN ADHESIN	
68:00:00	7pkn-R	3.2	10.9	57	80	18	MOLECULE:	CENTROMERE PROTEIN H	
69:00:00	3dxq-A	3.2	10.5	57	294	7	MOLECULE:	CHOLINE/ETHANOLAMINE KINASE FAMILY PROTEIN	
70:00:00	4dvy-P	3.2	3.2	78	656	6	MOLECULE:	CYTOTOXICITY-ASSOCIATED IMMUNODOMINANT ANTI	GEN
71:00:00	8h1l-B	3.2	4.5	43	423	7	MOLECULE:	N-ACYLGLUCOSAMINE 2-EPIMERASE	
72:00:00	6cj6-D	3.2	4	51	166	10	MOLECULE:	PROTEIN F9	
73:00:00	1kw2-A	3.2	6.5	68	455	6	MOLECULE:	VITAMIN D-BINDING PROTEIN	
74:00:00	6ddq-A	3.2	25.1	99	507	5	MOLECULE:	CYTOSOLIC PURINE 5'-NUCLEOTIDASE	
75:00:00	8qfc-C	3.2	15.9	91	380	5	MOLECULE:	60S RIBOSOMAL PROTEIN L10A	
76:00:00	8iht-N	3.2	9	58	295	7	MOLECULE:	HISTONE H3	
77:00:00	5vgz-D	3.2	9.9	66	107	2	MOLECULE:	26S PROTEASOME REGULATORY SUBUNIT 7	
78:00:00	7cy6-A	3.2	4.9	67	872	4	MOLECULE:	MALTODEXTRIN-BINDING PROTEIN,5-METHYLCYTOSI	NE-MOD
79:00:00	6rw3-B	3.2	4.4	62	439	15	MOLECULE:	HEXOSE TRANSPORTER 1	
80:00:00	6vq6-A	3.2	2.6	51	600	6	MOLECULE:	ATPASE H+-TRANSPORTING V1 SUBUNIT A	
81:00:00	7o4i-1	3.2	5.1	60	522	3	MOLECULE:	GENERAL TRANSCRIPTION AND DNA REPAIR FACTOR	IIH H
82:00:00	7qhm-I	3.2	14.7	98	135	7	MOLECULE:	CYTOCHROME BC1 COMPLEX RIESKE IRON-SULFUR S	UBUNIT
83:00:00	7v2y-B	3.2	32	108	1226	6	MOLECULE:	THO COMPLEX SUBUNIT HPR1	
84:00:00	8jh7-D	3.2	4.1	89	431	4	MOLECULE:	ANTI-BRIL FAB HEAVY CHAIN	
85:00:00	2ets-A	3.2	4.2	68	115	4	MOLECULE:	HYPOTHETICAL PROTEIN	
86:00:00	5doq-B	3.2	10.7	88	330	6	MOLECULE:	BD-TYPE QUINOL OXIDASE SUBUNIT I	
87:00:00	8yjb-B	3.2	13.3	86	1061	3	MOLECULE:	26S PROTEASOME COMPLEX SUBUNIT DSS1	
88:00:00	7wrg-A	3.2	17.6	112	812	7	MOLECULE:	KINESIN-LIKE PROTEIN	
89:00:00	7pde-A	3.2	18.1	125	846	6	MOLECULE:	ADENYLATE CYCLASE 9	
90:00:00	8d8k-M	3.2	3.8	50	83	10	MOLECULE:	PROBABLE S-ADENOSYL-L-METHIONINE-DEPENDENT	RNA
91:00:00	7jmn-O	3.2	3.4	59	164	7	MOLECULE:	MEDIATOR OF RNA POLYMERASE II TRANSCRIPTION	SUBUN
92:00:00	8tww-A	3.2	11.1	81	245	6	MOLECULE:	AETD	
93:00:00	6zxr-A	3.2	3.3	52	212	8	MOLECULE:	ER LUMEN PROTEIN-RETAINING RECEPTOR 2	
94:00:00	1qhh-C	3.2	2.9	48	102	2	MOLECULE:	PROTEIN (PCRA (SUBUNIT))	
95:00:00	7d7n-A	3.2	16.6	156	704	6	MOLECULE:	ATP-BINDING CASSETTE SUB-FAMILY B MEMBER 6,	MITOC
96:00:00	7u5d-A	3.2	24.1	73	630	5	MOLECULE:	CRRNA	
97:00:00	7ux0-A	3.2	2.4	50	115	0	MOLECULE:	SPERM-EGG FUSION PROTEIN TMEM95	
98:00:00	4orh-H	3.2	5.6	76	142	9	MOLECULE:	UBIQUITIN-CONJUGATING ENZYME E2 VARIANT 2	
99:00:00	4hkr-A	3.2	8.7	94	165	9	MOLECULE:	CALCIUM RELEASE-ACTIVATED CALCIUM CHANNEL P	ROTEIN
0:00	6br8-A	3.2	10.1	66	249	5	MOLECULE:	PROTEIN A6 HOMOLOG	
1:00	8tkp-A	3.2	18.4	65	614	3	MOLECULE:	TRANSMEMBRANE CHANNEL-LIKE PROTEIN 2	
2:00	7t7r-A	3.2	23.2	115	1132	8	MOLECULE:	PROTEIN UNC-13 HOMOLOG A	
3:00	7dgy-A	3.2	2.6	47	99	0	MOLECULE:	C2	
4:00	6bpz-A	3.2	4.8	66	900	2	MOLECULE:	PIEZO-TYPE MECHANOSENSITIVE ION CHANNEL COM	PONENT

5:00	2w6a-A	3.2	3.1	47	61	11	MOLECULE:	ARF GTPASE-ACTIVATING PROTEIN GIT1	
6:00	5vpe-A	3.2	2.7	46	67	15	MOLECULE:	PROTEIN FOSB	
7:00	1gmj-A	3.2	3.2	50	65	10	MOLECULE:	ATPASE INHIBITOR	
8:00	5ncn-B	3.2	2.3	44	76	2	MOLECULE:	DBF2 KINASE ACTIVATOR PROTEIN MOB1	
9:00	6rfq-3	3.2	5.5	58	108	3	MOLECULE:	SUBUNIT NUAM OF NADH:UBIQUINONE OXIDOREDUCT	ASE (C
10:00	4dl0-K	3.2	14	56	97	18	MOLECULE:	V-TYPE PROTON ATPASE SUBUNIT C	
11:00	8gkv-A	3.2	2.4	41	44	5	MOLECULE:	ANTI-ADAPTER PROTEIN IRAP	
12:00	5chx-A	3.2	4.2	52	210	2	MOLECULE:	XRCC4-MYH7-1590-1657	
13:00	5dw7-A	3.2	3.2	52	309	6	MOLECULE:	GERMACRADIENOL/GEOSMIN SYNTHASE	
14:00	7jw1-e	3.2	6.3	102	235	3	MOLECULE:	CAPSID PROTEINS	
15:00	7x6g-D	3.2	11.5	69	152	7	MOLECULE:	QUORUM-SENSING REGULATOR PROTEIN G	
16:00	5j1i-A	3.2	3.1	55	346	9	MOLECULE:	PLECTIN	
17:00	3aei-A	3.2	4.5	55	94	13	MOLECULE:	PREFOLDIN BETA SUBUNIT 2	
18:00	5i08-A	3.2	7.8	72	958	6	MOLECULE:	SPIKE GLYCOPROTEIN, ENVELOPE GLYCOPROTEIN C	HIMERA
19:00	8k9c-A	3.2	8.2	74	207	7	MOLECULE:	CAPRIN-2	
20:00	7sfm-A	3.1	26.2	93	472	5	MOLECULE:	HIP1	
21:00	7eew-A	3.1	31.7	87	611	7	MOLECULE:	TYPE I RESTRICTION-MODIFICATION SYSTEM METH	YLTRAN
22:00	6vej-A	3.1	31.7	141	1022	9	MOLECULE:	PROBABLE RESISTANCE-NODULATION-CELL DIVISIO	N (RND
23:00	6wb9-2	3.1	31.9	85	292	4	MOLECULE:	ENDOPLASMIC RETICULUM MEMBRANE PROTEIN COMP	LEX SU
24:00:00	5t58-D	3.1	36.6	113	201	5	MOLECULE:	KLLA0F02343P	
25:00:00	4qfh-B	3.1	26.8	121	611	7	MOLECULE:	GLUCOSE-6-PHOSPHATE ISOMERASE	
26:00:00	7tpg-B	3.1	32.5	128	403	7	MOLECULE:	PUTATIVE CELL SURFACE POLYSACCHARIDE POLYME	RASE/L
27:00:00	5lc5-n	3.1	26.4	78	166	9	MOLECULE:	NADH-UBIQUINONE OXIDOREDUCTASE CHAIN 3	
28:00:00	8at3-D	3.1	31.8	144	666	5	MOLECULE:	HAUS AUGMIN-LIKE COMPLEX SUBUNIT 1	
29:00:00	6uz3-A	3.1	29	137	1126	2	MOLECULE:	SODIUM CHANNEL PROTEIN TYPE 5 SUBUNIT ALPHA	,GREEN
30:00:00	6klb-A	3.1	33.8	144	1118	10	MOLECULE:	LBCAS12A	
31:00:00	8zxd-A	3.1	10.7	64	388	5	MOLECULE:	VANG-LIKE PROTEIN 1	
32:00:00	1y2o-A	3.1	6.4	98	248	3	MOLECULE:	BAI1-ASSOCIATED PROTEIN 2 ISOFORM 1	
33:00:00	7obq-y	3.1	18.9	85	454	2	MOLECULE:	SRP RNA	
34:00:00	7yh5-B	3.1	8.4	60	177	8	MOLECULE:	NUCLEOSIDE TRIPHOSPHATE PYROPHOSPHOHYDROLAS	E
35:00:00	2xra-A	3.1	6.5	105	203	3	MOLECULE:	TRANSMEMBRANE PROTEIN GP41	
36:00:00	6lo8-D	3.1	6.6	83	119	7	MOLECULE:	MITOCHONDRIAL IMPORT INNER MEMBRANE TRANSLO	CASE S
37:00:00	6t9i-H	3.1	20.3	63	227	5	MOLECULE:	TRANSCRIPTION FACTOR SPT20	
38:00:00	8syn-A	3.1	18.3	69	801	0	MOLECULE:	VPS35 ENDOSOMAL PROTEIN-SORTING FACTOR-LIKE	
39:00:00	6ypc-W	3.1	5.5	53	69	4	MOLECULE:	INNER KINETOCHORE SUBUNIT MCM22	
40:00:00	2luh-A	3.1	2.4	52	167	2	MOLECULE:	VACUOLAR PROTEIN SORTING-ASSOCIATED PROTEIN	VTA1
41:00:00	2nrk-A	3.1	3	42	165	17	MOLECULE:	HYPOTHETICAL PROTEIN GRPB	
42:00:00	7a6h-K	3.1	2.1	49	107	10	MOLECULE:	DNA-DIRECTED RNA POLYMERASE III SUBUNIT RPC	1
43:00:00	8z9y-B	3.1	2.3	52	164	6	MOLECULE:	PROTEIN TIC 214	
44:00:00	9asc-A	3.1	10.2	101	318	9	MOLECULE:	UNDECAPRENYL-PHOSPHATE 4-DEOXY-4-FORMAMIDO-	L-ARAB
45:00:00	8d2s-A	3.1	9.3	105	475	5	MOLECULE:	SODIUM-DEPENDENT LYSOPHOSPHATIDYLCHOLINE SY	MPORTE
46:00:00	8kc4-A	3.1	12.5	68	151	9	MOLECULE:	DE NOVO DESIGN PROTEIN -NA05	
47:00:00	3p3o-A	3.1	6.1	74	416	4	MOLECULE:	CYTOCHROME P450	
48:00:00	6dfk-I	3.1	10	62	236	8	MOLECULE:	SUBUNIT OF PROTEASEOME ACTIVATOR COMPLEX,PU	TATIVE
49:00:00	6kko-A	3.1	7	65	170	2	MOLECULE:	PUTATIVE SERINE PHOSPHATASE	
50:00:00	8h2h-D	3.1	16.5	69	599	4	MOLECULE:	LTRB	
51:00:00	6n7p-X	3.1	14.1	112	826	4	MOLECULE:	U1 SMALL NUCLEAR RIBONUCLEOPROTEIN 70 KDA H	OMOLOG
52:00:00	3f46-A	3.1	9.6	66	345	3	MOLECULE:	5,10-METHENYLTETRAHYDROMETHANOPTERIN HYDROG	ENASE
53:00:00	1k30-A	3.1	21.2	82	363	2	MOLECULE:	GLYCEROL-3-PHOSPHATE ACYLTRANSFERASE	
54:00:00	6qg0-G	3.1	8.7	72	355	6	MOLECULE:	TRANSLATION INITIATION FACTOR EIF-2B SUBUNI	T ALPH
55:00:00	6ln2-A	3.1	8.4	76	437	11	MOLECULE:	GLUCAGON-LIKE PEPTIDE 1 RECEPTOR,RUBREDOXIN	,GLUCA
56:00:00	9na9-E	3.1	6.9	80	153	5	MOLECULE:	AUGMIN SUBUNIT 1	
57:00:00	7bw0-R	3.1	6.3	111	280	6	MOLECULE:	SOLUBLE CYTOCHROME B562,G-PROTEIN COUPLED B	ILE AC
58:00:00	3s0x-A	3.1	11.5	75	225	4	MOLECULE:	PEPTIDASE A24B, FLAK DOMAIN PROTEIN	
59:00:00	5xyv-C	3.1	4.3	45	52	2	MOLECULE:	RHINO	
60:00:00	7y22-M	3.1	12.4	95	215	5	MOLECULE:	PHAGE CONNECTOR PROTEIN	
61:00:00	8hf3-A	3.1	6.9	98	295	7	MOLECULE:	PALMITOYLTRANSFERASE ZDHHC9	
62:00:00	7nad-5	3.1	13.2	70	123	11	MOLECULE:	25S RRNA	
63:00:00	7nh9-A	3.1	11.4	113	327	4	MOLECULE:	CMAX PROTEIN	
64:00:00	3hh0-A	3.1	5.9	65	134	8	MOLECULE:	TRANSCRIPTIONAL REGULATOR, MERR FAMILY	
65:00:00	6h2x-A	3.1	24.7	141	352	4	MOLECULE:	CHROMOSOME PARTITION PROTEIN MUKB,CHROMOSOM	E PART
66:00:00	9bkv-A	3.1	19.2	92	788	1	MOLECULE:	OXYGEN SENSOR PROTEIN DOSP	
67:00:00	6qld-Y	3.1	13.2	113	223	6	MOLECULE:	INNER KINETOCHORE SUBUNIT MIF2	
68:00:00	7mi8-A	3.1	20.5	132	868	4	MOLECULE:	FUSION PROTEIN OF DYNEIN AND ENDOLYSIN	
69:00:00	3vpq-A	3.1	4.2	70	203	6	MOLECULE:	GLUTATHIONE S-TRANSFERASE SIGMA	
70:00:00	2ymb-D	3.1	7.1	69	231	3	MOLECULE:	MIT DOMAIN-CONTAINING PROTEIN 1	
71:00:00	6tlj-S	3.1	18.7	66	293	3	MOLECULE:	ANAPHASE-PROMOTING COMPLEX SUBUNIT 1	
72:00:00	7wnq-A	3.1	5.4	67	368	3	MOLECULE:	GUARD CELL S-TYPE ANION CHANNEL SLAC1	
73:00:00	6vm0-A	3.1	6.6	97	363	3	MOLECULE:	GLYCINE RECEPTOR SUBUNIT ALPHAZ1	
74:00:00	8qbd-A	3.1	12.5	110	272	2	MOLECULE:	SPHINGOLIPID LONG CHAIN BASE-RESPONSIVE PRO	TEIN P
75:00:00	8uic-T	3.1	22.5	114	803	7	MOLECULE:	INTEGRATOR COMPLEX SUBUNIT 11	
76:00:00	8bsh-C	3.1	9.8	58	794	7	MOLECULE:	PROTEIN TRANSPORT PROTEIN SEC23	
77:00:00	2ilk-A	3.1	2.3	40	155	13	MOLECULE:	INTERLEUKIN-10	
78:00:00	5d80-G	3.1	24.9	87	223	5	MOLECULE:	V-TYPE PROTON ATPASE CATALYTIC SUBUNIT A	
79:00:00	8dke-P	3.1	7.4	62	333	3	MOLECULE:	ISOFORM 2 OF CYSTINOSIN	
80:00:00	8szh-A	3.1	6.7	103	836	5	MOLECULE:	EXTRACELLULAR CALCIUM-SENSING RECEPTOR	
81:00:00	2lor-A	3.1	5.4	60	108	3	MOLECULE:	TRANSMEMBRANE PROTEIN 141	
82:00:00	5xpd-A	3.1	10.8	64	269	13	MOLECULE:	SUGAR TRANSPORTER	
83:00:00	6pe4-A	3.1	13.4	97	758	2	MOLECULE:	V-TYPE PROTON ATPASE SUBUNIT A, VACUOLAR IS	OFORM
84:00:00	7r5s-T	3.1	13	69	112	4	MOLECULE:	CENTROMERE PROTEIN H	
85:00:00	7yil-B	3.1	9.5	58	94	9	MOLECULE:	GINS	
86:00:00	6xrx-A	3.1	7.6	51	551	0	MOLECULE:	MALTOSE/MALTODEXTRIN-BINDING PERIPLASMIC PR	OTEIN,
87:00:00	7qii-B	3.1	8	63	76	2	MOLECULE:	CHAPERONE PROTEIN YSCY	
88:00:00	9go3-A	3.1	25.2	93	253	5	MOLECULE:	MECHANOSENSITIVE CHANNEL PROTEIN	
89:00:00	1lqs-L	3.1	7.9	69	142	3	MOLECULE:	INTERLEUKIN-10 RECEPTOR ALPHA CHAIN	
90:00:00	7jmn-P	3.1	22	79	810	5	MOLECULE:	MEDIATOR OF RNA POLYMERASE II TRANSCRIPTION	SUBUN
91:00:00	6jql-A	3.1	16.5	104	678	9	MOLECULE:	BIFUNCTIONAL PROTEIN PAAZ	
92:00:00	4k2u-A	3.1	10.5	80	230	6	MOLECULE:	ERYTHROCYTE BINDING ANTIGEN 175	
93:00:00	6poo-A	3.1	9.6	102	273	8	MOLECULE:	BIBA	
94:00:00	5odw-D	3.1	4	65	198	6	MOLECULE:	FERRIPYOVERDINE RECEPTOR	
95:00:00	7etw-B	3.1	22.6	92	483	3	MOLECULE:	INSULIN-INDUCED GENE 2 PROTEIN	
96:00:00	3cl3-D	3.1	3.1	47	59	4	MOLECULE:	ORF K13	
97:00:00	4tt0-B	3.1	17.4	82	131	4	MOLECULE:	DENEDDYLASE	
98:00:00	2xco-A	3.1	10.5	63	636	5	MOLECULE:	DNA GYRASE SUBUNIT B, DNA GYRASE SUBUNIT A	
99:00:00	3wuv-Q	3.1	4.8	47	62	4	MOLECULE:	CENTROSOMAL PROTEIN OF 55 KDA	
0:00	5c9n-A	3.1	3.9	53	64	6	MOLECULE:	GEMININ COILED-COIL DOMAIN-CONTAINING PROTE	IN 1
1:00	6wxu-B	3.1	4.5	48	273	4	MOLECULE:	DUAL OXIDASE 1	
2:00	5fqf-A	3.1	2.5	46	583	11	MOLECULE:	BETA-N-ACETYLGALACTOSAMINIDASE	
3:00	7k36-D	3.1	2.6	55	74	4	MOLECULE:	SERINE/THREONINE-PROTEIN PHOSPHATASE 2A 65	KDA RE
4:00	8cqn-B	3.1	14.4	63	187	6	MOLECULE:	LIPOPROTEIN, PUTATIVE	
5:00	7ug2-A	3.1	1.6	45	49	2	MOLECULE:	TRIPARTITE MOTIF-CONTAINING PROTEIN 75	

6:00	1zhc-A	3.1	4.2	56	76	13	MOLECULE:	HYPOTHETICAL PROTEIN HP1242		
7:00	5l4k-V	3.1	4.1	52	293	4	MOLECULE:	26S PROTEASOME NON-ATPASE REGULATORY SUBUNI	T 4	
8:00	3jcm-I	3.1	21.6	91	416	5	MOLECULE:	PRE-MRNA-SPLICING FACTOR 8		
9:00	4mt8-A	3.1	3.4	51	100	4	MOLECULE:	ETHYLENE RESPONSE SENSOR 1		
10:00	7y1a-p	3.1	4.3	56	164	4	MOLECULE:	B-PHYCOERYTHRIN BETA CHAIN		
11:00	8b9z-K	3.1	5.6	51	96	8	MOLECULE:	NADH-UBIQUINONE OXIDOREDUCTASE CHAIN 3		
12:00	7qe5-A	3.1	5.1	56	616	5	MOLECULE:	SIALIC ACID TRAP TRANSPORTER PERMEASE PROTE	IN SIA	
13:00	2nps-D	3.1	3.1	50	63	2	MOLECULE:	VESICLE-ASSOCIATED MEMBRANE PROTEIN 4		
14:00	5dol-B	3.1	3.6	47	62	4	MOLECULE:	INITIATION-CONTROL PROTEIN YABA		
15:00	5ajs-B	3.1	3.5	52	64	17	MOLECULE:	THAP DOMAIN-CONTAINING PROTEIN 11		
16:00	8iw0-A	3	20.3	78	84	3	MOLECULE:	LIPRIN-BETA-1,KN MOTIF AND ANKYRIN REPEAT D	OMAIN-	
17:00	8c5v-I	3	94.5	188	516	7	MOLECULE:	CHEMOTAXIS PROTEIN CHEA		
18:00	5u1s-A	3	16.6	122	1488	9	MOLECULE:	SEPARIN		
19:00	1cm5-A	3	24.6	104	759	6	MOLECULE:	PROTEIN (PYRUVATE FORMATE-LYASE)		
20:00	8rt9-F	3	16.8	72	272	4	MOLECULE:	TRWJ PROTEIN		
21:00	7ni5-A	3	28.7	112	2791	6	MOLECULE:	SERINE-PROTEIN KINASE ATM		
22:00	8rhn-A	3	27.1	102	198	5	MOLECULE:	ATPASE FAMILY GENE 2 PROTEIN HOMOLOG A		
23:00	6g70-B	3	28.4	111	510	7	MOLECULE:	PRE-MRNA-PROCESSING FACTOR 39		
24:00:00	3cwz-B	3	6.1	87	310	8	MOLECULE:	RAS-RELATED PROTEIN RAB-6A		
25:00:00	8h2m-A	3	13.4	77	754	10	MOLECULE:	TAPE TAIL MEASURE PROTEIN		
26:00:00	3ufe-A	3	6.1	63	109	6	MOLECULE:	TRANSCRIPTIONAL ANTITERMINATOR (BGLG FAMILY	)	
27:00:00	3wvo-A	3	14	90	543	9	MOLECULE:	CRISPR-ASSOCIATED PROTEIN, CSE1 FAMILY		
28:00:00	2m64-A	3	3.4	50	231	10	MOLECULE:	PHLP5		
29:00:00	3r6n-A	3	31.9	141	450	4	MOLECULE:	DESMOPLAKIN		
30:00:00	7uqj-A	3	2.3	43	585	7	MOLECULE:	ATPASE HISTONE CHAPERONE YTA7		
31:00:00	7v2c-d	3	29.6	104	175	3	MOLECULE:	NADH DEHYDROGENASE [UBIQUINONE] FLAVOPROTEI	N 1,	
32:00:00	7w1m-D	3	28.2	119	967	6	MOLECULE:	STRUCTURAL MAINTENANCE OF CHROMOSOMES PROTE	IN 1A	
33:00:00	6bzf-A	3	11.2	77	197	4	MOLECULE:	SPORULATION-SPECIFIC PROTEIN 16		
34:00:00	5dlq-B	3	27.8	118	1052	9	MOLECULE:	EXPORTIN-4		
35:00:00	3erm-A	3	8.8	55	67	11	MOLECULE:	UNCHARACTERIZED CONSERVED PROTEIN		
36:00:00	3kdw-A	3	8	70	206	4	MOLECULE:	PUTATIVE SUGAR BINDING PROTEIN		
37:00:00	4ql6-B	3	2.7	43	509	2	MOLECULE:	CARBOXY-TERMINAL PROCESSING PROTEASE		
38:00:00	7emf-J	3	4.6	47	122	11	MOLECULE:	MEDIATOR OF RNA POLYMERASE II TRANSCRIPTION	SUBUN	
39:00:00	5vjx-J	3	3.2	51	64	8	MOLECULE:	CLOCK-INTERACTING PACEMAKER		
40:00:00	6zis-A	3	8.3	68	569	4	MOLECULE:	MALTOSE/MALTODEXTRIN-BINDING PERIPLASMIC PR	OTEIN,	
41:00:00	3sng-A	3	2.9	54	267	6	MOLECULE:	NUCLEASE		
42:00:00	7pp4-f	3	9.8	68	142	7	MOLECULE:	DNA-DIRECTED RNA POLYMERASE SUBUNIT ALPHA		
43:00:00	7ae8-A	3	6.4	59	152	3	MOLECULE:	HEPN TOXIN		
44:00:00	4hh0-A	3	8.7	65	383	6	MOLECULE:	APPA PROTEIN		
45:00:00	4ejo-A	3	6.1	66	112	8	MOLECULE:	TRANSCRIPTIONAL REGULATOR, PADR-LIKE FAMILY		
46:00:00	3w0l-B	3	7.3	67	598	6	MOLECULE:	GLUCOKINASE		
47:00:00	8ou0-C	3	5.6	63	153	11	MOLECULE:	TUBULIN BETA-4B CHAIN		
48:00:00	2rdp-A	3	10.7	82	140	7	MOLECULE:	PUTATIVE TRANSCRIPTIONAL REGULATOR MARR		
49:00:00	7uh4-A	3	3.4	84	110	4	MOLECULE:	LXG-ASSOCIATED ALPHA-HELICAL PROTEIN D2		
50:00:00	7ktr-E	3	13.2	106	124	6	MOLECULE:	TRANSFORMATION/TRANSCRIPTION DOMAIN-ASSOCIA	TED PR	
51:00:00	6ekr-A	3	4.4	51	303	8	MOLECULE:	TYPE II SITE-SPECIFIC DEOXYRIBONUCLEASE		
52:00:00	5cm8-A	3	6.7	97	419	2	MOLECULE:	RAL GUANINE NUCLEOTIDE DISSOCIATION STIMULA	TOR-LI	
53:00:00	4uj6-A	3	10	124	711	3	MOLECULE:	SURFACE LAYER PROTEIN		
54:00:00	8age-H	3	8.6	95	133	5	MOLECULE:	DOLICHYL-DIPHOSPHOOLIGOSACCHARIDE--PROTEIN		
55:00:00	6ffv-A	3	11.5	75	194	7	MOLECULE:	BTUM		
56:00:00	2iaz-B	3	15	93	113	9	MOLECULE:	HYPOTHETICAL PROTEIN SP1372		
57:00:00	7mop-A	3	30.5	102	2445	5	MOLECULE:	E3 UBIQUITIN-PROTEIN LIGASE HUWE1		
58:00:00	8cli-A	3	3.2	40	542	8	MOLECULE:	GENERAL TRANSCRIPTION FACTOR 3C POLYPEPTIDE		1
59:00:00	7xr2-a	3	2.9	45	274	2	MOLECULE:	VP3		
60:00:00	6l5d-B	3	7.6	84	244	8	MOLECULE:	GAS VESICLE PROTEIN		
61:00:00	7zvw-E	3	36.8	153	488	7	MOLECULE:	TRANSCRIPTION-ASSOCIATED PROTEIN		
62:00:00	6vbv-7	3	14.4	89	706	6	MOLECULE:	BARDET-BIEDL SYNDROME 18 PROTEIN		
63:00:00	8qyd-A	3	21.9	140	280	4	MOLECULE:	ANTI-PHAGE DEFENSE ZORAB SYSTEM ZORA		
64:00:00	7wjn-A	3	24.7	115	808	4	MOLECULE:	CHITIN SYNTHASE		
65:00:00	8q85-X	3	14.5	54	68	15	MOLECULE:	KINETOCHORE PROTEIN NDC80		
66:00:00	7xr2-B	3	17.4	84	846	7	MOLECULE:	VP3		
67:00:00	6z1b-B	3	2.2	44	202	7	MOLECULE:	HTH-TYPE TRANSCRIPTIONAL REGULATOR RUTR		
68:00:00	6m5r-B	3	20.1	104	689	4	MOLECULE:	TRIPARTITE TERMINASE SUBUNIT 3		
69:00:00	6rx4-B	3	7.2	85	379	4	MOLECULE:	CYTOCHROME BD-I UBIQUINOL OXIDASE SUBUNIT 1		
70:00:00	7rsl-A	3	9.8	68	235	4	MOLECULE:	SEIPIN		
71:00:00	8etq-A	3	5.9	57	277	7	MOLECULE:	K-CTRP5		
72:00:00	7qh2-C	3	6.4	61	467	5	MOLECULE:	LACTATE DEHYDROGENASE (NAD(+),FERREDOXIN) S	UBUNIT	
73:00:00	9l0d-B	3	7	79	466	6	MOLECULE:	VACUOLAR FUSION PROTEIN MON1 HOMOLOG A		
74:00:00	5f5p-A	3	9.5	118	181	6	MOLECULE:	PROTEIN SHROOM2		
75:00:00	2dg6-A	3	8.6	64	207	2	MOLECULE:	PUTATIVE TRANSCRIPTIONAL REGULATOR		
76:00:00	8xi9-A	3	5.3	87	201	7	MOLECULE:	FRB-FKBP FUSION PROTEIN		
77:00:00	8p2m-A	3	23.7	109	659	12	MOLECULE:	NAD(+) HYDROLASE TIR-1		
78:00:00	8vdq-A	3	14.8	111	2192	4	MOLECULE:	GREEN FLUORESCENT PROTEIN,TALIN-1		
79:00:00	6sp2-A	3	16.7	96	366	5	MOLECULE:	MEMBRANE PROTEIN TMS1D		
80:00:00	3a8t-A	3	15.6	92	289	4	MOLECULE:	ADENYLATE ISOPENTENYLTRANSFERASE		
81:00:00	6ysf-F	3	13.5	100	257	9	MOLECULE:	CHEMOTAXIS MOTB PROTEIN		
82:00:00	5td8-C	3	9.8	58	111	9	MOLECULE:	KINETOCHORE PROTEIN NDC80		
83:00:00	9cah-A	3	7.7	51	718	8	MOLECULE:	DNA TOPOISOMERASE 3-BETA-1		
84:00:00	8jw0-b	3	12.6	86	617	6	MOLECULE:	PHOTOSYSTEM I PSAA		
85:00:00	3rx6-A	3	7.5	89	187	6	MOLECULE:	POLARITY SUPPRESSION PROTEIN		
86:00:00	9h9q-C	3	28	130	735	5	MOLECULE:	TUBULIN GAMMA CHAIN		
87:00:00	9ayc-A	3	21.5	162	1481	6	MOLECULE:	METAL RESISTANCE PROTEIN YCF1		
88:00:00	5efv-B	3	25.3	98	635	5	MOLECULE:	PHI ETA ORF 56-LIKE PROTEIN		
89:00:00	8yw6-B	3	11.5	67	126	6	MOLECULE:	MITOCHONDRIAL PYRUVATE CARRIER 2		
90:00:00	8rc0-D	3	25.9	90	580	4	MOLECULE:	CD2 ANTIGEN CYTOPLASMIC TAIL-BINDING PROTEI	N 2	
91:00:00	7vcs-A	3	18.8	71	766	1	MOLECULE:	TRANSITIONAL ENDOPLASMIC RETICULUM ATPASE		
92:00:00	7loi-A	3	2.9	51	175	2	MOLECULE:	TRANSMEMBRANE PROTEIN GP41		
93:00:00	3jc5-A	3	17.5	81	208	6	MOLECULE:	DNA REPLICATION LICENSING FACTOR MCM2		
94:00:00	4c47-A	3	14.2	63	191	11	MOLECULE:	INNER MEMBRANE LIPOPROTEIN		
95:00:00	5mps-R	3	2.9	47	108	4	MOLECULE:	YEAST UBC4 GENE FOR UBIQUITIN-CONJUGATING E	NZYME	
96:00:00	5f3o-A	3	3.7	51	194	6	MOLECULE:	PUTATIVE UNCHARACTERIZED PROTEIN		
97:00:00	6r7f-E	3	4.5	52	311	6	MOLECULE:	COP9 SIGNALOSOME COMPLEX SUBUNIT 1		
98:00:00	5cj1-A	3	17.9	72	98	6	MOLECULE:	GP7-MYH7-(1526-1571) CHIMERA PROTEIN		
99:00:00	4kxr-B	3	3.3	90	174	9	MOLECULE:	PE25		
0:00	7f6j-C	3	4	54	106	4	MOLECULE:	RAS-RELATED PROTEIN RAB-7A		
1:00	8i8b-C	3	7	61	229	8	MOLECULE:	MAJOR VIRAL CAPSID PROTEIN		
2:00	5w10-A	3	4.3	43	173	5	MOLECULE:	CGMP-SPECIFIC PHOSPHODIESTERASE		
3:00	6otn-B	3	3	51	67	6	MOLECULE:	TROPOMYOSIN ALPHA-3 CHAIN		
4:00	1ifl-A	3	3.9	46	53	4	MOLECULE:	INOVIRUS		
5:00	1jad-A	2.9	31.9	130	242	10	MOLECULE:	PHOSPHOLIPASE C BETA		
6:00	6idp-A	2.9	34.4	116	438	6	MOLECULE:	MATE FAMILY EFFLUX TRANSPORTER		

7:00	4nqi-D	2.9	18.5	127	232	6	MOLECULE:	SH3 DOMAIN-CONTAINING PROTEIN		
8:00	4n5c-D	2.9	22.4	113	757	5	MOLECULE:	CARGO-TRANSPORT PROTEIN YPP1		
9:00	6fvb-A	2.9	28.9	137	1001	7	MOLECULE:	IMPORTIN BETA-LIKE PROTEIN KAP120		
10:00	7qoo-U	2.9	37.5	137	186	7	MOLECULE:	CENTROMERE PROTEIN C		
11:00	4btf-A	2.9	21.3	77	413	4	MOLECULE:	MIXED LINEAGE KINASE DOMAIN-LIKE PROTEIN		
12:00	8ce1-c	2.9	19.6	88	241	8	MOLECULE:	CYTOCHROME C BIOGENESIS ATP-BINDING EXPORT	PROTEI	
13:00	8ceg-B	2.9	19.8	127	214	6	MOLECULE:	CBY1-INTERACTING BAR DOMAIN-CONTAINING PROT	EIN 1	
14:00	6w17-F	2.9	3.9	60	168	3	MOLECULE:	ACTIN-RELATED PROTEIN 3		
15:00	5ijo-J	2.9	26.6	103	1256	4	MOLECULE:	NUCLEAR PORE COMPLEX PROTEIN NUP155		
16:00	8w20-A	2.9	38	135	732	9	MOLECULE:	SECRETED PROTEIN		
17:00	3zcj-A	2.9	33.8	109	205	5	MOLECULE:	CAGL		
18:00	6ptm-A	2.9	33.8	106	726	6	MOLECULE:	UNCHARACTERIZED PROTEIN		
19:00	8ka6-B	2.9	12	74	148	8	MOLECULE:	DE NOVO DESIGN PROTEIN -NA7		
20:00	7wiu-B	2.9	21.6	126	576	6	MOLECULE:	MYCOBACTIN IMPORT ATP-BINDING/PERMEASE PROT	EIN IR	
21:00	4hga-A	2.9	24.4	96	207	5	MOLECULE:	DEATH DOMAIN-ASSOCIATED PROTEIN 6		
22:00	5b86-A	2.9	35.2	124	579	6	MOLECULE:	TUMOR NECROSIS FACTOR ALPHA-INDUCED PROTEIN		2
23:00	8tek-E	2.9	51.9	151	219	7	MOLECULE:	DYNEIN REGULATORY COMPLEX PROTEIN 1/2 N-TER	MINAL	
24:00:00	8dc2-A	2.9	19.2	93	673	3	MOLECULE:	CASLAMBDA		
25:00:00	3iuk-A	2.9	24.8	101	552	5	MOLECULE:	UNCHARACTERIZED PROTEIN		
26:00:00	4jza-A	2.9	33.1	101	817	6	MOLECULE:	UNCHARACTERIZED PROTEIN		
27:00:00	5n9j-E	2.9	14	106	201	10	MOLECULE:	MEDIATOR OF RNA POLYMERASE II TRANSCRIPTION	SUBUN	
28:00:00	7xzi-A	2.9	49.2	166	1598	8	MOLECULE:	CTAP3		
29:00:00	5xjt-2	2.9	12.4	57	217	5	MOLECULE:	GEM-ASSOCIATED PROTEIN 2		
30:00:00	7vmc-B	2.9	8.9	51	111	2	MOLECULE:	ELONGATION FACTOR TU		
31:00:00	1nek-D	2.9	4.6	90	113	10	MOLECULE:	SUCCINATE DEHYDROGENASE FLAVOPROTEIN SUBUNI	T	
32:00:00	8tlp-A	2.9	5.2	80	226	5	MOLECULE:	D_3_633_8X, NO PEPTIDE		
33:00:00	8erc-A	2.9	5.5	56	441	5	MOLECULE:	LYSOPHOSPHOLIPID ACYLTRANSFERASE 7		
34:00:00	8yb7-C	2.9	23.2	69	455	1	MOLECULE:	PAPAIN-LIKE PROTEASE NSP3		
35:00:00	5ed9-B	2.9	20.3	69	72	7	MOLECULE:	SUN DOMAIN-CONTAINING PROTEIN 2		
36:00:00	8bbg-B	2.9	7.1	81	1426	4	MOLECULE:	WD REPEAT-CONTAINING PROTEIN 19		
37:00:00	6qm5-A	2.9	19.1	133	673	8	MOLECULE:	PREDICTED PROTEIN		
38:00:00	6dcf-D	2.9	23.5	133	1177	7	MOLECULE:	RNA POLYMERASE-BINDING PROTEIN RBPA		
39:00:00	5n9y-A	2.9	13	108	327	6	MOLECULE:	ZINC TRANSPORT PROTEIN ZNTB		
40:00:00	3mzy-A	2.9	9.5	64	123	9	MOLECULE:	RNA POLYMERASE SIGMA-H FACTOR		
41:00:00	4uis-B	2.9	9.9	84	241	2	MOLECULE:	GAMMA-SECRETASE		
42:00:00	7m5f-A	2.9	4.7	49	98	4	MOLECULE:	CDII		
43:00:00	8ymk-A	2.9	8.8	79	226	1	MOLECULE:	ISOFORM S OF LARGE ENVELOPE PROTEIN		
44:00:00	1kx5-A	2.9	7.6	67	135	3	MOLECULE:	DNA		
45:00:00	8th8-J	2.9	10.5	127	372	4	MOLECULE:	DYNEIN REGULATORY COMPLEX PROTEIN 1/2 N-TER	MINAL	
46:00:00	1joc-A	2.9	31.3	88	123	6	MOLECULE:	EARLY ENDOSOMAL AUTOANTIGEN 1		
47:00:00	8of0-M	2.9	4.7	62	140	6	MOLECULE:	DNA-DIRECTED RNA POLYMERASE II SUBUNIT RPB1		
48:00:00	7d6v-C	2.9	6.2	89	264	1	MOLECULE:	SUCCINATE DEHYDROGENASE SUBUNIT A		
49:00:00	8wg3-A	2.9	9.9	83	548	4	MOLECULE:	CSC1-LIKE PROTEIN 2,GREEN FLUORESCENT PROTE	IN	
50:00:00	8tzk-C	2.9	3.8	80	304	4	MOLECULE:	CELL DIVISION ATP-BINDING PROTEIN FTSE		
51:00:00	7paf-A	2.9	9.1	74	278	3	MOLECULE:	NANOBODY		
52:00:00	7t6d-A	2.9	14.3	60	389	8	MOLECULE:	LIPOPOLYSACCHARIDE ASSEMBLY PROTEIN B		
53:00:00	4iyp-A	2.9	10.7	84	205	4	MOLECULE:	IMMUNOGLOBULIN-BINDING PROTEIN 1		
54:00:00	2zy4-F	2.9	2.6	48	535	4	MOLECULE:	L-ASPARTATE BETA-DECARBOXYLASE		
55:00:00	1qv9-A	2.9	6.7	96	282	8	MOLECULE:	F420-DEPENDENT METHYLENETETRAHYDROMETHANOPT	ERIN	
56:00:00	8yzk-A	2.9	4.2	89	264	6	MOLECULE:	SOLUBLE CYTOCHROME B562,G-PROTEIN COUPLED R	ECEPTO	
57:00:00	5n3u-B	2.9	11.4	59	170	3	MOLECULE:	PHYCOCYANOBILIN LYASE SUBUNIT ALPHA		
58:00:00	8q7n-S	2.9	22.8	86	171	10	MOLECULE:	U5 SNRNA		
59:00:00	6urt-A	2.9	11.3	130	331	12	MOLECULE:	LOW CONDUCTANCE MECHANOSENSITIVE CHANNEL YN	AI	
60:00:00	4bzj-C	2.9	7.2	49	693	4	MOLECULE:	PROTEIN TRANSPORT PROTEIN SEC31		
61:00:00	8soi-B	2.9	33.8	161	515	7	MOLECULE:	RB1-INDUCIBLE COILED-COIL PROTEIN 1		
62:00:00	8xuj-A	2.9	9	73	424	4	MOLECULE:	MEMBRANE PROTEIN		
63:00:00	8gs1-D	2.9	3.4	55	337	5	MOLECULE:	AZI28		
64:00:00	8qae-A	2.9	4.8	86	211	3	MOLECULE:	SC-APCC-6-SLLA		
65:00:00	6tpk-A	2.9	15.5	121	461	13	MOLECULE:	OXYTOCIN RECEPTOR		
66:00:00	5hy3-A	2.9	2.6	44	252	7	MOLECULE:	MRNA ENDORIBONUCLEASE LSOA		
67:00:00	2ox6-C	2.9	11.7	74	163	15	MOLECULE:	HYPOTHETICAL PROTEIN SO3848		
68:00:00	7jhj-R	2.9	4.4	93	277	10	MOLECULE:	GUANINE NUCLEOTIDE-BINDING PROTEIN G(I) SUB	UNIT A	
69:00:00	8r5s-A	2.9	5.5	111	237	4	MOLECULE:	CHAIN A, B, C AND H OF THE SOLUBLE HOMOTETR	AMER	
70:00:00	4g80-I	2.9	3.9	82	139	5	MOLECULE:	VOLTAGE-SENSOR CONTAINING PHOSPHATASE		
71:00:00	6yrf-A	2.9	34.6	109	777	2	MOLECULE:	VEGETATIVE INSECTICIDAL PROTEIN		
72:00:00	6ffh-A	2.9	13	92	414	4	MOLECULE:	METABOTROPIC GLUTAMATE RECEPTOR 5,ENDOLYSIN		
73:00:00	9j2f-M	2.9	4.7	59	331	7	MOLECULE:	PHOTOSYNTHETIC REACTION CENTER CYTOCHROME C	SUBUN	
74:00:00	8j8y-A	2.9	6.1	70	129	14	MOLECULE:	IMMUNODOMINANT MEMBRANE PROTEIN		
75:00:00	1qgk-A	2.9	6.6	55	876	11	MOLECULE:	PROTEIN (IMPORTIN BETA SUBUNIT)		
76:00:00	8cmk-A	2.9	8.6	80	913	4	MOLECULE:	TRANSPORTIN-3		
77:00:00	5fu7-A	2.9	16	88	519	1	MOLECULE:	CCR4-NOT TRANSCRIPTION COMPLEX SUBUNIT 1		
78:00:00	8xks-O	2.9	18.1	95	197	5	MOLECULE:	CTAP1		
79:00:00	8exh-a	2.9	4	49	69	6	MOLECULE:	PROTEIN VIRB2		
80:00:00	4rh7-A	2.9	41.5	96	3005	8	MOLECULE:	GREEN FLUORESCENT PROTEIN/CYTOPLASMIC DYNEI	N 2 HE	
81:00:00	6rgv-A	2.9	3.7	62	406	5	MOLECULE:	FLAGELLIN		
82:00:00	3zjc-E	2.9	4.7	62	286	18	MOLECULE:	GTPASE IMAP FAMILY MEMBER 7		
83:00:00	5fiy-A	2.9	3.2	57	78	9	MOLECULE:	PRKC APOPTOSIS WT1 REGULATOR PROTEIN		
84:00:00	8uz8-B	2.9	2.4	41	60	5	MOLECULE:	2-OXOGLUTARATE:ACCEPTOR OXIDOREDUCTASE		
85:00:00	3bas-B	2.9	31	72	87	11	MOLECULE:	MYOSIN HEAVY CHAIN, STRIATED MUSCLE/GENERAL	CONTR	
86:00:00	7o4i-W	2.9	33	109	312	8	MOLECULE:	GENERAL TRANSCRIPTION AND DNA REPAIR FACTOR	IIH H	
87:00:00	3ctw-B	2.9	4	55	120	5	MOLECULE:	RCDA		
88:00:00	5n8k-C	2.9	5.6	57	80	12	MOLECULE:	GALACTOCEREBROSIDASE		
89:00:00	8w6e-B	2.9	3.2	53	161	11	MOLECULE:	HBC599 IN COMPLEX WITH WFAP1.1		
90:00:00	6tdv-f	2.9	15	74	274	4	MOLECULE:	ATPTB1		
91:00:00	6sih-A	2.9	24.5	81	488	7	MOLECULE:	FLAGELLAR HOOK-ASSOCIATED PROTEIN 2		
92:00:00	3k66-A	2.8	29.6	109	217	5	MOLECULE:	BETA-AMYLOID-LIKE PROTEIN		
93:00:00	8ywi-A	2.8	28.9	121	980	10	MOLECULE:	DNA POLYMERASE		
94:00:00	4wbd-A	2.8	23.1	92	540	3	MOLECULE:	BSHC		
95:00:00	4avm-A	2.8	20.2	120	230	4	MOLECULE:	BRIDGING INTEGRATOR 2		
96:00:00	8xzv-D	2.8	37	114	1216	4	MOLECULE:	DNA-DIRECTED RNA POLYMERASE SUBUNIT ALPHA		
97:00:00	7xge-A	2.8	8	89	168	4	MOLECULE:	BCL-XL AND MCL-1 DUAL BINDER 2		
98:00:00	8e2h-A	2.8	16.4	97	656	3	MOLECULE:	BACULOVIRAL IAP REPEAT-CONTAINING PROTEIN 6		
99:00:00	1u4q-A	2.8	16.6	85	318	6	MOLECULE:	SPECTRIN ALPHA CHAIN, BRAIN		
0:00	6yjd-A	2.8	17.3	91	104	3	MOLECULE:	CAPSID ASSEMBLY SCAFFOLDING PROTEIN,PRELAMI	N-A/C	
1:00	8r7n-D	2.8	46.4	135	221	6	MOLECULE:	U11 SNRNA		
2:00	6ikn-A	2.8	13.8	96	305	7	MOLECULE:	GROWTH ARREST-SPECIFIC PROTEIN 7		
3:00	4gwp-C	2.8	31.8	92	184	5	MOLECULE:	MEDIATOR OF RNA POLYMERASE II TRANSCRIPTION	SUBUN	
4:00	1s35-A	2.8	27.2	128	211	5	MOLECULE:	SPECTRIN BETA CHAIN, ERYTHROCYTE		
5:00	4v1a-w	2.8	31.1	100	387	5	MOLECULE:	MITORIBOSOMAL PROTEIN ML37, MRPL37		
6:00	5mpd-Q	2.8	36.3	127	434	8	MOLECULE:	26S PROTEASOME REGULATORY SUBUNIT RPN10		
7:00	5jm8-A	2.8	25	100	556	6	MOLECULE:	AEROBACTIN SYNTHASE IUCA		

8:00	2qe9-A	2.8	33.7	86	165	6	MOLECULE:	UNCHARACTERIZED PROTEIN YIZA		
9:00	9exz-A	2.8	7.1	97	602	4	MOLECULE:	DE NOVO PROTEIN P600		
10:00	7kyp-F	2.8	19.6	89	290	6	MOLECULE:	MANGANESE ABC TRANSPORTER, ATP-BINDING PROT	EIN	
11:00	6n88-A	2.8	12	71	619	6	MOLECULE:	MGC52556 PROTEIN		
12:00	7q4p-C	2.8	5.5	43	878	9	MOLECULE:	SPLICING FACTOR 3A SUBUNIT 2		
13:00	6j5i-b	2.8	19.7	87	209	8	MOLECULE:	ATP SYNTHASE SUBUNIT ALPHA, MITOCHONDRIAL		
14:00	8cih-A	2.8	5.6	103	188	7	MOLECULE:	CYCLIN-DEPENDENT KINASE 2-INTERACTING PROTE	IN	
15:00	7mwq-A	2.8	8.1	57	204	12	MOLECULE:	LHD29A53		
16:00	3h36-A	2.8	8.7	57	78	4	MOLECULE:	POLYRIBONUCLEOTIDE NUCLEOTIDYLTRANSFERASE		
17:00	8qf8-A	2.8	6.3	69	776	4	MOLECULE:	GLYCOSYL HYDROLASE		
18:00	6yxq-B	2.8	12.4	53	396	6	MOLECULE:	ACTIVATING SIGNAL COINTEGRATOR 1 COMPLEX SU	BUNIT	
19:00	7aqr-V	2.8	13.7	60	140	3	MOLECULE:	NADH DEHYDROGENASE [UBIQUINONE] IRON-SULFUR	PROTE	
20:00	5kkl-B	2.8	1.7	41	838	0	MOLECULE:	PUTATIVE POLYCOMB PROTEIN EED		
21:00	4oph-A	2.8	7.6	60	202	5	MOLECULE:	NONSTRUCTURAL PROTEIN 1		
22:00	4xb6-E	2.8	6	64	149	3	MOLECULE:	ALPHA-D-RIBOSE 1-METHYLPHOSPHONATE 5-TRIPHO	SPHATE	
23:00	6zh3-A	2.8	20.6	121	170	6	MOLECULE:	VACUOLAR PROTEIN-SORTING-ASSOCIATED PROTEIN		24
24:00:00	6cp3-Z	2.8	16.1	68	155	6	MOLECULE:	ATP SYNTHASE SUBUNIT 9, MITOCHONDRIAL		
25:00:00	7v9g-D	2.8	5.7	69	112	9	MOLECULE:	BEN DOMAIN-CONTAINING PROTEIN 3		
26:00:00	7vqo-A	2.8	28.2	106	1180	8	MOLECULE:	AMS1, NBR1 AND MALE FUSION PROTEIN		
27:00:00	7tt5-D	2.8	6.1	57	153	7	MOLECULE:	MALTOSE/MALTODEXTRIN-BINDING PERIPLASMIC PR	OTEIN,	
28:00:00	7vf2-C	2.8	38.4	119	184	10	MOLECULE:	PROTEIN VIRILIZER HOMOLOG		
29:00:00	5t76-A	2.8	6.1	74	208	7	MOLECULE:	ALANINE--TRNA LIGASE, CYTOPLASMIC		
30:00:00	6xgx-A	2.8	5.5	55	402	9	MOLECULE:	MUTATOR FAMILY TRANSPOSASE		
31:00:00	9kqi-A	2.8	6.2	74	378	3	MOLECULE:	PHOSPHATIDYLSERINE SYNTHASE 1		
32:00:00	8e1m-A	2.8	12.5	94	133	5	MOLECULE:	MITOCHONDRIAL IMPORT INNER MEMBRANE TRANSLO	CASE S	
33:00:00	3err-A	2.8	34.4	148	527	5	MOLECULE:	FUSION PROTEIN OF MICROTUBULE BINDING DOMAI	N FROM	
34:00:00	8at3-A	2.8	41.6	175	286	4	MOLECULE:	HAUS AUGMIN-LIKE COMPLEX SUBUNIT 1		
35:00:00	8rwv-C	2.8	29.7	94	678	5	MOLECULE:	DNA REPLICATION LICENSING FACTOR MCM2		
36:00:00	4qnd-A	2.8	9.1	58	97	7	MOLECULE:	CHEMICAL TRANSPORT PROTEIN		
37:00:00	8ket-B	2.8	8.4	71	267	7	MOLECULE:	E3 UBIQUITIN-PROTEIN LIGASE SYNOVIOLIN		
38:00:00	7mge-B	2.8	29	124	442	6	MOLECULE:	WD REPEAT-CONTAINING PROTEIN 41		
39:00:00	8bou-A	2.8	17.3	89	807	4	MOLECULE:	N,N'-DIACETYLCHITOBIOSE PHOSPHORYLASE		
40:00:00	8qx8-A	2.8	20.9	71	969	7	MOLECULE:	VACUOLAR PROTEIN SORTING-ASSOCIATED PROTEIN		8
41:00:00	8a6m-A	2.8	3.7	54	161	4	MOLECULE:	ISOFORM 1B OF SYNAPTOGYRIN-1		
42:00:00	8f7c-A	2.8	6.5	103	287	7	MOLECULE:	PANNEXIN-2, SOLUBLE CYTOCHROME B562 FUSION		
43:00:00	7rut-B	2.8	9.7	67	187	7	MOLECULE:	CORRINOID ADENOSYLTRANSFERASE		
44:00:00	2hsb-A	2.8	3.9	50	126	4	MOLECULE:	HYPOTHETICAL UPF0332 PROTEIN AF0298		
45:00:00	7syf-A	2.8	25.7	120	1391	8	MOLECULE:	PHOSPHATIDYLINOSITOL 3,4,5-TRISPHOSPHATE-DE	PENDEN	
46:00:00	6nsj-A	2.8	4.3	80	182	5	MOLECULE:	ACID-ACTIVATED UREA CHANNEL		
47:00:00	8vxq-A	2.8	4.1	56	155	2	MOLECULE:	GP72		
48:00:00	6al2-A	2.8	24.5	95	484	6	MOLECULE:	MEMBRANE PROTEIN INSERTASE YIDC		
49:00:00	2y7c-A	2.8	23.1	132	464	6	MOLECULE:	TYPE-1 RESTRICTION ENZYME ECOKI SPECIFICITY	PROTE	
50:00:00	7drj-A	2.8	9.9	67	189	9	MOLECULE:	CDP-DIACYLGLYCEROL--GLYCEROL-3-PHOSPHATE 3-		
51:00:00	1s3j-A	2.8	9.7	79	143	6	MOLECULE:	YUSO PROTEIN		
52:00:00	8afz-A	2.8	20.6	111	381	8	MOLECULE:	SORTING NEXIN-1		
53:00:00	3kmi-A	2.8	5.7	93	172	8	MOLECULE:	PUTATIVE MEMBRANE PROTEIN COG4129		
54:00:00	9crw-D	2.8	7.9	83	228	8	MOLECULE:	KINESIN-LIKE PROTEIN		
55:00:00	6lqo-L	2.8	4.9	82	94	2	MOLECULE:	CYTOPLASMIC ENVELOPMENT PROTEIN 1		
56:00:00	6c6r-A	2.8	4.8	83	451	5	MOLECULE:	SQUALENE MONOOXYGENASE		
57:00:00	8t9l-A	2.8	13.7	78	108	4	MOLECULE:	NUCLEOPORIN POM152		
58:00:00	6m3q-F	2.8	12	116	301	6	MOLECULE:	ANKYRIN-2		
59:00:00	6ec8-A	2.8	25.7	130	803	6	MOLECULE:	LANTIBIOTIC DEHYDRATASE DOMAIN PROTEIN		
60:00:00	4rs7-B	2.8	7.3	69	88	13	MOLECULE:	PARB-C		
61:00:00	1cqx-A	2.8	14.6	71	403	13	MOLECULE:	FLAVOHEMOPROTEIN		
62:00:00	8ua7-A	2.8	7.6	63	109	6	MOLECULE:	HISTONE H3		
63:00:00	3nr7-A	2.8	4.2	62	81	5	MOLECULE:	DNA-BINDING PROTEIN H-NS		
64:00:00	5b0l-B	2.8	14.7	99	334	3	MOLECULE:	MOEN5,DNA-BINDING PROTEIN 7D		
65:00:00	5oge-G	2.8	4.3	68	306	6	MOLECULE:	GDP-MANNOSE TRANSPORTER 1		
66:00:00	5ixp-A	2.8	3.3	40	393	3	MOLECULE:	EXTRACELLULAR SOLUTE-BINDING PROTEIN FAMILY		1
67:00:00	5xsj-L	2.8	4	75	122	11	MOLECULE:	PERIPLASMIC BINDING PROTEIN/LACI TRANSCRIPT	IONAL	
68:00:00	5mdx-A	2.8	11.2	71	307	3	MOLECULE:	PHOTOSYSTEM II PROTEIN D1		
69:00:00	8h0m-A	2.8	5.7	74	392	7	MOLECULE:	VIOD		
70:00:00	4mnd-A	2.8	8	63	408	8	MOLECULE:	CTP L-MYO-INOSITOL-1-PHOSPHATE CYTIDYLYLTRA	NSFERA	
71:00:00	3qt2-C	2.8	4.4	50	110	16	MOLECULE:	INTERLEUKIN-5 RECEPTOR SUBUNIT ALPHA		
72:00:00	5z25-A	2.8	4.3	62	96	8	MOLECULE:	CYTOCHROME C552		
73:00:00	2rvq-D	2.8	5.9	54	129	7	MOLECULE:	HISTONE H2A TYPE 1-B/E		
74:00:00	7ty0-C	2.8	33	89	488	11	MOLECULE:	GLYCOPROTEIN G		
75:00:00	6m15-A	2.8	26	88	965	3	MOLECULE:	SPIKE GLYCOPROTEIN		
76:00:00	7rtn-A	2.8	11.9	82	270	6	MOLECULE:	OUTER CAPSID PROTEIN VP5		
77:00:00	9c3f-A	2.8	25.1	99	485	7	MOLECULE:	ANHYDROMUROPEPTIDE PERMEASE,SOLUBLE CYTOCHR	OME B5	
78:00:00	8u54-B	2.8	28.5	124	680	6	MOLECULE:	INTEGRAL MEMBRANE PROTEIN GPR155		
79:00:00	9gm6-B	2.8	43.9	142	1148	5	MOLECULE:	CHROMOSOME PARTITION PROTEIN MUKF		
80:00:00	8v1g-A	2.8	14.4	111	990	11	MOLECULE:	NIEMANN-PICK TYPE C1-RELATED PROTEIN		
81:00:00	7ojn-L	2.8	17.8	87	2010	5	MOLECULE:	RNA-DIRECTED RNA POLYMERASE L		
82:00:00	6pw5-B	2.8	19.8	123	782	6	MOLECULE:	TRP-LIKE ION CHANNEL		
83:00:00	8ezj-A	2.8	3.6	48	1285	8	MOLECULE:	ARF GUANINE-NUCLEOTIDE EXCHANGE FACTOR 2		
84:00:00	4ap2-B	2.8	10.7	55	359	4	MOLECULE:	KELCH-LIKE PROTEIN 11		
85:00:00	7y9x-A	2.8	44.4	111	1458	10	MOLECULE:	CHAT DOMAIN-CONTAINING PROTEIN		
86:00:00	1lgh-A	2.8	4.8	45	56	7	MOLECULE:	LIGHT HARVESTING COMPLEX II		
87:00:00	7t92-B	2.8	22.7	117	359	5	MOLECULE:	PEROXIN-12		
88:00:00	6hd8-B	2.8	5.8	73	232	7	MOLECULE:	NANOBODY,MALTOSE/MALTODEXTRIN-BINDING PERIP	LASMIC	
89:00:00	7lma-A	2.8	6.5	96	1012	4	MOLECULE:	TELOMERASE LA-RELATED PROTEIN P65		
90:00:00	6bym-A	2.8	5.2	56	200	13	MOLECULE:	STEROL-BINDING PROTEIN		
91:00:00	7ktr-A	2.8	23.2	104	3042	8	MOLECULE:	TRANSFORMATION/TRANSCRIPTION DOMAIN-ASSOCIA	TED PR	
92:00:00	7zjk-A	2.8	5.3	100	221	6	MOLECULE:	CSPZ		
93:00:00	8eki-A	2.8	10.7	84	170	5	MOLECULE:	PROTEIN TRANSPORT PROTEIN SEC20		
94:00:00	6lcp-B	2.8	22.9	109	376	9	MOLECULE:	PHOSPHOLIPID-TRANSPORTING ATPASE		
95:00:00	7vrc-D	2.8	5.2	48	51	8	MOLECULE:	TRANSCRIPTION REGULATORY PROTEIN SNF11		
96:00:00	2x3j-A	2.8	19	105	586	4	MOLECULE:	ACSD		
97:00:00	2odu-A	2.8	6.8	92	217	9	MOLECULE:	PLECTIN 1		
98:00:00	7bii-B	2.8	24.4	143	2144	6	MOLECULE:	E3 UBIQUITIN-PROTEIN LIGASE HUWE1		
99:00:00	6cgh-A	2.8	2.4	44	89	16	MOLECULE:	DNAJ HOMOLOG SUBFAMILY C MEMBER 2		
0:00	5wst-A	2.8	3	54	71	9	MOLECULE:	UNCONVENTIONAL MYOSIN-VIIA		
1:00	6n6s-D	2.8	3.5	55	69	11	MOLECULE:	TNFAIP3-INTERACTING PROTEIN 1		
2:00	8sjj-A	2.8	3.5	58	74	7	MOLECULE:	SEPTIN-14		
3:00	5xau-E	2.8	14.5	54	72	7	MOLECULE:	LAMININ SUBUNIT ALPHA-5		
4:00	6yip-A	2.8	3.8	55	71	5	MOLECULE:	KINESIN-LIKE PROTEIN KIF20A		
5:00	7eqb-A	2.8	4.5	55	68	11	MOLECULE:	KINESIN-LIKE PROTEIN		
6:00	6hk9-B	2.8	4.7	59	78	7	MOLECULE:	TESTIS-EXPRESSED PROTEIN 12		
7:00	8fn2-a	2.8	4.4	47	65	6	MOLECULE:	23S RIBOSOMAL RNA		
8:00	4akk-A	2.8	11.3	68	368	6	MOLECULE:	NITRATE REGULATORY PROTEIN		

9:00	5mnt-B	2.8	10.2	87	421	3	MOLECULE:	A2 MATURATION PROTEIN	
10:00	9dva-G	2.8	4.3	47	71	9	MOLECULE:	ACTIN, ALPHA SKELETAL MUSCLE	
11:00	8sl3-A	2.8	5.8	66	578	11	MOLECULE:	ADENYLATE CYCLASE TYPE 5	
12:00	2e5t-A	2.8	3.5	41	46	5	MOLECULE:	ATP SYNTHASE EPSILON CHAIN	
13:00	6r5k-O	2.7	22	88	447	9	MOLECULE:	PAN2-PAN3 DEADENYLATION COMPLEX CATALYTIC S	UBUNIT
14:00	6nd4-O	2.7	7	64	832	13	MOLECULE:	ETS RRNA	
15:00	5yke-B	2.7	20.4	171	845	7	MOLECULE:	ATP-SENSITIVE INWARD RECTIFIER POTASSIUM CH	ANNEL
16:00	8pr3-C	2.7	34.4	86	106	1	MOLECULE:	C-JUN-AMINO-TERMINAL KINASE-INTERACTING PRO	TEIN 3
17:00	6h9m-A	2.7	22.3	73	94	4	MOLECULE:	COILED-COIL DOMAIN-CONTAINING PROTEIN 90B,	MITOCH
18:00	8b6l-O	2.7	22.8	71	580	6	MOLECULE:	PROTEIN TRANSPORT PROTEIN SEC61 SUBUNIT ALP	HA ISO
19:00	7eld-A	2.7	28.8	112	1137	6	MOLECULE:	ENDORIBONUCLEASE DICER HOMOLOG 1	
20:00	7b93-L	2.7	35.2	196	607	1	MOLECULE:	NADH-UBIQUINONE OXIDOREDUCTASE CHAIN 3	
21:00	8j98-A	2.7	16.5	59	184	8	MOLECULE:	NEAR-INFRARED FLUORESCENT PROTEIN	
22:00	6lul-A	2.7	21.4	102	223	7	MOLECULE:	RESPIRATORY SUPERCOMPLEX FACTOR 2, MITOCHON	DRIAL
23:00	6tgz-F	2.7	31.3	127	352	9	MOLECULE:	55 KDA IMMEDIATE-EARLY PROTEIN 1	
24:00:00	8tdl-A	2.7	37	121	364	5	MOLECULE:	MECHANOSENSITIVE ION CHANNEL PROTEIN 10	
25:00:00	2dh4-A	2.7	13.9	59	326	10	MOLECULE:	YPL069C	
26:00:00	8p0s-A	2.7	50.7	117	133	5	MOLECULE:	RHO-ASSOCIATED PROTEIN KINASE 1	
27:00:00	4lin-A	2.7	66.9	161	289	8	MOLECULE:	TAIL NEEDLE PROTEIN GP26	
28:00:00	6hux-A	2.7	16.1	85	369	7	MOLECULE:	H(2)-FORMING METHYLENETETRAHYDROMETHANOPTER	IN
29:00:00	8rg0-5	2.7	28.5	105	520	9	MOLECULE:	EUKARYOTIC TRANSLATION INITIATION FACTOR 3	SUBUNI
30:00:00	5y5a-A	2.7	37.2	147	514	3	MOLECULE:	KLLA0F20702P	
31:00:00	5cws-D	2.7	27.4	130	180	5	MOLECULE:	SAB-158 FAB LIGHT CHAIN	
32:00:00	2huj-A	2.7	30.2	80	125	1	MOLECULE:	LIN2004 PROTEIN	
33:00:00	6ysg-A	2.7	30.4	131	1264	10	MOLECULE:	MG-CHELATASE SUBUNIT CHLH	
34:00:00	8t1l-S	2.7	21.7	159	913	6	MOLECULE:	MEDIATOR OF RNA POLYMERASE II TRANSCRIPTION	SUBUN
35:00:00	6eqo-A	2.7	26.4	122	1804	4	MOLECULE:	ACETYL-COENZYME A SYNTHETASE	
36:00:00	9ewy-A	2.7	7.5	91	815	10	MOLECULE:	[F-ACTIN]-MONOOXYGENASE MICAL1	
37:00:00	5x11-E	2.7	3.9	64	181	5	MOLECULE:	DNA (28-MER)	
38:00:00	5lst-A	2.7	6.3	66	617	3	MOLECULE:	ATP-DEPENDENT DNA HELICASE Q4	
39:00:00	8fli-B	2.7	3.8	51	458	14	MOLECULE:	GROUP II INTRON	
40:00:00	8egr-A	2.7	31.6	131	609	9	MOLECULE:	GP15, RECEPTOR-BINDING PROTEIN, TAIL FIBER	
41:00:00	6em5-r	2.7	12.6	78	176	6	MOLECULE:	5.8S RIBOSOMAL RNA	
42:00:00	8e9h-J	2.7	4.2	51	231	8	MOLECULE:	TWO-COMPONENT SYSTEM RESPONSE REGULATOR	
43:00:00	8xku-R	2.7	8.9	91	267	7	MOLECULE:	PROBABLE INACTIVE ATP-DEPENDENT ZINC METALL	OPROTE
44:00:00	5b7j-A	2.7	5.1	62	110	2	MOLECULE:	SWITCH-ACTIVATING PROTEIN 1	
45:00:00	8gyz-B	2.7	16.9	87	215	3	MOLECULE:	TRANSCRIPTION FACTOR TGA7	
46:00:00	6a73-A	2.7	15.6	90	290	10	MOLECULE:	COP9 SIGNALOSOME COMPLEX SUBUNIT 2,ENDOLYSI	N
47:00:00	6g3e-A	2.7	11.9	133	497	5	MOLECULE:	ARGININOSUCCINATE LYASE	
48:00:00	8q3v-g	2.7	4	54	76	4	MOLECULE:	TETRAHYDROMETHANOPTERIN S-METHYLTRANSFERASE	SUBUN
49:00:00	3dxp-A	2.7	10.9	82	329	5	MOLECULE:	PUTATIVE ACYL-COA DEHYDROGENASE	
50:00:00	8yx6-A	2.7	10.6	83	324	0	MOLECULE:	LIGAND-GATED CATION CHANNEL ZACN	
51:00:00	7dvr-A	2.7	9.4	75	147	1	MOLECULE:	HTH MARR-TYPE DOMAIN-CONTAINING PROTEIN	
52:00:00	5lnf-A	2.7	10.3	93	139	6	MOLECULE:	PEROXISOMAL BIOGENESIS FACTOR 19	
53:00:00	8j0h-A	2.7	4.6	92	134	4	MOLECULE:	UNCHARACTERIZED PROTEIN C4H3.06	
54:00:00	3fgg-A	2.7	7.2	67	147	12	MOLECULE:	UNCHARACTERIZED PROTEIN BCE2196	
55:00:00	5ufl-A	2.7	2.3	39	523	5	MOLECULE:	PROTEIN CIP2A	
56:00:00	6l4o-A	2.7	7.9	57	429	5	MOLECULE:	APOPTOSIS INHIBITOR 5	
57:00:00	6xm1-C	2.7	29.8	128	570	0	MOLECULE:	VPS45	
58:00:00	7nwu-B	2.7	5.5	53	249	2	MOLECULE:	REGULATOR OF NONSENSE TRANSCRIPTS 3B	
59:00:00	5y58-A	2.7	18.6	88	548	7	MOLECULE:	ATP-DEPENDENT DNA HELICASE II SUBUNIT 1	
60:00:00	5y88-U	2.7	20.4	104	488	11	MOLECULE:	PRE-MRNA-SPLICING FACTOR 8	
61:00:00	9na8-C	2.7	4	45	234	7	MOLECULE:	AUGMIN SUBUNIT 1	
62:00:00	4l80-B	2.7	17.1	76	347	7	MOLECULE:	HPCH/HPAI ALDOLASE	
63:00:00	9eto-A	2.7	7.8	76	363	9	MOLECULE:	4-HYDROXYTRYPTAMINE KINASE	
64:00:00	8y1x-A	2.7	14.6	85	544	2	MOLECULE:	ASPARTATE/ALANINE ANTIPORTER	
65:00:00	4zi2-C	2.7	8.1	59	131	7	MOLECULE:	ADP-RIBOSYLATION FACTOR-LIKE PROTEIN 3	
66:00:00	7p2y-J	2.7	3.3	61	80	7	MOLECULE:	ATP SYNTHASE SUBUNIT ALPHA	
67:00:00	8rd2-B	2.7	13.1	124	400	6	MOLECULE:	INVARIANT SURFACE GLYCOPROTEIN	
68:00:00	7qj0-L	2.7	5.1	71	566	7	MOLECULE:	MITOTIC-SPINDLE ORGANIZING PROTEIN 1	
69:00:00	9h3q-C	2.7	5.2	62	158	8	MOLECULE:	LARGE RIBOSOMAL SUBUNIT PROTEIN BL34	
70:00:00	8hpo-D	2.7	44.4	94	165	9	MOLECULE:	TRANSCRIPTIONAL REGULATORY PROTEIN UME1	
71:00:00	6lum-G	2.7	8.3	78	123	1	MOLECULE:	SUCCINATE DEHYDROGENASE SUBUNIT C	
72:00:00	8tdj-A	2.7	14.2	156	467	4	MOLECULE:	MECHANOSENSITIVE ION CHANNEL PROTEIN 10	
73:00:00	6h4b-B	2.7	3.7	48	84	6	MOLECULE:	ORF026	
74:00:00	6cnn-A	2.7	7.7	86	360	6	MOLECULE:	INTERMEDIATE CONDUCTANCE CALCIUM-ACTIVATED	POTASS
75:00:00	7m7a-A	2.7	4.2	72	538	7	MOLECULE:	PHOSPHOINOSITIDE 3-KINASE MAVQ	
76:00:00	5cd4-I	2.7	9.5	90	494	6	MOLECULE:	CRISPR SYSTEM CASCADE SUBUNIT CASE	
77:00:00	5olk-A	2.7	12.5	73	395	4	MOLECULE:	RIBONUCLEOSIDE-DIPHOSPHATE REDUCTASE, BETA	SUBUNI
78:00:00	8wt9-A	2.7	2.8	40	319	8	MOLECULE:	IS621 TRANSPOSASE	
79:00:00	3gfi-A	2.7	9.8	77	143	1	MOLECULE:	146AA LONG HYPOTHETICAL TRANSCRIPTIONAL REG	ULATOR
80:00:00	4uig-A	2.7	3.6	69	91	10	MOLECULE:	COPPER SENSITIVE OPERON REPRESSOR	
81:00:00	3cfo-A	2.7	2.7	40	906	18	MOLECULE:	DNA POLYMERASE	
82:00:00	7oca-E	2.7	8.6	91	158	2	MOLECULE:	GLUTAMATE RECEPTOR 1	
83:00:00	8k58-G	2.7	9	62	129	3	MOLECULE:	DNA-DIRECTED RNA POLYMERASE SUBUNIT BETA	
84:00:00	2xnx-M	2.7	17.6	62	107	8	MOLECULE:	FIBRINOGEN ALPHA CHAIN	
85:00:00	5a63-D	2.7	3.7	60	100	10	MOLECULE:	NICASTRIN	
86:00:00	2gd5-D	2.7	4.9	88	162	11	MOLECULE:	CHARGED MULTIVESICULAR BODY PROTEIN 3	
87:00:00	5iwz-A	2.7	10.3	65	372	6	MOLECULE:	SYNAPTONEMAL COMPLEX PROTEIN 2	
88:00:00	5oc0-A	2.7	5.8	100	174	6	MOLECULE:	CYTOCHROME B561	
89:00:00	6ian-A	2.7	26	138	306	9	MOLECULE:	INTRAFLAGELLAR TRANSPORT PROTEIN 74	
90:00:00	4w8j-A	2.7	27.3	137	1017	4	MOLECULE:	PESTICIDAL CRYSTAL PROTEIN CRY1AC	
91:00:00	6v4m-A	2.7	2.5	42	163	5	MOLECULE:	BCL-2	
92:00:00	8z1y-A	2.7	13	125	331	9	MOLECULE:	DIPEPTIDE TRANSPORT SYSTEM PERMEASE PROTEIN	DPPB
93:00:00	8evm-C	2.7	4.9	85	237	7	MOLECULE:	CHLOROPHYLL DIMER PROTEIN DESIGNS, SPECIAL	PAIR 3
94:00:00	7uic-c	2.7	5.4	80	110	8	MOLECULE:	MEDIATOR OF RNA POLYMERASE II TRANSCRIPTION	SUBUN
95:00:00	6exn-D	2.7	30.8	77	97	5	MOLECULE:	U2 SNRNA	
96:00:00	1dce-A	2.7	7.7	73	567	7	MOLECULE:	PROTEIN (RAB GERANYLGERANYLTRANSFERASE ALPH	A
97:00:00	3vr8-D	2.7	9.9	99	129	2	MOLECULE:	FLAVOPROTEIN SUBUNIT OF COMPLEX II	
98:00:00	6xp5-V	2.7	25.2	107	112	9	MOLECULE:	MEDIATOR OF RNA POLYMERASE II TRANSCRIPTION	SUBUN
99:00:00	6jmg-A	2.7	2.9	48	267	13	MOLECULE:	DNAJ HOMOLOG SUBFAMILY C MEMBER 27-A	
0:00	8hhf-B	2.7	7.3	58	89	7	MOLECULE:	CELL DIVISION PROTEIN FTSQ	
1:00	8ifg-A	2.7	26.1	88	879	8	MOLECULE:	RBAP48-RELATED WD40 REPEAT-CONTAINING PROTE	IN PRW
2:00	2pf4-E	2.7	4.8	50	163	6	MOLECULE:	SERINE/THREONINE-PROTEIN PHOSPHATASE 2A 65	KDA RE
3:00	6v35-A	2.7	23	76	914	4	MOLECULE:	CALCIUM-ACTIVATED POTASSIUM CHANNEL SUBUNIT	ALPHA
4:00	6wvg-A	2.7	21.9	84	443	7	MOLECULE:	GREEN FLUORESCENT PROTEIN, LEUKOCYTE SURFAC	E ANTI
5:00	1tjl-A	2.7	4.5	68	145	9	MOLECULE:	DNAK SUPPRESSOR PROTEIN	
6:00	3jrt-A	2.7	5.4	56	166	7	MOLECULE:	INTEGRON CASSETTE PROTEIN VPC_CASS2	
7:00	4n9n-A	2.7	28.2	79	428	5	MOLECULE:	STEROL UPTAKE CONTROL PROTEIN 2, LYSOZYME	
8:00	6v85-C	2.7	3.3	58	74	5	MOLECULE:	RNA-DIRECTED RNA POLYMERASE L	
9:00	7bv6-H	2.7	3.1	56	68	7	MOLECULE:	VESICLE-ASSOCIATED MEMBRANE PROTEIN 8	

10:00	6abo-B	2.7	5.3	59	77	14	MOLECULE:	DNA REPAIR PROTEIN XRCC4		
11:00	6dm9-A	2.7	3.3	54	78	6	MOLECULE:	DHD15_EXTENDED_A		
12:00	8i3e-B	2.7	30.8	61	135	8	MOLECULE:	ELKS/RAB6-INTERACTING/CAST FAMILY MEMBER 1		
13:00	5hzp-A	2.7	2.9	54	71	7	MOLECULE:	M PROTEIN, SEROTYPE 49		
14:00	1nyh-A	2.7	3.8	55	76	2	MOLECULE:	REGULATORY PROTEIN SIR4		
15:00	5vpc-B	2.7	3.1	54	68	13	MOLECULE:	PROTEIN FOSB		
16:00	9itj-X	2.7	25.3	61	155	5	MOLECULE:	ATP SYNTHASE SUBUNIT ALPHA		
17:00	5yyl-D	2.7	5.5	41	43	10	MOLECULE:	MAJOR ROYAL JELLY PROTEIN 1		
18:00	7x5e-E	2.7	3.7	58	101	9	MOLECULE:	TRANSCRIPTION FACTOR MAFG		
19:00	2d3o-W	2.7	4.4	50	66	6	MOLECULE:	23S RIBOSOMAL RNA		
20:00	8fxf-A	2.7	4.9	57	78	4	MOLECULE:	E3 UBIQUITIN-PROTEIN LIGASE TRIM56		
21:00	4d7x-A	2.7	2.8	46	86	13	MOLECULE:	MEDIATOR OF RNA POLYMERASE II TRANSCRIPTION	SUBUN	
22:00	8kbe-B	2.7	5.4	54	131	4	MOLECULE:	THOERIS ANTI-DEFENSE 1		
23:00	5nen-A	2.7	4.3	72	266	3	MOLECULE:	LIPASE C		
24:00:00	7m2w-F	2.7	6.1	71	674	4	MOLECULE:	TUBULIN GAMMA CHAIN		
25:00:00	6ny2-Y	2.7	12.7	84	915	7	MOLECULE:	DNA TARGET STRAND		
26:00:00	7nvr-n	2.7	13	76	132	8	MOLECULE:	TFIIH BASAL TRANSCRIPTION FACTOR COMPLEX HE	LICASE	
27:00:00	7twd-B	2.7	4.3	41	45	7	MOLECULE:	ALPHA- AND GAMMA-ADAPTIN-BINDING PROTEIN P3		4
28:00:00	6whp-A	2.6	33	100	428	4	MOLECULE:	CHOLINE KINASE		
29:00:00	8k0b-A	2.6	30.2	129	689	7	MOLECULE:	CALCIUM PERMEABLE STRESS-GATED CATION CHANN	EL 1	
30:00:00	6bx3-F	2.6	18.6	94	215	5	MOLECULE:	HISTONE-LYSINE N-METHYLTRANSFERASE, H3 LYSI	NE-4 S	
31:00:00	8urn-A	2.6	26.9	69	152	9	MOLECULE:	ESCI INNER ROD PROTEIN TYPE III SECRETION S	YSTEM,	
32:00:00	2p0n-A	2.6	33.5	97	161	7	MOLECULE:	HYPOTHETICAL PROTEIN NMB1532		
33:00:00	3nl9-A	2.6	31.4	93	169	3	MOLECULE:	PUTATIVE NTP PYROPHOSPHOHYDROLASE		
34:00:00	8gaa-A	2.6	8.3	50	197	4	MOLECULE:	C6HR1_4R		
35:00:00	9f63-A	2.6	20.1	103	422	6	MOLECULE:	PROTEIN PNS1		
36:00:00	5yz0-A	2.6	46.6	141	2362	6	MOLECULE:	SERINE/THREONINE-PROTEIN KINASE ATR		
37:00:00	7sbe-A	2.6	33.3	116	228	8	MOLECULE:	TELOMERASE REVERSE TRANSCRIPTASE		
38:00:00	6zce-q	2.6	26	83	636	7	MOLECULE:	EUKARYOTIC TRANSLATION INITIATION FACTOR 3	SUBUNI	
39:00:00	9exk-A	2.6	31.8	118	1000	6	MOLECULE:	DE NOVO DESIGNED PROTEIN K12		
40:00:00	8v29-A	2.6	29.7	87	172	5	MOLECULE:	ONCOSTATIN-M		
41:00:00	7sfj-A	2.6	26.8	105	284	8	MOLECULE:	CHRMINE		
42:00:00	8dt0-A	2.6	15.3	54	140	7	MOLECULE:	SCAFFOLDING PROTEIN FUNCTIONAL SITES		
43:00:00	8ssc-A	2.6	28	83	1123	5	MOLECULE:	METHIONINE SYNTHASE		
44:00:00	8a5a-U	2.6	32.2	125	690	4	MOLECULE:	CHROMATIN-REMODELING ATPASE INO80		
45:00:00	8bd7-G	2.6	28.1	162	205	8	MOLECULE:	IFT88		
46:00:00	2v71-A	2.6	58.8	124	160	9	MOLECULE:	NUCLEAR DISTRIBUTION PROTEIN NUDE-LIKE 1		
47:00:00	8ovw-U	2.6	33.8	99	184	6	MOLECULE:	CENTROMERE-BINDING PROTEIN 1		
48:00:00	6zpp-A	2.6	33.3	90	157	3	MOLECULE:	VIRULENCE FACTOR		
49:00:00	2d1l-A	2.6	21.5	119	249	8	MOLECULE:	METASTASIS SUPPRESSOR PROTEIN 1		
50:00:00	8j2x-A	2.6	22.2	87	337	0	MOLECULE:	COBALAMIN-BINDING PROTEIN		
51:00:00	4wpc-B	2.6	32.6	133	288	6	MOLECULE:	RHO GTPASE-ACTIVATING PROTEIN RGD1		
52:00:00	8ibw-C	2.6	19.2	111	922	12	MOLECULE:	DNA (60-MER)		
53:00:00	3ggz-D	2.6	6.1	92	188	5	MOLECULE:	INCREASED SODIUM TOLERANCE PROTEIN 1		
54:00:00	4yif-B	2.6	10	79	141	11	MOLECULE:	MARR FAMILY PROTEIN RV0880		
55:00:00	6x0a-a	2.6	13.7	63	87	8	MOLECULE:	PLASMID STABILIZATION SYSTEM		
56:00:00	8pjn-b	2.6	31	104	296	9	MOLECULE:	E3 UBIQUITIN-PROTEIN TRANSFERASE RMND5A		
57:00:00	6pwn-A	2.6	18.5	147	280	7	MOLECULE:	SMALL-CONDUCTANCE MECHANOSENSITIVE CHANNEL		
58:00:00	9knu-M	2.6	8.3	76	140	7	MOLECULE:	PORTAL PROTEIN		
59:00:00	8ppr-F	2.6	17.4	77	112	5	MOLECULE:	KINETOCHORE-ASSOCIATED PROTEIN DSN1 HOMOLOG		
60:00:00	8jj6-A	2.6	13.1	101	554	5	MOLECULE:	NEGATIVE ELONGATION FACTOR B		
61:00:00	8i6v-D	2.6	10.5	78	678	4	MOLECULE:	VACUOLAR TRANSPORTER CHAPERONE COMPLEX SUBU	NIT 1	
62:00:00	4mi2-B	2.6	22.4	92	261	8	MOLECULE:	PUTATIVE ENOYL-COA HYDRATASE/ISOMERASE		
63:00:00	5o5j-O	2.6	5	69	88	4	MOLECULE:	16S RRNA		
64:00:00	5ajk-A	2.6	3.4	41	148	7	MOLECULE:	HOMOLOG OF VACCINIA VIRUS CDS F1L		
65:00:00	3dt5-A	2.6	4.2	51	119	10	MOLECULE:	UNCHARACTERIZED PROTEIN AF_0924		
66:00:00	6wgv-A	2.6	4	48	173	4	MOLECULE:	CORRINOID ADENOSYLTRANSFERASE		
67:00:00	3g9g-A	2.6	4.1	107	250	7	MOLECULE:	SUPPRESSOR OF YEAST PROFILIN DELETION		
68:00:00	1j1n-A	2.6	4.8	47	492	2	MOLECULE:	ALGQ2		
69:00:00	5abb-A	2.6	34.2	114	443	5	MOLECULE:	PROTEIN TRANSLOCASE SUBUNIT SECY		
70:00:00	6o0y-A	2.6	17.9	105	1146	8	MOLECULE:	CRISPR-ASSOCIATED ENDONUCLEASE CAS9/CSN1		
71:00:00	8p0v-L	2.6	10.4	61	416	10	MOLECULE:	COILED-COIL DOMAIN-CONTAINING PROTEIN 93		
72:00:00	6zz6-C	2.6	27.6	52	83	12	MOLECULE:	STRUCTURAL MAINTENANCE OF CHROMOSOMES PROTE	IN 1,S	
73:00:00	6s6b-B	2.6	3.6	61	154	7	MOLECULE:	CRISPR-ASSOCIATED PROTEIN, CMR5 FAMILY		
74:00:00	8k0e-A	2.6	17	122	646	4	MOLECULE:	PROLYL 3-HYDROXYLASE 1		
75:00:00	8zxc-A	2.6	3	47	205	2	MOLECULE:	ASH1L BROMODOMAIN AND PHD DOMAIN		
76:00:00	8ul9-A	2.6	20.9	112	387	5	MOLECULE:	CHOLINEPHOSPHOTRANSFERASE 1		
77:00:00	7uuy-A	2.6	8.4	106	503	7	MOLECULE:	SODIUM/IODIDE COTRANSPORTER		
78:00:00	4akf-A	2.6	19.9	105	560	8	MOLECULE:	VIPD		
79:00:00	6rd4-1	2.6	17.5	106	595	7	MOLECULE:	ASA-10: POLYTOMELLA F-ATP SYNTHASE ASSOCIAT	ED SUB	
80:00:00	6lu0-A	2.6	4.6	71	937	13	MOLECULE:	CAS12I2		
81:00:00	4xal-A	2.6	7.7	68	90	1	MOLECULE:	TEGUMENT PROTEIN VP22		
82:00:00	5tuu-B	2.6	5.8	50	106	6	MOLECULE:	TRANSCRIPTION FACTOR DP1		
83:00:00	7y7o-A	2.6	3.2	39	147	5	MOLECULE:	ENDORIBONUCLEASE YBEY		
84:00:00	8e4g-0	2.6	13.2	107	196	2	MOLECULE:	PORTAL PROTEIN		
85:00:00	4aur-A	2.6	5.5	94	564	2	MOLECULE:	LEOA		
86:00:00	5t2x-C	2.6	2.4	38	258	13	MOLECULE:	UNCHARACTERIZED PROTEIN LPG1670		
87:00:00	3kdq-C	2.6	11.2	76	153	3	MOLECULE:	UNCHARACTERIZED CONSERVED PROTEIN		
88:00:00	5ip0-D	2.6	3.9	86	108	8	MOLECULE:	PHA GRANULE-ASSOCIATED PROTEIN		
89:00:00	4wat-A	2.6	5.4	67	332	9	MOLECULE:	PFRH5		
90:00:00	8xlr-A	2.6	18.3	128	757	10	MOLECULE:	ANOCTAMIN-1		
91:00:00	6maf-A	2.6	21.3	111	259	5	MOLECULE:	BBVCI ENDONUCLEASE SUBUNIT 1		
92:00:00	6uwm-A	2.6	7.7	75	221	4	MOLECULE:	VOLTAGE-GATED POTASSIUM CHANNEL		
93:00:00	2yo3-C	2.6	32.1	106	257	5	MOLECULE:	GENERAL CONTROL PROTEIN GCN4, PUTATIVE INNE	R MEMB	
94:00:00	8xmd-A	2.6	13.5	88	1159	6	MOLECULE:	DNA-DIRECTED RNA POLYMERASE IV SUBUNIT 1		
95:00:00	6cbr-A	2.6	4.2	68	144	9	MOLECULE:	DNA PRIMASE,SINGLE-STRANDED DNA-BINDING PRO	TEIN C	
96:00:00	6pvp-A	2.6	7.4	105	661	10	MOLECULE:	TRANSIENT RECEPTOR POTENTIAL CATION CHANNEL	SUBFA	
97:00:00	7ljn-A	2.6	13.8	87	342	5	MOLECULE:	CD-NTASE		
98:00:00	5mym-C	2.6	4.9	53	205	6	MOLECULE:	HTH-TYPE TRANSCRIPTIONAL REGULATOR ETHR		
99:00:00	8wpe-D	2.6	6.7	48	69	10	MOLECULE:	DNA POLYMERASE		
0:00	2fsf-B	2.6	13.5	56	723	4	MOLECULE:	PREPROTEIN TRANSLOCASE SECA SUBUNIT		
1:00	3p2c-A	2.6	12.1	88	445	8	MOLECULE:	PUTATIVE GLYCOSYL HYDROLASE		
2:00	7wze-A	2.6	5.4	62	161	5	MOLECULE:	UNCHARACTERIZED HTH-TYPE TRANSCRIPTIONAL RE	GULATO	
3:00	7l7v-A	2.6	4.7	49	121	10	MOLECULE:	PROBABLE DISEASE RESISTANCE PROTEIN AT5G669		0
4:00	8tvh-A	2.6	5.3	53	86	6	MOLECULE:	4G5 LIGHT CHAIN		
5:00	6iu4-A	2.6	7.6	72	225	4	MOLECULE:	VIT1		
6:00	7xe1-A	2.6	9.3	65	751	5	MOLECULE:	LYSINE-SPECIFIC HISTONE DEMETHYLASE 1B		
7:00	4gnk-E	2.6	4.8	100	235	1	MOLECULE:	GUANINE NUCLEOTIDE-BINDING PROTEIN G(Q) SUB	UNIT A	
8:00	5a5t-E	2.6	13.9	91	419	4	MOLECULE:	EUKARYOTIC TRANSLATION INITIATION FACTOR 3	SUBUNI	
9:00	3a6m-A	2.6	11.3	90	168	8	MOLECULE:	PROTEIN GRPE		
10:00	8ih5-A	2.6	5.1	75	689	5	MOLECULE:	SYN-COPALYL DIPHOSPHATE SYNTHASE, CHLOROPLA	STIC	

11:00	9b9m-B	2.6	9.6	59	329	3	MOLECULE:	PYRROLOQUINOLINE QUINONE (COENZYME PQQ) BIO	SYNTHE	
12:00	6w98-A	2.6	16.2	90	1312	6	MOLECULE:	F5/8 TYPE C DOMAIN-CONTAINING PROTEIN		
13:00	4di3-A	2.6	17.6	66	273	9	MOLECULE:	TP33 PROTEIN		
14:00	8tzj-C	2.6	3.7	77	203	8	MOLECULE:	CELL DIVISION ATP-BINDING PROTEIN FTSE		
15:00	5xfa-A	2.6	9.9	94	581	7	MOLECULE:	NAD-REDUCING HYDROGENASE		
16:00	6lcn-F	2.6	10.1	72	309	4	MOLECULE:	SERINE O-ACETYLTRANSFERASE		
17:00	7uus-Q	2.6	34.2	109	170	6	MOLECULE:	HYDROGENASE-2, LARGE SUBUNIT		
18:00	5vmn-A	2.6	3.3	46	125	0	MOLECULE:	BAK PROTEIN		
19:00	7xyb-G	2.6	6.5	61	168	8	MOLECULE:	DNA-DIRECTED RNA POLYMERASE SUBUNIT ALPHA		
20:00	8s37-B	2.6	27.7	113	343	6	MOLECULE:	CRISPR TYPE AFERR-ASSOCIATED PROTEIN CSF2		
21:00	8p65-J	2.6	3.2	51	64	6	MOLECULE:	CYTOCHROME B-C1 COMPLEX SUBUNIT 1, MITOCHON	DRIAL	
22:00	5k8o-B	2.6	7	51	425	8	MOLECULE:	5-NITROANTHRANILIC ACID AMINOHYDROLASE		
23:00	8d7h-D	2.6	8.8	66	178	3	MOLECULE:	CILIARY NEUROTROPHIC FACTOR RECEPTOR SUBUNI	T ALPH	
24:00:00	5tk6-A	2.6	7.8	74	191	7	MOLECULE:	OXSA PROTEIN		
25:00:00	5nvu-A	2.6	29	127	3169	0	MOLECULE:	DYNEIN MOTOR DOMAIN		
26:00:00	8yzt-C	2.6	3.2	44	121	11	MOLECULE:	PROTEIN BANP		
27:00:00	6xrw-B	2.6	7.7	56	79	0	MOLECULE:	PLASMID STABILISATION SYSTEM PROTEIN		
28:00:00	8hk0-C	2.6	8.1	105	346	5	MOLECULE:	DEHYDROGENASE		
29:00:00	8zl2-A	2.6	6.7	54	542	6	MOLECULE:	PROTEIN I'M NOT DEAD YET		
30:00:00	5opt-P	2.6	18.3	62	249	8	MOLECULE:	ACTIVATED PROTEIN KINASE C RECEPTOR, PUTATI	VE	
31:00:00	6m9t-A	2.6	4.3	101	444	8	MOLECULE:	PROSTAGLANDIN E2 RECEPTOR EP3 SUBTYPE, ENDO	LYSIN	
32:00:00	2x0l-A	2.6	42.4	122	670	11	MOLECULE:	LYSINE-SPECIFIC HISTONE DEMETHYLASE 1		
33:00:00	2p5g-B	2.6	2.7	40	756	18	MOLECULE:	TEMPLATE DNA		
34:00:00	8i03-F	2.6	25.1	94	228	7	MOLECULE:	PAIRED AMPHIPATHIC HELIX PROTEIN PST1		
35:00:00	8p0w-H	2.6	10.6	76	183	7	MOLECULE:	COMM DOMAIN-CONTAINING PROTEIN 1		
36:00:00	6k9k-A	2.6	14.5	125	2501	9	MOLECULE:	SERINE-PROTEIN KINASE ATM		
37:00:00	3omb-A	2.6	5.8	49	503	6	MOLECULE:	EXTRACELLULAR SOLUTE-BINDING PROTEIN, FAMIL	Y 1	
38:00:00	2epo-A	2.6	18.1	88	626	8	MOLECULE:	N-ACETYL-BETA-D-GLUCOSAMINIDASE		
39:00:00	2qqy-A	2.6	6.1	61	139	5	MOLECULE:	SIGMA B OPERON		
40:00:00	8tcb-C	2.6	2.6	54	80	2	MOLECULE:	C4B-BINDING PROTEIN ALPHA CHAIN		
41:00:00	5djn-C	2.6	3.5	59	81	7	MOLECULE:	KINESIN-LIKE PROTEIN		
42:00:00	6wsm-A	2.6	3.1	53	74	9	MOLECULE:	SEPTIN-8		
43:00:00	4dhx-A	2.6	4.4	56	72	4	MOLECULE:	80 KDA MCM3-ASSOCIATED PROTEIN		
44:00:00	5hda-A	2.6	17	63	121	10	MOLECULE:	ZINC FINGER MYND DOMAIN-CONTAINING PROTEIN		11
45:00:00	2i7u-A	2.6	3.5	50	62	8	MOLECULE:	FOUR-ALPHA-HELIX BUNDLE		
46:00:00	6y09-C	2.6	2.9	59	86	5	MOLECULE:	RAS-RELATED PROTEIN RAB-33B		
47:00:00	3iv1-A	2.6	3.9	58	78	7	MOLECULE:	TUMOR SUSCEPTIBILITY GENE 101 PROTEIN		
48:00:00	4h22-B	2.6	3.6	57	83	11	MOLECULE:	LEUCINE-RICH REPEAT FLIGHTLESS-INTERACTING	PROTEI	
49:00:00	5xmk-G	2.6	5.6	49	54	6	MOLECULE:	VACUOLAR PROTEIN SORTING-ASSOCIATED PROTEIN		4
50:00:00	2vrz-A	2.6	4.6	59	98	8	MOLECULE:	VIRULENCE FACTOR ESXA		
51:00:00	2nr5-E	2.6	2.6	45	65	0	MOLECULE:	HYPOTHETICAL PROTEIN SO2669		
52:00:00	4djg-A	2.6	4.7	44	49	0	MOLECULE:	PLECTIN-RELATED PROTEIN		
53:00:00	3f0c-A	2.6	2.4	44	193	5	MOLECULE:	TRANSCRIPTIONAL REGULATOR		
54:00:00	7y3f-1	2.6	2.5	53	466	2	MOLECULE:	PHOTOSYSTEM I P700 CHLOROPHYLL A APOPROTEIN	A1	
55:00:00	8djk-A	2.6	4.1	54	245	2	MOLECULE:	3-HYDROXY-3-METHYLGLUTARYL-COENZYME A REDUC	TASE	
56:00:00	8on7-A	2.6	7.5	57	525	4	MOLECULE:	FMRFAMIDE-GATED SODIUM CHANNEL 1 (FANAC1)		
57:00:00	3c98-B	2.6	7.9	88	230	7	MOLECULE:	SYNTAXIN-BINDING PROTEIN 1		
58:00:00	2fup-A	2.6	4.3	50	128	2	MOLECULE:	HYPOTHETICAL PROTEIN PA3352		
59:00:00	6yj4-L	2.5	26.2	124	655	7	MOLECULE:	NADH-UBIQUINONE OXIDOREDUCTASE CHAIN 3		
60:00:00	4wpe-A	2.5	32	127	275	8	MOLECULE:	CYTOKINESIS PROTEIN 2		
61:00:00	1wgz-A	2.5	27.3	129	510	7	MOLECULE:	CARBOXYPEPTIDASE 1		
62:00:00	7khw-a	2.5	20.2	68	174	3	MOLECULE:	TRANSLOCON ESPA		
63:00:00	4ihq-A	2.5	27	73	512	7	MOLECULE:	FLAI ATPASE		
64:00:00	6tdv-H	2.5	31.6	126	388	6	MOLECULE:	ATPTB1		
65:00:00	8bfi-B	2.5	28.3	123	574	9	MOLECULE:	CCR4-NOT TRANSCRIPTION COMPLEX SUBUNIT 1		
66:00:00	4ymk-A	2.5	13.3	73	322	5	MOLECULE:	ACYL-COA DESATURASE 1		
67:00:00	3ksy-A	2.5	21.6	147	1007	11	MOLECULE:	SON OF SEVENLESS HOMOLOG 1		
68:00:00	4zuz-B	2.5	28.5	110	858	6	MOLECULE:	SIDC		
69:00:00	7mdy-B	2.5	31.9	103	411	5	MOLECULE:	LIPOPROTEIN TRANSPORTER SUBUNIT LOLE		
70:00:00	5h7b-A	2.5	11	58	230	12	MOLECULE:	IMMUNOGLOBULIN G-BINDING PROTEIN A		
71:00:00	5wgr-A	2.5	29	116	664	6	MOLECULE:	FLAVIN-DEPENDENT HALOGENASE		
72:00:00	7p5j-A	2.5	35.1	87	399	6	MOLECULE:	PROTEIN TWEETY HOMOLOG 1		
73:00:00	8wcn-A	2.5	29.8	123	379	7	MOLECULE:	DIGUANYLATE CYCLASE		
74:00:00	8d8j-d	2.5	26.1	90	660	6	MOLECULE:	PROBABLE S-ADENOSYL-L-METHIONINE-DEPENDENT	RNA	
75:00:00	7e40-D	2.5	26.7	113	345	7	MOLECULE:	PROTEIN PHOSPHATE STARVATION RESPONSE 2		
76:00:00	4uxv-A	2.5	35.7	116	522	7	MOLECULE:	SEPTATION RING FORMATION REGULATOR EZRA		
77:00:00	7bv6-B	2.5	23	45	61	4	MOLECULE:	VESICLE-ASSOCIATED MEMBRANE PROTEIN 8		
78:00:00	2pv4-A	2.5	16.2	45	145	2	MOLECULE:	UNCHARACTERIZED PROTEIN		
79:00:00	6yuf-D	2.5	32.9	119	1272	5	MOLECULE:	COHESIN SUBUNIT RAD21		
80:00:00	7zqy-B	2.5	12.6	71	177	10	MOLECULE:	DH DOMAIN-CONTAINING PROTEIN		
81:00:00	6lkc-B	2.5	17.8	65	533	9	MOLECULE:	POLYUNSATURATED FATTY ACID SYNTHASE PFAD		
82:00:00	6xm1-D	2.5	33.6	100	181	5	MOLECULE:	VPS45		
83:00:00	3etz-B	2.5	17.7	72	115	7	MOLECULE:	ADHESIN A		
84:00:00	7wji-C	2.5	45.9	149	1394	3	MOLECULE:	PROTEIN UNC-80 HOMOLOG		
85:00:00	4m9s-B	2.5	20.2	102	511	5	MOLECULE:	CELL DEATH PROTEIN 4		
86:00:00	7tn2-W	2.5	14.2	61	895	10	MOLECULE:	HISTONE H3		
87:00:00	1knz-A	2.5	9.4	59	154	8	MOLECULE:	5'-R(*UP*GP*AP*CP*C)-3'		
88:00:00	8arl-A	2.5	5.6	99	232	12	MOLECULE:	TRYPTOPHAN-RICH ANTIGEN		
89:00:00	6v6d-A	2.5	5.4	86	209	5	MOLECULE:	PANNEXIN-1		
90:00:00	5me8-A	2.5	5.6	75	107	7	MOLECULE:	INHIBITOR OF GROWTH PROTEIN 5		
91:00:00	6o8b-B	2.5	5.5	99	657	7	MOLECULE:	STIMULATOR OF INTERFERON GENES PROTEIN		
92:00:00	6jfk-A	2.5	22	109	428	4	MOLECULE:	MITOFUSIN-2,CDNA FLJ57997, HIGHLY SIMILAR T	O TRAN	
93:00:00	4w4u-B	2.5	4	46	96	13	MOLECULE:	UBIQUITIN CARBOXYL-TERMINAL HYDROLASE		
94:00:00	3f42-B	2.5	2.1	40	84	5	MOLECULE:	PROTEIN HP0035		
95:00:00	8kg9-E	2.5	24.5	100	568	7	MOLECULE:	DNA REPLICATION LICENSING FACTOR MCM2		
96:00:00	6hcy-A	2.5	9.5	116	436	3	MOLECULE:	METALLOREDUCTASE STEAP4		
97:00:00	3j9t-P	2.5	21.6	90	461	3	MOLECULE:	V-TYPE PROTON ATPASE SUBUNIT D		
98:00:00	9d80-C	2.5	21.7	93	275	6	MOLECULE:	PORTAL PROTEIN		
99:00:00	6inw-B	2.5	14	59	391	7	MOLECULE:	O-METHYLTRANSFERASE LEPI		
0:00	6k3b-B	2.5	3.9	50	106	8	MOLECULE:	LPG2147		
1:00	7zn7-D	2.5	5.8	61	128	7	MOLECULE:	DNA DAMAGE-BINDING PROTEIN 1		
2:00	5c8d-E	2.5	8.4	73	280	3	MOLECULE:	LIGHT-DEPENDENT TRANSCRIPTIONAL REGULATOR C	ARH	
3:00	7apk-m	2.5	44.3	160	549	8	MOLECULE:	THO COMPLEX SUBUNIT 1		
4:00	3oop-A	2.5	9.6	77	139	4	MOLECULE:	LIN2960 PROTEIN		
5:00	5fia-B	2.5	6.3	67	397	9	MOLECULE:	LPIR1		
6:00	1oxz-A	2.5	4.1	88	132	7	MOLECULE:	ADP-RIBOSYLATION FACTOR BINDING PROTEIN GGA		1
7:00	1t7s-A	2.5	11.1	85	129	5	MOLECULE:	BAG-1 COCHAPERONE		
8:00	7nhr-A	2.5	11	74	663	3	MOLECULE:	PUTATIVE TRANSMEMBRANE PROTEIN WZC		
9:00	2bb6-A	2.5	18.4	80	414	9	MOLECULE:	TRANSCOBALAMIN II		
10:00	6rd4-5	2.5	42.7	92	123	7	MOLECULE:	ASA-10: POLYTOMELLA F-ATP SYNTHASE ASSOCIAT	ED SUB	
11:00	7ugc-A	2.5	17.3	71	191	1	MOLECULE:	PROTEIN VIAA		

12:00	2pjw-H	2.5	4	48	88	6	MOLECULE:	UNCHARACTERIZED PROTEIN YHL002W		
13:00	6txb-A	2.5	6.4	65	263	3	MOLECULE:	UBIQUITIN CARBOXYL-TERMINAL HYDROLASE MINDY		-1
14:00	6thk-A	2.5	9.7	115	466	6	MOLECULE:	PYOCIN S5		
15:00	6rjw-A	2.5	5.9	72	161	10	MOLECULE:	LYSM DOMAIN PROTEIN		
16:00	7c7e-A	2.5	3.9	61	142	10	MOLECULE:	PUTATIVE DNA-BINDING TRANSCRIPTIONAL REGULA	TOR	
17:00	7emf-H	2.5	10.1	106	181	7	MOLECULE:	MEDIATOR OF RNA POLYMERASE II TRANSCRIPTION	SUBUN	
18:00	3fcn-A	2.5	6.1	62	158	6	MOLECULE:	AN ALPHA-HELICAL PROTEIN OF UNKNOWN FUNCTIO	N (PFA	
19:00	4nqw-B	2.5	7	55	73	5	MOLECULE:	ECF RNA POLYMERASE SIGMA FACTOR SIGK		
20:00	9f5y-I	2.5	14	54	135	9	MOLECULE:	NADH:UBIQUINONE OXIDOREDUCTASE 24 KD SUBUNI	T	
21:00	8urw-Z	2.5	8.3	69	1235	6	MOLECULE:	DNA-DIRECTED RNA POLYMERASE SUBUNIT ALPHA		
22:00	6wgm-A	2.5	11.8	74	305	5	MOLECULE:	TRAP-TYPE C4-DICARBOXYLATE TRANSPORT SYSTEM	, PERI	
23:00	7w0y-i	2.5	7.5	92	347	8	MOLECULE:	NADH DEHYDROGENASE [UBIQUINONE] FLAVOPROTEI	N 1,	
24:00:00	6n39-A	2.5	18.3	88	404	6	MOLECULE:	DEPHOSPHO-COA KINASE		
25:00:00	8dft-A	2.5	5	83	112	6	MOLECULE:	PILIN PROTEIN		
26:00:00	8fn4-4	2.5	30.5	107	741	2	MOLECULE:	RNA-EDITING SUBSTRATE-BINDING COMPLEX PROTE	IN 1 (	
27:00:00	7lhe-A	2.5	39	155	2222	10	MOLECULE:	INOSITOL 1,4,5-TRISPHOSPHATE RECEPTOR TYPE		1
28:00:00	4kk0-A	2.5	11.1	83	403	4	MOLECULE:	TUBEROUS SCLEROSIS 1 PROTEIN HOMOLOG		
29:00:00	9iz7-A	2.5	48.4	162	2611	7	MOLECULE:	SERINE/THREONINE-PROTEIN KINASE TEL1		
30:00:00	2g0u-A	2.5	4.2	67	92	4	MOLECULE:	TYPE III SECRETION SYSTEM NEEDLE PROTEIN		
31:00:00	6n63-A	2.5	5.8	86	141	3	MOLECULE:	ENCAPSULIN CARGO PROTEIN		
32:00:00	3ajb-A	2.5	2.8	52	293	2	MOLECULE:	PEROXISOMAL BIOGENESIS FACTOR 3		
33:00:00	9d80-B	2.5	11.6	83	234	4	MOLECULE:	PORTAL PROTEIN		
34:00:00	2pzi-A	2.5	20.9	62	654	10	MOLECULE:	PROBABLE SERINE/THREONINE-PROTEIN KINASE PK	NG	
35:00:00	6qyi-B	2.5	11.6	78	519	8	MOLECULE:	4-HYDROXYPHENYLACETATE 3-MONOOXYGENASE OXYG	ENASE	
36:00:00	5cgz-A	2.5	7.1	51	240	8	MOLECULE:	4-OXALMESACONATE HYDRATASE		
37:00:00	6v92-R	2.5	20	156	325	5	MOLECULE:	ACTIN-RELATED PROTEIN 7		
38:00:00	5mx5-L	2.5	5.4	86	214	7	MOLECULE:	PROTEASOME ACTIVATOR COMPLEX SUBUNIT 1		
39:00:00	2hsz-A	2.5	6.6	59	225	10	MOLECULE:	NOVEL PREDICTED PHOSPHATASE		
40:00:00	8c0v-B	2.5	22	86	1030	10	MOLECULE:	PEROXISOMAL ATPASE PEX1		
41:00:00	6whg-A	2.5	6.7	104	523	8	MOLECULE:	CALCIUM CHANNEL YVC1		
42:00:00	8pnu-A	2.5	7.2	61	155	5	MOLECULE:	STYRENE OXIDE ISOMERASE		
43:00:00	9im6-A	2.5	22.6	84	1095	5	MOLECULE:	IMPORTIN-5		
44:00:00	6ezn-B	2.5	5.8	80	110	1	MOLECULE:	DOLICHYL-DIPHOSPHOOLIGOSACCHARIDE--PROTEIN		
45:00:00	2zqm-A	2.5	2.8	76	114	7	MOLECULE:	PREFOLDIN BETA SUBUNIT 1		
46:00:00	2hh6-A	2.5	2.3	42	112	10	MOLECULE:	BH3980 PROTEIN		
47:00:00	8rc4-o	2.5	5.7	78	372	5	MOLECULE:	SERINE/THREONINE-PROTEIN PHOSPHATASE 2A 65	KDA RE	
48:00:00	7xsq-A	2.5	24.7	94	1289	6	MOLECULE:	RAMP SUPERFAMILY PROTEIN		
49:00:00	7mwa-A	2.5	6.2	63	383	0	MOLECULE:	2-POLYPRENYL-6-METHOXYPHENOL 4-HYDROXYLASE		
50:00:00	7w9l-A	2.5	20.8	120	1422	8	MOLECULE:	SODIUM CHANNEL PROTEIN TYPE 9 SUBUNIT ALPHA		
51:00:00	9f5y-U	2.5	7.6	82	105	1	MOLECULE:	NADH:UBIQUINONE OXIDOREDUCTASE 24 KD SUBUNI	T	
52:00:00	8rbz-j	2.5	13.8	96	668	4	MOLECULE:	UNK-UNK-UNK-UNK-UNK-UNK-UNK-UNK-UNK		
53:00:00	4ams-A	2.5	8.3	86	367	7	MOLECULE:	MG662		
54:00:00	7zr6-C	2.5	30	130	277	5	MOLECULE:	HEAT SHOCK PROTEIN HSP 90-BETA		
55:00:00	3p8c-D	2.5	21.9	121	204	5	MOLECULE:	CYTOPLASMIC FMR1-INTERACTING PROTEIN 1		
56:00:00	7qla-C	2.5	18.7	76	441	9	MOLECULE:	VACUOLAR FUSION PROTEIN MON1		
57:00:00	3aai-A	2.5	3.2	63	78	8	MOLECULE:	COPPER HOMEOSTASIS OPERON REGULATORY PROTEI	N	
58:00:00	5yfp-D	2.5	18.8	95	954	6	MOLECULE:	EXOCYST COMPLEX COMPONENT SEC3		
59:00:00	8j1p-A	2.5	32.5	95	1234	5	MOLECULE:	UBIQUITIN FUSION DEGRADATION PROTEIN 4		
60:00:00	1zgh-A	2.5	10.3	47	227	13	MOLECULE:	METHIONYL-TRNA FORMYLTRANSFERASE		
61:00:00	8g0l-B	2.5	7	88	925	7	MOLECULE:	N-ALPHA-ACETYLTRANSFERASE 20		
62:00:00	6edw-B	2.5	19.5	109	746	6	MOLECULE:	ISOCITRATE LYASE 2		
63:00:00	8e1m-C	2.5	11.5	81	264	7	MOLECULE:	MITOCHONDRIAL IMPORT INNER MEMBRANE TRANSLO	CASE S	
64:00:00	4m75-A	2.5	4.5	43	139	16	MOLECULE:	U6 SNRNA-ASSOCIATED SM-LIKE PROTEIN LSM1		
65:00:00	8gl3-A	2.5	4.1	92	189	5	MOLECULE:	PRLB-519		
66:00:00	7bc6-A	2.5	15.6	82	432	6	MOLECULE:	PROTON-COUPLED FOLATE TRANSPORTER		
67:00:00	6gvw-F	2.5	29.9	82	320	7	MOLECULE:	BRCA1-A COMPLEX SUBUNIT ABRAXAS 1		
68:00:00	8pmq-2	2.5	11.8	96	355	7	MOLECULE:	E3 UBIQUITIN-PROTEIN LIGASE RMD5		
69:00:00	7wwb-A	2.5	14.2	53	333	0	MOLECULE:	CHOLINE TRANSPORTER-LIKE PROTEIN 1		
70:00:00	6rgn-A	2.5	3.8	55	93	4	MOLECULE:	DUF3120 DOMAIN-CONTAINING PROTEIN		
71:00:00	6qo1-A	2.5	4.6	88	186	5	MOLECULE:	PUTATIVE SURFACE PROTEIN		
72:00:00	6c0g-A	2.5	5	48	114	4	MOLECULE:	LYSINOALANINE SYNTHASE		
73:00:00	6wlw-0	2.5	3.8	69	204	3	MOLECULE:	V-TYPE PROTON ATPASE 21 KDA PROTEOLIPID SUB	UNIT	
74:00:00	8iff-A	2.5	10.9	99	868	8	MOLECULE:	PHYTOCHROME A		
75:00:00	7xzj-G	2.5	4.4	55	73	2	MOLECULE:	CTAP3		
76:00:00	6mj1-A	2.5	9.6	73	200	7	MOLECULE:	PROBABLE HTH-TYPE TRANSCRIPTIONAL REGULATOR	YTTP	
77:00:00	7d60-B	2.5	20.9	101	288	7	MOLECULE:	CALCIUM HOMEOSTASIS MODULATOR PROTEIN 5		
78:00:00	6r6h-O	2.5	30.5	90	744	3	MOLECULE:	COP9 SIGNALOSOME COMPLEX SUBUNIT 1		
79:00:00	5no8-A	2.5	13.6	73	669	4	MOLECULE:	BACCELL_00875		
80:00:00	6s2e-A	2.5	26.7	100	1006	7	MOLECULE:	DNA POLYMERASE EPSILON CATALYTIC SUBUNIT A		
81:00:00	6tdw-C	2.5	3.5	48	157	6	MOLECULE:	ATPTB1		
82:00:00	6fpg-F	2.5	9.2	78	266	8	MOLECULE:	CHROMOSOME 16, WHOLE GENOME SHOTGUN SEQUEN	E	
83:00:00	2oap-1	2.5	3.1	38	498	5	MOLECULE:	TYPE II SECRETION SYSTEM PROTEIN		
84:00:00	6n8a-B	2.5	4.2	69	194	7	MOLECULE:	TRANSCRIPTION REGULATOR ACAB		
85:00:00	2pgc-A	2.5	15.6	58	206	2	MOLECULE:	UNCHARACTERIZED PROTEIN		
86:00:00	5ex8-A	2.5	5.4	71	414	13	MOLECULE:	CYTOCHROME P450		
87:00:00	8w8q-C	2.5	7.6	79	287	4	MOLECULE:	SOLUBLE CYTOCHROME B562,PROBABLE G-PROTEIN	COUPLE	
88:00:00	3kkz-A	2.5	7.8	58	257	3	MOLECULE:	UNCHARACTERIZED PROTEIN Q5LES9		
89:00:00	7edx-k	2.5	8.7	69	98	7	MOLECULE:	TRANSCRIPTION INITIATION FACTOR TFIID SUBUN	IT 1	
90:00:00	3jav-A	2.5	32.7	152	868	8	MOLECULE:	INOSITOL 1,4,5-TRISPHOSPHATE RECEPTOR TYPE		1
91:00:00	8ik3-A	2.5	19.1	113	328	4	MOLECULE:	STIMULATOR OF INTERFERON GENES PROTEIN,IMMU	NE PRO	
92:00:00	7xl9-A	2.5	11.7	88	164	6	MOLECULE:	TRANSCRIPTIONAL REGULATOR, MARR FAMILY		
93:00:00	7ldg-A	2.5	9.4	70	220	4	MOLECULE:	HEAT SHOCK FACTOR 2-BINDING PROTEIN		
94:00:00	2oeq-C	2.5	14.9	76	117	5	MOLECULE:	PROTEIN OF UNKNOWN FUNCTION, DUF964		
95:00:00	2o26-A	2.5	6.5	73	144	5	MOLECULE:	KIT LIGAND		
96:00:00	6nd4-M	2.5	12.5	94	431	5	MOLECULE:	ETS RRNA		
97:00:00	7nb6-A	2.5	7.9	81	340	4	MOLECULE:	AI-2 TRANSPORT PROTEIN TQSA		
98:00:00	6h3d-A	2.5	5.3	48	429	4	MOLECULE:	DUF2338 DOMAIN-CONTAINING PROTEIN		
99:00:00	3b8m-A	2.5	7.4	68	255	6	MOLECULE:	FERRIC ENTEROBACTIN (ENTEROCHELIN) TRANSPOR	T	
0:00	2lf0-A	2.5	8.6	71	120	7	MOLECULE:	UNCHARACTERIZED PROTEIN YIBL		
1:00	4om3-D	2.5	7.3	54	122	7	MOLECULE:	TRANSDUCIN-LIKE ENHANCER PROTEIN 1		
2:00	4zqa-A	2.5	3.8	58	86	2	MOLECULE:	SIN3 HISTONE DEACETYLASE COREPRESSOR COMPLE	X COMP	
3:00	8sdi-A	2.5	4.2	56	80	7	MOLECULE:	TRIPARTITE MOTIF-CONTAINING PROTEIN 45		
4:00	6oqa-G	2.5	17	58	87	9	MOLECULE:	PEPTIDYL-PROLYL CIS-TRANS ISOMERASE FKBP1A		
5:00	2jee-A	2.5	3.4	55	78	9	MOLECULE:	CELL DIVISION PROTEIN ZAPB		
6:00	4lnb-A	2.5	2.7	39	339	3	MOLECULE:	CAAX FARNESYLTRANSFERASE ALPHA SUBUNIT RAM2		
7:00	5yfp-E	2.5	15.1	110	776	8	MOLECULE:	EXOCYST COMPLEX COMPONENT SEC3		
8:00	5oi7-A	2.5	3.5	56	88	4	MOLECULE:	CENTROSOMAL PROTEIN OF 85 KDA		
9:00	8fck-F	2.5	29.9	88	393	5	MOLECULE:	HAUS AUGMIN-LIKE COMPLEX SUBUNIT 1		
10:00	2wz7-B	2.5	3.2	54	67	0	MOLECULE:	UNCHARACTERIZED PROTEIN YBGF		
11:00	8gt9-A	2.5	4.4	54	91	2	MOLECULE:	BECLIN-2		
12:00	6vhf-A	2.5	3.7	61	140	7	MOLECULE:	PHD-TYPE DOMAIN-CONTAINING PROTEIN		

13:00	6nbx-C	2.5	16.7	73	100	4	MOLECULE:	NAD(P)H-QUINONE OXIDOREDUCTASE SUBUNIT 1	
14:00	6t9i-T	2.5	21.9	57	3513	11	MOLECULE:	TRANSCRIPTION FACTOR SPT20	
15:00	6m17-A	2.5	11.1	72	606	6	MOLECULE:	SODIUM-DEPENDENT NEUTRAL AMINO ACID TRANSPO	RTER B
16:00	7aqq-N	2.5	10.4	94	488	3	MOLECULE:	NADH-UBIQUINONE OXIDOREDUCTASE CHAIN 3	
17:00	3vuq-C	2.5	10.3	56	179	2	MOLECULE:	TRANSCRIPTIONAL REGULATOR (TETR/ACRR FAMILY	)
18:00	5xvj-A	2.5	6.8	67	134	6	MOLECULE:	PHD FINGER PROTEIN ALFIN-LIKE 7	
19:00	7d3e-A	2.5	18.6	86	1380	2	MOLECULE:	DUAL OXIDASE 1	
20:00	1fxk-C	2.5	4.8	58	133	10	MOLECULE:	PREFOLDIN	
21:00	5xbj-A	2.4	32.2	126	500	9	MOLECULE:	FLAGELLAR HOOK-ASSOCIATED PROTEIN FLGK	
22:00	9mty-B	2.4	26.8	94	294	4	MOLECULE:	TRANSPOSASE IS116/IS110/IS902 C-TERMINAL DO	MAIN-C
23:00	6t8d-X	2.4	33.1	147	355	5	MOLECULE:	MAKB	
24:00:00	4whj-A	2.4	16.6	88	565	8	MOLECULE:	INTERFERON-INDUCED GTP-BINDING PROTEIN MX2	
25:00:00	8ti8-C	2.4	11.6	72	259	7	MOLECULE:	SHEDU PROTEIN SDUA	
26:00:00	7qpg-R	2.4	33.6	145	2208	8	MOLECULE:	PROTEIN ZWILCH HOMOLOG	
27:00:00	7s9w-A	2.4	25.4	105	1238	5	MOLECULE:	DRMA	
28:00:00	9fth-A	2.4	27.7	82	601	10	MOLECULE:	NEUROTRANSMITTER-GATED ION-CHANNEL LIGAND-B	INDING
29:00:00	7crc-E	2.4	17.7	55	237	5	MOLECULE:	NAD+ HYDROLASE (NADASE)	
30:00:00	6qum-N	2.4	23.4	101	649	5	MOLECULE:	V-TYPE ATP SYNTHASE ALPHA CHAIN	
31:00:00	5to5-A	2.4	32.7	97	141	10	MOLECULE:	NUCLEOPROTEIN TPR	
32:00:00	8u1a-A	2.4	14.1	66	324	11	MOLECULE:	DESIGNED NEAR INFRARED FLUORESCENT PROTEIN	MC7BP3
33:00:00	8jzr-B	2.4	34.3	131	456	5	MOLECULE:	LYSOSOMAL TRANSPORTER,ALFA TAG	
34:00:00	4owt-A	2.4	23.8	87	466	9	MOLECULE:	INTEGRATOR COMPLEX SUBUNIT 3	
35:00:00	7y8r-P	2.4	31	101	385	6	MOLECULE:	HISTONE H3	
36:00:00	5c1f-A	2.4	29.5	128	298	13	MOLECULE:	SEPTATION PROTEIN IMP2	
37:00:00	2ksr-A	2.4	19.4	58	140	5	MOLECULE:	NEURONAL ACETYLCHOLINE RECEPTOR SUBUNIT BET	A-2
38:00:00	6ek4-A	2.4	23.5	153	342	4	MOLECULE:	PAXB	
39:00:00	8csp-8	2.4	27.1	97	326	6	MOLECULE:	28S RIBOSOMAL PROTEIN S34, MITOCHONDRIAL	
40:00:00	7c9m-C	2.4	20.7	70	260	4	MOLECULE:	D-HISTIDINE 2-AMINOBUTANOYLTRANSFERASE	
41:00:00	8pqw-F	2.4	43.3	130	150	7	MOLECULE:	CYTOPLASMIC DYNEIN 1 HEAVY CHAIN 1	
42:00:00	7d2w-A	2.4	15.2	58	125	7	MOLECULE:	PRESAN DOMAIN-CONTAINING PROTEIN	
43:00:00	8gjg-A	2.4	25.8	91	162	8	MOLECULE:	GLUC_A04_0005 BINDER	
44:00:00	6jho-A	2.4	32.8	113	200	6	MOLECULE:	CAG PATHOGENICITY ISLAND PROTEIN (CAG6)	
45:00:00	5od9-A	2.4	4	41	95	5	MOLECULE:	MID1SC9	
46:00:00	7ar9-p	2.4	7.6	95	155	4	MOLECULE:	ND3	
47:00:00	5ve8-B	2.4	26.1	119	1030	9	MOLECULE:	KAP123	
48:00:00	1s5j-A	2.4	22.7	98	727	9	MOLECULE:	DNA POLYMERASE I	
49:00:00	5b04-B	2.4	15	56	319	7	MOLECULE:	TRANSLATION INITIATION FACTOR EIF-2B SUBUNI	T ALPH
50:00:00	7lga-D	2.4	3	48	91	8	MOLECULE:	RETROTRANSPOSON-DERIVED PROTEIN PEG10	
51:00:00	5d2s-A	2.4	15.7	72	91	6	MOLECULE:	FIBROIN-MODULATOR-BINDING PROTEIN-1	
52:00:00	6nr8-2	2.4	4.6	72	103	6	MOLECULE:	PREFOLDIN SUBUNIT 1	
53:00:00	3dfu-A	2.4	4.1	69	228	6	MOLECULE:	UNCHARACTERIZED PROTEIN FROM 6-PHOSPHOGLUCO	NATE
54:00:00	2id3-A	2.4	5.5	56	191	11	MOLECULE:	PUTATIVE TRANSCRIPTIONAL REGULATOR	
55:00:00	3cqx-D	2.4	2.9	65	84	6	MOLECULE:	HEAT SHOCK COGNATE 71 KDA PROTEIN	
56:00:00	5iqc-A	2.4	12.9	81	301	10	MOLECULE:	BIFUNCTIONAL AAC/APH	
57:00:00	6yxj-B	2.4	9	74	218	5	MOLECULE:	NON-STRUCTURAL PROTEIN 3	
58:00:00	9c2d-A	2.4	10.9	83	366	5	MOLECULE:	MAJOR CAPSID PROTEIN	
59:00:00	5tr1-A	2.4	20.4	87	606	2	MOLECULE:	CHLORIDE CHANNEL PROTEIN	
60:00:00	2h09-A	2.4	5.1	59	127	10	MOLECULE:	TRANSCRIPTIONAL REGULATOR MNTR	
61:00:00	3wxx-E	2.4	8.6	70	151	7	MOLECULE:	ACRH	
62:00:00	7vug-R	2.4	5.2	107	282	3	MOLECULE:	GUANINE NUCLEOTIDE-BINDING PROTEIN G(I) SUB	UNIT A
63:00:00	3k1m-B	2.4	16	83	305	7	MOLECULE:	HTH-TYPE TRANSCRIPTIONAL REGULATOR BENM	
64:00:00	3s84-B	2.4	6.6	81	241	5	MOLECULE:	APOLIPOPROTEIN A-IV	
65:00:00	1i49-A	2.4	17.7	103	201	8	MOLECULE:	ARFAPTIN 2	
66:00:00	8ck1-E	2.4	13.9	98	206	5	MOLECULE:	TAIL NOZZLE	
67:00:00	6djl-E	2.4	8.8	121	226	8	MOLECULE:	RAS-RELATED PROTEIN RAB-11A	
68:00:00	9irz-A	2.4	4.1	55	96	5	MOLECULE:	PROBABLE HTH-TYPE TRANSCRIPTIONAL REGULATOR	YHAJ
69:00:00	7eu3-G	2.4	8.2	74	176	1	MOLECULE:	NAD(P)H-QUINONE OXIDOREDUCTASE SUBUNIT 1, C	HLOROP
70:00:00	2qup-A	2.4	4.1	67	119	9	MOLECULE:	BH1478 PROTEIN	
71:00:00	5kay-B	2.4	5.2	80	200	6	MOLECULE:	SPELTER	
72:00:00	6b4h-A	2.4	12.5	80	318	3	MOLECULE:	NUCLEOPORIN AMO1	
73:00:00	4r3z-A	2.4	3.8	53	76	4	MOLECULE:	AMINOACYL TRNA SYNTHASE COMPLEX-INTERACTING	MULTI
74:00:00	7v1n-A	2.4	29.1	108	2344	8	MOLECULE:	TOXIN B	
75:00:00	3u9j-A	2.4	7.1	65	157	6	MOLECULE:	F-BOX/LRR-REPEAT PROTEIN 5	
76:00:00	8h6h-A	2.4	12.7	91	986	8	MOLECULE:	CELLODEXTRIN PHOSPHORYLASE	
77:00:00	6bq1-E	2.4	15.1	115	1510	7	MOLECULE:	PHOSPHATIDYLINOSITOL 4-KINASE III ALPHA (PI	4KA)
78:00:00	6db1-A	2.4	3.7	69	137	10	MOLECULE:	PUTATIVE METHYL-ACCEPTING CHEMOTAXIS PROTEI	N
79:00:00	2o34-A	2.4	6.7	61	249	8	MOLECULE:	HYPOTHETICAL PROTEIN	
80:00:00	7bi2-A	2.4	25.9	94	1021	9	MOLECULE:	PHOSPHATIDYLINOSITOL 4-PHOSPHATE 3-KINASE C	2 DOMA
81:00:00	5hmo-A	2.4	3.8	48	131	10	MOLECULE:	UNCONVENTIONAL MYOSIN-X	
82:00:00	8jxo-A	2.4	9.1	83	262	6	MOLECULE:	BLCHR2	
83:00:00	7pkq-w	2.4	36.5	118	155	8	MOLECULE:	MS35	
84:00:00	8gju-F	2.4	4.8	61	332	3	MOLECULE:	METHYLMALONIC ACIDURIA TYPE A PROTEIN, MITO	CHONDR
85:00:00	8xej-X	2.4	4.9	62	358	8	MOLECULE:	ISOFORM 2 OF BASIGIN	
86:00:00	7nvr-3	2.4	16.9	73	214	12	MOLECULE:	TFIIH BASAL TRANSCRIPTION FACTOR COMPLEX HE	LICASE
87:00:00	6j52-A	2.4	8.2	50	94	4	MOLECULE:	CASPASE RECRUITMENT DOMAIN-ONLY PROTEIN	
88:00:00	8eja-A	2.4	5.8	75	113	5	MOLECULE:	ABAK	
89:00:00	6xxv-C	2.4	4.9	71	111	6	MOLECULE:	ANTIBODY C57, HEAVY CHAIN	
90:00:00	2rkh-A	2.4	5	60	164	0	MOLECULE:	PUTATIVE APHA-LIKE TRANSCRIPTION FACTOR	
91:00:00	8jps-A	2.4	9.9	71	257	4	MOLECULE:	ATYPICAL CHEMOKINE RECEPTOR 1	
92:00:00	1kf6-D	2.4	5.6	96	119	6	MOLECULE:	FUMARATE REDUCTASE FLAVOPROTEIN	
93:00:00	5mq4-C	2.4	19	84	117	4	MOLECULE:	PROTEIN KINASE C-BINDING PROTEIN 1	
94:00:00	5xef-A	2.4	4.3	75	123	3	MOLECULE:	FLAGELLAR PROTEIN FLIS	
95:00:00	1na6-B	2.4	5.7	75	395	4	MOLECULE:	RESTRICTION ENDONUCLEASE ECORII	
96:00:00	8iq4-R	2.4	4.2	95	274	9	MOLECULE:	PROSTAGLANDIN F2-ALPHA RECEPTOR	
97:00:00	4dra-H	2.4	7.5	63	76	2	MOLECULE:	CENTROMERE PROTEIN S	
98:00:00	6xby-a	2.4	7.3	77	494	4	MOLECULE:	V-TYPE PROTON ATPASE CATALYTIC SUBUNIT A	
99:00:00	8hf2-A	2.4	6.1	83	120	4	MOLECULE:	PRA1 FAMILY PROTEIN	
0:00	4uw9-A	2.4	5.9	71	228	6	MOLECULE:	BETA-PHOSPHOGLUCOMUTASE	
1:00	8p62-D	2.4	21	88	245	10	MOLECULE:	DNA REPLICATION LICENSING FACTOR MCM2	
2:00	8qx8-E	2.4	5.3	46	216	13	MOLECULE:	VACUOLAR PROTEIN SORTING-ASSOCIATED PROTEIN	8
3:00	7jrl-A	2.4	20.4	96	457	4	MOLECULE:	F5/8 TYPE C DOMAIN PROTEIN	
4:00	6ox1-B	2.4	11.9	103	485	4	MOLECULE:	ACTIN, CYTOPLASMIC 1	
5:00	8vti-A	2.4	6.5	84	358	4	MOLECULE:	ISOFORM 4 OF ADHESION G PROTEIN-COUPLED REC	EPTOR
6:00	2xpl-A	2.4	4.2	54	142	9	MOLECULE:	IWS1	
7:00	6cv9-A	2.4	21.9	101	316	8	MOLECULE:	SHORT TRANSIENT RECEPTOR POTENTIAL CHANNEL	6
8:00	8j0o-C	2.4	29.6	80	258	5	MOLECULE:	ER MEMBRANE PROTEIN COMPLEX SUBUNIT 1	
9:00	8v14-A	2.4	14.2	49	167	4	MOLECULE:	NMDA RECEPTOR AUXILIARY PROTEIN	
10:00	7zet-A	2.4	23.6	91	377	4	MOLECULE:	CLUSTERIN	
11:00	5gqu-A	2.4	5.8	60	755	7	MOLECULE:	1,4-ALPHA-GLUCAN BRANCHING ENZYME GLGB	
12:00	5j65-A	2.4	15.4	101	402	5	MOLECULE:	PESTICIDAL CRYSTAL PROTEIN CRY6AA	
13:00	6prk-A	2.4	13.5	85	115	7	MOLECULE:	RICF	

14:00	7v7b-A	2.4	25.3	118	1110	4	MOLECULE:	DDB1- AND CUL4-ASSOCIATED FACTOR 1		
15:00	8ovb-C	2.4	4.6	100	266	10	MOLECULE:	COMPLEMENT C3F FRAGMENT		
16:00	5myi-D	2.4	6	62	165	8	MOLECULE:	DUTPASE FROM DI S. AUREUS PHAGE		
17:00	8eki-E	2.4	13.6	87	643	7	MOLECULE:	PROTEIN TRANSPORT PROTEIN SEC20		
18:00	2fo1-D	2.4	3.5	44	63	5	MOLECULE:	5'-		
19:00	8p1l-A	2.4	29.1	136	2036	3	MOLECULE:	RNA-DIRECTED RNA POLYMERASE L		
20:00	7usd-A	2.4	25.2	161	1185	4	MOLECULE:	CYTOPLASMIC FMR1-INTERACTING PROTEIN 1		
21:00	8ub7-B	2.4	8.5	58	122	7	MOLECULE:	REVERSE TRANSCRIPTASE		
22:00	5oqm-h	2.4	27	93	131	6	MOLECULE:	DNA-DIRECTED RNA POLYMERASE II SUBUNIT RPB1		
23:00	5yx5-B	2.4	10.8	71	270	7	MOLECULE:	49 PROTEIN		
24:00:00	5c0x-F	2.4	3.7	40	215	5	MOLECULE:	EXOSOME COMPLEX COMPONENT RRP45		
25:00:00	6v0t-A	2.4	22.2	81	402	6	MOLECULE:	[PYRUVATE DEHYDROGENASE [ACETYL-TRANSFERRIN	G]]-PH	
26:00:00	1wle-B	2.4	24.2	137	470	7	MOLECULE:	SERYL-TRNA SYNTHETASE		
27:00:00	6ch2-E	2.4	3.9	64	147	6	MOLECULE:	FLAGELLAR BIOSYNTHESIS PROTEIN FLHA		
28:00:00	6noy-A	2.4	3.9	47	80	4	MOLECULE:	MAINTENANCE OF CARBOXYSOME POSITIONING B PR	OTEIN,	
29:00:00	9l2q-A	2.4	10.4	59	105	14	MOLECULE:	H2C2		
30:00:00	4xwj-A	2.4	4	71	153	4	MOLECULE:	REGULATOR OF SIGMA D		
31:00:00	7z1n-O	2.4	8.8	84	570	6	MOLECULE:	DNA-DIRECTED RNA POLYMERASE III SUBUNIT RPC		1
32:00:00	7obq-u	2.4	6	78	441	6	MOLECULE:	SRP RNA		
33:00:00	5vkv-A	2.4	15.3	62	224	3	MOLECULE:	CYTOCHROME C-TYPE BIOGENESIS PROTEIN CCDA		
34:00:00	3edv-A	2.4	17	101	322	7	MOLECULE:	SPECTRIN BETA CHAIN, BRAIN 1		
35:00:00	8xku-B	2.4	26.3	95	845	7	MOLECULE:	PROBABLE INACTIVE ATP-DEPENDENT ZINC METALL	OPROTE	
36:00:00	8cbk-E	2.4	4.2	38	470	3	MOLECULE:	3-HYDROXYACYL-COA DEHYDROGENASE TYPE-2		
37:00:00	7f52-A	2.4	16.3	123	584	9	MOLECULE:	NON-STRUCTURAL PROTEIN 2		
38:00:00	5lxj-A	2.4	2.3	36	53	8	MOLECULE:	PHOSPHOPROTEIN		
39:00:00	2q1f-A	2.4	24.9	123	991	7	MOLECULE:	CHONDROITINASE		
40:00:00	6t9i-I	2.4	11.6	84	173	4	MOLECULE:	TRANSCRIPTION FACTOR SPT20		
41:00:00	6xz4-A	2.4	9.9	87	304	5	MOLECULE:	TALIN ROD DOMAIN-CONTAINING PROTEIN 1		
42:00:00	4bt9-B	2.4	22.7	107	238	7	MOLECULE:	PROLYL 4-HYDROXYLASE SUBUNIT ALPHA-1		
43:00:00	6vdq-A	2.4	10.9	77	305	4	MOLECULE:	3-METHYL-L-TYROSINE PEROXYGENASE		
44:00:00	6wuc-K	2.4	20.8	109	235	7	MOLECULE:	INNER KINETOCHORE SUBUNIT MCM16		
45:00:00	1ox3-A	2.4	7.8	68	108	1	MOLECULE:	FIBRITIN		
46:00:00	8pmq-9	2.4	28	114	412	7	MOLECULE:	E3 UBIQUITIN-PROTEIN LIGASE RMD5		
47:00:00	4k2p-A	2.4	7.7	80	238	13	MOLECULE:	T-LYMPHOMA INVASION AND METASTASIS-INDUCING	PROTE	
48:00:00	3hai-A	2.4	31	112	294	10	MOLECULE:	HUMAN PACSIN1 F-BAR		
49:00:00	5z7g-C	2.4	4.1	41	43	10	MOLECULE:	TAX1-BINDING PROTEIN 1		
50:00:00	6oap-A	2.4	4.8	44	305	7	MOLECULE:	DUAL SENSOR HISTIDINE KINASE		
51:00:00	5ool-v	2.4	2.7	41	69	7	MOLECULE:	16S RIBOSOMAL RNA		
52:00:00	4abn-B	2.4	14.3	86	426	2	MOLECULE:	TETRATRICOPEPTIDE REPEAT PROTEIN 5		
53:00:00	6vzf-A	2.4	3.9	59	86	8	MOLECULE:	AUTOPHAGY-RELATED PROTEIN 11		
54:00:00	5h07-D	2.4	13.4	57	78	9	MOLECULE:	POLYUBIQUITIN-C		
55:00:00	1x79-B	2.4	3.4	59	90	12	MOLECULE:	ADP-RIBOSYLATION FACTOR BINDING PROTEIN GGA		1
56:00:00	1fav-A	2.4	4.2	58	78	7	MOLECULE:	HIV-1 ENVELOPE PROTEIN CHIMERA		
57:00:00	5cff-B	2.4	3.3	55	88	9	MOLECULE:	MIRANDA		
58:00:00	6pse-A	2.4	4	58	78	7	MOLECULE:	PROTEIN BICAUDAL D HOMOLOG 2		
59:00:00	6k2j-B	2.4	6.4	65	83	11	MOLECULE:	UPF0335 PROTEIN CCNA_03428		
60:00:00	8sjj-C	2.4	18.7	56	86	13	MOLECULE:	SEPTIN-14		
61:00:00	7o97-A	2.4	3.4	53	73	6	MOLECULE:	HYPOTHETICAL PROTEIN UY81_C0065G0003 FROM C	ANDIDA	
62:00:00	8sde-A	2.4	3.8	56	89	11	MOLECULE:	TRIPARTITE MOTIF-CONTAINING PROTEIN 29		
63:00:00	5jxc-A	2.4	19.2	56	86	5	MOLECULE:	RAS/RAP GTPASE-ACTIVATING PROTEIN SYNGAP		
64:00:00	3a7p-A	2.4	3.7	59	88	8	MOLECULE:	AUTOPHAGY PROTEIN 16		
65:00:00	2olt-C	2.4	4.9	51	217	0	MOLECULE:	HYPOTHETICAL PROTEIN		
66:00:00	6rwy-g	2.4	5.2	59	86	7	MOLECULE:	INNER ROD PROTEIN		
67:00:00	7zl9-A	2.4	16.8	90	398	6	MOLECULE:	HYDROXYCARBOXYLIC ACID RECEPTOR 2,SOLUBLE C	YTOCHR	
68:00:00	6sl2-A	2.4	19.5	96	619	2	MOLECULE:	CALPONIN HOMOLOGY DOMAIN PROTEIN PUTATIVE		
69:00:00	7tds-A	2.4	11.7	98	290	6	MOLECULE:	34K2 SALIVARY PROTEIN		
70:00:00	8srm-B	2.4	34.6	109	235	6	MOLECULE:	RB1-INDUCIBLE COILED-COIL PROTEIN 1		
71:00:00	8egr-G	2.4	17.5	61	313	8	MOLECULE:	GP15, RECEPTOR-BINDING PROTEIN, TAIL FIBER		
72:00:00	8t1l-V	2.4	12.8	104	278	7	MOLECULE:	MEDIATOR OF RNA POLYMERASE II TRANSCRIPTION	SUBUN	
73:00:00	7nyx-A	2.3	98.3	194	1467	6	MOLECULE:	CHROMOSOME PARTITION PROTEIN MUKB		
74:00:00	7z8b-C	2.3	32.4	119	1224	4	MOLECULE:	CULLIN-7		
75:00:00	3k7d-A	2.3	25.9	91	497	2	MOLECULE:	GLUTAMATE-AMMONIA-LIGASE ADENYLYLTRANSFERAS	E	
76:00:00	2ewg-A	2.3	34.2	100	367	4	MOLECULE:	FARNESYL PYROPHOSPHATE SYNTHASE		
77:00:00	3u24-A	2.3	24.3	89	546	2	MOLECULE:	PUTATIVE LIPOPROTEIN		
78:00:00	8tw7-1	2.3	23.9	94	258	5	MOLECULE:	REPLICATION FACTOR C SUBUNIT 5		
79:00:00	9bax-A	2.3	22.6	101	1724	6	MOLECULE:	PROTEIN EFR3 HOMOLOG A		
80:00:00	5jjh-A	2.3	15.6	74	704	3	MOLECULE:	4-ALPHA-GLUCANOTRANSFERASE		
81:00:00	2vca-A	2.3	31.1	95	886	6	MOLECULE:	ALPHA-N-ACETYLGLUCOSAMINIDASE		
82:00:00	9j0n-W	2.3	37	123	543	7	MOLECULE:	DNA-DIRECTED RNA POLYMERASE SUBUNIT		
83:00:00	3jbz-A	2.3	25.9	100	960	4	MOLECULE:	SERINE/THREONINE-PROTEIN KINASE MTOR		
84:00:00	6aay-A	2.3	23.2	131	1199	2	MOLECULE:	BERGEYELLA ZOOHELCUM CAS13B (R1177A) MUTANT		
85:00:00	1qoy-A	2.3	32.8	120	303	9	MOLECULE:	HEMOLYSIN E		
86:00:00	8fti-A	2.3	20.7	86	737	7	MOLECULE:	INTEGRASE		
87:00:00	9dtr-S	2.3	28.2	95	604	5	MOLECULE:	U2 SNRNA		
88:00:00	6bfi-B	2.3	31.9	179	806	7	MOLECULE:	VIN1		
89:00:00	2rd9-A	2.3	35.6	96	189	5	MOLECULE:	BH0186 PROTEIN		
90:00:00	5vhx-E	2.3	32.8	89	179	6	MOLECULE:	GLUTAMATE RECEPTOR 2,GERM CELL-SPECIFIC GEN	E 1-LI	
91:00:00	4kpp-A	2.3	29.8	141	395	6	MOLECULE:	PUTATIVE UNCHARACTERIZED PROTEIN		
92:00:00	4yto-B	2.3	33.3	87	97	9	MOLECULE:	SYNAPTONEMAL COMPLEX PROTEIN 1		
93:00:00	9fwf-D	2.3	32.1	93	226	5	MOLECULE:	N-VELCROVAX HBCAG WITH SUMO-AFFIMER INSERTE	D AT N	
94:00:00	7b4m-A	2.3	30.7	137	459	6	MOLECULE:	SODIUM/HYDROGEN EXCHANGER 9B2		
95:00:00	8ki9-A	2.3	23.6	167	2238	8	MOLECULE:	L PROTEIN		
96:00:00	5ys3-A	2.3	28.4	85	188	8	MOLECULE:	SUCCINATE-ACETATE PERMEASE		
97:00:00	8jhq-A	2.3	31.6	137	446	4	MOLECULE:	SPHINGOSINE-1-PHOSPHATE TRANSPORTER SPNS2,G	LGA GL	
98:00:00	8cr1-C	2.3	29	126	266	10	MOLECULE:	ATPASE ASNA1		
99:00:00	6csm-D	2.3	8.9	95	277	5	MOLECULE:	GTACR1		
0:00	8ugc-A	2.3	15.7	88	384	3	MOLECULE:	FD15		
1:00	7zke-E	2.3	30.9	153	1623	10	MOLECULE:	DNA (36-MER)		
2:00	6vls-D	2.3	21.3	125	963	4	MOLECULE:	MALTOSE/MALTODEXTRIN-BINDING PERIPLASMIC PR	OTEIN,	
3:00	7zmg-d	2.3	4.7	70	101	4	MOLECULE:	NADH-UBIQUINONE OXIDOREDUCTASE CHAIN 1		
4:00	7cgp-A	2.3	11.5	75	147	7	MOLECULE:	MITOCHONDRIAL IMPORT INNER MEMBRANE TRANSLO	CASE S	
5:00	7zmb-3	2.3	19.7	103	130	6	MOLECULE:	NADH-UBIQUINONE OXIDOREDUCTASE CHAIN 1		
6:00	7dqk-B	2.3	7.7	87	479	2	MOLECULE:	PROTEIN DETOXIFICATION		
7:00	6iqc-A	2.3	3.8	38	103	11	MOLECULE:	DNA-BINDING PROTEIN SSO0352		
8:00	8wcs-A	2.3	14.6	98	1044	10	MOLECULE:	CAS13H1		
9:00	8v9u-A	2.3	2	38	81	3	MOLECULE:	DNA (CYTOSINE-5)-METHYLTRANSFERASE 1		
10:00	8umy-A	2.3	10.5	76	546	5	MOLECULE:	CHROMOSOME TRANSMISSION FIDELITY PROTEIN 18	HOMOL	
11:00	9ce3-A	2.3	29.6	104	1295	8	MOLECULE:	ISOFORM 4 OF TUBERIN		
12:00	6wg3-A	2.3	17.9	86	561	6	MOLECULE:	STRUCTURAL MAINTENANCE OF CHROMOSOMES PROTE	IN 1A	
13:00	9k9m-A	2.3	4.4	66	216	2	MOLECULE:	FLAGELLAR ASSOCIATED PROTEIN		
14:00	8upx-A	2.3	21.9	97	906	6	MOLECULE:	SPIKE GLYCOPROTEIN,SARS-COV-2 OMICRON SPIKE	WITH	

15:00	6k7x-C	2.3	14	92	276	3	MOLECULE:	CALCIUM UNIPORTER PROTEIN, MITOCHONDRIAL		
16:00	6hpn-A	2.3	4	73	212	3	MOLECULE:	ANTIGEN, P35		
17:00	5xj5-A	2.3	11.5	68	200	4	MOLECULE:	GLYCEROL-3-PHOSPHATE ACYLTRANSFERASE		
18:00	8esv-B	2.3	8.3	101	255	9	MOLECULE:	DISINTEGRIN AND METALLOPROTEINASE DOMAIN-CO	NTAINI	
19:00	7tjk-F	2.3	2.3	39	262	3	MOLECULE:	ORIGIN RECOGNITION COMPLEX SUBUNIT 1		
20:00	8uwq-D	2.3	12.9	64	280	13	MOLECULE:	BT1282		
21:00	5csk-A	2.3	22.7	78	1996	5	MOLECULE:	ACETYL-COA CARBOXYLASE		
22:00	7wgh-A	2.3	11.2	83	350	6	MOLECULE:	SQUALENE SYNTHASE		
23:00	9bgo-A	2.3	15.7	93	395	8	MOLECULE:	GP72		
24:00:00	8i8b-H	2.3	5.1	71	228	10	MOLECULE:	MAJOR VIRAL CAPSID PROTEIN		
25:00:00	7ot9-A	2.3	5.1	61	221	2	MOLECULE:	AI-2E MEMBER YDIK		
26:00:00	6s3l-K	2.3	11	83	193	6	MOLECULE:	FLAGELLAR BIOSYNTHETIC PROTEIN FLIP		
27:00:00	8s1x-B	2.3	3.7	44	61	2	MOLECULE:	PEPTIDE DEFORMYLASE		
28:00:00	8ixk-A	2.3	16.3	73	145	8	MOLECULE:	ATTACHMENT PROTEIN G3P		
29:00:00	6vbu-2	2.3	4.5	78	659	6	MOLECULE:	BARDET-BIEDL SYNDROME 18 PROTEIN		
30:00:00	6ukj-A	2.3	8.8	83	350	8	MOLECULE:	CHLOROQUINE RESISTANCE TRANSPORTER		
31:00:00	9b8v-E	2.3	21.3	129	845	2	MOLECULE:	CYCLIC DI-GMP-BINDING PROTEIN		
32:00:00	8yl8-A	2.3	7.4	60	211	2	MOLECULE:	DE NOVO PROTEIN		
33:00:00	8hpo-C	2.3	7.6	103	138	5	MOLECULE:	TRANSCRIPTIONAL REGULATORY PROTEIN UME1		
34:00:00	8ap7-c	2.3	7.4	57	64	9	MOLECULE:	ATP SYNTHASE SUBUNIT A		
35:00:00	9j0x-B	2.3	17.8	122	688	5	MOLECULE:	POTASSIUM CHANNEL GORK		
36:00:00	3hgf-A	2.3	13.9	74	98	9	MOLECULE:	RHOPTRY PROTEIN FRAGMENT		
37:00:00	5ctq-A	2.3	10.2	81	518	9	MOLECULE:	SQUAMOUS CELL CARCINOMA ANTIGEN RECOGNIZED	BY T-C	
38:00:00	7p2y-d	2.3	8.4	63	174	6	MOLECULE:	ATP SYNTHASE SUBUNIT ALPHA		
39:00:00	3let-B	2.3	9.9	85	310	11	MOLECULE:	ADENOSINE MONOPHOSPHATE-PROTEIN TRANSFERASE	VOPS	
40:00:00	6e7e-A	2.3	8.8	75	169	4	MOLECULE:	INCLUSION MEMBRANE PROTEIN A		
41:00:00	7nuv-A	2.3	6.1	60	76	10	MOLECULE:	AUX2PLS20		
42:00:00	7x1i-A	2.3	10.3	114	678	6	MOLECULE:	ISOFORM 1 OF SOLUTE CARRIER FAMILY 4 MEMBER		11
43:00:00	7zjr-A	2.3	27.6	105	268	6	MOLECULE:	PROTEIN PILJ		
44:00:00	3msv-A	2.3	8.9	62	352	8	MOLECULE:	NUCLEAR IMPORT ADAPTOR, NRO1		
45:00:00	5y06-A	2.3	4.3	88	235	6	MOLECULE:	MSMEG_4306		
46:00:00	9bw6-B	2.3	3	49	136	8	MOLECULE:	MAJOR VAULT PROTEIN		
47:00:00	3gp8-A	2.3	7.5	71	551	7	MOLECULE:	EXODEOXYRIBONUCLEASE V, SUBUNIT RECD, PUTAT	IVE	
48:00:00	5hc9-A	2.3	4.1	59	425	5	MOLECULE:	TRNA NUCLEOTIDYL TRANSFERASE-RELATED PROTEI	N	
49:00:00	7et3-1	2.3	7.2	76	285	4	MOLECULE:	TRIPLEX CAPSID PROTEIN 2		
50:00:00	6ait-A	2.3	3.7	57	418	9	MOLECULE:	BETA-BARREL ASSEMBLY-ENHANCING PROTEASE		
51:00:00	1szi-A	2.3	7.6	85	194	6	MOLECULE:	MANNOSE-6-PHOSPHATE RECEPTOR BINDING PROTEI	N 1	
52:00:00	7pgr-F	2.3	26.6	107	2423	6	MOLECULE:	NEUROFIBROMIN		
53:00:00	8wjo-B	2.3	43	127	283	6	MOLECULE:	STRUCTURAL MAINTENANCE OF CHROMOSOMES PROTE	IN 5	
54:00:00	2p01-A	2.3	5.1	77	323	4	MOLECULE:	ALPHA-2-MACROGLOBULIN RECEPTOR-ASSOCIATED		
55:00:00	6xy6-C	2.3	2.6	41	138	15	MOLECULE:	ANTI-APOPTOTIC MEMBRANE PROTEIN		
56:00:00	6ygh-D	2.3	16.7	87	193	7	MOLECULE:	CAPSID PROTEIN		
57:00:00	4it4-B	2.3	12.6	89	210	2	MOLECULE:	CG17282		
58:00:00	7sq3-A	2.3	7.8	51	146	4	MOLECULE:	DESIGNED TREFOIL KNOT PROTEIN, VARIANT 1		
59:00:00	6lum-D	2.3	5.5	97	146	6	MOLECULE:	SUCCINATE DEHYDROGENASE SUBUNIT C		
60:00:00	6mc9-A	2.3	21.6	62	150	6	MOLECULE:	SODIUM CHANNEL PROTEIN TYPE 4 SUBUNIT ALPHA		
61:00:00	6px3-C	2.3	8.1	60	179	3	MOLECULE:	HISTONE H3		
62:00:00	4xqk-A	2.3	14.1	105	1517	6	MOLECULE:	LLABIII		
63:00:00	3gp4-A	2.3	9.3	62	132	6	MOLECULE:	TRANSCRIPTIONAL REGULATOR, MERR FAMILY		
64:00:00	3i3l-A	2.3	8.7	84	550	2	MOLECULE:	ALKYLHALIDASE CMLS		
65:00:00	3iv5-B	2.3	3.2	52	98	2	MOLECULE:	DNA-BINDING PROTEIN FIS		
66:00:00	7w7c-B	2.3	4.7	72	159	1	MOLECULE:	PUTATIVE ABC TRANSPORT SYSTEM, ATP-BINDING	PROTEI	
67:00:00	8vwk-A	2.3	6.7	70	430	4	MOLECULE:	CYTOCHROME P450		
68:00:00	3mnl-A	2.3	6.5	60	188	10	MOLECULE:	TRANSCRIPTIONAL REGULATORY PROTEIN (PROBABL	Y TETR	
69:00:00	5vkq-A	2.3	17.1	110	1499	5	MOLECULE:	NO MECHANORECEPTOR POTENTIAL C ISOFORM L		
70:00:00	9b7y-A	2.3	12.5	87	385	3	MOLECULE:	TRANSCRIPTIONAL REPRESSOR MCE3R		
71:00:00	9ix4-A	2.3	33.6	101	1178	5	MOLECULE:	DDMD		
72:00:00	5dfz-D	2.3	7.7	77	341	8	MOLECULE:	VACUOLAR PROTEIN SORTING-ASSOCIATED PROTEIN		38
73:00:00	5jbr-A	2.3	19.6	79	149	11	MOLECULE:	UNCHARACTERIZED PROTEIN BCAV_2135		
74:00:00	5xe3-E	2.3	4.2	55	81	2	MOLECULE:	ENDORIBONUCLEASE MAZF4		
75:00:00	6tqe-A	2.3	30.2	135	634	3	MOLECULE:	ABC TRANSPORTER ATP-BINDING PROTEIN/PERMEAS	E	
76:00:00	6btm-C	2.3	6.7	74	457	7	MOLECULE:	ALTERNATIVE COMPLEX III SUBUNIT A		
77:00:00	7yoj-A	2.3	14	95	867	4	MOLECULE:	CASPI		
78:00:00	8dr5-A	2.3	26	62	646	11	MOLECULE:	REPLICATION FACTOR C SUBUNIT 1		
79:00:00	8qa2-A	2.3	5.6	95	215	3	MOLECULE:	GAP JUNCTION BETA-2 PROTEIN		
80:00:00	6wt8-A	2.3	22.5	93	356	6	MOLECULE:	STING-ASSOCIATED CDNE C-DI-GMP SYNTHASE		
81:00:00	5lm2-B	2.3	26.3	124	340	11	MOLECULE:	TYROSINE-PROTEIN PHOSPHATASE NON-RECEPTOR T	YPE 23	
82:00:00	5kqj-A	2.3	3.3	39	185	5	MOLECULE:	2''-AMINOGLYCOSIDE NUCLEOTIDYLTRANSFERASE		
83:00:00	4d0n-B	2.3	9.4	120	371	7	MOLECULE:	TRANSFORMING PROTEIN RHOA		
84:00:00	7ttt-A	2.3	21.1	92	351	3	MOLECULE:	TUBULIN BETA CHAIN		
85:00:00	8oyv-A	2.3	5.3	91	182	4	MOLECULE:	DE NOVO DESIGNED SOLUBLE CLAUDIN		
86:00:00	5zki-B	2.3	2.6	36	304	8	MOLECULE:	NUCLEASE EXOG, MITOCHONDRIAL		
87:00:00	1bvp-1	2.3	3.5	42	349	7	MOLECULE:	BLUETONGUE VIRUS COAT PROTEIN VP7		
88:00:00	3ter-B	2.3	5.6	70	121	4	MOLECULE:	MAMMALIAN STROMAL INTERACTION MOLECULE-1		
89:00:00	8kgn-A	2.3	24.1	105	1190	7	MOLECULE:	DNA TOPOISOMERASE 2		
90:00:00	8ity-W	2.3	4.4	42	111	5	MOLECULE:	SNRNA-ACTIVATING PROTEIN COMPLEX SUBUNIT 1		
91:00:00	2ipc-A	2.3	29.1	140	939	8	MOLECULE:	PREPROTEIN TRANSLOCASE SECA SUBUNIT		
92:00:00	8wag-B	2.3	7.2	72	235	7	MOLECULE:	PROTEIN CHUP1, CHLOROPLASTIC		
93:00:00	6pon-A	2.3	5	50	255	8	MOLECULE:	ADHERENCE AND VIRULENCE PROTEIN A		
94:00:00	5dyr-A	2.3	7.6	57	139	4	MOLECULE:	VIRULENCE-ASSOCIATED PROTEIN D		
95:00:00	5g49-A	2.3	9	67	97	4	MOLECULE:	NUCLEAR TRANSCRIPTION FACTOR Y SUBUNIT B-6		
96:00:00	6mcp-A	2.3	13.4	78	520	9	MOLECULE:	LEGK7		
97:00:00	6ka4-A	2.3	9.5	86	534	7	MOLECULE:	F22L4.1 PROTEIN		
98:00:00	4iuw-A	2.3	25.4	115	630	10	MOLECULE:	NEUTRAL ENDOPEPTIDASE		
99:00:00	8si6-A	2.3	14.6	124	1107	7	MOLECULE:	TRANSIENT RECEPTOR POTENTIAL CATION CHANNEL	SUBFA	
0:00	3h0g-A	2.3	10.2	96	1496	7	MOLECULE:	DNA-DIRECTED RNA POLYMERASE II SUBUNIT RPB1		
1:00	2e2e-A	2.3	14.8	62	171	10	MOLECULE:	FORMATE-DEPENDENT NITRITE REDUCTASE COMPLEX	NRFG	
2:00	2win-M	2.3	3.9	53	84	8	MOLECULE:	COMPLEMENT C3 BETA CHAIN		
3:00	3n9m-A	2.3	12.9	68	503	7	MOLECULE:	PUTATIVE UNCHARACTERIZED PROTEIN		
4:00	7mrw-C	2.3	24.7	95	706	7	MOLECULE:	CYTOADHERENCE LINKED ASEXUAL PROTEIN 3.1		
5:00	6c14-B	2.3	5.9	88	164	2	MOLECULE:	PROTOCADHERIN-15		
6:00	4ztx-A	2.3	11.4	82	768	9	MOLECULE:	COBALAMIN-INDEPENDENT METHIONINE SYNTHASE		
7:00	6nf1-A	2.3	7.4	104	550	4	MOLECULE:	PROTO-ONCOGENE VAV		
8:00	4gr6-B	2.3	7.4	57	105	4	MOLECULE:	ATRBCX2		
9:00	2qyw-A	2.3	1.9	62	96	6	MOLECULE:	VESICLE TRANSPORT THROUGH INTERACTION WITH	T-SNAR	
10:00	1ypy-A	2.3	9	62	182	3	MOLECULE:	VIRION MEMBRANE PROTEIN		
11:00	6hyd-A	2.3	20.1	108	1574	8	MOLECULE:	MIDASIN,MIDASIN,MIDASIN		
12:00	1s7o-C	2.3	8.7	68	108	4	MOLECULE:	HYPOTHETICAL UPF0122 PROTEIN		
13:00	5jw9-B	2.3	6.9	75	115	5	MOLECULE:	AF4/FMR2 FAMILY MEMBER 4		
14:00	5i1u-B	2.3	14.8	75	294	7	MOLECULE:	GERMACRADIEN-4-OL SYNTHASE		
15:00	6l82-A	2.3	21	58	98	14	MOLECULE:	SPINDLE POLE BODY COMPONENT		

16:00	4f43-A	2.3	8.9	64	320	6	MOLECULE:	PROTELOMERASE		
17:00	5yx4-A	2.3	24.1	64	232	9	MOLECULE:	CHALCONE-FLAVONONE ISOMERASE FAMILY PROTEIN		
18:00	6pwy-B	2.3	12.6	74	513	8	MOLECULE:	ZK177.8		
19:00	3vep-H	2.3	6.9	53	71	4	MOLECULE:	UNCHARACTERIZED PROTEIN RV3413C/MT3522		
20:00	6ogd-A	2.3	7.2	63	748	6	MOLECULE:	TOXIN SUBUNIT YENA1		
21:00	7arc-W	2.3	3.3	45	127	7	MOLECULE:	PSST		
22:00	8c06-A	2.3	26.4	80	925	4	MOLECULE:	E3 UBIQUITIN-PROTEIN LIGASE UBR5		
23:00	7y17-C	2.3	13.4	66	340	9	MOLECULE:	POLYNUCLEOTIDE 5'-HYDROXYL-KINASE GRC3		
24:00:00	7nnl-B	2.3	23.5	97	682	5	MOLECULE:	POTASSIUM-TRANSPORTING ATPASE POTASSIUM-BIN	DING S	
25:00:00	1nek-C	2.3	6.2	72	129	4	MOLECULE:	SUCCINATE DEHYDROGENASE FLAVOPROTEIN SUBUNI	T	
26:00:00	4zm1-B	2.3	6	97	238	5	MOLECULE:	CHAIN LENGTH DETERMINANT PROTEIN		
27:00:00	6bhc-A	2.3	5.2	52	371	10	MOLECULE:	PSEUDOPODIUM-ENRICHED ATYPICAL KINASE 1		
28:00:00	6e6y-A	2.3	8.4	61	268	3	MOLECULE:	DIECKMANN CYCLASE, NCMC		
29:00:00	8sfn-A	2.3	15.5	119	1302	8	MOLECULE:	CRISPR-ASSOCIATED ENDONUCLEASE CAS12A		
30:00:00	8afz-B	2.3	5.1	68	376	9	MOLECULE:	SORTING NEXIN-1		
31:00:00	6v8o-N	2.3	15.2	105	412	9	MOLECULE:	HIGH TEMPERATURE LETHAL PROTEIN 1		
32:00:00	7s4m-C	2.3	5.9	64	241	8	MOLECULE:	PARTICULATE METHANE MONOOXYGENASE ALPHA SUB	UNIT	
33:00:00	4h8s-C	2.3	33.7	155	382	6	MOLECULE:	DCC-INTERACTING PROTEIN 13-BETA		
34:00:00	4nad-A	2.3	9.8	91	133	11	MOLECULE:	REGULATION OF NUCLEAR PRE-MRNA DOMAIN-CONTA	INING	
35:00:00	6g7c-C	2.3	3.5	41	239	0	MOLECULE:	IMPA-RELATED DOMAIN PROTEIN		
36:00:00	8d8k-Q	2.3	4.1	50	205	4	MOLECULE:	PROBABLE S-ADENOSYL-L-METHIONINE-DEPENDENT	RNA	
37:00:00	3vou-A	2.3	10.2	100	139	3	MOLECULE:	ION TRANSPORT 2 DOMAIN PROTEIN, VOLTAGE-GAT	ED SOD	
38:00:00	4me7-F	2.3	8.1	55	79	4	MOLECULE:	MRNA INTERFERASE ENDOA		
39:00:00	5o9e-B	2.3	9.9	57	80	7	MOLECULE:	PUTATIVE U3 SMALL NUCLEOLAR RIBONUCLEOPROTE	IN	
40:00:00	3qwe-A	2.3	22.5	117	260	5	MOLECULE:	GEM-INTERACTING PROTEIN		
41:00:00	8j80-A	2.3	16.6	96	290	10	MOLECULE:	ZINC TRANSPORTER 7		
42:00:00	3cit-A	2.3	4.8	52	155	4	MOLECULE:	SENSOR HISTIDINE KINASE		
43:00:00	7zkq-C	2.3	15.3	77	416	5	MOLECULE:	NADH DEHYDROGENASE SUBUNIT 2		
44:00:00	6n7p-D	2.3	22.9	99	544	10	MOLECULE:	U1 SMALL NUCLEAR RIBONUCLEOPROTEIN 70 KDA H	OMOLOG	
45:00:00	3j9t-O	2.3	17.4	113	392	5	MOLECULE:	V-TYPE PROTON ATPASE SUBUNIT D		
46:00:00	5vxv-A	2.3	15.2	56	217	9	MOLECULE:	PEROXISOMAL MEMBRANE PROTEIN PEX15		
47:00:00	5l0w-B	2.3	25.6	64	209	8	MOLECULE:	SEC72		
48:00:00	8au0-B	2.3	4.9	38	41	5	MOLECULE:	SUN DOMAIN-CONTAINING PROTEIN 1		
49:00:00	7a48-B	2.3	3.3	37	40	5	MOLECULE:	NANOBODY 49		
50:00:00	6q6e-A	2.3	7.6	70	221	9	MOLECULE:	CONDENSIN COMPLEX SUBUNIT 2,STRUCTURAL MAIN	TENANC	
51:00:00	3c64-A	2.3	4.8	54	152	7	MOLECULE:	PFEMP1 VARIANT 2 OF STRAIN MC		
52:00:00	3pla-A	2.3	9.7	71	375	4	MOLECULE:	PRE MRNA SPLICING PROTEIN		
53:00:00	1i6z-A	2.3	6.2	60	135	10	MOLECULE:	BAG-FAMILY MOLECULAR CHAPERONE REGULATOR-1		
54:00:00	8ims-A	2.3	3.6	58	93	3	MOLECULE:	E3 UBIQUITIN-PROTEIN LIGASE TRAF7		
55:00:00	8hir-A	2.3	24.3	97	918	9	MOLECULE:	POTASSIUM CHANNEL SUBFAMILY T MEMBER 1		
56:00:00	7c7y-C	2.3	20	57	67	9	MOLECULE:	UNCHARACTERIZED PROTEIN		
57:00:00	7bji-B	2.3	3.7	56	92	11	MOLECULE:	CENTROSOMAL PROTEIN OF 135 KDA		
58:00:00	4ytd-B	2.3	3.1	55	99	4	MOLECULE:	PROTEIN BICAUDAL D HOMOLOG 1		
59:00:00	3wvz-A	2.3	5.9	47	192	0	MOLECULE:	PROTEIN HIKESHI		
60:00:00	6h9l-B	2.3	5.2	55	129	11	MOLECULE:	UNCHARACTERIZED PROTEIN		
61:00:00	6fkf-p	2.3	34.1	89	143	8	MOLECULE:	ATP SYNTHASE SUBUNIT ALPHA, CHLOROPLASTIC		
62:00:00	7xuz-A	2.3	3.7	56	120	13	MOLECULE:	HISTONE DEACETYLASE 4		
63:00:00	7xfr-B	2.3	4.1	52	69	0	MOLECULE:	ISOFORM 2 OF WD REPEAT DOMAIN PHOSPHOINOSIT	IDE-IN	
64:00:00	4nqf-B	2.3	2.8	50	151	10	MOLECULE:	NUCLEOTIDYLTRANSFERASE		
65:00:00	1izl-C	2.3	18.7	70	349	0	MOLECULE:	PHOTOSYSTEM II: SUBUNIT PSBA		
66:00:00	8vb0-H	2.3	4.3	44	61	16	MOLECULE:	MAJOR CAPSID PROTEIN (GP38)		
67:00:00	3m62-A	2.3	19.1	108	955	6	MOLECULE:	UBIQUITIN CONJUGATION FACTOR E4		
68:00:00	7c1i-A	2.3	4.3	49	115	2	MOLECULE:	HISTIDINE KINASE		
69:00:00	6uak-A	2.3	11	48	298	8	MOLECULE:	SAM DEPENDENT METHYLTRANSFERASE LAHSB		
70:00:00	7vyx-A	2.3	9.4	92	1211	4	MOLECULE:	SELENOMETHIONINE (SEMET)-LABELED CAS12C1 D9	69A MU	
71:00:00	6dql-A	2.3	3.2	41	227	2	MOLECULE:	REGULATOR OF PROTEINASE B ROPB		
72:00:00	6rd4-7	2.3	6	65	176	5	MOLECULE:	ASA-10: POLYTOMELLA F-ATP SYNTHASE ASSOCIAT	ED SUB	
73:00:00	6uuj-G	2.3	3.5	49	74	4	MOLECULE:	PE FAMILY IMMUNOMODULATOR PE5		
74:00:00	7lhf-A	2.2	38.3	166	2123	5	MOLECULE:	INOSITOL 1,4,5-TRISPHOSPHATE RECEPTOR TYPE		1
75:00:00	8qby-L	2.2	25.8	110	660	5	MOLECULE:	NADH-QUINONE OXIDOREDUCTASE SUBUNIT K		
76:00:00	6hsy-A	2.2	25.3	62	190	0	MOLECULE:	TOLUENE TOLERANCE PROTEIN TTG2D		
77:00:00	8wge-A	2.2	12.8	73	348	4	MOLECULE:	ZAC		
78:00:00	6hu9-c	2.2	13.1	89	269	4	MOLECULE:	CYTOCHROME B-C1 COMPLEX SUBUNIT 1, MITOCHON	DRIAL	
79:00:00	6rxd-A	2.2	18.3	94	509	4	MOLECULE:	HISTIDINE ACID PHOSPHATASE		
80:00:00	7ymo-A	2.2	22.4	42	206	2	MOLECULE:	RECOMBINATION PROTEIN RECO		
81:00:00	8yad-C	2.2	29.5	127	1811	6	MOLECULE:	SPATACSIN		
82:00:00	1gm5-A	2.2	13.2	91	729	8	MOLECULE:	RECG		
83:00:00	4ka7-A	2.2	19.5	137	695	8	MOLECULE:	OLIGOPEPTIDASE A		
84:00:00	2y44-A	2.2	13.4	68	184	4	MOLECULE:	GLUTAMIC ACID/ALANINE-RICH PROTEIN		
85:00:00	8ucj-c	2.2	27.3	101	269	4	MOLECULE:	SYNAPTIC VESICULAR AMINE TRANSPORTER		
86:00:00	8vlu-A	2.2	27.4	117	550	8	MOLECULE:	HEPARAN-ALPHA-GLUCOSAMINIDE N-ACETYLTRANSFE	RASE	
87:00:00	7znk-b	2.2	21.3	89	919	8	MOLECULE:	RNA		
88:00:00	7zr1-D	2.2	57.6	174	780	6	MOLECULE:	DOUBLE-STRAND BREAK REPAIR PROTEIN		
89:00:00	6ibl-A	2.2	5.9	121	397	2	MOLECULE:	THIOREDOXIN 1,BETA-1 ADRENERGIC RECEPTOR		
90:00:00	7yn2-B	2.2	29.8	94	349	5	MOLECULE:	CCBD		
91:00:00	8rjj-A	2.2	26.8	78	183	5	MOLECULE:	GENOME POLYPROTEIN		
92:00:00	5wco-A	2.2	15.6	62	196	8	MOLECULE:	NS2		
93:00:00	6fes-A	2.2	21.6	91	365	4	MOLECULE:	D12_BRIC2, A SYNTHETIC PROTEIN,D12_BRIC2, A	SYNTH	
94:00:00	1iq0-A	2.2	20.1	93	588	6	MOLECULE:	ARGINYL-TRNA SYNTHETASE		
95:00:00	6hqa-A	2.2	31.4	116	887	8	MOLECULE:	TAF2		
96:00:00	7lid-A	2.2	35	119	381	8	MOLECULE:	MHOR5		
97:00:00	3eo8-A	2.2	27.6	90	219	7	MOLECULE:	BLUB-LIKE FLAVOPROTEIN		
98:00:00	2jqq-A	2.2	14.9	65	154	6	MOLECULE:	CONSERVED OLIGOMERIC GOLGI COMPLEX SUBUNIT		2
99:00:00	1yvl-B	2.2	22.4	110	653	10	MOLECULE:	SIGNAL TRANSDUCER AND ACTIVATOR OF TRANSCRI	PTION	
0:00	3o0z-D	2.2	53.6	134	160	8	MOLECULE:	RHO-ASSOCIATED PROTEIN KINASE 1		
1:00	6j36-A	2.2	30.7	89	459	6	MOLECULE:	ENOLASE		
2:00	2j68-A	2.2	28.3	160	680	5	MOLECULE:	BACTERIAL DYNAMIN-LIKE PROTEIN		
3:00	7wmp-m	2.2	29.9	100	195	12	MOLECULE:	PORTAL PROTEIN		
4:00	7ag9-B	2.2	32.7	155	384	8	MOLECULE:	KAR9		
5:00	6s7t-H	2.2	6.6	78	112	5	MOLECULE:	DOLICHYL-DIPHOSPHOOLIGOSACCHARIDE--PROTEIN		
6:00	7qog-M	2.2	3.7	58	126	10	MOLECULE:	PORTAL PROTEIN GP20		
7:00	6vtk-A	2.2	3.7	61	446	5	MOLECULE:	ACID-SENSING ION CHANNEL 1		
8:00	3zhe-A	2.2	21.9	110	399	4	MOLECULE:	NONSENSE-MEDIATED MRNA DECAY PROTEIN		
9:00	7q21-f	2.2	14.7	80	143	3	MOLECULE:	CO-PURIFIED UNKNOWN TRANSMEMBRANE HELICES B	UILT A	
10:00	3lvg-D	2.2	15.2	64	180	9	MOLECULE:	CLATHRIN HEAVY CHAIN 1		
11:00	1x8z-A	2.2	4.3	89	151	7	MOLECULE:	INVERTASE/PECTIN METHYLESTERASE INHIBITOR F	AMILY	
12:00	2oer-B	2.2	11.8	70	189	6	MOLECULE:	PROBABLE TRANSCRIPTIONAL REGULATOR		
13:00	6y92-A	2.2	4.7	73	178	5	MOLECULE:	B-LYMPHOCYTE ANTIGEN CD20		
14:00	2uxx-B	2.2	24.1	82	134	4	MOLECULE:	LYSINE-SPECIFIC HISTONE DEMETHYLASE 1		
15:00	1yx4-A	2.2	16.4	69	112	6	MOLECULE:	26S PROTEASOME NON-ATPASE REGULATORY SUBUNI	T 4	
16:00	3k11-A	2.2	13.9	82	439	6	MOLECULE:	PUTATIVE GLYCOSYL HYDROLASE		

17:00	6jx7-A	2.2	19.9	77	1245	8	MOLECULE:	FELINE INFECTIOUS PERITONITIS VIRUS SPIKE P	ROTEIN
18:00	8w97-A	2.2	5.2	59	135	5	MOLECULE:	DE NOVO DESIGN PROTEIN	
19:00	9bq2-B	2.2	22.4	82	422	6	MOLECULE:	FLOTILLIN-2	
20:00	4boj-C	2.2	7.9	66	344	2	MOLECULE:	ALPHA-1,6-MANNANASE	
21:00	4o8w-D	2.2	7.8	43	120	7	MOLECULE:	SPORE GERMINATION PROTEIN	
22:00	3uau-A	2.2	4.2	71	344	4	MOLECULE:	SURFACE-EXPOSED LIPOPROTEIN	
23:00	4cej-A	2.2	30.4	106	1177	7	MOLECULE:	ATP-DEPENDENT HELICASE/NUCLEASE SUBUNIT A	
24:00:00	4gzu-A	2.2	20	119	436	9	MOLECULE:	FERM, RHOGEF AND PLECKSTRIN DOMAIN-CONTAINI	NG PRO
25:00:00	8cq4-A	2.2	13.5	90	393	7	MOLECULE:	BIFUNCTIONAL CYCLOHEXADIENYL DEHYDRATASE/CH	ORISMA
26:00:00	5fib-A	2.2	18.7	125	534	10	MOLECULE:	SPHINGOMYELIN PHOSPHODIESTERASE	
27:00:00	2x3b-A	2.2	7.2	69	295	3	MOLECULE:	TOXIC EXTRACELLULAR ENDOPEPTIDASE	
28:00:00	6em5-b	2.2	13.5	132	421	8	MOLECULE:	5.8S RIBOSOMAL RNA	
29:00:00	6xz6-A	2.2	8.5	87	213	9	MOLECULE:	GARP DOMAIN-CONTAINING PROTEIN	
30:00:00	7mk3-B	2.2	9.9	93	365	8	MOLECULE:	REGULATORY PROTEIN NPR1	
31:00:00	8ofi-C	2.2	10.3	91	415	10	MOLECULE:	POTASSIUM/SODIUM HYPERPOLARIZATION-ACTIVATE	D CYCL
32:00:00	8as8-E	2.2	30.9	111	498	5	MOLECULE:	JETC	
33:00:00	8eld-L	2.2	3.7	41	115	2	MOLECULE:	GP37	
34:00:00	2fb5-A	2.2	5.3	86	204	10	MOLECULE:	HYPOTHETICAL MEMBRANE SPANNING PROTEIN	
35:00:00	6s85-C	2.2	22.9	89	374	7	MOLECULE:	NUCLEASE SBCCD SUBUNIT C	
36:00:00	4igg-B	2.2	18.8	108	771	6	MOLECULE:	CATENIN ALPHA-1	
37:00:00	8jpa-A	2.2	9.1	70	139	11	MOLECULE:	DE NOVO DESIGN CAVITATED PROTEIN	
38:00:00	2yy5-B	2.2	20.8	75	346	9	MOLECULE:	TRYPTOPHANYL-TRNA SYNTHETASE	
39:00:00	8wai-A	2.2	8.8	83	277	6	MOLECULE:	RVY_06210	
40:00:00	6wq0-A	2.2	12.9	89	131	6	MOLECULE:	DNA (301-MER)	
41:00:00	6wq2-A	2.2	9.8	97	154	5	MOLECULE:	A-DNA	
42:00:00	8hpp-C	2.2	8.5	57	86	7	MOLECULE:	INTEGRATOR COMPLEX SUBUNIT 3	
43:00:00	9na9-A	2.2	5.5	74	103	5	MOLECULE:	AUGMIN SUBUNIT 1	
44:00:00	2ocy-A	2.2	31.5	81	149	12	MOLECULE:	RAB GUANINE NUCLEOTIDE EXCHANGE FACTOR SEC2	
45:00:00	9jfl-A	2.2	1.7	41	594	7	MOLECULE:	DUF262 DOMAIN-CONTAINING PROTEIN	
46:00:00	6ah0-w	2.2	22.3	129	443	5	MOLECULE:	U5SNRNA	
47:00:00	5ue0-A	2.2	8.3	76	280	4	MOLECULE:	CT622 PROTEIN	
48:00:00	7ykr-A	2.2	14.1	119	1035	5	MOLECULE:	TRANSIENT RECEPTOR POTENTIAL CATION CHANNEL	SUBFA
49:00:00	3tvr-A	2.2	2.7	51	172	2	MOLECULE:	POLYKETIDE CYCLASE	
50:00:00	4ckg-A	2.2	28.4	132	368	7	MOLECULE:	ARF-GAP WITH COILED-COIL, ANK REPEAT AND PH	DOMAI
51:00:00	7bst-C	2.2	6.8	60	992	2	MOLECULE:	TYPE I RESTRICTION ENZYME R PROTEIN	
52:00:00	6f0k-H	2.2	6.5	84	156	2	MOLECULE:	CYTOCHROME C FAMILY PROTEIN	
53:00:00	7ypx-a	2.2	3.9	58	162	14	MOLECULE:	PAM3 TAIL FIBER PROREINS	
54:00:00	9f4c-A	2.2	12.5	52	141	13	MOLECULE:	CYTOSKELETON-ASSOCIATED PROTEIN 5	
55:00:00	5c0x-K	2.2	25.4	114	350	4	MOLECULE:	EXOSOME COMPLEX COMPONENT RRP45	
56:00:00	6ulg-N	2.2	18.8	88	483	9	MOLECULE:	FOLLICULIN	
57:00:00	2v0o-A	2.2	29.3	123	273	6	MOLECULE:	FCH DOMAIN ONLY PROTEIN 2	
58:00:00	6bhp-A	2.2	4.8	89	170	6	MOLECULE:	MEMBRANE PROTEIN	
59:00:00	8fjg-A	2.2	6.7	75	104	4	MOLECULE:	H12	
60:00:00	8p23-B	2.2	15	55	715	7	MOLECULE:	ANAEROBIC RIBONUCLEOSIDE-TRIPHOSPHATE REDUC	TASE
61:00:00	8bd7-L	2.2	47.3	117	303	7	MOLECULE:	IFT88	
62:00:00	6ar7-C	2.2	4.8	102	207	7	MOLECULE:	UNCHARACTERIZED PROTEIN	
63:00:00	7but-A	2.2	3.2	51	142	10	MOLECULE:	ACINIFORM SPIDROIN	
64:00:00	3cdh-B	2.2	10.2	76	136	5	MOLECULE:	TRANSCRIPTIONAL REGULATOR, MARR FAMILY	
65:00:00	4xa6-A	2.2	20.4	121	168	3	MOLECULE:	GP7-MYH7(1777-1855)-EB1 CHIMERA PROTEIN	
66:00:00	6tpn-A	2.2	11	119	510	6	MOLECULE:	OREXIN RECEPTOR TYPE 2,GLGA GLYCOGEN SYNTHA	SE,ORE
67:00:00	8ap8-d	2.2	17.1	87	270	2	MOLECULE:	OSCP	
68:00:00	8fed-E	2.2	33	104	358	5	MOLECULE:	VIRULENCE FACTOR MCE FAMILY PROTEIN	
69:00:00	7k0i-C	2.2	9.9	51	62	8	MOLECULE:	SERINE PALMITOYLTRANSFERASE 1	
70:00:00	6h02-A	2.2	25.5	129	1334	4	MOLECULE:	MEDIATOR OF RNA POLYMERASE II TRANSCRIPTION	SUBUN
71:00:00	8ua7-B	2.2	9.1	62	81	15	MOLECULE:	HISTONE H3	
72:00:00	6k6l-B	2.2	10.1	68	263	7	MOLECULE:	PSEUDO DEUBIQUITINASE	
73:00:00	3woz-B	2.2	29	73	229	8	MOLECULE:	CLIP-ASSOCIATING PROTEIN 2	
74:00:00	8b1r-B	2.2	18.3	101	1170	9	MOLECULE:	RECBCD ENZYME SUBUNIT RECB	
75:00:00	9g40-G	2.2	13.3	118	797	7	MOLECULE:	GAMMA-TUBULIN COMPLEX COMPONENT 3	
76:00:00	7q3e-B	2.2	8.5	56	503	7	MOLECULE:	WD REPEAT-CONTAINING AND PLANAR CELL POLARI	TY EFF
77:00:00	8q4h-C	2.2	6.6	75	89	3	MOLECULE:	TETRACHLOROETHENE REDUCTIVE DEHALOGENASE	
78:00:00	5ncl-A	2.2	22.6	116	418	6	MOLECULE:	SERINE/THREONINE-PROTEIN KINASE CBK1	
79:00:00	2guz-B	2.2	3.5	45	65	7	MOLECULE:	MITOCHONDRIAL IMPORT INNER MEMBRANE TRANSLO	CASE S
80:00:00	7dhg-C	2.2	5.4	60	470	7	MOLECULE:	MITOCHONDRIAL IMPORT RECEPTOR SUBUNIT TOM70	
81:00:00	5tj4-H	2.2	6.6	79	537	3	MOLECULE:	SUGAR ABC TRANSPORTER SUBSTRATE-BINDING PRO	TEIN,G
82:00:00	6t5a-A	2.2	4.1	67	96	4	MOLECULE:	TEGUMENT PROTEIN UL51	
83:00:00	2ia9-D	2.2	3.9	42	99	2	MOLECULE:	PUTATIVE SEPTATION PROTEIN SPOVG	
84:00:00	6vz1-A	2.2	12.9	115	411	7	MOLECULE:	DIACYLGLYCEROL O-ACYLTRANSFERASE 1	
85:00:00	5guj-A	2.2	2.3	37	324	11	MOLECULE:	DNA PRIMASE	
86:00:00	7pw5-B	2.2	26.4	95	702	11	MOLECULE:	SMG1,SERINE/THREONINE-PROTEIN KINASE SMG1,S	MG1,
87:00:00	8hki-a	2.2	8.8	70	291	4	MOLECULE:	T-COMPLEX PROTEIN 1 SUBUNIT ALPHA	
88:00:00	7ztb-A	2.2	14.2	88	344	6	MOLECULE:	RELA/SPOT FAMILY PROTEIN	
89:00:00	8tn1-A	2.2	3.5	76	147	5	MOLECULE:	DE NOVO DESIGNED 4 HELIX BUNDLES	
90:00:00	1q6u-A	2.2	8	75	213	9	MOLECULE:	FKBP-TYPE PEPTIDYL-PROLYL CIS-TRANS ISOMERA	SE FKP
91:00:00	8ikg-R	2.2	5.9	64	275	3	MOLECULE:	GUANINE NUCLEOTIDE-BINDING PROTEIN G(I) SUB	UNIT A
92:00:00	7c79-L	2.2	3.3	73	131	1	MOLECULE:	RIBONUCLEASE MRP RNA SUBUNIT NME1	
93:00:00	5lc5-p	2.2	4.3	70	169	6	MOLECULE:	NADH-UBIQUINONE OXIDOREDUCTASE CHAIN 3	
94:00:00	5oxf-C	2.2	15.8	115	601	6	MOLECULE:	GTP-BINDING PROTEIN	
95:00:00	8r70-A	2.2	23	96	618	5	MOLECULE:	HEPARINASE	
96:00:00	6nyi-A	2.2	3.2	59	97	5	MOLECULE:	DESIGN CONSTRUCT XXA	
97:00:00	9dec-A	2.2	2.1	38	82	8	MOLECULE:	D9-THREADED	
98:00:00	1miu-A	2.2	16.1	84	671	8	MOLECULE:	DELETED IN SPLIT HAND/SPLIT FOOT PROTEIN 1	
99:00:00	1yqg-A	2.2	7.1	70	263	7	MOLECULE:	PYRROLINE-5-CARBOXYLATE REDUCTASE	
0:00	8q7n-X	2.2	5.7	54	81	15	MOLECULE:	U5 SNRNA	
1:00	8ssn-B	2.2	9.3	46	386	7	MOLECULE:	TYROSINE-PROTEIN KINASE ABL1	
2:00	3dpn-A	2.2	17	81	537	9	MOLECULE:	PROTEIN CT_858	
3:00	7wvt-A	2.2	2.8	71	364	8	MOLECULE:	PHOSPHATIDYLINOSITOL TRANSFER PROTEIN CSR1	
4:00	7pkq-P	2.2	9.7	75	337	8	MOLECULE:	MS35	
5:00	7dwb-A	2.2	13.6	124	421	5	MOLECULE:	PANNEXIN-1	
6:00	8p62-C	2.2	5.6	49	175	8	MOLECULE:	DNA REPLICATION LICENSING FACTOR MCM2	
7:00	8a9b-B	2.2	21.3	94	809	2	MOLECULE:	LIPID BINDING PROTEIN P116 (MPN213)	
8:00	5j1s-B	2.2	6	60	235	5	MOLECULE:	TORSIN-1A	
9:00	7ag4-C	2.2	10.5	90	425	8	MOLECULE:	SULFOQUINOVOSE ISOMERASE	
10:00	3ce2-A	2.2	23.2	103	595	4	MOLECULE:	PUTATIVE PEPTIDASE	
11:00	8ccr-A	2.2	3.7	63	105	5	MOLECULE:	4D2 (MUTANT T19D)	
12:00	8vxq-J	2.2	24.3	102	307	7	MOLECULE:	GP72	
13:00	7bc4-B	2.2	20.6	122	2054	6	MOLECULE:	FATTY ACID SYNTHASE SUBUNIT ALPHA	
14:00	8wzb-E	2.2	11.3	66	201	5	MOLECULE:	DPY30 DOMAIN CONTAINING 2	
15:00	6kn7-T	2.2	47.1	119	138	6	MOLECULE:	ACTIN, ALPHA SKELETAL MUSCLE	
16:00	8bgo-C	2.2	5.5	57	271	9	MOLECULE:	DIACETYLCHITOBIOSE DEACETYLASE	
17:00	3tdw-A	2.2	15.5	85	302	5	MOLECULE:	GENTAMICIN RESISTANCE PROTEIN	

18:00	6gsi-J	2.2	15.5	61	89	5	MOLECULE:	CAPSID PROTEIN	
19:00	7vw0-B	2.2	5.8	82	94	5	MOLECULE:	DUF305 DOMAIN-CONTAINING PROTEIN	
20:00	4epz-A	2.2	4.8	50	154	2	MOLECULE:	TRANSCRIPTION ANTI-TERMINATOR ANTAGONIST UP	XZ
21:00	1gqe-A	2.2	12.3	102	362	1	MOLECULE:	RELEASE FACTOR 2	
22:00	3hug-A	2.2	8.5	51	80	12	MOLECULE:	RNA POLYMERASE SIGMA FACTOR	
23:00	8x2q-A	2.2	3	79	109	11	MOLECULE:	ADENOMATOUS POLYPOSIS COLI PROTEIN	
24:00:00	8b9z-h	2.2	10.2	99	145	7	MOLECULE:	NADH-UBIQUINONE OXIDOREDUCTASE CHAIN 3	
25:00:00	6sc6-A	2.2	12.3	75	365	7	MOLECULE:	E3 UBIQUITIN-PROTEIN LIGASE RNF31	
26:00:00	7emf-D	2.2	9.8	88	158	6	MOLECULE:	MEDIATOR OF RNA POLYMERASE II TRANSCRIPTION	SUBUN
27:00:00	1zbd-B	2.2	5.1	80	124	5	MOLECULE:	RABPHILIN-3A	
28:00:00	8f2r-E	2.2	2	41	205	10	MOLECULE:	COMM DOMAIN-CONTAINING PROTEIN 1	
29:00:00	5xwp-A	2.2	23.2	115	1125	6	MOLECULE:	UNCHARACTERIZED PROTEIN	
30:00:00	7qd4-A	2.2	9.9	83	908	7	MOLECULE:	TRANSPOSASE FOR TRANSPOSON TN4430	
31:00:00	6tt7-M	2.2	26.5	70	156	11	MOLECULE:	ATP SYNTHASE SUBUNIT ALPHA	
32:00:00	8wzb-D	2.2	4.4	41	46	5	MOLECULE:	DPY30 DOMAIN CONTAINING 2	
33:00:00	7eqc-G	2.2	48.2	75	107	11	MOLECULE:	CYTOKINESIS DEFECT	
34:00:00	6rx3-C	2.2	3.4	48	99	8	MOLECULE:	SYNCYTIN-2	
35:00:00	5w0p-C	2.2	21.7	75	850	5	MOLECULE:	ENDOLYSIN,RHODOPSIN,S-ARRESTIN	
36:00:00	4bjm-A	2.2	5.6	61	226	7	MOLECULE:	AVRM	
37:00:00	2fbq-A	2.2	4.6	45	213	7	MOLECULE:	PROBABLE TRANSCRIPTIONAL REGULATOR	
38:00:00	8r5i-B	2.2	3.2	40	43	10	MOLECULE:	CORE PROTEIN A10	
39:00:00	8arf-B	2.2	3.8	59	97	8	MOLECULE:	PROTEIN SPINDLY	
40:00:00	8i03-G	2.2	21.2	88	166	7	MOLECULE:	PAIRED AMPHIPATHIC HELIX PROTEIN PST1	
41:00:00	6z26-B	2.2	21	61	103	7	MOLECULE:	SPINDLE ASSEMBLY ABNORMAL PROTEIN 6 HOMOLOG	
42:00:00	6r17-B	2.2	30.6	65	94	9	MOLECULE:	SYNAPTONEMAL COMPLEX CENTRAL ELEMENT PROTEI	N 2
43:00:00	4wij-B	2.2	19.4	64	301	6	MOLECULE:	SPLICING FACTOR, PROLINE- AND GLUTAMINE-RIC	H
44:00:00	5oag-A	2.2	3.6	55	98	13	MOLECULE:	RAB-3A-INTERACTING PROTEIN	
45:00:00	6zpm-A	2.2	3.8	56	103	4	MOLECULE:	TRYPANOSOMA CRUZI KKT4 117-218	
46:00:00	8fby-C	2.2	3.6	56	93	9	MOLECULE:	PLASMALEMMA VESICLE-ASSOCIATED PROTEIN	
47:00:00	4bbp-A	2.2	10.3	85	266	9	MOLECULE:	ZINC ABC TRANSPORTER, PERIPLASMIC ZINC-BIND	ING PR
48:00:00	7s0r-A	2.2	2.2	44	85	11	MOLECULE:	C PROTEIN BETA ANTIGEN	
49:00:00	9cjl-A	2.2	20.6	54	82	4	MOLECULE:	TRANSMEMBRANE EMP24 DOMAIN-CONTAINING PROTE	IN 9
50:00:00	6jxc-A	2.2	7.1	75	2345	9	MOLECULE:	SERINE/THREONINE-PROTEIN KINASE TEL1	
51:00:00	5x3q-A	2.2	9.9	97	313	8	MOLECULE:	ENVELOPE GLYCOPROTEIN	
52:00:00	1zw0-D	2.2	2.8	45	66	11	MOLECULE:	TYPE III SECRETION PROTEIN	
53:00:00	7wb4-g	2.2	8.3	57	672	11	MOLECULE:	OUTER NUP133	
54:00:00	7yim-A	2.2	3.1	39	592	8	MOLECULE:	ALPHA-FETOPROTEIN	
55:00:00	5fno-A	2.2	3	50	568	2	MOLECULE:	MANGANESE LIPOXYGENASE	
56:00:00	1zs4-A	2.2	8.7	61	82	5	MOLECULE:	DNA - 27MER	
57:00:00	7pgs-N	2.2	10.9	67	1787	4	MOLECULE:	NEUROFIBROMIN	
58:00:00	6yhs-U	2.2	4.5	46	61	4	MOLECULE:	23S RIBOSOMAL RNA	
59:00:00	1g5z-A	2.2	3.8	61	164	11	MOLECULE:	OUTER SURFACE PROTEIN C	
60:00:00	1z0x-A	2.2	3.5	50	216	2	MOLECULE:	TRANSCRIPTIONAL REGULATOR, TETR FAMILY	
61:00:00	7nnt-C	2.2	2.8	52	169	4	MOLECULE:	ENERGY-COUPLING FACTOR TRANSPORTER ATP-BIND	ING PR
62:00:00	2crb-A	2.2	3.1	56	97	9	MOLECULE:	NUCLEAR RECEPTOR BINDING FACTOR 2	
63:00:00	5tte-B	2.2	11.2	103	442	5	MOLECULE:	E3 UBIQUITIN-PROTEIN LIGASE ARIH1	
64:00:00	6h86-A	2.1	22.5	60	76	10	MOLECULE:	SYNAPTONEMAL COMPLEX CENTRAL ELEMENT PROTEI	N 3
65:00:00	6oht-A	2.1	24	63	206	5	MOLECULE:	3-BETA-HYDROXYSTEROID-DELTA(8),DELTA(7)-ISO	MERASE
66:00:00	9b3i-A	2.1	34.4	122	944	6	MOLECULE:	KAP114 ISOFORM 1	
67:00:00	8gji-A	2.1	28.8	82	167	10	MOLECULE:	GCG BINDER	
68:00:00	1z7q-c	2.1	18.4	105	230	4	MOLECULE:	PROTEASOME COMPONENT C7-ALPHA	
69:00:00	8dh9-D	2.1	30	78	146	8	MOLECULE:	LEPTIN RECEPTOR	
70:00:00	8d8j-O	2.1	37	78	210	6	MOLECULE:	PROBABLE S-ADENOSYL-L-METHIONINE-DEPENDENT	RNA
71:00:00	4acl-A	2.1	25.8	58	155	0	MOLECULE:	TSSL	
72:00:00	6yj4-g	2.1	12.6	74	198	7	MOLECULE:	NADH-UBIQUINONE OXIDOREDUCTASE CHAIN 3	
73:00:00	8jme-A	2.1	25.6	108	407	2	MOLECULE:	GUSTATORY RECEPTOR FOR SUGAR TASTE 64A	
74:00:00	7x3k-A	2.1	11.5	91	262	12	MOLECULE:	ZUOTIN	
75:00:00	4ll7-A	2.1	34.9	85	94	8	MOLECULE:	SWI5-DEPENDENT HO EXPRESSION PROTEIN 3	
76:00:00	3hnw-A	2.1	38.6	93	128	11	MOLECULE:	UNCHARACTERIZED PROTEIN	
77:00:00	7t3n-A	2.1	28.1	113	452	9	MOLECULE:	VESICULAR GLUTAMATE TRANSPORTER 2	
78:00:00	8upk-A	2.1	40.7	100	739	7	MOLECULE:	BACTERIOPHYTOCHROME (LIGHT-REGULATED SIGNAL	TRANS
79:00:00	7met-B	2.1	24.2	113	455	8	MOLECULE:	ATP-DEPENDENT LIPID A-CORE FLIPPASE	
80:00:00	1i5p-A	2.1	24.6	74	633	5	MOLECULE:	PESTICIDIAL CRYSTAL PROTEIN CRY2AA	
81:00:00	8p1u-A	2.1	24.3	87	370	2	MOLECULE:	CELL DIVISION PROTEIN FTSL	
82:00:00	5xis-D	2.1	33.3	78	82	9	MOLECULE:	E3 UBIQUITIN-PROTEIN LIGASE RNF168	
83:00:00	8ay2-A	2.1	36.7	124	400	7	MOLECULE:	EXOCYST COMPLEX COMPONENT 4	
84:00:00	8b17-A	2.1	32.9	125	451	10	MOLECULE:	DIPEPTIDE AND TRIPEPTIDE PERMEASE B	
85:00:00	1ng9-A	2.1	34.6	107	794	8	MOLECULE:	5'-D(*AP*GP*CP*TP*GP*CP*CP*AP*GP*GP*CP*AP*C	P*CP*A
86:00:00	3caz-B	2.1	22.4	138	210	9	MOLECULE:	BAR PROTEIN	
87:00:00	2f22-B	2.1	30.7	74	143	9	MOLECULE:	BH3987	
88:00:00	8i4t-R	2.1	48	108	501	4	MOLECULE:	ENVELOPMENT POLYPROTEIN	
89:00:00	2gnx-A	2.1	14.6	81	288	2	MOLECULE:	HYPOTHETICAL PROTEIN	
90:00:00	7wkk-I	2.1	18.8	71	171	4	MOLECULE:	MGC83295 PROTEIN	
91:00:00	8q85-G	2.1	61.5	113	178	6	MOLECULE:	KINETOCHORE PROTEIN NDC80	
92:00:00	2qih-B	2.1	43.1	119	137	5	MOLECULE:	PROTEIN USPA1	
93:00:00	8xca-A	2.1	22.3	83	668	7	MOLECULE:	CASJ19	
94:00:00	7jm7-B	2.1	27	82	218	2	MOLECULE:	OSTEOPETROSIS-ASSOCIATED TRANSMEMBRANE PROT	EIN 1
95:00:00	7d3u-F	2.1	4	67	85	10	MOLECULE:	MONOVALENT NA+/H+ ANTIPORTER SUBUNIT D	
96:00:00	9dtr-H	2.1	18.6	104	460	8	MOLECULE:	U2 SNRNA	
97:00:00	8c0v-A	2.1	5.9	58	823	5	MOLECULE:	PEROXISOMAL ATPASE PEX1	
98:00:00	9h2d-A	2.1	9.5	62	270	8	MOLECULE:	INTRAFLAGELLAR TRANSPORT PROTEIN 172 HOMOLO	G
99:00:00	3dby-C	2.1	6.9	84	267	10	MOLECULE:	UNCHARACTERIZED PROTEIN	
0:00	8jc5-C	2.1	5.2	53	196	4	MOLECULE:	ADP-RIBOSYLATION FACTOR-LIKE PROTEIN 8B	
1:00	8gel-A	2.1	20.1	94	498	6	MOLECULE:	SC4	
2:00	8t8k-A	2.1	9	69	183	10	MOLECULE:	DUF507 FAMILY PROTEIN	
3:00	7s9y-A	2.1	12.9	107	901	8	MOLECULE:	CYTOCHROME C BIOGENESIS PROTEIN	
4:00	4f52-E	2.1	8.3	72	523	11	MOLECULE:	CULLIN-1	
5:00	6u9f-A	2.1	19.4	92	717	9	MOLECULE:	PDPA	
6:00	8gmh-A	2.1	15.2	111	223	6	MOLECULE:	LXG DOMAIN-CONTAINING PROTEIN	
7:00	2nty-B	2.1	13.2	95	337	6	MOLECULE:	EMB|CAB41934.1	
8:00	7oci-C	2.1	4.8	74	119	5	MOLECULE:	DOLICHYL-DIPHOSPHOOLIGOSACCHARIDE--PROTEIN	
9:00	2efl-A	2.1	28.2	131	281	6	MOLECULE:	FORMIN-BINDING PROTEIN 1	
10:00	2eul-A	2.1	6.8	66	156	8	MOLECULE:	ANTI-CLEAVAGE ANTI-GREA TRANSCRIPTION FACTO	R GFH1
11:00	8p0u-A	2.1	17.7	114	621	3	MOLECULE:	POLYMERASE ACIDIC PROTEIN	
12:00	2gyq-A	2.1	7.4	81	162	6	MOLECULE:	YCFI, PUTATIVE STRUCTURAL PROTEIN	
13:00	5y3t-A	2.1	13.7	42	135	12	MOLECULE:	RANBP-TYPE AND C3HC4-TYPE ZINC FINGER-CONTA	INING
14:00	7ktr-D	2.1	9.9	83	219	8	MOLECULE:	TRANSFORMATION/TRANSCRIPTION DOMAIN-ASSOCIA	TED PR
15:00	4hwi-B	2.1	2.8	70	177	11	MOLECULE:	HEAT SHOCK COGNATE 71 KDA PROTEIN	
16:00	6c94-A	2.1	20.4	98	482	6	MOLECULE:	CYTOCHROME P450 4B1	
17:00	6qrj-A	2.1	21.8	87	463	5	MOLECULE:	HYBRID KINASE	
18:00	3b77-A	2.1	7.1	86	188	6	MOLECULE:	UNCHARACTERIZED PROTEIN	

19:00	8pnj-A	2.1	5.5	68	390	6	MOLECULE:	CHORISMATE MUTASE		
20:00	3bru-A	2.1	10.3	77	191	5	MOLECULE:	REGULATORY PROTEIN, TETR FAMILY		
21:00	6m3i-A	2.1	15.7	68	316	9	MOLECULE:	HISTONE PARYLATION FACTOR 1		
22:00	6y37-C	2.1	3.4	51	118	6	MOLECULE:	HAPB		
23:00	2yfb-B	2.1	12.4	110	238	10	MOLECULE:	METHYL-ACCEPTING CHEMOTAXIS TRANSDUCER		
24:00:00	6xtv-B	2.1	18.6	82	291	9	MOLECULE:	LYSINE EXPORT TRANSCRIPTIONAL REGULATORY PR	OTEIN	
25:00:00	4zrl-A	2.1	2.7	41	313	5	MOLECULE:	POLY(A) RNA POLYMERASE GLD-2		
26:00:00	4drf-A	2.1	7.2	107	408	7	MOLECULE:	METALLOPHOSPHOESTERASE		
27:00:00	7pkn-O	2.1	8.8	69	210	1	MOLECULE:	CENTROMERE PROTEIN H		
28:00:00	4cti-A	2.1	5.9	96	207	6	MOLECULE:	OSMOLARITY SENSOR PROTEIN ENVZ, AF1503		
29:00:00	6rmn-B	2.1	8.6	68	222	4	MOLECULE:	DNA MISMATCH REPAIR PROTEIN MLH1		
30:00:00	7kak-E	2.1	9.7	77	172	3	MOLECULE:	PROTEIN TRANSPORT CHANNEL SEC61 COMPLEX, AL	PHA SU	
31:00:00	8aw5-A	2.1	7.5	97	266	5	MOLECULE:	HEME O OXYGENASE		
32:00:00	8rt9-A	2.1	33.1	93	207	10	MOLECULE:	TRWJ PROTEIN		
33:00:00	8z4q-A	2.1	8.5	86	463	5	MOLECULE:	HYDROQUINONE DIOXYGENASE PAD		
34:00:00	7w72-U	2.1	10	88	420	6	MOLECULE:	PHOSPHATIDYLINOSITOL GLYCAN ANCHOR BIOSYNTH	ESIS C	
35:00:00	7tbq-A	2.1	6.1	88	375	7	MOLECULE:	D7 LOV2-DARPIN FUSION		
36:00:00	2vwb-A	2.1	2.6	43	519	5	MOLECULE:	PUTATIVE O-SIALOGLYCOPROTEIN ENDOPEPTIDASE		
37:00:00	6wi1-A	2.1	6.1	84	806	2	MOLECULE:	IONOTROPIC GLUTAMATE RECEPTOR , NMDA RECEPT	OR GLU	
38:00:00	5y69-A	2.1	3.1	40	147	8	MOLECULE:	CHAIN A		
39:00:00	9g8u-B	2.1	15.4	65	285	8	MOLECULE:	LACTONIZING LIPASE		
40:00:00	5da0-A	2.1	15.6	80	467	1	MOLECULE:	SULPHATE TRANSPORTER		
41:00:00	7zmb-9	2.1	7.7	74	103	3	MOLECULE:	NADH-UBIQUINONE OXIDOREDUCTASE CHAIN 1		
42:00:00	6ch3-B	2.1	7.4	72	162	4	MOLECULE:	FLAGELLAR BIOSYNTHESIS PROTEIN FLHA		
43:00:00	6grk-A	2.1	7.5	106	354	6	MOLECULE:	AHLB		
44:00:00	7xuh-A	2.1	24.8	114	707	5	MOLECULE:	CHLORIDE ANION EXCHANGER		
45:00:00	8t1l-Y	2.1	16.7	93	154	8	MOLECULE:	MEDIATOR OF RNA POLYMERASE II TRANSCRIPTION	SUBUN	
46:00:00	8ear-A	2.1	37	116	2389	3	MOLECULE:	INOSITOL 1,4,5-TRISPHOSPHATE RECEPTOR TYPE		1
47:00:00	6krd-D	2.1	10.5	71	429	3	MOLECULE:	T-COMPLEX PROTEIN 1 SUBUNIT ALPHA		
48:00:00	2gtv-X	2.1	3	46	104	11	MOLECULE:	CHORISMATE MUTASE		
49:00:00	6nr8-1	2.1	2.9	70	107	10	MOLECULE:	PREFOLDIN SUBUNIT 1		
50:00:00	5t58-A	2.1	33.4	122	233	7	MOLECULE:	KLLA0F02343P		
51:00:00	5lj3-G	2.1	3.2	58	97	10	MOLECULE:	U5 SNRNA (SMALL NUCLEAR RNA)		
52:00:00	5bz1-A	2.1	13.6	71	371	6	MOLECULE:	SUPPRESSOR PROTEIN MPT5		
53:00:00	8ppr-M	2.1	36.1	114	205	4	MOLECULE:	KINETOCHORE-ASSOCIATED PROTEIN DSN1 HOMOLOG		
54:00:00	1yf2-A	2.1	4.3	88	425	6	MOLECULE:	TYPE I RESTRICTION-MODIFICATION ENZYME, S S	UBUNIT	
55:00:00	3eff-K	2.1	11.3	96	139	2	MOLECULE:	FAB		
56:00:00	5eqw-B	2.1	4.3	69	123	6	MOLECULE:	PUTATIVE MAJOR COAT PROTEIN		
57:00:00	3f3s-A	2.1	6.2	52	312	4	MOLECULE:	LAMBDA-CRYSTALLIN HOMOLOG		
58:00:00	5muu-A	2.1	19.2	102	766	5	MOLECULE:	MAJOR INNER PROTEIN P1		
59:00:00	6o3v-B	2.1	15.2	74	831	7	MOLECULE:	PROTEIN VP3		
60:00:00	6tv6-A	2.1	2.7	44	362	9	MOLECULE:	PROTEIN-ARGININE KINASE		
61:00:00	8khl-A	2.1	10.8	62	275	16	MOLECULE:	PROBABLE ACYL-COA LYASE BETA CHAIN		
62:00:00	8g8i-A	2.1	15.1	80	465	6	MOLECULE:	C3HR3_9R_SHIFT4		
63:00:00	8ixk-E	2.1	8.2	81	109	5	MOLECULE:	ATTACHMENT PROTEIN G3P		
64:00:00	5uts-A	2.1	13.6	82	447	6	MOLECULE:	C-S LYASE EGT2		
65:00:00	7xym-A	2.1	22.3	104	488	4	MOLECULE:	ENVELOPE GLYCOPROTEIN		
66:00:00	2mn2-A	2.1	3.5	71	122	4	MOLECULE:	YMOB		
67:00:00	7way-A	2.1	30.9	136	960	7	MOLECULE:	DNA (33-MER)		
68:00:00	7faw-A	2.1	2.5	44	84	0	MOLECULE:	TRANSCRIPTION ELONGATION FACTOR S-II		
69:00:00	2xeq-C	2.1	15.5	79	241	6	MOLECULE:	PAT1 HOMOLOG 1,		
70:00:00	8fef-D	2.1	17.8	69	385	12	MOLECULE:	VIRULENCE FACTOR MCE FAMILY PROTEIN		
71:00:00	7vpt-A	2.1	23.9	90	469	10	MOLECULE:	C. GLABRATA IMPORTIN ALPHA ARM DOMAIN - UPC	2 NLS	
72:00:00	5yo8-A	2.1	17.6	77	349	9	MOLECULE:	TETRAPRENYL-BETA-CURCUMENE SYNTHASE		
73:00:00	7vcf-C	2.1	12.7	67	217	3	MOLECULE:	TIC214		
74:00:00	7wb4-E	2.1	23.8	132	1363	4	MOLECULE:	OUTER NUP133		
75:00:00	1sms-A	2.1	20.6	71	314	3	MOLECULE:	RIBONUCLEOSIDE-DIPHOSPHATE REDUCTASE SMALL	CHAIN	
76:00:00	4dnh-A	2.1	3	35	386	0	MOLECULE:	UNCHARACTERIZED PROTEIN		
77:00:00	4hts-A	2.1	14.4	66	228	5	MOLECULE:	SEC-INDEPENDENT PROTEIN TRANSLOCASE PROTEIN	TATC	
78:00:00	2o6i-B	2.1	11.4	76	430	7	MOLECULE:	HD DOMAIN PROTEIN		
79:00:00	8ap7-a	2.1	8.3	97	231	4	MOLECULE:	ATP SYNTHASE SUBUNIT A		
80:00:00	7tuk-A	2.1	17	80	142	6	MOLECULE:	SAG PROTEIN		
81:00:00	5wrr-B	2.1	7.2	85	438	13	MOLECULE:	PSEUDOKINASE FAM20A		
82:00:00	7u1t-A	2.1	3	40	176	18	MOLECULE:	EPSTEIN-BARR NUCLEAR ANTIGEN 1		
83:00:00	4u8u-G	2.1	3	42	151	2	MOLECULE:	GLOBIN A CHAIN		
84:00:00	6w4x-C	2.1	17.7	112	375	4	MOLECULE:	RIBONUCLEOSIDE-DIPHOSPHATE REDUCTASE 1 SUBU	NIT AL	
85:00:00	7zw1-A	2.1	8.6	102	317	4	MOLECULE:	PERIPHERIN-2		
86:00:00	5cow-A	2.1	5.5	70	234	1	MOLECULE:	PUTATIVE UNCHARACTERIZED PROTEIN		
87:00:00	8jbd-A	2.1	9.8	56	316	7	MOLECULE:	ADENYLOSUCCINATE LYASE		
88:00:00	2oqm-A	2.1	4.4	50	180	8	MOLECULE:	HYPOTHETICAL PROTEIN		
89:00:00	6zyx-Y	2.1	32.4	111	1067	11	MOLECULE:	DYNEIN HEAVY CHAIN, OUTER ARM PROTEIN		
90:00:00	9bi4-D	2.1	8.7	91	459	9	MOLECULE:	DNA REPAIR PROTEIN RAD50		
91:00:00	7vmb-A	2.1	1.5	38	359	3	MOLECULE:	IQ MOTIF AND SEC7 DOMAIN-CONTAINING PROTEIN		1
92:00:00	5njx-A	2.1	2.5	52	413	8	MOLECULE:	PEPTIDYL-PROLYL CIS-TRANS ISOMERASE FKBP5		
93:00:00	6mag-A	2.1	14.2	78	282	9	MOLECULE:	BBVCI ENDONUCLEASE SUBUNIT 2		
94:00:00	7xqk-B	2.1	8.8	46	276	4	MOLECULE:	GLUTATHIONE S-TRANSFERASE CLASS-MU 26 KDA I	SOZYME	
95:00:00	3gvd-A	2.1	8.1	64	273	3	MOLECULE:	SERINE ACETYLTRANSFERASE		
96:00:00	7rps-A	2.1	9	88	154	5	MOLECULE:	FIBRONECTIN-BINDING LIPOPROTEIN FBPB		
97:00:00	2qco-A	2.1	4.2	44	202	7	MOLECULE:	CMER		
98:00:00	4l6v-6	2.1	7.5	56	125	4	MOLECULE:	PHOTOSYSTEM I P700 CHLOROPHYLL A APOPROTEIN	A1	
99:00:00	8qa9-B	2.1	3.2	36	209	17	MOLECULE:	TRANSCRIPTIONAL REPRESSOR PROTEIN KORB		
0:00	7qen-D	2.1	16.6	124	1050	10	MOLECULE:	DNA (35-MER)		
1:00	5dwd-B	2.1	9.8	68	223	4	MOLECULE:	ESTERASE		
2:00	4b6i-C	2.1	3.5	43	102	5	MOLECULE:	SMA2266		
3:00	8ds9-A	2.1	8	77	197	6	MOLECULE:	VOLUME-REGULATED ANION CHANNEL SUBUNIT LRRC	8A,SOL	
4:00	8qat-C	2.1	5	51	606	14	MOLECULE:	PROTEIN HOOK HOMOLOG 3		
5:00	6mzc-I	2.1	23.8	105	338	9	MOLECULE:	TRANSCRIPTION INITIATION FACTOR TFIID SUBUN	IT 2	
6:00	6wtw-A	2.1	8.3	76	489	12	MOLECULE:	DASS FAMILY SODIUM-COUPLED ANION SYMPORTER		
7:00	3lxu-X	2.1	9.4	103	1217	13	MOLECULE:	TRIPEPTIDYL-PEPTIDASE 2		
8:00	6fze-A	2.1	3.1	70	209	3	MOLECULE:	PUTATIVE SURFACE PROTEIN		
9:00	4n2q-A	2.1	22	70	198	1	MOLECULE:	THA8 RNA BINDING PROTEIN		
10:00	7etr-A	2.1	7.5	55	88	2	MOLECULE:	TRANSCRIPTIONAL REGULATOR COPG FAMILY		
11:00	2yc2-C	2.1	4.1	43	149	7	MOLECULE:	INTRAFLAGELLAR TRANSPORT PROTEIN 25		
12:00	7zcv-B	2.1	11.9	81	285	6	MOLECULE:	TRANSCRIPTIONAL REGULATOR		
13:00	7rcy-A	2.1	26.2	75	829	13	MOLECULE:	PENICILLIN-BINDING PROTEIN		
14:00	4dci-B	2.1	8.8	87	148	7	MOLECULE:	UNCHARACTERIZED PROTEIN		
15:00	6m31-A	2.1	6.9	73	280	3	MOLECULE:	DIGERANYLGERANYLGLYCERYL PHOSPHATE SYNTHASE		
16:00	1uus-A	2.1	17	100	465	5	MOLECULE:	STAT PROTEIN		
17:00	8tie-a	2.1	17.2	112	1012	6	MOLECULE:	NUCLEOPORIN NUP120		
18:00	7ui4-A	2.1	8.5	77	413	5	MOLECULE:	DNA-GUANINE TRANSGLYCOSYLASE		
19:00	3iee-A	2.1	9.3	93	256	8	MOLECULE:	PUTATIVE EXPORTED PROTEIN		

20:00	7pkq-O	2.1	3.7	68	236	6	MOLECULE:	MS35	
21:00	9dof-D	2.1	9	51	166	16	MOLECULE:	GLYCOPROTEIN E	
22:00	6xdc-A	2.1	9.9	83	193	5	MOLECULE:	PROTEIN 3A	
23:00	5n9j-U	2.1	17.4	120	198	10	MOLECULE:	MEDIATOR OF RNA POLYMERASE II TRANSCRIPTION	SUBUN
24:00:00	6qd6-G	2.1	36.5	120	469	8	MOLECULE:	MB-CHOPQ-NB207,OUTER MEMBRANE PROTEIN,MB-CH	OPQ-NB
25:00:00	5v2c-b	2.1	16.2	86	506	8	MOLECULE:	PHOTOSYSTEM II PROTEIN D1	
26:00:00	4m1p-A	2.1	3.9	67	96	6	MOLECULE:	COPPER-SENSITIVE OPERON REPRESSOR (CSOR)	
27:00:00	5ouz-A	2.1	3.6	57	178	9	MOLECULE:	FERRITIN	
28:00:00	6tmi-A	2.1	7.8	60	109	7	MOLECULE:	ATP SYNTHASE SUBUNIT ALPHA	
29:00:00	8tl7-A	2.1	16.3	98	694	9	MOLECULE:	COMPUTATIONALLY DESIGNED PROTEIN	
30:00:00	6yjj-A	2.1	9.5	68	320	9	MOLECULE:	CYRI-B (FAM49B)	
31:00:00	5i4q-B	2.1	3.8	44	107	5	MOLECULE:	CONTACT-DEPENDENT INHIBITOR A	
32:00:00	7ogt-A	2.1	30.8	111	688	5	MOLECULE:	STRUCTURAL MAINTENANCE OF CHROMOSOMES PROTE	IN 1
33:00:00	3auy-A	2.1	10.5	94	366	4	MOLECULE:	DNA DOUBLE-STRAND BREAK REPAIR RAD50 ATPASE	
34:00:00	4r8g-E	2.1	34.8	94	324	6	MOLECULE:	UNCONVENTIONAL MYOSIN-IC	
35:00:00	4fgv-A	2.1	22	82	1067	5	MOLECULE:	CHROMOSOME REGION MAINTENANCE 1 (CRM1) OR E	XPORTI
36:00:00	5aww-G	2.1	8	53	75	8	MOLECULE:	PROTEIN TRANSLOCASE SUBUNIT SECY	
37:00:00	2no2-A	2.1	13.1	60	102	7	MOLECULE:	HUNTINGTIN-INTERACTING PROTEIN 1	
38:00:00	8q85-F	2.1	19.1	61	230	11	MOLECULE:	KINETOCHORE PROTEIN NDC80	
39:00:00	2qa7-C	2.1	3.3	56	102	13	MOLECULE:	HUNTINGTIN-INTERACTING PROTEIN 1	
40:00:00	8th8-C	2.1	5.4	91	533	7	MOLECULE:	DYNEIN REGULATORY COMPLEX PROTEIN 1/2 N-TER	MINAL
41:00:00	5zak-A	2.1	16.7	66	1314	8	MOLECULE:	ENDORIBONUCLEASE DICER	
42:00:00	6c0f-w	2.1	3	43	70	5	MOLECULE:	SACCHAROMYCES CEREVISIAE S288C 35S PRE-RIBO	SOMAL
43:00:00	6m1h-B	2.1	2.6	45	61	9	MOLECULE:	PITUITARY ADENYLATE CYCLASE-ACTIVATING POLY	PEPTID
44:00:00	2db7-A	2.1	2.5	37	57	3	MOLECULE:	HAIRY/ENHANCER-OF-SPLIT RELATED WITH YRPW M	OTIF
45:00:00	1c02-A	2.1	3.3	51	166	8	MOLECULE:	PHOSPHOTRANSFERASE YPD1P	
46:00:00	7q83-D	2.1	2.8	46	156	9	MOLECULE:	EXOCYST COMPLEX COMPONENT SEC3	
47:00:00	7zpo-M	2.1	7.1	56	438	11	MOLECULE:	KTR SYSTEM POTASSIUM UPTAKE PROTEIN A	
48:00:00	7bjg-A	2.1	34	77	98	9	MOLECULE:	SD21996P	
49:00:00	7k15-A	2.1	7.1	65	455	6	MOLECULE:	LEUKOTRIENE B4 RECEPTOR 1,FLAVODOXIN,LEUKOT	RIENE
50:00:00	6zbj-A	2.1	6	75	666	4	MOLECULE:	PRECURSOR OF THE MAJOR MEROZOITE SURFACE AN	TIGENS
51:00:00	3icq-T	2	26.4	100	949	8	MOLECULE:	EXPORTIN-T	
52:00:00	7p34-A	2	24.8	146	391	5	MOLECULE:	PEPTIDE ANTIBIOTIC TRANSPORTER SBMA	
53:00:00	9f3f-1	2	27.1	149	751	6	MOLECULE:	NUCLEAR CAP BINDING COMPLEX SUBUNIT CBP110	
54:00:00	6fln-B	2	31.9	105	367	8	MOLECULE:	E3 UBIQUITIN/ISG15 LIGASE TRIM25	
55:00:00	2o2k-B	2	16.2	59	333	5	MOLECULE:	METHIONINE SYNTHASE	
56:00:00	7w7g-B	2	31	134	1710	6	MOLECULE:	PROTEIN UNC-79 HOMOLOG	
57:00:00	8uw3-A	2	15.4	106	1265	6	MOLECULE:	LINE-1 RETROTRANSPOSABLE ELEMENT ORF2 PROTE	IN
58:00:00	8hnc-A	2	14.8	101	570	8	MOLECULE:	SOLUTE CARRIER ORGANIC ANION TRANSPORTER FA	MILY M
59:00:00	8vb2-E	2	46	160	844	4	MOLECULE:	TETRAMERIC EJECTION PROTEIN (GP48)	
60:00:00	5eri-A	2	23.8	73	153	8	MOLECULE:	MARR FAMILY TRANSCRIPTIONAL REGULATOR	
61:00:00	8g0p-A	2	28.9	106	143	5	MOLECULE:	KINETOCHORE PROTEIN NDC80 HOMOLOG	
62:00:00	7bss-A	2	33.7	145	816	7	MOLECULE:	ATP11C	
63:00:00	8hhe-B	2	11.4	99	647	3	MOLECULE:	CRYSTALINE ENTOMOCIDAL PROTOXIN	
64:00:00	8agg-A	2	2.7	35	489	9	MOLECULE:	PHOSPHOCHOLINE HYDROLASE LEM3	
65:00:00	7qpr-D	2	25.5	92	689	5	MOLECULE:	ACT DOMAIN PROTEIN	
66:00:00	7w0y-k	2	8.7	77	98	4	MOLECULE:	NADH DEHYDROGENASE [UBIQUINONE] FLAVOPROTEI	N 1,
67:00:00	9exv-C	2	4.6	70	99	9	MOLECULE:	NITROREDUCTASE	
68:00:00	3va9-A	2	4.9	76	127	8	MOLECULE:	SENSOR HISTIDINE KINASE	
69:00:00	5zle-A	2	3.7	64	225	9	MOLECULE:	CYTOCHROME B REDUCTASE 1	
70:00:00	7xp9-A	2	7.2	48	76	10	MOLECULE:	RXLR EFFECTOR PROTEIN AVR-VNT11	
71:00:00	3ezh-A	2	4.1	69	114	3	MOLECULE:	NITRATE/NITRITE SENSOR PROTEIN NARX	
72:00:00	4gcz-B	2	11.1	84	378	4	MOLECULE:	BLUE-LIGHT PHOTORECEPTOR, SENSOR PROTEIN FI	XL
73:00:00	6g49-A	2	9.6	43	288	5	MOLECULE:	PROTEIN-GLUTAMINE GAMMA-GLUTAMYLTRANSFERASE	
74:00:00	6h4j-A	2	32.1	136	488	7	MOLECULE:	UBIQUITIN CARBOXYL-TERMINAL HYDROLASE 25	
75:00:00	4mk6-A	2	7.1	80	188	5	MOLECULE:	PROBABLE DIHYDROXYACETONE KINASE REGULATOR	DHSK_R
76:00:00	7qcd-C	2	11.4	93	267	8	MOLECULE:	STRUCTURAL MAINTENANCE OF CHROMOSOMES PROTE	IN 5
77:00:00	6tg5-A	2	6.7	63	111	3	MOLECULE:	MACPD	
78:00:00	4wcx-A	2	11.2	81	456	4	MOLECULE:	BIOTIN AND THIAMIN SYNTHESIS ASSOCIATED	
79:00:00	6gts-B	2	6	52	71	4	MOLECULE:	ACETYLTRANSFERASE	
80:00:00	4iwb-A	2	6.9	82	165	1	MOLECULE:	FLIC, FLIS CHIMERA	
81:00:00	4mu6-A	2	22.7	111	274	10	MOLECULE:	KINECTIN 1 (KINESIN RECEPTOR)	
82:00:00	6s6b-I	2	11	77	284	6	MOLECULE:	CRISPR-ASSOCIATED PROTEIN, CMR5 FAMILY	
83:00:00	3mxz-A	2	6.7	67	107	7	MOLECULE:	TUBULIN-SPECIFIC CHAPERONE A	
84:00:00	3viq-A	2	10.7	58	122	3	MOLECULE:	SWI5-DEPENDENT RECOMBINATION DNA REPAIR PRO	TEIN 1
85:00:00	8qem-M	2	2.2	38	454	3	MOLECULE:	PUTATIVE NECK PROTEIN	
86:00:00	8ec3-A	2	9.2	90	155	11	MOLECULE:	FIBRONECTIN-BINDING PROTEIN	
87:00:00	7ar9-M	2	8.3	92	438	1	MOLECULE:	ND3	
88:00:00	6tdv-O	2	19.7	84	100	2	MOLECULE:	ATPTB1	
89:00:00	9b8e-B	2	8.4	57	225	7	MOLECULE:	CEG10	
90:00:00	7fh1-A	2	7.3	96	874	6	MOLECULE:	MECKELIN	
91:00:00	7y82-A	2	23.7	90	1341	3	MOLECULE:	RAMP SUPERFAMILY PROTEIN	
92:00:00	6giy-G	2	6.4	103	444	3	MOLECULE:	TSSF	
93:00:00	1wa8-A	2	7.8	72	99	7	MOLECULE:	ESAT-6 LIKE PROTEIN ESXB	
94:00:00	5y2v-A	2	17.4	66	304	11	MOLECULE:	RUBISCO OPERON TRANSCRIPTIONAL REGULATOR	
95:00:00	8fbi-A	2	6.5	93	274	4	MOLECULE:	KWOCA_39	
96:00:00	6iiv-A	2	4.8	97	461	1	MOLECULE:	SOLUBLE CYTOCHROME B562,THROMBOXANE A2 RECE	PTOR,R
97:00:00	8z9a-A	2	9.5	128	406	1	MOLECULE:	ODORANT RECEPTOR, APISORCO	
98:00:00	7z0s-C	2	13.3	118	604	6	MOLECULE:	FORMATE HYDROGENLYASE SUBUNIT 3	
99:00:00	6mev-A	2	3.4	36	342	3	MOLECULE:	BIFUNCTIONAL ARGININE DEMETHYLASE AND LYSYL	#NAME?
0:00	6z0c-A	2	5.5	97	199	5	MOLECULE:	MAQUETTE-3	
1:00	3zqq-C	2	12.2	82	134	9	MOLECULE:	TERMINASE SMALL SUBUNIT	
2:00	6o1q-A	2	4.5	74	119	4	MOLECULE:	NEPHROCYSTIN-1	
3:00	8d8j-5	2	9.2	54	270	4	MOLECULE:	PROBABLE S-ADENOSYL-L-METHIONINE-DEPENDENT	RNA
4:00	8ver-B	2	5.8	96	287	5	MOLECULE:	ENDORIBONUCLEASE YICC	
5:00	5m1m-A	2	4.4	65	154	9	MOLECULE:	MATRIX PROTEIN 1	
6:00	3l9f-A	2	2.9	58	170	7	MOLECULE:	PUTATIVE UNCHARACTERIZED PROTEIN SMU.1604C	
7:00	9c3i-S	2	3.3	75	231	5	MOLECULE:	CCR4-NOT TRANSCRIPTION COMPLEX SUBUNIT 3	
8:00	2wtt-D	2	2.6	43	47	5	MOLECULE:	TUMOR PROTEIN P73	
9:00	8qx8-D	2	12.4	43	613	2	MOLECULE:	VACUOLAR PROTEIN SORTING-ASSOCIATED PROTEIN	8
10:00	2k73-A	2	11.5	94	183	5	MOLECULE:	DISULFIDE BOND FORMATION PROTEIN B	
11:00	4bc2-A	2	8	55	528	9	MOLECULE:	XYLULOSE KINASE	
12:00	5yma-A	2	7.9	77	179	5	MOLECULE:	PUTATIVE RRNA PROCESSING PROTEIN	
13:00	6idy-A	2	15.1	50	421	16	MOLECULE:	LIPASE AFLB	
14:00	8rmm-E	2	19.8	119	290	3	MOLECULE:	CALCIUM HOMEOSTASIS MODULATOR PROTEIN 4	
15:00	6umm-E	2	5.6	87	343	11	MOLECULE:	ESX-3 SECRETION SYSTEM PROTEIN ECCE3	
16:00	6u8y-b	2	4.9	65	79	6	MOLECULE:	MONOVALENT CATION/H+ ANTIPORTER SUBUNIT E	
17:00	6iuq-B	2	2.9	37	296	3	MOLECULE:	PROLYL 4-HYDROXYLASE	
18:00	5cuf-C	2	14.8	70	370	7	MOLECULE:	SESTRIN-2	
19:00	8z1e-A	2	6.3	105	278	9	MOLECULE:	UNCHARACTERIZED PROTEIN UL78	
20:00	6tc0-C	2	25.7	127	994	6	MOLECULE:	PROBABLE CYTOSOLIC IRON-SULFUR PROTEIN ASSE	MBLY P

21:00	6m9a-C	2	8.7	74	157	7	MOLECULE:	SIGNALING PROTEIN	
22:00	5nmo-A	2	4.9	75	162	3	MOLECULE:	CHROMOSOME PARTITION PROTEIN SMC,CHROMOSOME	PARTI
23:00	6nr8-3	2	4.1	82	132	7	MOLECULE:	PREFOLDIN SUBUNIT 1	
24:00:00	6cum-A	2	5.4	48	51	4	MOLECULE:	LAO/AO TRANSPORT SYSTEM ATPASE	
25:00:00	8gi1-A	2	4.7	72	144	6	MOLECULE:	ACCESSORY PROTEIN CSX28	
26:00:00	5afr-A	2	7.9	77	332	5	MOLECULE:	DYNEIN HEAVY CHAIN, CYTOPLASMIC	
27:00:00	4cpg-A	2	2.5	40	69	5	MOLECULE:	SMALL GLUTAMINE-RICH TETRATRICOPEPTIDE REPE	AT-CON
28:00:00	6r24-A	2	5.9	57	159	9	MOLECULE:	TRANSPOSON TY3-I GAG-POL POLYPROTEIN	
29:00:00	5w3s-A	2	7.5	120	485	3	MOLECULE:	MUCOLIPIN-3 ISOFORM 1	
30:00:00	6bk4-A	2	5.1	60	106	3	MOLECULE:	CAPRIN HOMOLOG	
31:00:00	8qpk-S	2	6	48	73	6	MOLECULE:	PROBABLE ATP-DEPENDENT RNA HELICASE DDX23	
32:00:00	7pkq-C	2	26.5	118	369	11	MOLECULE:	MS35	
33:00:00	7k9y-A	2	3.2	57	425	0	MOLECULE:	TRT	
34:00:00	8gup-A	2	8.2	63	159	3	MOLECULE:	TYPE VII SECRETION SYSTEM PROTEIN ESAG	
35:00:00	2ptf-A	2	7.8	51	200	8	MOLECULE:	UNCHARACTERIZED PROTEIN MTH_863	
36:00:00	5ziy-A	2	3.2	86	195	6	MOLECULE:	FLAGELLAR HOOK-ASSOCIATED PROTEIN 3	
37:00:00	7woo-F	2	19.3	120	1622	1	MOLECULE:	NUCLEOPORIN NIC96	
38:00:00	8qt5-A	2	15.5	83	248	5	MOLECULE:	14-3-3-LIKE PROTEIN G-BOX FACTOR 14 LAMBDA,	PROTEI
39:00:00	8t5e-A	2	4	42	130	2	MOLECULE:	BIM_FULLDIFF	
40:00:00	7jqe-A	2	2.8	46	357	7	MOLECULE:	ESAT-6/WXG100 SECRETION SYSTEM PROTEIN	
41:00:00	4oe8-C	2	3.7	41	87	15	MOLECULE:	INTERLEUKIN-12 SUBUNIT BETA	
42:00:00	8th8-s	2	54	85	187	7	MOLECULE:	DYNEIN REGULATORY COMPLEX PROTEIN 1/2 N-TER	MINAL
43:00:00	6f62-A	2	3.6	59	105	5	MOLECULE:	SYNAPTONEMAL COMPLEX PROTEIN 1	
44:00:00	4ddg-A	2	6.4	55	399	16	MOLECULE:	UBIQUITIN-CONJUGATING ENZYME E2 D2, UBIQUIT	IN THI
45:00:00	7upq-F	2	4.3	51	77	10	MOLECULE:	DHT03 PROTEIN A	

DALI results: MnA-1 Cah

No:	Chain	Z	rmsd	lali	nres	%id	PDB Description	
1:00	2qih-A	8.1	7.5	136	136	6	MOLECULE:	PROTEIN USPA1
2:00	5jlh-J	8	7	134	135	0	MOLECULE:	ACTIN, CYTOPLASMIC 2
3:00	5jlh-H	7.9	7	134	135	0	MOLECULE:	ACTIN, CYTOPLASMIC 2
4:00	2b9c-B	7.8	7.1	139	142	5	MOLECULE:	STRIATED-MUSCLE ALPHA TROPOMYOSIN
5:00	4cpc-H	7.7	8.5	138	140	9	MOLECULE:	SYNAPTONEMAL COMPLEX PROTEIN 3
6:00	4pxu-B	7.7	6.8	136	136	6	MOLECULE:	BIPOLAR KINESIN KRP-130
7:00	3j8a-G	7.7	5.1	135	135	0	MOLECULE:	TROPOMYOSIN ALPHA-1
8:00	4cpc-A	7.5	7.7	141	143	9	MOLECULE:	SYNAPTONEMAL COMPLEX PROTEIN 3
9:00	4cpc-C	7.5	8	139	144	9	MOLECULE:	SYNAPTONEMAL COMPLEX PROTEIN 3
10:00	6vol-J	7.5	9.1	131	133	11	MOLECULE:	ATP SYNTHASE SUBUNIT ALPHA, CHLOROPLASTIC
11:00	2b9c-A	7.5	7.9	135	136	7	MOLECULE:	STRIATED-MUSCLE ALPHA TROPOMYOSIN
12:00	2d3e-D	7.5	5.2	130	130	12	MOLECULE:	GENERAL CONTROL PROTEIN GCN4 AND TROPOMYOSIN
13:00	2d3e-B	7.5	5.2	130	130	12	MOLECULE:	GENERAL CONTROL PROTEIN GCN4 AND TROPOMYOSIN
14:00	2efr-C	7.4	6.8	133	154	8	MOLECULE:	GENERAL CONTROL PROTEIN GCN4 AND TROPOMYOSIN
15:00	2efs-B	7.4	5.4	133	154	8	MOLECULE:	GENERAL CONTROL PROTEIN GCN4 AND TROPOMYOSIN
16:00	6x5z-P	7.4	8.6	151	166	6	MOLECULE:	ACTIN, ALPHA SKELETAL MUSCLE
17:00	4cpc-B	7.4	7	130	144	6	MOLECULE:	SYNAPTONEMAL COMPLEX PROTEIN 3
18:00	8h9g-K	7.4	5.6	136	198	4	MOLECULE:	ATP SYNTHASE F(0) COMPLEX SUBUNIT B1, MITOCH
19:00	4cpc-F	7.4	7	129	144	7	MOLECULE:	SYNAPTONEMAL COMPLEX PROTEIN 3
20:00	6gap-B	7.3	6.5	134	214	6	MOLECULE:	OUTER CAPSID PROTEIN SIGMA-1
21:00	4cpc-E	7.3	7.5	138	143	4	MOLECULE:	SYNAPTONEMAL COMPLEX PROTEIN 3
22:00	5to7-C	7.3	3.9	128	141	7	MOLECULE:	NUCLEOPROTEIN TPR
23:00	7z8k-W	7.3	7.6	152	165	9	MOLECULE:	CYTOPLASMIC DYNEIN 1 HEAVY CHAIN 1
24:00:00	7dl2-C	7.2	4.3	134	224	8	MOLECULE:	HAMARTIN
25:00:00	6snz-D	7.2	6.1	127	143	9	MOLECULE:	PRELAMIN-A/C
26:00:00	8enc-P	7.2	9.6	145	166	6	MOLECULE:	MYOSIN-7
27:00:00	8efi-P	7.2	10	146	166	6	MOLECULE:	MYOSIN-7
28:00:00	5to6-D	7.2	5.4	128	140	6	MOLECULE:	NUCLEOPROTEIN TPR
29:00:00	6snz-C	7.1	7	138	146	5	MOLECULE:	PRELAMIN-A/C
30:00:00	7qin-I	7.1	5.2	118	118	0	MOLECULE:	ACTIN, ALPHA SKELETAL MUSCLE
31:00:00	7jg5-b	7.1	8.9	142	145	8	MOLECULE:	ATP SYNTHASE SUBUNIT ALPHA
32:00:00	8aia-N	7.1	7.8	150	156	5	MOLECULE:	CRESCENTIN
33:00:00	8aix-N	7.1	7.8	150	156	5	MOLECULE:	CRESCENTIN
34:00:00	7ogt-A	7.1	6.7	129	688	5	MOLECULE:	STRUCTURAL MAINTENANCE OF CHROMOSOMES PROTEI
35:00:00	8aix-X	7.1	8	149	156	6	MOLECULE:	CRESCENTIN
36:00:00	5to6-B	7.1	7	127	139	7	MOLECULE:	NUCLEOPROTEIN TPR
37:00:00	6vm4-I	7	6.4	140	154	4	MOLECULE:	ATP SYNTHASE SUBUNIT ALPHA, CHLOROPLASTIC
38:00:00	6dd9-B	7	6	123	127	5	MOLECULE:	SYNAPTONEMAL COMPLEX PROTEIN 3
39:00:00	6dd8-A	7	5.7	127	131	2	MOLECULE:	SYNAPTONEMAL COMPLEX PROTEIN 3
40:00:00	8ajb-S	7	9	148	155	5	MOLECULE:	CRESCENTIN
41:00:00	6gao-B	7	7.7	145	225	8	MOLECULE:	OUTER CAPSID PROTEIN SIGMA-1
42:00:00	5to7-B	7	5.5	128	141	6	MOLECULE:	NUCLEOPROTEIN TPR
43:00:00	6snz-B	7	6.9	136	155	4	MOLECULE:	PRELAMIN-A/C
44:00:00	7z8k-x	7	4.7	131	135	10	MOLECULE:	CYTOPLASMIC DYNEIN 1 HEAVY CHAIN 1
45:00:00	6f1u-X	7	2.5	78	79	8	MOLECULE:	ARP1 ACTIN RELATED PROTEIN 1 HOMOLOG A
46:00:00	6wnq-Y	7	9.8	131	156	8	MOLECULE:	ATP SYNTHASE SUBUNIT DELTA
47:00:00	6yvu-B	7	11	150	1191	7	MOLECULE:	STRUCTURAL MAINTENANCE OF CHROMOSOMES PROTEI
48:00:00	5tvb-A	7	5.6	127	140	7	MOLECULE:	NUCLEOPROTEIN TPR
49:00:00	5to6-A	7	4.8	126	141	10	MOLECULE:	NUCLEOPROTEIN TPR

50:00:00	6oqu-Y	7	5	137	156	4	MOLECULE:	ATP SYNTHASE SUBUNIT DELTA
51:00:00	8tgt-A	6.9	3.7	76	76	12	MOLECULE:	M PROTEIN
52:00:00	8ahl-G	6.9	6.8	134	155	5	MOLECULE:	CRESCENTIN
53:00:00	8ga8-G	6.9	4.6	79	97	6	MOLECULE:	TRANSCRIPTIONAL REGULATORY PROTEIN SDS3
54:00:00	4wy4-C	6.9	3.4	78	78	8	MOLECULE:	VESICLE-ASSOCIATED MEMBRANE PROTEIN 8
55:00:00	5app-B	6.9	8	126	128	10	MOLECULE:	GENERAL CONTROL PROTEIN GCN4, OUTER MEMBRANE
56:00:00	6vol-I	6.9	8.4	143	150	8	MOLECULE:	ATP SYNTHASE SUBUNIT ALPHA, CHLOROPLASTIC
57:00:00	5fiy-E	6.9	2.6	78	78	4	MOLECULE:	PRKC APOPTOSIS WT1 REGULATOR PROTEIN
58:00:00	5bu5-F	6.9	3.2	81	174	9	MOLECULE:	DNA STABILIZATION PROTEIN
59:00:00	1gk4-D	6.9	2.2	78	78	5	MOLECULE:	VIMENTIN
60:00:00	6hk9-B	6.9	4.7	77	78	6	MOLECULE:	TESTIS-EXPRESSED PROTEIN 12
61:00:00	5zvm-B	6.9	4.8	76	76	3	MOLECULE:	SPIKE GLYCOPROTEIN
62:00:00	8csp-U	6.9	10.5	127	174	11	MOLECULE:	28S RIBOSOMAL PROTEIN S34, MITOCHONDRIAL
63:00:00	6z47-H	6.9	5.1	135	257	13	MOLECULE:	MYOSIN HEAVY CHAIN 11
64:00:00	2e7s-F	6.9	6	124	124	10	MOLECULE:	RAB GUANINE NUCLEOTIDE EXCHANGE FACTOR SEC2
65:00:00	2e7s-E	6.9	3.6	124	124	8	MOLECULE:	RAB GUANINE NUCLEOTIDE EXCHANGE FACTOR SEC2
66:00:00	8txr-C	6.8	8.4	148	431	6	MOLECULE:	EXODEOXYRIBONUCLEASE 7 LARGE SUBUNIT
67:00:00	6vm4-J	6.8	7.7	132	136	6	MOLECULE:	ATP SYNTHASE SUBUNIT ALPHA, CHLOROPLASTIC
68:00:00	6zpj-B	6.8	4.2	77	77	9	MOLECULE:	LEISHMANIA MEXICANA KKT4
69:00:00	8uzx-F	6.8	2.7	82	82	5	MOLECULE:	ACTIN, ALPHA CARDIAC MUSCLE 1
70:00:00	6voj-I	6.8	7	140	154	5	MOLECULE:	ATP SYNTHASE SUBUNIT ALPHA, CHLOROPLASTIC
71:00:00	8afe-F	6.8	4.9	79	79	10	MOLECULE:	CRESCENTIN
72:00:00	5ccg-C	6.8	2.5	74	74	5	MOLECULE:	VESICLE-ASSOCIATED MEMBRANE PROTEIN 2
73:00:00	8uzy-F	6.8	2.8	82	82	5	MOLECULE:	ACTIN, ALPHA CARDIAC MUSCLE 1
74:00:00	6fkf-p	6.8	9.2	141	143	6	MOLECULE:	ATP SYNTHASE SUBUNIT ALPHA, CHLOROPLASTIC
75:00:00	6mi4-B	6.8	3.1	121	123	10	MOLECULE:	NF-KB ESSENTIAL MODULATOR
76:00:00	8afh-F	6.8	4.9	79	79	10	MOLECULE:	CRESCENTIN
77:00:00	6igv-A	6.8	2.7	117	118	8	MOLECULE:	KINESIN-1 HEAVY CHAIN
78:00:00	5fiy-C	6.8	4.9	78	78	3	MOLECULE:	PRKC APOPTOSIS WT1 REGULATOR PROTEIN
79:00:00	8afh-P	6.8	4.9	79	79	10	MOLECULE:	CRESCENTIN
80:00:00	8v01-G	6.8	4	81	82	6	MOLECULE:	ACTIN, ALPHA CARDIAC MUSCLE 1
81:00:00	8v01-F	6.8	4.8	81	82	6	MOLECULE:	ACTIN, ALPHA CARDIAC MUSCLE 1
82:00:00	7bji-C	6.8	5.1	79	80	3	MOLECULE:	CENTROSOMAL PROTEIN OF 135 KDA
83:00:00	8fcf-D	6.8	3.6	77	79	6	MOLECULE:	PLASMALEMMA VESICLE-ASSOCIATED PROTEIN
84:00:00	8v0y-G	6.8	4.3	81	82	6	MOLECULE:	ACTIN, ALPHA CARDIAC MUSCLE 1
85:00:00	8uzy-G	6.8	4	81	82	6	MOLECULE:	ACTIN, ALPHA CARDIAC MUSCLE 1
86:00:00	7wxz-D	6.8	4.8	76	187	3	MOLECULE:	SPIKE PROTEIN S2'
87:00:00	5xis-A	6.8	4.8	79	79	4	MOLECULE:	E3 UBIQUITIN-PROTEIN LIGASE RNF168
88:00:00	6h9l-C	6.8	6.7	105	128	11	MOLECULE:	UNCHARACTERIZED PROTEIN
89:00:00	6l5h-A	6.8	4.8	78	82	4	MOLECULE:	ROOTLETIN
90:00:00	5dn6-V	6.8	3.7	78	78	0	MOLECULE:	CHAIN A
91:00:00	6l5h-B	6.8	4.2	81	82	6	MOLECULE:	ROOTLETIN
92:00:00	8fcf-C	6.8	3.4	81	83	7	MOLECULE:	PLASMALEMMA VESICLE-ASSOCIATED PROTEIN
93:00:00	6oqa-D	6.8	4.8	83	83	13	MOLECULE:	PEPTIDYL-PROLYL CIS-TRANS ISOMERASE FKBP1A
94:00:00	5whf-F	6.8	3.1	84	84	4	MOLECULE:	VIMENTIN
95:00:00	7jga-b	6.8	9.9	133	141	9	MOLECULE:	ATP SYNTHASE SUBUNIT ALPHA
96:00:00	8ajb-H	6.8	9	147	163	4	MOLECULE:	CRESCENTIN
97:00:00	8sjj-D	6.8	3	81	86	6	MOLECULE:	SEPTIN-14
98:00:00	8ahl-H	6.8	9	147	163	4	MOLECULE:	CRESCENTIN
99:00:00	6qum-K	6.8	3.5	85	98	5	MOLECULE:	V-TYPE ATP SYNTHASE ALPHA CHAIN
0:00	8jr0-b	6.8	8.6	134	144	3	MOLECULE:	ATP SYNTHASE SUBUNIT ALPHA

1:00	8j0t-b	6.8	9.9	138	144	7	MOLECULE:	ATP SYNTHASE SUBUNIT ALPHA
2:00	5noj-F	6.7	6.2	130	136	0	MOLECULE:	ACTIN, ALPHA SKELETAL MUSCLE
3:00	6vmg-I	6.7	6.8	135	148	8	MOLECULE:	ATP SYNTHASE SUBUNIT A, CHLOROPLASTIC
4:00	5to7-A	6.7	5.5	131	141	10	MOLECULE:	NUCLEOPROTEIN TPR
5:00	5adx-z	6.7	22.1	119	419	0	MOLECULE:	ACTIN RELATED PROTEIN 1
6:00	5to7-D	6.7	4.4	130	141	11	MOLECULE:	NUCLEOPROTEIN TPR
7:00	6aoz-A	6.7	3.6	74	74	5	MOLECULE:	CASP8-ASSOCIATED PROTEIN 2
8:00	4yv3-A	6.7	4.2	77	77	6	MOLECULE:	VIMENTIN
9:00	7rzt-A	6.7	4.7	71	71	10	MOLECULE:	SARS-COV-2 HR1 S940F LINKED TO A SCAFFOLD,SP
10:00	7rzv-B	6.7	4.7	71	71	11	MOLECULE:	SARS-COV-2 HR1 LINKED TO A SCAFFOLD,SPIKE PR
11:00	7tik-A	6.7	4.8	71	71	11	MOLECULE:	FERRITIN, DPS FAMILY PROTEIN AND SPIKE PROTE
12:00	7tik-C	6.7	4.8	71	71	11	MOLECULE:	FERRITIN, DPS FAMILY PROTEIN AND SPIKE PROTE
13:00	8v0y-F	6.7	2.7	82	82	4	MOLECULE:	ACTIN, ALPHA CARDIAC MUSCLE 1
14:00	6vmb-I	6.7	8.9	143	153	7	MOLECULE:	ATP SYNTHASE SUBUNIT ALPHA, CHLOROPLASTIC
15:00	1gk4-B	6.7	4.3	79	79	8	MOLECULE:	VIMENTIN
16:00	3bat-D	6.7	4.6	78	80	12	MOLECULE:	MYOSIN HEAVY CHAIN, STRIATED MUSCLE/GENERAL
17:00	7tj7-V	6.7	5	128	135	0	MOLECULE:	CARDIAC ACTIN
18:00	8fxf-A	6.7	4.1	78	78	4	MOLECULE:	E3 UBIQUITIN-PROTEIN LIGASE TRIM56
19:00	2fxo-A	6.7	3.6	127	129	6	MOLECULE:	MYOSIN HEAVY CHAIN, CARDIAC MUSCLE BETA ISOF
20:00	5cci-C	6.7	2.5	73	73	5	MOLECULE:	VESICLE-ASSOCIATED MEMBRANE PROTEIN 2
21:00	5ccg-I	6.7	2.6	74	74	5	MOLECULE:	VESICLE-ASSOCIATED MEMBRANE PROTEIN 2
22:00	7jh7-J	6.7	5	128	135	0	MOLECULE:	ACTIN, ALPHA CARDIAC MUSCLE 1
23:00	5cch-C	6.7	2.6	74	74	5	MOLECULE:	VESICLE-ASSOCIATED MEMBRANE PROTEIN 2
24:00:00	2fxo-C	6.7	4.5	126	127	6	MOLECULE:	MYOSIN HEAVY CHAIN, CARDIAC MUSCLE BETA ISOF
25:00:00	8i3e-B	6.7	4.2	107	135	10	MOLECULE:	ELKS/RAB6-INTERACTING/CAST FAMILY MEMBER 1
26:00:00	7uw5-B	6.7	24.7	132	723	8	MOLECULE:	MECHANOSENSITIVE CHANNEL MSCK
27:00:00	8uzx-G	6.7	4.2	81	82	6	MOLECULE:	ACTIN, ALPHA CARDIAC MUSCLE 1
28:00:00	8uwx-G	6.7	4.1	81	82	6	MOLECULE:	ACTIN, ALPHA CARDIAC MUSCLE 1
29:00:00	8v0k-G	6.7	4	81	82	6	MOLECULE:	ACTIN, ALPHA CARDIAC MUSCLE 1
30:00:00	8uz6-G	6.7	4.1	81	82	6	MOLECULE:	ACTIN, ALPHA CARDIAC MUSCLE 1
31:00:00	8uyd-F	6.7	4.9	81	82	6	MOLECULE:	ACTIN, ALPHA CARDIAC MUSCLE 1
32:00:00	8v0i-F	6.7	4.8	81	82	6	MOLECULE:	ACTIN, ALPHA CARDIAC MUSCLE 1
33:00:00	8uyd-G	6.7	4.1	81	82	6	MOLECULE:	ACTIN, ALPHA CARDIAC MUSCLE 1
34:00:00	8v0k-F	6.7	4.9	81	82	6	MOLECULE:	ACTIN, ALPHA CARDIAC MUSCLE 1
35:00:00	8uww-G	6.7	4.1	81	82	6	MOLECULE:	ACTIN, ALPHA CARDIAC MUSCLE 1
36:00:00	3bas-A	6.7	4.4	79	80	11	MOLECULE:	MYOSIN HEAVY CHAIN, STRIATED MUSCLE/GENERAL
37:00:00	5jxc-C	6.7	3.8	78	78	4	MOLECULE:	RAS/RAP GTPASE-ACTIVATING PROTEIN SYNGAP
38:00:00	6dm9-C	6.7	4.5	78	78	5	MOLECULE:	DHD15_EXTENDED_A
39:00:00	2bez-C	6.7	2.5	77	77	6	MOLECULE:	E2 GLYCOPROTEIN
40:00:00	7tk9-U	6.7	5.6	128	155	9	MOLECULE:	ATP SYNTHASE SUBUNIT 9, MITOCHONDRIAL
41:00:00	8uwy-F	6.7	3.5	80	82	8	MOLECULE:	ACTIN, ALPHA CARDIAC MUSCLE 1
42:00:00	2nps-C	6.7	4.1	79	79	9	MOLECULE:	VESICLE-ASSOCIATED MEMBRANE PROTEIN 4
43:00:00	8uwx-F	6.7	3.5	80	82	8	MOLECULE:	ACTIN, ALPHA CARDIAC MUSCLE 1
44:00:00	8uz5-F	6.7	3.5	80	82	8	MOLECULE:	ACTIN, ALPHA CARDIAC MUSCLE 1
45:00:00	5xis-D	6.7	4.6	81	82	6	MOLECULE:	E3 UBIQUITIN-PROTEIN LIGASE RNF168
46:00:00	8uww-F	6.7	3.5	80	82	8	MOLECULE:	ACTIN, ALPHA CARDIAC MUSCLE 1
47:00:00	2jee-A	6.7	2.4	77	78	6	MOLECULE:	CELL DIVISION PROTEIN ZAPB
48:00:00	2jee-B	6.7	2.4	77	78	6	MOLECULE:	CELL DIVISION PROTEIN ZAPB
49:00:00	2jee-C	6.7	2.4	77	78	6	MOLECULE:	CELL DIVISION PROTEIN ZAPB
50:00:00	2jee-D	6.7	2.4	77	78	6	MOLECULE:	CELL DIVISION PROTEIN ZAPB
51:00:00	4zqa-A	6.7	4.4	83	86	12	MOLECULE:	SIN3 HISTONE DEACETYLASE COREPRESSOR COMPLEX

52:00:00	8sdi-D	6.7	3.8	79	80	10	MOLECULE:	TRIPARTITE MOTIF-CONTAINING PROTEIN 45
53:00:00	8sjj-C	6.7	3.4	83	86	6	MOLECULE:	SEPTIN-14
54:00:00	2e7s-B	6.7	3.6	121	121	7	MOLECULE:	RAB GUANINE NUCLEOTIDE EXCHANGE FACTOR SEC2
55:00:00	2e7s-D	6.7	3.3	124	124	10	MOLECULE:	RAB GUANINE NUCLEOTIDE EXCHANGE FACTOR SEC2
56:00:00	8th8-D	6.6	9.9	148	292	9	MOLECULE:	DYNEIN REGULATORY COMPLEX PROTEIN 1/2 N-TERM
57:00:00	6mi3-A	6.6	3.7	119	122	8	MOLECULE:	NF-KB ESSENTIAL MODULATOR,NF-KAPPA-B ESSENTI
58:00:00	6jlb-A	6.6	8	148	202	5	MOLECULE:	LAMIN A/C
59:00:00	2ocy-A	6.6	9.6	144	149	9	MOLECULE:	RAB GUANINE NUCLEOTIDE EXCHANGE FACTOR SEC2
60:00:00	6ign-A	6.6	5.4	122	123	6	MOLECULE:	KINESIN-1 HEAVY CHAIN
61:00:00	7p2y-p	6.6	8.9	146	155	4	MOLECULE:	ATP SYNTHASE SUBUNIT ALPHA
62:00:00	5xau-B	6.6	4.1	71	71	4	MOLECULE:	LAMININ SUBUNIT ALPHA-5
63:00:00	8cst-U	6.6	10.9	126	174	13	MOLECULE:	28S RIBOSOMAL PROTEIN S34, MITOCHONDRIAL
64:00:00	6vof-I	6.6	6.7	138	151	4	MOLECULE:	ATP SYNTHASE SUBUNIT ALPHA, CHLOROPLASTIC
65:00:00	4mvd-B	6.6	6.8	97	253	7	MOLECULE:	CHOLINE-PHOSPHATE CYTIDYLYLTRANSFERASE A
66:00:00	1kil-C	6.6	1.8	72	72	7	MOLECULE:	SYNAPTOBREVIN SNARE MOTIF
67:00:00	6von-J	6.6	8.8	129	130	7	MOLECULE:	ATP SYNTHASE SUBUNIT ALPHA, CHLOROPLASTIC
68:00:00	5djn-C	6.6	4.8	80	81	6	MOLECULE:	KINESIN-LIKE PROTEIN
69:00:00	7tj7-T	6.6	4.5	129	135	0	MOLECULE:	CARDIAC ACTIN
70:00:00	7tit-N	6.6	4.9	128	135	0	MOLECULE:	CARDIAC ACTIN
71:00:00	4mvd-F	6.6	4.5	96	253	7	MOLECULE:	CHOLINE-PHOSPHATE CYTIDYLYLTRANSFERASE A
72:00:00	5djn-A	6.6	4.5	77	77	6	MOLECULE:	KINESIN-LIKE PROTEIN
73:00:00	4a7f-B	6.6	6.4	133	136	5	MOLECULE:	ACTIN, ALPHA SKELETAL MUSCLE
74:00:00	8uz5-G	6.6	4.1	81	82	6	MOLECULE:	ACTIN, ALPHA CARDIAC MUSCLE 1
75:00:00	6ano-A	6.6	3.8	75	75	4	MOLECULE:	CASP8-ASSOCIATED PROTEIN 2
76:00:00	8qrl-U	6.6	19.3	134	176	10	MOLECULE:	12S MITOCHONDRIAL RRNA
77:00:00	1fav-A	6.6	4.3	78	78	3	MOLECULE:	HIV-1 ENVELOPE PROTEIN CHIMERA
78:00:00	7bji-A	6.6	4.5	80	82	3	MOLECULE:	CENTROSOMAL PROTEIN OF 135 KDA
79:00:00	5lxn-A	6.6	4.2	80	81	5	MOLECULE:	TRANSFORMING ACIDIC COILED-COIL-CONTAINING P
80:00:00	6oqa-H	6.6	3.3	84	85	6	MOLECULE:	PEPTIDYL-PROLYL CIS-TRANS ISOMERASE FKBP1A
81:00:00	8uz6-F	6.6	3.5	80	82	8	MOLECULE:	ACTIN, ALPHA CARDIAC MUSCLE 1
82:00:00	5k7b-C	6.6	3.5	83	86	6	MOLECULE:	BECLIN-2
83:00:00	8afh-E	6.6	3.7	78	79	8	MOLECULE:	CRESCENTIN
84:00:00	8v0i-G	6.6	4.9	81	82	6	MOLECULE:	ACTIN, ALPHA CARDIAC MUSCLE 1
85:00:00	7tk4-U	6.6	7.2	140	155	4	MOLECULE:	ATP SYNTHASE SUBUNIT 9, MITOCHONDRIAL
86:00:00	4jpn-J	6.6	2.1	72	72	6	MOLECULE:	MINOR SPIKE PROTEIN H
87:00:00	5k7b-A	6.6	2	78	87	13	MOLECULE:	BECLIN-2
88:00:00	8gt9-B	6.6	2.5	87	88	9	MOLECULE:	BECLIN-2
89:00:00	3bas-B	6.6	3.4	82	87	4	MOLECULE:	MYOSIN HEAVY CHAIN, STRIATED MUSCLE/GENERAL
90:00:00	7qin-J	6.6	3.4	87	88	0	MOLECULE:	ACTIN, ALPHA SKELETAL MUSCLE
91:00:00	6vzf-A	6.6	3.4	82	86	5	MOLECULE:	AUTOPHAGY-RELATED PROTEIN 11
92:00:00	5d3a-A	6.6	2.8	79	82	6	MOLECULE:	KINESIN-LIKE PROTEIN KIF21A
93:00:00	5jxc-E	6.6	1.7	82	84	7	MOLECULE:	RAS/RAP GTPASE-ACTIVATING PROTEIN SYNGAP
94:00:00	6ys4-D	6.6	4.9	85	87	7	MOLECULE:	SPINDLE ASSEMBLY ABNORMAL PROTEIN 6 HOMOLOG
95:00:00	5yl9-A	6.6	3	86	88	8	MOLECULE:	SPIKE GLYCOPROTEIN
96:00:00	7eqc-C	6.6	5.9	102	106	6	MOLECULE:	CYTOKINESIS DEFECT
97:00:00	2e7s-C	6.6	2.3	118	118	3	MOLECULE:	RAB GUANINE NUCLEOTIDE EXCHANGE FACTOR SEC2
98:00:00	5hmo-A	6.5	3.9	124	131	10	MOLECULE:	UNCONVENTIONAL MYOSIN-X
99:00:00	4zry-A	6.5	2.4	110	110	5	MOLECULE:	KERATIN, TYPE I CYTOSKELETAL 10
0:00	6wvw-G	6.5	4.2	74	74	5	MOLECULE:	VESICLE-ASSOCIATED MEMBRANE PROTEIN 2
1:00	5zvk-A	6.5	4	70	70	6	MOLECULE:	HUMAN CORONAVIRUS MERS HR1 MOTIF
2:00	6klq-A	6.5	5.7	132	135	0	MOLECULE:	TROPOMYOSIN

3:00	5mq4-D	6.5	4.8	74	111	7	MOLECULE:	PROTEIN KINASE C-BINDING PROTEIN 1
4:00	6wsm-A	6.5	4.4	74	74	7	MOLECULE:	SEPTIN-8
5:00	5nog-G	6.5	6.3	131	135	0	MOLECULE:	CARDIAC MUSCLE ALPHA ACTIN 1
6:00	8fxf-C	6.5	4.3	78	78	4	MOLECULE:	E3 UBIQUITIN-PROTEIN LIGASE TRIM56
7:00	8fxf-D	6.5	4.2	78	78	3	MOLECULE:	E3 UBIQUITIN-PROTEIN LIGASE TRIM56
8:00	7eqc-B	6.5	4.5	101	101	6	MOLECULE:	CYTOKINESIS DEFECT
9:00	8fxf-B	6.5	4.1	78	78	3	MOLECULE:	E3 UBIQUITIN-PROTEIN LIGASE TRIM56
10:00	5ydk-F	6.5	5.7	80	82	10	MOLECULE:	E3 UBIQUITIN-PROTEIN LIGASE RNF168
11:00	5xit-E	6.5	3.3	75	75	7	MOLECULE:	UBIQUITIN-40S RIBOSOMAL PROTEIN S27A
12:00	5whf-D	6.5	3.6	85	85	5	MOLECULE:	VIMENTIN
13:00	6cp3-Z	6.5	6.6	138	155	4	MOLECULE:	ATP SYNTHASE SUBUNIT 9, MITOCHONDRIAL
14:00	7tkp-U	6.5	11.5	146	155	8	MOLECULE:	ATP SYNTHASE SUBUNIT 9
15:00	6abo-B	6.5	4.2	75	77	3	MOLECULE:	DNA REPAIR PROTEIN XRCC4
16:00	6ap0-B	6.5	4.3	88	88	8	MOLECULE:	CASP8-ASSOCIATED PROTEIN 2
17:00	2e7s-K	6.5	4.2	116	117	5	MOLECULE:	RAB GUANINE NUCLEOTIDE EXCHANGE FACTOR SEC2
18:00	7uw5-A	6.4	36.1	111	723	8	MOLECULE:	MECHANOSENSITIVE CHANNEL MSCK
19:00	4f61-I	6.4	5.2	146	234	3	MOLECULE:	TUBULIN ALPHA CHAIN
20:00	5oag-B	6.4	2.7	97	97	11	MOLECULE:	RAB-3A-INTERACTING PROTEIN
21:00	5jvd-E	6.4	4.3	104	122	3	MOLECULE:	TUBULIN ALPHA-1B CHAIN
22:00	4a7h-B	6.4	6.3	130	136	7	MOLECULE:	ACTIN, ALPHA SKELETAL MUSCLE
23:00	5zvk-C	6.4	4.1	70	70	6	MOLECULE:	HUMAN CORONAVIRUS MERS HR1 MOTIF
24:00:00	8a0l-E	6.4	4.4	104	123	3	MOLECULE:	TUBULIN ALPHA-1B CHAIN
25:00:00	5wst-A	6.4	4.2	71	71	4	MOLECULE:	UNCONVENTIONAL MYOSIN-VIIA
26:00:00	5zvk-B	6.4	4.2	70	70	6	MOLECULE:	HUMAN CORONAVIRUS MERS HR1 MOTIF
27:00:00	7db9-E	6.4	4.5	105	124	3	MOLECULE:	TUBULIN ALPHA-1B CHAIN
28:00:00	7dbb-E	6.4	1.6	100	123	3	MOLECULE:	TUBULIN ALPHA-1B CHAIN
29:00:00	6znn-S	6.4	3.3	68	68	0	MOLECULE:	ARP1 ACTIN RELATED PROTEIN 1 HOMOLOG A
30:00:00	4bl6-A	6.4	4.6	82	82	7	MOLECULE:	PROTEIN BICAUDAL D
31:00:00	1nkn-A	6.4	4.7	73	74	4	MOLECULE:	S2N51-GCN4
32:00:00	1sfc-K	6.4	1.7	73	73	5	MOLECULE:	PROTEIN (SYNAPTOBREVIN 2)
33:00:00	1jth-C	6.4	2.4	69	69	6	MOLECULE:	SNAP25
34:00:00	6gbo-F	6.4	4.8	73	73	0	MOLECULE:	POLYMERASE COFACTOR VP35
35:00:00	2n1t-C	6.4	3.8	75	77	7	MOLECULE:	VESICLE-ASSOCIATED MEMBRANE PROTEIN 2
36:00:00	7bv6-G	6.4	3.8	74	74	5	MOLECULE:	VESICLE-ASSOCIATED MEMBRANE PROTEIN 8
37:00:00	4mvd-H	6.4	6.5	99	253	10	MOLECULE:	CHOLINE-PHOSPHATE CYTIDYLYLTRANSFERASE A
38:00:00	6vq6-O	6.4	8.1	113	114	12	MOLECULE:	ATPASE H+-TRANSPORTING V1 SUBUNIT A
39:00:00	5ydk-A	6.4	4.2	80	81	9	MOLECULE:	E3 UBIQUITIN-PROTEIN LIGASE RNF168
40:00:00	7y5c-b	6.4	9	134	145	6	MOLECULE:	ATP SYNTHASE SUBUNIT ALPHA
41:00:00	7znk-o	6.4	8.4	134	164	8	MOLECULE:	RNA
42:00:00	6pqv-Y	6.4	5.6	129	156	9	MOLECULE:	ATP SYNTHASE SUBUNIT DELTA
43:00:00	7y5b-b	6.4	10.3	140	145	6	MOLECULE:	ATP SYNTHASE SUBUNIT ALPHA
44:00:00	5fiy-D	6.4	3.7	77	77	10	MOLECULE:	PRKC APOPTOSIS WT1 REGULATOR PROTEIN
45:00:00	6qum-L	6.4	4.1	81	186	7	MOLECULE:	V-TYPE ATP SYNTHASE ALPHA CHAIN
46:00:00	7tkb-U	6.4	7.2	139	155	4	MOLECULE:	ATP SYNTHASE SUBUNIT 9, MITOCHONDRIAL
47:00:00	5whf-H	6.4	3.2	84	84	5	MOLECULE:	VIMENTIN
48:00:00	4jpn-B	6.4	3.6	74	74	9	MOLECULE:	MINOR SPIKE PROTEIN H
49:00:00	3swk-B	6.4	2.6	85	85	6	MOLECULE:	VIMENTIN
50:00:00	7tk2-U	6.4	7.7	138	155	4	MOLECULE:	ATP SYNTHASE SUBUNIT 9, MITOCHONDRIAL
51:00:00	4jpn-A	6.4	3.6	74	75	8	MOLECULE:	MINOR SPIKE PROTEIN H
52:00:00	8sde-A	6.4	4	87	89	8	MOLECULE:	TRIPARTITE MOTIF-CONTAINING PROTEIN 29
53:00:00	7tkg-U	6.4	11.1	145	155	7	MOLECULE:	ATP SYNTHASE SUBUNIT 9

54:00:00	7jg8-b	6.4	9.3	129	138	9	MOLECULE:	ATP SYNTHASE SUBUNIT ALPHA
55:00:00	6y5k-B	6.4	3.3	87	133	9	MOLECULE:	X-31 INFLUENZA HAEMAGGLUTININ HA1
56:00:00	6y5k-D	6.4	3.3	87	133	9	MOLECULE:	X-31 INFLUENZA HAEMAGGLUTININ HA1
57:00:00	6y5k-F	6.4	3.3	87	133	7	MOLECULE:	X-31 INFLUENZA HAEMAGGLUTININ HA1
58:00:00	4ll8-B	6.4	4.2	112	113	10	MOLECULE:	MYOSIN-4
59:00:00	5d3a-B	6.4	4.3	82	82	4	MOLECULE:	KINESIN-LIKE PROTEIN KIF21A
60:00:00	8afh-O	6.4	2.4	79	79	9	MOLECULE:	CRESCENTIN
61:00:00	8j0s-b	6.4	6.5	127	144	6	MOLECULE:	ATP SYNTHASE SUBUNIT A
62:00:00	2e7s-Q	6.4	2.3	111	111	3	MOLECULE:	RAB GUANINE NUCLEOTIDE EXCHANGE FACTOR SEC2
63:00:00	6oqv-X	6.4	9.1	132	156	10	MOLECULE:	ATP SYNTHASE SUBUNIT DELTA
64:00:00	2e7s-A	6.4	2.2	113	114	7	MOLECULE:	RAB GUANINE NUCLEOTIDE EXCHANGE FACTOR SEC2
65:00:00	2e7s-M	6.4	3.4	117	117	6	MOLECULE:	RAB GUANINE NUCLEOTIDE EXCHANGE FACTOR SEC2
66:00:00	6vmg-J	6.4	5.9	112	131	6	MOLECULE:	ATP SYNTHASE SUBUNIT A, CHLOROPLASTIC
67:00:00	6fkj-E	6.3	1.9	100	122	2	MOLECULE:	TUBULIN ALPHA-1B CHAIN
68:00:00	5s4v-E	6.3	1.6	100	123	3	MOLECULE:	TUBULIN ALPHA-1B CHAIN
69:00:00	7cbz-E	6.3	2.1	100	123	3	MOLECULE:	TUBULIN ALPHA-1B CHAIN
70:00:00	7yhn-E	6.3	1.7	100	123	2	MOLECULE:	TUBULIN ALPHA-1B CHAIN
71:00:00	5d80-G	6.3	5.2	103	223	7	MOLECULE:	V-TYPE PROTON ATPASE CATALYTIC SUBUNIT A
72:00:00	7dae-E	6.3	1.9	101	124	2	MOLECULE:	TUBULIN ALPHA-1B CHAIN
73:00:00	7xqx-E	6.3	2	100	123	5	MOLECULE:	TUBULIN ALPHA-1B CHAIN
74:00:00	6a9p-C	6.3	5.3	104	104	9	MOLECULE:	GLIAL FIBRILLARY ACIDIC PROTEIN
75:00:00	6y6d-E	6.3	4.3	104	122	3	MOLECULE:	TUBULIN ALPHA-1B CHAIN
76:00:00	5s4t-E	6.3	4.4	104	123	3	MOLECULE:	TUBULIN ALPHA-1B CHAIN
77:00:00	5s5g-E	6.3	4.4	104	123	3	MOLECULE:	TUBULIN ALPHA-1B CHAIN
78:00:00	6fkl-E	6.3	1.6	100	122	3	MOLECULE:	TUBULIN ALPHA-1B CHAIN
79:00:00	5ov7-E	6.3	2	99	123	5	MOLECULE:	TUBULIN ALPHA-1B CHAIN
80:00:00	7z2p-E	6.3	2	100	123	5	MOLECULE:	TUBULIN ALPHA-1B CHAIN
81:00:00	5sb5-E	6.3	2	100	123	5	MOLECULE:	TUBULIN ALPHA-1B CHAIN
82:00:00	5vpc-B	6.3	4.2	68	68	10	MOLECULE:	PROTEIN FOSB
83:00:00	7ucc-J	6.3	4.3	68	68	10	MOLECULE:	PROTEIN FOSB
84:00:00	7emj-E	6.3	4.4	104	123	3	MOLECULE:	TUBULIN ALPHA-1B CHAIN
85:00:00	8c0f-E	6.3	2	100	122	5	MOLECULE:	TUBULIN ALPHA-1B CHAIN
86:00:00	7vmk-E	6.3	4.4	104	123	3	MOLECULE:	TUBULIN ALPHA-1B CHAIN
87:00:00	6n6s-D	6.3	3.6	69	69	12	MOLECULE:	TNFAIP3-INTERACTING PROTEIN 1
88:00:00	3cve-A	6.3	4.3	68	68	7	MOLECULE:	HOMER PROTEIN HOMOLOG 1
89:00:00	5sb8-E	6.3	4.4	104	123	3	MOLECULE:	TUBULIN ALPHA-1B CHAIN
90:00:00	7xr1-E	6.3	4.4	104	123	3	MOLECULE:	TUBULIN ALPHA-1B CHAIN
91:00:00	5wq4-C	6.3	4.5	72	72	8	MOLECULE:	UBIQUITIN
92:00:00	5nnv-A	6.3	5.9	119	233	8	MOLECULE:	CHROMOSOME PARTITION PROTEIN SMC,CHROMOSOME
93:00:00	1gk4-C	6.3	3	70	70	0	MOLECULE:	VIMENTIN
94:00:00	6ltj-I	6.3	21.4	118	551	7	MOLECULE:	HISTONE H3.3
95:00:00	6gbo-L	6.3	5	72	72	0	MOLECULE:	POLYMERASE COFACTOR VP35
96:00:00	7bv6-W	6.3	4.3	72	72	7	MOLECULE:	VESICLE-ASSOCIATED MEMBRANE PROTEIN 8
97:00:00	6gbo-B	6.3	5.2	73	73	4	MOLECULE:	POLYMERASE COFACTOR VP35
98:00:00	5vpe-A	6.3	2.9	67	67	7	MOLECULE:	PROTEIN FOSB
99:00:00	8b8d-D	6.3	4.7	103	103	7	MOLECULE:	PHOSPHOPROTEIN
0:00	6y4n-E	6.3	3.9	104	123	6	MOLECULE:	TUBULIN ALPHA-1B CHAIN
1:00	5s5x-E	6.3	3.5	107	123	11	MOLECULE:	TUBULIN ALPHA-1B CHAIN
2:00	5s5q-E	6.3	3.5	106	123	11	MOLECULE:	TUBULIN ALPHA-1B CHAIN
3:00	5s51-E	6.3	3.6	109	123	12	MOLECULE:	TUBULIN ALPHA-1B CHAIN
4:00	5s4y-E	6.3	3.5	106	123	11	MOLECULE:	TUBULIN ALPHA-1B CHAIN

5:00	5s4p-E	6.3	3.5	106	123	11	MOLECULE:	TUBULIN ALPHA-1B CHAIN
6:00	5s4u-E	6.3	3.3	107	123	12	MOLECULE:	TUBULIN ALPHA-1B CHAIN
7:00	5s67-E	6.3	3.4	107	123	12	MOLECULE:	TUBULIN ALPHA-1B CHAIN
8:00	5s5t-E	6.3	3.4	108	123	12	MOLECULE:	TUBULIN ALPHA-1B CHAIN
9:00	5s4l-E	6.3	3.4	106	123	11	MOLECULE:	TUBULIN ALPHA-1B CHAIN
10:00	5s55-E	6.3	3.5	106	123	11	MOLECULE:	TUBULIN ALPHA-1B CHAIN
11:00	6tiu-E	6.3	3.9	100	127	8	MOLECULE:	TUBULIN ALPHA-1 CHAIN
12:00	5hzp-A	6.3	4.4	71	71	8	MOLECULE:	M PROTEIN, SEROTYPE 49
13:00	3cvf-C	6.3	1.6	74	74	12	MOLECULE:	HOMER PROTEIN HOMOLOG 3
14:00	6gbp-F	6.3	5	73	73	0	MOLECULE:	POLYMERASE COFACTOR VP35
15:00	6hk9-A	6.3	4.9	74	74	7	MOLECULE:	TESTIS-EXPRESSED PROTEIN 12
16:00	6r0y-L	6.3	4.6	81	186	7	MOLECULE:	V-TYPE ATP SYNTHASE ALPHA CHAIN
17:00	4jpn-G	6.3	3.5	74	75	8	MOLECULE:	MINOR SPIKE PROTEIN H
18:00	5mq4-B	6.3	2.8	74	111	8	MOLECULE:	PROTEIN KINASE C-BINDING PROTEIN 1
19:00	4jpn-F	6.3	3.4	72	72	8	MOLECULE:	MINOR SPIKE PROTEIN H
20:00	7tkc-U	6.3	7.7	140	155	4	MOLECULE:	ATP SYNTHASE SUBUNIT 9, MITOCHONDRIAL
21:00	3swk-A	6.3	4	85	86	5	MOLECULE:	VIMENTIN
22:00	4lhy-E	6.3	3.8	76	76	9	MOLECULE:	RAS-RELATED PROTEIN RAB-8A
23:00	4lhz-E	6.3	3.9	76	76	9	MOLECULE:	RAS-RELATED PROTEIN RAB-8A
24:00:00	7tkf-U	6.3	12.4	147	155	9	MOLECULE:	ATP SYNTHASE SUBUNIT 9
25:00:00	5nfz-E	6.3	4	102	125	9	MOLECULE:	TUBULIN ALPHA-1B CHAIN
26:00:00	7jg6-b	6.3	9	129	138	9	MOLECULE:	ATP SYNTHASE SUBUNIT ALPHA
27:00:00	6ses-E	6.3	4.1	100	123	8	MOLECULE:	TUBULIN ALPHA-1B CHAIN
28:00:00	6qlf-U	6.3	14.6	121	171	7	MOLECULE:	INNER KINETOCHORE SUBUNIT IML3
29:00:00	6vq7-N	6.3	8.7	113	114	7	MOLECULE:	ATPASE H+-TRANSPORTING V1 SUBUNIT A
30:00:00	2msd-C	6.3	44.7	116	198	4	MOLECULE:	APOLIPOPROTEIN A-I
31:00:00	6zay-D	6.3	3.6	85	86	7	MOLECULE:	RAS-RELATED PROTEIN RAB-33B
32:00:00	6wnr-X	6.3	9.3	135	153	5	MOLECULE:	ATP SYNTHASE SUBUNIT DELTA
33:00:00	6lk8-M	6.3	4.6	73	73	0	MOLECULE:	MGC83295 PROTEIN
34:00:00	4c47-A	6.2	8.3	81	191	10	MOLECULE:	INNER MEMBRANE LIPOPROTEIN
35:00:00	7au5-E	6.2	1.8	100	124	2	MOLECULE:	TUBULIN ALPHA-1B CHAIN
36:00:00	6th4-E	6.2	6.2	103	128	2	MOLECULE:	TUBULIN ALPHA CHAIN
37:00:00	5s5o-E	6.2	1.9	100	123	2	MOLECULE:	TUBULIN ALPHA-1B CHAIN
38:00:00	5s4x-E	6.2	1.9	100	123	2	MOLECULE:	TUBULIN ALPHA-1B CHAIN
39:00:00	7e4z-E	6.2	1.8	99	123	2	MOLECULE:	TUBULIN ALPHA-1B CHAIN
40:00:00	5s58-E	6.2	1.7	99	123	2	MOLECULE:	TUBULIN ALPHA-1B CHAIN
41:00:00	5sb4-E	6.2	1.8	99	123	2	MOLECULE:	TUBULIN ALPHA-1B CHAIN
42:00:00	5s62-E	6.2	1.9	100	123	2	MOLECULE:	TUBULIN ALPHA-1B CHAIN
43:00:00	5s5e-E	6.2	1.9	100	123	2	MOLECULE:	TUBULIN ALPHA-1B CHAIN
44:00:00	5s5j-E	6.2	1.9	100	123	2	MOLECULE:	TUBULIN ALPHA-1B CHAIN
45:00:00	5s5h-E	6.2	1.8	99	123	2	MOLECULE:	TUBULIN ALPHA-1B CHAIN
46:00:00	5sbd-E	6.2	2.6	98	121	6	MOLECULE:	TUBULIN ALPHA-1B CHAIN
47:00:00	5s4r-E	6.2	1.8	99	123	2	MOLECULE:	TUBULIN ALPHA-1B CHAIN
48:00:00	7daf-E	6.2	1.9	100	123	2	MOLECULE:	TUBULIN ALPHA-1B CHAIN
49:00:00	5s4q-E	6.2	1.8	100	123	2	MOLECULE:	TUBULIN ALPHA-1B CHAIN
50:00:00	5s4w-E	6.2	1.8	100	123	2	MOLECULE:	TUBULIN ALPHA-1B CHAIN
51:00:00	7lz7-E	6.2	1.7	98	121	3	MOLECULE:	TUBULIN ALPHA-1B CHAIN
52:00:00	8dbs-Y	6.2	11.8	130	156	4	MOLECULE:	ATP SYNTHASE SUBUNIT ALPHA
53:00:00	7cnn-E	6.2	4.5	103	122	3	MOLECULE:	TUBULIN ALPHA-1B CHAIN
54:00:00	7ogn-E	6.2	4.4	102	120	4	MOLECULE:	TUBULIN ALPHA-1B CHAIN
55:00:00	6fjm-E	6.2	1.8	99	123	2	MOLECULE:	TUBULIN ALPHA-1B CHAIN

56:00:00	5xkg-E	6.2	2	98	121	5	MOLECULE:	TUBULIN ALPHA-1B CHAIN
57:00:00	7e4y-E	6.2	2.7	100	123	7	MOLECULE:	TUBULIN ALPHA-1B CHAIN
58:00:00	6v8o-J	6.2	8	86	115	3	MOLECULE:	HIGH TEMPERATURE LETHAL PROTEIN 1
59:00:00	6kw3-W	6.2	3.9	69	69	6	MOLECULE:	HISTONE H4
60:00:00	3cve-B	6.2	4.1	66	66	8	MOLECULE:	HOMER PROTEIN HOMOLOG 1
61:00:00	6n6r-D	6.2	3.4	67	67	12	MOLECULE:	UBIQUITIN
62:00:00	5sba-E	6.2	1.6	98	121	3	MOLECULE:	TUBULIN ALPHA-1B CHAIN
63:00:00	5sbb-E	6.2	1.6	97	120	2	MOLECULE:	TUBULIN ALPHA-1B CHAIN
64:00:00	5sbc-E	6.2	4.4	102	121	3	MOLECULE:	TUBULIN ALPHA-1B CHAIN
65:00:00	8amr-A	6.2	3.7	79	155	5	MOLECULE:	TRIPARTITE MOTIF-CONTAINING PROTEIN 3
66:00:00	6wvw-F	6.2	2.1	66	66	6	MOLECULE:	VESICLE-ASSOCIATED MEMBRANE PROTEIN 2
67:00:00	7bv6-S	6.2	4.2	74	74	9	MOLECULE:	VESICLE-ASSOCIATED MEMBRANE PROTEIN 8
68:00:00	6v86-C	6.2	3.9	74	74	8	MOLECULE:	RNA-DIRECTED RNA POLYMERASE L
69:00:00	7rsc-D	6.2	30.1	138	187	10	MOLECULE:	GTPASE KRAS
70:00:00	1jth-B	6.2	2.7	67	67	4	MOLECULE:	SNAP25
71:00:00	1urq-C	6.2	1.6	68	68	6	MOLECULE:	M-TOMOSYN ISOFORM
72:00:00	5vpd-B	6.2	4.2	67	67	10	MOLECULE:	PROTEIN FOSB
73:00:00	1gk4-E	6.2	2.8	69	70	0	MOLECULE:	VIMENTIN
74:00:00	1sfc-B	6.2	3.6	72	72	3	MOLECULE:	PROTEIN (SYNAPTOBREVIN 2)
75:00:00	1sfc-G	6.2	2	72	72	6	MOLECULE:	PROTEIN (SYNAPTOBREVIN 2)
76:00:00	1gk4-F	6.2	3.4	74	74	0	MOLECULE:	VIMENTIN
77:00:00	6cxi-T	6.2	5.1	125	127	0	MOLECULE:	ACTIN, CYTOPLASMIC 2
78:00:00	6cxj-T	6.2	5	125	127	0	MOLECULE:	ACTIN, CYTOPLASMIC 2
79:00:00	8pqw-F	6.2	14.3	138	150	9	MOLECULE:	CYTOPLASMIC DYNEIN 1 HEAVY CHAIN 1
80:00:00	3b5n-G	6.2	1.7	69	69	6	MOLECULE:	SYNAPTOBREVIN HOMOLOG 1
81:00:00	7c7y-C	6.2	2.5	67	67	4	MOLECULE:	UNCHARACTERIZED PROTEIN
82:00:00	5s5k-E	6.2	3.4	106	123	11	MOLECULE:	TUBULIN ALPHA-1B CHAIN
83:00:00	5s64-E	6.2	3.5	106	123	11	MOLECULE:	TUBULIN ALPHA-1B CHAIN
84:00:00	5vpb-D	6.2	4	66	66	11	MOLECULE:	PROTEIN FOSB
85:00:00	5s59-E	6.2	3.5	106	123	11	MOLECULE:	TUBULIN ALPHA-1B CHAIN
86:00:00	7yg4-E	6.2	4	67	67	7	MOLECULE:	PROTEIN VIRILIZER HOMOLOG
87:00:00	5s4n-E	6.2	3.3	106	123	12	MOLECULE:	TUBULIN ALPHA-1B CHAIN
88:00:00	5vpe-D	6.2	2.9	67	67	12	MOLECULE:	PROTEIN FOSB
89:00:00	6v85-E	6.2	4.8	74	74	4	MOLECULE:	RNA-DIRECTED RNA POLYMERASE L
90:00:00	6vag-A	6.2	4.8	74	74	4	MOLECULE:	PHOSPHOPROTEIN
91:00:00	6vqj-O	6.2	4	67	67	1	MOLECULE:	V-TYPE PROTON ATPASE SUBUNIT C 1
92:00:00	3cvf-D	6.2	2.3	71	72	14	MOLECULE:	HOMER PROTEIN HOMOLOG 3
93:00:00	4l2w-A	6.2	2.1	67	67	4	MOLECULE:	RHO-ASSOCIATED PROTEIN KINASE 1
94:00:00	6kpp-E	6.2	3.9	107	123	11	MOLECULE:	TUBULIN ALPHA-1B CHAIN
95:00:00	4l2w-D	6.2	1.6	68	68	7	MOLECULE:	RHO-ASSOCIATED PROTEIN KINASE 1
96:00:00	6xx0-B	6.2	4.5	75	75	9	MOLECULE:	INHIBITOR OF KAPPA LIGHT POLYPEPTIDE GENE EN
97:00:00	7njo-b	6.2	9.2	132	141	5	MOLECULE:	ATP SYNTHASE SUBUNIT ALPHA
98:00:00	6ff7-J	6.2	3.8	77	135	9	MOLECULE:	RNA-BINDING MOTIF PROTEIN, X-LINKED 2
99:00:00	6r10-K	6.2	3.8	98	98	7	MOLECULE:	V-TYPE ATP SYNTHASE ALPHA CHAIN
0:00	6vq6-N	6.2	8.1	113	114	9	MOLECULE:	ATPASE H+-TRANSPORTING V1 SUBUNIT A
1:00	6vq8-M	6.2	9.5	114	114	5	MOLECULE:	ATPASE H+-TRANSPORTING V1 SUBUNIT A
2:00	6vq7-M	6.2	8.7	113	114	9	MOLECULE:	ATPASE H+-TRANSPORTING V1 SUBUNIT A
3:00	3uf1-C	6.2	5.4	107	109	5	MOLECULE:	VIMENTIN
4:00	5hmo-C	6.2	5.6	111	122	11	MOLECULE:	UNCONVENTIONAL MYOSIN-X
5:00	7ttf-E	6.2	5	113	123	12	MOLECULE:	TUBULIN ALPHA-1B CHAIN
6:00	7y5d-b	6.2	7.1	130	145	8	MOLECULE:	ATP SYNTHASE SUBUNIT A

7:00	2e7s-L	6.2	3.1	114	114	3	MOLECULE:	RAB GUANINE NUCLEOTIDE EXCHANGE FACTOR SEC2
8:00	5t4q-J	6.2	9.5	148	155	10	MOLECULE:	ATP SYNTHASE SUBUNIT ALPHA
9:00	7jts-s	6.1	18.4	104	290	7	MOLECULE:	RADIAL SPOKE PROTEIN 3
10:00	8q85-b	6.1	2.7	68	70	6	MOLECULE:	KINETOCHORE PROTEIN NDC80
11:00	6eun-A	6.1	3.8	112	147	4	MOLECULE:	ADHESIN
12:00	8q85-G	6.1	7	126	178	6	MOLECULE:	KINETOCHORE PROTEIN NDC80
13:00	5bmv-E	6.1	2.1	99	123	2	MOLECULE:	TUBULIN ALPHA-1B CHAIN
14:00	4zhq-E	6.1	1.9	99	123	2	MOLECULE:	TUBULIN ALPHA-1B CHAIN
15:00	5s5n-E	6.1	1.9	100	122	2	MOLECULE:	TUBULIN ALPHA-1B CHAIN
16:00	6xes-E	6.1	5.9	101	125	3	MOLECULE:	TUBULIN ALPHA-1B CHAIN
17:00	5s4s-E	6.1	1.7	97	120	2	MOLECULE:	TUBULIN ALPHA-1B CHAIN
18:00	6br1-E	6.1	2	98	121	2	MOLECULE:	TUBULIN ALPHA-1B CHAIN
19:00	8ahl-D	6.1	6.6	145	234	6	MOLECULE:	CRESCENTIN
20:00	7cda-E	6.1	4.5	102	121	3	MOLECULE:	TUBULIN ALPHA-1B CHAIN
21:00	6nng-E	6.1	2	98	121	2	MOLECULE:	TUBULIN ALPHA-1B CHAIN
22:00	8ajb-D	6.1	9.4	154	234	9	MOLECULE:	CRESCENTIN
23:00	8ajb-P	6.1	6.5	146	234	12	MOLECULE:	CRESCENTIN
24:00:00	6h9b-E	6.1	6.4	110	136	4	MOLECULE:	TUBULIN ALPHA CHAIN
25:00:00	6s9e-E	6.1	1.9	98	120	2	MOLECULE:	TUBULIN ALPHA-1B CHAIN
26:00:00	4zi7-E	6.1	2	100	123	2	MOLECULE:	TUBULIN ALPHA-1B CHAIN
27:00:00	5h7o-E	6.1	2.1	98	121	2	MOLECULE:	TUBULIN ALPHA-1B CHAIN
28:00:00	6k9v-E	6.1	2	98	121	2	MOLECULE:	TUBULIN ALPHA-1B CHAIN
29:00:00	6y4m-E	6.1	2.2	100	123	2	MOLECULE:	TUBULIN ALPHA-1B CHAIN
30:00:00	8ahl-B	6.1	8.4	154	238	6	MOLECULE:	CRESCENTIN
31:00:00	5kx5-E	6.1	4.5	102	121	3	MOLECULE:	TUBULIN ALPHA CHAIN
32:00:00	6d88-E	6.1	4.5	102	121	3	MOLECULE:	TUBULIN ALPHA-1B CHAIN
33:00:00	8ajb-N	6.1	8.4	154	238	6	MOLECULE:	CRESCENTIN
34:00:00	5yl4-E	6.1	1.7	98	120	3	MOLECULE:	TUBULIN ALPHA CHAIN
35:00:00	8asn-E	6.1	2.3	101	123	2	MOLECULE:	TUBULIN ALPHA-1B CHAIN
36:00:00	7en3-E	6.1	2	98	121	5	MOLECULE:	TUBULIN ALPHA-1B CHAIN
37:00:00	6wm2-H	6.1	13.2	100	225	6	MOLECULE:	V-TYPE PROTON ATPASE SUBUNIT E 1
38:00:00	8clc-E	6.1	1.7	100	123	3	MOLECULE:	TUBULIN ALPHA-1B CHAIN
39:00:00	5wjb-C	6.1	3.7	97	112	4	MOLECULE:	CAPSID ASSEMBLY SCAFFOLDING PROTEIN,MYOSIN-7
40:00:00	7bjs-A	6.1	4.6	69	69	6	MOLECULE:	KINESIN HEAVY CHAIN
41:00:00	1wt6-B	6.1	3.9	67	67	7	MOLECULE:	MYOTONIN-PROTEIN KINASE
42:00:00	7c7y-A	6.1	4.7	66	66	6	MOLECULE:	UNCHARACTERIZED PROTEIN
43:00:00	8b8b-B	6.1	3	95	95	12	MOLECULE:	MUNIA BORNAVIRUS 1 PHOSPHOPROTEIN
44:00:00	7say-D	6.1	3.2	67	67	6	MOLECULE:	GENERAL CONTROL TRANSCRIPTION FACTOR GCN4/M
45:00:00	5sb9-E	6.1	4.4	102	123	3	MOLECULE:	TUBULIN ALPHA-1B CHAIN
46:00:00	7bv6-O	6.1	4.3	73	73	8	MOLECULE:	VESICLE-ASSOCIATED MEMBRANE PROTEIN 8
47:00:00	1sfc-F	6.1	4.7	73	73	4	MOLECULE:	PROTEIN (SYNAPTOBREVIN 2)
48:00:00	6g2t-P	6.1	4.9	129	135	0	MOLECULE:	ACTIN, CYTOPLASMIC 2
49:00:00	3gn4-A	6.1	4.5	89	129	3	MOLECULE:	MYOSIN-VI
50:00:00	3b5n-F	6.1	3.6	68	68	4	MOLECULE:	SYNAPTOBREVIN HOMOLOG 1
51:00:00	7bv6-K	6.1	4.4	73	73	7	MOLECULE:	VESICLE-ASSOCIATED MEMBRANE PROTEIN 8
52:00:00	7eqb-A	6.1	4.5	68	68	9	MOLECULE:	KINESIN-LIKE PROTEIN
53:00:00	6oqt-X	6.1	8.7	135	156	7	MOLECULE:	ATP SYNTHASE SUBUNIT DELTA
54:00:00	5bw9-i	6.1	9	126	217	7	MOLECULE:	V-TYPE PROTON ATPASE CATALYTIC SUBUNIT A
55:00:00	7e4r-E	6.1	6.8	119	121	5	MOLECULE:	TUBULIN ALPHA-1B CHAIN
56:00:00	5s66-E	6.1	3.3	105	123	11	MOLECULE:	TUBULIN ALPHA-1B CHAIN
57:00:00	5s52-E	6.1	3.3	106	123	12	MOLECULE:	TUBULIN ALPHA-1B CHAIN

58:00:00	4bwn-B	6.1	4.9	81	86	6	MOLECULE:	NF-KAPPA-B ESSENTIAL MODULATOR
59:00:00	5ylj-E	6.1	4.2	98	121	7	MOLECULE:	TUBULIN ALPHA-1B CHAIN
60:00:00	1sfc-C	6.1	4	77	77	5	MOLECULE:	PROTEIN (SYNAPTOBREVIN 2)
61:00:00	6gbq-A	6.1	4.3	70	70	3	MOLECULE:	POLYMERASE COFACTOR VP35
62:00:00	3lj5-C	6.1	8	131	692	8	MOLECULE:	PORTAL PROTEIN
63:00:00	3lj5-F	6.1	8	131	692	8	MOLECULE:	PORTAL PROTEIN
64:00:00	5gai-G	6.1	11.5	130	721	7	MOLECULE:	PORTAL PROTEIN
65:00:00	7syg-H	6.1	7.4	72	237	8	MOLECULE:	18S RRNA
66:00:00	6gbp-L	6.1	5.5	73	73	4	MOLECULE:	POLYMERASE COFACTOR VP35
67:00:00	8fby-A	6.1	3.5	83	89	6	MOLECULE:	PLASMALEMMA VESICLE-ASSOCIATED PROTEIN
68:00:00	7njl-b	6.1	9.3	131	145	5	MOLECULE:	ATP SYNTHASE SUBUNIT ALPHA
69:00:00	7tkj-U	6.1	12.5	146	155	9	MOLECULE:	ATP SYNTHASE SUBUNIT 9
70:00:00	7tmr-H	6.1	8.7	112	112	5	MOLECULE:	H(+)-TRANSPORTING TWO-SECTOR ATPASE
71:00:00	7njn-b	6.1	9.3	132	145	5	MOLECULE:	ATP SYNTHASE SUBUNIT ALPHA
72:00:00	5gon-E	6.1	2	98	121	5	MOLECULE:	TUBULIN ALPHA-1B CHAIN
73:00:00	7l05-E	6.1	5.8	111	121	8	MOLECULE:	TUBULIN ALPHA-1B CHAIN
74:00:00	3v6i-X	6.1	4.4	99	99	6	MOLECULE:	V-TYPE ATP SYNTHASE SUBUNIT E
75:00:00	6pc4-E	6.1	4.1	98	121	7	MOLECULE:	TUBULIN ALPHA-1B CHAIN
76:00:00	6vq8-O	6.1	8.6	112	113	9	MOLECULE:	ATPASE H+-TRANSPORTING V1 SUBUNIT A
77:00:00	5lov-E	6.1	1.9	97	117	5	MOLECULE:	TUBULIN ALPHA-1B CHAIN
78:00:00	4lhy-C	6.1	4.2	72	72	6	MOLECULE:	RAS-RELATED PROTEIN RAB-8A
79:00:00	4cpc-D	6.1	5.1	112	141	8	MOLECULE:	SYNAPTONEMAL COMPLEX PROTEIN 3
80:00:00	5vpe-C	6.1	2.6	66	66	8	MOLECULE:	PROTEIN FOSB
81:00:00	6eb8-E	6.1	3.9	75	103	8	MOLECULE:	PHOSPHOPROTEIN
82:00:00	2e7s-S	6.1	4.1	116	117	7	MOLECULE:	RAB GUANINE NUCLEOTIDE EXCHANGE FACTOR SEC2
83:00:00	2e7s-R	6.1	2.9	111	111	8	MOLECULE:	RAB GUANINE NUCLEOTIDE EXCHANGE FACTOR SEC2
84:00:00	4xa6-A	6	10.5	129	168	9	MOLECULE:	GP7-MYH7(1777-1855)-EB1 CHIMERA PROTEIN
85:00:00	8bd7-M	6	17.7	131	164	8	MOLECULE:	IFT88
86:00:00	6oei-A	6	10.8	100	243	8	MOLECULE:	SPINDLE POLE BODY COMPONENT SPC42,SIGMA-54-D
87:00:00	8clf-E	6	2	98	121	2	MOLECULE:	TUBULIN ALPHA-1B CHAIN
88:00:00	5xke-E	6	2	98	121	2	MOLECULE:	TUBULIN ALPHA-1B CHAIN
89:00:00	7s5u-A	6	6.3	139	179	7	MOLECULE:	KINESIN-LIKE PROTEIN KLP61F
90:00:00	4zol-E	6	2.3	99	122	2	MOLECULE:	TUBULIN ALPHA-1B CHAIN
91:00:00	8cld-E	6	2	98	121	2	MOLECULE:	DETYROSINATED TUBULIN ALPHA-1B CHAIN
92:00:00	6o61-E	6	2.1	97	121	2	MOLECULE:	TUBULIN ALPHA-1B CHAIN
93:00:00	8i03-G	6	7.7	112	166	8	MOLECULE:	PAIRED AMPHIPATHIC HELIX PROTEIN PST1
94:00:00	8tek-N	6	7.2	144	187	10	MOLECULE:	DYNEIN REGULATORY COMPLEX PROTEIN 1/2 N-TERM
95:00:00	6gze-E	6	4.3	100	116	2	MOLECULE:	TUBULIN ALPHA-1B CHAIN
96:00:00	6jcj-E	6	4.5	102	120	3	MOLECULE:	TUBULIN ALPHA-1B CHAIN
97:00:00	5xaf-E	6	2	98	121	5	MOLECULE:	TUBULIN ALPHA-1B CHAIN
98:00:00	6bs2-E	6	2	98	121	2	MOLECULE:	TUBULIN ALPHA-1B CHAIN
99:00:00	5wq4-E	6	4.7	69	69	4	MOLECULE:	UBIQUITIN
0:00	6uci-D	6	4	67	67	6	MOLECULE:	PROTEIN FOSB
1:00	7c7y-B	6	4.3	66	66	6	MOLECULE:	UNCHARACTERIZED PROTEIN
2:00	3cve-C	6	4.1	66	66	8	MOLECULE:	HOMER PROTEIN HOMOLOG 1
3:00	7yer-E	6	4.6	67	67	3	MOLECULE:	RNA-DIRECTED RNA POLYMERASE L
4:00	6lk8-K	6	3.8	68	69	0	MOLECULE:	MGC83295 PROTEIN
5:00	5wwl-N	6	5.9	73	155	7	MOLECULE:	CENTROMERE PROTEIN MIS12
6:00	7yes-E	6	4.7	67	67	3	MOLECULE:	RNA-DIRECTED RNA POLYMERASE L
7:00	6iak-F	6	3.5	71	71	8	MOLECULE:	UNCHARACTERIZED PROTEIN
8:00	7bv6-C	6	3.6	72	72	8	MOLECULE:	VESICLE-ASSOCIATED MEMBRANE PROTEIN 8

9:00	1n7s-B	6	3.6	68	68	7	MOLECULE:	VESICLE-ASSOCIATED MEMBRANE PROTEIN 2
10:00	5eof-B	6	4.2	73	74	1	MOLECULE:	OPTINEURIN
11:00	5d80-J	6	6	104	104	8	MOLECULE:	V-TYPE PROTON ATPASE CATALYTIC SUBUNIT A
12:00	6ptw-C	6	35.8	150	198	9	MOLECULE:	APOLIPOPROTEIN A-I
13:00	3wmi-A	6	2.4	56	57	4	MOLECULE:	EIAV GP45 WILD TYPE
14:00	6v85-D	6	4.7	71	72	4	MOLECULE:	RNA-DIRECTED RNA POLYMERASE L
15:00	5bw9-m	6	10.6	94	181	4	MOLECULE:	V-TYPE PROTON ATPASE CATALYTIC SUBUNIT A
16:00	5toi-C	6	3.1	70	70	9	MOLECULE:	POLYMERASE COFACTOR VP35
17:00	5toh-B	6	4.8	70	70	9	MOLECULE:	POLYMERASE COFACTOR VP35
18:00	6v86-E	6	4.9	74	74	5	MOLECULE:	RNA-DIRECTED RNA POLYMERASE L
19:00	6vag-B	6	2.9	71	72	10	MOLECULE:	PHOSPHOPROTEIN
20:00	1gu5-B	6	3.1	67	67	16	MOLECULE:	CAAT/ENHANCER BINDING PROTEIN BETA
21:00	1gu4-B	6	3.2	67	67	16	MOLECULE:	CAAT/ENHANCER BINDING PROTEIN BETA
22:00	6qld-Q	6	18.6	102	204	5	MOLECULE:	INNER KINETOCHORE SUBUNIT MIF2
23:00	7c4j-H	6	22.4	132	242	8	MOLECULE:	TRANSCRIPTION REGULATORY PROTEIN SNF12
24:00:00	6gbp-B	6	5.4	73	73	4	MOLECULE:	POLYMERASE COFACTOR VP35
25:00:00	6n6s-B	6	2.5	65	65	17	MOLECULE:	TNFAIP3-INTERACTING PROTEIN 1
26:00:00	6v85-B	6	4.7	71	72	4	MOLECULE:	RNA-DIRECTED RNA POLYMERASE L
27:00:00	5whf-B	6	4.7	82	82	9	MOLECULE:	VIMENTIN
28:00:00	4l2w-C	6	3.3	67	67	6	MOLECULE:	RHO-ASSOCIATED PROTEIN KINASE 1
29:00:00	5wq4-D	6	4	72	72	7	MOLECULE:	UBIQUITIN
30:00:00	6gbq-C	6	3.9	69	69	4	MOLECULE:	POLYMERASE COFACTOR VP35
31:00:00	4bry-A	6	3.5	69	69	7	MOLECULE:	GEMININ
32:00:00	7a5p-J	6	8.6	80	110	6	MOLECULE:	U2 SNRNA
33:00:00	7njv-b	6	3.9	62	63	10	MOLECULE:	ATP SYNTHASE SUBUNIT C
34:00:00	6fki-p	6	7.7	132	143	7	MOLECULE:	ATP SYNTHASE SUBUNIT A, CHLOROPLASTIC
35:00:00	8b8b-A	6	2.9	99	99	6	MOLECULE:	MUNIA BORNAVIRUS 1 PHOSPHOPROTEIN
36:00:00	6bry-E	6	4.2	98	121	7	MOLECULE:	TUBULIN ALPHA-1B CHAIN
37:00:00	5bw9-j	6	4.8	101	101	2	MOLECULE:	V-TYPE PROTON ATPASE CATALYTIC SUBUNIT A
38:00:00	5mqf-J	6	3.5	74	135	8	MOLECULE:	PRE-MRNA-PROCESSING-SPLICING FACTOR 8
39:00:00	7njr-b	6	7.2	113	145	6	MOLECULE:	ATP SYNTHASE SUBUNIT ALPHA
40:00:00	6vm1-J	6	7.5	123	131	11	MOLECULE:	ATP SYNTHASE SUBUNIT ALPHA, CHLOROPLASTIC
41:00:00	7njq-b	6	7.7	113	144	5	MOLECULE:	ATP SYNTHASE SUBUNIT ALPHA
42:00:00	6j5i-b	5.9	13.4	145	209	7	MOLECULE:	ATP SYNTHASE SUBUNIT ALPHA, MITOCHONDRIAL
43:00:00	8fef-B	5.9	23.9	149	335	13	MOLECULE:	VIRULENCE FACTOR MCE FAMILY PROTEIN
44:00:00	7apk-m	5.9	6.6	121	549	4	MOLECULE:	THO COMPLEX SUBUNIT 1
45:00:00	5bw9-g	5.9	3.3	87	141	6	MOLECULE:	V-TYPE PROTON ATPASE CATALYTIC SUBUNIT A
46:00:00	7zyw-E	5.9	1.9	96	119	2	MOLECULE:	TUBULIN ALPHA-1B CHAIN
47:00:00	8clb-E	5.9	2.1	98	121	2	MOLECULE:	TUBULIN ALPHA-1B CHAIN
48:00:00	5nvu-A	5.9	10.3	146	3169	0	MOLECULE:	DYNEIN MOTOR DOMAIN
49:00:00	8wd0-E	5.9	1.9	97	121	2	MOLECULE:	TUBULIN ALPHA-1B CHAIN
50:00:00	8cle-E	5.9	2.1	97	120	5	MOLECULE:	TUBULIN ALPHA-1B CHAIN
51:00:00	6cch-C	5.9	33.5	144	198	4	MOLECULE:	APOLIPOPROTEIN A-I
52:00:00	7c7y-D	5.9	4	64	64	6	MOLECULE:	UNCHARACTERIZED PROTEIN
53:00:00	7aw9-E	5.9	2.1	65	65	9	MOLECULE:	HAPB
54:00:00	1jth-A	5.9	1.5	62	62	8	MOLECULE:	SNAP25
55:00:00	5bw9-L	5.9	4.8	68	68	9	MOLECULE:	V-TYPE PROTON ATPASE CATALYTIC SUBUNIT A
56:00:00	3b5n-K	5.9	2.9	65	65	5	MOLECULE:	SYNAPTOBREVIN HOMOLOG 1
57:00:00	6kw4-V	5.9	4.1	69	69	6	MOLECULE:	HISTONE H3.2
58:00:00	5vpf-D	5.9	3.7	65	65	12	MOLECULE:	PROTEIN FOSB
59:00:00	7upz-B	5.9	4.6	68	68	6	MOLECULE:	CCAAT/ENHANCER-BINDING PROTEIN BETA

60:00:00	1s1c-X	5.9	3.5	69	69	4	MOLECULE:	TRANSFORMING PROTEIN RHOA
61:00:00	6wvw-D	5.9	4.9	63	63	8	MOLECULE:	VESICLE-ASSOCIATED MEMBRANE PROTEIN 2
62:00:00	8rtd-B	5.9	7.6	73	197	10	MOLECULE:	TRWJ PROTEIN
63:00:00	5toi-B	5.9	5.1	70	70	9	MOLECULE:	POLYMERASE COFACTOR VP35
64:00:00	3cvf-B	5.9	1.3	70	70	6	MOLECULE:	HOMER PROTEIN HOMOLOG 3
65:00:00	6zr2-Z	5.9	13	95	141	8	MOLECULE:	NADH-UBIQUINONE OXIDOREDUCTASE CHAIN 3
66:00:00	7n84-Z	5.9	3.4	63	63	0	MOLECULE:	NUCLEOPORIN NUP188
67:00:00	7tmp-J	5.9	9	108	108	6	MOLECULE:	H(+)-TRANSPORTING TWO-SECTOR ATPASE
68:00:00	8xwx-D	5.9	4.8	65	65	8	MOLECULE:	B-CELL RECEPTOR-ASSOCIATED PROTEIN 31
69:00:00	7l4v-B	5.9	2.6	69	69	9	MOLECULE:	CCAAT/ENHANCER-BINDING PROTEIN BETA
70:00:00	1gtw-B	5.9	3	67	67	16	MOLECULE:	CAAT/ENHANCER BINDING PROTEIN BETA
71:00:00	5toi-A	5.9	5.1	68	68	6	MOLECULE:	POLYMERASE COFACTOR VP35
72:00:00	7bv6-P	5.9	2.1	65	65	12	MOLECULE:	VESICLE-ASSOCIATED MEMBRANE PROTEIN 8
73:00:00	5cch-D	5.9	4.1	64	64	6	MOLECULE:	VESICLE-ASSOCIATED MEMBRANE PROTEIN 2
74:00:00	6mti-D	5.9	4.1	64	64	5	MOLECULE:	SYNAPTOTAGMIN-1
75:00:00	7tmq-J	5.9	10.6	108	108	6	MOLECULE:	H(+)-TRANSPORTING TWO-SECTOR ATPASE
76:00:00	6hx8-E	5.9	4.2	100	123	8	MOLECULE:	TUBULIN ALPHA-1B CHAIN
77:00:00	6wm2-M	5.9	8.8	112	114	9	MOLECULE:	V-TYPE PROTON ATPASE SUBUNIT E 1
78:00:00	4lhx-C	5.9	4.7	72	72	4	MOLECULE:	RAS-RELATED PROTEIN RAB-8A
79:00:00	5bw9-J	5.9	5.1	103	104	4	MOLECULE:	V-TYPE PROTON ATPASE CATALYTIC SUBUNIT A
80:00:00	7qin-K	5.9	4.6	88	88	0	MOLECULE:	ACTIN, ALPHA SKELETAL MUSCLE
81:00:00	4lhz-C	5.9	4.8	72	72	4	MOLECULE:	RAS-RELATED PROTEIN RAB-8A
82:00:00	4ytd-A	5.9	3.4	95	96	13	MOLECULE:	PROTEIN BICAUDAL D HOMOLOG 1
83:00:00	6vmb-J	5.9	6.5	112	136	5	MOLECULE:	ATP SYNTHASE SUBUNIT ALPHA, CHLOROPLASTIC
84:00:00	8fed-A	5.8	20.1	134	392	7	MOLECULE:	VIRULENCE FACTOR MCE FAMILY PROTEIN
85:00:00	1joc-A	5.8	27.3	81	123	7	MOLECULE:	EARLY ENDOSOMAL AUTOANTIGEN 1
86:00:00	5jcb-E	5.8	2.8	96	121	3	MOLECULE:	TUBULIN ALPHA-1B CHAIN
87:00:00	1x79-B	5.8	4.1	90	90	6	MOLECULE:	ADP-RIBOSYLATION FACTOR BINDING PROTEIN GGA1
88:00:00	3tnu-B	5.8	2.1	95	95	6	MOLECULE:	KERATIN, TYPE I CYTOSKELETAL 14
89:00:00	7tmm-I	5.8	10.3	91	220	7	MOLECULE:	H(+)-TRANSPORTING TWO-SECTOR ATPASE
90:00:00	5vpc-D	5.8	4.6	63	63	3	MOLECULE:	PROTEIN FOSB
91:00:00	8k8d-A	5.8	2.1	68	68	3	MOLECULE:	CCAAT/ENHANCER-BINDING PROTEIN BETA
92:00:00	7bv6-T	5.8	4.6	67	67	6	MOLECULE:	VESICLE-ASSOCIATED MEMBRANE PROTEIN 8
93:00:00	3cve-D	5.8	2.7	61	61	7	MOLECULE:	HOMER PROTEIN HOMOLOG 1
94:00:00	1l2p-A	5.8	2.9	61	61	10	MOLECULE:	ATP SYNTHASE B CHAIN
95:00:00	7eao-B	5.8	4.2	63	63	5	MOLECULE:	POLYUBIQUITIN-C
96:00:00	7bv6-D	5.8	4.6	67	67	6	MOLECULE:	VESICLE-ASSOCIATED MEMBRANE PROTEIN 8
97:00:00	6vqi-I	5.8	4.1	64	64	5	MOLECULE:	V-TYPE PROTON ATPASE SUBUNIT C 1
98:00:00	7eal-E	5.8	4.3	64	65	5	MOLECULE:	UBIQUITIN
99:00:00	6iak-E	5.8	3.8	67	67	9	MOLECULE:	UNCHARACTERIZED PROTEIN
0:00	6j6g-c	5.8	19.5	125	436	3	MOLECULE:	PRE-MRNA-SPLICING FACTOR 8
1:00	6vqk-I	5.8	4.3	64	64	5	MOLECULE:	V-TYPE PROTON ATPASE SUBUNIT C 1
2:00	6j6q-c	5.8	31.3	133	436	3	MOLECULE:	PRE-MRNA-SPLICING FACTOR 8
3:00	6j6h-c	5.8	31.3	133	436	3	MOLECULE:	PRE-MRNA-SPLICING FACTOR 8
4:00	1kil-D	5.8	3.4	66	66	2	MOLECULE:	SYNAPTOBREVIN SNARE MOTIF
5:00	1urq-B	5.8	4.4	63	63	3	MOLECULE:	M-TOMOSYN ISOFORM
6:00	5low-D	5.8	4.3	67	67	6	MOLECULE:	RABPHILIN-3A
7:00	7dp8-E	5.8	5	112	123	4	MOLECULE:	TUBULIN ALPHA-1B CHAIN
8:00	1n7s-D	5.8	3.6	66	66	2	MOLECULE:	VESICLE-ASSOCIATED MEMBRANE PROTEIN 2
9:00	3b5n-H	5.8	2.7	63	63	8	MOLECULE:	SYNAPTOBREVIN HOMOLOG 1
10:00	6j6n-c	5.8	31.3	133	436	3	MOLECULE:	PRE-MRNA-SPLICING FACTOR 8

11:00	6ucm-C	5.8	4.9	65	65	14	MOLECULE:	PROTEIN FOSB
12:00	4yv3-C	5.8	4	63	63	8	MOLECULE:	VIMENTIN
13:00	1wt6-A	5.8	4.7	65	65	5	MOLECULE:	MYOTONIN-PROTEIN KINASE
14:00	7xfr-D	5.8	3.7	62	62	5	MOLECULE:	ISOFORM 2 OF WD REPEAT DOMAIN PHOSPHOINOSITI
15:00	8rtd-A	5.8	4.6	68	197	9	MOLECULE:	TRWJ PROTEIN
16:00	6v92-J	5.8	3.9	80	115	6	MOLECULE:	ACTIN-RELATED PROTEIN 7
17:00	6gbr-A	5.8	3.5	67	67	3	MOLECULE:	POLYMERASE COFACTOR VP35
18:00	2gv5-C	5.8	3.2	72	73	10	MOLECULE:	CELL DIVISION CONTROL PROTEIN 31
19:00	6sct-D	5.8	11.8	85	104	12	MOLECULE:	CLATHRIN HEAVY CHAIN
20:00	6mg2-B	5.8	2.4	68	69	9	MOLECULE:	CCAAT/ENHANCER-BINDING PROTEIN BETA
21:00	1hjb-D	5.8	2.6	68	68	3	MOLECULE:	CCAAT/ENHANCER BINDING PROTEIN BETA
22:00	4jzl-C	5.8	3	62	62	8	MOLECULE:	B-CELL RECEPTOR-ASSOCIATED PROTEIN 31
23:00	2e42-B	5.8	2.1	65	67	5	MOLECULE:	CCAAT/ENHANCER-BINDING PROTEIN BETA
24:00:00	4wy4-D	5.8	4.6	64	64	6	MOLECULE:	VESICLE-ASSOCIATED MEMBRANE PROTEIN 8
25:00:00	5cci-D	5.8	4.1	64	64	5	MOLECULE:	VESICLE-ASSOCIATED MEMBRANE PROTEIN 2
26:00:00	6gbq-D	5.8	4.7	68	68	4	MOLECULE:	POLYMERASE COFACTOR VP35
27:00:00	6pph-l	5.8	6.9	83	83	6	MOLECULE:	CAPSID VERTEX COMPONENT 1
28:00:00	4bry-B	5.8	5	69	69	13	MOLECULE:	GEMININ
29:00:00	2e43-B	5.8	2.1	64	64	5	MOLECULE:	CCAAT/ENHANCER-BINDING PROTEIN BETA
30:00:00	6mti-H	5.8	4.1	64	64	5	MOLECULE:	SYNAPTOTAGMIN-1
31:00:00	6mti-L	5.8	4.1	64	64	5	MOLECULE:	SYNAPTOTAGMIN-1
32:00:00	6mti-P	5.8	4.1	64	64	5	MOLECULE:	SYNAPTOTAGMIN-1
33:00:00	6r2f-B	5.8	4.6	64	64	6	MOLECULE:	TESTIS-EXPRESSED PROTEIN 12
34:00:00	6h9l-B	5.8	4.3	110	129	8	MOLECULE:	UNCHARACTERIZED PROTEIN
35:00:00	6mti-T	5.8	4.1	64	64	5	MOLECULE:	SYNAPTOTAGMIN-1
36:00:00	7fde-H	5.8	8.5	112	112	5	MOLECULE:	V-TYPE PROTON ATPASE SUBUNIT C
37:00:00	6r2f-A	5.8	4.6	64	64	6	MOLECULE:	TESTIS-EXPRESSED PROTEIN 12
38:00:00	5vpd-D	5.8	2.3	63	63	13	MOLECULE:	PROTEIN FOSB
39:00:00	7n84-Y	5.8	4.4	63	63	0	MOLECULE:	NUCLEOPORIN NUP188
40:00:00	6mti-X	5.8	4.1	64	64	5	MOLECULE:	SYNAPTOTAGMIN-1
41:00:00	4o9b-C	5.8	3.6	92	92	12	MOLECULE:	STROMAL INTERACTION MOLECULE 1
42:00:00	5cff-B	5.8	4.3	88	88	3	MOLECULE:	MIRANDA
43:00:00	5cff-D	5.8	5.3	88	88	3	MOLECULE:	MIRANDA
44:00:00	8b8b-D	5.8	4.7	97	97	5	MOLECULE:	MUNIA BORNAVIRUS 1 PHOSPHOPROTEIN
45:00:00	8b8d-C	5.8	4.3	101	101	9	MOLECULE:	PHOSPHOPROTEIN
46:00:00	5wsu-D	5.8	4.9	91	91	8	MOLECULE:	CALMODULIN
47:00:00	7a5p-I	5.8	3.1	70	133	10	MOLECULE:	U2 SNRNA
48:00:00	6ian-A	5.7	36.4	121	306	9	MOLECULE:	INTRAFLAGELLAR TRANSPORT PROTEIN 74
49:00:00	8r1a-A	5.7	6.6	143	587	7	MOLECULE:	GUANYLATE BINDING PROTEIN 1
50:00:00	8fed-E	5.7	20.1	119	358	8	MOLECULE:	VIRULENCE FACTOR MCE FAMILY PROTEIN
51:00:00	7wkk-L	5.7	4.8	91	156	4	MOLECULE:	MGC83295 PROTEIN
52:00:00	5cws-C	5.7	4	91	169	5	MOLECULE:	SAB-158 FAB LIGHT CHAIN
53:00:00	5low-F	5.7	4.5	67	68	4	MOLECULE:	RABPHILIN-3A
54:00:00	5cws-I	5.7	3.6	91	169	11	MOLECULE:	SAB-158 FAB LIGHT CHAIN
55:00:00	7l4v-A	5.7	3.9	64	66	8	MOLECULE:	CCAAT/ENHANCER-BINDING PROTEIN BETA
56:00:00	7d2e-A	5.7	1.1	62	62	8	MOLECULE:	LIPRIN-ALPHA-2
57:00:00	6mti-U	5.7	4.2	62	62	6	MOLECULE:	SYNAPTOTAGMIN-1
58:00:00	7tmm-H	5.7	11.1	107	109	6	MOLECULE:	H(+)-TRANSPORTING TWO-SECTOR ATPASE
59:00:00	6vqi-J	5.7	3.9	64	64	11	MOLECULE:	V-TYPE PROTON ATPASE SUBUNIT C 1
60:00:00	5wsu-C	5.7	3	90	90	8	MOLECULE:	CALMODULIN
61:00:00	5gmk-c	5.7	31.5	133	436	2	MOLECULE:	PRE-MRNA-SPLICING FACTOR 8

62:00:00	8ovw-K	5.7	16.4	82	219	10	MOLECULE:	CENTROMERE-BINDING PROTEIN 1
63:00:00	1gd2-F	5.7	4.5	64	64	5	MOLECULE:	DNA (5'-
64:00:00	3b5n-J	5.7	3.1	64	64	5	MOLECULE:	SYNAPTOBREVIN HOMOLOG 1
65:00:00	2n9b-B	5.7	4	68	69	6	MOLECULE:	UNCONVENTIONAL MYOSIN-X, GENERAL CONTROL PRO
66:00:00	1hvv-B	5.7	2.6	62	62	3	MOLECULE:	SYNTAXIN 1A
67:00:00	9b8q-M	5.7	6.2	102	102	4	MOLECULE:	V-TYPE PROTON ATPASE SUBUNIT C 1
68:00:00	5vox-L	5.7	7	103	104	7	MOLECULE:	V-TYPE PROTON ATPASE CATALYTIC SUBUNIT A,V-T
69:00:00	1n7s-A	5.7	4.6	63	63	6	MOLECULE:	VESICLE-ASSOCIATED MEMBRANE PROTEIN 2
70:00:00	4om2-A	5.7	3.9	80	112	8	MOLECULE:	TRANSDUCIN-LIKE ENHANCER PROTEIN 1
71:00:00	5vox-G	5.7	7.1	117	217	6	MOLECULE:	V-TYPE PROTON ATPASE CATALYTIC SUBUNIT A,V-T
72:00:00	5wsg-c	5.7	31.5	133	436	2	MOLECULE:	PRE-MRNA-SPLICING FACTOR 8
73:00:00	6wvw-E	5.7	4.1	63	63	10	MOLECULE:	VESICLE-ASSOCIATED MEMBRANE PROTEIN 2
74:00:00	2n1t-A	5.7	2.4	69	69	6	MOLECULE:	VESICLE-ASSOCIATED MEMBRANE PROTEIN 2
75:00:00	3lj5-A	5.7	9.7	129	692	5	MOLECULE:	PORTAL PROTEIN
76:00:00	6yai-D	5.7	6.8	76	104	14	MOLECULE:	CLATHRIN HEAVY CHAIN
77:00:00	3lj5-K	5.7	8	131	692	8	MOLECULE:	PORTAL PROTEIN
78:00:00	5gai-E	5.7	11.7	126	721	9	MOLECULE:	PORTAL PROTEIN
79:00:00	5tsj-H	5.7	11	101	182	8	MOLECULE:	V-TYPE ATP SYNTHASE ALPHA CHAIN
80:00:00	6sbz-D	5.7	10.8	82	104	12	MOLECULE:	CLATHRIN HEAVY CHAIN
81:00:00	5gai-F	5.7	11.6	130	721	7	MOLECULE:	PORTAL PROTEIN
82:00:00	5d80-l	5.7	5.4	68	68	6	MOLECULE:	V-TYPE PROTON ATPASE CATALYTIC SUBUNIT A
83:00:00	6tt7-K	5.7	5.6	140	208	5	MOLECULE:	ATP SYNTHASE SUBUNIT ALPHA
84:00:00	3lj5-J	5.7	7.8	130	692	8	MOLECULE:	PORTAL PROTEIN
85:00:00	5gmk-o	5.7	8.6	79	126	9	MOLECULE:	PRE-MRNA-SPLICING FACTOR 8
86:00:00	1gtw-A	5.7	2.5	65	65	3	MOLECULE:	CAAT/ENHANCER BINDING PROTEIN BETA
87:00:00	6sbz-N	5.7	10.7	83	104	12	MOLECULE:	CLATHRIN HEAVY CHAIN
88:00:00	2gv5-F	5.7	4.1	73	73	7	MOLECULE:	CELL DIVISION CONTROL PROTEIN 31
89:00:00	6sbz-I	5.7	10.8	82	104	12	MOLECULE:	CLATHRIN HEAVY CHAIN
90:00:00	1h8a-B	5.7	3.8	65	67	18	MOLECULE:	CAAT/ENHANCER BINDING PROTEIN BETA
91:00:00	2e42-A	5.7	2.6	65	65	3	MOLECULE:	CCAAT/ENHANCER-BINDING PROTEIN BETA
92:00:00	1hjb-E	5.7	3.6	67	68	7	MOLECULE:	CCAAT/ENHANCER BINDING PROTEIN BETA
93:00:00	5kj7-G	5.7	4.6	63	63	10	MOLECULE:	VESICLE-ASSOCIATED MEMBRANE PROTEIN 3
94:00:00	6m3w-A	5.7	35.5	135	344	2	MOLECULE:	SPIKE GLYCOPROTEIN
95:00:00	1h88-A	5.7	3.3	68	70	7	MOLECULE:	CCAAT/ENHANCER BINDING PROTEIN BETA
96:00:00	1hjb-B	5.7	2.2	67	67	3	MOLECULE:	CCAAT/ENHANCER BINDING PROTEIN BETA
97:00:00	7znl-M	5.7	6.4	122	549	6	MOLECULE:	THO COMPLEX SUBUNIT 1
98:00:00	6wvw-H	5.7	4.8	62	62	6	MOLECULE:	VESICLE-ASSOCIATED MEMBRANE PROTEIN 2
99:00:00	5ccg-D	5.7	4.6	64	64	5	MOLECULE:	VESICLE-ASSOCIATED MEMBRANE PROTEIN 2
0:00	5low-K	5.7	4.8	72	72	1	MOLECULE:	RABPHILIN-3A
1:00	6kn7-T	5.7	15.7	80	138	9	MOLECULE:	ACTIN, ALPHA SKELETAL MUSCLE
2:00	5mw9-F	5.7	4.3	64	64	3	MOLECULE:	CENTROSOMIN
3:00	5cff-A	5.7	4.9	87	87	3	MOLECULE:	MIRANDA
4:00	5cff-C	5.7	4.9	87	87	3	MOLECULE:	MIRANDA
5:00	5dol-A	5.7	2.8	61	61	7	MOLECULE:	INITIATION-CONTROL PROTEIN YABA
6:00	6v86-D	5.7	2.1	71	72	14	MOLECULE:	RNA-DIRECTED RNA POLYMERASE L
7:00	5d60-D	5.7	3.2	61	61	7	MOLECULE:	PUTATIVE TRANSCRIPTION FACTOR
8:00	4lin-L	5.7	7.1	139	289	12	MOLECULE:	TAIL NEEDLE PROTEIN GP26
9:00	8ovw-U	5.6	13.8	132	184	8	MOLECULE:	CENTROMERE-BINDING PROTEIN 1
10:00	7woo-I	5.6	35	131	187	10	MOLECULE:	NUCLEOPORIN NIC96
11:00	3oja-A	5.6	8.7	118	482	6	MOLECULE:	LEUCINE-RICH IMMUNE MOLECULE 1
12:00	6fia-D	5.6	8.9	104	104	7	MOLECULE:	LINE-1 RETROTRANSPOSABLE ELEMENT ORF1 PROTEI

13:00	6wuc-K	5.6	20.8	121	235	5	MOLECULE:	INNER KINETOCHORE SUBUNIT MCM16
14:00	8tek-P	5.6	4.9	120	138	6	MOLECULE:	DYNEIN REGULATORY COMPLEX PROTEIN 1/2 N-TERM
15:00	3opc-B	5.6	2.1	72	131	6	MOLECULE:	UNCHARACTERIZED PROTEIN
16:00	7znk-M	5.6	9.9	128	549	9	MOLECULE:	RNA
17:00	5ajs-B	5.6	4.6	64	64	11	MOLECULE:	THAP DOMAIN-CONTAINING PROTEIN 11
18:00	7bv6-U	5.6	4.8	66	66	9	MOLECULE:	VESICLE-ASSOCIATED MEMBRANE PROTEIN 8
19:00	1qbz-C	5.6	4.2	63	120	2	MOLECULE:	PROTEIN (SIV GP41 ECTODOMAIN)
20:00	1wt6-D	5.6	3.4	63	63	6	MOLECULE:	MYOTONIN-PROTEIN KINASE
21:00	1x79-C	5.6	4.2	88	88	6	MOLECULE:	ADP-RIBOSYLATION FACTOR BINDING PROTEIN GGA1
22:00	6vqj-J	5.6	4.1	63	64	6	MOLECULE:	V-TYPE PROTON ATPASE SUBUNIT C 1
23:00	7bv6-M	5.6	4.6	66	66	9	MOLECULE:	VESICLE-ASSOCIATED MEMBRANE PROTEIN 8
24:00:00	1h89-A	5.6	2.3	64	64	5	MOLECULE:	CAAT/ENHANCER BINDING PROTEIN BETA
25:00:00	1gmj-C	5.6	2.7	59	59	8	MOLECULE:	ATPASE INHIBITOR
26:00:00	3jb9-T	5.6	10.8	73	134	11	MOLECULE:	PRE-MRNA-SPLICING FACTOR SPP42
27:00:00	3b5n-L	5.6	3	62	62	8	MOLECULE:	SYNAPTOBREVIN HOMOLOG 1
28:00:00	3gn4-E	5.6	4	83	117	2	MOLECULE:	MYOSIN-VI
29:00:00	6pse-B	5.6	2.2	67	68	6	MOLECULE:	PROTEIN BICAUDAL D HOMOLOG 2
30:00:00	4m3l-A	5.6	2.9	60	60	5	MOLECULE:	E3 UBIQUITIN-PROTEIN LIGASE TRIM63
31:00:00	9b8q-I	5.6	7.2	117	148	7	MOLECULE:	V-TYPE PROTON ATPASE SUBUNIT C 1
32:00:00	6o7x-L	5.6	7.4	105	105	7	MOLECULE:	V-TYPE PROTON ATPASE SUBUNIT C
33:00:00	8t9d-B	5.6	9.1	110	158	6	MOLECULE:	MEDIATOR OF RNA POLYMERASE II TRANSCRIPTION
34:00:00	5gai-D	5.6	6.7	114	721	4	MOLECULE:	PORTAL PROTEIN
35:00:00	6qle-U	5.6	14.2	124	172	9	MOLECULE:	CENTRAL KINETOCHORE SUBUNIT MCM16,CENTRAL KI
36:00:00	5gai-H	5.6	10.8	126	721	4	MOLECULE:	PORTAL PROTEIN
37:00:00	3lj5-E	5.6	8	131	692	8	MOLECULE:	PORTAL PROTEIN
38:00:00	7fdc-H	5.6	7.9	109	111	7	MOLECULE:	YEAST VACUOLAR ATPASE A SUBUNIT
39:00:00	2e43-A	5.6	2	62	62	3	MOLECULE:	CCAAT/ENHANCER-BINDING PROTEIN BETA
40:00:00	7saf-A	5.6	3.3	62	62	6	MOLECULE:	GENERAL CONTROL TRANSCRIPTION FACTOR GCN4/M
41:00:00	6wvw-A	5.6	4.6	62	62	10	MOLECULE:	VESICLE-ASSOCIATED MEMBRANE PROTEIN 2
42:00:00	7bv6-X	5.6	4.6	65	65	6	MOLECULE:	VESICLE-ASSOCIATED MEMBRANE PROTEIN 8
43:00:00	6iak-A	5.6	3.5	68	68	9	MOLECULE:	UNCHARACTERIZED PROTEIN
44:00:00	1h89-B	5.6	2.9	64	64	3	MOLECULE:	CAAT/ENHANCER BINDING PROTEIN BETA
45:00:00	7eao-C	5.6	4.3	62	62	5	MOLECULE:	POLYUBIQUITIN-C
46:00:00	5mqf-L	5.6	36.3	116	336	8	MOLECULE:	PRE-MRNA-PROCESSING-SPLICING FACTOR 8
47:00:00	5dn6-W	5.6	6.1	109	124	0	MOLECULE:	CHAIN A
48:00:00	6fki-b	5.6	10.3	125	161	6	MOLECULE:	ATP SYNTHASE SUBUNIT A, CHLOROPLASTIC
49:00:00	3oja-B	5.6	4.9	110	534	12	MOLECULE:	LEUCINE-RICH IMMUNE MOLECULE 1
50:00:00	5d80-n	5.6	7.8	105	105	2	MOLECULE:	V-TYPE PROTON ATPASE CATALYTIC SUBUNIT A
51:00:00	5w5c-C	5.6	4.7	65	65	2	MOLECULE:	VESICLE-ASSOCIATED MEMBRANE PROTEIN 2
52:00:00	6gbr-D	5.6	2.2	67	67	4	MOLECULE:	POLYMERASE COFACTOR VP35
53:00:00	8dbs-X	5.6	8.5	137	156	6	MOLECULE:	ATP SYNTHASE SUBUNIT ALPHA
54:00:00	7qoo-H	5.5	18.9	121	210	7	MOLECULE:	CENTROMERE PROTEIN C
55:00:00	2xnx-M	5.5	4.3	106	107	8	MOLECULE:	FIBRINOGEN ALPHA CHAIN
56:00:00	8ap7-L	5.5	3.6	63	65	5	MOLECULE:	ATP SYNTHASE SUBUNIT A
57:00:00	7bjs-B	5.5	4.4	62	62	5	MOLECULE:	KINESIN HEAVY CHAIN
58:00:00	6zkl-q	5.5	11	89	139	1	MOLECULE:	NADH DEHYDROGENASE [UBIQUINONE] FLAVOPROTEIN
59:00:00	1gd2-H	5.5	2.7	63	64	5	MOLECULE:	DNA (5'-
60:00:00	6fia-F	5.5	4.1	88	88	9	MOLECULE:	LINE-1 RETROTRANSPOSABLE ELEMENT ORF1 PROTEI
61:00:00	5gox-A	5.5	6.9	95	181	6	MOLECULE:	DNA REPAIR PROTEIN RAD50
62:00:00	1hvv-D	5.5	3.4	61	61	3	MOLECULE:	SYNTAXIN 1A
63:00:00	7fda-H	5.5	7.1	111	111	8	MOLECULE:	YEAST VACUOLAR ATPASE A SUBUNIT

64:00:00	1urq-D	5.5	4	62	62	6	MOLECULE:	M-TOMOSYN ISOFORM
65:00:00	6id1-t	5.5	3.2	67	67	12	MOLECULE:	PRE-MRNA-PROCESSING-SPLICING FACTOR 8
66:00:00	5jvp-B	5.5	5.7	68	90	6	MOLECULE:	CHIMERA PROTEIN OF CENTROMERE-ASSOCIATED PRO
67:00:00	6ip1-D	5.5	4.6	61	62	3	MOLECULE:	VESICLE-ASSOCIATED MEMBRANE PROTEIN 2
68:00:00	1sfc-L	5.5	2.7	68	73	4	MOLECULE:	PROTEIN (SYNAPTOBREVIN 2)
69:00:00	8t1l-B	5.5	8.8	105	158	8	MOLECULE:	MEDIATOR OF RNA POLYMERASE II TRANSCRIPTION
70:00:00	8uwy-G	5.5	2.2	81	82	10	MOLECULE:	ACTIN, ALPHA CARDIAC MUSCLE 1
71:00:00	6pfp-B	5.5	5.5	124	156	7	MOLECULE:	MYOSIN-7 FUSED TO GP7 AND EB1
72:00:00	1h8a-A	5.5	2.4	66	68	9	MOLECULE:	CAAT/ENHANCER BINDING PROTEIN BETA
73:00:00	1io4-B	5.5	3.9	68	70	6	MOLECULE:	CSF-1R PROMOTER
74:00:00	8aia-H	5.5	7.6	138	179	6	MOLECULE:	CRESCENTIN
75:00:00	5ccg-J	5.5	4.1	63	63	5	MOLECULE:	VESICLE-ASSOCIATED MEMBRANE PROTEIN 2
76:00:00	2n1t-D	5.5	2	67	74	12	MOLECULE:	VESICLE-ASSOCIATED MEMBRANE PROTEIN 2
77:00:00	5ylz-t	5.5	5.4	69	128	3	MOLECULE:	PRE-MRNA-SPLICING FACTOR 8
78:00:00	2zxx-A	5.5	6.7	69	70	7	MOLECULE:	GEMININ
79:00:00	6id1-q	5.5	11.6	90	132	10	MOLECULE:	PRE-MRNA-PROCESSING-SPLICING FACTOR 8
80:00:00	6vqj-I	5.5	2.4	63	64	8	MOLECULE:	V-TYPE PROTON ATPASE SUBUNIT C 1
81:00:00	4bl6-C	5.5	5	86	86	7	MOLECULE:	PROTEIN BICAUDAL D
82:00:00	6icz-L	5.5	39.3	124	454	8	MOLECULE:	PROTEIN MAGO NASHI HOMOLOG 2
83:00:00	7w59-L	5.5	37.9	124	437	7	MOLECULE:	PRE-MRNA-PROCESSING-SPLICING FACTOR 8
84:00:00	5whf-E	5.5	5.3	85	85	5	MOLECULE:	VIMENTIN
85:00:00	4ll7-G	5.5	4.6	87	87	7	MOLECULE:	SWI5-DEPENDENT HO EXPRESSION PROTEIN 3
86:00:00	7njx-d	5.5	3	59	61	5	MOLECULE:	ATP SYNTHASE SUBUNIT C
87:00:00	6mg3-A	5.5	3.9	64	64	6	MOLECULE:	CCAAT/ENHANCER-BINDING PROTEIN BETA
88:00:00	6mti-M	5.5	4.5	62	62	10	MOLECULE:	SYNAPTOTAGMIN-1
89:00:00	8qrm-U	5.5	16.4	137	176	5	MOLECULE:	12S MITOCHONDRIAL RRNA
90:00:00	5w5d-C	5.5	4.6	61	61	2	MOLECULE:	VESICLE-ASSOCIATED MEMBRANE PROTEIN 2
91:00:00	1uii-A	5.5	4.7	61	61	3	MOLECULE:	GEMININ
92:00:00	5y88-r	5.5	5.4	69	129	3	MOLECULE:	PRE-MRNA-SPLICING FACTOR 8
93:00:00	7usd-C	5.5	6	69	191	6	MOLECULE:	CYTOPLASMIC FMR1-INTERACTING PROTEIN 1
94:00:00	6o7v-J	5.5	5.2	105	105	5	MOLECULE:	V-TYPE PROTON ATPASE SUBUNIT D
95:00:00	6cfz-A	5.5	4.8	66	66	6	MOLECULE:	ASK1
96:00:00	6fkh-p	5.5	7.5	116	143	10	MOLECULE:	ATP SYNTHASE SUBUNIT A, CHLOROPLASTIC
97:00:00	7p3w-b	5.5	7.3	117	150	7	MOLECULE:	ATP SYNTHASE SUBUNIT ALPHA
98:00:00	4wpx-B	5.4	8.1	78	81	10	MOLECULE:	CELL DIVISION CONTROL PROTEIN 31-LIKE PROTEI
99:00:00	6exn-D	5.4	7.7	96	97	4	MOLECULE:	U2 SNRNA
0:00	6iac-E	5.4	23.5	128	146	5	MOLECULE:	PORTAL PROTEIN
1:00	8th8-A	5.4	3.3	134	290	6	MOLECULE:	DYNEIN REGULATORY COMPLEX PROTEIN 1/2 N-TERM
2:00	6qaj-B	5.4	10	123	441	6	MOLECULE:	ENDOLYSIN,TRANSCRIPTION INTERMEDIARY FACTOR
3:00	7d2e-B	5.4	3.1	58	58	7	MOLECULE:	LIPRIN-ALPHA-2
4:00	6iak-D	5.4	3.5	62	62	10	MOLECULE:	UNCHARACTERIZED PROTEIN
5:00	4n3y-C	5.4	3.8	83	83	12	MOLECULE:	RAB5 GDP/GTP EXCHANGE FACTOR
6:00	6fia-E	5.4	4.5	88	88	9	MOLECULE:	LINE-1 RETROTRANSPOSABLE ELEMENT ORF1 PROTEI
7:00	7pkq-w	5.4	12.1	137	155	9	MOLECULE:	MS35
8:00	1sfc-H	5.4	2.9	67	72	4	MOLECULE:	PROTEIN (SYNAPTOBREVIN 2)
9:00	1io4-A	5.4	3.7	63	63	8	MOLECULE:	CSF-1R PROMOTER
10:00	5afu-5	5.4	4.9	139	275	0	MOLECULE:	DYNEIN TAIL
11:00	5voy-L	5.4	6.6	104	104	4	MOLECULE:	V-TYPE PROTON ATPASE CATALYTIC SUBUNIT A,V-T
12:00	5gmk-p	5.4	5.2	69	128	7	MOLECULE:	PRE-MRNA-SPLICING FACTOR 8
13:00	8x7z-A	5.4	1.5	83	83	8	MOLECULE:	HR1
14:00	8pr4-x	5.4	2.6	58	92	2	MOLECULE:	ARP11

15:00	4m3l-B	5.4	1.8	58	58	5	MOLECULE:	E3 UBIQUITIN-PROTEIN LIGASE TRIM63
16:00	6id0-t	5.4	4.4	67	67	3	MOLECULE:	PRE-MRNA-PROCESSING-SPLICING FACTOR 8
17:00	7fdc-J	5.4	7.2	109	111	5	MOLECULE:	YEAST VACUOLAR ATPASE A SUBUNIT
18:00	5ajs-C	5.4	4.5	61	61	11	MOLECULE:	THAP DOMAIN-CONTAINING PROTEIN 11
19:00	6za9-T	5.4	6.8	69	71	7	MOLECULE:	ATP SYNTHASE F(0) COMPLEX SUBUNIT C1, MITOCH
20:00	5c9n-B	5.4	3.4	61	61	0	MOLECULE:	GEMININ COILED-COIL DOMAIN-CONTAINING PROTEI
21:00	6j6g-p	5.4	5.4	69	128	3	MOLECULE:	PRE-MRNA-SPLICING FACTOR 8
22:00	5i7c-A	5.4	2.7	58	59	2	MOLECULE:	CENTROSOMIN
23:00	8aix-H	5.4	7.6	138	179	6	MOLECULE:	CRESCENTIN
24:00:00	7nju-d	5.4	4.5	61	61	3	MOLECULE:	ATP SYNTHASE SUBUNIT C
25:00:00	6h9l-A	5.4	5.9	100	127	5	MOLECULE:	UNCHARACTERIZED PROTEIN
26:00:00	6oqa-G	5.4	2.7	87	87	8	MOLECULE:	PEPTIDYL-PROLYL CIS-TRANS ISOMERASE FKBP1A
27:00:00	5bvz-C	5.4	5.8	101	180	4	MOLECULE:	DNA STABILIZATION PROTEIN
28:00:00	5i7c-C	5.4	2.8	58	59	2	MOLECULE:	CENTROSOMIN
29:00:00	6r0z-L	5.4	5.3	112	185	10	MOLECULE:	V-TYPE ATP SYNTHASE ALPHA CHAIN
30:00:00	1m1j-B	5.4	8.2	128	402	10	MOLECULE:	FIBRINOGEN ALPHA SUBUNIT
31:00:00	5mq0-u	5.4	4.7	64	116	3	MOLECULE:	YEAST UBC4 GENE FOR UBIQUITIN-CONJUGATING EN
32:00:00	6gap-C	5.4	9.5	144	213	8	MOLECULE:	OUTER CAPSID PROTEIN SIGMA-1
33:00:00	2w4u-U	5.4	9.4	153	277	5	MOLECULE:	TROPONIN C, SKELETAL MUSCLE
34:00:00	6id1-L	5.4	39.6	124	475	8	MOLECULE:	PRE-MRNA-PROCESSING-SPLICING FACTOR 8
35:00:00	5fil-T	5.4	7.4	128	174	3	MOLECULE:	ATP SYNTHASE SUBUNIT ALPHA, MITOCHONDRIAL
36:00:00	5low-M	5.4	3.5	70	72	3	MOLECULE:	RABPHILIN-3A
37:00:00	7njs-b	5.4	6.6	105	144	3	MOLECULE:	ATP SYNTHASE SUBUNIT ALPHA
38:00:00	8fl8-Z	5.4	6.7	125	155	4	MOLECULE:	ATP SYNTHASE PROTEIN 8
39:00:00	8cqn-B	5.3	5.7	123	187	7	MOLECULE:	LIPOPROTEIN, PUTATIVE
40:00:00	8fed-F	5.3	21.8	149	399	5	MOLECULE:	VIRULENCE FACTOR MCE FAMILY PROTEIN
41:00:00	7nl9-d	5.3	3.9	60	61	8	MOLECULE:	ATP SYNTHASE SUBUNIT ALPHA
42:00:00	1gl2-C	5.3	4.1	59	59	7	MOLECULE:	ENDOBREVIN
43:00:00	6iak-B	5.3	4.3	62	62	6	MOLECULE:	UNCHARACTERIZED PROTEIN
44:00:00	6wcj-O	5.3	3.4	59	59	2	MOLECULE:	CLATHRIN HEAVY CHAIN 1
45:00:00	7fde-G	5.3	6.4	111	231	8	MOLECULE:	V-TYPE PROTON ATPASE SUBUNIT C
46:00:00	7njy-d	5.3	4	60	61	8	MOLECULE:	ATP SYNTHASE SUBUNIT C
47:00:00	5gas-I	5.3	6.4	98	99	5	MOLECULE:	V-TYPE ATP SYNTHASE ALPHA CHAIN
48:00:00	6wcj-N	5.3	3.1	59	59	5	MOLECULE:	CLATHRIN HEAVY CHAIN 1
49:00:00	3fwc-B	5.3	3.4	83	83	10	MOLECULE:	CELL DIVISION CONTROL PROTEIN 31
50:00:00	4n3y-B	5.3	3.2	83	83	0	MOLECULE:	RAB5 GDP/GTP EXCHANGE FACTOR
51:00:00	6id0-L	5.3	43.5	143	475	6	MOLECULE:	PRE-MRNA-PROCESSING-SPLICING FACTOR 8
52:00:00	3b5n-E	5.3	3.4	58	58	9	MOLECULE:	SYNAPTOBREVIN HOMOLOG 1
53:00:00	7bv6-Q	5.3	4.3	61	61	3	MOLECULE:	VESICLE-ASSOCIATED MEMBRANE PROTEIN 8
54:00:00	1hvv-C	5.3	4.1	66	66	14	MOLECULE:	SYNTAXIN 1A
55:00:00	5adx-Z	5.3	29	129	419	0	MOLECULE:	ACTIN RELATED PROTEIN 1
56:00:00	6v85-C	5.3	3.1	74	74	7	MOLECULE:	RNA-DIRECTED RNA POLYMERASE L
57:00:00	5gai-C	5.3	9.1	125	721	3	MOLECULE:	PORTAL PROTEIN
58:00:00	8ppr-P	5.3	4.6	86	176	10	MOLECULE:	KINETOCHORE-ASSOCIATED PROTEIN DSN1 HOMOLOG
59:00:00	6vqi-a	5.3	13.4	83	309	11	MOLECULE:	V-TYPE PROTON ATPASE SUBUNIT C 1
60:00:00	5apv-B	5.3	4	67	67	6	MOLECULE:	GENERAL CONTROL PROTEIN GCN4
61:00:00	4m3l-C	5.3	1.6	57	57	5	MOLECULE:	E3 UBIQUITIN-PROTEIN LIGASE TRIM63
62:00:00	5f5p-E	5.3	3.8	57	57	11	MOLECULE:	PROTEIN SHROOM2
63:00:00	6gbq-H	5.3	4.5	63	63	3	MOLECULE:	POLYMERASE COFACTOR VP35
64:00:00	4wij-B	5.3	3.6	118	301	7	MOLECULE:	SPLICING FACTOR, PROLINE- AND GLUTAMINE-RICH
65:00:00	6r0y-J	5.3	6.4	115	185	8	MOLECULE:	V-TYPE ATP SYNTHASE ALPHA CHAIN

66:00:00	5oi7-A	5.3	3.5	86	88	10	MOLECULE:	CENTROSOMAL PROTEIN OF 85 KDA
67:00:00	3wuv-A	5.3	2.7	54	56	17	MOLECULE:	CENTROSOMAL PROTEIN OF 55 KDA
68:00:00	4jpp-C	5.3	6.3	101	122	5	MOLECULE:	MINOR SPIKE PROTEIN H
69:00:00	5jlh-I	5.3	9.2	120	135	0	MOLECULE:	ACTIN, CYTOPLASMIC 2
70:00:00	5jvp-C	5.3	4	68	90	4	MOLECULE:	CHIMERA PROTEIN OF CENTROMERE-ASSOCIATED PRO
71:00:00	5t4q-I	5.3	13.5	117	155	7	MOLECULE:	ATP SYNTHASE SUBUNIT ALPHA
72:00:00	8txr-A	5.3	8.9	142	427	8	MOLECULE:	EXODEOXYRIBONUCLEASE 7 LARGE SUBUNIT
73:00:00	4n5b-A	5.2	5.2	81	104	5	MOLECULE:	PHOSPHOPROTEIN
74:00:00	2fo1-D	5.2	5.6	63	63	8	MOLECULE:	5'-
75:00:00	8th8-J	5.2	48.9	144	372	9	MOLECULE:	DYNEIN REGULATORY COMPLEX PROTEIN 1/2 N-TERM
76:00:00	6j9r-B	5.2	5.4	102	126	7	MOLECULE:	BRAIN TUMOR PROTEIN
77:00:00	5hda-A	5.2	6.7	95	121	6	MOLECULE:	ZINC FINGER MYND DOMAIN-CONTAINING PROTEIN 1
78:00:00	8p0v-K	5.2	27.9	128	304	9	MOLECULE:	COILED-COIL DOMAIN-CONTAINING PROTEIN 93
79:00:00	7puz-A	5.2	3.4	102	148	9	MOLECULE:	MICOS COMPLEX SUBUNIT MIC60
80:00:00	5cws-D	5.2	4.8	90	180	7	MOLECULE:	SAB-158 FAB LIGHT CHAIN
81:00:00	8iah-W	5.2	8.3	142	248	10	MOLECULE:	ADDUCIN 1
82:00:00	5aj3-d	5.2	15.5	139	177	9	MOLECULE:	MITORIBOSOMAL 12S RRNA
83:00:00	3bat-C	5.2	4.1	83	85	7	MOLECULE:	MYOSIN HEAVY CHAIN, STRIATED MUSCLE/GENERAL
84:00:00	3b5n-I	5.2	4.4	58	58	9	MOLECULE:	SYNAPTOBREVIN HOMOLOG 1
85:00:00	5mw9-H	5.2	3.4	57	57	7	MOLECULE:	CENTROSOMIN
86:00:00	5nw4-5	5.2	8	154	275	0	MOLECULE:	DYNEIN HEAVY CHAIN
87:00:00	6wcj-J	5.2	3.3	59	59	2	MOLECULE:	CLATHRIN HEAVY CHAIN 1
88:00:00	7qoo-K	5.2	15.7	132	253	7	MOLECULE:	CENTROMERE PROTEIN C
89:00:00	7qix-P	5.2	7.8	69	237	10	MOLECULE:	18S RRNA BODY
90:00:00	7oz3-A	5.2	10.5	78	202	9	MOLECULE:	GNTR FAMILY TRANSCRIPTIONAL REGULATOR
91:00:00	7woo-H	5.2	19.4	91	246	7	MOLECULE:	NUCLEOPORIN NIC96
92:00:00	6wm2-K	5.2	10	113	114	4	MOLECULE:	V-TYPE PROTON ATPASE SUBUNIT E 1
93:00:00	5nt2-N	5.2	3.5	96	173	9	MOLECULE:	NON-STRUCTURAL PROTEIN 1
94:00:00	7p3n-p	5.2	10.4	126	155	6	MOLECULE:	ATP SYNTHASE SUBUNIT ALPHA
95:00:00	7vf5-C	5.2	7.8	109	184	6	MOLECULE:	PROTEIN VIRILIZER HOMOLOG
96:00:00	6r0w-J	5.2	18.4	107	185	9	MOLECULE:	V-TYPE ATP SYNTHASE ALPHA CHAIN
97:00:00	6ip1-B	5.2	3.7	62	62	5	MOLECULE:	VESICLE-ASSOCIATED MEMBRANE PROTEIN 2
98:00:00	8x5f-B	5.2	28.7	91	616	4	MOLECULE:	SOLUTE CARRIER FAMILY 53 MEMBER 1
99:00:00	2e7s-O	5.2	7.5	112	124	7	MOLECULE:	RAB GUANINE NUCLEOTIDE EXCHANGE FACTOR SEC2
0:00	8bd7-L	5.1	34.6	155	303	7	MOLECULE:	IFT88
1:00	7woo-G	5.1	3.5	91	200	12	MOLECULE:	NUCLEOPORIN NIC96
2:00	8u95-A	5.1	7	143	1009	8	MOLECULE:	MYOSIN HEAVY CHAIN, ISOFORM U
3:00	7d7n-A	5.1	10.4	93	704	11	MOLECULE:	ATP-BINDING CASSETTE SUB-FAMILY B MEMBER 6,
4:00	7o3x-B	5.1	26.3	133	215	8	MOLECULE:	PROTEIN SLL0617
5:00	6cfz-I	5.1	12.4	106	106	13	MOLECULE:	ASK1
6:00	8a5a-G	5.1	7.9	104	110	7	MOLECULE:	CHROMATIN-REMODELING ATPASE INO80
7:00	4yv3-B	5.1	2.8	75	75	12	MOLECULE:	VIMENTIN
8:00	5jxc-D	5.1	4	78	78	8	MOLECULE:	RAS/RAP GTPASE-ACTIVATING PROTEIN SYNGAP
9:00	5jxc-B	5.1	3	78	78	1	MOLECULE:	RAS/RAP GTPASE-ACTIVATING PROTEIN SYNGAP
10:00	7bv6-B	5.1	1.8	61	61	15	MOLECULE:	VESICLE-ASSOCIATED MEMBRANE PROTEIN 8
11:00	2zvo-D	5.1	2.1	87	88	9	MOLECULE:	UBC PROTEIN
12:00	2n5e-B	5.1	25.9	110	167	8	MOLECULE:	APOLIPOPROTEIN A-I
13:00	3lj5-H	5.1	8.5	136	692	8	MOLECULE:	PORTAL PROTEIN
14:00	6von-I	5.1	14.4	128	153	9	MOLECULE:	ATP SYNTHASE SUBUNIT ALPHA, CHLOROPLASTIC
15:00	5f5p-F	5.1	3.2	58	58	10	MOLECULE:	PROTEIN SHROOM2
16:00	6ian-C	5.1	8.6	105	432	12	MOLECULE:	INTRAFLAGELLAR TRANSPORT PROTEIN 74

17:00	6wm3-K	5.1	9.5	111	114	5	MOLECULE:	V-TYPE PROTON ATPASE 116 KDA SUBUNIT A ISOFO
18:00	8fby-D	5.1	5.2	86	86	8	MOLECULE:	PLASMALEMMA VESICLE-ASSOCIATED PROTEIN
19:00	8k8a-A	5.1	3.2	61	62	2	MOLECULE:	NUCLEAR FACTOR INTERLEUKIN-3-REGULATED PROTE
20:00	3wut-D	5.1	2.9	53	54	17	MOLECULE:	CENTROSOMAL PROTEIN OF 55 KDA
21:00	2e7s-H	5.1	4.7	88	107	11	MOLECULE:	RAB GUANINE NUCLEOTIDE EXCHANGE FACTOR SEC2
22:00	4m8m-B	5	17.4	92	574	7	MOLECULE:	GCN4 COILED-COIL FUSED ZEBRAFISH PLEXINC1
23:00	6vq8-H	5	3.6	94	214	10	MOLECULE:	ATPASE H+-TRANSPORTING V1 SUBUNIT A
24:00:00	6aoz-C	5	3.2	74	74	9	MOLECULE:	CASP8-ASSOCIATED PROTEIN 2
25:00:00	7rzr-C	5	0.9	71	71	11	MOLECULE:	SARS-COV-2 HR1 D936Y LINKED TO A SCAFFOLD,SP
26:00:00	7rzv-C	5	0.9	71	71	11	MOLECULE:	SARS-COV-2 HR1 LINKED TO A SCAFFOLD,SPIKE PR
27:00:00	7rzr-A	5	0.9	71	71	11	MOLECULE:	SARS-COV-2 HR1 D936Y LINKED TO A SCAFFOLD,SP
28:00:00	7rzq-A	5	0.9	71	71	11	MOLECULE:	SARS-COV-2 HR1 LINKED TO A SCAFFOLD,SPIKE PR
29:00:00	7rzt-C	5	0.9	71	71	11	MOLECULE:	SARS-COV-2 HR1 S940F LINKED TO A SCAFFOLD,SP
30:00:00	7rzq-B	5	0.9	71	71	11	MOLECULE:	SARS-COV-2 HR1 LINKED TO A SCAFFOLD,SPIKE PR
31:00:00	1kil-B	5	2	59	59	12	MOLECULE:	SYNAPTOBREVIN SNARE MOTIF
32:00:00	6f3a-5	5	8	154	275	0	MOLECULE:	ARP1 ACTIN RELATED PROTEIN 1 HOMOLOG A
33:00:00	5fiy-A	5	2.3	77	78	1	MOLECULE:	PRKC APOPTOSIS WT1 REGULATOR PROTEIN
34:00:00	5xit-A	5	2.8	76	76	11	MOLECULE:	UBIQUITIN-40S RIBOSOMAL PROTEIN S27A
35:00:00	6fpr-B	5	2.4	58	58	9	MOLECULE:	SIGNAL RECOGNITION PARTICLE RECEPTOR FTSY
36:00:00	5ip0-M	5	3.8	57	107	5	MOLECULE:	PHA GRANULE-ASSOCIATED PROTEIN
37:00:00	8sdi-A	5	3.4	80	80	5	MOLECULE:	TRIPARTITE MOTIF-CONTAINING PROTEIN 45
38:00:00	2xqh-A	4.9	8.5	118	258	10	MOLECULE:	IMMUNOGLOBULIN-BINDING PROTEIN EIBD
39:00:00	4dl0-K	4.9	5.8	97	97	8	MOLECULE:	V-TYPE PROTON ATPASE SUBUNIT C
40:00:00	8tek-A	4.9	8.8	141	200	11	MOLECULE:	DYNEIN REGULATORY COMPLEX PROTEIN 1/2 N-TERM
41:00:00	5dfz-D	4.9	11.5	128	341	8	MOLECULE:	VACUOLAR PROTEIN SORTING-ASSOCIATED PROTEIN
42:00:00	4r8g-E	4.9	17.7	110	324	7	MOLECULE:	UNCONVENTIONAL MYOSIN-IC
43:00:00	8wjo-B	4.9	5.8	113	283	7	MOLECULE:	STRUCTURAL MAINTENANCE OF CHROMOSOMES PROTEI
44:00:00	3v6i-A	4.9	5.7	114	186	10	MOLECULE:	V-TYPE ATP SYNTHASE SUBUNIT E
45:00:00	8p0v-L	4.9	32.8	93	416	9	MOLECULE:	COILED-COIL DOMAIN-CONTAINING PROTEIN 93
46:00:00	8fwj-A	4.9	7.9	95	552	11	MOLECULE:	CIRCADIAN CLOCK PROTEIN KAIC
47:00:00	8g0q-B	4.9	14.7	88	230	9	MOLECULE:	KINETOCHORE PROTEIN NDC80
48:00:00	2eqb-C	4.9	4	91	93	5	MOLECULE:	RAS-RELATED PROTEIN SEC4
49:00:00	7tik-B	4.9	0.9	71	71	11	MOLECULE:	FERRITIN, DPS FAMILY PROTEIN AND SPIKE PROTE
50:00:00	6f5x-A	4.9	3.6	76	76	13	MOLECULE:	SYNAPTONEMAL COMPLEX PROTEIN 1
51:00:00	6mti-C	4.9	3.2	73	73	7	MOLECULE:	SYNAPTOTAGMIN-1
52:00:00	1gmj-D	4.9	1.9	56	56	7	MOLECULE:	ATPASE INHIBITOR
53:00:00	6mti-O	4.9	3.2	73	73	7	MOLECULE:	SYNAPTOTAGMIN-1
54:00:00	6mti-W	4.9	3.2	73	73	7	MOLECULE:	SYNAPTOTAGMIN-1
55:00:00	6mti-K	4.9	3.2	73	73	7	MOLECULE:	SYNAPTOTAGMIN-1
56:00:00	7y4s-A	4.9	10	93	343	3	MOLECULE:	TRIPARTITE MOTIF-CONTAINING PROTEIN 72
57:00:00	6mti-G	4.9	3.2	73	73	7	MOLECULE:	SYNAPTOTAGMIN-1
58:00:00	6mti-S	4.9	3.2	73	73	7	MOLECULE:	SYNAPTOTAGMIN-1
59:00:00	5nt1-A	4.9	5.2	105	173	8	MOLECULE:	E3 UBIQUITIN/ISG15 LIGASE TRIM25
60:00:00	3lj5-L	4.9	8	131	692	8	MOLECULE:	PORTAL PROTEIN
61:00:00	3lj5-B	4.9	8	131	692	8	MOLECULE:	PORTAL PROTEIN
62:00:00	3lj5-D	4.9	8	131	692	8	MOLECULE:	PORTAL PROTEIN
63:00:00	5gai-J	4.9	8.9	125	721	3	MOLECULE:	PORTAL PROTEIN
64:00:00	7z47-F	4.9	5.9	66	68	5	MOLECULE:	ADAPTOR PROTEIN
65:00:00	3wuv-J	4.9	4.3	56	56	4	MOLECULE:	CENTROSOMAL PROTEIN OF 55 KDA
66:00:00	4m3l-D	4.9	2.4	53	53	6	MOLECULE:	E3 UBIQUITIN-PROTEIN LIGASE TRIM63
67:00:00	5low-N	4.9	4.5	60	60	8	MOLECULE:	RABPHILIN-3A

68:00:00	6yjd-A	4.9	3.8	87	104	8	MOLECULE:	CAPSID ASSEMBLY SCAFFOLDING PROTEIN,PRELAMIN
69:00:00	1nyh-A	4.9	3.3	76	76	11	MOLECULE:	REGULATORY PROTEIN SIR4
70:00:00	5opt-P	4.8	8.7	87	249	6	MOLECULE:	ACTIVATED PROTEIN KINASE C RECEPTOR, PUTATIV
71:00:00	5ijn-F	4.8	29.7	130	335	10	MOLECULE:	NUCLEAR PORE COMPLEX PROTEIN NUP155
72:00:00	6znl-n	4.8	13.7	93	343	10	MOLECULE:	ARP1 ACTIN RELATED PROTEIN 1 HOMOLOG A
73:00:00	5chx-A	4.8	10.5	118	210	14	MOLECULE:	XRCC4-MYH7-1590-1657
74:00:00	1aa0-A	4.8	3.8	85	113	9	MOLECULE:	FIBRITIN
75:00:00	8srm-B	4.8	9.7	85	235	5	MOLECULE:	RB1-INDUCIBLE COILED-COIL PROTEIN 1
76:00:00	7qoo-U	4.8	15.5	132	186	6	MOLECULE:	CENTROMERE PROTEIN C
77:00:00	4lin-A	4.8	10.2	144	289	5	MOLECULE:	TAIL NEEDLE PROTEIN GP26
78:00:00	4q9u-C	4.8	3.9	83	83	6	MOLECULE:	RAB5 GDP/GTP EXCHANGE FACTOR
79:00:00	7r5v-U	4.8	7.2	111	165	5	MOLECULE:	CENTROMERE PROTEIN H
80:00:00	4q9u-G	4.8	5	84	84	0	MOLECULE:	RAB5 GDP/GTP EXCHANGE FACTOR
81:00:00	2yo3-B	4.8	9	134	258	9	MOLECULE:	GENERAL CONTROL PROTEIN GCN4, PUTATIVE INNER
82:00:00	5tby-A	4.8	36.5	76	954	7	MOLECULE:	MYOSIN-7
83:00:00	3uia-A	4.8	3.4	53	53	0	MOLECULE:	GLYCOPROTEIN 41
84:00:00	7tkd-U	4.8	15.5	130	155	11	MOLECULE:	ATP SYNTHASE SUBUNIT 9, MITOCHONDRIAL
85:00:00	8th8-E	4.8	10.1	140	291	5	MOLECULE:	DYNEIN REGULATORY COMPLEX PROTEIN 1/2 N-TERM
86:00:00	4jpn-C	4.8	5	77	77	1	MOLECULE:	MINOR SPIKE PROTEIN H
87:00:00	4mvd-E	4.8	15	110	252	12	MOLECULE:	CHOLINE-PHOSPHATE CYTIDYLYLTRANSFERASE A
88:00:00	5oqm-h	4.7	8.8	101	131	8	MOLECULE:	DNA-DIRECTED RNA POLYMERASE II SUBUNIT RPB1
89:00:00	2p2u-B	4.7	23.3	83	153	8	MOLECULE:	HOST-NUCLEASE INHIBITOR PROTEIN GAM, PUTATIV
90:00:00	8q85-V	4.7	6.8	96	117	8	MOLECULE:	KINETOCHORE PROTEIN NDC80
91:00:00	5dfz-A	4.7	23.1	130	343	7	MOLECULE:	VACUOLAR PROTEIN SORTING-ASSOCIATED PROTEIN
92:00:00	2p22-A	4.7	13.5	121	168	9	MOLECULE:	SUPPRESSOR PROTEIN STP22 OF TEMPERATURE-SENS
93:00:00	4q9u-H	4.7	4.1	81	82	11	MOLECULE:	RAB5 GDP/GTP EXCHANGE FACTOR
94:00:00	4mvd-A	4.7	17.8	100	252	8	MOLECULE:	CHOLINE-PHOSPHATE CYTIDYLYLTRANSFERASE A
95:00:00	4mvd-C	4.7	18.6	100	253	5	MOLECULE:	CHOLINE-PHOSPHATE CYTIDYLYLTRANSFERASE A
96:00:00	8amr-C	4.7	3.8	95	147	11	MOLECULE:	TRIPARTITE MOTIF-CONTAINING PROTEIN 3
97:00:00	4jpn-H	4.7	4.1	74	74	4	MOLECULE:	MINOR SPIKE PROTEIN H
98:00:00	5yfp-C	4.7	39	127	790	6	MOLECULE:	EXOCYST COMPLEX COMPONENT SEC3
99:00:00	4lin-C	4.7	9.7	137	289	6	MOLECULE:	TAIL NEEDLE PROTEIN GP26
0:00	5n9j-D	4.6	17	97	135	3	MOLECULE:	MEDIATOR OF RNA POLYMERASE II TRANSCRIPTION
1:00	8tek-B	4.6	9	145	208	8	MOLECULE:	DYNEIN REGULATORY COMPLEX PROTEIN 1/2 N-TERM
2:00	8ppr-N	4.6	6.1	103	217	9	MOLECULE:	KINETOCHORE-ASSOCIATED PROTEIN DSN1 HOMOLOG
3:00	6b85-A	4.6	5.1	66	215	3	MOLECULE:	TMHC4_R
4:00	6h9m-A	4.6	3.9	84	94	8	MOLECULE:	COILED-COIL DOMAIN-CONTAINING PROTEIN 90B, M
5:00	5mq4-C	4.6	7.2	95	117	16	MOLECULE:	PROTEIN KINASE C-BINDING PROTEIN 1
6:00	2eqb-B	4.6	8.3	76	93	11	MOLECULE:	RAS-RELATED PROTEIN SEC4
7:00	4mvd-G	4.6	17.5	100	252	8	MOLECULE:	CHOLINE-PHOSPHATE CYTIDYLYLTRANSFERASE A
8:00	6n6r-B	4.6	2.5	68	68	3	MOLECULE:	UBIQUITIN
9:00	7cld-E	4.6	10.2	78	123	6	MOLECULE:	TUBULIN ALPHA-1B CHAIN
10:00	3f1i-H	4.6	5.4	76	98	9	MOLECULE:	HEPATOCYTE GROWTH FACTOR-REGULATED TYROSINE
11:00	8dd0-O	4.6	1.4	70	70	6	MOLECULE:	ACTIN, ALPHA CARDIAC MUSCLE 1
12:00	1kfm-A	4.6	3.3	50	50	6	MOLECULE:	MAJOR OUTER MEMBRANE LIPOPROTEIN
13:00	1ik7-A	4.6	2.6	52	52	8	MOLECULE:	PROBABLE SERINE/THREONINE-PROTEIN KINASE PEL
14:00	3htk-B	4.6	3.7	73	73	10	MOLECULE:	STRUCTURAL MAINTENANCE OF CHROMOSOMES PROTEI
15:00	6aoz-D	4.6	1.8	68	68	9	MOLECULE:	CASP8-ASSOCIATED PROTEIN 2
16:00	8q5h-B	4.6	8.3	75	174	12	MOLECULE:	KINETOCHORE PROTEIN SPC24
17:00	6wko-A	4.6	2.6	60	90	5	MOLECULE:	HEMAGGLUTININ-ESTERASE-FUSION GLYCOPROTEIN
18:00	3wqa-B	4.6	6.3	118	201	10	MOLECULE:	TRIMERIC AUTOTRANSPORTER ADHESIN

19:00	2yo3-A	4.6	7.4	96	257	8	MOLECULE:	GENERAL CONTROL PROTEIN GCN4, PUTATIVE INNER
20:00	8at3-D	4.5	36.2	107	666	7	MOLECULE:	HAUS AUGMIN-LIKE COMPLEX SUBUNIT 1
21:00	8etc-F	4.5	17.3	55	218	13	MOLECULE:	RNA (2151-MER)
22:00	1lj2-B	4.5	7.3	66	110	2	MOLECULE:	NONSTRUCTURAL RNA-BINDING PROTEIN 34
23:00	8hpo-D	4.5	11.5	112	165	9	MOLECULE:	TRANSCRIPTIONAL REGULATORY PROTEIN UME1
24:00:00	4y66-C	4.5	19.2	115	197	6	MOLECULE:	MND1
25:00:00	8hd0-E	4.5	26.8	99	380	8	MOLECULE:	CELL DIVISION ATP-BINDING PROTEIN FTSE
26:00:00	6wm2-I	4.5	7.4	111	225	5	MOLECULE:	V-TYPE PROTON ATPASE SUBUNIT E 1
27:00:00	4xa6-B	4.5	12.8	124	165	7	MOLECULE:	GP7-MYH7(1777-1855)-EB1 CHIMERA PROTEIN
28:00:00	5wst-B	4.5	2.4	70	70	9	MOLECULE:	UNCONVENTIONAL MYOSIN-VIIA
29:00:00	5hzp-C	4.5	1.5	71	71	3	MOLECULE:	M PROTEIN, SEROTYPE 49
30:00:00	6ano-B	4.5	3.6	70	70	6	MOLECULE:	CASP8-ASSOCIATED PROTEIN 2
31:00:00	6cfz-D	4.5	3.3	72	73	1	MOLECULE:	ASK1
32:00:00	6aoz-B	4.5	3.7	69	69	6	MOLECULE:	CASP8-ASSOCIATED PROTEIN 2
33:00:00	6eb9-A	4.5	8.1	74	101	9	MOLECULE:	PHOSPHOPROTEIN
34:00:00	5kj8-B	4.5	3.6	66	66	2	MOLECULE:	VESICLE-ASSOCIATED MEMBRANE PROTEIN 3
35:00:00	3lj5-I	4.5	8	131	692	8	MOLECULE:	PORTAL PROTEIN
36:00:00	5zuv-C	4.5	1.3	84	119	8	MOLECULE:	SPIKE GLYCOPROTEIN,INHIBITOR EK1
37:00:00	2ba2-A	4.5	3.8	81	81	5	MOLECULE:	HYPOTHETICAL UPF0134 PROTEIN MPN010
38:00:00	7x5e-A	4.5	2.9	80	99	9	MOLECULE:	TRANSCRIPTION FACTOR MAFG
39:00:00	4aj5-B	4.5	11.5	86	87	5	MOLECULE:	SPINDLE AND KINETOCHORE-ASSOCIATED PROTEIN 1
40:00:00	8iw0-A	4.5	3.3	63	84	8	MOLECULE:	LIPRIN-BETA-1,KN MOTIF AND ANKYRIN REPEAT DO
41:00:00	6tda-L	4.5	57.3	120	596	6	MOLECULE:	HISTONE H3.2
42:00:00	6vy1-g	4.5	2.8	46	121	4	MOLECULE:	PREFOLDIN SUBUNIT ALPHA 2
43:00:00	6vy1-e	4.5	3.5	47	121	4	MOLECULE:	PREFOLDIN SUBUNIT ALPHA 2
44:00:00	8j0s-d	4.5	15.7	149	445	7	MOLECULE:	ATP SYNTHASE SUBUNIT A
45:00:00	4egx-C	4.5	4.2	66	174	11	MOLECULE:	KINESIN-LIKE PROTEIN KIF1A
46:00:00	3wpr-C	4.5	7.4	106	218	6	MOLECULE:	TRIMERIC AUTOTRANSPORTER ADHESIN
47:00:00	6h9m-C	4.5	4.1	84	94	7	MOLECULE:	COILED-COIL DOMAIN-CONTAINING PROTEIN 90B, M
48:00:00	3hgf-A	4.4	4.1	69	98	3	MOLECULE:	RHOPTRY PROTEIN FRAGMENT
49:00:00	6gvw-F	4.4	23.1	86	320	14	MOLECULE:	BRCA1-A COMPLEX SUBUNIT ABRAXAS 1
50:00:00	8at3-A	4.4	43.1	73	286	7	MOLECULE:	HAUS AUGMIN-LIKE COMPLEX SUBUNIT 1
51:00:00	3n7n-B	4.4	7.8	93	164	5	MOLECULE:	MONOPOLIN COMPLEX SUBUNIT CSM1
52:00:00	8akr-R	4.4	12.8	132	196	14	MOLECULE:	CHLOROPLAST MEMBRANE-ASSOCIATED 30 KD PROTEI
53:00:00	4q9u-D	4.4	3.8	75	75	11	MOLECULE:	RAB5 GDP/GTP EXCHANGE FACTOR
54:00:00	6vqk-M	4.4	4.1	67	67	4	MOLECULE:	V-TYPE PROTON ATPASE SUBUNIT C 1
55:00:00	5d80-k	4.4	9.3	91	178	4	MOLECULE:	V-TYPE PROTON ATPASE CATALYTIC SUBUNIT A
56:00:00	7ucd-J	4.4	2.6	68	68	7	MOLECULE:	PROTEIN FOSB
57:00:00	6vqk-O	4.4	1.9	66	66	9	MOLECULE:	V-TYPE PROTON ATPASE SUBUNIT C 1
58:00:00	6k15-H	4.4	21.3	123	393	7	MOLECULE:	CHROMATIN STRUCTURE-REMODELING COMPLEX SUBUN
59:00:00	5eoa-A	4.4	5.3	72	72	7	MOLECULE:	OPTINEURIN
60:00:00	6vqj-M	4.4	4.1	67	67	4	MOLECULE:	V-TYPE PROTON ATPASE SUBUNIT C 1
61:00:00	6w2d-x	4.4	3.5	68	68	6	MOLECULE:	MAJOR CAPSID PROTEIN
62:00:00	7uo7-B	4.4	10.8	90	186	4	MOLECULE:	RNA-DIRECTED RNA POLYMERASE
63:00:00	8pr3-B	4.4	3.3	84	105	11	MOLECULE:	C-JUN-AMINO-TERMINAL KINASE-INTERACTING PROT
64:00:00	2b9b-B	4.4	9.1	97	482	7	MOLECULE:	FUSION GLYCOPROTEIN F0
65:00:00	6wcj-F	4.4	7	77	109	16	MOLECULE:	CLATHRIN HEAVY CHAIN 1
66:00:00	4lin-F	4.4	9.7	95	289	7	MOLECULE:	TAIL NEEDLE PROTEIN GP26
67:00:00	5djo-A	4.4	3.9	57	158	5	MOLECULE:	KINESIN-LIKE PROTEIN
68:00:00	6h9m-B	4.4	4.9	84	94	7	MOLECULE:	COILED-COIL DOMAIN-CONTAINING PROTEIN 90B, M
69:00:00	2yny-A	4.4	9.7	71	106	11	MOLECULE:	GENERAL CONTROL PROTEIN GCN4, PUTATIVE INNER

70:00:00	7ty0-C	4.3	38.3	70	488	6	MOLECULE:	GLYCOPROTEIN G
71:00:00	8tek-T	4.3	18.6	110	137	4	MOLECULE:	DYNEIN REGULATORY COMPLEX PROTEIN 1/2 N-TERM
72:00:00	7mge-B	4.3	10.2	68	442	7	MOLECULE:	WD REPEAT-CONTAINING PROTEIN 41
73:00:00	3ub0-D	4.3	8	70	194	7	MOLECULE:	NON-STRUCTURAL PROTEIN 6, NSP6,
74:00:00	7eeb-C	4.3	24	88	278	8	MOLECULE:	ENHANCED GREEN FLUORESCENT PROTEIN,CATION CH
75:00:00	6wcj-B	4.3	7.5	78	109	6	MOLECULE:	CLATHRIN HEAVY CHAIN 1
76:00:00	8ppr-M	4.3	8.9	116	205	5	MOLECULE:	KINETOCHORE-ASSOCIATED PROTEIN DSN1 HOMOLOG
77:00:00	7emf-D	4.3	9.4	107	158	16	MOLECULE:	MEDIATOR OF RNA POLYMERASE II TRANSCRIPTION
78:00:00	7c4j-F	4.3	20	114	383	9	MOLECULE:	TRANSCRIPTION REGULATORY PROTEIN SNF12
79:00:00	7oqe-K	4.3	23.2	120	406	8	MOLECULE:	PROTEIN NAM8
80:00:00	8q85-X	4.3	32.1	68	68	10	MOLECULE:	KINETOCHORE PROTEIN NDC80
81:00:00	7a5p-K	4.3	12.7	96	138	5	MOLECULE:	U2 SNRNA
82:00:00	4efa-E	4.3	9.1	127	225	6	MOLECULE:	V-TYPE PROTON ATPASE SUBUNIT C
83:00:00	7yg4-B	4.3	4	67	67	3	MOLECULE:	PROTEIN VIRILIZER HOMOLOG
84:00:00	7vdv-X	4.3	32.6	120	252	5	MOLECULE:	HISTONE H4
85:00:00	6wcj-E	4.3	9	80	109	8	MOLECULE:	CLATHRIN HEAVY CHAIN 1
86:00:00	5zuv-B	4.3	2.2	85	129	9	MOLECULE:	SPIKE GLYCOPROTEIN,INHIBITOR EK1
87:00:00	5zuv-A	4.3	2.4	86	130	10	MOLECULE:	SPIKE GLYCOPROTEIN,INHIBITOR EK1
88:00:00	6w2e-x	4.3	3.4	68	68	4	MOLECULE:	MAJOR CAPSID PROTEIN
89:00:00	3wpr-A	4.3	7.7	97	225	6	MOLECULE:	TRIMERIC AUTOTRANSPORTER ADHESIN
90:00:00	7aej-B	4.3	4.2	80	145	9	MOLECULE:	ENVELOPE GLYCOPROTEIN GP160,ENVELOPE GLYCOPR
91:00:00	6abo-A	4.2	9	108	213	7	MOLECULE:	DNA REPAIR PROTEIN XRCC4
92:00:00	8ovw-Z	4.2	6.6	99	151	13	MOLECULE:	CENTROMERE-BINDING PROTEIN 1
93:00:00	8q3v-b	4.2	3.9	57	70	2	MOLECULE:	TETRAHYDROMETHANOPTERIN S-METHYLTRANSFERASE
94:00:00	7zmg-n	4.2	9.2	69	136	13	MOLECULE:	NADH-UBIQUINONE OXIDOREDUCTASE CHAIN 1
95:00:00	5tsj-K	4.2	4.3	94	209	2	MOLECULE:	V-TYPE ATP SYNTHASE ALPHA CHAIN
96:00:00	5y05-A	4.2	9.3	99	233	14	MOLECULE:	MSMEG_4306
97:00:00	6j5j-b	4.2	14.7	140	209	9	MOLECULE:	ATP SYNTHASE SUBUNIT ALPHA, MITOCHONDRIAL
98:00:00	8u95-B	4.2	6.5	145	1009	7	MOLECULE:	MYOSIN HEAVY CHAIN, ISOFORM U
99:00:00	2d3e-C	4.2	8.6	102	130	5	MOLECULE:	GENERAL CONTROL PROTEIN GCN4 AND TROPOMYOSIN
0:00	5vox-M	4.2	44	76	209	13	MOLECULE:	V-TYPE PROTON ATPASE CATALYTIC SUBUNIT A,V-T
1:00	5gai-I	4.2	10.5	126	721	3	MOLECULE:	PORTAL PROTEIN
2:00	6ztq-Z	4.2	13	95	141	8	MOLECULE:	NADH-UBIQUINONE OXIDOREDUCTASE CHAIN 3
3:00	7tkk-U	4.2	12.7	119	155	6	MOLECULE:	ATP SYNTHASE SUBUNIT 9
4:00	8j9s-B	4.2	4.8	67	67	12	MOLECULE:	AMINOACYL TRNA SYNTHASE COMPLEX-INTERACTING
5:00	6qld-U	4.2	17.2	111	171	7	MOLECULE:	INNER KINETOCHORE SUBUNIT MIF2
6:00	6zkp-q	4.2	10.8	88	139	1	MOLECULE:	NADH DEHYDROGENASE [UBIQUINONE] FLAVOPROTEIN
7:00	6zkg-q	4.2	11.3	88	139	1	MOLECULE:	NADH DEHYDROGENASE [UBIQUINONE] FLAVOPROTEIN
8:00	6zks-q	4.2	10.7	88	139	1	MOLECULE:	NADH DEHYDROGENASE [UBIQUINONE] FLAVOPROTEIN
9:00	4jpp-B	4.2	9.3	94	127	11	MOLECULE:	MINOR SPIKE PROTEIN H
10:00	7mf3-H	4.2	4.5	101	211	6	MOLECULE:	MYOSIN-11
11:00	7vhq-G	4.2	14.6	106	299	3	MOLECULE:	ATP-DEPENDENT ZINC METALLOPROTEASE FTSH
12:00	6zke-q	4.2	10.8	88	139	1	MOLECULE:	NADH DEHYDROGENASE [UBIQUINONE] FLAVOPROTEIN
13:00	6z47-G	4.2	49.8	135	257	8	MOLECULE:	MYOSIN HEAVY CHAIN 11
14:00	2yo1-C	4.2	6	109	273	7	MOLECULE:	GENERAL CONTROL PROTEIN GCN4, PUTATIVE INNER
15:00	3wpr-B	4.2	6.7	104	207	4	MOLECULE:	TRIMERIC AUTOTRANSPORTER ADHESIN
16:00	2yny-B	4.2	4.6	82	104	7	MOLECULE:	GENERAL CONTROL PROTEIN GCN4, PUTATIVE INNER
17:00	5t58-B	4.1	11.4	112	190	8	MOLECULE:	KLLA0F02343P
18:00	8rt9-A	4.1	6.9	91	207	0	MOLECULE:	TRWJ PROTEIN
19:00	3p8c-D	4.1	42.8	107	204	5	MOLECULE:	CYTOPLASMIC FMR1-INTERACTING PROTEIN 1
20:00	7tch-A	4.1	16	72	645	6	MOLECULE:	BACITRACIN EXPORT PERMEASE PROTEIN BCEB

21:00	5ed9-B	4.1	4	72	72	6	MOLECULE:	SUN DOMAIN-CONTAINING PROTEIN 2
22:00	7egm-H	4.1	11.8	82	388	7	MOLECULE:	TRANSCRIPTION REGULATORY PROTEIN SNF2
23:00	8ppr-F	4.1	2.7	70	112	4	MOLECULE:	KINETOCHORE-ASSOCIATED PROTEIN DSN1 HOMOLOG
24:00:00	8i21-A	4.1	61.5	133	256	10	MOLECULE:	STRUCTURAL MAINTENANCE OF CHROMOSOMES PROTEI
25:00:00	7vc4-C	4.1	15.2	84	107	5	MOLECULE:	MITOCHONDRIAL IMPORT RECEPTOR SUBUNIT TOM6 H
26:00:00	4r3z-A	4.1	2.1	71	76	11	MOLECULE:	AMINOACYL TRNA SYNTHASE COMPLEX-INTERACTING
27:00:00	5j6f-A	4.1	13.8	59	352	10	MOLECULE:	3-DEOXY-D-ARABINO-HEPTULOSONATE 7-PHOSPHATE
28:00:00	8qfc-C	4.1	8.2	97	380	9	MOLECULE:	60S RIBOSOMAL PROTEIN L10A
29:00:00	8ap7-c	4.1	4.9	60	64	3	MOLECULE:	ATP SYNTHASE SUBUNIT A
30:00:00	7tmq-M	4.1	4.9	93	214	8	MOLECULE:	H(+)-TRANSPORTING TWO-SECTOR ATPASE
31:00:00	5y88-J	4.1	30.8	133	431	3	MOLECULE:	PRE-MRNA-SPLICING FACTOR 8
32:00:00	5bw9-l	4.1	4.4	66	66	9	MOLECULE:	V-TYPE PROTON ATPASE CATALYTIC SUBUNIT A
33:00:00	3zx6-B	4.1	8.5	112	303	12	MOLECULE:	HAMP, METHYL-ACCEPTING CHEMOTAXIS PROTEIN I
34:00:00	5lhg-A	4.1	50.7	84	666	10	MOLECULE:	LYSINE-SPECIFIC HISTONE DEMETHYLASE 1A
35:00:00	8akr-u	4.1	8.7	121	196	9	MOLECULE:	CHLOROPLAST MEMBRANE-ASSOCIATED 30 KD PROTEI
36:00:00	8at4-E	4.1	22.4	108	217	7	MOLECULE:	HAUS AUGMIN-LIKE COMPLEX SUBUNIT 1
37:00:00	6hgc-A	4.1	9.2	66	304	6	MOLECULE:	UBIQUITIN CARBOXYL-TERMINAL HYDROLASE CALYPS
38:00:00	7b93-Z	4.1	11.2	97	143	5	MOLECULE:	NADH-UBIQUINONE OXIDOREDUCTASE CHAIN 3
39:00:00	3jbh-B	4.1	25.7	104	964	12	MOLECULE:	MYOSIN 2 HEAVY CHAIN STRIATED MUSCLE
40:00:00	7uus-Q	4	8.3	64	170	8	MOLECULE:	HYDROGENASE-2, LARGE SUBUNIT
41:00:00	2pjw-H	4	7	65	88	6	MOLECULE:	UNCHARACTERIZED PROTEIN YHL002W
42:00:00	4orh-H	4	2.6	77	142	6	MOLECULE:	UBIQUITIN-CONJUGATING ENZYME E2 VARIANT 2
43:00:00	7v2w-J	4	13.9	126	236	6	MOLECULE:	THO COMPLEX SUBUNIT HPR1
44:00:00	4lws-A	4	5	61	100	3	MOLECULE:	UNCHARACTERIZED PROTEIN
45:00:00	8dft-A	4	2.3	46	112	15	MOLECULE:	PILIN PROTEIN
46:00:00	7qog-A	4	9.9	86	657	2	MOLECULE:	PORTAL PROTEIN GP20
47:00:00	3jb9-i	4	30.1	107	161	7	MOLECULE:	PRE-MRNA-SPLICING FACTOR SPP42
48:00:00	8aud-A	4	3.8	94	350	13	MOLECULE:	CELL WALL-ASSOCIATED HYDROLASES (INVASION-AS
49:00:00	8i03-E	4	16.6	115	169	9	MOLECULE:	PAIRED AMPHIPATHIC HELIX PROTEIN PST1
50:00:00	1y9b-A	4	6.4	66	81	8	MOLECULE:	CONSERVED HYPOTHETICAL PROTEIN
51:00:00	5o31-Z	4	12.4	88	137	6	MOLECULE:	NADH-UBIQUINONE OXIDOREDUCTASE CHAIN 3
52:00:00	6zls-A	4	15.2	105	292	7	MOLECULE:	HISTIDINE KINASE
53:00:00	7sqk-C	4	8	131	600	3	MOLECULE:	HAUS AUGMIN-LIKE COMPLEX SUBUNIT 1
54:00:00	7tmo-M	4	4.3	94	214	7	MOLECULE:	H(+)-TRANSPORTING TWO-SECTOR ATPASE
55:00:00	8gwk-B	4	13.2	90	187	4	MOLECULE:	RNA-DIRECTED RNA POLYMERASE
56:00:00	8c5v-S	4	5.3	117	516	6	MOLECULE:	CHEMOTAXIS PROTEIN CHEA
57:00:00	6m3w-B	4	40.4	132	344	2	MOLECULE:	SPIKE GLYCOPROTEIN
58:00:00	7z47-D	4	3.8	72	87	6	MOLECULE:	ADAPTOR PROTEIN
59:00:00	7apk-E	4	14.6	111	523	5	MOLECULE:	THO COMPLEX SUBUNIT 1
60:00:00	6pse-A	4	9.1	78	78	9	MOLECULE:	PROTEIN BICAUDAL D HOMOLOG 2
61:00:00	2e50-P	4	10.7	84	186	13	MOLECULE:	PROTEIN SET
62:00:00	4g2k-B	4	2.2	76	108	8	MOLECULE:	GENERAL CONTROL PROTEIN GCN4, ENVELOPE GLYCO
63:00:00	3lvg-D	3.9	10.8	88	180	8	MOLECULE:	CLATHRIN HEAVY CHAIN 1
64:00:00	6r7l-E	3.9	14.4	72	98	3	MOLECULE:	SECG
65:00:00	5nnv-D	3.9	5.1	120	252	7	MOLECULE:	CHROMOSOME PARTITION PROTEIN SMC,CHROMOSOME
66:00:00	4cjd-A	3.9	17.4	80	123	8	MOLECULE:	NADA
67:00:00	7x2e-A	3.9	10.3	96	163	9	MOLECULE:	HARMONIN
68:00:00	6cv9-A	3.9	11.9	57	316	11	MOLECULE:	SHORT TRANSIENT RECEPTOR POTENTIAL CHANNEL 6
69:00:00	5xbt-A	3.9	10.4	75	271	11	MOLECULE:	PROBABLE TRANSCRIPTIONAL REGULATOR
70:00:00	8x5f-A	3.9	32.1	90	616	10	MOLECULE:	SOLUTE CARRIER FAMILY 53 MEMBER 1
71:00:00	8hmy-B	3.9	13.8	77	264	4	MOLECULE:	TRNA-SPLICING ENDONUCLEASE SUBUNIT SEN2

72:00:00	2p4w-B	3.9	13.8	64	198	16	MOLECULE:	TRANSCRIPTIONAL REGULATORY PROTEIN ARSR FAMI
73:00:00	8fck-E	3.9	35.3	142	222	4	MOLECULE:	HAUS AUGMIN-LIKE COMPLEX SUBUNIT 1
74:00:00	6ly8-G	3.9	4.4	94	210	7	MOLECULE:	V-TYPE ATP SYNTHASE ALPHA CHAIN
75:00:00	5nt1-E	3.9	5	85	173	8	MOLECULE:	E3 UBIQUITIN/ISG15 LIGASE TRIM25
76:00:00	3lj5-G	3.9	8.4	134	692	7	MOLECULE:	PORTAL PROTEIN
77:00:00	7yo1-B	3.9	18.1	72	263	10	MOLECULE:	CALCIUM-ACTIVATED POTASSIUM CHANNEL SUBUNIT
78:00:00	8jsm-D	3.9	3.3	68	68	9	MOLECULE:	RNA-DIRECTED RNA POLYMERASE L
79:00:00	4lin-K	3.9	34.1	123	289	10	MOLECULE:	TAIL NEEDLE PROTEIN GP26
80:00:00	3wpo-A	3.9	6.7	102	193	5	MOLECULE:	TRIMERIC AUTOTRANSPORTER ADHESIN
81:00:00	7emf-I	3.8	6.2	67	73	3	MOLECULE:	MEDIATOR OF RNA POLYMERASE II TRANSCRIPTION
82:00:00	7qoo-Q	3.8	17.2	97	209	6	MOLECULE:	CENTROMERE PROTEIN C
83:00:00	6gmh-M	3.8	14.5	53	991	8	MOLECULE:	RPB1
84:00:00	7emf-G	3.8	4.1	79	161	9	MOLECULE:	MEDIATOR OF RNA POLYMERASE II TRANSCRIPTION
85:00:00	7uxc-R	3.8	18.8	57	107	7	MOLECULE:	SERINE/THREONINE-PROTEIN KINASE MTOR
86:00:00	1z56-A	3.8	2.7	76	77	5	MOLECULE:	LIGASE INTERACTING FACTOR 1
87:00:00	6yvu-A	3.8	57.1	149	1127	9	MOLECULE:	STRUCTURAL MAINTENANCE OF CHROMOSOMES PROTEI
88:00:00	4wpe-A	3.8	4.7	113	275	6	MOLECULE:	CYTOKINESIS PROTEIN 2
89:00:00	6gmh-Q	3.8	9.5	101	884	13	MOLECULE:	RPB1
90:00:00	3eff-K	3.8	3.8	81	139	10	MOLECULE:	FAB
91:00:00	6qld-Y	3.8	14.2	129	223	9	MOLECULE:	INNER KINETOCHORE SUBUNIT MIF2
92:00:00	5n9j-E	3.8	6.9	85	201	9	MOLECULE:	MEDIATOR OF RNA POLYMERASE II TRANSCRIPTION
93:00:00	7p3w-p	3.8	11.8	107	155	9	MOLECULE:	ATP SYNTHASE SUBUNIT ALPHA
94:00:00	5w9a-A	3.8	10.3	96	193	5	MOLECULE:	TRIPARTITE MOTIF-CONTAINING PROTEIN 5
95:00:00	6gbq-G	3.8	4.7	66	66	6	MOLECULE:	POLYMERASE COFACTOR VP35
96:00:00	5lki-D	3.8	11.5	146	1427	5	MOLECULE:	TCDA1
97:00:00	5vwl-A	3.8	9.4	82	105	7	MOLECULE:	CYTOPLASMIC TAIL OF HIV-1 GP41 PROTEIN
98:00:00	4lin-E	3.8	10.3	139	289	6	MOLECULE:	TAIL NEEDLE PROTEIN GP26
99:00:00	5t96-B	3.7	5.4	68	330	4	MOLECULE:	HE PROTEIN
0:00	5y06-A	3.7	12	101	235	16	MOLECULE:	MSMEG_4306
1:00	4aur-A	3.7	10.3	71	564	8	MOLECULE:	LEOA
2:00	2ehw-C	3.7	2.1	47	117	9	MOLECULE:	HYPOTHETICAL PROTEIN TTHB059
3:00	8q85-Z	3.7	5.8	80	94	5	MOLECULE:	KINETOCHORE PROTEIN NDC80
4:00	6kn7-U	3.7	7.8	108	170	8	MOLECULE:	ACTIN, ALPHA SKELETAL MUSCLE
5:00	8x2q-A	3.7	5	63	109	6	MOLECULE:	ADENOMATOUS POLYPOSIS COLI PROTEIN
6:00	4om3-D	3.7	13.5	84	122	1	MOLECULE:	TRANSDUCIN-LIKE ENHANCER PROTEIN 1
7:00	2ve7-D	3.7	8	98	242	4	MOLECULE:	KINETOCHORE PROTEIN HEC1, KINETOCHORE PROTEI
8:00	6wuc-H	3.7	16.2	83	179	8	MOLECULE:	INNER KINETOCHORE SUBUNIT MCM16
9:00	8e4c-C	3.7	9.6	73	142	10	MOLECULE:	ISOFORM 2 OF IMMUNOGLOBULIN HEAVY CONSTANT M
10:00	2p22-C	3.7	8.5	107	186	5	MOLECULE:	SUPPRESSOR PROTEIN STP22 OF TEMPERATURE-SENS
11:00	8t1l-W	3.7	15.3	71	119	3	MOLECULE:	MEDIATOR OF RNA POLYMERASE II TRANSCRIPTION
12:00	4tt0-B	3.7	7.3	87	131	3	MOLECULE:	DENEDDYLASE
13:00	8th8-B	3.7	37	145	276	8	MOLECULE:	DYNEIN REGULATORY COMPLEX PROTEIN 1/2 N-TERM
14:00	7sc0-A	3.7	16.5	78	129	10	MOLECULE:	CAVEOLIN-1
15:00	8ixk-E	3.7	3	61	109	8	MOLECULE:	ATTACHMENT PROTEIN G3P
16:00	5xe3-E	3.7	10.3	65	81	9	MOLECULE:	ENDORIBONUCLEASE MAZF4
17:00	5ip0-D	3.7	1.5	51	108	2	MOLECULE:	PHA GRANULE-ASSOCIATED PROTEIN
18:00	3vou-A	3.7	5	68	139	7	MOLECULE:	ION TRANSPORT 2 DOMAIN PROTEIN, VOLTAGE-GATE
19:00	8tkp-A	3.7	29.1	68	614	9	MOLECULE:	TRANSMEMBRANE CHANNEL-LIKE PROTEIN 2
20:00	4mu6-A	3.7	4	98	274	6	MOLECULE:	KINECTIN 1 (KINESIN RECEPTOR)
21:00	6z6o-C	3.7	11.7	109	548	6	MOLECULE:	HISTONE DEACETYLASE HDA1
22:00	6f5d-G	3.7	7.2	68	277	4	MOLECULE:	ATP SYNTHASE ALPHA CHAIN, MITOCHONDRIAL

23:00	8tdj-A	3.7	31.8	70	467	11	MOLECULE:	MECHANOSENSITIVE ION CHANNEL PROTEIN 10
24:00:00	7kon-T	3.7	16.2	99	126	5	MOLECULE:	ACTIN, ALPHA SKELETAL MUSCLE
25:00:00	6vy1-F	3.7	3.3	47	121	4	MOLECULE:	PREFOLDIN SUBUNIT ALPHA 2
26:00:00	7e9t-A	3.7	10.5	111	371	3	MOLECULE:	SPIKE PROTEIN S2
27:00:00	5uhq-D	3.7	5.6	56	81	5	MOLECULE:	SUGAR TRANSPORTER SEMISWEET
28:00:00	3zmf-C	3.7	6.6	85	111	6	MOLECULE:	GENERAL CONTROL PROTEIN GCN4, PUTATIVE INNER
29:00:00	2yny-C	3.7	6	80	105	9	MOLECULE:	GENERAL CONTROL PROTEIN GCN4, PUTATIVE INNER
30:00:00	2yo1-A	3.7	6.8	103	273	11	MOLECULE:	GENERAL CONTROL PROTEIN GCN4, PUTATIVE INNER
31:00:00	3zmf-B	3.7	4.8	81	111	7	MOLECULE:	GENERAL CONTROL PROTEIN GCN4, PUTATIVE INNER
32:00:00	2efl-A	3.6	3.7	121	281	2	MOLECULE:	FORMIN-BINDING PROTEIN 1
33:00:00	7p64-L	3.6	4.8	84	606	4	MOLECULE:	NADH-QUINONE OXIDOREDUCTASE SUBUNIT F
34:00:00	8q3v-g	3.6	7.4	71	76	7	MOLECULE:	TETRAHYDROMETHANOPTERIN S-METHYLTRANSFERASE
35:00:00	5oxf-A	3.6	9.6	120	703	5	MOLECULE:	GTP-BINDING PROTEIN
36:00:00	9f63-A	3.6	24	82	422	5	MOLECULE:	PROTEIN PNS1
37:00:00	5c1f-A	3.6	4.6	123	298	7	MOLECULE:	SEPTATION PROTEIN IMP2
38:00:00	7jtk-C	3.6	12.2	122	427	8	MOLECULE:	FLAGELLAR RADIAL SPOKE PROTEIN 1
39:00:00	5odw-D	3.6	2.9	65	198	9	MOLECULE:	FERRIPYOVERDINE RECEPTOR
40:00:00	4gcz-B	3.6	7.2	82	378	7	MOLECULE:	BLUE-LIGHT PHOTORECEPTOR, SENSOR PROTEIN FIX
41:00:00	8qfc-D	3.6	13.5	103	185	10	MOLECULE:	60S RIBOSOMAL PROTEIN L10A
42:00:00	7tmp-M	3.6	4.4	94	214	7	MOLECULE:	H(+)-TRANSPORTING TWO-SECTOR ATPASE
43:00:00	7tmq-H	3.6	7.1	51	51	6	MOLECULE:	H(+)-TRANSPORTING TWO-SECTOR ATPASE
44:00:00	6sct-O	3.6	2.6	59	59	5	MOLECULE:	CLATHRIN HEAVY CHAIN
45:00:00	6sct-J	3.6	2.6	59	59	5	MOLECULE:	CLATHRIN HEAVY CHAIN
46:00:00	2yo1-B	3.6	8.7	114	273	10	MOLECULE:	GENERAL CONTROL PROTEIN GCN4, PUTATIVE INNER
47:00:00	2yo2-A	3.6	22.5	95	160	7	MOLECULE:	GENERAL CONTROL PROTEIN GCN4, PUTATIVE INNER
48:00:00	3wqa-C	3.6	28.6	86	201	7	MOLECULE:	TRIMERIC AUTOTRANSPORTER ADHESIN
49:00:00	7zr1-D	3.5	10	130	780	6	MOLECULE:	DOUBLE-STRAND BREAK REPAIR PROTEIN
50:00:00	2v0o-A	3.5	5.2	115	273	6	MOLECULE:	FCH DOMAIN ONLY PROTEIN 2
51:00:00	7nvr-n	3.5	12.1	93	132	11	MOLECULE:	TFIIH BASAL TRANSCRIPTION FACTOR COMPLEX HEL
52:00:00	8wjn-A	3.5	38.2	103	491	10	MOLECULE:	STRUCTURAL MAINTENANCE OF CHROMOSOMES PROTEI
53:00:00	6ted-R	3.5	12.8	82	244	5	MOLECULE:	DNA-DIRECTED RNA POLYMERASE SUBUNIT
54:00:00	7vf2-C	3.5	35.1	102	184	7	MOLECULE:	PROTEIN VIRILIZER HOMOLOG
55:00:00	8d8k-V	3.5	15.5	113	233	11	MOLECULE:	PROBABLE S-ADENOSYL-L-METHIONINE-DEPENDENT R
56:00:00	3jac-A	3.5	39.9	103	918	3	MOLECULE:	PIEZO-TYPE MECHANOSENSITIVE ION CHANNEL COMP
57:00:00	8rhn-A	3.5	3.9	64	198	13	MOLECULE:	ATPASE FAMILY GENE 2 PROTEIN HOMOLOG A
58:00:00	6sp2-A	3.5	9.7	59	366	7	MOLECULE:	MEMBRANE PROTEIN TMS1D
59:00:00	8cbk-F	3.5	34.3	87	344	6	MOLECULE:	3-HYDROXYACYL-COA DEHYDROGENASE TYPE-2
60:00:00	6vq7-H	3.5	3.7	94	214	10	MOLECULE:	ATPASE H+-TRANSPORTING V1 SUBUNIT A
61:00:00	4iff-C	3.5	2.3	66	82	8	MOLECULE:	FUSION OF PHAGE PHI29 GP7 PROTEIN AND CELL D
62:00:00	4mh6-A	3.5	3.6	85	159	6	MOLECULE:	PUTATIVE TYPE III SECRETION PROTEIN YSCO
63:00:00	6g72-Z	3.5	11.9	91	139	7	MOLECULE:	NADH-UBIQUINONE OXIDOREDUCTASE CHAIN 3
64:00:00	1jch-C	3.5	15.3	82	468	12	MOLECULE:	COLICIN E3
65:00:00	6w1s-W	3.5	20.2	89	118	10	MOLECULE:	MEDIATOR OF RNA POLYMERASE II TRANSCRIPTION
66:00:00	5lki-E	3.5	11.5	145	1427	6	MOLECULE:	TCDA1
67:00:00	7pgg-A	3.5	3.3	77	147	12	MOLECULE:	ION TRANSPORT PROTEIN
68:00:00	8ia3-E	3.5	7.6	87	111	5	MOLECULE:	UPSTREAM STIMULATORY FACTOR 2
69:00:00	6sct-E	3.5	1.7	59	59	8	MOLECULE:	CLATHRIN HEAVY CHAIN
70:00:00	5ylz-r	3.5	4.7	63	125	3	MOLECULE:	PRE-MRNA-SPLICING FACTOR 8
71:00:00	6xl0-P	3.5	9.4	82	271	7	MOLECULE:	FLAGELLIN
72:00:00	8ebt-K	3.4	10.6	84	172	10	MOLECULE:	GENERAL TRANSCRIPTION AND DNA REPAIR FACTOR
73:00:00	7zu0-F	3.4	8.5	59	112	8	MOLECULE:	E3 UBIQUITIN-PROTEIN LIGASE PEP5

74:00:00	5oqm-n	3.4	15.1	93	136	5	MOLECULE:	DNA-DIRECTED RNA POLYMERASE II SUBUNIT RPB1
75:00:00	8gb3-F	3.4	4.3	70	90	13	MOLECULE:	CHAPERONE PROTEIN DNAK
76:00:00	8cr1-C	3.4	7.8	73	266	3	MOLECULE:	ATPASE ASNA1
77:00:00	3p8c-F	3.4	10.6	105	156	8	MOLECULE:	CYTOPLASMIC FMR1-INTERACTING PROTEIN 1
78:00:00	8b9z-h	3.4	8.7	84	145	12	MOLECULE:	NADH-UBIQUINONE OXIDOREDUCTASE CHAIN 3
79:00:00	7jrg-G	3.4	9.4	64	70	5	MOLECULE:	MITOCHONDRIAL-PROCESSING PEPTIDASE SUBUNIT B
80:00:00	5wkq-A	3.4	3.7	85	166	7	MOLECULE:	INVASIN IPAB
81:00:00	8b9z-Z	3.4	33.6	100	146	7	MOLECULE:	NADH-UBIQUINONE OXIDOREDUCTASE CHAIN 3
82:00:00	5n9j-U	3.4	23	103	198	3	MOLECULE:	MEDIATOR OF RNA POLYMERASE II TRANSCRIPTION
83:00:00	8h36-G	3.4	7.8	79	379	4	MOLECULE:	E3 UBIQUITIN-PROTEIN LIGASE RBX1
84:00:00	4cgk-A	3.4	2.4	84	351	5	MOLECULE:	SECRETED 45 KDA PROTEIN
85:00:00	4cq4-A	3.4	3.7	78	106	10	MOLECULE:	ENGINEERED VERSION OF TRANSMEMBRANE RECEPTOR
86:00:00	4zwt-N	3.4	3.6	61	125	10	MOLECULE:	RECOMBINATION PROTEIN UVSY
87:00:00	6qum-G	3.4	3.8	93	207	9	MOLECULE:	V-TYPE ATP SYNTHASE ALPHA CHAIN
88:00:00	7fde-M	3.4	5.6	104	218	7	MOLECULE:	V-TYPE PROTON ATPASE SUBUNIT C
89:00:00	5sxi-B	3.4	3.3	75	209	3	MOLECULE:	PHOSPHATIDYLINOSITOL 4,5-BISPHOSPHATE 3-KINA
90:00:00	5sxf-B	3.4	3.6	72	183	13	MOLECULE:	PHOSPHATIDYLINOSITOL 4,5-BISPHOSPHATE 3-KINA
91:00:00	6gol-A	3.4	2.7	45	65	2	MOLECULE:	HEMAGGLUTININ TRI-STALK
92:00:00	8h37-J	3.4	5.1	79	168	8	MOLECULE:	CULLIN-3
93:00:00	8pjn-i	3.4	6	60	395	3	MOLECULE:	E3 UBIQUITIN-PROTEIN TRANSFERASE RMND5A
94:00:00	8t1i-B	3.4	11.4	92	158	8	MOLECULE:	MEDIATOR OF RNA POLYMERASE II TRANSCRIPTION
95:00:00	5b83-B	3.4	2.4	58	59	3	MOLECULE:	TETRA UBIQUITIN
96:00:00	5ip0-A	3.4	3.7	57	107	5	MOLECULE:	PHA GRANULE-ASSOCIATED PROTEIN
97:00:00	8h37-G	3.4	3.4	79	168	11	MOLECULE:	CULLIN-3
98:00:00	6h2f-B	3.4	5.5	112	344	7	MOLECULE:	AHLB
99:00:00	6e95-A	3.4	16.4	74	240	14	MOLECULE:	STAPHYLOCOCCUS AUREUS AGRC HISTIDINE KINASE
0:00	7aej-A	3.4	7.3	72	129	8	MOLECULE:	ENVELOPE GLYCOPROTEIN GP160,ENVELOPE GLYCOPR
1:00	5jeq-A	3.3	4.9	88	227	10	MOLECULE:	NITRATE/NITRITE SENSOR PROTEIN NARQ
2:00	8afz-B	3.3	2.4	74	376	9	MOLECULE:	SORTING NEXIN-1
3:00	7wkk-l	3.3	4.9	88	171	10	MOLECULE:	MGC83295 PROTEIN
4:00	5n9j-A	3.3	24.9	124	566	4	MOLECULE:	MEDIATOR OF RNA POLYMERASE II TRANSCRIPTION
5:00	6ikn-A	3.3	4.1	115	305	6	MOLECULE:	GROWTH ARREST-SPECIFIC PROTEIN 7
6:00	8gju-F	3.3	12.9	81	332	4	MOLECULE:	METHYLMALONIC ACIDURIA TYPE A PROTEIN, MITOC
7:00	3mhh-C	3.3	10.3	65	93	6	MOLECULE:	UBIQUITIN CARBOXYL-TERMINAL HYDROLASE 8
8:00	6bpz-A	3.3	39.8	107	900	5	MOLECULE:	PIEZO-TYPE MECHANOSENSITIVE ION CHANNEL COMP
9:00	8tek-E	3.3	15.8	116	219	7	MOLECULE:	DYNEIN REGULATORY COMPLEX PROTEIN 1/2 N-TERM
10:00	7w5a-X	3.3	10.3	87	87	0	MOLECULE:	PRE-MRNA-PROCESSING-SPLICING FACTOR 8
11:00	8fef-D	3.3	25.2	139	385	6	MOLECULE:	VIRULENCE FACTOR MCE FAMILY PROTEIN
12:00	5j9u-G	3.3	9.9	59	297	8	MOLECULE:	HISTONE ACETYLTRANSFERASE ESA1
13:00	7xzj-G	3.3	2.6	53	73	9	MOLECULE:	CTAP3
14:00	8i4t-R	3.3	12.3	103	501	2	MOLECULE:	ENVELOPMENT POLYPROTEIN
15:00	8pmq-9	3.3	10	68	412	4	MOLECULE:	E3 UBIQUITIN-PROTEIN LIGASE RMD5
16:00	6cnn-A	3.3	38.8	68	360	3	MOLECULE:	INTERMEDIATE CONDUCTANCE CALCIUM-ACTIVATED P
17:00	8t1i-F	3.3	5	60	73	13	MOLECULE:	MEDIATOR OF RNA POLYMERASE II TRANSCRIPTION
18:00	7emf-K	3.3	14.2	82	112	6	MOLECULE:	MEDIATOR OF RNA POLYMERASE II TRANSCRIPTION
19:00	5yfp-F	3.3	16.3	137	725	8	MOLECULE:	EXOCYST COMPLEX COMPONENT SEC3
20:00	7aqw-m	3.3	8.8	60	70	7	MOLECULE:	NADH-UBIQUINONE OXIDOREDUCTASE CHAIN 5
21:00	6oee-A	3.3	36.4	110	243	7	MOLECULE:	TYPE IV SECRETION SYSTEM APPARATUS PROTEIN C
22:00	6rd4-5	3.3	5.8	99	123	5	MOLECULE:	ASA-10: POLYTOMELLA F-ATP SYNTHASE ASSOCIATE
23:00	6lom-A	3.3	19.5	98	295	5	MOLECULE:	CALCIUM HOMEOSTASIS MODULATOR PROTEIN
24:00:00	7zqy-B	3.3	4	74	177	9	MOLECULE:	DH DOMAIN-CONTAINING PROTEIN

25:00:00	6r0w-G	3.3	3.7	93	207	9	MOLECULE:	V-TYPE ATP SYNTHASE ALPHA CHAIN
26:00:00	5sxc-B	3.3	4.5	77	208	10	MOLECULE:	PHOSPHATIDYLINOSITOL 4,5-BISPHOSPHATE 3-KINA
27:00:00	6wm2-G	3.3	4.4	97	213	11	MOLECULE:	V-TYPE PROTON ATPASE SUBUNIT E 1
28:00:00	5knb-G	3.3	4	79	164	8	MOLECULE:	V-TYPE SODIUM ATPASE CATALYTIC SUBUNIT A
29:00:00	6r0y-G	3.3	3.2	91	206	9	MOLECULE:	V-TYPE ATP SYNTHASE ALPHA CHAIN
30:00:00	6wm4-G	3.3	4.2	97	213	11	MOLECULE:	V-TYPE PROTON ATPASE 116 KDA SUBUNIT A ISOFO
31:00:00	5sxe-B	3.3	3.3	73	225	3	MOLECULE:	PHOSPHATIDYLINOSITOL 4,5-BISPHOSPHATE 3-KINA
32:00:00	6w1s-Q	3.3	8.4	78	131	12	MOLECULE:	MEDIATOR OF RNA POLYMERASE II TRANSCRIPTION
33:00:00	6o7v-a	3.3	36.2	83	625	4	MOLECULE:	V-TYPE PROTON ATPASE SUBUNIT D
34:00:00	1ujw-B	3.3	4.2	62	115	8	MOLECULE:	VITAMIN B12 RECEPTOR
35:00:00	6m3w-C	3.3	40.4	132	344	2	MOLECULE:	SPIKE GLYCOPROTEIN
36:00:00	5j9t-L	3.3	4.2	60	119	10	MOLECULE:	HISTONE ACETYLTRANSFERASE ESA1
37:00:00	5lgt-A	3.3	51.8	86	666	10	MOLECULE:	LYSINE-SPECIFIC HISTONE DEMETHYLASE 1A
38:00:00	8pmq-2	3.3	7.2	84	355	8	MOLECULE:	E3 UBIQUITIN-PROTEIN LIGASE RMD5
39:00:00	6ip1-A	3.3	5	63	63	5	MOLECULE:	VESICLE-ASSOCIATED MEMBRANE PROTEIN 2
40:00:00	8bjw-F	3.2	5.2	57	97	12	MOLECULE:	PROTEIN C
41:00:00	5wlz-C	3.2	12	100	206	7	MOLECULE:	DNA REPAIR PROTEIN XRCC4,MYOSIN-7
42:00:00	4p1m-B	3.2	2.9	61	107	10	MOLECULE:	CELL DIVISION PROTEIN ZAPA
43:00:00	1kmi-Z	3.2	9.5	97	177	8	MOLECULE:	CHEMOTAXIS PROTEIN CHEY
44:00:00	4h8s-C	3.2	12	122	382	3	MOLECULE:	DCC-INTERACTING PROTEIN 13-BETA
45:00:00	5vjx-J	3.2	4.3	52	64	12	MOLECULE:	CLOCK-INTERACTING PACEMAKER
46:00:00	3na7-A	3.2	12.8	100	237	13	MOLECULE:	HP0958
47:00:00	1llm-C	3.2	5.2	54	87	4	MOLECULE:	5'-D(*TP*CP*CP*CP*AP*CP*GP*CP*GP*TP*GP*GP*G)
48:00:00	4f52-E	3.2	23.6	99	523	7	MOLECULE:	CULLIN-1
49:00:00	5vk5-B	3.2	4.1	61	282	5	MOLECULE:	POTASSIUM CHANNEL SUBFAMILY K MEMBER 2
50:00:00	7x6g-D	3.2	7.2	79	152	0	MOLECULE:	QUORUM-SENSING REGULATOR PROTEIN G
51:00:00	7w5a-2	3.2	1.8	61	123	18	MOLECULE:	PRE-MRNA-PROCESSING-SPLICING FACTOR 8
52:00:00	4jio-B	3.2	5.5	59	320	7	MOLECULE:	BRO1
53:00:00	7nb6-A	3.2	26.6	70	340	9	MOLECULE:	AI-2 TRANSPORT PROTEIN TQSA
54:00:00	5l4k-S	3.2	10.4	83	491	12	MOLECULE:	26S PROTEASOME NON-ATPASE REGULATORY SUBUNIT
55:00:00	8hhf-B	3.2	3.9	68	89	6	MOLECULE:	CELL DIVISION PROTEIN FTSQ
56:00:00	6djl-C	3.2	28.7	109	224	5	MOLECULE:	RAS-RELATED PROTEIN RAB-11A
57:00:00	6ek8-A	3.2	4.7	82	249	9	MOLECULE:	YAXB
58:00:00	4ke2-B	3.2	5.3	91	196	8	MOLECULE:	TYPE I HYPERACTIVE ANTIFREEZE PROTEIN
59:00:00	6qum-J	3.2	11.3	93	185	12	MOLECULE:	V-TYPE ATP SYNTHASE ALPHA CHAIN
60:00:00	1ebo-A	3.2	5.1	77	112	6	MOLECULE:	EBOLA VIRUS ENVELOPE PROTEIN CHIMERA CONSIST
61:00:00	8v8h-D	3.2	3.5	81	239	6	MOLECULE:	PHOSPHATIDYLINOSITOL 4,5-BISPHOSPHATE 3-KINA
62:00:00	4egx-A	3.2	5.3	67	180	10	MOLECULE:	KINESIN-LIKE PROTEIN KIF1A
63:00:00	3zmf-A	3.2	3.7	79	111	5	MOLECULE:	GENERAL CONTROL PROTEIN GCN4, PUTATIVE INNER
64:00:00	6mdm-H	3.1	20.4	79	147	8	MOLECULE:	VESICLE-FUSING ATPASE
65:00:00	8c5v-I	3.1	8.7	141	516	13	MOLECULE:	CHEMOTAXIS PROTEIN CHEA
66:00:00	5yfp-A	3.1	36.1	93	672	8	MOLECULE:	EXOCYST COMPLEX COMPONENT SEC3
67:00:00	5twv-B	3.1	11	86	1375	5	MOLECULE:	ATP-SENSITIVE INWARD RECTIFIER POTASSIUM CHA
68:00:00	8ftk-A	3.1	26.9	106	1584	8	MOLECULE:	5'-3' RNA HELICASE-LIKE PROTEIN
69:00:00	1esx-A	3.1	16.6	72	96	7	MOLECULE:	VPR PROTEIN
70:00:00	4dyl-A	3.1	4.4	115	376	8	MOLECULE:	TYROSINE-PROTEIN KINASE FES/FPS
71:00:00	8ikg-R	3.1	8.4	61	275	5	MOLECULE:	GUANINE NUCLEOTIDE-BINDING PROTEIN G(I) SUBU
72:00:00	6v8o-C	3.1	8.6	53	60	9	MOLECULE:	HIGH TEMPERATURE LETHAL PROTEIN 1
73:00:00	8b6j-k	3.1	6.3	57	58	9	MOLECULE:	PEPTIDASE M16 INACTIVE DOMAIN PROTEIN
74:00:00	8e1m-C	3.1	17.4	70	264	6	MOLECULE:	MITOCHONDRIAL IMPORT INNER MEMBRANE TRANSLOC
75:00:00	7oi8-q	3.1	16.5	109	164	4	MOLECULE:	39S RIBOSOMAL PROTEIN L2, MITOCHONDRIAL

76:00:00	8ppr-D	3.1	16.4	129	248	8	MOLECULE:	KINETOCHORE-ASSOCIATED PROTEIN DSN1 HOMOLOG
77:00:00	7m2w-K	3.1	15.4	88	95	9	MOLECULE:	TUBULIN GAMMA CHAIN
78:00:00	1ezj-A	3.1	7.3	72	114	7	MOLECULE:	NUCLEOCAPSID PHOSPHOPROTEIN
79:00:00	8j8p-R	3.1	10.7	60	69	2	MOLECULE:	CTR9-LIKE PROTEIN
80:00:00	8u7i-A	3.1	54.6	94	626	12	MOLECULE:	ENDONUCLEASE GAJA
81:00:00	3f6n-B	3.1	21.9	70	74	7	MOLECULE:	VIRION-ASSOCIATED PROTEIN
82:00:00	6nct-B	3.1	3.2	72	225	3	MOLECULE:	PHOSPHATIDYLINOSITOL 4,5-BISPHOSPHATE 3-KINA
83:00:00	5nen-B	3.1	3.7	91	213	15	MOLECULE:	LIPASE C
84:00:00	6el1-Q	3.1	5	112	332	6	MOLECULE:	YAXA
85:00:00	2yo3-C	3.1	11.5	125	257	6	MOLECULE:	GENERAL CONTROL PROTEIN GCN4, PUTATIVE INNER
86:00:00	6grj-A	3.1	5.7	108	338	7	MOLECULE:	AHLB
87:00:00	5lm1-A	3.1	11.6	118	339	6	MOLECULE:	TYROSINE-PROTEIN PHOSPHATASE NON-RECEPTOR TY
88:00:00	5n9j-G	3.1	18.2	110	163	5	MOLECULE:	MEDIATOR OF RNA POLYMERASE II TRANSCRIPTION
89:00:00	1fzb-A	3.1	2.3	55	81	9	MOLECULE:	FIBRINOGEN
90:00:00	7yo1-H	3.1	21.2	68	263	7	MOLECULE:	CALCIUM-ACTIVATED POTASSIUM CHANNEL SUBUNIT
91:00:00	5lki-C	3.1	11.5	145	1427	6	MOLECULE:	TCDA1
92:00:00	7tke-U	3.1	8.3	100	155	11	MOLECULE:	ATP SYNTHASE SUBUNIT 9
93:00:00	2hod-D	3.1	3.7	46	71	7	MOLECULE:	FIBRINOGEN ALPHA CHAIN
94:00:00	5vox-J	3.1	10.8	82	104	9	MOLECULE:	V-TYPE PROTON ATPASE CATALYTIC SUBUNIT A,V-T
95:00:00	5ip0-P	3.1	3.3	55	108	5	MOLECULE:	PHA GRANULE-ASSOCIATED PROTEIN
96:00:00	6el1-O	3.1	61	116	332	9	MOLECULE:	YAXA
97:00:00	6vy2-D	3.1	3.9	44	121	9	MOLECULE:	GLYCOPROTEIN 120
98:00:00	6vy2-F	3.1	3.9	44	121	9	MOLECULE:	GLYCOPROTEIN 120
99:00:00	6vy2-B	3.1	3.9	44	121	9	MOLECULE:	GLYCOPROTEIN 120
0:00	1y2o-A	3	5.6	99	248	5	MOLECULE:	BAI1-ASSOCIATED PROTEIN 2 ISOFORM 1
1:00	4nqi-D	3	5	97	232	7	MOLECULE:	SH3 DOMAIN-CONTAINING PROTEIN
2:00	4tko-B	3	37.6	112	326	6	MOLECULE:	EMRA
3:00	6em5-t	3	16.9	77	290	8	MOLECULE:	5.8S RIBOSOMAL RNA
4:00	8ap7-m	3	9.8	59	129	5	MOLECULE:	ATP SYNTHASE SUBUNIT A
5:00	8hqo-x	3	7.3	77	80	8	MOLECULE:	PORTAL PROTEIN
6:00	6djl-E	3	28.9	89	226	3	MOLECULE:	RAS-RELATED PROTEIN RAB-11A
7:00	2kn8-A	3	10	68	68	7	MOLECULE:	DNA CLEAVAGE AND PACKAGING PROTEIN LARGE SUB
8:00	7b93-L	3	22.2	94	607	9	MOLECULE:	NADH-UBIQUINONE OXIDOREDUCTASE CHAIN 3
9:00	6c0f-8	3	9	71	98	10	MOLECULE:	SACCHAROMYCES CEREVISIAE S288C 35S PRE-RIBOS
10:00	8q85-d	3	21.1	72	108	11	MOLECULE:	KINETOCHORE PROTEIN NDC80
11:00	2py8-C	3	7.2	61	123	8	MOLECULE:	HYPOTHETICAL PROTEIN RBCX
12:00	8t1l-X	3	11.2	72	127	13	MOLECULE:	MEDIATOR OF RNA POLYMERASE II TRANSCRIPTION
13:00	8p1u-C	3	9.9	82	93	5	MOLECULE:	CELL DIVISION PROTEIN FTSL
14:00	1ox3-A	3	3.1	60	108	12	MOLECULE:	FIBRITIN
15:00	8kde-3	3	11	90	97	7	MOLECULE:	PHOTOSYSTEM II CP47 REACTION CENTER PROTEIN
16:00	8i4u-A	3	7.7	124	453	6	MOLECULE:	STRUCTURAL MAINTENANCE OF CHROMOSOMES PROTEI
17:00	6el1-S	3	5	112	332	6	MOLECULE:	YAXA
18:00	6r0z-J	3	11.5	88	185	14	MOLECULE:	V-TYPE ATP SYNTHASE ALPHA CHAIN
19:00	1jad-A	3	3.7	95	242	7	MOLECULE:	PHOSPHOLIPASE C BETA
20:00	4mvd-D	3	5	100	253	6	MOLECULE:	CHOLINE-PHOSPHATE CYTIDYLYLTRANSFERASE A
21:00	3uum-B	3	2.3	51	123	10	MOLECULE:	UTROPHIN
22:00	1qce-B	3	3.6	62	123	15	MOLECULE:	PROTEIN (GP41)
23:00	1qce-C	3	3.6	62	123	15	MOLECULE:	PROTEIN (GP41)
24:00:00	6el1-N	3	5	112	332	6	MOLECULE:	YAXA
25:00:00	1qce-A	3	3.6	62	123	15	MOLECULE:	PROTEIN (GP41)
26:00:00	6el1-K	3	5.1	112	332	6	MOLECULE:	YAXA

27:00:00	5uky-A	3	2.7	43	74	5	MOLECULE:	ATP-BINDING PROTEIN
28:00:00	6vo3-B	3	4.2	44	124	7	MOLECULE:	PGV04 HEAVY CHAIN
29:00:00	6ot1-D	3	4.8	47	132	4	MOLECULE:	ENVELOPE GLYCOPROTEIN GP41
30:00:00	3wpo-C	3	6.9	98	193	8	MOLECULE:	TRIMERIC AUTOTRANSPORTER ADHESIN
31:00:00	4lin-B	3	51.5	111	289	4	MOLECULE:	TAIL NEEDLE PROTEIN GP26
32:00:00	6vo3-E	3	4.2	44	124	7	MOLECULE:	PGV04 HEAVY CHAIN
33:00:00	1llm-D	3	4.9	56	85	7	MOLECULE:	5'-D(*TP*CP*CP*CP*AP*CP*GP*CP*GP*TP*GP*GP*G)
34:00:00	6vo3-F	3	4.2	44	124	7	MOLECULE:	PGV04 HEAVY CHAIN
35:00:00	5vo5-A	2.9	3.3	76	179	7	MOLECULE:	COILED-COIL AND C2 DOMAIN-CONTAINING PROTEIN
36:00:00	6zz8-A	2.9	4.6	91	215	10	MOLECULE:	CENTRIOLE PROTEIN
37:00:00	8cpz-B	2.9	8.1	146	2122	11	MOLECULE:	TCDA1
38:00:00	8f7n-A	2.9	7.5	111	364	10	MOLECULE:	METHYL-ACCEPTING CHEMOTAXIS PROTEIN
39:00:00	6ah0-w	2.9	17.4	86	443	10	MOLECULE:	U5SNRNA
40:00:00	5c21-A	2.9	5.1	92	267	8	MOLECULE:	CHROMOSOMAL HEMOLYSIN D
41:00:00	8auc-B	2.9	33.5	123	493	7	MOLECULE:	CELL WALL-ASSOCIATED HYDROLASES (INVASION-AS
42:00:00	5td8-D	2.9	3.6	65	128	11	MOLECULE:	KINETOCHORE PROTEIN NDC80
43:00:00	4ilo-A	2.9	18.4	102	236	8	MOLECULE:	CT398
44:00:00	3wpa-A	2.9	6.6	112	346	4	MOLECULE:	TRIMERIC AUTOTRANSPORTER ADHESIN
45:00:00	4hkr-A	2.9	10.9	67	165	6	MOLECULE:	CALCIUM RELEASE-ACTIVATED CALCIUM CHANNEL PR
46:00:00	8eki-C	2.9	29.5	109	701	7	MOLECULE:	PROTEIN TRANSPORT PROTEIN SEC20
47:00:00	7sn7-a	2.9	7	104	546	10	MOLECULE:	FLAGELLIN
48:00:00	6fj3-A	2.9	9.7	56	562	5	MOLECULE:	PARATHYROID HORMONE/PARATHYROID HORMONE-RELA
49:00:00	4he8-D	2.9	26.5	72	160	8	MOLECULE:	NADH-QUINONE OXIDOREDUCTASE SUBUNIT 7
50:00:00	5nen-A	2.9	3.7	91	266	3	MOLECULE:	LIPASE C
51:00:00	8f7c-A	2.9	9.6	65	287	6	MOLECULE:	PANNEXIN-2, SOLUBLE CYTOCHROME B562 FUSION
52:00:00	6tpi-A	2.9	7.4	105	380	10	MOLECULE:	MUREIN HYDROLASE ACTIVATOR ENVC
53:00:00	7xzj-A	2.9	3.7	49	423	10	MOLECULE:	CTAP3
54:00:00	8av6-H	2.9	8.1	78	98	12	MOLECULE:	RUVB-LIKE HELICASE
55:00:00	4gwp-D	2.9	8.7	77	121	6	MOLECULE:	MEDIATOR OF RNA POLYMERASE II TRANSCRIPTION
56:00:00	8hpo-C	2.9	13	84	138	4	MOLECULE:	TRANSCRIPTIONAL REGULATORY PROTEIN UME1
57:00:00	7xym-A	2.9	15.4	114	488	10	MOLECULE:	ENVELOPE GLYCOPROTEIN
58:00:00	8xud-D	2.9	10.7	64	651	8	MOLECULE:	LIPOPROTEIN NLPI
59:00:00	6zh3-A	2.9	16.6	88	170	8	MOLECULE:	VACUOLAR PROTEIN-SORTING-ASSOCIATED PROTEIN
60:00:00	3j9v-M	2.9	4.1	96	210	6	MOLECULE:	V-TYPE PROTON ATPASE SUBUNIT A, VACUOLAR ISO
61:00:00	2ezp-B	2.9	3.8	64	123	11	MOLECULE:	GP41
62:00:00	8ap9-G	2.9	2.7	54	279	6	MOLECULE:	ATP SYNTHASE GAMMA SUBUNIT
63:00:00	2ezs-B	2.9	3.9	63	123	11	MOLECULE:	GP41
64:00:00	7yo1-D	2.9	19	66	263	9	MOLECULE:	CALCIUM-ACTIVATED POTASSIUM CHANNEL SUBUNIT
65:00:00	2ezr-A	2.9	3.9	63	123	11	MOLECULE:	GP41
66:00:00	2ezq-B	2.9	3.9	64	123	11	MOLECULE:	GP41
67:00:00	2ezr-C	2.9	3.9	63	123	11	MOLECULE:	GP41
68:00:00	2ezp-C	2.9	3.8	64	123	11	MOLECULE:	GP41
69:00:00	2ezr-B	2.9	3.9	63	123	11	MOLECULE:	GP41
70:00:00	2ezs-A	2.9	3.9	63	123	11	MOLECULE:	GP41
71:00:00	2ezs-C	2.9	3.9	63	123	11	MOLECULE:	GP41
72:00:00	7jh6-D	2.9	5.9	48	174	13	MOLECULE:	TWO-DOMAIN DI-ZN(II) AND PORPHYRIN-BINDING P
73:00:00	6o58-E	2.9	16.4	106	270	6	MOLECULE:	CALCIUM UNIPORTER PROTEIN, MITOCHONDRIAL
74:00:00	5lhh-A	2.9	50.5	84	666	11	MOLECULE:	LYSINE-SPECIFIC HISTONE DEMETHYLASE 1A
75:00:00	5t8f-B	2.9	6.6	82	169	9	MOLECULE:	PHOSPHATIDYLINOSITOL 4,5-BISPHOSPHATE 3-KINA
76:00:00	7jh6-C	2.9	5.9	48	174	13	MOLECULE:	TWO-DOMAIN DI-ZN(II) AND PORPHYRIN-BINDING P
77:00:00	7khw-D	2.9	3.2	62	174	3	MOLECULE:	TRANSLOCON ESPA

78:00:00	5k7b-D	2.9	11	59	88	12	MOLECULE:	BECLIN-2
79:00:00	6ot1-B	2.9	3.8	44	132	5	MOLECULE:	ENVELOPE GLYCOPROTEIN GP41
80:00:00	6ot1-O	2.9	3.8	44	132	5	MOLECULE:	ENVELOPE GLYCOPROTEIN GP41
81:00:00	7qen-B	2.9	11.8	75	419	4	MOLECULE:	DNA (35-MER)
82:00:00	7nuv-A	2.8	5.5	52	76	4	MOLECULE:	AUX2PLS20
83:00:00	3qwe-A	2.8	5.4	93	260	9	MOLECULE:	GEM-INTERACTING PROTEIN
84:00:00	6tdv-M	2.8	19.2	89	166	12	MOLECULE:	ATPTB1
85:00:00	4o8w-D	2.8	9.4	67	120	10	MOLECULE:	SPORE GERMINATION PROTEIN
86:00:00	6gsi-J	2.8	17.4	70	89	6	MOLECULE:	CAPSID PROTEIN
87:00:00	9b8o-U	2.8	3.8	56	214	5	MOLECULE:	ATPASE H+-TRANSPORTING V1 SUBUNIT D
88:00:00	6m3q-F	2.8	6.4	98	301	5	MOLECULE:	ANKYRIN-2
89:00:00	7y22-e	2.8	4.2	64	172	5	MOLECULE:	PHAGE CONNECTOR PROTEIN
90:00:00	7zmg-j	2.8	12.2	65	75	5	MOLECULE:	NADH-UBIQUINONE OXIDOREDUCTASE CHAIN 1
91:00:00	4nnh-A	2.8	3.1	42	164	7	MOLECULE:	30S RIBOSOMAL PROTEIN S1
92:00:00	7bqx-G	2.8	8	73	84	3	MOLECULE:	TRIPLEX CAPSID PROTEIN 1
93:00:00	7c4j-C	2.8	37.4	98	154	8	MOLECULE:	TRANSCRIPTION REGULATORY PROTEIN SNF12
94:00:00	8q85-a	2.8	7.3	78	234	4	MOLECULE:	KINETOCHORE PROTEIN NDC80
95:00:00	3viq-A	2.8	16.3	75	122	9	MOLECULE:	SWI5-DEPENDENT RECOMBINATION DNA REPAIR PROT
96:00:00	7wtt-z	2.8	25.1	59	82	3	MOLECULE:	18S RRNA
97:00:00	2wwb-B	2.8	10.1	57	68	9	MOLECULE:	PROTEIN TRANSPORT PROTEIN SEC61 SUBUNIT ALPH
98:00:00	7qo4-J	2.8	18.7	101	405	4	MOLECULE:	26S PROTEASOME REGULATORY SUBUNIT RPN1
99:00:00	6yvd-D	2.8	8.3	73	414	8	MOLECULE:	CONDENSIN COMPLEX SUBUNIT 2
0:00	6h2x-A	2.8	8	126	352	10	MOLECULE:	CHROMOSOME PARTITION PROTEIN MUKB,CHROMOSOME
1:00	6rw8-B	2.8	58	136	2337	5	MOLECULE:	A COMPONENT OF INSECTICIDAL TOXIN COMPLEX (T
2:00	5knd-G	2.8	4.8	84	178	7	MOLECULE:	V-TYPE SODIUM ATPASE CATALYTIC SUBUNIT A
3:00	5y5y-G	2.8	4.6	95	210	13	MOLECULE:	V-TYPE ATP SYNTHASE ALPHA CHAIN
4:00	2ezo-B	2.8	3.8	63	123	11	MOLECULE:	GP41
5:00	6o58-C	2.8	16.4	112	268	4	MOLECULE:	CALCIUM UNIPORTER PROTEIN, MITOCHONDRIAL
6:00	2ezq-C	2.8	3.7	63	123	11	MOLECULE:	GP41
7:00	2ezq-A	2.8	3.7	63	123	11	MOLECULE:	GP41
8:00	2ezp-A	2.8	4	63	123	11	MOLECULE:	GP41
9:00	6wdo-C	2.8	15.8	119	268	3	MOLECULE:	CALCIUM UNIPORTER PROTEIN, MITOCHONDRIAL
10:00	6ud4-H	2.8	4.6	41	134	5	MOLECULE:	GLUTAMATE RECEPTOR 2
11:00	6ud4-F	2.8	4.6	41	134	5	MOLECULE:	GLUTAMATE RECEPTOR 2
12:00	5j9t-D	2.8	4.5	58	119	7	MOLECULE:	HISTONE ACETYLTRANSFERASE ESA1
13:00	6vqa-N	2.8	6.7	46	47	4	MOLECULE:	ATPASE H+-TRANSPORTING V1 SUBUNIT A
14:00	4r61-A	2.8	2.1	52	140	19	MOLECULE:	GP41-BASED CONSTRUCT COVNHR3-ABC
15:00	7llk-B	2.8	4.2	47	126	19	MOLECULE:	ENVELOPE GLYCOPROTEIN GP120
16:00	7rai-B	2.8	4.1	47	128	4	MOLECULE:	ENVELOPE GLYCOPROTEIN GP160
17:00	8tni-F	2.8	4.1	49	126	2	MOLECULE:	HIV-1 BG505 DS-SOSIP GP120
18:00	4wbd-A	2.7	7	65	540	8	MOLECULE:	BSHC
19:00	8odu-C	2.7	4.8	70	232	7	MOLECULE:	ATPASE GET3
20:00	8w8q-C	2.7	8.5	53	287	6	MOLECULE:	SOLUBLE CYTOCHROME B562,PROBABLE G-PROTEIN C
21:00	5lc5-Z	2.7	14.7	91	138	5	MOLECULE:	NADH-UBIQUINONE OXIDOREDUCTASE CHAIN 3
22:00	1lgh-A	2.7	5.1	47	56	9	MOLECULE:	LIGHT HARVESTING COMPLEX II
23:00	8b6j-i	2.7	16.7	73	117	3	MOLECULE:	PEPTIDASE M16 INACTIVE DOMAIN PROTEIN
24:00:00	2mi2-A	2.7	15.2	84	104	8	MOLECULE:	SEC-INDEPENDENT PROTEIN TRANSLOCASE PROTEIN
25:00:00	5lm2-B	2.7	13.7	113	340	4	MOLECULE:	TYROSINE-PROTEIN PHOSPHATASE NON-RECEPTOR TY
26:00:00	5w82-B	2.7	6.3	55	101	11	MOLECULE:	PROTEIN DELTA
27:00:00	8i3j-A	2.7	5.1	96	277	7	MOLECULE:	DYNEIN AXONEMAL HEAVY CHAIN 1
28:00:00	5n9j-Z	2.7	6.9	75	119	4	MOLECULE:	MEDIATOR OF RNA POLYMERASE II TRANSCRIPTION

29:00:00	6yj4-i	2.7	8.6	61	86	10	MOLECULE:	NADH-UBIQUINONE OXIDOREDUCTASE CHAIN 3
30:00:00	5efv-B	2.7	27.1	93	635	8	MOLECULE:	PHI ETA ORF 56-LIKE PROTEIN
31:00:00	7xe4-F	2.7	27.8	95	1503	6	MOLECULE:	1,3-BETA-GLUCAN SYNTHASE COMPONENT FKS1
32:00:00	8bf9-G	2.7	9.2	64	158	5	MOLECULE:	RNA (1766)
33:00:00	6wg3-C	2.7	31.6	58	248	10	MOLECULE:	STRUCTURAL MAINTENANCE OF CHROMOSOMES PROTEI
34:00:00	4wxa-E	2.7	5.6	50	69	14	MOLECULE:	EKC/KEOPS COMPLEX SUBUNIT PCC1
35:00:00	7w76-D	2.7	5.4	64	168	2	MOLECULE:	UBIQUITIN-CONJUGATING ENZYME E2 2
36:00:00	6szw-C	2.7	13.7	57	186	9	MOLECULE:	COMPLEMENT COMPONENT 1 Q SUBCOMPONENT-BINDIN
37:00:00	6foc-G	2.7	3.8	71	193	8	MOLECULE:	ATP SYNTHASE SUBUNIT ALPHA,ATP SYNTHASE SUBU
38:00:00	5sxa-B	2.7	3.2	70	218	7	MOLECULE:	PHOSPHATIDYLINOSITOL 4,5-BISPHOSPHATE 3-KINA
39:00:00	7zqy-A	2.7	3.5	73	175	4	MOLECULE:	DH DOMAIN-CONTAINING PROTEIN
40:00:00	3j9t-M	2.7	4.8	97	210	5	MOLECULE:	V-TYPE PROTON ATPASE SUBUNIT D
41:00:00	6xj1-B	2.7	6.8	124	277	3	MOLECULE:	CELL DIVISION CONTROL PROTEIN 15
42:00:00	7tmo-I	2.7	10	91	217	8	MOLECULE:	H(+)-TRANSPORTING TWO-SECTOR ATPASE
43:00:00	7fda-L	2.7	14.2	91	111	7	MOLECULE:	YEAST VACUOLAR ATPASE A SUBUNIT
44:00:00	4zwr-C	2.7	6	58	137	9	MOLECULE:	RECOMBINATION PROTEIN UVSY
45:00:00	8dk2-C	2.7	15.9	144	723	3	MOLECULE:	JETA
46:00:00	2ezo-C	2.7	3.5	61	123	15	MOLECULE:	GP41
47:00:00	7khw-k	2.7	3.2	62	174	3	MOLECULE:	TRANSLOCON ESPA
48:00:00	7khw-A	2.7	3.2	62	174	3	MOLECULE:	TRANSLOCON ESPA
49:00:00	7khw-C	2.7	3.2	62	174	3	MOLECULE:	TRANSLOCON ESPA
50:00:00	6wdn-G	2.7	11.8	88	174	13	MOLECULE:	CALCIUM UPTAKE PROTEIN 2, MITOCHONDRIAL
51:00:00	7khw-v	2.7	3.2	62	174	3	MOLECULE:	TRANSLOCON ESPA
52:00:00	7khw-V	2.7	3.2	62	174	3	MOLECULE:	TRANSLOCON ESPA
53:00:00	7khw-x	2.7	3.2	62	174	3	MOLECULE:	TRANSLOCON ESPA
54:00:00	7khw-Q	2.7	3.2	62	174	3	MOLECULE:	TRANSLOCON ESPA
55:00:00	5lgn-A	2.7	50.4	84	670	10	MOLECULE:	LYSINE-SPECIFIC HISTONE DEMETHYLASE 1A
56:00:00	7khw-J	2.7	3.2	62	174	3	MOLECULE:	TRANSLOCON ESPA
57:00:00	7khw-e	2.7	3.2	62	174	3	MOLECULE:	TRANSLOCON ESPA
58:00:00	6vqb-M	2.7	6.2	47	47	0	MOLECULE:	ATPASE H+-TRANSPORTING V1 SUBUNIT A
59:00:00	7khw-w	2.7	3.2	62	174	3	MOLECULE:	TRANSLOCON ESPA
60:00:00	7khw-p	2.7	3.2	62	174	3	MOLECULE:	TRANSLOCON ESPA
61:00:00	7rai-D	2.7	3.4	47	128	9	MOLECULE:	ENVELOPE GLYCOPROTEIN GP160
62:00:00	6u59-B	2.7	3.6	45	120	4	MOLECULE:	SOSIP.664 GP120,SOSIP.664 GP120
63:00:00	8euu-D	2.7	3.9	44	130	5	MOLECULE:	ENVELOPE GLYCOPROTEIN GP120
64:00:00	7l7u-D	2.7	4.3	47	145	11	MOLECULE:	BG505 SOSIP.V5.2(7S) - GP120
65:00:00	7ur6-I	2.7	4.3	45	132	4	MOLECULE:	GP120
66:00:00	8ovw-Q	2.6	19.1	110	258	4	MOLECULE:	CENTROMERE-BINDING PROTEIN 1
67:00:00	7jw1-e	2.6	9.6	100	235	4	MOLECULE:	CAPSID PROTEINS
68:00:00	8t8k-A	2.6	3.3	45	183	13	MOLECULE:	DUF507 FAMILY PROTEIN
69:00:00	4u6u-D	2.6	11.1	90	283	7	MOLECULE:	COG7
70:00:00	6jfk-A	2.6	4.5	81	428	9	MOLECULE:	MITOFUSIN-2,CDNA FLJ57997, HIGHLY SIMILAR TO
71:00:00	5a31-N	2.6	28.7	78	703	3	MOLECULE:	ANAPHASE-PROMOTING COMPLEX SUBUNIT 1
72:00:00	5t58-A	2.6	25.6	95	233	9	MOLECULE:	KLLA0F02343P
73:00:00	4ad8-A	2.6	9.1	104	452	11	MOLECULE:	DNA REPAIR PROTEIN RECN
74:00:00	1yx4-A	2.6	37.7	83	112	6	MOLECULE:	26S PROTEASOME NON-ATPASE REGULATORY SUBUNIT
75:00:00	6r1j-J	2.6	4.3	90	264	4	MOLECULE:	UNCHARACTERIZED PROTEIN
76:00:00	8soi-B	2.6	6.4	99	515	3	MOLECULE:	RB1-INDUCIBLE COILED-COIL PROTEIN 1
77:00:00	8eup-3	2.6	11.1	61	194	7	MOLECULE:	RNA (1422-MER)
78:00:00	2gf4-A	2.6	2.5	47	89	4	MOLECULE:	PROTEIN VNG1086C
79:00:00	5ncl-A	2.6	25.2	69	418	7	MOLECULE:	SERINE/THREONINE-PROTEIN KINASE CBK1

80:00:00	8ixk-A	2.6	4.7	43	145	5	MOLECULE:	ATTACHMENT PROTEIN G3P
81:00:00	7loi-A	2.6	18.3	81	175	9	MOLECULE:	TRANSMEMBRANE PROTEIN GP41
82:00:00	8i8b-H	2.6	10.2	65	228	6	MOLECULE:	MAJOR VIRAL CAPSID PROTEIN
83:00:00	5oxf-C	2.6	21	107	601	6	MOLECULE:	GTP-BINDING PROTEIN
84:00:00	6tmi-B	2.6	8.4	97	246	5	MOLECULE:	ATP SYNTHASE SUBUNIT ALPHA
85:00:00	1flc-B	2.6	3	57	162	5	MOLECULE:	HAEMAGGLUTININ-ESTERASE-FUSION GLYCOPROTEIN
86:00:00	6gy6-A	2.6	6	99	365	10	MOLECULE:	XAXA
87:00:00	5c0w-K	2.6	38.5	99	576	6	MOLECULE:	EXOSOME COMPLEX COMPONENT RRP45
88:00:00	7mjp-A	2.6	6.8	61	366	3	MOLECULE:	ATP-SENSITIVE INWARD RECTIFIER POTASSIUM CHA
89:00:00	8b9z-m	2.6	12.2	79	108	5	MOLECULE:	NADH-UBIQUINONE OXIDOREDUCTASE CHAIN 3
90:00:00	6xky-A	2.6	5.3	101	271	8	MOLECULE:	FLAGELLIN
91:00:00	6xf1-A	2.6	6.1	78	230	5	MOLECULE:	NESPRIN-2
92:00:00	6v22-E	2.6	3.4	65	199	3	MOLECULE:	CALCIUM-ACTIVATED POTASSIUM CHANNEL SUBUNIT
93:00:00	8h2j-A	2.6	6.9	49	92	8	MOLECULE:	P26
94:00:00	7w6x-A	2.6	7.2	47	447	6	MOLECULE:	REGULATOR OF SIGMA-E PROTEASE RSEP
95:00:00	7yzp-C	2.6	7.5	92	448	8	MOLECULE:	DNA HAIRPIN (59-MER)
96:00:00	5f5p-A	2.6	3.1	56	181	9	MOLECULE:	PROTEIN SHROOM2
97:00:00	6zka-p	2.6	21.6	89	128	2	MOLECULE:	NADH-UBIQUINONE OXIDOREDUCTASE CHAIN 3
98:00:00	5knc-G	2.6	6.6	88	195	10	MOLECULE:	V-TYPE SODIUM ATPASE CATALYTIC SUBUNIT A
99:00:00	6ixv-B	2.6	33.8	102	226	5	MOLECULE:	SH3 DOMAIN-BINDING PROTEIN 5
0:00	7jtk-D	2.6	15.3	127	427	5	MOLECULE:	FLAGELLAR RADIAL SPOKE PROTEIN 1
1:00	6sih-E	2.6	6.8	108	488	5	MOLECULE:	FLAGELLAR HOOK-ASSOCIATED PROTEIN 2
2:00	3etz-B	2.6	2.8	52	115	6	MOLECULE:	ADHESIN A
3:00	8at4-B	2.6	10.4	149	597	7	MOLECULE:	HAUS AUGMIN-LIKE COMPLEX SUBUNIT 1
4:00	6lth-I	2.6	13.1	84	120	8	MOLECULE:	TRANSCRIPTION ACTIVATOR BRG1
5:00	7khw-d	2.6	3.2	62	174	3	MOLECULE:	TRANSLOCON ESPA
6:00	7khw-t	2.6	3.2	62	174	3	MOLECULE:	TRANSLOCON ESPA
7:00	7khw-R	2.6	3.2	62	174	3	MOLECULE:	TRANSLOCON ESPA
8:00	7khw-I	2.6	3.2	62	174	3	MOLECULE:	TRANSLOCON ESPA
9:00	6elu-G	2.6	8.6	49	168	0	MOLECULE:	SERUM RESISTANCE ASSOCIATED VSG PROTEIN
10:00	7khw-T	2.6	3.2	62	174	3	MOLECULE:	TRANSLOCON ESPA
11:00	7khw-K	2.6	3.2	62	174	3	MOLECULE:	TRANSLOCON ESPA
12:00	7khw-m	2.6	3.2	62	174	3	MOLECULE:	TRANSLOCON ESPA
13:00	7khw-f	2.6	3.2	62	174	3	MOLECULE:	TRANSLOCON ESPA
14:00	7khw-X	2.6	3.2	62	174	3	MOLECULE:	TRANSLOCON ESPA
15:00	7khw-o	2.6	3.2	62	174	3	MOLECULE:	TRANSLOCON ESPA
16:00	7khw-h	2.6	3.2	62	174	3	MOLECULE:	TRANSLOCON ESPA
17:00	7khw-q	2.6	3.2	62	174	3	MOLECULE:	TRANSLOCON ESPA
18:00	7khw-M	2.6	3.2	62	174	3	MOLECULE:	TRANSLOCON ESPA
19:00	7khw-s	2.6	3.2	62	174	3	MOLECULE:	TRANSLOCON ESPA
20:00	7khw-O	2.6	3.2	62	174	3	MOLECULE:	TRANSLOCON ESPA
21:00	7khw-Z	2.6	3.2	62	174	3	MOLECULE:	TRANSLOCON ESPA
22:00	6vq9-N	2.6	6.8	46	47	9	MOLECULE:	ATPASE H+-TRANSPORTING V1 SUBUNIT A
23:00	7khw-G	2.6	3.2	62	174	3	MOLECULE:	TRANSLOCON ESPA
24:00:00	7khw-E	2.6	3.2	62	174	3	MOLECULE:	TRANSLOCON ESPA
25:00:00	7khw-H	2.6	3.2	62	174	3	MOLECULE:	TRANSLOCON ESPA
26:00:00	7khw-j	2.6	3.2	62	174	3	MOLECULE:	TRANSLOCON ESPA
27:00:00	7khw-c	2.6	3.2	62	174	3	MOLECULE:	TRANSLOCON ESPA
28:00:00	7khw-B	2.6	3.2	62	174	3	MOLECULE:	TRANSLOCON ESPA
29:00:00	7khw-S	2.6	3.2	62	174	3	MOLECULE:	TRANSLOCON ESPA
30:00:00	7khw-l	2.6	3.2	62	174	3	MOLECULE:	TRANSLOCON ESPA

31:00:00	7khw-u	2.6	3.2	62	174	3	MOLECULE:	TRANSLOCON ESPA
32:00:00	6wdo-G	2.6	22.7	109	255	9	MOLECULE:	CALCIUM UNIPORTER PROTEIN, MITOCHONDRIAL
33:00:00	7khw-g	2.6	3.2	62	174	3	MOLECULE:	TRANSLOCON ESPA
34:00:00	7khw-L	2.6	3.2	62	174	3	MOLECULE:	TRANSLOCON ESPA
35:00:00	7khw-n	2.6	3.2	62	174	3	MOLECULE:	TRANSLOCON ESPA
36:00:00	7khw-U	2.6	3.2	62	174	3	MOLECULE:	TRANSLOCON ESPA
37:00:00	7khw-N	2.6	3.2	62	174	3	MOLECULE:	TRANSLOCON ESPA
38:00:00	7khw-P	2.6	3.2	62	174	3	MOLECULE:	TRANSLOCON ESPA
39:00:00	7khw-b	2.6	3.2	62	174	3	MOLECULE:	TRANSLOCON ESPA
40:00:00	7khw-i	2.6	3.2	62	174	3	MOLECULE:	TRANSLOCON ESPA
41:00:00	7khw-F	2.6	3.2	62	174	3	MOLECULE:	TRANSLOCON ESPA
42:00:00	7khw-Y	2.6	3.2	62	174	3	MOLECULE:	TRANSLOCON ESPA
43:00:00	8q0e-B	2.6	3.2	49	241	8	MOLECULE:	VARIANT SURFACE GLYCOPROTEIN 545
44:00:00	7khw-W	2.6	3.2	62	174	3	MOLECULE:	TRANSLOCON ESPA
45:00:00	7khw-r	2.6	3.2	62	174	3	MOLECULE:	TRANSLOCON ESPA
46:00:00	6x5b-E	2.6	3.8	50	149	12	MOLECULE:	ENVELOPE GLYCOPROTEIN GP120
47:00:00	6x9r-B	2.6	3.6	42	121	5	MOLECULE:	HIV-1 ENVELOPE GLYCOPROTEIN BG505 SOSIP.664
48:00:00	6x9t-B	2.6	3.5	42	121	17	MOLECULE:	HIV-1 ENVELOPE GLYCOPROTEIN BG505 SOSIP.664
49:00:00	8gpj-U	2.6	3.8	43	128	9	MOLECULE:	8ANC195 FAB HEAVY CHAIN
50:00:00	6vo0-E	2.6	4.2	47	122	4	MOLECULE:	43A2 LIGHT CHAIN
51:00:00	6nc3-M	2.6	5.1	47	128	6	MOLECULE:	HIV-1 ENV AMC011 V4.2 SOSIP GP120
52:00:00	6nc2-N	2.6	12	45	129	7	MOLECULE:	AMC011 V4.2 SOSIP GP120
53:00:00	6nc3-K	2.6	3.5	44	128	9	MOLECULE:	HIV-1 ENV AMC011 V4.2 SOSIP GP120
54:00:00	6vo1-B	2.6	3.8	44	129	7	MOLECULE:	ENVELOPE GLYCOPROTEIN GP120
55:00:00	8tni-D	2.6	3.7	46	126	11	MOLECULE:	HIV-1 BG505 DS-SOSIP GP120
56:00:00	7lpn-E	2.6	4	43	121	9	MOLECULE:	HIV-1 ENVELOPE GLYCOPROTEIN BG505 SOSIP.664
57:00:00	7l6o-f	2.6	3.8	43	126	5	MOLECULE:	CH848.3.D0949.10.17CHIM.6R.DS.SOSIP.664 - GP
58:00:00	6nc3-B	2.6	3.5	44	128	9	MOLECULE:	HIV-1 ENV AMC011 V4.2 SOSIP GP120
59:00:00	6vo0-F	2.6	4.2	47	122	4	MOLECULE:	43A2 LIGHT CHAIN
60:00:00	8g4m-F	2.6	4.1	45	131	11	MOLECULE:	ENVELOPE GLYCOPROTEIN GP120
61:00:00	6vo0-B	2.6	4.2	47	122	4	MOLECULE:	43A2 LIGHT CHAIN
62:00:00	6vo1-F	2.6	3.8	44	129	7	MOLECULE:	ENVELOPE GLYCOPROTEIN GP120
63:00:00	2vky-B	2.6	8.6	59	152	12	MOLECULE:	TAIL PROTEIN, PIIGCN4
64:00:00	4gnk-E	2.5	42.2	72	235	11	MOLECULE:	GUANINE NUCLEOTIDE-BINDING PROTEIN G(Q) SUBU
65:00:00	8ceg-B	2.5	2.7	79	214	4	MOLECULE:	CBY1-INTERACTING BAR DOMAIN-CONTAINING PROTE
66:00:00	6a6x-C	2.5	9.3	61	75	5	MOLECULE:	PROBABLE ENDORIBONUCLEASE MAZF7
67:00:00	6f7j-A	2.5	8.9	59	97	8	MOLECULE:	SERRATE RNA EFFECTOR MOLECULE HOMOLOG
68:00:00	5yfp-B	2.5	24.9	144	927	6	MOLECULE:	EXOCYST COMPLEX COMPONENT SEC3
69:00:00	7p5j-A	2.5	38.4	59	399	7	MOLECULE:	PROTEIN TWEETY HOMOLOG 1
70:00:00	6y1y-B	2.5	5.5	68	128	10	MOLECULE:	CHEA
71:00:00	7o6t-A	2.5	3.1	46	73	4	MOLECULE:	PROTEIN VERNALIZATION INSENSITIVE 3
72:00:00	7jve-A	2.5	14.2	85	253	4	MOLECULE:	DSBA FAMILY PROTEIN
73:00:00	8k32-C	2.5	30.6	64	333	5	MOLECULE:	KETOL-ACID REDUCTOISOMERASE (NADP(+))
74:00:00	8hpo-H	2.5	27.7	99	233	7	MOLECULE:	TRANSCRIPTIONAL REGULATORY PROTEIN UME1
75:00:00	5sva-V	2.5	6.9	58	85	12	MOLECULE:	DNA-DIRECTED RNA POLYMERASE II SUBUNIT RPB1
76:00:00	5wjt-A	2.5	6.1	105	302	3	MOLECULE:	FLAGELLIN
77:00:00	7nyx-A	2.5	12.7	127	1467	11	MOLECULE:	CHROMOSOME PARTITION PROTEIN MUKB
78:00:00	6zyy-C	2.5	41.4	142	2901	6	MOLECULE:	DYNEIN HEAVY CHAIN, OUTER ARM PROTEIN
79:00:00	2yjf-M	2.5	15.6	78	121	8	MOLECULE:	ACTIN, ALPHA SKELETAL MUSCLE
80:00:00	5n0c-A	2.5	34.6	99	1293	5	MOLECULE:	TETANUS TOXIN
81:00:00	2yfb-B	2.5	4.8	71	238	7	MOLECULE:	METHYL-ACCEPTING CHEMOTAXIS TRANSDUCER

82:00:00	4abx-A	2.5	6.6	82	167	7	MOLECULE:	DNA REPAIR PROTEIN RECN
83:00:00	7nad-8	2.5	8.3	58	58	5	MOLECULE:	25S RRNA
84:00:00	2lzs-A	2.5	11	47	48	13	MOLECULE:	SEC-INDEPENDENT PROTEIN TRANSLOCASE PROTEIN
85:00:00	7vcf-C	2.5	7.1	75	217	8	MOLECULE:	TIC214
86:00:00	1few-A	2.5	4	75	173	8	MOLECULE:	SECOND MITOCHONDRIA-DERIVED ACTIVATOR OF CAS
87:00:00	6x0a-a	2.5	41	66	87	6	MOLECULE:	PLASMID STABILIZATION SYSTEM
88:00:00	7qtt-Y	2.5	47.1	110	306	8	MOLECULE:	SPLICING FACTOR 3B SUBUNIT 3
89:00:00	1ej6-D	2.5	12.4	60	417	7	MOLECULE:	LAMBDA2
90:00:00	6uz8-A	2.5	51.8	119	734	9	MOLECULE:	SHORT TRANSIENT RECEPTOR POTENTIAL CHANNEL 6
91:00:00	7jrj-G	2.5	11.2	78	148	9	MOLECULE:	RADIAL SPOKE PROTEIN 10
92:00:00	6h7w-O	2.5	4.4	81	220	7	MOLECULE:	VACUOLAR PROTEIN SORTING-ASSOCIATED PROTEIN
93:00:00	5wwl-M	2.5	24.3	99	214	2	MOLECULE:	CENTROMERE PROTEIN MIS12
94:00:00	8fbi-A	2.5	8.6	48	274	2	MOLECULE:	KWOCA_39
95:00:00	7egm-A	2.5	5.8	68	109	4	MOLECULE:	TRANSCRIPTION REGULATORY PROTEIN SNF2
96:00:00	8eja-A	2.5	3.6	39	113	10	MOLECULE:	ABAK
97:00:00	6xl0-B	2.5	9.4	82	271	7	MOLECULE:	FLAGELLIN
98:00:00	6xl0-D	2.5	9.4	82	271	7	MOLECULE:	FLAGELLIN
99:00:00	6xl0-G	2.5	9.4	82	271	7	MOLECULE:	FLAGELLIN
0:00	6xl0-I	2.5	9.4	82	271	7	MOLECULE:	FLAGELLIN
1:00	5xi5-E	2.5	10.9	61	120	5	MOLECULE:	TUBULIN ALPHA CHAIN
2:00	6xl0-R	2.5	9.4	82	271	7	MOLECULE:	FLAGELLIN
3:00	8v8j-B	2.5	4.4	84	239	5	MOLECULE:	PHOSPHATIDYLINOSITOL 4,5-BISPHOSPHATE 3-KINA
4:00	6xl0-F	2.5	9.4	82	271	7	MOLECULE:	FLAGELLIN
5:00	6xl0-H	2.5	9.4	82	271	7	MOLECULE:	FLAGELLIN
6:00	6vqb-O	2.5	6.8	46	47	9	MOLECULE:	ATPASE H+-TRANSPORTING V1 SUBUNIT A
7:00	6xl0-J	2.5	9.4	82	271	7	MOLECULE:	FLAGELLIN
8:00	8g6u-B	2.5	2.9	46	120	11	MOLECULE:	CRF01_AE T/F100 HIV-1 GP120
9:00	3wpo-B	2.5	9.1	54	196	4	MOLECULE:	TRIMERIC AUTOTRANSPORTER ADHESIN
10:00	6ce0-B	2.5	3.7	43	137	9	MOLECULE:	ENVELOPE GLYCOPROTEIN GP160
11:00	6x5c-G	2.5	3	50	148	6	MOLECULE:	ENVELOPE GLYCOPROTEIN GP160
12:00	7rai-F	2.5	3.9	46	128	4	MOLECULE:	ENVELOPE GLYCOPROTEIN GP160
13:00	6nc3-J	2.5	3.4	43	128	9	MOLECULE:	HIV-1 ENV AMC011 V4.2 SOSIP GP120
14:00	8d5c-Y	2.5	4.4	50	129	4	MOLECULE:	ENVELOPE GLYCOPROTEIN GP41
15:00	7mdt-D	2.5	4.3	44	122	7	MOLECULE:	SURFACE PROTEIN GP120
16:00	8g6u-F	2.5	2.9	46	120	11	MOLECULE:	CRF01_AE T/F100 HIV-1 GP120
17:00	8saq-F	2.5	4.6	50	127	2	MOLECULE:	CH848.0526.25 GP120
18:00	6u0n-Z	2.5	3.8	50	124	2	MOLECULE:	ENVELOPE GLYCOPROTEIN GP120
19:00	6opq-B	2.5	2.9	47	128	9	MOLECULE:	ENVELOPE GLYCOPROTEIN GP160
20:00	6nc3-I	2.5	3.4	43	128	9	MOLECULE:	HIV-1 ENV AMC011 V4.2 SOSIP GP120
21:00	6vo1-E	2.5	4	44	129	9	MOLECULE:	ENVELOPE GLYCOPROTEIN GP120
22:00	6um6-J	2.5	4.4	45	132	7	MOLECULE:	CH848 10.17DT GP120
23:00	6x9s-B	2.5	3.8	42	121	5	MOLECULE:	HIV-1 ENVELOPE GLYCOPROTEIN BG505 SOSIP.664
24:00:00	8g6u-J	2.5	2.9	46	120	11	MOLECULE:	CRF01_AE T/F100 HIV-1 GP120
25:00:00	6nc3-N	2.5	3.4	43	128	9	MOLECULE:	HIV-1 ENV AMC011 V4.2 SOSIP GP120
26:00:00	6um6-F	2.5	4.2	44	132	7	MOLECULE:	CH848 10.17DT GP120
27:00:00	7nnl-B	2.4	17	70	682	1	MOLECULE:	POTASSIUM-TRANSPORTING ATPASE POTASSIUM-BIND
28:00:00	4d6k-A	2.4	7	46	76	4	MOLECULE:	DEOXYNUCLEOTIDYLTRANSFERASE TERMINAL-INTERAC
29:00:00	7wb4-E	2.4	24	60	1363	5	MOLECULE:	OUTER NUP133
30:00:00	6sl1-A	2.4	18.2	77	2651	5	MOLECULE:	SERINE/THREONINE-PROTEIN KINASE TEL1
31:00:00	6zbj-A	2.4	36.7	130	666	5	MOLECULE:	PRECURSOR OF THE MAJOR MEROZOITE SURFACE ANT
32:00:00	6nr8-4	2.4	2.8	50	104	2	MOLECULE:	PREFOLDIN SUBUNIT 1

33:00:00	6s3l-K	2.4	5.1	58	193	5	MOLECULE:	FLAGELLAR BIOSYNTHETIC PROTEIN FLIP
34:00:00	1fxk-C	2.4	5.7	60	133	7	MOLECULE:	PREFOLDIN
35:00:00	5jr9-A	2.4	7.4	58	188	14	MOLECULE:	NEQ131
36:00:00	6qum-N	2.4	8.5	65	649	9	MOLECULE:	V-TYPE ATP SYNTHASE ALPHA CHAIN
37:00:00	7xdi-D	2.4	4.9	51	124	4	MOLECULE:	VP1
38:00:00	5d2s-A	2.4	16	58	91	16	MOLECULE:	FIBROIN-MODULATOR-BINDING PROTEIN-1
39:00:00	4uy3-A	2.4	4.5	71	194	7	MOLECULE:	SEPTATION RING FORMATION REGULATOR EZRA
40:00:00	6xby-a	2.4	46.8	82	494	7	MOLECULE:	V-TYPE PROTON ATPASE CATALYTIC SUBUNIT A
41:00:00	5z25-A	2.4	6.1	51	96	8	MOLECULE:	CYTOCHROME C552
42:00:00	3edv-A	2.4	9.7	88	322	6	MOLECULE:	SPECTRIN BETA CHAIN, BRAIN 1
43:00:00	6ye4-A	2.4	3.9	83	236	8	MOLECULE:	BIOPOLYMER TRANSPORT PROTEIN EXBB
44:00:00	5j1i-A	2.4	6.6	105	346	10	MOLECULE:	PLECTIN
45:00:00	5i4q-B	2.4	2.7	45	107	9	MOLECULE:	CONTACT-DEPENDENT INHIBITOR A
46:00:00	3caz-B	2.4	6.1	82	210	5	MOLECULE:	BAR PROTEIN
47:00:00	2kog-A	2.4	21.9	76	116	7	MOLECULE:	VESICLE-ASSOCIATED MEMBRANE PROTEIN 2
48:00:00	2iaz-B	2.4	13.6	55	113	5	MOLECULE:	HYPOTHETICAL PROTEIN SP1372
49:00:00	8z9a-A	2.4	8.8	64	406	14	MOLECULE:	ODORANT RECEPTOR, APISORCO
50:00:00	4yxw-G	2.4	4.1	77	213	5	MOLECULE:	ATP SYNTHASE SUBUNIT ALPHA, MITOCHONDRIAL
51:00:00	3g3z-A	2.4	12.3	58	142	2	MOLECULE:	TRANSCRIPTIONAL REGULATOR, MARR FAMILY
52:00:00	7uh4-A	2.4	2.3	63	110	5	MOLECULE:	LXG-ASSOCIATED ALPHA-HELICAL PROTEIN D2
53:00:00	4uig-A	2.4	5.3	44	91	7	MOLECULE:	COPPER SENSITIVE OPERON REPRESSOR
54:00:00	6uz3-A	2.4	30.6	67	1126	4	MOLECULE:	SODIUM CHANNEL PROTEIN TYPE 5 SUBUNIT ALPHA,
55:00:00	7pde-A	2.4	12.6	86	846	3	MOLECULE:	ADENYLATE CYCLASE 9
56:00:00	5z7b-B	2.4	10	49	197	4	MOLECULE:	PADR FAMILY TRANSCRIPTIONAL REGULATOR
57:00:00	5td8-C	2.4	9.2	73	111	8	MOLECULE:	KINETOCHORE PROTEIN NDC80
58:00:00	2nb1-A	2.4	7.5	46	60	4	MOLECULE:	TUMOR PROTEIN 63
59:00:00	7qj0-L	2.4	10.4	64	566	5	MOLECULE:	MITOTIC-SPINDLE ORGANIZING PROTEIN 1
60:00:00	8ulg-A	2.4	7.2	60	827	7	MOLECULE:	ROD CGMP-SPECIFIC 3',5'-CYCLIC PHOSPHODIESTE
61:00:00	1x04-A	2.4	2.7	72	200	8	MOLECULE:	SH3-CONTAINING GRB2-LIKE PROTEIN 2
62:00:00	6gje-C	2.4	7.8	64	83	5	MOLECULE:	PROTEIN AMNIONLESS
63:00:00	3r6n-A	2.4	5.1	123	450	11	MOLECULE:	DESMOPLAKIN
64:00:00	3rx6-A	2.4	8.7	73	187	4	MOLECULE:	POLARITY SUPPRESSION PROTEIN
65:00:00	4tql-B	2.4	3.5	83	235	11	MOLECULE:	THREE HELIX BUNDLE
66:00:00	6sl2-A	2.4	4.5	121	619	5	MOLECULE:	CALPONIN HOMOLOGY DOMAIN PROTEIN PUTATIVE
67:00:00	5j9q-D	2.4	5.6	63	120	8	MOLECULE:	HISTONE ACETYLTRANSFERASE ESA1
68:00:00	2zdi-C	2.4	11.9	71	148	8	MOLECULE:	PREFOLDIN SUBUNIT BETA
69:00:00	1sc7-A	2.4	5.9	55	567	7	MOLECULE:	5'-D(*AP*AP*AP*AP*AP*GP*AP*CP*TP*T)-3'
70:00:00	8i4u-B	2.4	7.1	129	458	9	MOLECULE:	STRUCTURAL MAINTENANCE OF CHROMOSOMES PROTEI
71:00:00	6sih-G	2.4	7.6	102	488	8	MOLECULE:	FLAGELLAR HOOK-ASSOCIATED PROTEIN 2
72:00:00	6h7w-M	2.4	3.3	85	220	6	MOLECULE:	VACUOLAR PROTEIN SORTING-ASSOCIATED PROTEIN
73:00:00	6nyk-B	2.4	3.1	51	90	8	MOLECULE:	DESIGN CONSTRUCT XAX_GGDQ
74:00:00	6kn7-b	2.4	40	93	170	11	MOLECULE:	ACTIN, ALPHA SKELETAL MUSCLE
75:00:00	6el1-I	2.4	8.1	109	351	9	MOLECULE:	YAXA
76:00:00	7lic-A	2.4	5.2	51	377	4	MOLECULE:	MHOR5
77:00:00	7lic-C	2.4	5	51	377	2	MOLECULE:	MHOR5
78:00:00	7lic-D	2.4	5	51	377	2	MOLECULE:	MHOR5
79:00:00	5xiw-E	2.4	6.1	55	121	5	MOLECULE:	TUBULIN ALPHA-1B CHAIN
80:00:00	8eav-F	2.4	2.4	44	159	0	MOLECULE:	YAR027W OR YAR028W
81:00:00	6v8x-J	2.4	4.6	54	128	9	MOLECULE:	ENVELOPE GLYCOPROTEIN GP120
82:00:00	2mk3-A	2.4	12.3	56	68	5	MOLECULE:	TRANSMEMBRANE GLYCOPROTEIN, CHIMERIC CONSTRU
83:00:00	8euu-F	2.4	3.9	44	130	5	MOLECULE:	ENVELOPE GLYCOPROTEIN GP120

84:00:00	7mep-E	2.4	3.4	44	123	16	MOLECULE:	RM19R MAB LIGHT CHAIN
85:00:00	6osy-A	2.4	4.5	44	132	7	MOLECULE:	BG505 GP120
86:00:00	7tcn-F	2.4	10.1	49	122	2	MOLECULE:	ENVELOPE GLYCOPROTEIN GP160
87:00:00	6cue-1	2.4	4.2	45	132	2	MOLECULE:	ENVELOPE GLYCOPROTEIN GP120
88:00:00	6v8x-B	2.4	4.6	54	128	9	MOLECULE:	ENVELOPE GLYCOPROTEIN GP120
89:00:00	5i8h-B	2.4	4.9	45	132	7	MOLECULE:	BG505 SOSIP.664 GP120
90:00:00	7uoj-B	2.4	4.7	45	131	7	MOLECULE:	ENVELOPE GLYCOPROTEIN GP120
91:00:00	7mxe-C	2.4	5.7	49	124	8	MOLECULE:	8ANC195 G52K5 FAB HEAVY CHAIN
92:00:00	8dok-B	2.4	3.4	44	121	7	MOLECULE:	CRF-1_AE T/F100 ENV GP120
93:00:00	6u0n-X	2.4	2.1	41	129	5	MOLECULE:	ENVELOPE GLYCOPROTEIN GP120
94:00:00	6v8x-F	2.4	4.6	54	128	9	MOLECULE:	ENVELOPE GLYCOPROTEIN GP120
95:00:00	6x9v-B	2.4	3.9	42	121	5	MOLECULE:	HIV-1 ENVELOPE GLYCOPROTEIN BG505 SOSIP.664
96:00:00	1qo0-E	2.3	5.7	72	194	7	MOLECULE:	AMIC
97:00:00	7kzn-E	2.3	18.9	75	474	8	MOLECULE:	HEAVY CHAIN ALPHA
98:00:00	6rwb-A	2.3	29.3	153	1873	10	MOLECULE:	TOXIN,TOXIN COMPLEX SUBUNIT TCAB,PUTATIVE TO
99:00:00	2aze-A	2.3	1.5	52	149	13	MOLECULE:	TRANSCRIPTION FACTOR DP-1
0:00	6vy1-A	2.3	5.1	58	121	9	MOLECULE:	PREFOLDIN SUBUNIT ALPHA 2
1:00	6ogd-E	2.3	10.4	119	1078	6	MOLECULE:	TOXIN SUBUNIT YENA1
2:00	5dfz-C	2.3	38.6	77	783	6	MOLECULE:	VACUOLAR PROTEIN SORTING-ASSOCIATED PROTEIN
3:00	7z7s-A	2.3	21	63	131	5	MOLECULE:	NADH-QUINONE OXIDOREDUCTASE SUBUNIT F
4:00	3lss-B	2.3	35.4	58	468	2	MOLECULE:	SERYL-TRNA SYNTHETASE
5:00	3euh-B	2.3	21.5	78	321	9	MOLECULE:	CHROMOSOME PARTITION PROTEIN MUKF
6:00	6zz6-C	2.3	29.4	40	83	8	MOLECULE:	STRUCTURAL MAINTENANCE OF CHROMOSOMES PROTEI
7:00	6x80-C	2.3	5.1	115	574	6	MOLECULE:	FLAGELLIN A
8:00	6thk-A	2.3	9.6	108	466	9	MOLECULE:	PYOCIN S5
9:00	8anq-C	2.3	8.2	45	227	7	MOLECULE:	BACTERIORHODOPSIN
10:00	7etr-A	2.3	10.1	50	88	8	MOLECULE:	TRANSCRIPTIONAL REGULATOR COPG FAMILY
11:00	2d1l-A	2.3	4.6	87	249	7	MOLECULE:	METASTASIS SUPPRESSOR PROTEIN 1
12:00	8k49-T	2.3	16.9	59	627	12	MOLECULE:	VP2
13:00	1u4q-A	2.3	6.5	102	318	11	MOLECULE:	SPECTRIN ALPHA CHAIN, BRAIN
14:00	8t9l-B	2.3	16.4	70	110	4	MOLECULE:	NUCLEOPORIN POM152
15:00	2xzb-B	2.3	13.3	60	175	8	MOLECULE:	POTASSIUM-TRANSPORTING ATPASE ALPHA CHAIN 1
16:00	5lc5-h	2.3	20.5	95	134	1	MOLECULE:	NADH-UBIQUINONE OXIDOREDUCTASE CHAIN 3
17:00	5me8-A	2.3	2.6	51	107	6	MOLECULE:	INHIBITOR OF GROWTH PROTEIN 5
18:00	6cfw-I	2.3	44.5	95	114	6	MOLECULE:	MONOVALENT CATION/H+ ANTIPORTER SUBUNIT D
19:00	5n9j-V	2.3	10.6	67	99	7	MOLECULE:	MEDIATOR OF RNA POLYMERASE II TRANSCRIPTION
20:00	3b0z-A	2.3	4.1	39	41	5	MOLECULE:	FLAGELLAR BIOSYNTHETIC PROTEIN FLHB
21:00	8fck-F	2.3	12.5	100	393	6	MOLECULE:	HAUS AUGMIN-LIKE COMPLEX SUBUNIT 1
22:00	2zqm-A	2.3	3.6	58	114	3	MOLECULE:	PREFOLDIN BETA SUBUNIT 1
23:00	1jfi-B	2.3	18.7	78	135	13	MOLECULE:	5'-
24:00:00	4gwp-A	2.3	9.9	71	112	6	MOLECULE:	MEDIATOR OF RNA POLYMERASE II TRANSCRIPTION
25:00:00	8bd7-G	2.3	37.8	89	205	10	MOLECULE:	IFT88
26:00:00	3pwx-A	2.3	2.7	66	224	20	MOLECULE:	PUTATIVE FLAGELLAR HOOK-ASSOCIATED PROTEIN
27:00:00	4wid-A	2.3	3.2	67	353	10	MOLECULE:	RHUL123
28:00:00	1ylf-C	2.3	2.8	38	119	8	MOLECULE:	RRF2 FAMILY PROTEIN
29:00:00	6tgz-F	2.3	5.6	94	352	5	MOLECULE:	55 KDA IMMEDIATE-EARLY PROTEIN 1
30:00:00	6hb4-A	2.3	26.9	60	197	8	MOLECULE:	TRANSCRIPTION FACTOR A, MITOCHONDRIAL
31:00:00	5g06-P	2.3	6	56	95	7	MOLECULE:	EXOSOME COMPLEX COMPONENT RRP45
32:00:00	6wg3-B	2.3	14.9	85	693	7	MOLECULE:	STRUCTURAL MAINTENANCE OF CHROMOSOMES PROTEI
33:00:00	7v7b-A	2.3	39.6	96	1110	6	MOLECULE:	DDB1- AND CUL4-ASSOCIATED FACTOR 1
34:00:00	6xp5-D	2.3	5.5	93	134	5	MOLECULE:	MEDIATOR OF RNA POLYMERASE II TRANSCRIPTION

35:00:00	6n7p-H	2.3	10.8	57	105	2	MOLECULE:	U1 SMALL NUCLEAR RIBONUCLEOPROTEIN 70 KDA HO
36:00:00	5s5z-E	2.3	6.9	56	123	5	MOLECULE:	TUBULIN ALPHA-1B CHAIN
37:00:00	5t1y-E	2.3	8.1	49	110	6	MOLECULE:	MLA10
38:00:00	6c5w-B	2.3	7.7	89	217	2	MOLECULE:	CALCIUM UNIPORTER
39:00:00	6nrb-5	2.3	2.8	50	127	8	MOLECULE:	T-COMPLEX PROTEIN 1 SUBUNIT ALPHA
40:00:00	6xjx-E	2.3	6.3	71	153	3	MOLECULE:	CALCIUM UNIPORTER PROTEIN, MITOCHONDRIAL
41:00:00	7dad-E	2.3	10.6	60	121	8	MOLECULE:	TUBULIN ALPHA-1B CHAIN
42:00:00	5tj5-J	2.3	4	46	150	7	MOLECULE:	V-TYPE PROTON ATPASE SUBUNIT A
43:00:00	8jtd-B	2.3	4.4	46	126	7	MOLECULE:	GP120 PROTEIN OF HIV ENVELOPE TRIMER
44:00:00	6nnj-B	2.3	3.5	45	127	11	MOLECULE:	ENVELOPE GLYCOPROTEIN GP41
45:00:00	7tfn-Z	2.3	3.3	50	125	12	MOLECULE:	ENVELOPE GLYCOPROTEIN BG505 SOSIP.664 - GP12
46:00:00	8g9y-D	2.3	4.5	45	132	7	MOLECULE:	ENVELOPE GLYCOPROTEIN GP41
47:00:00	8tni-B	2.3	3.6	46	127	7	MOLECULE:	HIV-1 BG505 DS-SOSIP GP120
48:00:00	8dok-J	2.3	3.3	43	120	7	MOLECULE:	CRF-1_AE T/F100 ENV GP120
49:00:00	6ohy-E	2.3	3.5	44	120	11	MOLECULE:	ENVELOPE GLYCOPROTEIN GP160
50:00:00	8sb5-G	2.3	3.6	43	132	9	MOLECULE:	CH848.10.17.SOSIP GP120
51:00:00	6x5b-B	2.3	4	49	149	10	MOLECULE:	ENVELOPE GLYCOPROTEIN GP120
52:00:00	6uda-F	2.3	4.7	47	126	2	MOLECULE:	CH505.N279K.G458Y.SOSIP.664 GP120
53:00:00	6h4j-A	2.2	23.4	101	488	7	MOLECULE:	UBIQUITIN CARBOXYL-TERMINAL HYDROLASE 25
54:00:00	8bbt-A	2.2	4.2	58	230	7	MOLECULE:	MOBP
55:00:00	5c0w-B	2.2	6.6	52	244	12	MOLECULE:	EXOSOME COMPLEX COMPONENT RRP45
56:00:00	6j6q-F	2.2	12.7	54	161	7	MOLECULE:	PRE-MRNA-SPLICING FACTOR 8
57:00:00	1t7s-A	2.2	3.2	41	129	0	MOLECULE:	BAG-1 COCHAPERONE
58:00:00	7r6k-w	2.2	26.1	82	214	5	MOLECULE:	25S RRNA
59:00:00	5w0p-C	2.2	7.7	54	850	7	MOLECULE:	ENDOLYSIN,RHODOPSIN,S-ARRESTIN
60:00:00	5i6r-A	2.2	2.6	73	419	10	MOLECULE:	SLIT-ROBO RHO GTPASE-ACTIVATING PROTEIN 2
61:00:00	2kjy-A	2.2	4.1	46	60	4	MOLECULE:	PROTEIN PHOSPHATASE 1 REGULATORY SUBUNIT 12A
62:00:00	7e84-A	2.2	22.6	65	460	8	MOLECULE:	POTASSIUM VOLTAGE-GATED CHANNEL SUBFAMILY D
63:00:00	6xz6-A	2.2	4.3	67	213	7	MOLECULE:	GARP DOMAIN-CONTAINING PROTEIN
64:00:00	2pih-A	2.2	27.2	66	123	6	MOLECULE:	PROTEIN YMCA
65:00:00	8t1l-Y	2.2	33.6	93	154	6	MOLECULE:	MEDIATOR OF RNA POLYMERASE II TRANSCRIPTION
66:00:00	8gkh-P	2.2	20.6	79	600	4	MOLECULE:	DNA (5'-D(P*CP*GP*GP*TP*AP*CP*CP*CP*GP*GP*GP
67:00:00	7cun-B	2.2	22.5	77	1058	3	MOLECULE:	INTEGRATOR COMPLEX SUBUNIT 1
68:00:00	7wb4-b	2.2	28.2	91	636	4	MOLECULE:	OUTER NUP133
69:00:00	8cwo-T	2.2	3.5	45	87	9	MOLECULE:	16S RIBOSOMAL RNA
70:00:00	5vtl-A	2.2	3.7	61	210	11	MOLECULE:	TB427.07.360- PUTATIVE UNCHARACTERIZED METAC
71:00:00	3whj-A	2.2	5.8	47	111	11	MOLECULE:	PROBABLE 26S PROTEASOME REGULATORY SUBUNIT P
72:00:00	7byl-A	2.2	16.9	69	354	7	MOLECULE:	GREEN FLUORESCENT PROTEIN,POTASSIUM VOLTAGE-
73:00:00	8v8b-A	2.2	33.3	75	476	7	MOLECULE:	PORTAL PROTEIN
74:00:00	6y6k-A	2.2	27.7	80	1299	8	MOLECULE:	RNA-DEPENDENT RNA POLYMERASE
75:00:00	6nr8-3	2.2	11.3	68	132	12	MOLECULE:	PREFOLDIN SUBUNIT 1
76:00:00	7lid-A	2.2	6.8	67	381	9	MOLECULE:	MHOR5
77:00:00	3cqx-D	2.2	4.8	47	84	2	MOLECULE:	HEAT SHOCK COGNATE 71 KDA PROTEIN
78:00:00	1s35-A	2.2	3.1	71	211	14	MOLECULE:	SPECTRIN BETA CHAIN, ERYTHROCYTE
79:00:00	7vxs-o	2.2	23.3	93	128	6	MOLECULE:	NADH DEHYDROGENASE [UBIQUINONE] IRON-SULFUR
80:00:00	3on0-A	2.2	12.4	67	121	4	MOLECULE:	PROTEIN TRAM
81:00:00	8ip4-A	2.2	10.6	106	320	4	MOLECULE:	MAGNESIUM TRANSPORTER MRS2 HOMOLOG, MITOCHON
82:00:00	8cli-B	2.2	7.9	53	689	11	MOLECULE:	GENERAL TRANSCRIPTION FACTOR 3C POLYPEPTIDE
83:00:00	7x29-A	2.2	24	87	991	9	MOLECULE:	SPIKE GLYCOPROTEIN
84:00:00	2w0c-S	2.2	22.4	72	84	3	MOLECULE:	MAJOR CAPSID PROTEIN P2
85:00:00	6epg-C	2.2	9.8	51	56	6	MOLECULE:	EPSILON_1 ANTITOXIN

86:00:00	2qri-A	2.2	18.5	60	398	8	MOLECULE:	H-2 CLASS I HISTOCOMPATIBILITY ANTIGEN K-B A
87:00:00	8wjn-B	2.2	45.5	67	481	9	MOLECULE:	STRUCTURAL MAINTENANCE OF CHROMOSOMES PROTEI
88:00:00	7pv1-D	2.2	6	56	141	14	MOLECULE:	MICOS COMPLEX SUBUNIT MIC60 FUSED TO MIC19
89:00:00	7zu0-E	2.2	6.3	47	343	2	MOLECULE:	E3 UBIQUITIN-PROTEIN LIGASE PEP5
90:00:00	5hdi-A	2.2	15.6	66	397	6	MOLECULE:	CYTOCHROME P450 144
91:00:00	6a68-A	2.2	12.3	80	162	3	MOLECULE:	CALCIUM-DEPENDENT SECRETION ACTIVATOR 1
92:00:00	6tdx-O	2.2	3.7	40	81	10	MOLECULE:	ATP SYNTHASE F1 SUBUNIT GAMMA
93:00:00	6xl0-N	2.2	4.7	98	271	8	MOLECULE:	FLAGELLIN
94:00:00	6xl0-O	2.2	4.7	98	271	8	MOLECULE:	FLAGELLIN
95:00:00	6xky-E	2.2	5.4	97	271	8	MOLECULE:	FLAGELLIN
96:00:00	6xl0-K	2.2	4.9	98	271	9	MOLECULE:	FLAGELLIN
97:00:00	6xky-I	2.2	4.1	97	271	7	MOLECULE:	FLAGELLIN
98:00:00	6xky-M	2.2	4.2	95	271	6	MOLECULE:	FLAGELLIN
99:00:00	6xky-D	2.2	5.5	97	271	7	MOLECULE:	FLAGELLIN
0:00	6xky-L	2.2	5.5	97	271	7	MOLECULE:	FLAGELLIN
1:00	6xky-K	2.2	5.6	96	271	6	MOLECULE:	FLAGELLIN
2:00	3k8v-A	2.2	8.1	97	323	3	MOLECULE:	FLAGELLIN HOMOLOG
3:00	6xky-G	2.2	5.5	98	271	7	MOLECULE:	FLAGELLIN
4:00	6xky-C	2.2	5.6	96	271	6	MOLECULE:	FLAGELLIN
5:00	6x80-I	2.2	7.1	108	574	10	MOLECULE:	FLAGELLIN A
6:00	6xky-F	2.2	4.1	97	271	7	MOLECULE:	FLAGELLIN
7:00	6ek7-A	2.2	3.3	83	364	4	MOLECULE:	YAXA
8:00	4zwq-E	2.2	3.2	50	135	10	MOLECULE:	RECOMBINATION PROTEIN UVSY
9:00	1fxk-A	2.2	2.8	48	107	2	MOLECULE:	PREFOLDIN
10:00	8jsn-C	2.2	12.3	67	99	6	MOLECULE:	RNA-DIRECTED RNA POLYMERASE L
11:00	5jsb-L	2.2	2.6	44	116	9	MOLECULE:	INDUCED MYELOID LEUKEMIA CELL DIFFERENTIATIO
12:00	6u59-I	2.2	3.5	44	120	5	MOLECULE:	SOSIP.664 GP120,SOSIP.664 GP120
13:00	6w03-B	2.2	4.9	45	124	7	MOLECULE:	ENVELOPE GLYCOPROTEIN GP41
14:00	5wdu-J	2.2	3.9	44	126	0	MOLECULE:	ENVELOPE GLYCOPROTEIN GP160
15:00	6opn-B	2.2	3.4	42	124	19	MOLECULE:	ENVELOPE GLYCOPROTEIN GP160
16:00	6ieq-B	2.2	4.1	47	131	4	MOLECULE:	ENVELOPE GLYCOPROTEIN GP160
17:00	6mug-B	2.2	3.4	44	123	9	MOLECULE:	ENVELOPE GLYCOPROTEIN GP160
18:00	6opq-F	2.2	3.3	47	128	2	MOLECULE:	ENVELOPE GLYCOPROTEIN GP160
19:00	8sb1-B	2.2	3.5	48	124	8	MOLECULE:	CH848.10.17 GP120
20:00	6opq-G	2.2	3.3	47	128	2	MOLECULE:	ENVELOPE GLYCOPROTEIN GP160
21:00	8sb1-G	2.2	3.5	48	124	8	MOLECULE:	CH848.10.17 GP120
22:00	6nf2-I	2.2	15	51	132	4	MOLECULE:	ENVELOPE GLYCOPROTEIN GP120
23:00	8sax-B	2.2	10.5	56	132	0	MOLECULE:	CH848.10.17.SOSIP GP120
24:00:00	7lpn-B	2.2	5.1	44	121	5	MOLECULE:	HIV-1 ENVELOPE GLYCOPROTEIN BG505 SOSIP.664
25:00:00	6oz4-B	2.2	4	44	122	2	MOLECULE:	ENVELOPE GLYCOPROTEIN GP160
26:00:00	6muf-B	2.2	3.4	44	122	9	MOLECULE:	ENVELOPE GLYCOPROTEIN GP160
27:00:00	6nf5-B	2.2	4	44	122	5	MOLECULE:	HIV-1 ENV BG505 MD64 N332-GT5 SOSIP GP120
28:00:00	8g9w-O	2.2	17.2	48	132	0	MOLECULE:	ENVELOPE GLYCOPROTEIN GP41
29:00:00	8tnh-D	2.2	9.3	47	126	9	MOLECULE:	HIV-1 BG505 DS-SOSIP GP120
30:00:00	8d5c-Z	2.2	7.5	43	129	7	MOLECULE:	ENVELOPE GLYCOPROTEIN GP41
31:00:00	7sq1-B	2.2	4.2	42	121	5	MOLECULE:	TRANSMEMBRANE PROTEIN GP41
32:00:00	6uda-J	2.2	4.7	45	126	4	MOLECULE:	CH505.N279K.G458Y.SOSIP.664 GP120
33:00:00	8sav-F	2.2	3.8	46	136	9	MOLECULE:	CH848.0526.25 GP120
34:00:00	5t3s-B	2.2	3.7	40	122	5	MOLECULE:	ENVELOPE GLYCOPROTEIN GP160
35:00:00	6opn-E	2.2	3.4	42	124	19	MOLECULE:	ENVELOPE GLYCOPROTEIN GP160
36:00:00	8dok-F	2.2	3.4	44	117	7	MOLECULE:	CRF-1_AE T/F100 ENV GP120

37:00:00	4gfq-A	2.1	2.9	70	186	6	MOLECULE:	RIBOSOME-RECYCLING FACTOR
38:00:00	8f2r-E	2.1	3.1	43	205	12	MOLECULE:	COMM DOMAIN-CONTAINING PROTEIN 1
39:00:00	7x5e-B	2.1	12.6	64	106	6	MOLECULE:	TRANSCRIPTION FACTOR MAFG
40:00:00	3g5o-A	2.1	8.4	52	92	2	MOLECULE:	UNCHARACTERIZED PROTEIN RV2865
41:00:00	8p65-J	2.1	7.1	54	64	7	MOLECULE:	CYTOCHROME B-C1 COMPLEX SUBUNIT 1, MITOCHOND
42:00:00	6j72-A	2.1	3.3	80	561	8	MOLECULE:	ISONIAZID INDUCIBLE GENE PROTEIN INIA
43:00:00	5szj-B	2.1	2.2	57	145	5	MOLECULE:	RAS-RELATED PROTEIN RAB-10
44:00:00	7e40-D	2.1	18.9	87	345	5	MOLECULE:	PROTEIN PHOSPHATE STARVATION RESPONSE 2
45:00:00	5yud-C	2.1	2.9	45	75	2	MOLECULE:	BACULOVIRAL IAP REPEAT-CONTAINING PROTEIN 1E
46:00:00	8eki-A	2.1	3.7	71	170	3	MOLECULE:	PROTEIN TRANSPORT PROTEIN SEC20
47:00:00	6ww6-B	2.1	3.6	64	192	9	MOLECULE:	RESPONSE REGULATOR
48:00:00	6qeq-A	2.1	2.5	37	111	5	MOLECULE:	PCFF
49:00:00	7qlr-A	2.1	17.2	92	603	2	MOLECULE:	CDHS1_22 PUTATIVE TAIL FIBER PROTEIN
50:00:00	2oar-A	2.1	20.9	62	125	6	MOLECULE:	LARGE-CONDUCTANCE MECHANOSENSITIVE CHANNEL
51:00:00	8ox6-A	2.1	13.7	55	575	5	MOLECULE:	PHOSPHOLIPID-TRANSPORTING ATPASE IC
52:00:00	7nyw-A	2.1	15.1	102	685	7	MOLECULE:	CHROMOSOME PARTITION PROTEIN MUKB
53:00:00	6th1-R	2.1	5.3	91	360	7	MOLECULE:	IMMEDIATE EARLY PROTEIN 1
54:00:00	7f6j-C	2.1	1.8	40	106	8	MOLECULE:	RAS-RELATED PROTEIN RAB-7A
55:00:00	6cum-A	2.1	4.9	39	51	3	MOLECULE:	LAO/AO TRANSPORT SYSTEM ATPASE
56:00:00	4rp5-A	2.1	5.9	48	94	8	MOLECULE:	DISKS LARGE 1 TUMOR SUPPRESSOR PROTEIN
57:00:00	3cf4-A	2.1	34.9	73	766	7	MOLECULE:	ACETYL-COA DECARBOXYLASE/SYNTHASE ALPHA SUBU
58:00:00	4y66-D	2.1	17.5	93	208	4	MOLECULE:	MND1
59:00:00	5che-A	2.1	7.6	66	427	2	MOLECULE:	GLUTAMYL-TRNA REDUCTASE 1, CHLOROPLASTIC
60:00:00	2odu-A	2.1	3.1	77	217	13	MOLECULE:	PLECTIN 1
61:00:00	6yj4-g	2.1	7.5	79	198	5	MOLECULE:	NADH-UBIQUINONE OXIDOREDUCTASE CHAIN 3
62:00:00	6t9i-K	2.1	11.5	68	279	3	MOLECULE:	TRANSCRIPTION FACTOR SPT20
63:00:00	6yj4-o	2.1	6.1	46	83	4	MOLECULE:	NADH-UBIQUINONE OXIDOREDUCTASE CHAIN 3
64:00:00	5lbm-C	2.1	3.3	41	90	12	MOLECULE:	TRANSCRIPTIONAL REPRESSOR FRMR
65:00:00	2ox6-C	2.1	11.1	46	163	11	MOLECULE:	HYPOTHETICAL PROTEIN SO3848
66:00:00	6rlr-A	2.1	4.8	46	80	2	MOLECULE:	CD9 ANTIGEN
67:00:00	3av0-B	2.1	12	74	365	11	MOLECULE:	DNA DOUBLE-STRAND BREAK REPAIR PROTEIN MRE11
68:00:00	6swy-1	2.1	21.8	68	542	1	MOLECULE:	VACUOLAR IMPORT AND DEGRADATION PROTEIN 28
69:00:00	2q83-A	2.1	27.1	63	332	16	MOLECULE:	YTAA PROTEIN
70:00:00	6xl0-A	2.1	4.6	98	271	9	MOLECULE:	FLAGELLIN
71:00:00	6xky-B	2.1	5.6	102	271	8	MOLECULE:	FLAGELLIN
72:00:00	6xky-R	2.1	5.3	98	271	8	MOLECULE:	FLAGELLIN
73:00:00	6xky-S	2.1	5.5	97	271	7	MOLECULE:	FLAGELLIN
74:00:00	6xl0-L	2.1	4.7	98	271	8	MOLECULE:	FLAGELLIN
75:00:00	6xl0-Q	2.1	4.2	92	271	9	MOLECULE:	FLAGELLIN
76:00:00	5ik2-G	2.1	4	82	285	10	MOLECULE:	ATP SYNTHASE SUBUNIT ALPHA
77:00:00	6xl0-T	2.1	4.2	92	271	10	MOLECULE:	FLAGELLIN
78:00:00	6xky-Q	2.1	5.3	98	271	8	MOLECULE:	FLAGELLIN
79:00:00	6xl0-M	2.1	4.2	92	271	10	MOLECULE:	FLAGELLIN
80:00:00	6xl0-C	2.1	4.2	92	271	10	MOLECULE:	FLAGELLIN
81:00:00	2vrz-A	2.1	6.8	55	98	9	MOLECULE:	VIRULENCE FACTOR ESXA
82:00:00	5j9w-H	2.1	5.7	63	118	13	MOLECULE:	HISTONE ACETYLTRANSFERASE ESA1
83:00:00	6cnm-A	2.1	19.1	98	359	3	MOLECULE:	INTERMEDIATE CONDUCTANCE CALCIUM-ACTIVATED P
84:00:00	5t1y-A	2.1	8	49	108	6	MOLECULE:	MLA10
85:00:00	7u1s-E	2.1	12.1	68	1440	3	MOLECULE:	ATP-SENSITIVE INWARD RECTIFIER POTASSIUM CHA
86:00:00	1i4l-A	2.1	4	60	185	10	MOLECULE:	ARFAPTIN 2
87:00:00	7yo0-H	2.1	22.7	59	262	8	MOLECULE:	CALCIUM-ACTIVATED POTASSIUM CHANNEL SUBUNIT

88:00:00	1i4d-A	2.1	3.9	60	188	5	MOLECULE:	ARFAPTIN 2
89:00:00	5da9-A	2.1	13.6	75	433	4	MOLECULE:	PUTATIVE UNCHARACTERIZED PROTEIN,PUTATIVE UN
90:00:00	1i4t-B	2.1	4.6	57	179	4	MOLECULE:	ARFAPTIN 2
91:00:00	5vox-b	2.1	24.2	100	627	9	MOLECULE:	V-TYPE PROTON ATPASE CATALYTIC SUBUNIT A,V-T
92:00:00	7sb2-A	2.1	5.4	45	299	2	MOLECULE:	GLDM
93:00:00	6cnm-C	2.1	25.9	84	359	6	MOLECULE:	INTERMEDIATE CONDUCTANCE CALCIUM-ACTIVATED P
94:00:00	6ysl-C	2.1	6.6	70	255	9	MOLECULE:	MOTILITY PROTEIN A
95:00:00	6nfc-I	2.1	3.7	42	120	5	MOLECULE:	BASE-BINDING RM20A3 FRAGMENT ANTIGEN BINDING
96:00:00	7l7u-F	2.1	4.3	47	145	15	MOLECULE:	BG505 SOSIP.V5.2(7S) - GP120
97:00:00	6x9u-B	2.1	3.2	42	121	10	MOLECULE:	HIV-1 ENVELOPE GLYCOPROTEIN BG505 SOSIP.664
98:00:00	7lu9-b	2.1	9.9	49	135	10	MOLECULE:	DH851.3 LIGHT CHAIN
99:00:00	8g9w-D	2.1	4.3	47	132	4	MOLECULE:	ENVELOPE GLYCOPROTEIN GP41
0:00	8g9x-O	2.1	15.4	50	132	4	MOLECULE:	ENVELOPE GLYCOPROTEIN GP41
1:00	7mxd-B	2.1	4.9	47	136	9	MOLECULE:	ENVELOPE GLYCOPROTEIN GP120
2:00	8czz-B	2.1	7.9	43	125	2	MOLECULE:	CRF01_AE T/F100 HIV-1 GP120
3:00	7sq1-D	2.1	4.2	42	121	5	MOLECULE:	TRANSMEMBRANE PROTEIN GP41
4:00	8tnh-B	2.1	3.5	47	127	9	MOLECULE:	HIV-1 BG505 DS-SOSIP GP120
5:00	7lpn-A	2.1	4.6	43	121	5	MOLECULE:	HIV-1 ENVELOPE GLYCOPROTEIN BG505 SOSIP.664
6:00	6nfc-G	2.1	4.1	41	120	7	MOLECULE:	BASE-BINDING RM20A3 FRAGMENT ANTIGEN BINDING
7:00	5v8l-B	2.1	4.5	46	126	2	MOLECULE:	GP120
8:00	6x5b-K	2.1	3.5	49	149	4	MOLECULE:	ENVELOPE GLYCOPROTEIN GP120
9:00	6ohy-F	2.1	7	49	120	10	MOLECULE:	ENVELOPE GLYCOPROTEIN GP160
10:00	8ttw-J	2.1	4.9	44	123	5	MOLECULE:	ENVELOPE GLYCOPROTEIN GP160
11:00	8flw-B	2.1	3	45	123	7	MOLECULE:	PGT145 DU303 HEAVY
12:00	8dow-J	2.1	10.9	48	132	6	MOLECULE:	ENVELOPE GLYCOPROTEIN GP120
13:00	5g06-J	2	27.1	80	993	8	MOLECULE:	EXOSOME COMPLEX COMPONENT RRP45
14:00	8ffz-A	2	10.2	46	314	4	MOLECULE:	TRANSCRIPTION FACTOR IIIA
15:00	1sjj-A	2	14.5	139	863	11	MOLECULE:	ACTININ
16:00	4mer-D	2	7.1	60	97	10	MOLECULE:	STREPTOCOCCAL HISTIDINE-RICH GLYCOPROTEIN IN
17:00	6ezn-E	2	7.4	52	85	4	MOLECULE:	DOLICHYL-DIPHOSPHOOLIGOSACCHARIDE--PROTEIN
18:00	6lcn-F	2	23.7	81	309	2	MOLECULE:	SERINE O-ACETYLTRANSFERASE
19:00	7x29-B	2	12.9	83	1190	6	MOLECULE:	SPIKE GLYCOPROTEIN
20:00	6q0x-A	2	5.6	92	373	4	MOLECULE:	SORTING NEXIN MVP1
21:00	6wg3-A	2	21.8	79	561	5	MOLECULE:	STRUCTURAL MAINTENANCE OF CHROMOSOMES PROTEI
22:00	4drf-A	2	4.5	54	408	11	MOLECULE:	METALLOPHOSPHOESTERASE
23:00	6zsi-D	2	1.6	34	133	21	MOLECULE:	RAS-RELATED PROTEIN RAB-8A
24:00:00	8ap7-j	2	40	81	168	1	MOLECULE:	ATP SYNTHASE SUBUNIT A
25:00:00	8pjn-b	2	6.1	80	296	6	MOLECULE:	E3 UBIQUITIN-PROTEIN TRANSFERASE RMND5A
26:00:00	7o4i-1	2	8.5	44	522	11	MOLECULE:	GENERAL TRANSCRIPTION AND DNA REPAIR FACTOR
27:00:00	7rz4-A	2	9.5	54	993	9	MOLECULE:	GLUTAMATE RECEPTOR 2
28:00:00	8ebw-H	2	5	75	532	12	MOLECULE:	TFIIH BASAL TRANSCRIPTION FACTOR COMPLEX HEL
29:00:00	6al9-B	2	3.5	41	91	12	MOLECULE:	CHORISMATE MUTASE
30:00:00	7nyw-B	2	16.8	97	858	5	MOLECULE:	CHROMOSOME PARTITION PROTEIN MUKB
31:00:00	7cgp-C	2	32.2	98	223	11	MOLECULE:	MITOCHONDRIAL IMPORT INNER MEMBRANE TRANSLOC
32:00:00	6zyd-A	2	29.3	50	323	2	MOLECULE:	LOW CONDUCTANCE MECHANOSENSITIVE CHANNEL YNA
33:00:00	3o1j-A	2	4.4	86	273	9	MOLECULE:	SENSOR PROTEIN TORS
34:00:00	1pug-A	2	3.5	43	94	12	MOLECULE:	HYPOTHETICAL UPF0133 PROTEIN YBAB
35:00:00	6xky-N	2	4.1	97	271	7	MOLECULE:	FLAGELLIN
36:00:00	3zgx-B	2	34.6	77	351	8	MOLECULE:	CHROMOSOME PARTITION PROTEIN SMC
37:00:00	5j9u-K	2	5.8	61	120	16	MOLECULE:	HISTONE ACETYLTRANSFERASE ESA1
38:00:00	8fef-F	2	34.8	72	399	1	MOLECULE:	VIRULENCE FACTOR MCE FAMILY PROTEIN

39:00:00	3jac-B	2	37.6	94	918	1	MOLECULE:	PIEZO-TYPE MECHANOSENSITIVE ION CHANNEL COMP
40:00:00	5j9u-D	2	5.5	63	120	8	MOLECULE:	HISTONE ACETYLTRANSFERASE ESA1
41:00:00	7nh9-A	2	6	91	327	11	MOLECULE:	CMAX PROTEIN
42:00:00	2x2v-B	2	1.9	37	69	5	MOLECULE:	ATP SYNTHASE SUBUNIT C
43:00:00	7ur6-B	2	11.3	49	132	6	MOLECULE:	GP120
44:00:00	6um5-B	2	4.1	48	131	2	MOLECULE:	CH848 10.17 DT GP120
45:00:00	7ucg-M	2	4.4	44	134	7	MOLECULE:	ENVELOPE GLYCOPROTEIN GP41
46:00:00	6mu6-B	2	3.5	45	128	11	MOLECULE:	ENVELOPE GLYCOPROTEIN GP160
47:00:00	6u59-D	2	4.2	44	120	7	MOLECULE:	SOSIP.664 GP120,SOSIP.664 GP120
48:00:00	6vtt-B	2	5.1	46	129	7	MOLECULE:	ENVELOPE GLYCOPROTEIN GP120
49:00:00	6udj-F	2	3.3	44	123	9	MOLECULE:	10-1074 FAB HEAVY CHAIN

DALI results: MnA-1 Penton

No:	Chain	Z	rmsd	lali	nres	%id	PDB Description
1:00	6z7q-B	11.8	3.9	224	416	14	MOLECULE: PENTON PROTEIN
2:00	6z7q-C	11.7	3.9	224	416	14	MOLECULE: PENTON PROTEIN
3:00	6z7q-D	11.7	3.9	224	416	14	MOLECULE: PENTON PROTEIN
4:00	6z7q-E	11.7	3.9	224	416	14	MOLECULE: PENTON PROTEIN
5:00	2c6s-K	11.6	3.9	222	443	15	MOLECULE: ADENOVIRUS 2,12 PENTON BASE CHIMERA
6:00	2c6s-H	11.6	3.9	223	443	15	MOLECULE: ADENOVIRUS 2,12 PENTON BASE CHIMERA
7:00	2c6s-D	11.6	3.9	222	443	15	MOLECULE: ADENOVIRUS 2,12 PENTON BASE CHIMERA
8:00	2c6s-G	11.6	3.9	223	443	15	MOLECULE: ADENOVIRUS 2,12 PENTON BASE CHIMERA
9:00	2c6s-C	11.6	3.9	223	443	15	MOLECULE: ADENOVIRUS 2,12 PENTON BASE CHIMERA
10:00	2c6s-L	11.6	3.9	222	443	15	MOLECULE: ADENOVIRUS 2,12 PENTON BASE CHIMERA
11:00	2c6s-F	11.6	3.9	222	443	15	MOLECULE: ADENOVIRUS 2,12 PENTON BASE CHIMERA
12:00	2c6s-A	11.6	3.9	222	443	15	MOLECULE: ADENOVIRUS 2,12 PENTON BASE CHIMERA
13:00	6z7q-A	11.6	3.9	222	416	14	MOLECULE: PENTON PROTEIN
14:00	2c6s-J	11.6	3.9	223	443	15	MOLECULE: ADENOVIRUS 2,12 PENTON BASE CHIMERA
15:00	2c6s-N	11.6	3.9	224	443	15	MOLECULE: ADENOVIRUS 2,12 PENTON BASE CHIMERA
16:00	2c6s-O	11.6	3.9	222	443	15	MOLECULE: ADENOVIRUS 2,12 PENTON BASE CHIMERA
17:00	2c6s-E	11.6	3.9	223	443	15	MOLECULE: ADENOVIRUS 2,12 PENTON BASE CHIMERA
18:00	2c6s-I	11.6	3.9	222	443	15	MOLECULE: ADENOVIRUS 2,12 PENTON BASE CHIMERA
19:00	2c6s-B	11.6	3.9	223	443	15	MOLECULE: ADENOVIRUS 2,12 PENTON BASE CHIMERA
20:00	2c6s-M	11.5	3.9	222	443	14	MOLECULE: ADENOVIRUS 2,12 PENTON BASE CHIMERA
21:00	2c9g-B	11.5	4	223	447	15	MOLECULE: PENTON PROTEIN
22:00	2c9g-A	11.5	4	223	447	15	MOLECULE: PENTON PROTEIN
23:00	2c9g-D	11.5	4	223	447	15	MOLECULE: PENTON PROTEIN
24:00:00	2c9g-C	11.5	4	223	447	15	MOLECULE: PENTON PROTEIN
25:00:00	2c9g-E	11.5	4	223	447	15	MOLECULE: PENTON PROTEIN
26:00:00	1x9p-A	11.4	4	222	460	15	MOLECULE: PENTON PROTEIN
27:00:00	2c9f-B	11.4	4	222	440	15	MOLECULE: PENTON PROTEIN
28:00:00	2c9f-D	11.4	4.1	223	440	15	MOLECULE: PENTON PROTEIN
29:00:00	1x9t-A	11.4	4.1	223	440	15	MOLECULE: PENTON PROTEIN
30:00:00	2c9f-A	11.4	4	222	440	15	MOLECULE: PENTON PROTEIN
31:00:00	2c9f-E	11.4	4.1	223	440	15	MOLECULE: PENTON PROTEIN
32:00:00	2c9f-C	11.4	4.1	223	440	15	MOLECULE: PENTON PROTEIN
33:00:00	3izo-A	11.2	4	224	455	14	MOLECULE: PENTON PROTEIN
34:00:00	3izo-E	11.2	4	224	455	14	MOLECULE: PENTON PROTEIN
35:00:00	3izo-D	11.2	4	224	455	14	MOLECULE: PENTON PROTEIN
36:00:00	3izo-C	11.2	4	224	455	14	MOLECULE: PENTON PROTEIN
37:00:00	6g41-F	11.2	3.2	153	301	7	MOLECULE: MINOR CAPSID PR OTEIN
38:00:00	4aqq-A	11.1	4.1	226	462	14	MOLECULE: L2 PROTEIN III (PENTON BASE)
39:00:00	3izo-B	11.1	3.9	222	455	14	MOLECULE: PENTON PROTEIN
40:00:00	4ar2-A	11	4.2	226	455	13	MOLECULE: L2 PROTEIN III (PENTON BASE)
41:00:00	6g42-D	10.9	3.2	155	302	6	MOLECULE: MINOR CAPSID PR OTEIN
42:00:00	6g41-I	10.9	3.2	154	301	6	MOLECULE: MINOR CAPSID PR OTEIN

43:00:00	6g42-E	10.8	3.1	154	301	6	MOLECULE:	MINOR CAPSID PR	OTEIN
44:00:00	6g41-J	10.8	3.2	154	301	6	MOLECULE:	MINOR CAPSID PR	OTEIN
45:00:00	6g42-C	10.7	3.2	155	302	6	MOLECULE:	MINOR CAPSID PR	OTEIN
46:00:00	6g41-A	10.7	3.5	155	297	6	MOLECULE:	MINOR CAPSID PR	OTEIN
47:00:00	6g42-B	10.7	3.2	154	301	6	MOLECULE:	MINOR CAPSID PR	OTEIN
48:00:00	6g42-A	10.7	3.1	153	301	7	MOLECULE:	MINOR CAPSID PR	OTEIN
49:00:00	6g41-C	10.7	3.2	154	301	6	MOLECULE:	MINOR CAPSID PR	OTEIN
50:00:00	6g41-E	10.7	3.1	152	301	7	MOLECULE:	MINOR CAPSID PR	OTEIN
51:00:00	6g41-H	10.7	3.1	152	301	7	MOLECULE:	MINOR CAPSID PR	OTEIN
52:00:00	6g41-D	10.6	3.2	153	291	7	MOLECULE:	MINOR CAPSID PR	OTEIN
53:00:00	6g41-B	10.6	3.1	152	295	7	MOLECULE:	MINOR CAPSID PR	OTEIN
54:00:00	8rbt-S	8.9	3.3	209	663	12	MOLECULE:	MAJOR CAPSID PR	OTEIN
55:00:00	8coi-I	8.6	2.6	99	249	15	MOLECULE:	ADDOBODY	
56:00:00	6g41-G	8.1	3.1	152	287	7	MOLECULE:	MINOR CAPSID PR	OTEIN
57:00:00	8coi-A	8	2.7	96	245	15	MOLECULE:	ADDOBODY	
58:00:00	8coi-N	8	2.6	95	232	16	MOLECULE:	ADDOBODY	
59:00:00	3j26-N	8	4	165	362	7	MOLECULE:	CAPSID PROTEIN	V20
60:00:00	8coi-C	7.9	2.7	99	249	15	MOLECULE:	ADDOBODY	
61:00:00	8coi-Q	7.9	2.6	96	232	16	MOLECULE:	ADDOBODY	
62:00:00	8coi-J	7.9	2.6	94	243	15	MOLECULE:	ADDOBODY	
63:00:00	8coi-E	7.8	2.5	94	228	15	MOLECULE:	ADDOBODY	
64:00:00	8coi-P	7.6	2.6	92	243	16	MOLECULE:	ADDOBODY	
65:00:00	8coi-H	7.5	2.5	96	243	16	MOLECULE:	ADDOBODY	
66:00:00	8coi-F	7.2	2.6	98	240	15	MOLECULE:	ADDOBODY	
67:00:00	8coi-B	6.7	2.5	96	246	16	MOLECULE:	ADDOBODY	
68:00:00	8coi-G	6.7	2.6	96	243	16	MOLECULE:	ADDOBODY	
69:00:00	8coi-M	6.7	2.7	96	237	16	MOLECULE:	ADDOBODY	
70:00:00	8coi-R	6.7	2.6	93	227	15	MOLECULE:	ADDOBODY	
71:00:00	8coi-D	6.7	2.6	96	244	16	MOLECULE:	ADDOBODY	
72:00:00	8coi-K	6.6	2.6	95	250	16	MOLECULE:	ADDOBODY	
73:00:00	8qb3-E	6.5	2.9	89	233	15	MOLECULE:	ADDOBODY	
74:00:00	8coi-S	6.5	2.6	94	230	15	MOLECULE:	ADDOBODY	
75:00:00	8coi-L	6.5	2.6	95	244	16	MOLECULE:	ADDOBODY	
76:00:00	8coi-T	6.3	2.7	85	212	15	MOLECULE:	ADDOBODY	
77:00:00	8coi-O	6.3	2.5	91	229	14	MOLECULE:	ADDOBODY	
78:00:00	6wpc-C	6	3.3	112	588	8	MOLECULE:	CRY1A.2	
79:00:00	6ds5-E	5.9	3.8	105	160	13	MOLECULE:	SEIPIN	
80:00:00	6ds5-G	5.9	3.8	105	160	13	MOLECULE:	SEIPIN	
81:00:00	7cz6-C	5.9	3.2	99	126	7	MOLECULE:	CAPSID PROTEIN	
82:00:00	7rsl-A	5.8	5.3	108	235	12	MOLECULE:	SEIPIN	
83:00:00	7rsl-F	5.8	3.2	106	235	12	MOLECULE:	SEIPIN	
84:00:00	7rsl-D	5.8	3.2	106	235	12	MOLECULE:	SEIPIN	
85:00:00	1ciy-A	5.8	3.8	107	577	7	MOLECULE:	CRYIA(A)	
86:00:00	7rsl-G	5.8	5.2	109	235	13	MOLECULE:	SEIPIN	

87:00:00	6ds5-F	5.8	3.8	105	160	13	MOLECULE:	SEIPIN			
88:00:00	6ds5-C	5.8	3.8	105	160	13	MOLECULE:	SEIPIN			
89:00:00	6ds5-H	5.8	3.8	105	160	13	MOLECULE:	SEIPIN			
90:00:00	6ds5-K	5.8	3.8	105	160	13	MOLECULE:	SEIPIN			
91:00:00	6ds5-D	5.8	3.8	105	160	13	MOLECULE:	SEIPIN			
92:00:00	6ds5-A	5.8	3.8	105	160	13	MOLECULE:	SEIPIN			
93:00:00	6ds5-B	5.8	3.8	105	160	13	MOLECULE:	SEIPIN			
94:00:00	6ds5-I	5.8	3.8	105	160	13	MOLECULE:	SEIPIN			
95:00:00	6ds5-J	5.8	3.6	103	160	13	MOLECULE:	SEIPIN			
96:00:00	7rsl-I	5.8	5.2	110	235	13	MOLECULE:	SEIPIN			
97:00:00	7rsl-B	5.8	3.1	102	235	13	MOLECULE:	SEIPIN			
98:00:00	7rsl-C	5.8	5.2	111	235	13	MOLECULE:	SEIPIN			
99:00:00	6owk-A	5.7	3.2	106	582	7	MOLECULE:	PESTICIDAL CRYS	TAL PRO	TEIN	CRY1BE, C
0:00	6za2-A	5.7	3.9	102	1081	7	MOLECULE:	POR SECRETION S	YSTEM P	ROTEI	N PORU
1:00	7rsl-E	5.7	5.3	110	235	12	MOLECULE:	SEIPIN			
2:00	6wpc-B	5.7	3.8	110	588	10	MOLECULE:	CRY1A.2			
3:00	9buv-A	5.6	4	106	573	8	MOLECULE:	PESTICIDAL CRYS	TAL PRO	TEIN	CRY1FA
4:00	6wpc-A	5.6	3.9	111	588	9	MOLECULE:	CRY1A.2			
5:00	8qb3-C	5.5	2.7	88	216	15	MOLECULE:	ADDOBODY			
6:00	2c9k-A	5.5	4	108	598	7	MOLECULE:	PESTICIDAL CRYS	TAL PRO	TEIN	CRY4AA
7:00	7rsl-J	5.4	3.2	106	235	10	MOLECULE:	SEIPIN			
8:00	7rsl-H	5.4	3.2	106	235	10	MOLECULE:	SEIPIN			
9:00	3eb7-A	5.3	3.5	106	589	8	MOLECULE:	INSECTICIDAL DE	LTA-END	OTOXI	N CRY8EA1
10:00	3eb7-C	5.3	3.2	104	588	10	MOLECULE:	INSECTICIDAL DE	LTA-END	OTOXI	N CRY8EA1
11:00	8qb3-D	5.3	2.8	85	206	15	MOLECULE:	ADDOBODY			
12:00	8qb3-A	5.2	2.7	87	233	15	MOLECULE:	ADDOBODY			
13:00	3eb7-B	5.2	3.5	112	589	13	MOLECULE:	INSECTICIDAL DE	LTA-END	OTOXI	N CRY8EA1
14:00	4qx0-A	5.2	3.7	113	584	5	MOLECULE:	PESTICIDAL CRYS	TAL PRO	TEIN	CRY3AA
15:00	6za2-B	5.2	3.9	100	1083	7	MOLECULE:	POR SECRETION S	YSTEM P	ROTEI	N PORU
16:00	4qx2-A	5.2	3.6	113	584	4	MOLECULE:	PESTICIDAL CRYS	TAL PRO	TEIN	CRY3AA
17:00	4qx3-A	5.2	3.5	112	584	5	MOLECULE:	PESTICIDAL CRYS	TAL PRO	TEIN	CRY3AA
18:00	6ovb-A	5.1	4.4	110	566	10	MOLECULE:	ACTIVE CORE CRY	STAL TO	XIN P	ROTEIN 1D
19:00	8j3x-B	5.1	3.3	91	390	7	MOLECULE:	PUTATIVE POLYSA	CCHARID	E-BIN	DING PROT
20:00	6lfp-A	5.1	3.6	111	582	7	MOLECULE:	CRY3AA PROTEIN			
21:00	8qb3-B	5.1	3	89	233	15	MOLECULE:	ADDOBODY			
22:00	8j3y-A	5	4.2	93	392	5	MOLECULE:	PUTATIVE POLYSA	CCHARID	E-BIN	DING PROT
23:00	7ear-A	5	3.3	108	584	5	MOLECULE:	CRYSTALINE ENTO	MOCIDAL	PROT	OXIN
24:00:00	4qx1-A	5	3.9	116	584	5	MOLECULE:	PESTICIDAL CRYS	TAL PRO	TEIN	CRY3AA
25:00:00	4arx-D	5	4	105	576	8	MOLECULE:	PESTICIDAL CRYS	TAL PRO	TEIN	CRY1AC
26:00:00	1dlc-A	5	3.3	109	584	6	MOLECULE:	DELTA-ENDOTOXIN	CRYIII	A	
27:00:00	6mlu-B	5	4.2	97	153	6	MOLECULE:	SEIPIN			
28:00:00	4arx-A	5	4.1	106	578	8	MOLECULE:	PESTICIDAL CRYS	TAL PRO	TEIN	CRY1AC
29:00:00	4arx-C	4.9	4	106	577	8	MOLECULE:	PESTICIDAL CRYS	TAL PRO	TEIN	CRY1AC
30:00:00	4arx-B	4.9	4	106	578	8	MOLECULE:	PESTICIDAL CRYS	TAL PRO	TEIN	CRY1AC

31:00:00	8j3x-A	4.9	4.2	92	389	5	MOLECULE:	PUTATIVE POLYSA	CCHARID	E-BIN	DING PROT
32:00:00	4ary-B	4.9	4	105	580	9	MOLECULE:	PESTICIDAL CRYS	TAL PRO	TEIN	CRY1AC
33:00:00	4ary-A	4.9	4	106	578	8	MOLECULE:	PESTICIDAL CRYS	TAL PRO	TEIN	CRY1AC
34:00:00	4ary-C	4.9	4	106	577	8	MOLECULE:	PESTICIDAL CRYS	TAL PRO	TEIN	CRY1AC
35:00:00	4w8j-A	4.8	3.9	103	1017	8	MOLECULE:	PESTICIDAL CRYS	TAL PRO	TEIN	CRY1AC
36:00:00	5hly-A	4.8	4.3	101	299	10	MOLECULE:	INHIBIN BETA A	CHAIN		
37:00:00	5hlz-A	4.8	4.3	104	204	11	MOLECULE:	INHIBIN BETA A	CHAIN		
38:00:00	5hlz-C	4.7	4.4	100	204	8	MOLECULE:	INHIBIN BETA A	CHAIN		
39:00:00	6dj4-A	4.7	3.7	112	571	5	MOLECULE:	CRY1A.105			
40:00:00	1ji6-A	4.6	3.5	110	589	7	MOLECULE:	PESTICIDIAL CRY	STAL PR	OTEIN	CRY3BB
41:00:00	5g56-A	4.6	4.4	100	706	12	MOLECULE:	CARBOHYDRATE BI	NDING F	AMILY	6
42:00:00	4ary-D	4.6	4	99	577	8	MOLECULE:	PESTICIDAL CRYS	TAL PRO	TEIN	CRY1AC
43:00:00	1w99-A	4.6	4.4	100	558	10	MOLECULE:	PESTICIDIAL CRY	STAL PR	OTEIN	CRY4BA
44:00:00	5zi1-A	4.5	3.5	114	590	7	MOLECULE:	INSECTICIDAL CR	YSTAL P	ROTEI	N CRY7CAI
45:00:00	5la2-B	4.5	4.5	97	465	12	MOLECULE:	CARBOHYDRATE BI	NDING F	AMILY	6
46:00:00	6mlu-A	4.5	4.3	93	153	8	MOLECULE:	SEIPIN			
47:00:00	3iii-A	4.4	3.3	110	556	10	MOLECULE:	COCE/NOND FAMIL	Y HYDRO	LASE	
48:00:00	1stm-A	4.4	3.6	100	141	5	MOLECULE:	SATELLITE PANIC	UM MOSA	IC VI	RUS
49:00:00	5zi1-B	4.4	3.5	114	590	7	MOLECULE:	INSECTICIDAL CR	YSTAL P	ROTEI	N CRY7CAI
50:00:00	5hlz-G	4.4	4.4	98	204	9	MOLECULE:	INHIBIN BETA A	CHAIN		
51:00:00	3wnm-A	4.4	3.4	92	701	9	MOLECULE:	CYCLOISOMALTOOL	IGOSACC	HARID	E GLUCAN
52:00:00	8w7n-A	4.4	4.8	98	1011	10	MOLECULE:	PESTICIDAL CRYS	TAL PRO	TEIN	CRY1AA
53:00:00	1pnf-A	4.3	3.7	97	314	8	MOLECULE:	PEPTIDE-N(4)-(N	#NAME?	####	-D-GLUCOS
54:00:00	2y8k-A	4.3	4.4	101	480	9	MOLECULE:	CARBOHYDRATE BI	NDING F	AMILY	6
55:00:00	3wnl-A	4.3	3.4	91	701	8	MOLECULE:	CYCLOISOMALTOOL	IGOSACC	HARID	E GLUCAN
56:00:00	4jdm-C	4.2	3.5	96	269	7	MOLECULE:	VIRULENCE PLASM	ID PROT	EIN P	GP3-D
57:00:00	6i56-A	4.2	4.8	89	279	4	MOLECULE:	PHAGE-LIKE ELEM	ENT PBS	X PRO	TEIN XEPA
58:00:00	3wnk-A	4.2	3.1	88	712	8	MOLECULE:	CYCLOISOMALTOOL	IGOSACC	HARID	E GLUCAN
59:00:00	3wno-A	4.2	3.2	87	700	8	MOLECULE:	CYCLOISOMALTOOL	IGOSACC	HARID	E GLUCAN
60:00:00	5jsd-A	4.1	3.8	87	548	7	MOLECULE:	PHIAB6 TAILSPIK	E		
61:00:00	8erm-B	4.1	2.6	72	203	8	MOLECULE:	B-TYPE FLAGELLI	N		
62:00:00	8j3y-B	4.1	4	92	392	4	MOLECULE:	PUTATIVE POLYSA	CCHARID	E-BIN	DING PROT
63:00:00	7r1m-A	4	3.6	98	156	8	MOLECULE:	EXOSPORIUM PROT	EIN		
64:00:00	3qd6-D	4	3.8	95	135	9	MOLECULE:	CD40 LIGAND			
65:00:00	7vt4-A	4	4.5	83	438	5	MOLECULE:	ENDOGLUCANASE H			
66:00:00	5la0-A	4	4.6	96	476	9	MOLECULE:	CARBOHYDRATE BI	NDING F	AMILY	6
67:00:00	4mtm-A	3.9	4.4	81	137	10	MOLECULE:	PUTATIVE TAIL F	IBER PR	OTEIN	
68:00:00	8xcg-J	3.9	6.5	106	598	7	MOLECULE:	TAIL TIP PROTEI	N M		
69:00:00	1yq8-A	3.9	3.6	95	191	6	MOLECULE:	MINOR CAPSID PR	OTEIN		
70:00:00	5ak1-A	3.9	4.1	100	842	12	MOLECULE:	CARBOHYDRATE BI	NDING F	AMILY	6
71:00:00	3wnn-A	3.9	3.1	79	699	11	MOLECULE:	CYCLOISOMALTOOL	IGOSACC	HARID	E GLUCAN
72:00:00	4moa-A	3.9	4	99	602	10	MOLECULE:	PESTICIDAL CRYS	TAL PRO	TEIN	CRY4BA
73:00:00	7crc-B	3.8	3.3	91	1112	5	MOLECULE:	NAD+ HYDROLASE	(NADASE	)	
74:00:00	6s44-A	3.8	3.4	92	146	3	MOLECULE:	CAPSID PROTEIN			

75:00:00	3a21-A	3.8	3.3	89	614	6	MOLECULE:	PUTATIVE SECRET	ED ALPH	A-GAL	ACTOSIDAS	
76:00:00	3wno-B	3.8	3.1	78	699	10	MOLECULE:	CYCLOISOMALTOOL	IGOSACC	HARID	E GLUCAN(	
77:00:00	8yzo-A	3.7	7.3	101	506	12	MOLECULE:	LIPASE				
78:00:00	7pug-A	3.7	4.7	106	829	4	MOLECULE:	XYLAN ALPHA-1,2	#NAME?	ONID/	SE	
79:00:00	5zru-A	3.7	5.2	103	576	6	MOLECULE:	ALPHA-1,3-GLUCA	NASE			
80:00:00	6wpc-D	3.7	3.6	115	588	10	MOLECULE:	CRY1A.2				
81:00:00	5la2-A	3.7	4.4	100	464	10	MOLECULE:	CARBOHYDRATE BI	NDING F	AMILY		6
82:00:00	5hlz-E	3.7	4.5	105	204	7	MOLECULE:	INHIBIN BETA A	CHAIN			
83:00:00	6nff-A	3.6	7.3	102	794	10	MOLECULE:	XAA-PRO DIPEPTI	DYL-PEP	TIDAS	E	
84:00:00	2hey-F	3.6	4.1	96	135	11	MOLECULE:	TUMOR NECROSIS	FACTOR	LIGAN	D SUPERFA	
85:00:00	5e7t-H	3.6	4.5	97	173	7	MOLECULE:	MINOR STRUCTURA	L PROTE	IN 4		
86:00:00	7ufg-A	3.6	4.3	103	1151	6	MOLECULE:	PAPPALYSIN-1				
87:00:00	2wz8-A	3.6	3.5	87	135	10	MOLECULE:	CELLULOSOME PRO	TEIN DO	CKERI	N TYPE I	
88:00:00	3wnn-B	3.6	3.4	86	698	8	MOLECULE:	CYCLOISOMALTOOL	IGOSACC	HARID	E GLUCAN(	
89:00:00	3wqb-A	3.5	3.9	104	597	13	MOLECULE:	EXTRACELLULAR S	ERINE P	ROTE/	SE	
90:00:00	7a02-A	3.5	6	83	112	10	MOLECULE:	DUF3992 DOMAIN-	CONTAIN	ING P	ROTEIN	
91:00:00	3c7g-A	3.5	4.1	101	488	3	MOLECULE:	ENDO-1,4-BETA-X	YLANASE			
92:00:00	4ind-A	3.4	4.6	89	377	7	MOLECULE:	C381 TURRET PRO	TEIN			
93:00:00	4r4x-A	3.3	4.2	109	544	13	MOLECULE:	PNGF-II				
94:00:00	8gld-A	3.3	3.3	85	365	8	MOLECULE:	SGNH HYDROLASE-	TYPE ES	TERAS	E DOMAIN-	
95:00:00	2wys-B	3.2	3.8	91	516	7	MOLECULE:	ENDO-1,4-BETA-X	YLANASE	Y		
96:00:00	7kx0-A	3.2	4	93	140	5	MOLECULE:	CD70 ANTIGEN				
97:00:00	5la1-A	3.2	5.8	93	474	13	MOLECULE:	CARBOHYDRATE BI	NDING F	AMILY		6
98:00:00	4qpw-A	3.1	3.6	90	142	9	MOLECULE:	GLYCOSYL HYDROL	ASE FAM	ILY 1		0
99:00:00	7w63-B	3.1	4.2	94	395	10	MOLECULE:	TOXIN-COREGULAT	ED PILU	S BIO	SYNTHESIS	
0:00	1a6c-A	3.1	6.8	91	513	4	MOLECULE:	TOBACCO RINGSPO	T VIRUS	CAPS	ID PROTEIN	
1:00	6iwy-A	3	2.7	73	366	11	MOLECULE:	FLAGELLAR HOOK-	ASSOCIA	TED P	ROTEIN 2	
2:00	3b9t-A	3	5.5	118	419	5	MOLECULE:	TWIN-ARGININE T	RANSLOC	ATION	PATHWAY S	
3:00	6q5u-R	2.9	3.2	87	125	7	MOLECULE:	MAJOR CAPSID PR	OTEIN (	P3)		
4:00	5ubu-B	2.7	5.2	116	331	7	MOLECULE:	PUTATIVE ACETAM	IDASE/F	ORMA	IDASE	
5:00	8hqo-P	2.5	4	68	170	6	MOLECULE:	PORTAL PROTEIN				
6:00	6x80-C	2.5	16.8	80	574	14	MOLECULE:	FLAGELLIN A				

DALI results: MnA-1 Macc

No:	Chain	Z	rmsd	lali	nres	%id	PDB Description			
1:00	5jdo-C	5.9	3	84	141	7	MOLECULE:	HAPTOGLOBI	N-HAEMOG	LOBIN RECEPTOR
2:00	7kyr-A	5.7	3.9	88	151	11	MOLECULE:	MYOGLOBIN		
3:00	3e5o-A	5.6	4.1	88	153	11	MOLECULE:	MYOGLOBIN		
4:00	6g5b-A	5.6	4	88	154	11	MOLECULE:	MYOGLOBIN		
5:00	7sph-A	5.6	4	91	154	12	MOLECULE:	MYOGLOBIN		
6:00	7a44-A	5.5	4	91	154	12	MOLECULE:	MYOGLOBIN		
7:00	1jw8-A	5.4	4.1	90	154	11	MOLECULE:	MYOGLOBIN		
8:00	6e03-A	5.4	4.1	88	153	11	MOLECULE:	MYOGLOBIN		
9:00	4tyx-A	5.4	4	88	153	11	MOLECULE:	MYOGLOBIN		
10:00	5m3s-A	5.4	4.2	90	154	11	MOLECULE:	MYOGLOBIN		
11:00	1ch9-A	5.4	4.1	90	154	12	MOLECULE:	PROTEIN (MYOGLOBIN)		
12:00	5b84-A	5.4	4.1	88	153	11	MOLECULE:	MYOGLOBIN		
13:00	7spe-A	5.3	4.1	90	154	11	MOLECULE:	MYOGLOBIN		
14:00	2r4w-A	5.3	3.3	84	145	8	MOLECULE:	GLOBIN-1		
15:00	2blh-A	5.3	4	90	153	12	MOLECULE:	MYOGLOBIN		
16:00	7l3u-A	5.3	4.1	91	153	11	MOLECULE:	MYOGLOBIN		
17:00	8eko-A	5.3	4.1	88	153	11	MOLECULE:	MYOGLOBIN		
18:00	2ri4-C	5.3	3	83	138	7	MOLECULE:	HEMOGLOBIN	SUBUNIT	ALPHA-1/2
19:00	6az6-A	5.2	8.3	105	209	8	MOLECULE:	GNTR FAMIL	Y TRANSC	RIPTIONAL REGULATOR
20:00	1myt-A	5.2	4.1	85	145	9	MOLECULE:	MYOGLOBIN		
21:00	5zzf-A	5.2	4.2	88	153	11	MOLECULE:	MYOGLOBIN		
22:00	7xcq-A	5.2	4.1	88	153	11	MOLECULE:	MYOGLOBIN		
23:00	7xc9-A	5.2	4.1	88	153	11	MOLECULE:	MYOGLOBIN		
24:00:00	1dtm-A	5.2	4.1	91	152	11	MOLECULE:	RECOMBINAN	T SPERM	WHALE MYOGLOBIN VARIANT H93G
25:00:00	8j4k-A	5.2	4.1	88	153	11	MOLECULE:	MYOGLOBIN		
26:00:00	5yzf-A	5.2	4	89	154	12	MOLECULE:	SPERM WHAL	E MYOGLO	BIN
27:00:00	7xzi-B	5.1	4.4	88	166	2	MOLECULE:	CTAP3		
28:00:00	5hav-A	5.1	4.1	90	153	9	MOLECULE:	MYOGLOBIN		
29:00:00	6krc-A	5.1	4.1	90	153	9	MOLECULE:	MYOGLOBIN		
30:00:00	7l3y-A	5.1	4	88	153	11	MOLECULE:	MYOGLOBIN		
31:00:00	3qm9-A	5.1	4	84	144	7	MOLECULE:	MYOGLOBIN		
32:00:00	3a59-B	5.1	3.5	85	146	9	MOLECULE:	HEMOGLOBIN	SUBUNIT	ALPHA-A
33:00:00	5yce-A	5.1	4.1	88	151	11	MOLECULE:	MYOGLOBIN		
34:00:00	5yci-B	5	4.1	93	157	16	MOLECULE:	ANCESTRAL	MYOGLOBI	N AMBWB' OF BASILOSAURUS RELATI
35:00:00	7spf-A	5	4.1	92	152	11	MOLECULE:	MYOGLOBIN		
36:00:00	6ls8-I	5	4	91	153	13	MOLECULE:	MYOGLOBIN		
37:00:00	5zzg-A	5	4.1	88	153	11	MOLECULE:	MYOGLOBIN		
38:00:00	2nrm-A	5	4	85	146	7	MOLECULE:	MYOGLOBIN		
39:00:00	8oup-D	5	4.1	91	151	5	MOLECULE:	NERVE HEMO	GLOBIN	
40:00:00	1v75-A	5	3.9	89	141	8	MOLECULE:	HEMOGLOBIN	D ALPHA	CHAIN
41:00:00	3w4u-A	5	3.6	86	141	3	MOLECULE:	HEMOGLOBIN	SUBUNIT	ZETA
42:00:00	3sxy-B	5	4.4	105	201	6	MOLECULE:	TRANSCRIPT	IONAL RE	GULATOR, GNTR FAMILY
43:00:00	8oup-B	5	3.9	91	151	5	MOLECULE:	NERVE HEMO	GLOBIN	
44:00:00	1wmu-A	5	3.9	88	141	8	MOLECULE:	HEMOGLOBIN	D ALPHA	CHAIN
45:00:00	6az6-B	5	7.6	105	205	6	MOLECULE:	GNTR FAMIL	Y TRANSC	RIPTIONAL REGULATOR
46:00:00	3w4u-E	5	3.1	85	138	6	MOLECULE:	HEMOGLOBIN	SUBUNIT	ZETA
47:00:00	3ecx-A	5	4.1	88	153	11	MOLECULE:	MYOGLOBIN		
48:00:00	7spg-A	5	4.2	92	152	11	MOLECULE:	MYOGLOBIN		
49:00:00	6zca-H	4.9	10.3	123	771	7	MOLECULE:	PROBABLE D	NA-DIREC	TED RNA POLYMERASE SUBUNIT DELT
50:00:00	3pt7-B	4.9	4.1	87	152	6	MOLECULE:	HEMOGLOBIN	II	
51:00:00	2bnl-C	4.9	3.7	83	134	10	MOLECULE:	MODULATOR	PROTEIN	RSBR
52:00:00	3sxk-A	4.9	4.4	100	140	10	MOLECULE:	TRANSCRIPT	IONAL RE	GULATOR, GNTR FAMILY

53:00:00	6bmg-A	4.9	4.1	90	154	14	MOLECULE:	MYOGLOBIN		
54:00:00	6ls8-G	4.9	4.1	89	153	17	MOLECULE:	MYOGLOBIN		
55:00:00	6f1a-A	4.9	4.2	90	154	9	MOLECULE:	MYOGLOBIN		
56:00:00	1wvp-A	4.9	4.1	89	151	12	MOLECULE:	MYOGLOBIN		
57:00:00	3qm5-A	4.9	4	84	145	7	MOLECULE:	MYOGLOBIN		
58:00:00	3w4u-C	4.9	3.6	86	141	3	MOLECULE:	HEMOGLOBIN	SUBUNIT	ZETA
59:00:00	2w6x-A	4.9	4.2	90	154	13	MOLECULE:	MYOGLOBIN		
60:00:00	3s1j-A	4.9	3.6	85	131	9	MOLECULE:	HEMOGLOBIN	#NAME?	AVOPROTEIN
61:00:00	3s1i-A	4.9	3.6	85	131	9	MOLECULE:	HEMOGLOBIN	#NAME?	AVOPROTEIN
62:00:00	6oty-B	4.9	4.2	88	152	6	MOLECULE:	HEMOGLOBIN	II	
63:00:00	3s1i-C	4.9	3.7	84	131	8	MOLECULE:	HEMOGLOBIN	#NAME?	AVOPROTEIN
64:00:00	3ubc-D	4.9	3.7	85	131	9	MOLECULE:	HEMOGLOBIN	#NAME?	AVOPROTEIN
65:00:00	8oup-C	4.9	4.1	91	151	5	MOLECULE:	NERVE HEMO	GLOBIN	
66:00:00	8oup-A	4.9	4	90	150	6	MOLECULE:	NERVE HEMO	GLOBIN	
67:00:00	5ycj-B	4.9	4.2	94	157	15	MOLECULE:	ANCESTRAL	MYOGLOBI	N AMBWB' OF BASILOSAURUS RELATI
68:00:00	2z6n-A	4.9	3.9	88	141	8	MOLECULE:	HEMOGLOBIN	D SUBUN	IT ALPHA
69:00:00	6ls8-C	4.9	4.2	90	153	17	MOLECULE:	MYOGLOBIN		
70:00:00	3ogb-A	4.9	4	91	154	12	MOLECULE:	MYOGLOBIN		
71:00:00	3sxm-A	4.9	4.1	96	137	8	MOLECULE:	TRANSCRIPT	IONAL RE	GULATOR, GNTR FAMILY
72:00:00	1nz3-A	4.9	4.2	89	153	17	MOLECULE:	MYOGLOBIN		
73:00:00	1jeb-A	4.9	3.4	86	141	6	MOLECULE:	HEMOGLOBIN	ZETA CH	AIN
74:00:00	4nxa-A	4.9	4	89	153	12	MOLECULE:	MYOGLOBIN		
75:00:00	1ebc-A	4.9	4.1	90	153	12	MOLECULE:	PROTEIN (M	YOGLOBIN	)
76:00:00	5m3r-A	4.9	4	91	154	12	MOLECULE:	MYOGLOBIN		
77:00:00	1l2k-A	4.9	4.1	88	151	11	MOLECULE:	MYOGLOBIN		
78:00:00	3rgk-A	4.9	3.4	85	149	15	MOLECULE:	MYOGLOBIN		
79:00:00	1lhs-A	4.9	3.9	89	153	9	MOLECULE:	MYOGLOBIN		
80:00:00	4h07-A	4.8	4.2	90	154	12	MOLECULE:	MYOGLOBIN		
81:00:00	1emy-A	4.8	4	89	153	11	MOLECULE:	MYOGLOBIN		
82:00:00	2frk-X	4.8	4.2	91	152	15	MOLECULE:	MYOGLOBIN		
83:00:00	6n03-A	4.8	4	91	154	12	MOLECULE:	MYOGLOBIN,	CO-BOUN	D
84:00:00	6ls8-A	4.8	4.1	91	153	13	MOLECULE:	MYOGLOBIN		
85:00:00	2w6y-A	4.8	4.1	88	153	11	MOLECULE:	MYOGLOBIN		
86:00:00	8f9i-B	4.8	4.1	91	154	12	MOLECULE:	MYOGLOBIN		
87:00:00	6ls8-E	4.8	4.2	91	153	15	MOLECULE:	MYOGLOBIN		
88:00:00	1n9f-A	4.8	4.3	90	154	12	MOLECULE:	MYOGLOBIN		
89:00:00	5xl0-A	4.8	4.1	88	151	11	MOLECULE:	MYOGLOBIN		
90:00:00	2zsz-A	4.8	4.1	88	153	11	MOLECULE:	MYOGLOBIN		
91:00:00	3rj6-B	4.8	4.1	90	152	13	MOLECULE:	MYOGLOBIN		
92:00:00	1hbr-C	4.8	3.1	86	140	5	MOLECULE:	PROTEIN (H	EMOGLOBI	N D)
93:00:00	4rol-C	4.8	2.9	84	141	7	MOLECULE:	HEMOGLOBIN	SUBUNIT	ALPHA
94:00:00	3qm6-A	4.8	4	83	145	6	MOLECULE:	MYOGLOBIN		
95:00:00	1mlq-A	4.8	4.2	90	154	11	MOLECULE:	MYOGLOBIN		
96:00:00	2r1h-C	4.8	3.3	85	142	8	MOLECULE:	HEMOGLOBIN	SUBUNIT	ALPHA-4
97:00:00	8wf5-A	4.8	4.1	90	152	13	MOLECULE:	MYOGLOBIN		
98:00:00	4pqb-A	4.8	4.2	89	153	13	MOLECULE:	MYOGLOBIN		
99:00:00	3rj6-A	4.8	4.2	90	152	16	MOLECULE:	MYOGLOBIN		
0:00	3hxn-C	4.8	3	84	141	7	MOLECULE:	HEMOGLOBIN	SUBUNIT	ALPHA
1:00	8ess-A	4.8	4	89	154	11	MOLECULE:	MYOGLOBIN		
2:00	5ilp-A	4.8	4.1	88	151	11	MOLECULE:	MYOGLOBIN		
3:00	2ekt-A	4.8	4.2	90	153	13	MOLECULE:	MYOGLOBIN		
4:00	1m6m-A	4.8	4	90	153	13	MOLECULE:	PROTEIN (M	YOGLOBIN	)
5:00	5iks-A	4.8	4.1	88	151	11	MOLECULE:	MYOGLOBIN		
6:00	1n9x-A	4.8	4.1	90	154	11	MOLECULE:	MYOGLOBIN		

7:00	3pt8-B	4.8	4.1	87	152	6	MOLECULE:	HEMOGLOBIN	II	
8:00	7sli-A	4.8	4.2	89	152	11	MOLECULE:	MYOGLOBIN		
9:00	1n9h-A	4.8	4.3	90	154	12	MOLECULE:	MYOGLOBIN		
10:00	1fcs-A	4.8	4.1	90	154	11	MOLECULE:	MYOGLOBIN		
11:00	1iop-A	4.8	4.1	91	153	11	MOLECULE:	MYOGLOBIN		
12:00	112m-A	4.8	4	90	154	11	MOLECULE:	MYOGLOBIN		
13:00	3ase-A	4.8	4.2	90	154	11	MOLECULE:	MYOGLOBIN		
14:00	3bom-A	4.8	2.8	83	142	8	MOLECULE:	HEMOGLOBIN	SUBUNIT	ALPHA-4
15:00	1mlk-A	4.8	4	90	154	11	MOLECULE:	MYOGLOBIN		
16:00	3m3b-A	4.8	4	91	153	11	MOLECULE:	MYOGLOBIN		
17:00	3a2g-A	4.8	4.1	90	154	11	MOLECULE:	MYOGLOBIN		
18:00	4twv-A	4.8	4.1	90	153	13	MOLECULE:	MYOGLOBIN		
19:00	1mob-A	4.8	4	90	154	11	MOLECULE:	MYOGLOBIN		
20:00	1mtk-A	4.8	4	90	154	11	MOLECULE:	MYOGLOBIN		
21:00	4rol-A	4.8	3	84	141	7	MOLECULE:	HEMOGLOBIN	SUBUNIT	ALPHA
22:00	1mlr-A	4.8	4.2	90	154	11	MOLECULE:	MYOGLOBIN		
23:00	1yog-A	4.8	4.1	89	153	12	MOLECULE:	MYOGLOBIN		
24:00:00	2nx0-A	4.8	3.9	84	146	7	MOLECULE:	MYOGLOBIN		
25:00:00	2spl-A	4.8	4.1	90	154	11	MOLECULE:	MYOGLOBIN		
26:00:00	3s1j-C	4.8	3.7	84	131	8	MOLECULE:	HEMOGLOBIN	#NAME?	AVOPROTEIN
27:00:00	7vdn-A	4.8	4.1	88	151	11	MOLECULE:	MYOGLOBIN		
28:00:00	3h57-A	4.8	4.1	90	154	11	MOLECULE:	MYOGLOBIN		
29:00:00	5ile-A	4.8	4.2	90	154	11	MOLECULE:	MYOGLOBIN		
30:00:00	1j3f-A	4.8	3.6	88	152	13	MOLECULE:	MYOGLOBIN		
31:00:00	5kkk-A	4.8	4.2	90	154	12	MOLECULE:	MYOGLOBIN		
32:00:00	2mb5-A	4.8	4.1	89	153	12	MOLECULE:	MYOGLOBIN		
33:00:00	1spe-A	4.8	4.2	90	153	12	MOLECULE:	MYOGLOBIN		
34:00:00	6otw-B	4.8	4.1	87	152	6	MOLECULE:	HEMOGLOBIN	II	
35:00:00	1h1x-A	4.8	4.2	90	154	12	MOLECULE:	MYOGLOBIN		
36:00:00	1hbr-A	4.8	3.6	87	141	3	MOLECULE:	PROTEIN (H	EMOGLOBI	N D)
37:00:00	1xzu-C	4.8	3	84	141	7	MOLECULE:	HEMOGLOBIN	ALPHA C	HAIN
38:00:00	2mga-A	4.8	4.1	90	154	11	MOLECULE:	MYOGLOBIN		
39:00:00	3bom-C	4.8	3.3	85	142	8	MOLECULE:	HEMOGLOBIN	SUBUNIT	ALPHA-4
40:00:00	2r1h-A	4.8	3.3	85	142	8	MOLECULE:	HEMOGLOBIN	SUBUNIT	ALPHA-4
41:00:00	3ubc-G	4.8	3.8	84	131	8	MOLECULE:	HEMOGLOBIN	#NAME?	AVOPROTEIN
42:00:00	3h58-A	4.8	4.1	90	154	11	MOLECULE:	MYOGLOBIN		
43:00:00	8esu-A	4.8	4.2	90	154	11	MOLECULE:	MYOGLOBIN		
44:00:00	1abs-A	4.8	4.1	90	154	11	MOLECULE:	MYOGLOBIN		
45:00:00	3sxz-A	4.8	3.9	97	139	7	MOLECULE:	TRANSCRIPT	IONAL RE	GULATOR, GNTR FAMILY
46:00:00	2oha-A	4.8	4.1	90	154	11	MOLECULE:	MYOGLOBIN		
47:00:00	6m8f-A	4.8	4.2	90	154	11	MOLECULE:	MYOGLOBIN		
48:00:00	1n9i-A	4.8	4.2	90	154	11	MOLECULE:	MYOGLOBIN		
49:00:00	2o5m-X	4.8	4.2	91	152	13	MOLECULE:	MYOGLOBIN		
50:00:00	1jeb-C	4.8	3.4	86	141	6	MOLECULE:	HEMOGLOBIN	ZETA CH	AIN
51:00:00	2v1j-A	4.8	4.1	90	153	13	MOLECULE:	MYOGLOBIN		
52:00:00	2o5t-X	4.8	4.1	90	152	13	MOLECULE:	MYOGLOBIN		
53:00:00	2o5o-X	4.8	4.2	91	152	15	MOLECULE:	MYOGLOBIN		
54:00:00	4twu-A	4.8	4.1	88	153	16	MOLECULE:	MYOGLOBIN		
55:00:00	6mv0-A	4.8	4	88	154	11	MOLECULE:	MYOGLOBIN		
56:00:00	2o58-X	4.8	4.2	91	152	13	MOLECULE:	MYOGLOBIN		
57:00:00	5vzq-A	4.8	4.1	91	153	11	MOLECULE:	MYOGLOBIN		
58:00:00	2o5q-X	4.8	4.2	91	152	15	MOLECULE:	MYOGLOBIN		
59:00:00	1ufp-A	4.8	4.1	88	154	11	MOLECULE:	MYOGLOBIN		
60:00:00	1bzp-A	4.8	4.1	89	153	12	MOLECULE:	PROTEIN (M	YOGLOBIN	)

61:00:00	2oh9-A	4.8	4.1	90	154	11	MOLECULE:	MYOGLOBIN		
62:00:00	1c7c-A	4.8	9.4	114	283	7	MOLECULE:	PROTEIN (D	EOXYHEMO	GLOBIN (ALPHA CHAIN))
63:00:00	2mgj-A	4.8	4.2	90	154	11	MOLECULE:	MYOGLOBIN		
64:00:00	1cpw-A	4.8	4.2	90	154	11	MOLECULE:	PROTEIN (M	YOGLOBIN	)
65:00:00	1vxc-A	4.8	4.1	89	153	12	MOLECULE:	MYOGLOBIN		
66:00:00	1mlj-A	4.8	4.1	90	154	11	MOLECULE:	MYOGLOBIN		
67:00:00	1cp0-A	4.8	4.2	90	154	11	MOLECULE:	PROTEIN (M	YOGLOBIN	)
68:00:00	1moc-A	4.8	4.2	90	154	11	MOLECULE:	MYOGLOBIN		
69:00:00	1ofj-A	4.8	4.1	90	154	11	MOLECULE:	MYOGLOBIN		
70:00:00	104m-A	4.8	4.1	89	153	12	MOLECULE:	MYOGLOBIN		
71:00:00	103m-A	4.8	4.2	91	154	12	MOLECULE:	MYOGLOBIN		
72:00:00	2mya-A	4.8	4.1	89	153	12	MOLECULE:	MYOGLOBIN	(ETHYL I	SOCYANIDE)
73:00:00	2spo-A	4.8	4.1	90	154	11	MOLECULE:	MYOGLOBIN		
74:00:00	1dti-A	4.8	4.1	91	154	12	MOLECULE:	PROTEIN (M	YOGLOBIN	)
75:00:00	2oh8-A	4.8	4.1	90	154	11	MOLECULE:	MYOGLOBIN		
76:00:00	1vxe-A	4.8	4.1	89	153	12	MOLECULE:	MYOGLOBIN		
77:00:00	1co9-A	4.8	4.2	90	154	11	MOLECULE:	PROTEIN (M	YOGLOBIN	)
78:00:00	1cio-A	4.8	4.1	90	154	11	MOLECULE:	PROTEIN (M	YOGLOBIN	)
79:00:00	5zze-A	4.8	4.1	90	152	13	MOLECULE:	MYOGLOBIN		
80:00:00	1mlm-A	4.8	4.3	90	154	11	MOLECULE:	MYOGLOBIN		
81:00:00	1bz6-A	4.8	4.1	89	153	12	MOLECULE:	PROTEIN (M	YOGLOBIN	)
82:00:00	4it8-A	4.8	4.1	88	153	11	MOLECULE:	MYOGLOBIN		
83:00:00	1j52-A	4.8	4.2	90	154	11	MOLECULE:	MYOGLOBIN		
84:00:00	1ch1-A	4.8	4.2	90	154	11	MOLECULE:	PROTEIN (M	YOGLOBIN	)
85:00:00	1mln-A	4.8	4.3	90	154	11	MOLECULE:	MYOGLOBIN		
86:00:00	1o16-A	4.8	4.2	90	154	11	MOLECULE:	MYOGLOBIN		
87:00:00	1myz-A	4.8	4.1	90	154	11	MOLECULE:	MYOGLOBIN		
88:00:00	1vxd-A	4.8	4.1	88	153	11	MOLECULE:	MYOGLOBIN		
89:00:00	1o1l-A	4.8	10.2	115	283	7	MOLECULE:	HEMOGLOBIN	ALPHA C	HAIN
90:00:00	1mll-A	4.8	4.1	90	154	11	MOLECULE:	MYOGLOBIN		
91:00:00	4mbn-A	4.8	4.1	90	153	12	MOLECULE:	MYOGLOBIN		
92:00:00	1mz0-A	4.8	4.1	90	154	11	MOLECULE:	MYOGLOBIN		
93:00:00	102m-A	4.8	4.2	90	154	11	MOLECULE:	MYOGLOBIN		
94:00:00	1ltw-A	4.8	4.2	90	154	11	MOLECULE:	MYOGLOBIN		
95:00:00	1ch7-A	4.8	4.1	90	154	11	MOLECULE:	PROTEIN (M	YOGLOBIN	)
96:00:00	1tes-A	4.8	4.2	90	154	11	MOLECULE:	MYOGLOBIN		
97:00:00	1cik-A	4.8	4.1	90	154	11	MOLECULE:	PROTEIN (M	YOGLOBIN	)
98:00:00	106m-A	4.8	4.2	90	154	11	MOLECULE:	MYOGLOBIN		
99:00:00	2mge-A	4.8	4.1	90	154	11	MOLECULE:	MYOGLOBIN		
0:00	1mlu-A	4.8	4.2	90	154	11	MOLECULE:	MYOGLOBIN		
1:00	2mgm-A	4.8	4.1	90	154	11	MOLECULE:	MYOGLOBIN		
2:00	108m-A	4.8	4.2	90	154	11	MOLECULE:	MYOGLOBIN		
3:00	2spm-A	4.8	4.2	90	154	11	MOLECULE:	MYOGLOBIN		
4:00	1mlf-A	4.8	4.1	90	154	11	MOLECULE:	MYOGLOBIN		
5:00	1a6g-A	4.8	4.1	88	151	11	MOLECULE:	MYOGLOBIN		
6:00	1nz4-A	4.8	4.2	89	153	17	MOLECULE:	MYOGLOBIN		
7:00	6q6p-A	4.8	4.1	90	156	2	MOLECULE:	CYTOGLOBIN		
8:00	111m-A	4.8	4.2	90	154	11	MOLECULE:	MYOGLOBIN		
9:00	2mgb-A	4.8	4.2	90	154	11	MOLECULE:	MYOGLOBIN		
10:00	1mlo-A	4.8	4.3	90	154	11	MOLECULE:	MYOGLOBIN		
11:00	5cn4-A	4.8	4.1	89	152	15	MOLECULE:	MYOGLOBIN		
12:00	2mgk-A	4.8	4.1	90	154	11	MOLECULE:	MYOGLOBIN		
13:00	101m-A	4.8	4.2	90	154	11	MOLECULE:	MYOGLOBIN		
14:00	1cq2-A	4.8	4.1	89	153	12	MOLECULE:	MYOGLOBIN		

15:00	2v1g-A	4.8	4.1	90	153	13	MOLECULE:	MYOGLOBIN		
16:00	2ohb-A	4.8	4.2	90	154	11	MOLECULE:	MYOGLOBIN		
17:00	1o1p-A	4.8	10.6	115	283	8	MOLECULE:	HEMOGLOBIN	ALPHA C	HAIN
18:00	109m-A	4.8	4.2	90	154	11	MOLECULE:	MYOGLOBIN		
19:00	5mbn-A	4.8	4.1	90	153	12	MOLECULE:	MYOGLOBIN		
20:00	1mls-A	4.8	4.1	90	154	11	MOLECULE:	MYOGLOBIN		
21:00	1cp5-A	4.8	4.1	90	154	11	MOLECULE:	PROTEIN (M	YOGLOBIN	)
22:00	1o1j-A	4.8	9.5	112	283	8	MOLECULE:	HEMOGLOBIN	ALPHA C	HAIN
23:00	5cn5-A	4.8	4.1	90	152	13	MOLECULE:	MYOGLOBIN		
24:00:00	1yoi-A	4.8	4.1	89	153	12	MOLECULE:	MYOGLOBIN		
25:00:00	2mgc-A	4.8	4.1	90	154	11	MOLECULE:	MYOGLOBIN		
26:00:00	2mgl-A	4.8	4.2	90	154	11	MOLECULE:	MYOGLOBIN		
27:00:00	1ofk-A	4.8	4.1	90	154	11	MOLECULE:	MYOGLOBIN		
28:00:00	2mye-A	4.8	4.1	89	153	12	MOLECULE:	MYOGLOBIN	(ETHYL I	SOCYANIDE)
29:00:00	1lht-A	4.8	4	89	153	9	MOLECULE:	MYOGLOBIN		
30:00:00	2g0s-A	4.8	4.1	90	154	11	MOLECULE:	MYOGLOBIN		
31:00:00	1lue-A	4.8	4.2	90	154	11	MOLECULE:	MYOGLOBIN		
32:00:00	1vxh-A	4.8	4.1	89	153	12	MOLECULE:	MYOGLOBIN		
33:00:00	1ufj-A	4.8	4.1	88	154	11	MOLECULE:	MYOGLOBIN		
34:00:00	1f63-A	4.8	4.1	90	154	11	MOLECULE:	MYOGLOBIN		
35:00:00	1mym-A	4.8	4.2	90	154	11	MOLECULE:	MYOGLOBIN		
36:00:00	1swm-A	4.8	4.1	89	153	12	MOLECULE:	MYOGLOBIN		
37:00:00	1mbc-A	4.8	4.1	88	153	11	MOLECULE:	MYOGLOBIN		
38:00:00	2mgi-A	4.8	4.2	90	154	11	MOLECULE:	MYOGLOBIN		
39:00:00	2mgf-A	4.8	4.2	90	154	11	MOLECULE:	MYOGLOBIN		
40:00:00	6q6p-B	4.8	4.2	91	157	2	MOLECULE:	CYTOGLOBIN		
41:00:00	1obm-A	4.8	4.2	90	154	11	MOLECULE:	MYOGLOBIN		
42:00:00	6ird-B	4.7	6.5	90	254	4	MOLECULE:	1-PHOSPHAT	IDYLINOS	ITOL 4,5-BISPHOSPHATE PHOSPHODI
43:00:00	1abw-A	4.7	9.3	111	287	7	MOLECULE:	HEMOGLOBIN	#NAME?	LOOD SUBSTITUTE
44:00:00	1mwd-A	4.7	4	90	153	13	MOLECULE:	PROTEIN (M	YOGLOBIN	)
45:00:00	2v1i-A	4.7	4.1	90	153	13	MOLECULE:	MYOGLOBIN		
46:00:00	1myj-B	4.7	4.1	90	153	13	MOLECULE:	MYOGLOBIN		
47:00:00	1mnk-B	4.7	4.2	90	151	13	MOLECULE:	MYOGLOBIN		
48:00:00	5ilm-A	4.7	4.1	88	153	11	MOLECULE:	MYOGLOBIN		
49:00:00	2w6w-A	4.7	4.1	88	153	11	MOLECULE:	MYOGLOBIN		
50:00:00	2d6c-B	4.7	4	89	151	12	MOLECULE:	MYOGLOBIN		
51:00:00	8f9i-A	4.7	4.2	92	154	11	MOLECULE:	MYOGLOBIN		
52:00:00	1lfl-P	4.7	3	84	141	7	MOLECULE:	HEMOGLOBIN	ALPHA C	HAIN
53:00:00	1j3z-C	4.7	3	84	141	7	MOLECULE:	HEMOGLOBIN	ALPHA C	HAIN
54:00:00	2dn2-A	4.7	3	84	141	7	MOLECULE:	HEMOGLOBIN	ALPHA S	UBUNIT
55:00:00	1hgb-A	4.7	3	84	141	7	MOLECULE:	HEMOGLOBIN	(AQUO M	ET) (ALPHA CHAIN)
56:00:00	6e04-A	4.7	4.1	88	153	11	MOLECULE:	MYOGLOBIN		
57:00:00	6f18-A	4.7	4	88	154	11	MOLECULE:	MYOGLOBIN		
58:00:00	3szk-A	4.7	3.4	85	140	5	MOLECULE:	HEMOGLOBIN	SUBUNIT	ALPHA
59:00:00	1j41-G	4.7	3	84	141	7	MOLECULE:	HEMOGLOBIN	ALPHA C	HAIN
60:00:00	1dxv-C	4.7	3	84	141	7	MOLECULE:	HEMOGLOBIN	(DEOXY)	(ALPHA CHAIN)
61:00:00	3m39-A	4.7	4	88	153	11	MOLECULE:	MYOGLOBIN		
62:00:00	2dn2-C	4.7	3	84	141	7	MOLECULE:	HEMOGLOBIN	ALPHA S	UBUNIT
63:00:00	1a3o-C	4.7	3	84	141	7	MOLECULE:	HEMOGLOBIN	(ALPHA	CHAIN)
64:00:00	1do1-A	4.7	4.1	88	153	11	MOLECULE:	MYOGLOBIN		
65:00:00	1hjt-A	4.7	4.1	91	153	11	MOLECULE:	MYOGLOBIN		
66:00:00	6f17-A	4.7	4.1	89	153	11	MOLECULE:	MYOGLOBIN		
67:00:00	1y7g-C	4.7	3	84	141	7	MOLECULE:	HEMOGLOBIN	ALPHA C	HAIN
68:00:00	3qje-A	4.7	3	84	141	7	MOLECULE:	HEMOGLOBIN	SUBUNIT	ALPHA

69:00:00	1myg-B	4.7	4.1	90	153	13	MOLECULE:	MYOGLOBIN		
70:00:00	5ut7-A	4.7	4.1	90	154	11	MOLECULE:	MYOGLOBIN		
71:00:00	1do7-A	4.7	4.1	87	153	11	MOLECULE:	MYOGLOBIN		
72:00:00	6nbd-A	4.7	3	84	140	7	MOLECULE:	HEMOGLOBIN	SUBUNIT	ALPHA
73:00:00	2d5z-A	4.7	3	84	141	7	MOLECULE:	HEMOGLOBIN	ALPHA S	UBUNIT
74:00:00	1yh9-C	4.7	3	84	141	7	MOLECULE:	HEMOGLOBIN	ALPHA C	HAIN
75:00:00	1y0t-C	4.7	3	84	141	7	MOLECULE:	HEMOGLOBIN	ALPHA C	HAIN
76:00:00	3ecz-A	4.7	4.1	88	153	11	MOLECULE:	MYOGLOBIN		
77:00:00	5ut9-A	4.7	4.2	88	153	13	MOLECULE:	MYOGLOBIN		
78:00:00	8f9j-A	4.7	4.2	88	153	11	MOLECULE:	MYOGLOBIN		
79:00:00	3at5-A	4.7	2.9	84	141	8	MOLECULE:	ALPHAA-GLO	BIN	
80:00:00	1a01-A	4.7	3	84	141	7	MOLECULE:	HEMOGLOBIN	(ALPHA	CHAIN)
81:00:00	3d17-C	4.7	3.5	87	141	6	MOLECULE:	HEMOGLOBIN	SUBUNIT	ALPHA
82:00:00	3s1j-B	4.7	3.7	85	131	9	MOLECULE:	HEMOGLOBIN	#NAME?	AVOPROTEIN
83:00:00	1c7b-C	4.7	3	84	141	7	MOLECULE:	PROTEIN (D	EOXYHEMO	GLOBIN (ALPHA CHAIN))
84:00:00	1ch5-A	4.7	4.1	90	154	12	MOLECULE:	PROTEIN (M	YOGLOBIN	)
85:00:00	1bz0-C	4.7	3	84	141	7	MOLECULE:	PROTEIN (H	EMOGLOBI	N ALPHA CHAIN)
86:00:00	1j3y-C	4.7	3	84	141	7	MOLECULE:	HEMOGLOBIN	ALPHA C	HAIN
87:00:00	1xz4-A	4.7	3	84	141	8	MOLECULE:	HEMOGLOBIN	ALPHA C	HAIN
88:00:00	1a00-A	4.7	3	84	141	7	MOLECULE:	HEMOGLOBIN	(ALPHA	CHAIN)
89:00:00	6kye-K	4.7	3.1	84	140	7	MOLECULE:	HEMOGLOBIN	SUBUNIT	ALPHA
90:00:00	5kdq-A	4.7	3	84	141	7	MOLECULE:	HEMOGLOBIN	SUBUNIT	ALPHA
91:00:00	1gli-A	4.7	3	84	141	7	MOLECULE:	DEOXYHEMOG	LOBIN	
92:00:00	1vwt-C	4.7	3	84	141	7	MOLECULE:	HEMOGLOBIN		
93:00:00	1a6n-A	4.7	4.1	88	151	11	MOLECULE:	MYOGLOBIN		
94:00:00	1hgc-C	4.7	3.1	85	141	6	MOLECULE:	HEMOGLOBIN	(OXY) (	ALPHA CHAIN)
95:00:00	2zso-A	4.7	4.1	88	153	11	MOLECULE:	MYOGLOBIN		
96:00:00	1j7y-A	4.7	3	84	141	7	MOLECULE:	HEMOGLOBIN		
97:00:00	1xz2-A	4.7	3	84	141	7	MOLECULE:	HEMOGLOBIN	ALPHA C	HAIN
98:00:00	3d7o-A	4.7	3.4	85	141	7	MOLECULE:	HEMOGLOBIN	SUBUNIT	ALPHA
99:00:00	1y0d-C	4.7	3	84	140	7	MOLECULE:	HEMOGLOBIN	ALPHA C	HAIN
0:00	1gbu-C	4.7	3	84	141	7	MOLECULE:	HEMOGLOBIN		
1:00	1y5k-C	4.7	3	84	141	7	MOLECULE:	HEMOGLOBIN	ALPHA C	HAIN
2:00	2zt2-A	4.7	4.1	88	153	11	MOLECULE:	MYOGLOBIN		
3:00	3dut-A	4.7	3	84	141	7	MOLECULE:	HEMOGLOBIN	SUBUNIT	ALPHA
4:00	1y5j-C	4.7	3	84	141	7	MOLECULE:	HEMOGLOBIN	ALPHA C	HAIN
5:00	1gzx-C	4.7	3	84	141	7	MOLECULE:	HEMOGLOBIN	SUBUNIT	ALPHA
6:00	2d60-C	4.7	3	84	141	7	MOLECULE:	HEMOGLOBIN	ALPHA S	UBUNIT
7:00	2hbd-A	4.7	2.9	83	141	7	MOLECULE:	HEMOGLOBIN	A (METH	YL ISOCYANIDE) (ALPHA CHAIN)
8:00	1yhr-A	4.7	3	84	141	7	MOLECULE:	HEMOGLOBIN	ALPHA C	HAIN
9:00	1coh-A	4.7	3	84	141	7	MOLECULE:	HEMOGLOBIN	(FERROU	S CARBONMONOXY) (ALPHA CHAIN)
10:00	3s1i-B	4.7	3.8	85	131	9	MOLECULE:	HEMOGLOBIN	#NAME?	AVOPROTEIN
11:00	1a0u-C	4.7	3	84	141	7	MOLECULE:	HEMOGLOBIN	(ALPHA	CHAIN)
12:00	1o1n-A	4.7	9.8	115	282	6	MOLECULE:	HEMOGLOBIN	ALPHA C	HAIN
13:00	2mgh-A	4.7	4.2	90	154	11	MOLECULE:	MYOGLOBIN		
14:00	2spn-A	4.7	4.1	89	154	11	MOLECULE:	MYOGLOBIN		
15:00	1y09-C	4.7	3	84	141	7	MOLECULE:	HEMOGLOBIN	ALPHA C	HAIN
16:00	1xz7-C	4.7	3	84	141	7	MOLECULE:	HEMOGLOBIN	ALPHA C	HAIN
17:00	6cf0-A	4.7	4.2	88	153	13	MOLECULE:	MYOGLOBIN		
18:00	1mwc-A	4.7	4.1	90	153	13	MOLECULE:	PROTEIN (M	YOGLOBIN	)
19:00	1dxv-A	4.7	3	84	141	7	MOLECULE:	HEMOGLOBIN	(DEOXY)	(ALPHA CHAIN)
20:00	3wft-A	4.7	4.1	90	153	13	MOLECULE:	MYOGLOBIN		
21:00	3ba2-A	4.7	4.2	88	152	14	MOLECULE:	MYOGLOBIN		
22:00	2hbc-A	4.7	3	84	141	7	MOLECULE:	HEMOGLOBIN	A (ETHY	L ISOCYANIDE) (ALPHA CHAIN)

23:00	8fdl-A	4.7	3	84	141	7	MOLECULE:	HEMOGLOBIN	SUBUNIT	ALPHA
24:00:00	1gbv-A	4.7	3	84	141	7	MOLECULE:	HEMOGLOBIN		
25:00:00	1xz2-C	4.7	3	84	141	7	MOLECULE:	HEMOGLOBIN	ALPHA C	HAIN
26:00:00	3mn0-A	4.7	3.9	88	153	11	MOLECULE:	MYOGLOBIN		
27:00:00	2hhe-C	4.7	3	84	141	7	MOLECULE:	HEMOGLOBIN	(DEOXY)	(ALPHA CHAIN)
28:00:00	1dxu-C	4.7	3	84	141	7	MOLECULE:	HEMOGLOBIN	(DEOXY)	(ALPHA CHAIN)
29:00:00	2hbs-C	4.7	3	84	141	7	MOLECULE:	HEMOGLOBIN	S (DEOX	Y), ALPHA CHAIN
30:00:00	1y4v-C	4.7	3	84	141	7	MOLECULE:	HEMOGLOBIN	ALPHA C	HAIN
31:00:00	4n7n-K	4.7	3	84	141	7	MOLECULE:	HEMOGLOBIN	SUBUNIT	ALPHA
32:00:00	2mgg-A	4.7	4.2	90	154	11	MOLECULE:	MYOGLOBIN		
33:00:00	8dov-G	4.7	3.4	85	140	7	MOLECULE:	HEMOGLOBIN	SUBUNIT	ALPHA
34:00:00	3ed9-A	4.7	4.1	88	153	11	MOLECULE:	MYOGLOBIN		
35:00:00	1dxt-A	4.7	3	84	141	7	MOLECULE:	HEMOGLOBIN	(DEOXY)	(ALPHA CHAIN)
36:00:00	1irc-A	4.7	4	89	152	12	MOLECULE:	MYOGLOBIN	(METAQUO	)
37:00:00	1sdk-A	4.7	3	84	141	7	MOLECULE:	HEMOGLOBIN	A	
38:00:00	1ajh-A	4.7	4.1	88	153	11	MOLECULE:	MYOGLOBIN		
39:00:00	3hhb-C	4.7	3	84	141	7	MOLECULE:	HEMOGLOBIN	(DEOXY)	(ALPHA CHAIN)
40:00:00	1y4r-C	4.7	3	84	141	7	MOLECULE:	HEMOGLOBIN	ALPHA C	HAIN
41:00:00	1hbb-C	4.7	3	84	141	7	MOLECULE:	HEMOGLOBIN	A (DEOX	Y) (ALPHA CHAIN)
42:00:00	3ecl-A	4.7	4.1	88	153	11	MOLECULE:	MYOGLOBIN		
43:00:00	1yhe-C	4.7	3	84	141	7	MOLECULE:	HEMOGLOBIN	ALPHA C	HAIN
44:00:00	2eku-A	4.7	4.1	88	153	11	MOLECULE:	MYOGLOBIN		
45:00:00	3fms-A	4.7	4.2	101	209	7	MOLECULE:	TRANSCRIPT	IONAL RE	GULATOR, GNTR FAMILY
46:00:00	1dxu-A	4.7	3	84	141	7	MOLECULE:	HEMOGLOBIN	(DEOXY)	(ALPHA CHAIN)
47:00:00	1qsh-A	4.7	3	84	141	7	MOLECULE:	PROTEIN (H	EMOGLOBI	N ALPHA CHAIN)
48:00:00	1mno-A	4.7	4	90	153	13	MOLECULE:	PROTEIN (M	YOGLOBIN	)
49:00:00	2zt3-A	4.7	4.1	88	153	11	MOLECULE:	MYOGLOBIN		
50:00:00	3eda-A	4.7	4.1	88	153	11	MOLECULE:	MYOGLOBIN		
51:00:00	8f9n-A	4.7	4.2	89	153	11	MOLECULE:	MYOGLOBIN		
52:00:00	1mnj-B	4.7	4.2	90	151	13	MOLECULE:	MYOGLOBIN		
53:00:00	3k9z-A	4.7	4	88	153	11	MOLECULE:	MYOGLOBIN		
54:00:00	4h0b-A	4.7	4	89	154	11	MOLECULE:	MYOGLOBIN		
55:00:00	1do4-A	4.7	4.1	88	153	11	MOLECULE:	MYOGLOBIN		
56:00:00	3m38-A	4.7	4.1	89	153	12	MOLECULE:	MYOGLOBIN		
57:00:00	6kae-G	4.7	3	84	141	7	MOLECULE:	HEMOGLOBIN	SUBUNIT	ALPHA
58:00:00	1dxd-A	4.7	4.1	88	153	11	MOLECULE:	MYOGLOBIN		
59:00:00	1j3y-G	4.7	3	84	141	7	MOLECULE:	HEMOGLOBIN	ALPHA C	HAIN
60:00:00	4m4a-A	4.7	3.4	85	140	7	MOLECULE:	HEMOGLOBIN	SUBUNIT	ALPHA
61:00:00	1r1y-A	4.7	3.3	85	141	7	MOLECULE:	HEMOGLOBIN	ALPHA C	HAIN
62:00:00	1vwt-A	4.7	3	84	141	7	MOLECULE:	HEMOGLOBIN		
63:00:00	1k1k-A	4.7	3	84	141	7	MOLECULE:	HEMOGLOBIN	ALPHA C	HAIN
64:00:00	6hit-A	4.7	3.3	86	142	5	MOLECULE:	HEMOGLOBIN	ALPHA 2	CHAIN
65:00:00	3e4n-A	4.7	4.1	88	153	11	MOLECULE:	MYOGLOBIN		
66:00:00	1yen-C	4.7	2.9	84	138	6	MOLECULE:	HEMOGLOBIN	ALPHA C	HAIN
67:00:00	1do3-A	4.7	4.2	88	153	13	MOLECULE:	MYOGLOBIN		
68:00:00	1ye2-C	4.7	3	84	141	7	MOLECULE:	HEMOGLOBIN	ALPHA C	HAIN
69:00:00	1y7z-C	4.7	3	84	141	7	MOLECULE:	HEMOGLOBIN	ALPHA C	HAIN
70:00:00	3e55-A	4.7	4.1	88	153	11	MOLECULE:	MYOGLOBIN		
71:00:00	3ubv-D	4.7	3.7	85	131	9	MOLECULE:	HEMOGLOBIN	#NAME?	AVOPROTEIN
72:00:00	4rom-A	4.7	3	84	141	7	MOLECULE:	HEMOGLOBIN	SUBUNIT	ALPHA
73:00:00	1qsh-C	4.7	3	84	141	7	MOLECULE:	PROTEIN (H	EMOGLOBI	N ALPHA CHAIN)
74:00:00	1duo-A	4.7	4.1	89	152	12	MOLECULE:	SPERM WHAL	E METAQU	OMYOGLOBIN VARIANT H93G
75:00:00	7ud7-A	4.7	3	84	141	7	MOLECULE:	HEMOGLOBIN	SUBUNIT	ALPHA
76:00:00	1xxt-C	4.7	3	84	141	7	MOLECULE:	HEMOGLOBIN	ALPHA C	HAIN

77:00:00	1y22-C	4.7	3	84	141	7	MOLECULE:	HEMOGLOBIN	ALPHA C	HAIN
78:00:00	6nq5-A	4.7	3.1	85	140	7	MOLECULE:	HEMOGLOBIN	SUBUNIT	ALPHA
79:00:00	1bzz-C	4.7	3	84	141	7	MOLECULE:	PROTEIN (H	EMOGLOBI	N ALPHA CHAIN)
80:00:00	5yci-A	4.7	4.2	92	154	15	MOLECULE:	ANCESTRAL	MYOGLOBI	N AMBWB' OF BASILOSAURUS RELATI
81:00:00	2zt0-A	4.7	4.1	88	153	11	MOLECULE:	MYOGLOBIN		
82:00:00	1ye0-A	4.7	3	84	141	7	MOLECULE:	HEMOGLOBIN	ALPHA C	HAIN
83:00:00	3sdn-A	4.7	4.1	89	152	12	MOLECULE:	MYOGLOBIN		
84:00:00	3oo5-A	4.7	3.3	85	140	7	MOLECULE:	HEMOGLOBIN	SUBUNIT	ALPHA
85:00:00	6g5a-A	4.7	4	89	153	12	MOLECULE:	MYOGLOBIN		
86:00:00	1y45-A	4.7	3	84	141	7	MOLECULE:	HEMOGLOBIN	ALPHA C	HAIN
87:00:00	1xy0-C	4.7	3	84	141	7	MOLECULE:	HEMOGLOBIN	ALPHA C	HAIN
88:00:00	1g9v-A	4.7	3	84	141	7	MOLECULE:	HEMOGLOBIN	ALPHA C	HAIN
89:00:00	1y0d-A	4.7	3	84	140	7	MOLECULE:	HEMOGLOBIN	ALPHA C	HAIN
90:00:00	4pqc-A	4.7	4	88	153	11	MOLECULE:	MYOGLOBIN		
91:00:00	1coh-C	4.7	3	84	141	7	MOLECULE:	HEMOGLOBIN	(FERROU	S CARBONMONOXY) (ALPHA CHAIN)
92:00:00	1pmb-B	4.7	4.1	89	153	15	MOLECULE:	MYOGLOBIN		
93:00:00	4lpi-A	4.7	4.1	88	153	11	MOLECULE:	MYOGLOBIN		
94:00:00	1vxa-A	4.7	4.1	89	153	12	MOLECULE:	MYOGLOBIN		
95:00:00	2ef2-A	4.7	3.6	87	153	11	MOLECULE:	MYOGLOBIN		
96:00:00	4m4b-A	4.7	3.4	85	140	7	MOLECULE:	HEMOGLOBIN	SUBUNIT	ALPHA
97:00:00	3p5q-A	4.7	3.4	85	139	7	MOLECULE:	HEMOGLOBIN	SUBUNIT	ALPHA
98:00:00	5c6y-A	4.7	4.1	88	153	11	MOLECULE:	MYOGLOBIN		
99:00:00	1y0c-C	4.7	3	84	141	7	MOLECULE:	HEMOGLOBIN	ALPHA C	HAIN
0:00	1yih-C	4.7	3	84	141	7	MOLECULE:	HEMOGLOBIN	ALPHA C	HAIN
1:00	1dxt-C	4.7	3	84	141	7	MOLECULE:	HEMOGLOBIN	(DEOXY)	(ALPHA CHAIN)
2:00	4n7o-I	4.7	3.1	85	141	7	MOLECULE:	HEMOGLOBIN	SUBUNIT	ALPHA
3:00	1bz0-A	4.7	3	84	141	7	MOLECULE:	PROTEIN (H	EMOGLOBI	N ALPHA CHAIN)
4:00	1bab-A	4.7	3	84	142	7	MOLECULE:	HEMOGLOBIN	THIONVI	LLE (DEOXY) (ALPHA CHAIN)
5:00	1ydz-C	4.7	3	84	141	7	MOLECULE:	HEMOGLOBIN	ALPHA C	HAIN
6:00	5vzp-A	4.7	4.2	89	153	11	MOLECULE:	MYOGLOBIN		
7:00	1a3n-C	4.7	3	84	141	7	MOLECULE:	HEMOGLOBIN	(ALPHA	CHAIN)
8:00	2d5z-C	4.7	3	84	141	7	MOLECULE:	HEMOGLOBIN	ALPHA S	UBUNIT
9:00	1o1o-C	4.7	3	84	141	7	MOLECULE:	HEMOGLOBIN	ALPHA C	HAIN
10:00	6otx-B	4.7	4.1	88	152	6	MOLECULE:	HEMOGLOBIN	II	
11:00	1a6m-A	4.7	4.1	88	151	11	MOLECULE:	MYOGLOBIN		
12:00	1rvw-A	4.7	3.3	85	141	7	MOLECULE:	HEMOGLOBIN		
13:00	1j3z-G	4.7	3	84	141	7	MOLECULE:	HEMOGLOBIN	ALPHA C	HAIN
14:00	1y83-C	4.7	3	84	141	7	MOLECULE:	HEMOGLOBIN	ALPHA C	HAIN
15:00	1bzz-A	4.7	3	84	141	7	MOLECULE:	PROTEIN (H	EMOGLOBI	N ALPHA CHAIN)
16:00	2hhd-A	4.7	3.3	85	141	7	MOLECULE:	HEMOGLOBIN	(DEOXY)	(ALPHA CHAIN)
17:00	1hsy-A	4.7	4.2	90	153	16	MOLECULE:	MYOGLOBIN		
18:00	1qsi-C	4.7	3	84	141	7	MOLECULE:	PROTEIN (H	EMOGLOBI	N ALPHA CHAIN)
19:00	1y4g-C	4.7	3	84	141	7	MOLECULE:	HEMOGLOBIN	ALPHA C	HAIN
20:00	1c7b-A	4.7	3	84	141	7	MOLECULE:	PROTEIN (D	EOXYHEMO	GLOBIN (ALPHA CHAIN))
21:00	1myj-A	4.7	4.1	89	153	15	MOLECULE:	MYOGLOBIN		
22:00	1xz7-A	4.7	3	84	141	7	MOLECULE:	HEMOGLOBIN	ALPHA C	HAIN
23:00	1y8w-C	4.7	3.1	85	141	6	MOLECULE:	HEMOGLOBIN	ALPHA C	HAIN
24:00:00	8fdm-C	4.7	3.4	85	139	7	MOLECULE:	HEMOGLOBIN	SUBUNIT	ALPHA
25:00:00	3szk-D	4.7	3.4	85	140	5	MOLECULE:	HEMOGLOBIN	SUBUNIT	ALPHA
26:00:00	2zsn-A	4.7	4.1	88	153	11	MOLECULE:	MYOGLOBIN		
27:00:00	1hdb-C	4.7	3	84	141	7	MOLECULE:	HEMOGLOBIN	(DEOXY)	BETA-V67T
28:00:00	1a0z-C	4.7	3	84	141	7	MOLECULE:	HEMOGLOBIN	(ALPHA	CHAIN)
29:00:00	3wfu-A	4.7	4.1	90	152	13	MOLECULE:	MYOGLOBIN		
30:00:00	1lfl-A	4.7	3.1	85	141	6	MOLECULE:	HEMOGLOBIN	ALPHA C	HAIN

31:00:00	2zt1-A	4.7	4.1	88	153	11	MOLECULE:	MYOGLOBIN		
32:00:00	6hbw-A	4.7	3	84	141	7	MOLECULE:	PROTEIN (H	EMOGLOBI	N ALPHA 1)
33:00:00	1yhr-C	4.7	3	85	141	6	MOLECULE:	HEMOGLOBIN	ALPHA C	HAIN
34:00:00	1a0z-A	4.7	3	84	141	7	MOLECULE:	HEMOGLOBIN	(ALPHA	CHAIN)
35:00:00	1y35-C	4.7	3	84	141	7	MOLECULE:	HEMOGLOBIN	ALPHA C	HAIN
36:00:00	5e6e-A	4.7	3.4	85	141	7	MOLECULE:	HEMOGLOBIN	SUBUNIT	ALPHA
37:00:00	1k0y-A	4.7	3	84	141	7	MOLECULE:	HEMOGLOBIN	ALPHA C	HAIN
38:00:00	3qjd-A	4.7	3	84	141	7	MOLECULE:	HEMOGLOBIN	SUBUNIT	ALPHA
39:00:00	1y0w-C	4.7	3	84	141	7	MOLECULE:	HEMOGLOBIN	ALPHA C	HAIN
40:00:00	2zsq-A	4.7	4.1	88	153	11	MOLECULE:	MYOGLOBIN		
41:00:00	3v2v-A	4.7	4.1	89	152	17	MOLECULE:	MYOGLOBIN		
42:00:00	1mbn-A	4.7	4.1	90	153	12	MOLECULE:	MYOGLOBIN		
43:00:00	3v2z-A	4.7	4.2	88	152	16	MOLECULE:	MYOGLOBIN		
44:00:00	7ddt-A	4.7	4.2	90	153	12	MOLECULE:	ANCESTRAL	MYOGLOBI	N AMBSE
45:00:00	1y4q-C	4.7	3	84	141	7	MOLECULE:	HEMOGLOBIN	ALPHA C	HAIN
46:00:00	3hhb-A	4.7	3	84	141	7	MOLECULE:	HEMOGLOBIN	(DEOXY)	(ALPHA CHAIN)
47:00:00	1g08-C	4.7	3.1	85	141	7	MOLECULE:	HEMOGLOBIN	ALPHA C	HAIN
48:00:00	5vzo-A	4.7	4.2	88	153	13	MOLECULE:	MYOGLOBIN		
49:00:00	4of9-A	4.7	4.1	88	153	11	MOLECULE:	MYOGLOBIN		
50:00:00	1y0a-C	4.7	3	84	141	7	MOLECULE:	HEMOGLOBIN	ALPHA C	HAIN
51:00:00	8fb0-A	4.7	4.1	88	153	11	MOLECULE:	MYOGLOBIN		
52:00:00	2zsr-A	4.7	4.1	88	153	11	MOLECULE:	MYOGLOBIN		
53:00:00	1k0y-C	4.7	3	84	141	7	MOLECULE:	HEMOGLOBIN	ALPHA C	HAIN
54:00:00	1qsi-A	4.7	3	84	141	7	MOLECULE:	PROTEIN (H	EMOGLOBI	N ALPHA CHAIN)
55:00:00	5ut8-A	4.7	4.2	89	153	11	MOLECULE:	MYOGLOBIN		
56:00:00	1bz1-C	4.7	3	84	142	7	MOLECULE:	PROTEIN (H	EMOGLOBI	N ALPHA CHAIN)
57:00:00	3onz-A	4.7	3.4	85	140	7	MOLECULE:	HEMOGLOBIN	SUBUNIT	ALPHA
58:00:00	7k4m-G	4.7	3	85	140	6	MOLECULE:	HEMOGLOBIN	SUBUNIT	ALPHA
59:00:00	1bz1-A	4.7	3	84	142	7	MOLECULE:	PROTEIN (H	EMOGLOBI	N ALPHA CHAIN)
60:00:00	5kd1-A	4.7	4.1	88	153	11	MOLECULE:	MYOGLOBIN		
61:00:00	1y7d-C	4.7	3	84	141	7	MOLECULE:	HEMOGLOBIN	ALPHA C	HAIN
62:00:00	1lfl-C	4.7	3.1	85	141	6	MOLECULE:	HEMOGLOBIN	ALPHA C	HAIN
63:00:00	1sdl-C	4.7	3	84	141	7	MOLECULE:	HEMOGLOBIN	A	
64:00:00	8fdm-A	4.7	3.4	85	139	7	MOLECULE:	HEMOGLOBIN	SUBUNIT	ALPHA
65:00:00	2z6t-A	4.7	4.1	88	151	11	MOLECULE:	MYOGLOBIN		
66:00:00	6ka9-C	4.7	3	84	141	7	MOLECULE:	HEMOGLOBIN	SUBUNIT	ALPHA
67:00:00	3edb-A	4.7	4.1	88	153	11	MOLECULE:	MYOGLOBIN		
68:00:00	1mod-A	4.7	4.2	90	154	11	MOLECULE:	MYOGLOBIN		
69:00:00	1yev-C	4.7	3	84	138	7	MOLECULE:	HEMOGLOBIN	ALPHA C	HAIN
70:00:00	1gli-C	4.7	3	84	141	7	MOLECULE:	DEOXYHEMOG	LOBIN	
71:00:00	2zss-A	4.7	4.1	88	153	11	MOLECULE:	MYOGLOBIN		
72:00:00	1y4p-C	4.7	3	84	141	7	MOLECULE:	HEMOGLOBIN	ALPHA C	HAIN
73:00:00	1rq3-C	4.7	3	84	141	7	MOLECULE:	HEMOGLOBIN	ALPHA C	HAIN
74:00:00	2zt4-A	4.7	4.1	88	153	11	MOLECULE:	MYOGLOBIN		
75:00:00	1o1o-A	4.7	3	84	141	7	MOLECULE:	HEMOGLOBIN	ALPHA C	HAIN
76:00:00	1ajg-A	4.7	4.1	88	153	11	MOLECULE:	MYOGLOBIN		
77:00:00	3m3a-A	4.7	4	89	153	12	MOLECULE:	MYOGLOBIN		
78:00:00	1xzv-C	4.7	3	84	141	7	MOLECULE:	HEMOGLOBIN	ALPHA C	HAIN
79:00:00	7slh-A	4.7	4.2	89	152	11	MOLECULE:	MYOGLOBIN		
80:00:00	1mnk-A	4.7	4.1	90	151	13	MOLECULE:	MYOGLOBIN		
81:00:00	1xz4-C	4.7	3.6	86	141	5	MOLECULE:	HEMOGLOBIN	ALPHA C	HAIN
82:00:00	5uta-A	4.7	4.2	88	153	13	MOLECULE:	MYOGLOBIN		
83:00:00	2eb9-A	4.7	3.6	86	150	12	MOLECULE:	MYOGLOBIN		
84:00:00	1y5f-C	4.7	3	84	141	7	MOLECULE:	HEMOGLOBIN	ALPHA C	HAIN

85:00:00	4l7y-A	4.7	3	84	141	7	MOLECULE:	HEMOGLOBIN	SUBUNIT	ALPHA
86:00:00	2jho-A	4.7	4.1	89	153	12	MOLECULE:	MYOGLOBIN		
87:00:00	6e02-A	4.7	4.2	89	153	12	MOLECULE:	MYOGLOBIN		
88:00:00	5ilr-A	4.7	4.1	88	151	11	MOLECULE:	MYOGLOBIN		
89:00:00	6zmx-A	4.7	3	85	138	6	MOLECULE:	HEMOGLOBIN	SUBUNIT	ALPHA-A
90:00:00	3e5i-A	4.7	4.1	88	153	11	MOLECULE:	MYOGLOBIN		
91:00:00	4fwx-A	4.7	4	88	153	11	MOLECULE:	MYOGLOBIN		
92:00:00	1gbv-C	4.7	3	84	141	7	MOLECULE:	HEMOGLOBIN		
93:00:00	2zsp-A	4.7	4.1	88	153	11	MOLECULE:	MYOGLOBIN		
94:00:00	5vrt-A	4.7	4.1	88	153	11	MOLECULE:	MYOGLOBIN		
95:00:00	1dke-C	4.7	2.9	84	141	6	MOLECULE:	HEMOGLOBIN	: ALPHA	CHAIN
96:00:00	2o5b-X	4.7	4.1	89	152	16	MOLECULE:	MYOGLOBIN		
97:00:00	5ych-A	4.7	4.1	90	153	13	MOLECULE:	ANCESTRAL	MYOGLOBI	N AMBWB OF BASILOSAURUS RELATIV
98:00:00	5yl3-A	4.7	4.1	90	151	13	MOLECULE:	MYOGLOBIN		
99:00:00	1nz2-A	4.7	4.2	90	153	16	MOLECULE:	MYOGLOBIN		
0:00	1mbd-A	4.7	4.2	90	153	12	MOLECULE:	MYOGLOBIN		
1:00	2yrs-I	4.7	3.3	85	141	7	MOLECULE:	HEMOGLOBIN	SUBUNIT	ALPHA
2:00	1mcy-A	4.7	4.1	89	154	11	MOLECULE:	MYOGLOBIN	(CARBONM	ONOXY)
3:00	5jnz-A	4.7	3.1	85	142	1	MOLECULE:	ALPHA CHAI	N	
4:00	1cls-A	4.7	3	84	141	7	MOLECULE:	HEMOGLOBIN		
5:00	4pq6-A	4.7	4	88	153	11	MOLECULE:	MYOGLOBIN		
6:00	6krf-A	4.7	4.1	88	153	11	MOLECULE:	MYOGLOBIN		
7:00	2hbs-G	4.7	3	84	141	7	MOLECULE:	HEMOGLOBIN	S (DEOX	Y), ALPHA CHAIN
8:00	1mtj-A	4.7	4.2	90	154	11	MOLECULE:	MYOGLOBIN		
9:00	2zsy-A	4.7	4.1	88	153	11	MOLECULE:	MYOGLOBIN		
10:00	8qbc-A	4.7	4.1	89	156	11	MOLECULE:	MYOGLOBIN		
11:00	4mxk-A	4.7	4	88	153	11	MOLECULE:	MYOGLOBIN		
12:00	3ubv-A	4.7	3.7	84	131	8	MOLECULE:	HEMOGLOBIN	#NAME?	AVOPROTEIN
13:00	2mgd-A	4.7	4.2	90	154	11	MOLECULE:	MYOGLOBIN		
14:00	1ch2-A	4.7	4.1	89	154	11	MOLECULE:	PROTEIN (M	YOGLOBIN	)
15:00	1nz5-A	4.7	4.2	90	153	16	MOLECULE:	MYOGLOBIN		
16:00	1myg-A	4.7	4.1	89	153	15	MOLECULE:	MYOGLOBIN		
17:00	107m-A	4.7	4.2	90	154	11	MOLECULE:	MYOGLOBIN		
18:00	4ns2-A	4.7	4	88	153	14	MOLECULE:	MYOGLOBIN		
19:00	1jpb-A	4.7	4.1	88	151	11	MOLECULE:	MYOGLOBIN		
20:00	2bwh-A	4.7	4	88	153	13	MOLECULE:	MYOGLOBIN		
21:00	6kai-G	4.7	3	84	141	7	MOLECULE:	HEMOGLOBIN	SUBUNIT	ALPHA
22:00	5wjk-A	4.7	4.2	89	153	11	MOLECULE:	MYOGLOBIN		
23:00	4qau-A	4.7	4.1	88	153	11	MOLECULE:	MYOGLOBIN		
24:00:00	8fdj-A	4.7	4.1	88	151	11	MOLECULE:	MYOGLOBIN		
25:00:00	3at6-A	4.7	2.9	84	141	8	MOLECULE:	ALPHAA-GLO	BIN	
26:00:00	7xcf-A	4.7	4.1	88	153	11	MOLECULE:	MYOGLOBIN		
27:00:00	3nmm-C	4.7	3	84	141	7	MOLECULE:	HEMOGLOBIN	SUBUNIT	ALPHA
28:00:00	3ubc-A	4.7	3.8	84	131	8	MOLECULE:	HEMOGLOBIN	#NAME?	AVOPROTEIN
29:00:00	6g5t-A	4.7	4.1	89	155	11	MOLECULE:	MYOGLOBIN		
30:00:00	5vnu-A	4.7	4	88	153	11	MOLECULE:	MYOGLOBIN		
31:00:00	1a01-C	4.7	3	84	141	7	MOLECULE:	HEMOGLOBIN	(ALPHA	CHAIN)
32:00:00	1y46-C	4.7	3	84	141	7	MOLECULE:	HEMOGLOBIN	ALPHA C	HAIN
33:00:00	6kah-G	4.7	3	84	141	7	MOLECULE:	HEMOGLOBIN	SUBUNIT	ALPHA
34:00:00	1ch3-A	4.7	4.1	89	154	11	MOLECULE:	PROTEIN (M	YOGLOBIN	)
35:00:00	2bli-A	4.7	4	89	153	11	MOLECULE:	MYOGLOBIN		
36:00:00	2zsx-A	4.7	4.1	88	153	11	MOLECULE:	MYOGLOBIN		
37:00:00	1moa-A	4.7	4.1	89	154	11	MOLECULE:	MYOGLOBIN		
38:00:00	1j7w-A	4.7	3	84	141	7	MOLECULE:	HEMOGLOBIN		

39:00:00	5xkw-A	4.7	4.1	89	153	11	MOLECULE:	MYOGLOBIN		
40:00:00	6iyi-A	4.7	3	84	142	2	MOLECULE:	ALPHA CHAI	N	
41:00:00	1xy0-A	4.7	3	84	141	7	MOLECULE:	HEMOGLOBIN	ALPHA C	HAIN
42:00:00	4mxl-A	4.7	4.1	89	153	12	MOLECULE:	MYOGLOBIN		
43:00:00	1r1y-C	4.7	3.3	85	141	7	MOLECULE:	HEMOGLOBIN	ALPHA C	HAIN
44:00:00	1c7d-A	4.7	11.4	111	284	9	MOLECULE:	PROTEIN (D	EOXYHEMO	GLOBIN (ALPHA CHAIN))
45:00:00	1hba-C	4.7	3	84	141	7	MOLECULE:	HEMOGLOBIN	ROTHSCH	ILD (DEOXY) (ALPHA CHAIN)
46:00:00	2d6c-A	4.7	4.2	88	153	11	MOLECULE:	MYOGLOBIN		
47:00:00	6n02-A	4.7	4	89	154	12	MOLECULE:	MYOGLOBIN,	CO-BOUN	D
48:00:00	1j40-G	4.7	3	84	141	7	MOLECULE:	HEMOGLOBIN	ALPHA C	HAIN
49:00:00	2hbs-E	4.7	3	84	141	7	MOLECULE:	HEMOGLOBIN	S (DEOX	Y), ALPHA CHAIN
50:00:00	1rse-A	4.7	4.2	90	153	16	MOLECULE:	HORSE HEAR	T MYOGLO	BIN
51:00:00	5oja-A	4.7	4.1	90	152	12	MOLECULE:	MYOGLOBIN		
52:00:00	4fwy-A	4.7	4.1	89	153	12	MOLECULE:	MYOGLOBIN		
53:00:00	2zst-A	4.7	4.1	88	153	11	MOLECULE:	MYOGLOBIN		
54:00:00	2nss-A	4.7	4.1	90	152	16	MOLECULE:	MYOGLOBIN		
55:00:00	2hbf-A	4.7	2.9	83	141	7	MOLECULE:	HEMOGLOBIN	A (N-PR	OPYL ISOCYANIDE) (ALPHA CHAIN)
56:00:00	7cen-A	4.7	4.1	88	153	11	MOLECULE:	MYOGLOBIN		
57:00:00	1bje-A	4.7	4.2	90	153	16	MOLECULE:	MYOGLOBIN		
58:00:00	1mbo-A	4.7	4.1	90	153	12	MOLECULE:	MYOGLOBIN		
59:00:00	1g9v-C	4.7	3	84	141	7	MOLECULE:	HEMOGLOBIN	ALPHA C	HAIN
60:00:00	1xye-C	4.7	3	84	141	7	MOLECULE:	HEMOGLOBIN	ALPHA C	HAIN
61:00:00	6bwp-A	4.7	3	84	141	7	MOLECULE:	HEMOGLOBIN	SUBUNIT	ALPHA
62:00:00	2myc-A	4.7	4.1	89	153	12	MOLECULE:	MYOGLOBIN	(N-BUTYL	ISOCYANIDE)
63:00:00	1d8u-A	4.6	4.8	95	165	9	MOLECULE:	NON-SYMBIO	TIC HEMO	GLOBIN
64:00:00	5ca9-A	4.6	20.1	110	656	6	MOLECULE:	PROTEIN SE	Y1	
65:00:00	1mwd-B	4.6	4.3	90	153	16	MOLECULE:	PROTEIN (M	YOGLOBIN	)
66:00:00	7pcf-A	4.6	3.6	87	141	6	MOLECULE:	HEMOGLOBIN	SUBUNIT	ALPHA
67:00:00	5ojb-A	4.6	3.6	89	154	12	MOLECULE:	MYOGLOBIN		
68:00:00	1mno-B	4.6	4.2	90	153	16	MOLECULE:	PROTEIN (M	YOGLOBIN	)
69:00:00	1ycb-B	4.6	4.2	89	153	15	MOLECULE:	MYOGLOBIN		
70:00:00	6lcw-C	4.6	3	84	141	7	MOLECULE:	HEMOGLOBIN	SUBUNIT	ALPHA
71:00:00	1spg-A	4.6	3.4	86	142	6	MOLECULE:	HEMOGLOBIN		
72:00:00	1kd2-C	4.6	3.1	85	141	7	MOLECULE:	HEMOGLOBIN	ALPHA C	HAIN
73:00:00	1g09-A	4.6	3.1	85	141	7	MOLECULE:	HEMOGLOBIN	ALPHA C	HAIN
74:00:00	4rom-C	4.6	3	84	141	7	MOLECULE:	HEMOGLOBIN	SUBUNIT	ALPHA
75:00:00	1hab-C	4.6	3.3	85	141	7	MOLECULE:	HEMOGLOBIN	A	
76:00:00	6bnr-A	4.6	2.9	83	141	7	MOLECULE:	HEMOGLOBIN	SUBUNIT	ALPHA
77:00:00	3sxz-B	4.6	3.8	94	140	7	MOLECULE:	TRANSCRIPT	IONAL RE	GULATOR, GNTR FAMILY
78:00:00	6kat-A	4.6	3.1	85	141	6	MOLECULE:	HEMOGLOBIN	SUBUNIT	ALPHA
79:00:00	1yeq-C	4.6	2.9	84	141	6	MOLECULE:	HEMOGLOBIN	ALPHA C	HAIN
80:00:00	1b86-C	4.6	3.1	85	141	6	MOLECULE:	PROTEIN (H	EMOGLOBI	N ALPHA CHAIN)
81:00:00	1yie-A	4.6	3.3	85	141	7	MOLECULE:	HEMOGLOBIN	ALPHA C	HAIN
82:00:00	1xxt-A	4.6	3.1	85	141	7	MOLECULE:	HEMOGLOBIN	ALPHA C	HAIN
83:00:00	3odq-C	4.6	3.4	86	141	6	MOLECULE:	HEMOGLOBIN	SUBUNIT	ALPHA
84:00:00	1ye0-C	4.6	2.9	83	141	7	MOLECULE:	HEMOGLOBIN	ALPHA C	HAIN
85:00:00	2qsp-C	4.6	3.1	84	141	7	MOLECULE:	HEMOGLOBIN	SUBUNIT	ALPHA
86:00:00	6kau-C	4.6	3	84	141	6	MOLECULE:	HEMOGLOBIN	SUBUNIT	ALPHA
87:00:00	5jdo-E	4.6	3.4	85	140	7	MOLECULE:	HAPTOGLOBI	N-HAEMOG	LOBIN RECEPTOR
88:00:00	6ka9-G	4.6	3	84	141	7	MOLECULE:	HEMOGLOBIN	SUBUNIT	ALPHA
89:00:00	1hda-C	4.6	3	84	141	7	MOLECULE:	HEMOGLOBIN	(DEOXY)	(ALPHA CHAIN)
90:00:00	1thb-A	4.6	3	84	141	7	MOLECULE:	HEMOGLOBIN	A (OXY)	(ALPHA CHAIN)
91:00:00	5ee4-E	4.6	3.5	85	141	5	MOLECULE:	HPUA		
92:00:00	2w72-C	4.6	2.8	83	141	7	MOLECULE:	HUMAN HEMO	GLOBIN A	

93:00:00	2hco-A	4.6	3	84	141	7	MOLECULE:	HEMOGLOBIN	(CARBON	MONOXY) (ALPHA CHAIN)
94:00:00	3oo4-A	4.6	3.4	85	141	7	MOLECULE:	HEMOGLOBIN	SUBUNIT	ALPHA
95:00:00	1j3y-E	4.6	3	84	141	7	MOLECULE:	HEMOGLOBIN	ALPHA C	HAIN
96:00:00	3d17-A	4.6	3.5	86	141	6	MOLECULE:	HEMOGLOBIN	SUBUNIT	ALPHA
97:00:00	4ni0-A	4.6	3.4	86	141	6	MOLECULE:	HEMOGLOBIN	SUBUNIT	ALPHA
98:00:00	7dy4-E	4.6	3.1	85	141	7	MOLECULE:	HEMOGLOBIN	SUBUNIT	ALPHA
99:00:00	3dut-C	4.6	3	84	141	7	MOLECULE:	HEMOGLOBIN	SUBUNIT	ALPHA
0:00	5zeo-A	4.6	4.1	89	153	11	MOLECULE:	MYOGLOBIN		
1:00	6l5y-C	4.6	3.1	85	141	6	MOLECULE:	HEMOGLOBIN	SUBUNIT	ALPHA
2:00	3wcp-C	4.6	3	84	141	7	MOLECULE:	HEMOGLOBIN	SUBUNIT	ALPHA
3:00	5kdq-C	4.6	3	85	141	7	MOLECULE:	HEMOGLOBIN	SUBUNIT	ALPHA
4:00	3wtg-A	4.6	3	85	138	9	MOLECULE:	HEMOGLOBIN	SUBUNIT	ALPHA-A
5:00	1yeo-A	4.6	3	84	141	7	MOLECULE:	HEMOGLOBIN	ALPHA C	HAIN
6:00	3qje-C	4.6	3	84	141	7	MOLECULE:	HEMOGLOBIN	SUBUNIT	ALPHA
7:00	1yeo-C	4.6	3	84	141	7	MOLECULE:	HEMOGLOBIN	ALPHA C	HAIN
8:00	6kao-A	4.6	3.4	85	141	7	MOLECULE:	HEMOGLOBIN	SUBUNIT	ALPHA
9:00	6l5y-A	4.6	3	84	141	6	MOLECULE:	HEMOGLOBIN	SUBUNIT	ALPHA
10:00	7dy4-A	4.6	3	84	141	7	MOLECULE:	HEMOGLOBIN	SUBUNIT	ALPHA
11:00	4n8t-A	4.6	3.4	85	140	7	MOLECULE:	HEMOGLOBIN	SUBUNIT	ALPHA
12:00	6di4-A	4.6	3.5	85	141	5	MOLECULE:	HEMOGLOBIN	SUBUNIT	ALPHA
13:00	1xzu-A	4.6	3.1	85	141	7	MOLECULE:	HEMOGLOBIN	ALPHA C	HAIN
14:00	3bj3-A	4.6	3.3	86	142	3	MOLECULE:	HEMOGLOBIN	ALPHA	
15:00	6ihx-A	4.6	3.1	84	140	7	MOLECULE:	HEMOGLOBIN	SUBUNIT	ALPHA
16:00	5hlx-A	4.6	4.2	88	153	11	MOLECULE:	MYOGLOBIN		
17:00	4b3w-B	4.6	4.2	89	154	9	MOLECULE:	CYTOGLOBIN		
18:00	1hgc-A	4.6	2.9	84	141	7	MOLECULE:	HEMOGLOBIN	(OXY) (	ALPHA CHAIN)
19:00	1hab-A	4.6	3	84	141	7	MOLECULE:	HEMOGLOBIN	A	
20:00	1qi8-C	4.6	3	84	141	7	MOLECULE:	HEMOGLOBIN		
21:00	6ihx-C	4.6	3.1	84	140	7	MOLECULE:	HEMOGLOBIN	SUBUNIT	ALPHA
22:00	3vrf-A	4.6	3.2	86	141	7	MOLECULE:	HEMOGLOBIN	SUBUNIT	ALPHA
23:00	4hhb-A	4.6	3	84	141	7	MOLECULE:	HEMOGLOBIN	SUBUNIT	ALPHA
24:00:00	1bbb-A	4.6	3.4	85	141	5	MOLECULE:	HEMOGLOBIN	A (CARB	ONMONOXY) (ALPHA CHAIN)
25:00:00	2hbe-A	4.6	2.9	83	141	7	MOLECULE:	HEMOGLOBIN	A (N-BU	TYL ISOCYANIDE) (ALPHA CHAIN)
26:00:00	1yen-A	4.6	3.1	85	138	6	MOLECULE:	HEMOGLOBIN	ALPHA C	HAIN
27:00:00	1y45-C	4.6	3	84	141	7	MOLECULE:	HEMOGLOBIN	ALPHA C	HAIN
28:00:00	3qjd-C	4.6	3	84	141	7	MOLECULE:	HEMOGLOBIN	SUBUNIT	ALPHA
29:00:00	1y4p-A	4.6	3	84	141	7	MOLECULE:	HEMOGLOBIN	ALPHA C	HAIN
30:00:00	4hhb-C	4.6	3	84	141	7	MOLECULE:	HEMOGLOBIN	SUBUNIT	ALPHA
31:00:00	1mni-B	4.6	4.2	90	153	13	MOLECULE:	MYOGLOBIN		
32:00:00	6kai-C	4.6	3	84	141	7	MOLECULE:	HEMOGLOBIN	SUBUNIT	ALPHA
33:00:00	1y4q-A	4.6	3.6	86	141	5	MOLECULE:	HEMOGLOBIN	ALPHA C	HAIN
34:00:00	8dov-A	4.6	3.4	86	140	7	MOLECULE:	HEMOGLOBIN	SUBUNIT	ALPHA
35:00:00	6xdt-A	4.6	2.9	83	141	7	MOLECULE:	HEMOGLOBIN	SUBUNIT	ALPHA
36:00:00	6kat-C	4.6	3.1	85	141	6	MOLECULE:	HEMOGLOBIN	SUBUNIT	ALPHA
37:00:00	1y22-A	4.6	3	84	141	7	MOLECULE:	HEMOGLOBIN	ALPHA C	HAIN
38:00:00	1qxd-A	4.6	2.9	83	141	7	MOLECULE:	HEMOGLOBIN	ALPHA C	HAIN
39:00:00	6kas-C	4.6	3.1	85	141	6	MOLECULE:	HEMOGLOBIN	SUBUNIT	ALPHA
40:00:00	1y09-A	4.6	3	84	141	7	MOLECULE:	HEMOGLOBIN	ALPHA C	HAIN
41:00:00	6kav-C	4.6	3	84	141	6	MOLECULE:	HEMOGLOBIN	SUBUNIT	ALPHA
42:00:00	1fdh-B	4.6	3	84	141	7	MOLECULE:	HEMOGLOBIN	F (DEOX	Y) (ALPHA CHAIN)
43:00:00	1hac-C	4.6	3.4	86	141	6	MOLECULE:	HEMOGLOBIN	A	
44:00:00	7uvb-C	4.6	2.9	83	141	7	MOLECULE:	HEMOGLOBIN	SUBUNIT	ALPHA
45:00:00	7jy3-A	4.6	2.9	83	141	7	MOLECULE:	HEMOGLOBIN	SUBUNIT	ALPHA
46:00:00	6l5w-A	4.6	3.4	85	141	7	MOLECULE:	HEMOGLOBIN	SUBUNIT	ALPHA

47:00:00	1y0w-A	4.6	3	84	141	7	MOLECULE:	HEMOGLOBIN	ALPHA C	HAIN
48:00:00	2evp-A	4.6	4.1	89	153	11	MOLECULE:	MYOGLOBIN		
49:00:00	1y83-A	4.6	3	84	141	7	MOLECULE:	HEMOGLOBIN	ALPHA C	HAIN
50:00:00	2w6v-C	4.6	3	84	141	7	MOLECULE:	HEMOGLOBIN	SUBUNIT	ALPHA
51:00:00	5ucu-A	4.6	3.4	85	140	7	MOLECULE:	HEMOGLOBIN	SUBUNIT	ALPHA
52:00:00	1rq4-C	4.6	3	84	141	7	MOLECULE:	HEMOGLOBIN	ALPHA C	HAIN
53:00:00	1sdk-C	4.6	3.1	84	141	7	MOLECULE:	HEMOGLOBIN	A	
54:00:00	6bwu-A	4.6	3.4	85	140	7	MOLECULE:	HEMOGLOBIN	SUBUNIT	ALPHA
55:00:00	1ye1-C	4.6	3	84	141	7	MOLECULE:	HEMOGLOBIN	ALPHA C	HAIN
56:00:00	1pmb-A	4.6	4.1	89	153	15	MOLECULE:	MYOGLOBIN		
57:00:00	6kap-A	4.6	3.4	85	141	7	MOLECULE:	HEMOGLOBIN	SUBUNIT	ALPHA
58:00:00	6xdt-C	4.6	3.5	86	141	3	MOLECULE:	HEMOGLOBIN	SUBUNIT	ALPHA
59:00:00	1y7c-C	4.6	3	84	141	7	MOLECULE:	HEMOGLOBIN	ALPHA C	HAIN
60:00:00	1lft-A	4.6	3	84	141	7	MOLECULE:	HEMOGLOBIN	ALPHA C	HAIN
61:00:00	7pch-A	4.6	3.4	85	141	7	MOLECULE:	HEMOGLOBIN	SUBUNIT	ALPHA
62:00:00	1v4w-C	4.6	3.3	85	143	5	MOLECULE:	HEMOGLOBIN	ALPHA C	HAIN
63:00:00	1j7s-C	4.6	3	84	141	7	MOLECULE:	HEMOGLOBIN		
64:00:00	3qjb-A	4.6	3.4	85	141	7	MOLECULE:	HEMOGLOBIN	SUBUNIT	ALPHA
65:00:00	4n7n-G	4.6	3.1	86	141	6	MOLECULE:	HEMOGLOBIN	SUBUNIT	ALPHA
66:00:00	1gbu-A	4.6	3	84	141	7	MOLECULE:	HEMOGLOBIN		
67:00:00	1v4x-A	4.6	3.3	87	143	5	MOLECULE:	HEMOGLOBIN	ALPHA C	HAIN
68:00:00	2hhe-A	4.6	3	84	141	7	MOLECULE:	HEMOGLOBIN	(DEOXY)	(ALPHA CHAIN)
69:00:00	3fs4-A	4.6	3.6	87	141	5	MOLECULE:	HEMOGLOBIN	SUBUNIT	ALPHA-A
70:00:00	6kar-A	4.6	3.4	85	141	7	MOLECULE:	HEMOGLOBIN	SUBUNIT	ALPHA
71:00:00	6kas-A	4.6	3	84	141	7	MOLECULE:	HEMOGLOBIN	SUBUNIT	ALPHA
72:00:00	1j7y-C	4.6	3	84	141	7	MOLECULE:	HEMOGLOBIN		
73:00:00	1xz5-A	4.6	3	84	141	7	MOLECULE:	HEMOGLOBIN	ALPHA C	HAIN
74:00:00	6hbw-C	4.6	2.9	84	141	7	MOLECULE:	PROTEIN (H	EMOGLOBI	N ALPHA 1)
75:00:00	1ye2-A	4.6	3.6	86	141	5	MOLECULE:	HEMOGLOBIN	ALPHA C	HAIN
76:00:00	1kd2-A	4.6	3	84	141	7	MOLECULE:	HEMOGLOBIN	ALPHA C	HAIN
77:00:00	1yeu-A	4.6	3	84	141	7	MOLECULE:	HEMOGLOBIN	ALPHA C	HAIN
78:00:00	3sxy-A	4.6	4.4	103	201	6	MOLECULE:	TRANSCRIPT	IONAL RE	GULATOR, GNTR FAMILY
79:00:00	7uf6-C	4.6	3	84	141	6	MOLECULE:	HEMOGLOBIN	SUBUNIT	ALPHA
80:00:00	5sw7-A	4.6	3.4	85	142	7	MOLECULE:	HEMOGLOBIN	SUBUNIT	ALPHA
81:00:00	1hgb-C	4.6	3	84	141	7	MOLECULE:	HEMOGLOBIN	(AQUO M	ET) (ALPHA CHAIN)
82:00:00	1y85-A	4.6	3.1	85	141	7	MOLECULE:	HEMOGLOBIN	ALPHA C	HAIN
83:00:00	3bcq-A	4.6	3.3	85	142	8	MOLECULE:	ALPHA-CHAI	N HEMOGL	OBIN
84:00:00	7dy4-G	4.6	3	84	141	7	MOLECULE:	HEMOGLOBIN	SUBUNIT	ALPHA
85:00:00	1y5j-A	4.6	3.6	86	141	5	MOLECULE:	HEMOGLOBIN	ALPHA C	HAIN
86:00:00	1qi8-A	4.6	3	84	141	7	MOLECULE:	HEMOGLOBIN		
87:00:00	1yvq-A	4.6	2.9	83	141	7	MOLECULE:	HEMOGLOBIN	ALPHA C	HAIN
88:00:00	6ii1-C	4.6	3.1	84	138	7	MOLECULE:	HEMOGLOBIN	SUBUNIT	ALPHA
89:00:00	6lcw-E	4.6	3	84	141	7	MOLECULE:	HEMOGLOBIN	SUBUNIT	ALPHA
90:00:00	3wtg-C	4.6	3.3	86	138	9	MOLECULE:	HEMOGLOBIN	SUBUNIT	ALPHA-A
91:00:00	1cls-C	4.6	3	84	141	7	MOLECULE:	HEMOGLOBIN		
92:00:00	3whm-E	4.6	3	84	140	7	MOLECULE:	HEMOGLOBIN	SUBUNIT	ALPHA
93:00:00	5e29-A	4.6	3	84	141	7	MOLECULE:	HEMOGLOBIN	SUBUNIT	ALPHA
94:00:00	1j7w-C	4.6	3	84	141	7	MOLECULE:	HEMOGLOBIN		
95:00:00	6ka9-E	4.6	3	84	141	7	MOLECULE:	HEMOGLOBIN	SUBUNIT	ALPHA
96:00:00	1a0u-A	4.6	3	84	141	7	MOLECULE:	HEMOGLOBIN	(ALPHA	CHAIN)
97:00:00	6z4t-A	4.6	4.1	88	154	11	MOLECULE:	MYOGLOBIN		
98:00:00	1yvt-A	4.6	3.4	85	141	7	MOLECULE:	HEMOGLOBIN	ALPHA C	HAIN
99:00:00	6lcx-C	4.6	3	84	141	7	MOLECULE:	HEMOGLOBIN	SUBUNIT	ALPHA
0:00	1fsx-C	4.6	3	84	141	7	MOLECULE:	HEMOGLOBIN	ALPHA C	HAIN

1:00	1y4g-A	4.6	3	84	141	7	MOLECULE:	HEMOGLOBIN	ALPHA C	HAIN
2:00	1xye-A	4.6	3	84	141	7	MOLECULE:	HEMOGLOBIN	ALPHA C	HAIN
3:00	2hbs-A	4.6	3	84	141	7	MOLECULE:	HEMOGLOBIN	S (DEOX	Y), ALPHA CHAIN
4:00	6ka9-A	4.6	3	84	141	7	MOLECULE:	HEMOGLOBIN	SUBUNIT	ALPHA
5:00	1y7d-A	4.6	3	84	141	7	MOLECULE:	HEMOGLOBIN	ALPHA C	HAIN
6:00	6lcx-E	4.6	3	84	141	7	MOLECULE:	HEMOGLOBIN	SUBUNIT	ALPHA
7:00	8fdk-C	4.6	3.1	84	141	7	MOLECULE:	HEMOGLOBIN	SUBUNIT	ALPHA
8:00	4mqc-A	4.6	3.1	85	141	7	MOLECULE:	HEMOGLOBIN	SUBUNIT	ALPHA
9:00	1ygd-A	4.6	3	84	141	7	MOLECULE:	HEMOGLOBIN	ALPHA C	HAIN
10:00	4mqh-A	4.6	3.2	86	139	6	MOLECULE:	HEMOGLOBIN	SUBUNIT	ALPHA
11:00	1y85-C	4.6	3.6	86	141	5	MOLECULE:	HEMOGLOBIN	ALPHA C	HAIN
12:00	7cue-C	4.6	3.5	86	141	6	MOLECULE:	HEMOGLOBIN	SUBUNIT	ALPHA
13:00	1yhe-A	4.6	3	84	141	7	MOLECULE:	HEMOGLOBIN	ALPHA C	HAIN
14:00	1yg5-C	4.6	3	84	141	7	MOLECULE:	HEMOGLOBIN	ALPHA C	HAIN
15:00	1j3z-E	4.6	3	84	141	7	MOLECULE:	HEMOGLOBIN	ALPHA C	HAIN
16:00	2yrs-C	4.6	3.3	86	141	7	MOLECULE:	HEMOGLOBIN	SUBUNIT	ALPHA
17:00	1rqa-C	4.6	3	84	141	7	MOLECULE:	HEMOGLOBIN	ALPHA C	HAIN
18:00	2hhb-A	4.6	3	84	141	7	MOLECULE:	HEMOGLOBIN	(DEOXY)	(ALPHA CHAIN)
19:00	7ehx-A	4.6	4.1	88	153	11	MOLECULE:	MYOGLOBIN		
20:00	1y2z-C	4.6	3	84	141	7	MOLECULE:	HEMOGLOBIN	ALPHA C	HAIN
21:00	6hit-E	4.6	3.4	86	142	5	MOLECULE:	HEMOGLOBIN	ALPHA 2	CHAIN
22:00	1buw-A	4.6	3.4	85	141	5	MOLECULE:	PROTEIN (H	EMOGLOBI	N)
23:00	1y7z-A	4.6	3	84	141	7	MOLECULE:	HEMOGLOBIN	ALPHA C	HAIN
24:00:00	1y31-A	4.6	3.6	86	141	5	MOLECULE:	HEMOGLOBIN	ALPHA C	HAIN
25:00:00	8fdk-A	4.6	3.4	85	141	7	MOLECULE:	HEMOGLOBIN	SUBUNIT	ALPHA
26:00:00	6kye-I	4.6	3.6	86	141	5	MOLECULE:	HEMOGLOBIN	SUBUNIT	ALPHA
27:00:00	2hhd-C	4.6	3	85	141	7	MOLECULE:	HEMOGLOBIN	(DEOXY)	(ALPHA CHAIN)
28:00:00	1vxb-A	4.6	4.1	88	153	11	MOLECULE:	MYOGLOBIN		
29:00:00	1ye1-A	4.6	3	84	141	7	MOLECULE:	HEMOGLOBIN	ALPHA C	HAIN
30:00:00	5ee4-C	4.6	2.9	83	141	7	MOLECULE:	HPUA		
31:00:00	1yie-C	4.6	3	84	141	7	MOLECULE:	HEMOGLOBIN	ALPHA C	HAIN
32:00:00	8dov-C	4.6	3.4	86	140	7	MOLECULE:	HEMOGLOBIN	SUBUNIT	ALPHA
33:00:00	1y35-A	4.6	3	84	141	7	MOLECULE:	HEMOGLOBIN	ALPHA C	HAIN
34:00:00	5hu6-A	4.6	2.9	83	141	7	MOLECULE:	HEMOGLOBIN	SUBUNIT	ALPHA
35:00:00	1y4b-A	4.6	3	84	141	7	MOLECULE:	HEMOGLOBIN	ALPHA C	HAIN
36:00:00	5vmm-A	4.6	3.5	88	139	6	MOLECULE:	HEMOGLOBIN	SUBUNIT	ALPHA
37:00:00	3bj1-C	4.6	3.4	86	142	3	MOLECULE:	HEMOGLOBIN	ALPHA	
38:00:00	1yeq-A	4.6	2.9	83	141	7	MOLECULE:	HEMOGLOBIN	ALPHA C	HAIN
39:00:00	1xzv-A	4.6	3	84	141	7	MOLECULE:	HEMOGLOBIN	ALPHA C	HAIN
40:00:00	1nqp-A	4.6	2.9	84	141	6	MOLECULE:	HEMOGLOBIN	ALPHA C	HAIN
41:00:00	1rq4-A	4.6	3	84	141	7	MOLECULE:	HEMOGLOBIN	ALPHA C	HAIN
42:00:00	6kye-C	4.6	3.1	85	140	6	MOLECULE:	HEMOGLOBIN	SUBUNIT	ALPHA
43:00:00	7jy0-C	4.6	2.9	83	141	7	MOLECULE:	HEMOGLOBIN	SUBUNIT	ALPHA
44:00:00	1lfl-R	4.6	3	85	141	6	MOLECULE:	HEMOGLOBIN	ALPHA C	HAIN
45:00:00	2evk-A	4.6	4.1	88	153	11	MOLECULE:	MYOGLOBIN		
46:00:00	7jy1-A	4.6	3	84	141	7	MOLECULE:	HEMOGLOBIN	SUBUNIT	ALPHA
47:00:00	6kau-A	4.6	3.1	85	141	6	MOLECULE:	HEMOGLOBIN	SUBUNIT	ALPHA
48:00:00	6kye-A	4.6	3.5	87	140	6	MOLECULE:	HEMOGLOBIN	SUBUNIT	ALPHA
49:00:00	1hdb-A	4.6	3	84	141	7	MOLECULE:	HEMOGLOBIN	(DEOXY)	BETA-V67T
50:00:00	6l5x-A	4.6	3	84	141	7	MOLECULE:	HEMOGLOBIN	SUBUNIT	ALPHA
51:00:00	1hga-A	4.6	3.3	85	141	7	MOLECULE:	HEMOGLOBIN	(DEOXY)	(ALPHA CHAIN)
52:00:00	1y5k-A	4.6	3	84	141	7	MOLECULE:	HEMOGLOBIN	ALPHA C	HAIN
53:00:00	7uvb-A	4.6	3.1	85	141	6	MOLECULE:	HEMOGLOBIN	SUBUNIT	ALPHA
54:00:00	2yrs-M	4.6	3.3	85	141	7	MOLECULE:	HEMOGLOBIN	SUBUNIT	ALPHA

55:00:00	5u3i-A	4.6	3	84	141	6	MOLECULE:	HEMOGLOBIN	SUBUNIT	ALPHA
56:00:00	3fs4-C	4.6	3.1	85	141	7	MOLECULE:	HEMOGLOBIN	SUBUNIT	ALPHA-A
57:00:00	1yeu-C	4.6	3	84	141	7	MOLECULE:	HEMOGLOBIN	ALPHA C	HAIN
58:00:00	1y7c-A	4.6	3	84	141	7	MOLECULE:	HEMOGLOBIN	ALPHA C	HAIN
59:00:00	1y31-C	4.6	3	84	141	7	MOLECULE:	HEMOGLOBIN	ALPHA C	HAIN
60:00:00	6lcw-G	4.6	2.9	84	141	7	MOLECULE:	HEMOGLOBIN	SUBUNIT	ALPHA
61:00:00	8fdn-A	4.6	3.1	84	141	7	MOLECULE:	HEMOGLOBIN	SUBUNIT	ALPHA
62:00:00	6bwp-C	4.6	2.9	84	141	7	MOLECULE:	HEMOGLOBIN	SUBUNIT	ALPHA
63:00:00	5hlq-A	4.6	4.2	88	152	11	MOLECULE:	MYOGLOBIN		
64:00:00	1nih-A	4.6	3	85	141	7	MOLECULE:	HEMOGLOBIN	(NICKEL	OUS DEOXY) (ALPHA CHAIN)
65:00:00	6l5v-A	4.6	3.4	85	141	7	MOLECULE:	HEMOGLOBIN	SUBUNIT	ALPHA
66:00:00	1y5f-A	4.6	3	84	141	7	MOLECULE:	HEMOGLOBIN	ALPHA C	HAIN
67:00:00	1yih-A	4.6	3	84	141	7	MOLECULE:	HEMOGLOBIN	ALPHA C	HAIN
68:00:00	7jjq-A	4.6	3.4	85	140	7	MOLECULE:	HEMOGLOBIN	SUBUNIT	ALPHA
69:00:00	1g08-A	4.6	3.6	87	141	5	MOLECULE:	HEMOGLOBIN	ALPHA C	HAIN
70:00:00	3bj1-A	4.6	3.4	86	142	3	MOLECULE:	HEMOGLOBIN	ALPHA	
71:00:00	4esa-A	4.6	3	84	142	5	MOLECULE:	HEMOGLOBIN	ALPHA C	HAIN
72:00:00	1y0a-A	4.6	3	85	141	7	MOLECULE:	HEMOGLOBIN	ALPHA C	HAIN
73:00:00	4x0i-A	4.6	2.9	83	141	7	MOLECULE:	HEMOGLOBIN	SUBUNIT	ALPHA
74:00:00	1r1x-A	4.6	3.1	84	141	7	MOLECULE:	HEMOGLOBIN	ALPHA C	HAIN
75:00:00	3vrg-A	4.6	3.2	85	141	7	MOLECULE:	HEMOGLOBIN	SUBUNIT	ALPHA
76:00:00	6kye-E	4.6	3.4	86	140	6	MOLECULE:	HEMOGLOBIN	SUBUNIT	ALPHA
77:00:00	6kah-C	4.6	3	84	141	7	MOLECULE:	HEMOGLOBIN	SUBUNIT	ALPHA
78:00:00	1ycb-A	4.6	4.1	90	153	13	MOLECULE:	MYOGLOBIN		
79:00:00	7k4m-A	4.6	3	84	141	7	MOLECULE:	HEMOGLOBIN	SUBUNIT	ALPHA
80:00:00	1gzx-A	4.6	3	84	141	7	MOLECULE:	HEMOGLOBIN	SUBUNIT	ALPHA
81:00:00	7ud8-A	4.6	3	84	141	6	MOLECULE:	HEMOGLOBIN	SUBUNIT	ALPHA
82:00:00	4fwz-A	4.6	4	88	153	11	MOLECULE:	MYOGLOBIN		
83:00:00	7vw4-A	4.6	4.2	88	152	11	MOLECULE:	MYOGLOBIN		
84:00:00	6kae-C	4.6	3	84	141	7	MOLECULE:	HEMOGLOBIN	SUBUNIT	ALPHA
85:00:00	1ljw-A	4.6	3.1	84	141	7	MOLECULE:	HEMOGLOBIN	ALPHA C	HAIN
86:00:00	6kaq-A	4.6	3.4	85	141	7	MOLECULE:	HEMOGLOBIN	SUBUNIT	ALPHA
87:00:00	2hhb-C	4.6	3.6	86	141	5	MOLECULE:	HEMOGLOBIN	(DEOXY)	(ALPHA CHAIN)
88:00:00	7uf7-A	4.6	3.5	85	141	5	MOLECULE:	HEMOGLOBIN	SUBUNIT	ALPHA
89:00:00	1bab-C	4.6	3	84	142	7	MOLECULE:	HEMOGLOBIN	THIONVI	LLE (DEOXY) (ALPHA CHAIN)
90:00:00	1yev-A	4.6	3	84	138	7	MOLECULE:	HEMOGLOBIN	ALPHA C	HAIN
91:00:00	2w6v-A	4.6	3	85	141	7	MOLECULE:	HEMOGLOBIN	SUBUNIT	ALPHA
92:00:00	1j41-C	4.6	3.1	85	141	7	MOLECULE:	HEMOGLOBIN	ALPHA C	HAIN
93:00:00	1y0t-A	4.6	3	84	141	7	MOLECULE:	HEMOGLOBIN	ALPHA C	HAIN
94:00:00	1j3z-A	4.6	3	84	141	7	MOLECULE:	HEMOGLOBIN	ALPHA C	HAIN
95:00:00	5ksi-C	4.6	3	84	141	7	MOLECULE:	HEMOGLOBIN	SUBUNIT	ALPHA
96:00:00	1fsx-A	4.6	3.5	85	141	5	MOLECULE:	HEMOGLOBIN	ALPHA C	HAIN
97:00:00	4ni1-A	4.6	3.4	86	141	7	MOLECULE:	HEMOGLOBIN	SUBUNIT	ALPHA
98:00:00	1y4v-A	4.6	3	84	141	7	MOLECULE:	HEMOGLOBIN	ALPHA C	HAIN
99:00:00	3ic2-A	4.6	3	84	141	6	MOLECULE:	HEMOGLOBIN	SUBUNIT	ALPHA
0:00	8fdn-C	4.6	3.4	85	141	7	MOLECULE:	HEMOGLOBIN	SUBUNIT	ALPHA
1:00	4xs0-A	4.6	2.9	83	141	7	MOLECULE:	HEMOGLOBIN	SUBUNIT	ALPHA
2:00	7dy4-C	4.6	3	84	141	7	MOLECULE:	HEMOGLOBIN	SUBUNIT	ALPHA
3:00	1xz5-C	4.6	3	84	141	7	MOLECULE:	HEMOGLOBIN	ALPHA C	HAIN
4:00	1hbb-A	4.6	3.1	85	141	7	MOLECULE:	HEMOGLOBIN	A (DEOX	Y) (ALPHA CHAIN)
5:00	1buw-C	4.6	3.5	86	141	3	MOLECULE:	PROTEIN (H	EMOGLOBI	N)
6:00	1bvd-A	4.6	4.1	88	153	11	MOLECULE:	APOMYOGLOB	IN	
7:00	1y4b-C	4.6	3.5	85	141	5	MOLECULE:	HEMOGLOBIN	ALPHA C	HAIN
8:00	5ksi-A	4.6	3	84	141	7	MOLECULE:	HEMOGLOBIN	SUBUNIT	ALPHA

9:00	1thb-C	4.6	3	84	141	7	MOLECULE:	HEMOGLOBIN	A (OXY)	(ALPHA CHAIN)
10:00	1y2z-A	4.6	3.6	86	141	5	MOLECULE:	HEMOGLOBIN	ALPHA C	HAIN
11:00	1ygd-C	4.6	3.1	85	141	6	MOLECULE:	HEMOGLOBIN	ALPHA C	HAIN
12:00	1hda-A	4.6	3	84	141	7	MOLECULE:	HEMOGLOBIN	(DEOXY)	(ALPHA CHAIN)
13:00	1ygf-C	4.6	3	84	141	7	MOLECULE:	HEMOGLOBIN	ALPHA C	HAIN
14:00	4mqg-A	4.6	3.4	85	141	7	MOLECULE:	HEMOGLOBIN	SUBUNIT	ALPHA
15:00	7dy3-E	4.6	3.1	85	141	7	MOLECULE:	HEMOGLOBIN	SUBUNIT	ALPHA
16:00	3vre-C	4.6	3.1	86	141	7	MOLECULE:	HEMOGLOBIN	SUBUNIT	ALPHA
17:00	5ufj-A	4.6	3	84	141	7	MOLECULE:	HEMOGLOBIN	SUBUNIT	ALPHA
18:00	1y4f-A	4.6	3	84	141	7	MOLECULE:	HEMOGLOBIN	ALPHA C	HAIN
19:00	1y8w-A	4.6	3	84	141	7	MOLECULE:	HEMOGLOBIN	ALPHA C	HAIN
20:00	1rps-A	4.6	3	84	141	7	MOLECULE:	HEMOGLOBIN	ALPHA C	HAIN
21:00	1yh9-A	4.6	3.6	86	141	5	MOLECULE:	HEMOGLOBIN	ALPHA C	HAIN
22:00	1o1i-A	4.6	3	83	141	7	MOLECULE:	HEMOGLOBIN	ALPHA C	HAIN
23:00	3cy5-A	4.6	3.6	86	141	5	MOLECULE:	HEMOGLOBIN	SUBUNIT	ALPHA-2
24:00:00	1yca-B	4.6	4.1	90	153	13	MOLECULE:	MYOGLOBIN		
25:00:00	3bj3-C	4.6	3.3	86	142	3	MOLECULE:	HEMOGLOBIN	ALPHA	
26:00:00	1a00-C	4.6	3	85	141	7	MOLECULE:	HEMOGLOBIN	(ALPHA	CHAIN)
27:00:00	2w72-A	4.6	3	84	141	7	MOLECULE:	HUMAN HEMO	GLOBIN A	
28:00:00	1yca-A	4.6	4.1	90	153	13	MOLECULE:	MYOGLOBIN		
29:00:00	1a3n-A	4.6	3	84	141	7	MOLECULE:	HEMOGLOBIN	(ALPHA	CHAIN)
30:00:00	8fdl-C	4.6	3	85	141	7	MOLECULE:	HEMOGLOBIN	SUBUNIT	ALPHA
31:00:00	1bbb-C	4.6	3.5	85	141	5	MOLECULE:	HEMOGLOBIN	A (CARB	ONMONOXY) (ALPHA CHAIN)
32:00:00	3nl7-A	4.6	3.4	85	141	7	MOLECULE:	HEMOGLOBIN	SUBUNIT	ALPHA
33:00:00	1uiw-C	4.6	3	84	141	7	MOLECULE:	HEMOGLOBIN	ALPHA C	HAIN
34:00:00	1v4w-A	4.6	3.3	85	143	5	MOLECULE:	HEMOGLOBIN	ALPHA C	HAIN
35:00:00	1nqp-C	4.6	2.9	83	141	7	MOLECULE:	HEMOGLOBIN	ALPHA C	HAIN
36:00:00	3nmm-A	4.6	3	84	141	7	MOLECULE:	HEMOGLOBIN	SUBUNIT	ALPHA
37:00:00	1rps-C	4.6	3	84	141	7	MOLECULE:	HEMOGLOBIN	ALPHA C	HAIN
38:00:00	3qjc-A	4.6	3.1	84	141	7	MOLECULE:	HEMOGLOBIN	SUBUNIT	ALPHA
39:00:00	1hba-A	4.6	3.5	85	141	5	MOLECULE:	HEMOGLOBIN	ROTHSCH	ILD (DEOXY) (ALPHA CHAIN)
40:00:00	2qss-C	4.6	3.4	85	141	7	MOLECULE:	HEMOGLOBIN	SUBUNIT	ALPHA
41:00:00	6lcx-G	4.6	2.9	84	141	7	MOLECULE:	HEMOGLOBIN	SUBUNIT	ALPHA
42:00:00	1y0c-A	4.6	3.1	85	141	7	MOLECULE:	HEMOGLOBIN	ALPHA C	HAIN
43:00:00	1y4f-C	4.6	3	84	141	7	MOLECULE:	HEMOGLOBIN	ALPHA C	HAIN
44:00:00	1b86-A	4.6	3	84	141	7	MOLECULE:	PROTEIN (H	EMOGLOBI	N ALPHA CHAIN)
45:00:00	1rq3-A	4.6	3	84	141	7	MOLECULE:	HEMOGLOBIN	ALPHA C	HAIN
46:00:00	6kae-E	4.6	3	84	141	7	MOLECULE:	HEMOGLOBIN	SUBUNIT	ALPHA
47:00:00	4esa-C	4.6	3.3	85	142	5	MOLECULE:	HEMOGLOBIN	ALPHA C	HAIN
48:00:00	7jjq-C	4.6	3.4	86	139	7	MOLECULE:	HEMOGLOBIN	SUBUNIT	ALPHA
49:00:00	5wog-A	4.6	3.1	85	137	6	MOLECULE:	HEMOGLOBIN	SUBUNIT	ALPHA
50:00:00	2nsr-A	4.6	4.4	91	152	15	MOLECULE:	MYOGLOBIN		
51:00:00	1yvq-C	4.6	3	84	141	6	MOLECULE:	HEMOGLOBIN	ALPHA C	HAIN
52:00:00	5woh-A	4.6	3.1	85	137	6	MOLECULE:	HEMOGLOBIN	SUBUNIT	ALPHA
53:00:00	7dy3-C	4.6	3	84	141	7	MOLECULE:	HEMOGLOBIN	SUBUNIT	ALPHA
54:00:00	3ubv-G	4.6	3.5	83	131	8	MOLECULE:	HEMOGLOBIN	#NAME?	AVOPROTEIN
55:00:00	1j40-E	4.6	3	84	141	7	MOLECULE:	HEMOGLOBIN	ALPHA C	HAIN
56:00:00	4l7y-C	4.6	3	84	141	7	MOLECULE:	HEMOGLOBIN	SUBUNIT	ALPHA
57:00:00	1y4r-A	4.6	3	84	141	7	MOLECULE:	HEMOGLOBIN	ALPHA C	HAIN
58:00:00	4b3w-A	4.6	4.2	89	154	9	MOLECULE:	CYTOGLOBIN		
59:00:00	7ddu-A	4.6	4.1	88	154	10	MOLECULE:	MYOGLOBIN		
60:00:00	6f19-A	4.6	4.1	88	154	11	MOLECULE:	MYOGLOBIN		
61:00:00	1a3o-A	4.6	3	84	141	7	MOLECULE:	HEMOGLOBIN	(ALPHA	CHAIN)
62:00:00	5b85-A	4.6	4.2	88	153	13	MOLECULE:	MYOGLOBIN		

63:00:00	1o1k-A	4.6	3.1	84	140	7	MOLECULE:	HEMOGLOBIN	ALPHA C	HAIN
64:00:00	1mnh-A	4.6	4.1	89	153	17	MOLECULE:	MYOGLOBIN		
65:00:00	1o1k-C	4.6	3.1	84	140	7	MOLECULE:	HEMOGLOBIN	ALPHA C	HAIN
66:00:00	6ii1-A	4.6	3.1	84	137	7	MOLECULE:	HEMOGLOBIN	SUBUNIT	ALPHA
67:00:00	7uf6-A	4.6	2.9	83	141	7	MOLECULE:	HEMOGLOBIN	SUBUNIT	ALPHA
68:00:00	5jgg-A	4.6	3.5	87	142	1	MOLECULE:	ALPHA CHAI	N	
69:00:00	1urv-B	4.6	4.2	90	154	10	MOLECULE:	CYTOGLOBIN		
70:00:00	7dy3-G	4.6	2.9	83	141	7	MOLECULE:	HEMOGLOBIN	SUBUNIT	ALPHA
71:00:00	1aby-A	4.6	9.3	105	283	8	MOLECULE:	HEMOGLOBIN		
72:00:00	6kae-A	4.6	3	84	141	7	MOLECULE:	HEMOGLOBIN	SUBUNIT	ALPHA
73:00:00	4hrt-E	4.6	3.4	86	149	3	MOLECULE:	GLOBIN-2 A	CHAIN	
74:00:00	1myh-A	4.6	4	89	153	15	MOLECULE:	MYOGLOBIN		
75:00:00	6zmx-C	4.6	3.1	86	138	6	MOLECULE:	HEMOGLOBIN	SUBUNIT	ALPHA-A
76:00:00	5ycj-A	4.6	4.2	89	154	17	MOLECULE:	ANCESTRAL	MYOGLOBI	N AMBWB' OF BASILOSAURUS RELATI
77:00:00	1y46-A	4.6	3	84	141	7	MOLECULE:	HEMOGLOBIN	ALPHA C	HAIN
78:00:00	3ciu-A	4.6	3	84	140	7	MOLECULE:	HEMOGLOBIN	SUBUNIT	ALPHA
79:00:00	3hxn-A	4.6	3.3	85	141	7	MOLECULE:	HEMOGLOBIN	SUBUNIT	ALPHA
80:00:00	1ux9-B	4.6	4.2	90	154	9	MOLECULE:	CYTOGLOBIN		
81:00:00	1m6c-A	4.6	4.1	90	153	13	MOLECULE:	PROTEIN (M	YOGLOBIN	)
82:00:00	6iyh-A	4.6	3.4	85	142	2	MOLECULE:	ALPHA CHAI	N	
83:00:00	2bnl-D	4.6	4	79	129	8	MOLECULE:	MODULATOR	PROTEIN	RSBR
84:00:00	1ygf-A	4.6	3	84	141	7	MOLECULE:	HEMOGLOBIN	ALPHA C	HAIN
85:00:00	2bnl-E	4.6	3.6	77	129	8	MOLECULE:	MODULATOR	PROTEIN	RSBR
86:00:00	1j7s-A	4.6	3	84	141	7	MOLECULE:	HEMOGLOBIN		
87:00:00	6kai-A	4.6	3	84	141	7	MOLECULE:	HEMOGLOBIN	SUBUNIT	ALPHA
88:00:00	2bnl-A	4.6	4	79	129	8	MOLECULE:	MODULATOR	PROTEIN	RSBR
89:00:00	1ut0-B	4.6	4.2	90	154	10	MOLECULE:	CYTOGLOBIN		
90:00:00	6zmy-C	4.6	3.1	85	137	6	MOLECULE:	HEMOGLOBIN	SUBUNIT	ALPHA-A
91:00:00	6lcw-A	4.6	3	84	141	7	MOLECULE:	HEMOGLOBIN	SUBUNIT	ALPHA
92:00:00	8j4l-A	4.6	4.1	88	153	11	MOLECULE:	MYOGLOBIN		
93:00:00	7pcq-C	4.6	3.4	85	141	7	MOLECULE:	HEMOGLOBIN	SUBUNIT	ALPHA
94:00:00	1uiw-A	4.6	3	84	141	7	MOLECULE:	HEMOGLOBIN	ALPHA C	HAIN
95:00:00	1yma-A	4.6	4.3	90	153	14	MOLECULE:	MYOGLOBIN		
96:00:00	6lcx-A	4.6	3	84	141	7	MOLECULE:	HEMOGLOBIN	SUBUNIT	ALPHA
97:00:00	1fdh-A	4.6	3	84	141	7	MOLECULE:	HEMOGLOBIN	F (DEOX	Y) (ALPHA CHAIN)
98:00:00	1hbg-A	4.5	3.5	87	147	8	MOLECULE:	HEMOGLOBIN	(CARBON	MONOXY)
99:00:00	4hrr-A	4.5	3.3	87	149	3	MOLECULE:	GLOBIN-2 A	CHAIN	
0:00	1yhu-U	4.5	3.3	84	145	12	MOLECULE:	HEMOGLOBIN	A1 CHAI	N
1:00	3bj2-C	4.5	3.9	88	142	2	MOLECULE:	HEMOGLOBIN	ALPHA	
2:00	8u5a-B	4.5	3.7	84	149	7	MOLECULE:	DESIGNED M	YOGLOBIN	
3:00	8u5a-A	4.5	3.6	85	149	8	MOLECULE:	DESIGNED M	YOGLOBIN	
4:00	1hco-A	4.5	3.7	88	141	3	MOLECULE:	HEMOGLOBIN	(CARBON	MONOXY) (ALPHA CHAIN)
5:00	1ymb-A	4.5	4.1	90	153	13	MOLECULE:	METMYOGLOB	IN	
6:00	1sdl-A	4.5	3.4	86	141	7	MOLECULE:	HEMOGLOBIN	A	
7:00	3eu1-C	4.5	3.5	87	141	6	MOLECULE:	HEMOGLOBIN	SUBUNIT	ALPHA-1/2
8:00	1g0a-A	4.5	3.5	85	141	5	MOLECULE:	HEMOGLOBIN	ALPHA C	HAIN
9:00	5hy8-C	4.5	3.5	85	141	5	MOLECULE:	HEMOGLOBIN	SUBUNIT	ALPHA
10:00	6hk2-A	4.5	3.4	85	141	7	MOLECULE:	HEMOGLOBIN	SUBUNIT	ALPHA
11:00	1g09-C	4.5	3.6	85	141	5	MOLECULE:	HEMOGLOBIN	ALPHA C	HAIN
12:00	1aj9-A	4.5	3.4	86	141	7	MOLECULE:	HEMOGLOBIN	(ALPHA	CHAIN)
13:00	1y7g-A	4.5	3.1	85	141	7	MOLECULE:	HEMOGLOBIN	ALPHA C	HAIN
14:00	6kah-E	4.5	3.1	85	141	7	MOLECULE:	HEMOGLOBIN	SUBUNIT	ALPHA
15:00	7jy1-C	4.5	2.9	83	141	7	MOLECULE:	HEMOGLOBIN	SUBUNIT	ALPHA
16:00	8egi-C	4.5	3.5	86	141	3	MOLECULE:	HEMOGLOBIN	SUBUNIT	ALPHA

17:00	1yzi-A	4.5	3.4	86	141	6	MOLECULE:	HEMOGLOBIN	ALPHA C	HAIN
18:00	1fhj-C	4.5	3.6	86	141	5	MOLECULE:	HEMOGLOBIN	(ALPHA	CHAIN)
19:00	1xq5-C	4.5	3.4	86	142	6	MOLECULE:	HEMOGLOBIN	ALPHA-1	CHAIN
20:00	5e29-C	4.5	3.1	85	141	7	MOLECULE:	HEMOGLOBIN	SUBUNIT	ALPHA
21:00	5e83-A	4.5	2.9	83	141	7	MOLECULE:	HEMOGLOBIN	SUBUNIT	ALPHA
22:00	7jy0-A	4.5	3	84	141	7	MOLECULE:	HEMOGLOBIN	SUBUNIT	ALPHA
23:00	6r2o-C	4.5	2.9	83	141	7	MOLECULE:	HEMOGLOBIN	SUBUNIT	ALPHA
24:00:00	1a9w-A	4.5	3.5	85	141	5	MOLECULE:	HEMOGLOBIN	(ALPHA	CHAIN)
25:00:00	4f4o-G	4.5	3.1	84	141	7	MOLECULE:	HEMOGLOBIN	SUBUNIT	ALPHA
26:00:00	1yff-C	4.5	3.5	85	141	5	MOLECULE:	HEMOGLOBIN	ALPHA C	HAIN
27:00:00	1yff-A	4.5	3.1	84	141	7	MOLECULE:	HEMOGLOBIN	ALPHA C	HAIN
28:00:00	3gqg-C	4.5	3.3	85	142	6	MOLECULE:	HEMOGLOBIN	SUBUNIT	ALPHA
29:00:00	1dke-A	4.5	3.1	85	141	7	MOLECULE:	HEMOGLOBIN	: ALPHA	CHAIN
30:00:00	7jxz-C	4.5	3.5	85	141	5	MOLECULE:	HEMOGLOBIN	SUBUNIT	ALPHA
31:00:00	3gdj-A	4.5	3.2	86	141	7	MOLECULE:	HEMOGLOBIN	SUBUNIT	ALPHA
32:00:00	6kye-G	4.5	3	84	140	7	MOLECULE:	HEMOGLOBIN	SUBUNIT	ALPHA
33:00:00	1jy7-A	4.5	2.9	83	141	7	MOLECULE:	HEMOGLOBIN	ALPHA C	HAIN
34:00:00	3gys-G	4.5	3.1	85	138	8	MOLECULE:	HEMOGLOBIN	SUBUNIT	ALPHA
35:00:00	7cue-A	4.5	3.4	85	141	7	MOLECULE:	HEMOGLOBIN	SUBUNIT	ALPHA
36:00:00	1ird-A	4.5	3.4	85	141	7	MOLECULE:	HEMOGLOBIN	ALPHA C	HAIN
37:00:00	4fc3-A	4.5	2.9	83	140	7	MOLECULE:	HEMOGLOBIN	SUBUNIT	ALPHA
38:00:00	1yff-E	4.5	3.5	85	141	5	MOLECULE:	HEMOGLOBIN	ALPHA C	HAIN
39:00:00	2dn3-A	4.5	3.4	85	141	7	MOLECULE:	HEMOGLOBIN	ALPHA S	UBUNIT
40:00:00	1jy7-C	4.5	3.5	85	141	5	MOLECULE:	HEMOGLOBIN	ALPHA C	HAIN
41:00:00	4wjg-Z	4.5	3.6	85	141	5	MOLECULE:	HEMOGLOBIN	SUBUNIT	ALPHA
42:00:00	3pia-A	4.5	3.5	85	141	5	MOLECULE:	HEMOGLOBIN	SUBUNIT	ALPHA
43:00:00	1hho-A	4.5	3.1	84	141	7	MOLECULE:	HEMOGLOBIN	A (OXY)	(ALPHA CHAIN)
44:00:00	6hit-C	4.5	3.1	85	142	5	MOLECULE:	HEMOGLOBIN	ALPHA 2	CHAIN
45:00:00	6hit-G	4.5	3	84	142	6	MOLECULE:	HEMOGLOBIN	ALPHA 2	CHAIN
46:00:00	4mqi-A	4.5	3.1	85	140	7	MOLECULE:	HEMOGLOBIN	SUBUNIT	ALPHA
47:00:00	1jy7-P	4.5	3.5	85	141	5	MOLECULE:	HEMOGLOBIN	ALPHA C	HAIN
48:00:00	6xd9-A	4.5	3.5	85	141	5	MOLECULE:	HEMOGLOBIN	SUBUNIT	ALPHA
49:00:00	1bij-C	4.5	2.9	83	141	7	MOLECULE:	HEMOGLOBIN	A	
50:00:00	3s65-C	4.5	3.5	86	141	3	MOLECULE:	HEMOGLOBIN	SUBUNIT	ALPHA
51:00:00	3ic0-A	4.5	3	84	141	6	MOLECULE:	HEMOGLOBIN	SUBUNIT	ALPHA
52:00:00	5hlu-A	4.5	4.2	89	152	11	MOLECULE:	MYOGLOBIN		
53:00:00	1j40-C	4.5	3.1	85	141	7	MOLECULE:	HEMOGLOBIN	ALPHA C	HAIN
54:00:00	1jy7-W	4.5	2.9	83	141	7	MOLECULE:	HEMOGLOBIN	ALPHA C	HAIN
55:00:00	5ksj-C	4.5	3.5	85	141	5	MOLECULE:	HEMOGLOBIN	SUBUNIT	ALPHA
56:00:00	6bnr-C	4.5	3	84	141	6	MOLECULE:	HEMOGLOBIN	SUBUNIT	ALPHA
57:00:00	4mqk-A	4.5	3.5	85	141	7	MOLECULE:	HEMOGLOBIN	SUBUNIT	ALPHA
58:00:00	1j3y-A	4.5	3.1	85	141	7	MOLECULE:	HEMOGLOBIN	ALPHA C	HAIN
59:00:00	3gqg-A	4.5	3.3	85	142	6	MOLECULE:	HEMOGLOBIN	SUBUNIT	ALPHA
60:00:00	7pch-C	4.5	3.9	87	141	5	MOLECULE:	HEMOGLOBIN	SUBUNIT	ALPHA
61:00:00	3pi8-C	4.5	2.9	83	141	7	MOLECULE:	HEMOGLOBIN	SUBUNIT	ALPHA
62:00:00	6xe7-A	4.5	3.5	85	141	5	MOLECULE:	HEMOGLOBIN	SUBUNIT	ALPHA
63:00:00	1bij-A	4.5	3.5	85	141	5	MOLECULE:	HEMOGLOBIN	A	
64:00:00	7uf7-C	4.5	3.5	86	141	3	MOLECULE:	HEMOGLOBIN	SUBUNIT	ALPHA
65:00:00	3whm-A	4.5	2.9	83	140	7	MOLECULE:	HEMOGLOBIN	SUBUNIT	ALPHA
66:00:00	6hk2-C	4.5	3.5	86	141	7	MOLECULE:	HEMOGLOBIN	SUBUNIT	ALPHA
67:00:00	3ng6-A	4.5	3.3	85	142	6	MOLECULE:	HEMOGLOBIN	SUBUNIT	ALPHA-1
68:00:00	2qsp-A	4.5	3.5	85	141	5	MOLECULE:	HEMOGLOBIN	SUBUNIT	ALPHA
69:00:00	1hbh-A	4.5	3.3	85	142	6	MOLECULE:	HEMOGLOBIN	(DEOXY)	(ALPHA CHAIN)
70:00:00	6kai-E	4.5	3.1	85	141	7	MOLECULE:	HEMOGLOBIN	SUBUNIT	ALPHA

71:00:00	2h8d-A	4.5	3.3	85	142	6	MOLECULE:	HEMOGLOBIN	ALPHA S	UBUNIT
72:00:00	6nbc-C	4.5	3	84	140	7	MOLECULE:	HEMOGLOBIN	SUBUNIT	ALPHA
73:00:00	1cmy-A	4.5	3	84	141	7	MOLECULE:	HEMOGLOBIN	YPSILAN	TI (CARBONMONOXY) (ALPHA
74:00:00	2aa1-C	4.5	3	84	142	6	MOLECULE:	HEMOGLOBIN	ALPHA-1	CHAIN
75:00:00	1mko-A	4.5	3.1	84	141	7	MOLECULE:	HEMOGLOBIN	ALPHA C	HAIN
76:00:00	1faw-C	4.5	3.1	85	141	8	MOLECULE:	HEMOGLOBIN	(ALPHA	SUBUNIT)
77:00:00	3cy5-C	4.5	3.4	86	141	7	MOLECULE:	HEMOGLOBIN	SUBUNIT	ALPHA-2
78:00:00	2yrs-A	4.5	3	85	141	7	MOLECULE:	HEMOGLOBIN	SUBUNIT	ALPHA
79:00:00	7qu4-A	4.5	3.5	86	142	3	MOLECULE:	HEMOGLOBIN	SUBUNIT	GAMMA-2
80:00:00	4n7n-E	4.5	3	84	141	7	MOLECULE:	HEMOGLOBIN	SUBUNIT	ALPHA
81:00:00	3s48-D	4.5	2.9	83	140	7	MOLECULE:	IRON-REGUL	ATED SUR	FACE DETERMINANT PROTEIN H
82:00:00	5ksj-A	4.5	3	84	141	7	MOLECULE:	HEMOGLOBIN	SUBUNIT	ALPHA
83:00:00	3pia-C	4.5	3.5	85	141	5	MOLECULE:	HEMOGLOBIN	SUBUNIT	ALPHA
84:00:00	6l5x-C	4.5	3.1	85	141	6	MOLECULE:	HEMOGLOBIN	SUBUNIT	ALPHA
85:00:00	7qu4-B	4.5	3.1	84	141	7	MOLECULE:	HEMOGLOBIN	SUBUNIT	GAMMA-2
86:00:00	3vre-A	4.5	3.1	85	141	7	MOLECULE:	HEMOGLOBIN	SUBUNIT	ALPHA
87:00:00	1jy7-U	4.5	2.9	83	141	7	MOLECULE:	HEMOGLOBIN	ALPHA C	HAIN
88:00:00	4hrr-E	4.5	3.3	87	149	3	MOLECULE:	GLOBIN-2 A	CHAIN	
89:00:00	1v4u-C	4.5	3	84	143	5	MOLECULE:	HEMOGLOBIN	ALPHA C	HAIN
90:00:00	2qss-A	4.5	3.1	84	141	7	MOLECULE:	HEMOGLOBIN	SUBUNIT	ALPHA
91:00:00	4mqj-C	4.5	3.5	85	141	7	MOLECULE:	HEMOGLOBIN	SUBUNIT	ALPHA
92:00:00	1lfq-A	4.5	3.5	85	141	5	MOLECULE:	HEMOGLOBIN	ALPHA C	HAIN
93:00:00	1cmy-C	4.5	3	84	141	7	MOLECULE:	HEMOGLOBIN	YPSILAN	TI (CARBONMONOXY) (ALPHA
94:00:00	1nej-C	4.5	2.9	83	141	7	MOLECULE:	HEMOGLOBIN	ALPHA C	HAIN
95:00:00	3bcq-C	4.5	3.3	85	142	8	MOLECULE:	ALPHA-CHAI	N HEMOGL	OBIN
96:00:00	1j40-A	4.5	3.1	85	141	7	MOLECULE:	HEMOGLOBIN	ALPHA C	HAIN
97:00:00	7ud7-C	4.5	2.9	84	141	7	MOLECULE:	HEMOGLOBIN	SUBUNIT	ALPHA
98:00:00	3s66-A	4.5	3.1	85	139	7	MOLECULE:	HEMOGLOBIN	SUBUNIT	ALPHA
99:00:00	6kav-A	4.5	3	84	141	6	MOLECULE:	HEMOGLOBIN	SUBUNIT	ALPHA
0:00	1uiw-G	4.5	3	84	141	7	MOLECULE:	HEMOGLOBIN	ALPHA C	HAIN
1:00	4n7o-G	4.5	3.4	86	141	7	MOLECULE:	HEMOGLOBIN	SUBUNIT	ALPHA
2:00	1ouu-A	4.5	3.3	85	142	6	MOLECULE:	HEMOGLOBIN	I	
3:00	1a9w-C	4.5	2.9	83	141	7	MOLECULE:	HEMOGLOBIN	(ALPHA	CHAIN)
4:00	6zmy-A	4.5	3.1	85	139	6	MOLECULE:	HEMOGLOBIN	SUBUNIT	ALPHA-A
5:00	6xe7-C	4.5	3	84	141	6	MOLECULE:	HEMOGLOBIN	SUBUNIT	ALPHA
6:00	3wcp-A	4.5	3.1	85	141	7	MOLECULE:	HEMOGLOBIN	SUBUNIT	ALPHA
7:00	7vde-A	4.5	3	84	141	7	MOLECULE:	HEMOGLOBIN	SUBUNIT	ALPHA
8:00	4mqj-A	4.5	3.5	86	141	6	MOLECULE:	HEMOGLOBIN	SUBUNIT	ALPHA
9:00	7vde-C	4.5	3	84	141	7	MOLECULE:	HEMOGLOBIN	SUBUNIT	ALPHA
10:00	1m9p-C	4.5	3.5	85	141	5	MOLECULE:	HEMOGLOBIN	ALPHA C	HAIN
11:00	4f4o-J	4.5	3.1	85	141	6	MOLECULE:	HEMOGLOBIN	SUBUNIT	ALPHA
12:00	2qmb-A	4.5	3.6	87	141	3	MOLECULE:	HEMOGLOBIN	SUBUNIT	ALPHA-A
13:00	2dxm-C	4.5	3.7	88	141	3	MOLECULE:	HEMOGLOBIN	SUBUNIT	ALPHA
14:00	2aa1-A	4.5	3.3	85	142	6	MOLECULE:	HEMOGLOBIN	ALPHA-1	CHAIN
15:00	3r5i-A	4.5	3	84	141	7	MOLECULE:	HEMOGLOBIN	SUBUNIT	ALPHA
16:00	3bj2-A	4.5	3.4	86	142	3	MOLECULE:	HEMOGLOBIN	ALPHA	
17:00	1pbx-A	4.5	3.3	85	142	6	MOLECULE:	HEMOGLOBIN	(CARBON	MONOXY) (ALPHA CHAIN)
18:00	1lfv-A	4.5	3.1	84	141	7	MOLECULE:	HEMOGLOBIN	ALPHA C	HAIN
19:00	1m9p-A	4.5	3.5	85	141	5	MOLECULE:	HEMOGLOBIN	ALPHA C	HAIN
20:00	4mqk-E	4.5	3.3	86	138	6	MOLECULE:	HEMOGLOBIN	SUBUNIT	ALPHA
21:00	1g0a-C	4.5	3.5	85	141	5	MOLECULE:	HEMOGLOBIN	ALPHA C	HAIN
22:00	2qls-C	4.5	3.1	85	141	7	MOLECULE:	HEMOGLOBIN	SUBUNIT	ALPHA
23:00	8egi-A	4.5	3.5	85	141	5	MOLECULE:	HEMOGLOBIN	SUBUNIT	ALPHA
24:00:00	1j41-A	4.5	3.1	85	141	7	MOLECULE:	HEMOGLOBIN	ALPHA C	HAIN

25:00:00	1nih-C	4.5	3	84	141	7	MOLECULE:	HEMOGLOBIN	(NICKEL	OUS DEOXY) (ALPHA CHAIN)	
26:00:00	2dxm-A	4.5	3	84	141	7	MOLECULE:	HEMOGLOBIN	SUBUNIT	ALPHA	
27:00:00	6di4-C	4.5	2.9	83	141	7	MOLECULE:	HEMOGLOBIN	SUBUNIT	ALPHA	
28:00:00	1v4x-C	4.5	3.3	85	143	5	MOLECULE:	HEMOGLOBIN	ALPHA C	HAIN	
29:00:00	1si4-A	4.5	3	84	141	6	MOLECULE:	HEMOGLOBIN	ALPHA C	HAIN	
30:00:00	5ker-E	4.5	3.2	86	140	7	MOLECULE:	ALPHA-GLOB	IN		
31:00:00	5urc-C	4.5	3.5	86	141	3	MOLECULE:	HEMOGLOBIN	SUBUNIT	ALPHA	
32:00:00	1hac-A	4.5	3.1	86	141	6	MOLECULE:	HEMOGLOBIN	A		
33:00:00	4n7o-K	4.5	3.1	86	141	6	MOLECULE:	HEMOGLOBIN	SUBUNIT	ALPHA	
34:00:00	3pi8-A	4.5	3.5	85	141	5	MOLECULE:	HEMOGLOBIN	SUBUNIT	ALPHA	
35:00:00	7jy3-C	4.5	3	84	141	7	MOLECULE:	HEMOGLOBIN	SUBUNIT	ALPHA	
36:00:00	6nbd-C	4.5	3.4	86	140	7	MOLECULE:	HEMOGLOBIN	SUBUNIT	ALPHA	
37:00:00	8pur-A	4.5	3.4	85	140	8	MOLECULE:	HEMOGLOBIN	, ALPHA		1
38:00:00	1yg5-A	4.5	3	84	141	7	MOLECULE:	HEMOGLOBIN	ALPHA C	HAIN	
39:00:00	1v4u-A	4.5	3.3	85	143	5	MOLECULE:	HEMOGLOBIN	ALPHA C	HAIN	
40:00:00	2d60-A	4.5	3.1	85	141	7	MOLECULE:	HEMOGLOBIN	ALPHA S	UBUNIT	
41:00:00	3pi9-C	4.5	3.5	85	141	5	MOLECULE:	HEMOGLOBIN	SUBUNIT	ALPHA	
42:00:00	5ufj-C	4.5	2.9	83	141	7	MOLECULE:	HEMOGLOBIN	SUBUNIT	ALPHA	
43:00:00	6kah-A	4.5	3.1	85	141	7	MOLECULE:	HEMOGLOBIN	SUBUNIT	ALPHA	
44:00:00	1rqa-A	4.5	3.5	85	141	5	MOLECULE:	HEMOGLOBIN	ALPHA C	HAIN	
45:00:00	6nbc-A	4.5	3	84	140	7	MOLECULE:	HEMOGLOBIN	SUBUNIT	ALPHA	
46:00:00	5hy8-S	4.5	2.9	83	141	7	MOLECULE:	HEMOGLOBIN	SUBUNIT	ALPHA	
47:00:00	7pcq-A	4.5	3.6	86	141	5	MOLECULE:	HEMOGLOBIN	SUBUNIT	ALPHA	
48:00:00	1j41-E	4.5	3.1	85	141	7	MOLECULE:	HEMOGLOBIN	ALPHA C	HAIN	
49:00:00	1jy7-R	4.5	2.9	83	141	7	MOLECULE:	HEMOGLOBIN	ALPHA C	HAIN	
50:00:00	7k4m-C	4.5	3	84	140	7	MOLECULE:	HEMOGLOBIN	SUBUNIT	ALPHA	
51:00:00	5e83-C	4.5	3	84	141	6	MOLECULE:	HEMOGLOBIN	SUBUNIT	ALPHA	
52:00:00	4n7o-E	4.5	3.1	84	141	7	MOLECULE:	HEMOGLOBIN	SUBUNIT	ALPHA	
53:00:00	1ydz-A	4.5	3.3	85	141	7	MOLECULE:	HEMOGLOBIN	ALPHA C	HAIN	
54:00:00	1qxe-A	4.5	3	84	141	6	MOLECULE:	HEMOGLOBIN	ALPHA C	HAIN	
55:00:00	1uiw-E	4.5	2.9	83	141	7	MOLECULE:	HEMOGLOBIN	ALPHA C	HAIN	
56:00:00	1lfy-A	4.5	3.1	84	141	7	MOLECULE:	HEMOGLOBIN	ALPHA C	HAIN	
57:00:00	1hga-C	4.5	3	85	141	6	MOLECULE:	HEMOGLOBIN	(DEOXY)	(ALPHA CHAIN)	
58:00:00	1yff-G	4.5	3.5	85	141	5	MOLECULE:	HEMOGLOBIN	ALPHA C	HAIN	
59:00:00	4mqk-C	4.5	3.5	86	141	6	MOLECULE:	HEMOGLOBIN	SUBUNIT	ALPHA	
60:00:00	3wr1-A	4.5	3.6	87	141	6	MOLECULE:	HEMOGLOBIN	SUBUNIT	ALPHA-A	
61:00:00	5hy8-A	4.5	3.5	85	141	5	MOLECULE:	HEMOGLOBIN	SUBUNIT	ALPHA	
62:00:00	1mnj-A	4.5	4.3	90	153	13	MOLECULE:	MYOGLOBIN			
63:00:00	3gqr-G	4.5	3.1	85	141	8	MOLECULE:	HEMOGLOBIN	SUBUNIT	ALPHA	
64:00:00	8f9h-A	4.5	4	88	151	11	MOLECULE:	MYOGLOBIN			
65:00:00	5vzn-A	4.5	4	88	151	11	MOLECULE:	MYOGLOBIN			
66:00:00	1a4f-A	4.5	2.9	84	141	8	MOLECULE:	HEMOGLOBIN	(ALPHA	CHAIN)	
67:00:00	5x2r-G	4.5	3.4	85	140	7	MOLECULE:	HEMOGLOBIN	SUBUNIT	ALPHA	
68:00:00	1ux9-A	4.5	4.1	90	154	9	MOLECULE:	CYTOGLOBIN			
69:00:00	4mqk-H	4.5	3.3	85	145	6	MOLECULE:	HEMOGLOBIN	SUBUNIT	ALPHA	
70:00:00	5x2s-G	4.5	3.5	86	140	6	MOLECULE:	HEMOGLOBIN	SUBUNIT	ALPHA	
71:00:00	1s5y-C	4.5	3.1	84	142	6	MOLECULE:	HEMOGLOBIN	ALPHA C	HAIN	
72:00:00	8dov-E	4.5	3.4	85	138	7	MOLECULE:	HEMOGLOBIN	SUBUNIT	ALPHA	
73:00:00	105m-A	4.5	4.2	90	153	9	MOLECULE:	MYOGLOBIN			
74:00:00	4mqk-D	4.5	3.4	85	145	6	MOLECULE:	HEMOGLOBIN	SUBUNIT	ALPHA	
75:00:00	5x2u-G	4.5	3.5	86	140	6	MOLECULE:	HEMOGLOBIN	SUBUNIT	ALPHA	
76:00:00	2ri4-I	4.5	3	84	137	6	MOLECULE:	HEMOGLOBIN	SUBUNIT	ALPHA-1/2	
77:00:00	2dn1-A	4.5	3.4	85	140	7	MOLECULE:	HEMOGLOBIN	ALPHA S	UBUNIT	
78:00:00	3d1k-A	4.5	3.4	85	142	6	MOLECULE:	HEMOGLOBIN	SUBUNIT	ALPHA-1	

79:00:00	6hal-A	4.5	3.1	84	139	7	MOLECULE:	HEMOGLOBIN	SUBUNIT	ALPHA
80:00:00	8vyl-C	4.5	3	84	140	7	MOLECULE:	HEMOGLOBIN	SUBUNIT	ALPHA
81:00:00	6bb5-A	4.5	3.4	85	139	7	MOLECULE:	HEMOGLOBIN	SUBUNIT	ALPHA
82:00:00	5ycg-A	4.5	4.1	88	154	14	MOLECULE:	ANCESTRAL	MYOGLOBI	N AMBWP OF PAKICETUS RELATIVE
83:00:00	5wog-B	4.5	3.4	85	137	7	MOLECULE:	HEMOGLOBIN	SUBUNIT	ALPHA
84:00:00	5x2s-I	4.5	3.1	85	140	6	MOLECULE:	HEMOGLOBIN	SUBUNIT	ALPHA
85:00:00	1la6-A	4.5	3.4	85	142	6	MOLECULE:	HEMOGLOBIN	ALPHA-1	CHAIN
86:00:00	1fhj-A	4.5	3.1	85	141	7	MOLECULE:	HEMOGLOBIN	(ALPHA	CHAIN)
87:00:00	1i3d-A	4.5	3.4	86	146	6	MOLECULE:	HEMOGLOBIN	GAMMA C	HAINS
88:00:00	4hrt-G	4.5	3.4	85	149	4	MOLECULE:	GLOBIN-2 A	CHAIN	
89:00:00	1i3d-B	4.5	3.4	86	146	6	MOLECULE:	HEMOGLOBIN	GAMMA C	HAINS
90:00:00	6rp5-A	4.5	3.5	85	142	5	MOLECULE:	HEMOGLOBIN	SUBUNIT	ALPHA 1
91:00:00	5x2t-I	4.5	3.1	84	140	7	MOLECULE:	HEMOGLOBIN	SUBUNIT	ALPHA
92:00:00	3gqp-C	4.5	3.2	85	141	8	MOLECULE:	HEMOGLOBIN	SUBUNIT	ALPHA
93:00:00	2bnl-B	4.5	3.6	76	128	8	MOLECULE:	MODULATOR	PROTEIN	RSBR
94:00:00	4mqj-F	4.5	3.4	86	146	6	MOLECULE:	HEMOGLOBIN	SUBUNIT	ALPHA
95:00:00	3a59-C	4.5	3.4	86	141	7	MOLECULE:	HEMOGLOBIN	SUBUNIT	ALPHA-A
96:00:00	5x2u-C	4.5	3.5	86	140	6	MOLECULE:	HEMOGLOBIN	SUBUNIT	ALPHA
97:00:00	2r80-C	4.5	3.1	85	140	8	MOLECULE:	HEMOGLOBIN	SUBUNIT	ALPHA-A
98:00:00	4mqj-D	4.5	3.3	85	145	6	MOLECULE:	HEMOGLOBIN	SUBUNIT	ALPHA
99:00:00	5woh-C	4.5	3.4	85	137	7	MOLECULE:	HEMOGLOBIN	SUBUNIT	ALPHA
0:00	5x2u-K	4.5	3	83	140	7	MOLECULE:	HEMOGLOBIN	SUBUNIT	ALPHA
1:00	4hrt-C	4.5	3.5	86	149	3	MOLECULE:	GLOBIN-2 A	CHAIN	
2:00	5ziq-A	4.4	4.4	91	155	8	MOLECULE:	GLOBIN PRO	TEIN	
3:00	7m8i-A	4.4	7.8	145	572	9	MOLECULE:	ADRENODOXI	N, CYTOC	HROME P450 11B2, MITOCHONDRIAL
4:00	6p5b-A	4.4	10.9	94	388	4	MOLECULE:	MAVC		
5:00	7d3u-F	4.4	7.5	76	85	9	MOLECULE:	MONOVALENT	NA+/H+	ANTIPORTER SUBUNIT D
6:00	6vxm-A	4.4	14.8	100	277	6	MOLECULE:	MECHANOSEN	SITIVE I	ON CHANNEL PROTEIN 1, MITOCHOND
7:00	5utd-A	4.4	4.5	92	151	8	MOLECULE:	MYOGLOBIN		
8:00	6ls8-K	4.4	4.2	91	153	14	MOLECULE:	MYOGLOBIN		
9:00	3pi2-B	4.4	4	86	151	7	MOLECULE:	HEMOGLOBIN	II	
10:00	1bvc-A	4.4	4.3	90	153	10	MOLECULE:	APOMYOGLOB	IN	
11:00	2eb8-A	4.4	3.8	90	154	8	MOLECULE:	MYOGLOBIN		
12:00	2olp-B	4.4	4	84	151	5	MOLECULE:	HEMOGLOBIN	II	
13:00	1u7s-A	4.4	4.3	91	153	10	MOLECULE:	MYOGLOBIN		
14:00	3pi1-B	4.4	4	85	151	6	MOLECULE:	HEMOGLOBIN	II	
15:00	3pt8-A	4.4	3.8	84	151	7	MOLECULE:	HEMOGLOBIN	II	
16:00	3a59-G	4.4	3.1	85	141	7	MOLECULE:	HEMOGLOBIN	SUBUNIT	ALPHA-A
17:00	4n7p-I	4.4	3.4	85	141	7	MOLECULE:	HEMOGLOBIN	SUBUNIT	ALPHA
18:00	1ouu-C	4.4	3.4	86	142	6	MOLECULE:	HEMOGLOBIN	I	
19:00	1si4-C	4.4	3.5	85	141	5	MOLECULE:	HEMOGLOBIN	ALPHA C	HAIN
20:00	4wjg-F	4.4	3.1	84	141	7	MOLECULE:	HEMOGLOBIN	SUBUNIT	ALPHA
21:00	3eok-A	4.4	3	83	141	7	MOLECULE:	HEMOGLOBIN	SUBUNIT	ALPHA-A
22:00	3b75-C	4.4	2.9	83	141	7	MOLECULE:	HEMOGLOBIN	SUBUNIT	ALPHA
23:00	4wjg-P	4.4	3.6	85	141	5	MOLECULE:	HEMOGLOBIN	SUBUNIT	ALPHA
24:00:00	4wjg-U	4.4	3.1	84	141	7	MOLECULE:	HEMOGLOBIN	SUBUNIT	ALPHA
25:00:00	3dhr-C	4.4	3.6	87	141	6	MOLECULE:	HEMOGLOBIN	SUBUNIT	ALPHA-A
26:00:00	2qu0-A	4.4	3.7	88	141	3	MOLECULE:	HEMOGLOBIN	SUBUNIT	ALPHA-1/2
27:00:00	3gys-C	4.4	3.6	87	139	6	MOLECULE:	HEMOGLOBIN	SUBUNIT	ALPHA
28:00:00	1ns9-A	4.4	3.2	85	141	7	MOLECULE:	HEMOGLOBIN	ALPHA S	UBUNIT
29:00:00	6xd9-C	4.4	3.5	86	141	3	MOLECULE:	HEMOGLOBIN	SUBUNIT	ALPHA
30:00:00	4wjg-A	4.4	3.6	85	141	5	MOLECULE:	HEMOGLOBIN	SUBUNIT	ALPHA
31:00:00	3d1a-C	4.4	3.4	85	141	7	MOLECULE:	HEMOGLOBIN	SUBUNIT	ALPHA-1/2
32:00:00	1lfz-A	4.4	3.4	86	141	6	MOLECULE:	HEMOGLOBIN	ALPHA C	HAIN

33:00:00	2zlu-A	4.4	3.1	84	140	7	MOLECULE:	HEMOGLOBIN	SUBUNIT	ALPHA
34:00:00	3d4x-C	4.4	3	83	141	10	MOLECULE:	HEMOGLOBIN	SUBUNIT	ALPHA
35:00:00	1qpw-C	4.4	3.2	85	141	7	MOLECULE:	PORCINE HE	MOGLOBIN	(ALPHA SUBUNIT)
36:00:00	1y8i-C	4.4	3	83	141	7	MOLECULE:	HEMOGLOBIN	ALPHA C	HAINS
37:00:00	6tb2-A	4.4	3.6	85	141	5	MOLECULE:	HEMOGLOBIN	SUBUNIT	ALPHA
38:00:00	1g0b-A	4.4	3.1	84	141	7	MOLECULE:	HEMOGLOBIN	ALPHA C	HAIN
39:00:00	1y8k-C	4.4	2.9	83	141	7	MOLECULE:	HEMOGLOBIN	ALPHA C	HAINS
40:00:00	3ic2-C	4.4	3.5	85	141	5	MOLECULE:	HEMOGLOBIN	SUBUNIT	ALPHA
41:00:00	1out-A	4.4	3	84	142	6	MOLECULE:	HEMOGLOBIN	I	
42:00:00	1qxe-C	4.4	3	83	141	7	MOLECULE:	HEMOGLOBIN	ALPHA C	HAIN
43:00:00	3dht-A	4.4	3.2	86	139	7	MOLECULE:	HEMOGLOBIN	SUBUNIT	ALPHA-1/2
44:00:00	7jxz-A	4.4	3	84	141	7	MOLECULE:	HEMOGLOBIN	SUBUNIT	ALPHA
45:00:00	3kmf-E	4.4	3	84	141	7	MOLECULE:	HEMOGLOBIN	SUBUNIT	ALPHA
46:00:00	4wjg-K	4.4	3.6	85	141	5	MOLECULE:	HEMOGLOBIN	SUBUNIT	ALPHA
47:00:00	3d1a-A	4.4	3.8	88	141	3	MOLECULE:	HEMOGLOBIN	SUBUNIT	ALPHA-1/2
48:00:00	4mqj-G	4.4	3.2	84	141	7	MOLECULE:	HEMOGLOBIN	SUBUNIT	ALPHA
49:00:00	3dhr-A	4.4	3.1	85	141	8	MOLECULE:	HEMOGLOBIN	SUBUNIT	ALPHA-A
50:00:00	2zlt-A	4.4	3.1	84	141	7	MOLECULE:	HEMOGLOBIN	SUBUNIT	ALPHA
51:00:00	3gou-A	4.4	3.2	86	141	7	MOLECULE:	HEMOGLOBIN	SUBUNIT	ALPHA
52:00:00	1qxd-C	4.4	3.1	84	141	7	MOLECULE:	HEMOGLOBIN	ALPHA C	HAIN
53:00:00	4mqk-G	4.4	3.3	86	140	6	MOLECULE:	HEMOGLOBIN	SUBUNIT	ALPHA
54:00:00	4n7o-A	4.4	3.8	87	141	5	MOLECULE:	HEMOGLOBIN	SUBUNIT	ALPHA
55:00:00	3b75-E	4.4	3.3	86	141	6	MOLECULE:	HEMOGLOBIN	SUBUNIT	ALPHA
56:00:00	3r5i-C	4.4	3.1	84	141	7	MOLECULE:	HEMOGLOBIN	SUBUNIT	ALPHA
57:00:00	3gys-E	4.4	2.9	83	138	10	MOLECULE:	HEMOGLOBIN	SUBUNIT	ALPHA
58:00:00	2qu0-C	4.4	3.1	84	141	7	MOLECULE:	HEMOGLOBIN	SUBUNIT	ALPHA-1/2
59:00:00	3pel-A	4.4	3.1	85	141	7	MOLECULE:	HEMOGLOBIN	SUBUNIT	ALPHA
60:00:00	3dhr-G	4.4	3.5	86	141	6	MOLECULE:	HEMOGLOBIN	SUBUNIT	ALPHA-A
61:00:00	3dhr-E	4.4	3	84	141	8	MOLECULE:	HEMOGLOBIN	SUBUNIT	ALPHA-A
62:00:00	1nej-A	4.4	2.9	83	141	7	MOLECULE:	HEMOGLOBIN	ALPHA C	HAIN
63:00:00	5c6e-A	4.4	3.1	84	141	7	MOLECULE:	HEMOGLOBIN	SUBUNIT	ALPHA
64:00:00	1mko-C	4.4	3.1	85	141	6	MOLECULE:	HEMOGLOBIN	ALPHA C	HAIN
65:00:00	2bw9-M	4.4	4.2	90	153	9	MOLECULE:	MYOGLOBIN		
66:00:00	3d4x-A	4.4	3.1	85	141	9	MOLECULE:	HEMOGLOBIN	SUBUNIT	ALPHA
67:00:00	3gqr-E	4.4	3.1	85	141	8	MOLECULE:	HEMOGLOBIN	SUBUNIT	ALPHA
68:00:00	3gou-C	4.4	3.4	85	141	8	MOLECULE:	HEMOGLOBIN	SUBUNIT	ALPHA
69:00:00	4h2l-A	4.4	3.2	85	141	8	MOLECULE:	ALPHA-GLOB	IN	
70:00:00	1iwh-A	4.4	3.1	84	141	7	MOLECULE:	HEMOGLOBIN	ALPHA C	HAIN
71:00:00	3b75-S	4.4	3.5	86	141	3	MOLECULE:	HEMOGLOBIN	SUBUNIT	ALPHA
72:00:00	3s65-A	4.4	2.9	83	141	7	MOLECULE:	HEMOGLOBIN	SUBUNIT	ALPHA
73:00:00	2rao-A	4.4	3.5	85	141	6	MOLECULE:	HEMOGLOBIN	SUBUNIT	ALPHA-1/2
74:00:00	1shr-C	4.4	3	83	141	7	MOLECULE:	HEMOGLOBIN	ALPHA C	HAIN
75:00:00	4n7p-C	4.4	3.1	84	141	7	MOLECULE:	HEMOGLOBIN	SUBUNIT	ALPHA
76:00:00	3pi9-A	4.4	3.5	85	141	5	MOLECULE:	HEMOGLOBIN	SUBUNIT	ALPHA
77:00:00	3b75-G	4.4	3.5	86	141	7	MOLECULE:	HEMOGLOBIN	SUBUNIT	ALPHA
78:00:00	3a59-A	4.4	3.6	87	141	5	MOLECULE:	HEMOGLOBIN	SUBUNIT	ALPHA-A
79:00:00	4yu4-A	4.4	3.5	85	141	7	MOLECULE:	HEMOGLOBIN		
80:00:00	1a01-B	4.4	3.6	86	146	5	MOLECULE:	HEMOGLOBIN	(ALPHA	CHAIN)
81:00:00	3nfe-A	4.4	3	84	142	6	MOLECULE:	HEMOGLOBIN	SUBUNIT	ALPHA-1
82:00:00	7ud8-C	4.4	3.1	84	141	7	MOLECULE:	HEMOGLOBIN	SUBUNIT	ALPHA
83:00:00	1fn3-A	4.4	3.1	85	141	7	MOLECULE:	HEMOGLOBIN	ALPHA C	HAIN
84:00:00	4g51-A	4.4	3.3	85	142	6	MOLECULE:	HEMOGLOBIN	SUBUNIT	ALPHA
85:00:00	4mqj-E	4.4	3.2	84	141	7	MOLECULE:	HEMOGLOBIN	SUBUNIT	ALPHA
86:00:00	2h8d-C	4.4	3.3	85	142	6	MOLECULE:	HEMOGLOBIN	ALPHA S	UBUNIT

87:00:00	2mhb-A	4.4	3.4	85	141	7	MOLECULE:	HEMOGLOBIN	(AQUO M	ET) (ALPHA CHAIN)
88:00:00	2d5x-A	4.4	3.4	85	141	7	MOLECULE:	HEMOGLOBIN	ALPHA S	UBUNIT
89:00:00	1fn3-C	4.4	3.1	85	141	7	MOLECULE:	HEMOGLOBIN	ALPHA C	HAIN
90:00:00	1hbs-E	4.4	2.8	83	141	7	MOLECULE:	HEMOGLOBIN	S (DEOX	Y) (ALPHA CHAIN)
91:00:00	1ibe-A	4.4	3.3	85	141	8	MOLECULE:	HEMOGLOBIN	(DEOXY)	
92:00:00	2h8f-C	4.4	3.3	85	142	6	MOLECULE:	HEMOGLOBIN	ALPHA S	UBUNIT
93:00:00	2h8f-A	4.4	3.3	85	142	6	MOLECULE:	HEMOGLOBIN	ALPHA S	UBUNIT
94:00:00	2qls-A	4.4	3.6	87	141	5	MOLECULE:	HEMOGLOBIN	SUBUNIT	ALPHA
95:00:00	1hbh-C	4.4	2.8	83	142	6	MOLECULE:	HEMOGLOBIN	(DEOXY)	(ALPHA CHAIN)
96:00:00	3b75-A	4.4	3	84	141	6	MOLECULE:	HEMOGLOBIN	SUBUNIT	ALPHA
97:00:00	4f4o-A	4.4	3.6	86	141	3	MOLECULE:	HEMOGLOBIN	SUBUNIT	ALPHA
98:00:00	3gqr-C	4.4	3.1	84	141	10	MOLECULE:	HEMOGLOBIN	SUBUNIT	ALPHA
99:00:00	3a0g-A	4.4	3.6	86	139	5	MOLECULE:	HEMOGLOBIN	SUBUNIT	ALPHA
0:00	3hyu-A	4.4	3.6	86	141	5	MOLECULE:	HEMOGLOBIN	SUBUNIT	ALPHA
1:00	7dy3-A	4.4	3.1	85	141	7	MOLECULE:	HEMOGLOBIN	SUBUNIT	ALPHA
2:00	3gys-A	4.4	3.1	85	138	8	MOLECULE:	HEMOGLOBIN	SUBUNIT	ALPHA
3:00	4f4o-D	4.4	3.1	84	141	7	MOLECULE:	HEMOGLOBIN	SUBUNIT	ALPHA
4:00	4yu4-C	4.4	3.5	85	141	7	MOLECULE:	HEMOGLOBIN		
5:00	3kmf-A	4.4	3.1	85	141	7	MOLECULE:	HEMOGLOBIN	SUBUNIT	ALPHA
6:00	2zlv-A	4.4	3	83	141	7	MOLECULE:	HEMOGLOBIN	SUBUNIT	ALPHA
7:00	3nfe-C	4.4	3	84	142	6	MOLECULE:	HEMOGLOBIN	SUBUNIT	ALPHA-1
8:00	1y8i-A	4.4	2.9	83	141	7	MOLECULE:	HEMOGLOBIN	ALPHA C	HAINS
9:00	3ng6-C	4.4	3	84	142	6	MOLECULE:	HEMOGLOBIN	SUBUNIT	ALPHA-1
10:00	2b7h-C	4.4	3.4	86	141	8	MOLECULE:	HEMOGLOBIN	ALPHA C	HAIN
11:00	4iro-C	4.4	3.3	85	142	6	MOLECULE:	HEMOGLOBIN	SUBUNIT	ALPHA
12:00	1qpw-A	4.4	3	84	141	7	MOLECULE:	PORCINE HE	MOGLOBIN	(ALPHA SUBUNIT)
13:00	4n7p-K	4.4	3.4	85	141	7	MOLECULE:	HEMOGLOBIN	SUBUNIT	ALPHA
14:00	1ouu-B	4.4	3.5	85	146	7	MOLECULE:	HEMOGLOBIN	I	
15:00	1mdn-A	4.4	4.4	91	153	13	MOLECULE:	PROTEIN (M	YOGLOBIN	)
16:00	1o1p-D	4.4	3.3	85	146	6	MOLECULE:	HEMOGLOBIN	ALPHA C	HAIN
17:00	5vmm-C	4.4	3.1	84	141	7	MOLECULE:	HEMOGLOBIN	SUBUNIT	ALPHA
18:00	5x2u-I	4.4	3.1	84	140	7	MOLECULE:	HEMOGLOBIN	SUBUNIT	ALPHA
19:00	5x2r-I	4.4	3.1	84	140	7	MOLECULE:	HEMOGLOBIN	SUBUNIT	ALPHA
20:00	1a01-D	4.4	3.2	85	146	6	MOLECULE:	HEMOGLOBIN	(ALPHA	CHAIN)
21:00	1c40-B	4.4	3.5	85	146	9	MOLECULE:	PROTEIN (H	EMOGLOBI	N (ALPHA CHAIN))
22:00	1faw-D	4.4	3.5	85	146	9	MOLECULE:	HEMOGLOBIN	(ALPHA	SUBUNIT)
23:00	1i3e-A	4.4	3.4	86	146	6	MOLECULE:	HEMOGLOBIN	GAMMA C	HAINS
24:00:00	1yeo-B	4.4	3.5	86	146	6	MOLECULE:	HEMOGLOBIN	ALPHA C	HAIN
25:00:00	1out-B	4.4	3.6	85	146	7	MOLECULE:	HEMOGLOBIN	I	
26:00:00	1j7s-B	4.4	3.1	84	146	6	MOLECULE:	HEMOGLOBIN		
27:00:00	3pi3-B	4.4	3.9	84	151	7	MOLECULE:	HEMOGLOBIN	II	
28:00:00	5lfg-A	4.4	3.3	85	142	6	MOLECULE:	HEMOGLOBIN	SUBUNIT	ALPHA-1
29:00:00	1faw-B	4.4	3.5	85	146	9	MOLECULE:	HEMOGLOBIN	(ALPHA	SUBUNIT)
30:00:00	5x2t-G	4.4	3.4	85	140	7	MOLECULE:	HEMOGLOBIN	SUBUNIT	ALPHA
31:00:00	4nxc-A	4.4	4.4	92	153	9	MOLECULE:	MYOGLOBIN		
32:00:00	3s48-C	4.4	3.4	86	138	6	MOLECULE:	IRON-REGUL	ATED SUR	FACE DETERMINANT PROTEIN H
33:00:00	1y4b-D	4.4	3.2	85	146	6	MOLECULE:	HEMOGLOBIN	ALPHA C	HAIN
34:00:00	4n7p-E	4.4	3.2	86	141	6	MOLECULE:	HEMOGLOBIN	SUBUNIT	ALPHA
35:00:00	1y4g-D	4.4	3.5	86	146	6	MOLECULE:	HEMOGLOBIN	ALPHA C	HAIN
36:00:00	1ns6-A	4.4	3.2	85	141	7	MOLECULE:	HEMOGLOBIN	ALPHA S	UBUNIT
37:00:00	2blj-M	4.4	4.7	91	153	10	MOLECULE:	MYOGLOBIN		
38:00:00	5hy8-G	4.4	3.1	83	138	7	MOLECULE:	HEMOGLOBIN	SUBUNIT	ALPHA
39:00:00	5ni1-C	4.4	3.1	85	141	6	MOLECULE:	HEMOGLOBIN	SUBUNIT	ALPHA
40:00:00	1ouu-D	4.4	3.6	85	146	7	MOLECULE:	HEMOGLOBIN	I	

41:00:00	1yev-B	4.4	3.5	86	146	6	MOLECULE:	HEMOGLOBIN	ALPHA C	HAIN
42:00:00	1yeu-D	4.4	3.5	86	146	6	MOLECULE:	HEMOGLOBIN	ALPHA C	HAIN
43:00:00	7qu4-H	4.4	3.4	84	144	6	MOLECULE:	HEMOGLOBIN	SUBUNIT	GAMMA-2
44:00:00	5u3i-C	4.4	2.9	82	140	7	MOLECULE:	HEMOGLOBIN	SUBUNIT	ALPHA
45:00:00	1t1n-A	4.4	3.3	85	142	6	MOLECULE:	PROTEIN (H	EMOGLOBI	N)
46:00:00	1m6c-B	4.4	4.1	89	153	13	MOLECULE:	PROTEIN (M	YOGLOBIN	)
47:00:00	1yih-D	4.4	3.5	85	146	6	MOLECULE:	HEMOGLOBIN	ALPHA C	HAIN
48:00:00	5ni1-A	4.4	3.1	85	141	6	MOLECULE:	HEMOGLOBIN	SUBUNIT	ALPHA
49:00:00	1j7s-D	4.4	3.3	85	146	6	MOLECULE:	HEMOGLOBIN		
50:00:00	1rqa-B	4.4	3.5	86	146	6	MOLECULE:	HEMOGLOBIN	ALPHA C	HAIN
51:00:00	6hal-C	4.4	3.1	84	139	7	MOLECULE:	HEMOGLOBIN	SUBUNIT	ALPHA
52:00:00	1a0z-D	4.4	3.5	86	146	6	MOLECULE:	HEMOGLOBIN	(ALPHA	CHAIN)
53:00:00	3gkv-A	4.4	3.4	85	142	6	MOLECULE:	HEMOGLOBIN	SUBUNIT	ALPHA
54:00:00	1yeu-B	4.4	3.5	86	146	6	MOLECULE:	HEMOGLOBIN	ALPHA C	HAIN
55:00:00	1o1p-B	4.4	3.3	85	146	6	MOLECULE:	HEMOGLOBIN	ALPHA C	HAIN
56:00:00	1a00-D	4.4	3.1	84	146	6	MOLECULE:	HEMOGLOBIN	(ALPHA	CHAIN)
57:00:00	1v9q-A	4.4	4.6	92	154	9	MOLECULE:	MYOGLOBIN		
58:00:00	1ye2-D	4.4	3.5	86	146	6	MOLECULE:	HEMOGLOBIN	ALPHA C	HAIN
59:00:00	6sva-A	4.4	3.1	84	139	7	MOLECULE:	HEMOGLOBIN	SUBUNIT	ALPHA
60:00:00	4n7n-I	4.4	3.2	86	141	7	MOLECULE:	HEMOGLOBIN	SUBUNIT	ALPHA
61:00:00	2peg-A	4.4	3.4	85	142	6	MOLECULE:	HEMOGLOBIN	SUBUNIT	ALPHA
62:00:00	5lfg-C	4.4	3.1	84	142	6	MOLECULE:	HEMOGLOBIN	SUBUNIT	ALPHA-1
63:00:00	1y4g-B	4.4	3.5	86	146	6	MOLECULE:	HEMOGLOBIN	ALPHA C	HAIN
64:00:00	4n7p-A	4.4	3.4	85	141	7	MOLECULE:	HEMOGLOBIN	SUBUNIT	ALPHA
65:00:00	5hy8-E	4.4	3	82	137	7	MOLECULE:	HEMOGLOBIN	SUBUNIT	ALPHA
66:00:00	4odc-A	4.4	3.4	85	142	6	MOLECULE:	HEMOGLOBIN	SUBUNIT	ALPHA
67:00:00	1dm1-A	4.3	3.5	81	146	7	MOLECULE:	MYOGLOBIN		
68:00:00	6myo-D	4.3	8.1	84	102	7	MOLECULE:	SUCCINATE	DEHYDROG	ENASE [UBIQUINONE] FLAVOPROTEIN
69:00:00	5hv1-A	4.3	11.2	108	847	8	MOLECULE:	PHOSPHOENO	LPYRUVAT	E SYNTHASE
70:00:00	1f65-A	4.3	4.4	91	154	9	MOLECULE:	MYOGLOBIN		
71:00:00	6oty-A	4.3	4	86	151	6	MOLECULE:	HEMOGLOBIN	II	
72:00:00	5oj9-A	4.3	4.1	90	154	9	MOLECULE:	MYOGLOBIN		
73:00:00	7cez-A	4.3	4.3	90	153	10	MOLECULE:	MYOGLOBIN		
74:00:00	1hbs-A	4.3	3.5	86	141	5	MOLECULE:	HEMOGLOBIN	S (DEOX	Y) (ALPHA CHAIN)
75:00:00	3pt7-A	4.3	3.9	85	151	6	MOLECULE:	HEMOGLOBIN	II	
76:00:00	3pi2-A	4.3	3.9	85	151	8	MOLECULE:	HEMOGLOBIN	II	
77:00:00	8qba-A	4.3	4.3	91	156	8	MOLECULE:	MYOGLOBIN		
78:00:00	6bmg-B	4.3	4.4	91	154	8	MOLECULE:	MYOGLOBIN		
79:00:00	6zfb-h	4.3	19.6	126	771	7	MOLECULE:	DNA-DIRECT	ED RNA P	OLYMERASE SUBUNIT DELTA
80:00:00	1hrm-A	4.3	4.7	92	153	14	MOLECULE:	MYOGLOBIN		
81:00:00	3wtg-B	4.3	3.5	85	146	11	MOLECULE:	HEMOGLOBIN	SUBUNIT	ALPHA-A
82:00:00	1flp-A	4.3	3.4	78	142	5	MOLECULE:	HEMOGLOBIN	I (AQUO	MET)
83:00:00	6d45-A	4.3	4.3	90	153	10	MOLECULE:	MYOGLOBIN		
84:00:00	1moh-A	4.3	3.4	78	142	5	MOLECULE:	MONOMERIC	HEMOGLOB	IN I
85:00:00	2e2y-A	4.3	4.2	91	154	8	MOLECULE:	MYOGLOBIN		
86:00:00	5ker-C	4.3	3	83	140	8	MOLECULE:	ALPHA-GLOB	IN	
87:00:00	1shr-A	4.3	3.5	85	141	5	MOLECULE:	HEMOGLOBIN	ALPHA C	HAIN
88:00:00	4x0l-A	4.3	3.6	85	141	5	MOLECULE:	HEMOGLOBIN	SUBUNIT	ALPHA
89:00:00	1y4f-B	4.3	3.5	86	146	5	MOLECULE:	HEMOGLOBIN	ALPHA C	HAIN
90:00:00	1dxu-B	4.3	3.6	86	146	5	MOLECULE:	HEMOGLOBIN	(DEOXY)	(ALPHA CHAIN)
91:00:00	4hrt-F	4.3	3.9	88	151	9	MOLECULE:	GLOBIN-2 A	CHAIN	
92:00:00	1o1l-B	4.3	3.6	86	146	5	MOLECULE:	HEMOGLOBIN	ALPHA C	HAIN
93:00:00	4n7n-C	4.3	3.1	85	141	7	MOLECULE:	HEMOGLOBIN	SUBUNIT	ALPHA
94:00:00	1c40-A	4.3	3.5	85	141	7	MOLECULE:	PROTEIN (H	EMOGLOBI	N (ALPHA CHAIN))

95:00:00	3a59-E	4.3	3.4	86	141	7	MOLECULE:	HEMOGLOBIN	SUBUNIT	ALPHA-A
96:00:00	3k8b-C	4.3	3.5	87	141	3	MOLECULE:	HEMOGLOBIN	SUBUNIT	ALPHA-A
97:00:00	3sxm-B	4.3	3.8	93	137	8	MOLECULE:	TRANSCRIPT	IONAL RE	GULATOR, GNTR FAMILY
98:00:00	1yih-B	4.3	3.4	84	146	5	MOLECULE:	HEMOGLOBIN	ALPHA C	HAIN
99:00:00	1y8k-A	4.3	2.9	83	141	7	MOLECULE:	HEMOGLOBIN	ALPHA C	HAINS
0:00	6r2o-A	4.3	2.9	83	141	7	MOLECULE:	HEMOGLOBIN	SUBUNIT	ALPHA
1:00	1rqa-D	4.3	3.6	86	146	5	MOLECULE:	HEMOGLOBIN	ALPHA C	HAIN
2:00	1y8h-A	4.3	3	83	141	7	MOLECULE:	HEMOGLOBIN	ALPHA C	HAINS
3:00	5ker-G	4.3	3.1	84	140	8	MOLECULE:	ALPHA-GLOB	IN	
4:00	2zlx-C	4.3	3	84	139	6	MOLECULE:	HEMOGLOBIN	SUBUNIT	ALPHA
5:00	2hbs-H	4.3	3.4	84	146	5	MOLECULE:	HEMOGLOBIN	S (DEOX	Y), ALPHA CHAIN
6:00	2r80-A	4.3	3.2	86	141	8	MOLECULE:	HEMOGLOBIN	SUBUNIT	ALPHA-A
7:00	3fh9-A	4.3	3.7	86	141	6	MOLECULE:	HEMOGLOBIN	ALPHA C	HAIN
8:00	7pcf-B	4.3	3.3	84	146	8	MOLECULE:	HEMOGLOBIN	SUBUNIT	ALPHA
9:00	3mju-A	4.3	3.4	86	141	8	MOLECULE:	HEMOGLOBIN	SUBUNIT	ALPHA-A
10:00	2pgh-C	4.3	3.2	85	141	7	MOLECULE:	HEMOGLOBIN	(AQUO M	ET) (ALPHA CHAIN)
11:00	3ic0-C	4.3	3	83	141	7	MOLECULE:	HEMOGLOBIN	SUBUNIT	ALPHA
12:00	1y4p-D	4.3	3.6	86	146	5	MOLECULE:	HEMOGLOBIN	ALPHA C	HAIN
13:00	7k4m-E	4.3	3.5	85	142	5	MOLECULE:	HEMOGLOBIN	SUBUNIT	ALPHA
14:00	1o1n-D	4.3	3.6	86	146	5	MOLECULE:	HEMOGLOBIN	ALPHA C	HAIN
15:00	2b7h-A	4.3	3.5	86	141	9	MOLECULE:	HEMOGLOBIN	ALPHA C	HAIN
16:00	4g51-C	4.3	3.4	85	142	6	MOLECULE:	HEMOGLOBIN	SUBUNIT	ALPHA
17:00	1y46-B	4.3	3.5	86	146	5	MOLECULE:	HEMOGLOBIN	ALPHA C	HAIN
18:00	1o1n-B	4.3	3.6	86	146	5	MOLECULE:	HEMOGLOBIN	ALPHA C	HAIN
19:00	3gqr-A	4.3	3.5	87	141	8	MOLECULE:	HEMOGLOBIN	SUBUNIT	ALPHA
20:00	5ker-A	4.3	3.6	85	140	6	MOLECULE:	ALPHA-GLOB	IN	
21:00	3eu1-A	4.3	3.6	87	141	6	MOLECULE:	HEMOGLOBIN	SUBUNIT	ALPHA-1/2
22:00	3gqp-A	4.3	3.2	85	141	9	MOLECULE:	HEMOGLOBIN	SUBUNIT	ALPHA
23:00	1ye2-B	4.3	3.5	86	146	5	MOLECULE:	HEMOGLOBIN	ALPHA C	HAIN
24:00:00	4yu3-A	4.3	3.6	85	141	7	MOLECULE:	HEMOGLOBIN		
25:00:00	1yg5-B	4.3	3.5	86	146	5	MOLECULE:	HEMOGLOBIN	ALPHA C	HAIN
26:00:00	2dhb-A	4.3	2.9	83	141	6	MOLECULE:	HEMOGLOBIN	(DEOXY)	(ALPHA CHAIN)
27:00:00	3mjp-A	4.3	3.4	87	141	6	MOLECULE:	HEMOGLOBIN	SUBUNIT	ALPHA-A
28:00:00	2pgh-A	4.3	3.1	84	141	7	MOLECULE:	HEMOGLOBIN	(AQUO M	ET) (ALPHA CHAIN)
29:00:00	1dxu-D	4.3	3.6	86	146	5	MOLECULE:	HEMOGLOBIN	(DEOXY)	(ALPHA CHAIN)
30:00:00	1y8h-C	4.3	3	83	141	7	MOLECULE:	HEMOGLOBIN	ALPHA C	HAINS
31:00:00	1y4f-D	4.3	3.6	86	146	5	MOLECULE:	HEMOGLOBIN	ALPHA C	HAIN
32:00:00	2ri4-A	4.3	3	84	139	7	MOLECULE:	HEMOGLOBIN	SUBUNIT	ALPHA-1/2
33:00:00	1o1l-D	4.3	3.6	86	146	5	MOLECULE:	HEMOGLOBIN	ALPHA C	HAIN
34:00:00	1s0h-A	4.3	3.1	84	141	8	MOLECULE:	HEMOGLOBIN	ALPHA C	HAIN
35:00:00	1fdh-G	4.3	3.6	86	146	5	MOLECULE:	HEMOGLOBIN	F (DEOX	Y) (ALPHA CHAIN)
36:00:00	1hbs-C	4.3	3.1	85	141	6	MOLECULE:	HEMOGLOBIN	S (DEOX	Y) (ALPHA CHAIN)
37:00:00	1y4p-B	4.3	3.5	86	146	5	MOLECULE:	HEMOGLOBIN	ALPHA C	HAIN
38:00:00	1dxc-A	4.3	4.7	92	154	9	MOLECULE:	MYOGLOBIN		
39:00:00	1yie-D	4.3	3.5	85	146	6	MOLECULE:	HEMOGLOBIN	ALPHA C	HAIN
40:00:00	1a9w-F	4.3	3.5	85	145	5	MOLECULE:	HEMOGLOBIN	(ALPHA	CHAIN)
41:00:00	6zmx-B	4.3	3.5	85	146	8	MOLECULE:	HEMOGLOBIN	SUBUNIT	ALPHA-A
42:00:00	1a9w-E	4.3	3.5	85	145	5	MOLECULE:	HEMOGLOBIN	(ALPHA	CHAIN)
43:00:00	3wr1-B	4.3	3.5	86	146	9	MOLECULE:	HEMOGLOBIN	SUBUNIT	ALPHA-A
44:00:00	1naz-A	4.3	4.7	92	154	9	MOLECULE:	MYOGLOBIN		
45:00:00	3hf4-E	4.3	3	84	141	7	MOLECULE:	HEMOGLOBIN	SUBUNIT	ALPHA-1/2
46:00:00	1yev-D	4.3	3.5	86	146	6	MOLECULE:	HEMOGLOBIN	ALPHA C	HAIN
47:00:00	3nml-A	4.3	4.5	91	154	9	MOLECULE:	MYOGLOBIN		
48:00:00	1mdn-B	4.3	4.3	89	153	16	MOLECULE:	PROTEIN (M	YOGLOBIN	)

49:00:00	1yeo-D	4.3	3.5	86	146	6	MOLECULE:	HEMOGLOBIN	ALPHA C	HAIN
50:00:00	1gli-D	4.3	3.3	85	146	6	MOLECULE:	DEOXYHEMOG	LOBIN	
51:00:00	5me2-C	4.3	2.9	83	140	7	MOLECULE:	HEMOGLOBIN	SUBUNIT	ALPHA
52:00:00	4hrr-G	4.3	3.3	87	149	3	MOLECULE:	GLOBIN-2 A	CHAIN	
53:00:00	1yie-B	4.3	3.4	84	146	6	MOLECULE:	HEMOGLOBIN	ALPHA C	HAIN
54:00:00	1hbr-D	4.3	3.6	86	146	8	MOLECULE:	PROTEIN (H	EMOGLOBI	N D)
55:00:00	1yg5-D	4.3	3.5	86	146	6	MOLECULE:	HEMOGLOBIN	ALPHA C	HAIN
56:00:00	2hbs-B	4.3	3.1	83	146	6	MOLECULE:	HEMOGLOBIN	S (DEOX	Y), ALPHA CHAIN
57:00:00	1y4v-D	4.3	3.5	86	146	6	MOLECULE:	HEMOGLOBIN	ALPHA C	HAIN
58:00:00	1o1m-A	4.3	9.7	102	282	9	MOLECULE:	HEMOGLOBIN	ALPHA C	HAIN
59:00:00	2cmm-A	4.3	4.4	91	153	9	MOLECULE:	MYOGLOBIN		
60:00:00	3k8b-D	4.3	3.5	86	146	8	MOLECULE:	HEMOGLOBIN	SUBUNIT	ALPHA-A
61:00:00	5me2-A	4.3	2.9	83	140	7	MOLECULE:	HEMOGLOBIN	SUBUNIT	ALPHA
62:00:00	1s5y-A	4.3	3.5	85	142	6	MOLECULE:	HEMOGLOBIN	ALPHA C	HAIN
63:00:00	1yeq-D	4.3	3.5	86	146	6	MOLECULE:	HEMOGLOBIN	ALPHA C	HAIN
64:00:00	1dxt-B	4.3	3.1	83	147	6	MOLECULE:	HEMOGLOBIN	(DEOXY)	(ALPHA CHAIN)
65:00:00	8puq-A	4.3	3.2	85	139	6	MOLECULE:	HEMOGLOBIN	SUBUNIT	ALPHA
66:00:00	1y0t-D	4.3	3.5	86	146	6	MOLECULE:	HEMOGLOBIN	ALPHA C	HAIN
67:00:00	1hbs-H	4.3	3.4	86	146	6	MOLECULE:	HEMOGLOBIN	S (DEOX	Y) (ALPHA CHAIN)
68:00:00	1co8-A	4.3	4.5	92	154	8	MOLECULE:	PROTEIN (M	YOGLOBIN	)
69:00:00	1y0t-B	4.3	3	83	146	6	MOLECULE:	HEMOGLOBIN	ALPHA C	HAIN
70:00:00	1y01-B	4.3	3.2	85	126	7	MOLECULE:	ALPHA-HEMO	GLOBIN S	TABILIZING PROTEIN
71:00:00	2hbs-F	4.3	2.9	82	146	6	MOLECULE:	HEMOGLOBIN	S (DEOX	Y), ALPHA CHAIN
72:00:00	2r80-B	4.3	3.6	86	146	8	MOLECULE:	HEMOGLOBIN	SUBUNIT	ALPHA-A
73:00:00	1y4b-B	4.3	3.2	85	146	6	MOLECULE:	HEMOGLOBIN	ALPHA C	HAIN
74:00:00	3pi1-A	4.3	3.9	86	151	7	MOLECULE:	HEMOGLOBIN	II	
75:00:00	1s5x-A	4.3	3.1	84	142	6	MOLECULE:	HEMOGLOBIN	ALPHA C	HAIN
76:00:00	5x2r-E	4.3	3.1	84	140	7	MOLECULE:	HEMOGLOBIN	SUBUNIT	ALPHA
77:00:00	3ciu-C	4.3	3	83	141	7	MOLECULE:	HEMOGLOBIN	SUBUNIT	ALPHA
78:00:00	1ygd-B	4.3	3.5	86	146	6	MOLECULE:	HEMOGLOBIN	ALPHA C	HAIN
79:00:00	1y2z-B	4.3	3.1	83	146	6	MOLECULE:	HEMOGLOBIN	ALPHA C	HAIN
80:00:00	2qmb-B	4.3	3.5	85	146	8	MOLECULE:	HEMOGLOBIN	SUBUNIT	ALPHA-A
81:00:00	1gli-B	4.3	3.3	85	146	6	MOLECULE:	DEOXYHEMOG	LOBIN	
82:00:00	2rao-C	4.3	3	83	141	8	MOLECULE:	HEMOGLOBIN	SUBUNIT	ALPHA-1/2
83:00:00	1y35-B	4.3	3	83	146	6	MOLECULE:	HEMOGLOBIN	ALPHA C	HAIN
84:00:00	1y46-D	4.3	3.2	85	146	6	MOLECULE:	HEMOGLOBIN	ALPHA C	HAIN
85:00:00	1y7c-D	4.3	3.5	86	146	6	MOLECULE:	HEMOGLOBIN	ALPHA C	HAIN
86:00:00	1a0u-D	4.3	3.5	86	146	6	MOLECULE:	HEMOGLOBIN	(ALPHA	CHAIN)
87:00:00	5x2t-A	4.3	3.5	85	140	7	MOLECULE:	HEMOGLOBIN	SUBUNIT	ALPHA
88:00:00	2olp-A	4.3	3.9	84	151	6	MOLECULE:	HEMOGLOBIN	II	
89:00:00	1y4v-B	4.3	3	83	146	6	MOLECULE:	HEMOGLOBIN	ALPHA C	HAIN
90:00:00	3pi3-A	4.3	3.9	85	151	7	MOLECULE:	HEMOGLOBIN	II	
91:00:00	3at5-B	4.3	3.5	86	146	9	MOLECULE:	ALPHAA-GLO	BIN	
92:00:00	1a4f-B	4.3	3.5	85	146	9	MOLECULE:	HEMOGLOBIN	(ALPHA	CHAIN)
93:00:00	7wb4-b	4.2	24	137	636	4	MOLECULE:	OUTER NUP1	33	
94:00:00	8fef-I	4.2	10.3	97	253	6	MOLECULE:	VIRULENCE	FACTOR M	CE FAMILY PROTEIN
95:00:00	6u8y-b	4.2	6.2	70	79	7	MOLECULE:	MONOVALENT	CATION/	H+ ANTIPORTER SUBUNIT E
96:00:00	3hen-A	4.2	4.2	90	153	12	MOLECULE:	MYOGLOBIN		
97:00:00	1ebt-A	4.2	3.4	78	142	5	MOLECULE:	HEMOGLOBIN		
98:00:00	1hv4-A	4.2	3.6	85	141	7	MOLECULE:	HEMOGLOBIN	ALPHA-A	CHAIN
99:00:00	7vuc-A	4.2	4.4	91	153	9	MOLECULE:	MYOGLOBIN		
0:00	2in4-A	4.2	4.1	90	153	12	MOLECULE:	MYOGLOBIN		
1:00	3hep-A	4.2	4.3	90	153	14	MOLECULE:	MYOGLOBIN		
2:00	5ojc-A	4.2	4.2	91	154	9	MOLECULE:	MYOGLOBIN		

3:00	1hv4-C	4.2	3.6	85	141	7	MOLECULE:	HEMOGLOBIN	ALPHA-A	CHAIN
4:00	4pnj-A	4.2	4.4	91	154	9	MOLECULE:	MYOGLOBIN		
5:00	3rjn-B	4.2	4.2	90	152	12	MOLECULE:	MYOGLOBIN		
6:00	3u3e-A	4.2	4.4	90	153	10	MOLECULE:	MYOGLOBIN		
7:00	3heo-A	4.2	4.2	90	153	14	MOLECULE:	MYOGLOBIN		
8:00	6jp1-A	4.2	4.5	91	151	9	MOLECULE:	MYOGLOBIN		
9:00	3g52-A	4.2	3.3	85	145	8	MOLECULE:	GLOBIN-1		
10:00	7ylk-A	4.2	4.6	91	153	8	MOLECULE:	MYOGLOBIN		
11:00	1jp8-A	4.2	3.9	88	152	9	MOLECULE:	MYOGLOBIN		
12:00	1y35-D	4.2	3.6	86	146	5	MOLECULE:	HEMOGLOBIN	ALPHA C	HAIN
13:00	5urc-A	4.2	4.2	87	141	7	MOLECULE:	HEMOGLOBIN	SUBUNIT	ALPHA
14:00	3hc9-A	4.2	4.3	90	153	14	MOLECULE:	MYOGLOBIN		
15:00	1gbu-B	4.2	3.6	86	146	5	MOLECULE:	HEMOGLOBIN		
16:00	1y0w-D	4.2	3.6	86	146	5	MOLECULE:	HEMOGLOBIN	ALPHA C	HAIN
17:00	1a00-B	4.2	3.5	84	146	5	MOLECULE:	HEMOGLOBIN	(ALPHA	CHAIN)
18:00	6hbi-B	4.2	3.3	85	145	8	MOLECULE:	HEMOGLOBIN		
19:00	3hrw-A	4.2	3.1	85	141	7	MOLECULE:	HEMOGLOBIN	SUBUNIT	ALPHA
20:00	3k8b-A	4.2	3.5	85	141	6	MOLECULE:	HEMOGLOBIN	SUBUNIT	ALPHA-A
21:00	3gdj-C	4.2	3.6	86	141	7	MOLECULE:	HEMOGLOBIN	SUBUNIT	ALPHA
22:00	4sdh-B	4.2	3.3	84	145	11	MOLECULE:	HEMOGLOBIN	I (DEOX	Y)
23:00	3uhs-B	4.2	3.2	84	145	11	MOLECULE:	GLOBIN-1		
24:00:00	2zlx-A	4.2	3.2	85	140	6	MOLECULE:	HEMOGLOBIN	SUBUNIT	ALPHA
25:00:00	2zlw-C	4.2	3.1	83	141	7	MOLECULE:	HEMOGLOBIN	SUBUNIT	ALPHA
26:00:00	1nwn-B	4.2	3.3	85	145	9	MOLECULE:	GLOBIN I		
27:00:00	3ui0-B	4.2	3.4	84	145	11	MOLECULE:	GLOBIN-1		
28:00:00	1jp9-A	4.2	4	90	151	9	MOLECULE:	MYOGLOBIN		
29:00:00	1a6k-A	4.2	4.2	89	151	9	MOLECULE:	MYOGLOBIN		
30:00:00	3uhw-B	4.2	3.3	85	145	8	MOLECULE:	GLOBIN-1		
31:00:00	3uhx-B	4.2	3.3	85	145	8	MOLECULE:	GLOBIN-1		
32:00:00	3lqd-A	4.2	3	83	141	8	MOLECULE:	HEMOGLOBIN	SUBUNIT	ALPHA
33:00:00	1hba-B	4.2	3.4	84	146	5	MOLECULE:	HEMOGLOBIN	ROTHSCH	ILD (DEOXY) (ALPHA CHAIN)
34:00:00	4hrt-A	4.2	3.7	88	149	5	MOLECULE:	GLOBIN-2 A	CHAIN	
35:00:00	2zfb-A	4.2	3.1	85	141	8	MOLECULE:	HEMOGLOBIN	SUBUNIT	ALPHA
36:00:00	1gbu-D	4.2	3.5	84	146	5	MOLECULE:	HEMOGLOBIN		
37:00:00	3g4y-A	4.2	3.4	86	145	9	MOLECULE:	GLOBIN-1		
38:00:00	1hba-D	4.2	3.5	84	146	5	MOLECULE:	HEMOGLOBIN	ROTHSCH	ILD (DEOXY) (ALPHA CHAIN)
39:00:00	4hbi-B	4.2	3.3	84	145	11	MOLECULE:	HEMOGLOBIN		
40:00:00	3uhv-B	4.2	3.3	84	145	11	MOLECULE:	GLOBIN-1		
41:00:00	6jp1-B	4.2	4.2	89	151	10	MOLECULE:	MYOGLOBIN		
42:00:00	6z4r-A	4.2	4.6	91	154	9	MOLECULE:	MYOGLOBIN		
43:00:00	3uhr-B	4.2	3.4	84	145	11	MOLECULE:	GLOBIN-1		
44:00:00	3o89-A	4.2	4.7	91	153	10	MOLECULE:	MYOGLOBIN		
45:00:00	3vre-B	4.2	3.3	84	146	7	MOLECULE:	HEMOGLOBIN	SUBUNIT	ALPHA
46:00:00	1jzk-B	4.2	3.3	84	145	12	MOLECULE:	GLOBIN I -	ARK SHE	LL
47:00:00	5jgg-B	4.2	3.7	85	144	7	MOLECULE:	ALPHA CHAI	N	
48:00:00	3uhi-A	4.2	3.3	84	145	11	MOLECULE:	GLOBIN-1		
49:00:00	1yeq-B	4.2	3.3	85	146	6	MOLECULE:	HEMOGLOBIN	ALPHA C	HAIN
50:00:00	1jzm-B	4.2	3.3	84	145	11	MOLECULE:	GLOBIN I -	ARK SHE	LL
51:00:00	2grf-B	4.2	3.1	83	145	11	MOLECULE:	GLOBIN-1		
52:00:00	3uhz-B	4.2	3.3	84	145	11	MOLECULE:	GLOBIN-1		
53:00:00	2zfb-B	4.2	3.6	85	146	8	MOLECULE:	HEMOGLOBIN	SUBUNIT	ALPHA
54:00:00	3uhn-B	4.2	3.4	84	145	11	MOLECULE:	GLOBIN-1		
55:00:00	3uht-B	4.2	3.4	84	145	12	MOLECULE:	GLOBIN-1		
56:00:00	7xgy-C	4.2	3.5	84	141	7	MOLECULE:	HEMOGLOBIN	SUBUNIT	ALPHA

57:00:00	7dy4-B	4.2	3.2	84	146	6	MOLECULE:	HEMOGLOBIN	SUBUNIT	ALPHA
58:00:00	7xgy-A	4.2	3.6	84	141	7	MOLECULE:	HEMOGLOBIN	SUBUNIT	ALPHA
59:00:00	3eok-B	4.2	3.5	85	146	8	MOLECULE:	HEMOGLOBIN	SUBUNIT	ALPHA-A
60:00:00	1jzk-A	4.2	3.3	85	145	9	MOLECULE:	GLOBIN I -	ARK SHE	LL
61:00:00	7pcq-B	4.2	3.5	84	146	6	MOLECULE:	HEMOGLOBIN	SUBUNIT	ALPHA
62:00:00	1hv4-H	4.2	3.5	85	146	9	MOLECULE:	HEMOGLOBIN	ALPHA-A	CHAIN
63:00:00	1nwi-A	4.2	3.3	85	145	8	MOLECULE:	GLOBIN I		
64:00:00	1b0b-A	4.2	3.4	78	142	5	MOLECULE:	HEMOGLOBIN		
65:00:00	3uhy-B	4.2	3.3	84	145	11	MOLECULE:	GLOBIN-1		
66:00:00	4iro-A	4.2	3.3	85	142	6	MOLECULE:	HEMOGLOBIN	SUBUNIT	ALPHA
67:00:00	3g4q-A	4.2	3.4	85	145	8	MOLECULE:	GLOBIN-1		
68:00:00	1y0w-B	4.2	3.1	83	146	6	MOLECULE:	HEMOGLOBIN	ALPHA C	HAIN
69:00:00	4hrr-C	4.2	3.7	87	149	3	MOLECULE:	GLOBIN-2 A	CHAIN	
70:00:00	7pch-B	4.2	3.4	84	146	6	MOLECULE:	HEMOGLOBIN	SUBUNIT	ALPHA
71:00:00	3a59-F	4.2	3.5	85	146	9	MOLECULE:	HEMOGLOBIN	SUBUNIT	ALPHA-A
72:00:00	1y7c-B	4.2	3.4	84	146	6	MOLECULE:	HEMOGLOBIN	ALPHA C	HAIN
73:00:00	1hv4-B	4.2	3.5	85	146	9	MOLECULE:	HEMOGLOBIN	ALPHA-A	CHAIN
74:00:00	1hv4-F	4.2	3.6	86	146	9	MOLECULE:	HEMOGLOBIN	ALPHA-A	CHAIN
75:00:00	7dy4-F	4.2	2.9	82	146	6	MOLECULE:	HEMOGLOBIN	SUBUNIT	ALPHA
76:00:00	3fs4-D	4.2	3.5	85	146	9	MOLECULE:	HEMOGLOBIN	SUBUNIT	ALPHA-A
77:00:00	1i3e-B	4.2	3.5	86	146	6	MOLECULE:	HEMOGLOBIN	GAMMA C	HAINS
78:00:00	1hds-A	4.2	3.4	85	141	6	MOLECULE:	HEMOGLOBIN	S (DEOX	Y) (ALPHA CHAIN)
79:00:00	4ij2-A	4.2	3.5	84	138	5	MOLECULE:	HEMOGLOBIN	SUBUNIT	ALPHA
80:00:00	7dy3-F	4.2	3.1	83	146	6	MOLECULE:	HEMOGLOBIN	SUBUNIT	ALPHA
81:00:00	3mju-B	4.2	3.5	85	146	8	MOLECULE:	HEMOGLOBIN	SUBUNIT	ALPHA-A
82:00:00	3bcq-B	4.2	3.6	86	146	7	MOLECULE:	ALPHA-CHAI	N HEMOGL	OBIN
83:00:00	8egi-D	4.2	3.6	86	146	6	MOLECULE:	HEMOGLOBIN	SUBUNIT	ALPHA
84:00:00	3uhu-B	4.2	3.4	84	145	11	MOLECULE:	GLOBIN-1		
85:00:00	3uhk-A	4.2	3.2	84	145	8	MOLECULE:	GLOBIN-1		
86:00:00	4ij2-C	4.2	3.5	84	138	5	MOLECULE:	HEMOGLOBIN	SUBUNIT	ALPHA
87:00:00	3g4v-A	4.2	3.3	84	145	11	MOLECULE:	GLOBIN-1		
88:00:00	6xdt-D	4.2	3.5	86	146	6	MOLECULE:	HEMOGLOBIN	SUBUNIT	ALPHA
89:00:00	3gqp-B	4.2	3.5	85	145	7	MOLECULE:	HEMOGLOBIN	SUBUNIT	ALPHA
90:00:00	7dy4-D	4.2	3	82	146	6	MOLECULE:	HEMOGLOBIN	SUBUNIT	ALPHA
91:00:00	3ovu-C	4.2	3.6	86	139	7	MOLECULE:	ALPHA-HEMO	GLOBIN-S	TABILIZING PROTEIN
92:00:00	7dds-A	4.2	4.5	92	154	12	MOLECULE:	ANCESTRAL	MYOGLOBI	N AMBSP
93:00:00	2zlw-A	4.2	3	83	141	7	MOLECULE:	HEMOGLOBIN	SUBUNIT	ALPHA
94:00:00	3vre-D	4.2	3.5	84	146	7	MOLECULE:	HEMOGLOBIN	SUBUNIT	ALPHA
95:00:00	3dhr-D	4.2	3.6	85	145	8	MOLECULE:	HEMOGLOBIN	SUBUNIT	ALPHA-A
96:00:00	5x2s-C	4.2	3.1	84	140	6	MOLECULE:	HEMOGLOBIN	SUBUNIT	ALPHA
97:00:00	1a0z-B	4.2	3.1	83	146	6	MOLECULE:	HEMOGLOBIN	(ALPHA	CHAIN)
98:00:00	3uhk-D	4.2	3.3	84	145	11	MOLECULE:	GLOBIN-1		
99:00:00	1hlb-A	4.1	4.4	91	157	11	MOLECULE:	HEMOGLOBIN	(DEOXY)	
0:00	6wb9-6	4.1	3.8	74	98	4	MOLECULE:	ENDOPLASMI	C RETICU	LUM MEMBRANE PROTEIN COMPLEX SU
1:00	7ol3-B	4.1	12.6	88	425	5	MOLECULE:	ATLASTIN-1		
2:00	1mwc-B	4.1	4.3	90	153	13	MOLECULE:	PROTEIN (M	YOGLOBIN	)
3:00	7ddr-A	4.1	4.3	91	154	13	MOLECULE:	ANCESTRAL	MYOGLOBI	N AMBSP
4:00	1m6m-B	4.1	4.3	90	153	13	MOLECULE:	PROTEIN (M	YOGLOBIN	)
5:00	4sdh-A	4.1	3.3	84	145	8	MOLECULE:	HEMOGLOBIN	I (DEOX	Y)
6:00	1hv4-G	4.1	3.6	85	141	7	MOLECULE:	HEMOGLOBIN	ALPHA-A	CHAIN
7:00	4hrr-B	4.1	3.9	88	151	9	MOLECULE:	GLOBIN-2 A	CHAIN	
8:00	1hbi-A	4.1	3.3	84	145	8	MOLECULE:	HEMOGLOBIN	(OXY)	
9:00	2z85-A	4.1	3.3	84	145	8	MOLECULE:	GLOBIN-1		
10:00	3g53-A	4.1	3.3	85	145	9	MOLECULE:	GLOBIN-1		

11:00	3uhz-A	4.1	3.3	84	145	8	MOLECULE:	GLOBIN-1		
12:00	3g4r-A	4.1	3.3	84	145	8	MOLECULE:	GLOBIN-1		
13:00	1nwi-D	4.1	3.3	84	145	8	MOLECULE:	GLOBIN I		
14:00	3lqd-C	4.1	3	83	141	8	MOLECULE:	HEMOGLOBIN	SUBUNIT	ALPHA
15:00	2qmb-C	4.1	3.5	85	141	6	MOLECULE:	HEMOGLOBIN	SUBUNIT	ALPHA-A
16:00	3uhw-A	4.1	3.3	84	145	11	MOLECULE:	GLOBIN-1		
17:00	4hrt-H	4.1	3.4	87	148	9	MOLECULE:	GLOBIN-2 A	CHAIN	
18:00	1nwi-C	4.1	3.2	84	145	8	MOLECULE:	GLOBIN I		
19:00	3g4u-A	4.1	3.3	85	145	9	MOLECULE:	GLOBIN-1		
20:00	3ugy-A	4.1	3.3	85	145	9	MOLECULE:	GLOBIN-1		
21:00	3uhe-A	4.1	3.3	84	145	8	MOLECULE:	GLOBIN-1		
22:00	2auq-A	4.1	3.3	84	145	8	MOLECULE:	GLOBIN I		
23:00	3uh7-A	4.1	3.3	85	145	9	MOLECULE:	GLOBIN-1		
24:00:00	2r4x-B	4.1	3.3	84	145	8	MOLECULE:	GLOBIN-1		
25:00:00	2r4y-B	4.1	3.3	84	145	8	MOLECULE:	GLOBIN-1		
26:00:00	1hv4-E	4.1	3.6	85	141	7	MOLECULE:	HEMOGLOBIN	ALPHA-A	CHAIN
27:00:00	6hbi-A	4.1	3.3	84	145	8	MOLECULE:	HEMOGLOBIN		
28:00:00	3uhv-A	4.1	3.3	84	145	8	MOLECULE:	GLOBIN-1		
29:00:00	4n7o-C	4.1	3	83	141	6	MOLECULE:	HEMOGLOBIN	SUBUNIT	ALPHA
30:00:00	3ui0-A	4.1	3.3	84	145	8	MOLECULE:	GLOBIN-1		
31:00:00	1nxf-B	4.1	3.5	85	145	9	MOLECULE:	GLOBIN I		
32:00:00	3uhg-A	4.1	3.3	84	145	8	MOLECULE:	GLOBIN-1		
33:00:00	3uhn-A	4.1	3.3	84	145	8	MOLECULE:	GLOBIN-1		
34:00:00	3uhu-A	4.1	3.3	84	145	8	MOLECULE:	GLOBIN-1		
35:00:00	3uhq-B	4.1	3.3	84	145	8	MOLECULE:	GLOBIN-1		
36:00:00	2z85-B	4.1	3.5	85	145	9	MOLECULE:	GLOBIN-1		
37:00:00	4hrr-F	4.1	3.7	87	151	10	MOLECULE:	GLOBIN-2 A	CHAIN	
38:00:00	3g4w-A	4.1	3.6	87	145	9	MOLECULE:	GLOBIN-1		
39:00:00	3sdh-A	4.1	3.3	84	145	8	MOLECULE:	HEMOGLOBIN	I (CARB	ONMONOXY)
40:00:00	1faw-A	4.1	3.6	85	141	7	MOLECULE:	HEMOGLOBIN	(ALPHA	SUBUNIT)
41:00:00	2av3-B	4.1	3.5	85	145	8	MOLECULE:	GLOBIN I		
42:00:00	2grf-A	4.1	3.3	84	145	8	MOLECULE:	GLOBIN-1		
43:00:00	2z8a-A	4.1	3.6	86	145	8	MOLECULE:	GLOBIN-1		
44:00:00	5xkv-A	4.1	3.6	80	153	10	MOLECULE:	MYOGLOBIN		
45:00:00	1jzk-D	4.1	3.3	84	145	8	MOLECULE:	GLOBIN I -	ARK SHE	LL
46:00:00	2auo-A	4.1	3.3	84	145	8	MOLECULE:	GLOBIN I		
47:00:00	1jwn-A	4.1	3.3	84	145	8	MOLECULE:	GLOBIN I -	ARK SHE	LL
48:00:00	3mjp-C	4.1	3.9	88	141	2	MOLECULE:	HEMOGLOBIN	SUBUNIT	ALPHA-A
49:00:00	1jzm-A	4.1	3.3	84	145	8	MOLECULE:	GLOBIN I -	ARK SHE	LL
50:00:00	3uhr-A	4.1	3.2	82	145	11	MOLECULE:	GLOBIN-1		
51:00:00	2av0-A	4.1	3.5	85	145	8	MOLECULE:	GLOBIN I		
52:00:00	2aup-B	4.1	3.4	84	145	11	MOLECULE:	GLOBIN I		
53:00:00	3uhs-A	4.1	3.3	84	145	8	MOLECULE:	GLOBIN-1		
54:00:00	4hbi-A	4.1	3.3	84	145	8	MOLECULE:	HEMOGLOBIN		
55:00:00	3qob-A	4.1	3.4	85	145	8	MOLECULE:	GLOBIN-1		
56:00:00	3uh6-A	4.1	3.3	85	145	9	MOLECULE:	GLOBIN-1		
57:00:00	3a59-D	4.1	3.5	85	146	9	MOLECULE:	HEMOGLOBIN	SUBUNIT	ALPHA-A
58:00:00	2aur-B	4.1	3.4	84	145	11	MOLECULE:	GLOBIN I		
59:00:00	1jwn-B	4.1	3.2	83	145	12	MOLECULE:	GLOBIN I -	ARK SHE	LL
60:00:00	3g46-B	4.1	3.2	83	146	11	MOLECULE:	GLOBIN-1		
61:00:00	7xgy-B	4.1	3.4	84	146	7	MOLECULE:	HEMOGLOBIN	SUBUNIT	ALPHA
62:00:00	2ri4-K	4.1	3	84	138	6	MOLECULE:	HEMOGLOBIN	SUBUNIT	ALPHA-1/2
63:00:00	3g4u-B	4.1	3.4	84	145	8	MOLECULE:	GLOBIN-1		
64:00:00	1qpw-B	4.1	3.6	86	146	5	MOLECULE:	PORCINE HE	MOGLOBIN	(ALPHA SUBUNIT)

65:00:00	7xgy-D	4.1	3.4	84	146	7	MOLECULE:	HEMOGLOBIN	SUBUNIT	ALPHA
66:00:00	3g4w-B	4.1	3.4	85	145	8	MOLECULE:	GLOBIN-1		
67:00:00	7ud8-D	4.1	3.5	84	146	6	MOLECULE:	HEMOGLOBIN	SUBUNIT	ALPHA
68:00:00	3uhk-C	4.1	3.3	85	145	8	MOLECULE:	GLOBIN-1		
69:00:00	1fhj-B	4.1	3.5	84	146	7	MOLECULE:	HEMOGLOBIN	(ALPHA	CHAIN)
70:00:00	2qmb-D	4.1	3.5	85	146	8	MOLECULE:	HEMOGLOBIN	SUBUNIT	ALPHA-A
71:00:00	3uhy-A	4.1	3.4	85	146	8	MOLECULE:	GLOBIN-1		
72:00:00	3fh9-B	4.1	3.6	86	146	6	MOLECULE:	HEMOGLOBIN	ALPHA C	HAIN
73:00:00	5e83-D	4.1	3.4	84	146	6	MOLECULE:	HEMOGLOBIN	SUBUNIT	ALPHA
74:00:00	2pgh-D	4.1	3.5	85	146	5	MOLECULE:	HEMOGLOBIN	(AQUO M	ET) (ALPHA CHAIN)
75:00:00	8dov-B	4.1	3.5	84	146	6	MOLECULE:	HEMOGLOBIN	SUBUNIT	ALPHA
76:00:00	8fdm-B	4.1	3.4	84	146	6	MOLECULE:	HEMOGLOBIN	SUBUNIT	ALPHA
77:00:00	7dy3-H	4.1	3.5	84	146	6	MOLECULE:	HEMOGLOBIN	SUBUNIT	ALPHA
78:00:00	5hbi-A	4.1	3.4	84	145	8	MOLECULE:	HEMOGLOBIN		
79:00:00	3gou-D	4.1	3.5	84	146	7	MOLECULE:	HEMOGLOBIN	SUBUNIT	ALPHA
80:00:00	7hbi-A	4.1	3.3	84	145	8	MOLECULE:	HEMOGLOBIN		
81:00:00	3g46-A	4.1	3.3	84	146	8	MOLECULE:	GLOBIN-1		
82:00:00	3uh5-A	4.1	3.3	84	145	8	MOLECULE:	GLOBIN-1		
83:00:00	7ddu-C	4.1	4.6	92	154	10	MOLECULE:	MYOGLOBIN		
84:00:00	2r1h-B	4.1	3.6	86	147	6	MOLECULE:	HEMOGLOBIN	SUBUNIT	ALPHA-4
85:00:00	3uhb-A	4.1	3.4	85	145	8	MOLECULE:	GLOBIN-1		
86:00:00	3uh3-A	4.1	3.4	85	145	9	MOLECULE:	GLOBIN-1		
87:00:00	7jy1-D	4.1	3.5	84	146	6	MOLECULE:	HEMOGLOBIN	SUBUNIT	ALPHA
88:00:00	2grh-A	4.1	3.3	84	145	8	MOLECULE:	GLOBIN-1		
89:00:00	3uhq-A	4.1	3.3	84	145	8	MOLECULE:	GLOBIN-1		
90:00:00	3hrw-C	4.1	3.3	86	141	7	MOLECULE:	HEMOGLOBIN	SUBUNIT	ALPHA
91:00:00	6xe7-D	4.1	3.6	86	146	6	MOLECULE:	HEMOGLOBIN	SUBUNIT	ALPHA
92:00:00	8fdm-D	4.1	3.4	84	146	6	MOLECULE:	HEMOGLOBIN	SUBUNIT	ALPHA
93:00:00	5e6e-B	4.1	3.4	84	146	6	MOLECULE:	HEMOGLOBIN	SUBUNIT	ALPHA
94:00:00	1jwn-D	4.1	3.5	86	145	10	MOLECULE:	GLOBIN I -	ARK SHE	LL
95:00:00	3bcq-D	4.1	3.6	87	146	7	MOLECULE:	ALPHA-CHAI	N HEMOGL	OBIN
96:00:00	3g4v-B	4.1	3.4	84	145	8	MOLECULE:	GLOBIN-1		
97:00:00	7dy3-D	4.1	3.5	84	146	6	MOLECULE:	HEMOGLOBIN	SUBUNIT	ALPHA
98:00:00	7jjq-D	4.1	3.4	83	144	6	MOLECULE:	HEMOGLOBIN	SUBUNIT	ALPHA
99:00:00	5x2s-E	4.1	3.2	83	140	7	MOLECULE:	HEMOGLOBIN	SUBUNIT	ALPHA
0:00	3uhx-A	4.1	3.4	85	145	8	MOLECULE:	GLOBIN-1		
1:00	7rb4-A	4	10.8	116	603	9	MOLECULE:	DIPHTHERIA	TOXIN,	C DOMAIN
2:00	5tsz-A	4	4.8	86	130	9	MOLECULE:	PV CELL-TR	AVERSAL	PROTEIN
3:00	2peg-B	4	3.9	84	139	6	MOLECULE:	HEMOGLOBIN	SUBUNIT	ALPHA
4:00	7ewp-A	4	15.3	109	577	4	MOLECULE:	PROBABLE G	#NAME?	COUPLED RECEPTOR 158
5:00	7uw5-A	4	20.6	123	723	7	MOLECULE:	MECHANOSEN	SITIVE C	HANNEL MSCK
6:00	5o5e-A	4	6.2	118	381	2	MOLECULE:	UDP-N-ACET	YLGLUCOS	AMINE--DOLICHYL-PHOSPHATE N-
7:00	6z16-d	4	13.7	105	489	6	MOLECULE:	MULTISUBUN	IT NA+/H	+ ANTIPORTER, A SUBUNIT
8:00	1nwi-B	4	3.3	84	145	8	MOLECULE:	GLOBIN I		
9:00	2auo-B	4	3.5	85	145	8	MOLECULE:	GLOBIN I		
10:00	3hf4-A	4	4.3	86	141	7	MOLECULE:	HEMOGLOBIN	SUBUNIT	ALPHA-1/2
11:00	1nxf-A	4	3.3	84	145	8	MOLECULE:	GLOBIN I		
12:00	3uhd-A	4	3.3	85	145	9	MOLECULE:	GLOBIN-1		
13:00	3uhi-D	4	3.3	84	145	8	MOLECULE:	GLOBIN-1		
14:00	3g4y-B	4	3.5	85	145	8	MOLECULE:	GLOBIN-1		
15:00	1a0u-B	4	3.7	83	146	5	MOLECULE:	HEMOGLOBIN	(ALPHA	CHAIN)
16:00	3g4q-B	4	3.5	85	145	8	MOLECULE:	GLOBIN-1		
17:00	3uh7-B	4	3.5	85	145	8	MOLECULE:	GLOBIN-1		
18:00	2auq-B	4	3.5	85	145	8	MOLECULE:	GLOBIN I		

19:00	1jzk-C	4	3.3	84	145	8	MOLECULE:	GLOBIN I -	ARK SHE	LL
20:00	3uhi-C	4	3.3	85	145	9	MOLECULE:	GLOBIN-1		
21:00	4mqj-B	4	4.4	87	146	2	MOLECULE:	HEMOGLOBIN	SUBUNIT	ALPHA
22:00	1hbr-B	4	3.9	84	143	6	MOLECULE:	PROTEIN (H	EMOGLOBI	N D)
23:00	4n7n-A	4	4.1	87	141	7	MOLECULE:	HEMOGLOBIN	SUBUNIT	ALPHA
24:00:00	3g52-B	4	3.5	85	145	8	MOLECULE:	GLOBIN-1		
25:00:00	3uhb-B	4	3.5	85	145	8	MOLECULE:	GLOBIN-1		
26:00:00	1nwn-A	4	3.3	85	145	9	MOLECULE:	GLOBIN I		
27:00:00	2r4x-A	4	3.3	84	145	8	MOLECULE:	GLOBIN-1		
28:00:00	3uhc-B	4	3.5	85	145	8	MOLECULE:	GLOBIN-1		
29:00:00	3uhi-B	4	3.4	84	145	8	MOLECULE:	GLOBIN-1		
30:00:00	3uht-A	4	3.3	84	145	10	MOLECULE:	GLOBIN-1		
31:00:00	1jwn-C	4	3.3	84	145	8	MOLECULE:	GLOBIN I -	ARK SHE	LL
32:00:00	2av3-A	4	3.3	84	145	8	MOLECULE:	GLOBIN I		
33:00:00	1jzl-A	4	3.3	84	145	8	MOLECULE:	GLOBIN I -	ARK SHE	LL
34:00:00	3uhh-A	4	3.3	84	145	8	MOLECULE:	GLOBIN-1		
35:00:00	4hrt-D	4	3.4	87	151	9	MOLECULE:	GLOBIN-2 A	CHAIN	
36:00:00	2aup-A	4	3.4	85	145	8	MOLECULE:	GLOBIN I		
37:00:00	8vyl-A	4	3.3	84	137	7	MOLECULE:	HEMOGLOBIN	SUBUNIT	ALPHA
38:00:00	3uhk-B	4	3.3	84	145	8	MOLECULE:	GLOBIN-1		
39:00:00	2aur-A	4	3.3	84	145	8	MOLECULE:	GLOBIN I		
40:00:00	3ugz-A	4	3.3	84	145	8	MOLECULE:	GLOBIN-1		
41:00:00	3g4r-B	4	3.5	85	145	8	MOLECULE:	GLOBIN-1		
42:00:00	3uhc-A	4	3.3	85	145	9	MOLECULE:	GLOBIN-1		
43:00:00	7k4m-I	4	4.1	86	140	7	MOLECULE:	HEMOGLOBIN	SUBUNIT	ALPHA
44:00:00	2r4y-A	4	3.3	84	145	8	MOLECULE:	GLOBIN-1		
45:00:00	5hbi-B	4	3.5	85	145	8	MOLECULE:	HEMOGLOBIN		
46:00:00	2qls-B	4	3.5	84	146	7	MOLECULE:	HEMOGLOBIN	SUBUNIT	ALPHA
47:00:00	1hbi-B	4	3.4	84	145	8	MOLECULE:	HEMOGLOBIN	(OXY)	
48:00:00	3a0g-B	4	3.6	86	144	8	MOLECULE:	HEMOGLOBIN	SUBUNIT	ALPHA
49:00:00	4x0l-B	4	3.5	84	144	6	MOLECULE:	HEMOGLOBIN	SUBUNIT	ALPHA
50:00:00	6wg7-F	4	8.3	106	228	10	MOLECULE:	DNA (35-ME	R)	
51:00:00	5jdo-F	4	3.5	84	144	6	MOLECULE:	HAPTOGLOBI	N-HAEMOG	LOBIN RECEPTOR
52:00:00	3gys-D	4	3.6	85	145	7	MOLECULE:	HEMOGLOBIN	SUBUNIT	ALPHA
53:00:00	3gqr-F	4	3.5	84	145	7	MOLECULE:	HEMOGLOBIN	SUBUNIT	ALPHA
54:00:00	1qpw-D	4	3.5	84	146	5	MOLECULE:	PORCINE HE	MOGLOBIN	(ALPHA SUBUNIT)
55:00:00	1hbs-B	4	3.1	83	146	6	MOLECULE:	HEMOGLOBIN	S (DEOX	Y) (ALPHA CHAIN)
56:00:00	5x2s-A	4	3.9	86	140	6	MOLECULE:	HEMOGLOBIN	SUBUNIT	ALPHA
57:00:00	2bnl-F	4	4.4	80	129	8	MOLECULE:	MODULATOR	PROTEIN	RSBR
58:00:00	7ud8-B	4	3.5	84	146	6	MOLECULE:	HEMOGLOBIN	SUBUNIT	ALPHA
59:00:00	1hbs-D	4	3.4	85	146	6	MOLECULE:	HEMOGLOBIN	S (DEOX	Y) (ALPHA CHAIN)
60:00:00	2m6z-C	4	3.6	88	141	8	MOLECULE:	HEMOGLOBIN	SUBUNIT	ALPHA
61:00:00	7jy1-B	4	3.6	85	146	6	MOLECULE:	HEMOGLOBIN	SUBUNIT	ALPHA
62:00:00	7vcf-W	4	4.1	87	164	6	MOLECULE:	TIC214		
63:00:00	6wg7-C	4	8	103	228	11	MOLECULE:	DNA (35-ME	R)	
64:00:00	7xk3-E	3.9	6.6	93	198	6	MOLECULE:	NA(+)-TRAN	SLOCATIN	G NADH-QUINONE REDUCTASE SUBUNI
65:00:00	8a5y-T	3.9	22.2	108	650	5	MOLECULE:	ANAPHASE-P	ROMOTING	COMPLEX SUBUNIT CDC27
66:00:00	1or4-A	3.9	6.3	96	169	13	MOLECULE:	HEME-BASED	AEROTAC	TIC TRANSDUCER HEMAT
67:00:00	6v3f-A	3.9	16.3	131	1234	9	MOLECULE:	NPC1-LIKE	INTRACEL	LULAR CHOLESTEROL TRANSPORTER 1
68:00:00	8q6o-2	3.9	14	98	267	4	MOLECULE:	DNA REPLIC	ATION LI	CENSING FACTOR MCM2
69:00:00	8q4h-C	3.9	4.1	70	89	4	MOLECULE:	TETRACHLOR	OETHENE	REDUCTIVE DEHALOGENASE
70:00:00	5y0r-A	3.9	12.3	101	423	5	MOLECULE:	TMTMCAL(SE	MET)	
71:00:00	8xh6-A	3.9	10.6	80	161	9	MOLECULE:	LATENT MEM	BRANE PR	OTEIN 1
72:00:00	5ex8-A	3.9	11.4	140	414	9	MOLECULE:	CYTOCHROME	P450	

73:00:00	6ygb-B	3.9	23.2	143	725	6	MOLECULE:	N-ALPHA-AC	ETYLTRAN	SFERASE 30
74:00:00	5azr-A	3.9	3.7	81	153	12	MOLECULE:	MYOGLOBIN		
75:00:00	1dwr-A	3.9	4.1	82	152	12	MOLECULE:	MYOGLOBIN		
76:00:00	2vlz-A	3.9	4.1	82	153	12	MOLECULE:	MYOGLOBIN		
77:00:00	1xch-A	3.9	3.7	81	153	11	MOLECULE:	MYOGLOBIN		
78:00:00	6zfb-H	3.9	19.6	126	771	7	MOLECULE:	DNA-DIRECT	ED RNA P	OLYMERASE SUBUNIT DELTA
79:00:00	3lr7-A	3.9	4	82	151	12	MOLECULE:	MYOGLOBIN		
80:00:00	5z7f-A	3.9	4	82	152	12	MOLECULE:	MYOGLOBIN		
81:00:00	5x2u-E	3.9	4.5	86	140	7	MOLECULE:	HEMOGLOBIN	SUBUNIT	ALPHA
82:00:00	3ugy-B	3.9	3.5	85	145	8	MOLECULE:	GLOBIN-1		
83:00:00	1y85-D	3.9	3.5	82	145	5	MOLECULE:	HEMOGLOBIN	ALPHA C	HAIN
84:00:00	5x2r-C	3.9	4.4	86	140	7	MOLECULE:	HEMOGLOBIN	SUBUNIT	ALPHA
85:00:00	3uhh-B	3.9	3.5	85	145	8	MOLECULE:	GLOBIN-1		
86:00:00	7dy4-H	3.9	3.4	82	146	4	MOLECULE:	HEMOGLOBIN	SUBUNIT	ALPHA
87:00:00	5x2r-K	3.9	4.3	86	140	7	MOLECULE:	HEMOGLOBIN	SUBUNIT	ALPHA
88:00:00	3uhe-B	3.9	3.5	85	145	8	MOLECULE:	GLOBIN-1		
89:00:00	2r4w-B	3.9	3.4	84	145	8	MOLECULE:	GLOBIN-1		
90:00:00	3qob-B	3.9	3.4	85	145	9	MOLECULE:	GLOBIN-1		
91:00:00	5x2t-C	3.9	4.5	86	140	7	MOLECULE:	HEMOGLOBIN	SUBUNIT	ALPHA
92:00:00	1ygd-D	3.9	4.1	86	146	5	MOLECULE:	HEMOGLOBIN	ALPHA C	HAIN
93:00:00	3odq-A	3.9	4	86	141	7	MOLECULE:	HEMOGLOBIN	SUBUNIT	ALPHA
94:00:00	2av0-B	3.9	3.5	85	145	8	MOLECULE:	GLOBIN I		
95:00:00	3uh5-B	3.9	3.5	85	145	8	MOLECULE:	GLOBIN-1		
96:00:00	3uh6-B	3.9	3.5	85	145	8	MOLECULE:	GLOBIN-1		
97:00:00	7ud7-D	3.9	3.9	83	146	5	MOLECULE:	HEMOGLOBIN	SUBUNIT	ALPHA
98:00:00	2grh-B	3.9	3.5	85	145	8	MOLECULE:	GLOBIN-1		
99:00:00	1jzl-B	3.9	3.5	85	145	8	MOLECULE:	GLOBIN I -	ARK SHE	LL
0:00	2r80-D	3.9	4.1	86	146	7	MOLECULE:	HEMOGLOBIN	SUBUNIT	ALPHA-A
1:00	1fdh-H	3.9	3.9	86	146	2	MOLECULE:	HEMOGLOBIN	F (DEOX | Y) (ALPHA CHAIN)
2:00	3g53-B	3.9	3.5	85	145	8	MOLECULE:	GLOBIN-1		
3:00	7hbi-B	3.9	3.5	85	145	8	MOLECULE:	HEMOGLOBIN		
4:00	3sdh-B	3.9	3.5	85	145	8	MOLECULE:	HEMOGLOBIN	I (CARB | ONMONOXY)
5:00	1hds-C	3.9	3.5	86	141	5	MOLECULE:	HEMOGLOBIN	S (DEOX | Y) (ALPHA CHAIN)
6:00	7dy3-B	3.9	3.8	83	146	5	MOLECULE:	HEMOGLOBIN	SUBUNIT	ALPHA
7:00	3uhg-B	3.9	3.5	85	145	8	MOLECULE:	GLOBIN-1		
8:00	1y2z-D	3.9	4.4	86	146	5	MOLECULE:	HEMOGLOBIN	ALPHA C	HAIN
9:00	3uh3-B	3.9	3.5	85	145	9	MOLECULE:	GLOBIN-1		
10:00	4n7p-G	3.9	4.4	87	141	7	MOLECULE:	HEMOGLOBIN	SUBUNIT	ALPHA
11:00	3lr9-A	3.9	4	82	152	12	MOLECULE:	MYOGLOBIN		
12:00	3ugz-B	3.9	3.5	85	145	8	MOLECULE:	GLOBIN-1		
13:00	2o5s-X	3.9	4.1	82	152	12	MOLECULE:	MYOGLOBIN		
14:00	7vde-B	3.9	3.6	85	146	6	MOLECULE:	HEMOGLOBIN	SUBUNIT	ALPHA
15:00	7vde-D	3.9	3.6	85	146	6	MOLECULE:	HEMOGLOBIN	SUBUNIT	ALPHA
16:00	4h2l-B	3.9	3.5	84	146	6	MOLECULE:	ALPHA-GLOB	IN	
17:00	5ufj-B	3.9	3.6	84	146	6	MOLECULE:	HEMOGLOBIN	SUBUNIT	ALPHA
18:00	5ker-F	3.9	3.5	84	146	5	MOLECULE:	ALPHA-GLOB	IN	
19:00	2v1k-A	3.9	4.1	82	153	12	MOLECULE:	MYOGLOBIN		
20:00	5ker-D	3.9	3.6	84	146	5	MOLECULE:	ALPHA-GLOB	IN	
21:00	5ker-H	3.9	3.7	86	146	5	MOLECULE:	ALPHA-GLOB	IN	
22:00	7jy0-D	3.9	3.6	84	146	6	MOLECULE:	HEMOGLOBIN	SUBUNIT	ALPHA
23:00	2m6z-A	3.9	3.5	88	141	7	MOLECULE:	HEMOGLOBIN	SUBUNIT	ALPHA
24:00:00	3gys-B	3.9	3.5	84	145	10	MOLECULE:	HEMOGLOBIN	SUBUNIT	ALPHA
25:00:00	4f4o-E	3.9	3.7	86	146	5	MOLECULE:	HEMOGLOBIN	SUBUNIT	ALPHA
26:00:00	4f4o-K	3.9	3.6	85	146	5	MOLECULE:	HEMOGLOBIN	SUBUNIT	ALPHA

27:00:00	3mjp-D	3.9	3.6	85	146	8	MOLECULE:	HEMOGLOBIN	SUBUNIT	ALPHA-A
28:00:00	2hvr-A	3.8	13.9	77	319	5	MOLECULE:	5'-		
29:00:00	1it2-A	3.8	3.2	77	146	5	MOLECULE:	HEMOGLOBIN		
30:00:00	2vwa-A	3.8	2.9	70	99	13	MOLECULE:	PUTATIVE U	NCHARACT	ERIZED PROTEIN PF13_0012
31:00:00	8cr1-C	3.8	9.8	114	266	4	MOLECULE:	ATPASE ASN	A1	
32:00:00	6rmg-A	3.8	7	118	1026	8	MOLECULE:	PROTEIN PA	TCHED HO	MOLOG 1,GFP-LIKE FLUORESCENT
33:00:00	7ctp-A	3.8	4.3	113	553	6	MOLECULE:	PROTEIN NI	BAN 2	
34:00:00	7dgy-A	3.8	3.5	61	99	3	MOLECULE:	C2		
35:00:00	4bja-A	3.8	5.2	99	182	6	MOLECULE:	PROTEIN GL	B-12	
36:00:00	5lbm-C	3.8	4	72	90	6	MOLECULE:	TRANSCRIPT	IONAL RE	PRESSOR FRMR
37:00:00	7etm-A	3.8	9.1	105	498	12	MOLECULE:	PORTAL PRO	TEIN	
38:00:00	1cqx-A	3.8	9	122	403	7	MOLECULE:	FLAVOHEMOP	ROTEIN	
39:00:00	7w0y-k	3.8	6.6	82	98	5	MOLECULE:	NADH DEHYD	ROGENASE	[UBIQUINONE] FLAVOPROTEIN 1,
40:00:00	1x46-A	3.8	4.1	82	150	10	MOLECULE:	HEMOGLOBIN	COMPONE	NT VII
41:00:00	8fib-A	3.8	4.7	123	418	7	MOLECULE:	CYTOCHROME	P450	
42:00:00	8b6l-M	3.8	6.7	76	110	1	MOLECULE:	PROTEIN TR	ANSPORT	PROTEIN SEC61 SUBUNIT ALPHA ISO
43:00:00	2p6v-A	3.8	3.2	76	97	11	MOLECULE:	TRANSCRIPT	ION INIT	IATION FACTOR TFIID SUBUNIT 4
44:00:00	1azi-A	3.8	3.7	81	153	12	MOLECULE:	MYOGLOBIN		
45:00:00	2vlx-A	3.8	3.9	85	153	12	MOLECULE:	MYOGLOBIN		
46:00:00	3wtg-D	3.8	3.9	84	146	8	MOLECULE:	HEMOGLOBIN	SUBUNIT	ALPHA-A
47:00:00	5x2u-A	3.8	4.5	86	140	7	MOLECULE:	HEMOGLOBIN	SUBUNIT	ALPHA
48:00:00	3uhd-B	3.8	3.5	84	144	7	MOLECULE:	GLOBIN-1		
49:00:00	3at6-B	3.8	4.2	88	146	7	MOLECULE:	ALPHAA-GLO	BIN	
50:00:00	5x2s-K	3.8	4.3	86	140	7	MOLECULE:	HEMOGLOBIN	SUBUNIT	ALPHA
51:00:00	3pel-B	3.8	3.6	82	146	6	MOLECULE:	HEMOGLOBIN	SUBUNIT	ALPHA
52:00:00	7pch-D	3.8	3.8	84	146	4	MOLECULE:	HEMOGLOBIN	SUBUNIT	ALPHA
53:00:00	7cue-D	3.8	3.6	82	146	5	MOLECULE:	HEMOGLOBIN	SUBUNIT	ALPHA
54:00:00	3dht-B	3.8	3.7	85	145	6	MOLECULE:	HEMOGLOBIN	SUBUNIT	ALPHA-1/2
55:00:00	1nej-B	3.8	3.6	84	146	6	MOLECULE:	HEMOGLOBIN	ALPHA C	HAIN
56:00:00	1xq5-A	3.8	3.3	85	142	7	MOLECULE:	HEMOGLOBIN	ALPHA-1	CHAIN
57:00:00	6wfq-C	3.8	8	103	228	11	MOLECULE:	HTH-TYPE T	RANSCRIP	TIONAL REPRESSOR NANR
58:00:00	5x2t-E	3.8	3.4	84	140	8	MOLECULE:	HEMOGLOBIN	SUBUNIT	ALPHA
59:00:00	6wg7-E	3.8	8	103	228	11	MOLECULE:	DNA (35-ME	R)	
60:00:00	1hbs-G	3.8	3.4	85	141	4	MOLECULE:	HEMOGLOBIN	S (DEOX	Y) (ALPHA CHAIN)
61:00:00	5cne-A	3.8	4	82	152	12	MOLECULE:	MYOGLOBIN		
62:00:00	1nek-D	3.7	8.7	84	113	7	MOLECULE:	SUCCINATE	DEHYDROG	ENASE FLAVOPROTEIN SUBUNIT
63:00:00	8egr-G	3.7	12.5	94	313	4	MOLECULE:	GP15, RECE	PTOR-BIN	DING PROTEIN, TAIL FIBER
64:00:00	7qru-F	3.7	7.5	76	91	5	MOLECULE:	NA+/H+ ANT	IPORTER	SUBUNIT D
65:00:00	7vgh-B	3.7	24.2	146	1182	3	MOLECULE:	CELL CYCLE	CONTROL	PROTEIN 50B
66:00:00	7qe7-A	3.7	21.6	122	1648	6	MOLECULE:	ANAPHASE-P	ROMOTING	COMPLEX SUBUNIT 10
67:00:00	4zi2-C	3.7	7.8	102	131	4	MOLECULE:	ADP-RIBOSY	LATION F	ACTOR-LIKE PROTEIN 3
68:00:00	8jem-B	3.7	13.4	112	419	4	MOLECULE:	TEICHOIC A	CID D-AL	ANYLTRANSFERASE
69:00:00	6zka-K	3.7	6.5	84	98	6	MOLECULE:	NADH-UBIQU	INONE OX	IDOREDUCTASE CHAIN 3
70:00:00	3a59-H	3.7	4	85	146	7	MOLECULE:	HEMOGLOBIN	SUBUNIT	ALPHA-A
71:00:00	2myd-A	3.7	3.6	80	153	10	MOLECULE:	MYOGLOBIN	(N-PROPY	L ISOCYANIDE)
72:00:00	1y85-B	3.7	3.8	82	145	2	MOLECULE:	HEMOGLOBIN	ALPHA C	HAIN
73:00:00	1myh-B	3.7	4.5	89	153	7	MOLECULE:	MYOGLOBIN		
74:00:00	1mni-A	3.7	4.8	90	153	7	MOLECULE:	MYOGLOBIN		
75:00:00	7pcq-D	3.7	3.9	83	146	2	MOLECULE:	HEMOGLOBIN	SUBUNIT	ALPHA
76:00:00	7ud7-B	3.7	3.9	84	146	4	MOLECULE:	HEMOGLOBIN	SUBUNIT	ALPHA
77:00:00	1mbi-A	3.7	3.7	80	153	10	MOLECULE:	MYOGLOBIN		
78:00:00	3hf4-B	3.7	3.7	84	145	6	MOLECULE:	HEMOGLOBIN	SUBUNIT	ALPHA-1/2
79:00:00	2r4z-B	3.7	3.8	82	146	12	MOLECULE:	GLOBIN-1		
80:00:00	2r4z-A	3.7	3.7	82	145	11	MOLECULE:	GLOBIN-1		

81:00:00	2myb-A	3.7	3.6	80	153	10	MOLECULE:	MYOGLOBIN	(METHYL	ISOCYANIDE)
82:00:00	1vxg-A	3.7	3.6	80	153	10	MOLECULE:	MYOGLOBIN		
83:00:00	3gou-B	3.7	3.8	84	146	5	MOLECULE:	HEMOGLOBIN	SUBUNIT	ALPHA
84:00:00	1yoh-A	3.7	3.6	80	153	10	MOLECULE:	MYOGLOBIN		
85:00:00	1ch4-B	3.7	4	85	146	6	MOLECULE:	MODULE-SUB	STITUTED	CHIMERA HEMOGLOBIN BETA-ALPHA
86:00:00	2z6s-A	3.7	3.6	80	151	10	MOLECULE:	MYOGLOBIN		
87:00:00	3eqm-A	3.6	4.8	155	452	6	MOLECULE:	CYTOCHROME	P450 19	A1
88:00:00	8djk-A	3.6	12.3	119	245	8	MOLECULE:	3-HYDROXY-	3-METHYL	GLUTARYL-COENZYME A REDUCTASE
89:00:00	7yug-A	3.6	3	64	117	3	MOLECULE:	PROTEIN BA	NP	
90:00:00	6wu0-B	3.6	6.1	114	854	4	MOLECULE:	HOPANOID B	IOSYNTHE	SIS ASSOCIATED RND TRANSPORTER
91:00:00	6uak-A	3.6	17.9	96	298	11	MOLECULE:	SAM DEPEND	ENT METH	YLTRANSFERASE LAHSB
92:00:00	3zjh-B	3.6	5.5	98	193	8	MOLECULE:	PROTOGLOBI	N	
93:00:00	5yo8-A	3.6	11.3	95	349	4	MOLECULE:	TETRAPRENY	L-BETA-C	URCUMENE SYNTHASE
94:00:00	2hjm-A	3.6	5.4	73	91	5	MOLECULE:	HYPOTHETIC	AL PROTE	IN PF1176
95:00:00	8h17-A	3.6	4.9	91	157	8	MOLECULE:	TLR1989 PR	OTEIN	
96:00:00	6s8h-F	3.6	14	93	239	8	MOLECULE:	LIPOPOLYSA	CCHARIDE	ABC TRANSPORTER, ATP-BINDING P
97:00:00	5jki-A	3.6	5.2	99	201	14	MOLECULE:	PUTATIVE L	IPID PHO	SPHATE PHOSPHATASE YODM
98:00:00	3p3o-A	3.6	5	142	416	5	MOLECULE:	CYTOCHROME	P450	
99:00:00	4zvb-A	3.6	4.1	92	150	5	MOLECULE:	DIGUANYLAT	E CYCLAS	E DOSC
0:00	2gb7-B	3.6	16	85	295	5	MOLECULE:	DNA STRAND		1
1:00	1it3-B	3.6	3.1	76	146	7	MOLECULE:	HEMOGLOBIN		
2:00	1it3-D	3.6	3.2	76	146	7	MOLECULE:	HEMOGLOBIN		
3:00	4f4o-B	3.6	3.8	85	146	1	MOLECULE:	HEMOGLOBIN	SUBUNIT	ALPHA
4:00	2mbw-A	3.6	3.6	80	154	10	MOLECULE:	MYOGLOBIN		
5:00	7k4m-F	3.6	4.3	85	146	4	MOLECULE:	HEMOGLOBIN	SUBUNIT	ALPHA
6:00	110m-A	3.6	3.8	84	154	10	MOLECULE:	MYOGLOBIN		
7:00	1myi-B	3.6	4.4	89	153	8	MOLECULE:	MYOGLOBIN		
8:00	1bzr-A	3.6	4.4	89	153	7	MOLECULE:	PROTEIN (M	YOGLOBIN	)
9:00	1duk-A	3.6	4	81	152	10	MOLECULE:	WILD-TYPE	RECOMBIN	ANT SPERM WHALE
10:00	2g0r-A	3.6	3.8	84	154	10	MOLECULE:	MYOGLOBIN		
11:00	1u7r-A	3.6	3.7	80	153	10	MOLECULE:	MYOGLOBIN		
12:00	1shr-D	3.6	4.4	83	146	2	MOLECULE:	HEMOGLOBIN	ALPHA C	HAIN
13:00	1mti-A	3.6	3.8	84	154	10	MOLECULE:	MYOGLOBIN		
14:00	1vxf-A	3.6	3.6	80	153	10	MOLECULE:	MYOGLOBIN		
15:00	5utc-A	3.6	4.5	89	152	8	MOLECULE:	MYOGLOBIN		
16:00	1ch4-A	3.6	4	85	146	6	MOLECULE:	MODULE-SUB	STITUTED	CHIMERA HEMOGLOBIN BETA-ALPHA
17:00	1mgn-A	3.6	3.8	84	154	10	MOLECULE:	METMYOGLOB	IN	
18:00	2z8a-B	3.6	3.8	82	146	12	MOLECULE:	GLOBIN-1		
19:00	5v7p-A	3.5	6.2	108	281	6	MOLECULE:	PROTEIN-S-	ISOPRENY	LCYSTEINE O-METHYLTRANSFERASE
20:00	3vw4-A	3.5	13	91	122	7	MOLECULE:	REP		
21:00	5eik-A	3.5	5	76	234	5	MOLECULE:	UNCHARACTE	RIZED PR	OTEIN Y57A10A.28
22:00	7bc6-A	3.5	17.5	114	432	6	MOLECULE:	PROTON-COU	PLED FOL	ATE TRANSPORTER
23:00	1us7-B	3.5	14.8	94	193	7	MOLECULE:	HEAT SHOCK	PROTEIN	HSP82
24:00:00	5zle-A	3.5	5.8	98	225	8	MOLECULE:	CYTOCHROME	B REDUC	TASE 1
25:00:00	7nnt-C	3.5	5	87	169	3	MOLECULE:	ENERGY-COU	PLING FA	CTOR TRANSPORTER ATP-BINDING PR
26:00:00	3jc6-5	3.5	12.3	86	254	5	MOLECULE:	DNA REPLIC	ATION LI	CENSING FACTOR MCM2
27:00:00	8slm-A	3.5	4.4	89	248	11	MOLECULE:	ZN DEPENDE	NT HYDRO	LASE FUSED TO HTH DOMAIN, IRRE
28:00:00	4p9f-A	3.5	4.5	94	212	9	MOLECULE:	HTH-TYPE T	RANSCRIP	TIONAL REGULATOR MCBR
29:00:00	8wu3-B	3.5	12.6	131	592	7	MOLECULE:	RNA POLYME	RASE	
30:00:00	4m8m-B	3.5	16.3	104	574	5	MOLECULE:	GCN4 COILE	D-COIL F	USED ZEBRAFISH PLEXINC1
31:00:00	5t8v-A	3.5	19.2	109	1315	6	MOLECULE:	PUTATIVE U	NCHARACT	ERIZED PROTEIN
32:00:00	7tch-A	3.5	13.2	104	645	3	MOLECULE:	BACITRACIN	EXPORT	PERMEASE PROTEIN BCEB
33:00:00	6g2j-K	3.5	6.6	81	98	5	MOLECULE:	NADH-UBIQU	INONE OX	IDOREDUCTASE CHAIN 3
34:00:00	6csm-D	3.5	9.6	112	277	10	MOLECULE:	GTACR1		

35:00:00	7obq-u	3.5	16.4	88	441	6	MOLECULE:	SRP RNA		
36:00:00	6wg3-E	3.5	16.3	103	1226	6	MOLECULE:	STRUCTURAL	MAINTEN	ANCE OF CHROMOSOMES PROTEIN 1A
37:00:00	6iuy-A	3.5	18.1	93	585	8	MOLECULE:	GLYCEROL-3	#NAME?	TE DEHYDROGENASE [NAD(+)]
38:00:00	4dwl-B	3.5	4.8	77	110	10	MOLECULE:	BBP7		
39:00:00	7dpd-B	3.5	15.3	91	273	4	MOLECULE:	DNA HELICA	SE MCM9	
40:00:00	1jdo-A	3.5	4	85	154	9	MOLECULE:	MYOGLOBIN		
41:00:00	2pgh-B	3.5	4.1	85	146	2	MOLECULE:	HEMOGLOBIN	(AQUO M | ET) (ALPHA CHAIN)
42:00:00	1mlg-A	3.5	3.8	84	154	10	MOLECULE:	MYOGLOBIN		
43:00:00	1myi-A	3.5	3.7	80	153	14	MOLECULE:	MYOGLOBIN		
44:00:00	4f4o-H	3.5	4.2	87	146	1	MOLECULE:	HEMOGLOBIN	SUBUNIT	ALPHA
45:00:00	1hbs-F	3.5	3.8	84	146	4	MOLECULE:	HEMOGLOBIN	S (DEOX | Y) (ALPHA CHAIN)
46:00:00	6on4-B	3.5	13	102	214	10	MOLECULE:	HTH-TYPE T	RANSCRIP	TIONAL REPRESSOR NANR
47:00:00	3qma-A	3.5	4	78	145	5	MOLECULE:	MYOGLOBIN		
48:00:00	2nrl-A	3.5	4	78	145	5	MOLECULE:	MYOGLOBIN		
49:00:00	1mlh-A	3.5	3.8	84	154	10	MOLECULE:	MYOGLOBIN		
50:00:00	1z8u-B	3.5	3.7	84	135	8	MOLECULE:	ALPHA-HEMO	GLOBIN S	TABILIZING PROTEIN
51:00:00	4k0f-A	3.4	10.6	146	531	8	MOLECULE:	LANOSTEROL	14-ALPH	A DEMETHYLASE
52:00:00	4p96-A	3.4	4.7	115	278	10	MOLECULE:	FATTY ACID	METABOL	ISM REGULATOR PROTEIN
53:00:00	7x2q-A	3.4	16.3	97	467	7	MOLECULE:	SUGIOL SYN	THASE	
54:00:00	7b1s-D	3.4	16.5	122	593	11	MOLECULE:	ETHYL-COEN	ZYME M R	EDUCTASE ALPHA SUBUNIT
55:00:00	1lqs-L	3.4	12.1	118	142	12	MOLECULE:	INTERLEUKI	N-10 REC	EPTOR ALPHA CHAIN
56:00:00	8j5t-C	3.4	10	107	288	12	MOLECULE:	UNCHARACTE	RIZED PR	OTEIN RV1280C
57:00:00	7tt9-A	3.4	3.8	66	116	6	MOLECULE:	GROUP 1 TR	UNCATED	HEMOGLOBIN
58:00:00	6m9a-C	3.4	4.4	87	157	8	MOLECULE:	SIGNALING	PROTEIN	
59:00:00	7z8b-C	3.4	14.3	109	1224	13	MOLECULE:	CULLIN-7		
60:00:00	7eu3-G	3.4	7	98	176	5	MOLECULE:	NAD(P)H-QU	INONE OX	IDOREDUCTASE SUBUNIT 1, CHLOROP
61:00:00	7pkq-o	3.4	13.5	88	163	2	MOLECULE:	MS35		
62:00:00	2pke-A	3.4	5.4	95	233	2	MOLECULE:	HALOACID D	ELAHOGEN	ASE-LIKE FAMILY HYDROLASE
63:00:00	7lt2-A	3.4	20.7	96	387	7	MOLECULE:	MAB-21 DOM	AIN-CONT	AINING PROTEIN
64:00:00	6ezn-B	3.4	7.4	77	110	3	MOLECULE:	DOLICHYL-D	IPHOSPHO	OLIGOSACCHARIDE--PROTEIN
65:00:00	3l9w-B	3.4	9	103	357	4	MOLECULE:	GLUTATHION	E-REGULA	TED POTASSIUM-EFFLUX SYSTEM PRO
66:00:00	8dl7-A	3.4	16	115	467	5	MOLECULE:	SOLUTE CAR	RIER FAM	ILY 40 MEMBER 1
67:00:00	7ar9-J	3.4	7.1	83	145	2	MOLECULE:	ND3		
68:00:00	2dc3-B	3.4	5	91	168	4	MOLECULE:	CYTOGLOBIN		
69:00:00	1ury-B	3.4	4.2	84	154	5	MOLECULE:	CYTOGLOBIN		
70:00:00	5x2t-K	3.4	3.6	80	140	9	MOLECULE:	HEMOGLOBIN	SUBUNIT	ALPHA
71:00:00	5cnc-A	3.4	4.8	81	152	10	MOLECULE:	MYOGLOBIN		
72:00:00	3wi8-A	3.4	4.5	79	151	10	MOLECULE:	MYOGLOBIN		
73:00:00	3qm8-A	3.4	4.1	81	145	5	MOLECULE:	MYOGLOBIN		
74:00:00	6otw-A	3.4	4.9	86	151	7	MOLECULE:	HEMOGLOBIN	II	
75:00:00	4hrt-B	3.4	4.1	86	146	12	MOLECULE:	GLOBIN-2 A	CHAIN	
76:00:00	3qm7-A	3.4	4.3	77	144	5	MOLECULE:	MYOGLOBIN		
77:00:00	5cnf-A	3.4	4.8	81	152	10	MOLECULE:	MYOGLOBIN		
78:00:00	5x2r-A	3.4	3.6	79	140	9	MOLECULE:	HEMOGLOBIN	SUBUNIT	ALPHA
79:00:00	3lhb-H	3.4	3	76	149	3	MOLECULE:	PROTEIN (H	EMOGLOBI	N)
80:00:00	1uc3-K	3.4	3.1	76	149	3	MOLECULE:	GLOBIN		
81:00:00	1f5p-F	3.4	3.2	77	149	3	MOLECULE:	HEMOGLOBIN	V	
82:00:00	3lhb-B	3.4	3.2	77	149	3	MOLECULE:	PROTEIN (H	EMOGLOBI	N)
83:00:00	3lhb-C	3.4	3.2	77	149	3	MOLECULE:	PROTEIN (H	EMOGLOBI	N)
84:00:00	1f5p-D	3.4	3.2	77	149	3	MOLECULE:	HEMOGLOBIN	V	
85:00:00	3lhb-A	3.4	3.1	76	149	3	MOLECULE:	PROTEIN (H	EMOGLOBI	N)
86:00:00	3lhb-D	3.4	3.2	77	149	3	MOLECULE:	PROTEIN (H	EMOGLOBI	N)
87:00:00	1dwt-A	3.4	4.6	81	152	10	MOLECULE:	MYOGLOBIN		
88:00:00	3lhb-F	3.4	3.2	77	149	3	MOLECULE:	PROTEIN (H	EMOGLOBI	N)

89:00:00	3lhb-I	3.4	3.2	77	149	3	MOLECULE:	PROTEIN (H	EMOGLOBI	N)	
90:00:00	1f5p-B	3.4	3.2	77	149	3	MOLECULE:	HEMOGLOBIN	V		
91:00:00	1f5p-A	3.4	3.2	77	149	3	MOLECULE:	HEMOGLOBIN	V		
92:00:00	3lhb-L	3.4	3.2	77	149	3	MOLECULE:	PROTEIN (H	EMOGLOBI	N)	
93:00:00	3lhb-G	3.4	3.2	77	149	3	MOLECULE:	PROTEIN (H	EMOGLOBI	N)	
94:00:00	3lhb-K	3.4	3.1	76	149	3	MOLECULE:	PROTEIN (H	EMOGLOBI	N)	
95:00:00	3hrw-D	3.4	3.7	83	146	7	MOLECULE:	HEMOGLOBIN	SUBUNIT	ALPHA	
96:00:00	1f5p-C	3.4	3.6	78	149	3	MOLECULE:	HEMOGLOBIN	V		
97:00:00	3lhb-J	3.4	3.2	77	149	3	MOLECULE:	PROTEIN (H	EMOGLOBI	N)	
98:00:00	1f5p-E	3.4	3.2	77	149	3	MOLECULE:	HEMOGLOBIN	V		
99:00:00	3lhb-E	3.4	3.1	76	149	3	MOLECULE:	PROTEIN (H	EMOGLOBI	N)	
0:00	6wq0-A	3.3	14.2	80	131	5	MOLECULE:	DNA (301-M	ER)		
1:00	6z3w-F	3.3	14.1	76	110	8	MOLECULE:	ER MEMBRAN	E PROTEI	N COMPLEX SUBUNIT 1,ER MEMBRANE	
2:00	2cqn-A	3.3	4.2	66	77	2	MOLECULE:	FORMIN-BIN	DING PRO	TEIN 3	
3:00	5k94-A	3.3	19.3	98	506	8	MOLECULE:	MALTOSE-BI	NDING PE	RIPLASMIC PROTEIN,PROTEIN TRANS	
4:00	7a7c-A	3.3	17.4	127	560	6	MOLECULE:	CATALASE-P	EROXIDAS	E	
5:00	2mjm-A	3.3	3.6	67	101	3	MOLECULE:	PROTEIN NL	RC5		
6:00	3al0-B	3.3	16.7	90	482	7	MOLECULE:	GLUTAMYL-T	RNA(GLN)	AMIDOTRANSFERASE SUBUNIT A	
7:00	7c79-L	3.3	9.3	85	131	4	MOLECULE:	RIBONUCLEA	SE MRP R	NA SUBUNIT NME1	
8:00	4gr6-B	3.3	14.8	79	105	3	MOLECULE:	ATRBCX2			
9:00	8ce1-c	3.3	15.7	99	241	3	MOLECULE:	CYTOCHROME	C BIOGE	NESIS ATP-BINDING EXPORT PROTEI	
10:00	6zgd-A	3.3	16.6	90	288	10	MOLECULE:	PROTON-GAT	ED ION C	HANNEL	
11:00	3ua3-A	3.3	12.1	105	663	2	MOLECULE:	PROTEIN AR	GININE N	-METHYLTRANSFERASE 5	
12:00	7etw-B	3.3	13.3	99	483	6	MOLECULE:	INSULIN-IN	DUCED GE	NE 2 PROTEIN	
13:00	1q1v-A	3.3	6.4	60	70	3	MOLECULE:	DEK PROTEI	N		
14:00	3dan-A	3.3	6.1	100	473	2	MOLECULE:	CYTOCHROME	P450 74	A2	
15:00	2zop-A	3.3	3.3	72	113	6	MOLECULE:	PUTATIVE U	NCHARACT	ERIZED PROTEIN TTHB164	
16:00	6c94-A	3.3	18.1	106	482	8	MOLECULE:	CYTOCHROME	P450 4B		1
17:00	7dgu-A	3.3	4.6	71	97	7	MOLECULE:	A1			
18:00	7mhz-A	3.3	10.9	92	490	3	MOLECULE:	SONIC HEDG	EHOG PRO	TEIN N-PRODUCT PEPTIDE	
19:00	7pq1-A	3.3	7	82	395	4	MOLECULE:	CYTOCHROME	P450 PR	OTEIN	
20:00	1gzs-B	3.3	4.1	85	165	8	MOLECULE:	SOPE			
21:00	8p50-A	3.3	25.6	111	1574	5	MOLECULE:	TOXIN PROT	EIN		
22:00	1dws-A	3.3	4.8	81	152	10	MOLECULE:	MYOGLOBIN			
23:00	5jom-A	3.3	4.4	79	154	9	MOLECULE:	MYOGLOBIN			
24:00:00	1npf-A	3.3	4.8	81	152	10	MOLECULE:	MYOGLOBIN			
25:00:00	2frj-X	3.3	4.8	81	152	10	MOLECULE:	MYOGLOBIN			
26:00:00	5o41-A	3.3	4.4	78	154	9	MOLECULE:	MYOGLOBIN			
27:00:00	5cn8-A	3.3	4.8	81	152	10	MOLECULE:	MYOGLOBIN			
28:00:00	5cnd-A	3.3	4.8	81	152	10	MOLECULE:	MYOGLOBIN			
29:00:00	1gjn-A	3.3	4.8	81	153	10	MOLECULE:	MYOGLOBIN			
30:00:00	1wla-A	3.3	5.4	82	153	10	MOLECULE:	MYOGLOBIN			
31:00:00	2vly-A	3.3	4.9	81	153	10	MOLECULE:	MYOGLOBIN			
32:00:00	1z8u-D	3.3	3.3	80	135	6	MOLECULE:	ALPHA-HEMO	GLOBIN S	TABILIZING PROTEIN	
33:00:00	1npg-A	3.3	4.8	81	152	10	MOLECULE:	MYOGLOBIN			
34:00:00	2frf-A	3.3	4.8	81	152	10	MOLECULE:	MYOGLOBIN			
35:00:00	5cn9-A	3.3	4.8	81	152	10	MOLECULE:	MYOGLOBIN			
36:00:00	2v1f-A	3.3	4.8	81	153	10	MOLECULE:	MYOGLOBIN			
37:00:00	4dc7-A	3.3	4.8	81	152	10	MOLECULE:	MYOGLOBIN			
38:00:00	4dc8-A	3.3	4.8	81	152	10	MOLECULE:	MYOGLOBIN			
39:00:00	5z7e-A	3.3	4.8	81	153	10	MOLECULE:	MYOGLOBIN			
40:00:00	5cnb-A	3.3	4.8	81	152	10	MOLECULE:	MYOGLOBIN			
41:00:00	5cng-A	3.3	4.5	79	152	10	MOLECULE:	MYOGLOBIN			
42:00:00	2v1e-A	3.3	4.8	81	153	10	MOLECULE:	MYOGLOBIN			

43:00:00	5cmv-A	3.3	4.8	81	152	10	MOLECULE:	MYOGLOBIN		
44:00:00	2o5l-X	3.3	4.9	81	152	10	MOLECULE:	MYOGLOBIN		
45:00:00	2fri-X	3.3	4.8	81	152	10	MOLECULE:	MYOGLOBIN		
46:00:00	2vm0-A	3.3	4.8	81	153	10	MOLECULE:	MYOGLOBIN		
47:00:00	5azq-A	3.3	5.6	84	153	10	MOLECULE:	MYOGLOBIN		
48:00:00	3vau-A	3.3	4.8	81	151	10	MOLECULE:	MYOGLOBIN		
49:00:00	2v1h-A	3.3	4.8	81	153	10	MOLECULE:	MYOGLOBIN		
50:00:00	1uc3-D	3.3	3.2	77	149	3	MOLECULE:	GLOBIN		
51:00:00	2h35-C	3.3	4	86	141	8	MOLECULE:	HEMOGLOBIN	ALPHA S	UBUNIT
52:00:00	1uc3-G	3.3	3.4	78	149	3	MOLECULE:	GLOBIN		
53:00:00	2h35-A	3.3	4	86	141	8	MOLECULE:	HEMOGLOBIN	ALPHA S	UBUNIT
54:00:00	1uc3-H	3.3	3.6	78	149	3	MOLECULE:	GLOBIN		
55:00:00	1uc3-A	3.3	3.2	77	149	3	MOLECULE:	GLOBIN		
56:00:00	1qoy-A	3.2	4.5	93	303	5	MOLECULE:	HEMOLYSIN	E	
57:00:00	4cru-A	3.2	10.3	103	242	7	MOLECULE:	CCR4-NOT T	RANSCRIP	TION COMPLEX SUBUNIT 1
58:00:00	7xdi-F	3.2	9.3	83	456	5	MOLECULE:	VP1		
59:00:00	2hs5-A	3.2	4.5	98	207	15	MOLECULE:	PUTATIVE T	RANSCRIP	TIONAL REGULATOR GNTR
60:00:00	6dk9-F	3.2	4.9	122	232	8	MOLECULE:	DNA DAMAGE	#NAME?	LE PROTEIN
61:00:00	6btx-A	3.2	19.8	106	377	15	MOLECULE:	SOLUTE CAR	RIER FAM	ILY 39 (IRON-REGULATED TRANSPOR
62:00:00	4cej-B	3.2	18.6	125	1156	8	MOLECULE:	ATP-DEPEND	ENT HELI	CASE/NUCLEASE SUBUNIT A
63:00:00	5w65-P	3.2	13.4	86	481	6	MOLECULE:	DNA-DIRECT	ED RNA P	OLYMERASE I SUBUNIT RPA190
64:00:00	5ajk-A	3.2	3.8	66	148	2	MOLECULE:	HOMOLOG OF	VACCINI	A VIRUS CDS F1L
65:00:00	5tpm-A	3.2	5.3	102	155	9	MOLECULE:	PYRUVATE D	EHYDROGE	NASE COMPLEX REPRESSOR
66:00:00	3bni-A	3.2	4.9	62	174	11	MOLECULE:	PUTATIVE T	ETR-FAMI	LY TRANSCRIPTIONAL REGULATOR
67:00:00	7v9g-D	3.2	15.6	80	112	5	MOLECULE:	BEN DOMAIN	#NAME?	ING PROTEIN 3
68:00:00	6khj-E	3.2	6.3	80	101	3	MOLECULE:	NAD(P)H-QU	INONE OX	IDOREDUCTASE SUBUNIT 1
69:00:00	7dqk-B	3.2	8.3	84	479	4	MOLECULE:	PROTEIN DE	TOXIFICA	TION
70:00:00	8uw3-A	3.2	11.2	122	1265	5	MOLECULE:	LINE-1 RET	ROTRANSP	OSABLE ELEMENT ORF2 PROTEIN
71:00:00	8q7h-A	3.2	17.9	155	1403	9	MOLECULE:	CULLIN-9		
72:00:00	5z1n-A	3.2	3.8	83	166	12	MOLECULE:	G-PROTEIN	INTERACT	ING PROTEIN 1
73:00:00	8j2x-A	3.2	11.1	74	337	7	MOLECULE:	COBALAMIN-	BINDING	PROTEIN
74:00:00	6zyy-C	3.2	26.7	126	2901	7	MOLECULE:	DYNEIN HEA	VY CHAIN	, OUTER ARM PROTEIN
75:00:00	8q3v-e	3.2	11.8	111	294	8	MOLECULE:	TETRAHYDRO	METHANOP	TERIN S-METHYLTRANSFERASE SUBUN
76:00:00	5d2m-C	3.2	4.7	76	158	16	MOLECULE:	SUMO-CONJU	GATING E	NZYME UBC9
77:00:00	3h36-A	3.2	3.9	67	78	0	MOLECULE:	POLYRIBONU	CLEOTIDE	NUCLEOTIDYLTRANSFERASE
78:00:00	4zsf-A	3.2	5.1	68	272	6	MOLECULE:	BSAWI ENDO	NUCLEASE	
79:00:00	7kfu-C	3.2	14.5	147	909	5	MOLECULE:	CAS2		
80:00:00	4y9j-B	3.2	25.6	125	593	7	MOLECULE:	PROTEIN AC	DH-11, I	SOFORM B
81:00:00	8p1u-A	3.2	7.8	88	370	10	MOLECULE:	CELL DIVIS	ION PROT	EIN FTSL
82:00:00	6xdc-A	3.2	12.7	98	193	9	MOLECULE:	PROTEIN 3A		
83:00:00	2yvx-A	3.2	10.8	96	442	10	MOLECULE:	MG2+ TRANS	PORTER M	GTE
84:00:00	6rao-I	3.2	20.8	110	443	3	MOLECULE:	AFP1		
85:00:00	2khm-A	3.2	10.6	82	140	7	MOLECULE:	FIBROIN-3		
86:00:00	7pik-C	3.2	24.7	87	557	1	MOLECULE:	TRANSPOSON	TN7 TRA	NSPOSITION PROTEIN TNSB
87:00:00	4hkr-A	3.2	9.2	97	165	2	MOLECULE:	CALCIUM RE	LEASE-AC	TIVATED CALCIUM CHANNEL PROTEIN
88:00:00	3ia3-B	3.2	3.6	82	135	6	MOLECULE:	ALPHA-HEMO	GLOBIN-S	TABILIZING PROTEIN
89:00:00	1ymc-A	3.2	5.6	82	153	10	MOLECULE:	CYANOMET-S	ULFMYOGL	OBIN
90:00:00	3ia3-D	3.2	3.6	82	135	6	MOLECULE:	ALPHA-HEMO	GLOBIN-S	TABILIZING PROTEIN
91:00:00	1umo-B	3.2	4.7	88	154	5	MOLECULE:	CYTOGLOBIN		
92:00:00	7a45-A	3.2	4.4	80	154	9	MOLECULE:	MYOGLOBIN		
93:00:00	6otx-A	3.2	4.2	76	151	5	MOLECULE:	HEMOGLOBIN	II	
94:00:00	7dgo-A	3.2	11.3	87	153	3	MOLECULE:	MYOGLOBIN		
95:00:00	7uvb-B	3.2	3.3	81	146	11	MOLECULE:	HEMOGLOBIN	SUBUNIT	ALPHA
96:00:00	3hyu-B	3.2	3.6	84	146	11	MOLECULE:	HEMOGLOBIN	SUBUNIT	ALPHA

97:00:00	3hrw-B	3.2	4.2	83	146	6	MOLECULE:	HEMOGLOBIN	SUBUNIT	ALPHA	
98:00:00	8q7e-J	3.1	18.3	126	895	10	MOLECULE:	CULLIN-9			
99:00:00	6wtw-A	3.1	7.7	107	489	7	MOLECULE:	DASS FAMIL	Y SODIUM	-COUPLED ANION SYMPORTER	
0:00	7c7e-A	3.1	4.7	97	142	5	MOLECULE:	PUTATIVE D	NA-BINDI	NG TRANSCRIPTIONAL REGULATOR	
1:00	3h9p-A	3.1	4.3	82	226	9	MOLECULE:	PUTATIVE T	RIPHOSPH	ORIBOSYL-DEPHOSPHO-COA SYNTHASE	
2:00	5do7-D	3.1	12.5	101	571	7	MOLECULE:	ATP-BINDIN	G CASSET	TE SUB-FAMILY G MEMBER 5	
3:00	9b8q-J	3.1	14.5	74	149	11	MOLECULE:	V-TYPE PRO	TON ATPA	SE SUBUNIT C 1	
4:00	2ilk-A	3.1	14.7	118	155	10	MOLECULE:	INTERLEUKI	N-10		
5:00	4q9v-B	3.1	16.4	93	186	14	MOLECULE:	TUMOR NECR	OSIS FAC	TOR ALPHA-INDUCED PROTEIN 8-LIK	
6:00	7ccl-C	3.1	9.2	72	147	6	MOLECULE:	BCL-2-LIKE	PROTEIN		15
7:00	8v46-D	3.1	4.4	89	305	8	MOLECULE:	ARIA ANTIT	OXIN		
8:00	4mu6-A	3.1	4.7	85	274	7	MOLECULE:	KINECTIN 1	(KINESI	N RECEPTOR)	
9:00	6w6x-A	3.1	8.2	78	126	9	MOLECULE:	DE NOVO DE	SIGNED A	BLE PROTEIN	
10:00	6vjy-B	3.1	12.7	104	280	8	MOLECULE:	ERAD-ASSOC	IATED E3	UBIQUITIN-PROTEIN LIGASE HRD1	
11:00	3v8d-A	3.1	11.2	92	479	5	MOLECULE:	CHOLESTERO	L 7-ALPH	A-MONOOXYGENASE	
12:00	8j98-A	3.1	8.3	79	184	6	MOLECULE:	NEAR-INFRA	RED FLUO	RESCENT PROTEIN	
13:00	7z0o-G	3.1	4	58	84	7	MOLECULE:	HISTONE H3			
14:00	1r5i-D	3.1	17.7	79	214	4	MOLECULE:	HLA CLASS	II HISTO	COMPATIBILITY ANTIGEN, DR	
15:00	7vw0-B	3.1	9.1	76	94	9	MOLECULE:	DUF305 DOM	AIN-CONT	AINING PROTEIN	
16:00	8uc3-C	3.1	7.4	70	98	3	MOLECULE:	ALBONOURSI	N SYNTHA	SE	
17:00	5e2q-A	3.1	20.6	105	725	7	MOLECULE:	DIPEPTIDYL	PEPTIDA	SE 3	
18:00	8w6c-A	3.1	10.8	112	530	8	MOLECULE:	SOLUTE CAR	RIER FAM	ILY 13 MEMBER 2	
19:00	1w9r-A	3.1	10.9	93	119	6	MOLECULE:	CHOLINE BI	NDING PR	OTEIN A	
20:00	8tzk-C	3.1	6.2	81	304	7	MOLECULE:	CELL DIVIS	ION ATP-	BINDING PROTEIN FTSE	
21:00	4uis-B	3.1	4.2	88	241	7	MOLECULE:	GAMMA-SECR	ETASE		
22:00	7yet-A	3.1	29	131	999	6	MOLECULE:	RNA-DIRECT	ED RNA P	OLYMERASE L	
23:00	2qup-A	3.1	7.9	78	119	4	MOLECULE:	BH1478 PRO	TEIN		
24:00:00	8pm6-A	3.1	23.1	108	872	6	MOLECULE:	BILE SALT	EXPORT P	UMP	
25:00:00	3rh2-A	3.1	12	77	210	6	MOLECULE:	HYPOTHETIC	AL TETR-	LIKE TRANSCRIPTIONAL REGULATOR	
26:00:00	3bm3-A	3.1	4.8	95	259	7	MOLECULE:	DNA (5'-D(	*CP*AP*T	P*CP*CP*AP*GP*GP*TP*AP*C)-3')	
27:00:00	6lum-G	3.1	7.3	77	123	8	MOLECULE:	SUCCINATE	DEHYDROG	ENASE SUBUNIT C	
28:00:00	7wuq-R	3.1	8.9	94	278	4	MOLECULE:	GUANINE NU	CLEOTIDE	#NAME?	
29:00:00	2f4q-A	3.1	14.5	74	309	7	MOLECULE:	TYPE I TOP	OISOMERA	SE, PUTATIVE	
30:00:00	6cfw-G	3.1	8.4	80	113	6	MOLECULE:	MONOVALENT	CATION/	H+ ANTIPORTER SUBUNIT D	
31:00:00	3ps5-A	3.1	7.6	103	529	7	MOLECULE:	TYROSINE-P	ROTEIN P	HOSPHATASE NON-RECEPTOR TYPE 6	
32:00:00	5yhf-A	3.1	7.8	121	734	7	MOLECULE:	PROTEIN TR	ANSLOCAS	E SUBUNIT SECDF	
33:00:00	8if8-B	3.1	9.7	99	620	6	MOLECULE:	GALACTAN 5	#NAME?	NOFURANOSYLTRANSFERASE	
34:00:00	7d3u-G	3.1	6.1	79	111	6	MOLECULE:	MONOVALENT	NA+/H+	ANTIPORTER SUBUNIT D	
35:00:00	7op8-A	3.1	21.7	111	1118	5	MOLECULE:	CATION-TRA	NSPORTIN	G ATPASE	
36:00:00	6lum-D	3.1	8.9	82	146	5	MOLECULE:	SUCCINATE	DEHYDROG	ENASE SUBUNIT C	
37:00:00	7ar9-K	3.1	6.5	72	104	10	MOLECULE:	ND3			
38:00:00	7tmw-R	3.1	16.7	100	499	7	MOLECULE:	RELAXIN RE	CEPTOR 1	, GUANINE NUCLEOTIDE-BINDING PR	
39:00:00	4ap2-B	3.1	20.1	125	359	7	MOLECULE:	KELCH-LIKE	PROTEIN		11
40:00:00	8fm9-M	3.1	12.5	108	333	8	MOLECULE:	RNA-DIRECT	ED RNA P	OLYMERASE	
41:00:00	2wus-R	3.1	2.9	60	87	7	MOLECULE:	PUTATIVE U	NCHARACT	ERIZED PROTEIN	
42:00:00	6zmx-D	3.1	4	77	146	17	MOLECULE:	HEMOGLOBIN	SUBUNIT	ALPHA-A	
43:00:00	4hrr-D	3.1	4.2	87	151	7	MOLECULE:	GLOBIN-2 A	CHAIN		
44:00:00	6zmy-B	3.1	3.9	77	146	17	MOLECULE:	HEMOGLOBIN	SUBUNIT	ALPHA-A	
45:00:00	5eui-A	3.1	4.1	75	134	11	MOLECULE:	HBA PROTEI	N		
46:00:00	1jp6-A	3.1	4.9	80	152	9	MOLECULE:	MYOGLOBIN			
47:00:00	4ood-A	3.1	4.9	88	154	6	MOLECULE:	MYOGLOBIN			
48:00:00	4mqk-B	3.1	4.4	80	145	14	MOLECULE:	HEMOGLOBIN	SUBUNIT	ALPHA	
49:00:00	1hv4-D	3.1	4	76	146	17	MOLECULE:	HEMOGLOBIN	ALPHA-A	CHAIN	
50:00:00	3pi4-A	3.1	4.2	77	151	5	MOLECULE:	HEMOGLOBIN	II		

51:00:00	1ch4-D	3.1	3.5	77	146	8	MOLECULE:	MODULE-SUB	STITUTED	CHIMERA HEMOGLOBIN BETA-ALPHA
52:00:00	7k4m-D	3.1	3.4	81	147	11	MOLECULE:	HEMOGLOBIN	SUBUNIT	ALPHA
53:00:00	5ufj-D	3.1	3.3	82	146	11	MOLECULE:	HEMOGLOBIN	SUBUNIT	ALPHA
54:00:00	1ch4-C	3.1	3.6	78	146	9	MOLECULE:	MODULE-SUB	STITUTED	CHIMERA HEMOGLOBIN BETA-ALPHA
55:00:00	3fs4-B	3.1	3.6	85	146	12	MOLECULE:	HEMOGLOBIN	SUBUNIT	ALPHA-A
56:00:00	1myf-A	3.1	4.2	79	153	9	MOLECULE:	MYOGLOBIN		
57:00:00	7ot9-A	3	8.2	79	221	3	MOLECULE:	AI-2E MEMB	ER YDIK	
58:00:00	6zy2-E	3	17	93	259	8	MOLECULE:	YRBD PROTE	IN	
59:00:00	8gkh-P	3	23.2	114	600	7	MOLECULE:	DNA (5'-D(	P*CP*GP*	GP*TP*AP*CP*CP*CP*GP*GP*GP*CP*A
60:00:00	6gkw-A	3	4.8	76	351	8	MOLECULE:	PUTATIVE P	HAGE XKD	K-LIKE PROTEIN
61:00:00	7nur-A	3	15.7	145	1044	10	MOLECULE:	PROTEIN KI	NASE DOM	AIN-CONTAINING PROTEIN
62:00:00	7rpk-A	3	19.8	147	953	7	MOLECULE:	PROTEIN DI	SPATCHED	HOMOLOG 1
63:00:00	2z3r-E	3	3.5	70	119	4	MOLECULE:	INTERLEUKI	N-15	
64:00:00	7lhe-A	3	13.3	107	2222	5	MOLECULE:	INOSITOL 1	,4,5-TRI	SPHOSPHATE RECEPTOR TYPE 1
65:00:00	7vu7-A	3	4.2	64	89	11	MOLECULE:	FLAGELLIFO	RM FIBRO	IN
66:00:00	7d3u-C	3	7.9	90	129	10	MOLECULE:	MONOVALENT	NA+/H+	ANTIPORTER SUBUNIT D
67:00:00	2oeb-A	3	3.5	79	152	5	MOLECULE:	HYPOTHETIC	AL PROTE	IN
68:00:00	1iie-A	3	5.2	51	75	6	MOLECULE:	PROTEIN (H	LA-DR AN	TIGENS ASSOCIATED INVARIANT
69:00:00	6zvh-R	3	14.4	80	135	3	MOLECULE:	18S RRNA		
70:00:00	5yi4-A	3	12.5	83	145	7	MOLECULE:	DISRUPTED	IN SCHIZ	OPHRENIA 1 HOMOLOG,NUCLEAR DIST
71:00:00	7awt-D	3	20.4	114	589	11	MOLECULE:	NADH-QUINO	NE OXIDO	REDUCTASE SUBUNIT B
72:00:00	8j3r-B	3	9.8	91	387	7	MOLECULE:	TRANSPOSAS	E IS605	ORFB C-TERMINAL DOMAIN-CONTAINI
73:00:00	7sbe-A	3	4	90	228	7	MOLECULE:	TELOMERASE	REVERSE	TRANSCRIPTASE
74:00:00	1xsz-A	3	14.9	69	356	4	MOLECULE:	GUANINE NU	CLEOTIDE	EXCHANGE PROTEIN
75:00:00	7wst-A	3	17.4	103	601	6	MOLECULE:	IRON-PHYTO	SIDEROPH	ORE TRANSPORTER
76:00:00	6ebu-A	3	4.9	91	165	8	MOLECULE:	LPXE		
77:00:00	5mps-R	3	11.2	56	108	0	MOLECULE:	YEAST UBC4	GENE FO	R UBIQUITIN-CONJUGATING ENZYME
78:00:00	8tl7-A	3	8	106	694	6	MOLECULE:	COMPUTATIO	NALLY DE	SIGNED PROTEIN
79:00:00	1knz-A	3	6.1	79	154	3	MOLECULE:	5'-R(*UP*G	P*AP*CP*	C)-3'
80:00:00	5m0n-A	3	13.5	132	423	7	MOLECULE:	TERMINAL O	LEFIN-FO	RMING FATTY ACID DECARBOXYLASE
81:00:00	1tu9-A	3	4.5	76	131	9	MOLECULE:	HYPOTHETIC	AL PROTE	IN PA3967
82:00:00	3k3u-B	3	4.6	72	137	4	MOLECULE:	DEHALOPERO	XIDASE A	
83:00:00	8kdb-A	3	11.7	91	2117	3	MOLECULE:	RNA-DIRECT	ED RNA P	OLYMERASE L
84:00:00	6gys-B	3	27.1	112	579	4	MOLECULE:	CENTROMERE	DNA-BIN	DING PROTEIN COMPLEX CBF3 SUBUN
85:00:00	3f7c-A	3	5.7	67	199	9	MOLECULE:	PROTEIN OF	UNKNOWN	FUNCTION (DUF416)
86:00:00	4raf-A	3	17.9	88	362	10	MOLECULE:	PROTEIN PH	OSPHATAS	E 1A
87:00:00	8a3t-C	3	31.5	117	1410	9	MOLECULE:	ANAPHASE-P	ROMOTING	COMPLEX SUBUNIT CDC27
88:00:00	8qq7-A	3	19.1	120	399	8	MOLECULE:	FAD-BINDIN	G FR-TYP	E DOMAIN-CONTAINING PROTEIN
89:00:00	7zmb-6	3	7	87	190	11	MOLECULE:	NADH-UBIQU	INONE OX	IDOREDUCTASE CHAIN 1
90:00:00	7ryq-B	3	13.9	101	527	8	MOLECULE:	KIF-BINDIN	G PROTEI	N
91:00:00	4jbw-F	3	15.8	99	492	3	MOLECULE:	MALTOSE TR	ANSPORT	SYSTEM PERMEASE PROTEIN MALF
92:00:00	8ca1-A	3	12.9	129	589	8	MOLECULE:	VERY LONG-	CHAIN SP	ECIFIC ACYL-COA DEHYDROGENASE,
93:00:00	3tix-A	3	10.3	75	153	12	MOLECULE:	UBIQUITIN-	LIKE PRO	TEIN SMT3,RNA-INDUCED TRANSCRIP
94:00:00	8c3y-A	3	17.3	114	929	4	MOLECULE:	PFEMP1		
95:00:00	6ysg-A	3	19.1	133	1264	7	MOLECULE:	MG-CHELATA	SE SUBUN	IT CHLH
96:00:00	5w7g-B	3	9.5	66	126	9	MOLECULE:	ORF140		
97:00:00	7yh5-B	3	9.1	91	177	15	MOLECULE:	NUCLEOSIDE	TRIPHOS	PHATE PYROPHOSPHOHYDROLASE
98:00:00	7mxb-A	3	10.7	75	672	12	MOLECULE:	SERINE/THR	EONINE P	ROTEIN KINASES
99:00:00	5w7g-A	3	10.7	72	131	6	MOLECULE:	ORF140		
0:00	1uc3-J	3	3.9	79	149	4	MOLECULE:	GLOBIN		
1:00	2lhb-A	3	3.9	78	149	4	MOLECULE:	HEMOGLOBIN	V (CYAN	O MET)
2:00	2di3-B	3	9.5	101	230	6	MOLECULE:	BACTERIAL	REGULATO	RY PROTEINS, GNTR FAMILY
3:00	3pi4-B	3	4.3	76	151	4	MOLECULE:	HEMOGLOBIN	II	
4:00	4mqj-H	3	4.5	79	146	11	MOLECULE:	HEMOGLOBIN	SUBUNIT	ALPHA

5:00	4mqk-F	3	4.5	79	145	14	MOLECULE:	HEMOGLOBIN	SUBUNIT	ALPHA
6:00	6zmy-D	3	4.7	79	146	15	MOLECULE:	HEMOGLOBIN	SUBUNIT	ALPHA-A
7:00	3d4x-D	3	3.9	76	145	14	MOLECULE:	HEMOGLOBIN	SUBUNIT	ALPHA
8:00	3gys-F	3	4.2	77	145	13	MOLECULE:	HEMOGLOBIN	SUBUNIT	ALPHA
9:00	3dhr-B	3	4.6	79	145	16	MOLECULE:	HEMOGLOBIN	SUBUNIT	ALPHA-A
10:00	3dhr-F	3	4.8	79	145	15	MOLECULE:	HEMOGLOBIN	SUBUNIT	ALPHA-A
11:00	3d4x-B	3	4.5	79	145	14	MOLECULE:	HEMOGLOBIN	SUBUNIT	ALPHA
12:00	2hbs-D	3	4.1	76	146	13	MOLECULE:	HEMOGLOBIN	S (DEOX	Y), ALPHA CHAIN
13:00	1dxt-D	3	4.2	80	147	13	MOLECULE:	HEMOGLOBIN	(DEOXY)	(ALPHA CHAIN)
14:00	3dhr-H	3	4.8	79	145	15	MOLECULE:	HEMOGLOBIN	SUBUNIT	ALPHA-A
15:00	3gys-H	3	3.7	78	145	10	MOLECULE:	HEMOGLOBIN	SUBUNIT	ALPHA
16:00	3gqr-D	3	4	77	145	14	MOLECULE:	HEMOGLOBIN	SUBUNIT	ALPHA
17:00	7jy3-B	3	3.4	82	146	10	MOLECULE:	HEMOGLOBIN	SUBUNIT	ALPHA
18:00	1si4-B	3	3.5	84	146	10	MOLECULE:	HEMOGLOBIN	ALPHA C	HAIN
19:00	7qu4-G	3	4	77	143	12	MOLECULE:	HEMOGLOBIN	SUBUNIT	GAMMA-2
20:00	7uf7-D	3	3.6	79	146	11	MOLECULE:	HEMOGLOBIN	SUBUNIT	ALPHA
21:00	2di3-A	2.9	7	104	231	8	MOLECULE:	BACTERIAL	REGULATO	RY PROTEINS, GNTR FAMILY
22:00	7kpv-B	2.9	10	92	290	8	MOLECULE:	MEIOTIC MR	NA STABI	LITY PROTEIN KINASE SSN3
23:00	6mpb-A	2.9	7.4	110	447	6	MOLECULE:	NEUTRAL AM	INO ACID	TRANSPORTER B(0)
24:00:00	7kzn-B	2.9	22.5	110	683	5	MOLECULE:	HEAVY CHAI	N ALPHA	
25:00:00	2wtg-A	2.9	4.2	76	158	5	MOLECULE:	GLOBIN-LIK	E PROTEI	N
26:00:00	7ro2-A	2.9	8.4	61	94	10	MOLECULE:	MAJOR CAPS	ID PROTE	IN
27:00:00	2be4-A	2.9	8.2	103	270	9	MOLECULE:	HYPOTHETIC	AL PROTE	IN LOC449832
28:00:00	5cd4-I	2.9	18.5	83	494	7	MOLECULE:	CRISPR SYS	TEM CASC	ADE SUBUNIT CASE
29:00:00	7qru-G	2.9	9.2	93	113	4	MOLECULE:	NA+/H+ ANT	IPORTER	SUBUNIT D
30:00:00	8eki-E	2.9	18.1	115	643	6	MOLECULE:	PROTEIN TR	ANSPORT	PROTEIN SEC20
31:00:00	6i3m-E	2.9	6.2	94	354	10	MOLECULE:	TRANSLATIO	N INITIA	TION FACTOR EIF-2B SUBUNIT ALPH
32:00:00	3qbg-A	2.9	10.3	103	261	8	MOLECULE:	HALORHODOP	SIN	
33:00:00	4ymt-B	2.9	4	83	216	10	MOLECULE:	ABC-TYPE P	OLAR AMI	NO ACID TRANSPORT SYSTEM, ATPAS
34:00:00	5lnk-J	2.9	6.7	80	175	5	MOLECULE:	MITOCHONDR	IAL COMP	LEX I, 51 KDA SUBUNIT
35:00:00	1whu-A	2.9	5.5	74	104	9	MOLECULE:	POLYNUCLEO	TIDE PHO	SPHORYLASE 3'-5' RNA
36:00:00	4c0n-A	2.9	4.7	86	150	8	MOLECULE:	2-ON-2 HEM	OGLOBIN	
37:00:00	8qa2-A	2.9	10.6	72	215	7	MOLECULE:	GAP JUNCTI	ON BETA-	2 PROTEIN
38:00:00	6vm0-A	2.9	18.1	85	363	4	MOLECULE:	GLYCINE RE	CEPTOR S	UBUNIT ALPHAZ1
39:00:00	8wm8-B	2.9	5.3	89	265	10	MOLECULE:	NITRATE TR	ANSPORT	ATP-BINDING PROTEIN
40:00:00	3h3m-A	2.9	5.6	73	89	12	MOLECULE:	FLAGELLAR	PROTEIN	FLIT
41:00:00	7xdi-D	2.9	6.1	79	124	5	MOLECULE:	VP1		
42:00:00	8odu-C	2.9	23.2	90	232	10	MOLECULE:	ATPASE GET	3	
43:00:00	3nnq-A	2.9	4.4	66	95	8	MOLECULE:	N-TERMINAL	DOMAIN	OF MOLONEY MURINE LEUKEMIA VIRU
44:00:00	3c1d-B	2.9	16.7	76	154	8	MOLECULE:	REGULATORY	PROTEIN	RECX
45:00:00	6zyd-A	2.9	21.6	106	323	4	MOLECULE:	LOW CONDUC	TANCE ME	CHANOSENSITIVE CHANNEL YNAI,LOW
46:00:00	2q5z-B	2.9	15.7	69	94	7	MOLECULE:	HYPOTHETIC	AL PROTE	IN
47:00:00	5o5j-O	2.9	5.9	63	88	16	MOLECULE:	16S RRNA		
48:00:00	7woo-Z	2.9	30	109	746	4	MOLECULE:	NUCLEOPORI	N NIC96	
49:00:00	3i4m-D	2.9	10.4	84	187	7	MOLECULE:	DNA-DIRECT	ED RNA P	OLYMERASE II SUBUNIT RPB1
50:00:00	4cej-A	2.9	30	128	1177	7	MOLECULE:	ATP-DEPEND	ENT HELI	CASE/NUCLEASE SUBUNIT A
51:00:00	8t53-A	2.9	5.6	122	398	6	MOLECULE:	UNDECAPREN	YL-PHOSP	HATE GALACTOSE PHOSPHOTRANSFERA
52:00:00	6rfl-I	2.9	8.6	79	773	4	MOLECULE:	DNA-DEPEND	ENT RNA	POLYMERASE SUBUNIT RPO132
53:00:00	7way-A	2.9	33.4	131	960	5	MOLECULE:	DNA (33-ME	R)	
54:00:00	8kee-C	2.9	13.9	89	505	7	MOLECULE:	SHEATH		
55:00:00	1jog-D	2.9	7.5	77	136	6	MOLECULE:	HYPOTHETIC	AL PROTE	IN HI0074
56:00:00	2k85-A	2.9	3.1	58	65	5	MOLECULE:	GLUCOCORTI	COID REC	EPTOR DNA-BINDING FACTOR 1
57:00:00	6u8y-d	2.9	6.5	72	89	3	MOLECULE:	MONOVALENT	CATION/	H+ ANTIPORTER SUBUNIT E
58:00:00	8arl-A	2.9	4.6	80	232	5	MOLECULE:	TRYPTOPHAN	#NAME?	TIGEN

59:00:00	8eau-e	2.9	13.9	67	72	4	MOLECULE:	V-TYPE PRO	TON ATPA	SE SUBUNIT A, VACUOLAR ISOFORM
60:00:00	5d67-D	2.9	7.6	74	95	5	MOLECULE:	EF-HAND CA	LCIUM-BI	NDING DOMAIN-CONTAINING PROTEIN
61:00:00	8iqi-C	2.9	19.7	115	916	3	MOLECULE:	PUTATIVE P	RIMASE C	962R
62:00:00	1x8z-A	2.9	8.9	92	151	8	MOLECULE:	INVERTASE/	PECTIN M	ETHYLESTERASE INHIBITOR FAMILY
63:00:00	3vr8-D	2.9	14.7	83	129	10	MOLECULE:	FLAVOPROTE	IN SUBUN	IT OF COMPLEX II
64:00:00	3mjp-B	2.9	4.3	84	146	5	MOLECULE:	HEMOGLOBIN	SUBUNIT	ALPHA-A
65:00:00	5u3i-D	2.9	4	77	145	14	MOLECULE:	HEMOGLOBIN	SUBUNIT	ALPHA
66:00:00	2hhe-B	2.9	4.7	79	145	13	MOLECULE:	HEMOGLOBIN	(DEOXY)	(ALPHA CHAIN)
67:00:00	3k8b-B	2.9	4	77	146	17	MOLECULE:	HEMOGLOBIN	SUBUNIT	ALPHA-A
68:00:00	8vyl-D	2.9	4.5	78	146	14	MOLECULE:	HEMOGLOBIN	SUBUNIT	ALPHA
69:00:00	3gqp-D	2.9	4.4	78	145	14	MOLECULE:	HEMOGLOBIN	SUBUNIT	ALPHA
70:00:00	3vrf-B	2.9	4.5	79	146	13	MOLECULE:	HEMOGLOBIN	SUBUNIT	ALPHA
71:00:00	2hhe-D	2.9	4.7	80	145	13	MOLECULE:	HEMOGLOBIN	(DEOXY)	(ALPHA CHAIN)
72:00:00	1fhj-D	2.9	4	76	146	16	MOLECULE:	HEMOGLOBIN	(ALPHA	CHAIN)
73:00:00	3vrg-B	2.9	4	77	146	14	MOLECULE:	HEMOGLOBIN	SUBUNIT	ALPHA
74:00:00	6wg7-D	2.9	11.8	104	219	7	MOLECULE:	DNA (35-ME	R)	
75:00:00	7uvb-D	2.9	3.3	79	145	11	MOLECULE:	HEMOGLOBIN	SUBUNIT	ALPHA
76:00:00	6on4-A	2.9	15.5	93	217	4	MOLECULE:	HTH-TYPE T	RANSCRIP	TIONAL REPRESSOR NANR
77:00:00	6wg7-H	2.9	11.8	104	219	7	MOLECULE:	DNA (35-ME	R)	
78:00:00	3kdw-A	2.8	15.1	100	206	3	MOLECULE:	PUTATIVE S	UGAR BIN	DING PROTEIN
79:00:00	4pi0-A	2.8	27.6	99	390	7	MOLECULE:	UNKNOWN PE	PTIDE	
80:00:00	7lsy-Y	2.8	10.4	108	855	7	MOLECULE:	X-RAY REPA	IR CROSS	-COMPLEMENTING PROTEIN 6
81:00:00	7zw6-A	2.8	26	115	793	5	MOLECULE:	SLR0869 PR	OTEIN	
82:00:00	1uzc-A	2.8	2.7	54	69	7	MOLECULE:	HYPOTHETIC	AL PROTE	IN FLJ21157
83:00:00	2lor-A	2.8	7.2	64	108	5	MOLECULE:	TRANSMEMBR	ANE PROT	EIN 141
84:00:00	5gm2-B	2.8	22.8	90	283	8	MOLECULE:	O-METHYLRA	NSFERASE	
85:00:00	5hdi-A	2.8	4.9	123	397	2	MOLECULE:	CYTOCHROME	P450 14	4
86:00:00	7s5l-A	2.8	5.6	90	378	12	MOLECULE:	CEMBRENE A	SYNTHAS	E
87:00:00	5udb-4	2.8	22.7	95	751	9	MOLECULE:	DNA REPLIC	ATION LI	CENSING FACTOR MCM2
88:00:00	4uig-A	2.8	5.4	56	91	7	MOLECULE:	COPPER SEN	SITIVE O	PERON REPRESSOR
89:00:00	7zpo-M	2.8	5.8	124	438	5	MOLECULE:	KTR SYSTEM	POTASSI	UM UPTAKE PROTEIN A
90:00:00	6zz6-B	2.8	26.1	93	423	8	MOLECULE:	STRUCTURAL	MAINTEN	ANCE OF CHROMOSOMES PROTEIN 1,S
91:00:00	8iic-B	2.8	12.7	122	425	7	MOLECULE:	POLYMERASE	POLYPRO	TEIN
92:00:00	2ijd-1	2.8	14.8	119	644	6	MOLECULE:	PICORNAIN	3C, RNA-	DIRECTED RNA POLYMERASE
93:00:00	7r5s-I	2.8	25.3	121	622	6	MOLECULE:	CENTROMERE	PROTEIN	H
94:00:00	6s6b-B	2.8	3.7	79	154	5	MOLECULE:	CRISPR-ASS	OCIATED	PROTEIN, CMR5 FAMILY
95:00:00	7e38-B	2.8	8.3	96	305	4	MOLECULE:	DEOXYPODOP	HYLLOTOX	IN SYNTHASE
96:00:00	6tmi-B	2.8	19.5	104	246	8	MOLECULE:	ATP SYNTHA	SE SUBUN	IT ALPHA
97:00:00	6e11-E	2.8	15.9	99	210	6	MOLECULE:	UNKNOWN (C	LAW)	
98:00:00	2wj9-B	2.8	6	79	145	6	MOLECULE:	INTERGENIC	#NAME?	PROTEIN
99:00:00	3bjd-C	2.8	14.8	90	324	3	MOLECULE:	PUTATIVE 3	#NAME?	-(ACYL-CARRIER-PROTEIN) SYNTHAS
0:00	5abb-A	2.8	18.7	72	443	7	MOLECULE:	PROTEIN TR	ANSLOCAS	E SUBUNIT SECY
1:00	8tsh-C	2.8	5.5	97	259	5	MOLECULE:	ABC TRANSP	ORTER AT	P-BINDING PROTEIN
2:00	8hra-C	2.8	15.7	109	852	6	MOLECULE:	ARCHAEAL A	TPASE	
3:00	8ciw-A	2.8	14.5	105	555	9	MOLECULE:	(2S)-METHY	LSUCCINY	L-COA DEHYDROGENASE
4:00	6lo8-D	2.8	8	83	119	11	MOLECULE:	MITOCHONDR	IAL IMPO	RT INNER MEMBRANE TRANSLOCASE S
5:00	7zr6-C	2.8	16.8	90	277	6	MOLECULE:	HEAT SHOCK	PROTEIN	HSP 90-BETA
6:00	8ipr-A	2.8	24.2	120	504	8	MOLECULE:	COMPONENT	LINKED W	ITH THE ASSEMBLY OF CYTOCHROME'
7:00	7bmh-A	2.8	8.4	92	247	4	MOLECULE:	OPSIN		
8:00	6zvh-y	2.8	2.7	57	72	11	MOLECULE:	18S RRNA		
9:00	6n39-A	2.8	11.3	76	404	3	MOLECULE:	DEPHOSPHO-	COA KINA	SE
10:00	7v99-A	2.8	20.7	156	991	6	MOLECULE:	TELOMERASE	REVERSE	TRANSCRIPTASE
11:00	6k7l-A	2.8	26	92	992	10	MOLECULE:	PHOSPHOLIP	ID-TRANS	PORTING ATPASE
12:00	5b7j-A	2.8	3.7	82	110	5	MOLECULE:	SWITCH-ACT	IVATING	PROTEIN 1

13:00	6whg-A	2.8	21.1	140	523	8	MOLECULE:	CALCIUM CH	ANNEL YV	C1
14:00	8esv-B	2.8	4.5	71	255	6	MOLECULE:	DISINTEGRI	N AND ME	TALLOPROTEINASE DOMAIN-CONTAINI
15:00	6dlu-P	2.8	24.6	121	747	6	MOLECULE:	DYNAMIN-1		
16:00	6kex-A	2.8	7.3	66	349	5	MOLECULE:	PHOSPHORIB	ULOKINAS	E
17:00	5dn6-H	2.8	4.3	68	110	3	MOLECULE:	CHAIN A		
18:00	6urt-A	2.8	18.7	125	331	8	MOLECULE:	LOW CONDUC	TANCE ME	CHANOSENSITIVE CHANNEL YNAI
19:00	4kha-A	2.8	16.1	75	360	7	MOLECULE:	SPT16M-HIS	TONE H2B	1.1 CHIMERA
20:00	7bw0-R	2.8	14.6	108	280	8	MOLECULE:	SOLUBLE CY	TOCHROME	B562,G-PROTEIN COUPLED BILE AC
21:00	6ql4-A	2.8	25.5	106	660	7	MOLECULE:	PUTATIVE M	ITOCHOND	RIAL DYNAMIN PROTEIN
22:00	5iy7-M	2.8	16.1	82	310	5	MOLECULE:	DNA-DIRECT	ED RNA P	OLYMERASE II SUBUNIT RPB1
23:00	7jr7-A	2.8	16.9	119	588	7	MOLECULE:	ATP-BINDIN	G CASSET	TE SUB-FAMILY G MEMBER 5
24:00:00	8anq-C	2.8	9.3	96	227	5	MOLECULE:	BACTERIORH	ODOPSIN	
25:00:00	5ys9-A	2.8	33.7	130	692	5	MOLECULE:	ACYL-COENZ	YME A OX	IDASE 3
26:00:00	8otx-A	2.8	18.6	132	1054	6	MOLECULE:	SPERM-SPEC	IFIC SOD	IUM PROTON EXCHANGER
27:00:00	3pux-G	2.8	10.3	85	293	2	MOLECULE:	MALTOSE-BI	NDING PE	RIPLASMIC PROTEIN
28:00:00	7eeb-K	2.8	7.9	87	143	2	MOLECULE:	ENHANCED G	REEN FLU	ORESCENT PROTEIN,CATION CHANNEL
29:00:00	8qfs-A	2.8	30.1	140	1183	7	MOLECULE:	ELONGATION	FACTOR	TU
30:00:00	6wgv-A	2.8	13.1	92	173	3	MOLECULE:	CORRINOID	ADENOSYL	TRANSFERASE
31:00:00	7o3d-A	2.8	11.7	112	709	11	MOLECULE:	TRANSCRIPT	ION ELON	GATION FACTOR SPT6
32:00:00	3k1z-A	2.8	5.6	105	241	5	MOLECULE:	HALOACID D	EHALOGEN	ASE-LIKE HYDROLASE DOMAIN-CONTA
33:00:00	8axa-A	2.8	8.4	88	465	3	MOLECULE:	CAS12K		
34:00:00	1zp2-A	2.8	15.5	84	227	6	MOLECULE:	RNA POLYME	RASE II	HOLOENZYME CYCLIN-LIKE SUBUNIT
35:00:00	3ed5-A	2.8	5.3	89	232	8	MOLECULE:	YFNB		
36:00:00	8yb7-C	2.8	15	90	455	7	MOLECULE:	PAPAIN-LIK	E PROTEA	SE NSP3
37:00:00	5xj5-A	2.8	9.9	86	200	7	MOLECULE:	GLYCEROL-3	#NAME?	TE ACYLTRANSFERASE
38:00:00	5edl-A	2.8	12	95	197	9	MOLECULE:	PUTATIVE H	MP/THIAM	INE PERMEASE PROTEIN YKOE
39:00:00	7drt-B	2.8	18.4	121	496	4	MOLECULE:	PROTEIN WN	T-3A	
40:00:00	8e4y-A	2.8	17	100	651	5	MOLECULE:	GLYCEROL-3	#NAME?	TE ACYLTRANSFERASE 1, MITOCHOND
41:00:00	1gak-A	2.8	2.9	65	137	8	MOLECULE:	FERTILIZAT	ION PROT	EIN
42:00:00	8j56-C	2.8	4	74	160	7	MOLECULE:	FLAGELLAR	TRANSCRI	PTIONAL REGULATOR FLHD
43:00:00	8fed-J	2.8	7.4	98	263	1	MOLECULE:	VIRULENCE	FACTOR M	CE FAMILY PROTEIN
44:00:00	2voh-A	2.8	4.5	76	155	9	MOLECULE:	BCL-2-RELA	TED PROT	EIN A1
45:00:00	8ovw-Q	2.8	8.2	83	258	8	MOLECULE:	CENTROMERE	#NAME?	PROTEIN 1
46:00:00	2rld-C	2.8	4.9	77	116	5	MOLECULE:	UNCHARACTE	RIZED PR	OTEIN
47:00:00	7w6k-A	2.8	25.7	113	410	3	MOLECULE:	GMALMT12/Q	UAC1	
48:00:00	7m2w-G	2.8	19.5	108	712	4	MOLECULE:	TUBULIN GA	MMA CHAI	N
49:00:00	5ean-A	2.8	25.1	135	1051	8	MOLECULE:	DNA REPLIC	ATION AT	P-DEPENDENT HELICASE/NUCLEASE D
50:00:00	7k4m-B	2.8	4.2	75	146	13	MOLECULE:	HEMOGLOBIN	SUBUNIT	ALPHA
51:00:00	5e83-B	2.8	4	77	146	14	MOLECULE:	HEMOGLOBIN	SUBUNIT	ALPHA
52:00:00	5u3i-B	2.8	4.1	77	146	14	MOLECULE:	HEMOGLOBIN	SUBUNIT	ALPHA
53:00:00	1ut0-A	2.8	5.1	89	154	8	MOLECULE:	CYTOGLOBIN		
54:00:00	3bom-D	2.8	4.5	80	147	13	MOLECULE:	HEMOGLOBIN	SUBUNIT	ALPHA-4
55:00:00	7jy0-B	2.8	4.6	79	146	14	MOLECULE:	HEMOGLOBIN	SUBUNIT	ALPHA
56:00:00	1mbs-A	2.8	4.6	79	153	8	MOLECULE:	MYOGLOBIN		
57:00:00	1shr-B	2.8	4.1	77	146	13	MOLECULE:	HEMOGLOBIN	ALPHA C	HAIN
58:00:00	7uf6-D	2.8	4.5	76	146	13	MOLECULE:	HEMOGLOBIN	SUBUNIT	ALPHA
59:00:00	3w4u-B	2.8	4	77	145	14	MOLECULE:	HEMOGLOBIN	SUBUNIT	ZETA
60:00:00	3w4u-F	2.8	3.9	76	145	14	MOLECULE:	HEMOGLOBIN	SUBUNIT	ZETA
61:00:00	7jxz-D	2.8	4.1	77	146	14	MOLECULE:	HEMOGLOBIN	SUBUNIT	ALPHA
62:00:00	2qls-D	2.8	4.5	75	146	15	MOLECULE:	HEMOGLOBIN	SUBUNIT	ALPHA
63:00:00	7jxz-B	2.8	4.1	77	146	14	MOLECULE:	HEMOGLOBIN	SUBUNIT	ALPHA
64:00:00	8egi-B	2.8	3.9	76	146	14	MOLECULE:	HEMOGLOBIN	SUBUNIT	ALPHA
65:00:00	7uf6-B	2.8	4	77	146	14	MOLECULE:	HEMOGLOBIN	SUBUNIT	ALPHA
66:00:00	3w4u-D	2.8	4.4	79	145	14	MOLECULE:	HEMOGLOBIN	SUBUNIT	ZETA

67:00:00	2b7h-B	2.8	4	76	144	14	MOLECULE:	HEMOGLOBIN	ALPHA C	HAIN
68:00:00	3gqr-B	2.8	4	76	145	14	MOLECULE:	HEMOGLOBIN	SUBUNIT	ALPHA
69:00:00	7cue-B	2.8	4.6	79	146	14	MOLECULE:	HEMOGLOBIN	SUBUNIT	ALPHA
70:00:00	1urv-A	2.8	5.1	89	154	8	MOLECULE:	CYTOGLOBIN		
71:00:00	6xe7-B	2.8	4.1	77	146	14	MOLECULE:	HEMOGLOBIN	SUBUNIT	ALPHA
72:00:00	1nej-D	2.8	4.6	78	146	14	MOLECULE:	HEMOGLOBIN	ALPHA C	HAIN
73:00:00	3bom-B	2.8	4.1	78	147	13	MOLECULE:	HEMOGLOBIN	SUBUNIT	ALPHA-4
74:00:00	8dov-F	2.8	4	76	144	14	MOLECULE:	HEMOGLOBIN	SUBUNIT	ALPHA
75:00:00	6wg7-G	2.8	12.6	106	219	8	MOLECULE:	DNA (35-ME	R)	
76:00:00	6wfq-D	2.8	12.6	106	219	8	MOLECULE:	HTH-TYPE T	RANSCRIP	TIONAL REPRESSOR NANR
77:00:00	6u4b-A	2.7	19.8	120	570	8	MOLECULE:	WBBM PROTE	IN	
78:00:00	3bhn-A	2.7	10.1	64	214	5	MOLECULE:	THIJ/PFPI	DOMAIN P	ROTEIN
79:00:00	3f0c-A	2.7	3.7	66	193	9	MOLECULE:	TRANSCRIPT	IONAL RE	GULATOR
80:00:00	7yim-A	2.7	6.3	116	592	8	MOLECULE:	ALPHA-FETO	PROTEIN	
81:00:00	5nzz-A	2.7	12.1	103	378	10	MOLECULE:	TGF-BETA-A	CTIVATED	KINASE 1 AND MAP3K7-BINDING PR
82:00:00	6tgb-A	2.7	22.6	99	1450	3	MOLECULE:	DEDICATOR	OF CYTOK	INESIS PROTEIN 2
83:00:00	6ppi-A	2.7	18.2	104	413	8	MOLECULE:	PORTAL PRO	TEIN	
84:00:00	6vy1-A	2.7	10.4	71	121	7	MOLECULE:	PREFOLDIN	SUBUNIT	ALPHA 2
85:00:00	5hzi-A	2.7	7	85	476	8	MOLECULE:	INTERSECTI	N-1,NPH1	-1,INTERSECTIN-1
86:00:00	2gnx-A	2.7	13	80	288	5	MOLECULE:	HYPOTHETIC	AL PROTE	IN
87:00:00	8tdl-A	2.7	18	116	364	4	MOLECULE:	MECHANOSEN	SITIVE I	ON CHANNEL PROTEIN 10
88:00:00	2p01-A	2.7	11.3	93	323	5	MOLECULE:	ALPHA-2-MA	CROGLOBU	LIN RECEPTOR-ASSOCIATED
89:00:00	2vyw-A	2.7	4.4	66	148	6	MOLECULE:	HEMOGLOBIN		
90:00:00	2xwu-B	2.7	15.3	123	910	10	MOLECULE:	SUMO-CONJU	GATING E	NZYME UBC9
91:00:00	7roa-A	2.7	4.8	69	117	6	MOLECULE:	ENTV		
92:00:00	8osg-B	2.7	13.4	89	323	7	MOLECULE:	MAGNESIUM-	CHELATAS	E SUBUNIT CHLI
93:00:00	2js9-A	2.7	3.2	57	81	4	MOLECULE:	SAPOSIN-LI	KE PROTE	IN FAMILY PROTEIN 5
94:00:00	3lwj-A	2.7	4.9	71	193	10	MOLECULE:	PUTATIVE T	ETR-FAMI	LY TRANSCRIPTIONAL REGULATOR
95:00:00	3ajb-A	2.7	6.6	104	293	5	MOLECULE:	PEROXISOMA	L BIOGEN	ESIS FACTOR 3
96:00:00	8eki-A	2.7	20.7	82	170	6	MOLECULE:	PROTEIN TR	ANSPORT	PROTEIN SEC20
97:00:00	8ppr-D	2.7	24.3	88	248	11	MOLECULE:	KINETOCHOR	E-ASSOCI	ATED PROTEIN DSN1 HOMOLOG
98:00:00	5v8f-6	2.7	18.1	100	692	8	MOLECULE:	DNA REPLIC	ATION LI	CENSING FACTOR MCM2
99:00:00	7eew-A	2.7	17.2	89	611	9	MOLECULE:	TYPE I RES	TRICTION	#NAME?
0:00	2qkm-B	2.7	9.7	87	266	1	MOLECULE:	SPBC3B9.21	PROTEIN	
1:00	7p4a-A	2.7	12.2	98	246	3	MOLECULE:	STL		
2:00	7vf2-A	2.7	23.3	121	1196	12	MOLECULE:	PROTEIN VI	RILIZER	HOMOLOG
3:00	7sjr-A	2.7	29.2	96	911	13	MOLECULE:	DNA HELICA	SE	
4:00	6c66-I	2.7	3.8	71	177	8	MOLECULE:	CRISPR-ASS	OCIATED	HELICASE, CAS3 FAMILY
5:00	2e87-A	2.7	15.2	102	356	5	MOLECULE:	HYPOTHETIC	AL PROTE	IN PH1320
6:00	7yyg-A	2.7	7.7	87	275	7	MOLECULE:	PUTATIVE O	UTER PRO	TEIN N
7:00	8ucq-A	2.7	23.2	114	1232	6	MOLECULE:	SEC7 DOMAI	N-CONTAI	NING PROTEIN
8:00	2fbq-A	2.7	14.9	64	213	6	MOLECULE:	PROBABLE T	RANSCRIP	TIONAL REGULATOR
9:00	7pgr-F	2.7	22	151	2423	6	MOLECULE:	NEUROFIBRO	MIN	
10:00	2k3q-A	2.7	4.1	81	118	12	MOLECULE:	TUSP1		
11:00	5wrr-B	2.7	6.5	64	438	8	MOLECULE:	PSEUDOKINA	SE FAM20	A
12:00	6vrb-C	2.7	7.4	73	229	4	MOLECULE:	RNA (52-ME	R)	
13:00	7pds-A	2.7	15.8	88	457	6	MOLECULE:	SIMILAR TO	D. NODO	SUS VAPE
14:00	5dca-A	2.7	15.1	134	1948	7	MOLECULE:	PRE-MRNA-S	PLICING	HELICASE BRR2
15:00	7bss-A	2.7	23.4	84	816	8	MOLECULE:	ATP11C		
16:00	7obq-y	2.7	14	82	454	7	MOLECULE:	SRP RNA		
17:00	1sf9-A	2.7	9.1	80	118	3	MOLECULE:	YFHH HYPOT	HETICAL	PROTEIN
18:00	7ody-C	2.7	11.6	73	100	5	MOLECULE:	MAZG-LIKE	PYROPHOS	PHOHYDROLASE (MAZZ)
19:00	6nf4-A	2.7	19.3	98	421	6	MOLECULE:	OTOPETRIN1		
20:00	1tjf-B	2.7	10	89	186	2	MOLECULE:	ADENYLYL C	YCLASE-A	SSOCIATED PROTEIN

21:00	8ovt-A	2.7	5	70	288	4	MOLECULE:	YEGT GLYCO	SYLTRANS	FERASE
22:00	6cfw-C	2.7	10	86	114	7	MOLECULE:	MONOVALENT	CATION/	H+ ANTIPORTER SUBUNIT D
23:00	2xl4-A	2.7	9.4	81	147	5	MOLECULE:	LMO0438 PR	OTEIN	
24:00:00	7exx-A	2.7	28.3	117	440	7	MOLECULE:	DNA PHOSPH	OROTHIOA	TION-DEPENDENT RESTRICTION PROT
25:00:00	7t5p-B	2.7	9.6	82	419	7	MOLECULE:	SUMO-INTER	ACTING M	OTIF-CONTAINING PROTEIN 1
26:00:00	6d5f-A	2.7	13.4	93	193	9	MOLECULE:	FIMBRIAL P	ROTEIN	
27:00:00	5kkl-B	2.7	9.4	87	838	2	MOLECULE:	PUTATIVE P	OLYCOMB	PROTEIN EED
28:00:00	5h72-A	2.7	2.7	58	67	9	MOLECULE:	FLAGELLAR	BIOSYNTH	ETIC PROTEIN FLIP
29:00:00	8rf0-A	2.7	30	136	2780	4	MOLECULE:	CYCLIC BET	A-(1,2)-	GLUCAN SYNTHASE NDVB
30:00:00	5t58-A	2.7	13.5	93	233	10	MOLECULE:	KLLA0F0234	3P	
31:00:00	5htf-B	2.7	7.9	91	260	7	MOLECULE:	FOLDASE PR	OTEIN PR	SA 1
32:00:00	5yet-A	2.7	16.3	93	397	4	MOLECULE:	UNCHARACTE	RIZED PR	OTEIN R354
33:00:00	5eqw-B	2.7	12.2	75	123	3	MOLECULE:	PUTATIVE M	AJOR COA	T PROTEIN
34:00:00	6w2q-A	2.7	4.2	66	195	5	MOLECULE:	JUNCTION 3	4	
35:00:00	2dh4-A	2.7	10.8	113	326	3	MOLECULE:	YPL069C		
36:00:00	5yfp-C	2.7	13.4	89	790	8	MOLECULE:	EXOCYST CO	MPLEX CO	MPONENT SEC3
37:00:00	7nhr-A	2.7	13.1	88	663	6	MOLECULE:	PUTATIVE T	RANSMEMB	RANE PROTEIN WZC
38:00:00	8j0h-A	2.7	8.4	82	134	6	MOLECULE:	UNCHARACTE	RIZED PR	OTEIN C4H3.06
39:00:00	2ly9-A	2.7	5.2	61	74	8	MOLECULE:	ZINC FINGE	RS AND H	OMEOBOXES PROTEIN 1
40:00:00	8c81-B	2.7	17.6	95	539	9	MOLECULE:	PROTEIN OR	M1	
41:00:00	3wvo-A	2.7	17.4	89	543	10	MOLECULE:	CRISPR-ASS	OCIATED	PROTEIN, CSE1 FAMILY
42:00:00	7os0-A	2.7	9.2	111	1130	5	MOLECULE:	CAS13A		
43:00:00	3egw-C	2.7	17.1	104	225	9	MOLECULE:	RESPIRATOR	Y NITRAT	E REDUCTASE 1 ALPHA CHAIN
44:00:00	3mw6-A	2.7	6.4	80	122	13	MOLECULE:	UNCHARACTE	RIZED PR	OTEIN NMB1681
45:00:00	6ekr-A	2.7	10.8	91	303	11	MOLECULE:	TYPE II SI	TE-SPECI	FIC DEOXYRIBONUCLEASE
46:00:00	3rgc-B	2.7	4.8	85	249	5	MOLECULE:	POSSIBLE P	ERIPLASM	IC PROTEIN
47:00:00	7x07-A	2.7	24	89	633	10	MOLECULE:	ATP-BINDIN	G CASSET	TE SUB-FAMILY D MEMBER 1
48:00:00	8hp7-A	2.7	4.6	97	241	9	MOLECULE:	(S)-2-HALO	ACID DEH	ALOGENASE
49:00:00	6xpd-A	2.7	27.1	115	303	8	MOLECULE:	ZINC TRANS	PORTER 8	
50:00:00	7zke-E	2.7	19.7	112	1623	6	MOLECULE:	DNA (36-ME	R)	
51:00:00	7xzj-A	2.7	30.3	97	423	9	MOLECULE:	CTAP3		
52:00:00	3utk-A	2.7	3.4	60	95	5	MOLECULE:	LIPOPROTEI	N OUTS	
53:00:00	6xdt-B	2.7	4	74	146	14	MOLECULE:	HEMOGLOBIN	SUBUNIT	ALPHA
54:00:00	2b7h-D	2.7	4	74	145	15	MOLECULE:	HEMOGLOBIN	ALPHA C	HAIN
55:00:00	1si4-D	2.7	4.7	79	146	13	MOLECULE:	HEMOGLOBIN	ALPHA C	HAIN
56:00:00	7uf7-B	2.7	4.5	75	146	15	MOLECULE:	HEMOGLOBIN	SUBUNIT	ALPHA
57:00:00	5ker-B	2.7	4.3	75	146	13	MOLECULE:	ALPHA-GLOB	IN	
58:00:00	2r1h-D	2.7	4.5	76	147	13	MOLECULE:	HEMOGLOBIN	SUBUNIT	ALPHA-4
59:00:00	3hf4-F	2.7	3.9	73	146	14	MOLECULE:	HEMOGLOBIN	SUBUNIT	ALPHA-1/2
60:00:00	3gqr-H	2.7	4.4	75	145	15	MOLECULE:	HEMOGLOBIN	SUBUNIT	ALPHA
61:00:00	1v5h-A	2.7	4.6	78	151	8	MOLECULE:	CYTOGLOBIN		
62:00:00	5aqd-M	2.6	4.4	104	184	4	MOLECULE:	PHYCOERYTH	RIN ALPH	A SUBUNIT
63:00:00	8agg-A	2.6	9.2	60	489	5	MOLECULE:	PHOSPHOCHO	LINE HYD	ROLASE LEM3
64:00:00	5lpa-A	2.6	3.9	70	267	9	MOLECULE:	STREPTOMYC	IN 3''-A	DENYLYLTRANSFERASE
65:00:00	5e6g-B	2.6	8	72	114	10	MOLECULE:	DE NOVO DE	SIGNED P	ROTEIN CA01
66:00:00	4me9-A	2.6	4.3	67	188	4	MOLECULE:	TRANSCRIPT	IONAL RE	GULATOR, TETR FAMILY
67:00:00	7f5v-A	2.6	16.3	134	689	7	MOLECULE:	DELTA-1-PY	RROLINE-	5-CARBOXYLATE SYNTHASE
68:00:00	3ut2-A	2.6	16.7	117	735	4	MOLECULE:	CATALASE-P	EROXIDAS	E 2
69:00:00	7uic-b	2.6	5.3	49	65	6	MOLECULE:	MEDIATOR O	F RNA PO	LYMERASE II TRANSCRIPTION SUBUN
70:00:00	8t1l-X	2.6	20.3	80	127	9	MOLECULE:	MEDIATOR O	F RNA PO	LYMERASE II TRANSCRIPTION SUBUN
71:00:00	7m5t-A	2.6	8.6	73	100	8	MOLECULE:	DE NOVO DE	SIGNED P	ROTEIN 0515
72:00:00	8dmg-B	2.6	10.3	187	1033	7	MOLECULE:	BIFUNCTION	AL CYTOC	HROME P450/NADPH--P450 REDUCTAS
73:00:00	7w7c-B	2.6	4.6	82	159	9	MOLECULE:	PUTATIVE A	BC TRANS	PORT SYSTEM, ATP-BINDING PROTEI
74:00:00	8d0b-F	2.6	18.2	110	915	7	MOLECULE:	CST COMPLE	X SUBUNI	T CTC1

75:00:00	4a01-A	2.6	7.4	132	740	3	MOLECULE:	PROTON PYR	OPHOSPHA	TASE
76:00:00	2a6o-A	2.6	5.4	78	152	6	MOLECULE:	5'-D(*CP*C	P*CP*CP*	TP*AP*GP*CP*TP*TP*TP*AP*GP*CP*T
77:00:00	8cws-A	2.6	12.3	94	451	5	MOLECULE:	F4132-2 CH	AIN A	
78:00:00	6xjh-A	2.6	5.2	93	251	6	MOLECULE:	PHENOL-SOL	UBLE MOD	ULIN EXPORT ABC TRANSPORTER PER
79:00:00	8d4a-A	2.6	18.7	113	1207	8	MOLECULE:	ORFB_ZN_RI	BBON DOM	AIN-CONTAINING PROTEIN
80:00:00	4hts-A	2.6	4.7	98	228	7	MOLECULE:	SEC-INDEPE	NDENT PR	OTEIN TRANSLOCASE PROTEIN TATC
81:00:00	8r33-A	2.6	12.5	102	489	3	MOLECULE:	PHO90 ISOF	ORM 1	
82:00:00	6d0j-B	2.6	4.9	99	396	5	MOLECULE:	CLC-TYPE F	LUORIDE/	PROTON ANTIPORTER
83:00:00	8ap9-G	2.6	7.2	62	279	5	MOLECULE:	ATP SYNTHA	SE GAMMA	SUBUNIT
84:00:00	6hgc-A	2.6	19.1	81	304	2	MOLECULE:	UBIQUITIN	CARBOXYL	#NAME?
85:00:00	4i1s-A	2.6	8.7	87	238	11	MOLECULE:	MELANOMA D	IFFERENT	IATION ASSOCIATED PROTEIN-5
86:00:00	7s8u-A	2.6	8.5	97	667	4	MOLECULE:	SOLUTE CAR	RIER FAM	ILY 15 MEMBER 1
87:00:00	8ea3-W	2.6	24.5	96	514	3	MOLECULE:	TARGET_LE		
88:00:00	8csp-F	2.6	11.8	86	208	1	MOLECULE:	28S RIBOSO	MAL PROT	EIN S34, MITOCHONDRIAL
89:00:00	8cqv-A	2.6	5	96	202	6	MOLECULE:	NITROREDUC	TASE	
90:00:00	3e00-D	2.6	13.8	123	363	2	MOLECULE:	RETINOIC A	CID RECE	PTOR RXR-ALPHA
91:00:00	8jpj-A	2.6	30.2	137	733	4	MOLECULE:	H(+)/CL(-)	EXCHANG	E TRANSPORTER 6
92:00:00	2qeu-B	2.6	3.1	54	133	6	MOLECULE:	PUTATIVE C	ARBOXYMU	CONOLACTONE DECARBOXYLASE
93:00:00	8es4-C	2.6	10.6	81	181	6	MOLECULE:	GP35		
94:00:00	6zxr-A	2.6	6.4	89	212	6	MOLECULE:	ER LUMEN P	ROTEIN-R	ETAINING RECEPTOR 2
95:00:00	3o7q-A	2.6	7	107	414	7	MOLECULE:	L-FUCOSE-P	ROTON SY	MPORTER
96:00:00	4xom-A	2.6	5.7	98	206	4	MOLECULE:	COENZYME F	420:L-GL	UTAMATE LIGASE
97:00:00	7y7q-A	2.6	18.8	138	951	10	MOLECULE:	RNA-DEPEND	ENT RNA	POLYMERASE
98:00:00	7wkk-A	2.6	23.2	116	1684	4	MOLECULE:	MGC83295 P	ROTEIN	
99:00:00	6csv-A	2.6	6.9	58	90	9	MOLECULE:	CENTROSOMA	L PROTEI	N OF 63 KDA,CENTROSOMAL PROTEIN
0:00	7mdy-B	2.6	5.9	78	411	4	MOLECULE:	LIPOPROTEI	N TRANSP	ORTER SUBUNIT LOLE
1:00	1nek-C	2.6	5.7	79	129	4	MOLECULE:	SUCCINATE	DEHYDROG	ENASE FLAVOPROTEIN SUBUNIT
2:00	8b9z-K	2.6	8.1	78	96	8	MOLECULE:	NADH-UBIQU	INONE OX	IDOREDUCTASE CHAIN 3
3:00	6hux-A	2.6	25.9	113	369	9	MOLECULE:	H(2)-FORMI	NG METHY	LENETETRAHYDROMETHANOPTERIN
4:00	8h2m-A	2.6	28	109	754	8	MOLECULE:	TAPE TAIL	MEASURE	PROTEIN
5:00	2da3-A	2.6	6.7	70	80	9	MOLECULE:	ALPHA-FETO	PROTEIN	ENHANCER BINDING PROTEIN
6:00	6rfl-C	2.6	11.2	66	304	8	MOLECULE:	DNA-DEPEND	ENT RNA	POLYMERASE SUBUNIT RPO132
7:00	6thh-C	2.6	18.7	98	816	7	MOLECULE:	SIRV2 ACRI	D1 (GP02	) ANTI-CRISPR PROTEIN
8:00	3cit-A	2.6	4	72	155	11	MOLECULE:	SENSOR HIS	TIDINE K	INASE
9:00	6ahp-A	2.6	3.7	68	110	6	MOLECULE:	FLAGELLAR	PROTEIN	FLIL
10:00	2xyk-B	2.6	3.8	72	130	10	MOLECULE:	2-ON-2 HEM	OGLOBIN	
11:00	8syn-A	2.6	26	117	801	9	MOLECULE:	VPS35 ENDO	SOMAL PR	OTEIN-SORTING FACTOR-LIKE
12:00	1dek-A	2.6	8.7	96	241	5	MOLECULE:	DEOXYNUCLE	OSIDE MO	NOPHOSPHATE KINASE
13:00	2py8-C	2.6	16	84	123	8	MOLECULE:	HYPOTHETIC	AL PROTE	IN RBCX
14:00	1ypy-A	2.6	6.4	86	182	8	MOLECULE:	VIRION MEM	BRANE PR	OTEIN
15:00	7w6x-A	2.6	11.5	82	447	12	MOLECULE:	REGULATOR	OF SIGMA	-E PROTEASE RSEP
16:00	6idp-A	2.6	12.1	94	438	9	MOLECULE:	MATE FAMIL	Y EFFLUX	TRANSPORTER
17:00	2d7d-A	2.6	27.7	100	621	6	MOLECULE:	5'-D(P*TP*	TP*T)-3'	
18:00	6yxp-B	2.6	8.1	75	272	0	MOLECULE:	SWI/SNF CO	MPLEX SU	BUNIT SMARCC1
19:00	5dw7-A	2.6	14.1	94	309	3	MOLECULE:	GERMACRADI	ENOL/GEO	SMIN SYNTHASE
20:00	6xlp-A	2.6	13.8	98	586	8	MOLECULE:	LPS BINDIN	G PROTEI	N
21:00	7f3t-A	2.6	16	107	330	5	MOLECULE:	TRANSMEMBR	ANE PROT	EIN 120A
22:00	6v4a-C	2.6	16.3	88	593	3	MOLECULE:	NEUR_CHAN_	LBD DOMA	IN-CONTAINING PROTEIN
23:00	8psn-B	2.6	14.3	111	515	11	MOLECULE:	POLYMERASE	ACIDIC	PROTEIN (PA-LIKE)
24:00:00	6vrc-A	2.6	13.8	105	1007	6	MOLECULE:	CRISPR-ASS	OCIATED	ENDORIBONUCLEASE CAS13A
25:00:00	7znk-b	2.6	16.1	109	919	6	MOLECULE:	RNA		
26:00:00	1jud-A	2.6	4.8	95	220	7	MOLECULE:	L-2-HALOAC	ID DEHAL	OGENASE
27:00:00	6lo8-C	2.6	4	61	108	10	MOLECULE:	MITOCHONDR	IAL IMPO	RT INNER MEMBRANE TRANSLOCASE S
28:00:00	5gko-A	2.6	11.8	98	650	5	MOLECULE:	MACROLIDE	EXPORT A	TP-BINDING/PERMEASE PROTEIN MAC

29:00:00	3fay-A	2.6	24.7	139	379	9	MOLECULE:	RAS GTPASE	#NAME?	ING-LIKE PROTEIN IQGAP1	
30:00:00	8sgi-A	2.6	10.7	100	751	9	MOLECULE:	SODIUM/CAL	CIUM EXC	HANGER 1	
31:00:00	6w2r-B	2.6	8	81	221	15	MOLECULE:	JUNCTION 1	9 DHR54-	DHR79	
32:00:00	5csk-A	2.6	23.1	100	1996	6	MOLECULE:	ACETYL-COA	CARBOXY	LASE	
33:00:00	7nnh-X	2.6	7.7	122	637	11	MOLECULE:	ERYTHROCYT	E MEMBRA	NE PROTEIN 1	
34:00:00	8iq4-R	2.6	12	99	274	5	MOLECULE:	PROSTAGLAN	DIN F2-A	LPHA RECEPTOR	
35:00:00	7yca-O	2.6	4.2	63	96	3	MOLECULE:	LHCA1			
36:00:00	6zpa-A	2.6	4.5	65	173	2	MOLECULE:	DATZ			
37:00:00	6ny2-Y	2.6	22.1	135	915	8	MOLECULE:	DNA TARGET	STRAND		
38:00:00	8hdu-B	2.6	4	85	162	12	MOLECULE:	DE NOVO DE	SIGN CAV	ITATED PROTEIN	
39:00:00	6rfq-2	2.6	12.8	99	469	5	MOLECULE:	SUBUNIT NU	AM OF NA	DH:UBIQUINONE OXIDOREDUCTASE (C	
40:00:00	5ir2-A	2.6	10.4	67	222	9	MOLECULE:	CELLULASE			
41:00:00	2mab-A	2.6	3.6	76	109	7	MOLECULE:	ACINIFORM	SPIDROIN		
42:00:00	5jeq-A	2.6	32.8	110	227	5	MOLECULE:	NITRATE/NI	TRITE SE	NSOR PROTEIN NARQ	
43:00:00	8cqr-A	2.6	6.2	70	103	4	MOLECULE:	NINJURIN-1			
44:00:00	7lsv-B	2.6	6.2	83	158	4	MOLECULE:	CALMODULIN	#NAME?	NT PROTEIN KINASE	
45:00:00	5k8o-B	2.6	7.8	70	425	10	MOLECULE:	5-NITROANT	HRANILIC	ACID AMINOHYDROLASE	
46:00:00	4btp-A	2.6	12.3	114	748	5	MOLECULE:	P1			
47:00:00	8xje-B	2.6	9.3	64	149	2	MOLECULE:	YQEY			
48:00:00	5z58-1	2.6	21.5	93	1038	5	MOLECULE:	PRE-MRNA-P	ROCESSIN	G-SPLICING FACTOR 8	
49:00:00	7z1n-O	2.6	21	91	570	4	MOLECULE:	DNA-DIRECT	ED RNA P	OLYMERASE III SUBUNIT RPC1	
50:00:00	2odm-B	2.6	6.4	72	83	4	MOLECULE:	UPF0358 PR	OTEIN MW		995
51:00:00	2nnu-A	2.6	15	99	201	13	MOLECULE:	REGULATORY	PROTEIN	E2	
52:00:00	3u8p-A	2.6	9.6	78	336	5	MOLECULE:	CYTOCHROME	B562 IN	TEGRAL FUSION WITH ENHANCED GRE	
[truncated: 400,534 more chars]
